# The tomato *HIGH PIGMENT1/DAMAGED DNA BINDING PROTEIN 1* gene contributes to regulation of fruit ripening

**DOI:** 10.1038/s41438-018-0093-3

**Published:** 2019-02-01

**Authors:** Anquan Wang, Danyang Chen, Qiyue Ma, Jocelyn K. C. Rose, Zhangjun Fei, Yongsheng Liu, James J. Giovannoni

**Affiliations:** 1 School of Biotechnology and Food Engineering, Hefei University of Technology, Hefei, 230009 China; 2 Boyce Thompson Institute, Cornell University, Ithaca, NY 14853 USA; 3 Plant Biology Section, School of Integrative Plant Science, Cornell University, Ithaca, NY 14853 USA; 4 United States Department of Agriculture, Robert W. Holley Center, Cornell University, Ithaca, NY 14853 USA

**Keywords:** Systems analysis, Plant development

## Abstract

Fleshy fruit ripening is governed by multiple external and internal cues and accompanied by changes in color, texture, volatiles, and nutritional quality traits. While extended shelf-life and increased phytonutrients are desired, delaying ripening via genetic or postharvest means can be accompanied by reduced nutritional value. Here we report that the *high pigment 1* (*hp1*) mutation at the *UV-DAMAGED DNA BINDING PROTEIN 1* (*DDB1*) locus, previously shown to influence carotenoid and additional phytonutrient accumulation via altered light signal transduction, also results in delayed ripening and firmer texture, resulting at least in part from decreased ethylene evolution. Transcriptome analysis revealed multiple ethylene biosynthesis and signaling-associated genes downregulated in *hp1*. Furthermore, the *hp1* mutation impedes softening of the pericarp, placenta, columella as well as the whole fruit, in addition to reduced expression of the FRUITFUL2 (FUL2) MADS-box transcription factor and xyloglucan endotransglucosylase/hydrolase 5 (XTH5). These results indicate that *DDB1* influences a broader range of fruit development and ripening processes than previously thought and present an additional genetic target for increasing fruit quality and shelf-life.

## Fruit development: genetically improving fruit quality and shelf-life

A mutation in the tomato gene encoding UV-damaged DNA binding protein 1 (DDB1) delays fruit ripening and softening. Previous studies have established that DDB1 mutants produce fruit with high levels of carotenoids, flavonoids, and vitamin C. However, little was known about the effects of the mutation on fruit ripening processes. Yongsheng Liu at Hefei University of Technology, China, and colleagues showed that DDB1 is implicated in the synthesis of the fruit ripening hormone ethylene, the expression of ripening-associated genes, particularly during early fruit development, and cell wall-related genes. The slower ripening and increased firmness of DDB1 mutant tomatoes highlight this gene as a useful target for improving both the shelf-life and nutritional value of fleshy fruits.

## Introduction

As sessile organisms, plants have evolved fleshy fruits to disperse seeds by attracting animals, which consume them and release their seeds. Tomato (*Solanum lycopersicum*) is a model system for fleshy fruit ripening that, like many fruits, undergoes changes in color, aroma, texture, nutrient composition, and additional quality traits. These changes are coordinated by multiple internal and external factors, including the gaseous hormone ethylene, key transcription factors, epigenetic changes, and environmental stimuli, such as light and temperature^1,2^.

Several MADS-box transcription factors, including RIPENING-INHIBITOR (RIN), are essential for manifestation of the ripening phenotype^3^. While RIN is necessary for normal ripening, recent evidence indicates the mutant *rin* allele has repressor activity, resulting in ripening repression greater than null mutations^4,5^. RIN function is conserved across diverse fruiting species, as shown through transgenic repression in strawberry and banana^6,7^. Additional MADS-box proteins, TOMATO AGAMOUS-LIKE 1 (TAGL1), FRUITFULL 1 (FUL1), and FRUITFULL 2 (FUL2) interact with RIN to regulate fruit development and ripening^8–12^. Additional MADS-box and non-MADS-box ripening transcription factors, including COLORLESS NON-RIPENING (CNR), APETALA2a (AP2a), STAY-GREEN 1 (SGR1), MADS-box transcription factor MADS1, and HD-Zip homeobox protein HB-1^13-17^, have been characterized in tomato, suggesting multiple, and sometimes interacting, factors mediating genetic control of fruit ripening.

In addition to the accumulating knowledge pertaining to genetic regulation of ripening, intensive efforts have been made to improve fruit quality through targeted manipulation of softening, light responses^18–20^, and carotenoid pathway genes^21–23^. While extended shelf-life and increased phytonutrient levels are desired traits, genetic or postharvest approaches to delay ripening often negatively influence phytonutrients. For instance, mutations of RIN and CNR delay ripening but also confer reduced carotenoid accumulation^3,13^. Conversely, the tomato *high pigment 1* (*hp1*) mutant has attracted attention for its increased phytonutrients level^24,25^, including enhanced flavonoids, lycopene, and β-carotene accumulation, though the effect on additional aspects of fruit ripening has not been thoroughly examined. HP1 encodes a UV-DAMAGED DNA BINDING PROTEIN 1 (DDB1) homolog that associates with the CUL4-DDB1-DET1 E3 ligase complex to target substrates for 26S proteasomal degradation^26–28^. The stability of SlGLK2, a member of the MYB transcription factor superfamily exhibiting gradient longitudinal expression in tomato fruit, has been shown to be influenced by the CUL4-DDB1-DET1 complex, while its transcription in the *hp1* loss-of-function mutant is upregulated^29,30^. *Arabidopsis* DET1 was shown to function together with CCA1 and LHY as a transcriptional repressor^31^, suggesting a role for DDB1-DET1 E3 ligase complex in similar transcriptional regulation.

Here we used *S. lycopersicum* cv. Ailsa Craig (AC) and its nearly isogenic *hp1* mutant line to further characterize the influence of *DDB1* on fruit ripening. We show that in addition to previously reported effects on pigmentation, this gene influences additional ripening activities including texture and shelf-life suggesting a re-examination of its utility in modifying agriculturally important fleshy fruit traits.

## Results

### Defective *DDB1* mutation influences tomato fruit maturation and ripening initiation

**Fig. 1:**
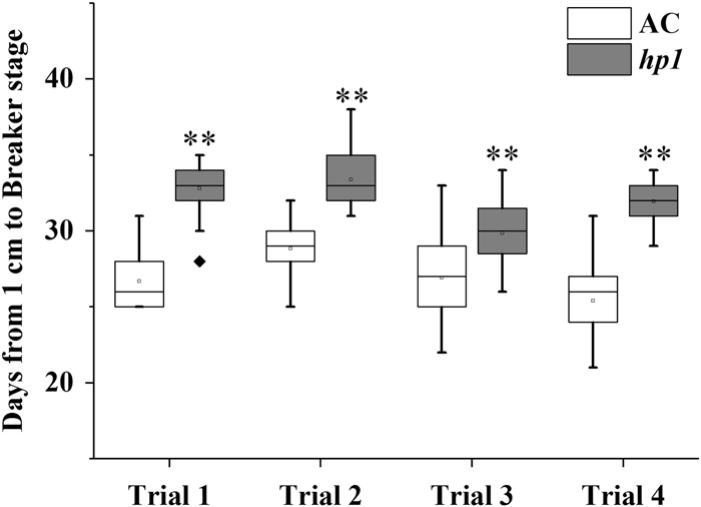
Days from 1 cm fruit to the breaker stage for wild-type tomato AC (Ailsa Craig) and *hp1* mutant. **P < 0.01

**Fig. 2:**
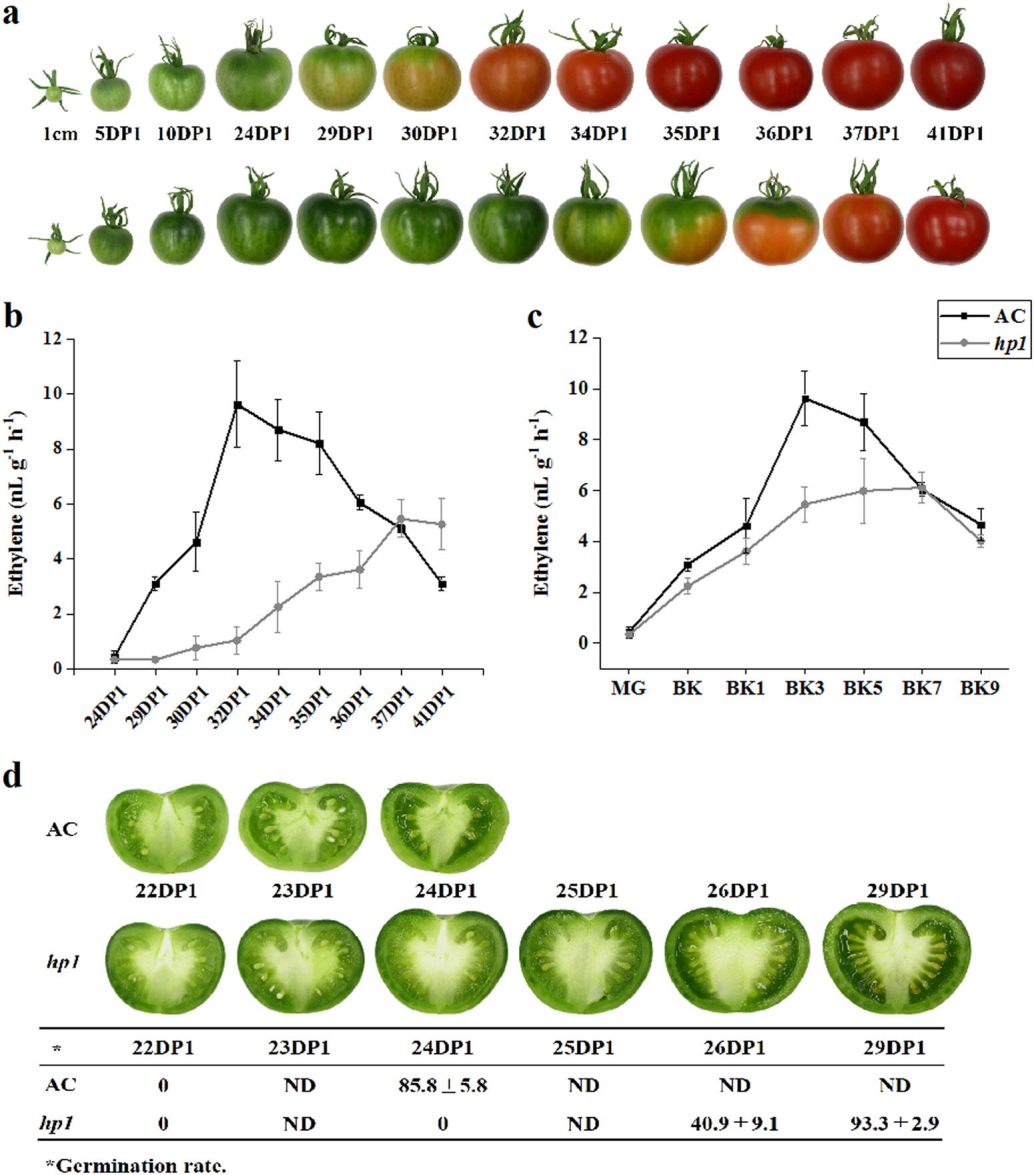
*hp1* mutation influences fruit development and ripening. **a** Upper and lower rows of pictures represent fruits throughout fruit development for AC and the *hp1* mutant, respectively. **b**, **c** Ethylene production of AC and *hp1* fruits based on 1 cm or breaker (BK) stage, respectively. DP1, days post 1 cm fruit. **d** Cross-sectioned AC and *hp1* fruits at different developmental stages and germination rates of seeds isolated from the corresponding fruits. Error bars indicate standard error.

The tomato *DDB1*-defective mutant, *hp1*, has been shown to present whole plant constitutive light responsiveness, including an enhanced accumulation of fruit carotenoids. This is due, at least in part, to increased plastid number and elevated carotenoid pathway gene expression^27,32^. Fruit development was monitored in the *hp1* mutant from 1 cm fruit (7 days post anthesis (DPA)) up to the initial ripening or breaker (BK) stage, showing that ripening initiation was delayed 4–5 days compared to the wild-type (WT) AC control (Figs. 1 and 2a). Consistent results were observed in plants grown in three additional trials with minor variation (Fig. 1) and *hp1* fruit showed an average of 4.7 days ripening delay. To further confirm delayed ripening initiation, ethylene production was measured at various developmental stages. Ethylene synthesis was also delayed in *hp1* fruit consistent with the delay in ripening initiation (Fig. 2b). Ethylene reached a peak at BK + 3 days and then gradually declined in AC control fruit, whereas in *hp1*, ethylene achieved a reduced plateau as compared to WT spanning BK + 3 (BK3) to BK + 7 days (BK7), then declined (Fig. 2c). In order to accurately harvest fruit at the mature green (MG) stage, we compared fruit gel liquidity and seed germination rate at different DPA pre-BK. *hp1* fruit reached MG slightly later than AC (Fig. 2d).

### *DDB1* loss of function impedes fruit softening

**Fig. 3:**
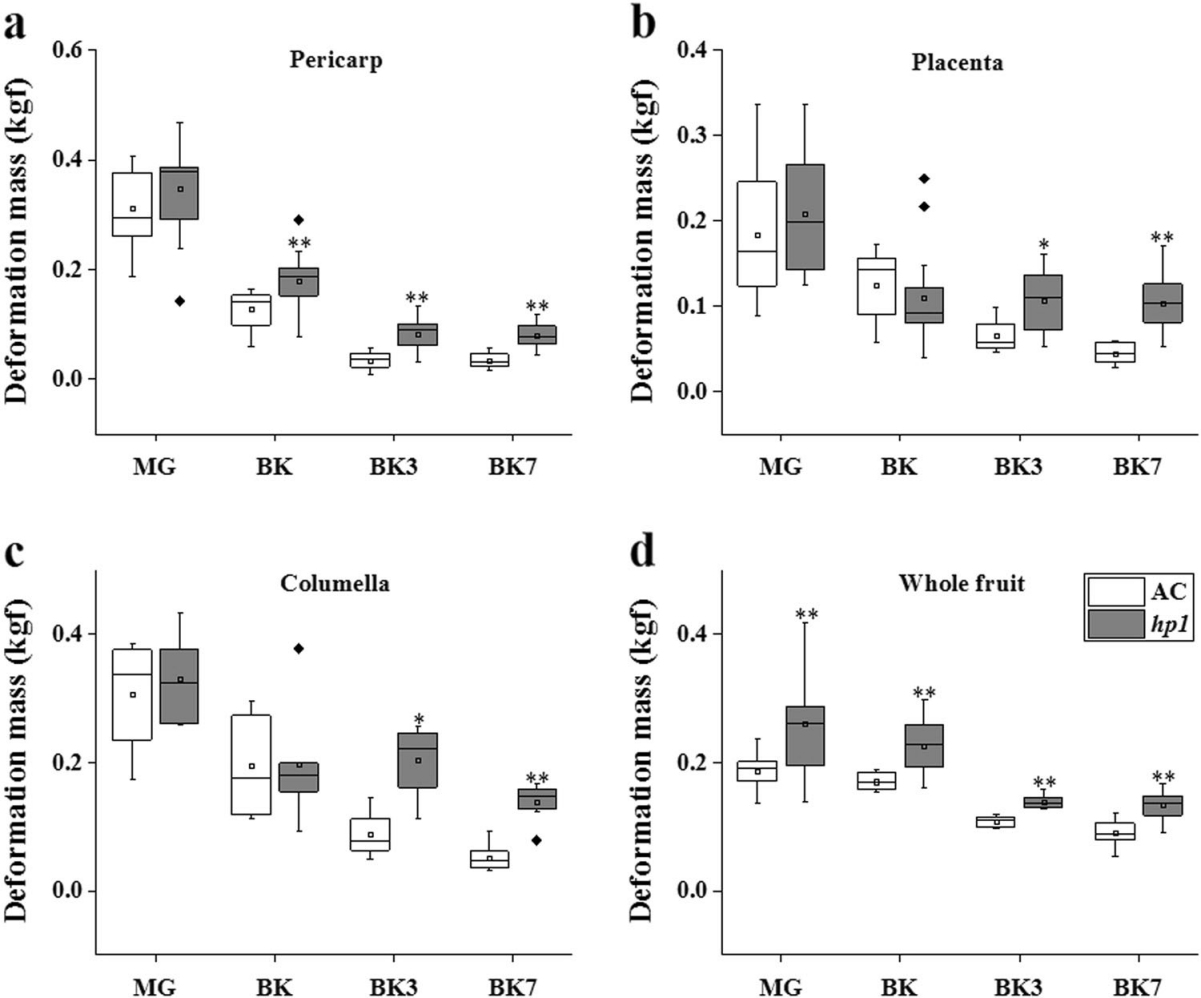
*hp1* mutation impedes fruit softening process. Deformation mass of AC and *hp1* fruits measured on pericarp (**a**), placenta (**b**), columella (**c**), and intact fruit (**d**), respectively. *0.01 < *P* < 0.05, **P < 0.01

Given that *hp1* delays tomato ripening initiation and ethylene production, we next asked whether it influences the key quality and postharvest trait of fruit softening. Fruit firmness was measured by independently quantifying resistance to mechanical deformation of the fruit pericarp, placenta, and columella tissue. There were no significant differences between *hp1* and AC at the MG stage, while at BK, BK3, and BK7, *hp1* fruits exhibited higher pericarp deformation resistance (Fig. 3a), indicating reduced softening during ripening. Differences in placenta and columella firmness were significant at the BK3 and BK7 stages, suggesting effects later than in pericarp in these tissues (Fig. 3b, c). Furthermore, intact *hp1* fruits exhibited higher deformation resistance at all developmental stages examined, including MG, as compared with AC (Fig. 3d), implying additional textural influence of the mutation at pre-ripening stages.

### *DDB1* influences ethylene synthesis and signaling at the transcriptional level

**Fig. 4:**
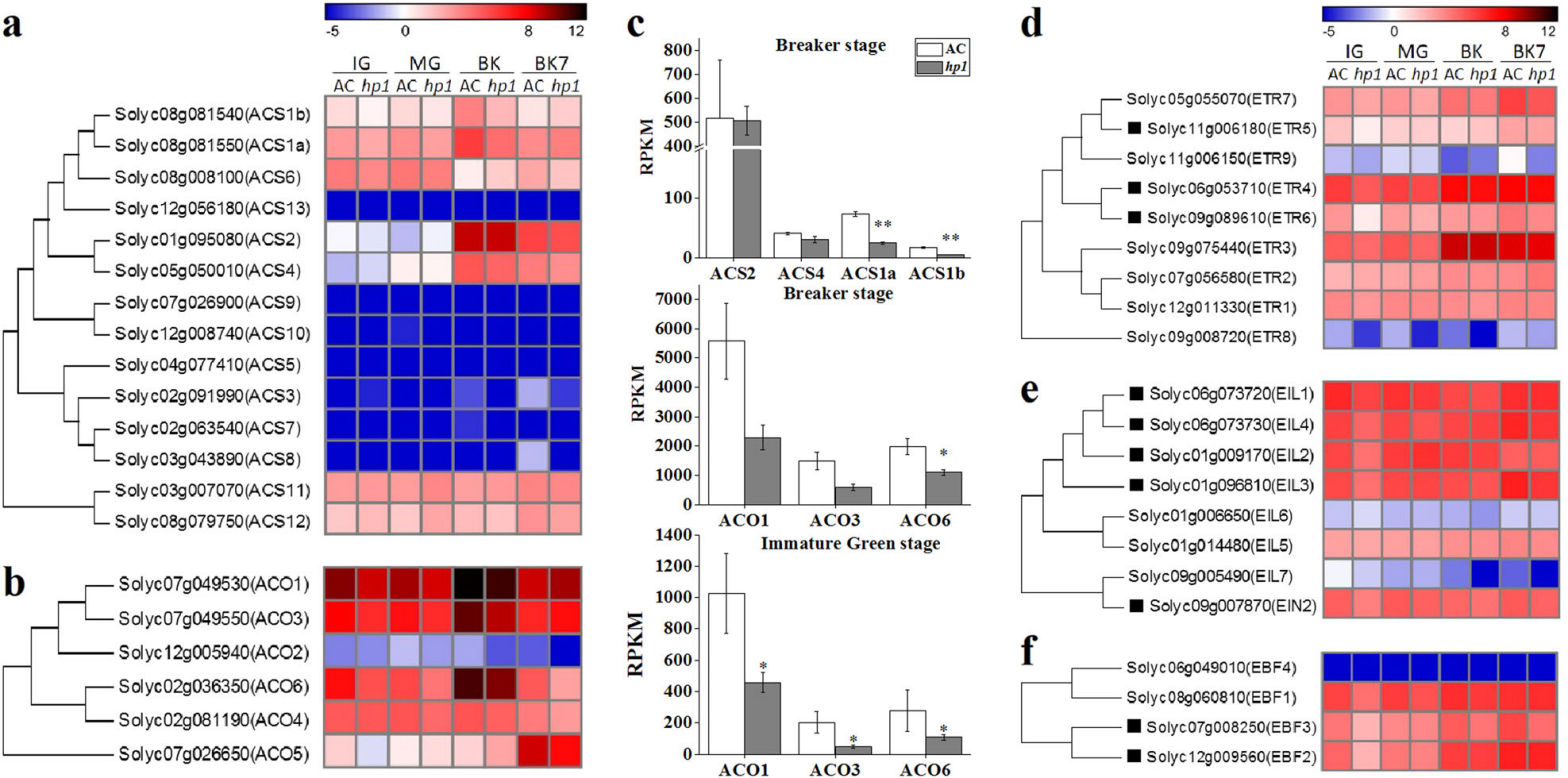
*hp1* mutation influences the expression of ethylene biosynthesis and signaling genes. **a**, **b** Phylogenetic tree and expression heatmap of ethylene biosynthesis genes. **c**. Expression of *ACS1a*, *ACS1b*, *ACS2*, *ACS4*, *ACO1*, *ACO3*, and *ACO6* at the breaker (BK) or immature green (IG) stage. *0.01 < *P* < 0.05. **P < 0.01. **d**–**f** Phylogenetic tree and expression heatmap of ethylene receptor genes (**d**), ethylene insensitive 3 (EIN3) transcription factors homologous (**e**), and EIN3 binding F-box genes (**f**). Genes marked with black rectangle are downregulated in *hp1* at the IG stage. Error bars indicate standard error

To better understand the mechanism behind the *hp1* phenotype and thus more fully understand DDB1 function, transcriptome analysis was performed on *hp1* and nearly isogenic AC control fruit tissues (Supplemental Table 1; Supplemental Dataset 1). It has been proposed that ethylene production is mediated by system 1, or repressive, ethylene prior to the MG stage, while system 2 ripening promotive ethylene operates during later fruit development and ripening^33^. At the immature green (IG) stage, expression levels of the ACC oxidase genes *ACO1*, *ACO3*, and *ACO6* were lower in *hp1* than in AC (Fig. 4a–c), suggesting that DDB1 loss of function may initially affect system 1 ethylene production, consistent with our observations of delayed ethylene production during fruit development. At the BK stage, expression of ACC synthases *ACS1a* and *ACS1b* were significantly lower in the *hp1* mutant, while normally limiting enzymes in ethylene biosynthesis *ACS2* and *ACS4* (i.e. those most strongly induced during ripening) remained unchanged in *hp1*. Expression of *ACO1*, *ACO3*, and *ACO6* was slightly lower in the *hp1* mutant at BK stage.

We next examined expression of genes involved in ethylene perception and signaling. At the IG stage, transcripts of multiple ethylene signaling components were downregulated in *hp1* (Fig. 4d–f), including ethylene receptors (*ETR5* and *ETR6*), ethylene insensitive 3 transcription factors homologous (*EIL2-4*), and ethylene insensitive 3 binding F-box proteins (*EBF2* and *EBF3*). Given the repressive role of ethylene receptors in mediating downstream signaling in the presence of ethylene^34,35^, these fruit might be anticipated to be more ethylene-sensitive than their WT counterparts. Furthermore, multiple ethylene responsive factors were also found to be downregulated in the *hp1* mutant at either IG or MG stages (Supplemental Fig. 1). As such, both promotive ethylene signaling functions including those encoded by *EILs* and repressive functions as encoded by the *ETR* receptors were downregulated in *hp1*. Given the fact that *ERT3* and *ETR4* are the predominantly expressed receptors in maturing tomato fruit, and that they are not altered by *hp1*, the transcriptome changes of ethylene signaling genes would be consistent with reduced ethylene response and the observed inhibited ripening phenotypes.

### Defective *DDB1* influences ripening-regulated gene expression

**Fig. 5:**
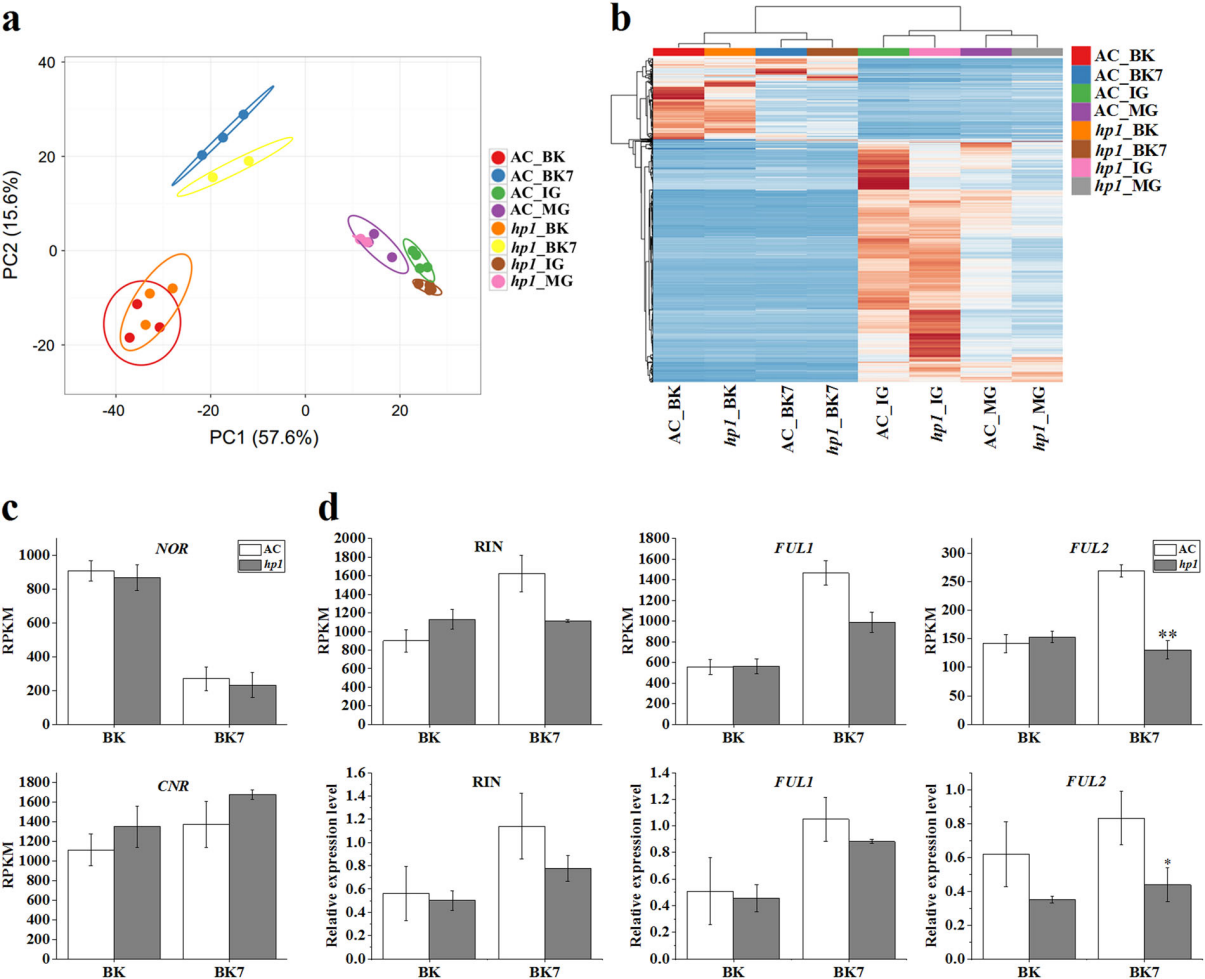
*hp1* mutation influences fruit ripening by affecting ripening-related gene expression. **a** PCA analysis of AC and *hp1* fruit samples using expression data (RPKM) of ripening-associated genes. **b** Heatmap analysis of AC and *hp1* fruit samples using expression data (RPKM) of ripening-associated genes. **c** Expression (RPKM) of *NOR* and *CNR* at BK and BK + 7 stages. **d** Expression (RPKM) and relative expression levels of *RIN*, *FUL1* and *FUL2* at BK and BK + 7 stages determined by RNA-Seq (upper panel) and qRT-PCR (lower panel), respectively. *0.01 < *P* < 0.05, **P < 0.01. Error bars indicate standard error

Differential gene expression analysis between IG and BK was performed (Supplemental Dataset 2). Differentially expressed genes (DEGs, adjusted *P* < 0.05, ratio >2 or <0.5) between AC IG stage and AC BK stage were defined as ripening-associated genes (Supplemental Table 2). Principal component analysis (PCA) of ripening-associated transcripts was performed to describe the relationships among different developmental stages. IG, MG, BK, and BK7 stages of AC were separated in PCA with a predominance of IG and MG localizing to the first principal component (PC1) and separation of BK and BK7 in PC2 (Fig. 5a).

To better visualize alteration of ripening-associated genes, a heatmap was generated. As shown in Fig. 5b, clusters of up- or downregulated transcripts were identified in *hp1* as compared to WT at all stages examined, confirming the effects of DDB1 across all fruit development and ripening stages tested. A large set of ripening-associated genes showed distinct patterns in *hp1* at the IG stage, indicating an especially large effect during early fruit development. Among previously described ripening-related genes, expression of the *NOR* and *CNR* transcription factors displayed similar expression patterns in AC and *hp1* at the BK and BK7 stages (Fig. 5c). Expression of *RIN* and *FUL1* were not significantly different at either BK or BK7 stage, while reduced expression of *FUL2* was observed in *hp1* at BK7 stage, which was confirmed by qRT-PCR analysis (Fig. 5d).

### The *hp1* mutation alters cell wall-related gene expression

**Fig. 6:**
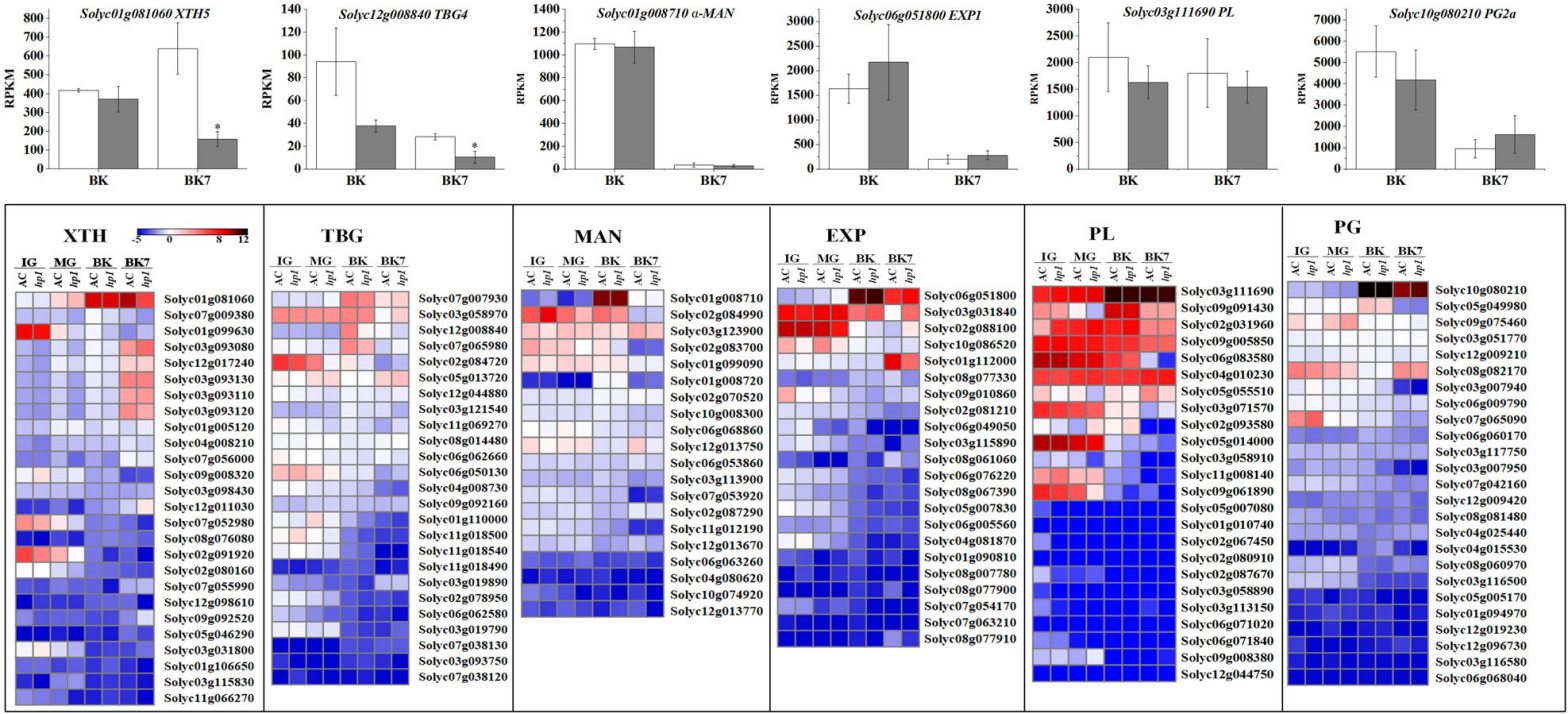
Expression (RPKM) (upper panel) and heatmap (lower panel) of genes encoding cell wall-modifying enzymes. *0.01 < *P* < 0.05. Error bars indicate standard error

Ripening-related softening results in part from remodeling of cell wall components. To better understand the delayed softening of *hp1* fruit, the expression of cell wall-related genes was examined. Compared to AC controls, expression of *xyloglucan endotransglucosylase/hydrolase 5* (*XTH5*) and *β-galactosidase 4* (*TBG4*) was downregulated in *hp1* at BK7, while expression of *α-mannose* (*α-MAN*), *expansin 1* (*EXP1*)*, pectate lyase* (*PL*), and *polygalacturonase 2a* (*PG2a*) was unchanged. Each of these cell wall-modifying enzyme classes is encoded by a large gene family and all have been associated to greater or lesser degrees with changes in maturing fruit texture. We examined the annotations and expression of homologous genes (Fig. 6). *XTH5*, *α-MAN*, *EXP1*, *PL*, and *PG2a* are the highest expressed respective family members at the BK stage. Interestingly, *TBG4* is exceeded in expression by two members of the TBG family. Transgenic downregulation of *TBG4* was previously shown to result in decreased fruit softening^36,37^. Furthermore, multiple additional cell wall genes were found downregulated in *hp1* at the MG stage, including *Solyc07g009380*, *Solyc02g091920*, *Solyc02g080160*, and *Solyc03g031800*, which belong to XTH gene family, and *Solyc11g069270*, *Solyc06g062660*, and *Solyc02g078950*, which belong to the TBG gene family. These previously uncharacterized genes emerge as additional candidates for ripening-related textural changes as a result of this analysis.

## Discussion

### *DDB1/hp1* influences both fruit nutritional quality and firmness

Fruit ripening is a process modulated by complex regulatory networks. Multiple ripening or pigmentation regulators have been identified by positional cloning of the known mutants^3,13,27,38–40^, or through functional characterization using reverse genetics approaches^8,9,15,16,41^. RIN has been characterized as one of the central ripening regulators and the *rin* mutant exhibits significant delayed ripening, which is beneficial for prolonged shelf-life during the postharvest stage; however, the *rin* mutant also shows reduced phytonutrient levels^3,12,42,43^. We note that suppression of a pectate lyase, *PL*, was reported to result in firmer fruit without affecting other aspects of ripening^20,44^, suggesting that global ripening and textural changes can be uncoupled. According to previous studies, *hp1* mutant fruit exhibit increased accumulation of chlorophyll and carotenoids at the MG and red ripe stages, respectively, due to increased plastid number/size and elevated expression of carotenoid biosynthesis genes^27,32^. Here we reveal that expression of neither *RIN* nor *PL* is significantly altered in the *hp1* mutant. As such, these key regulators of ripening and softening do not appear to be the genetic means through which DDB1 exerts its effects on ripening.

Tomato DDB1 clearly influences ripening initiation and softening as revealed through characterization of the *hp1* mutation for ripening phenotypes beyond those previously characterized (Figs. 1–3). Another *HP1* (*DDB1*) mutant allele, *hp1*^*w*^, is available and exhibited a similar delay in ripening initiation (5.2 days) compared to its corresponding WT GT (Supplemental Fig. 2) and consistent with the initial *hp1* observations, indicating a significant contribution of tomato DDB1 in the initiation and manifestation of ripening phenotypes. Indeed, *DDB1* expression exhibits a significant increase at BK (Reads Per Kilobase per Million mapped reads, RPKM 31.69) as compared to its expression at IG stage (RPKM 20.48, fold-change 1.55) in AC (Supplemental Fig. 3), consistent with a previous report^45^. Moreover, *hp1* impaired expression of *FUL2*, *XTH5*, and *TBG4* (Figs. 5 and 6) likely contributing to the observed textural changes. FUL2, a MADS-box transcription factor, is involved in regulating cell wall modification gene expression and acts redundantly with FUL1 in controlling fruit ripening^11^ as part of a complex with RIN^10^. Transcripts of *XTH5*, the mostly highly expressed XTH at BK (Fig. 6), is expressed at high levels throughout ripening and XTH5 protein has been shown to degrade xyloglucan, a major component of the primary cell wall of dicotyledons^46^. Tomato β-galactosidase TBG4 degrades β-(1, 4)-galactan in the pericarp cell wall, and downregulation of *TBG4* resulted in decreased fruit softening in transgenic fruit^36,37^. In short, the *hp1* mutation exhibits decreased ethylene production and delayed ripening in addition to increased fruit firmness likely due at least in part to alteration of transcription factors such as FUL2 and cell wall-modifying enzymes including TBG4, which are known to modulate these fruit phenotypes, respectively.

### The effects of DDB1 on plastids and photosynthesis are predominantly in young fruit tissues

Chlorophylls are the major photosynthetic pigments, where they act to harvest solar energy, whereas carotenoids function as accessory pigments in light absorption^47^. Xanthophyll cycle carotenoids, violaxanthin, antheraxanthin, and zeathaxin, also play a protective role in photosynthesis by dissipating excess energy^48^. Carotenoid biosynthesis genes, such as *GGPS1*, *PSY1*, *PSY2*, *LCYE*, *CHYB1*, and *CHYB2*, were found to be upregulated in the *hp1* mutant at the IG and MG stages, but not at the BK or BK7 stages (Supplemental Table 3). Most carotenoid biosynthesis genes, including the ethylene-regulated rate-limiting enzyme encoding fruit PSY (*PSY1*), showed no significant difference in expression at the onset of fruit ripening (BK). *ZEP* and *VDE1* are genes involved in the xanthophyll cycle of carotogenesis and *VDE1* was significantly upregulated at IG, MG, and BK7, while *ZEP* was slightly upregulated at IG and BK7 (Supplemental Table 3). These results suggest that the *hp1* mutation influences the expression of carotenoid biosynthesis genes primarily in the early photosynthetic fruit stages of pre-ripening fruit, which are also typified by elevated plastid numbers^27^.

Altered pathway analysis using Plant MetGenMAP^49^ showed that photosynthesis-associated pathways changed in *hp1* throughout fruit development and the related genes were generally upregulated (Supplemental Table 4). For example, genes coding ribulose bisphosphate carboxylase small subunit and glyceraldehyde-3-phosphate dehydrogenase involved in carbon fixation via the Calvin-Benson-Bassham cycle were significantly upregulated in *hp1*, especially at the IG stage. A previous study suggested that carbon fixation via Calvin-Benson-Bassham cycle is modulated by HP1 and can lead to improving secondary metabolites^50^. Another study showed that tomato fruit photosynthetic rates have little effect on fruit ripening and primary metabolism, but may more substantively influence seed development^51^. This result is consistent with our observed effect on delayed seed maturation detected in the *hp1* mutant (Fig. 2d).

### *DDB1* influences ethylene signaling at early developmental stages

Both ethylene and abscisic acid (ABA) play a role in regulating fruit ripening and ABA was suggested to act upstream of ethylene^52,53^. DDB1 functions as part of the CUL4-DDB1-DET1 E3 ligase complex that targets substrates for degradation. Its *Arabidopsis* counterpart, AtDDB1, has been shown to be associated with the AtCOP1-SPA1 complex^54^. AtCOP1, which is the central regulator of light signaling, directly targets the F-box protein AtEBF1 and AtEBF2 for ubiquitination and degradation to stabilize AtEIN3. In *Arabidopsis*, a mutation in AtCOP1 leads to reduced expression of *AtEIN3*, a primary transcription factor mediating multiple ethylene responses^55^. Our study found that in *hp1*, expression levels of *AtEIN3* orthologs, *EIL2*, *EIL3*, and *EIL4*, were downregulated at the IG stage, and reduced levels of *EBF2*, *EBF3* transcription factors, in addition to the *ETR5* and *ETR6* ethylene receptors, were also observed (Fig. 4b–d). In brief, DDB1 influences both ethylene biosynthesis and signaling (Fig. 4 and Supplemental Fig. 1) contributing the observed reduction in ethylene evolution and delayed ripening, but not likely through the activity of important regulators of ripening and softening, such as RIN and PL. While the increased pigmentation and nutritional quality of *hp1* fruit has made it an attractive target for breeding, pleiotropic whole plant effects have limited its use. Demonstration that loss of DDB1 function through renewed *hp1* mutant analysis may provide new incentives to deploy this allele for enhanced fruit characteristics beyond pigmentation, possibly through fruit-specific repression, as described previously^18^.

## Materials and methods

### Plant materials

The *hp1* mutants “AC” (*hp1*/*hp1*) (LA3538) and its nearly isogenic WT “AC” (+/+) (LA2838A) were kindly provided by Tomato Genetics Resource Center, Davis, CA, USA. Plants were grown under greenhouse conditions with a light (16 h, 27 °C) and dark (8 h, 19 °C) cycle at the Boyce Thompson Institute, Ithaca, NY, USA. Tomato fruits at 1 cm diameter were tagged after 7–8 DPA. Fruits of various developmental stages described in the text were collected for further analysis.

### Ethylene measurement

Tomato fruits were harvested at indicated stages and placed in 250 mL open jars overnight at room temperature to reduce harvest stress. The jars were then sealed for 4 h and 1 mL headspace air samples were then taken and injected into a gas chromatograph (Agilent 6850 Series II Network GC System) equipped with a flame ionization detector and an activated alumina column. Ethylene concentrations were calculated using an ethylene standard of known concentration, and normalized by fruit mass^9^.

### Fruit firmness measurement

Firmness testing was adopted from a previous report^56^ with slight modifications. Intact fruit compression measurements were made by recording a force-deformation curve using Instron Force Transducer (2519 series S-beam, Instron) with flat probe, 1 mm s^−1^ loading speed and followed by 10 s stress relaxation. Force was recorded at 0.04 s intervals and maximum values were used as an estimation of the fruit firmness with two or three compressions against locule per fruit. Fruit were then sliced into 1 cm-thick slices along the equator using a double-blade slicer, and the pericarp, placenta, and columella deformation mass of the center slice was analyzed using a flat-faced cylinder probe with the same force-deformation method as used in intact fruit compression measurement. Six fruits per stage were measured and a *t*-test was used for statistical analysis.

### Quantitative real-time PCR analysis

Quantitative real-time PCR was performed on the ABI PRISM 7900HT sequence detection system using Power SYBR Green 1-Step kit (Applied Biosystems) following the manufacturer’s instructions. A minimum of three biological replicates were performed for each sample and a tomato *TIP41* gene was used as internal standard^57^. *TIP41* and gene-specific primers are listed in Supplemental Table 5.

### Transcriptome sequencing and data processing

Fruits from three biological replicates were harvested at IG (10 days post 1 cm diameter (DP1)), MG (24 and 29 DP1), BK (29 and 34 DP1), and BK7 (36 and 41 DP1) for AC and *hp1*, respectively. For analysis of different developmental stages AC and *hp1*, fruits were equally cross-sectioned into three parts: shoulder, middle, and bottom. The middle section was discarded and pericarp tissues from shoulder and bottom parts were kept in −80 °C freezer until use. The shoulder sections are currently being analyzed for a separate purpose and bottom parts were used in this study since ripening process was more uniform in this section. Total RNA extracted from bottom section pericarp tissue was used for construction of strand-specific RNA-Seq libraries as described^58^. Two or three biological replicates were sequenced for each sample using Illumina HiSeq2000 sequencing system following manufacturer’s instruction. RNA-Seq reads were first cleaned by removing adaptor, polyA/T, rRNA, and plant virus sequences. The resulting reads were mapped to tomato genome by using HISAT allowing one segment mismatch^59^. For each tomato gene model, raw counts of mapped read were then derived and normalized to RPKM.

### DEGs and significantly changed pathways analysis

DEGs between indicated comparisons were identified using DESeq package, with a cutoff of adjusted *P* < 0.05 and ratio >2 or <0.5. DEGs were then fed to Plant MetGenMAP^49^ to identify significantly changed pathways with false discovery rate correction and a cutoff *P* value of 0.05.

### PCA, heatmap, and phylogenetic analysis

PCA and heatmap were performed using ClustVis^60^. Phylogenetic analysis was performed as previously described^61^.

### Accession numbers

Transcriptome sequencing reads were submitted to the National Center for Biotechnology Information Sequence Read Archive (accession number SRP125614).

## Electronic supplementary material

Supplemental files

## References

[1] Klee HJ, Giovannoni JJ (2011). Genetics and control of tomato fruit ripening and quality attributes. Annu. Rev. Genet..

[2] Giovannoni J, Nguyen C, Ampofo B, Zhong S, Fei Z (2017). The epigenome and transcriptional dynamics of fruit ripening. Annu. Rev. Plant Biol..

[3] Vrebalov J (2002). A MADS-box gene necessary for fruit ripening at the tomato *ripening-inhibitor (rin)* locus. Science.

[4] Ito Y (2017). Re-evaluation of the rin mutation and the role of RIN in the induction of tomato ripening. Nat. Plants.

[5] Li S (2017). The RIN-MC fusion of MADS-box transcription factors has transcriptional activity. Plant Physiol..

[6] Seymour GB (2011). A SEPALLATA gene is involved in the development and ripening of strawberry (*Fragaria x ananassa* Duch.) fruit, a non-climacteric tissue. J. Exp. Bot..

[7] Elitzur T (2016). Banana MaMADS transcription factors are necessary for fruit ripening and molecular tools to promote shelf-life and food security. Plant Physiol..

[8] Itkin M (2009). TOMATO AGAMOUS‐LIKE 1 is a component of the fruit ripening regulatory network. Plant J..

[9] Vrebalov J (2009). Fleshy fruit expansion and ripening are regulated by the Tomato SHATTERPROOF gene TAGL1. Plant Cell.

[10] Martel C, Vrebalov J, Tafelmeyer P, Giovannoni JJ (2011). The tomato MADS-box transcription factor ripening inhibitor interacts with promoters involved in numerous ripening processes in a colorless nonripening-dependent manner. Plant Physiol..

[11] Bemer M (2012). The tomato FRUITFULL homologs TDR4/FUL1 and MBP7/FUL2 regulate ethylene-independent aspects of fruit ripening. Plant Cell.

[12] Fujisawa M (2014). Transcriptional regulation of fruit ripening by tomato FRUITFULL homologs and associated MADS box proteins. Plant Cell.

[13] Manning K (2006). A naturally occurring epigenetic mutation in a gene encoding an SBP-box transcription factor inhibits tomato fruit ripening. Nat. Genet..

[14] Lin Z (2008). A tomato HD-Zip homeobox protein, LeHB-1, plays an important role in floral organogenesis and ripening. Plant J..

[15] Chung MY (2010). A tomato (*Solanum lycopersicum*) APETALA2/ERF gene, SlAP2a, is a negative regulator of fruit ripening. Plant J..

[16] Karlova R (2011). Transcriptome and metabolite profiling show that APETALA2a is a major regulator of tomato fruit ripening. Plant Cell.

[17] Dong T (2013). A tomato MADS-box transcription factor, SlMADS1, acts as a negative regulator of fruit ripening. Plant Physiol..

[18] Davuluri GR (2005). Fruit-specific RNAi-mediated suppression of DET1 enhances carotenoid and flavonoid content in tomatoes. Nat. Biotechnol..

[19] Wang S (2008). Altered plastid levels and potential for improved fruit nutrient content by downregulation of the tomato DDB1-interacting protein CUL4. Plant J..

[20] Uluisik S (2016). Genetic improvement of tomato by targeted control of fruit softening. Nat. Biotechnol..

[21] Fraser PD (2007). Manipulation of phytoene levels in tomato fruit: effects on isoprenoids, plastids, and intermediary metabolism. Plant Cell.

[22] McQuinn RP, Giovannoni JJ, Pogson BJ (2015). More than meets the eye: from carotenoid biosynthesis, to new insights into apocarotenoid signaling. Curr. Opin. Plant. Biol..

[23] McQuinn R, Wong B, Giovannoni J (2017). AtPDS overexpression in tomato: exposing unique patterns of carotenoid self-regulation and an alternative strategy for the enhancement of fruit carotenoid content. Plant Biotechnol. J..

[24] Rohrmann J (2011). Combined transcription factor profiling, microarray analysis and metabolite profiling reveals the transcriptional control of metabolic shifts occurring during tomato fruit development. Plant J..

[25] Kilambi HV, Kumar R, Sharma R, Sreelakshmi Y (2013). Chromoplast-specific carotenoid-associated protein appears to be important for enhanced accumulation of carotenoids in hp1 tomato fruits. Plant Physiol..

[26] Lieberman M, Segev O, Gilboa N, Lalazar A, Levin I (2004). The tomato homolog of the gene encoding UV-damaged DNA binding protein 1 (DDB1) underlined as the gene that causes the *high pigment-1* mutant phenotype. Theor. Appl. Genet..

[27] Liu Y (2004). Manipulation of light signal transduction as a means of modifying fruit nutritional quality in tomato. Proc. Natl Acad. Sci. USA.

[28] Tang X (2016). Ubiquitin-conjugated degradation of golden 2-like transcription factor is mediated by CUL4-DDB1-based E3 ligase complex in tomato. New Phytol..

[29] Nadakuduti SS, Holdsworth WL, Klein CL, Barry CS (2014). KNOX genes influence a gradient of fruit chloroplast development through regulation of GOLDEN2-LIKE expression in tomato. Plant J..

[30] Nguyen CV (2014). Tomato GOLDEN2-LIKE transcription factors reveal molecular gradients that function during fruit development and ripening. Plant Cell.

[31] Lau OS (2011). Interaction of Arabidopsis DET1 with CCA1 and LHY in mediating transcriptional repression in the plant circadian clock. Mol. Cell.

[32] Cookson PJ (2003). Increases in cell elongation, plastid compartment size and phytoene synthase activity underlie the phenotype of the *high pigment-1* mutant of tomato. Planta.

[33] McMurchie E, McGlasson W, Eaks I (1972). Treatment of fruit with propylene gives information about the biogenesis of ethylene. Nature.

[34] Tieman DM, Taylor MG, Ciardi JA, Klee HJ (2000). The tomato ethylene receptors NR and LeETR4 are negative regulators of ethylene response and exhibit functional compensation within a multigene family. Proc. Natl Acad. Sci. USA.

[35] Kevany BM, Tieman DM, Taylor MG, Cin VD, Klee HJ (2007). Ethylene receptor degradation controls the timing of ripening in tomato fruit. Plant J..

[36] Smith DL, Abbott JA, Gross KC (2002). Down-regulation of tomato β-galactosidase 4 results in decreased fruit softening. Plant Physiol..

[37] Eda M, Matsumoto T, Ishimaru M, Tada T (2016). Structural and functional analysis of tomato β-galactosidase 4: insight into the substrate specificity of the fruit softening-related enzyme. Plant J..

[38] Mustilli AC, Fenzi F, Ciliento R, Alfano F, Bowler C (1999). Phenotype of the tomato *high pigment-2* mutant is caused by a mutation in the tomato homolog of DEETIOLATED1. Plant Cell.

[39] Barry CS, Giovannoni JJ (2006). Ripening in the tomato Green-ripe mutant is inhibited by ectopic expression of a protein that disrupts ethylene signaling. Proc. Natl Acad. Sci. USA.

[40] Barry CS, McQuinn RP, Chung MY, Besuden A, Giovannoni JJ (2008). Amino acid substitutions in homologs of the STAY-GREEN protein are responsible for the green-flesh and chlorophyll retainer mutations of tomato and pepper. Plant Physiol..

[41] Lee JM (2012). Combined transcriptome, genetic diversity and metabolite profiling in tomato fruit reveals that the ethylene response factor SlERF6 plays an important role in ripening and carotenoid accumulation. Plant J..

[42] Fujisawa M, Nakano T, Shima Y, Ito Y (2013). A large-scale identification of direct targets of the tomato MADS box transcription factor RIPENING INHIBITOR reveals the regulation of fruit ripening. Plant Cell.

[43] Fujisawa M (2012). Direct targets of the tomato-ripening regulator RIN identified by transcriptome and chromatin immunoprecipitation analyses. Planta.

[44] Yang L (2017). Silencing of SlPL (Solyc03g111690), which encodes a pectate lyase in tomato, confers enhanced fruit firmness, prolonged shelf-life, and reduced susceptibility to gray mold. Plant Biotechnol. J..

[45] Li Y (2015). Tomato MBD5, a methyl CpG binding domain protein, physically interacting with UV-damaged DNA binding protein-1, functions in multiple processes. New Phytol..

[46] Saladié M, Rose JK, Cosgrove DJ, Catalá C (2006). Characterization of a new xyloglucan endotransglucosylase/hydrolase (XTH) from ripening tomato fruit and implications for the diverse modes of enzymic action. Plant J..

[47] Green B, Durnford D (1996). The chlorophyll-carotenoid proteins of oxygenic photosynthesis. Annu. Rev. Plant Biol..

[48] Demmig-Adams B, Adams WW (1996). The role of xanthophyll cycle carotenoids in the protection of photosynthesis. Trends Plant Sci..

[49] Joung JG (2009). Plant MetGenMAP: an integrative analysis system for plant systems biology. Plant Physiol..

[50] Enfissi EMA (2010). Integrative transcript and metabolite analysis of nutritionally enhanced DE-ETIOLATED1 downregulated tomato fruit. Plant Cell.

[51] Lytovchenko A (2011). Tomato fruit photosynthesis is seemingly unimportant in primary metabolism and ripening but plays a considerable role in seed development. Plant Physiol..

[52] Ji K (2014). SlNCED1 and SlCYP707A2: key genes involved in ABA metabolism during tomato fruit ripening. J. Exp. Bot..

[53] Leng P, Yuan B, Guo Y, Chen P (2014). The role of abscisic acid in fruit ripening and responses to abiotic stress. J. Exp. Bot..

[54] Chen H (2010). Arabidopsis CULLIN4-damaged DNA binding protein 1 interacts with CONSTITUTIVELY PHOTOMORPHOGENIC1-SUPPRESSOR OF PHYA complexes to regulate photomorphogenesis and flowering time. Plant Cell.

[55] Shi H (2015). Seedlings transduce the depth and mechanical pressure of covering soil using COP1 and ethylene to regulate EBF1/EBF2 for soil emergence. Curr. Biol..

[56] Wu T, Abbott JA (2002). Firmness and force relaxation characteristics of tomatoes stored intact or as slices. Postharvest Biol. Technol..

[57] Expósito-Rodríguez M, Borges AA, Borges-Pérez A, Pérez JA (2008). Selection of internal control genes for quantitative real-time RT-PCR studies during tomato development process. BMC Plant Biol..

[58] Zhong S (2011). High-throughput illumina strand-specific RNA sequencing library preparation. Cold Spring Harb. Protoc..

[59] Kim D, Langmead B, Salzberg SL (2015). HISAT: a fast spliced aligner with low memory requirements. Nat. Methods.

[60] Metsalu T, Vilo J (2015). ClustVis: a web tool for visualizing clustering of multivariate data using principal component analysis and heatmap. Nucleic Acids Res..

[61] Hall BG (2013). Building phylogenetic trees from molecular data with MEGA. Mol. Biol. Evol..

