# Supplementary material for: The tomato HIGH PIGMENT1/DAMAGED DNA BINDING PROTEIN 1 gene contributes to regulation of fruit ripening
Source: Hortic Res. 2019 Feb 1;6:15. doi: 10.1038/s41438-018-0093-3 (PMC6355878; doi:10.1038/s41438-018-0093-3)
Supplement: Supplementary file 1 — Supplemental files [file 41438_2018_93_MOESM1_ESM.pdf]

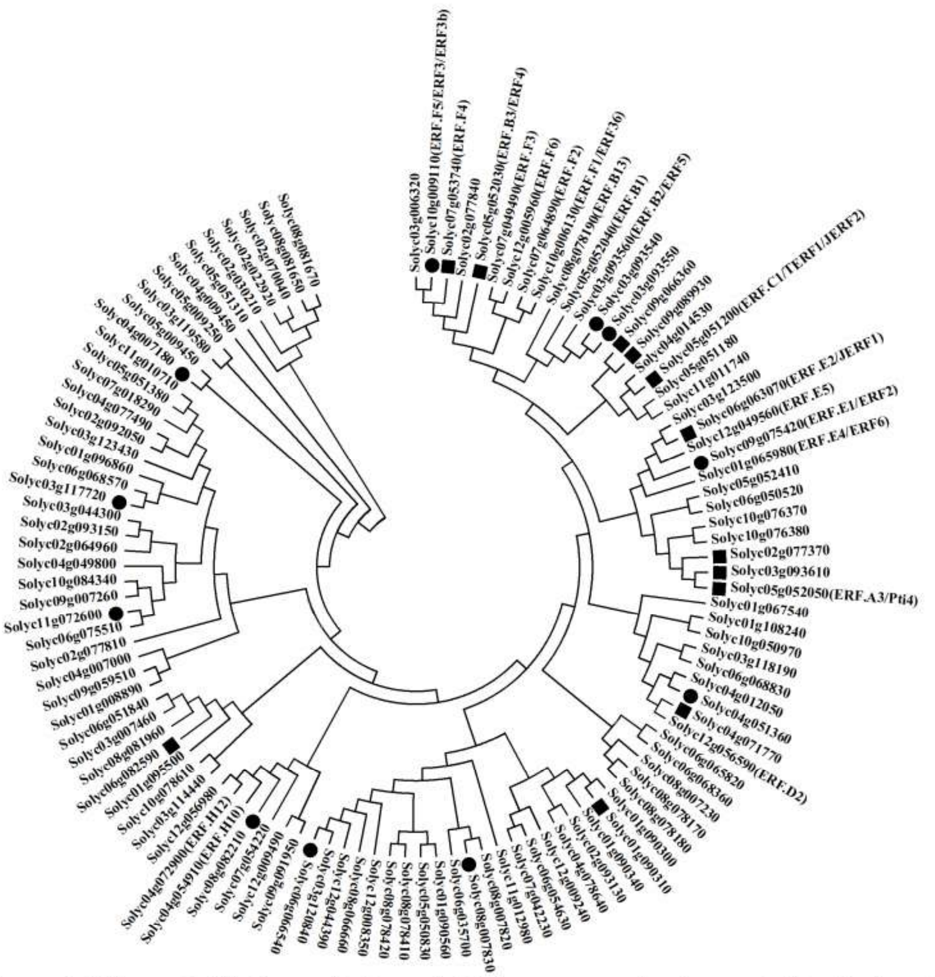

Supplemental Figure 1. Phylogenetic tree of ethylene responsive transcription factors in tomato. Genes marked with black rectangle or black circle are DEGs (downregulated) in *hp1* at IG or MG, respectively.

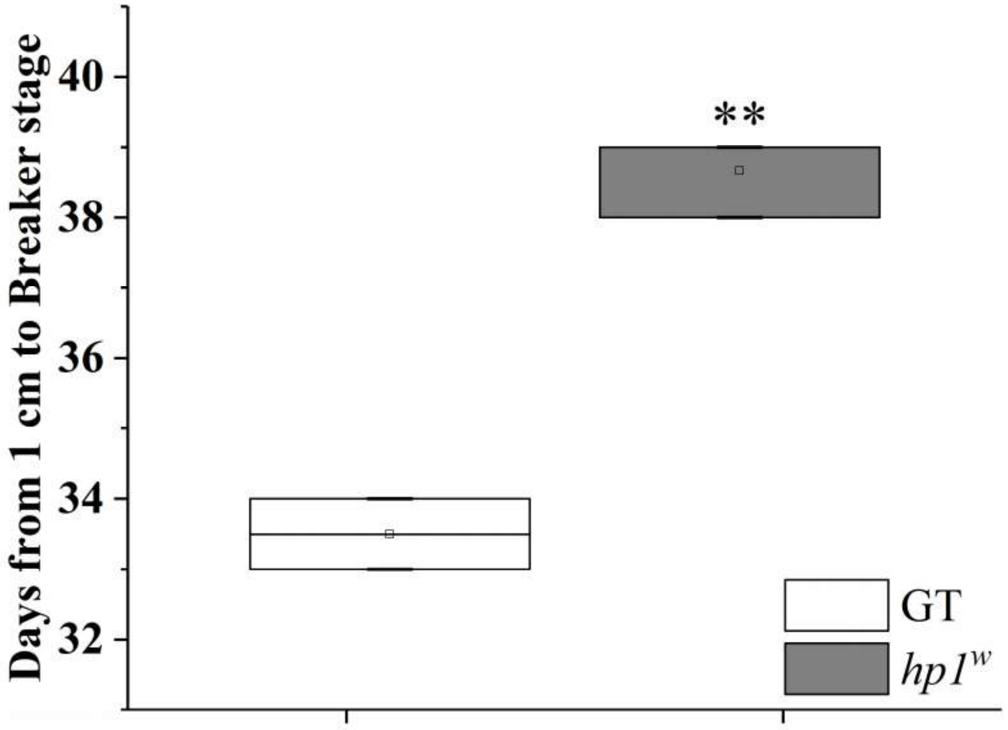

Suppemental Figure 2. Days from 1 cm fruit to the Breaker stage for wild type tomato GT and *hp1*^{w} mutant. ** indicates *P*< 0.01.

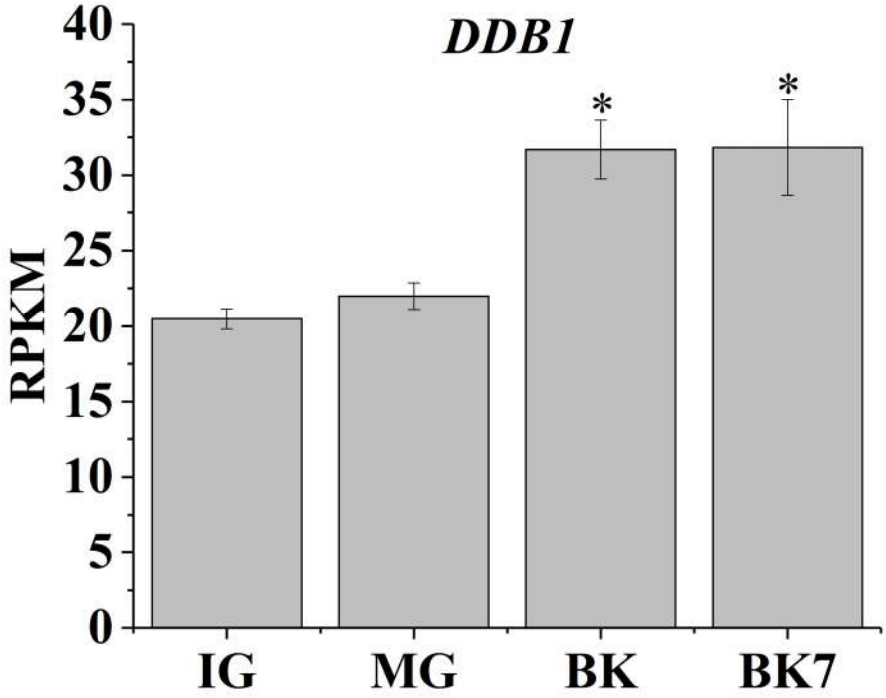

Supplemental Figure 3. Expression (RPKM) of DDB1/HP1 at various developmental stages in AC. * indicates 0.01 <*P*< 0.05. Error bars indicate standard error.

Supplemental Table 1. Summary statistics of AC and *hp1* transcriptome data.

sample	total raw reads	rRNA		final clean	mapped	
		No. reads	%rRNA		No. reads	%mapped
AC_IG_rep1	5,486,091	28,534	0.52	5,457,557	5,052,718	92.58
AC_IG_rep2	6,163,037	25,129	0.41	6,137,908	5,677,245	92.49
AC_IG_rep3	4,733,388	101,736	2.15	4,631,652	4,282,730	92.47
AC_IG_rep4	3,900,845	36,346	0.93	3,864,499	3,572,098	92.43
AC_MG_rep1	5,901,514	24,923	0.42	5,876,591	5,431,977	92.43
AC_MG_rep2	6,338,010	29,034	0.46	6,308,976	5,812,541	92.13
AC_MG_rep3	6,509,215	67,910	1.04	6,441,305	5,982,208	92.87
AC_BK_rep1	6,772,402	23,444	0.35	6,748,958	6,254,081	92.67
AC_BK_rep2	6,744,515	44,571	0.66	6,699,944	6,200,677	92.55
AC_BK_rep3	4,494,328	50,906	1.13	4,443,422	4,126,808	92.87
AC_BK7_rep1	4,188,664	180,665	4.31	4,007,999	3,553,753	88.67
AC_BK7_rep2	7,973,828	105,256	1.32	7,868,572	6,956,748	88.41
AC_BK7_rep3	3,759,667	36,711	0.98	3,722,956	3,285,797	88.26
HP1_IG_rep1	5,149,893	75,778	1.47	5,074,115	4,545,536	89.58
HP1_IG_rep2	7,020,636	36,150	0.51	6,984,486	6,400,074	91.63
HP1_IG_rep3	6,212,915	129,614	2.09	6,083,301	5,495,454	90.34
HP1_IG_rep4	7,669,284	36,291	0.47	7,632,993	7,037,944	92.20
HP1_MG_rep1	7,991,390	42,383	0.53	7,949,007	7,284,995	91.65
HP1_MG_rep2	10,623,949	50,182	0.47	10,573,767	9,758,705	92.29
HP1_MG_rep3	7,012,287	25,232	0.36	6,987,055	6,429,451	92.02
HP1_BK_rep1	7,593,702	43,848	0.58	7,549,854	6,904,726	91.46
HP1_BK_rep2	8,409,127	42,794	0.51	8,366,333	7,691,395	91.93
HP1_BK_rep3	7,578,773	33,603	0.44	7,545,170	6,933,756	91.90
HP1_BK7_rep1	6,072,946	43,549	0.72	6,029,397	5,519,633	91.55
HP1_BK7_rep2	6,283,246	47,228	0.75	6,236,018	5,762,765	92.41

Supplemental Table 2. List of ripening-associated genes defined as DEGs between AC IG and BK fruit bottom part tissues.

		AC_IG					AC_BK					
Gene ID	Annotation	rep1	rep2	rep3	rep4	mean	rep1	rep2	rep3	mean	ratio	adjust p
Solyc08g066690.2.1	Exostosin family prote	4.56	7.47	11.61	8.2	7.96	1121.28	1108.99	959.93	1063.4	133.59	6.69E-74
Solyc08g081220.1.1	Cytochrome P450	176.04	194.76	139.68	142.24	163.18	0.1	0.1	0.35	0.18	0	4.48E-69
Solyc03g097700.2.1	O-methyltransferase (A	3.54	2.65	7.92	6.59	5.17	313.17	425.35	260.45	332.99	64.35	1.03E-68
Solyc08g066700.2.1	Polyribonucleotide nuc	24.07	21.92	26.06	18.42	22.62	1037.83	996.94	981.67	1005.48	44.46	2.83E-62
Solyc02g091920.2.1	Xyloglucan endotransg	156.01	191.81	115.7	176.86	160.09	0.18	0.36	1.99	0.84	0.01	1.73E-54
Solyc07g054860.1.1	Aromatic amino acid d	77.34	102.66	108.5	104.22	98.18	0.21	0.21	0.65	0.36	0	1.86E-53
Solyc03g098240.2.1	Glutamate decarboxyla	39.22	29.07	30.64	27.46	31.6	707.5	1030.69	698.17	812.12	25.7	6.61E-53
Solyc01g010490.2.1	Cytochrome P450	0.91	0.93	2.31	0.74	1.22	52.77	65.47	38.38	52.21	42.7	2.28E-48
Solyc01g111570.2.1	Receptor-like kinase (A	248.11	315.78	287.41	209.76	265.26	2.63	4.75	3.51	3.63	0.01	3.96E-48
Solyc10g083670.1.1	Cellulose synthase-like	79.19	119.19	121.56	115.51	108.86	1.1	0.91	0.37	0.79	0.01	2.48E-46
Solyc01g104130.2.1	Oxidoreductase 2OG-F	7.33	8.15	6.49	3.89	6.46	173.96	150.7	83.67	136.11	21.05	8.03E-46
Solyc01g058320.2.1	T6A9.7 protein (AHRI	278.96	264.63	218.49	224.96	246.76	2.28	4.03	3.74	3.35	0.01	8.76E-45
Solyc11g007540.1.1	Cytochrome P450	99.48	113.7	65.63	77.95	89.19	0.21	0	0.39	0.2	0	2.63E-44
Solyc10g005210.2.1	DNA-3-methyladenine	93.57	83.95	64.45	65.24	76.8	0	0.77	0.19	0.32	0	5.92E-44
Solyc03g043850.2.1	Cytochrome c oxidase	9.53	10.44	22.48	21.77	16.05	407.44	442.01	608.26	485.9	30.26	2.94E-42
Solyc12g009300.1.1	Sucrose synthase (AHI	617.23	615.4	620.43	919.15	693.05	2.84	3.94	5.77	4.18	0.01	5.19E-42
Solyc01g097770.2.1	Serine/threonine protei	101.85	118.1	128.15	108.15	114.06	0.22	2.02	1.16	1.13	0.01	1.40E-41
Solyc01g107890.2.1	Filament-like plant pro	48.41	52.84	54.87	55.76	52.97	0.33	0.67	0.74	0.58	0.01	2.09E-41
Solyc02g087840.2.1	Homeobox-leucine zip	129.6	137.13	119.01	111.08	124.2	1.2	1.04	1.69	1.31	0.01	5.28E-41
Solyc10g050110.1.1	LRR receptor-like seri	59.57	78.06	83.98	48.87	67.62	0.36	0	0.3	0.22	0	1.19E-40
Solyc07g052960.1.1	GRAS family transcrip	0.92	1.91	2.17	2.82	1.96	313.84	318.17	335.48	322.5	164.96	5.70E-39
Solyc09g092500.1.1	UDP-glucosyltransfera	9.94	16.7	14.05	4.95	11.41	367.54	592.93	371.9	444.12	38.92	5.16E-38
Solyc10g083630.1.1	Repressor of silencing	29.21	29.37	32.97	31.96	30.88	406.5	461.08	345.1	404.23	13.09	2.32E-37
Solyc10g018780.1.1	Squamosa promoter bi	48.69	44.34	47.92	49.51	47.61	0	0.18	1.11	0.43	0.01	9.50E-37
Solyc03g113620.2.1	MYB transcription fac	615.66	737.64	804.43	472.49	657.56	9.95	14.63	12.43	12.34	0.02	9.90E-37
Solyc09g011140.2.1	Tropinone reductase I	83.74	96.85	65.77	63.7	77.51	0	1.19	0.97	0.72	0.01	1.07E-36
Solyc03g006010.2.1	Os05g0272200 protein	0	0.92	0.46	0	0.35	78.96	64.49	28.06	57.17	165.71	1.65E-36
Solyc02g083880.2.1	Gibberellin-regulated p	583.59	386.72	410.86	413.64	448.7	0.51	8.79	2.52	3.94	0.01	1.89E-36
Solyc01g105720.2.1	Unknown Protein (AH	375.81	376.8	361.77	285.38	349.94	12.55	11.87	10.65	11.69	0.03	1.92E-36
Solyc01g112120.2.1	Dimethylaniline mono	4.06	5.49	7.7	6.24	5.87	113.87	99.9	105.93	106.57	18.15	2.44E-36
Solyc11g072470.1.1	LOB domain protein 1	4.86	5.87	7.37	10.8	7.23	126.51	166.93	201.96	165.13	22.86	2.93E-36
Solyc05g014120.1.1	Os08g0119500 protein	5.85	8.52	6.9	4.52	6.45	132.82	135.91	144.96	137.9	21.39	4.83E-36
Solyc04g071650.2.1	Cellulose synthase (AH	192.29	213.01	279.22	255.06	234.89	3.52	8.03	3.48	5.01	0.02	8.19E-36
Solyc06g035720.2.1	BCL-2 binding anthano	449.84	398.05	593.26	390.74	457.97	7.89	15.1	6.73	9.91	0.02	1.68E-35
Solyc04g010250.2.1	Lipase-like protein (AI	318.52	407.51	526.34	311.15	390.88	1	3.19	2.25	2.15	0.01	1.70E-34
Solyc08g082620.2.1	3-oxoacyl-(Acyl carrie	38.69	35.52	24.82	42.63	35.41	0	0.33	0.13	0.15	0	3.17E-34
Solyc02g064940.1.1	LRR receptor-like seri	58.88	57.07	78	71.04	66.25	1.15	1.97	1.27	1.46	0.02	6.23E-34
Solyc01g097290.2.1	Auxin responsive prote	105.5	115.8	131.88	74.06	106.81	1.69	1.49	1.82	1.67	0.02	7.68E-34
Solyc01g094700.2.1	Glycerol-3-phosphate a	100.67	104.21	86.82	72.31	91	2.12	1.39	0.26	1.26	0.01	8.32E-34
Solyc05g055400.2.1	Cytochrome P450	361.17	412.49	255.84	265.37	323.72	4.48	4.93	1.66	3.69	0.01	2.16E-33
Solyc11g010810.1.1	Glucosyltransferase (A	108.55	114.69	156.66	106.79	121.67	3.06	2.85	2.5	2.8	0.02	2.95E-33
Solyc09g007480.2.1	Unknown Protein (AH	323.42	378.06	249.63	265.15	304.06	6.5	5.9	3.99	5.46	0.02	4.40E-33
Solyc01g081060.2.1	Xyloglucan endotransg	9.76	24.15	35.46	13.25	20.66	433.64	411.29	405.87	416.93	20.19	2.27E-32
Solyc05g005950.2.1	Solute carrier family 1	123.71	134.42	171.45	154.61	146.05	2.86	7.36	2.61	4.28	0.03	3.24E-32
Solyc11g010790.1.1	Glucosyltransferase (A	61.21	61.33	86.59	57.46	66.65	1.81	1.6	1.39	1.6	0.02	3.40E-32
Solyc08g067030.2.1	Os01g0611000 protein	1062.81	1029.55	903.23	1274.56	1067.54	20.35	44.22	14.96	26.51	0.02	1.14E-31
Solyc01g066740.2.1	Transducin family prot	36.48	42.41	44.98	38.75	40.65	1.37	0.98	0.69	1.01	0.02	1.30E-31
Solyc09g014350.2.1	Glycerol-3-phosphate a	139.65	190.79	105.73	105.33	135.38	0.11	0.21	0.78	0.37	0	1.30E-31
Solyc12g009930.1.1	UDP-glucuronosyltran	159.74	169.74	195.05	287.86	203.1	0.13	0.26	1.59	0.66	0	1.40E-31
Solyc11g066360.1.1	Unknown Protein (AH	16.35	17.27	18.31	23.13	18.77	189.68	281.89	233.04	234.87	12.52	1.55E-31
Solyc06g074070.2.1	Receptor like kinase, R	23.97	30.17	30.75	35.34	30.06	0.71	0.83	0.54	0.69	0.02	2.16E-31
Solyc05g007180.2.1	Homeobox-leucine zip	72.65	85.19	58.76	103.68	80.07	0.52	0.7	0.64	0.62	0.01	2.54E-31
Solyc12g044820.1.1	Multidrug resistance p	7.96	10.79	12.8	12.98	11.13	0.15	0.07	0.04	0.09	0.01	2.54E-31
Solyc09g075440.2.1	Ethylene receptor (AH	29.15	32.31	50.66	31.25	35.84	384.07	330.07	738.01	484.05	13.5	1.69E-30
Solyc02g078950.2.1	Beta-galactosidase (AI	20.45	24.18	18.33	23.19	21.54	0.19	0.64	0.15	0.33	0.02	3.16E-30
Solyc02g065530.2.1	Glycosyltransferase far	24.85	25.86	21.56	25.85	24.53	0	0	0.18	0.06	0	3.76E-30
Solyc12g098600.1.1	UDP-glucosyltransfera	75.78	95.03	70.79	67.17	77.19	1.95	1.85	1.41	1.74	0.02	5.25E-30
Solyc12g009800.1.1	Purple acid phosphatas	22.63	21.99	18.24	28.11	22.74	0.11	0	0	0.04	0	5.42E-30
Solyc07g053540.1.1	Fasciclin-like arabinog	38.04	33.14	42.37	40.64	38.55	0.21	0	0.79	0.33	0.01	7.10E-30
Solyc09g065240.2.1	Patatin-like protein 3 (	75.94	66.36	71.75	66.83	70.22	0.14	2.1	1.53	1.26	0.02	7.46E-30
Solyc07g049300.2.1	Endoglucanase 1 (AHI	52.62	68.97	88.8	102.2	78.15	0.11	0.21	0.91	0.41	0.01	7.70E-30
Solyc01g095320.2.1	BCL-2-associated atha	402.47	434.53	226.54	161.79	306.33	3167.89	3089.76	4099.81	3452.49	11.27	7.84E-30
Solyc02g063360.2.1	C2 domain-containing	19.36	19.69	10.72	18.44	17.05	195.64	188.96	666.19	350.26	20.54	8.31E-30
Solyc02g090680.2.1	Cyclin-dependent kina	70	71.12	41.29	56.52	59.73	0.25	0.25	0.31	0.27	0	1.52E-29
Solyc01g095340.2.1	Unknown Protein (AH	1609.93	1654.29	1022.74	674.49	1240.36	11885.57	12487.79	18302.85	14225.4	11.47	2.27E-29
Solyc01g108490.2.1	Serine carboxypeptidas	106.28	142.46	113.31	73.88	108.98	0.73	1.69	1.42	1.28	0.01	4.56E-29
Solyc06g083070.2.1	Fimbrin-like potential	22.75	33.78	36.22	35.13	31.97	0.4	0.57	0.69	0.55	0.02	5.49E-29
Solyc08g078650.2.1	Glycosyl transferase fa	0	0.5	0.33	0.4	0.31	51.14	32.48	28.7	37.44	121.76	6.41E-29
Solyc02g078040.2.1	Pistil extensin like pro	105.09	80.79	82.66	132.38	100.23	0.21	0.83	0	0.35	0	6.90E-29
Solyc08g079550.1.1	HAT family dimerisati	9.95	12.14	13.92	13.62	12.41	0.06	0.07	0.08	0.07	0.01	7.06E-29
Solyc03g025320.2.1	Hydroxycinnamoyl tra	286.31	295.51	193.81	206	245.41	6.59	7.83	5.06	6.49	0.03	1.60E-28
Solyc05g005080.2.1	Endo-1 4-beta-glucana	670.3	649.28	792.57	776.62	722.19	17.28	31.28	17.41	21.99	0.03	1.76E-28
Solyc08g007240.2.1	Hydrolase NUDIX fam	75.78	123.41	117.93	79.69	99.2	1.03	1.78	1.08	1.3	0.01	4.90E-28
Solyc09g061890.2.1	Pectate lyase 1-27 (AH	38.68	33.36	22.82	27.15	30.5	0	0.61	0.3	0.3	0.01	8.26E-28
Solyc10g083230.1.1	F-box family protein (A	3.87	4.04	3.77	1.9	3.4	44.32	51.29	51.95	49.19	14.49	1.39E-27
Solyc09g082580.2.1	Os12g0604200 protein	21.24	28.35	22.88	22.03	23.62	0.11	0.46	0	0.19	0.01	2.28E-27
Solyc02g063030.2.1	LuxR family transcript	50.29	56.18	41.8	73.16	55.36	0.33	0.66	0.4	0.46	0.01	3.42E-27
Solyc01g091170.2.1	Agmatinase (AHRD V	174.95	163.15	218.11	121.94	169.54	2.04	5.87	2.12	3.34	0.02	4.50E-27

Solyc08g061560.2.1	Receptor like kinase, R	10.19	12.09	6.75	11.02	10.01	0	0.22	0.13	0.12	0.01	5.13E-27
Solyc04g064690.2.1	Peroxidase (AHRD V1	39.96	61.14	42.13	36.8	45.01	1.79	0.83	0.84	1.15	0.03	9.15E-27
Solyc08g075490.2.1	Carotenoid cleavage di	25.06	25.46	24.72	21.49	24.18	0.27	0.81	0.44	0.51	0.02	1.27E-26
Solyc12g011310.1.1	Glutathione S-transfera	0	0.27	0	0	0.07	52.09	86.75	83.64	74.16	1098.67	1.57E-26
Solyc10g054080.1.1	Kinesin like protein (A	22.59	21.48	17.23	26.26	21.89	0.1	1.16	0.12	0.46	0.02	3.50E-26
Solyc10g006880.2.1	NAC domain protein (	1.48	2.97	4.59	1.83	2.72	1024.35	832.49	870.77	909.2	334.57	4.08E-26
Solyc09g090510.2.1	Cyclopropane-fatty-acy	31.49	37.69	34.72	18.92	30.7	0.37	0.12	0.23	0.24	0.01	4.89E-26
Solyc07g053640.1.1	Arabinogalactan-protei	2.43	2.89	2.39	1.72	2.36	137.4	189.36	174.98	167.25	70.94	5.67E-26
Solyc06g051800.2.1	Expansin (AHRD V1 *	0.25	2.02	3.27	6.77	3.08	1117.64	1627.91	2150.17	1631.91	530.27	6.68E-26
Solyc06g051460.2.1	ATP-dependent chaper	14.3	15.64	15.72	12.66	14.58	0.11	0.43	0.52	0.35	0.02	8.58E-26
Solyc10g076710.1.1	1-phosphatidylinositol-	33.86	44.18	47.32	34.72	40.02	0.8	0.89	0.44	0.71	0.02	8.58E-26
Solyc03g117310.1.1	Jp18 (AHRD V1 ***-	3.44	1.67	2.58	3.1	2.7	43.45	54.27	59.28	52.33	19.4	3.27E-25
Solyc04g054500.2.1	Metal ion binding prot	164.28	137.77	124.12	103.07	132.31	5.23	6.32	3	4.85	0.04	3.82E-25
Solyc03g122130.2.1	L-lactate dehydrogenas	0.19	0.17	0.23	0.54	0.28	17.97	18.44	17.32	17.91	63.4	4.25E-25
Solyc07g062040.2.1	ATP-binding/protein se	24.92	26.47	30.64	26.37	27.1	0	0.17	0.21	0.13	0	4.41E-25
Solyc02g079510.2.1	Peroxidase (AHRD V1	281.46	227.92	167.21	226.62	225.8	5.42	6.35	2.34	4.7	0.02	5.36E-25
Solyc07g056510.2.1	Glutathione S-transfera	0	0	0	0	0	103.93	180.82	241.09	175.28	17528	8.01E-25
Solyc12g096870.1.1	UDP-glucosyltransfera	23.71	23.99	15.66	15.33	19.67	0.11	0	0	0.04	0	1.06E-24
Solyc01g111220.2.1	Glutamyl-tRNA(Gln) a	14.4	16.71	15.02	22.92	17.26	0.1	0.21	0.26	0.19	0.01	1.08E-24
Solyc01g102310.2.1	Unknown Protein (AH	89.48	86.43	106.4	118.26	100.14	3.26	6.15	3.31	4.24	0.04	1.08E-24
Solyc09g089580.2.1	1-aminocyclopropane-	23.56	29.2	52.39	59.48	41.16	19191.72	13804.77	17420.25	16805.58	408.32	1.37E-24
Solyc03g013340.2.1	Aquaporin Z transmem	167.48	162.7	83.31	116.64	132.53	0.38	0.76	0.46	0.53	0	1.67E-24
Solyc03g083100.2.1	Calmodulin-binding pr	184.86	168.52	127.84	163.4	161.16	5.44	10.87	4.74	7.02	0.04	1.82E-24
Solyc05g005490.2.1	Carbonic anhydrase (A	75.29	86.43	131.94	98.17	97.96	773.52	842.93	794.55	803.67	8.2	2.26E-24
Solyc02g080530.2.1	Peroxidase (AHRD V1	222.12	223.36	221.36	296.5	240.84	7.17	24.99	9.21	13.79	0.06	2.38E-24
Solyc08g014130.2.1	2-isopropylmalate synt	10.94	10.98	11.14	8.04	10.28	88.31	94.32	154.39	112.34	10.93	2.38E-24
Solyc03g112060.2.1	Quinolinate synthase A	16.54	15.03	14.48	18.86	16.23	0.14	0.22	0.53	0.3	0.02	3.01E-24
Solyc10g009410.1.1	Aspartic proteinase ne	57.49	67.28	91.4	82.13	74.58	2.32	1.99	2.43	2.25	0.03	3.03E-24
Solyc02g081830.2.1	HAD-superfamily hydr	9.75	11.19	6.66	6.17	8.44	88.14	91.62	90.96	90.24	10.69	3.73E-24
Solyc05g005960.2.1	Peptide transporter 1 (	112.73	120.38	172.39	150.57	139.02	7.85	10.53	4.31	7.56	0.05	4.39E-24
Solyc01g005590.2.1	Unknown Protein (AH	181.95	144.89	123.02	179.11	157.24	1.72	3.47	3.17	2.79	0.02	6.98E-24
Solyc03g112390.2.1	Myb transcription facto	29.55	33.32	16.95	28.91	27.18	0	0.16	0.8	0.32	0.01	7.42E-24
Solyc03g115000.2.1	AT1G74160 protein (F	16.06	14.5	21.43	17.25	17.31	0.49	0.44	0.18	0.37	0.02	8.63E-24
Solyc10g078700.1.1	Squamosa promoter-bi	25.47	31.48	23.37	30.02	27.59	0.71	0	0	0.24	0.01	8.87E-24
Solyc08g079870.1.1	Subtilisin-like protease	56.6	47.07	81.69	45.16	57.63	0.29	0.29	0.26	0.28	0	1.15E-23
Solyc07g052230.2.1	Laccase-22 (AHRD V1	20.85	14.73	15.19	14.92	16.42	0.2	0.2	0.12	0.17	0.01	1.21E-23
Solyc03g078440.2.1	Cinnamyl alcohol dehy	26.42	21.12	26.21	24.04	24.45	0.27	0.55	0.33	0.38	0.02	1.37E-23
Solyc08g065430.2.1	N-myc downstream reg	38.86	41.5	29.3	42.1	37.94	0.61	1.85	2.07	1.51	0.04	1.59E-23
Solyc11g010800.1.1	Anthocyanidin 3-O-glu	69.04	69.64	105.34	81.16	81.3	1.98	2.25	1.52	1.92	0.02	1.68E-23
Solyc10g054840.1.1	Aquaporin (AHRD V1	61.14	57.28	32.51	40.11	47.76	0.32	0	0.2	0.17	0	2.09E-23
Solyc08g065610.2.1	Vacuolar processing en	20.35	26.14	97.17	33.04	44.17	421.42	558.17	609.4	529.66	11.99	2.22E-23
Solyc04g079560.2.1	Lysine/histidine transp	41.89	44.76	40.69	65.64	48.25	0	1.12	0.25	0.46	0.01	2.28E-23
Solyc04g063210.2.1	Caffeoyl-CoA O-methy	42.66	34.23	41.25	27.04	36.29	0.19	1.14	0.93	0.75	0.02	2.35E-23
Solyc08g083110.2.1	Cystathionine gamma-	248.76	188.41	314.13	302.18	263.37	6.47	13.53	6.47	8.82	0.03	2.64E-23
Solyc02g065770.2.1	COBRA-like protein 4	323.63	329.06	317.1	350.83	330.16	11.98	26.7	8.09	15.59	0.05	3.64E-23
Solyc02g091560.2.1	Serine hydroxymethylt	313.71	293	375.21	330.83	328.19	8.93	28.38	18.55	18.62	0.06	5.24E-23
Solyc05g009120.2.1	Os03g0731050 protein	0.17	0.3	0.8	0.24	0.38	12.81	16.77	27.63	19.07	50.52	5.26E-23
Solyc03g112760.2.1	Unknown Protein (AH	11.05	18.47	18.14	12.6	15.06	0.1	0.5	0.55	0.38	0.03	5.26E-23
Solyc03g121600.2.1	Choline dehydrogenase	143.86	138.9	74.32	80.68	109.44	0.24	0	0.74	0.33	0	5.69E-23
Solyc01g079410.2.1	SMP-30/Gluconolacon	54.88	67.87	34.27	45.94	50.74	0.98	0.49	0.6	0.69	0.01	9.97E-23
Solyc02g094330.2.1	Pollen proteins Ole e I	76.16	117.43	73.25	72.05	84.72	2.35	2.37	4.33	3.02	0.04	1.10E-22
Solyc11g008440.1.1	Amino acid transporter	1.9	3.48	2.38	0.67	2.11	27.9	24.75	29.21	27.29	12.95	1.75E-22
Solyc12g017250.1.1	photosystem II polypep	1.43	2.98	6.2	4.73	3.83	798.32	1141.37	675.7	871.8	227.33	1.94E-22
Solyc06g060620.2.1	Nitrate transporter (AH	31.82	34.85	59.06	46.89	43.16	0.09	0.54	0	0.21	0	2.82E-22
Solyc12g042830.1.1	class I heat shock prote	4.12	2.94	0.97	5.83	3.47	157.9	264.76	326.59	249.75	72.08	2.88E-22
Solyc07g062550.2.1	UDP-N-acetylglucosan	38.88	31.31	18.51	27.01	28.93	0	0.24	0.3	0.18	0.01	3.45E-22
Solyc01g006540.2.1	Lipoxygenase (AHRD	0.29	0.79	0.17	0.42	0.42	208.62	266.33	205.06	226.67	542.92	3.90E-22
Solyc09g092380.2.1	Adenosylhomocysteina	991.33	959.11	1450.78	1604.99	1251.55	7503.91	7282.16	11101.53	8629.2	6.89	4.03E-22
Solyc08g078300.2.1	Homeobox-leucine zip	28.88	32.8	43.21	46.16	37.76	0.19	0.57	0.47	0.41	0.01	5.55E-22
Solyc10g005200.2.1	Novel protein (AHRD	64.33	66.28	59.22	48.11	59.48	1.88	5.03	1.91	2.94	0.05	6.95E-22
Solyc09g074430.2.1	Monooxygenase (AHR	17.3	19.47	12.21	12.71	15.42	0.14	0.14	0.17	0.15	0.01	1.08E-21
Solyc03g093610.1.1	Ethylene responsive tra	48.87	35.76	32.67	33.41	37.68	0.88	0.66	0.54	0.69	0.02	1.67E-21
Solyc06g007130.2.1	Omega-3 fatty acid des	19.9	22.37	29.24	27.9	24.85	0.42	0.85	0.17	0.48	0.02	1.69E-21
Solyc05g005570.2.1	BURP domain-contain	24.15	23.66	17.3	26.84	22.99	0.46	0.9	0.43	0.61	0.03	2.34E-21
Solyc08g005560.2.1	Binding protein (AHRI	150.77	159.51	126.39	128.83	141.38	5.42	11.89	8.4	8.57	0.06	2.64E-21
Solyc02g063150.2.1	Ribulose bisphosphate	768.45	598.11	374.62	461.96	550.78	1.46	6.5	11.51	6.49	0.01	4.87E-21
Solyc06g008590.2.1	Auxin responsive prote	29.04	20.79	17.88	28.13	23.96	0	0	0.31	0.1	0	5.19E-21
Solyc10g075160.1.1	Ferredoxin I (AHRD V	322.58	287.09	433.71	218.17	315.39	2.57	6.3	4.52	4.46	0.01	5.93E-21
Solyc11g013330.1.1	F2E2.8 (AHRD V1 ***	1.44	0.73	0.97	0.58	0.93	29.31	19.49	43.59	30.8	33.11	6.12E-21
Solyc11g066930.1.1	AT5G28150-like prote	2.89	3.85	1.7	2.04	2.62	31.81	34.44	22.92	29.72	11.34	6.38E-21
Solyc07g062470.2.1	Light-dependent short	184.54	208.67	117.8	223.2	183.55	5.04	4.72	2.65	4.14	0.02	7.57E-21
Solyc07g065110.1.1	Protease inhibitor/seed	78.54	65.98	47.44	65.77	64.43	0.51	0	0.62	0.38	0.01	7.75E-21
Solyc07g063640.1.1	Wound induced protei	21.77	21.14	20.24	20.53	20.92	566.03	384.9	972.41	641.11	30.65	1.13E-20
Solyc07g017600.2.1	Pectinesterase (AHRD	95.54	119.49	123.81	140.84	119.92	8.88	6.77	6.79	7.48	0.06	1.28E-20
Solyc03g063600.2.1	Guanylate kinase (AHI	51.89	62.36	68.95	67.61	62.7	374.28	359.63	467.15	400.35	6.38	1.60E-20
Solyc04g008040.2.1	microtubule associated	18.04	16.06	13.98	23.71	17.95	0.43	0.52	0.53	0.49	0.03	1.78E-20
Solyc11g027880.1.1	Phosphatidylinositol tr	55.22	44.96	53.42	47.32	50.23	1.85	2.41	2.27	2.18	0.04	1.85E-20
Solyc06g083660.2.1	Genomic DNA chromo	35.59	41.56	33.02	48.55	39.68	1.48	2.87	2.3	2.22	0.06	2.18E-20
Solyc08g078670.2.1	Aspartyl protease (AHI	110.79	117.15	198.74	140.75	141.86	2.15	2.81	3.73	2.9	0.02	2.44E-20
Solyc01g010870.2.1	Dehydration-responsiv	15.73	19.6	24.45	20.26	20.01	136.93	103.62	114.16	118.24	5.91	2.70E-20
Solyc12g055840.1.1	Glucan endo-1 3-beta-g	57.55	55.94	55.94	83.83	63.31	1.13	3.43	0.46	1.67	0.03	2.81E-20
Solyc02g071090.2.1	Purine permease famil	2.98	3.9	7.45	2.48	4.2	99.08	102.8	104.32	102.07	24.29	2.83E-20

Solyc06g082940.2.1	Photosystem I reaction	313.44	235.39	254.27	228.01	257.78	16.64	25.78	18.96	20.46	0.08	3.23E-20
Solyc06g065970.1.1	Cortical cell-delineatin	48.83	53.63	36.77	71.27	52.62	0	0	0	0	0	3.50E-20
Solyc05g007070.2.1	Alpha amylase 2 (IC) (	49.57	51.42	61.36	47.44	52.45	240.41	369.56	308.57	306.18	5.84	3.77E-20
Solyc06g084050.2.1	Photosystem II reactio	149.84	144.28	141.18	126.59	140.47	7.85	10.83	6.09	8.26	0.06	3.89E-20
Solyc02g068120.2.1	Gda-1 (AHRD V1 **--	33.97	34.88	27.74	33.88	32.62	0.53	1.24	0.86	0.88	0.03	4.85E-20
Solyc06g060340.2.1	Chloroplast photosyste	214.11	270.47	248.95	156.99	222.63	26.94	11.26	11.35	16.52	0.07	5.04E-20
Solyc11g044450.1.1	Chaperone protein dna	13.96	18.72	13.31	11.63	14.41	0.15	0	0.19	0.11	0.01	6.15E-20
Solyc09g008780.2.1	Os01g0786800 protein	12.19	24.25	27.24	15.42	19.77	596.6	517.24	265.63	459.82	23.25	6.90E-20
Solyc06g074630.2.1	Cellulose synthase-like	20.75	31.78	20.41	29.88	25.7	1	1.71	1.35	1.35	0.05	7.09E-20
Solyc02g069290.2.1	Transducin-like (AHR	13.35	16.74	14.43	15.26	14.94	0.58	1.02	0.72	0.77	0.05	8.25E-20
Solyc04g078460.2.1	N(4)-(Beta-N-acetylglu	4.65	5.04	12.18	2.58	6.11	134.55	94.65	191.63	140.28	22.95	8.90E-20
Solyc12g005840.1.1	Ras-related protein Ra	37.48	57.35	45.25	40.92	45.25	0.71	2.14	0.22	1.02	0.02	1.13E-19
Solyc06g068390.1.1	Zinc finger family prot	26.31	25.4	26.67	23.07	25.36	0	0	0	0	0	1.13E-19
Solyc02g093310.2.1	MAP-like protein kina	35.78	32.98	29.4	45.42	35.89	1.03	1.82	0.95	1.27	0.04	1.27E-19
Solyc05g007120.2.1	Receptor like kinase, R	131.14	172.43	100.88	93.94	124.6	2.44	3.55	1.32	2.44	0.02	1.39E-19
Solyc10g005960.1.1	Fasciclin-like arabinog	59.47	58.71	74.65	105.88	74.68	0.51	0.65	0.63	0.6	0.01	2.09E-19
Solyc05g051460.2.1	Homeobox-leucine zip	76.06	81.71	121.46	120.95	100.04	2.38	3.94	1.25	2.52	0.03	2.17E-19
Solyc01g099290.2.1	Cullin-like protein1 (A	54.36	54.1	44.89	35.65	47.25	1.2	4.03	0.49	1.91	0.04	2.31E-19
Solyc04g082690.1.1	Ring finger protein (A	10.04	4.15	7.19	12.68	8.52	69.23	69.53	83.63	74.13	8.71	2.33E-19
Solyc03g034220.2.1	Ribulose bisphosphate	2552.46	2000.81	1696.82	1542.55	1948.16	51.24	115.53	144.35	103.71	0.05	2.55E-19
Solyc09g010380.2.1	Inorganic pyrophospha	266.32	328.74	189.74	248.99	258.45	23.82	22.05	9.05	18.31	0.07	2.55E-19
Solyc04g081240.2.1	Auxin response factor	0.43	1.2	1.5	0.9	1.01	9.85	11.09	9.21	10.05	9.98	2.55E-19
Solyc02g083930.1.1	Unknown Protein (AH	95.29	102.75	76.39	67.39	85.45	1.48	3.98	0	1.82	0.02	2.95E-19
Solyc11g008540.1.1	Ribonuclease 3-like pr	43.83	44.35	54.76	61.59	51.13	2.4	2.38	5.52	3.43	0.07	3.03E-19
Solyc07g049660.2.1	Acetyl coenzyme A cis	30.45	25.47	37.42	24.92	29.57	0.57	2.41	0.84	1.27	0.04	3.44E-19
Solyc02g071080.1.1	Purine permease famil	3.69	5.64	3.42	3.36	4.03	47.12	42.79	45.84	45.25	11.24	3.82E-19
Solyc02g089620.2.1	Proline dehydrogenase	601.04	534.92	382.43	503	505.35	24.88	46.19	23.32	31.46	0.06	4.25E-19
Solyc07g053720.2.1	Tyrosine aminotransfe	38.05	48.16	29.07	34.41	37.42	1.51	2.92	1.08	1.84	0.05	4.95E-19
Solyc02g036350.2.1	1-aminocyclopropane-	117.72	143.48	380.4	172.93	203.63	2506.86	1780.89	1639.92	1975.89	9.7	5.08E-19
Solyc08g082080.2.1	Amino acid transporte	21.6	19.74	15.91	14.77	18	114.8	113.66	70.95	99.8	5.54	5.11E-19
Solyc09g015830.1.1	Pto-like, Serine/threon	31.36	29.54	37.24	65.32	40.86	201.06	228.43	425.63	285.04	6.98	5.64E-19
Solyc01g010180.2.1	Unknown Protein (AH	96.68	81.66	67.54	57.92	75.95	5.51	4.02	2.26	3.93	0.05	5.92E-19
Solyc04g071770.2.1	Ethylene responsive tra	2.18	1.94	6.01	3.3	3.36	27.88	30.97	28.04	28.96	8.63	6.57E-19
Solyc01g112260.2.1	Unknown Protein (AH	198.78	156.44	188.44	257.23	200.22	10.52	16.62	11.2	12.78	0.06	6.87E-19
Solyc11g065320.1.1	Zinc finger CCCH don	28.64	29.27	32.34	35.03	31.32	1.15	2.77	0.75	1.56	0.05	1.01E-18
Solyc02g091000.2.1	Harpin-induced protei	8.04	6.05	5.84	7.87	6.95	46.47	60.81	53.61	53.63	7.72	1.08E-18
Solyc07g007260.2.1	Metallocarboxypeptida	123.99	123.79	66.58	73.08	96.86	0	0	0.79	0.26	0	1.28E-18
Solyc01g080220.2.1	Dienelactone hydrolase	2.76	1.72	2.93	0.39	1.95	46.83	48.36	32.06	42.42	21.75	1.31E-18
Solyc05g014280.2.1	Heat shock protein (A	0.3	0.26	0.35	0	0.23	92.43	164.42	171.08	142.64	627	1.61E-18
Solyc05g009950.2.1	Cytochrome B561 fam	32.44	39.39	34.38	26.05	33.07	0.44	1.12	0.27	0.61	0.02	1.63E-18
Solyc11g010600.1.1	Os01g0841200 protein	552.15	522.3	492.99	495.22	515.66	36.72	56.45	41.97	45.05	0.09	2.15E-18
Solyc11g013500.1.1	Unknown Protein (AH	2.15	1.6	2.96	1.01	1.93	76.18	88.82	56.23	73.74	38.21	2.34E-18
Solyc08g076220.2.1	Phosphoribulokinase/u	172.08	181.12	173.91	151.49	169.65	10.77	18.37	15.02	14.72	0.09	2.43E-18
Solyc04g071890.2.1	Peroxidase 4 (AHRD V	104.91	116.42	137.82	86.9	111.51	5.35	6.78	3.38	5.17	0.05	2.44E-18
Solyc03g007430.2.1	Mitochondrial carrier f	9.49	8.31	14.93	9.85	10.65	63.01	64.33	108.98	78.77	7.4	2.81E-18
Solyc10g080390.1.1	Serine/threonine prote	27.35	28.52	20.81	19.34	24.01	1.1	1.36	0.21	0.89	0.04	3.17E-18
Solyc08g006310.2.1	Cellulose synthase-like	5.98	8.79	10.64	11.41	9.21	0	0.31	0	0.1	0.01	3.37E-18
Solyc03g006400.2.1	Protein kinase (AHRD	2.33	1.83	2.1	1.94	2.05	26.7	41.24	22.6	30.18	14.72	3.88E-18
Solyc03g119540.2.1	CONSTANS-like zinc	26.61	25.84	26.64	17.34	24.11	0.26	1.05	0.32	0.54	0.02	3.88E-18
Solyc10g051120.1.1	Brain protein 44-like p	0	0	0	0	0	32.76	20.16	37.52	30.15	3014.67	4.14E-18
Solyc03g119610.1.1	Genomic DNA chromo	83.27	73.63	106.86	121.62	96.34	2.18	5.73	4.83	4.25	0.04	4.15E-18
Solyc02g071610.2.1	GDSL esterase/lipase A	20.44	30	26.44	29.92	26.7	0.14	1.31	0.18	0.54	0.02	4.17E-18
Solyc09g091050.2.1	Lipase (AHRD V1 *-*-	7.48	8.56	6.76	8.21	7.75	43.55	52.58	50.48	48.87	6.3	4.39E-18
Solyc07g042390.1.1	Pectinesterase (AHRD	96.85	97.44	51.34	68.5	78.53	1.13	1.72	1.39	1.41	0.02	4.58E-18
Solyc01g079260.2.1	WRKY transcription fa	17.06	15	11.4	11.63	13.77	89.5	95.62	99.35	94.82	6.88	4.73E-18
Solyc12g055970.1.1	Endoglucanase 1 (AHI	10.4	10.91	11.6	7.66	10.14	0.12	0	0.14	0.09	0.01	4.89E-18
Solyc09g007550.2.1	Zinc finger family prot	24.52	20.93	34.01	39.56	29.76	0.46	0.12	0.43	0.34	0.01	4.97E-18
Solyc09g011670.2.1	Universal stress protei	106.35	147.32	100.47	78.05	108.05	4.85	4.24	1.98	3.69	0.03	5.61E-18
Solyc04g081190.2.1	BZIP transcription fact	17.05	19.13	20.64	16.57	18.35	90.07	141.82	82.85	104.91	5.72	6.90E-18
Solyc02g079500.2.1	Peroxidase (AHRD V1	1328.64	1018.89	695.8	1163	1051.58	33.73	26.81	14.53	25.02	0.02	7.58E-18
Solyc09g098540.2.1	Chitinase-like protein (	2990.76	2904.87	2834.79	3709.3	3109.93	231.85	348.25	243.51	274.54	0.09	8.10E-18
Solyc04g050620.2.1	Cytochrome P450	38.52	31.08	19.18	32.05	30.21	0.43	0.65	0.79	0.62	0.02	1.01E-17
Solyc08g078460.2.1	Inositol 2-dehydrogena	0	0	0	0	0	22.54	31.88	28.39	27.6	2760.33	1.03E-17
Solyc01g008700.2.1	cDNA clone 001-102-(	11.65	9	10.38	7.47	9.62	185.12	185.82	116.56	162.5	16.88	1.05E-17
Solyc08g016720.1.1	9-cis-epoxycarotenoid	48.06	38.94	40.25	28.86	39.03	2.66	2.31	0.68	1.88	0.05	1.08E-17
Solyc01g110360.2.1	Fructose-bisphosphate	333.17	264.36	321.69	279.95	299.79	13.39	27.28	28.46	23.04	0.08	1.15E-17
Solyc03g118970.2.1	Multidrug resistance p	27.4	36.7	26.52	22.39	28.25	0.64	1.73	1.06	1.14	0.04	1.20E-17
Solyc07g054060.2.1	Unknown Protein (AH	58.84	88.86	96.76	71.88	79.08	5738.96	5021.97	5116.46	5292.46	66.92	1.54E-17
Solyc02g062460.2.1	2-oxoglutarate-depend	63.72	75.85	92.58	95.34	81.87	412.48	413.37	536.67	454.17	5.55	1.61E-17
Solyc05g005560.2.1	BURP domain-contain	1041.21	1166.09	816.31	1073.5	1024.28	56.93	118.51	28.95	68.13	0.07	1.70E-17
Solyc10g086660.1.1	Rop guanine nucleotid	10.28	14.76	10.3	13.59	12.23	65.53	80.56	56.72	67.6	5.53	1.86E-17
Solyc01g097520.2.1	Annexin 11 (AHRD V	823.7	660.3	970.32	583.15	759.37	19.67	63.76	21.9	35.11	0.05	1.87E-17
Solyc01g006400.2.1	Cysteine-rich extensin-	50.26	45.9	80.92	47.77	56.21	1.27	1.71	0.78	1.25	0.02	2.02E-17
Solyc03g115870.2.1	Thioredoxin 2 (AHRD	29.89	46.49	39.07	27.8	35.81	2.39	1.32	1.07	1.59	0.04	2.05E-17
Solyc10g005690.2.1	Chloride channel E (A	10.63	15.05	9.46	15.16	12.57	0.28	0.36	0.09	0.24	0.02	2.05E-17
Solyc06g068950.2.1	Phosphoethanolamine	44.11	46.14	50.32	67.3	51.97	2.05	5.43	1.06	2.85	0.05	2.16E-17
Solyc08g079840.1.1	Subtilisin-like protease	6.26	5.26	7.18	5.49	6.05	0	0.07	0.09	0.05	0.01	2.35E-17
Solyc05g049990.2.1	Heavy metal-associate	173.06	224.4	178.59	131.66	176.93	14.24	13.26	3.14	10.21	0.06	2.36E-17
Solyc02g082690.2.1	Syntaxin binding prote	12.57	18.7	20.51	9.91	15.42	86.89	82.5	69.45	79.61	5.16	2.38E-17
Solyc01g111340.2.1	Endo-1 4-beta-xylanas	14.33	19.61	18.04	25.03	19.25	106.89	91.92	74.21	91.01	4.73	2.41E-17
Solyc05g026240.1.1	LRR receptor-like seri	77.58	85.67	48.64	74.27	71.54	3.67	5.76	1.17	3.53	0.05	2.62E-17
Solyc07g049290.2.1	Nitrate transporter (AH	35.1	43.44	55.88	38.46	43.22	3.15	4.54	1.11	2.93	0.07	2.67E-17

Solyc04g016330.2.1	Glycerol-3-phosphate d	63.56	75.25	47.87	54.38	60.27	6.65	4.97	2.11	4.58	0.08	2.81E-17
Solyc07g056140.2.1	Glucose-1-phosphate a	325.31	248.24	224.55	297.11	273.8	8.99	40.16	9.53	19.56	0.07	3.07E-17
Solyc11g062440.1.1	Laccase (AHRD V1 **	12.99	18.82	15.86	11.22	14.72	0.09	1.03	0.11	0.41	0.03	3.15E-17
Solyc01g099040.2.1	GDSL esterase/lipase A	0.17	0.31	0.2	0.73	0.35	294.33	215.03	163.1	224.15	635.9	3.19E-17
Solyc03g117300.2.1	Nodulin-like protein (A	18.25	28	26.22	24.98	24.36	0.76	1.24	0.58	0.86	0.04	3.23E-17
Solyc11g008010.1.1	NAC domain protein I	18.26	17.77	56.29	29.64	30.49	166.99	139.01	93.73	133.24	4.37	3.95E-17
Solyc08g080140.2.1	dTDP-4-dehydrorhamn	321.77	245.55	321.89	371.72	315.23	12.71	36.14	17.13	21.99	0.07	3.98E-17
Solyc09g092430.2.1	Selenium binding prote	44.56	43.98	46.38	68.56	50.87	1.41	3.4	1.47	2.09	0.04	5.13E-17
Solyc02g093480.2.1	Lysine ketoglutarate re	12.05	10.19	10.13	10.23	10.65	0.12	0	0.15	0.09	0.01	5.30E-17
Solyc03g044740.2.1	Methyl jasmonate este	29.99	32.24	14.72	26.13	25.77	0.19	0.2	0.24	0.21	0.01	5.44E-17
Solyc03g095870.1.1	Unknown Protein (AH	0	2.94	1.3	0	1.06	58.63	61.82	50.2	56.88	53.66	6.33E-17
Solyc08g006870.2.1	BZIP domain class tra	8.57	14.49	14.28	10.3	11.91	0.52	0.52	0.43	0.49	0.04	7.49E-17
Solyc07g006030.2.1	Protein TIF31 homolog	6.55	6.72	10.28	7.17	7.68	58.74	46.08	69.17	58	7.55	7.67E-17
Solyc11g006250.1.1	GDSL esterase/lipase A	768.94	990.37	349.71	458.61	641.91	0.44	0.44	3.97	1.62	0	9.29E-17
Solyc03g113350.2.1	CHY zinc finger conta	10.84	12.75	11.66	8.22	10.87	0.26	0.52	0.21	0.33	0.03	1.02E-16
Solyc05g014000.2.1	Pectate lyase (AHRD V	216.13	288.9	107.26	153.21	191.38	0.53	0.27	1.63	0.81	0	1.10E-16
Solyc08g066330.1.1	Cell division protein k	47.34	55.47	45.99	54.23	50.76	3.29	4.01	4.04	3.78	0.07	1.27E-16
Solyc11g066100.1.1	heat shock protein (AH	14.6	12.55	10.34	9.4	11.72	231.5	499.49	429.78	386.92	33.01	1.28E-16
Solyc03g117840.2.1	Unknown Protein (AH	142.25	182.87	188.09	111.78	156.25	12.21	12.32	2.05	8.86	0.06	1.46E-16
Solyc04g072850.2.1	Alpha-L-arabinofurano	27.71	32.69	20.86	43.05	31.08	0.55	0.28	0	0.28	0.01	1.46E-16
Solyc01g008550.2.1	Cinnamoyl CoA reduc	52.82	55.6	35.89	38.37	45.67	3.82	3.35	2.45	3.21	0.07	1.65E-16
Solyc03g120380.2.1	Auxin response factor	108.36	94.31	36.7	83.16	80.63	0	0	1.7	0.57	0.01	1.75E-16
Solyc07g055930.2.1	Glycosyltransferase (A	6.27	11.59	10.1	9.21	9.29	58.07	71.74	55.31	61.71	6.64	1.98E-16
Solyc09g074270.2.1	Acetyl esterase (AHRI	2.67	6.45	9	4.58	5.68	42.21	39.93	66.62	49.59	8.74	2.18E-16
Solyc08g007040.2.1	Glycine cleavage syste	42.35	35.52	29.31	21.89	32.27	0.33	0.66	0.81	0.6	0.02	2.25E-16
Solyc01g098400.2.1	Histidine phosphotrans	42.5	46.1	40.22	22.55	37.84	1.43	1.08	1.76	1.42	0.04	2.43E-16
Solyc12g088700.1.1	UDP-glucosyltransfera	24.59	28.84	32.15	23.04	27.16	0.54	1.76	0.49	0.93	0.03	2.43E-16
Solyc09g056340.2.1	Ras-related protein Ra	41.58	33.39	23.98	38.92	34.47	143.22	242.8	273.72	219.91	6.38	2.75E-16
Solyc10g081730.1.1	Os02g0200800 protein	99.7	97.77	134.71	88.88	105.27	6.39	14.47	7.85	9.57	0.09	2.89E-16
Solyc03g007920.2.1	Glutamine cyclotransfe	65.74	78.15	49.92	58.89	63.17	6.25	5.01	1.58	4.28	0.07	3.69E-16
Solyc05g056070.2.1	Chlorophyll a-b bindin	175.74	154.27	120.06	120.14	142.55	15.97	13.93	6.63	12.18	0.09	3.84E-16
Solyc01g105360.2.1	UDP-glucuronosyltran	21.95	27.51	19.1	15.9	21.12	0.65	2.4	0.53	1.19	0.06	3.95E-16
Solyc02g020940.2.1	Glyceraldehyde-3-phos	115.53	113.13	111.05	89.67	107.34	4.42	12.98	5.75	7.72	0.07	3.97E-16
Solyc09g009420.1.1	Unknown Protein (AH	35.33	26.89	31.13	33.11	31.61	0	0.69	0.42	0.37	0.01	4.12E-16
Solyc05g014380.2.1	Multidrug resistance pr	6.74	7.24	7.33	3.94	6.31	62.64	46.61	79.14	62.8	9.95	4.28E-16
Solyc02g091700.2.1	Hydroxyproline-rich gl	60.09	61.33	72.87	106.99	75.32	308.93	367.4	331.16	335.83	4.46	5.71E-16
Solyc12g088240.1.1	Cellulose synthase-like	4.02	2.22	1.81	2.17	2.56	15.08	22.08	9.69	15.62	6.11	5.77E-16
Solyc03g034140.2.1	Flavoprotein wrbA (AI	0	0	0.33	0	0.08	9.9	12.25	9.12	10.42	126.34	5.83E-16
Solyc02g070890.2.1	Receptor like kinase, R	0.39	0.6	1.46	0.8	0.81	8.2	6.75	31.57	15.51	19.09	5.93E-16
Solyc04g054990.2.1	Lipoxygenase homolog	497.39	495.38	348.25	709.2	512.56	17.11	12.75	10.66	13.51	0.03	5.93E-16
Solyc05g005980.2.1	Solute carrier family 1	11.6	14.58	16.91	16.89	15	0.37	0.74	0.79	0.63	0.04	6.17E-16
Solyc06g082460.1.1	Plant-specific domain	9.56	13.18	7.74	6.89	9.34	0	0	0.19	0.06	0.01	6.76E-16
Solyc08g077300.2.1	Adenylate kinase (AHI	15.81	19.48	16.14	13.33	16.19	95.3	98.6	107.43	100.44	6.2	7.31E-16
Solyc06g061170.2.1	Solute carrier family 2	16.35	16.85	19.97	17.04	17.55	220.61	222.98	263.64	235.74	13.43	7.60E-16
Solyc04g009030.2.1	Glyceraldehyde-3-phos	271.47	254.67	281.36	255.22	265.68	27.85	35.08	28.98	30.64	0.12	7.67E-16
Solyc07g022910.2.1	Myosin heavy chain-lil	7.08	8.31	16.15	10.78	10.58	46.2	61.3	68.88	58.79	5.56	7.97E-16
Solyc04g074850.2.1	Multidrug resistance pr	30.73	37.68	70.58	56.8	48.95	233.94	208.02	241.19	227.72	4.65	9.48E-16
Solyc01g107730.2.1	Cyclin (AHRD V1 ***	24.58	42.15	20.68	28.12	28.88	0.44	0.29	0.36	0.36	0.01	9.68E-16
Solyc11g018500.1.1	Beta-galactosidase (AI	41.14	43.87	22.25	34.86	35.53	0.31	2.86	0	1.06	0.03	1.03E-15
Solyc01g095700.2.1	Abscisic acid receptor	133.35	158.25	124.03	86.83	125.61	13.4	9.49	5.6	9.5	0.08	1.05E-15
Solyc06g068440.2.1	Cinnamoyl-CoA reduc	10.3	7.76	10.52	11.77	10.09	45.62	69.26	72.56	62.48	6.19	1.10E-15
Solyc04g055260.2.1	Carboxyl methyltransfe	40.74	43.31	38.92	26.77	37.44	0	5.73	0	1.91	0.05	1.13E-15
Solyc11g011920.1.1	Glutamate decarboxyla	180.9	218.55	264.23	350.49	253.54	4.97	9.07	8.06	7.37	0.03	1.14E-15
Solyc06g006100.2.1	Anthranilate synthase c	15.03	18.57	21.05	26.03	20.17	99.07	89.6	146	111.56	5.53	1.18E-15
Solyc12g006520.1.1	Cycloartenol synthase	45.87	57.79	29.84	28.97	40.62	0.23	1	0.37	0.53	0.01	1.48E-15
Solyc06g066540.1.1	Ethylene-responsive tra	36.92	43.2	37.1	36.26	38.37	2.21	1.67	2.04	1.97	0.05	1.48E-15
Solyc05g012380.2.1	Glucan endo-1 3-beta-g	87.74	74.33	46.01	67.11	68.8	3.41	3.71	2.58	3.23	0.05	1.78E-15
Solyc07g053970.2.1	Two-pore calcium cha	16.14	21.18	18.51	15.01	17.71	1.15	0.94	0.71	0.93	0.05	1.89E-15
Solyc06g060840.1.1	Oleosin (AHRD V1 **	3.91	13.92	28.83	11.75	14.6	528.64	678.14	432.67	546.48	37.42	2.06E-15
Solyc04g082630.2.1	Glyceraldehyde-3-phos	154.76	129.61	120.91	120.12	131.35	10.39	17.13	17.3	14.94	0.11	2.75E-15
Solyc09g083400.2.1	Os02g0200800 protein	12.42	12.7	10.1	8.07	10.82	0	0.41	0	0.14	0.01	2.93E-15
Solyc12g099650.1.1	Photosystem II 5 kDa p	223.38	231.25	259.44	239.02	238.27	29.92	22.63	17.81	23.45	0.1	3.01E-15
Solyc07g056170.2.1	Subtilisin-like proteas	10.13	8.26	13.76	10.6	10.69	0.21	0.49	0	0.23	0.02	3.10E-15
Solyc12g099930.1.1	Serine-glyoxylate amin	121.44	128.09	151.99	92.16	123.42	9.94	12.97	11.89	11.6	0.09	3.46E-15
Solyc04g014530.1.1	Ethylene responsive tra	25.37	30.86	22.62	21.53	25.1	0.68	1.38	0.28	0.78	0.03	3.66E-15
Solyc02g083680.1.1	Multi-sensor hybrid his	6.75	8.14	7.08	5.51	6.87	0.24	0.18	0.22	0.21	0.03	4.07E-15
Solyc03g083400.2.1	CCT motif family prot	151.54	135.3	78.96	111.24	119.26	3.09	6.23	3.56	4.29	0.04	4.69E-15
Solyc05g007060.2.1	Bifunctional N-succiny	87.49	100.06	77.12	57.47	80.53	373.9	421.21	321.62	372.24	4.62	4.84E-15
Solyc03g031800.2.1	Xyloglucan endotransg	24.74	34.32	13.15	29.32	25.38	0.18	0	0.22	0.13	0.01	5.20E-15
Solyc07g006910.2.1	Solute carrier family 3	45.01	43.14	29.5	29.11	36.69	1.09	4.07	2.1	2.42	0.07	5.39E-15
Solyc03g005760.1.1	Chlorophyll a-b bindin	729.87	655.71	556.44	604.12	636.53	93.47	67	48.38	69.62	0.11	5.63E-15
Solyc10g085870.1.1	UDP-glucosyltransfera	0.68	2.29	1.92	1.92	1.7	342.56	322.66	385.77	350.33	205.77	5.95E-15
Solyc08g068390.2.1	Fatty acid oxidation co	19.3	27.58	27.59	28.74	25.8	2.02	2.19	1.66	1.96	0.08	6.09E-15
Solyc03g032020.2.1	Hydroxy-methylglutary	12.01	9.73	23.56	11.94	14.31	64.92	60.25	90.14	71.77	5.02	6.65E-15
Solyc03g114030.2.1	Nucleobase ascorbate t	179.98	200.14	179.34	188.94	187.1	19.35	23.71	21.66	21.57	0.12	7.34E-15
Solyc01g086920.2.1	LRR receptor-like seri	1025.95	966.47	1281.16	2231.66	1376.31	1.29	1.3	4.37	2.32	0	7.68E-15
Solyc10g007690.2.1	Chlorophyll a-b bindin	199.12	187.07	132.37	129.08	161.91	18.48	16.09	9.08	14.55	0.09	8.97E-15
Solyc03g114730.2.1	Os03g0169000 protein	83.52	92.77	86.24	103.77	91.57	4.48	11.93	10.87	9.09	0.1	9.24E-15
Solyc04g005670.1.1	Kelch-like protein 17 (	12.38	10.29	16.53	19.58	14.7	79.75	65.84	86.34	77.31	5.26	9.31E-15
Solyc09g008060.2.1	UDP-glucosyltransfera	8.3	7.03	12.89	13.97	10.55	0	0	0	0	0	9.54E-15
Solyc03g115940.2.1	Dof zinc finger protein	30.09	34.27	40	44.98	37.34	3.93	3.32	2.61	3.29	0.09	9.64E-15
Solyc01g096370.2.1	BHLH transcription fa	9.65	6.25	7.77	6.21	7.47	0	0.12	0	0.04	0.01	1.01E-14

Solyc04g081910.2.1	Calcium-dependent pro	93.4	102.69	157.01	116.2	117.33	13.38	9.37	15.43	12.73	0.11	1.04E-14
Solyc04g074640.2.1	L-ascorbate peroxidase	15.83	11.37	9	14.29	12.62	0.15	0.31	0.38	0.28	0.02	1.09E-14
Solyc05g054090.2.1	Unknown Protein (AH	4437.35	4079.2	3197.55	1926.17	3410.07	141.88	53.39	76.32	90.53	0.03	1.13E-14
Solyc08g061610.2.1	Copper-exporting P-ty	21.23	21.58	26.26	21.58	22.66	1.43	3.06	0.51	1.67	0.07	1.15E-14
Solyc03g083900.2.1	Laccase-22 (AHRD V1	1.89	1.88	2.23	2.99	2.25	18.78	39.07	26.83	28.23	12.56	1.42E-14
Solyc01g067510.2.1	Receptor-like kinase (A	13.09	9.6	8.14	16.01	11.71	0	0.14	0	0.05	0	1.74E-14
Solyc03g025720.2.1	Long-chain-fatty-acid-	58.42	63.87	86.45	46.48	63.8	311.96	251.55	325.41	296.31	4.64	1.96E-14
Solyc03g078800.2.1	Glucosyltransferase-2 (	26.48	20.14	19.6	17.71	20.98	0.39	1.37	0.72	0.83	0.04	2.16E-14
Solyc09g010700.2.1	CM0545.320.nc protei	19.32	23.27	13.67	19.76	19.01	0	0.97	0.59	0.52	0.03	2.17E-14
Solyc08g005650.2.1	Cytochrome P450	22.77	38.33	40.99	21.9	31	1.16	0.42	0.65	0.74	0.02	2.22E-14
Solyc03g097470.2.1	3-oxoacyl- (AHRD V1	39.86	33.16	30.45	38.67	35.53	190.25	153.81	363.94	236	6.64	2.36E-14
Solyc04g064620.2.1	RAG1-activating prote	50.69	52.27	41.13	40.21	46.08	1.52	1.53	0.53	1.19	0.03	2.41E-14
Solyc12g056220.1.1	Aquaporin (AHRD V1	421.85	473.17	364.38	384.24	410.91	37.62	62.31	27.42	42.45	0.1	2.68E-14
Solyc02g088230.2.1	Xenotropic and polytro	8.15	8.81	6.87	5.53	7.34	0.27	0.47	0.41	0.38	0.05	2.68E-14
Solyc01g106820.2.1	Peptidase M50 family	1.12	1.5	3.71	2.06	2.1	14.5	28.52	32.74	25.25	12.04	3.04E-14
Solyc01g111250.2.1	Phosphatidylinositol-sp	3.93	3.13	4.39	2.93	3.59	143.19	92.01	141.02	125.41	34.88	3.07E-14
Solyc11g012860.1.1	Filament-like plant pro	65.28	67.25	56.94	62.82	63.07	7.75	6.45	3.93	6.04	0.1	3.21E-14
Solyc01g100040.2.1	Integrin-linked kinase-	47.86	44.19	43.36	34.23	42.41	3.77	3.65	3.2	3.54	0.08	3.23E-14
Solyc07g052690.2.1	Beta-amylase (AHRD	7.79	8.05	10.13	5.51	7.87	35.07	43.77	28.88	35.91	4.56	3.33E-14
Solyc05g056050.2.1	Chlorophyll a-b bindin	72.38	65.61	43.8	46.47	57.07	4.75	3.26	1.86	3.29	0.06	3.40E-14
Solyc02g089250.2.1	Pollen Ole e 1 allergen	14.4	18.67	30	27.92	22.75	0.43	0.29	0.35	0.36	0.02	3.45E-14
Solyc12g013690.1.1	Monooxygenase FAD-	14.88	16.98	16.98	20.13	17.24	1180.54	789.5	1155.57	1041.87	60.42	3.94E-14
Solyc12g009920.1.1	Glucosyltransferase (A	16.41	20.23	21.6	12.06	17.57	0.26	0.26	0.31	0.28	0.02	4.21E-14
Solyc10g079070.1.1	Transcription factor (A	27.34	28.71	23.44	28.87	27.09	2.06	1.93	2.17	2.05	0.08	4.23E-14
Solyc09g007940.2.1	Adenosine kinase (AH	25.73	19.63	13.69	28.19	21.81	0.47	0.63	0.58	0.56	0.03	4.55E-14
Solyc06g068620.2.1	Unknown Protein (AH	103.79	125.26	74.72	63.45	91.8	3.55	2.87	0.87	2.43	0.03	5.36E-14
Solyc09g064470.2.1	Galactosyltransferase-l	75.31	77.89	119	112.73	96.23	7.24	6.99	3.97	6.07	0.06	5.55E-14
Solyc06g048470.2.1	COP1-Interacting Prot	21.87	26.65	24.44	28.87	25.46	1.82	3.37	1.81	2.33	0.09	5.66E-14
Solyc05g009270.2.1	Fatty acid elongase 3-k	11.86	17.79	8.02	10.56	12.06	0.22	0.11	0.4	0.24	0.02	5.80E-14
Solyc02g088270.2.1	Genomic DNA chromo	1.05	1.17	2.79	5.2	2.55	1572.2	1285.47	1515.74	1457.8	571.13	5.91E-14
Solyc02g084390.2.1	Kinesin like protein (A	34.02	33.62	27.93	33.58	32.29	3.45	2.79	2.57	2.94	0.09	6.15E-14
Solyc11g013270.1.1	Os01g0841200 protein	8.22	9.64	7.45	6.74	8.01	0.1	0.19	0.12	0.14	0.02	6.16E-14
Solyc02g070260.2.1	Protein phosphatase 1	25.99	33.73	38.67	36.76	33.79	3.31	4.54	4.63	4.16	0.12	6.34E-14
Solyc06g011370.2.1	Chaperone protein clpl	9.05	10.21	11.97	15.92	11.79	51.16	68.32	64.61	61.36	5.21	6.49E-14
Solyc08g076720.2.1	Uncharacterized ABC	19.35	23.71	19.74	23.74	21.63	1.86	2.56	1.93	2.12	0.1	6.49E-14
Solyc02g093980.2.1	Os12g0236050 protein	17.51	20.35	14	12.75	16.15	1.79	1.71	1.73	1.74	0.11	6.93E-14
Solyc11g069190.1.1	Auxin response factor	93.68	118.8	120.87	130.44	115.95	14.51	12.51	18.95	15.32	0.13	7.05E-14
Solyc08g076620.1.1	Xylanase inhibitor (Fra	6.04	5.91	10.69	5.98	7.16	0	0	0	0	0	7.23E-14
Solyc06g011400.2.1	ATP-dependent chaper	14.56	14.29	24.38	24.65	19.47	70.39	92.71	108.73	90.61	4.65	7.99E-14
Solyc12g011450.1.1	Chlorophyll a-b bindin	68.7	83.21	70.22	31.92	63.51	1.2	0.81	0.98	1	0.02	8.04E-14
Solyc08g016500.2.1	Potassium channel (AH	0.29	0.34	0.46	0.27	0.34	4.68	5.27	3.06	4.34	12.75	8.43E-14
Solyc12g056800.1.1	Oxidoreductase family	36.55	37.25	49.59	36.19	39.89	1.77	5.21	1.09	2.69	0.07	8.68E-14
Solyc04g082970.2.1	Os03g0859900 protein	8.89	7.63	5.15	7.5	7.29	0	0	0.31	0.1	0.01	9.21E-14
Solyc02g079170.2.1	NADH dehydrogenase	3.83	3.11	7.45	7.98	5.59	28.61	23.43	40.18	30.74	5.5	9.46E-14
Solyc01g112080.2.1	LysM-domain GPI-anc	43.66	33.26	35.99	39.65	38.14	1.85	2.49	0.95	1.76	0.05	9.73E-14
Solyc03g097050.2.1	Cellulose synthase-like	1.33	1.85	2.46	5.08	2.68	10.19	13.96	38.83	20.99	7.83	9.73E-14
Solyc12g089240.1.1	Zinc finger protein CO	3.4	3.2	4.25	1.41	3.06	84.63	83.57	115.88	94.69	30.9	9.74E-14
Solyc01g099190.2.1	Lipoxygenase (AHRD	69.35	99.79	100.85	99.94	92.48	6500.5	4828.23	8202.28	6510.34	70.4	1.06E-13
Solyc04g057940.2.1	U-box domain-contain	2.4	3.17	4.51	4.09	3.54	0.07	0.18	0.22	0.16	0.04	1.08E-13
Solyc08g077060.2.1	Zinc finger protein LSI	251.68	182.58	191.38	338.75	241.1	4.75	8.84	8.52	7.37	0.03	1.08E-13
Solyc05g005920.2.1	Peptide transporter (AI	49.25	57.61	55.58	58.48	55.23	3.56	8.74	2.24	4.85	0.09	1.11E-13
Solyc06g083140.2.1	Auxin-induced in root	10.46	15.04	11.39	12.9	12.45	0.22	0	0.27	0.16	0.01	1.24E-13
Solyc01g102340.2.1	Myb-like transcription	16.07	17.05	24.61	27.54	21.32	0.75	1.13	0.31	0.73	0.03	1.26E-13
Solyc10g085880.1.1	UDP-glucosyltransfera	15.87	39.65	48.73	39.49	35.94	8155.29	6240.4	7798.08	7397.92	205.87	1.43E-13
Solyc03g005770.1.1	Chlorophyll a-b bindin	365.55	343.08	325.27	323.47	339.34	51.51	35.7	30.54	39.25	0.12	1.51E-13
Solyc02g071030.1.1	Chlorophyll a/b bindin	166.42	120.96	133.43	85.95	126.69	8.62	6.47	3.94	6.34	0.05	1.58E-13
Solyc06g068750.2.1	Transferase transferrin	84.52	84.42	65.64	70.95	76.38	7.61	8.42	6.64	7.56	0.1	1.64E-13
Solyc02g087830.2.1	ATP binding / serine-tl	10.97	8.04	5.33	7.76	8.02	0	0	0	0	0	1.65E-13
Solyc01g008710.2.1	Mannan endo-1 4-beta	0	0.44	0.19	1.87	0.62	1113.54	1171.51	1004.82	1096.62	1754.6	1.65E-13
Solyc08g079900.1.1	Subtilisin-like protease	4.84	3.76	6.44	3.86	4.72	0.07	0	0.09	0.05	0.01	1.76E-13
Solyc04g050170.2.1	Receptor like kinase, F	2.47	2.14	2.77	3.59	2.74	0	0	0	0	0	1.82E-13
Solyc09g009990.2.1	Transposon protein Mu	37.8	33.64	38.34	36.7	36.62	2.82	4.89	3.18	3.63	0.1	1.92E-13
Solyc09g005180.2.1	FAD-binding domain-c	9	11.15	8.33	7.9	9.09	0.28	0.46	0	0.25	0.03	2.33E-13
Solyc06g082810.2.1	Oligoribonuclease (AH	41.7	44.23	43.46	40.4	42.45	3.92	3.03	4.82	3.92	0.09	2.45E-13
Solyc10g078160.1.1	Hydroxyproline-rich gl	15.31	15.38	16.03	21.65	17.09	0.75	0.97	0.65	0.79	0.05	2.45E-13
Solyc03g020080.2.1	Proteinase inhibitor II	306.35	326.6	186.31	164.47	245.93	7.33	4.7	3.27	5.1	0.02	2.50E-13
Solyc03g121040.2.1	Taurine catabolism dio	21.38	20.66	18.74	23.62	21.1	126.34	104.2	143.9	124.81	5.92	2.73E-13
Solyc04g016220.2.1	UDP-glucose glucosylt	221.85	216.15	191.48	262.61	223.02	18.4	40.67	11.88	23.65	0.11	2.80E-13
Solyc03g033660.2.1	GATA transcription fa	14.04	15.27	16.04	11.66	14.25	1.26	0	0.66	0.64	0.04	3.13E-13
Solyc02g093930.2.1	Double-strand-break re	7.25	8.79	8.66	7.19	7.97	0.23	0.46	0.65	0.45	0.06	3.13E-13
Solyc02g082670.2.1	WUSCHEL-related ho	83.72	82.84	82.93	52.26	75.44	266.11	376.89	227.64	290.21	3.85	3.78E-13
Solyc12g057040.1.1	Cryptochrome 1b	45.64	55.2	57.57	45.06	50.87	3.92	8.38	0.9	4.4	0.09	3.78E-13
Solyc05g007830.2.1	Expansin 2 (AHRD V1	19.26	36.4	25.84	38.82	30.08	0.21	1.72	0	0.64	0.02	3.79E-13
Solyc05g005140.2.1	Receptor like kinase, F	10.38	14.19	14.69	11.59	12.71	0.97	0.98	1.41	1.12	0.09	3.85E-13
Solyc03g118340.2.1	ATP-dependent clp pro	17.86	17.8	22	17.21	18.72	64.05	85.48	96.78	82.1	4.39	3.95E-13
Solyc05g009800.2.1	LRR receptor-like seri	9.38	12.34	13.53	15.48	12.68	48.5	59.87	54.31	54.23	4.28	4.08E-13
Solyc01g096230.2.1	ATPase AAA family p	14.08	13.01	16.45	15.17	14.68	99.88	96.7	85.31	93.96	6.4	4.80E-13
Solyc04g005610.2.1	NAC domain transcrip	0.48	0.21	0.56	0.68	0.48	9.85	12.27	6.88	9.67	20.03	4.91E-13
Solyc07g043500.1.1	UDP-glucosyltransfera	49.85	54.33	36.8	70.51	52.87	3.26	4.74	1.04	3.01	0.06	5.09E-13
Solyc02g081600.2.1	Zinc transporter protei	39.25	49.57	41.23	30.4	40.11	4.08	6.09	1.67	3.95	0.1	5.12E-13
Solyc08g083280.2.1	Alcohol dehydrogenase	11.74	8.76	13.65	9.77	10.98	0	0.42	0.17	0.2	0.02	5.20E-13
Solyc05g014390.2.1	ABC transporter C fam	12.63	15.2	14.63	9.27	12.93	99.6	76.63	150.01	108.75	8.41	5.32E-13

Solyc03g114710.2.1	Glucosyltransferase (A	19.06	10.7	19.03	37.95	21.69	84.92	79.32	128.2	97.48	4.5	5.51E-13
Solyc02g078570.2.1	Epoxide hydrolase 3 (A	199.37	245.82	145.57	145.88	184.16	24.29	14.29	12.43	17	0.09	5.58E-13
Solyc07g063930.2.1	Solute carrier family 2	13.26	14.71	51.15	18.53	24.41	76.1	109.68	93.19	92.99	3.81	5.99E-13
Solyc06g071820.2.1	Speckle-type poz prote	326.84	322.26	267.52	193.56	277.54	51.01	30.41	50.51	43.98	0.16	6.04E-13
Solyc08g066840.2.1	Aquaporin-like protein	24.74	48.06	25.11	27.09	31.25	0	0.65	0.26	0.3	0.01	6.26E-13
Solyc01g097510.2.1	Annexin 3 (AHRD V1	192.12	192.52	248.14	113.21	186.5	7.35	11.46	7.18	8.66	0.05	6.62E-13
Solyc10g008470.2.1	Engulfment and cell m	2.21	4.15	4.92	1.73	3.25	109.17	90.33	121.71	107.07	32.92	7.33E-13
Solyc12g049400.1.1	Protein TIFY 3B (AHR	166.73	127.07	195.55	100.74	147.52	2.39	14.71	0.65	5.92	0.04	7.43E-13
Solyc06g064900.2.1	Unknown Protein (AH	1.74	3.09	4.1	0	2.23	47.69	34.66	34.46	38.94	17.44	7.61E-13
Solyc05g024260.2.1	RAG1-activating prote	8.92	14.88	8.15	1.89	8.46	151.25	227.02	80.53	152.93	18.08	7.81E-13
Solyc09g011170.2.1	Uncharacterized basic	28.31	25.51	26.57	22.29	25.67	2.59	2.83	2.47	2.63	0.1	7.94E-13
Solyc02g083340.2.1	Phospholipase D (AHR	11.95	11.58	11.58	12.38	11.87	0.61	1.43	0.38	0.81	0.07	8.06E-13
Solyc11g012040.1.1	Cysteine-type endopep	4.41	6.49	8.25	7.53	6.67	0	0.12	0.15	0.09	0.01	8.24E-13
Solyc02g090510.2.1	Calcium-dependent pro	10.74	12.17	5.89	8.75	9.39	0.35	0.71	0.54	0.53	0.06	9.65E-13
Solyc04g071800.2.1	Cytochrome P450	42.56	53.3	26.86	37.51	40.06	1.99	4.32	1.54	2.62	0.07	9.84E-13
Solyc09g092130.2.1	Sucrose phosphate syn	19.02	21.61	17.98	15.33	18.48	1.6	2.47	2.03	2.03	0.11	1.02E-12
Solyc04g053120.2.1	4-alpha-glucanotransfe	99.82	82.08	82.59	82.57	86.77	10.14	13.7	10.63	11.49	0.13	1.02E-12
Solyc06g084390.2.1	ER auxin binding prote	43.87	26.9	32.21	41.37	36.09	132.33	186.17	170	162.83	4.51	1.07E-12
Solyc04g077020.2.1	Tubulin alpha-3 chain	3743.09	3574.26	2808.34	4048.58	3543.57	337.87	683.23	192.24	404.45	0.11	1.08E-12
Solyc06g036640.2.1	Coatomer (AHRD V1	19.54	24.59	21.88	20.6	21.65	90.81	90.48	42.38	74.56	3.44	1.10E-12
Solyc03g063750.2.1	Exostosin-like glycosy	16.12	16.66	18.42	13.65	16.21	68.49	66.48	84.73	73.23	4.52	1.12E-12
Solyc05g012210.2.1	Ninja-family protein 1	4.98	5.31	7.38	4.02	5.42	0	0	0.14	0.05	0.01	1.15E-12
Solyc03g116230.2.1	Agglutinin isolectin I (	666.17	1065.98	415.98	403.53	637.91	4056.63	3737.24	2951.35	3581.74	5.61	1.16E-12
Solyc01g106330.2.1	Glutamyl-tRNA(Gln) a	68.1	76.36	38.48	61.21	61.04	4.39	5.47	3.6	4.49	0.07	1.22E-12
Solyc07g051880.1.1	Protein serine/threonin	0.2	1.44	0.95	0.29	0.72	145.39	99.62	188.44	144.48	200.67	1.28E-12
Solyc07g006500.2.1	Alpha alpha-trehalose-	0.68	1.34	3.19	2.34	1.89	39.08	58.17	23.67	40.31	21.35	1.31E-12
Solyc11g007250.1.1	Protein kinase like pro	7.17	4.98	4.06	5.48	5.42	0	0	0	0	0	1.31E-12
Solyc03g019670.2.1	Phosphoesterase family	42.89	52.13	52.09	59.61	51.68	3.85	7.87	3.86	5.19	0.1	1.35E-12
Solyc11g066670.1.1	UDP-glucosyltransfera	50.47	43.28	57.54	82.15	58.36	2.73	3.68	1.96	2.79	0.05	1.35E-12
Solyc10g018670.1.1	DNA-3-methyladenine	63.33	52.84	73.16	54.12	60.86	0	0	0	0	0	1.35E-12
Solyc05g016310.1.1	Aspartic proteinase ne	0.13	0.33	0	0.89	0.34	5.06	5.2	17.27	9.18	27.19	1.35E-12
Solyc02g068590.2.1	Potassium transporter (	11.96	14.42	18.33	16.92	15.41	54.31	67.37	55.67	59.12	3.84	1.63E-12
Solyc06g068540.2.1	Transcription regulator	4	3.67	5.31	5.3	4.57	22.11	21.69	18.48	20.76	4.54	1.64E-12
Solyc07g049370.2.1	Glucan endo-1 3-beta-g	11.53	16.78	13.94	25.67	16.98	0.23	0.11	0.14	0.16	0.01	1.65E-12
Solyc01g104860.2.1	Peroxidase (AHRD V1	4.32	10.24	8.73	7.56	7.71	0	0	0	0	0	1.65E-12
Solyc07g043580.2.1	BHLH transcription fac	15.43	15.98	13.5	18.81	15.93	1.5	0.98	0.79	1.09	0.07	1.75E-12
Solyc02g082740.1.1	Dirigent-like protein 1	227.96	177.37	108.39	132.4	161.53	3.07	4.78	1.37	3.07	0.02	1.84E-12
Solyc03g123740.2.1	Receptor like kinase, R	34.15	33.28	31.96	48.04	36.86	3.6	5.22	4.88	4.57	0.12	1.95E-12
Solyc01g109340.2.1	RING finger protein 5	38.04	44.96	31.55	51.28	41.46	2.88	3.63	1.77	2.76	0.07	1.97E-12
Solyc02g069800.1.1	CXE carboxylesterase	25.33	24.11	46.1	79.71	43.81	167.83	230.71	314.16	237.57	5.42	1.97E-12
Solyc05g018510.2.1	ABC transporter G fam	35.07	51.86	23.81	29.46	35.05	1.42	0.98	0.78	1.06	0.03	1.97E-12
Solyc06g011380.2.1	Chaperone ClpB (AHR	10.89	8.84	12.47	19.03	12.81	52.26	68.89	72.79	64.65	5.05	1.97E-12
Solyc08g006150.2.1	ChaC cation transport	237.09	202.29	208.68	251.02	224.77	25.37	33.1	33.46	30.64	0.14	1.97E-12
Solyc11g067160.1.1	Aldo/keto reductase fa	25.46	22.51	26.92	27.83	25.68	105.66	113.04	146.39	121.7	4.74	1.97E-12
Solyc03g019790.2.1	Alpha-galactosidase (A	14.96	23.92	15.57	29.59	21.01	0.52	0.26	0.16	0.31	0.01	2.10E-12
Solyc04g081300.2.1	Endoglucanase 1 (AHR	123.44	132.22	116.15	172.81	136.16	14.86	26.88	14.48	18.74	0.14	2.12E-12
Solyc02g071260.2.1	Phytochrome E	5.22	5.06	7.18	4.59	5.51	0.14	0.33	0.23	0.23	0.04	2.17E-12
Solyc04g015850.2.1	Unknown Protein (AH	71.22	81.7	65.46	63.35	70.43	5.54	14.3	4.41	8.08	0.11	2.19E-12
Solyc10g008980.2.1	Triose phosphate/phos	206.16	235.56	242.85	276.36	240.23	32.03	33.34	45.96	37.11	0.15	2.32E-12
Solyc12g089220.1.1	Wound responsive pro	9.99	14.63	11.29	5.89	10.45	54.14	62.92	64.86	60.64	5.8	2.47E-12
Solyc07g063740.2.1	Ferredoxin-like (AHRI	21.91	23	20.62	30.99	24.13	1.99	1.81	1.47	1.76	0.07	2.51E-12
Solyc03g095820.2.1	Cell number regulator	15.69	14.57	9.95	15.99	14.05	0.55	0.82	0	0.46	0.03	2.54E-12
Solyc04g074770.2.1	Unknown Protein (AH	49.95	19.01	32.62	28.88	32.62	0	0	0	0	0	2.69E-12
Solyc02g083670.2.1	Histidine kinase (AHR	43.4	35.54	36.87	52.8	42.15	0	0.71	0.86	0.52	0.01	2.81E-12
Solyc03g118510.2.1	Receptor like kinase, R	22.62	31.66	29.75	40.88	31.23	1.29	4.55	0.49	2.11	0.07	3.12E-12
Solyc09g083130.2.1	Peptidase S9 prolyl oli	20.79	24.62	22.9	22.11	22.61	1.25	3.01	2.9	2.39	0.11	3.19E-12
Solyc06g063070.2.1	Ethylene responsive tra	249.03	286.64	258.12	334.49	282.07	1067.83	954.09	1689.9	1237.27	4.39	3.25E-12
Solyc07g066010.2.1	Amino acid permease (	10.54	9.76	7.15	11.43	9.72	80.82	58.93	52.73	64.16	6.6	3.57E-12
Solyc03g096840.2.1	Seed specific protein B	1333.56	1232.53	1173.04	1574.52	1328.41	143.4	249.59	88.87	160.62	0.12	3.59E-12
Solyc08g041770.2.1	Os04g0226400 protein	20.41	32.11	26.45	26.81	26.45	1.77	3.27	1.27	2.1	0.08	3.61E-12
Solyc03g116730.2.1	Stearoyl-CoA 9-desatu	17.68	16.34	21.06	17.32	18.1	0.69	2.49	0.84	1.34	0.07	3.61E-12
Solyc03g114930.2.1	Photosystem II oxygen	36.86	35.18	24.26	24.93	30.31	1.29	3.26	1.86	2.14	0.07	3.63E-12
Solyc05g005930.1.1	UDP-glucosyltransfera	37.19	39.01	37.96	44.26	39.6	4.77	4.69	1.17	3.54	0.09	3.75E-12
Solyc10g018300.1.1	Transketolase 1 (AHRI	117.18	110.7	106.47	84.26	104.65	13	18.18	10.85	14.01	0.13	3.80E-12
Solyc10g078410.1.1	F-box family protein (A	27.07	42.3	16.56	21.3	26.81	0.14	0	0.34	0.16	0.01	3.80E-12
Solyc01g094250.2.1	Short-chain dehydroge	22.68	32.84	25.05	31.2	27.94	2.83	2.02	1.02	1.96	0.07	3.82E-12
Solyc06g065670.2.1	ATP-binding cassette t	18.54	28.29	12.26	15.83	18.73	0.49	0.11	0.37	0.32	0.02	3.82E-12
Solyc04g055000.2.1	Tetratricopeptide repea	8.41	10.38	4.9	6.98	7.67	0.18	0.37	0.11	0.22	0.03	3.94E-12
Solyc03g097100.1.1	Calmodulin (AHRD V	6.03	5.37	4.75	3.41	4.89	29.57	44.58	37.93	37.36	7.64	3.97E-12
Solyc08g016670.2.1	Calcyclin-binding prot	61.77	52.58	50.24	51.75	54.09	152.87	279.78	248.83	227.16	4.2	4.06E-12
Solyc06g053620.2.1	Calcium-dependent pro	7.8	6.1	6.97	12.38	8.31	584.76	447.79	484.9	505.82	60.85	4.10E-12
Solyc09g082460.2.1	Homocysteine s-methy	0.39	0.35	2.3	0.28	0.83	147.32	93.56	336.3	192.39	231.8	4.10E-12
Solyc11g072820.1.1	Kinesin-5 (AHRD V1	15.35	15.56	13.6	21	16.38	0.81	2.4	0.37	1.19	0.07	4.37E-12
Solyc01g100320.2.1	Thioredoxin/protein di	363.94	329.12	283.01	365.23	335.32	1260.36	1285.1	1307.1	1284.19	3.83	4.49E-12
Solyc02g085380.2.1	Integral membrane pro	16.74	16.62	8.87	18.53	15.19	0	0.99	0	0.33	0.02	4.71E-12
Solyc12g006370.1.1	Amine oxidase family	4.18	6	5.57	7.82	5.89	24.2	27.15	37.23	29.53	5.01	5.17E-12
Solyc03g006100.2.1	Receptor like kinase, R	6.86	10.91	7.92	4.65	7.58	0.12	0.24	0.29	0.22	0.03	5.66E-12
Solyc11g008810.1.1	Beta-hexosaminidase b	13.38	10.49	10	19.49	13.34	0	0	0	0	0	5.82E-12
Solyc06g074730.2.1	ARGONAUTE 1 (AHI	11.28	14.62	21	15.58	15.62	2.11	1.27	1.94	1.77	0.11	6.06E-12
Solyc10g084540.1.1	Pentatricopeptide repea	0.08	0.15	0.2	0.06	0.12	1.91	1.96	1.21	1.69	13.82	6.09E-12
Solyc01g097950.2.1	Cysteine synthase (AH	13.85	11.78	10.57	17.28	13.37	0.16	0.66	0.4	0.41	0.03	6.38E-12
Solyc04g011770.2.1	Unknown Protein (AH	64.93	57.79	41.67	36.74	50.28	0.84	4.23	0.52	1.86	0.04	6.38E-12

Solyc11g011210.1.1	Gibberellin regulated p	3.4	5.45	8.02	6.73	5.9	482.43	926.63	1048.39	819.15	138.84	6.42E-12
Solyc05g014560.2.1	3-oxoacyl-reductase (A	19.2	24.9	13.01	14.45	17.89	1.16	1	0	0.72	0.04	6.50E-12
Solyc09g065560.2.1	Sulfate transporter (AH	1	1.69	0.35	0.57	0.9	58.56	46.82	22.85	42.74	47.36	6.95E-12
Solyc05g050220.2.1	G-box binding factor 3	4.59	3.1	2.99	0.9	2.9	24.03	19.34	33.76	25.71	8.88	7.65E-12
Solyc09g091470.2.1	3-ketoacyl CoA thiolas	120.76	122.72	155.33	152.59	137.85	950.34	793.1	1032.96	925.47	6.71	8.63E-12
Solyc02g093520.2.1	Copine-like protein (A	43.62	35.97	42.15	51.11	43.21	160.22	153.86	195.38	169.82	3.93	8.87E-12
Solyc01g107170.2.1	Zinc finger protein (AH	7.42	4.91	9.01	4.8	6.54	109.34	108.55	155.81	124.57	19.06	9.51E-12
Solyc12g088250.1.1	Serine carboxypeptidas	5.4	6.71	7.38	2.82	5.58	0	0	0	0	0	9.98E-12
Solyc03g043770.2.1	Receptor like kinase, R	4.85	5.27	5.08	3.24	4.61	0	0	0.13	0.04	0.01	1.00E-11
Solyc01g109180.2.1	Long-chain-fatty-acid-(	172.42	207.1	106.49	103.5	147.38	8.32	4.24	3.87	5.48	0.04	1.01E-11
Solyc03g114850.2.1	Squamosa promoter bi	42.66	41.06	28.38	29.82	35.48	3.81	3.84	3.45	3.7	0.1	1.02E-11
Solyc02g086300.2.1	Glutamine amidotransf	28.67	27.3	12.91	24.42	23.32	0.62	0.5	3.21	1.44	0.06	1.18E-11
Solyc09g064480.1.1	Galactosyltransferase-l	43.34	48.47	66.97	59.14	54.48	4.65	3.13	2.28	3.35	0.06	1.18E-11
Solyc03g005060.2.1	Os12g0236050 protein	3.47	3.47	3.59	3.79	3.58	0.05	0.2	0.18	0.14	0.04	1.19E-11
Solyc02g078890.1.1	Unknown Protein (AH	230.39	167.11	210.75	109.11	179.34	0.82	12.41	2.01	5.08	0.03	1.19E-11
Solyc01g090340.2.1	Ethylene responsive tra	23.99	28.29	45.99	61.93	40.05	0	0	0.3	0.1	0	1.25E-11
Solyc06g069190.2.1	Nucellin-like aspartic p	35.27	38.69	39.4	53.87	41.81	2.08	5.16	5	4.08	0.1	1.25E-11
Solyc05g005550.2.1	BURP domain-contain	200.11	258.62	128.98	178.49	191.55	8.38	11.95	2.7	7.68	0.04	1.27E-11
Solyc06g053950.1.1	Heat stress transcriptio	58.81	79.2	114.03	47.74	74.94	702.38	500.49	532.49	578.45	7.72	1.27E-11
Solyc02g088610.2.1	ATP-dependent chaper	21.65	22.98	22.53	26.35	23.38	90.19	181.55	189.91	153.88	6.58	1.34E-11
Solyc01g090700.2.1	Enoyl-CoA-hydratase (	40.89	40.3	38.82	35.25	38.81	128.71	163.53	94.59	128.94	3.32	1.38E-11
Solyc07g006600.1.1	GDU1 (AHRD V1 *-*-	7.09	11.36	13.39	8.03	9.97	53.59	87.86	45.06	62.17	6.24	1.38E-11
Solyc02g085950.2.1	Ribulose bisphosphate	314.55	197.88	178.02	176.32	216.69	5.01	11.58	19.17	11.92	0.06	1.53E-11
Solyc01g090560.2.1	Ethylene-responsive tr	18.71	17.93	37.93	22.4	24.24	0.39	0.2	0.24	0.28	0.01	1.53E-11
Solyc07g048090.1.1	Fasciclin-like arabinog	14.72	18.14	22.05	37.25	23.04	62.91	101.06	36.54	66.84	2.9	1.54E-11
Solyc09g009970.2.1	Rho GTPase activating	10.33	11.3	16.01	8.1	11.44	0.5	0.71	0.37	0.53	0.05	1.61E-11
Solyc05g005760.2.1	NHL repeat-containing	57.94	53.32	38.22	59.98	52.36	3.51	9.76	3.4	5.56	0.11	1.75E-11
Solyc01g008720.1.1	Mannan endo-1 4-beta	0	0	0.19	0.23	0.11	26.91	20.59	33.07	26.86	255.78	1.76E-11
Solyc03g118120.2.1	Transferase transferrin	300.92	330.14	190.14	196.05	254.31	21.04	21.22	15.8	19.35	0.08	1.77E-11
Solyc04g063230.2.1	Dehydration-responsiv	177.07	180.42	151.82	189.54	174.71	19.89	34.53	21.88	25.43	0.15	1.81E-11
Solyc02g081980.2.1	Ectonucleoside triphos	10.62	15.25	9.13	12.87	11.97	0.25	0.87	0.6	0.57	0.05	1.81E-11
Solyc11g012260.1.1	Membrane bound O-ac	3.56	4.75	7.52	3.98	4.95	54.98	38.42	45.76	46.39	9.37	1.83E-11
Solyc01g099100.2.1	Long-chain-fatty-acid c	38.05	42.03	44.89	38.56	40.88	206.39	188.42	198.04	197.62	4.83	1.85E-11
Solyc04g005540.2.1	Cc-nbs-lrr, resistance p	13.31	15	16.36	17.9	15.64	2.04	1.86	2.32	2.07	0.13	1.89E-11
Solyc11g066720.1.1	Bifunctional polymyxi	108.93	92.55	77.43	125.14	101.01	10.74	17.5	7.28	11.84	0.12	1.97E-11
Solyc02g021680.2.1	WRKY transcription fa	0	0	0	0	0	24.14	19.98	16.8	20.31	2030.67	2.01E-11
Solyc09g075820.2.1	Solute carrier family 2	7.3	7.51	8.02	4.27	6.77	0.31	0.1	0.12	0.18	0.03	2.12E-11
Solyc00g145170.1.1	Proteinase inhibitor II (	740.44	586.32	374.46	351.73	513.24	8.95	13.53	5.95	9.48	0.02	2.17E-11
Solyc02g070580.1.1	Cytochrome p450	1.31	0.7	2.33	1.3	1.41	10.19	8.67	23.22	14.03	9.95	2.17E-11
Solyc02g078670.2.1	COP1-Interacting Prot	45.3	45.56	46.77	52.33	47.49	3.39	8.21	3.65	5.08	0.11	2.18E-11
Solyc09g092390.2.1	Adenosylhomocysteina	511.75	499.07	679.19	814.5	626.13	1907.45	1857.51	2730.31	2165.09	3.46	2.43E-11
Solyc04g009860.2.1	1-aminocyclopropane-	29.82	41.34	26.44	21.99	29.9	3.21	1.91	1.79	2.3	0.08	2.74E-11
Solyc08g078450.2.1	Exostosin family prote	0	0	0	0	0	2.98	3.21	4.64	3.61	361	2.76E-11
Solyc01g100930.2.1	GDSL esterase/lipase A	22.88	27.62	21.76	18.06	22.58	1	2.02	0.35	1.12	0.05	2.83E-11
Solyc02g082930.2.1	Endochitinase (Chitina	5.59	5.68	8.16	6.02	6.36	36.32	31.86	30.89	33.02	5.19	2.94E-11
Solyc07g045320.2.1	Unknown Protein (AH	8.21	8.88	4.18	5.46	6.68	30.16	36.45	26.19	30.93	4.63	2.99E-11
Solyc12g094640.1.1	Glyceraldehyde-3-phos	11.85	12.63	14.15	6.41	11.26	0	0.95	1.74	0.9	0.08	3.10E-11
Solyc01g005990.2.1	Non-specific lipid-tran	46.93	42.98	34.5	31.27	38.92	3.57	3.6	1.35	2.84	0.07	3.11E-11
Solyc08g079880.1.1	Subtilisin-like protease	3.84	3.42	5.36	2.47	3.77	0	0.07	0	0.02	0.01	3.11E-11
Solyc02g090070.2.1	Phototropic-responsive	16.39	16.12	22.56	21.9	19.24	0.98	3.38	2.01	2.12	0.11	3.30E-11
Solyc08g081890.2.1	Multidrug resistance pr	8.42	7.62	6.15	5.61	6.95	27.15	25.72	25.26	26.04	3.75	3.48E-11
Solyc02g092210.1.1	Alpha-6-galactosyltran	0	0.66	0.88	0.42	0.49	8.4	17.19	9.73	11.77	24.03	3.48E-11
Solyc02g090620.2.1	Unknown Protein (AH	9.81	10.96	10.89	4.9	9.14	0.31	0.16	0	0.16	0.02	3.53E-11
Solyc09g075890.2.1	Pyrimidine 5&apos-nu	129.63	237.37	180.8	118.01	166.45	6.22	10.04	7.13	7.8	0.05	3.57E-11
Solyc03g118290.2.1	Auxin response factor	70.41	65.44	82.98	89.02	76.96	267.63	243.02	293.01	267.89	3.48	3.62E-11
Solyc02g079190.2.1	Auxin F-box protein 5	54.08	55.92	96.63	77.92	71.14	3.63	5.67	6.35	5.22	0.07	3.86E-11
Solyc12g088910.1.1	Unknown Protein (AH	11.99	12.05	6.85	9.85	10.19	0.47	0.47	0	0.31	0.03	3.86E-11
Solyc01g109030.2.1	B3 domain-containing	113	93.83	75.23	85.73	91.95	13.01	12.35	10.65	12	0.13	4.01E-11
Solyc09g015040.1.1	Os08g0119500 protein	3.36	4.83	4.57	6.57	4.83	15.33	23.88	38.06	25.76	5.33	4.03E-11
Solyc08g082170.2.1	Glycoside hydrolase fa	88.67	107.77	81.32	112.26	97.5	20.16	13.55	34	22.57	0.23	4.08E-11
Solyc09g009710.2.1	General transcription f	8.81	7.71	7.82	9.58	8.48	0.41	1.36	0.65	0.81	0.1	4.12E-11
Solyc04g051280.2.1	F16G16.9 protein (Frag	3.34	3.19	5.49	2.7	3.68	18.31	18.18	12.2	16.23	4.41	4.13E-11
Solyc08g077460.2.1	S-ribonuclease binding	188.46	178.5	235.73	126.2	182.22	674.55	627.84	438.86	580.42	3.19	4.13E-11
Solyc03g062710.2.1	Aldose 1-epimerase fa	8.89	5.78	6.65	7.98	7.33	0.14	0.14	0.17	0.15	0.02	4.78E-11
Solyc12g014630.1.1	Cortical cell-delineatin	111.01	223	75.59	124.72	133.58	0.51	0.52	0.63	0.55	0	5.11E-11
Solyc07g018070.2.1	Heat shock protein-rela	3.42	5.28	4.49	5.66	4.71	0.47	0.05	0.13	0.22	0.05	5.14E-11
Solyc03g120500.2.1	Auxin responsive prote	90.33	130.9	104.05	113.72	109.75	11.27	18.88	6.57	12.24	0.11	5.19E-11
Solyc01g111830.2.1	24-sterol C-methyltran	156.2	157.66	215.93	225.88	188.92	21.53	35.04	29.18	28.58	0.15	5.30E-11
Solyc08g076730.2.1	TPR domain protein (A	144.79	146.49	114.31	98.24	125.96	12.66	27.85	11.02	17.18	0.14	5.30E-11
Solyc05g013060.2.1	Unknown Protein (AH	3.74	4.16	5.24	6.62	4.94	24.76	28.21	28.1	27.02	5.47	5.45E-11
Solyc01g109040.2.1	Plastoquinol-plastocya	218.09	177.6	133.15	153.47	170.58	27.75	22.21	21.11	23.69	0.14	5.69E-11
Solyc04g081870.2.1	Expansin (AHRD V1 *	29.41	31.41	12.67	19.53	23.25	0.41	0.42	0.25	0.36	0.02	5.75E-11
Solyc03g123600.2.1	Alanine aminotransfer	10.2	7.99	8.34	7.89	8.6	65.61	54.09	56.17	58.62	6.81	5.76E-11
Solyc01g079580.2.1	DNAJ heat shock N-te	19.67	22.17	30.27	40.38	28.12	0.52	0.87	0.53	0.64	0.02	5.77E-11
Solyc06g009190.2.1	Pectinesterase (AHRD	85	102.57	130.87	249.06	141.88	0	0.38	0.35	0.24	0	5.88E-11
Solyc06g052050.2.1	Heat shock protein (AH	1.28	0.85	1.13	0.3	0.89	6.19	8.32	5.7	6.74	7.57	5.94E-11
Solyc07g051870.1.1	Protein serine/threonin	0.8	1.07	0.24	0	0.53	19.22	16.13	25	20.12	38.14	6.23E-11
Solyc10g086620.1.1	Tropinone reductase (A	30.58	52.93	37.21	27.59	37.08	1.75	3.73	1.91	2.46	0.07	6.34E-11
Solyc07g009380.2.1	Xyloglucan endotransg	7.65	9.57	2.26	3.04	5.63	26.07	52	12.81	30.29	5.38	6.36E-11
Solyc00g136560.2.1	Undecaprenyl pyropho	3.58	1.77	5.16	1.12	2.91	18.3	30.44	14.99	21.24	7.31	6.54E-11
Solyc09g075860.2.1	Lipoxygenase (AHRD	26.82	27.15	23.05	30.5	26.88	2.12	4.1	2.68	2.97	0.11	6.54E-11
Solyc06g084460.2.1	Shikimate dehydrogen	30.62	25.84	29.67	24.92	27.76	2.75	4.46	0.96	2.72	0.1	6.66E-11

Solyc10g008540.2.1	Unknown Protein (AH	28.45	27.52	29.19	18.08	25.81	0.33	3.36	1.23	1.64	0.06	6.73E-11
Solyc12g044330.1.1	Aquaporin (AHRD V1	22.52	17.92	38.13	35.6	28.54	0.43	0.86	0	0.43	0.02	6.73E-11
Solyc07g065410.1.1	Unknown Protein (AH	5.17	17.84	6.87	6.4	9.07	134.29	132.29	59.7	108.76	11.99	6.84E-11
Solyc05g056060.2.1	Ubiquitin-like protein	626.73	574.65	406.55	416.94	506.22	99.23	81.78	45.98	75.66	0.15	6.84E-11
Solyc02g085340.1.1	GRAS family transcrip	14.17	14.73	15.69	16.22	15.2	1.57	1.59	0.97	1.38	0.09	7.12E-11
Solyc07g005960.2.1	Serine carboxypeptidas	21.09	27.97	26.56	26.03	25.41	2.86	4.04	2.67	3.19	0.13	7.17E-11
Solyc02g088100.2.1	Expansin (AHRD V1 *	680.6	642.18	325.6	555.62	551	25.76	60.93	14.19	33.63	0.06	7.19E-11
Solyc07g005480.2.1	Formin 2B (AHRD V1	12.52	13.8	21.19	16.66	16.04	55.89	56.01	36.77	49.56	3.09	7.19E-11
Solyc04g014680.2.1	Cysteine-type endopep	69.91	79.37	92.86	116.27	89.6	7.11	12.85	10.54	10.17	0.11	7.64E-11
Solyc12g010050.1.1	DNA-3-methyladenine	5.13	6.14	4.59	6.5	5.59	0	0	0	0	0	7.65E-11
Solyc04g050920.2.1	Os02g0504100 protein	36.09	35.26	38.82	34.43	36.15	1.49	3.69	2.5	2.56	0.07	8.25E-11
Solyc07g052240.2.1	Laccase-22 (AHRD V1	4.49	8.2	3.86	4.97	5.38	0	0.1	0	0.03	0.01	9.04E-11
Solyc01g096340.2.1	Auxin-induced SAUR-	39.58	50.71	38.92	22.9	38.03	328.03	252.17	226.21	268.8	7.07	9.56E-11
Solyc02g062490.2.1	2-oxoglutarate-depend	7.29	7.46	7.31	8.25	7.58	36.22	33.26	78.87	49.45	6.53	9.63E-11
Solyc02g055440.2.1	Genomic DNA chromo	66.27	75.53	52.71	35.21	57.43	1.45	5.13	1.79	2.79	0.05	9.75E-11
Solyc11g022530.1.1	Transmembrane protei	0.81	1.44	1.53	0.69	1.12	7.84	10.41	7.86	8.7	7.79	9.82E-11
Solyc12g038520.1.1	Squamosa promoter bi	12.66	13.89	13.03	10.62	12.55	1.67	0.72	0.73	1.04	0.08	9.91E-11
Solyc06g063370.2.1	Chlorophyll a-b bindin	379.51	327.53	317.02	274.09	324.54	80.78	37.09	63.2	60.36	0.19	9.93E-11
Solyc01g108780.2.1	Alpha-hydroxynitrile l	7.44	7.73	11.41	8.42	8.75	0	0	0.25	0.08	0.01	1.05E-10
Solyc02g062560.2.1	TIM-barrel enzyme far	3.15	3.89	2.78	3.46	3.32	0	0.07	0.17	0.08	0.02	1.05E-10
Solyc07g065420.1.1	Plant-specific domain	0	0.74	0.65	0.78	0.54	7.17	12.65	4.4	8.07	14.88	1.08E-10
Solyc10g086730.1.1	Fructose-1 6-bisphosph	133.07	131.03	93.59	88.76	111.61	15.6	20.72	8.78	15.03	0.13	1.08E-10
Solyc02g071050.2.1	Purine permease famil	4.25	8.78	7.82	2.89	5.94	39.7	39.07	9.96	29.58	4.98	1.09E-10
Solyc11g061960.1.1	Serine/threonine kinas	6.5	6.3	9.03	6.53	7.09	0.23	0.24	0.29	0.25	0.04	1.10E-10
Solyc03g032010.2.1	Hydroxy-methylglutary	16.53	12.37	22.75	13.36	16.25	52.13	55.57	72.38	60.03	3.69	1.15E-10
Solyc06g083030.2.1	Serine carboxypeptidas	4.22	8.69	9.96	6.91	7.45	0.32	0.22	0.26	0.27	0.04	1.17E-10
Solyc10g045380.1.1	Vacuolar protein sortin	8.39	9.77	9.65	12.79	10.15	0.78	0.79	0.11	0.56	0.06	1.21E-10
Solyc11g009020.1.1	HAD-superfamily hydr	14.37	14.53	11.82	8.32	12.26	0	0.71	0.65	0.45	0.04	1.21E-10
Solyc01g065530.2.1	COBRA-like protein (	8.74	6.06	9.59	13.23	9.41	31.95	29.74	83.04	48.24	5.13	1.23E-10
Solyc08g076820.2.1	BHLH transcription fa	7.22	10.17	3.79	9.36	7.63	0	0	0.4	0.13	0.02	1.24E-10
Solyc04g080040.2.1	UBA/TS-N domain pro	5.19	4.78	6.12	3.99	5.02	0.22	0.15	0	0.12	0.02	1.26E-10
Solyc08g041710.2.1	Unknown Protein (AH	21.95	31.06	26.77	26.69	26.62	2.69	2.91	3.3	2.97	0.11	1.31E-10
Solyc10g080670.1.1	Unknown Protein (AH	25.81	28.78	29.53	22.13	26.56	73.73	108.58	118.02	100.11	3.77	1.32E-10
Solyc02g076690.2.1	Cathepsin B-like cyste	25.37	30.88	22.17	35.5	28.48	2.88	5.03	2.72	3.54	0.12	1.46E-10
Solyc12g036170.1.1	Unknown Protein (AH	43.98	43.87	35.34	36.47	39.91	1.84	5.87	1.88	3.2	0.08	1.47E-10
Solyc11g018610.1.1	Calcium-dependent pro	10.69	9.51	8.61	9.22	9.51	0.63	0.32	0.39	0.45	0.05	1.50E-10
Solyc12g044300.2.1	Acyl-CoA synthetase (	155.08	171.99	117.59	129.21	143.47	20.17	31.92	22.77	24.95	0.17	1.53E-10
Solyc01g099180.2.1	Lipoxygenase (AHRD	2.08	3.5	2	2.73	2.58	242.12	163.41	215.06	206.86	80.26	1.56E-10
Solyc09g007270.2.1	Ascorbate peroxidase (	180.3	157.43	126.83	149.45	153.5	1375.78	1611.87	1420.52	1469.39	9.57	1.57E-10
Solyc10g074540.1.1	Expressed protein (Fra	20.46	26.55	29.33	40.57	29.23	0.46	0.93	0.19	0.53	0.02	1.63E-10
Solyc03g005090.2.1	4-coumarate-coa ligase	10.8	11.06	10.41	10.84	10.78	37.86	40.98	41.96	40.27	3.74	1.79E-10
Solyc01g108400.2.1	Calcium-dependent pro	2.58	3.19	3.83	2.22	2.96	112.39	62.25	113.73	96.12	32.53	1.87E-10
Solyc09g011720.2.1	Unknown Protein (AH	12.22	12.7	12.09	19.96	14.24	1.15	1.54	0.39	1.03	0.07	1.92E-10
Solyc07g054500.2.1	Receptor like kinase, R	10.25	8.23	8.45	13.55	10.12	1.14	0.88	0.58	0.87	0.09	2.10E-10
Solyc11g006990.1.1	Os06g0207500 protein	18.8	26.34	38.4	38.9	30.61	134.86	293.47	215.28	214.54	7.01	2.17E-10
Solyc09g066070.2.1	Homeodomain-contain	0	0	0.36	0	0.09	3.7	3.48	4.4	3.86	42.89	2.18E-10
Solyc03g113680.2.1	Microtubule-associated	30.83	35.21	57.35	58.04	45.36	0.43	2.14	0.42	1	0.02	2.26E-10
Solyc04g014830.1.1	GRAS family transcrip	10.6	10.81	19.81	10.61	12.96	0.29	0.68	0.12	0.36	0.03	2.31E-10
Solyc07g006180.2.1	Tetratricopeptide repea	44.06	41.23	33.87	35.97	38.78	124.5	165.99	124.97	138.49	3.57	2.42E-10
Solyc12g094620.1.1	Catalase (AHRD V1 *	265.62	336.44	358.22	346.57	326.71	1002.29	1200.88	1159.95	1121.04	3.43	2.45E-10
Solyc06g059800.2.1	Protein-P-II uridylyltra	67.15	74.57	61.5	60.41	65.91	12.25	7.92	8.48	9.55	0.14	2.56E-10
Solyc05g026570.2.1	Unknown Protein (AH	61.93	61.11	51.36	42.53	54.23	2.9	9.87	3.12	5.3	0.1	2.56E-10
Solyc08g078070.2.1	Ras-related protein Ra	62.41	54.97	46.93	46.66	52.74	234.1	217.67	205.11	218.96	4.15	2.57E-10
Solyc04g078290.2.1	Cytochrome P450	16.46	14.19	13.2	10.55	13.6	0.62	0.73	1.15	0.83	0.06	2.58E-10
Solyc01g099630.2.1	Xyloglucan endotransg	250.78	257.79	266.52	412.54	296.91	23.51	31.68	24.47	26.55	0.09	2.85E-10
Solyc04g054740.2.1	Inositol-3-phosphate sy	34.34	37.8	38.54	14.06	31.19	0.73	1.26	1.28	1.09	0.03	2.85E-10
Solyc08g067870.1.1	Homology to unknown	3.57	4	4.13	2.62	3.58	14.37	15.08	13.39	14.28	3.99	2.86E-10
Solyc12g009490.1.1	Ethylene-responsive tr	53.55	95.57	37.35	37.66	56.03	0.24	0	0	0.08	0	2.87E-10
Solyc07g063690.1.1	Calcium-binding EF ha	9.84	21.12	22.53	14.73	17.05	0	0	0	0	0	3.01E-10
Solyc01g098640.2.1	UPF0603 protein At1g	82.58	74.91	62.27	59.53	69.82	7.35	13.9	11.52	10.92	0.16	3.04E-10
Solyc05g010320.2.1	Chalcone--flavonone is	37.2	39.57	30.52	42.75	37.51	2.58	4.97	2.02	3.19	0.09	3.07E-10
Solyc05g050010.2.1	1-aminocyclopropane-	0.55	0.62	0.33	0	0.38	39	38.21	44.75	40.65	108.41	3.07E-10
Solyc10g079640.1.1	Iaa-amino acid hydrola	36.79	43.13	67.9	51.27	49.77	0.66	9.96	0.4	3.67	0.07	3.09E-10
Solyc03g007180.1.1	Unknown Protein (AH	12.39	13.82	13.59	16.79	14.15	1.34	2.2	0.52	1.35	0.1	3.10E-10
Solyc06g018100.2.1	Nucellin-like aspartic	24.82	23.18	23.1	27.25	24.59	2.74	3.77	1.38	2.63	0.11	3.18E-10
Solyc02g085590.2.1	Vicilin (Fragment) (AH	2.5	2.82	3.18	2.86	2.84	0	0	0	0	0	3.22E-10
Solyc08g081280.2.1	Cell division protein ki	10.52	11.88	13.16	13.33	12.22	48.1	45.95	55.82	49.96	4.09	3.24E-10
Solyc04g082510.2.1	Serine/threonine-prote	7.11	9.02	10.7	9.81	9.16	36.06	38.54	74.31	49.64	5.42	3.53E-10
Solyc06g062600.2.1	Choline dehydrogenase	16.55	18.75	11.58	5.77	13.16	79.23	57.62	126.8	87.88	6.68	3.53E-10
Solyc05g009330.2.1	COSII_At1g13380 (Fra	69.43	70.09	45.64	68.4	63.39	3.63	8.16	1.37	4.39	0.07	3.58E-10
Solyc04g082720.2.1	Small heat shock prote	35.13	29.36	19.21	21.21	26.23	87.22	131.6	93.12	103.98	3.96	3.64E-10
Solyc07g041280.2.1	Nitrilase 2 (AHRD V1	10.72	8.41	10.94	12.08	10.54	0.44	0.74	0.36	0.51	0.05	3.66E-10
Solyc01g081640.2.1	Legume lectin beta dom	3.15	3.74	2.97	4.01	3.47	0	0.09	0	0.03	0.01	3.71E-10
Solyc06g007240.2.1	BCL-2 binding anthan	13.24	14.1	13.11	8.7	12.29	0.38	0.39	0.23	0.33	0.03	3.73E-10
Solyc06g074000.1.1	Aspartic proteinase ne	14.99	18.17	6.57	10.91	12.66	0.12	0.23	0	0.12	0.01	3.74E-10
Solyc03g122250.2.1	Transmembrane protei	37.22	42.84	34.98	45.57	40.15	3.32	6.28	2.55	4.05	0.1	3.97E-10
Solyc07g005500.2.1	Formin 2B (AHRD V1	4.76	4.66	6.74	5.73	5.47	22.99	22.32	10.87	18.73	3.42	3.97E-10
Solyc00g006820.2.1	Peptide transporter-lik	12.51	13.74	10.35	9.13	11.43	1.3	0.99	1	1.1	0.1	4.14E-10
Solyc02g069680.2.1	Charged multivesicula	11.83	11.32	12.22	13.81	12.29	0	0.48	0.59	0.36	0.03	4.20E-10
Solyc06g048950.2.1	Receptor like kinase, R	3.83	5.05	3.91	3.75	4.13	0.18	0.24	0.15	0.19	0.05	4.20E-10
Solyc01g097390.2.1	Aldo/keto reductase fa	12.81	11.23	9.02	11.63	11.17	0.31	0.78	0.76	0.62	0.06	4.46E-10
Solyc06g060560.2.1	Pentatricopeptide repe	16.59	16.48	9.1	16.92	14.77	0.31	0.63	0	0.31	0.02	4.54E-10

Solyc07g063110.2.1	Beta-1,3-galactosyl-O-	26.49	26.52	39.67	21.62	28.58	202.9	226.03	168.13	199.02	6.96	4.63E-10
Solyc02g067180.2.1	Cystathionine gamma s	192.79	195.46	258.53	199.22	211.5	700.76	672.12	542.74	638.54	3.02	5.02E-10
Solyc04g007980.2.1	1-aminocyclopropane-	65.25	71.51	99.25	83.65	79.91	217.41	324.01	231.24	257.55	3.22	5.25E-10
Solyc09g075450.2.1	Fumarate hydratase cla	27.32	24.55	23.11	31.86	26.71	94.86	86.12	107.81	96.26	3.6	5.51E-10
Solyc11g010960.1.1	Alcohol dehydrogenase	245.19	296.83	101.18	118.2	190.35	1.18	0.45	0.91	0.85	0	5.63E-10
Solyc04g054310.2.1	Alanine-glyoxylate am	20.04	21.43	16.91	13.58	17.99	111.43	93.34	117.15	107.31	5.96	5.77E-10
Solyc12g011420.1.1	Integral membrane sing	12.5	12.1	29.27	14.3	17.04	54.19	54.28	49.52	52.66	3.09	5.85E-10
Solyc03g083360.2.1	Pectinesterase (AHRD	24.97	24.03	20.26	30.84	25.03	4.2	2.39	2.92	3.17	0.13	5.91E-10
Solyc09g074050.2.1	Oxidoreductase (AHRI	14.87	15.59	19.81	14.93	16.3	2.04	1.95	2.14	2.04	0.13	5.94E-10
Solyc01g007980.2.1	RLK, Receptor like pro	44.29	37.4	56.39	42.5	45.14	5.09	5.55	2.77	4.47	0.1	6.15E-10
Solyc06g065440.1.1	Zinc finger family prot	34.86	37.86	44.22	32.08	37.25	3.48	5.8	0.56	3.28	0.09	6.84E-10
Solyc02g090350.2.1	Cytochrome P450	42.7	43.77	85.94	33.29	51.42	0.21	0.65	0.26	0.37	0.01	7.12E-10
Solyc07g053350.2.1	Os06g0207500 protein	26.64	31.94	40.63	10.51	27.43	0.15	0.15	0	0.1	0	7.24E-10
Solyc10g078530.1.1	Patatin-like protein 3 (	82.77	81.34	162.27	134.62	115.25	5.62	3.58	4.51	4.57	0.04	7.24E-10
Solyc07g054670.2.1	NADH dehydrogenase	1.15	2.24	2.16	1.62	1.79	10.83	10.08	12.62	11.18	6.24	7.24E-10
Solyc02g064690.2.1	Acetyltransferase-like	2.39	1.28	1.88	1.58	1.78	26.39	16.49	24.67	22.52	12.63	7.36E-10
Solyc02g081150.1.1	Unknown Protein (AH	18.85	17.61	22.24	22.93	20.41	1.37	2.46	0.94	1.59	0.08	7.40E-10
Solyc11g017470.1.1	NAC domain protein I	104.6	94.71	145	61.68	101.5	695.98	537.58	528.34	587.3	5.79	7.73E-10
Solyc10g044680.1.1	Myb-like transcription	9.59	7.95	4.1	6.78	7.11	0	0.27	0.16	0.14	0.02	7.76E-10
Solyc04g057980.2.1	NAD(P)H-quinone oxi	42.63	29.36	22.76	20.69	28.86	0.25	1.27	1.24	0.92	0.03	7.91E-10
Solyc05g012510.2.1	Phosphorylase (AHRD	66.86	76.36	67.14	83.39	73.44	5.07	17.44	12.72	11.74	0.16	8.02E-10
Solyc04g078600.2.1	Genomic DNA chromo	47.63	44.9	54.11	43.91	47.64	182.41	251.54	172.28	202.08	4.24	8.35E-10
Solyc06g071750.2.1	Octicosapeptide/Phox/	3.4	2.12	4.41	1.92	2.96	56.05	70.66	165.06	97.26	32.83	8.48E-10
Solyc08g083320.2.1	Granule-bound starch s	137.08	107.59	107.13	86.35	109.54	9.46	23.27	13.47	15.4	0.14	8.58E-10
Solyc09g063130.2.1	Photosystem I reaction	549.76	475.12	542.62	448.05	503.89	61.99	122.38	69.2	84.52	0.17	8.99E-10
Solyc02g065170.2.1	Laccase-22 (AHRD V1	33.47	47.3	34.3	76.21	47.82	0.2	0.9	1.09	0.73	0.02	9.00E-10
Solyc03g116480.1.1	F-box family protein (A	1.07	1.59	1.48	1.77	1.48	9.24	11.51	9.05	9.93	6.72	9.29E-10
Solyc03g026150.2.1	Ring-h2 finger protein	43.52	48.85	43.17	46.27	45.45	4.7	5.87	2.2	4.26	0.09	9.51E-10
Solyc10g084180.1.1	C2H2L domain class tr	30.45	29	35.13	23.8	29.6	1.83	5.66	1.72	3.07	0.1	9.55E-10
Solyc01g080280.2.1	Glutamine synthetase (	66.28	52.61	50.87	44.4	53.54	5.79	9.81	12.85	9.48	0.18	9.76E-10
Solyc07g017610.2.1	Saccharopine dehydrog	52.15	54.91	49	36.08	48.03	156.14	148.93	155.1	153.39	3.19	1.03E-09
Solyc05g005940.2.1	Solute carrier family 1	72.23	88.58	83.33	89.13	83.32	11.21	19.46	5.08	11.92	0.14	1.04E-09
Solyc06g066370.2.1	WRKY transcription fa	7.75	6.79	3.92	9.4	6.96	18.79	30.39	37.63	28.94	4.15	1.04E-09
Solyc05g006780.2.1	NPH3 domain containi	16.69	17.43	15.04	30.67	19.96	50.01	73.35	61.31	61.56	3.08	1.06E-09
Solyc03g080080.2.1	Carbon catabolite repr	15.13	20.14	21.15	11.85	17.07	1.54	2.04	1.17	1.58	0.09	1.07E-09
Solyc03g083030.2.1	Protein ABIL1 (AHRD	15.78	20.53	21.49	23.19	20.25	148.78	156.49	144.62	149.96	7.41	1.07E-09
Solyc01g109590.2.1	Receptor-like protein k	66.11	68.46	62.52	75.72	68.2	11.69	13.66	15.16	13.5	0.2	1.07E-09
Solyc02g087210.2.1	Zinc finger AN1 doma	3.84	1.86	2.06	4.44	3.05	16.36	19.91	63.4	33.22	10.89	1.10E-09
Solyc10g080900.1.1	3-oxoacyl-reductase (A	6.41	7.54	6.76	8.75	7.37	2609.59	1356.07	2729.79	2231.82	303.03	1.11E-09
Solyc10g083360.1.1	Calmodulin binding pr	34.89	31.31	49.67	50.95	41.7	5.02	7.6	14.58	9.07	0.22	1.15E-09
Solyc10g086580.1.1	Ribulose-1 5-bisphospl	309.29	315.82	299.64	226.8	287.89	42.18	62.19	45.37	49.91	0.17	1.16E-09
Solyc01g104710.2.1	Unknown Protein (AH	213.75	293.57	363.22	225.2	273.94	25.58	50.89	34.92	37.13	0.14	1.20E-09
Solyc05g005540.2.1	BURP domain-contain	182.91	230.96	111.65	150.51	169.01	10.27	14.27	3	9.18	0.05	1.20E-09
Solyc05g025600.1.1	Chloroplast photosyste	619.66	514.8	359.97	354.93	462.34	56.15	55.2	37.42	49.59	0.11	1.23E-09
Solyc02g093720.2.1	Os02g0200800 protein	6.7	6.08	7.75	4.65	6.29	0.11	0.75	0	0.29	0.05	1.25E-09
Solyc09g098050.2.1	Ankyrin repeat-contair	0	0.29	0.13	0.15	0.14	3.49	3.79	2.04	3.11	21.8	1.27E-09
Solyc09g074630.2.1	Phototropic-responsive	7.6	7.45	5.32	3.65	6	0.31	0.21	0	0.17	0.03	1.31E-09
Solyc02g031960.2.1	Oil palm polygalacturo	4.71	8.39	0.93	2.22	4.06	43.15	30.72	21.05	31.64	7.79	1.33E-09
Solyc03g006330.2.1	LRR receptor-like seri	12.48	13.55	9.35	14.51	12.47	39.8	77.45	64.64	60.63	4.86	1.34E-09
Solyc10g009580.2.1	UDP-glucosyltransfera	11.62	12.43	7.83	6.46	9.58	1.12	0.45	0.27	0.61	0.06	1.37E-09
Solyc01g100600.2.1	Kelch domain-containi	6.73	7.16	2.65	5.79	5.58	0	0.11	0	0.04	0.01	1.39E-09
Solyc12g096310.1.1	DUF599 family protein	2.76	7.37	2.61	1.95	3.67	625.76	516.88	206.31	449.65	122.44	1.40E-09
Solyc04g076830.2.1	Phosphoglycerate muta	22.24	21.44	21.64	13.63	19.74	75.31	66.89	73.58	71.93	3.64	1.44E-09
Solyc06g065570.2.1	N-acetyltransferase (A	100.15	97.27	98.46	98.94	98.7	334.24	342.3	312.03	329.52	3.34	1.47E-09
Solyc02g081170.2.1	Plastid-lipid-associated	93.41	72.18	100.92	81.33	86.96	230.31	331.75	306.18	289.41	3.33	1.61E-09
Solyc02g084400.2.1	Uncharacterized aarF d	23.24	24.73	25.33	19.26	23.14	4.56	3.52	1.1	3.06	0.13	1.61E-09
Solyc12g088230.1.1	Early-responsive to del	35.82	39.68	26.66	23	31.29	111.07	81.68	97.15	96.63	3.09	1.66E-09
Solyc06g006090.1.1	Unknown Protein (AH	10.37	18.45	16.12	12	14.23	0	0	0	0	0	1.73E-09
Solyc10g078680.1.1	Phosphoribosylanthran	2.27	3.66	3.4	5.44	3.69	0	0.14	0.17	0.1	0.03	1.78E-09
Solyc10g084770.1.1	Receptor-like protein k	23.42	17.3	23.69	32.91	24.33	1.42	1.43	1.74	1.53	0.06	1.79E-09
Solyc11g020960.1.1	Proteinase inhibitor II	273.31	282.79	257.85	185.36	249.83	18.85	76.8	25.84	40.5	0.16	1.80E-09
Solyc01g080070.2.1	Copper chaperone (AH	55.91	46.86	53.35	73.38	57.38	1.5	8.08	1.38	3.65	0.06	1.84E-09
Solyc03g117680.2.1	Transcription regulator	5.43	8.45	14.55	6.98	8.85	25.01	30.55	33.41	29.66	3.35	1.85E-09
Solyc04g079960.1.1	Geranylgeranyl pyroph	2.9	1.29	3.64	4.36	3.05	97.67	58.78	113.69	90.05	29.55	1.88E-09
Solyc05g041440.2.1	Genomic DNA chromo	8.77	10.11	9.88	8.74	9.38	0.32	0.16	0	0.16	0.02	1.89E-09
Solyc09g008350.2.1	Amino acid binding pr	52.84	65.55	41.56	40.77	50.18	8.8	9.26	5.72	7.93	0.16	1.89E-09
Solyc03g112880.1.1	Fasciclin-like arabinog	6.17	5.35	9.46	11.34	8.08	0.25	0	0	0.08	0.01	1.90E-09
Solyc08g076090.2.1	At1g32160/F3C3_6 (A	7.69	5.39	8.3	7.41	7.2	24.33	33.48	26.65	28.15	3.91	1.93E-09
Solyc01g005740.2.1	Unknown Protein (AH	2.64	4.7	0	3.73	2.77	108.73	60.21	83.82	84.25	30.44	2.04E-09
Solyc04g078810.2.1	Remorin (AHRD V1 *	14.24	10.34	13.58	23.21	15.34	95.73	97.77	89.23	94.24	6.14	2.07E-09
Solyc05g056170.2.1	Phenylalanine ammoni	21.46	25.72	19.51	18.93	21.41	6.76	2.57	0.92	3.42	0.16	2.11E-09
Solyc03g083910.2.1	Acid beta-fructofurano	68.51	61.79	83.23	90.44	75.99	2148.61	980.97	4589.61	2573.06	33.86	2.12E-09
Solyc04g011440.2.1	heat shock protein (AH	28.33	18.46	22.92	50.52	30.06	193	421.32	570.76	395.03	13.14	2.16E-09
Solyc08g075080.2.1	Ring finger protein 12	6.01	5.57	3.76	9.19	6.13	0	0	0	0	0	2.16E-09
Solyc02g021260.1.1	B3 domain-containing	0.85	0.85	1.36	1.42	1.12	5.82	4.24	7.55	5.87	5.24	2.18E-09
Solyc10g024420.1.1	ABC transporter C fan	0.31	0.52	1.26	0.88	0.74	403.96	198.52	374.86	325.78	438.76	2.21E-09
Solyc03g119600.1.1	NIMIN2c protein (AH	280.38	267.55	267.99	160.12	244.01	31.49	45.21	14.88	30.53	0.13	2.28E-09
Solyc03g065260.2.1	Unknown Protein (AH	1.57	2.8	3.71	0	2.02	192.89	188.15	115.37	165.47	81.92	2.29E-09
Solyc07g042990.1.1	LRR receptor-like seri	3.39	3.59	4.19	6.39	4.39	0	0	0.16	0.05	0.01	2.34E-09
Solyc07g054310.1.1	Gty37 protein (AHRD	1074.76	1064.25	289.27	487.89	729.04	3.36	0.42	1.03	1.6	0	2.43E-09
Solyc04g071150.2.1	Cytochrome P450	28.29	32.3	42.82	18.94	30.59	120.31	118.87	93.84	111.01	3.63	2.47E-09
Solyc03g116710.2.1	GTP binding protein (A	46.37	45.97	30.47	30.81	38.41	5.53	5.58	1.85	4.32	0.11	2.47E-09

Solyc07g061970.2.1	60S ribosomal protein	84.48	73.69	102.06	103.79	91.01	8.16	20.02	7.68	11.95	0.13	2.53E-09
Solyc06g050840.2.1	DNA binding protein (	42.06	42.2	27.73	34.41	36.6	0.67	4.03	0.41	1.7	0.05	2.60E-09
Solyc05g009990.2.1	LRR receptor-like seri	3.37	4.75	3.48	2.98	3.65	0	0	0	0	0	2.61E-09
Solyc03g119860.2.1	Peptidyl-prolyl cis-tran	24.69	22.53	14.69	9.68	17.9	0.17	0.68	0	0.28	0.02	2.61E-09
Solyc04g016470.2.1	Beta-1 3-glucanase (Al	7.63	6.11	4.5	3.78	5.51	162.05	100.83	69.84	110.91	20.15	2.61E-09
Solyc03g097380.2.1	Heavy metal-associated	11.76	15.61	7.99	11.56	11.73	1.61	0.81	0.4	0.94	0.08	2.61E-09
Solyc09g008240.2.1	Uncharacterized ABC	25.44	25.05	39.54	37.61	31.91	4.99	4.05	2.34	3.79	0.12	2.66E-09
Solyc08g005760.1.1	Alcohol acetyltransfera	0	0	0.35	0.21	0.14	32	17.04	36.98	28.67	204.81	2.87E-09
Solyc02g093100.2.1	Receptor like kinase, R	8.41	7.57	11.9	11.05	9.73	0.24	1.45	0.98	0.89	0.09	2.90E-09
Solyc04g007060.2.1	Cc-nbs-lrr, resistance p	26.84	25.82	39	27.68	29.84	3.09	3.97	3.35	3.47	0.12	2.90E-09
Solyc04g064900.1.1	Endo-1 4-beta-glucana	105.32	130.82	148.15	108.87	123.29	2.34	24.52	2.87	9.91	0.08	2.90E-09
Solyc09g074520.2.1	Auxin F-box protein 5	51.24	54.48	68.34	63.33	59.35	7.88	10.44	6.41	8.24	0.14	2.90E-09
Solyc03g005190.2.1	Ferredoxin (AHRD V1	35.01	37.94	25.94	21.27	30.04	2.18	6.03	2.23	3.48	0.12	2.91E-09
Solyc02g080540.1.1	ATP synthase gamma	180.46	161.23	194.99	199.96	184.16	23.55	40.53	18.71	27.6	0.15	2.99E-09
Solyc05g012450.2.1	Dehydration-responsiv	18.28	18.18	13.25	23.14	18.21	57.88	62.91	39.27	53.35	2.93	3.08E-09
Solyc11g071620.1.1	Aldehyde oxidase (AH	8.96	7.2	4.8	5.96	6.73	0.82	0.47	0.77	0.69	0.1	3.08E-09
Solyc01g090590.2.1	Os11g0109966 protein	12.09	12.31	13.23	9.48	11.78	45.3	40.7	53.48	46.49	3.95	3.18E-09
Solyc05g025680.1.1	Respiratory burst oxida	0.11	0.38	0.87	0.3	0.41	4.86	2.84	4.61	4.1	9.89	3.20E-09
Solyc12g094730.1.1	AT-rich interactive do	0.62	0.56	2.39	0.88	1.11	9.07	6.23	2.94	6.08	5.47	3.22E-09
Solyc01g096220.2.1	Ras-related protein Ra	62.19	52.7	45.3	65.02	56.3	150.07	210.62	111.11	157.27	2.79	3.38E-09
Solyc08g078050.1.1	CTF2A (AHRD V1 **	5.94	5.28	3.11	0	3.58	22.92	30.64	41.25	31.6	8.82	3.46E-09
Solyc01g111970.2.1	Laccase 1a (AHRD V1	0.37	0	0.14	0	0.13	103.22	47.95	109.14	86.77	680.55	3.52E-09
Solyc01g074030.2.1	Beta-glucosidase 01 (A	20.38	35.02	16.08	16.03	21.88	0.21	1.04	0.51	0.59	0.03	3.72E-09
Solyc03g026210.2.1	Dihydrodipicolinate re	23.47	30.15	11.49	24.74	22.46	0.36	1.08	0.66	0.7	0.03	3.74E-09
Solyc04g072160.2.1	Prostaglandin E syntha	13.93	7.18	6.05	14.52	10.42	126.44	175.31	113.5	138.42	13.28	3.74E-09
Solyc02g032930.2.1	B-cell receptor-associa	74.25	70.56	73.29	66.53	71.16	206.98	231.66	215.83	218.16	3.07	3.74E-09
Solyc07g065270.1.1	GRAS family transcrip	9.42	8.71	10.4	13.5	10.51	30.46	37.3	31.59	33.12	3.15	3.74E-09
Solyc08g065940.2.1	Zinc finger CCCH don	431.81	471.85	769.83	537.84	552.83	18.41	90.9	52.39	53.9	0.1	3.76E-09
Solyc07g065290.2.1	Zinc finger CCCH don	0	0.23	0.6	0	0.21	10.64	7.64	5.28	7.85	37.85	3.84E-09
Solyc01g087850.2.1	Subtilisin-like protease	21.47	19.8	22.68	37.47	25.35	0.42	2.89	0.09	1.13	0.04	3.96E-09
Solyc10g054420.1.1	Photosystem II oxygen	43.98	37.6	24.92	20.87	31.84	0.7	2.36	1.15	1.4	0.04	3.98E-09
Solyc07g045160.2.1	Phosphofructokinase fa	1.88	2.39	3.17	1.52	2.24	7.82	13.58	9.74	10.38	4.63	4.02E-09
Solyc10g054900.1.1	Proline-rich protein (A	83.68	61.76	35.04	27.47	51.99	0.23	0.23	0.28	0.25	0	4.09E-09
Solyc10g080890.1.1	3-oxoacyl-reductase (A	5.89	8.07	8.29	7.05	7.33	32.97	30.48	28.13	30.53	4.17	4.19E-09
Solyc02g082140.2.1	Unknown Protein (AH	85.98	75.6	89.71	94.41	86.42	10.43	17.67	20.5	16.2	0.19	4.20E-09
Solyc02g069670.2.1	Alpha-glucosidase 1 (A	36.82	32.98	35.87	41.78	36.86	4.3	6.13	3.03	4.49	0.12	4.27E-09
Solyc02g077370.1.1	Ethylene-responsive tra	16.29	13.77	13.93	6.91	12.72	0	0.33	0.4	0.24	0.02	4.29E-09
Solyc11g066440.1.1	Pheophorbide a oxyger	44.76	38.85	37.32	28.1	37.26	99.07	122	128.9	116.66	3.13	4.31E-09
Solyc07g066080.2.1	Ubiquitin-conjugating	26.12	20.88	24.03	19.41	22.61	84.06	96.69	62.41	81.05	3.58	4.39E-09
Solyc01g098490.2.1	Solute carrier family 2	3.29	3.31	2.19	1.21	2.5	13.53	10.96	6.11	10.2	4.08	4.43E-09
Solyc05g009360.2.1	Unknown Protein (AH	137.38	123.23	192.87	143.05	149.13	465.04	438.04	357.63	420.24	2.82	4.43E-09
Solyc06g035580.2.1	Choline dehydrogenase	399.21	455.99	137.62	183.99	294.2	2.26	1.05	2.43	1.91	0.01	4.45E-09
Solyc04g077780.2.1	LIM domain protein (A	340.4	250.59	235.9	360.28	296.79	35.99	75.93	46.24	52.72	0.18	4.61E-09
Solyc03g078650.2.1	Os12g0581300 protein	5.48	5.02	3.7	8.64	5.71	0	0	0	0	0	4.82E-09
Solyc05g012940.2.1	Ribonuclease H2 subu	3.54	2.98	2.56	2.51	2.9	16.23	14.76	20.52	17.17	5.93	4.82E-09
Solyc03g113480.1.1	Unknown Protein (AH	0.69	0.61	0	1.46	0.69	34.14	104.44	79.47	72.68	105.34	4.88E-09
Solyc04g054890.2.1	Acyl-CoA oxidase (AH	8.33	6.39	7.34	7.04	7.27	79.99	70.22	66.34	72.18	9.92	4.95E-09
Solyc07g024000.2.1	Dehydrogenase/ reduct	47.34	51.45	63.55	45.79	52.03	162.42	156.51	138.8	152.58	2.93	4.95E-09
Solyc12g017460.1.1	GDSL esterase/lipase A	25.32	30.15	65.21	82.72	50.85	0	0	0	0	0	4.95E-09
Solyc00g095860.1.1	1-aminocyclopropane-	10.6	10.48	11.12	12.08	11.07	81.85	92.88	76.01	83.58	7.55	4.96E-09
Solyc08g007890.2.1	Mitochondrial aldehyd	1.4	2.5	1.66	0	1.39	40.82	26.31	62.7	43.28	31.13	4.96E-09
Solyc08g061010.2.1	Nitrate transporter (AH	5.19	8.58	3.79	7.34	6.22	0	0	0	0	0	5.00E-09
Solyc07g007130.1.1	NHL repeat-containing	22.82	18.46	22.03	16.43	19.94	69.39	76.41	86.49	77.43	3.88	5.20E-09
Solyc06g066680.2.1	Mitochondrial carrier p	2.09	2.42	3.2	2.07	2.44	12.31	16.16	4.97	11.15	4.56	5.42E-09
Solyc04g081420.2.1	Unknown Protein (AH	7.33	10.55	6.74	7.17	7.95	19.86	35.84	57.03	37.58	4.73	5.46E-09
Solyc07g055810.2.1	Receptor like kinase, R	3.85	3.94	4.39	7.99	5.04	0.05	0.05	0	0.03	0.01	5.47E-09
Solyc11g008630.1.1	Hydroxycinnamoyl Co	96.53	109.17	48.35	55.14	77.3	4.27	2.33	1.7	2.77	0.04	5.47E-09
Solyc03g007000.2.1	Peptidase M48 Ste24p	11.02	11.13	11.26	14.89	12.07	42.13	39.68	57.97	46.59	3.86	5.58E-09
Solyc03g120790.1.1	Unknown Protein (AH	9.75	10.72	5.75	12.98	9.8	0.23	0.23	0	0.15	0.02	5.62E-09
Solyc02g062340.2.1	Fructose-bisphosphate	139.27	129.88	136.6	94.73	125.12	21.4	23.89	12.73	19.34	0.15	5.62E-09
Solyc12g006440.1.1	Unknown Protein (AH	89.2	55.41	62.48	39.43	61.63	2.25	4.54	0.92	2.57	0.04	5.63E-09
Solyc12g014570.1.1	Glycerophosphoryl die	3.16	5.31	5.17	7.45	5.27	0.21	0.21	0.17	0.2	0.04	5.65E-09
Solyc06g083580.2.1	Pectate lyase 1-27 (AH	166.52	159.81	153.34	217.74	174.35	17.89	39.42	13.25	23.52	0.13	5.75E-09
Solyc04g076300.2.1	Transporter small cond	16.4	13.43	21.89	18.42	17.54	1.67	3.67	0.93	2.09	0.12	5.80E-09
Solyc03g098290.2.1	Sucrose synthase (AHI	2	3.03	1.48	1.57	2.02	44.99	40.44	40.96	42.13	20.86	5.96E-09
Solyc06g061180.1.1	Abscisic acid receptor	28.36	28.53	33.46	37.94	32.07	1.49	4.77	3.37	3.21	0.1	6.20E-09
Solyc01g090790.2.1	BHLH transcription fac	13.34	13.64	24.6	12.44	16	1.15	0.35	0.14	0.55	0.03	6.24E-09
Solyc04g063240.2.1	Senescence-inducible	39.37	47.36	40.36	23.04	37.53	5.26	5.75	5.66	5.56	0.15	6.25E-09
Solyc02g087780.2.1	Aldose-1-epimerase-lil	36.37	38.87	36.22	29.73	35.3	5.46	6.55	0.73	4.25	0.12	6.41E-09
Solyc03g117590.2.1	Chaperone protein dna	41.94	52.19	47.08	24.77	41.49	6.91	4.31	2.43	4.55	0.11	6.64E-09
Solyc04g016200.1.1	UDP-glucosyltransfera	7.44	4.16	5.34	3.92	5.21	0.24	0.24	0	0.16	0.03	7.13E-09
Solyc09g008320.2.1	Xyloglucan endotransg	15	30.44	27.07	21.85	23.59	3.39	3.06	1.75	2.73	0.12	7.39E-09
Solyc01g065500.2.1	Phosphoribosylanthran	7.64	6.62	8.23	12.12	8.65	0.86	0.7	0.86	0.81	0.09	7.40E-09
Solyc01g107700.2.1	Kynurenine formamida	56.23	50.48	57.73	71.97	59.1	180.74	167.22	204.18	184.05	3.11	7.47E-09
Solyc02g091860.2.1	Receptor like kinase, R	7.93	8.54	10.85	10.93	9.56	1.18	1.09	0.79	1.02	0.11	7.95E-09
Solyc06g076520.1.1	class I heat shock prot	164.29	126.14	187.8	155.93	158.54	1513.68	2299.09	1923.91	1912.23	12.06	8.04E-09
Solyc11g018710.1.1	Unknown Protein (AH	14.99	12.45	11.01	5.18	10.91	0	0	0.66	0.22	0.02	8.04E-09
Solyc02g062470.1.1	2-oxoglutarate-depend	1.71	3.05	2.02	0	1.69	16.96	11.87	24.66	17.83	10.52	8.21E-09
Solyc09g084440.2.1	Proteinase inhibitor I (	581.37	1335.74	387.77	594.89	724.94	0.95	3.84	5.85	3.55	0	8.34E-09
Solyc06g059720.2.1	Stearoyl-acyl carrier pr	0	0	0	0	0	9.34	4.02	9.45	7.6	760.33	8.59E-09
Solyc10g081010.1.1	Hydroxyproline-rich gl	14.73	10.58	10.66	24.51	15.12	0	0.4	0.12	0.17	0.01	8.71E-09
Solyc07g007870.2.1	NADH flavin oxidored	229.32	199.66	264.67	144.09	209.44	14.23	39.81	11.38	21.81	0.1	8.76E-09

Solyc10g081650.1.1	Carotenoid isomerase,	21.31	24.5	24.89	18.03	22.18	74.24	71.39	94.29	79.97	3.61	8.81E-09
Solyc01g066640.2.1	Os04g0405500 protein	19.41	24.46	21.48	35.72	25.27	2.22	2.52	0.68	1.81	0.07	8.90E-09
Solyc04g082290.2.1	Os03g0133300 protein	25.97	21.09	19.06	27.12	23.31	3.66	3.69	4.2	3.85	0.17	9.00E-09
Solyc12g057110.2.1	14-3-3 (Fragment) (AH	135.56	139.07	125.14	159.19	139.74	25.71	30.57	17.21	24.5	0.18	9.34E-09
Solyc09g098290.2.1	Receptor like kinase, R	25.2	30.26	34.96	49.1	34.88	1.63	3.4	0.84	1.96	0.06	9.37E-09
Solyc01g068460.2.1	Pathogen-induced caln	77.37	78.19	108.55	85.2	87.33	13.31	17.35	12.06	14.24	0.16	9.43E-09
Solyc01g111040.2.1	EF-Hand containing pr	50.4	59.54	48.97	22.8	45.43	538.97	383.7	445.87	456.18	10.04	9.54E-09
Solyc01g005370.2.1	Calmodulin-2 (AHRD	17.54	19.68	15.3	17.8	17.58	61.92	70.23	60.94	64.36	3.66	9.67E-09
Solyc06g068380.2.1	PPPDE peptidase dom	114.02	101.25	118.95	117.61	112.96	15.69	28.4	7.41	17.17	0.15	1.00E-08
Solyc05g055800.2.1	Aspartyl protease fami	9.8	8.84	6.68	7.22	8.13	0.22	1.24	1.23	0.9	0.11	1.01E-08
Solyc06g054070.2.1	Non-specific lipid-tran	28.86	18.35	20	31.11	24.58	1.85	1.87	0.91	1.54	0.06	1.04E-08
Solyc04g045600.2.1	Serine/threonine kinase	17.15	25.83	14.23	19.2	19.1	2.28	2.92	1.87	2.36	0.12	1.05E-08
Solyc01g096360.2.1	Rho GTPase activating	8.11	6.98	6.12	9.97	7.79	0.54	0.87	0.4	0.6	0.08	1.09E-08
Solyc07g022900.2.1	Chlorophyll a-b bindin	71.99	86.17	42.02	48.61	62.2	1.82	8.38	3.73	4.64	0.07	1.12E-08
Solyc01g057000.2.1	Universal stress protei	471.33	518.58	475.09	464.42	482.36	2963.15	3084.38	2563.65	2870.39	5.95	1.13E-08
Solyc09g008200.2.1	Heavy metal-associate	117.91	163.34	214.66	102.25	149.54	385.85	455.94	348.31	396.7	2.65	1.17E-08
Solyc02g085600.1.1	GRAS family transcrip	4.05	7.54	10.29	11.99	8.47	80.39	56.76	101.82	79.66	9.41	1.19E-08
Solyc06g073260.2.1	NAD-dependent epime	18.06	23.33	22.33	16.7	20.11	2.52	2.4	2.58	2.5	0.12	1.20E-08
Solyc03g116340.2.1	Transcription factor (F	65.43	51.19	66.25	43.57	56.61	181.7	163.94	178.32	174.65	3.09	1.20E-08
Solyc12g088430.1.1	Unknown Protein (AH	43.42	38.14	43.91	21.53	36.75	0.46	6.89	0	2.45	0.07	1.21E-08
Solyc02g066920.2.1	Genomic DNA chromo	43.41	51.52	35.65	38.53	42.28	5.5	7.28	4.22	5.67	0.13	1.24E-08
Solyc04g079120.2.1	Protein phosphatase 2C	9.38	8.62	5.44	13.7	9.29	21.99	34.84	42.74	33.19	3.57	1.25E-08
Solyc03g116500.2.1	Polygalacturonase (AH	6.55	7.65	5.5	7.62	6.83	0.24	0.47	0.29	0.33	0.05	1.25E-08
Solyc01g090730.2.1	Squamosa promoter bi	32.99	23.88	29.32	65.63	37.95	0	0.18	0.22	0.13	0	1.27E-08
Solyc04g011450.1.1	Heat shock cognate pro	24.1	14.68	7.48	44.86	22.78	145.55	362.86	338.74	282.38	12.4	1.27E-08
Solyc08g005770.2.1	Alcohol acetyltransfera	0.15	0.27	3.69	2.32	1.61	1177.14	628.34	834.57	880.02	547.44	1.27E-08
Solyc01g091370.2.1	AT-hook motif nuclear	11.79	13.07	11.47	19.01	13.84	1.34	1.18	0.41	0.98	0.07	1.29E-08
Solyc02g068340.2.1	Kinesin-like calmoduli	11.46	11.46	8.36	12.01	10.82	1.77	1.66	0.88	1.44	0.13	1.29E-08
Solyc05g007490.2.1	Dehydration-responsiv	40.14	28.45	21.84	49.63	35.02	1.22	0.96	2.35	1.51	0.04	1.31E-08
Solyc09g092340.2.1	3&apos-5&apos exonu	10.96	11.12	10.34	19.84	13.07	0.18	0.71	0.22	0.37	0.03	1.32E-08
Solyc01g104660.2.1	Exocyst subunit Exo70	18.85	24.71	28	22.14	23.43	80.22	66.65	79.68	75.52	3.22	1.33E-08
Solyc10g086190.1.1	Adenosine kinase (AH	70.82	67.35	50.36	71.36	64.97	7.68	14.86	5.39	9.31	0.14	1.33E-08
Solyc07g043000.2.1	Unknown Protein (AH	476.46	343.59	386.28	946.98	538.33	3.95	0	4.85	2.93	0.01	1.35E-08
Solyc11g019910.1.1	Pectinesterase (AHRD	12.06	8.64	8.3	7.11	9.03	0	0	0	0	0	1.36E-08
Solyc12g014430.1.1	Cellulose synthase (AH	10.32	10.71	13.99	10.5	11.38	1.9	1.55	1.35	1.6	0.14	1.36E-08
Solyc02g063510.1.1	Unknown Protein (AH	15.75	19.69	14.81	14.66	16.23	0.97	3.25	0.2	1.47	0.09	1.40E-08
Solyc06g053290.1.1	Auxin responsive SAU	2.84	3.54	4.7	1.61	3.17	33.08	93.15	41.21	55.81	17.59	1.40E-08
Solyc04g080940.2.1	Nodulin-like protein (A	9.57	11.26	7.06	5.32	8.3	0.69	0.42	0.17	0.43	0.05	1.48E-08
Solyc04g015200.2.1	6-phosphofructokinase	10.25	8.99	8	6.13	8.34	23.68	35.05	25.79	28.17	3.38	1.51E-08
Solyc04g040190.1.1	Lycopene beta-cyclase	13.69	17.23	11.96	8.94	12.96	1.17	1.82	0.52	1.17	0.09	1.52E-08
Solyc03g115120.1.1	Chaperone protein dna	11.5	8.66	5.22	10.26	8.91	0.57	0.43	0.35	0.45	0.05	1.52E-08
Solyc02g093150.2.1	AP2-like ethylene-resp	61.69	52.67	58.16	37.79	52.58	7.27	10.22	4.83	7.44	0.14	1.54E-08
Solyc03g113100.2.1	Ubiquitin-conjugating	295.36	259.8	261.47	281.17	274.45	744.44	803.9	866.25	804.86	2.93	1.58E-08
Solyc06g007910.2.1	Gibberellin regulated p	25.69	40.79	24.58	31.43	30.62	0.56	3.4	1.38	1.78	0.06	1.62E-08
Solyc05g046120.2.1	Formin 3 (AHRD V1 *	0.5	0.64	2.2	1.12	1.12	4.35	4.91	3.56	4.27	3.83	1.65E-08
Solyc12g096770.1.1	Hydroxycinnamoyl Co	17.16	14.74	17.61	5.07	13.64	0.12	0.24	0.44	0.27	0.02	1.69E-08
Solyc03g096730.2.1	GDP-D-mannose pyrop	395.1	375.64	248.55	343.88	340.79	62.72	80.34	50.65	64.57	0.19	1.69E-08
Solyc07g006280.2.1	Senescence-associated	213.97	202.53	90.08	251.99	189.64	1225.87	1027.03	1311.12	1188.01	6.26	1.75E-08
Solyc03g111680.2.1	DNA polymerase III ga	2.33	3.51	1.37	3.11	2.58	0.05	0.11	0	0.05	0.02	1.78E-08
Solyc08g082710.2.1	Protein ABIL1 (AHRD	20.94	21.27	20.04	8.94	17.8	2.07	0.97	0.78	1.27	0.07	1.78E-08
Solyc07g042930.2.1	Rhomboid domain-con	10.33	7.78	5.16	5.9	7.29	24.56	32.87	25.64	27.69	3.8	1.83E-08
Solyc07g014670.2.1	Cytochrome P450	44.75	47.15	68.12	41.84	50.47	6.44	9.75	3.96	6.72	0.13	1.93E-08
Solyc01g080130.2.1	Unknown Protein (AH	13.28	18.78	15.67	17.57	16.33	1.73	2.44	1.17	1.78	0.11	1.97E-08
Solyc03g044380.2.1	Histone-lysine N-meth	7.66	8.09	8.02	10.32	8.52	0.58	1.28	1.07	0.98	0.11	1.98E-08
Solyc04g014600.2.1	Universal stress protei	70.02	84.65	40.59	42.36	59.41	446.66	439.62	349.1	411.79	6.93	1.98E-08
Solyc08g078950.2.1	Nitrate transporter (AH	4.58	3.68	6.06	5.84	5.04	0.27	0.36	0.44	0.36	0.07	1.98E-08
Solyc09g009590.1.1	Zinc finger A20 and A	59.46	50.6	54.28	9.82	43.54	394.13	302.74	270.54	322.47	7.41	2.09E-08
Solyc01g099810.2.1	Arginine/serine-rich sp	95.35	97.71	89.58	38.38	80.25	246.58	231.06	216.46	231.37	2.88	2.13E-08
Solyc00g060810.2.1	Major latex-like protei	0	0.4	1.06	0	0.36	170.77	78.26	60.14	103.06	282.35	2.18E-08
Solyc12g011280.1.1	Chlorophyll a-b bindin	6.74	5.14	3.69	9.2	6.19	0	0	0	0	0	2.18E-08
Solyc12g099830.1.1	Receptor-like protein k	17.47	26.04	24.13	21.84	22.37	3.83	3.53	2.55	3.3	0.15	2.18E-08
Solyc00g049520.2.1	Unknown Protein (AH	484.07	468.19	292.12	319.94	391.08	74.06	57.25	47.62	59.64	0.15	2.31E-08
Solyc06g049030.2.1	Ring finger protein (Al	36.89	38.68	30.25	26.98	33.2	3.28	5.35	1.86	3.5	0.11	2.31E-08
Solyc02g078760.1.1	CK25 (AHRD V1 ***-	28.05	15.72	10.72	27.55	20.51	0	0.42	0	0.14	0.01	2.35E-08
Solyc09g014480.1.1	Polygalacturonase inhi	16.3	13.98	22.7	6.67	14.91	0	0.32	0	0.11	0.01	2.37E-08
Solyc03g006870.2.1	Phosphoglucomutase (	18.34	20.68	14.75	15.48	17.31	2.61	2.8	2.28	2.56	0.15	2.37E-08
Solyc01g112100.2.1	Dimethylaniline mono	116.97	135.35	122.16	100.8	118.82	702.27	717.57	643.8	687.88	5.79	2.40E-08
Solyc01g109790.2.1	Glucose-1-phosphate a	92.74	71.69	41.51	32.35	59.57	0.41	0.82	1.25	0.83	0.01	2.45E-08
Solyc09g011810.2.1	Fructose-1 6-bisphospl	24	30.66	20.42	18.25	23.33	3.56	3.73	2.43	3.24	0.14	2.51E-08
Solyc02g071770.2.1	DUF1264 domain prot	8.81	4.52	10.71	9.44	8.37	34.09	27.42	15.91	25.81	3.08	2.55E-08
Solyc00g011890.2.1	Galactokinase like prot	26.34	29.33	32.2	25.65	28.38	90.66	77.29	103.24	90.4	3.19	2.61E-08
Solyc01g107830.2.1	UDP-glucosyltransfera	9.18	8.55	8.33	4.2	7.57	179.64	117.76	260.39	185.93	24.58	2.61E-08
Solyc06g036310.2.1	Heavy metal transport/	835.8	682.12	414.91	526.96	614.95	29.78	59.69	18.3	35.92	0.06	2.65E-08
Solyc12g094420.1.1	Predicted mechanosens	5.77	7.39	7.42	4.33	6.23	17.3	22.36	23.16	20.94	3.36	2.69E-08
Solyc05g050800.2.1	Phosphoglycerate muta	263.88	241.28	183.94	276.3	241.35	32.69	40.32	56.79	43.27	0.18	2.72E-08
Solyc03g120710.2.1	Harpin-induced protein	424.46	458.76	479.25	388.27	437.69	70.44	110.45	64.64	81.84	0.19	2.72E-08
Solyc11g020060.1.1	Glyoxylate reductase/h	16.61	16.03	18.49	11.91	15.76	48.01	48.8	58.28	51.7	3.28	2.78E-08
Solyc09g075950.1.1	Heat shock protein 1 (A	6.63	8.34	5.67	5.66	6.58	36.21	91.58	93.06	73.62	11.2	2.80E-08
Solyc08g081550.2.1	1-aminocyclopropane-	9.9	10.05	8.72	8.48	9.29	75.5	78.87	64.51	72.96	7.86	2.88E-08
Solyc06g036340.2.1	Unknown Protein (AH	9.59	8.42	9.15	7.06	8.55	31.96	27.41	27.65	29.01	3.39	2.90E-08
Solyc03g118090.2.1	Calcium-binding mitoc	6.95	6.3	6.19	9.09	7.13	61.46	43.28	56.24	53.66	7.52	2.90E-08
Solyc01g058270.2.1	CAS1 domain containi	14.11	18.14	8.96	10.92	13.03	0.97	1.87	0.84	1.23	0.09	2.91E-08

Solyc02g077330.2.1	GDSL esterase/lipase A	102.34	121.33	42.9	35.41	75.5	0.44	0	0.18	0.21	0	2.93E-08
Solyc01g091520.2.1	Enoyl-CoA hydratase/i	98.82	91.96	109.09	116.55	104.11	14.98	25.04	15.25	18.42	0.18	2.98E-08
Solyc05g055010.2.1	RNA-binding protein (	15.84	15.64	8.84	8.15	12.12	39.8	84.98	66.05	63.61	5.25	3.06E-08
Solyc03g121720.2.1	2-hydroxy-3-oxopropio	30.49	40.32	39.83	45.07	38.93	5.29	7.16	4.45	5.63	0.14	3.18E-08
Solyc01g111230.2.1	Disease resistance resp	117.96	119.34	38.07	61.37	84.19	0.58	0.29	0.71	0.53	0.01	3.20E-08
Solyc07g008210.2.1	TPR domain protein (A	312.15	253.92	127.36	85.24	194.67	0	0.23	1.98	0.74	0	3.21E-08
Solyc11g008350.1.1	Kinesin-like protein (A	3.59	3.79	4.16	4.04	3.89	15.92	13.08	12.61	13.87	3.56	3.23E-08
Solyc06g076820.1.1	Transcription factor (F	40.52	34.52	48.82	56.09	44.99	6.57	8.18	6.7	7.15	0.16	3.24E-08
Solyc05g054210.2.1	Proline-rich protein (A	124.28	121.02	123.29	177.5	136.52	27.55	30.08	29.04	28.89	0.21	3.27E-08
Solyc12g005860.1.1	3-isopropylmalate deh	104.87	112.53	152.34	137.41	126.79	324.57	329.33	427.1	360.33	2.84	3.29E-08
Solyc08g006890.2.1	Tubulin alpha-3 chain	3692.63	3343.97	2491.48	3300.94	3207.26	620.6	816	356.96	597.85	0.19	3.31E-08
Solyc09g018010.2.1	Non-specific lipid-tran	12.48	17.98	14.02	8.41	13.22	0	0	0	0	0	3.33E-08
Solyc01g094260.2.1	Short-chain dehydroge	11.75	17.25	14.84	19.83	15.92	1.67	1.51	0.41	1.2	0.08	3.45E-08
Solyc11g069330.1.1	Arabidopsis thaliana g	212	165.34	227.57	131.39	184.08	32.45	34.89	21.32	29.55	0.16	3.48E-08
Solyc04g005630.2.1	Mitochondrial transcri	7.19	8.36	7.48	5.69	7.18	27.68	24.74	33.04	28.49	3.97	3.61E-08
Solyc06g076220.2.1	Expansin-1 (AHRD V1	12.13	10.8	16.4	19.31	14.66	0.82	1.24	0.25	0.77	0.05	3.66E-08
Solyc05g051010.2.1	Dihydroflavonol 4-red	17.19	18.86	14.15	29.41	19.9	0.65	0.33	1.19	0.72	0.04	3.66E-08
Solyc07g045140.2.1	Uncharacterized memb	1.45	1.19	1.98	0.32	1.23	13.46	9.38	14.43	12.42	10.06	3.79E-08
Solyc01g010200.2.1	LTRGag-pol-polymera	656.86	810.4	542.42	419.92	607.4	111.53	105.52	66.69	94.58	0.16	3.80E-08
Solyc08g082250.2.1	Endoglucanase 1 (AHI	177.47	232.79	251.03	207.35	217.16	47.51	58.82	47.07	51.13	0.24	3.82E-08
Solyc09g082860.2.1	Sulfate adenylyltransfe	7.38	11.24	15.73	10.44	11.2	46.99	39.65	73.09	53.24	4.75	3.83E-08
Solyc03g117720.2.1	AP2-like ethylene-resp	12.59	19.73	15.27	14.8	15.6	3.73	1.16	1.94	2.28	0.15	4.00E-08
Solyc06g051960.2.1	Pectinesterase (AHRD	4.54	2.76	6.92	8.14	5.59	0	0.09	0	0.03	0.01	4.08E-08
Solyc06g069280.2.1	Protein LSM14 homol	13.18	13.24	12.64	13.01	13.02	37.98	42.17	31.71	37.29	2.86	4.08E-08
Solyc12g100140.1.1	BHLH transcription fa	10.82	16.14	9.84	14.9	12.92	0.83	2.15	0.87	1.28	0.1	4.08E-08
Solyc06g068900.2.1	Nudix hydrolase 4 (AH	1.25	3.13	2.66	3.9	2.73	212.99	128.16	196.94	179.36	65.58	4.13E-08
Solyc06g083370.2.1	Os01g0917200 protein	8.86	7.45	6.1	9.05	7.86	0.2	0.8	1.1	0.7	0.09	4.14E-08
Solyc05g053930.2.1	ATP binding / serine-t	25.1	22.63	26.77	35.05	27.39	2.34	3.28	1.92	2.51	0.09	4.33E-08
Solyc06g073190.2.1	Fructokinase-like (AH	428.71	410.64	615.32	699.16	538.46	89.26	85.46	78.62	84.45	0.16	4.33E-08
Solyc01g093990.2.1	Glyoxylate reductase/h	24.03	25.19	23.3	23.04	23.89	1.65	5.97	2.22	3.28	0.14	4.33E-08
Solyc02g080070.2.1	RLK, Receptor like pr	2.34	1.91	2.64	2.76	2.41	33.46	28.74	47.01	36.4	15.09	4.43E-08
Solyc07g005180.2.1	Unknown Protein (AH	126.12	122.61	93.4	131.19	118.33	16.93	26.88	10.01	17.94	0.15	4.53E-08
Solyc01g091530.2.1	Fasciclin-like arabinog	127.97	122.95	180.23	253.41	171.14	5.39	8.42	4.58	6.13	0.04	4.54E-08
Solyc10g085830.1.1	O-methyltransferase 1	12.57	9.57	6.02	6.19	8.59	287.11	255.57	193.73	245.47	28.58	4.60E-08
Solyc08g080090.2.1	Senescence-inducible	14.02	18.28	34.21	5.81	18.08	1596.61	1173.21	1090.65	1286.82	71.17	4.64E-08
Solyc04g015280.2.1	Zinc finger RING-type	10.64	7.71	10.37	13.13	10.46	0.8	2.02	0.61	1.14	0.11	4.71E-08
Solyc06g074200.2.1	Sex-linked protein 9 (I	212.37	257.38	162.59	124	189.09	31.4	17.31	25.56	24.76	0.13	4.79E-08
Solyc08g075430.2.1	ABC transporter G fan	2.14	2.72	1.92	1.01	1.95	6.92	10.3	4.86	7.36	3.78	4.95E-08
Solyc10g085460.1.1	Cc-nbs-lrr, resistance	12.45	18.11	19	14.27	15.96	2.47	2.36	1.44	2.09	0.13	5.05E-08
Solyc01g096940.2.1	Receptor like kinase, R	2.12	4.37	3.1	3.35	3.23	0.34	0	0.08	0.14	0.04	5.15E-08
Solyc06g048820.1.1	Wound-induced protei	87.76	84.57	124.96	108.73	101.5	8.31	15.29	9.01	10.87	0.11	5.45E-08
Solyc01g091690.2.1	Dehydration-responsiv	36.25	44.7	59.75	93.61	58.58	175.63	386.6	394.02	318.75	5.44	5.53E-08
Solyc06g083410.2.1	Os04g0376600 protein	9.33	9.06	6.76	5.1	7.56	25.19	30.94	26.74	27.62	3.65	5.65E-08
Solyc09g015840.2.1	Receptor-like kinase (A	33.73	58.05	19.24	50.85	40.47	346.74	269.69	362.36	326.26	8.06	5.71E-08
Solyc01g079600.2.1	Lipase (AHRD V1 *-*-	13.7	15.08	15.84	19.5	16.03	35.9	57.58	33.81	42.43	2.65	5.84E-08
Solyc12g096710.1.1	Receptor like kinase, R	0.24	0.22	0.58	0.35	0.35	2.18	3.19	3.24	2.87	8.26	6.15E-08
Solyc12g096820.1.1	UDP-glucosyltransfera	11.78	12.75	9.54	12.84	11.73	0.8	1.62	0.56	0.99	0.08	6.28E-08
Solyc02g062390.2.1	Unknown Protein (AH	0	0.88	0.58	1.39	0.71	272.06	139.61	191.07	200.91	281.98	6.32E-08
Solyc03g098480.1.1	Unknown Protein (AH	0.2	0	0.48	0	0.17	18.97	61.18	8.64	29.6	174.1	6.32E-08
Solyc08g075940.2.1	DNA-directed RNA po	13.75	13.83	15.45	11.38	13.6	1.23	2.88	2.05	2.05	0.15	6.35E-08
Solyc01g049650.2.1	Pyruvate kinase (AHR	105.97	129.8	132.45	140.99	127.3	18.33	33.02	17.05	22.8	0.18	6.40E-08
Solyc03g005780.1.1	Chlorophyll a-b bindin	48	43.38	31.37	33.77	39.13	8.75	5.82	1.95	5.51	0.14	6.64E-08
Solyc08g080650.1.1	Osmotin-like protein (	3.21	1.9	4.1	11.71	5.23	1049.14	1690.21	157.48	965.61	184.63	6.64E-08
Solyc02g077050.2.1	Cathepsin B-like cyste	7.52	4.12	7.05	10.64	7.33	0	0	0	0	0	6.69E-08
Solyc08g061480.2.1	Chalcone isomerase (A	12.94	15.21	13.95	14.69	14.2	48.65	36.19	40.43	41.76	2.94	6.74E-08
Solyc07g066150.1.1	Photosystem I reaction	277.8	289.97	288.56	275.5	282.96	54.02	71.68	84.64	70.11	0.25	6.99E-08
Solyc01g111210.2.1	Glutamyl-tRNA(Gln) a	10.53	10.54	5.43	6.17	8.17	0.27	0.67	0.65	0.53	0.06	7.09E-08
Solyc11g009010.1.1	Hydrolase alpha/beta f	31.44	45	51.65	26.16	38.56	120.54	135.65	102.2	119.46	3.1	7.20E-08
Solyc08g083310.2.1	Glucan endo-1 3-beta-	9.49	6.23	10.83	12.32	9.72	0.63	1.01	0.46	0.7	0.07	7.21E-08
Solyc03g117500.1.1	VQ motif-containing p	4.68	1.39	4.14	3.31	3.38	17.66	18.77	26.35	20.93	6.19	7.26E-08
Solyc02g087870.2.1	Uncharacterized ABC	23.06	28.32	43.77	55.02	37.54	0.72	1.46	0.58	0.92	0.02	7.26E-08
Solyc10g055770.1.1	Multidrug resistance p	6.97	5.67	4.2	10.07	6.73	0	0.36	0	0.12	0.02	7.43E-08
Solyc06g075180.1.1	Ribosomal protein L12	496.96	411	652.02	727.53	571.88	51.38	77.9	64.7	64.66	0.11	7.62E-08
Solyc05g053100.2.1	Dihydrolipoyl dehydro	18.34	15.4	15.65	19.58	17.24	1.12	4.7	2.98	2.93	0.17	7.67E-08
Solyc12g087890.1.1	Transcription factor/ tr	36.65	32.81	39.53	54.51	40.88	5.92	5.46	4.37	5.25	0.13	7.77E-08
Solyc02g084950.2.1	Carboxyl methyltransf	1.24	1.27	1.89	0.5	1.23	8.91	8.11	2.82	6.61	5.4	7.89E-08
Solyc07g044990.2.1	Transmembrane protei	3.94	9.64	5.81	2.79	5.54	26.85	23.27	32.74	27.62	4.98	7.94E-08
Solyc02g087770.2.1	Aldose-1-epimerase-lil	168.47	150.93	118.95	183.2	155.39	30.56	41.61	21.64	31.27	0.2	7.97E-08
Solyc01g099650.2.1	Formin 3 (AHRD V1 *	1.24	2.34	1.55	3.2	2.08	0	0	0	0	0	8.04E-08
Solyc02g077670.2.1	Chaperone protein dna	14	15.87	9.05	14.92	13.46	45.71	103.14	167.7	105.52	7.84	8.09E-08
Solyc08g081010.2.1	Glutamate-cysteine lig	48.72	46.95	58.52	54.85	52.26	460.87	319.37	587.59	455.94	8.72	8.35E-08
Solyc01g079790.2.1	Glucose-1-phosphate a	12.02	9.33	9.65	9.22	10.05	0.93	1.56	0.63	1.04	0.1	8.38E-08
Solyc11g072480.1.1	Senescence-associated	23.53	27.3	26.67	25.45	25.74	2.05	4.7	2.06	2.94	0.11	8.55E-08
Solyc07g062220.2.1	Os06g0207500 protein	4.88	7.4	9.17	4.09	6.39	17.96	27.25	20.81	22.01	3.45	8.57E-08
Solyc08g016310.2.1	LRR receptor-like seri	11.2	13.29	7.93	8.45	10.22	0.88	1.6	0.2	0.89	0.09	8.60E-08
Solyc02g067930.2.1	Glycerol-3-phosphate	4.64	4.92	5.26	8.32	5.79	0.14	0.44	0	0.19	0.03	8.65E-08
Solyc01g105030.2.1	Chlorophyll a-b bindin	242.58	186.65	208.36	175.74	203.33	44.38	47.69	30.07	40.71	0.2	8.69E-08
Solyc01g095530.2.1	Unknown Protein (AH	383.26	339.7	194.58	255.51	293.26	726.51	872.28	401.47	666.75	2.27	8.87E-08
Solyc01g107010.2.1	DNA polymerase IV (A	3.93	5.45	5.08	4.88	4.83	17.56	14.75	17.3	16.54	3.42	9.04E-08
Solyc05g007220.2.1	Kelch repeat-containin	6.96	8.37	11.46	13.45	10.06	29.42	23.42	39.39	30.74	3.06	9.19E-08
Solyc02g080160.2.1	Xyloglucan endotransg	26.04	33.78	15.62	47.44	30.72	0.53	0.54	1.1	0.72	0.02	9.63E-08
Solyc03g115230.2.1	ClpB chaperone (AHR	12.51	10.11	7.42	13.1	10.79	98.01	292.42	387.51	259.31	24.04	9.81E-08

Solyc01g105050.2.1	Chlorophyll a-b bindin	149.65	130.68	105.09	81.33	116.69	24.06	20.08	16.82	20.32	0.17	9.82E-08
Solyc12g055930.1.1	UDP-glucose 4-epimer	6.62	6.06	4.68	5.61	5.74	25.66	27.88	30.02	27.85	4.85	9.88E-08
Solyc11g065920.1.1	Xanthine dehydrogena	22.2	23.62	37.35	28.74	27.98	70.73	66.81	87.95	75.16	2.69	1.00E-07
Solyc05g008640.1.1	RING finger protein (A	0.38	0.34	0	0.54	0.32	6.2	9.38	7.23	7.6	24.14	1.01E-07
Solyc09g097780.2.1	Glycine-rich protein (A	95.33	75.68	45.89	49.09	66.5	4.62	2.8	1.14	2.85	0.04	1.01E-07
Solyc01g103430.2.1	Glycogen synthase kin	218.5	213.06	179.4	195.01	201.49	31.88	49.31	27.4	36.2	0.18	1.01E-07
Solyc05g014080.1.1	Unknown Protein (AH	68.56	63.32	102.25	87.83	80.49	9.41	4.22	16.69	10.11	0.13	1.06E-07
Solyc02g084870.2.1	Mitogen-activated prot	34.85	35.77	51.82	20.63	35.77	116.37	108.82	117.37	114.19	3.19	1.09E-07
Solyc09g008840.2.1	Pyruvate kinase (AHR	12.37	12.23	11.65	14.32	12.64	38.76	38.69	28.4	35.28	2.79	1.09E-07
Solyc03g078360.1.1	Receptor-like protein k	1.16	2.52	2.06	1.65	1.85	15.19	10.1	24.28	16.52	8.94	1.09E-07
Solyc00g247300.2.1	Cytochrome P450	3.21	4.28	2.05	3.21	3.19	0	0.11	0	0.04	0.01	1.09E-07
Solyc10g079570.1.1	Acyltransferase (AHR	0	0	0	0	0	58.07	52.96	14.27	41.77	4176.67	1.10E-07
Solyc09g098070.2.1	Necrotic spotted lesion	16.89	15.04	15.77	17.24	16.23	39.91	48.44	37.46	41.94	2.58	1.10E-07
Solyc04g007410.2.1	Os06g0731300 protein	36.7	46.27	40.2	38.32	40.37	5.65	6.41	5.2	5.75	0.14	1.11E-07
Solyc11g065530.1.1	Lipase (Fragment) (AH	79.3	78.36	63.96	39.36	65.25	334.78	374.6	221.47	310.28	4.76	1.13E-07
Solyc11g066510.1.1	Ring H2 finger protein	2.23	4.71	3.61	3.54	3.52	14.84	20.19	8.84	14.62	4.15	1.14E-07
Solyc06g071070.1.1	Short-chain dehydroge	59.42	46.39	54.36	64.11	56.07	7.73	13.75	8.5	9.99	0.18	1.15E-07
Solyc10g054780.1.1	Acetyl esterase (AHRI	47.53	46.19	49.79	37.92	45.36	6.14	10.22	6.04	7.47	0.16	1.18E-07
Solyc12g056830.1.1	ATP synthase delta sub	273.61	243.74	320.94	307.83	286.53	34.61	93.81	32.61	53.68	0.19	1.19E-07
Solyc03g097120.2.1	Heat stress transcriptio	26.42	25.06	31.8	25.23	27.13	75.07	78.12	73.07	75.42	2.78	1.23E-07
Solyc10g086150.1.1	Single-stranded DNA b	79.98	65.35	62.64	44.1	63.02	9.49	16.02	9.19	11.57	0.18	1.24E-07
Solyc12g042470.1.1	Reductase 2 (AHRD V	29.81	22.64	16.64	12.09	20.3	0	1.3	0.2	0.5	0.02	1.25E-07
Solyc08g007640.1.1	Lrr, resistance protein	8.38	7.46	12.11	7.41	8.84	0.34	0.85	0.21	0.47	0.05	1.26E-07
Solyc01g005030.2.1	Serine/threonine-prote	8.47	12.17	12.56	9.06	10.57	24.92	26.39	19.76	23.69	2.24	1.28E-07
Solyc10g051110.1.1	NAD dependent epime	59.96	42.82	54.64	40.75	49.54	11.75	10.39	6.59	9.58	0.19	1.31E-07
Solyc09g090070.1.1	Inorganic phosphate tr	9.79	9.69	18.05	8.66	11.55	48.65	45.08	40.09	44.61	3.86	1.33E-07
Solyc06g060130.2.1	Nucellin-like aspartic p	51.05	60.44	65.33	72.63	62.36	10.49	12.41	10.07	10.99	0.18	1.33E-07
Solyc07g062980.2.1	Squamosa promoter bi	9.92	10.15	12.87	15.44	12.09	0.4	0	0	0.13	0.01	1.35E-07
Solyc06g050630.2.1	Prephenate dehydroger	1.45	3.23	2	3.76	2.61	10.15	12.6	24.47	15.74	6.03	1.36E-07
Solyc01g087990.2.1	MADS-box transcripti	18.92	15.33	15.45	10.29	15	42.72	49.61	32.98	41.77	2.79	1.36E-07
Solyc03g118370.2.1	Serine carboxypeptidas	3.47	5.67	2.9	7.78	4.96	0	0	0	0	0	1.36E-07
Solyc07g041640.2.1	Growth-regulating fact	17.02	13.96	7.67	16.5	13.79	1.24	1.25	1.71	1.4	0.1	1.38E-07
Solyc12g056200.1.1	Glycosyltransferase-lik	17.24	19.84	5.81	11.18	13.52	0.1	0.21	0	0.1	0.01	1.39E-07
Solyc07g054450.2.1	Transcription factor (F	72.51	77.19	61.39	76.42	71.88	424.32	365.18	266.73	352.08	4.9	1.40E-07
Solyc05g041200.2.1	4-hydroxyphenylpyruv	44.93	44.1	69.38	27.45	46.46	176.05	159.81	133.93	156.6	3.37	1.44E-07
Solyc03g098020.2.1	Hydrolase alpha/beta f	23.39	21.25	23.22	28.06	23.98	2.87	5.39	3.36	3.87	0.16	1.45E-07
Solyc06g050350.2.1	Myosin heavy chain-lil	26.39	23.49	25.3	23.51	24.67	3.18	5.38	1.89	3.48	0.14	1.45E-07
Solyc06g051860.1.1	Inorganic phosphate tr	1.49	0.78	2.5	1.23	1.5	5.13	9.13	5.56	6.61	4.4	1.45E-07
Solyc01g008020.2.1	Equilibrative nucleosid	3.96	5.5	3.55	3.36	4.09	0.13	0.13	0.16	0.14	0.03	1.47E-07
Solyc09g061310.2.1	PPPDE peptidase dom	51.68	44.51	44.96	44.81	46.49	115.83	144	164.06	141.3	3.04	1.48E-07
Solyc01g109500.2.1	BURP domain-contain	2.92	9.54	6.33	6.21	6.25	0	0.13	0	0.04	0.01	1.52E-07
Solyc06g084140.2.1	High affinity sulfate tr	2.2	4.09	2.83	1.27	2.6	0	0	0	0	0	1.52E-07
Solyc04g076990.2.1	Receptor like kinase, R	37.5	44.09	48.83	47.72	44.54	3.14	6.23	16.31	8.56	0.19	1.54E-07
Solyc01g108340.2.1	Peptidyl-prolyl cis-tran	75.42	129.4	100.35	72.75	94.48	19.23	17.18	11.18	15.86	0.17	1.57E-07
Solyc12g099000.1.1	S-adenosylmethionine	419.1	273	395.48	449.05	384.16	59.12	97.96	134.45	97.18	0.25	1.57E-07
Solyc09g082340.2.1	Vicilin-like protein (Fr	7.75	26.37	37.14	16.7	21.99	0.09	0	0.34	0.14	0.01	1.58E-07
Solyc08g013730.2.1	Aquaporin (AHRD V1	41.68	39.47	50.49	36.57	42.05	4.86	7.45	4.2	5.5	0.13	1.61E-07
Solyc09g010080.2.1	Beta-fructofuranosidas	4.85	5.02	3.86	0.8	3.63	13.85	13.78	13.77	13.8	3.8	1.61E-07
Solyc03g082920.2.1	Heat shock protein (AI	88.78	73.22	69.79	113.99	86.45	156.54	241.1	265.95	221.2	2.56	1.61E-07
Solyc03g006030.2.1	Receptor like kinase, R	1.24	1.61	0.98	0.95	1.19	0	0	0.15	0.05	0.04	1.64E-07
Solyc03g119310.2.1	Unknown Protein (AH	78.17	76.02	44.99	59.33	64.63	6.47	10.88	4.92	7.42	0.11	1.64E-07
Solyc12g010590.1.1	O-acyltransferase WSI	23.47	32.82	11.39	9.86	19.39	0.11	0	0	0.04	0	1.66E-07
Solyc02g087970.1.1	Zinc finger-homeodom	1451.36	1696.25	2159.62	2690.78	1999.5	268.84	288.28	222.35	259.82	0.13	1.68E-07
Solyc12g006470.1.1	Aminotransferase-like	88.39	152.48	128.36	265.31	158.63	2.09	2.58	2.14	2.27	0.01	1.69E-07
Solyc03g116490.1.1	Os05g0176300 protein	50.54	44.51	45.82	51.93	48.2	113.9	172.54	107.2	131.21	2.72	1.74E-07
Solyc03g111990.2.1	Cytochrome P450 pseu	1.43	1.16	3.54	1.48	1.9	59.62	42.18	64.32	55.37	29.11	1.75E-07
Solyc08g076150.2.1	Pentatricopeptide repe	7.85	8.13	4.72	3.64	6.08	0.35	0.23	0	0.19	0.03	1.75E-07
Solyc08g061590.2.1	Pentatricopeptide repe	191.29	188.18	176.83	182.34	184.66	40.14	32.61	39.45	37.4	0.2	1.78E-07
Solyc01g060180.2.1	AMP-dependent synthe	24.74	19.85	30.01	29.41	26	4.22	4.92	2.54	3.89	0.15	1.81E-07
Solyc05g015060.2.1	26S protease regulator	0.41	0.48	0.16	1.92	0.74	6.46	6.4	2.55	5.14	6.92	1.81E-07
Solyc11g072890.1.1	Unknown Protein (AH	37.7	39.65	46.5	16.36	35.05	205.58	206.3	163.28	191.72	5.47	1.81E-07
Solyc06g071330.2.1	Nucleobase ascorbate	35.68	36.78	50.92	67.82	47.8	1.68	3.6	2.8	2.69	0.06	1.83E-07
Solyc03g118410.2.1	Acyl carrier protein (A	145.73	154.67	121.39	146.94	142.18	31.82	29.29	16.13	25.75	0.18	1.84E-07
Solyc02g030450.2.1	Protein SEY1 (AHRD	4.29	4.4	8.08	6.42	5.8	13.06	18.61	16.12	15.93	2.75	1.86E-07
Solyc07g007930.2.1	Alkaline alpha galacto	10.92	10.99	14.13	9.27	11.33	61.57	138.2	111.83	103.87	9.17	1.88E-07
Solyc01g111360.2.1	Peptidyl-prolyl cis-tran	39.76	36.92	28.89	34.64	35.05	89.61	117.37	80.92	95.97	2.74	1.88E-07
Solyc03g123630.2.1	Pectinesterase (AHRD	490.15	529.63	649.04	1302.59	742.85	1.55	9.85	4.93	5.44	0.01	1.91E-07
Solyc04g072810.2.1	CRAL/TRIO domain c	46.6	48.12	53.55	45.94	48.55	7.17	10.19	3.2	6.85	0.14	1.91E-07
Solyc09g008650.2.1	Peptidyl-prolyl cis-tran	16.99	18.65	17.7	8.81	15.54	1.14	1.38	0.84	1.12	0.07	1.93E-07
Solyc09g082720.2.1	Aldo/keto reductase fa	106.58	97.4	84.81	96.55	96.34	304.08	265.53	332.71	300.77	3.12	1.98E-07
Solyc01g005440.2.1	Jasmonate ZIM-domai	363.91	331.26	572.19	353.4	405.19	63.44	86.53	46.68	65.55	0.16	1.99E-07
Solyc12g055810.1.1	AAA-ATPase (AHRD	5.31	4.27	6.41	4.83	5.21	0.2	0.31	0.38	0.3	0.06	1.99E-07
Solyc08g069060.2.1	Beta-1 3-galactosyltrar	6.31	8.43	10.98	10.81	9.13	174.53	121.6	181.27	159.13	17.42	2.00E-07
Solyc01g098910.2.1	Mitochondrial carrier p	28.84	23.45	24.23	28.17	26.17	60.51	78.27	95.55	78.11	2.98	2.02E-07
Solyc06g072650.1.1	Auxin responsive SAU	0	0	0	0	0	5.61	5.34	1.91	4.29	428.67	2.02E-07
Solyc09g065480.1.1	Inositol-1 4 5-trisphosp	3.88	6.07	7.49	8.32	6.44	1.05	0.48	0.35	0.63	0.1	2.06E-07
Solyc01g094010.2.1	CXE carboxylesterase	154.06	175.45	79.21	91.39	125.03	7.21	11.07	4.04	7.44	0.06	2.07E-07
Solyc01g098760.2.1	Heavy metal-associate	163.08	141.57	169.3	205.16	169.78	23.04	37.43	21.57	27.35	0.16	2.10E-07
Solyc07g005690.2.1	Unknown Protein (AH	53.17	49.09	44.25	55.86	50.59	124.07	130.89	109.94	121.63	2.4	2.10E-07
Solyc06g068270.2.1	1-aminocyclopropane-	2.04	1.15	3.28	2.1	2.14	20.21	18.12	36.79	25.04	11.69	2.10E-07
Solyc10g079090.1.1	Chaperone protein dna	11.56	12.31	4.01	7.7	8.9	34.43	29.19	29.03	30.88	3.47	2.11E-07
Solyc01g066440.2.1	Unknown Protein (AH	38.16	34.91	27.77	43.88	36.18	4.9	6.1	2.83	4.61	0.13	2.11E-07

Solyc03g119930.1.1	Sulfate transporter like	7.33	7.16	7.97	8.34	7.7	0.58	0.7	0.71	0.66	0.09	2.11E-07
Solyc04g005060.2.1	F3I6.9 protein (AHRD	63.86	72.65	48.77	59.72	61.25	9.24	18.49	1.75	9.83	0.16	2.12E-07
Solyc11g044530.1.1	PAP fibrillin (AHRD V	19.37	22.49	23.18	19.46	21.12	0.91	4.12	2.51	2.51	0.12	2.22E-07
Solyc08g067850.2.1	Homology to unknown	6.04	6.29	7.82	9.17	7.33	19.24	19.17	30.07	22.83	3.11	2.25E-07
Solyc04g078640.1.1	Ethylene-responsive tr	38.08	38.62	54.85	11.9	35.86	184.22	238.12	155.58	192.64	5.37	2.27E-07
Solyc03g044300.2.1	AP2-like ethylene-resp	69.42	57.4	60.6	76.83	66.06	388.73	362.26	347.28	366.09	5.54	2.27E-07
Solyc04g074310.2.1	RNA-binding protein 6	11.67	8.39	5.56	7.3	8.23	0.91	0.55	0.22	0.56	0.07	2.30E-07
Solyc05g012020.2.1	MADS-box transcripti	0	2.66	2.88	3.46	2.25	1124.75	713.45	862.05	900.08	400.04	2.34E-07
Solyc06g082950.2.1	Photosystem I reaction	197.61	136.29	164.82	143.14	160.46	36.42	38.68	28.15	34.42	0.21	2.34E-07
Solyc10g084170.1.1	Bcl-2-associated athan	0.77	0	0	1.64	0.6	4.99	13.52	21.44	13.32	22.1	2.36E-07
Solyc03g111380.2.1	Tubulin beta-1 chain (A	17.36	13.6	18.84	17.29	16.77	1.91	2.83	2.48	2.41	0.14	2.38E-07
Solyc01g091270.2.1	AT2G45380 protein (F	39.41	35.46	33.39	24.33	33.15	195.6	292.21	217.19	235	7.09	2.38E-07
Solyc03g116510.1.1	Unknown Protein (AH	12.63	20.61	12.42	5.96	12.91	133.81	133.82	98.92	122.18	9.47	2.41E-07
Solyc10g011940.1.1	Chromodomain helicas	2.98	3.15	5.04	2.07	3.31	0.09	0.18	0.17	0.15	0.04	2.47E-07
Solyc07g007280.2.1	Conserved domain pro	6.28	9.55	7.1	5.55	7.12	0.21	0.43	0	0.21	0.03	2.54E-07
Solyc01g006230.2.1	Cysteine protease (AH	20.36	24.41	24.22	26.42	23.85	62	69.39	63.48	64.96	2.72	2.54E-07
Solyc01g087210.2.1	Cellulose synthase (AH	183.06	211.67	194.8	207.46	199.25	35.65	46.57	34.19	38.8	0.19	2.68E-07
Solyc02g089080.2.1	Rapid alkalinization fa	425.87	459.24	195.13	212.48	323.18	10.76	13.63	10.45	11.61	0.04	2.70E-07
Solyc11g006670.1.1	Plant-specific domain	10.5	13.9	6.67	6.86	9.48	0.44	0.22	0	0.22	0.02	2.73E-07
Solyc03g044670.1.1	Phosphoglycerate dehy	6.57	5.85	13.24	5.42	7.77	21.23	26.77	30.16	26.05	3.35	2.73E-07
Solyc06g005710.2.1	cDNA clone 002-143-C	21.77	19.37	21.3	23.09	21.38	3.65	4	2.64	3.43	0.16	2.73E-07
Solyc12g015690.1.1	Fasciclin-like arabinog	54.4	48.42	27.59	83.57	53.49	0.99	1.6	1.22	1.27	0.02	2.73E-07
Solyc02g084720.2.1	Beta-galactosidase (AH	151.44	190.72	208.56	168.65	179.84	48.51	47.72	28.48	41.57	0.23	2.77E-07
Solyc07g019670.2.1	Fatty acid oxidation co	70.71	56.61	68.59	44.98	60.22	157.02	142.49	178.72	159.41	2.65	2.79E-07
Solyc06g084290.2.1	Os06g0207500 protein	7.77	8.61	7.96	7.05	7.85	32.22	22.94	46.13	33.76	4.3	2.82E-07
Solyc06g034150.2.1	8-amino-7-oxononanoa	15.19	16.65	16.76	20.29	17.22	3.98	2.41	2.37	2.92	0.17	2.85E-07
Solyc07g008020.2.1	Auxin response factor	7.37	13.11	7.51	8.05	9.01	0	0	0	0	0	2.88E-07
Solyc01g104140.2.1	Phosphatidylinositol-4	14.22	16.06	14.36	12.28	14.23	40.71	33.23	36.25	36.73	2.58	2.89E-07
Solyc04g077190.2.1	Endo-1 4-beta-xylanase	2.48	2.05	2.31	2.44	2.32	0.05	0.19	0.17	0.14	0.06	2.92E-07
Solyc10g008520.2.1	Auxin-responsive GH3	7.77	10.22	12.52	4.02	8.63	0.27	0.09	0.33	0.23	0.03	2.93E-07
Solyc12g019550.1.1	Unknown Protein (AH	13.83	17.05	12.55	19.57	15.75	0	0.87	0.53	0.47	0.03	3.00E-07
Solyc12g040640.1.1	Beta-glucosidase (AHF	29.92	25.23	8.48	13.86	19.37	0.11	0.21	0	0.11	0.01	3.03E-07
Solyc06g036170.1.1	Scarecrow-like 1 trans	32.71	34.8	50.55	33.03	37.77	109.18	91.9	156.44	119.17	3.16	3.04E-07
Solyc05g007770.2.1	NAC domain transcrip	0.7	1.24	3.3	1.32	1.64	39.74	29.06	20.59	29.8	18.17	3.04E-07
Solyc05g013530.2.1	Octicosapeptide/Phox/	10.2	14.68	10.26	8.48	10.91	30.04	34.21	34.82	33.02	3.03	3.05E-07
Solyc01g091630.2.1	Homeobox-leucine zip	25.76	25.93	28.88	43.14	30.93	69.18	73.05	72.57	71.6	2.32	3.09E-07
Solyc01g058670.2.1	Transcription factor (A	139.88	151.05	174.4	212.57	169.48	32.73	34.8	48.91	38.81	0.23	3.22E-07
Solyc06g009140.2.1	Late embryogenesis ab	555.55	339.3	562.85	157.16	403.71	980.69	1122.68	1954.82	1352.73	3.35	3.22E-07
Solyc06g074750.1.1	Histone H2B (AHRD V	2.09	2.07	4.66	6.57	3.85	9.95	17.79	22.83	16.86	4.38	3.25E-07
Solyc09g015080.2.1	Phosphatidylinositol tr	6.99	8.84	5.86	2.63	6.08	0.13	0.13	0.15	0.14	0.02	3.34E-07
Solyc07g007760.2.1	Defensin protein (AHR	2744.06	3667.76	2432.5	3316.85	3040.29	1081.49	755.33	366.37	734.4	0.24	3.35E-07
Solyc05g013640.1.1	Unknown Protein (AH	100.11	135.45	75.78	72.03	95.84	265.56	321.6	99.95	229.04	2.39	3.35E-07
Solyc08g005800.2.1	Pectinacetylesterase lik	561.08	560.81	670.23	1063.06	713.79	21.07	57.69	32.41	37.06	0.05	3.37E-07
Solyc02g087800.2.1	Aldose-1-epimerase-lik	22.61	18.64	15.09	19.13	18.87	0.75	4.53	0.18	1.82	0.1	3.37E-07
Solyc02g077940.1.1	Unknown Protein (AH	64.77	100.35	142.93	110.28	104.58	279.09	301.04	384.56	321.56	3.07	3.38E-07
Solyc10g080940.1.1	Tubulin beta chain (AH	423.21	396.09	385.38	546.71	437.85	43.53	133.62	38.45	71.87	0.16	3.39E-07
Solyc04g050370.1.1	60S ribosomal protein	92.74	82.54	80.09	128.48	95.96	7.72	11.69	8.54	9.32	0.1	3.50E-07
Solyc11g009000.1.1	Carboxylesterase bioH	16.35	22.86	22.39	12.39	18.5	65.33	47.81	38.83	50.66	2.74	3.57E-07
Solyc08g080050.2.1	PGR5-like protein 1A,	118.06	127.12	80.85	70.06	99.02	15.18	18.27	11.44	14.96	0.15	3.69E-07
Solyc05g005250.2.1	Pantothenate kinase (A	26.42	25.99	23.51	23.17	24.77	61.41	73.78	46.77	60.65	2.45	3.74E-07
Solyc12g098580.1.1	UDP-glucosyltransfera	0.28	0.13	0.33	0.4	0.29	3.2	3.69	2.53	3.14	11.02	3.77E-07
Solyc02g067250.1.1	Structural constituent c	3.33	9.16	3.93	2.57	4.75	23.96	20.22	19.22	21.13	4.45	3.78E-07
Solyc01g096450.2.1	Aspartic proteinase ne	56.91	53.29	55.57	124.84	72.65	0.84	1.82	1.18	1.28	0.02	3.79E-07
Solyc02g065400.2.1	Oxygen-evolving enha	544.56	436.44	403.78	347.25	433.01	106.11	118.1	100.8	108.34	0.25	3.80E-07
Solyc02g067380.2.1	Transcription factor sty	33.33	37.08	9.01	22.59	25.5	0	0	0	0	0	3.80E-07
Solyc08g080780.2.1	SKIP interacting protei	24.96	26.18	25.92	36	28.27	4.24	8.06	2.79	5.03	0.18	3.83E-07
Solyc07g062410.2.1	Unknown Protein (AH	27.38	28.6	24	24.74	26.18	2.92	5.58	2.64	3.71	0.14	3.84E-07
Solyc07g043510.2.1	Cysteine-type peptidas	23.26	21.54	10.64	16.78	18.05	92.03	66.91	117.3	92.08	5.1	3.93E-07
Solyc08g067860.1.1	Homology to unknown	5.01	5.41	4.01	5.31	4.93	17.48	16.03	23.25	18.92	3.83	3.93E-07
Solyc08g080380.2.1	Cytochrome P450	7.17	9.45	8.61	13.14	9.59	0.54	1.73	0.79	1.02	0.11	3.94E-07
Solyc11g006020.1.1	NAD(P)H-quinone oxi	60.64	41.21	30.46	23.18	38.87	0	3.01	1.63	1.55	0.04	4.03E-07
Solyc05g047550.2.1	Receptor-like protein k	14.75	14.04	13.31	14.87	14.24	1.44	3.25	0.67	1.79	0.13	4.10E-07
Solyc10g054820.1.1	Aquaporin (AHRD V1	4.65	4.5	3.34	2.86	3.84	0	0	0	0	0	4.10E-07
Solyc06g076560.1.1	class I heat shock prote	83	62.5	79.84	65.62	72.74	1039.15	1571.11	1591.85	1400.7	19.26	4.12E-07
Solyc03g044200.2.1	Alcohol dehydrogenase	11.17	14.76	12.15	9.38	11.87	39.2	34.56	31.87	35.21	2.97	4.13E-07
Solyc02g087050.2.1	Nodulin MtN21 family	12.42	12.53	12.02	12.32	12.32	0.6	2.11	0.37	1.03	0.08	4.15E-07
Solyc01g104950.2.1	Alpha-L-arabinofurano	30.83	42.13	44.76	35.7	38.36	14.85	6.21	8.91	9.99	0.26	4.17E-07
Solyc08g006460.2.1	RING-finger protein li	13.14	17.99	12.73	7.54	12.85	38.47	35.77	24.62	32.95	2.56	4.21E-07
Solyc02g067040.2.1	cDNA clone J075153F	0.12	0.31	0.28	0	0.18	13.68	8.15	12.37	11.4	64.23	4.25E-07
Solyc04g081790.2.1	GDSL esterase/lipase A	4.51	6.36	3.99	3.19	4.51	0	0.15	0	0.05	0.01	4.31E-07
Solyc09g025210.2.1	Legumin 11S-globulin	39.3	133	236.12	58.94	116.84	0	0	0.14	0.05	0	4.39E-07
Solyc12g044280.1.1	Photosystem I reaction	39.21	29.97	22.86	15.66	26.93	1.12	2.63	0.92	1.56	0.06	4.46E-07
Solyc11g066680.1.1	UDP-glucuronosyltran	7.52	8.84	11.38	15.45	10.8	1.15	1.04	0.7	0.96	0.09	4.53E-07
Solyc11g008820.1.1	Endoglucanase (AHRD	25.55	27.42	27.34	28.13	27.11	3.3	5.58	3.01	3.96	0.15	4.55E-07
Solyc06g073910.2.1	Strictosidine synthase (	40.23	36.19	42.87	31.82	37.78	7.16	9.34	4.51	7	0.19	4.57E-07
Solyc11g066130.1.1	Thaumatin-like protein	43.81	42.7	56.3	72.29	53.78	9.9	9.56	8.8	9.42	0.18	4.58E-07
Solyc05g007800.2.1	Negatively light-regula	75.18	61.44	39.82	65.1	60.38	186.42	173.77	162.33	174.17	2.88	4.67E-07
Solyc06g082300.2.1	UDP-glucosyltransfera	3.79	4.54	2.06	2.06	3.11	0	0.12	0	0.04	0.01	4.67E-07
Solyc02g090430.2.1	Protein serine/threonin	4.03	3.4	5.24	5.11	4.45	0.17	0.42	0.41	0.33	0.07	4.68E-07
Solyc02g081560.1.1	Unknown Protein (AH	6.89	9.64	5.81	5.57	6.98	27.84	44.13	136.83	69.6	9.97	4.74E-07
Solyc07g066610.2.1	Phosphoglycerate kina	243.4	219.3	211.58	210.2	221.12	42.48	67.34	42.98	50.93	0.23	4.74E-07
Solyc08g075750.2.1	ATP-dependent Clp pr	59.44	51.35	60.62	60.98	58.1	139.14	166.06	180.02	161.74	2.78	4.75E-07

Solyc01g104650.2.1	BZIP transcription fact	36.53	37.24	46.49	27.56	36.96	121.08	287.01	263.69	223.93	6.06	4.87E-07
Solyc06g082970.2.1	DnaJ domain family (A	35.16	34.34	23.5	25.09	29.52	3.27	6.34	2.47	4.03	0.14	4.87E-07
Solyc11g006930.1.1	4&apos-phosphopante	8	12.45	9.43	8.48	9.59	0.46	0.7	1.13	0.76	0.08	4.95E-07
Solyc01g103650.2.1	Hydrolase alpha/beta f	7.15	8.88	14.23	13.48	10.94	0.12	1.21	0.74	0.69	0.06	5.00E-07
Solyc03g112620.2.1	Carboxyl-terminal prot	5.78	8.86	7.2	9.08	7.73	0.26	0.52	0	0.26	0.03	5.03E-07
Solyc03g117810.2.1	Phosphate import ATP	49.98	51.6	30.07	27.57	39.8	4.24	4.89	5.46	4.86	0.12	5.11E-07
Solyc08g082540.2.1	Sad1/UNC domain pro	10.68	10.5	7.96	14.47	10.9	29.69	26.74	39.04	31.82	2.92	5.13E-07
Solyc06g062430.2.1	Inositol oxygenase (AH	3.88	6.72	3.31	3.05	4.24	12.89	15.81	4.49	11.06	2.61	5.24E-07
Solyc02g067320.1.1	Zinc finger-homeodom	5.96	4.95	7.03	8.15	6.52	0.32	0.32	0	0.21	0.03	5.37E-07
Solyc03g095860.2.1	Ankyrin repeat domain	8.3	6.65	8.81	8.02	7.95	40.45	44.74	33.22	39.47	4.97	5.54E-07
Solyc03g098710.1.1	Kunitz-type proteinase	7.33	6	2.42	6.22	5.49	0	0	0	0	0	5.55E-07
Solyc03g114210.2.1	Protein kinase (AHRD	9.26	8.99	11.91	9.76	9.98	0.14	1.23	0.17	0.51	0.05	5.58E-07
Solyc07g039310.1.1	Polyamine oxidase (AH	3.47	4.34	2.88	2.54	3.31	0.31	0	0.25	0.19	0.06	5.85E-07
Solyc03g111700.2.1	Inactivated Zn-depend	77.49	85.29	76.22	51.63	72.66	14.82	18.93	12.59	15.45	0.21	5.89E-07
Solyc09g008190.2.1	Unknown Protein (AH	137.44	171.25	84.32	114.05	126.77	950.49	913.88	587.52	817.3	6.45	5.92E-07
Solyc07g043310.2.1	Aminotransferase (AH	35.93	47.56	66.37	22.42	43.07	127.26	99.28	106.47	111	2.58	6.25E-07
Solyc08g077100.2.1	Baculoviral IAP repeat	10.11	12.74	7.16	16.73	11.69	0.38	1.39	0.31	0.69	0.06	6.32E-07
Solyc06g071050.2.1	Spfh domain / band 7 f	65.29	54.26	79.98	60.49	65.01	10.84	10.01	13.77	11.54	0.18	6.34E-07
Solyc04g012020.1.1	Hydroxycinnamoyl Co	3.54	3.28	1.92	2.09	2.71	0	0	0	0	0	6.36E-07
Solyc06g007980.2.1	Sterol 3-beta-glucosylt	85.2	94.52	116.38	170.73	116.71	6.62	12.84	13.45	10.97	0.09	6.37E-07
Solyc07g007270.2.1	Unknown Protein (AH	16.73	23.83	15.79	6.09	15.61	0	0	0	0	0	6.37E-07
Solyc06g063090.2.1	Alanine aminotransfer	18.59	10.7	12.9	12.89	13.77	30.53	37.62	29.79	32.65	2.37	6.37E-07
Solyc01g005410.2.1	Calcium binding prote	20.62	18.96	8.51	10.21	14.57	0.56	0	0	0.19	0.01	6.39E-07
Solyc10g083720.1.1	Pyruvate kinase (AHR	14.12	14.34	12.09	9.01	12.39	87.22	124.42	136.55	116.06	9.37	6.40E-07
Solyc12g095880.1.1	Phosphofructokinase f	22.94	24.77	26.1	26.31	25.03	60.1	76.54	53.89	63.51	2.54	6.40E-07
Solyc02g079340.2.1	Unknown Protein (AH	35.08	43.34	30.89	33.33	35.66	3.81	5.12	3.64	4.19	0.12	6.43E-07
Solyc05g012290.2.1	DUF647 domain-conta	28.57	33.42	19.98	19.16	25.28	4.64	3.6	2.78	3.67	0.15	6.51E-07
Solyc10g085140.1.1	Undecaprenyl pyropho	4.83	4.3	4.43	2.28	3.96	27.73	53.32	11.71	30.92	7.81	6.69E-07
Solyc04g074840.2.1	Multidrug resistance p	0	0.35	0.46	1.11	0.48	467	235.08	817.23	506.44	1055.08	6.75E-07
Solyc11g008140.1.1	Pectate lyase family p	5.86	6.65	4.67	6.01	5.8	0.59	0.48	0	0.36	0.06	6.80E-07
Solyc03g112130.1.1	AT5G28150-like prote	2.9	3.5	4.64	4.68	3.93	0	0	0	0	0	6.82E-07
Solyc05g013380.2.1	Alanine aminotransfer	71.86	75.77	76.22	76.86	75.18	11.72	20.52	10.46	14.23	0.19	6.85E-07
Solyc06g071290.2.1	Betaine aldehyde dehy	63.75	59.88	55.64	83.15	65.6	14.56	18.1	10.37	14.34	0.22	6.89E-07
Solyc03g113340.2.1	Nodulin-like protein (I	4.36	5.39	6.85	4.97	5.39	12.32	18.05	5.65	12.01	2.23	7.17E-07
Solyc08g076170.1.1	Unknown Protein (AH	12.72	26.17	12.19	15.74	16.7	0	0	0	0	0	7.24E-07
Solyc06g036130.2.1	Multidrug resistance p	13.46	13.95	12.48	18.11	14.5	94.35	100.17	106.85	100.46	6.93	7.33E-07
Solyc07g043230.2.1	Low affinity zinc trans	9.42	16.78	9.76	11.97	11.98	0.47	1.25	0.19	0.64	0.05	7.34E-07
Solyc06g053960.2.1	Heat stress transcriptio	158.24	204.88	237.85	85.49	171.62	1822.21	1272.51	1185.55	1426.76	8.31	7.40E-07
Solyc06g071550.2.1	Anthranilate phosphor	21.01	21.62	22.27	20.43	21.33	3.45	4.81	2.44	3.57	0.17	7.40E-07
Solyc08g007630.1.1	Cc-nbs-lrr, resistance p	2.73	2.91	4.96	2.83	3.36	0.38	0.38	0.2	0.32	0.1	7.44E-07
Solyc03g116370.2.1	Microtubule plus-end b	7.63	9.42	4.36	5.22	6.66	0.2	0.2	0	0.13	0.02	7.54E-07
Solyc02g065060.2.1	Short-chain dehydroge	32.12	42.88	93.57	92.27	65.21	0.8	1.21	0.49	0.83	0.01	7.67E-07
Solyc09g074660.2.1	Thymidylate kinase (A	18.53	18.34	26.56	16.04	19.87	51.48	49.1	48.9	49.83	2.51	7.75E-07
Solyc02g081390.2.1	Amine oxidase family	84.46	87.83	79.56	106.49	89.58	367.5	266.3	407.42	347.07	3.87	7.77E-07
Solyc06g008230.2.1	Calmodulin-binding pr	6.19	7.94	6.33	6.05	6.63	19.48	19.95	24.57	21.33	3.22	7.83E-07
Solyc06g066020.2.1	Auxin response factor	7.33	4.83	5.12	2.3	4.9	0	0	0	0	0	7.85E-07
Solyc06g034390.1.1	Expressed protein (Fra	4.56	3.38	3.59	4.84	4.09	0.15	0	0	0.05	0.01	7.91E-07
Solyc01g103590.2.1	Glyoxalase/bleomycin	48.38	76.46	48.43	42.3	53.89	1.9	15.53	3.2	6.88	0.13	8.13E-07
Solyc03g113930.1.1	class IV heat shock pro	0	0	0	0.49	0.12	90.24	116.9	121.96	109.7	895.51	8.18E-07
Solyc11g072510.1.1	Cinnamoyl-CoA reduc	31.46	38.12	23.47	18.66	27.93	3.28	5.13	1.41	3.27	0.12	8.18E-07
Solyc05g046020.2.1	Peroxidase (AHRD V1	3.42	5.37	3.08	2.56	3.61	0	0	0	0	0	8.27E-07
Solyc03g120550.2.1	Peptide transporter 1 (	40.34	45.34	74.43	95.21	63.83	226.08	226.27	499.85	317.4	4.97	8.47E-07
Solyc04g076980.2.1	LRR receptor-like seri	36.56	47.71	48	46.66	44.73	3.11	6.5	17.46	9.02	0.2	8.52E-07
Solyc05g010810.1.1	Phosphatidylinositol k	68.96	65.68	117.29	116.61	92.14	205.05	230.21	428.04	287.77	3.12	8.58E-07
Solyc07g045440.1.1	Fasciclin-like arabinog	215.77	183.02	316.83	458.16	293.44	3.9	12.59	3.67	6.72	0.02	8.62E-07
Solyc05g008060.2.1	Auxin efflux carrier (A	23	28.82	21.66	26.68	25.04	8.56	4.03	4.71	5.77	0.23	8.71E-07
Solyc07g045090.2.1	Alcohol dehydrogenas	0.76	0.34	0.45	3.25	1.2	121.89	97.39	51.82	90.37	75.31	8.72E-07
Solyc08g006510.2.1	NAD dependent epime	29.37	29.88	21.73	25.06	26.51	77.03	76.17	59	70.73	2.67	8.79E-07
Solyc11g068730.1.1	Nitrilase 4A (AHRD V	29.42	27.69	30.73	37.11	31.24	59.66	104.46	94.69	86.27	2.76	8.81E-07
Solyc12g056780.1.1	Prostaglandin E syntha	59.77	53.85	43.43	46.92	50.99	134.87	132.76	147.24	138.29	2.71	8.87E-07
Solyc04g005380.2.1	Ninja-family protein O	54.83	48.65	40.63	13.09	39.3	0.55	1.95	0.85	1.12	0.03	8.92E-07
Solyc10g007590.2.1	Polyadenylate-binding	59.84	72.08	47.4	51.44	57.69	163.31	147.32	111.87	140.83	2.44	8.97E-07
Solyc03g117490.2.1	Glycerol kinase (AHR	24.98	32.89	22.17	19.98	25.01	4.38	5.96	2	4.11	0.16	9.07E-07
Solyc01g095300.2.1	Glycosyl transferase g	9.65	12.09	9.96	11.32	10.76	1.69	1.71	0.63	1.34	0.12	9.14E-07
Solyc01g104500.2.1	Os07g0419800 protein	107.92	128.57	120.94	132.56	122.5	545.01	444.4	469.12	486.18	3.97	9.35E-07
Solyc03g121610.2.1	Receptor-like kinase (	8.18	10	11.85	11.68	10.43	1.68	1.94	2.44	2.02	0.19	9.38E-07
Solyc05g005970.2.1	Peptide transporter (A	7.49	6.16	10.18	11.56	8.85	0.73	1.85	0.23	0.94	0.11	9.61E-07
Solyc09g020190.2.1	Phosphoesterase famil	29.35	31.19	41.92	31.22	33.42	63.68	89.13	55.13	69.31	2.07	9.62E-07
Solyc11g066280.1.1	U3 small nucleolar rib	50.26	49.45	53.51	36.38	47.4	5.23	11.36	2.34	6.31	0.13	9.69E-07
Solyc09g083190.2.1	Genomic DNA chromo	112.49	145.57	59.54	57.62	93.81	4.42	2.97	3.62	3.67	0.04	9.74E-07
Solyc03g121890.1.1	Os01g0584100 protein	8.47	7.34	18.66	17.1	12.89	34.95	41.33	69.52	48.6	3.77	9.81E-07
Solyc03g096990.2.1	Cytochrome P450 89A	27.38	29.41	46.39	41.8	36.25	77.17	92.38	92.9	87.48	2.41	9.83E-07
Solyc09g075810.2.1	Blue copper protein (A	35.5	37.33	14.8	11.66	24.82	0.29	0	0	0.1	0	9.83E-07
Solyc00g156980.2.1	Choline dehydrogenas	37.93	57.61	13.13	21.58	32.56	0	0	0	0	0	9.88E-07
Solyc07g053530.1.1	Fasciclin-like arabinog	5.78	4.68	3.72	4.83	4.75	0	0	0	0	0	9.88E-07
Solyc11g006230.1.1	Calcium-responsive tra	9.17	10.27	11.17	10.04	10.16	0.24	0.48	0.59	0.44	0.04	9.90E-07
Solyc01g005420.2.1	SRC2-like protein (AH	18.35	21.89	8.75	24.3	18.32	0.32	1.59	0.77	0.89	0.05	1.01E-06
Solyc01g109580.2.1	Adenylyl cyclase-asso	7.95	6.83	4.61	10.65	7.51	0.11	1.02	0.55	0.56	0.07	1.03E-06
Solyc03g117360.2.1	WD-40 repeat protein-	36.92	37.95	46.38	33.17	38.61	7.82	8.7	10.02	8.85	0.23	1.03E-06
Solyc05g052600.2.1	Fructose-1 6-bisphosp	99.54	108.96	82.76	64.26	88.88	21.18	21.64	15.91	19.58	0.22	1.04E-06
Solyc07g055060.2.1	Phosphoenolpyruvate c	38.76	40.1	70.41	61.79	52.76	129.19	104.45	138.36	124	2.35	1.05E-06
Solyc04g039990.2.1	Cc-nbs, resistance prot	9.49	14.26	11.38	11	11.53	1.38	1.14	0.77	1.1	0.1	1.06E-06

Solyc12g010910.1.1	Lipase (Fragment) (AH	10.75	8.14	6.82	2.5	7.05	0	0	0.32	0.11	0.02	1.07E-06
Solyc02g078020.2.1	Binding protein (Fragn	6.88	5.17	8.51	5.95	6.63	19.58	16.54	28.76	21.63	3.26	1.08E-06
Solyc03g062720.2.1	Galactose mutarotase-l	3	3	3.98	3.71	3.42	0	0	0	0	0	1.08E-06
Solyc02g079990.2.1	RLK, Receptor like pro	16.12	15.05	10.85	15.11	14.28	1.84	3.22	1.57	2.21	0.15	1.08E-06
Solyc00g007220.2.1	Ring finger protein (Al	4.3	6.06	10.57	8.11	7.26	58.8	40.61	37.72	45.71	6.3	1.09E-06
Solyc02g078660.2.1	Phosphoinositide-bind	1.42	2.33	3.93	3.11	2.7	0.06	0.06	0	0.04	0.01	1.09E-06
Solyc11g012700.1.1	Oligopeptide transport	11.34	12.19	16.88	22.71	15.78	42.3	63.71	58.55	54.85	3.48	1.10E-06
Solyc03g117270.1.1	F-box family protein (A	5.45	5.54	7.53	3.52	5.51	0.25	0.63	0	0.29	0.05	1.10E-06
Solyc01g087950.2.1	Serine carboxypeptidas	0.13	0	0.47	0.19	0.2	123.68	64.44	135.75	107.96	546.62	1.10E-06
Solyc02g072440.2.1	Receptor like kinase, R	7.44	12.72	7.65	5.88	8.42	0.53	1.01	1.09	0.88	0.1	1.12E-06
Solyc03g044150.2.1	Subtilisin-like protease	69.13	63.96	50.41	120.75	76.06	2.83	4.39	2.54	3.25	0.04	1.13E-06
Solyc04g082810.2.1	AT-hook DNA-binding	0.8	1.19	0.95	3.41	1.59	5.42	11.8	6.12	7.78	4.9	1.13E-06
Solyc03g122350.2.1	Cytochrome P450	17.89	13.03	35.78	23.28	22.5	66.18	53.86	156.05	92.03	4.09	1.14E-06
Solyc04g012030.2.1	Palmitoyltransferase P	14.62	17.86	8.43	10.34	12.81	1.92	1.52	0.84	1.43	0.11	1.14E-06
Solyc10g055410.1.1	MYB transcription fac	18.3	30.64	17.61	17.37	20.98	4.08	3.14	1.67	2.96	0.14	1.16E-06
Solyc07g006730.2.1	Multidrug resistance p	33.66	39.9	36.69	38.66	37.23	6.53	7.46	4.14	6.04	0.16	1.19E-06
Solyc06g071210.2.1	Ribosomal protein S6 l	35.6	33.32	36.17	28.97	33.52	126.8	112.58	111.89	117.09	3.49	1.20E-06
Solyc02g081820.2.1	HAD-superfamily hydr	0.27	0.48	0	0	0.19	3.71	7.71	4.03	5.15	27.47	1.23E-06
Solyc10g009090.2.1	Mitochondrial ornithin	7.11	5.76	3.56	4.88	5.33	0	0.35	0.43	0.26	0.05	1.24E-06
Solyc03g095510.2.1	Protein kinase 2 (AHR	2.27	2.53	2.01	1.01	1.96	12.29	10.77	9.6	10.89	5.57	1.25E-06
Solyc02g069240.1.1	Protein-O-fucosyltrans	0.45	0.94	0.71	0.21	0.58	5	4.06	7.34	5.47	9.47	1.27E-06
Solyc06g050810.2.1	Os03g0816700 protein	9.8	10.46	3.85	7.85	7.99	0	0.27	0	0.09	0.01	1.29E-06
Solyc07g051920.1.1	Protein serine/threonin	0.82	0.18	0.24	0.29	0.38	10.4	7.32	7.3	8.34	21.8	1.30E-06
Solyc03g117630.1.1	heat shock protein (AH	2.12	1.7	1.19	1.71	1.68	30.43	57.12	68.08	51.88	30.88	1.32E-06
Solyc04g072400.2.1	Endoribonuclease E-lil	33.51	33.68	26.47	23.06	29.18	67.68	92.89	93.99	84.85	2.91	1.33E-06
Solyc04g011670.2.1	BZIP transcription fact	7.39	10.02	13.49	14.43	11.33	1.14	0.86	1.57	1.19	0.11	1.33E-06
Solyc11g013480.1.1	Auxin response factor	4.42	3.08	2.5	1.09	2.77	10.26	13.95	9.93	11.38	4.1	1.34E-06
Solyc01g106350.2.1	Glutamyl-tRNA(Gln) a	5.05	3.32	3.5	4.35	4.05	0.27	0.27	0.22	0.25	0.06	1.35E-06
Solyc06g035970.2.1	Tubulin beta chain (AH	637.03	577.44	416.78	556.98	547.06	110.4	158.99	103.49	124.29	0.23	1.35E-06
Solyc01g080020.2.1	Xylanase inhibitor (Fra	31.88	52.81	36.5	36.39	39.39	6.5	9.9	3.13	6.51	0.17	1.40E-06
Solyc07g026930.1.1	Tetracycline transport	0	0	0.2	0	0.05	3.35	2.11	2.91	2.79	55.8	1.41E-06
Solyc03g095710.2.1	Alpha-amylase (AHRD	7.85	11.25	8.23	8.63	8.99	35.45	30.67	28.99	31.7	3.53	1.44E-06
Solyc04g072570.2.1	Receptor like kinase, R	13.4	13.23	11.49	17.92	14.01	1.86	3.07	1.97	2.3	0.16	1.44E-06
Solyc06g065720.1.1	DOMON domain-cont	5.12	5.47	6.04	10.55	6.79	14.18	16.45	21.78	17.47	2.57	1.46E-06
Solyc07g053480.2.1	Ras-related protein Ra	54.17	44.44	31.78	45.8	44.05	5.62	8.88	2.7	5.73	0.13	1.47E-06
Solyc03g097670.2.1	ATP binding protein (A	16.31	22.61	28.02	23.83	22.69	4.67	4.56	5.51	4.91	0.22	1.51E-06
Solyc09g008020.2.1	Cysteine-type peptidas	13.7	17.34	13.43	8.73	13.3	98.96	66.02	72.57	79.18	5.95	1.51E-06
Solyc07g063180.2.1	Cytoplasmic dynein lig	9.81	12.3	4.73	8.83	8.92	0.36	0	0	0.12	0.01	1.51E-06
Solyc12g005430.1.1	Acyltransferase-like pr	5.47	5	2.55	1.83	3.71	0	0	0	0	0	1.52E-06
Solyc01g080460.2.1	Pyruvate phosphate dik	282.19	269.62	283.21	218.8	263.45	630.2	562.23	471.12	554.52	2.1	1.58E-06
Solyc05g011830.2.1	Unknown Protein (AH	6.18	5.5	7.71	5.88	6.32	30.73	21.31	36.81	29.62	4.69	1.59E-06
Solyc07g062850.2.1	Genomic DNA chromo	10.39	11.52	13.12	9.54	11.14	29.89	28.36	33.46	30.57	2.74	1.60E-06
Solyc02g076710.2.1	Cathepsin B-like cyste	219.51	246.11	282.43	419.83	291.97	763.09	863.13	903.29	843.17	2.89	1.63E-06
Solyc01g059870.2.1	Phytochrome B1	12.3	17.27	22.21	19.62	17.85	3.11	4.89	3.18	3.73	0.21	1.64E-06
Solyc01g087560.2.1	24-sterol C-methyltran	63.31	58.38	58.32	59.7	59.93	159.9	131.22	136.06	142.39	2.38	1.64E-06
Solyc02g092120.2.1	Phytosulfokines 3 (AH	0	0	0.93	0	0.23	8.25	32	12.47	17.57	75.58	1.64E-06
Solyc03g114100.1.1	AT3g15630/MSJ11_3	35.08	54.99	61.15	73.32	56.13	6.77	10.67	5.72	7.72	0.14	1.65E-06
Solyc01g099800.2.1	Unknown Protein (AH	164.4	183.6	176.67	91.83	154.12	412.85	360.94	435.51	403.1	2.62	1.65E-06
Solyc12g009840.1.1	Pyrophosphate-energiz	11.14	9.38	11.42	11.39	10.83	1.25	2.58	2.38	2.07	0.19	1.66E-06
Solyc01g067830.2.1	U1 small nuclear ribon	13.52	17.32	14.01	18.2	15.76	33.31	56.71	55.66	48.56	3.08	1.69E-06
Solyc02g014450.1.1	TBC1 domain family r	6.08	7.27	5.57	7.19	6.53	0.81	1.4	0.72	0.98	0.15	1.70E-06
Solyc06g051940.2.1	Protein phosphatase 2C	261.5	201.85	207.84	120.7	197.97	20.09	50.72	5.76	25.52	0.13	1.70E-06
Solyc02g081400.2.1	Intracellular protease I	113.63	106.41	140.86	155.93	129.21	289.37	337.61	351.82	326.27	2.53	1.75E-06
Solyc02g068070.2.1	Lipase (AHRD V1 ***	5.98	5.89	5.57	11.14	7.15	0.51	0.26	0.31	0.36	0.05	1.75E-06
Solyc01g068130.2.1	Os05g0272200 protein	33.66	38.9	19.38	43.78	33.93	2.96	4.42	4.18	3.85	0.11	1.76E-06
Solyc12g049510.1.1	Erect panical 2 (AHRD	3.53	3.66	4.41	3.88	3.87	0.3	0.64	0.21	0.38	0.1	1.78E-06
Solyc02g092460.2.1	Speckle-type POZ prot	18.1	31.19	29.73	21.71	25.18	3.52	5.6	4.09	4.4	0.17	1.78E-06
Solyc09g014520.2.1	Chlorophyll a-b bindin	291.33	241.01	333.1	277.01	285.61	89.45	61.46	61.13	70.68	0.25	1.78E-06
Solyc01g111350.2.1	Nodulin family protein	161.47	206.45	267.64	239.86	218.85	424.37	500.41	666.29	530.36	2.42	1.78E-06
Solyc07g052460.2.1	Genomic DNA chromo	23.12	40.86	32.5	12.51	27.25	0.55	0	1.01	0.52	0.02	1.78E-06
Solyc08g006930.2.1	Photosystem I reaction	689.93	669.61	402.83	393.92	539.07	121.65	65.66	45.49	77.6	0.14	1.80E-06
Solyc04g054370.1.1	RING finger protein 13	20.67	31.48	31.41	26.42	27.5	0.96	6.15	3.55	3.55	0.13	1.80E-06
Solyc02g077920.2.1	Squamosa promoter bi	419.42	379.71	230.66	227.5	314.32	1241.43	792.24	1302.47	1112.05	3.54	1.86E-06
Solyc05g054560.2.1	UPF0497 membrane p	41.51	43.53	49.74	34.6	42.34	100.36	105.94	107.33	104.54	2.47	1.86E-06
Solyc01g008320.2.1	DDHD domain contain	15.64	17.65	19.04	14.58	16.73	39	42	45.58	42.19	2.52	1.90E-06
Solyc03g007870.2.1	Heavy metal-associate	79	84.45	52.37	85.25	75.27	204.64	214.7	106.71	175.35	2.33	1.90E-06
Solyc12g006630.1.1	Dynein light chain 1 cy	5.86	5.74	3.11	5.39	5.03	0	0	0	0	0	1.92E-06
Solyc06g051010.1.1	Peptide-N4-(N-acetyl-l	3.68	3.38	1.45	3.79	3.08	8.57	10.01	18.95	12.51	4.07	1.93E-06
Solyc07g016170.2.1	Galactokinase like pro	11.63	14.12	15	17.88	14.66	2.72	2.79	2.07	2.53	0.17	2.06E-06
Solyc02g082900.2.1	Strictosidine synthase	56.22	33.78	18.41	46.16	38.64	1	2.89	2.99	2.29	0.06	2.06E-06
Solyc08g080490.2.1	2S albumin seed storag	57.33	202.87	305.42	68.86	158.62	0	0	0.9	0.3	0	2.15E-06
Solyc01g009310.2.1	Sterol reductase (AHR	238.56	273.05	239.58	160.03	227.81	49.75	59.2	43.96	50.97	0.22	2.16E-06
Solyc06g005390.1.1	Histone H2B (AHRD V	2.67	1.59	1.05	1.26	1.64	18.01	15.62	12.83	15.49	9.43	2.17E-06
Solyc05g050830.1.1	Ethylene-responsive tr	26.23	28.89	22.06	29.76	26.74	4.41	6.77	2.58	4.59	0.17	2.19E-06
Solyc03g118070.2.1	5&apos-AMP-activate	8.48	10.06	6.17	6.19	7.73	0.8	1.15	0.84	0.93	0.12	2.20E-06
Solyc04g076400.2.1	Vernalization insensiti	2.7	1.83	1.02	3.21	2.19	0	0	0	0	0	2.22E-06
Solyc08g076880.2.1	Unknown Protein (AH	1.51	1.34	3.56	0.71	1.78	144.43	183.43	97.97	141.94	79.74	2.23E-06
Solyc03g043940.2.1	Unknown Protein (AH	19.94	15.02	14.48	8.68	14.53	74.37	80.01	54.82	69.73	4.8	2.24E-06
Solyc05g056480.2.1	Pyruvate dehydrogenas	17.06	16.43	14.8	16.55	16.21	33.03	52.84	22.67	36.18	2.23	2.25E-06
Solyc01g011350.2.1	cDNA clone J013057I	228.31	252.51	196.87	193.34	217.76	58.52	62.72	51.36	57.53	0.26	2.25E-06
Solyc10g007310.1.1	Light-dependent short	375.17	372.31	310.96	276.9	333.84	927.49	959.6	652.37	846.49	2.54	2.26E-06
Solyc03g095250.2.1	cDNA clone 001-032-I	9.83	9.79	9.66	15.55	11.21	0.89	2.34	1.58	1.6	0.14	2.29E-06

Solyc05g054570.2.1	CRAL/TRIO domain c	24.27	16.95	25.15	19.35	21.43	60.02	44.03	86.51	63.52	2.96	2.29E-06
Solyc08g066180.2.1	HMP-PP phosphatase (	7.07	7.89	5.29	6.82	6.77	0.45	1.37	0.33	0.72	0.11	2.33E-06
Solyc08g075470.2.1	Nodulin-like protein (A	11.02	11.19	8.33	9.26	9.95	1.39	1.82	1.37	1.53	0.15	2.34E-06
Solyc02g094060.2.1	Voltage-gated chloride	9.33	7.53	10.39	4.52	7.94	0.84	1.55	0.77	1.05	0.13	2.36E-06
Solyc07g008670.2.1	AT1G74160 protein (F	4.04	3.74	3.69	5.01	4.12	0.2	0.47	0	0.22	0.05	2.37E-06
Solyc07g018140.2.1	Serine/threonine protei	9.92	15.03	7.19	5.66	9.45	0	0.28	0.35	0.21	0.02	2.39E-06
Solyc06g053710.2.1	Ethylene receptor (AH	71.43	64.88	110.31	53.03	74.91	218.86	151.18	302.58	224.21	2.99	2.42E-06
Solyc01g090910.2.1	Vesicle-associated mer	67.94	56.46	67.85	62.21	63.62	14.58	15.39	15.39	15.12	0.24	2.45E-06
Solyc08g061270.1.1	Unknown Protein (AH	54.13	44.66	80.49	48.65	56.98	960.74	663.47	653.7	759.3	13.33	2.49E-06
Solyc01g099170.2.1	Lipoxygenase (AHRD	1.68	1.03	3.65	1.28	1.91	37.68	24.46	29.67	30.6	16.02	2.49E-06
Solyc01g095720.2.1	Lipase (AHRD V1 *-*	5.27	7.2	8.22	6.39	6.77	1.44	0.92	2.15	1.5	0.22	2.50E-06
Solyc03g080020.2.1	DUF895 domain meml	6.14	6.04	3.82	6.4	5.6	67.15	45.85	92.12	68.37	12.21	2.54E-06
Solyc09g011010.2.1	One helix protein (AH	106.84	97.68	78.52	72.67	88.93	20.75	20.46	15.65	18.95	0.21	2.55E-06
Solyc08g081540.2.1	1-aminocyclopropane-	2.45	2.43	2.73	1.35	2.24	17.4	18.22	13.39	16.34	7.29	2.55E-06
Solyc02g076730.2.1	Unknown Protein (AH	33.36	34.42	45.63	61.14	43.64	6.13	5.56	4.52	5.4	0.12	2.55E-06
Solyc03g111800.2.1	Receptor like kinase, R	6.38	3.29	10.44	4.91	6.25	0	0	0	0	0	2.57E-06
Solyc07g007670.2.1	Purple acid phosphatas	4.55	5.04	3.58	2.54	3.93	0.33	0.22	0.27	0.27	0.07	2.58E-06
Solyc02g084210.1.1	GDP-mannose 4 6-deh	38.27	29.36	21.07	54.13	35.71	1.91	2.97	1.45	2.11	0.06	2.59E-06
Solyc12g099310.1.1	Ubiquitin-conjugating	81.26	78.16	94.58	93.01	86.75	324.56	250.28	316.79	297.21	3.43	2.60E-06
Solyc04g011540.2.1	Elicitor-responsive pro	1.65	0.37	0.97	0	0.75	29.65	20.83	26.6	25.69	34.37	2.60E-06
Solyc11g012680.1.1	Proline-rich protein (A	14.66	15.82	19.65	10.68	15.2	2.51	2.17	0.66	1.78	0.12	2.63E-06
Solyc03g025760.2.1	Glycosyltransferase CA	7.56	6.5	3.17	2.54	4.94	0.1	0	0.13	0.08	0.02	2.72E-06
Solyc05g008900.2.1	COSII_At1g13380 (Fra	79.84	94.85	78.63	80.4	83.43	20.5	19.57	14.44	18.17	0.22	2.73E-06
Solyc06g066330.2.1	Poly polymerase cataly	14.75	19.34	20.14	16.94	17.79	51.33	41.56	43.36	45.42	2.55	2.74E-06
Solyc02g090030.2.1	Oxygen-evolving enha	376.86	323.1	343.25	329.4	343.15	70.37	104.14	72.74	82.42	0.24	2.79E-06
Solyc01g106480.2.1	Malate dehydrogenase	60.26	71.51	66.74	81.85	70.09	14.14	18.02	17.38	16.51	0.24	2.80E-06
Solyc10g047320.1.1	Cc-nbs-lrr, resistance p	34.47	38.3	47	49.61	42.34	6.98	7.95	7.76	7.56	0.18	2.80E-06
Solyc09g011660.2.1	Universal stress protei	180.32	269.37	127.83	165.14	185.66	31.98	26.39	27.38	28.58	0.15	2.81E-06
Solyc01g065980.2.1	Ethylene responsive tra	427.13	478	826.24	294.95	506.58	1026.68	1272.47	1075.19	1124.78	2.22	2.83E-06
Solyc02g076800.1.1	Unknown Protein (AH	101.71	90.21	64.05	43.26	74.81	13.32	5.04	4.09	7.48	0.1	2.83E-06
Solyc10g018140.1.1	Dihydroflavonol 4-redu	0	0.23	0	0	0.06	20.94	11.73	19.75	17.47	303.88	2.83E-06
Solyc11g013150.1.1	GRAS family transcrip	17.84	17.81	22.17	25.05	20.72	3.49	3.08	1.88	2.82	0.14	2.83E-06
Solyc08g006530.2.1	CONSTANS-like prote	26.95	17.61	28.69	17.86	22.78	3.35	4.15	1.5	3	0.13	2.88E-06
Solyc02g085000.2.1	Genomic DNA chromo	47.31	44.89	42.7	33.86	42.19	141.82	131.2	132.66	135.23	3.21	2.88E-06
Solyc07g051890.1.1	Protein serine/threonin	1.6	1.07	1.18	0.57	1.1	14.86	18.9	16.67	16.81	15.21	2.92E-06
Solyc10g085920.1.1	Bifunctional polymyxi	4.11	3.24	1.49	3.36	3.05	0.13	0	0	0.04	0.01	2.92E-06
Solyc03g013240.2.1	Homology to unknown	17.12	21.28	20.72	11.06	17.55	4.17	3.01	0.73	2.64	0.15	2.92E-06
Solyc02g085730.2.1	Allene oxide cyclase (A	636.02	531.54	578.5	335.94	520.5	82.01	162.37	69.22	104.53	0.2	2.94E-06
Solyc10g080020.1.1	F-box/LRR-repeat prot	10.91	10.05	10.65	7.81	9.86	23.1	29.74	28.63	27.16	2.76	2.95E-06
Solyc09g064940.2.1	Phenazine biosynthesis	99.07	130.45	143.98	132.17	126.42	572.64	569.5	553.36	565.17	4.47	2.96E-06
Solyc02g089150.2.1	MAPprotein kinase-lik	2.99	5.32	3.94	0.75	3.25	61.4	50.32	46.8	52.84	16.26	3.00E-06
Solyc05g051220.2.1	Potassium channel (AH	0.4	0.5	0.19	0	0.27	1.81	2.67	2.14	2.21	8.1	3.01E-06
Solyc07g040730.1.1	Unknown Protein (AH	479.6	445.01	551	640.62	529.06	116.33	165.54	112.26	131.38	0.25	3.07E-06
Solyc04g007070.2.1	Cc-nbs-lrr, resistance p	29.42	28.88	38.74	57.17	38.55	0.98	1.11	1.58	1.22	0.03	3.10E-06
Solyc03g118060.2.1	Unknown Protein (AH	0	0	0	0	0	22.24	43.43	45.53	37.07	3706.67	3.10E-06
Solyc02g078600.2.1	Solute carrier family 2	3.34	2.4	2.57	1.45	2.44	0	0	0	0	0	3.13E-06
Solyc01g079610.2.1	DNAJ chaperone (AHI	21.16	19.68	16.2	29.67	21.68	36.05	69.61	56.59	54.08	2.49	3.19E-06
Solyc09g091180.2.1	chaperonin (AHRD V1	54.26	46.26	42	49.88	48.1	86.29	140.42	115.16	113.96	2.37	3.22E-06
Solyc07g047850.2.1	Chlorophyll a-b bindin	144.59	117.87	106.8	63.85	108.28	23.04	10.31	10.34	14.56	0.13	3.27E-06
Solyc05g013690.2.1	GDSL esterase/lipase A	2.22	3.64	1.41	4.1	2.84	0	0	0.17	0.06	0.02	3.29E-06
Solyc02g092760.2.1	Hydrolase alpha/beta f	20.08	28.29	13.82	26.03	22.05	1.54	2.92	0.71	1.72	0.08	3.33E-06
Solyc10g007080.2.1	Aberrant lateral root fo	25.27	34.98	22.36	12.91	23.88	1.13	0	0.7	0.61	0.03	3.40E-06
Solyc08g082770.2.1	Nodulin MtN3 family p	7.33	10.25	5.25	2.59	6.35	0	0	0	0	0	3.41E-06
Solyc08g077500.2.1	Vacuolar protein sortir	1.86	2.55	3.36	2.48	2.56	6.63	5.03	7.21	6.29	2.45	3.42E-06
Solyc01g111560.2.1	Ras-like GTP-binding	215.81	252.85	183.82	147.71	200.05	58.39	50.25	38.5	49.05	0.25	3.43E-06
Solyc10g009080.2.1	Squamosa promoter bi	62.58	71.67	46.36	50.77	57.85	16.46	7.91	8.19	10.85	0.19	3.47E-06
Solyc05g052350.2.1	Receptor like kinase, R	5.82	8.44	6.48	4.82	6.39	1.48	0.68	0.83	1	0.16	3.49E-06
Solyc02g080490.2.1	MATE efflux family p	19.2	15.93	10.02	21.8	16.74	1.58	3.19	0.78	1.85	0.11	3.52E-06
Solyc01g010520.2.1	Uncharacterized plant-	9.92	13.08	11.27	12.22	11.62	0.89	2.55	0.36	1.27	0.11	3.52E-06
Solyc01g008420.2.1	Mate efflux family pro	4.42	4.37	3.62	3.82	4.06	0.3	0.4	0.12	0.27	0.07	3.54E-06
Solyc09g091010.2.1	Unknown Protein (AH	78.67	71.41	76.52	86.06	78.16	13.5	21.61	9.73	14.95	0.19	3.57E-06
Solyc04g016230.2.1	Glucosyltransferase (A	6.99	6.34	2.47	2.97	4.69	0	0.11	0	0.04	0.01	3.61E-06
Solyc04g071900.2.1	Peroxidase (AHRD V1	15.93	15.01	7.74	7.42	11.53	1.36	0.46	0.19	0.67	0.06	3.61E-06
Solyc09g014450.2.1	Unknown Protein (AH	44.26	37.9	57.8	37.41	44.34	111.77	104.11	98.91	104.93	2.37	3.61E-06
Solyc03g120150.2.1	Plant cell wall protein	22.88	27.51	32.61	32.37	28.84	73.47	79.91	83.77	79.05	2.74	3.61E-06
Solyc01g096100.2.1	AT2G46550 protein (F	60.55	69.22	72.1	81.14	70.75	187.95	142.26	174.94	168.38	2.38	3.64E-06
Solyc07g039510.2.1	Esterase/lipase/thioeste	43.9	44.26	31.73	41.27	40.29	6.76	8.06	3.27	6.03	0.15	3.65E-06
Solyc07g042300.1.1	Disease resistance resp	16.14	17.18	14.49	20.35	17.04	0.28	2.57	0.7	1.18	0.07	3.65E-06
Solyc07g005440.1.1	CBL-interacting protei	165.16	159.56	210.6	134.37	167.42	33.48	48.45	26.66	36.2	0.22	3.67E-06
Solyc02g092630.1.1	Unknown Protein (AH	37.48	62.72	63.68	44.54	52.1	164.74	140.5	196.45	167.23	3.21	3.68E-06
Solyc01g008850.2.1	CBL-interacting protei	69.43	85.6	104.43	110.11	92.39	154.68	218.95	323.09	232.24	2.51	3.69E-06
Solyc02g078940.2.1	Zinc-binding dehydrog	171.81	152.91	179.54	156.29	165.14	458.76	493.55	426.84	459.72	2.78	3.71E-06
Solyc10g080770.1.1	BZIP transcription fact	14.92	12.24	19.16	21.93	17.06	37.7	35.64	76.23	49.86	2.92	3.72E-06
Solyc11g045250.1.1	N-acetylated alpha-link	7.18	12.68	9.27	6.19	8.83	1.63	1.46	1.45	1.51	0.17	3.72E-06
Solyc01g098020.2.1	Acetyltransferase GNA	13.83	16.17	9.79	8.94	12.18	1.91	0.32	0.78	1	0.08	3.72E-06
Solyc10g084930.1.1	Serine/threonine-prote	9.46	8.75	9.76	8.71	9.17	19.52	24.93	24.08	22.84	2.49	3.79E-06
Solyc10g011820.2.1	Delta-6 desaturase (AH	7.07	9.19	6.2	8.46	7.73	0.73	0.59	0.54	0.62	0.08	3.82E-06
Solyc09g092330.1.1	NAD dependent epime	101.51	101.23	121.64	162.53	121.73	13.41	36.67	18.94	23.01	0.19	3.88E-06
Solyc03g098740.1.1	Kunitz trypsin inhibito	0.66	0.29	0.39	0	0.33	63.69	32.52	47.47	47.89	142.97	3.89E-06
Solyc02g077160.2.1	Lipase-like protein (AH	30.18	32.14	23.67	22.7	27.17	4.8	8.24	4.46	5.83	0.21	3.89E-06
Solyc12g088050.1.1	Rhamnogalacturonate	17.09	17.72	30.99	12.97	19.69	0.84	1.7	0.31	0.95	0.05	3.91E-06
Solyc09g064720.2.1	Dienelactone hydrolase	61.02	60.43	44.1	47.82	53.34	152.12	131.26	213.36	165.58	3.1	3.93E-06

Solyc03g033540.2.1	Calcium-dependent pro	84.62	80.08	97.36	134.2	99.06	11.58	10.92	42.11	21.54	0.22	4.04E-06
Solyc02g091850.1.1	Unknown Protein (AH	0	0	3.11	0	0.78	25.58	19.35	26.19	23.71	30.49	4.04E-06
Solyc01g056850.2.1	CTP synthase (AHRD	23.59	44.97	44.35	19.98	33.22	2.81	2.93	2.73	2.82	0.08	4.07E-06
Solyc01g111930.2.1	Rop guanine nucleotid	15.75	16.46	15.49	20.99	17.17	2.31	4	3.17	3.16	0.18	4.09E-06
Solyc07g041190.2.1	PHD finger family prot	25.64	28.29	36.55	33.25	30.93	158.39	152.78	109.23	140.13	4.53	4.13E-06
Solyc03g026050.2.1	Terminal flower 1 (Fra	13.12	7.67	7.96	3.71	8.12	0	0	0	0	0	4.21E-06
Solyc09g061930.2.1	Receptor like kinase, R	8.2	11.65	6.84	7.21	8.47	1.78	1.3	2.78	1.95	0.23	4.24E-06
Solyc01g098740.2.1	Receptor protein kinas	2.28	2.3	3.23	2.37	2.54	41.14	47.07	43.6	43.94	17.26	4.28E-06
Solyc02g071880.2.1	Receptor like kinase, R	6.09	7.49	6.41	7.96	6.99	0.8	1.46	0.33	0.86	0.12	4.30E-06
Solyc02g037530.2.1	Auxin response factor	18.31	17.55	15.88	15.83	16.89	4.24	4.53	3.57	4.11	0.24	4.33E-06
Solyc01g089880.2.1	Unknown Protein (AH	32.62	37.51	12.97	17.63	25.18	0.59	0.6	0.73	0.64	0.03	4.42E-06
Solyc03g020060.2.1	Proteinase inhibitor II (	2817	2803.8	1144.17	909.72	1918.67	30.13	38.44	22.46	30.34	0.02	4.42E-06
Solyc08g078700.2.1	Heat shock protein 22 (	3.13	1.39	1.84	2.21	2.14	53.55	178.85	77.28	103.23	48.18	4.45E-06
Solyc09g018460.1.1	GRAS family transcrip	1.58	2.23	2.02	1.12	1.74	7.98	7.62	3.27	6.29	3.62	4.46E-06
Solyc04g074110.2.1	Acetyl xylan esterase A	32.99	24.77	10.82	17.98	21.64	1.43	0.79	0.64	0.95	0.04	4.46E-06
Solyc08g065360.2.1	Glutathione-regulated	25.07	37.48	18.81	25.82	26.8	6.31	4.3	4.37	4.99	0.19	4.46E-06
Solyc04g080460.2.1	Glucose transporter 8 (	17.75	19.89	13.11	14.95	16.43	2.74	3.97	3.36	3.36	0.2	4.55E-06
Solyc05g056030.2.1	Patatin-like protein 3 (	27.97	39.48	65.83	59.75	48.26	104.43	101.87	97.45	101.25	2.1	4.63E-06
Solyc02g081730.2.1	Sterol-4-alpha-carboxy	17.44	16.08	19.09	27.33	19.98	3.17	3.84	2.18	3.06	0.15	4.64E-06
Solyc07g005760.2.1	Hydroxycinnamoyl Co	31.23	26.43	67.9	57.16	45.68	140.23	128.1	89.64	119.32	2.61	4.70E-06
Solyc08g008550.2.1	Serine/threonine protei	5.55	5.85	5.68	6.28	5.84	10.02	20.58	13.5	14.7	2.52	4.74E-06
Solyc02g070790.2.1	3-oxoacyl-(Acyl-carrie	26.95	25.86	14.41	15.49	20.68	1.93	4.58	1.11	2.54	0.12	4.75E-06
Solyc05g008390.2.1	RING zinc finger-conta	19.82	21.43	32.26	13.84	21.84	49.65	63.36	49.8	54.27	2.49	4.76E-06
Solyc03g111710.2.1	Speckle-type POZ prot	60.63	40.56	50.61	42.07	48.47	5.39	14.29	16.09	11.92	0.25	4.80E-06
Solyc10g078390.1.1	Calcium-dependent pro	71.82	77.71	74.72	61.83	71.52	16.31	20.01	10.36	15.56	0.22	4.87E-06
Solyc06g082210.1.1	Light-dependent short	32.84	32.91	32.46	35.99	33.55	87	83.17	69.01	79.73	2.38	4.88E-06
Solyc08g005530.2.1	Unknown Protein (AH	4.4	9.3	3.89	5.44	5.76	19.99	21.5	29.47	23.65	4.11	4.88E-06
Solyc05g005810.2.1	Uncharacterized basic	9.99	6.85	4.64	5.79	6.82	0.79	0.4	0.49	0.56	0.08	4.97E-06
Solyc12g019320.1.1	Multidrug resistance pr	2.49	2.33	2.32	1.11	2.06	0	0	0	0	0	4.97E-06
Solyc02g090960.1.1	Rapid alkalinization fa	74.36	66.68	17.15	29.26	46.86	0	0	0.55	0.18	0	4.98E-06
Solyc05g005180.2.1	Naphthoate synthase (A	31.23	27.1	26.48	21.81	26.66	4.57	7.48	3.29	5.11	0.19	4.98E-06
Solyc02g084740.2.1	Cytochrome P450	15.06	14.71	16.35	8.29	13.6	3.23	2.5	3.04	2.92	0.21	4.99E-06
Solyc03g095900.2.1	1-aminocyclopropane-	1.42	3.63	3.77	3.26	3.02	5683.89	2728.77	4141.93	4184.86	1385.72	5.00E-06
Solyc01g108940.1.1	B3 domain-containing	6.01	6.3	4.59	4.75	5.41	0.43	0.14	0	0.19	0.04	5.00E-06
Solyc05g008350.2.1	ABC transporter G fam	34.52	39.39	35.03	31.24	35.05	4.95	11.19	5.89	7.34	0.21	5.02E-06
Solyc08g061980.2.1	Mechanosensitive ion (	14.7	17.78	16.46	11.73	15.17	1.62	3.17	0.62	1.8	0.12	5.07E-06
Solyc02g084850.2.1	Unknown Protein (AH	13.09	5.83	9.51	4.99	8.36	0	0	0.5	0.17	0.02	5.07E-06
Solyc12g099960.1.1	Unknown Protein (AH	45.38	40.39	49.08	33.28	42.03	4.41	8.9	4.17	5.83	0.14	5.11E-06
Solyc07g051860.1.1	Protein serine/threonin	0.61	0.72	0.24	0	0.39	31.46	24.99	33.44	29.96	76.34	5.12E-06
Solyc02g086740.1.1	50S ribosomal protein	69.5	60.28	101.14	104.29	83.8	6.84	21.56	15.76	14.72	0.18	5.21E-06
Solyc10g055630.1.1	Aquaporin (AHRD V1	43.21	46.72	23.57	44.69	39.55	5.82	11.55	4.15	7.17	0.18	5.25E-06
Solyc02g030170.2.1	FAD linked oxidase do	358.84	349.6	432.52	461.82	400.69	99.48	116.96	145.81	120.75	0.3	5.40E-06
Solyc02g091380.1.1	Unknown Protein (AH	14.94	13.29	5.87	11.44	11.38	0	0	1.24	0.41	0.04	5.40E-06
Solyc11g006500.1.1	Charged multivesicula	41.62	27.47	22.05	39.25	32.6	78.69	91.6	110.17	93.49	2.87	5.42E-06
Solyc02g078030.1.1	DNA-binding WRKY	49.23	40.25	66.63	109.58	66.42	218.24	171.86	307.32	232.47	3.5	5.61E-06
Solyc08g005090.1.1	Unknown Protein (AH	13.67	15.59	10.47	6.11	11.46	0.97	0.98	0.71	0.89	0.08	5.68E-06
Solyc09g092080.2.1	50S ribosomal protein	125.29	87.39	98.35	75.23	96.56	22.31	22.92	15.74	20.32	0.21	5.70E-06
Solyc12g095780.1.1	Hydroxyproline-rich gl	26.82	24.41	23.01	41.81	29.01	2.34	5.34	1.97	3.22	0.11	5.70E-06
Solyc10g045290.1.1	Kinase interacting fam	1	0.61	1.1	1.72	1.11	0.03	0	0	0.01	0.01	5.71E-06
Solyc02g083810.2.1	Ferredoxin--NADP red	342.13	288.82	249.86	252.28	283.27	66.95	99.55	63.19	76.56	0.27	5.72E-06
Solyc07g042000.2.1	Sodium/calcium excha	10.1	16.49	10.44	9	11.51	2.79	1.47	1.24	1.83	0.16	5.77E-06
Solyc02g088070.2.1	Dof zinc finger protein	6.13	7.99	8.24	6.45	7.2	1.04	0.93	1.13	1.03	0.14	5.78E-06
Solyc09g009010.2.1	Cellulose synthase-like	1.98	5.48	2.59	3.73	3.45	0	0	0	0	0	5.85E-06
Solyc05g041470.2.1	Transient receptor pote	5.68	5.58	5.08	5.26	5.4	0.32	0.16	0	0.16	0.03	5.93E-06
Solyc04g074430.1.1	Phi-1 protein (Fragmen	25.79	28.25	10.53	22.45	21.75	0	2.43	6.4	2.94	0.14	5.97E-06
Solyc06g005970.2.1	Beta-D-glucosidase (A	43.51	42.02	46.83	45.96	44.58	106.51	104.23	129.45	113.4	2.54	5.99E-06
Solyc05g050130.2.1	Acidic chitinase (AHR	33.77	31.06	46.75	16.24	31.95	3.46	2.94	2.46	2.95	0.09	6.00E-06
Solyc06g009810.2.1	Unknown Protein (AH	217.59	198.8	314.06	273.4	250.96	43.01	80.16	33.32	52.16	0.21	6.08E-06
Solyc09g065590.2.1	Os12g0604200 protein	1.89	3.73	1.44	3.63	2.67	0	0.11	0	0.04	0.01	6.10E-06
Solyc02g088220.2.1	Xenotropic and polytro	10.6	13.13	8.6	13.31	11.41	2.33	2.9	2.19	2.47	0.22	6.13E-06
Solyc08g082570.2.1	CccP Conserved Cys-c	93.87	76.5	76.94	85.32	83.16	17.66	23.65	12.72	18.01	0.22	6.16E-06
Solyc10g086180.1.1	Phenylalanine ammoni	10.84	8.91	10.82	8.78	9.84	67.15	65.91	54.81	62.62	6.37	6.18E-06
Solyc03g083090.2.1	Glycogen synthase (AH	48.3	56.88	34.92	44.62	46.18	118.72	111.45	156.97	129.05	2.79	6.19E-06
Solyc07g054460.2.1	H/ACA ribonucleoprot	15.41	14.92	21.08	17.49	17.22	2.19	2.48	1.93	2.2	0.13	6.27E-06
Solyc01g107360.2.1	Coatomer subunit beta	4.99	4.87	4.53	3.7	4.52	10.1	14.12	12.17	12.13	2.68	6.33E-06
Solyc11g008680.1.1	Acyl- (AHRD V1 ***-	17.08	19.52	15.41	19.42	17.86	45.45	45.84	41.55	44.28	2.48	6.34E-06
Solyc01g108330.2.1	Unknown Protein (AH	6.16	6.26	10.38	4.98	6.95	0	0	0.44	0.15	0.02	6.37E-06
Solyc02g031950.2.1	Pathogenesis-related pr	162.96	340.59	287.76	219.72	252.76	658.69	547.64	553.86	586.73	2.32	6.42E-06
Solyc07g056500.2.1	Glutathione transferase	36.72	54.46	42.97	49.4	45.89	129.03	135.49	98.08	120.87	2.63	6.46E-06
Solyc12g007090.1.1	Os08g0387500 protein	12.61	16.05	19.37	15.35	15.85	42.4	41.93	42.2	42.18	2.66	6.54E-06
Solyc11g011380.1.1	Glutamine synthetase (	842.01	734.67	844.31	910.03	832.75	1947.27	1942.69	1538.74	1809.57	2.17	6.59E-06
Solyc12g011010.1.1	Meiosis 5 (AHRD V1	3.72	4.03	11.06	8.46	6.82	0.13	0	0	0.04	0.01	6.61E-06
Solyc01g006370.2.1	Glucan synthase like 3	25.93	29.16	28.11	33.32	29.13	7.03	7.25	6.79	7.02	0.24	6.62E-06
Solyc04g045560.2.1	Squamosa promoter-bi	11.47	11.04	15.08	9.04	11.66	0.76	1.84	0.56	1.05	0.09	6.66E-06
Solyc06g053780.2.1	Os02g0200800 protein	14.72	14.72	9.01	11.83	12.57	0.59	3.7	0.72	1.67	0.13	6.66E-06
Solyc09g075120.2.1	Bifunctional polymyxi	346.86	276.61	425.83	297.37	336.67	79.12	109.9	83.24	90.75	0.27	6.66E-06
Solyc05g008450.2.1	Oxidoreductase FAD/N	167.21	131.57	140.77	178.32	154.47	302.2	422.79	448	391	2.53	6.67E-06
Solyc08g013670.2.1	Photosystem I reaction	130.33	138.91	70.24	44.38	95.97	2.27	4.59	1.6	2.82	0.03	6.67E-06
Solyc03g123460.2.1	Os10g0422600 protein	123	143.19	147.13	99.94	128.31	20.63	46.13	12.67	26.48	0.21	6.70E-06
Solyc07g052390.2.1	Glutamate-gated kaina	9.12	10.21	9.5	8.71	9.38	1.6	1.98	2.04	1.87	0.2	6.71E-06
Solyc06g076690.2.1	Acyl-coenzyme A thio	33.19	39.14	30.35	36.97	34.91	242.69	183.93	212.51	213.04	6.1	6.78E-06
Solyc02g069120.2.1	Zinc-finger protein ZP	13.66	10.54	12.28	10.49	11.74	31.43	31.28	31.64	31.45	2.68	6.88E-06

Solyc03g005390.2.1	Magnesium transporter	43.15	50.11	42.65	49.01	46.23	117.77	104.8	90.29	104.29	2.26	6.90E-06
Solyc04g078200.2.1	Gibberellin-regulated f	1565.31	2312.97	641.52	1064.65	1396.11	36.34	59.46	29.76	41.85	0.03	6.96E-06
Solyc12g096060.1.1	Mps one binder kinase	27.79	33.71	29.19	37.59	32.07	83.65	78.4	84.88	82.31	2.57	6.97E-06
Solyc06g065680.2.1	Cyclin-dependent prote	4.85	2.59	5.07	3.72	4.06	0.11	0.45	0	0.19	0.05	6.98E-06
Solyc01g104850.2.1	Serine carboxypeptidas	20.38	29.54	7.47	13.25	17.66	0.11	0.56	0.14	0.27	0.02	7.12E-06
Solyc01g097000.2.1	Beta-D-glucosidase (A	9.85	8.94	4.81	12.53	9.03	0.08	1.3	0.3	0.56	0.06	7.21E-06
Solyc07g049690.2.1	Cytochrome P450	65.83	83.7	109.65	124.81	96	217.78	212.89	252.73	227.8	2.37	7.23E-06
Solyc09g091420.2.1	Nucleoside-triphospha	19.25	17.98	14.15	15.63	16.75	1.28	2.57	0.94	1.6	0.1	7.23E-06
Solyc12g006510.1.1	Cycloartenol Synthase	4.02	5.83	2.06	1.85	3.44	0	0	0	0	0	7.31E-06
Solyc11g005380.1.1	GT-2 factor (Fragment	13.83	14.92	14.91	13.17	14.21	41.69	65.79	107.99	71.82	5.06	7.34E-06
Solyc09g072560.2.1	Legumin 11S-globulin	26.42	88.31	141.62	27.88	71.06	0.11	0	0.27	0.13	0	7.41E-06
Solyc12g042460.1.1	4-coumarate CoA ligas	2.76	4.81	2.13	2.04	2.93	0	0.1	0.12	0.07	0.02	7.41E-06
Solyc06g061030.2.1	Two-component respo	17.32	13.84	11.54	16.83	14.88	3.52	3.55	0.47	2.51	0.17	7.47E-06
Solyc07g006680.1.1	Hydroxycinnamoyl Co	37.28	53.14	30.41	18.75	34.89	3.06	3.8	1.16	2.67	0.08	7.53E-06
Solyc07g063750.2.1	Serine/threonine-prote	0.27	0.08	0.11	0	0.12	5.42	2.84	5.33	4.53	39.39	7.55E-06
Solyc02g068110.2.1	Enhancer of yellow 2 t	238.23	221.81	181.61	182.74	206.1	500.41	557.73	435.64	497.93	2.42	7.61E-06
Solyc08g007770.2.1	PGR5-like protein 1A,	143.51	148.69	124.58	87.86	126.16	17.65	33.16	17.86	22.89	0.18	7.71E-06
Solyc02g087850.1.1	Glutaredoxin family pr	15.24	15.1	12.97	16.66	14.99	1.4	2.18	2.34	1.97	0.13	7.74E-06
Solyc04g080540.2.1	ST225 (AHRD V1 ***	6.97	5.79	7.13	7.89	6.95	17.27	38.99	37.35	31.2	4.49	7.74E-06
Solyc01g095010.2.1	Diacylglycerol kinase	7.53	9.49	8.24	7.8	8.26	22.27	28.15	14.23	21.55	2.61	7.98E-06
Solyc07g044860.2.1	Oxygen-evolving enha	1072.6	832.65	777.12	777.87	865.06	201.05	315.49	203.02	239.85	0.28	7.99E-06
Solyc04g011650.2.1	Telomere repeat-bindi	5.14	4.66	5.01	6.15	5.24	36.78	24.06	27.06	29.3	5.59	7.99E-06
Solyc12g013700.1.1	Aluminum-induced pro	1.03	2.76	1.83	6.22	2.96	1866.91	989.14	1846.34	1567.46	529.55	8.02E-06
Solyc06g082570.1.1	Unknown Protein (AH	40.2	40.83	32.92	22.41	34.09	7.83	4.43	4.46	5.57	0.16	8.05E-06
Solyc06g053140.2.1	Senescence-associated	49.48	46.57	43.61	12.87	38.13	1.15	3.71	1.13	2	0.05	8.21E-06
Solyc01g088760.2.1	Cytochrome P450	6.33	7.7	10.36	5.48	7.47	29.41	25.67	21.53	25.54	3.42	8.23E-06
Solyc08g005960.1.1	Cortical cell-delineatin	7.22	17.57	6.25	8.85	9.97	0	0	0	0	0	8.29E-06
Solyc03g007030.2.1	CDGSH iron sulfur do	71.97	84.81	80.98	46.19	70.99	17.77	14.12	0.66	10.85	0.15	8.34E-06
Solyc07g042080.2.1	Inter-alpha-trypsin inhi	8.32	9.43	8.58	10.41	9.18	16.78	24.42	23.39	21.53	2.34	8.34E-06
Solyc05g005460.2.1	Nucleoredoxin 2 (AHR	20.29	24.51	33.3	26.94	26.26	7.14	5.45	9	7.2	0.27	8.39E-06
Solyc08g066220.2.1	Decarboxylase family	0	0	0	0.63	0.16	168.04	54	302.84	174.96	1110.86	8.55E-06
Solyc03g096460.2.1	Lipoxygenase homolog	13.19	10.16	3.79	18.16	11.32	0	0	0.35	0.12	0.01	8.56E-06
Solyc03g115920.2.1	Zinc finger protein-like	2.74	2.44	5.29	1.06	2.88	14.08	9.53	5.93	9.85	3.42	8.68E-06
Solyc06g083860.2.1	Light-dependent short	20.48	23.59	36.96	20.88	25.48	3.16	5.4	2.39	3.65	0.14	8.83E-06
Solyc09g066060.1.1	Homeobox leucine-zip	0.26	0	0	0	0.07	2.57	6.48	3.42	4.16	63.95	8.90E-06
Solyc07g051820.2.1	Cellulose synthase (AH	25.18	39.77	36.09	15.8	29.21	2.36	3.61	0.62	2.2	0.08	8.91E-06
Solyc03g031860.2.1	Phytoene synthase 1	118.05	155.53	202.21	99.19	143.75	6047.12	4401.48	6935.09	5794.56	40.31	8.94E-06
Solyc04g078550.2.1	WRKY transcription fa	15.93	19.18	13.05	17.76	16.48	33.01	45.05	64.92	47.66	2.89	8.94E-06
Solyc09g011690.2.1	Unknown Protein (AH	21.78	28.85	22.07	36.73	27.36	5.86	5.52	5.44	5.61	0.2	8.97E-06
Solyc04g007130.1.1	Genomic DNA chromo	5.67	9.79	7.95	11.54	8.74	18.34	33.82	33.45	28.54	3.27	9.05E-06
Solyc03g119280.2.1	Histidyl-tRNA syntheta	15.94	13.25	13.72	13.86	14.19	29.02	40.77	35.01	34.93	2.46	9.08E-06
Solyc05g007930.2.1	Beta-1 3-galactosyltran	5.76	6.8	3.95	7.7	6.05	0.51	1.11	1.77	1.13	0.19	9.16E-06
Solyc02g067160.2.1	Xenotropic and polytro	7.93	11.82	13.14	10.3	10.8	31.32	28.97	26.79	29.03	2.69	9.20E-06
Solyc09g018070.1.1	Multidrug resistance p	0	0	0	0	0	1.6	1.27	1.96	1.61	161	9.28E-06
Solyc08g075240.2.1	Glucose-methanol-cho	1.1	2.34	1.5	1.8	1.68	0	0.14	0.17	0.1	0.06	9.31E-06
Solyc04g081260.2.1	Filament-like plant pro	17.93	25.91	21.47	37.91	25.8	2.3	3.77	1.84	2.64	0.1	9.42E-06
Solyc02g031920.2.1	Pathogen-related prote	78.92	147.61	137.48	95.93	114.98	286.92	296.42	240.51	274.62	2.39	9.58E-06
Solyc07g017500.2.1	Lateral signaling target	18.46	17.88	21.14	18.43	18.98	89.38	58.85	79.63	75.95	4	9.58E-06
Solyc08g066360.2.1	Malic enzyme (AHRD	15.71	12.89	11.18	20.33	15.03	3.96	2.27	3.32	3.18	0.21	9.58E-06
Solyc01g097870.2.1	40S ribosomal protein	895.04	937.67	989.16	1099.59	980.37	218.76	264.78	247.76	243.77	0.25	9.63E-06
Solyc12g099810.1.1	Crt homolog 1 (AHRD	1.74	1.29	1.71	2.25	1.75	10.75	8.84	16.51	12.03	6.89	9.70E-06
Solyc11g010690.1.1	Nucleobase ascorbate	9.24	11.51	5.78	8.23	8.69	0.67	2.33	0.37	1.12	0.13	9.70E-06
Solyc01g104400.2.1	Blue copper protein (A	6.88	9.7	10.15	8.11	8.71	0	0	0	0	0	9.90E-06
Solyc09g098080.2.1	UDP-glucosyltransfera	0	0	0	0	0	3.58	1.35	2.06	2.33	233	1.00E-05
Solyc08g079860.1.1	Subtilisin-like proteas	0.97	1.57	1.56	1.25	1.34	0	0	0	0	0.01	1.00E-05
Solyc08g008630.2.1	Homology to unknown	88.78	80.54	57.29	33.3	64.98	4.36	9.59	4.87	6.27	0.1	1.00E-05
Solyc09g042750.2.1	Acyl-CoA thioesterase	35.07	38.48	31.2	31.74	34.12	78.15	84.75	66.22	76.37	2.24	1.01E-05
Solyc11g071610.1.1	Xanthine dehydrogena	2.56	2.77	1.24	2.7	2.32	0.32	0.2	0.25	0.26	0.11	1.02E-05
Solyc07g041070.2.1	Unknown Protein (AH	3.47	2.99	4.47	3.98	3.73	0.18	0.35	0	0.18	0.05	1.02E-05
Solyc01g107390.2.1	Auxin-responsive GH3	5.63	3.34	3.64	2.96	3.89	17.74	15.46	22.56	18.59	4.77	1.02E-05
Solyc10g083650.1.1	Peroxiredoxin ahpC/TS	90.17	74.1	58.12	80.28	75.67	655.64	914.35	663.4	744.46	9.84	1.02E-05
Solyc07g053430.2.1	Glucan endo-1 3-beta-	70.64	82.62	67.16	63.59	71	20.99	17.84	15.07	17.97	0.25	1.03E-05
Solyc02g082510.1.1	Amino acid transporter	9.91	13.37	11.69	13.58	12.14	43.04	29.02	49.8	40.62	3.35	1.03E-05
Solyc06g011450.1.1	Phosphatidylinositol bi	13.62	13.17	12.28	13.5	13.14	1.99	2.53	1.6	2.04	0.16	1.04E-05
Solyc01g005140.2.1	Cytochrome b561 (AH	0	0.79	1.05	0.42	0.57	9.6	9.2	6.49	8.43	14.92	1.05E-05
Solyc10g078740.1.1	Enoyl reductase (AHR	42.64	43.33	33.67	37.75	39.35	5.56	11.63	7.66	8.28	0.21	1.06E-05
Solyc01g067710.2.1	Sodium/hydrogen exch	5.89	7.42	8.82	5.72	6.96	16.35	19.98	23.25	19.86	2.85	1.06E-05
Solyc12g010980.1.1	Acyltransferase-like pr	3.11	3.03	2.79	1.26	2.55	0	0	0	0	0	1.06E-05
Solyc00g110870.2.1	Receptor like kinase, R	3.77	4.16	2.67	2.13	3.18	0.12	0.25	0.45	0.27	0.09	1.06E-05
Solyc10g007930.2.1	Cytochrome P450	9.28	10.81	7.37	3.34	7.7	0.27	0.41	0.17	0.28	0.04	1.06E-05
Solyc01g097860.2.1	Membrane protein (AH	52.5	60.75	52.52	44.56	52.58	9.59	16.93	8.61	11.71	0.22	1.06E-05
Solyc01g010250.2.1	6-phosphogluconolacto	18.78	14.07	18.07	13.98	16.23	39.33	38.46	31.39	36.39	2.24	1.07E-05
Solyc12g008980.1.1	Lycopene epsilon cycla	7.12	7.78	4.86	3.89	5.91	0.2	1.63	0	0.61	0.1	1.08E-05
Solyc07g052980.2.1	Xyloglucan endotransg	89.74	67.61	159.59	36.76	88.42	0.18	2.35	0.22	0.92	0.01	1.09E-05
Solyc05g045650.2.1	Glutamate-gated kaina	2.18	3.61	3.9	3.19	3.22	0.3	0.12	0.45	0.29	0.09	1.10E-05
Solyc07g015860.2.1	Peptide deformylase (A	59.09	46.46	42.56	52.03	50.04	10.93	15.28	6.6	10.94	0.22	1.10E-05
Solyc11g072100.1.1	1-AMINOCYCLOPRO	6.23	9.3	4.11	8.55	7.05	18.65	22.25	20.01	20.3	2.88	1.10E-05
Solyc02g078820.2.1	Lysine ketoglutarate re	26	26.18	35.47	29.65	29.33	68.79	71.1	75.8	71.9	2.45	1.10E-05
Solyc03g025390.2.1	Retinol dehydrogenase	75.64	84.14	98.16	115.9	93.46	17.82	27.22	11.05	18.7	0.2	1.11E-05
Solyc06g019170.2.1	Gamma-glutamyl phos	64.41	74.66	74.47	62.38	68.98	211.71	167.56	172.83	184.03	2.67	1.12E-05
Solyc02g079220.2.1	Solute carrier family 2	3.65	5.38	3.71	7.66	5.1	0.61	0.21	0	0.27	0.05	1.13E-05
Solyc04g007650.2.1	Phosphatidylinositol-4	22.64	30.12	39.79	22.55	28.78	6.76	5.26	3.6	5.21	0.18	1.14E-05

Solyc02g092980.2.1	Cyclin D-like protein (	17.04	21.85	19.46	17.11	18.87	2.96	2.24	2.36	2.52	0.13	1.14E-05
Solyc07g052790.1.1	Nbs-lrr, resistance prot	1.85	1.78	4.02	3.56	2.8	0	0	0	0	0	1.14E-05
Solyc03g098700.1.1	Cysteine protease inhib	26.99	26.69	5.66	12.72	18.02	0	0.24	0	0.08	0	1.15E-05
Solyc03g019890.2.1	Beta-galactosidase (AH	2.35	2.83	4.56	4.61	3.59	0.55	0.06	0.45	0.35	0.1	1.15E-05
Solyc10g019270.1.1	ABC transporter C fam	0	0	0.06	0	0.01	3.45	1.37	3.7	2.84	189.33	1.15E-05
Solyc10g080610.1.1	Kelch-like protein 14 (	178.03	173.76	242.52	219.09	203.35	25.95	44.88	23.73	31.52	0.16	1.16E-05
Solyc03g020070.2.1	Proteinase inhibitor II	1014.72	997.4	409.59	285.2	676.73	11.99	11.09	6.55	9.88	0.01	1.17E-05
Solyc02g079320.2.1	Unknown Protein (AH	24.61	25.07	18.51	17.65	21.46	2.88	3.78	2.48	3.05	0.14	1.17E-05
Solyc05g032700.2.1	Unknown Protein (AH	35.13	21.35	26.28	10.91	23.42	0	2.09	1.7	1.26	0.05	1.20E-05
Solyc01g005550.2.1	Carboxyl-terminal pep	60.41	81.13	74.73	46.11	65.59	16.23	18.88	11.81	15.64	0.24	1.20E-05
Solyc02g070980.1.1	Chlorophyll a/b bindin	17.11	13.02	13.75	6.67	12.64	1.8	1.21	0.74	1.25	0.1	1.21E-05
Solyc10g081750.1.1	Mal d 1-associated pro	36.2	36.43	39.91	39.24	37.95	70.52	107.52	111.15	96.4	2.54	1.21E-05
Solyc02g063430.2.1	BHLH transcription fa	4.51	7.11	1.69	5.51	4.71	0.17	0	0	0.06	0.01	1.21E-05
Solyc06g071920.2.1	Glyceraldehyde-3-phos	253.54	237.46	214.84	286.85	248.17	572.09	568.91	629.64	590.21	2.38	1.22E-05
Solyc01g106340.2.1	Glutamyl-tRNA(Gln) a	13.28	16.34	8.15	6.87	11.16	1.05	0.7	0.43	0.73	0.07	1.22E-05
Solyc07g056360.1.1	Unknown Protein (AH	32.67	26.84	34.1	3.55	24.29	0	0	0	0	0	1.22E-05
Solyc08g068290.2.1	Os01g0786800 protein	42.74	54.9	61.51	40.5	49.91	107.71	116.53	91.98	105.41	2.11	1.22E-05
Solyc10g047240.1.1	Annexin (AHRD V1 *	70.51	70.82	23.59	28.87	48.45	1.5	1.01	0.82	1.11	0.02	1.23E-05
Solyc11g070110.1.1	Epsin (AHRD V1 *-*-	8.32	9.77	5.55	5.97	7.4	22.02	21.52	16.16	19.9	2.69	1.23E-05
Solyc02g069470.2.1	Inter-alpha-trypsin inh	9.49	9.85	13.94	12.5	11.45	27.21	29.12	31.4	29.24	2.56	1.23E-05
Solyc03g045070.1.1	Ammonium transporte	1.86	3.06	2.36	1.01	2.07	0	0	0	0	0	1.24E-05
Solyc10g009570.2.1	Rhodanese-related sul	38.73	36.81	21.23	23.15	29.98	3.94	6.41	6.7	5.68	0.19	1.25E-05
Solyc06g074890.1.1	Trigger factor (AHRD	28.96	18.07	14.44	20.69	20.54	0.72	5.35	1.78	2.62	0.13	1.25E-05
Solyc01g081480.2.1	Genomic DNA chromo	15.93	15.63	14.22	10.4	14.04	2.97	2.5	0.81	2.09	0.15	1.27E-05
Solyc03g096800.2.1	At5g24290-like protei	5.16	5.15	4.22	4.33	4.71	0.46	0.69	0.28	0.48	0.1	1.28E-05
Solyc04g079690.2.1	Receptor-like protein k	4.31	4.16	4.44	2.86	3.94	0.3	0.52	0.36	0.39	0.1	1.28E-05
Solyc07g006130.1.1	Eukaryotic peptide cha	12.02	10.41	9.78	8.27	10.12	67.35	48.99	53.55	56.63	5.6	1.29E-05
Solyc07g065840.2.1	Heat shock protein 90	2456.01	2155.81	3068.91	2672.3	2588.26	401.72	712.52	611.98	575.41	0.22	1.29E-05
Solyc07g056350.2.1	Mitogen activated prot	94.18	89.49	119.5	100.82	101	18.85	31.55	16.95	22.45	0.22	1.30E-05
Solyc07g052680.1.1	Unknown Protein (AH	12.59	9.27	8.19	9.82	9.97	0.35	0.71	0.43	0.5	0.05	1.31E-05
Solyc02g066950.2.1	Alpha amylase 2 (IC) (	14.27	11.6	14.43	13.08	13.34	2.12	3.56	1.59	2.42	0.18	1.31E-05
Solyc05g053470.2.1	chaperonin (AHRD V1	58.73	44.6	45.7	38.85	46.97	80.23	120.06	121.84	107.38	2.29	1.31E-05
Solyc03g121090.2.1	Cold induced protein-l	19.98	45.9	41.11	42.06	37.26	7.51	6.44	4.61	6.19	0.17	1.31E-05
Solyc01g099990.2.1	F-box protein PP2-B1	0.23	0.81	0.8	0.64	0.62	5.49	5.73	5.63	5.62	9.06	1.32E-05
Solyc07g056330.2.1	Ribose-phosphate pyro	6.78	4.94	8.24	6.69	6.66	0.17	0.84	0	0.34	0.05	1.33E-05
Solyc07g065540.1.1	Fasciclin-like arabinog	28.98	27.79	36.65	49.15	35.64	2.2	9.53	2.07	4.6	0.13	1.34E-05
Solyc07g042500.2.1	Abhydrolase domain c	6.83	8.43	9.08	5.06	7.35	18.31	14.82	19.59	17.57	2.39	1.41E-05
Solyc10g083520.1.1	cDNA clone J033118E	103.01	151.08	88.76	96.04	109.72	32.1	32.38	24.51	29.66	0.27	1.45E-05
Solyc08g066680.2.1	Exostosin family prote	15.98	17.67	18.38	17.14	17.29	45.16	32.85	41.73	39.91	2.31	1.48E-05
Solyc03g097900.2.1	40S ribosomal protein	989.82	728.32	1049.25	1141.16	977.14	215.84	253.6	246.93	238.79	0.24	1.48E-05
Solyc03g113330.2.1	Nodulin-like protein (	12.58	11.2	11.1	7.65	10.63	23.75	29.42	17.16	23.44	2.2	1.48E-05
Solyc06g072700.2.1	Metal ion binding prot	57.75	50.2	37	38.14	45.77	6.03	10.44	4.75	7.07	0.15	1.48E-05
Solyc04g074050.2.1	Receptor like kinase, F	4.93	4.17	2.19	5.83	4.28	0.26	0.33	0.96	0.52	0.12	1.51E-05
Solyc02g069510.1.1	Light-dependent short	4.35	6.45	4.7	5.64	5.29	0	0	0.72	0.24	0.05	1.54E-05
Solyc04g054520.2.1	Peptidyl-prolyl cis-tran	18.22	19.85	15.94	13.78	16.95	2.03	4.35	1.87	2.75	0.16	1.55E-05
Solyc06g069000.2.1	Uncharacterized GPI-a	4.01	3.57	2.13	5.96	3.92	0	0.16	0	0.05	0.01	1.55E-05
Solyc07g017490.2.1	Red family protein (AH	41.19	40.75	40.24	47.41	42.4	94.01	90.88	107.3	97.4	2.3	1.59E-05
Solyc03g113430.2.1	Peptide transporter (A	3.6	5.11	3.72	3.34	3.94	25.65	31.19	21.9	26.25	6.66	1.60E-05
Solyc02g088340.2.1	WRKY transcription fa	13.31	13.88	8.27	9.11	11.14	29.71	29.85	19.32	26.29	2.36	1.60E-05
Solyc03g005580.2.1	Legumin 11S-globulin	26.04	82.01	170.47	40.99	79.88	0.11	0	0.13	0.08	0	1.60E-05
Solyc04g050390.1.1	60S ribosomal protein	48.23	56.28	76.22	84.31	66.26	4.04	8.16	9.94	7.38	0.11	1.62E-05
Solyc01g010310.2.1	Glucan endo-1 3-beta-	86.46	67.06	82.75	67.29	75.89	14.31	20.37	9.65	14.78	0.19	1.64E-05
Solyc01g006320.2.1	Non-race specific dise	26.52	13.28	9.39	25.79	18.74	0.8	0.27	0.33	0.47	0.02	1.66E-05
Solyc11g012510.1.1	GRAS family transcrip	40.09	34.17	35.26	61.18	42.68	4.91	8.42	4.1	5.81	0.14	1.66E-05
Solyc07g064440.2.1	Genomic DNA chromo	6.62	10.23	11.94	6.54	8.83	25.84	18.94	27.53	24.1	2.73	1.67E-05
Solyc03g095880.1.1	Unknown Protein (AH	0	0	0	0	0	129.59	87.49	314.57	177.22	17721.67	1.71E-05
Solyc11g010200.1.1	14-3-3 protein beta/alp	250.2	231.38	296.05	301.38	269.75	51.32	85.22	43.17	59.9	0.22	1.72E-05
Solyc02g092110.2.1	Phytosulfokines 3 (AH	2.38	1.41	2.81	1.12	1.93	16.05	44.04	18.15	26.08	13.51	1.73E-05
Solyc06g065190.1.1	TCP family transcripti	8.3	9.01	11.94	18.03	11.82	0.98	0.99	0.4	0.79	0.07	1.77E-05
Solyc08g065320.2.1	Transmembrane protei	10.54	16.84	16.27	5.74	12.35	44.12	39	39.72	40.95	3.32	1.77E-05
Solyc01g099660.2.1	Heat shock protein (AI	0	0.26	0.12	0	0.1	2.47	7.3	5.57	5.11	53.82	1.77E-05
Solyc04g082410.2.1	BTB/POZ domain-con	18.79	31.99	34.22	20.51	26.38	53.46	70.73	66.7	63.63	2.41	1.78E-05
Solyc02g076870.2.1	Potential lipid particle	24.99	19.01	22.46	20.14	21.65	83.12	74.36	95.92	84.47	3.9	1.79E-05
Solyc02g089350.2.1	Gibberellin regulated p	1244.69	789.26	699.8	1383.22	1029.24	78.3	231.21	74.75	128.09	0.12	1.79E-05
Solyc09g009770.2.1	Calmodulin binding pr	25.84	24.78	37.14	39.21	31.74	9.83	3.78	5.73	6.45	0.2	1.79E-05
Solyc08g069230.2.1	Unknown Protein (AH	24.39	32.56	13.08	16.47	21.62	1.34	2.71	0.55	1.53	0.07	1.80E-05
Solyc01g098800.2.1	Sodium/calcium excha	7.36	14.26	8.12	20.48	12.55	28.76	26.59	59.71	38.35	3.05	1.81E-05
Solyc04g076780.2.1	Oligopeptide transport	17.89	21.75	12.77	20.12	18.13	2.47	6.04	1.21	3.24	0.18	1.82E-05
Solyc10g006230.2.1	Chlorophyll a-b bindin	989.32	790.36	576.99	551.98	727.16	189.99	152.15	166.48	169.54	0.23	1.84E-05
Solyc03g005330.1.1	CBL-interacting protei	1.23	0.68	2	0.65	1.14	6.09	5.51	6.11	5.9	5.18	1.84E-05
Solyc08g061100.2.1	Cellulose synthase (AH	235.06	249.82	287.63	290.16	265.67	50.31	79.13	60.18	63.21	0.24	1.84E-05
Solyc12g017230.1.1	Os04g0431000 protein	6.8	7.03	7.26	7.15	7.06	17.82	17.89	14.32	16.68	2.36	1.85E-05
Solyc04g077920.2.1	Interferon-related deve	36.8	33.82	38.47	26.45	33.88	106.36	89.23	105.57	100.39	2.96	1.85E-05
Solyc02g076680.2.1	Unknown Protein (AH	16.97	19.15	20.46	23.06	19.91	98.1	74.77	95.31	89.39	4.49	1.86E-05
Solyc06g062580.2.1	Beta-galactosidase (AI	5.8	6.55	4.53	12.75	7.41	0.19	0.32	0.47	0.33	0.04	1.86E-05
Solyc02g079200.1.1	Armadillo/beta-cateni	6.06	8.45	6.74	9.38	7.66	1.66	1.49	0.23	1.13	0.15	1.89E-05
Solyc12g010920.1.1	Oleosin (AHRD V1 **	5.34	11.88	15.75	5.93	9.72	0	0	0.38	0.13	0.01	1.91E-05
Solyc11g068360.1.1	Cc-nbs-lrr, resistance p	1.99	2.52	2.77	3.5	2.69	0.34	0.34	0.12	0.27	0.1	1.91E-05
Solyc06g050700.2.1	Early response to dehy	155.69	252.08	233.5	190.36	207.91	31.27	78.13	26.19	45.2	0.22	1.94E-05
Solyc11g012150.1.1	Transcription initiatio	35.16	27.31	34.8	24.4	30.42	71.49	72.21	63.39	69.03	2.27	1.95E-05
Solyc06g074090.2.1	Sterol reductase (AHR	33.67	39.95	44.91	62.43	45.24	8.21	11.25	5.72	8.39	0.19	1.95E-05
Solyc02g062320.2.1	BURP domain-contain	2.23	4.68	6.92	2.58	4.1	0	0	0	0	0	1.95E-05

Solyc12g056630.1.1	Holocarboxylase synth	23.24	22.51	42.34	24.18	28.07	72.08	65.73	69.21	69.01	2.46	1.96E-05
Solyc11g043070.1.1	Cc-nbs-lrr, resistance p	3.39	5.89	2.4	4.32	4	0.27	0.14	0.34	0.25	0.06	1.97E-05
Solyc12g013710.1.1	Protochlorophyllide re	4.48	3.54	3.52	4.45	4	0.4	0.14	0.16	0.23	0.06	1.98E-05
Solyc02g087110.2.1	Alpha-dioxygenase (A	5.21	4.08	1.23	0.74	2.81	235.34	267.85	182.79	228.66	81.23	2.00E-05
Solyc01g102960.2.1	class IV heat shock pro	9.03	9.27	2.46	15.72	9.12	1051.94	1115.33	733.06	966.78	106.01	2.02E-05
Solyc11g011760.1.1	Genomic DNA chromo	237.14	280.35	237	213.53	242	57.14	75.55	50.73	61.14	0.25	2.02E-05
Solyc09g014400.2.1	Alpha-amylase (AHRD	18.83	26.17	26.07	14.97	21.51	4.15	6.21	2.01	4.12	0.19	2.03E-05
Solyc10g008200.2.1	Tyrosine aminotransfe	2.83	3.36	3.89	6.44	4.13	0	0.26	0.16	0.14	0.03	2.03E-05
Solyc06g053830.2.1	Auxin responsive prote	5.42	3.75	5.69	2.13	4.25	0	0	0.3	0.1	0.02	2.03E-05
Solyc09g007860.2.1	Plant synaptotagmin (A	3.38	5.27	6.98	5.98	5.4	11.03	15.16	7.2	11.13	2.06	2.03E-05
Solyc02g083310.2.1	Wound responsive prot	23.01	28.27	41.32	18.72	27.83	2.14	6.64	1.62	3.47	0.12	2.06E-05
Solyc07g049200.2.1	Unknown Protein (AH	29.49	22.33	24.72	24.15	25.17	61.45	66.41	62.4	63.42	2.52	2.07E-05
Solyc12g005750.1.1	Zinc finger protein CO	21.59	22.42	6.37	9.33	14.93	0	0	0	0	0	2.07E-05
Solyc10g008630.2.1	NADH dehydrogenase	24.43	25.29	25.18	26.96	25.47	50.99	66.87	56.42	58.09	2.28	2.09E-05
Solyc04g009800.2.1	Calcium-dependent pro	27.43	29.16	25.14	55.96	34.42	54.4	82.02	126.8	87.74	2.55	2.10E-05
Solyc10g050440.1.1	Unknown Protein (AH	211.53	174.28	150.72	127.38	165.98	27.92	52.05	12.47	30.81	0.19	2.10E-05
Solyc02g078240.2.1	2,3-bisphosphoglycera	13.56	13.07	10.4	7.35	11.1	1.64	2.03	0	1.22	0.11	2.11E-05
Solyc06g053240.2.1	AP2 transcription facto	41.23	31.19	9.73	34.99	29.29	0	0	0	0	0	2.12E-05
Solyc02g064720.2.1	Phototropic-responsive	11.76	15.6	13.36	6.31	11.76	1.14	2.66	0.54	1.45	0.12	2.13E-05
Solyc04g079720.2.1	Transferase family pro	113.48	99.54	131.59	109.54	113.54	24.66	42.84	22.62	30.04	0.26	2.14E-05
Solyc01g097470.2.1	Neurogenic locus notc	27.94	43.59	9.52	26.08	26.78	0.47	0.94	1.72	1.04	0.04	2.15E-05
Solyc12g099760.1.1	Homeobox protein (AH	17.73	17.13	14.49	14.51	15.96	39.69	36.32	42.1	39.37	2.47	2.15E-05
Solyc02g070970.1.1	Chlorophyll a/b bindin	23.07	16.11	12	8.42	14.9	1.2	0.2	0	0.47	0.03	2.15E-05
Solyc01g109370.2.1	1-acylglycerol-3-phosp	5.03	10.72	6.36	7.88	7.5	0.29	1.32	0.36	0.66	0.09	2.16E-05
Solyc10g055240.1.1	Phosphatidylinositol-sp	3.31	5.34	3.42	1.17	3.31	0	0	0.21	0.07	0.02	2.16E-05
Solyc07g063860.2.1	POT family domain co	29.01	38.91	22.97	26.42	29.33	2.89	7.77	1.18	3.95	0.13	2.17E-05
Solyc11g064850.1.1	Os04g0226400 protein	19.4	35.64	28.3	26.2	27.39	94.77	158.4	73.36	108.84	3.97	2.17E-05
Solyc10g083740.1.1	60S ribosomal protein	119.07	113.13	88.27	67.71	97.04	18.85	32.78	12.78	21.47	0.22	2.17E-05
Solyc01g099590.2.1	Glutathione-S-transfera	173.58	218.68	111.04	84.19	146.87	322.4	358.62	337.31	339.44	2.31	2.19E-05
Solyc01g106080.2.1	Methylmalonate-semia	34.33	38.52	33.71	37.99	36.14	81.43	87.13	78.26	82.27	2.28	2.20E-05
Solyc03g119690.1.1	Aspartic proteinase ne	110.86	108.98	201.2	144.77	141.45	27.89	28.74	41.61	32.75	0.23	2.21E-05
Solyc12g089130.1.1	CRAL/TRIO domain c	7.05	7.03	10.98	10.37	8.86	19.13	26.65	18.19	21.32	2.41	2.23E-05
Solyc04g054980.2.1	Lipoxygenase homolog	1595.17	1365.33	1375.03	1644.63	1495.04	310.2	597.31	208.92	372.14	0.25	2.23E-05
Solyc05g041430.2.1	Unknown Protein (AH	57.82	65.97	51.16	38.79	53.43	17.67	9.66	2.21	9.85	0.18	2.24E-05
Solyc06g060940.1.1	GATA transcription fa	4.04	5.01	4.77	5.54	4.84	13.25	12.67	22.11	16.01	3.31	2.26E-05
Solyc08g078040.2.1	Monooxygenase FAD-	5.15	2.84	4.63	1.73	3.59	18.03	22.58	24.59	21.73	6.06	2.28E-05
Solyc02g092610.2.1	Transposon protein Mu	8.33	23.37	20.4	11.78	15.97	45.02	43.84	64.85	51.24	3.21	2.32E-05
Solyc03g114500.2.1	Enolase (AHRD V1 **	51.81	45.27	41.54	58.78	49.35	14.39	17.59	17.41	16.46	0.33	2.33E-05
Solyc03g033640.2.1	DUF726 domain-conta	13.95	11.71	16.58	12.4	13.66	34.3	27.93	39.37	33.87	2.48	2.34E-05
Solyc02g092150.2.1	ACT domain-containin	3.66	3.47	2.16	1.72	2.75	59.01	67.37	49.14	58.51	21.26	2.34E-05
Solyc04g079310.2.1	RNA Binding Protein 4	51.81	48.84	35.3	45.42	45.34	5.31	15.7	7.68	9.56	0.21	2.34E-05
Solyc08g082060.2.1	Leaf senescence protei	13.01	17.98	10.11	14.29	13.85	1.11	4.24	0.91	2.09	0.15	2.34E-05
Solyc03g117870.2.1	4-coumarate CoA ligas	28.88	32.58	47.75	47.85	39.27	217.88	157.43	234.82	203.38	5.18	2.34E-05
Solyc11g010850.1.1	1-deoxy-D-xylulose 5-	3.69	7.06	15.57	9.92	9.06	0	0.15	0	0.05	0.01	2.35E-05
Solyc11g044940.1.1	Pto-like, Serine/threon	0.89	1.66	0.7	1.69	1.23	0	0.06	0	0.02	0.02	2.36E-05
Solyc07g066000.2.1	Amino acid permease (	9.02	13.55	5.99	11.17	9.93	119.35	68.92	59.89	82.72	8.33	2.38E-05
Solyc00g053440.2.1	Transmembrane BAX	18.07	20.94	15.86	35.07	22.48	40.06	65.74	62.15	55.98	2.49	2.39E-05
Solyc12g056930.1.1	Sulfate transporter 2 (A	5.72	4.74	2.01	3.69	4.04	0.41	0.33	0.3	0.35	0.09	2.39E-05
Solyc05g006550.2.1	Microtubule-associated	1.49	2.03	1.05	3.37	1.98	0	0	0	0	0.01	2.39E-05
Solyc11g012880.1.1	Solute carrier family 3	4.1	2.95	2.76	3.04	3.21	0	0.16	0.19	0.12	0.04	2.40E-05
Solyc11g008760.1.1	Pentatricopeptide repe	1.05	1.07	0.47	1.14	0.93	3.96	3.08	5.35	4.13	4.43	2.40E-05
Solyc03g013160.2.1	Amino acid transporter	13.13	17.6	11.67	3.93	11.58	0.37	0.5	0	0.29	0.03	2.42E-05
Solyc03g120930.1.1	Avr9/Cf-9 rapidly elici	0.85	2.26	2.49	5.98	2.9	8.88	23.43	8.4	13.57	4.69	2.43E-05
Solyc01g090550.2.1	DNAJ chaperone (AHI	84.6	81.09	84.79	76.19	81.67	169.93	177.72	183.81	177.15	2.17	2.44E-05
Solyc08g079710.2.1	Kinesin (AHRD V1 *-*	7.13	6.73	5.23	5.35	6.11	1.12	1.36	0.79	1.09	0.18	2.47E-05
Solyc12g089000.1.1	Ariadne-like ubiquitin	20.72	21.61	31.83	16.3	22.61	53.5	51.36	51.85	52.24	2.31	2.47E-05
Solyc02g062500.2.1	2-oxoglutarate-depend	122.31	156.81	123.03	97.95	125.02	2491.13	1490.35	1835.74	1939.07	15.51	2.48E-05
Solyc12g011040.1.1	Lipoxygenase (AHRD	61.46	59.24	168.65	48.28	84.41	0.12	1.08	0.44	0.55	0.01	2.49E-05
Solyc01g081070.2.1	Protein transport prote	16.73	16.81	16.79	20.25	17.64	42.01	35.21	37.75	38.32	2.17	2.52E-05
Solyc01g111110.2.1	Unknown Protein (AH	165.71	264.91	139.91	78.87	162.35	3.81	14.08	3.12	7	0.04	2.52E-05
Solyc06g074290.1.1	Unknown Protein (AH	138.54	147.76	110.26	80.87	119.36	406.85	293.87	311.04	337.25	2.83	2.53E-05
Solyc08g077510.2.1	Poly(U)-specific endor	65.19	58.16	49.13	51.18	55.91	132.3	130.9	92.11	118.44	2.12	2.53E-05
Solyc05g025500.2.1	Glucan endo-1 3-beta-g	9.56	10.82	6.45	10.24	9.27	1.66	1.45	1.76	1.62	0.18	2.53E-05
Solyc07g062970.2.1	Serine/threonine phosp	65.74	61.83	76.46	48.8	63.21	416.59	323.31	394.05	377.98	5.98	2.53E-05
Solyc09g008460.2.1	Ras-related protein Ra	38.61	47.35	27.26	32.68	36.48	4.42	9.41	5.13	6.32	0.17	2.54E-05
Solyc08g081170.2.1	UDP-N-acetylmuramo	5.98	5.4	6.55	5.44	5.84	10.42	16.78	17.39	14.86	2.54	2.54E-05
Solyc08g067100.2.1	Aspartic proteinase ne	16.16	25.73	27.75	22.68	23.08	3.93	6.85	3.52	4.77	0.21	2.57E-05
Solyc05g052460.2.1	Cation-independent ma	7.2	8.68	5.76	5.59	6.81	18.58	29.91	20.98	23.16	3.4	2.57E-05
Solyc02g091890.1.1	MRNA complete cds c	48.53	50.95	41.16	45.76	46.6	8.69	17.74	6.39	10.94	0.23	2.58E-05
Solyc06g082590.1.1	Ethylene responsive tra	18.28	11.55	29.07	17.99	19.22	0.64	1.94	1.05	1.21	0.06	2.60E-05
Solyc05g015950.2.1	Glucose-6-phosphate 1	10.73	9.55	11.46	11.5	10.81	26.38	22.83	27.58	25.6	2.37	2.60E-05
Solyc08g067960.2.1	CHY zinc finger famil	94.09	120.63	117.5	67	99.81	182.1	217.96	280.82	226.96	2.27	2.61E-05
Solyc03g031750.1.1	Vacuolar protein sortin	16.34	19.85	18.53	43.99	24.68	84.52	53.76	145.93	94.74	3.84	2.67E-05
Solyc06g050870.2.1	Hypoxia induced prote	1.76	3.13	1.04	1.24	1.79	16.34	20.79	4.37	13.83	7.72	2.68E-05
Solyc01g088590.2.1	Invertase inhibitor (AH	6	11.93	5.41	2.5	6.46	179.28	133.1	145.32	152.57	23.62	2.69E-05
Solyc09g092460.2.1	Receptor-like kinase (A	12.08	10.19	9.94	14.62	11.71	23.37	41.32	26.19	30.29	2.59	2.72E-05
Solyc07g061790.2.1	Heme-binding protein	213.4	261.92	186.8	253.98	229.03	85.74	72.46	56.66	71.62	0.31	2.72E-05
Solyc03g120720.2.1	Protein disulfide isome	40.88	40.63	42.45	39.65	40.9	64.37	107.32	89.05	86.91	2.12	2.74E-05
Solyc05g052700.2.1	TPR domain protein (A	0.88	3.12	1.03	0.62	1.41	5.78	6.18	4.49	5.48	3.88	2.75E-05
Solyc05g006980.2.1	Homeobox-leucine zip	28.99	29.46	32.32	29.71	30.12	5.72	8.56	3.4	5.89	0.2	2.76E-05
Solyc12g049350.1.1	Myb 12 transcription f	11.67	19.57	21.97	15.55	17.19	4.71	4.02	1.11	3.28	0.19	2.78E-05
Solyc02g084610.1.1	LRR receptor-like seri	5.07	4.8	3.37	5.16	4.6	0.26	0.39	0	0.22	0.05	2.78E-05

Solyc06g076670.2.1	Nuclear SR-like RNA	55.1	33.38	28.53	24.68	35.42	67.77	129.59	190.07	129.14	3.65	2.78E-05
Solyc12g094550.1.1	AT1G76250-like prote	4.26	3.79	4.85	5.2	4.53	10.92	14.01	11.96	12.3	2.72	2.79E-05
Solyc02g083110.1.1	UNE1-like protein (AH	45.23	40.13	49.77	62.21	49.34	9.05	15.55	6.04	10.21	0.21	2.79E-05
Solyc05g006050.2.1	Cyclin D1 (AHRD V1	5.07	13.55	17.96	9.11	11.42	0.79	0.16	0.19	0.38	0.03	2.79E-05
Solyc01g099160.2.1	Lipoxygenase (AHRD	1.91	2.59	5.5	2.16	3.04	75.35	48.71	51.52	58.53	19.25	2.79E-05
Solyc09g010630.2.1	heat shock protein (AH	3105.72	2218.84	3408.2	2789.86	2880.65	409.25	843.91	559.32	604.16	0.21	2.80E-05
Solyc05g054120.1.1	Cysteine proteinase inh	14.72	14.9	11.38	15.07	14.02	1.64	0.83	1.51	1.33	0.09	2.82E-05
Solyc06g076570.1.1	class I heat shock prote	191.1	120.84	89.38	142.68	136	1212.11	1639.09	1190.09	1347.1	9.91	2.83E-05
Solyc06g083880.2.1	Unknown Protein (AH	9.05	9.21	8.65	10.98	9.47	0	1.41	0	0.47	0.05	2.84E-05
Solyc06g066030.2.1	Os02g0200800 protein	154.12	139.38	167.44	196.63	164.39	31.69	48.11	31.64	37.15	0.23	2.85E-05
Solyc07g056490.2.1	Glutathione transferase	343.35	395.79	227.13	261.71	307	82.13	102.87	57.74	80.91	0.26	2.85E-05
Solyc03g119150.2.1	Peptidyl-prolyl cis-tran	30.33	24.52	26.24	25.03	26.53	1.63	10.3	3.26	5.06	0.19	2.87E-05
Solyc07g047800.2.1	Short-chain dehydroge	351.85	339.09	368.17	257.23	329.09	3574.59	2277.95	3412.43	3088.32	9.38	2.87E-05
Solyc12g005870.1.1	NEDD8 activating enz	16.99	15	16	15.09	15.77	31.06	39.38	36.99	35.81	2.27	2.91E-05
Solyc03g117480.2.1	Pyrophosphate-energiz	43.64	41.89	58.98	58.08	50.65	102.78	175.13	244.36	174.09	3.44	2.92E-05
Solyc06g076540.1.1	class I heat shock prote	5.53	3.03	3.51	8.43	5.12	47.8	102.31	124.2	91.44	17.84	2.92E-05
Solyc04g079990.2.1	DNA-3-methyladenine	4.93	6.41	5.37	5.09	5.45	0.46	0.46	0.38	0.43	0.08	2.92E-05
Solyc10g083580.1.1	Phytosulfokines 5 (AH	70.23	99.12	29.29	27.09	56.43	735.86	637.58	268.97	547.47	9.7	2.93E-05
Solyc01g099150.2.1	Lipoxygenase (AHRD	0.31	0.54	1.89	0.97	0.93	58.55	32.08	42.04	44.22	47.68	2.97E-05
Solyc06g076770.2.1	Myb family transcripti	49.26	60.43	67.72	45.45	55.72	16.44	16.93	15.73	16.37	0.29	3.01E-05
Solyc07g062860.2.1	Genomic DNA chromo	26.55	32.94	33.22	28.45	30.29	67.92	57.04	88.64	71.2	2.35	3.02E-05
Solyc08g065150.1.1	Genomic DNA chromo	30.88	27.65	48.19	30.65	34.34	6.04	10.78	1.9	6.24	0.18	3.05E-05
Solyc10g084310.1.1	30S ribosomal protein	520.39	497.63	457.74	448.31	481.02	134.55	122.55	103.49	120.2	0.25	3.09E-05
Solyc01g108500.2.1	Polyadenylate-binding	1376.79	1027.78	1316.56	1393.65	1278.69	308.98	372.15	365.53	348.89	0.27	3.11E-05
Solyc01g067480.2.1	Kelch-domain-contain	12.23	10.71	10.53	14.32	11.95	0.97	3.84	0.9	1.9	0.16	3.14E-05
Solyc04g012140.1.1	Unknown Protein (AH	1.59	0.71	2.81	0	1.28	233.74	63.47	347.13	214.78	168.13	3.21E-05
Solyc03g124040.2.1	Mitochondrial transcri	16.52	19.71	16.78	11.04	16.01	3.99	3.83	2.51	3.44	0.22	3.24E-05
Solyc06g061200.1.1	Glycine-rich protein T	46.44	55.04	32	19.03	38.13	5.08	1.77	4.09	3.65	0.1	3.24E-05
Solyc10g084280.1.1	Calmodulin-binding pr	55.92	42.53	63.7	66.04	57.05	9.79	15.49	10.25	11.84	0.21	3.24E-05
Solyc09g063060.2.1	GDSL esterase/lipase	17.21	20.42	7.19	4.06	12.22	0.14	0	0	0.05	0	3.28E-05
Solyc08g080170.2.1	Hydroxymethylglutary	59.27	55.67	59.51	97.75	68.05	8.75	15.33	6.08	10.05	0.15	3.31E-05
Solyc11g005200.1.1	Prolyl 4-hydroxylase a	19.08	30.69	24.14	24.06	24.49	90.44	82.04	79.86	84.11	3.43	3.31E-05
Solyc02g093590.2.1	CONSTANS-like zinc	44.19	41.15	33.46	39.83	39.66	7.8	11.89	6.32	8.67	0.22	3.35E-05
Solyc08g079240.2.1	Homology to unknown	69.18	66.08	84.45	63.47	70.79	11.65	24.38	13.26	16.43	0.23	3.36E-05
Solyc08g080150.1.1	TCP family transcripti	9.07	6.65	14.69	13.83	11.06	1.01	0.87	1.06	0.98	0.09	3.38E-05
Solyc06g066010.2.1	U11/U12 small nuclea	20.7	13.76	17.26	15.62	16.84	3.12	2.7	2.74	2.85	0.17	3.38E-05
Solyc08g082230.2.1	Unknown Protein (AH	12.13	16.59	11.52	14.23	13.62	38.96	36.88	17.62	31.15	2.29	3.39E-05
Solyc08g082180.2.1	Abscisic acid receptor	181.94	201.79	155.25	98.23	159.3	45.72	40.74	26.88	37.78	0.24	3.39E-05
Solyc01g088250.2.1	IQ-domain 11 (AHRD	62.09	66.13	63.08	47.17	59.62	20.09	18.97	7.56	15.54	0.26	3.40E-05
Solyc02g090900.2.1	Remorin (AHRD V1 *	7.85	5.9	10.91	6.67	7.83	0.14	1.42	0.17	0.58	0.07	3.40E-05
Solyc06g075580.2.1	Kinesin-5 (AHRD V1	12.06	12.87	9.91	16.79	12.91	0.77	4.63	0.81	2.07	0.16	3.42E-05
Solyc01g058540.2.1	WRKY transcription fa	4.06	5.6	3.59	2.3	3.89	0	0	0	0	0	3.44E-05
Solyc06g065990.1.1	ATP synthase subunit	32.69	28.31	43.09	56.66	40.19	3.09	9.12	5.55	5.92	0.15	3.44E-05
Solyc10g081910.1.1	Receptor like kinase, R	5.22	7.72	6.58	4.09	5.9	0.74	1.32	0.28	0.78	0.13	3.44E-05
Solyc01g094750.2.1	Cytochrome P450	4.63	5.9	4.58	1.59	4.18	0.1	0.1	0.25	0.15	0.04	3.46E-05
Solyc01g101060.2.1	S-adenosylmethionine	2137.2	1853.21	3312.6	2899.66	2550.67	289.35	615.21	391.9	432.15	0.17	3.48E-05
Solyc01g006430.2.1	Omega-6 fatty acid des	163.04	143.27	234.51	218.47	189.82	40.25	59.92	53.03	51.07	0.27	3.50E-05
Solyc09g083020.1.1	Myrosinase-binding pr	91.44	97.36	115.27	108.08	103.04	25.3	19.73	11.19	18.74	0.18	3.50E-05
Solyc10g083760.1.1	Threonine dehydratase	159.66	138.42	228.11	226.6	188.2	26.61	38.17	33.87	32.88	0.17	3.52E-05
Solyc06g075370.2.1	Dof zinc finger protein	7.6	13.52	13.83	11.36	11.58	1.4	2.65	0.86	1.64	0.14	3.56E-05
Solyc02g082290.2.1	MORC family CW-typ	8.07	9.91	13.03	11	10.5	1.94	2.43	1.81	2.06	0.2	3.56E-05
Solyc12g006230.1.1	RING-H2 finger protei	12.57	17.47	21.31	16.66	17	43.15	59.52	66.26	56.31	3.31	3.56E-05
Solyc12g098370.1.1	Myb family transcripti	42.46	37.98	38.27	41.88	40.15	6.86	13.31	6.7	8.96	0.22	3.57E-05
Solyc02g063520.2.1	Homeobox-leucine zip	41.98	45.49	29.84	36.88	38.55	6.53	9.35	7.25	7.71	0.2	3.58E-05
Solyc10g084530.1.1	Unknown Protein (AH	75.29	79.14	106.6	130.85	97.97	152.94	237.82	213.53	201.43	2.06	3.59E-05
Solyc06g069760.2.1	Dof zinc finger protein	10.57	10.24	18.79	13.25	13.21	2.27	2.51	1.99	2.26	0.17	3.62E-05
Solyc06g051130.1.1	Transferase family pro	5.31	4.18	2.5	5.36	4.34	0.12	0.74	0.15	0.34	0.08	3.63E-05
Solyc02g087890.2.1	Unknown Protein (AH	2.13	1.42	1.88	0	1.36	218.35	117.05	157.9	164.43	121.13	3.64E-05
Solyc05g011890.1.1	Sulfotransferase family	70.67	123.18	75.06	32.4	75.33	7.75	3.03	3.5	4.76	0.06	3.65E-05
Solyc08g068100.2.1	Bzip-like transcription	43.62	42.86	60.04	56.56	50.77	11.16	11.33	12.45	11.65	0.23	3.67E-05
Solyc08g081070.2.1	Mitochondrial carrier f	14	14.43	10.1	11.11	12.41	2.03	3.78	0.8	2.2	0.18	3.68E-05
Solyc09g089690.2.1	1-aminocyclopropane-	26.49	33.73	25.46	29.04	28.68	3.4	10.15	3.48	5.68	0.2	3.71E-05
Solyc06g051560.2.1	Flavoprotein wrbA (AH	2.03	1.81	0	1.44	1.32	11.48	20.68	5.04	12.4	9.39	3.72E-05
Solyc02g092620.2.1	BZIP domain class tran	6.63	9.18	10.87	4.69	7.84	25.31	19.22	32.92	25.82	3.29	3.74E-05
Solyc02g063390.2.1	DUF1295 domain prot	7.65	7.18	9.52	13.46	9.45	0.84	1.85	0.21	0.97	0.1	3.78E-05
Solyc11g069890.1.1	BEL1-like homeodoma	2.73	5.46	4.55	2.73	3.87	0.37	0.46	0.11	0.31	0.08	3.82E-05
Solyc12g005240.1.1	Unknown Protein (AH	13.93	17.14	7.98	10.43	12.37	1.16	1.84	0	1	0.08	3.83E-05
Solyc06g084420.1.1	LRR receptor-like seri	2.63	4.24	6.2	5.11	4.54	0.13	0.53	0.16	0.27	0.06	3.83E-05
Solyc07g008780.2.1	Unknown Protein (AH	29.37	25.55	25.86	32.55	28.33	64.99	70.01	52.43	62.48	2.21	3.86E-05
Solyc02g065680.2.1	Amino acid transporter	147.01	191.83	152.48	86.68	144.5	37.45	36.15	18.63	30.74	0.21	3.88E-05
Solyc09g090150.2.1	Legumin 11S-globulin	9.57	30.16	81.19	27.99	37.23	0	0	0	0	0	3.88E-05
Solyc11g011060.1.1	AT5g16610/MTG13_5	2.55	2.55	3.76	4.51	3.34	8.24	14.54	8.22	10.33	3.09	3.88E-05
Solyc10g086550.1.1	Cell division protease	21.15	21.49	27.25	23.04	23.23	52.25	48.34	49.88	50.16	2.16	3.93E-05
Solyc09g061280.2.1	Cyclin dependent kinas	4.89	4.04	0.82	3.95	3.42	15.51	10.24	41.58	22.44	6.55	3.94E-05
Solyc10g085000.1.1	Receptor-like kinase (	26.28	26.73	27.37	47.42	31.95	4.12	5.35	5.99	5.15	0.16	3.97E-05
Solyc11g066820.1.1	Cellulose synthase-like	230.33	343.67	306.73	245.29	281.5	64.16	103.21	62.74	76.7	0.27	3.97E-05
Solyc06g083680.2.1	Photosystem I reaction	236.36	206.55	212.22	198.72	213.46	55.6	69.23	64.54	63.12	0.3	4.00E-05
Solyc07g006370.1.1	Sodium/calcium excha	24.16	45.38	50.84	23.49	35.97	7.32	2.46	3.46	4.41	0.12	4.01E-05
Solyc03g025960.1.1	Unknown Protein (AH	3.94	1.75	1.16	2.79	2.41	32.62	28.88	43	34.83	14.45	4.06E-05
Solyc03g083960.2.1	Trehalose 6-phosphate	5.2	4.47	2.96	4.56	4.3	0.29	0	1.78	0.69	0.16	4.06E-05
Solyc10g051020.1.1	Cytochrome P450	0.64	1.7	2.26	2.17	1.69	9.79	5.3	13.43	9.51	5.62	4.08E-05
Solyc04g072780.2.1	Genomic DNA chromo	709.68	725.41	730.88	521.8	671.94	189.86	246.84	96.48	177.73	0.26	4.09E-05

Solyc06g072130.2.1	Aquaporin (AHRD V1	3.3	6.32	5.69	1.44	4.19	0	0	0	0	0	4.10E-05
Solyc06g068230.2.1	Protein TIF31 homolog	42.61	42.63	59.81	19.59	41.16	5.78	4.53	5.73	5.35	0.13	4.13E-05
Solyc10g085810.1.1	Vacuolar protein-sortin	40.14	29.48	25.5	59.56	38.67	72.12	97.91	114.25	94.76	2.45	4.13E-05
Solyc03g120390.2.1	Auxin responsive prote	1320.2	1071.94	1045.96	1271.01	1177.28	356.45	404.56	184.02	315.01	0.27	4.16E-05
Solyc06g083190.2.1	Peptidyl-prolyl cis-tran	144.68	127.74	127.64	125.29	131.34	194.75	325.82	286.76	269.11	2.05	4.16E-05
Solyc12g005150.1.1	Pollen ole e 1 allergen	487.38	329.12	375.92	490.07	420.62	124	147.74	73.05	114.93	0.27	4.19E-05
Solyc05g015800.2.1	Acetyl coenzyme A cis	2.2	4.45	4.16	0.83	2.91	20.54	11.13	22.17	17.95	6.17	4.19E-05
Solyc11g071270.1.1	Class E vacuolar prote	12.89	7.62	10.32	12.51	10.84	23.15	26.1	22.62	23.96	2.21	4.21E-05
Solyc01g005460.2.1	Dynein light chain 1 cy	9.06	8.96	2.38	5.7	6.53	0	0	0	0	0	4.21E-05
Solyc05g009570.2.1	Ribosomal protein PSI	62.83	74.09	47.26	40.55	56.18	15.55	15.68	5.46	12.23	0.22	4.25E-05
Solyc12g011130.1.1	Ras-related protein Ra	24.27	26.86	16.52	12.76	20.1	49.78	65.17	40.77	51.91	2.58	4.25E-05
Solyc08g081310.2.1	Os10g0358600 protein	41.45	49.62	54.76	46.66	48.12	12.97	9.51	16.52	13	0.27	4.27E-05
Solyc07g064210.2.1	2S albumin seed storag	34.87	52.42	96.73	11.33	48.84	0	0	0	0	0	4.27E-05
Solyc02g081970.2.1	Vacuolar sorting recep	2.79	2.76	2.8	2.48	2.71	15.76	10.43	20.09	15.43	5.7	4.31E-05
Solyc03g123390.2.1	Hydrolase (AHRD V1	3.73	3.96	4.83	4.78	4.33	0.14	0.14	0	0.09	0.02	4.32E-05
Solyc08g076390.2.1	Lysine-specific demeth	2.18	2.98	2.11	4.08	2.84	8.12	8	21.42	12.51	4.41	4.32E-05
Solyc04g009820.2.1	Calcium-responsive tra	17.05	12.64	12.29	16.52	14.62	1.28	3.34	2.19	2.27	0.16	4.32E-05
Solyc00g206460.1.1	Os06g0220000 protein	12.32	12.11	7.27	18.52	12.55	0.41	1.46	6.88	2.92	0.23	4.33E-05
Solyc05g012270.2.1	Argininosuccinate synt	26.92	26.69	22.33	20.55	24.12	52.87	61.14	55.56	56.52	2.34	4.37E-05
Solyc06g005490.2.1	Triosephosphate isome	41.42	35.73	34.09	28.57	34.95	137.17	113.97	97.97	116.37	3.33	4.37E-05
Solyc09g064630.2.1	TPR domain protein (A	32.43	31.33	14.57	17.99	24.08	1.04	3.9	0.73	1.89	0.08	4.37E-05
Solyc02g067660.2.1	Phytoalexin deficient 4	21.84	12.92	8.08	21.85	16.17	1.13	0.85	0.58	0.85	0.05	4.42E-05
Solyc05g055070.2.1	Ethylene receptor (AH	6.81	9.13	14.29	12.63	10.71	24.08	21.69	26.68	24.15	2.25	4.42E-05
Solyc02g090460.2.1	Unknown Protein (AH	104.23	75.15	105.85	50.39	83.91	9.59	23.65	13.1	15.45	0.18	4.46E-05
Solyc01g073700.2.1	26S protease regulator	2.88	2.9	1.18	1.6	2.14	0	0.1	0	0.03	0.02	4.46E-05
Solyc10g038080.1.1	Shikimate dehydrogen	1.92	3.76	3.17	3.44	3.07	40.26	29.44	46.66	38.79	12.62	4.48E-05
Solyc08g082990.2.1	Oligopeptide transport	2.73	2.74	14.01	4.35	5.96	80.23	74.76	74.39	76.46	12.83	4.48E-05
Solyc06g083270.2.1	ER lumen retaining rec	7.44	8.75	2.83	4.41	5.86	24.81	19.35	23.1	22.42	3.83	4.50E-05
Solyc03g120450.2.1	Aminotransferase (AH	29.39	26.61	30.34	16.77	25.78	4.99	8.85	3.65	5.83	0.23	4.50E-05
Solyc04g048900.2.1	Calreticulin 2 calcium-	216.24	148.34	195.9	95.57	164.01	13.26	21.17	11.86	15.43	0.09	4.53E-05
Solyc04g082830.2.1	Auxin efflux carrier fa	13.29	12.57	15.88	14.81	14.14	56.39	41.16	36.62	44.72	3.16	4.56E-05
Solyc01g097160.2.1	FAD dependent oxidor	26.07	29.53	27.19	24.4	26.8	6.06	6.97	10.98	8	0.3	4.59E-05
Solyc08g081940.2.1	Receptor like kinase, R	10.67	12.14	12.95	13.06	12.21	2.4	3.68	3.16	3.08	0.25	4.66E-05
Solyc02g065780.1.1	Unknown Protein (AH	129.9	279.04	85.05	73.11	141.78	1.1	1.11	1.35	1.19	0.01	4.67E-05
Solyc07g005470.2.1	Reticulon-like protein	1.16	1.03	0.84	1.77	1.2	0	0	0	0	0.01	4.67E-05
Solyc08g076290.2.1	Unknown Protein (AH	2.57	4.34	2.88	2.18	2.99	0.31	0	0.38	0.23	0.08	4.67E-05
Solyc11g066840.1.1	Histone deacetylase-lik	928.37	699.58	745.77	776.46	787.55	138.27	235.32	125.82	166.47	0.21	4.67E-05
Solyc09g072850.1.1	Unknown Protein (AH	30.33	44.54	34	19.31	32.05	5.51	6.8	4.52	5.61	0.18	4.69E-05
Solyc01g088030.2.1	Structure-specific endo	3.28	2.04	2.71	3.71	2.94	11.67	15.24	14.01	13.64	4.65	4.70E-05
Solyc05g008090.2.1	Os02g0200800 protein	13.85	17.29	21.67	17	17.45	2.8	5.49	2	3.43	0.2	4.70E-05
Solyc07g063600.2.1	Chlorophyll a-b bindin	12.54	10.27	5.03	6.03	8.47	0.61	0.82	0.5	0.64	0.08	4.70E-05
Solyc04g012160.2.1	Serine-threonine prote	12.2	14.42	14.62	14.29	13.88	203.8	88.09	213.67	168.52	12.14	4.71E-05
Solyc09g011030.2.1	Hsp70 nucleotide exch	53.71	50.16	62.38	62.6	57.21	108.2	122.91	174.4	135.17	2.36	4.72E-05
Solyc02g076750.1.1	Cathepsin B-like cyste	10.53	10.54	17.74	23.66	15.62	1.67	1.07	1.68	1.47	0.09	4.77E-05
Solyc12g044310.1.1	Solute carrier family 1	3.04	3.17	0.62	1.78	2.15	0	0	0.1	0.03	0.02	4.79E-05
Solyc10g085010.1.1	PAR-1c protein (AHRI	1.4	0.94	0.41	3.47	1.55	16.73	37.74	92.3	48.92	31.46	4.80E-05
Solyc07g055660.2.1	Unknown Protein (AH	26.55	25.06	25.15	22.76	24.88	61.1	68.18	63.49	64.26	2.58	4.84E-05
Solyc08g080480.2.1	Unknown Protein (AH	7.71	17.54	28.81	8.48	15.64	0	0	0	0	0	4.86E-05
Solyc01g091770.2.1	Ring H2 finger protein	3.24	5.45	3.4	7.14	4.81	0	0	0	0	0	4.87E-05
Solyc02g071100.2.1	Purine permease famil	5.44	6.4	5.59	2.98	5.1	34.87	35.46	24.9	31.74	6.22	4.89E-05
Solyc11g044320.1.1	Single myb histone (A	15.34	9.75	12.15	14.57	12.95	35.24	26.08	32.63	31.32	2.42	4.90E-05
Solyc03g082870.2.1	WD-40 repeat family p	7.16	6.91	7.22	10	7.82	1.74	0.91	1.28	1.31	0.17	4.93E-05
Solyc09g010280.2.1	Os08g0482100 protein	32.99	41.01	45.27	35.44	38.68	96.63	82.95	83.52	87.7	2.27	4.93E-05
Solyc08g028970.2.1	Unknown Protein (AH	24.5	40.26	24.46	24	28.3	89.85	113.66	65.48	89.66	3.17	4.97E-05
Solyc01g009080.2.1	FHA domain containin	41.92	40.78	36.42	18.85	34.49	8.66	6.89	5.81	7.12	0.21	4.99E-05
Solyc01g104380.2.1	Blue copper protein (A	4.83	10.5	12.02	3.79	7.79	0	0	0	0	0	5.07E-05
Solyc12g096900.1.1	Tir, resistance protein	41.04	37.29	21.25	30.37	32.49	6.85	5.64	3.61	5.37	0.17	5.07E-05
Solyc04g076690.2.1	Unknown Protein (AH	73.64	86.02	99.55	82.47	85.42	787.08	445.06	1015.74	749.29	8.77	5.09E-05
Solyc05g012850.2.1	Unknown Protein (AH	1.48	0.66	0.58	2.79	1.38	4.18	10.03	12.95	9.05	6.57	5.13E-05
Solyc03g120410.1.1	Hypothetical conserved	41.91	50.96	34.76	62.8	47.61	3.35	12.85	3.71	6.64	0.14	5.20E-05
Solyc01g103050.2.1	Auxin response factor	52.58	58.71	62.62	70.66	61.14	19.12	16.58	15.8	17.17	0.28	5.30E-05
Solyc03g098100.2.1	Reductase 2 (AHRD V	0.34	0.6	0.79	0.95	0.67	5.44	5.21	3.68	4.78	7.13	5.44E-05
Solyc05g052860.2.1	Os12g0581300 protein	0.3	0.4	1.23	0.21	0.54	2.64	2.78	5.16	3.53	6.59	5.44E-05
Solyc06g069790.2.1	Gibberellin-regulated p	15.08	15.1	2.97	9.78	10.73	0	0	0	0	0	5.44E-05
Solyc07g063590.2.1	Myosin-like protein (A	1.94	2.32	2.23	2.67	2.29	0.22	0.36	0.5	0.36	0.16	5.44E-05
Solyc05g046030.2.1	Peroxidase (AHRD V1	2.41	4.3	1.42	1.99	2.53	0	0	0	0	0	5.52E-05
Solyc09g065850.2.1	Auxin responsive prote	11.7	18.94	6.28	7.53	11.11	53.3	35.84	124.63	71.26	6.41	5.53E-05
Solyc11g073130.1.1	Protein tyrosine phospl	6.41	7.61	3.6	5.18	5.7	0.25	0.5	0	0.25	0.04	5.55E-05
Solyc03g062900.2.1	Unknown Protein (AH	5.55	4.6	5.66	2.68	4.62	0.2	0.31	0.38	0.3	0.06	5.55E-05
Solyc02g078780.2.1	Receptor like kinase, R	7.95	9.53	11.69	12.37	10.38	2.6	2.18	2.22	2.33	0.22	5.56E-05
Solyc03g122360.2.1	Cytochrome P450	2.08	1.85	6.6	2.58	3.28	20.18	10.71	36.68	22.52	6.87	5.58E-05
Solyc02g090820.2.1	Heat stress transcriptio	80.83	78.16	115.97	156.04	107.75	169.07	208.62	416.09	264.59	2.46	5.58E-05
Solyc03g118390.2.1	Chromosome 14 contig	28.46	25.95	23.43	41.07	29.73	48.51	59.53	73.21	60.42	2.03	5.64E-05
Solyc12g088690.1.1	Glucosyltransferase (A	5.77	6.97	6.81	7.58	6.78	0.17	1.51	0.2	0.63	0.09	5.67E-05
Solyc03g121910.1.1	Threonine synthase (A	42.5	33.65	51.19	56.89	46.06	6.97	12.72	11.71	10.47	0.23	5.73E-05
Solyc03g112340.1.1	Ring H2 finger protein	3.47	4.27	4.88	2.81	3.86	12.56	39.34	28.23	26.71	6.92	5.74E-05
Solyc03g111830.2.1	Protein-L-isoaspartate	46.49	49.06	43.09	38.83	44.37	88	111.48	95.37	98.28	2.22	5.78E-05
Solyc10g084670.1.1	Eukaryotic translation	3.84	4.29	3.55	4.55	4.06	0.23	1.08	0.62	0.64	0.16	5.78E-05
Solyc08g065970.2.1	RNA polymerase sigm	17.45	19.68	18.89	13.86	17.47	2.8	5.55	1.54	3.3	0.19	5.78E-05
Solyc00g007300.2.1	Lrr, resistance protein	5.69	5.17	6.58	5.59	5.76	9.48	15.99	34.7	20.06	3.48	5.79E-05
Solyc10g018120.1.1	Unknown Protein (AH	0.13	1.05	2.64	1.67	1.37	7.96	8.25	3.78	6.66	4.85	5.86E-05
Solyc12g098960.1.1	Receptor-like protein k	13.46	14.64	12.74	25.39	16.56	1.61	2.44	0.99	1.68	0.1	5.86E-05

Solyc07g017950.2.1	Ribulose bisphosphate	9.31	14.16	5.49	5.49	8.61	0.31	0.32	0.39	0.34	0.04	5.86E-05
Solyc01g098570.2.1	Unknown Protein (AH	5.89	3.07	5.75	3.45	4.54	190.4	142.25	90.87	141.17	31.1	5.89E-05
Solyc02g078840.2.1	Secretory carrier meml	25.32	24.05	31.38	28.6	27.34	62.24	57.05	67.59	62.29	2.28	5.89E-05
Solyc01g091130.2.1	Nitroreductase (AHRD	2.35	1.43	3.15	2.57	2.38	4.92	7.05	10.28	7.42	3.12	6.03E-05
Solyc04g077230.1.1	Unknown Protein (AH	45.74	47.75	61.23	58.48	53.3	6.4	7.88	7.86	7.38	0.14	6.12E-05
Solyc01g100460.2.1	BZIP transcription fact	3.32	5.49	3.36	1.34	3.38	21.09	28.62	25.91	25.21	7.46	6.13E-05
Solyc07g051840.2.1	WRKY transcription fa	3.76	2.14	2.59	5.61	3.53	8.01	12.16	11.5	10.56	2.99	6.13E-05
Solyc10g084250.1.1	Receptor-like kinase (A	9.5	11.87	19.08	16.16	14.15	1.03	3.13	2.18	2.11	0.15	6.13E-05
Solyc09g015000.2.1	class I heat shock prote	49.84	40.45	31.13	25.58	36.75	668.01	1325.22	433.15	808.79	22.01	6.19E-05
Solyc03g115900.2.1	Chlorophyll a-b bindin	265.72	229.03	126.32	86.65	176.93	15.86	7.68	3.12	8.89	0.05	6.19E-05
Solyc09g009100.2.1	Heat stress transcriptio	22.12	27.51	24.42	23.97	24.51	4.81	5.16	8.47	6.15	0.25	6.27E-05
Solyc01g065510.2.1	Unknown Protein (AH	6.03	4.03	11.12	11.73	8.23	0	0.61	0	0.2	0.02	6.33E-05
Solyc06g082910.2.1	Formin 3 (AHRD V1 *	10.35	12.28	17.19	29.18	17.25	0.46	0.57	0.28	0.44	0.03	6.35E-05
Solyc07g064060.2.1	Breast carcinoma ampl	15.75	16.79	23.46	15.81	17.95	39.87	36.73	41.93	39.51	2.2	6.39E-05
Solyc04g064850.2.1	Transmembrane protei	2.33	4.64	4.53	3.69	3.8	18.95	12.41	17.43	16.26	4.28	6.39E-05
Solyc09g005260.2.1	Calcium/proton exchar	2.75	4.9	11.12	8.2	6.74	0.12	0.12	0	0.08	0.01	6.57E-05
Solyc02g089980.2.1	Ternary complex facto	27.33	29.47	24.96	35.69	29.36	6.58	6.53	4.57	5.89	0.2	6.58E-05
Solyc03g007170.2.1	FK506-binding protein	20.85	22.95	24.11	21.15	22.27	3.1	6.26	2.18	3.85	0.17	6.60E-05
Solyc08g007130.2.1	Beta-amylase 8 (AHRI	1.21	3.78	3.3	1.72	2.5	0.2	0	0	0.07	0.03	6.60E-05
Solyc04g078060.1.1	Small hydrophobic pro	6.2	5.02	4.66	6.38	5.56	15.49	24.35	14.55	18.13	3.26	6.64E-05
Solyc05g051020.2.1	Dihydroflavonol 4-red	7.76	6.41	8.5	16.47	9.79	0	0	0.55	0.18	0.02	6.64E-05
Solyc10g079240.1.1	Calmodulin binding pr	10.47	11.69	10.33	19.46	12.99	1.64	2.17	2.48	2.1	0.16	6.64E-05
Solyc10g074800.1.1	Unknown Protein (AH	3.43	4.31	2.62	1.43	2.95	0	0.16	0	0.05	0.02	6.64E-05
Solyc03g121400.1.1	Dof zinc finger protein	1.25	2.23	1.11	0.88	1.37	6.06	10.96	23.28	13.43	9.82	6.71E-05
Solyc03g006910.2.1	BHLH transcription fa	2.93	9.23	10.67	6.4	7.31	0	0.87	0	0.29	0.04	6.72E-05
Solyc07g014640.2.1	Galactokinase-like pro	14.23	11.48	9.27	10.48	11.37	1.32	3.38	1.03	1.91	0.17	6.73E-05
Solyc06g048620.2.1	Receptor like kinase, R	1.56	2.42	1.52	1.82	1.83	0.22	0.17	0.27	0.22	0.12	6.74E-05
Solyc01g060460.1.1	DNA repair protein (A	0	0.15	0.1	0	0.06	0.91	1.48	0.43	0.94	15.04	6.77E-05
Solyc01g089980.2.1	Engulfment and cell m	54.61	52.56	55.68	57.67	55.13	14.17	16.11	18.88	16.39	0.3	6.77E-05
Solyc02g090480.2.1	Peptidyl-prolyl cis-trar	4.74	5.51	4.3	7.48	5.51	0.15	0.74	0	0.3	0.05	6.77E-05
Solyc09g010860.2.1	Expansin (AHRD V1 *	43.73	49.15	68.99	117.35	69.81	4.24	4.07	6.95	5.09	0.07	6.78E-05
Solyc10g006890.2.1	WD-40 repeat family p	16.62	16.85	12.93	17.4	15.95	36.74	28.37	42.4	35.84	2.25	6.78E-05
Solyc09g083440.2.1	Proteinase inhibitor I (	98.96	116.9	25.71	33.33	68.73	0	1.44	0.58	0.67	0.01	6.85E-05
Solyc10g080210.1.1	Polygalacturonase A (A	1.87	1.92	4.59	11.61	5	5605.89	3389.31	7538.32	5511.17	1102.79	6.85E-05
Solyc10g055720.1.1	Kinase family protein (	20	19.05	18.49	17.7	18.81	43.28	42.22	48.3	44.6	2.37	6.88E-05
Solyc02g088490.2.1	Saccharopine dehydrog	53.13	55.6	82.31	84.03	68.77	12.85	20.58	14.78	16.07	0.23	6.92E-05
Solyc12g005980.1.1	Zinc ion binding protei	15.6	14.65	14.47	13.24	14.49	26.34	34.11	49.03	36.49	2.52	7.03E-05
Solyc11g008280.1.1	Serine carboxypeptidas	11.7	14.5	17.36	16.36	14.98	4.25	3.32	3.13	3.57	0.24	7.04E-05
Solyc07g055050.2.1	ATP synthase I-like pr	49.43	50.57	40.41	56.71	49.28	7.86	17.13	11.4	12.13	0.25	7.07E-05
Solyc12g099360.1.1	Acyl-CoA synthetase/A	67.02	57.99	95.37	60.79	70.29	138.59	193.92	161.05	164.52	2.34	7.07E-05
Solyc04g072460.2.1	BZIP transcription fact	14.76	16.71	22.58	18.04	18.02	4.12	2.97	3.62	3.57	0.2	7.20E-05
Solyc04g082750.2.1	3-beta hydroxysteroid	13.63	14.1	12.84	18.31	14.72	0.89	4.15	1.78	2.27	0.15	7.27E-05
Solyc12g056940.1.1	Acetyl-CoA carboxylas	8.5	10.09	15.07	8.57	10.56	107.06	71.8	94.06	90.97	8.62	7.29E-05
Solyc02g082340.2.1	RNA polymerase I-spe	161.51	169.87	221.78	183.16	184.08	38.55	53.93	53.61	48.7	0.26	7.30E-05
Solyc01g098950.1.1	Glyceraldehyde-3-phos	51.31	40.83	47.15	65.89	51.3	96.66	117.15	138.94	117.58	2.29	7.30E-05
Solyc08g014150.2.1	GRAM domain contai	16.27	12.37	20.97	13.81	15.86	3.24	4.08	2.73	3.35	0.21	7.44E-05
Solyc08g007800.2.1	Transporter major faci	3.64	4.73	6.39	4.18	4.74	15.19	13.32	15.93	14.81	3.13	7.45E-05
Solyc05g008110.2.1	Unknown Protein (AH	411.66	460.79	778.32	525.55	544.08	2047.09	2024.3	2036.76	2036.05	3.74	7.47E-05
Solyc08g077980.2.1	Bax inhibitor (AHRD V	88.67	97	97.76	103.77	96.8	155.21	224.92	260.1	213.41	2.2	7.48E-05
Solyc07g005390.2.1	Aldehyde dehydrogena	40.49	40.64	38.52	25.91	36.39	7.08	9.41	3.56	6.68	0.18	7.51E-05
Solyc03g005270.2.1	Transmembrane emp24	73.88	67.37	59.54	58.91	64.92	149.09	147.15	108.03	134.76	2.08	7.57E-05
Solyc03g043860.2.1	Nudix hydrolase 1 (Atl	1.31	1.17	1.55	1.85	1.47	18.71	15.31	4.77	12.93	8.8	7.64E-05
Solyc10g086500.1.1	3-oxo-5-alpha-steroid	25.83	26.63	54.9	28.94	34.07	4.96	4.38	1.52	3.62	0.11	7.65E-05
Solyc07g056590.2.1	Polyadenylate-binding	25.18	23.45	26.71	29.54	26.22	51.41	63.46	63.16	59.34	2.26	7.71E-05
Solyc11g069070.1.1	Metal ion binding prot	13.98	10.12	10.11	28.21	15.61	0.15	0.15	0	0.1	0.01	7.77E-05
Solyc01g095080.2.1	1-aminocyclopropane-	0.41	0.72	0.16	2.5	0.95	583.87	246.89	715.32	515.36	543.92	7.79E-05
Solyc07g054560.1.1	CK25 (AHRD V1 ***-	4.57	5.28	2.15	2.91	3.73	0.18	0.19	0	0.12	0.03	7.79E-05
Solyc03g111180.2.1	Aspartyl aminopeptida	35.38	39.02	34	34.39	35.7	79.67	71.9	68.06	73.21	2.05	7.85E-05
Solyc02g063170.2.1	Homology to unknown	5.34	6.42	7.56	3.4	5.68	0.65	0.22	0.27	0.38	0.07	7.90E-05
Solyc06g076400.2.1	Protein phosphatase 2C	56.98	58.71	73.29	26.11	53.77	6.87	9.02	4.3	6.73	0.13	7.92E-05
Solyc08g041700.1.1	F-box domain containi	3.6	3.5	2.12	1.39	2.65	0	0.13	0	0.04	0.02	7.93E-05
Solyc07g049430.2.1	GDSL esterase/lipase A	3.54	5.14	3.08	1.32	3.27	0	0.15	0	0.05	0.02	7.97E-05
Solyc03g083420.2.1	OBP3-responsive gene	19.47	21.44	24.01	18.08	20.75	41.38	46.31	48.31	45.33	2.18	8.00E-05
Solyc07g066100.2.1	GPI-anchored protein (	50.56	35.86	41.01	87.73	53.79	97.34	113.63	115.28	108.75	2.02	8.03E-05
Solyc11g068430.1.1	Ferredoxin (AHRD V1	121.91	146.08	191.78	173.94	158.43	475.42	418.02	431.66	441.7	2.79	8.10E-05
Solyc03g120140.2.1	Bzip-like transcription	45.58	45.05	56.48	65.45	53.14	118.63	97.06	149.61	121.77	2.29	8.17E-05
Solyc01g111170.2.1	Peptidyl-prolyl cis-trar	3330.01	3179.77	2590.63	2833.11	2983.38	959.06	1027.02	718.4	901.49	0.3	8.25E-05
Solyc03g113030.2.1	Aldose 1-epimerase-lik	40.21	65.31	45.47	64.22	53.8	188.04	206.12	166.1	186.75	3.47	8.32E-05
Solyc05g013460.2.1	Multidrug resistance pi	22.8	26.91	27.96	31.35	27.26	5.07	8.56	3.05	5.56	0.2	8.36E-05
Solyc03g111970.2.1	Cytochrome P450	13.27	16.44	19.19	15.09	16	640.74	381.5	537.16	519.8	32.49	8.43E-05
Solyc02g093600.2.1	class I heat shock prote	4.82	5.57	5.11	3.41	4.73	103.48	120.46	40.63	88.19	18.65	8.51E-05
Solyc11g069460.1.1	DsRNA-binding protei	11.76	9.05	12	18.89	12.93	1.03	1.68	1.74	1.48	0.11	8.55E-05
Solyc09g009700.2.1	GDSL esterase/lipase A	2.68	3.82	2.74	2.53	2.94	0.14	0.15	0.18	0.16	0.05	8.57E-05
Solyc07g047820.1.1	Pentatricopeptide repea	2.67	2.95	2.9	2.86	2.84	7.99	6.18	7.88	7.35	2.58	8.70E-05
Solyc02g069620.2.1	Ribose 5-phosphate iso	31.76	36.37	16.51	26.71	27.84	4.67	7.42	3.31	5.13	0.18	8.75E-05
Solyc03g097370.2.1	DNA helicase-like (AH	12.49	12.3	14.18	11.62	12.65	57.94	37.33	64.82	53.36	4.22	8.77E-05
Solyc02g079100.2.1	Riboflavin kinase (AH	29.74	32.7	38.51	31.67	33.16	64.9	68.52	83.63	72.35	2.18	8.79E-05
Solyc04g056320.1.1	Zinc finger family prot	29.46	33.44	39.33	38.92	35.29	6.98	10.64	7.88	8.5	0.24	8.87E-05
Solyc02g080480.2.1	Multidrug resistance pi	5.28	5.99	2.33	2.05	3.91	0	0.32	0.13	0.15	0.04	8.91E-05
Solyc01g095540.2.1	UDP-galactose transpo	9.37	16.85	11.29	9.3	11.7	22.22	32.48	24.53	26.41	2.26	8.93E-05
Solyc02g091930.2.1	Homeobox-leucine zip	101.78	107.82	92.88	57.59	90.02	32.1	26.78	17.04	25.31	0.28	8.95E-05
Solyc12g056710.1.1	Xanthoxin dehydrogen	0	0	0.55	0	0.14	9.76	3.03	20.52	11.1	80.75	8.95E-05

Solyc05g026310.2.1	Derlin-3 (AHRD V1 *	16.08	21.47	11.16	9.7	14.6	34.96	37.57	42.48	38.34	2.63	8.98E-05
Solyc02g080110.2.1	Unknown Protein (AH	12.45	14.68	11.52	4.28	10.73	76.41	64.73	62.14	67.76	6.31	9.00E-05
Solyc09g090640.2.1	Unknown Protein (AH	17.04	18.64	15.49	19.03	17.55	30.51	40.5	60.1	43.7	2.49	9.00E-05
Solyc12g099280.1.1	Pre-mRNA polyadenyl	7.06	6.02	9.08	8.25	7.6	0.8	2	0.81	1.2	0.16	9.02E-05
Solyc09g008970.1.1	Unknown Protein (AH	8.67	7.29	3.41	8.17	6.88	143.56	441.44	188.8	257.93	37.46	9.22E-05
Solyc08g082160.2.1	Unknown Protein (AH	57.72	72.78	18.65	41.8	47.74	3.33	3.36	0.68	2.46	0.05	9.25E-05
Solyc01g086960.2.1	Zinc finger A20 and A	157.77	147.56	184.08	216.26	176.42	308.79	395.64	557.12	420.52	2.38	9.28E-05
Solyc01g080260.2.1	At4g14280-like protei	0	0.07	0.28	0	0.09	1.35	1.04	0.32	0.9	10.32	9.37E-05
Solyc06g062790.2.1	Casein kinase I-like (A	41.3	44.66	42.74	37.88	41.64	91.22	82.47	98.36	90.68	2.18	9.37E-05
Solyc04g055110.2.1	Mitochondrial import r	7.77	9.16	13.88	11.44	10.56	17.31	22.68	24.5	21.5	2.04	9.38E-05
Solyc03g025870.2.1	MYB transcription fac	4.48	5.45	3.61	3.33	4.22	0	0.58	0	0.19	0.05	9.40E-05
Solyc02g070020.1.1	UDP-glucosyltransfera	0.82	1.1	1.46	1.36	1.19	448.82	190.62	437.91	359.12	303.05	9.40E-05
Solyc01g005000.2.1	Glutamate decarboxyla	44.48	42.86	18.94	20.2	31.62	3.3	2.66	1.76	2.57	0.08	9.44E-05
Solyc07g065500.1.1	Nuclear transcription f	24.87	31.44	17.44	18.87	23.16	7.86	4.41	8.59	6.95	0.3	9.61E-05
Solyc01g096240.2.1	Homology to unknown	236.02	240.15	194.72	206.17	219.26	56.38	84.42	54.84	65.21	0.3	9.66E-05
Solyc08g083220.1.1	Unknown Protein (AH	6.52	4.41	3.38	9.96	6.07	0	0.21	0	0.07	0.01	9.72E-05
Solyc03g115890.2.1	Expansin (AHRD V1 *	14.19	12.16	10.54	4.46	10.34	1.06	1.5	0.26	0.94	0.09	9.72E-05
Solyc06g005670.2.1	mRNA binding protein	23.78	22.84	30.36	22.69	24.92	67.21	45.57	55.79	56.19	2.26	9.72E-05
Solyc08g066260.2.1	Histidine decarboxylas	0	0.34	0.44	0.8	0.4	489.28	132.09	817.78	479.72	1214.47	9.73E-05
Solyc05g047460.2.1	Auxin response factor	11	9.41	14.67	16.15	12.81	4.66	2.35	5.31	4.11	0.32	9.74E-05
Solyc03g115160.2.1	PHD-finger family pro	3.28	2.16	1.68	0.81	1.98	0	0	0.14	0.05	0.02	9.78E-05
Solyc09g007880.1.1	Unknown Protein (AH	24.19	33.27	16.86	10.89	21.3	73.73	83.32	46.93	67.99	3.19	9.81E-05
Solyc06g062780.2.1	Phospholipid-transport	23.37	18.62	19.23	28.44	22.41	3.6	7.09	3.72	4.8	0.21	9.87E-05
Solyc02g080610.2.1	Ankyrin repeat domain	1.46	0.5	1.73	2.07	1.44	0	0	0	0	0.01	9.87E-05
Solyc01g006130.2.1	Homology to unknown	165.07	161.53	110.46	105.54	135.65	43.52	49.3	33.46	42.09	0.31	9.92E-05
Solyc04g081490.2.1	Tubulin beta-1 chain (	459.61	467.89	409.13	613.78	487.6	106.36	168.53	94.17	123.02	0.25	9.92E-05
Solyc05g053210.2.1	CBL-interacting protei	88.15	109.4	187.07	94.13	119.69	487.32	521.72	364.63	457.89	3.83	9.92E-05
Solyc08g065190.2.1	Zinc transporter protei	13.74	14.82	14.12	11.21	13.47	32.53	28.33	31.14	30.67	2.28	9.92E-05
Solyc12g099290.1.1	Aldehyde dehydrogena	6.33	6.92	8.91	10.68	8.21	1.44	1.99	2.2	1.88	0.23	0.000101
Solyc03g020050.2.1	Proteinase inhibitor II	50.29	57.26	16.18	23.1	36.71	0.53	1.86	0.97	1.12	0.03	0.000101
Solyc06g008050.2.1	Monooxygenase (AHR	1.44	1.14	1.13	1.13	1.21	81.11	44.36	29.09	51.52	42.58	0.000102
Solyc11g071420.1.1	Cc-nbs-lrr, resistance p	1.36	1.46	1.22	1.77	1.45	0.18	0.09	0.22	0.16	0.11	0.000102
Solyc01g102860.2.1	Unknown Protein (AH	24.65	29.68	24.23	22.9	25.37	4.49	8.86	4.08	5.81	0.23	0.000102
Solyc12g056440.1.1	Major facilitator super	8.13	7.23	5.69	5.48	6.63	14.97	16.48	18.95	16.8	2.53	0.000103
Solyc04g006970.2.1	Phosphoenolpyruvate c	197.85	204.07	280.08	314.93	249.23	10.95	60.15	10.4	27.17	0.11	0.000103
Solyc10g079580.1.1	RNA-binding region R	34.62	27.61	36.99	33.72	33.23	67.55	78.77	102.44	82.92	2.49	0.000103
Solyc10g080530.1.1	Unknown Protein (AH	5.23	7.52	8.54	3.41	6.17	0.49	0.98	0.2	0.56	0.09	0.000103
Solyc11g013470.1.1	Auxin response factor	1.94	1.73	1.14	0.55	1.34	4.39	6.48	6.74	5.87	4.38	0.000104
Solyc11g066890.1.1	Prephenate dehydratase	10.33	7.41	15.46	24.42	14.41	197.5	197.19	183.59	192.76	13.38	0.000104
Solyc03g006760.2.1	Lrr, resistance protein	3.53	3.79	5.12	7.17	4.9	0.29	0.52	0.18	0.33	0.07	0.000106
Solyc09g065180.2.1	NAD-dependent epime	95.56	90.52	62.95	54.89	75.98	10.06	27.8	7.86	15.24	0.2	0.000107
Solyc01g087640.2.1	Cinnamoyl CoA reduc	38.3	42.3	36.67	33.19	37.61	94.45	79.41	70.86	81.57	2.17	0.000107
Solyc04g015740.2.1	DUF288 containing pr	16.96	20.43	14.7	16.17	17.07	5	4.66	4.28	4.65	0.27	0.00011
Solyc09g011740.2.1	Phosphatidylcholine:c	13.11	12.04	7.61	13.84	11.65	28.75	25.78	55.98	36.84	3.16	0.00011
Solyc11g020040.1.1	Chaperone DnaK (AH	19.04	15.59	20.1	21.54	19.07	68.76	199.9	120.38	129.68	6.8	0.00011
Solyc02g086710.2.1	Monodehydroascorbat	39.42	43.24	41.66	34.65	39.74	84.92	80.64	82.6	82.72	2.08	0.000111
Solyc12g042950.1.1	ADP/ATP carrier prot	176.55	149.21	143.93	124.42	148.53	36.72	49.92	47.5	44.71	0.3	0.000111
Solyc01g088660.2.1	Os04g0585900 protein	1143.94	1356.88	1386.19	1398.8	1321.45	310.73	450.49	264.55	341.92	0.26	0.000113
Solyc10g007360.2.1	Multidrug resistance p	1.37	2.31	1.61	1.16	1.61	3.86	6.12	3.8	4.59	2.85	0.000113
Solyc02g071280.2.1	Ribosome maturation	5.94	8.87	7.25	4.27	6.58	13.67	19.53	22.79	18.66	2.84	0.000113
Solyc01g107740.2.1	Protein unc-13 homolo	17.94	16.71	18.89	26.16	19.93	48.84	39.81	89.04	59.23	2.97	0.000113
Solyc03g117330.2.1	Prolyl-tRNA synthetas	6.66	5.23	12.79	9.79	8.62	0.42	0.85	1.17	0.81	0.09	0.000115
Solyc02g087400.1.1	Ankyrin repeat domain	58.1	46.14	47.04	34.14	46.36	10.26	16.61	5.5	10.79	0.23	0.000115
Solyc01g010270.2.1	Nitrilase associated pr	564.21	564.21	441.05	532.34	525.45	147.21	196.6	109.76	151.19	0.29	0.000116
Solyc12g096610.1.1	Poly(ADP-ribose) glyc	15.45	15.61	21.62	13.7	16.59	2.96	2.99	3.12	3.02	0.18	0.000117
Solyc10g005050.2.1	Thylakoid membrane p	212.49	186.52	216.3	225.81	210.28	55.36	73.66	61.86	63.63	0.3	0.000117
Solyc11g042950.1.1	Carbohydrate kinase F	32.63	45.46	40.17	31.11	37.34	1.72	9.25	4.22	5.06	0.14	0.000117
Solyc06g066000.1.1	ATP synthase subunit	210.87	208.12	252.26	237.87	227.28	54.49	85.68	74.84	71.67	0.32	0.000117
Solyc08g068330.2.1	Aspartate aminotransf	39.61	30.72	28.35	39.14	34.45	115.54	104.43	111.02	110.33	3.2	0.000117
Solyc10g049890.1.1	Phosphoglycerate deh	15.5	15.17	9.14	12.53	13.09	2.06	4.87	2.31	3.08	0.24	0.000118
Solyc02g092600.2.1	Phototropic-responsive	6.34	11.54	16.65	10.19	11.18	23.97	24.88	47.75	32.2	2.88	0.000118
Solyc02g079790.2.1	POT family domain co	24.37	32.54	19.83	31.5	27.06	11.54	2.4	0.42	4.79	0.18	0.00012
Solyc08g066940.2.1	Peptide transporter 1 (	25.93	32.67	48	22.69	32.32	3.94	7.84	3.44	5.07	0.16	0.00012
Solyc10g039290.1.1	Peptide transporter-lik	4.03	5.64	2.99	1.47	3.53	0.19	0.09	0.23	0.17	0.05	0.00012
Solyc07g045210.1.1	Unknown Protein (AH	63.26	65.66	65.95	75.71	67.64	16.64	22.04	18.08	18.92	0.28	0.000121
Solyc02g091470.2.1	Serine/threonine prote	3.57	2.95	1.65	3.06	2.81	0.31	0.21	0.25	0.26	0.09	0.000122
Solyc02g091950.2.1	Lysosomal Pro-X carb	7.95	11.02	6.15	7.56	8.17	1.05	2.23	1.94	1.74	0.21	0.000124
Solyc03g116460.2.1	Aspartate racemase (A	65.74	70.12	78.66	76.83	72.84	132.59	161.27	154.8	149.55	2.05	0.000126
Solyc01g068560.2.1	Unknown Protein (AH	320.43	364.87	441.97	269.95	349.31	102.79	67.2	56.13	75.37	0.22	0.000127
Solyc11g011610.1.1	Fatty acid-binding prot	54.38	48.04	75.47	51.47	57.34	12.6	16.29	3.57	10.82	0.19	0.000128
Solyc04g071820.2.1	Cytochrome P450	3	4.19	1.7	3.14	3.01	0.21	0.32	0.13	0.22	0.07	0.000129
Solyc12g089300.1.1	Gibberellin-regulated p	6.42	18.71	7.58	17.34	12.51	186.78	349.66	108.36	214.93	17.18	0.00013
Solyc01g111630.2.1	Glyoxylate/hydroxypy	247.18	238.95	200.71	125.14	203	56.47	65.98	36.38	52.94	0.26	0.00013
Solyc04g015100.2.1	Acetyl-CoA C-acetyltr	36.19	39.05	41.18	27.41	35.96	129.75	97.08	127.47	118.1	3.28	0.00013
Solyc08g075970.2.1	Transmembrane protei	13.67	21.78	13.59	13.57	15.65	91.29	70.96	59.53	73.93	4.72	0.000131
Solyc02g071040.2.1	Glycogen synthase (A	7.57	9.9	6.6	6.52	7.65	17.77	16.15	16.21	16.71	2.19	0.000131
Solyc11g005680.1.1	40S ribosomal protein	968.44	856.53	1038.27	1166.14	1007.35	282.52	271.95	313.72	289.4	0.29	0.000132
Solyc01g110960.2.1	Glutamic acid-rich pro	6.02	4.92	5.92	5.26	5.53	0.57	1.15	0.3	0.67	0.12	0.000132
Solyc05g008790.2.1	Ceramide kinase (AHR	7.09	11.16	7.33	10.03	8.9	20.17	19.19	26.3	21.89	2.46	0.000132
Solyc09g090910.1.1	Auxin responsive prot	6.76	7.05	4.13	5.94	5.97	0.57	0.76	0.23	0.52	0.09	0.000133
Solyc03g082420.2.1	Heat shock protein (A	4.19	2.99	2.64	5.54	3.84	180.9	406.37	194.23	260.5	67.84	0.000133
Solyc02g092140.1.1	Phloem protein (Fragm	2.68	2.67	2.79	2.46	2.65	20.91	20.83	19.43	20.39	7.69	0.000133

Solyc03g082560.2.1	Aldo/keto reductase-lil	19.03	18.99	16.36	18.36	18.18	2.87	4.93	2.12	3.31	0.18	0.000134
Solyc09g007360.2.1	Interactor of constituti	1.18	0.86	1.39	1.82	1.31	0	0	0	0	0.01	0.000135
Solyc10g007290.2.1	Phosphoenolpyruvate c	20.03	16	32.34	33.46	25.46	2.54	3.79	3.05	3.13	0.12	0.000135
Solyc08g077290.1.1	DnaJ homolog subfam	17.32	17.06	16.54	19.54	17.61	34.64	45.02	45.43	41.7	2.37	0.000136
Solyc12g010110.1.1	Formin 2B (AHRD V1	2.49	3.39	3.47	3.59	3.24	7.79	6.29	5.74	6.61	2.04	0.000136
Solyc10g076600.1.1	Acyl-CoA dehydrogena	20.39	23.35	20.87	20.78	21.35	59.36	60.6	54.02	57.99	2.72	0.000137
Solyc09g074510.2.1	Tubby-like F-box prote	43.87	51.47	53.91	55.47	51.18	109.15	96.14	104.41	103.23	2.02	0.000137
Solyc05g009630.2.1	Disease resistance prot	73.01	64.59	92.88	97.05	81.88	9.24	21.14	19.21	16.53	0.2	0.000138
Solyc06g050520.1.1	Ethylene-responsive tr	0.44	0.98	0	0	0.35	4.43	3.93	1.74	3.37	9.48	0.000142
Solyc10g084050.1.1	26S protease regulator	30.74	34.84	47.72	41.76	38.77	82.75	71.96	81.99	78.9	2.04	0.000143
Solyc12g008560.1.1	Unknown Protein (AH	1051.47	1091.64	1449.02	1754.57	1336.67	250.04	536.25	140.67	308.99	0.23	0.000143
Solyc01g067910.2.1	Unknown Protein (AH	0	2.26	5.99	3.59	2.96	266.49	153	181.34	200.28	67.66	0.000145
Solyc02g092470.2.1	Formin 3 (AHRD V1 *	15.42	14.65	18.36	13.63	15.52	2.87	5.68	1.18	3.24	0.21	0.000145
Solyc09g042700.2.1	Proactivator polypeptic	32.33	44.79	27.69	26.93	32.94	7.37	11.47	6.47	8.44	0.26	0.000146
Solyc03g122120.2.1	3-oxoacyl-(Acyl-carrie	20.77	19.54	26.73	38.39	26.36	44.62	50.39	66.65	53.89	2.04	0.000147
Solyc11g007770.1.1	Exostosin-2 (AHRD V	1.37	2.37	4.25	2.79	2.7	4.92	6.92	17.12	9.65	3.58	0.000147
Solyc07g006810.2.1	Adenosylmethionine-8	5.87	4.47	3.65	3.06	4.26	0.31	1.01	0.77	0.7	0.16	0.000147
Solyc11g010310.1.1	ATP dependent RNA h	3.56	4.58	8.35	8.1	6.15	10.28	11.53	16.62	12.81	2.08	0.000148
Solyc05g053600.2.1	ATP-binding cassette t	1.82	1.83	1.65	1.32	1.66	4.19	3.31	3.01	3.5	2.12	0.000148
Solyc10g077120.1.1	Photosystem II core co	508.82	431.92	541.93	449.7	483.09	113.68	202.73	113.2	143.2	0.3	0.00015
Solyc08g006640.2.1	Processive diacylglyce	47.03	54.09	64.4	47.97	53.37	11.1	16.54	9.71	12.45	0.23	0.000151
Solyc10g074950.1.1	Unknown Protein (AH	0.28	0.25	0	0	0.13	5.03	3.46	2.25	3.58	27.02	0.000151
Solyc04g007000.1.1	Ethylene-responsive tr	0.35	0.31	2.5	0.25	0.85	3.57	3.31	2.81	3.23	3.79	0.000152
Solyc10g007670.2.1	Unknown Protein (AH	116.06	106.19	40.36	52.99	78.9	2.63	7.63	2.02	4.09	0.05	0.000153
Solyc06g066640.2.1	Photosystem I reaction	37.05	22.52	13.33	19.81	23.18	1.83	2.58	2.69	2.37	0.1	0.000156
Solyc03g019760.2.1	ABC transporter G fan	12.8	16.68	7.77	4.72	10.49	24.29	28.66	16.3	23.08	2.2	0.000157
Solyc10g083190.1.1	Os04g0625000 protein	19.83	22.62	19.63	26.08	22.04	5.72	7.15	3.26	5.38	0.24	0.000157
Solyc01g094240.2.1	Short-chain dehydroge	3.31	4.6	4.15	7.61	4.92	0.17	0.67	0	0.28	0.06	0.000158
Solyc12g018990.1.1	Ribonucleoside hydrol	65.77	70.75	63.81	63.18	65.88	147.98	128.88	128.73	135.2	2.05	0.000158
Solyc04g056590.2.1	Aspartyl protease fami	9.68	12.49	9.01	11.38	10.64	30.72	24.66	35.18	30.19	2.84	0.000159
Solyc03g033840.2.1	26S protease regulator	7.04	2.9	5.69	4.24	4.97	0.53	0.42	0.26	0.4	0.08	0.000162
Solyc08g079500.2.1	AT2G45380 protein (F	81.12	76.99	66.82	66.03	72.74	143.47	152.14	164.94	153.52	2.11	0.000163
Solyc06g082600.2.1	Ubiquitin-conjugating	276.72	273.08	183.87	222.33	239	468.6	596.02	432.45	499.02	2.09	0.000163
Solyc06g073540.2.1	Argonaute 4-like prote	69.29	74.2	89.33	78.61	77.86	16.33	24.76	19.56	20.22	0.26	0.000163
Solyc10g008410.1.1	RING finger protein 5	26.08	37.89	17.65	76.5	39.53	0	0	0.76	0.25	0.01	0.000163
Solyc03g034400.2.1	Multidrug resistance pi	5.32	5.36	2.31	1.58	3.64	0	0.11	0.14	0.08	0.02	0.000163
Solyc02g068350.1.1	Uroporphyrinogen-III s	9.27	7.88	4.97	7.75	7.47	1.36	1.03	0.84	1.08	0.14	0.000165
Solyc12g056950.1.1	Genomic DNA chromo	28.95	40.13	47.04	40.28	39.1	85.89	98.62	86.21	90.24	2.31	0.000165
Solyc01g110910.2.1	Unknown Protein (AH	4.3	11.49	0	4.06	4.96	32.44	36.23	29.89	32.85	6.62	0.000165
Solyc04g082420.2.1	BTB/POZ domain-con	9.66	19.32	11.73	11.89	13.15	24.22	33.1	25.03	27.45	2.09	0.000165
Solyc01g087240.2.1	Nuclear transcription f	85.07	72.04	98.83	48.5	76.11	19.11	24.23	16.8	20.05	0.26	0.000168
Solyc07g008430.1.1	Unknown Protein (AH	264.85	300.48	40.06	144.09	187.37	4.7	3.95	2.89	3.85	0.02	0.000169
Solyc10g079850.1.1	Genomic DNA chromo	5.83	8.41	8.26	7.92	7.61	15.46	16.02	16.96	16.15	2.12	0.000169
Solyc01g095590.2.1	Extra-large G-protein-l	15.4	16.07	23.08	23.81	19.59	4.46	5.28	4.19	4.64	0.24	0.00017
Solyc08g076320.2.1	Cellulose synthase-like	15.57	17	19.8	30.2	20.64	34.13	46.97	67.09	49.4	2.39	0.000171
Solyc07g008840.2.1	TBC1 domain family r	53.23	58.5	81.43	77.1	67.56	17.42	20.31	26.81	21.51	0.32	0.000171
Solyc09g065020.2.1	F-box family protein (/	5.9	6.57	6.71	5.96	6.29	25.37	23.36	25.11	24.61	3.92	0.000171
Solyc11g072160.1.1	Ubiquitin-associated /T	6.55	5.91	8.9	10.28	7.91	17.48	15.94	20.94	18.12	2.29	0.000172
Solyc08g082980.2.1	Serine/threonine protei	1.91	3.7	2.78	1.27	2.42	36.32	20.33	24.65	27.1	11.22	0.000174
Solyc09g063010.2.1	BHLH transcription fac	8.51	4.1	8.23	5.18	6.5	12.32	17.53	10.33	13.39	2.06	0.000174
Solyc08g062450.1.1	class II heat shock prot	4.56	5.17	4.9	2.93	4.39	51.29	239.37	78.66	123.11	28.04	0.000174
Solyc04g080290.2.1	CRAL/TRIO domain c	96.21	83.99	155.39	199.59	133.79	9.99	12.32	7.05	9.79	0.07	0.000176
Solyc06g035960.2.1	4-coumarate-CoA ligas	5.43	6.52	4.03	5.54	5.38	24.51	12.26	51.31	29.36	5.46	0.000177
Solyc03g114860.2.1	Alpha-1 4-glucan-prote	23.24	20.52	22.77	25.98	23.13	1.06	6.72	2.42	3.4	0.15	0.000178
Solyc02g079950.2.1	Oxygen-evolving enha	754.53	618.4	628.38	622.51	655.95	195.66	275.54	230.46	233.89	0.36	0.000179
Solyc11g008780.1.1	Acetolactate synthase s	6.31	4.78	7.93	7.98	6.75	0.43	1.75	0.4	0.86	0.13	0.00018
Solyc05g009310.2.1	Zinc finger protein CO	1.36	3.75	1.78	3.83	2.68	0	0.12	0	0.04	0.01	0.00018
Solyc05g015490.2.1	Non-specific lipid-tran	8.11	5.65	4.58	2.99	5.33	95.77	61.23	57.43	71.48	13.4	0.000183
Solyc07g052470.2.1	Syntaxin (AHRD V1 *	32.55	43.17	34.59	24.4	33.68	338.78	273.71	325.49	312.66	9.28	0.000184
Solyc06g009770.1.1	Binding protein (AHRI	163.51	177.82	46.14	35.99	105.86	1.9	0.77	1.4	1.36	0.01	0.000184
Solyc08g066500.2.1	Class III homeodomain	0.8	1.91	1.03	1.13	1.22	0.06	0	0	0.02	0.02	0.000184
Solyc08g048450.2.1	Ornithine-oxo-acid trai	27.26	26.46	27.22	22.78	25.93	57.93	48.71	58.72	55.12	2.13	0.000185
Solyc10g083610.1.1	Protein kinase (AHRD	10.17	12.31	15.85	13.49	12.96	26.14	25.84	31.01	27.66	2.14	0.000186
Solyc02g085700.1.1	Geranylgeranyl pyroph	26.13	22.77	19.4	20.68	22.24	39.86	59.86	40.81	46.84	2.11	0.000189
Solyc09g008140.2.1	Membrane magnesium	84.02	71.45	82.24	92.44	82.54	181.01	184.09	179.77	181.62	2.2	0.00019
Solyc05g013570.2.1	AT1G67900-like prote	2.92	3.08	3.57	1.68	2.81	0.35	0.35	0.21	0.3	0.11	0.00019
Solyc09g089950.1.1	Unknown Protein (AH	14.96	18.76	47.34	13.47	23.63	0	0	0	0	0	0.00019
Solyc08g076060.2.1	Serine/threonine kinase	26.56	30.67	31.24	31.19	29.91	5.33	9.34	3.62	6.1	0.2	0.000191
Solyc08g079060.2.1	Trehalose 6-phosphate	1.7	2.12	2.82	2.89	2.38	0	0.14	0	0.05	0.02	0.000191
Solyc08g005630.2.1	Glucose-methanol-cho	0	0.68	1	1.69	0.84	366.56	162.45	495.55	341.52	405.36	0.000191
Solyc01g008070.2.1	Alpha/beta superfamily	49.21	45.02	56.44	43.32	48.5	99.21	103.79	120.53	107.84	2.22	0.000191
Solyc10g085240.1.1	UDP-glucosyltransfera	4.05	4.23	6.76	7.51	5.64	328.49	217.99	331.57	292.68	51.92	0.000191
Solyc02g070040.1.1	Ethylene-responsive nu	6.75	5.67	5.01	9.55	6.75	0.47	0.47	0	0.31	0.05	0.000192
Solyc03g059260.2.1	Carboxyl-terminal-proc	38.68	55.88	55.01	39.35	47.23	14.45	12.52	8.68	11.88	0.25	0.000193
Solyc08g075760.2.1	CYCLOPS (AHRD V1	10.03	14.06	7.84	8.52	10.11	2.43	2.56	1.62	2.2	0.22	0.000193
Solyc09g005120.2.1	Chaperone protein Dna	2.18	1.84	1.9	2.76	2.17	5.84	17.86	24.94	16.21	7.47	0.000193
Solyc04g078590.2.1	Receptor like kinase, F	1.87	1.49	0.81	1.12	1.32	0	0.08	0	0.03	0.02	0.000193
Solyc03g111940.2.1	Cytochrome P450	7.62	6.43	2.79	8.55	6.35	0.32	0.86	0.39	0.52	0.08	0.000195
Solyc08g082120.2.1	Methanol inducible pro	81.28	60.59	34.56	45.91	55.59	7.67	10.32	6.02	8	0.14	0.000196
Solyc08g079790.1.1	Organic anion transpor	11.64	10.69	6.76	10.98	10.02	3.43	1.36	0.55	1.78	0.18	0.000196
Solyc01g006670.2.1	UDP-glucosyltransfera	1.9	2.65	1.6	1.53	1.92	6.57	6.73	4.3	5.87	3.06	0.000197
Solyc02g079490.2.1	Hydroxycinnamoyl-Co	0.14	0.38	0	0.2	0.18	3.6	5.97	7.85	5.81	32.26	0.000197

Solyc04g071970.2.1	Light stress-responsive	118.62	122.51	116.75	134.14	123	31.01	40.32	15.74	29.02	0.24	0.000199
Solyc08g059710.2.1	Genomic DNA chromo	0	0	0.14	0.33	0.12	1.14	4.81	1.99	2.65	22.52	0.000199
Solyc12g009220.1.1	Jasmonate ZIM-domai	13.3	10.91	22.15	19.18	16.38	0.21	2.97	1.29	1.49	0.09	0.0002
Solyc09g089730.2.1	1-aminocyclopropane-	5.12	4.55	6.24	0.75	4.17	38.91	33.49	30.99	34.46	8.27	0.000201
Solyc01g081310.2.1	Glutathione-S-transfera	54.29	54.3	31	22.3	40.47	389.13	344.43	156.75	296.77	7.33	0.000201
Solyc10g009380.2.1	Unknown Protein (AH	88.67	164.78	66.95	50.17	92.64	4.58	4.62	4.93	4.71	0.05	0.000202
Solyc02g087740.2.1	Cupin RmlC-type (AH	55.95	51.54	49.29	80.53	59.33	119.82	96.76	280.36	165.65	2.79	0.000202
Solyc02g068060.2.1	Receptor like kinase, R	1.63	1.53	1.21	1.45	1.46	0.14	0.07	0.09	0.1	0.07	0.000202
Solyc05g015050.2.1	Os12g0236050 protein	30.24	34.31	43.21	39.7	36.86	7.85	10.31	9.77	9.31	0.25	0.000204
Solyc07g062310.2.1	Os03g0825600 protein	17.75	20.67	43.28	22.18	25.97	2.75	2.17	3.96	2.96	0.11	0.000204
Solyc06g074970.1.1	ABC transporter G fam	4.38	6.72	6.71	6.04	5.96	0.71	1.25	0.54	0.83	0.14	0.000204
Solyc03g114150.2.1	Aldehyde dehydrogena	56.49	53.89	54.89	51.53	54.2	8.75	19.36	5.99	11.37	0.21	0.000204
Solyc09g075560.1.1	Glycosyltransferase fa	8.6	9.73	14.8	15.72	12.21	1.88	3.21	0.53	1.87	0.15	0.000205
Solyc01g091000.2.1	Zinc ion binding prote	2.89	2.57	4.65	1.86	2.99	0	0	0	0	0	0.000205
Solyc01g005010.2.1	Protein XRI1 (AHRD V	13.4	18.25	16.12	10.04	14.45	2.97	2.14	1.57	2.23	0.15	0.000206
Solyc04g074420.1.1	Phi-1 protein (Fragmen	18.33	18.27	12.97	33.18	20.69	0	2.99	3.64	2.21	0.11	0.000207
Solyc02g023950.2.1	Receptor like kinase, R	1.38	2.01	2.15	1.95	1.87	0.1	0.2	0	0.1	0.05	0.000207
Solyc05g054370.2.1	Acyl-CoA dehydrogena	79.4	83.12	128.94	111.75	100.8	18.82	28.82	22.33	23.32	0.23	0.000208
Solyc03g111720.2.1	Peptide methionine sul	109.84	92.09	148.95	127.42	119.58	8999.88	3189.43	9119.64	7102.98	59.4	0.000208
Solyc12g044240.1.1	Dynein light chain 1 cy	92.36	109.39	102.41	81.53	96.42	29.73	15.84	26.88	24.15	0.25	0.000208
Solyc02g064950.2.1	CBS domain-containin	37.02	30.22	33.5	41.74	35.62	64.09	87.33	103.06	84.83	2.38	0.00021
Solyc04g054930.2.1	Trehalose-6-phosphate	21.16	31.39	19.58	17.02	22.29	5.53	7.44	0.62	4.53	0.2	0.00021
Solyc03g026120.2.1	Dehydration-responsiv	3.55	2.98	1.43	7.45	3.85	0	0	0	0	0	0.000211
Solyc05g014510.2.1	Ankyrin repeat domain	3.6	3.38	1.53	1.7	2.55	0.24	0.24	0.5	0.33	0.13	0.000212
Solyc05g025690.1.1	Respiratory burst oxida	0.54	0.24	0	0	0.2	3.95	1.55	6.2	3.9	20	0.000214
Solyc11g010450.1.1	Phosphofructokinase fa	32.18	30.07	37.38	34.49	33.53	86.2	76.05	67.94	76.73	2.29	0.000215
Solyc01g111660.2.1	Aquaporin-like protein	8.83	13.64	7.67	8.21	9.59	2.25	1.7	0.23	1.39	0.15	0.000215
Solyc02g092870.1.1	Os12g0236050 protein	1.49	1.58	1.42	0.8	1.32	8.31	5.66	10.07	8.01	6.06	0.000218
Solyc08g016620.2.1	Os11g0198100 protein	2.03	3.36	1.2	1.23	1.96	0.12	0	0	0.04	0.02	0.000221
Solyc03g019710.2.1	MADS-box transcripti	1.48	0.33	1.31	2.62	1.44	7.79	6.95	4.41	6.38	4.45	0.000222
Solyc08g075810.2.1	Acyl-CoA synthetase/A	5.09	4.95	7.41	4.86	5.58	14.45	13.8	13.64	13.96	2.5	0.000223
Solyc02g078400.2.1	Allantoinase (AHRD V	4.06	6.64	16.37	3.33	7.6	181.36	113.49	320.65	205.17	27	0.000223
Solyc04g078450.2.1	Unknown Protein (AH	0	1.63	2.16	0	0.95	329.41	152.31	125.51	202.41	213.63	0.000224
Solyc12g044290.1.1	Acetyl-coenzyme A sy	137.49	146.29	72.27	83.51	109.89	20.15	29.14	18.43	22.57	0.21	0.000225
Solyc08g068420.2.1	FAD-linked oxidoredu	4.28	6.51	3.98	7.96	5.68	0.64	1.01	0.45	0.7	0.12	0.000226
Solyc10g076550.1.1	Receptor-like protein k	6.74	6.14	7.39	13.17	8.36	0.52	0.13	0.48	0.38	0.05	0.000227
Solyc02g061760.2.1	Mate efflux family pro	5.26	6.17	3.95	3.21	4.65	0.68	0.88	0.12	0.56	0.12	0.000227
Solyc01g111740.2.1	Bzip-like transcription	5.06	4.66	5.12	4.22	4.76	0.51	0.81	0.36	0.56	0.12	0.000227
Solyc02g078470.2.1	Unknown Protein (AH	41.7	55.94	47.73	42.26	46.91	125.7	126.28	116.76	122.91	2.62	0.000227
Solyc01g105450.2.1	ABC transporter G fam	1.79	3.09	2.48	1.93	2.32	0.34	0.17	0.1	0.2	0.09	0.000229
Solyc05g009760.1.1	Nbs-lrr, resistance prot	3.93	5.77	4.96	4.52	4.79	0.59	0.6	1.27	0.82	0.17	0.000229
Solyc01g108620.2.1	Os11g0614900 protein	13.16	17.79	17.06	15.86	15.97	3.54	2.65	4.84	3.68	0.23	0.000232
Solyc07g055180.2.1	Receptor protein kinas	9.17	12.09	12.55	14.59	12.1	2.12	2.53	1.62	2.09	0.17	0.000233
Solyc04g007910.2.1	Glucan endo-1 3-beta-g	4.95	5.34	5.23	4.79	5.08	0.63	1.27	0.39	0.76	0.15	0.000233
Solyc06g068990.2.1	Mitogen-activated prot	0.11	0.2	0.13	0.16	0.15	1.86	0.89	4.46	2.4	16.02	0.000237
Solyc04g070980.2.1	Lanosterol synthase (A	26.96	30.86	23.32	48.07	32.3	2.17	7.35	1.2	3.57	0.11	0.000239
Solyc02g080520.2.1	Os04g0625000 protein	3.12	3.52	4.79	4.71	4.04	8.49	11.87	8.88	9.75	2.42	0.000239
Solyc02g077430.2.1	Lipase-like (AHRD V1	16.86	8.53	14.63	1.64	10.42	0	0.13	0	0.04	0	0.000239
Solyc06g060300.1.1	Unknown Protein (AH	9.96	8.86	11.75	8.8	9.84	24.14	43.11	50.04	39.1	3.97	0.00024
Solyc03g120420.2.1	Unknown Protein (AH	811.2	662.16	368.98	314.51	539.21	2091.47	3943.22	1373.91	2469.53	4.58	0.00024
Solyc11g072990.1.1	Fatty acid elongase 3-k	12.01	18.1	7	6.59	10.93	1.37	0.35	0.14	0.62	0.06	0.00024
Solyc01g106470.2.1	Genomic DNA chromo	39.86	43.08	35.67	32.62	37.81	106.6	103.41	108.38	106.13	2.81	0.000241
Solyc05g056620.1.1	MADS-MC (AHRD V	30.16	20.61	33.04	30.09	28.48	542.12	319.47	421.76	427.78	15.02	0.000242
Solyc01g014560.2.1	D-mannose binding lec	0	0	0	0	0	22.86	12.57	10.1	15.18	1517.67	0.000242
Solyc01g097190.2.1	Ankyrin repeat protein	58.17	46.72	46.03	37.93	47.21	80.81	105.09	106.74	97.55	2.07	0.000243
Solyc11g010710.1.1	AP2-like ethylene-resp	0.36	0.54	0.43	1.03	0.59	17.15	8	12.88	12.68	21.49	0.000243
Solyc01g073770.2.1	PRLI-interacting factor	15.06	8.51	14.38	13.52	12.87	19.7	36.03	22.54	26.09	2.03	0.000243
Solyc07g008900.2.1	Subtilisin-like serine p	158.15	192.22	235.11	230.44	203.98	44.05	70.19	50.59	54.94	0.27	0.000244
Solyc03g115370.2.1	Diacylglycerol kinase	8.48	10.92	11.61	10.98	10.5	17.64	22.06	35.47	25.06	2.39	0.000244
Solyc12g010200.1.1	Glycosyltransferase (A	0.44	0.3	0.66	0	0.35	2.42	2.44	2.76	2.54	7.26	0.000244
Solyc09g008270.2.1	Unknown Protein (AH	8.96	15.95	7.61	10.14	10.67	2.03	1.46	0.36	1.28	0.12	0.000245
Solyc04g076140.2.1	Rho GTPase activating	2.9	2.38	2.07	2.59	2.48	0.12	0.56	0.3	0.33	0.13	0.000246
Solyc09g010510.2.1	Short-chain dehydroge	8.43	11.16	3.39	2.03	6.25	0	0	0	0	0	0.000247
Solyc06g084480.2.1	Serine protease (AHRI	9.64	7.71	6.39	6.9	7.66	1.75	2.12	0.75	1.54	0.2	0.000248
Solyc12g010320.1.1	Outer membrane lipop	295.81	327.03	215.92	177.1	253.96	4563.33	3672.28	2848.48	3694.7	14.55	0.000251
Solyc01g009870.2.1	Glycerophosphodiester	11.55	22.2	11.46	26.53	17.94	0.54	1.91	3.82	2.09	0.12	0.000252
Solyc01g102300.2.1	BHLH transcription fac	64.77	64.45	85.44	61.39	69.01	23.23	22.6	19.66	21.83	0.32	0.000252
Solyc09g007710.2.1	Tir-nbs-lrr, resistance p	1.48	1.86	1.52	0.7	1.39	0.05	0	0	0.02	0.01	0.000255
Solyc06g065340.1.1	Unknown Protein (AH	4.1	2.61	3.81	2.49	3.25	0	0	0.29	0.1	0.03	0.000255
Solyc07g042170.2.1	Jasmonate ZIM-domai	232.57	168.84	185.79	240.39	206.9	27.51	85.47	24.44	45.81	0.22	0.000256
Solyc09g010110.2.1	Chaperone protein dna	36.25	40.89	31.58	31.3	35.01	93.14	89.82	98.75	93.9	2.68	0.000257
Solyc01g067800.2.1	E3 ubiquitin-protein lig	63.4	57.65	61.66	56.47	59.79	10.66	19.55	12.59	14.27	0.24	0.000257
Solyc03g019780.2.1	40S ribosomal protein	1050.24	725.97	958.19	1052.38	946.7	244.21	344.3	258.21	282.24	0.3	0.000257
Solyc08g061020.1.1	Nitrate transporter (AH	2.63	3.9	1.29	1.55	2.34	0	0	0	0	0	0.000258
Solyc05g012560.1.1	RING zinc finger prote	23.9	25.48	24.67	16.9	22.74	41.23	56.6	45.96	47.93	2.11	0.000261
Solyc08g006900.2.1	Ribosomal protein L32	236.31	180.96	271.25	248.61	234.28	64.04	75.02	76.23	71.76	0.31	0.000261
Solyc01g005780.1.1	LRR receptor-like seri	2.32	2.21	4.66	3.03	3.05	0.28	0.28	0.43	0.33	0.11	0.000261
Solyc06g066240.2.1	Cytochrome P450	3.59	3.54	3.18	0.18	2.62	0	0	0	0	0	0.000264
Solyc08g005400.2.1	Cytosine-specific meth	8.95	8.24	4.77	7.98	7.48	1.93	2	1.76	1.9	0.25	0.000268
Solyc06g069370.2.1	Transcription factor pr	5.4	5.87	6.65	4.41	5.58	14.54	13	9.64	12.39	2.22	0.000268
Solyc04g008250.1.1	Acid phosphatase (AH	0	0	0	0	0	2.82	1.22	0.74	1.59	159.33	0.000269
Solyc06g007770.2.1	ER lumen retaining rec	8.49	10.79	8.87	7.89	9.01	0.98	2.37	2.41	1.92	0.21	0.000273

Solyc02g069580.2.1	UDP-glucose 4-epimer	31.41	26.27	30.72	32.82	30.3	42.97	63.4	101.51	69.29	2.29	0.000274
Solyc12g007180.1.1	Tesmin/TSO1-like CX	3.04	2.35	4.37	2.99	3.19	14.21	10.02	19.82	14.68	4.61	0.000274
Solyc01g102750.2.1	PHD finger family pro	53.3	50.49	53.86	45.16	50.7	117.9	88.38	124.94	110.41	2.18	0.000275
Solyc01g090680.2.1	Genomic DNA chromo	21.66	28.14	25.85	22.18	24.46	5.03	6.09	2.97	4.7	0.19	0.000281
Solyc08g005010.2.1	Unknown Protein (AH	34.12	32.08	33.24	25.25	31.17	6.93	7.13	5.73	6.6	0.21	0.000282
Solyc08g076250.1.1	Cytochrome P450	0.79	2.47	2.33	1.31	1.73	0	0.11	0	0.04	0.02	0.000282
Solyc06g061020.2.1	Baculoviral IAP repeat	66.57	68.44	74.08	51.44	65.13	549.68	351.21	302.36	401.08	6.16	0.000283
Solyc05g052260.2.1	Appr-1-p processing do	58.79	58.11	53.64	50.77	55.33	104.66	120.61	113.66	112.98	2.04	0.000284
Solyc12g019480.1.1	Formin 3 (AHRD V1 *	0.67	0.73	0.88	1.05	0.83	0	0	0	0	0.01	0.000286
Solyc06g050130.2.1	Alpha-galactosidase-lil	71.7	68.22	52.98	36.58	57.37	14.26	21.58	12.45	16.1	0.28	0.000287
Solyc01g109130.2.1	Proteasome assembly c	14.09	14.84	10.53	10.63	12.52	26.18	35.58	22.6	28.12	2.25	0.000288
Solyc01g109680.2.1	Unknown Protein (AH	18.25	23.74	29.81	33.75	26.39	57.27	74.92	44.58	58.92	2.23	0.000288
Solyc07g063900.2.1	RNA binding protein (	256.94	197.77	216.09	198.3	217.28	419.02	462.24	431.23	437.5	2.01	0.000288
Solyc08g081370.1.1	RING-H2 finger protei	24.64	27.23	14.53	35.98	25.59	5.14	4.21	1.18	3.51	0.14	0.000289
Solyc10g061970.1.1	60S ribosomal protein	17.02	13.26	12.55	16.05	14.72	1.15	1.73	0.7	1.19	0.08	0.000289
Solyc12g006460.1.1	Cytochrome P450	15.01	29.96	46.73	88.92	45.16	0.33	0.66	0.81	0.6	0.01	0.000289
Solyc08g023270.2.1	Unknown Protein (AH	0.21	0.75	0	0.3	0.32	3.25	3.79	5.67	4.24	13.45	0.00029
Solyc09g065910.1.1	Photosystem II reaction	944.04	769.07	562.36	584.87	715.09	226.71	265	197.37	229.69	0.32	0.00029
Solyc07g020800.2.1	Rac-like GTP-binding	8.75	4.17	3.32	4.42	5.16	0.51	0	0	0.17	0.03	0.000293
Solyc04g078190.2.1	Lipase-like protein (AH	7.4	6.39	5.56	13.33	8.17	0.36	0.73	0.45	0.51	0.06	0.000293
Solyc12g009060.1.1	Charged multivesicula	138.13	118.21	127.52	142.06	131.48	288.62	302.15	319.23	303.33	2.31	0.000294
Solyc05g054080.2.1	Ubiquitin-protein ligas	11.83	14.28	16.06	9.94	13.03	24.68	23.2	38.1	28.66	2.2	0.000297
Solyc10g083180.1.1	Xanthoxin dehydrogen	4.61	4.85	4.94	5.04	4.86	0.17	0.68	0.21	0.35	0.07	0.000298
Solyc04g051580.2.1	Mitochondrial carrier (	17.44	21.89	13.46	26.25	19.76	2.53	5.1	2	3.21	0.16	0.000299
Solyc01g057680.2.1	Hcr2-p4.1 (AHRD V1	1.05	0	0	1.48	0.63	13.54	12.8	10.39	12.24	19.36	0.000303
Solyc08g065850.1.1	Unknown Protein (AH	191.82	146.33	88.61	167.97	148.68	36.08	43.01	33.24	37.44	0.25	0.000304
Solyc01g068490.2.1	U-box domain-contain	0.69	0.34	0.81	0.54	0.59	2.35	1.56	2.13	2.01	3.38	0.000306
Solyc12g088190.1.1	Amino acid permease (	4.18	4.68	4.46	1.34	3.67	0.11	0.77	0.8	0.56	0.15	0.000306
Solyc01g079400.2.1	Os06g0207500 protein	13.15	18.52	10.77	9.12	12.89	2.56	4.56	1.45	2.86	0.22	0.000307
Solyc06g054090.1.1	Ribosomal-protein-alar	18.36	24.82	12.84	7.7	15.93	0.82	1.11	0.34	0.76	0.05	0.000307
Solyc03g119200.2.1	Mandelate racemase/m	10.85	12.13	10.9	9.12	10.75	0.71	4.42	0	1.71	0.16	0.000307
Solyc06g051260.2.1	Transcription factor bH	30.65	41.05	15.15	19.82	26.67	3.07	3.33	1.74	2.71	0.1	0.00031
Solyc03g114540.1.1	Strictosidine synthase	5.29	3.83	3	4.98	4.28	0.16	0.48	0	0.21	0.05	0.000313
Solyc03g123610.2.1	Alanine aminotransfera	35.65	35.4	39.24	41.35	37.91	70.25	73.33	90.51	78.03	2.06	0.000313
Solyc07g005020.2.1	DNA replication licens	12.25	9.15	8.96	6.58	9.23	16.47	18.5	21.12	18.7	2.02	0.000313
Solyc07g041490.1.1	Stress responsive alpha	93.54	89.17	66.51	89.49	84.68	26.05	32.7	21.26	26.67	0.31	0.000313
Solyc07g007250.2.1	Metallocarboxypeptida	1826.05	1100.51	229.5	355.32	877.85	1.37	2.07	5.04	2.83	0	0.000314
Solyc03g120560.2.1	Proton-dependent oligo	19.44	21.09	22.42	22.87	21.46	39.62	38.45	67.33	48.47	2.26	0.000315
Solyc05g045670.2.1	Glucose-6-phosphate/p	24.78	17.96	3.1	5.8	12.91	0	0	0	0	0	0.000316
Solyc02g067340.2.1	MYB transcription fac	10.01	13.8	4.63	10.83	9.82	0.48	0.64	0.39	0.5	0.05	0.000316
Solyc00g095760.1.1	1-aminocyclopropane-	0.4	0.53	0.93	0.28	0.54	4.63	7.56	3.92	5.37	10.04	0.000316
Solyc09g091060.2.1	Unknown Protein (AH	8.5	14.35	19.37	6.22	12.11	0.47	1.19	0.29	0.65	0.05	0.000317
Solyc04g008980.2.1	F-box/LRR-repeat prot	52.49	77.68	81.9	60.51	68.15	22.41	20.43	19.44	20.76	0.3	0.000318
Solyc11g072490.1.1	Interactor of constituti	43.57	51.21	54.8	79.94	57.38	16.6	16.66	16.83	16.7	0.29	0.000318
Solyc11g072600.1.1	AP2-like ethylene-resp	14.65	18.88	23.09	12.39	17.25	0.33	5.24	0.41	1.99	0.12	0.00032
Solyc12g038160.1.1	Lipase family protein (	17.57	18.18	34.8	21.74	23.07	3.39	4.36	3.22	3.66	0.16	0.00032
Solyc01g080210.2.1	Ribonucleoside-diphos	3.29	2.93	2.05	2.19	2.61	32.51	20.34	23.62	25.49	9.75	0.000323
Solyc06g067950.2.1	Acyl-protein thioestera	32.86	32.9	50.88	50.47	41.78	2.9	12.55	5.61	7.02	0.17	0.000324
Solyc10g085040.1.1	Soul heme-binding fan	93.68	97.17	62.27	41.53	73.66	792.55	814.43	649.91	752.3	10.21	0.000324
Solyc12g008800.1.1	MYB transcription fac	6.98	9.32	21	10.37	11.92	0.85	0.57	0.69	0.7	0.06	0.000324
Solyc04g051510.1.1	Receptor like kinase, R	12.83	15.5	22.04	24.72	18.77	1.94	3.74	2.01	2.56	0.14	0.000325
Solyc12g005060.1.1	ATP synthase gamma c	35.25	29.69	23.13	21.86	27.48	52.38	76.18	69.79	66.12	2.41	0.000327
Solyc01g099010.2.1	GDSL esterase/lipase A	0.18	0.16	0.41	0.25	0.25	6.38	3.43	2.61	4.14	16.56	0.000329
Solyc06g007760.2.1	Ycf54 protein (AHRD	183.29	167.37	114.81	121.18	146.66	36.18	68.35	40	48.18	0.33	0.000331
Solyc08g007010.2.1	FeS assembly protein S	17.24	17.4	18.26	15.55	17.11	32.68	46.35	40.42	39.82	2.33	0.000331
Solyc11g010120.1.1	Peroxidase 17 (AHRD	4.49	5.73	3.45	2.76	4.11	0.32	0.64	0	0.32	0.08	0.000331
Solyc02g090810.2.1	Calmodulin (AHRD V	85.49	94.15	63.56	61.29	76.12	20.81	32.45	10.07	21.11	0.28	0.000335
Solyc01g016410.2.1	RING finger protein 43	11.92	10.61	6.87	11.37	10.19	25.08	27.56	22.84	25.16	2.47	0.000338
Solyc10g008400.1.1	RING finger protein 5	72.51	109.49	143.74	166.46	123.05	12.96	26.64	19.47	19.69	0.16	0.000339
Solyc07g006340.2.1	GCN5-related N-acety	18.52	21.22	13.38	16.7	17.45	60.03	55.46	40.44	51.98	2.98	0.00034
Solyc09g007260.2.1	AP2-like ethylene-resp	3.91	2.58	4.95	3.27	3.68	0.12	0.12	0	0.08	0.02	0.000343
Solyc10g009290.1.1	BHLH transcription fa	15.18	12.73	20.49	1.65	12.51	0.12	0.24	0	0.12	0.01	0.000345
Solyc02g091330.2.1	C20orf4 homolog (AH	9.65	12.27	11.93	8.45	10.57	23.59	25.66	27.61	25.62	2.42	0.000347
Solyc06g008250.2.1	TBC1 domain family n	8.23	8.68	10.25	12.07	9.81	22.03	22.84	21.02	21.96	2.24	0.000347
Solyc08g062340.2.1	Class II small heat sho	0.85	2.63	1	1.79	1.57	42.02	111.99	61.28	71.76	45.78	0.000348
Solyc01g088600.2.1	PDE320 (AHRD V1 **	11.21	12.02	10.34	13.01	11.64	1.28	2.93	1.14	1.78	0.15	0.000348
Solyc08g079830.2.1	Cu/Zn-superoxide dism	57.09	47.23	39.17	50.25	48.44	80.29	121.82	111.87	104.66	2.16	0.000348
Solyc05g006440.2.1	Homology to unknown	115.62	102.9	74.29	64.57	89.34	27.73	31.77	16.87	25.46	0.28	0.000349
Solyc04g074340.2.1	UDP-glucuronosyltran	5.08	9.88	4.89	1.51	5.34	0	0	0	0	0	0.000349
Solyc08g081850.1.1	Unknown Protein (AH	0.43	0	0.25	0	0.17	5.17	2.78	3.39	3.78	22.24	0.00035
Solyc07g056540.2.1	L-lactate dehydrogenas	50.19	44.19	63.6	28.35	46.58	2.87	10.26	6.16	6.43	0.14	0.000351
Solyc03g118110.2.1	Succinate dehydrogena	140.14	132.03	143.46	156.37	143	589.74	465.22	567.39	540.78	3.78	0.000354
Solyc11g066620.1.1	Aspartyl protease fami	9.63	10.1	17.12	25.57	15.61	97.43	48.92	202.99	116.45	7.46	0.000354
Solyc03g006610.2.1	Arf-GAP with GTPase	1.41	1.13	1	1	1.14	4.68	2.65	13.6	6.98	6.15	0.000355
Solyc03g093340.2.1	Genomic DNA chromo	0.18	0.82	0.87	1.31	0.8	2.54	4.07	1.47	2.69	3.39	0.000355
Solyc04g015690.2.1	Insulin degrading enzy	11.15	14.38	14.51	11.76	12.95	3.33	3.75	2.42	3.17	0.24	0.000356
Solyc07g055160.2.1	Lipase (Fragment) (AH	16.42	26.23	11.1	16.48	17.56	3.18	3.09	2.79	3.02	0.17	0.000358
Solyc06g083080.2.1	Unknown Protein (AH	12.59	11.59	10.75	10.44	11.34	0.7	2.83	0.43	1.32	0.12	0.000359
Solyc02g063160.2.1	Lipase-like protein (AH	8.66	6.68	6.35	5.44	6.78	18.34	17.08	11.84	15.75	2.32	0.000361
Solyc06g065950.2.1	Vesicle-associated mer	95.82	87.67	104.25	101.76	97.38	19.53	30.16	21.63	23.77	0.24	0.000361
Solyc03g118300.1.1	Unknown Protein (AH	27.85	42.4	29.4	40.44	35.02	120.22	64.51	121.51	102.08	2.91	0.000363
Solyc03g117690.2.1	Aspartic proteinase ne	16.32	25.23	11.1	15.06	16.93	3.24	3.94	1.24	2.81	0.17	0.000365

Solyc12g094720.1.1	PsbP domain-containin	9.14	8.8	7.29	6.64	7.97	1.6	1.61	1.23	1.48	0.19	0.000365
Solyc12g094380.1.1	DSBA oxidoreductase	27.27	42.27	28.71	12.86	27.78	84.09	80.04	35.21	66.45	2.39	0.000367
Solyc07g045340.2.1	RPM1 interacting prot	340	289.05	297.36	282.34	302.19	785.81	579.62	585.98	650.47	2.15	0.000367
Solyc07g062180.2.1	Predicted membrane p	13.74	13.03	12.58	14.06	13.35	27.01	31.96	39.65	32.87	2.46	0.000367
Solyc09g008260.2.1	Unknown Protein (AH	8.32	8.55	9.48	5.27	7.91	1.04	2.8	0.71	1.52	0.19	0.000368
Solyc05g008510.2.1	Multidrug resistance p	5.78	6.01	10.07	8.96	7.71	18.25	15.04	22.69	18.66	2.42	0.000368
Solyc10g008500.2.1	Mpv17 (AHRD V1 *--	22.38	25.69	27.8	18.61	23.62	60.28	49.28	44.04	51.2	2.17	0.000371
Solyc01g057310.2.1	Kinesin (Centromeric	1.23	1.22	1.92	1.68	1.51	0.11	0.25	0.04	0.13	0.09	0.000373
Solyc08g066430.1.1	Lachrymatory factor sy	0.77	0.68	0	1.09	0.64	8.37	12.5	8.38	9.75	15.35	0.000373
Solyc07g054430.2.1	Glutamate formiminot	7.7	5.74	12.03	7.36	8.21	1.85	1.7	0.21	1.25	0.15	0.000374
Solyc07g019650.2.1	Pentatricopeptide repe	6.42	5.71	5.47	3.53	5.28	1.44	1.53	0.88	1.28	0.24	0.000374
Solyc04g080250.2.1	Deoxyuridine 5&apos-	0	0	0.52	0	0.13	33.53	12.1	79.36	41.66	320.49	0.000377
Solyc08g061890.2.1	Retinoblastoma bindin	1.24	1.65	1.58	1.31	1.44	3.41	3.7	4.19	3.77	2.61	0.000377
Solyc06g048570.2.1	Hydrolase alpha/beta f	3.29	6.69	4.15	1.66	3.95	0.19	0	0	0.06	0.02	0.00038
Solyc09g083060.2.1	U-box domain-contain	35.8	26.34	39.25	44.47	36.47	4.82	3.83	7.29	5.31	0.15	0.00038
Solyc10g083940.1.1	Nodulin family protein	0.11	0.1	0.13	0.32	0.17	5.04	4.9	6.41	5.45	33.03	0.00038
Solyc04g040160.2.1	Pheophorbide a oxyger	19.13	24.24	20.99	9.59	18.49	3.91	5.03	0.84	3.26	0.18	0.000381
Solyc10g006900.2.1	Protochlorophyllide re	8	4.6	3.54	5.42	5.39	0.4	1.09	0	0.5	0.09	0.000382
Solyc02g093990.2.1	Adenylate kinase fami	5.33	2.71	2.99	8.25	4.82	0	0.21	0.25	0.15	0.03	0.000383
Solyc03g122040.2.1	COP1-Interacting Prot	26.6	28.63	28.39	44.71	32.08	4.05	9.03	4.55	5.88	0.18	0.000384
Solyc11g069320.1.1	Kelch repeat-containin	11.75	19.57	16.35	19.17	16.71	31.64	38.54	32.29	34.16	2.04	0.000384
Solyc03g006420.2.1	Origin recognition con	13.32	12.84	13.28	12.9	13.08	2.11	4.52	2.05	2.89	0.22	0.000384
Solyc11g013100.1.1	Unknown Protein (AH	353.56	399.07	306.46	361.6	355.17	1084.29	1157.47	887.08	1042.95	2.94	0.000385
Solyc03g007970.1.1	UDP-N-acetylmuramat	2.98	3.08	5.49	6.41	4.49	0.39	0.39	0.83	0.54	0.12	0.000388
Solyc06g071280.2.1	Enhanced disease susc	40.7	26.48	25.81	50.76	35.94	1.59	2.23	4.67	2.83	0.08	0.000388
Solyc03g098560.1.1	Unknown Protein (AH	0.31	0.27	0	0	0.15	3.97	3	0.61	2.53	17.43	0.000393
Solyc03g005030.2.1	Unknown Protein (AH	18.99	17.47	15.95	16.84	17.31	232.69	127.97	151.09	170.58	9.85	0.000394
Solyc07g018010.2.1	Transcription Factor (	24.41	34.9	31.87	16.53	26.93	0.42	3.6	0.77	1.6	0.06	0.000394
Solyc10g081900.1.1	Digalactosyldiacylglyc	14.7	14.18	20.85	15.3	16.26	25.15	30.34	48.35	34.61	2.13	0.000394
Solyc12g038350.1.1	Unknown Protein (AH	3.15	3.21	6.11	3.5	3.99	0.36	0.37	0	0.24	0.06	0.000394
Solyc01g073640.2.1	Uncharacterized oxido	199.69	346.86	403.83	1226.03	544.1	0.41	2.48	3.27	2.05	0	0.000395
Solyc06g063240.2.1	Os03g0169000 protein	280.58	339.01	240.19	288.2	287	79.62	87.31	73.69	80.21	0.28	0.000395
Solyc07g062060.2.1	Peptide methionine sul	64.2	66.59	74.18	61.38	66.59	389.9	443.41	330.93	388.08	5.83	0.000395
Solyc04g082940.2.1	CHP-rich zinc finger p	0.21	0	0.75	0.6	0.39	3.44	2.95	0.42	2.27	5.82	0.000395
Solyc04g009510.2.1	CCR4-NOT transcripti	16.28	20.79	22.53	21.49	20.27	4.97	7.6	4.67	5.75	0.28	0.000397
Solyc10g086060.1.1	IQ-domain 10 (Fragme	65.97	50.88	58.7	73.74	62.32	12.99	22.78	9.71	15.16	0.24	0.000399
Solyc08g006070.2.1	AIG2-like protein (AH	72.14	88.24	47.34	56.75	66.12	17.45	25.46	14.17	19.03	0.29	0.0004
Solyc12g011430.1.1	Hydroxyacylglutathion	10.65	12.77	7.37	7.2	9.5	25.43	25.84	15.39	22.22	2.34	0.0004
Solyc02g071210.2.1	Cold acclimation prote	32.54	43.71	20.94	16.74	28.48	4.3	3.13	2.06	3.16	0.11	0.000402
Solyc06g071380.2.1	Unknown Protein (AH	7.33	11.92	7.16	12.16	9.64	24.91	17.1	20.32	20.78	2.15	0.000403
Solyc10g085230.1.1	UDP-glucosyltransfera	20.1	32.19	30.41	21.37	26.02	1900.38	1155.56	1630.45	1562.13	60.04	0.000403
Solyc02g062310.1.1	BURP domain-contain	3.47	9.27	9.95	2.81	6.37	0	0	0	0	0	0.000407
Solyc03g117890.2.1	Unknown protein DS1	127.36	149.84	95.4	73.88	111.62	333.3	268.41	199.63	267.11	2.39	0.000408
Solyc03g114960.2.1	Kelch-like protein 20 (	17.86	16	14.05	17.87	16.45	4.91	5.54	4.22	4.89	0.3	0.000411
Solyc12g009880.1.1	Serine-rich protein (Al	31.59	41.63	63.21	29.97	41.6	6.14	8.15	4.76	6.35	0.15	0.000413
Solyc04g072030.1.1	Interferon-induced GT	17.86	15.72	14.85	13.25	15.42	4.97	3.98	5.62	4.86	0.31	0.000415
Solyc11g012790.1.1	SEC14-like protein 1 (	44.37	47.63	42.7	35.78	42.62	14.78	13	9.27	12.35	0.29	0.000421
Solyc03g116190.1.1	Chitinase (AHRD V1 -	0	0	0	0	0	16.9	13.11	10.65	13.55	1355.33	0.000422
Solyc09g082470.2.1	Kinase-like protein (A	12.66	14.23	18.08	14.14	14.78	24.63	33.67	34.39	30.9	2.09	0.000423
Solyc02g079360.1.1	Octicosapeptide/Phox/	4.1	3.65	5.44	2.9	4.02	0.14	0.7	0.17	0.34	0.08	0.000425
Solyc03g118240.2.1	Magnesium-protoporpl	56.72	45.47	47.93	51.21	50.33	14.14	19.5	16.77	16.8	0.33	0.000425
Solyc07g056580.2.1	Ethylene receptor (AH	3.31	7.17	7.18	3.67	5.33	9.91	12.47	17.5	13.29	2.49	0.000427
Solyc11g066020.1.1	Ankyrin repeat domair	66.86	55.56	100.12	111.69	83.56	7.75	19.6	10.4	12.58	0.15	0.00043
Solyc01g103170.2.1	Lysine ketoglutarate re	5.23	5.47	8.16	5.22	6.02	21.24	11.15	34.04	22.14	3.68	0.00043
Solyc02g065570.1.1	DVL1 (AHRD V1 **--	65.97	67.92	12.21	31.11	44.3	0	2.11	0	0.7	0.02	0.00043
Solyc02g094390.2.1	Palmitoyltransferase-li	65.82	70.24	66.33	79.52	70.48	14.16	24.95	8.92	16.01	0.23	0.00043
Solyc05g053300.2.1	Dihydrolipoyl dehydro	42.27	39.14	43.19	56.44	45.26	84.04	89.49	101.16	91.56	2.02	0.000433
Solyc08g080080.2.1	WD-repeat cell cycle r	6.57	7.92	5.01	6.78	6.57	10.84	16.95	17.12	14.97	2.28	0.000434
Solyc09g008170.1.1	Auxin responsive SAU	0	0.47	0.63	1.51	0.65	586.71	178.18	733.51	499.47	765.47	0.000434
Solyc08g067410.1.1	Fatty acid elongase 3-k	12.78	21.12	19.69	16.41	17.5	0.53	2.76	0.52	1.27	0.07	0.000437
Solyc08g005000.2.1	Glucan endo-1 3-beta-g	7.36	6.55	7.09	8.72	7.43	1.34	1.71	0.89	1.31	0.18	0.000439
Solyc08g066250.2.1	Decarboxylase family	0.16	0.15	0.58	3.01	0.97	1259.46	328.55	1642.74	1076.92	1104.53	0.000439
Solyc06g076700.1.1	UPF0503 protein At3g	4.79	4.91	3.31	8.1	5.28	10.76	13.31	11.15	11.74	2.22	0.00044
Solyc05g054620.2.1	Amino acid binding pr	73.98	82.48	145.29	85.98	96.93	19.04	22.78	21.2	21.01	0.22	0.000444
Solyc01g090440.2.1	Unknown Protein (AH	2.82	4.88	3.33	2.85	3.47	0.33	1.12	0.08	0.51	0.15	0.000448
Solyc02g094450.2.1	Unknown Protein (AH	65.19	67.4	40.07	37	52.41	11.35	17.5	5.81	11.55	0.22	0.000451
Solyc01g008540.2.1	Cinnamoyl CoA reduc	0.61	0.72	1.2	0.29	0.71	7.24	12.61	4.85	8.23	11.68	0.000452
Solyc01g008510.2.1	Photosystem II 5 kDa	0	2.02	2.01	0	1.01	13.78	61.17	25.4	33.45	33.2	0.000452
Solyc03g096070.2.1	1-phosphatidylinositol-	3.65	2.26	3.17	4.74	3.46	0.6	0.73	0.22	0.52	0.15	0.000453
Solyc01g095500.2.1	Ethylene-responsive tr	4.04	5.79	6.35	7.3	5.87	0.18	0.73	0.22	0.38	0.06	0.000453
Solyc12g096860.1.1	Acyl-CoA thioesterase	2.69	2.52	1.23	1.9	2.08	0	0.12	0.15	0.09	0.04	0.000453
Solyc03g031920.2.1	Yellow stripe-like prot	14.01	12.72	22.33	11.78	15.21	44.73	25.91	70.65	47.1	3.1	0.000454
Solyc08g014010.2.1	Unknown Protein (AH	0.13	0	0	0	0.03	1.8	3.32	0.52	1.88	57.85	0.000454
Solyc12g011140.1.1	Subtilisin-like proteas	1.19	1.67	0.6	1.33	1.2	0	0.14	0	0.05	0.04	0.000456
Solyc09g011240.2.1	Reductase 2 (AHRD V	46.26	46.53	46.26	43.14	45.55	9.05	16.23	7.41	10.9	0.24	0.000456
Solyc01g010190.2.1	Unknown Protein (AH	38.61	42.48	42.32	47.3	42.68	14.25	10.4	8.75	11.13	0.26	0.000458
Solyc03g118100.2.1	Dehydrogenase/reduct	0	0	0	0	0	1.87	2.05	3.13	2.35	235	0.000458
Solyc09g074930.2.1	REF-like stress related	150.5	116	174.58	180.48	155.39	46.45	55.25	39.56	47.09	0.3	0.000458
Solyc04g082060.2.1	F-box family protein (	26.78	49.99	27.55	33.03	34.34	8.05	12.37	5.67	8.7	0.25	0.000458
Solyc05g053900.2.1	Aspartic proteinase ne	3.58	5.27	2.27	1.17	3.07	11.02	8.53	5.6	8.38	2.73	0.000461
Solyc06g075010.2.1	chaperonin (AHRD V1	65.52	52.05	70.84	65.48	63.47	13.37	27.24	12.76	17.79	0.28	0.000462
Solyc04g005420.2.1	Glucan endo-1 3-beta-	53.33	43.08	70.25	60.9	56.89	114.9	110.66	124.16	116.57	2.05	0.000463

Solyc01g110570.2.1	Auxin-induced SAUR-	7.47	8.86	4.41	4.4	6.29	0	0	0	0	0	0.000463
Solyc03g081320.2.1	Membrane related prot	31.89	31.17	27.06	24.66	28.7	10.03	10.11	7.95	9.36	0.33	0.000463
Solyc11g018590.1.1	Subtilase family protei	1.28	0.57	0.76	3.62	1.56	23.29	12	20.98	18.76	12.04	0.000464
Solyc04g054480.2.1	C2 domain-containing	74.93	74.03	155.66	158.93	115.89	3.57	10.54	3.98	6.03	0.05	0.000465
Solyc01g100200.2.1	GRAS family transcrip	5.44	2.2	3.03	2.24	3.23	7.27	6.93	9.92	8.04	2.49	0.000467
Solyc03g098230.2.1	Pyridoxal phosphate-de	61.63	66.98	53.72	44.25	56.65	131.62	115.34	113.9	120.29	2.12	0.000477
Solyc02g064980.1.1	Protein serine/threonin	0.18	0.16	0.65	1.56	0.64	4.29	4.03	6	4.77	7.49	0.000478
Solyc03g025270.2.1	rRNA 2&apos-O-meth	95.29	87.23	84.26	67.54	83.58	22.33	25.94	18.29	22.19	0.27	0.000486
Solyc03g113060.2.1	ATP-binding cassette t	8.24	8.64	5.6	7.11	7.4	1.07	1.93	1.11	1.37	0.19	0.000487
Solyc03g111100.1.1	AtIII18x5-like protein	0.42	0	1.5	1.2	0.78	8.23	6.91	9.05	8.06	10.34	0.000489
Solyc05g008500.1.1	Mate efflux family pro	0.43	0.39	0.51	0.61	0.48	2.68	2.82	2.72	2.74	5.65	0.00049
Solyc08g082630.2.1	Auxin response factor	1.23	1.27	1.69	0.87	1.26	0	0.08	0	0.03	0.02	0.000491
Solyc01g108560.2.1	Acetyl esterase (AHRD	5.24	5.83	4.9	7.11	5.77	69.36	34.71	119.69	74.59	12.93	0.000493
Solyc06g066290.2.1	At1g31940/F5M6.6 (A	2.81	0.83	2.76	0	1.6	10.21	6.48	0.93	5.87	3.67	0.000493
Solyc05g013480.2.1	ATP-dependent protea	43.79	46.11	34.3	37.17	40.34	101.48	101.74	93.41	98.88	2.45	0.000494
Solyc07g016180.2.1	Auxin response factor	12.86	13.82	12.87	21.21	15.19	4.98	3.72	3.06	3.92	0.26	0.000496
Solyc01g068000.2.1	Serine/threonine phosp	17.57	23.12	16.54	24.85	20.52	32.31	42.64	53.24	42.73	2.08	0.000497
Solyc09g009080.2.1	Repressor of silencing	13.52	12.76	13.67	17.42	14.34	2.44	5.78	1.73	3.32	0.23	0.000497
Solyc02g068140.2.1	Nodulin-like protein (A	11.38	10.62	20.6	3.91	11.63	0.36	0.18	0.22	0.25	0.02	0.000499
Solyc03g097230.1.1	Protein containing AIG	2.71	0.6	4.79	4.79	3.22	9.57	8.82	4.37	7.59	2.35	0.000502
Solyc07g039190.2.1	Unknown Protein (AH	10.7	10.26	9.59	5.97	9.13	1.33	2.6	1.12	1.68	0.18	0.000508
Solyc11g068830.1.1	Orotidine 5&apos-pho	171	141.26	156.64	168.87	159.44	50.06	59.83	59.32	56.4	0.35	0.000508
Solyc03g110970.2.1	GDP-mannose transpo	14.79	15.66	12.26	17.81	15.13	1.62	5.7	2.18	3.17	0.21	0.000509
Solyc02g081490.2.1	Receptor-like kinase (A	43.52	47.37	21.58	44.7	39.29	3.58	6.1	8.53	6.07	0.15	0.000509
Solyc04g051820.2.1	Os03g0169900 protein	185.25	177.92	146.23	194.17	175.89	48.99	61.36	94.59	68.31	0.39	0.000509
Solyc03g111200.2.1	Adenylate kinase (AHI	95.86	89.39	108.33	108.93	100.63	27.19	34.01	22.99	28.06	0.28	0.00051
Solyc10g081530.1.1	V-type proton ATPase	173.55	149.79	145.05	168.6	159.25	312.67	315.37	343.97	324	2.03	0.000513
Solyc03g005020.2.1	Lipase (AHRD V1 ***	0.65	2.02	1.15	2.07	1.47	628.42	219.27	396.12	414.6	281.56	0.000522
Solyc04g078920.2.1	Peroxisomal multifunc	269.64	269.34	148.87	148.12	208.99	964.87	884.01	575.83	808.24	3.87	0.000522
Solyc03g006840.2.1	Genomic DNA chromo	2.09	1.78	1.35	4.58	2.45	0.08	0	0	0.03	0.01	0.000527
Solyc11g012190.1.1	Mannan endo-1 4-beta	13.11	17.69	13.26	11	13.77	4.42	3.99	2.29	3.57	0.26	0.00053
Solyc09g066460.2.1	Serine/threonine-prote	37.33	33.99	47.36	21.12	34.95	6.63	10.37	5.59	7.53	0.22	0.000531
Solyc05g053490.2.1	Beta-1,3-galactosyl-O-	2.5	3.27	6.76	5.61	4.54	9.14	10.9	12.1	10.71	2.36	0.000532
Solyc03g082620.2.1	Oligopeptide transport	10.21	15.41	9.8	7.93	10.84	0.89	1.88	0.4	1.06	0.1	0.000538
Solyc12g020110.1.1	DNA repair protein rad	2.47	2.86	3.71	2.54	2.89	0.3	0.67	0.3	0.42	0.15	0.000542
Solyc09g007760.2.1	Aquaporin 2 (AHRD V	29.65	53.55	66.93	17.33	41.86	2.95	1.93	3.41	2.76	0.07	0.000545
Solyc05g054320.2.1	Epoxide hydrolase (AH	41.39	40.48	23.93	46.95	38.19	8.94	15.53	5.9	10.12	0.27	0.000548
Solyc06g072330.2.1	Mitochondrial import i	1.2	3.2	1.42	0.85	1.67	13.08	13.2	13.69	13.32	7.99	0.000549
Solyc09g031970.2.1	Phosphorylase (AHRD	66.6	55.77	48.95	54.23	56.39	13.48	23.16	14.22	16.95	0.3	0.000551
Solyc10g007050.2.1	Enolase-phosphatase E	11.93	8.69	7.04	8.69	9.09	1.46	2.36	1.08	1.63	0.18	0.000553
Solyc12g007230.1.1	Auxin responsive prote	19.39	20.83	12.36	16.71	17.32	4.14	4.18	3.98	4.1	0.24	0.000563
Solyc02g081500.2.1	Receptor-like kinase (A	11.63	8.82	4.48	10.56	8.87	0.4	0.8	1.94	1.05	0.12	0.000563
Solyc02g088500.1.1	C-glucosyltransferase (	10.54	11.79	10.09	6.65	9.77	1.04	3.02	0.85	1.64	0.17	0.000565
Solyc12g096340.1.1	UDP-sugar transporter	19.86	28.67	18.74	21.85	22.28	52.23	41.71	42.05	45.33	2.03	0.000565
Solyc02g068010.2.1	DNA-binding protein (	23.13	20.84	26.62	14.54	21.28	47.3	45.85	55.84	49.66	2.33	0.000566
Solyc03g117430.2.1	Cobalamin synthesis p	67.26	61.65	51.61	50.11	57.66	112.97	124.11	144.35	127.14	2.21	0.000566
Solyc01g097240.2.1	Pathogenesis-related p	21.07	17.12	44.86	10.37	23.36	161.75	228.84	123.69	171.43	7.34	0.000568
Solyc07g062140.2.1	Alpha alpha-trehalose-	41.85	39.46	30.48	30.9	35.67	9.43	11.37	12.57	11.12	0.31	0.000569
Solyc11g062370.1.1	Plectin-related protein-	22.58	26.1	21.5	18.21	22.1	4.8	8.72	8.26	7.26	0.33	0.000573
Solyc11g010730.1.1	Receptor-like kinase (A	5.49	6.74	5.86	4.62	5.68	1.27	1.17	0.26	0.9	0.16	0.000576
Solyc12g049620.1.1	Unknown Protein (AH	19.24	14.68	12.97	11.66	14.64	37.75	67.2	49.11	51.35	3.51	0.000576
Solyc06g007970.2.1	Male sterility 5 family	0	0	0	0.32	0.08	1.48	3.17	0.68	1.78	22.21	0.000576
Solyc03g117980.2.1	Respiratory burst oxida	23.54	22.7	18.57	49.09	28.48	0.4	1.77	1.46	1.21	0.04	0.00058
Solyc10g009550.2.1	WRKY transcription fa	7.03	4.04	3.48	8.66	5.8	0.37	0.55	0.45	0.46	0.08	0.000582
Solyc03g097190.2.1	Cingulin-like (AHRD V	61.41	61.44	69.81	92.93	71.4	17.64	21.45	21.13	20.07	0.28	0.000583
Solyc11g042640.1.1	Rieske (2Fe-2S) domai	37.15	37.31	45.8	31.67	37.98	9.81	12.23	6.86	9.63	0.25	0.000583
Solyc08g077880.2.1	Light harvesting-like p	211.26	209.57	127.97	115.68	166.12	41.52	49.73	30.9	40.72	0.25	0.000585
Solyc06g036250.1.1	Genomic DNA chromo	0	0.56	0.74	0.44	0.43	25.89	12.54	26.19	21.54	49.52	0.000585
Solyc09g082700.2.1	Early light-induced pro	35.67	23.21	33.03	11.94	25.96	1059.46	1047.02	438.58	848.35	32.68	0.000586
Solyc06g053740.2.1	Ubiquitin carboxyl-ter	54.58	51.67	44.05	28.42	44.68	118.48	113.06	81.97	104.5	2.34	0.000586
Solyc02g090340.2.1	Cytochrome P450	23.52	28.42	105.58	46.1	50.9	0	0.75	0.26	0.34	0.01	0.000588
Solyc12g042500.1.1	Gibberellin-regulated f	77.09	91.04	37.6	45.09	62.7	7.79	12.08	3.68	7.85	0.13	0.000594
Solyc05g055870.2.1	F-box protein PP2-B1 (	53.59	66.69	63.47	69.46	63.3	22.94	19.48	19.28	20.57	0.32	0.000594
Solyc04g005510.2.1	Eukaryotic translation	176.47	146.78	132.31	114.9	142.62	36.31	55.44	31.92	41.22	0.29	0.000596
Solyc04g007690.2.1	Auxin efflux carrier (A	2.79	2.96	1.9	6.38	3.51	0	0.18	0	0.06	0.02	0.000598
Solyc09g007770.2.1	Aquaporin 2 (AHRD V	34.25	62.33	75.74	19.38	47.92	3.48	2.27	3.53	3.09	0.06	0.0006
Solyc01g066460.2.1	Epoxide hydrolase (AH	0	0.3	0.4	0	0.17	12.03	8.55	1.34	7.31	41.75	0.000605
Solyc09g009940.2.1	Signal recognition part	48.67	59.33	50.56	50.27	52.21	12.76	20.25	14.06	15.69	0.3	0.000611
Solyc03g097630.2.1	Palmitoyltransferase-li	15.93	18.46	24.47	24.66	20.88	46.99	34.16	47.14	42.76	2.05	0.000612
Solyc07g056570.1.1	9-cis-epoxycarotenoid	62.49	60.65	113.67	227.44	116.06	294.9	282.8	797.56	458.42	3.95	0.000612
Solyc02g071490.2.1	1-aminocyclopropane-	7.47	6.97	7.1	7.99	7.38	22.82	30.74	14.29	22.62	3.06	0.000615
Solyc10g011810.2.1	Fatty acid delta-6 desat	3.73	3.65	3.08	3.95	3.6	0.45	0.3	0.74	0.5	0.14	0.00062
Solyc08g061240.1.1	Catalytic/ hydrolase (A	5.62	5.53	4.89	3.35	4.85	0.24	0.24	0.59	0.36	0.07	0.000622
Solyc01g107430.2.1	Zinc finger protein 7 (A	9.86	9.45	1.34	6.44	6.77	0	0	0.38	0.13	0.02	0.000623
Solyc11g005730.1.1	Histone-lysine N-meth	4.75	4.68	4.8	4.56	4.7	9.45	13.27	8.75	10.49	2.23	0.000629
Solyc03g119110.2.1	Uncharacterized GPI-a	10.16	14.37	13.64	8.18	11.59	2.09	3.41	0.79	2.1	0.18	0.000631
Solyc04g039980.2.1	Lrr, resistance protein	9.54	10.24	5.96	10.32	9.02	1.36	1.83	1.11	1.43	0.16	0.000632
Solyc03g026110.2.1	Calmodulin binding pr	40.37	30.02	40.11	37.36	36.97	10.94	10.27	6.86	9.36	0.25	0.000638
Solyc04g008970.2.1	FIP1 (AHRD V1 **-- I	43.21	45.64	37.05	12.63	34.63	7.22	2.35	5.72	5.1	0.15	0.000643
Solyc07g054740.2.1	Membrane associated	13.79	15.34	10.16	14.28	13.39	2.78	4.81	1.22	2.94	0.22	0.000643
Solyc06g075600.2.1	Ankyrin repeat family	2.34	2.3	1.45	1.22	1.83	0.2	0.1	0	0.1	0.05	0.000647
Solyc08g080390.2.1	Cytochrome P450	0.82	1.46	2.26	1.16	1.42	4.31	15.5	12.91	10.91	7.65	0.000651

Solyc01g107350.2.1	Os12g0581300 protein	6.86	11.47	6.11	14.66	9.78	1.24	1.83	0.42	1.16	0.12	0.000653
Solyc10g079790.1.1	Pathogen-induced caln	4.55	3.07	3.38	7.36	4.59	0.21	0.53	0.13	0.29	0.06	0.000654
Solyc09g091030.2.1	Beta-amylase (AHRD	54.84	47.7	45.82	50.75	49.78	152.19	110.48	199.38	154.02	3.09	0.000655
Solyc04g076100.2.1	Genomic DNA chromo	1.47	3.1	2.89	4	2.87	0.26	0.44	0.54	0.41	0.14	0.000658
Solyc05g025810.2.1	Strictosidine synthase	55.94	71.45	45.16	33.12	51.42	12.01	16.2	5.72	11.31	0.22	0.000661
Solyc12g013610.1.1	Calmodulin-binding pr	2.48	3.44	2.61	2.34	2.72	0	0	0	0	0	0.000663
Solyc08g082240.1.1	Unknown Protein (AH	32.28	29.98	22.36	21.84	26.62	54.43	77.21	40.4	57.35	2.15	0.00067
Solyc01g080680.2.1	Glucose transporter 8 (	11.53	21.78	16.26	4.38	13.49	0.68	1.15	1.4	1.08	0.08	0.000672
Solyc04g081850.2.1	UPF0436 protein C9or	34.6	33.14	47.74	50.6	41.52	7.82	13.14	11.64	10.87	0.26	0.000673
Solyc07g064690.1.1	Unknown Protein (AH	63.94	57.36	60.47	35.17	54.23	202.94	196.42	145.57	181.64	3.35	0.000675
Solyc06g082560.1.1	ClpB chaperone (AHR	0.31	0.89	0.73	1.09	0.76	2.93	6.66	11.79	7.13	9.44	0.000676
Solyc04g056640.1.1	LRR receptor-like seri	1.41	1.89	1.56	1.37	1.56	0	0.07	0.09	0.05	0.03	0.000678
Solyc02g066960.2.1	UDP-glycosyltransfera	1.24	2.71	0.49	1.96	1.6	0	0	0	0	0.01	0.000678
Solyc06g075650.2.1	Aquaporin (AHRD V1	3.9	5.55	4.9	0.37	3.68	0	0	0.26	0.09	0.02	0.000679
Solyc07g055100.2.1	Transcription factor (A	2.15	2.59	2.53	5.64	3.23	0.12	0.13	0.15	0.13	0.04	0.000679
Solyc03g111810.2.1	Sieve element-occludi	7.92	6.8	4.4	4.12	5.81	1.03	2.08	0	1.04	0.18	0.000682
Solyc09g011480.2.1	Rop guanine nucleotid	8.06	14.16	17.71	12.2	13.03	62.08	43.26	49.47	51.6	3.96	0.000683
Solyc05g053870.2.1	Copine I-like protein (	11.38	12.34	7.23	13.63	11.14	2.24	2.85	1.01	2.03	0.18	0.000684
Solyc07g054850.2.1	Uncharacterized GPI-a	26.47	30.37	60.26	43.88	40.24	4.51	9.44	2.26	5.4	0.13	0.000688
Solyc06g051970.2.1	Calcineurin B-like calc	5.52	4.92	3.62	4.34	4.6	0.5	0	0.3	0.27	0.06	0.000689
Solyc04g079350.1.1	Pheromone receptor-lil	6.22	9.05	6.61	4.4	6.57	13.58	19.78	11.33	14.9	2.27	0.000689
Solyc12g006320.1.1	ATP-dependent RNA h	162.98	142.04	197.12	161.56	165.93	34.93	57.1	58.16	50.06	0.3	0.000693
Solyc09g011080.2.1	Ribulose-1 5-bisphosp	26.84	25.89	17.51	11.45	20.42	3.39	6.23	1.79	3.8	0.19	0.000696
Solyc09g092110.2.1	Light regulated protein	4.57	7.68	2.39	5.02	4.92	0	0	0.5	0.17	0.03	0.000697
Solyc03g123760.2.1	Phytoene desaturase	44.85	43.63	44.51	36.91	42.48	88.53	74.84	110.22	91.2	2.15	0.000705
Solyc08g007860.2.1	Lysophospholipid acyl	28.44	31.38	35.56	35.4	32.7	78.22	59.2	91.59	76.34	2.33	0.000705
Solyc09g007790.1.1	Arabidopsis thaliana g	65.56	83.82	99.37	42.26	72.75	128.39	239.71	86.07	151.39	2.08	0.000708
Solyc05g051490.2.1	LMBR1 domain protei	7.6	9.47	7.38	9.86	8.58	16.54	17.55	19.79	17.96	2.09	0.00071
Solyc06g062960.1.1	Unknown Protein (AH	10.86	7.52	18.03	9.67	11.52	0.32	1.64	0.4	0.79	0.07	0.000711
Solyc01g081610.2.1	Beta-hexosaminidase	13.29	14.37	30.13	22.03	19.95	48.95	41.72	83.33	58	2.91	0.000711
Solyc03g096290.2.1	Aquaporin-like protein	37.47	75.49	76.2	16.26	51.35	2.6	3.93	0.91	2.48	0.05	0.000718
Solyc05g010590.2.1	WPP domain-interacti	2.63	3.09	2.44	3.98	3.04	0.23	0.54	0.65	0.47	0.16	0.000719
Solyc11g072320.1.1	Something about silen	12.25	16.86	24.13	13.6	16.71	55.81	59.9	36.76	50.82	3.04	0.000719
Solyc01g094810.2.1	Ubiquitin-conjugating	106.27	92.04	110.23	64.56	93.28	23.34	31.96	29.73	28.34	0.3	0.000724
Solyc09g065160.2.1	Dynamin family protei	16.78	16.68	16.42	14.01	15.97	3.77	5.6	3.74	4.37	0.27	0.000724
Solyc04g076390.2.1	Cysteinyl tRNA synthe	15.39	14.21	13.38	15.06	14.51	3.83	4.81	3.22	3.95	0.27	0.000724
Solyc04g081070.2.1	Heat shock protein Dn	21.8	22.84	29.71	34.48	27.21	7.21	9.77	9.66	8.88	0.33	0.000725
Solyc01g104570.2.1	Kinetochore protein nd	8.67	4.77	7.27	8.72	7.36	0.92	2.05	0.79	1.25	0.17	0.000726
Solyc09g083280.2.1	Auxin responsive prote	5.02	4.17	2.37	4.26	3.95	0	0.27	0.33	0.2	0.05	0.000726
Solyc04g081440.2.1	Neutral invertase like	127.44	149.92	239.77	200.85	179.5	50.96	50.18	58.03	53.06	0.3	0.000739
Solyc06g034370.1.1	Pectinesterase (AHRD	3.42	5.78	4.03	5.32	4.64	0	0.56	0.68	0.41	0.09	0.000746
Solyc02g092840.1.1	Galactosyltransferase-	66.1	71.82	124.23	113.79	93.98	14.9	27.24	19.07	20.4	0.22	0.000746
Solyc04g064910.1.1	Endo-1 4-beta-glucana	9.27	13.1	9.01	6.17	9.39	0	0.89	0	0.3	0.03	0.000746
Solyc01g088690.2.1	ATP binding / serine-t	7.21	9.97	11.77	10.42	9.84	18.22	19	29.39	22.2	2.26	0.000754
Solyc04g074020.2.1	Receptor like kinase, F	3.53	2.89	1.58	4.3	3.08	0.57	0.4	0.84	0.6	0.2	0.000755
Solyc04g007880.2.1	Serine/threonine-prote	26.72	22.16	30.16	33.36	28.1	47.3	54.97	262.75	121.67	4.33	0.000759
Solyc09g098590.2.1	Sucrose synthase (AH	22.8	24.99	18.09	15.95	20.46	6.36	7.54	4.11	6	0.29	0.000761
Solyc02g082260.2.1	Hydroxy-methylglutary	60.05	88.27	116.1	195	114.85	5.31	7.86	12.94	8.7	0.08	0.000771
Solyc08g083500.1.1	cytochrome P450	0.51	1.91	2.24	0.89	1.39	0	0	0	0	0.01	0.000772
Solyc02g065650.1.1	RING finger protein 1	7.46	7.52	3.91	7.03	6.48	15.4	14.72	16.12	15.41	2.38	0.000773
Solyc02g087910.1.1	Non-specific lipid-tran	1.1	12.72	11.67	7.78	8.32	0	0	0	0	0	0.000773
Solyc04g078520.2.1	Uncharacterized basic	9.66	11.74	13.15	3.68	9.56	0.45	1.36	1.11	0.97	0.1	0.000776
Solyc02g071720.2.1	GDSL esterase/lipase	1.81	2.78	2.52	1.63	2.18	0.13	0	0	0.04	0.02	0.00078
Solyc01g008230.2.1	TCP family transcripti	150.15	164.87	212.33	185.51	178.22	40.08	65.58	66.79	57.48	0.32	0.00078
Solyc01g088480.2.1	Adenylate kinase (AH	101.47	84.16	80.06	91.09	89.19	21.83	27.65	18.09	22.52	0.25	0.000781
Solyc05g013810.2.1	Glycosyl hydrolase fan	1.85	1.52	4.36	0.6	2.08	5.97	5.45	8.75	6.72	3.23	0.000785
Solyc01g090360.2.1	Non-specific lipid-tran	3.98	9.62	12.75	3.22	7.39	0	0	1.13	0.38	0.05	0.000786
Solyc09g008940.2.1	AT-hook motif nuclea	6.31	4.35	6.33	4.24	5.31	0.89	1.16	0.94	1	0.19	0.000789
Solyc06g083440.2.1	Cytochrome b5 reduct	106.83	96.4	62.15	83.57	87.24	34.6	30.49	22.97	29.35	0.34	0.000794
Solyc04g072020.2.1	Choline kinase (AHRD	20.55	17.97	9.99	15.55	16.02	5.82	2.35	5.19	4.45	0.28	0.000798
Solyc04g074700.2.1	Homeobox-leucine zip	15.49	18.31	11.01	7.5	13.08	3.08	2.07	1.47	2.21	0.17	0.000798
Solyc08g067390.2.1	Blight-associated prote	10.15	6.98	8.16	9.79	8.77	0.37	1.13	0.46	0.65	0.07	0.000804
Solyc11g066690.1.1	OB-fold nucleic acid b	6.64	5.07	6.16	8.06	6.48	0.38	0.39	0	0.26	0.04	0.000811
Solyc02g080370.2.1	Tobamovirus multiplic	262.51	310.23	232.69	241.2	261.66	99.96	94.12	91.77	95.28	0.36	0.000812
Solyc11g069180.1.1	Isovaleryl-CoA dehydr	9.26	14.79	16.02	11.3	12.84	2.19	4.17	1.11	2.49	0.19	0.000812
Solyc04g080840.1.1	Beta-galactosidase (AH	4.12	3.99	0.85	1.01	2.49	0	0	0	0	0	0.000815
Solyc01g109920.2.1	Dehydrin (AHRD V1	1.32	3.05	3.74	4.11	3.05	34.96	26.88	63.39	41.74	13.66	0.000815
Solyc01g087040.2.1	Thylakoid lumenal 19	26.72	21.84	36.34	25.84	27.69	5.73	11.33	7.04	8.03	0.29	0.000816
Solyc06g030470.2.1	Auxin-regulated protei	3.31	4.27	3.32	2.57	3.37	46.35	27.62	53.17	42.38	12.59	0.000817
Solyc04g054830.2.1	Inositol-1 4 5-trisphos	0	0	0.14	0	0.04	1.05	1.44	1.52	1.34	38.19	0.000824
Solyc01g088090.2.1	Hydrolase alpha/beta f	38.32	43.95	45.06	22.01	37.34	397.4	290.48	205.76	297.88	7.98	0.000824
Solyc06g005250.2.1	mRNA clone RAFL24	10.34	20.63	16.41	14.12	15.37	39.76	26.73	34.33	33.61	2.19	0.000828
Solyc07g005030.2.1	Ribonuclease 3-like pr	14.77	16.5	19.45	12.2	15.73	5.03	4.83	4.86	4.91	0.31	0.000832
Solyc05g055330.2.1	ATP-binding cassette t	5.53	5.99	4.16	2.4	4.52	1.3	0.95	0.71	0.99	0.22	0.000832
Solyc06g051660.1.1	ELF4-like protein (AH	80.57	54.86	106.54	115.5	89.37	20.1	24.23	19.32	21.22	0.24	0.000839
Solyc07g041180.2.1	Unknown Protein (AH	0.87	1.04	1.72	1.07	1.18	0	0.14	0	0.05	0.04	0.000842
Solyc12g010750.1.1	Secretory carrier mem	3.84	2.09	1.76	5.13	3.21	0	0.17	0.21	0.13	0.04	0.000847
Solyc11g068550.1.1	Os12g0119800 protein	99.48	82.32	114.07	118	103.47	24.53	44.37	28.06	32.32	0.31	0.000853
Solyc02g080600.1.1	Unknown Protein (AH	15.99	16.01	15.33	11.31	14.66	0.81	1.63	0	0.81	0.06	0.000859
Solyc04g014820.2.1	RNA-binding protein (	30.41	26.63	30.67	36.77	31.12	4.76	10.79	3.16	6.24	0.2	0.000861
Solyc03g116280.2.1	1-aminocyclopropane-	12.09	11.2	1.78	9.97	8.76	0	0	0	0	0	0.000862
Solyc04g011900.2.1	Os04g0639300 protein	17.74	14.9	11.52	12.12	14.07	1.61	6.17	0.99	2.92	0.21	0.000868

Solyc09g082990.2.1	GDP-D-mannose-3芒耶	145.42	142.35	193.44	138.36	154.89	44.11	55.04	37	45.38	0.29	0.000883
Solyc02g063000.2.1	Glucosyltransferase (A	3.49	2.49	2.31	1.19	2.37	0.45	0.11	0	0.19	0.08	0.000886
Solyc07g007660.1.1	Unknown Protein (AH	7.62	9.95	6.12	8.48	8.04	36.54	24.44	29.44	30.14	3.75	0.000899
Solyc11g066630.1.1	MKI67 FHA domain-in	43.83	35.39	52.24	33.24	41.18	5.48	14.36	7.62	9.15	0.22	0.000899
Solyc10g075090.1.1	Non-specific lipid-tran	10947.96	8281.55	3829.08	5953.86	7253.11	1248.58	1196.77	548.55	997.97	0.14	0.0009
Solyc06g009120.2.1	Unknown Protein (AH	38.12	46.54	30.84	34.56	37.52	10.86	11.95	4.61	9.14	0.24	0.000901
Solyc04g008210.1.1	Xyloglucan endotransg	0.4	2.13	2.35	2.82	1.92	4.67	7.15	6.92	6.25	3.25	0.000905
Solyc03g114970.2.1	Nitrilase associated pro	243.86	228.56	214.04	309.11	248.89	59.01	103.2	42.1	68.1	0.27	0.000909
Solyc08g081690.2.1	Respiratory burst oxida	2.05	2.01	5.89	5.71	3.92	0.11	0.22	0.34	0.22	0.06	0.000928
Solyc12g088930.1.1	Plant-specific domain	8.16	9.39	5.66	2.38	6.4	0.39	0.98	0.24	0.54	0.08	0.000933
Solyc05g009050.2.1	Receptor-like protein k	8.22	9.68	5.27	7.86	7.76	1.56	1.38	1.2	1.38	0.18	0.000934
Solyc09g009040.2.1	Delta14-sterol reductas	21.75	24.91	29.03	36.32	28	4.61	8.57	6.73	6.64	0.24	0.000935
Solyc05g009870.2.1	Baculoviral IAP repeat	11.87	17.35	16.5	8.39	13.53	49.08	32.46	48.38	43.31	3.2	0.000946
Solyc03g005450.2.1	Cellulose synthase (AH	1.6	1.9	2.31	1.64	1.86	0.29	0.29	0.18	0.25	0.14	0.000951
Solyc07g064270.2.1	Glucose-6-phosphate/p	149.1	176.14	175.52	157.41	164.54	337.96	276.93	380.16	331.68	2.02	0.000952
Solyc10g045270.1.1	Serine/threonine/tyrosi	2.95	3.34	2.85	5.31	3.61	0	0.22	1.33	0.52	0.14	0.000955
Solyc10g012130.1.1	Non-specific lipid-tran	2.95	5.77	9.73	4.17	5.65	0	0	0	0	0	0.000962
Solyc02g021220.1.1	Subtilisin-like proteas	2.49	3.18	5.68	3.99	3.83	23.53	17.11	16.72	19.12	4.99	0.000963
Solyc05g053810.2.1	Serine hydroxymethylt	277.72	292.82	366.57	481.21	354.58	100.16	111.5	92.81	101.49	0.29	0.000963
Solyc03g095600.1.1	CENP-E like kinetoch	14.73	12.36	9.75	8.64	11.37	1.26	4.2	1.66	2.37	0.21	0.000963
Solyc12g005230.1.1	Breast carcinoma ampl	2.8	4.64	4.57	4.91	4.23	0.7	0.92	0.27	0.63	0.15	0.000969
Solyc03g120570.2.1	Proton-dependent olig	0	1.78	0.59	1.41	0.95	239.04	66.79	560.57	288.8	305.61	0.00097
Solyc03g115200.2.1	Glucan endo-1 3-beta-g	30.18	35.6	70.38	103.74	59.97	2.84	3.15	1.04	2.34	0.04	0.000979
Solyc02g063260.2.1	Multidrug resistance pr	11.11	14.89	7.58	13.44	11.75	3.03	2.07	2.13	2.41	0.21	0.000981
Solyc03g034360.2.1	Unknown Protein (AH	12.91	29.36	11.84	10.14	16.06	0	0	0	0	0	0.000991
Solyc10g017960.1.1	F-box protein PP2-B1 (	0	0	0	0	0	2.73	1.28	2.01	2.01	200.67	0.000991
Solyc11g011990.1.1	Plastid terminal oxyda	24.45	27.2	14.85	19.83	21.58	75.37	75	69.41	73.26	3.39	0.001
Solyc08g074480.1.1	Cortical cell-delineatin	1874.68	1942.01	1517.72	2649.6	1996	572.96	619.43	473.26	555.22	0.28	0.001003
Solyc01g010390.2.1	Beta-glucosidase (AHR	2.47	3.35	3.22	4.22	3.32	0.31	0.85	0.13	0.43	0.13	0.001012
Solyc08g008490.2.1	WD-40 repeat family p	6.93	10.49	12.74	12.86	10.76	41.88	29.12	28.94	33.31	3.1	0.001012
Solyc06g053600.2.1	Oxidoreductase aldo/k	24.94	28.48	19.55	20.66	23.41	4.91	10.3	3.91	6.37	0.27	0.001015
Solyc05g005070.2.1	RLK, Receptor like pro	1.47	1.55	2.16	3.11	2.07	0.07	0.07	0.45	0.2	0.09	0.001023
Solyc07g065130.2.1	Calcium-binding prote	1.76	2.99	1.13	0.68	1.64	0	0	0	0	0.01	0.001023
Solyc01g090760.2.1	GATA transcription fa	2.78	4.05	5.67	0.72	3.31	0	0	0	0	0	0.001023
Solyc07g064870.2.1	Endoglucanase 1 (AHR	27.18	24.08	26.24	39.49	29.25	7.19	9.66	5.27	7.37	0.25	0.001023
Solyc06g071410.2.1	Protein kinase (AHRD	11.36	14.42	13.2	26.7	16.42	1.08	3.13	1.33	1.85	0.11	0.001023
Solyc01g091360.2.1	Homology to unknown	9.99	7.54	14.64	3.42	8.9	0	0.99	0.3	0.43	0.05	0.001025
Solyc01g100230.2.1	Chaperone protein dna	16.79	11.03	6.6	9.05	10.87	19.7	49.52	77.78	49	4.51	0.001027
Solyc04g080580.2.1	Kinase PfkB family (A	55.12	64.48	60.08	56.68	59.09	16.4	24.53	19.11	20.01	0.34	0.00103
Solyc10g055420.1.1	Unknown Protein (AH	8.44	12.29	9.05	4.34	8.53	0	0.63	0	0.21	0.02	0.001032
Solyc02g070990.1.1	Chlorophyll a/b bindin	8.18	5.96	8.19	4.21	6.63	1.2	1.01	0	0.74	0.11	0.001032
Solyc12g042010.1.1	Unknown Protein (AH	24.22	36.7	29.8	23.33	28.51	0.83	10.08	3.07	4.66	0.16	0.001033
Solyc07g054270.2.1	Os08g0100600 protein	33.84	53.41	37.66	54.18	44.77	12.72	11.62	21.33	15.22	0.34	0.001034
Solyc08g063000.2.1	Nucleosome assembly	74.19	68.36	41.45	57.89	60.47	8.76	28.17	6.12	14.35	0.24	0.00104
Solyc07g052380.2.1	Multidrug resistance pr	3.27	4.89	3.4	4.63	4.05	0.63	0.53	0.91	0.69	0.17	0.001042
Solyc01g010230.2.1	Unknown Protein (AH	66.53	71.65	58.04	43.49	59.93	20.33	18.68	4.44	14.48	0.24	0.001052
Solyc03g093600.1.1	Unknown Protein (AH	10.71	8.61	15.08	10.26	11.16	1.67	1.69	1.71	1.69	0.15	0.001055
Solyc08g076890.2.1	Unknown Protein (AH	20.99	27.76	11.32	11.03	17.77	2.91	0.49	5.95	3.12	0.18	0.001066
Solyc01g080200.2.1	At2g23590-like protein	4.35	5.34	8.08	8.21	6.5	1.33	1.55	0.65	1.18	0.18	0.001073
Solyc01g103510.2.1	Ribosomal protein L3-	25.26	20.76	27.35	27.53	25.23	7.92	8.96	7.82	8.23	0.33	0.001074
Solyc03g096940.2.1	NADH ubiquinone oxi	140.71	122.63	95.07	102.17	115.15	32.38	57.16	33.57	41.04	0.36	0.001077
Solyc10g083330.1.1	Calcium/calmodulin pr	6.04	7.87	3.05	10.67	6.91	0.17	0.7	0.21	0.36	0.05	0.001078
Solyc03g025970.2.1	Methyl binding domain	13.01	12.98	17.34	13.49	14.21	73.69	46.17	94.1	71.32	5.02	0.001083
Solyc01g099980.2.1	Glycine-rich protein (A	56.01	61.95	65.8	28.34	53.02	14.96	18.24	7.09	13.43	0.25	0.0011
Solyc06g071390.2.1	Deleted in split hand/s	64.89	66.41	52.31	42.83	56.61	12.23	20.27	6.44	12.98	0.23	0.0011
Solyc08g067340.2.1	WRKY transcription fa	65.45	58.48	76.3	26.45	56.67	4.62	3.17	5.67	4.49	0.08	0.0011
Solyc04g077630.2.1	Serine carboxypeptidas	1.09	1.7	0.48	1.35	1.16	13.99	13.33	13.12	13.48	11.67	0.001103
Solyc03g006430.2.1	Os11g0198100 protein	9.71	12.77	8.66	12.78	10.98	2.65	3.59	1.35	2.53	0.23	0.001104
Solyc03g033730.1.1	BZIP transcription fact	6.82	11.64	6.71	3.22	7.1	0.46	0.46	0	0.31	0.04	0.001107
Solyc03g025370.2.1	Short-chain dehydroge	5.67	4.86	3.72	4.75	4.75	0.51	0.86	0.42	0.6	0.13	0.001107
Solyc02g030230.2.1	UDP-glucose 4-epimer	21.2	15.49	12.69	14.55	15.98	32.08	67.42	41.3	46.93	2.94	0.001113
Solyc10g076300.1.1	UPF0497 membrane p	2.33	4.45	5.5	2.36	3.66	29.61	28.78	24.47	27.62	7.55	0.001113
Solyc11g069470.1.1	Class III homeodomain	16.45	17.43	23.2	15.18	18.06	4.87	5.87	4.12	4.95	0.27	0.001115
Solyc03g118770.2.1	WUSCHEL-related ho	2.18	1.34	0.79	3.32	1.91	0	0	0	0	0.01	0.001116
Solyc01g099840.2.1	Auxin-repressed protei	439.81	416.91	716.78	460.79	508.57	99.99	137.94	175.63	137.85	0.27	0.001117
Solyc02g071810.2.1	Receptor like kinase, R	0.98	0.93	1.39	0.83	1.03	0.11	0.05	0.13	0.1	0.09	0.001117
Solyc11g045030.1.1	Cytoplasmic membran	9.19	7.01	10.46	7.43	8.52	1.33	1.07	1.3	1.23	0.14	0.001119
Solyc02g067580.2.1	B12D-like protein (AH	367.14	392.49	272.41	272.85	326.22	840.61	951.28	657.67	816.52	2.5	0.001132
Solyc02g089550.2.1	Receptor like kinase, R	6.94	8.62	7.2	6.85	7.4	0.68	1.37	1.36	1.14	0.15	0.001139
Solyc01g010940.2.1	Transmembrane emp24	35.27	35.99	33.36	30.96	33.9	68.53	71.35	66.99	68.96	2.03	0.00114
Solyc03g121620.1.1	Harpin-induced protein	38.65	39	63.6	40.39	45.41	2.59	17.25	13.68	11.17	0.25	0.001152
Solyc08g081620.2.1	Endoglucanase 1 (AHR	0	0	0.47	0	0.12	4.88	0.86	6.78	4.17	35.52	0.001152
Solyc07g052610.2.1	Cyclin (AHRD V1 *-*	24.26	28.87	18.65	8.1	19.97	1.32	3.33	1.35	2	0.1	0.001152
Solyc08g068400.2.1	Dienelactone hydrolase	48.01	48.77	35.02	38.36	42.54	11.55	16.13	9.09	12.26	0.29	0.001155
Solyc06g069630.2.1	Unknown Protein (AH	4.34	4.02	5.23	4.89	4.62	0.5	1.3	0.44	0.75	0.16	0.001172
Solyc08g075540.2.1	Alternative oxidase (A	2.21	7.2	14.53	14.56	9.62	76.46	34.29	101.96	70.9	7.37	0.001172
Solyc10g005610.1.1	Unknown Protein (AH	2.31	1.58	4.18	4.77	3.21	0.29	0.29	0	0.19	0.06	0.001179
Solyc03g031680.2.1	Uncharacterized GPI-a	8.83	11.58	5.3	6.13	7.96	2.25	1.14	1.69	1.69	0.21	0.001182
Solyc04g009740.2.1	Exocyst complex prote	44.82	52.41	57.78	56.53	52.88	10.71	20.86	10.45	14.01	0.26	0.001202
Solyc01g098500.2.1	Glucose transporter 8 (	98.24	117.81	97.21	85.44	99.67	31.49	39.91	26.65	32.68	0.33	0.001207
Solyc01g066860.2.1	Unknown Protein (AH	23.68	28.23	14.47	11.96	19.59	2.73	4.48	1.68	2.96	0.15	0.001208
Solyc06g065330.2.1	Ring finger protein 13	13.32	22.19	18.51	20.67	18.67	40.1	38	51.83	43.31	2.32	0.001217

Solyc08g076800.2.1	Ring H2 finger protein	0.23	0.21	0	0	0.11	12.39	9.46	13.37	11.74	106.73	0.001219
Solyc02g083270.2.1	Vitamin K epoxide red	31.22	30.46	28.72	26.7	29.28	7.13	10.78	4.73	7.55	0.26	0.00122
Solyc11g007930.1.1	Histone H2B (AHRD V	5.7	6.76	2.8	5.37	5.16	11.5	14.7	7.07	11.09	2.15	0.001227
Solyc12g098810.1.1	Chitinase-like protein (	9.83	13.56	13.25	13.61	12.56	28.35	59.64	28.86	38.95	3.1	0.001232
Solyc05g007160.2.1	Transcription factor C-	18.75	22.57	34.86	34.16	27.59	2.4	4.62	4.82	3.95	0.14	0.001237
Solyc06g008500.2.1	Ankyrin repeat-contair	6.71	4.72	2.78	3.67	4.47	0.57	1.06	0.47	0.7	0.16	0.001243
Solyc05g043330.2.1	GDSL esterase/lipase A	35.57	62.81	17.61	25.28	35.32	6.1	0.75	4.21	3.69	0.1	0.001251
Solyc10g085900.1.1	Tetratricopeptide TPR	10.53	10.33	9.38	9.42	9.92	393.58	275.44	274.3	314.44	31.71	0.001253
Solyc03g121840.2.1	Histone H1-like protei	18.85	22.02	19.23	18.05	19.54	36.8	38.56	44.43	39.93	2.04	0.001254
Solyc09g090040.2.1	Germin-like protein 5 (	40.04	52.3	25.83	46.05	41.05	9.54	15.26	5.43	10.08	0.25	0.001255
Solyc03g026200.2.1	Os10g0522500 protein	14.68	12.48	10.86	20.15	14.54	3.19	4.46	5.44	4.36	0.3	0.001258
Solyc07g049610.1.1	Galactosyltransferase-l	11.49	11.55	12.12	23.14	14.57	0.33	1.32	2.01	1.22	0.08	0.00127
Solyc10g050980.1.1	Unknown Protein (AH	0	0.88	1.16	0	0.51	9.55	10.43	1.95	7.31	14.33	0.001272
Solyc05g056200.1.1	Receptor-like kinase (A	1.94	1.73	1.76	2.54	1.99	8.46	5.36	5.94	6.59	3.31	0.001279
Solyc03g031970.2.1	Auxin response factor	79.71	77.13	78.29	94.2	82.33	27.56	29.33	26.35	27.75	0.34	0.001287
Solyc01g103630.2.1	Glyoxalase/bleomycin	3.59	8.14	9.25	5.54	6.63	0.26	1.6	0.65	0.84	0.13	0.001288
Solyc05g008140.2.1	Unknown Protein (AH	65.65	56.41	52.65	50.32	56.26	18.55	25.56	13.48	19.2	0.34	0.001292
Solyc10g012370.2.1	Cysteine synthase (AH	27.36	38.86	32.95	30.75	32.48	113.87	79.87	103.99	99.24	3.06	0.001299
Solyc01g104210.2.1	Elongation of very lon	0	0	0	0	0	100.29	33.33	104.21	79.28	7927.67	0.001308
Solyc03g117340.1.1	Jp18 (AHRD V1 ***-	32.99	26.13	6.41	15.89	20.36	1.46	0	1.44	0.97	0.05	0.00131
Solyc11g072620.1.1	Unknown Protein (AH	7.61	8.28	16.96	22.73	13.89	0.68	0.69	0	0.46	0.03	0.00131
Solyc02g084770.2.1	Mitochondrial carrier p	6.51	6.36	8.18	8.02	7.27	0.68	1.2	2.71	1.53	0.21	0.001322
Solyc03g120900.1.1	Protein transport SEC1	1.31	1.75	1.8	1.24	1.53	5.65	6.41	3.9	5.32	3.49	0.001325
Solyc03g119320.1.1	At1g67050 (Fragment)	12.4	16.31	17.3	14.36	15.09	2.05	3.22	1.96	2.41	0.16	0.001332
Solyc09g092450.2.1	Long-chain-fatty-acid (	24.06	25.06	14.73	13.92	19.44	3.75	7.13	5.97	5.62	0.29	0.001332
Solyc12g019680.1.1	Cytochrome P450	0	0	0	0	0	2.18	0.94	3.18	2.1	210	0.001342
Solyc12g099250.1.1	Kinase family protein (	5.16	5.59	7.92	4.74	5.85	1.11	1.82	0.77	1.23	0.21	0.001345
Solyc04g050490.2.1	Carboxyl-terminal prot	10.79	16.5	9.5	6.67	10.87	2.58	1.24	0.45	1.42	0.13	0.001358
Solyc09g007510.1.1	Receptor-like kinase (A	3.63	6.27	6.7	6.91	5.88	1.83	1.11	0.45	1.13	0.19	0.001362
Solyc08g076010.2.1	Myb family transcripti	3.83	2.43	2.8	3.61	3.17	9.28	8.17	13.39	10.28	3.25	0.001366
Solyc04g079600.2.1	Myb family transcripti	1.92	2.47	3.53	1.51	2.36	0	0.17	0.21	0.13	0.05	0.001367
Solyc09g015180.2.1	Os04g0479300 protein	26.89	22.25	34.95	42.2	31.57	49.06	66.26	76.95	64.09	2.03	0.00137
Solyc09g092310.1.1	Cc-nbs-lrr, resistance p	1.94	1.59	1.55	2.63	1.93	0.13	0.25	0.31	0.23	0.12	0.00137
Solyc10g080150.1.1	24-sterol C-methyltran	6	8.9	10.42	11.78	9.28	1.75	2.04	1.98	1.92	0.21	0.00137
Solyc06g063340.2.1	cDNA clone J023065D	4.63	4.81	3.1	9.62	5.54	0.12	0.38	0.31	0.27	0.05	0.001379
Solyc04g082140.2.1	Laccase-22 (AHRD V1	101.58	70.98	94.23	147.65	103.61	8.77	33.98	16.34	19.7	0.19	0.001381
Solyc02g081370.2.1	Acyl-CoA synthetase /	1.86	0.93	0.96	3.29	1.76	0	0	0	0	0.01	0.001383
Solyc03g007890.2.1	Heat shock protein 90	4.62	4.53	6.23	11.6	6.75	19.04	107.13	148.18	91.45	13.56	0.001389
Solyc04g014480.2.1	class I heat shock prote	107.99	71.58	49.58	65.83	73.74	477.5	725.73	403.66	535.63	7.26	0.00139
Solyc05g012790.2.1	Palmitoyltransferase en	14.74	18.98	27.75	33.42	23.72	1.35	3.41	4.36	3.04	0.13	0.001407
Solyc09g015020.1.1	class I heat shock prote	14.05	14.77	5.02	8.43	10.57	115.54	307.29	120.4	181.08	17.14	0.001425
Solyc04g077510.2.1	Growth regulating fact	1.99	2.66	4.18	2.51	2.83	0.18	0.63	0.22	0.34	0.12	0.001427
Solyc04g009420.2.1	Photosystem II oxygen	23.43	19.17	19.7	14.85	19.29	5	6.8	2.41	4.74	0.25	0.001429
Solyc09g075020.2.1	Multidrug resistance p	4.36	2.87	4.06	2.53	3.46	11.02	8.1	10.81	9.98	2.89	0.001431
Solyc12g005020.1.1	Ring H2 finger protein	2.95	3.93	4.17	1.67	3.18	0	0	0	0	0	0.001431
Solyc11g066010.1.1	CM0545.320.nc protei	3	4.27	0.71	2.54	2.63	0	0	0	0	0	0.001432
Solyc07g005610.2.1	Endonuclease/exonucl	16.89	19.57	19.92	24.44	20.21	46.64	41.28	42.88	43.6	2.16	0.001433
Solyc03g093500.2.1	WD-repeat protein (AI	8.44	7.51	7.24	1.63	6.2	0.31	0.31	0.38	0.33	0.05	0.001434
Solyc05g005150.1.1	F-box/Kelch repeat-co	5.87	5.7	3.15	4.28	4.75	0.43	1.01	0.88	0.77	0.16	0.001436
Solyc06g033850.2.1	Dehydration-responsiv	1.96	1.16	0.77	0	0.97	5.28	11.18	5.84	7.43	7.64	0.001437
Solyc11g043060.1.1	Lrr, resistance protein	5.13	5.48	2.82	3.38	4.2	0	0.28	0	0.09	0.02	0.001437
Solyc03g093330.2.1	Receptor like kinase, R	0.27	0.78	0.95	1.04	0.76	0	0.05	0.07	0.04	0.05	0.00144
Solyc12g017240.1.1	Xyloglucan endotransg	2.27	3.64	2.95	6.11	3.74	11.76	21.5	36.35	23.2	6.2	0.001443
Solyc03g113240.2.1	Unknown Protein (AH	91.88	113.43	117.77	106.02	107.27	30.97	38.56	37.03	35.52	0.33	0.001444
Solyc03g097530.2.1	TPD1 (AHRD V1 ***-	3.35	5.31	1.76	2.64	3.27	0	0	0.37	0.12	0.04	0.001444
Solyc01g103230.2.1	Non-lysosomal glucosy	16.51	17.09	16.71	18.08	17.1	4.19	5.74	3.29	4.41	0.26	0.001445
Solyc02g081210.2.1	Expansin (AHRD V1 *	11.7	12.63	11.45	17.25	13.26	2.41	2.64	7.17	4.07	0.31	0.001445
Solyc01g073660.2.1	Uncharacterized MFS-	1.74	1.55	1.71	0.82	1.46	10.52	6.96	5.74	7.74	5.32	0.001448
Solyc09g082690.2.1	Early light-induced pro	148.43	137	213.63	69.5	142.14	6084.92	5017.65	2138.06	4413.54	31.05	0.00145
Solyc03g121540.2.1	Beta-galactosidase (AI	4.17	5.95	5.84	4.56	5.13	23.38	10.32	28.18	20.63	4.02	0.001452
Solyc05g016060.2.1	Unknown Protein (AH	17.17	11.26	15.99	17.9	15.58	25.55	53.76	22.43	33.91	2.18	0.001462
Solyc09g091430.2.1	Pectate lyase 1-27 (AH	5.84	7.14	5.34	9.29	6.9	68.16	78.02	153.27	99.82	14.46	0.001467
Solyc04g026100.1.1	30S ribosomal protein	315.59	344.35	231.92	250.94	285.7	99.75	104.61	99.99	101.45	0.36	0.001471
Solyc10g007900.2.1	Cytochrome P450	8.6	9.76	5.37	2.39	6.53	0.14	0.82	0.17	0.38	0.06	0.001486
Solyc11g013370.1.1	E3 ubiquitin-protein lig	14.78	15.3	16.61	17.83	16.13	36.77	27.96	39.46	34.73	2.15	0.001489
Solyc06g036290.2.1	Heat shock protein 90	4.01	2.41	0.51	2.3	2.31	10.78	73.56	115.46	66.6	28.86	0.001489
Solyc12g014530.1.1	L-aspartate oxidase (A	13.5	10.27	23.12	18.17	16.27	4.31	3.46	3.78	3.85	0.24	0.001493
Solyc11g011070.1.1	Genomic DNA chromo	3.5	2.7	3.3	4.29	3.45	6.78	10.83	8.33	8.65	2.51	0.0015
Solyc01g108930.2.1	B3 domain-containing	63.43	48.24	15.78	32.63	40.02	0.75	4.7	0	1.82	0.05	0.001509
Solyc12g062800.1.1	Unknown Protein (AH	5.17	21.87	10.68	9.15	11.72	0	0	0	0	0	0.001511
Solyc05g052030.1.1	Ethylene responsive tra	21.88	10.75	11.94	12.47	14.26	1.06	2.66	1.62	1.78	0.12	0.001512
Solyc12g010950.1.1	Alcohol dehydrogenase	12.55	22	13.46	12.91	15.23	36.25	29.9	31.14	32.43	2.13	0.001514
Solyc05g051400.2.1	Mitochondrial 2-oxogl	59.64	51.71	112.88	117.42	85.41	6.18	16.2	10.19	10.86	0.13	0.001516
Solyc01g107590.2.1	Cinnamyl alcohol dehy	12.35	18.86	10.22	6.26	11.92	2.53	1.95	0.37	1.62	0.14	0.001521
Solyc08g082960.2.1	Zinc finger HIT domai	2.41	2.72	1.33	2.28	2.19	4.55	8.39	7.99	6.98	3.19	0.001532
Solyc04g005020.2.1	WD-40 repeat family p	1.69	1.5	1.09	0.65	1.23	15.62	10.25	9.29	11.72	9.51	0.001547
Solyc10g006710.2.1	Serine/threonine kinase	9.24	9.43	9.31	8.68	9.17	15.77	16.3	27.36	19.81	2.16	0.001547
Solyc12g056350.1.1	Unknown Protein (AH	2.03	2.61	0.53	2.87	2.01	0	0	0	0	0	0.001547
Solyc09g098120.2.1	DUF1264 domain prot	1.1	2.95	3.91	1.95	2.48	0	0	0	0	0	0.001553
Solyc09g075420.2.1	Ethylene responsive tra	28.31	24.07	21.17	33.61	26.79	31.24	59.32	120.43	70.33	2.63	0.001568
Solyc11g020280.1.1	Receptor like kinase, R	20.02	26.49	29.69	27.65	25.96	7.53	9.72	6.19	7.81	0.3	0.001568
Solyc06g062920.2.1	ATP binding / serine-tl	4.11	5.05	3.18	0.6	3.23	0	0	0	0	0	0.001569

Solyc02g086490.2.1	Oleosin (AHRD V1 **	6.16	27.4	53.44	19.29	26.57	0.36	0	0	0.12	0	0.001578
Solyc10g006000.2.1	Dek protein (AHRD V	2.85	2.12	2.52	2.52	2.5	0.38	0.29	0.12	0.26	0.11	0.001579
Solyc01g097540.2.1	Annexin 3 (AHRD V1	14.06	24.11	14.88	17.26	17.58	44.61	39.43	37.15	40.4	2.3	0.00159
Solyc08g008080.1.1	RING finger protein (A	17.84	16.99	13.71	26.41	18.74	2.35	3.72	2.06	2.71	0.14	0.001591
Solyc05g014260.2.1	Response regulator 11	30.72	29.36	31.69	22.61	28.59	8.79	10.16	7.88	8.94	0.31	0.001599
Solyc08g066240.2.1	Decarboxylase family	0.89	0.92	2.45	8.6	3.21	2792.96	664.54	3105.92	2187.81	680.5	0.001604
Solyc05g052450.2.1	Os03g0291800 protein	1.39	1.1	0.37	0.66	0.88	2.25	4.29	2.46	3	3.41	0.001611
Solyc03g082690.2.1	U-box domain-contain	6.02	6.06	7.8	7.68	6.89	1.64	1.39	1.82	1.62	0.23	0.001623
Solyc01g107650.2.1	Receptor like kinase, R	0.6	1.14	1.01	0.97	0.93	0	0.07	0	0.02	0.03	0.001624
Solyc07g014590.2.1	Glycogen debranching	13.73	14.53	9.98	11.72	12.49	0.68	3.27	1.5	1.82	0.15	0.001624
Solyc02g094570.2.1	Receptor-like kinase (A	7.3	9.44	5.84	11	8.39	1.71	1.92	2.1	1.91	0.23	0.00163
Solyc06g083170.2.1	Transcription factor (A	9.93	5.68	4.18	3.61	5.85	0.34	0.35	0	0.23	0.04	0.00163
Solyc08g005050.2.1	Transcription factor (A	72.36	51.77	123.53	105.36	88.25	10.09	19.65	18.45	16.06	0.18	0.00163
Solyc07g066240.2.1	F-box/LRR-repeat prot	4.67	5.31	5.25	4.45	4.92	9.73	11.85	9.15	10.24	2.08	0.001642
Solyc12g040800.1.1	C2 domain-containing	27.26	26.72	23.77	16.76	23.63	4.15	9.66	0.39	4.73	0.2	0.001648
Solyc11g068770.1.1	CCR4-NOT transcripti	11.53	8.83	13.61	21.53	13.88	32.99	23.12	84.94	47.02	3.39	0.001653
Solyc09g075790.2.1	Long-chain fatty acid C	5.56	6.16	4.95	6.53	5.8	1.02	1.37	0.42	0.94	0.16	0.001655
Solyc06g059870.1.1	Unknown Protein (AH	859.59	1182.17	536.69	217.27	698.93	51.71	49.75	22.48	41.31	0.06	0.001656
Solyc02g085710.2.1	Geranylgeranyl pyroph	3.68	3.73	2.96	4.03	3.6	8.39	8.73	9.31	8.81	2.45	0.00166
Solyc12g098890.1.1	50S ribosomal protein	159.46	142.28	71.15	97.32	117.55	29.1	36.61	14.06	26.59	0.23	0.001663
Solyc11g020330.1.1	class IV heat shock pro	7.6	11.07	9.37	10.75	9.7	333.46	205.18	254.73	264.46	27.27	0.001663
Solyc12g038800.1.1	Unknown Protein (AH	59.2	54.57	72.34	46.06	58.04	14.01	24.12	12.17	16.77	0.29	0.001676
Solyc10g079470.2.1	L-galactono-1,4-lacton	19.26	14.35	20.48	19.8	18.47	3.26	7.03	4	4.76	0.26	0.00168
Solyc01g099640.2.1	Unknown Protein (AH	48.87	54.36	40.36	38.02	45.4	175.69	129.42	167.34	157.48	3.47	0.001681
Solyc11g071830.1.1	Chaperone protein dna	14.8	10.79	10.59	6.9	10.77	18.19	33.87	68.45	40.17	3.73	0.001689
Solyc02g083120.1.1	CM0216.240.nc protei	11.41	7.39	15.91	9.39	11.03	1.84	1.69	2.68	2.07	0.19	0.001691
Solyc01g111050.2.1	Ch-cobra (AHRD V1 *	5.75	7.62	3.31	1.79	4.62	75.64	48.72	44.58	56.31	12.2	0.001696
Solyc03g116870.2.1	Glycosyl transferase fa	30.28	31.51	26	24.74	28.13	5.17	14.25	5.5	8.31	0.3	0.001697
Solyc10g085590.1.1	Tumor susceptibility p	11.43	12.64	19.84	19.4	15.83	23.35	33.8	54.78	37.31	2.36	0.001697
Solyc01g110150.2.1	CM0216.450.nc protei	20.87	18.27	16.68	18.57	18.6	36.44	40.87	45.1	40.8	2.19	0.001727
Solyc01g095710.2.1	ATP-dependent RNA h	1.47	1.14	1.86	1.6	1.52	5.88	4.21	4.78	4.96	3.27	0.001737
Solyc08g074630.1.1	Polyphenol oxidase (A	0.79	1.1	1.32	1.75	1.24	0.09	0	0	0.03	0.02	0.001742
Solyc07g063230.2.1	Choline transporter lik	8.67	13.42	6.67	8.8	9.39	0.69	1.69	0.47	0.95	0.1	0.001746
Solyc02g076930.1.1	Armadillo/beta-catenin	4.16	7.13	2.55	2.83	4.17	0.12	0.75	0.46	0.44	0.11	0.00175
Solyc04g074070.1.1	High-affinity glucose t	1.29	1.5	0.92	0.73	1.11	0	0	0	0	0.01	0.001751
Solyc03g120630.2.1	30S ribosomal protein	345.21	230.06	246.22	278.77	275.06	89.84	127.46	100.9	106.07	0.39	0.001758
Solyc07g063820.2.1	Serine/threonine-prote	3.18	3.61	2.63	2.7	3.03	0.77	0.65	0.08	0.5	0.17	0.001758
Solyc06g048960.2.1	Ribonuclease 3-like pr	3.96	4.74	5.28	3.27	4.31	1.37	1.04	0.75	1.05	0.24	0.00176
Solyc04g011350.2.1	2-oxoglutarate dehydro	6.72	5.52	6.33	8.59	6.79	9.45	21.32	10.58	13.78	2.03	0.001763
Solyc07g007550.2.1	Heparanase (AHRD V	12.65	15.64	15.07	7.82	12.79	4.27	4.2	2.68	3.72	0.29	0.001763
Solyc11g006590.1.1	Cytochrome P450	4.24	8.02	6.56	0.94	4.94	0	0.32	0	0.11	0.02	0.001773
Solyc11g061720.1.1	Kinase family protein (	2.62	2.91	3.67	5.56	3.69	0	0.53	0.32	0.28	0.08	0.001775
Solyc10g077050.1.1	Palmitoyltransferase er	12.57	14.47	10.31	10.82	12.04	36.3	29.27	26.3	30.62	2.54	0.001784
Solyc04g077360.2.1	Serine/threonine-prote	0.65	0.29	0.48	0.46	0.47	1.05	1.99	1.45	1.5	3.18	0.001784
Solyc06g076610.2.1	Zinc finger family prot	17.9	20.03	20.21	19.17	19.33	4.96	6.88	4.57	5.47	0.28	0.001791
Solyc01g098710.2.1	Aspartyl protease fami	5.77	5.49	6.45	8.87	6.64	0.98	1.07	1.1	1.05	0.16	0.0018
Solyc10g005330.2.1	Homeobox-leucine zip	8.05	7	2.84	4.84	5.68	1.27	0.53	0.73	0.84	0.15	0.001802
Solyc01g106430.2.1	Inorganic pyrophospha	158.92	146.28	160.48	147.51	153.3	35.72	68.91	26.78	43.8	0.29	0.001804
Solyc12g007190.1.1	Unknown Protein (AH	1.61	1.43	5.06	4.55	3.16	10.4	12.24	14.37	12.34	3.9	0.00181
Solyc01g094360.2.1	MYB transcription fac	15.96	14.39	11.55	4.82	11.68	1.72	2.08	0.42	1.41	0.12	0.001811
Solyc01g097880.2.1	Deaminase (AHRD V1	131.59	156.68	129.03	125.25	135.64	2539.52	1491.13	1544.61	1858.42	13.7	0.001826
Solyc12g036330.1.1	Receptor-like protein k	2.43	1.62	1.67	1.72	1.86	0.08	0.16	0.1	0.11	0.06	0.001829
Solyc11g072190.1.1	Elongation factor beta-	602.71	564.74	880.39	742.84	697.67	173.92	233.42	204.2	203.85	0.29	0.001842
Solyc04g076050.2.1	Divalent cation toleran	95.66	99.16	84.75	83.47	90.76	228.59	215.03	181.17	208.26	2.29	0.001851
Solyc08g007270.2.1	Homeobox-leucine zip	6.21	3.62	5.64	1.01	4.12	0	0.39	0	0.13	0.03	0.001852
Solyc07g043050.1.1	UDP-glucosyltransfera	6.22	6.89	9.14	8.02	7.57	1.01	2.59	1.24	1.61	0.21	0.001854
Solyc01g067670.2.1	Unknown Protein (AH	0	1.01	5.37	4.83	2.8	117.62	38	232.57	129.4	46.17	0.001862
Solyc01g098590.2.1	Phosphoglycerate muta	212.26	159.87	156.76	289.12	204.5	63.4	49.04	60.2	57.55	0.28	0.001862
Solyc01g079860.2.1	Carbohydrate kinase Y	32.88	39.99	30.36	28.89	33.03	82.01	74.73	62.04	72.93	2.21	0.001866
Solyc12g037970.1.1	Peptidyl-tRNA hydrola	13.01	13.05	14.79	18.06	14.73	25.03	35.26	37.08	32.46	2.2	0.001866
Solyc01g110000.2.1	Beta-galactosidase (AI	15.59	22.9	28.01	32.17	24.67	4.61	7.31	3.78	5.23	0.21	0.001866
Solyc12g013750.1.1	Mannan endo-1,4-beta	47.75	48.65	42.99	44.44	45.96	13.71	11.26	13.72	12.9	0.28	0.001873
Solyc06g068510.1.1	Serine/threonine-prote	2.01	2.61	2.01	0.87	1.87	8.49	6.42	7.21	7.37	3.93	0.001873
Solyc04g009900.2.1	Calcium-dependent pro	6.36	7.76	5.56	9.66	7.33	15.99	19.39	17.07	17.48	2.38	0.001878
Solyc11g013820.1.1	Phosphatidylinositol-4	5.77	5.36	5.03	3.9	5.01	11.75	12.47	12.45	12.22	2.44	0.001897
Solyc01g100530.2.1	Protein EFR3 homolog	1.49	1.87	0.96	1.44	1.44	0.11	0.22	0	0.11	0.08	0.001903
Solyc11g010300.1.1	3 4-dihydroxy-2-butano	10.88	8.19	8.88	9.64	9.4	19.89	18.8	18.15	18.95	2.02	0.001913
Solyc02g077590.1.1	Homeobox-leucine zip	5.5	7.5	7.35	2.59	5.74	0	0.6	0	0.2	0.03	0.001918
Solyc05g010080.2.1	Unknown Protein (AH	10.52	6.83	1.34	3.62	5.58	0	0	0	0	0	0.001923
Solyc12g014390.1.1	50S ribosomal protein	86.91	70.2	59.22	68.97	71.33	22.25	30.15	30.18	27.53	0.39	0.001926
Solyc11g010350.1.1	Glucan 1 3-beta-glucos	7.67	7.62	4.35	4.38	6	0.12	2.16	0.73	1	0.17	0.001928
Solyc08g082400.1.1	Genome sequencing da	99.09	113.45	119.69	84.76	104.25	36.31	40.48	26.35	34.38	0.33	0.001928
Solyc11g044910.1.1	Beta-xylosidase 1 (AH	0.09	0.08	0.1	0	0.07	7.11	3.55	1.36	4.01	59.36	0.001928
Solyc03g112860.2.1	S-ribonuclease binding	5.29	5.8	5.77	5.18	5.51	12.5	14.27	12.93	13.23	2.4	0.001932
Solyc08g082830.2.1	NH(3)-dependent NAI	6.85	6.58	7.97	6.12	6.88	16.31	11.68	19.23	15.74	2.29	0.001936
Solyc09g082790.2.1	DNA repair and recom	0.39	0.35	0.46	0.56	0.44	1.27	3.37	0.78	1.81	4.11	0.001937
Solyc01g009610.2.1	Unknown Protein (AH	168.66	145.68	125.56	143.5	145.85	62.35	54.26	34.59	50.4	0.35	0.00194
Solyc09g042710.2.1	Myosin heavy chain-lil	10.69	14.63	16.27	10.08	12.92	3.44	5.24	1.25	3.31	0.26	0.00194
Solyc08g080190.2.1	Choline dehydrogenase	29.89	35.68	26.48	22.68	28.68	10.55	14.19	5.8	10.18	0.35	0.001942
Solyc05g008750.2.1	Splicing factor arginine	23.95	23.93	20.82	21.99	22.67	57.37	43.49	43.38	48.08	2.12	0.001945
Solyc08g080830.2.1	Receptor like kinase, R	4.71	7.48	5.55	6.66	6.1	1.29	1.47	2.09	1.62	0.27	0.001948
Solyc12g005480.1.1	Targeting protein for X	0.89	0.87	0.42	0.88	0.77	0	0	0	0	0.01	0.001963

Solyc12g088320.1.1	LIM domain protein (A	7.02	9.99	3.73	7.45	7.05	1.13	0.86	0	0.66	0.09	0.001987
Solyc02g032100.2.1	Low affinity zinc trans	0.38	0	0.45	0	0.21	3.22	1.85	1.69	2.25	10.86	0.001987
Solyc11g006900.1.1	Calcium/calmodulin pr	6.3	8.56	9.39	12.19	9.11	17.41	24.04	15.14	18.86	2.07	0.002006
Solyc01g094210.2.1	Carbonyl reductase 3 (	7.08	9.85	7.05	3.13	6.78	0.54	0.54	0.66	0.58	0.09	0.002007
Solyc10g086320.1.1	Phosphatidylinositol-4	1.84	2.45	1.87	2.72	2.22	0.4	0.2	0.33	0.31	0.14	0.002015
Solyc10g005790.2.1	Genomic DNA chromo	6.63	8.35	6.86	5.91	6.94	1.07	2.33	0.61	1.34	0.19	0.002019
Solyc06g054260.1.1	Photosystem I reaction	392.35	276.71	494.92	433.99	399.49	145.1	149.18	143.18	145.82	0.37	0.002024
Solyc09g074980.2.1	MutL DNA mismatch	1.9	1.92	1.95	0.82	1.65	0.13	0.27	0.08	0.16	0.1	0.002033
Solyc01g108440.2.1	Calmodulin-binding pr	9.71	4.32	8.59	7.16	7.45	0.16	0.99	0.2	0.45	0.06	0.002046
Solyc03g098300.1.1	Ornithine decarboxylas	1.53	1.51	4.8	6.72	3.64	0	0	0	0	0	0.002046
Solyc10g049630.1.1	Serine/threonine protei	5.89	8.94	5.52	18.86	9.8	17.77	27.8	74.76	40.11	4.09	0.00205
Solyc12g097010.1.1	Lrr, resistance protein	1.12	1.07	0.51	0.85	0.89	0	0	0	0	0.01	0.002068
Solyc02g077710.1.1	E6-2 protein kinase (A	528.54	497.27	249.83	277.5	388.28	2999.95	4703.24	5145.03	4282.74	11.03	0.002073
Solyc04g079050.1.1	UDP-glucosyltransfera	3.29	4.29	6.98	7.75	5.58	1.06	0.89	1.09	1.01	0.18	0.002073
Solyc03g123590.2.1	Remorin family protei	1.41	2.26	4.5	1.8	2.49	0.11	0.12	0	0.08	0.03	0.002074
Solyc11g069010.1.1	Alpha-L-fucosidase 1 (	22.37	30.77	29.39	26.61	27.29	7.09	9.32	6.69	7.7	0.28	0.002074
Solyc08g077790.2.1	Peptidyl-prolyl cis-tran	54.85	50.18	49.55	50.73	51.33	10.07	19.69	13.13	14.3	0.28	0.002075
Solyc01g097830.2.1	AT5G28150-like prote	0	0.17	0	0.27	0.11	1.08	3.41	0.57	1.69	15.33	0.002077
Solyc03g123710.2.1	Unknown Protein (AH	29.92	30.04	28.96	40.15	32.27	102.26	166.9	124.11	131.09	4.06	0.002083
Solyc02g067830.2.1	Ribosomal protein S6	1.79	1.87	1.61	1.93	1.8	0.25	0.17	0.1	0.17	0.1	0.002098
Solyc03g111610.2.1	HAD-superfamily hydr	19.92	15.69	13.22	14.38	15.8	3.35	6.42	5.35	5.04	0.32	0.002098
Solyc08g078160.2.1	Oleosin (AHRD V1 **	13.1	17.07	33.12	15.22	19.63	1.13	3.43	1.39	1.98	0.1	0.002102
Solyc01g104580.2.1	Harpin-induced protein	27.49	27.81	22.53	24.15	25.49	7.95	8.49	2.58	6.34	0.25	0.002104
Solyc10g054850.1.1	ER lumen protein retai	17.71	21.37	10.87	13.38	15.83	3.72	5.93	1.44	3.7	0.23	0.002106
Solyc06g005520.2.1	ATP binding / serine-t	6.37	9.24	6.12	9.44	7.79	1.8	1.45	1.47	1.57	0.2	0.002114
Solyc04g080550.2.1	Phenylcoumaran benzy	1.07	2.09	2.52	2.42	2.02	19.15	26.1	20.98	22.08	10.9	0.002117
Solyc03g118820.2.1	Ras-related protein Ra	14.93	14.98	10.89	10.75	12.89	62.95	38.05	44.46	48.49	3.76	0.00212
Solyc09g075290.2.1	Ribosomal protein L18	599.35	523.43	621	649.74	598.38	184.56	209.03	204.81	199.47	0.33	0.00212
Solyc05g010400.2.1	Receptor like kinase, R	5.62	6.8	4.5	4.5	5.36	1.63	1.3	0.84	1.26	0.23	0.00212
Solyc04g050820.1.1	Inositol-3-phosphate sy	3.11	2.37	4.98	0.94	2.85	0.18	0	0.22	0.13	0.05	0.002121
Solyc06g076140.2.1	Metallothionein-like pr	2807.34	2409.65	2018.37	2612.84	2462.05	7178.74	13597.03	6006.67	8927.48	3.63	0.002121
Solyc06g008820.2.1	Sodium/hydrogen exch	5.18	5.82	8.15	4.19	5.83	1.89	1.31	0.37	1.19	0.2	0.00213
Solyc01g107850.2.1	UDP-glucosyltransfera	1.21	3.23	2.14	3.42	2.5	107.58	35.51	105.13	82.74	33.1	0.002131
Solyc07g056270.2.1	Receptor-like protein k	13.09	15	11.01	18.7	14.45	4.4	4.33	4.37	4.37	0.3	0.002147
Solyc05g054680.1.1	Pto-like, Serine/threon	1.88	1.81	1.85	2.88	2.1	0.32	0.32	0.31	0.32	0.15	0.002148
Solyc04g077640.2.1	Serine carboxypeptidas	1.88	4.4	2.82	2.65	2.94	35.81	29.45	31.47	32.24	10.98	0.002183
Solyc04g078700.2.1	Receptor-like protein k	13.58	13.34	19.19	9.97	14.02	2.38	4.7	1.91	3	0.21	0.002189
Solyc01g066720.2.1	Hypoxia induced prote	435.54	349.89	460.64	475.15	430.31	1420.03	1125.07	1097.16	1214.09	2.82	0.002193
Solyc03g113790.2.1	Mannose-1-phosphate	50.27	52.93	56.32	51.82	52.84	12.94	21.32	9.92	14.73	0.28	0.002193
Solyc07g064410.1.1	Ubiquitin-conjugating	0.4	1.06	0.7	0	0.54	60.66	34.06	15.14	36.62	67.81	0.002193
Solyc03g116020.2.1	DNA-directed RNA po	168.27	137.57	137.62	137.49	145.24	52.53	52.72	39.54	48.26	0.33	0.002196
Solyc06g005110.2.1	Ribosomal protein S23	75.97	74.73	57.78	38.17	61.66	21	13.03	16.87	16.97	0.28	0.002197
Solyc06g060400.2.1	Ribosomal protein L15	625.28	476.59	608.99	548.97	564.96	171.86	203.75	189.42	188.34	0.33	0.002201
Solyc05g052410.1.1	Ethylene-responsive tr	1.65	1.47	1.75	0.7	1.39	17.19	9.95	18.01	15.05	10.81	0.002205
Solyc07g065090.1.1	Polygalacturonase inhi	67.58	71.6	76.65	153.06	92.22	15.76	9.67	13.58	13	0.14	0.002208
Solyc12g094660.1.1	Cc-nbs-lrr, resistance	7.36	5.84	7.03	7.38	6.9	0.92	2.4	1.13	1.48	0.21	0.00221
Solyc08g081210.2.1	Protein serine/threonin	7.06	7.33	7.4	9.43	7.8	1.84	1.66	1.4	1.63	0.21	0.002219
Solyc11g042940.1.1	Carbohydrate kinase F	12.14	19.24	9.37	7.8	12.14	1.07	3.06	0.88	1.67	0.14	0.002236
Solyc12g010800.1.1	BZIP transcription fact	1.07	2.68	1.52	5.17	2.61	11.63	5.25	10.02	8.97	3.44	0.002241
Solyc02g087960.2.1	MYB transcription fac	29.95	30.06	19.66	24.52	26.05	12.19	6.97	2.91	7.36	0.28	0.002242
Solyc12g044410.1.1	Class III homeodomain	7.56	8.34	7.89	4.9	7.17	1.91	1.99	1.88	1.93	0.27	0.002252
Solyc04g017650.2.1	Mediator of DNA dam	9.59	9.91	10.95	8.17	9.65	28.31	20.83	14.84	21.33	2.21	0.002253
Solyc12g005290.1.1	Receptor-like kinase (	3.42	3.89	3.94	3.94	3.8	8.48	7.9	7.97	8.12	2.14	0.002255
Solyc03g116630.2.1	Unknown Protein (AH	17.21	19.15	18.05	14.2	17.15	124.75	209.58	141.4	158.58	9.25	0.002269
Solyc06g084540.2.1	Universal stress protei	71.4	82.87	39.41	35.44	57.28	607.54	444.01	336.94	462.83	8.08	0.002269
Solyc12g005740.1.1	Unknown Protein (AH	2.64	2.68	4.45	3.2	3.24	0	0	0	0	0	0.002269
Solyc11g008720.1.1	Beta-glucosidase G4 (	0.78	0.7	0.77	0.55	0.7	158	27.68	192.3	125.99	179.99	0.00227
Solyc11g027830.1.1	Unknown Protein (AH	14.66	14.43	8.39	8.8	11.57	2.87	2.17	1.76	2.27	0.2	0.002271
Solyc01g087590.2.1	Polyamine oxidase (AI	1.72	2.71	4.84	2.62	2.97	0	0.32	0.26	0.19	0.07	0.002272
Solyc12g017530.1.1	Unknown Protein (AH	23.56	13.18	23.03	14.28	18.51	97.35	55.95	89.54	80.95	4.37	0.002279
Solyc05g012670.1.1	Glucosyltransferase-3	1.63	1.19	1.75	2.73	1.83	0	0	0	0	0.01	0.002283
Solyc05g011970.2.1	Cytochrome P450	2.94	3.19	3.92	3.8	3.46	7.54	7.81	21.95	12.43	3.59	0.002286
Solyc06g053610.2.1	Myb-related transcripti	4.22	5.96	3.22	4.21	4.4	0.2	0.81	0.25	0.42	0.1	0.002291
Solyc01g095900.2.1	Protein TIF31 homolog	8.79	9.47	10.58	8.9	9.43	76.16	53.19	78.52	69.29	7.34	0.002324
Solyc10g051030.1.1	Ternary complex facto	12.47	17.45	11.49	16.53	14.49	3.25	5.29	1.68	3.41	0.24	0.002324
Solyc02g083380.2.1	Unknown Protein (AH	37.05	27.35	28.79	20.45	28.41	202.97	206.19	200.93	203.36	7.16	0.002341
Solyc08g069010.2.1	Pentatricopeptide repe	4.06	4.73	2.58	2.8	3.54	0.25	0.76	0.21	0.41	0.11	0.002343
Solyc05g013150.2.1	Set domain protein (AI	12.57	11.94	15.33	10.39	12.56	2.62	5.06	2.38	3.35	0.27	0.002343
Solyc12g007010.1.1	Cytosolic Fe-S cluster	34.71	39.11	37.32	20.02	32.79	12.83	11.73	6.47	10.34	0.32	0.002349
Solyc06g069850.2.1	MYB family transcript	31.07	32.62	19.97	14.32	24.49	6.49	6.68	1.04	4.74	0.19	0.002349
Solyc02g071920.2.1	Transmembrane water	25.39	28.45	11.5	17	20.59	6.59	3.51	1.8	3.97	0.19	0.002371
Solyc04g072880.2.1	RNA polymerase II ho	19.31	22.2	19.93	13.09	18.63	37.37	34.09	42.52	37.99	2.04	0.002376
Solyc05g012370.2.1	Hydrolase alpha/beta f	8.43	6.51	8.62	7.42	7.74	1.06	1.68	1.49	1.41	0.18	0.002376
Solyc05g006420.2.1	Two-component respo	36.65	52.42	66.1	61.84	54.25	14.59	18.77	7.8	13.72	0.25	0.002412
Solyc08g079760.2.1	Unknown Protein (AH	3.17	2.82	2.81	2.24	2.76	0.53	0.32	0	0.28	0.1	0.002412
Solyc12g088420.1.1	Mpv17 protein (AHRD	4.16	4.16	4.29	4.04	4.16	21.82	25.4	19.08	22.1	5.31	0.002417
Solyc04g074230.2.1	14-3-3 protein sigma g	312.81	315.12	222.71	223.33	268.49	1777.82	1004.48	1311.85	1364.72	5.08	0.00243
Solyc01g008160.2.1	Tetratricopeptide repea	30.83	22.68	33.7	36.05	30.82	52.3	58.95	77.31	62.85	2.04	0.002434
Solyc02g093270.2.1	Caffeoyl-CoA O-methy	101.54	78.77	40.04	63.36	70.93	13.6	9.96	4.04	9.2	0.13	0.002438
Solyc07g007150.1.1	Unknown Protein (AH	33.22	32.52	76.15	51.69	48.4	1.53	5.41	2.83	3.26	0.07	0.00244
Solyc08g042040.2.1	Unknown Protein (AH	12.67	9.83	14.57	12.41	12.37	1.58	3.18	1.61	2.12	0.17	0.002444
Solyc09g082510.2.1	Kinase interacting fam	14.78	18.96	27.56	26.17	21.87	4.84	6.56	5.81	5.74	0.26	0.002457

Solyc09g082130.2.1	Unknown Protein (AH	10.01	9.05	7.69	10.12	9.22	24.53	16.84	25.88	22.42	2.43	0.002462
Solyc01g090900.2.1	Unknown Protein (AH	18.57	23.91	19.57	23.47	21.38	40.85	41.2	48.23	43.43	2.03	0.002468
Solyc08g079930.1.1	Subtilisin-like proteas	0.8	0.94	1.36	0.5	0.9	0	0	0	0	0.01	0.002471
Solyc12g009940.1.1	UDP-glucosyltransfera	8.73	8.28	5.4	7.89	7.57	1.73	1.75	1.56	1.68	0.22	0.002472
Solyc01g005930.2.1	Unknown Protein (AH	4.39	6.8	7.48	3.45	5.53	13.52	12.71	9.68	11.97	2.16	0.002486
Solyc08g078820.2.1	Autophagy-related pro	74.26	88.51	65.32	66.12	73.55	515.84	384.14	325.44	408.47	5.55	0.002506
Solyc02g080670.2.1	Laccase-22 (AHRD V1	1.08	0.53	0.85	1.7	1.04	0	0	0	0	0.01	0.002519
Solyc06g082710.2.1	Nucleic acid binding p	34.24	30.68	33.93	21.31	30.04	66.58	60.08	55.74	60.8	2.02	0.002524
Solyc01g107920.2.1	Os07g0175100 protein	2.07	2.26	2.25	0.45	1.76	5.01	6.35	2.05	4.47	2.54	0.002537
Solyc12g014260.1.1	Light-dependent short	30.74	27.93	30.23	37.6	31.62	5.95	12.27	3.18	7.13	0.23	0.002538
Solyc06g062900.2.1	Transcription initiatio	16.15	15.84	21.64	17.04	17.67	3.32	6.02	5.71	5.02	0.28	0.002539
Solyc08g067180.2.1	Zinc finger (Ran-bindi	12.77	12.31	8.79	9.03	10.72	0.86	0	0	0.29	0.03	0.002544
Solyc05g050120.2.1	Malic enzyme (AHRD	498.54	444.61	536.24	476.88	489.07	110.64	214.75	212.14	179.18	0.37	0.002551
Solyc10g012170.2.1	Receptor-like kinase (	8.89	8.45	8.53	10.01	8.97	19.59	14.73	25.56	19.96	2.23	0.002551
Solyc01g007100.2.1	Digalactosyldiacylglyc	13.84	14.66	16.33	8.65	13.37	6.04	2.77	3.12	3.98	0.3	0.002558
Solyc10g079200.1.1	Mitochondrial carrier	16.6	16.35	16.72	17.85	16.88	4.29	5.59	4.39	4.76	0.28	0.002563
Solyc11g020230.1.1	Pto-like, Serine/threon	0.85	1.36	0.6	1.68	1.12	0.07	0	0.08	0.05	0.04	0.002564
Solyc08g081390.2.1	Phosphoglycerate muta	3.29	3.08	2.45	1.71	2.63	0.42	0.14	0	0.19	0.07	0.002568
Solyc07g052990.1.1	Unknown Protein (AH	8.25	11.74	3.89	8.17	8.01	16.66	24.86	41.74	27.75	3.46	0.002576
Solyc09g098030.2.1	Cytochrome P450	2.27	4.03	1.89	1.13	2.33	0.22	0	0.13	0.12	0.05	0.002583
Solyc06g071600.2.1	Kinetochore protein Sp	8.17	8.2	6.92	6.22	7.38	0.85	2.56	0.42	1.28	0.17	0.002585
Solyc04g081770.2.1	GDSL esterase/lipase	22.18	28.16	25.72	10.64	21.68	3.74	5.03	1.34	3.37	0.16	0.002629
Solyc02g092800.2.1	Manganese transport p	34.02	43.29	38.15	27.08	35.64	121.96	69.36	110.42	100.58	2.82	0.002631
Solyc05g053410.2.1	Phytochrome B2	3.53	4.97	3.05	4.49	4.01	8.31	7.76	9.28	8.45	2.11	0.002635
Solyc04g072310.2.1	Unknown Protein (AH	5.54	4.11	2.72	2.61	3.75	0	0	0	0	0	0.002642
Solyc11g006220.1.1	Myosin XI (AHRD V1	1.17	0.81	1.12	1.47	1.14	0.21	0.14	0.21	0.19	0.16	0.002654
Solyc06g082870.2.1	60S ribosomal protein	586.75	525.31	495.08	443.71	512.71	163.95	194.92	168.04	175.64	0.34	0.002663
Solyc01g014530.2.1	Receptor like kinase (	0	0.33	0	0	0.08	3.89	4.23	1.84	3.32	40.24	0.002665
Solyc08g006830.2.1	Caffeoyl-CoA O-meth	5.94	6.91	4.85	5.49	5.8	0.37	1.12	0.91	0.8	0.14	0.002665
Solyc08g075950.1.1	Growth-regulating fact	2.55	3.73	4.52	3.87	3.67	0.44	0.59	0.72	0.58	0.16	0.002665
Solyc12g014040.1.1	Chloroplast protein HC	6.78	8.12	6.46	7.38	7.18	0.42	2.76	0.52	1.23	0.17	0.002666
Solyc04g008880.2.1	UDP-N-acetylmuramo	1.78	1.13	1.5	3.6	2	0.1	0.1	0	0.07	0.03	0.002685
Solyc10g086570.2.1	Palmitoyltransferase P	31.69	51.32	51.48	89.64	56.03	3.36	16.08	3.09	7.51	0.13	0.002688
Solyc02g069970.2.1	Pto-like, Serine/threon	1.18	2.02	3.02	4.46	2.67	0.08	0.24	0.1	0.14	0.05	0.002705
Solyc04g076250.2.1	Unknown Protein (AH	10.84	8.27	8.04	7.89	8.76	18.27	24.23	24.29	22.26	2.54	0.002705
Solyc05g017980.2.1	microtubule associated	58.24	49.76	47.1	71.3	56.6	16.25	23.82	11.67	17.25	0.3	0.002708
Solyc07g065980.2.1	Alkaline alpha galacto	21.18	21.64	46.06	15.9	26.2	44.99	150.69	71.28	88.99	3.4	0.002716
Solyc10g008480.2.1	Os08g0100600 protein	2.09	2.42	1.72	2.66	2.22	6.92	6.12	8.08	7.04	3.17	0.002737
Solyc11g071240.1.1	U-box domain-contain	2.36	2.28	2.56	0.56	1.94	0.16	0	0	0.05	0.03	0.002738
Solyc11g072500.1.1	Dof zinc finger protein	11.3	16.42	22.04	11.75	15.38	4.1	5.64	2.75	4.16	0.27	0.002743
Solyc10g076540.1.1	Wall-associated recept	15.87	22.3	11.82	5.91	13.98	2.02	0	0.83	0.95	0.07	0.002747
Solyc06g008710.1.1	Cullin 1B (AHRD V1	2.66	2.21	1.78	3.64	2.57	0.36	0.51	0.26	0.38	0.15	0.002748
Solyc05g023680.2.1	E3 ubiquitin-protein li	2.99	3.61	3.19	4.78	3.64	0.16	0.55	0.19	0.3	0.08	0.002766
Solyc08g061930.2.1	Cytokinin oxidase/deh	1.28	1.36	0.9	0.54	1.02	0	0	0	0	0.01	0.002768
Solyc12g006380.1.1	1-aminocyclopropane-	2.17	2.25	3.84	5.11	3.34	0.58	0.15	0.18	0.3	0.09	0.002773
Solyc11g007680.1.1	Chloroplast outer enve	52.68	59.98	39.76	40.95	48.34	103.91	128.01	113.53	115.15	2.38	0.002796
Solyc04g071340.2.1	Fructose-1 6-bisphosp	47.01	44.25	70.07	48.71	52.51	10.78	13.08	11.52	11.79	0.22	0.002801
Solyc04g080130.2.1	Zinc finger protein (A	9.2	9.82	15.35	21.01	13.84	1.59	1.82	3.13	2.18	0.16	0.002808
Solyc03g119710.2.1	Glucose-repressible al	16.99	21.06	17	16.09	17.79	5.27	5.93	3.61	4.94	0.28	0.002821
Solyc03g007330.2.1	Cell division protease	19.15	20.08	21.73	19.05	20	4.52	8.2	6.44	6.39	0.32	0.002833
Solyc08g075120.2.1	Pollen-specific kinase	22.22	24.15	21.39	18.53	21.57	7.56	7.53	2.67	5.92	0.27	0.002837
Solyc03g006630.2.1	D-erythro-sphingosine	1.63	0.48	0.96	0.77	0.96	4.05	2.1	4.44	3.53	3.68	0.002843
Solyc01g095090.2.1	E3 ubiquitin-protein li	0.58	0.62	1.24	0.82	0.81	13.65	6.84	7.87	9.45	11.6	0.002847
Solyc06g076590.2.1	Unknown Protein (AH	15.22	11.76	19.61	17.45	16.01	5.37	5.06	7.82	6.08	0.38	0.002847
Solyc12g005010.1.1	Os04g0625000 protein	26.21	23.15	29.53	27.77	26.66	4.76	11.52	5.26	7.18	0.27	0.002851
Solyc07g053010.1.1	Cc-nbs-lrr, resistance	0.6	0.6	0.71	0.85	0.69	1.27	2.44	2.6	2.1	3.05	0.002891
Solyc01g068270.2.1	Receptor-like kinase (	58.16	61.25	85.75	102.15	76.83	21.38	28.15	18.33	22.62	0.29	0.002906
Solyc05g008810.2.1	Lipid phosphate phosp	37.79	39.99	30.12	45.94	38.46	11.22	10.65	18.85	13.57	0.35	0.002925
Solyc02g069450.2.1	Photosystem I reaction	360.3	380.57	220.26	212.7	293.46	95.81	116	71.05	94.29	0.32	0.002953
Solyc02g080810.2.1	Aminomethyltransfera	131.46	129.55	107.66	125.87	123.63	40.2	61.95	32.34	44.83	0.36	0.002963
Solyc10g086710.1.1	PAR-1b protein (AHR	1.5	2.67	0.88	1.06	1.53	9.39	7.64	23.81	13.61	8.91	0.002963
Solyc02g089940.2.1	BEL1-like homeodom	8.35	5.98	8.67	3.85	6.71	1.83	2.14	1.71	1.89	0.28	0.002972
Solyc03g031760.2.1	Homeobox-leucine zip	1.05	1.21	0.86	1.04	1.04	0	0	0	0	0.01	0.002974
Solyc12g009600.1.1	Thylakoid lumenal pro	43.15	48.42	23.98	26.98	35.63	8.59	14.01	3.1	8.57	0.24	0.002987
Solyc07g066290.2.1	Chaperone protein dna	34.69	38.2	36.9	38.09	36.97	9.88	16.58	10.04	12.17	0.33	0.002989
Solyc08g007000.2.1	Protein phosphatase 2C	1.52	2.85	2.39	3.58	2.58	0.55	0.55	0.42	0.51	0.2	0.002992
Solyc01g108750.2.1	Hydrolase alpha/beta f	0	0.75	0.33	0.4	0.37	4.08	2.06	3.07	3.07	8.3	0.002994
Solyc07g063460.1.1	Methyltransferase fam	51.56	65.53	82.61	101.6	75.32	8.91	17.25	16.31	14.16	0.19	0.003011
Solyc08g078210.2.1	Hydrolase NUDIX fan	13.08	19.35	9.57	4.69	11.67	0.89	1.8	0.55	1.08	0.09	0.003011
Solyc12g096500.1.1	CONSTANS-like prote	93.17	89.95	114.47	153.1	112.67	15.59	42.68	10.03	22.77	0.2	0.003015
Solyc12g056660.1.1	Mitochondrial carrier	13.49	12.23	11.5	14.85	13.02	0.61	3.05	1.49	1.72	0.13	0.003028
Solyc02g077880.2.1	Auxin-repressed prote	1543.4	1865.59	1983.46	1907.71	1825.04	572.53	890.47	420.35	627.78	0.34	0.003028
Solyc05g007880.2.1	Dof zinc finger protein	8.21	7.7	7.57	6.12	7.4	2.53	1.34	1.04	1.64	0.22	0.003034
Solyc02g093970.2.1	Receptor like kinase, R	7.4	7.5	6.09	6.69	6.92	1.67	2.66	0.94	1.76	0.25	0.003041
Solyc08g008100.2.1	1-aminocyclopropane-	21.63	14.76	28.71	11.47	19.14	0.29	3.82	0.54	1.55	0.08	0.003045
Solyc07g047950.1.1	GRAS family transcrip	11.72	10	12.4	19.83	13.49	4	3.35	3.24	3.53	0.26	0.003062
Solyc03g123560.2.1	Chaperone protein dna	12.35	11.8	7.46	17.04	12.16	22.15	55.97	77.44	51.85	4.26	0.003078
Solyc12g100120.1.1	Acetyl-CoA carboxyla	18.13	20.75	20.85	16.92	19.16	5.79	6.83	6.65	6.42	0.34	0.003078
Solyc02g062330.1.1	Kinesin like protein (A	0.13	0	0.38	0.09	0.15	0.57	1.1	0.64	0.77	5.13	0.003082
Solyc04g005070.2.1	Peptide transporter (A	4.71	4.09	4.07	13.98	6.71	0.19	0.09	0	0.09	0.01	0.003082
Solyc03g044160.1.1	Pto-like, Serine/threon	7.82	9.62	6.76	10.84	8.76	1.17	2.5	1.36	1.68	0.19	0.003101
Solyc12g006140.1.1	Chlorophyll a-b bindin	2.73	6.62	3.22	3.16	3.93	0.2	0.2	0	0.13	0.03	0.003114

Solyc06g082610.2.1	Receptor like kinase, R	24.03	22.04	22.63	34.29	25.75	7.92	10.3	6.59	8.27	0.32	0.003121
Solyc01g096930.2.1	Phospholipid-transport	0.9	1.15	2.26	2.55	1.71	3.09	4.08	4.91	4.03	2.35	0.003132
Solyc10g086220.1.1	Flavin oxidoreductase/	21.7	23.05	13.42	10.89	17.27	1.56	4.28	0	1.95	0.11	0.003132
Solyc11g006910.1.1	Ferredoxin (AHRD V1	0	0	0	0	0	22.42	7.25	28.06	19.24	1924.33	0.003165
Solyc12g008340.1.1	Exostosin family prote	7.72	9.05	9.8	6.08	8.16	1.42	2.31	2.13	1.95	0.24	0.003185
Solyc03g114250.2.1	Phosphoglycerate muta	23.09	23.09	16.86	17.42	20.12	4.8	8.42	3.71	5.64	0.28	0.003194
Solyc01g111580.2.1	BZIP family transcript	80.82	83.41	84.44	66.11	78.69	29.11	30.75	25.85	28.57	0.36	0.003208
Solyc03g122190.2.1	Jasmonate ZIM domai	164.08	103.98	194.08	129.44	147.89	9.28	55.67	17.11	27.35	0.18	0.003213
Solyc04g075000.1.1	Serine/threonine protei	4.78	4.73	4.23	7.7	5.36	1.29	1.84	0.4	1.18	0.22	0.003218
Solyc09g082060.2.1	Cysteine synthase (AH	281.89	260.43	276.47	322.03	285.2	77.17	124.01	63.07	88.08	0.31	0.003226
Solyc05g044480.2.1	Chromodomain-helicas	6.75	8.44	8.83	6.29	7.58	1.44	2.96	1.25	1.88	0.25	0.003231
Solyc09g010710.2.1	Unknown Protein (AH	4.33	1.93	2.3	2.45	2.75	0.17	0.18	0	0.12	0.04	0.003234
Solyc10g077130.1.1	Atcambp25-binding pr	12.81	16.25	24.56	29.9	20.88	2.33	4.44	6.04	4.27	0.2	0.003234
Solyc04g005520.2.1	Aspartate/glutamate/ur	7.44	4.48	1.66	4.55	4.53	0.49	0.33	0.2	0.34	0.08	0.003247
Solyc08g005490.2.1	Dehydration-responsiv	190.63	185.57	162.04	217.68	188.98	42.87	83.6	25.19	50.55	0.27	0.003248
Solyc12g038670.1.1	Os03g0169000 protein	22.33	21.37	22.39	25.93	23.01	6.36	9.15	6.02	7.18	0.31	0.003252
Solyc02g021060.2.1	Uracil DNA glycosylas	13.15	10.06	12.12	10.17	11.38	0.83	3.52	2.04	2.13	0.19	0.003281
Solyc01g087940.1.1	Serine carboxypeptidas	0	0	0	0	0	6.81	3.16	8.63	6.2	620	0.003285
Solyc01g097200.1.1	Ankyrin repeat protein	1.11	1.97	1.96	0	1.26	8.96	6.32	9.9	8.39	6.66	0.003292
Solyc08g076510.2.1	Unknown Protein (AH	23.22	15.88	18.27	14.76	18.03	29.1	43.88	40.43	37.8	2.1	0.003302
Solyc03g007930.2.1	Unknown Protein (AH	11.2	11.08	11.75	15.85	12.47	0	0	1.24	0.41	0.03	0.003303
Solyc12g096070.1.1	Growth-regulating fact	3.05	3.56	6.07	6.47	4.79	14.02	9.79	14	12.6	2.63	0.003311
Solyc12g014360.1.1	Os06g0207500 protein	2.04	2.47	1.31	1.31	1.78	0	0.15	0	0.05	0.03	0.003324
Solyc03g097600.2.1	MtN3-like protein (AH	0	0	0	0	0	1.62	5.43	0	2.35	235	0.003336
Solyc11g010250.1.1	Avr9/Cf-9 rapidly elici	13.47	12.82	8.22	17.74	13.06	0.75	1.14	3.69	1.86	0.14	0.003344
Solyc06g064940.2.1	Phosphatidylinositol tr	27.48	27.79	31.08	29.87	29.05	43.94	145.45	79.57	89.65	3.09	0.003346
Solyc04g005200.2.1	2-succinyl-6-hydroxy-2	2.13	3.31	6.59	4.52	4.14	0.64	0	0.26	0.3	0.07	0.003366
Solyc08g075520.2.1	Binding protein (AHRI	25.88	40.24	41.77	21.12	32.25	8.5	11.47	8.04	9.34	0.29	0.003367
Solyc00g227860.1.1	UDP-glucosyltransfera	1.26	1.87	1.65	1.58	1.59	0	0.23	0.14	0.12	0.08	0.003375
Solyc05g041530.2.1	Dienelactone hydrolase	58.49	59.62	54.16	53.87	56.53	14.84	28.4	12.32	18.52	0.33	0.003375
Solyc10g086040.1.1	Mitochondrial import i	56.63	26.5	38.57	28.9	37.65	84.43	93.24	66.64	81.44	2.16	0.003377
Solyc05g052800.2.1	60S ribosomal protein	894.07	795.31	539.38	583.39	703.04	314.57	283.82	173.55	257.31	0.37	0.003377
Solyc10g083690.2.1	Cytochrome P450	0.79	1.41	1.09	0.75	1.01	21.64	21.07	15.32	19.34	19.15	0.003377
Solyc09g092260.2.1	Chaperone protein dna	54.85	108.85	84.39	128.96	94.26	22.46	33.52	4.41	20.13	0.21	0.003385
Solyc11g044560.1.1	BZIP transcription fact	90.71	103.63	75.11	75.59	86.26	26.65	33.2	20.63	26.83	0.31	0.003394
Solyc08g081060.2.1	Os10g0550700 protein	7.69	9.33	8.85	13.47	9.83	3.26	2.98	1.81	2.68	0.27	0.003397
Solyc02g070390.2.1	PTAC12 (AHRD V1 *	20.58	21.09	18.69	11.11	17.87	35.26	32.72	40.97	36.32	2.03	0.003404
Solyc06g069430.2.1	MADS box transcriptio	48.54	42.25	63.91	44.76	49.86	684.65	425.03	558.2	555.96	11.15	0.003405
Solyc04g016290.2.1	Unknown Protein (AH	4.46	3.97	5.9	2.15	4.12	0.44	1.24	0.32	0.67	0.16	0.003406
Solyc09g074340.2.1	Rho GTPase activating	2.84	3.37	3.72	2.9	3.21	0.64	0.26	0.16	0.35	0.11	0.003411
Solyc09g092320.2.1	RNA binding protein-l	56.39	55.7	36.59	27.12	43.95	6.38	16.54	7.28	10.07	0.23	0.003421
Solyc02g088170.1.1	Pentatricopeptide repe	8.92	9.89	5.91	8.81	8.38	2.37	2.29	1.33	2	0.24	0.003434
Solyc06g049040.2.1	BZIP transcription fact	3.78	4.42	4.69	9.28	5.54	0.32	0.49	0.2	0.34	0.06	0.003438
Solyc04g082610.2.1	Glutamate-gated kaina	5.75	6.57	8.13	8.14	7.15	2.07	2.2	1.48	1.92	0.27	0.003443
Solyc08g062990.1.1	Calcium-dependent pro	39.21	31.62	37.01	30.02	34.47	6.71	15.04	3.21	8.32	0.24	0.003452
Solyc01g008250.2.1	Thioredoxin X (AHRD	48.67	54.86	30.62	37.73	42.97	14.56	17.33	10.73	14.21	0.33	0.003456
Solyc03g025410.2.1	Dehydrogenase/reducta	26.26	26.93	16.11	16.94	21.56	3.06	10.96	3.75	5.92	0.27	0.003456
Solyc08g076530.2.1	RNA recognition moti	27.37	29.98	31.74	37.39	31.62	7.56	12.01	6.5	8.69	0.27	0.003467
Solyc01g079150.2.1	AE family transporter	2.84	3.66	2.27	2.07	2.71	0.59	0.6	0.36	0.52	0.19	0.00347
Solyc02g086260.1.1	Unknown Protein (AH	53.98	35.46	62.67	52.72	51.21	10.73	19.9	19.56	16.73	0.33	0.003474
Solyc01g095920.2.1	O-methyltransferase fa	2.03	0.77	0.34	0	0.78	53.3	39.14	33.32	41.92	53.4	0.003483
Solyc08g062960.2.1	Heat stress transcriptio	20.99	15.35	9.07	12.19	14.4	22.41	65.21	57.11	48.24	3.35	0.003484
Solyc11g011030.1.1	Pto-responsive gene 1	52.72	41.02	66.62	10.19	42.64	0	9.16	0.57	3.24	0.08	0.003505
Solyc12g005630.1.1	Cytochrome b6-f comp	293.17	241.69	236.85	226.85	249.64	90.29	106.82	67.46	88.19	0.35	0.003522
Solyc02g092510.2.1	UPF0553 protein C9or	9.46	8.61	12.42	9.73	10.05	1.22	2.63	1.07	1.64	0.16	0.003522
Solyc10g011770.2.1	Threonine endopeptida	124.18	123.99	76	79.04	100.8	39.5	42.37	32.74	38.2	0.38	0.003528
Solyc07g062500.2.1	Cytochrome P450	7.56	15.05	11.79	4.89	9.82	0.83	3.13	0	1.32	0.13	0.003531
Solyc08g076310.2.1	Uncharacterized memb	11.4	12.88	18.03	14.18	14.12	3.66	5.8	4.65	4.7	0.33	0.003534
Solyc12g056760.1.1	Unknown Protein (AH	6.03	4.47	5.93	10.42	6.71	0.81	0.82	0.66	0.76	0.11	0.003543
Solyc04g016000.2.1	Heat stress transcriptio	3.05	3.7	6.87	2.35	3.99	0	0.9	0.28	0.39	0.1	0.003551
Solyc08g083130.2.1	Homeobox leucine zip	46.88	33.68	43.85	9.47	33.47	67.1	170.28	76.12	104.5	3.12	0.003552
Solyc03g095240.2.1	SET and MYND doma	4.1	3.83	3.69	4.29	3.98	1.03	0.72	0.97	0.91	0.23	0.003559
Solyc04g049920.2.1	RNA binding protein-l	3.27	1.21	1.93	2.31	2.18	0	0	0.27	0.09	0.04	0.003565
Solyc03g120980.2.1	ATP-binding cassette t	1.58	3.09	5.34	4.1	3.53	257.47	101.82	115.48	158.26	44.86	0.003582
Solyc08g075680.2.1	Unknown Protein (AH	5.21	3.99	2.05	3.48	3.68	0.47	1.06	0.43	0.65	0.18	0.003585
Solyc05g006970.2.1	Small multidrug expor	10.05	9.32	7.91	5.04	8.08	1.86	2.39	1.66	1.97	0.24	0.00361
Solyc08g077550.2.1	Novel plant SNARE 1	10.07	8.74	2.97	13.18	8.74	0.41	1.85	0.25	0.84	0.1	0.003623
Solyc02g078410.1.1	Acyl-CoA thioesterase	23.88	27.74	26.26	17.75	23.91	51.66	67.28	44.19	54.38	2.27	0.003627
Solyc05g013500.2.1	Glycosyltransferase-lik	0.68	0.8	0.89	1.06	0.86	0.06	0	0.22	0.09	0.11	0.003659
Solyc03g120590.2.1	Peptide transporter 1 (	1.85	1.37	1.45	1.31	1.5	7.72	4.02	19.28	10.34	6.92	0.003676
Solyc08g029090.2.1	B3 domain-containing	10.6	7.03	8.25	7.18	8.27	1.28	2.21	0.9	1.46	0.18	0.003706
Solyc01g103940.2.1	Serine/threonine protei	332.77	305.25	203.61	223.49	266.28	99.53	123.26	79.77	100.85	0.38	0.003716
Solyc09g089640.2.1	At5g51670-like protei	1.05	1.4	0.62	1.77	1.21	3.13	2.81	2.39	2.78	2.29	0.003721
Solyc05g008920.2.1	Os08g0100600 protein	28.94	35.4	35.67	20.81	30.21	123.32	85.64	119.89	109.62	3.63	0.003727
Solyc06g073660.2.1	Non-specific lipid-tran	7.45	8.84	4.6	9.03	7.48	0.57	1.73	0.7	1	0.13	0.003731
Solyc01g014100.2.1	Unknown Protein (AH	5.21	7.73	7.17	7.37	6.87	0	0	0	0	0	0.003746
Solyc09g014300.2.1	Glycosyl transferase gr	17.83	16.21	14.12	17.49	16.41	52.35	31.49	41.98	41.94	2.56	0.003761
Solyc10g052650.1.1	Unknown Protein (AH	5.5	5.87	5.84	7.78	6.25	0.44	0.45	0	0.3	0.05	0.003762
Solyc09g011060.2.1	Receptor like protein k	0.87	1.41	1.12	0.9	1.07	0	0.13	0	0.04	0.04	0.003765
Solyc10g005310.2.1	Adenosine deaminase-	39.19	43.68	45.6	15.76	36.06	6.54	11.57	3.57	7.23	0.2	0.003797
Solyc06g051170.2.1	RNA-dependent RNA	4.58	2.96	7.3	7.29	5.53	0.74	0.75	2.16	1.22	0.22	0.003823
Solyc06g082500.2.1	Solute carrier family 3	20.74	32.77	12.79	17.45	20.94	4.23	5.48	2.97	4.23	0.2	0.003828

Solyc12g017630.1.1	Nucleolar essential pro	25.54	26.52	35.99	20.74	27.2	6.11	9.44	3.52	6.36	0.23	0.003837
Solyc01g111280.2.1	Cold shock protein-1 (	65.2	50.18	66.97	69.44	62.95	14.56	23.75	14.85	17.72	0.28	0.003843
Solyc05g013870.2.1	Sieve element-occludin	6.1	6.24	4.04	2.13	4.63	0.34	1.5	0.17	0.67	0.14	0.003843
Solyc11g071690.1.1	Cellular nucleic acid b	24.49	31.32	32.21	20.25	27.07	9.19	8.94	8.1	8.74	0.32	0.003852
Solyc11g040150.1.1	Heparan-alpha-glucosa	22.4	26.18	18.65	29.36	24.15	3.89	10.19	2.32	5.47	0.23	0.003853
Solyc01g102820.2.1	2-C-methyl-D-erythrito	32.77	33.55	23	23.63	28.24	6.58	12.74	9.35	9.56	0.34	0.003867
Solyc12g096100.1.1	ABC-type Co2+ transp	28.56	21.49	23.67	28.38	25.52	6.59	10.25	6.08	7.64	0.3	0.003881
Solyc11g069940.1.1	Glutaredoxin (AHRD V	109.34	94.02	264.52	251.23	179.78	10.44	16.92	16.03	14.46	0.08	0.003881
Solyc04g015040.2.1	Peptidyl-prolyl cis-tran	35.22	29.36	24.08	39.15	31.95	7.68	14.12	9.16	10.32	0.32	0.003883
Solyc04g051500.2.1	Integral membrane pro	36.06	40.55	39.97	31.67	37.06	11.52	16.3	8.85	12.22	0.33	0.003896
Solyc06g054320.1.1	Disease resistance resp	66.33	60.3	42.49	164.94	83.52	3.17	4.94	3.89	4	0.05	0.003918
Solyc06g007850.2.1	Pentatricopeptide repe	9.23	9.56	6.96	5.99	7.94	1.22	2.47	0.75	1.48	0.19	0.003925
Solyc03g115380.1.1	UDP-glucose dehydrog	3.29	1.1	4.37	2.33	2.77	0	0.56	0.14	0.23	0.08	0.003937
Solyc01g099140.2.1	Transmembrane protei	0.62	1.66	2.2	0.88	1.34	9.53	5.82	6.01	7.12	5.31	0.003942
Solyc06g051630.2.1	1-phosphatidylinositol-	10.22	6.52	5.37	11.31	8.36	2.42	1.9	0.77	1.7	0.2	0.003975
Solyc06g050370.1.1	Unknown Protein (AH	18.19	18.72	3.79	15.13	13.96	1.44	2.32	2.12	1.96	0.14	0.003975
Solyc03g118810.1.1	Calmodulin (AHRD V	5.6	6.05	5.66	19.79	9.27	25.84	16.94	59.93	34.24	3.69	0.00398
Solyc02g087090.2.1	Prostaglandin G/H synt	0	0.18	0	0	0.04	2.07	4.35	1.93	2.78	61.85	0.003997
Solyc12g015850.1.1	Solute carrier family 3	8.18	9.1	6.44	8.44	8.04	1.51	3.19	1.18	1.96	0.24	0.004006
Solyc08g081650.1.1	Ethylene-responsive tr	2.47	1.81	3.68	1.64	2.4	0.64	0.3	0.14	0.36	0.15	0.004022
Solyc01g105180.2.1	LIM zinc-binding prote	18.78	13.55	9.38	13.4	13.78	1.91	1.79	1.68	1.79	0.13	0.004022
Solyc04g011400.2.1	UDP-glucose 4-epimer	713.22	660.19	728.54	752.77	713.68	248.06	303.32	221.76	257.71	0.36	0.004023
Solyc08g077490.1.1	Vacuolar protein sortin	0.5	0.98	2.01	0.57	1.01	2.59	2.21	1.99	2.26	2.23	0.004024
Solyc11g071430.1.1	Cc-nbs-lrr, resistance p	2.78	3.33	3.78	3.77	3.42	0.91	0.87	0.79	0.86	0.25	0.004024
Solyc02g076660.2.1	Receptor like kinase, R	8.5	8.63	10.57	9.93	9.41	4.25	2.26	3.67	3.39	0.36	0.004057
Solyc01g080750.2.1	HEAT repeat family pr	1.55	2.17	1.83	1.26	1.7	0	0	0	0	0.01	0.004058
Solyc02g087580.2.1	cDNA clone J023121N	3.26	3.93	1.59	5.3	3.52	0.08	0.16	0	0.08	0.02	0.004062
Solyc07g066230.2.1	Receptor like kinase, R	5.94	6.79	6.74	6.56	6.51	2.51	1.89	2.47	2.29	0.35	0.004079
Solyc03g114800.1.1	Sulfotransferase family	41	39.08	14.49	17.37	27.98	3.36	3.56	3.92	3.61	0.13	0.004085
Solyc05g010420.1.1	S-adenosylmethionine	814.31	851.92	1278.73	1222.15	1041.78	4377.71	2708.43	4470.2	3852.11	3.7	0.004088
Solyc03g115020.2.1	Unknown Protein (AH	300	298.82	230.02	209.57	259.6	104.81	101.21	68.41	91.48	0.35	0.004089
Solyc04g077160.1.1	Unknown Protein (AH	72.3	76.09	49.19	50.76	62.08	22.17	18.06	5.76	15.33	0.25	0.004089
Solyc10g083500.1.1	Protein kinase (AHRD	9.12	7.65	15.09	15.77	11.91	1.75	2.89	2.54	2.39	0.2	0.004097
Solyc09g092670.2.1	Cytochrome P450	19.51	27.64	18.79	12.2	19.54	7.4	6.71	3.69	5.93	0.3	0.004148
Solyc08g060970.2.1	Polygalacturonase (AH	5.47	4.43	2.74	3.99	4.16	0.4	0.27	1.15	0.61	0.15	0.00415
Solyc04g009440.2.1	NAC domain protein (	35.39	27.41	21.39	29.97	28.54	5.65	9.79	5.85	7.1	0.25	0.004154
Solyc05g056120.2.1	GATA transcription fa	61.35	49.94	70.71	100.14	70.53	13.65	21.31	14.57	16.51	0.23	0.004159
Solyc08g079910.1.1	Subtilisin-like protease	0.62	1.26	1.25	0.13	0.81	0	0	0	0	0.01	0.00418
Solyc11g072000.1.1	Disease resistance prot	54.55	90.33	94.3	69.99	77.29	15.89	32.56	19.52	22.66	0.29	0.004202
Solyc11g007990.1.1	Malate dehydrogenase	162.92	173.49	135.46	113.96	146.46	52.58	63.34	45.82	53.91	0.37	0.004202
Solyc12g088220.1.1	Branched-chain-amino	0	0.31	1.02	0.24	0.39	121.99	31.32	154.68	102.66	261.56	0.004203
Solyc07g008820.2.1	Os12g0554100 protein	4.86	4.58	2.7	6.06	4.55	0.58	0.93	0.57	0.69	0.15	0.004207
Solyc10g080730.1.1	Thioredoxin family pro	5.11	4.32	8.45	3.62	5.38	0.62	1.46	0.76	0.95	0.18	0.004214
Solyc02g084630.2.1	MADS-box transcriptio	256	250.18	123.64	144.52	193.59	54.71	58.99	22.89	45.53	0.24	0.004251
Solyc06g066410.2.1	Profilin (AHRD V1 **	0	0	0	0	0	3.23	2.44	1.49	2.39	238.67	0.00426
Solyc08g069180.2.1	Receptor-like protein k	1.91	1.79	2.82	1.89	2.1	0.23	0.39	0.28	0.3	0.14	0.004295
Solyc07g062170.1.1	Receptor protein kinas	5.1	5.7	3.09	2.27	4.04	0.94	0.95	1.01	0.97	0.24	0.004299
Solyc12g007300.1.1	B3 domain-containing	0.28	0.75	2.97	1.98	1.5	4.07	6.61	6.66	5.78	3.87	0.004326
Solyc04g008330.1.1	Glucosyltransferase (A	10.31	5.37	18.24	1.46	8.85	0	0.12	0.15	0.09	0.01	0.004358
Solyc10g078420.1.1	Unknown Protein (AH	11.7	13.26	27.62	16.56	17.29	47.28	31.21	59.15	45.88	2.65	0.004374
Solyc01g091140.2.1	Nitroreductase (AHRD	6.22	4.23	7.15	6.25	5.96	8.46	12.94	15.92	12.44	2.09	0.004382
Solyc06g065910.2.1	At1g62390-like protein	0	0	0	0	0	0.7	0.47	0.19	0.45	45.33	0.004382
Solyc11g062280.1.1	Glyoxalase/bleomycin	21.99	25.59	21.29	15.15	21.01	6.83	2.3	3.92	4.35	0.21	0.004392
Solyc03g071570.2.1	Pectate lyase-like prote	22.99	20.46	14.49	44.53	25.62	1.69	3.41	1.04	2.05	0.08	0.004398
Solyc02g072180.2.1	Ras-related protein Ra	40.55	35.55	40.7	71.06	46.97	10.02	13.07	9.01	10.7	0.23	0.004406
Solyc03g043640.2.1	Metal ion binding prote	0	0.79	1.05	1.26	0.78	5.04	6.17	2.65	4.62	5.96	0.004428
Solyc10g006950.1.1	Unknown Protein (AH	9.96	8.86	14.69	5.28	9.7	42.24	26.37	27.18	31.93	3.29	0.004447
Solyc03g005960.2.1	Receptor like kinase, R	0.55	1.78	1.3	0.2	0.96	0	0	0	0	0.01	0.004448
Solyc11g062350.1.1	Plastocyanin-like doma	1.96	3.73	2.31	0.4	2.1	0	0	0	0	0	0.004448
Solyc07g052400.2.1	Glutamate-gated kaina	3.25	5.64	9.3	8.39	6.65	0.53	0.93	0.24	0.57	0.09	0.004487
Solyc01g028800.1.1	Pumilio-family RNA b	0.71	1.03	1.78	0.88	1.1	0.22	0	0.53	0.25	0.23	0.004505
Solyc01g090240.2.1	Protein kinase 5 (AHR	6.21	6.28	7.99	17.44	9.48	0.23	0.54	0.09	0.29	0.03	0.004525
Solyc10g012320.1.1	Ubiquitin-conjugating	4.89	2.61	2.31	4.84	3.66	10.66	15.13	7.28	11.02	3.01	0.004533
Solyc11g069570.1.1	Cytokinin riboside 5&a	7.58	7.75	5.3	2.78	5.85	0.45	1.83	0.84	1.04	0.18	0.004535
Solyc04g016460.2.1	Unknown Protein (AH	0	0	0	0	0	193.45	65.7	133.39	130.85	13084.67	0.004545
Solyc09g010690.2.1	Ankyrin repeat domain	157.63	142.89	232.12	264.05	199.17	33.49	60.06	33.9	42.48	0.21	0.004545
Solyc09g083210.2.1	Receptor-like protein k	31.52	34.02	33.85	52.88	38.07	4.57	5.63	4.89	5.03	0.13	0.004545
Solyc11g011840.1.1	Oxidoreductase family	15.81	18.12	12.44	8.74	13.78	4.55	5.48	2.71	4.25	0.31	0.004548
Solyc06g062690.2.1	Nucleosome assembly	5.84	8.56	8.9	8.8	8.03	14.01	32.1	14.97	20.36	2.54	0.004561
Solyc07g025370.2.1	Cytochrome P450	16.49	22.72	25.17	17.03	20.35	4.23	8.53	2.99	5.25	0.26	0.004562
Solyc04g082900.2.1	Mitotic checkpoint ser	1.35	1.35	2.59	2.15	1.86	9.98	3.58	9.23	7.6	4.08	0.004599
Solyc05g007910.2.1	50S ribosomal protein	18.52	14.76	8.65	14.73	14.16	1.87	3.46	2.3	2.54	0.18	0.004604
Solyc12g070120.1.1	Unknown Protein (AH	3.26	5.8	2.88	1.15	3.27	19.74	55.75	8.08	27.86	8.51	0.004613
Solyc04g057890.2.1	Ganglioside-induced d	29.18	31.87	34.72	19.08	28.71	75.29	51.56	65.73	64.19	2.24	0.004618
Solyc08g075020.2.1	Pectinacetylesterase lik	5.51	4.33	2.49	3.67	4	121.66	63.53	120.02	101.74	25.43	0.004667
Solyc12g044230.1.1	Os01g0786800 protein	34.6	45.32	37.17	42.18	39.82	13.41	11.23	10.89	11.84	0.3	0.004675
Solyc03g080180.2.1	O-methyltransferase (A	5.34	7.05	8.7	4.17	6.31	1.34	1.5	0.91	1.25	0.2	0.004676
Solyc01g109780.2.1	Unknown Protein (AH	19.88	22.61	36.01	24.15	25.66	96.94	65.62	61.99	74.85	2.92	0.0047
Solyc06g051270.2.1	GDP-D-mannose pyrop	116.82	115.81	128.79	99.76	115.29	39.16	48.86	30.57	39.53	0.34	0.004714
Solyc04g078780.2.1	WD repeat protein 18 (	9.26	8.37	11.61	10.18	9.86	2.02	2.63	1.75	2.13	0.22	0.004721
Solyc05g053440.2.1	60S ribosomal protein	1358.36	1178.13	1020.75	1020.36	1144.4	472.7	452.09	347.81	424.2	0.37	0.004721
Solyc06g052010.1.1	IQ-domain 21 (AHRD	9.28	7.38	4.5	10.79	7.99	1.74	1.49	1.65	1.63	0.2	0.004757

Solyc07g017650.2.1	Alpha-aminoadipic sei	13.69	16.06	9.91	10.12	12.44	24.14	23.08	27.8	25.01	2.01	0.004758
Solyc02g093440.2.1	GHMP kinase family p	24.04	25.87	24.4	32.42	26.68	8.43	11.24	8.69	9.45	0.35	0.004777
Solyc01g087730.2.1	50S ribosomal protein	149.65	142.37	136.61	109.83	134.62	36.16	68.51	48.48	51.05	0.38	0.004783
Solyc11g065520.1.1	Unknown Protein (AH	17.8	14.86	15.57	10.57	14.7	5.01	4.15	8.99	6.05	0.41	0.004783
Solyc03g120910.2.1	Class III homeodomair	10.8	10.17	7.25	7.14	8.84	1.21	1.8	0.63	1.21	0.14	0.004787
Solyc01g108200.2.1	cDNA clone J013057C	8.41	7.77	9.54	9.61	8.83	1.83	2.11	2.57	2.17	0.25	0.004791
Solyc02g067690.2.1	Glucosyltransferase-lik	97.69	87.87	59.74	83.42	82.18	17.33	37.86	13.3	22.83	0.28	0.004791
Solyc05g054760.2.1	Dehydroascorbate redu	280.46	295.52	262.27	335.23	293.37	112.91	116.18	93.41	107.5	0.37	0.004801
Solyc02g072460.1.1	Ycf20-like protein (AH	4.68	3.41	2.51	3.61	3.55	8.94	11.45	9.72	10.04	2.83	0.004802
Solyc06g082190.2.1	Serine/threonine/tyrosi	4.96	4.41	5.59	9.35	6.08	23.67	16.33	19.24	19.75	3.25	0.004804
Solyc02g087100.1.1	Alpha-dioxygenase (A	0	0	0	0	0	12.51	24.14	18.71	18.45	1845.33	0.004811
Solyc12g014270.1.1	Peptide-N4-(N-acetyl-l	2.46	2.52	4.65	6.62	4.06	0.2	0.7	0.24	0.38	0.09	0.004839
Solyc05g055780.2.1	Protein recA (AHRD V	7.09	8.55	10.4	7.35	8.35	1.53	3.34	1.09	1.99	0.24	0.004857
Solyc09g066150.1.1	Cytochrome P450	9.84	11.32	10.99	12.43	11.14	310.47	129.21	568.38	336.02	30.15	0.004857
Solyc02g037500.1.1	Unknown Protein (AH	5.41	0.96	6.38	7.65	5.1	25.34	21.15	42.94	29.81	5.85	0.004861
Solyc11g066080.1.1	Genomic DNA chromo	11.19	10.39	7.59	10.44	9.9	2.5	1.94	3.31	2.58	0.26	0.004876
Solyc10g085220.1.1	Mitochondrial carrier p	10.49	12.72	11.83	12.53	11.89	3.45	4.94	2.93	3.77	0.32	0.004883
Solyc12g088370.1.1	Interferon-related deve	7.02	4.38	4.23	5.28	5.23	15.43	10.09	14.52	13.35	2.55	0.004887
Solyc01g111010.2.1	Aquaporin (AHRD V1	1.01	3.04	1.49	1.07	1.65	0.2	0	0.13	0.11	0.07	0.004899
Solyc05g005280.2.1	Poly polymerase cataly	4.33	4.04	5.36	5.2	4.73	58.37	47.24	4.29	36.63	7.74	0.004903
Solyc03g065250.2.1	CER1 (AHRD V1 **--	12.73	12.08	16.14	9.08	12.51	124.53	75.02	64.33	87.96	7.03	0.004918
Solyc02g070940.1.1	Chlorophyll a/b bindin	88.29	71.07	59.98	33.68	63.26	25.05	6.67	4.68	12.13	0.19	0.00494
Solyc02g078380.2.1	Aluminum-induced pro	81.04	62.41	50.25	29.76	55.86	11.54	17.99	8.25	12.59	0.23	0.004948
Solyc02g069130.2.1	Peptidyl-prolyl cis-tran	54.37	57.63	42.52	35.43	47.49	16.53	20.66	10.61	15.93	0.34	0.004951
Solyc06g069380.2.1	Os03g0169000 protein	4.53	5.46	3.3	6.03	4.83	1.83	0.98	0.26	1.02	0.21	0.004953
Solyc09g075300.2.1	Epoxide hydrolase 2 (A	27.3	23.56	27.08	47.68	31.41	8.19	8.26	8.42	8.29	0.26	0.004966
Solyc09g091800.2.1	Unknown Protein (AH	41.62	84.33	5.22	16.91	37.02	0.36	0	0	0.12	0	0.004966
Solyc11g062390.1.1	Os03g0364500 protein	29.45	30.67	35.79	26.66	30.64	8.57	11.04	7.02	8.88	0.29	0.004969
Solyc07g007140.2.1	Serine/threonine-prote	3.59	2.82	5.55	4.22	4.04	22.02	10.25	18.08	16.78	4.15	0.004993
Solyc01g109330.2.1	Cytochrome c biogene	0.43	0.64	0.51	0	0.4	4.87	3.04	1.71	3.21	8.12	0.005021
Solyc02g067840.2.1	Sucrase/ferredoxin dor	26.19	24.35	23.81	36.23	27.65	64.74	46.48	77.62	62.95	2.28	0.005048
Solyc08g042100.2.1	Armadillo/beta-catenin	3.43	2.56	3.56	9.12	4.67	0	0	0	0	0	0.005048
Solyc04g007350.2.1	Unknown Protein (AH	3.55	5.42	6.59	11.13	6.67	20.5	18.82	14.1	17.81	2.67	0.005056
Solyc06g084120.2.1	Protein TIFY 4B (AHR	6.8	6.61	4.62	4.96	5.75	0.83	0.34	0.61	0.59	0.1	0.005084
Solyc09g072900.2.1	Dienelactone hydrolase	58	46.97	48.32	53.66	51.74	14.66	21.73	14.73	17.04	0.33	0.005087
Solyc07g008360.1.1	p-coumarate CoA-ligas	3.48	3.61	1.5	0.82	2.35	0.37	0	0.12	0.16	0.07	0.005103
Solyc09g063070.2.1	Senescence-associated	30.45	26.65	22.75	21.89	25.43	3.89	9.51	3.27	5.56	0.22	0.005131
Solyc08g075990.2.1	Os02g0129300 protein	7.4	12.61	7.73	7.18	8.73	2.22	2.07	2.31	2.2	0.25	0.005144
Solyc02g064800.2.1	Histone-binding protei	19.49	22.35	16.37	10.6	17.2	4.75	7.42	4.14	5.44	0.32	0.005151
Solyc01g095220.2.1	Unknown Protein (AH	92.96	133.44	86.68	67.87	95.24	26.65	30.54	25.3	27.5	0.29	0.005156
Solyc08g014440.2.1	CREB-binding protein	7.81	10.82	12.08	4.91	8.91	1.54	1.98	1.38	1.63	0.18	0.005164
Solyc06g064440.2.1	Ring finger protein 12	51.54	48.93	45.67	64.15	52.57	18.69	24.08	11.37	18.05	0.34	0.005168
Solyc07g052060.2.1	Acyltransferase 1 (AH	1.95	2.6	1.15	5.51	2.8	0	0	0.24	0.08	0.03	0.005168
Solyc03g112580.2.1	Receptor like kinase, R	2.56	1.86	2.81	5.32	3.14	0.1	0.34	0.18	0.21	0.07	0.005173
Solyc10g009370.2.1	Mutator-like transposa	0.45	0.67	0.18	0.85	0.54	2.56	4.55	1.5	2.87	5.34	0.00518
Solyc06g051990.2.1	At5g24280-like protein	1.97	2.38	1.5	2.19	2.01	0	0.34	0.14	0.16	0.08	0.005194
Solyc04g079680.2.1	Cytochrome P450	11.9	13.3	10.91	13.97	12.52	2.05	4.75	2.26	3.02	0.24	0.005224
Solyc02g021430.1.1	Mitochondrial transcri	4.73	4.78	5.83	3.65	4.75	0.87	0.53	0	0.47	0.1	0.005235
Solyc04g076010.2.1	Zinc ion binding protei	216.56	327.08	199.67	155.85	224.79	44.37	55.73	45.25	48.45	0.22	0.005235
Solyc06g074420.1.1	Cytochrome P450	0.41	1.57	1.44	0.58	1	0	0	0	0	0.01	0.005241
Solyc05g013280.2.1	Cc-nbs-lrr, resistance p	5.2	7.81	7.2	7.15	6.84	1.98	1.74	1.61	1.78	0.26	0.005251
Solyc03g121440.2.1	Receptor-like protein k	10.18	9.55	14.14	13.51	11.85	2.84	4.63	1.25	2.91	0.25	0.005282
Solyc03g097440.2.1	11-beta-hydroxysteroid	0.19	0.67	1.1	0.53	0.62	155.37	50.86	111.3	105.84	170.03	0.005293
Solyc01g073710.2.1	Calcium-binding EF ha	25.92	31.87	18.9	21.33	24.5	6.85	7.68	5.61	6.71	0.27	0.005295
Solyc07g008560.2.1	Purple acid phosphatas	3.21	3.04	3.3	1.61	2.79	0.42	0.76	1.13	0.77	0.28	0.005307
Solyc01g095170.2.1	Harpin-induced protein	16.27	21.99	12.69	5.76	14.18	33.81	52.1	18.46	34.79	2.45	0.005308
Solyc11g006720.1.1	MYB transcription fac	12.82	13.36	13.65	15.04	13.72	25.77	20.64	49.34	31.92	2.33	0.005319
Solyc06g005000.2.1	IBR finger domain-cor	4.63	0.33	3.99	6.37	3.83	0.3	0	0.37	0.22	0.06	0.005325
Solyc08g061650.2.1	F-box/LRR-repeat prot	1.91	2.05	0.62	1.3	1.47	0.16	0.37	0.07	0.2	0.14	0.005328
Solyc10g080930.1.1	Transcription elongatio	9.55	12.49	12.63	12.69	11.84	22.91	22.4	29.15	24.82	2.1	0.005344
Solyc05g015840.2.1	Squamosa promoter-bi	17.49	11.26	16.91	12.22	14.47	5.11	3.61	4.18	4.3	0.3	0.005377
Solyc06g069860.2.1	60S ribosomal protein	604.64	469.71	714.64	791.26	645.06	170.03	276.78	183.46	210.09	0.33	0.005413
Solyc03g082540.2.1	Unknown Protein (AH	23.63	28.17	25.94	43.02	30.19	2.82	11.39	4.9	6.37	0.21	0.00546
Solyc06g074110.2.1	Uncharacterized basic	3.18	2.7	3.93	4.09	3.48	0.41	1.12	1.01	0.85	0.24	0.005463
Solyc06g073530.1.1	Argonaute 4-like prote	4.08	4.02	6.02	4.23	4.59	1.12	1.13	2.03	1.43	0.31	0.00547
Solyc03g032240.2.1	Cation/H(+) antiporter	0.67	0.3	0.99	0.36	0.58	1.08	2.52	1.08	1.56	2.69	0.005488
Solyc01g108020.2.1	Thioredoxin m (AHRD	117	114.18	101.05	85.58	104.45	37.4	52.87	28.58	39.62	0.38	0.005502
Solyc12g044610.1.1	Myb transcription facto	5.97	7.34	7.45	5.21	6.49	1.13	2	0.52	1.22	0.19	0.005502
Solyc03g083530.2.1	40S ribosomal protein	1380.18	1196.3	1463.45	1448.24	1372.04	473.02	469.32	514.81	485.72	0.35	0.005568
Solyc06g049050.2.1	Expansin (AHRD V1 *	14.36	8.29	9.1	8.65	10.1	2.58	4.34	0	2.31	0.23	0.005575
Solyc05g054670.2.1	Os07g0175100 protein	2.33	2.08	0.79	1.65	1.71	3.63	5.43	4.96	4.67	2.73	0.005576
Solyc06g005500.2.1	ATP binding / serine-tl	49.48	35.11	42.92	57.64	46.29	12.67	17.26	14.61	14.85	0.32	0.005576
Solyc06g068910.2.1	Receptor like kinase, R	0.42	1.39	0.86	2.06	1.18	0	0.08	0	0.03	0.02	0.005594
Solyc09g007460.2.1	Unknown Protein (AH	2.48	2.33	0.81	1.36	1.75	0.22	0.22	0	0.15	0.08	0.005616
Solyc10g052880.1.1	LRR receptor-like serin	9.78	11.4	8.29	13.44	10.73	3.56	2.47	3.14	3.06	0.28	0.005616
Solyc09g075710.1.1	Gibberellin receptor G	6.84	3.94	1.66	1.71	3.54	0	0.16	0.4	0.19	0.05	0.005616
Solyc03g093400.2.1	Solute carrier family 2	1.6	1.67	1.11	0.8	1.29	0.08	0.15	0	0.08	0.06	0.005634
Solyc10g006800.2.1	Cyclic nucleotide gated	0.42	1.12	2.23	1.48	1.31	0.08	0.09	0	0.06	0.04	0.005655
Solyc02g083190.1.1	F-box family protein (A	5.06	3.14	4.34	10.42	5.74	0.37	0.63	0.3	0.43	0.08	0.005671
Solyc08g077330.2.1	Expansin-like protein (	0.26	0.69	1.52	0.73	0.8	8.33	2.31	5.37	5.34	6.67	0.005671
Solyc11g007200.1.1	Copper chaperone (AH	14.47	27.81	14.34	6.55	15.79	70.6	50.93	43.65	55.06	3.49	0.005717
Solyc02g079160.2.1	Unknown Protein (AH	0.99	0.44	1.17	0	0.65	5.99	3.02	7.6	5.54	8.52	0.005721

Solyc10g007570.2.1	Gibberellin 2-oxidase 1	21.76	39.96	19.15	31.27	28.04	11.16	6.47	3.09	6.91	0.25	0.005753
Solyc10g085130.1.1	Zinc finger family prot	2.46	2.92	3.88	1.94	2.8	9.07	6.47	5.43	6.99	2.5	0.005753
Solyc11g010270.1.1	Homeobox-leucine zip	188.14	255.6	247.91	318.23	252.47	77.44	97.41	66.6	80.48	0.32	0.005755
Solyc08g068690.1.1	N-acetyltransferase (A	361.34	316.71	106.9	138.23	230.79	14.82	20.52	18.75	18.03	0.08	0.005772
Solyc09g005570.2.1	Unknown Protein (AH	11.35	20.02	6.06	19.39	14.21	1.21	2.79	3.4	2.47	0.17	0.005787
Solyc01g080410.2.1	Peptide methionine sul	12.13	13.82	13.16	17.15	14.06	789.68	301.2	1323.61	804.83	57.22	0.005801
Solyc05g049980.2.1	Glycoside hydrolase fa	35.76	33.76	21.1	20.89	27.88	66.43	69.65	32.67	56.25	2.02	0.00581
Solyc12g014610.1.1	WRKY transcription fa	8.84	7.1	11.7	10.67	9.58	1.48	2.64	1.93	2.02	0.21	0.005817
Solyc01g087540.2.1	Os01g0611000 protein	23.66	25.21	29.61	39.3	29.45	2.32	9.5	2.67	4.83	0.16	0.005853
Solyc07g062740.2.1	Unknown Protein (AH	2.51	5.22	3.95	3.08	3.69	12.17	12.69	7.81	10.89	2.95	0.005866
Solyc02g094300.2.1	Uridylyltransferase PII	11.96	10.32	11.55	4.61	9.61	3.22	2.22	0.72	2.05	0.21	0.005878
Solyc07g062530.2.1	Phosphoenolpyruvate c	782.29	783.85	1187.64	764.9	879.67	288.14	411.96	189.05	296.38	0.34	0.005909
Solyc10g012270.1.1	Ubiquitin-conjugating	5.15	2.75	2.43	5.1	3.86	10.83	15.54	7.67	11.35	2.94	0.005915
Solyc02g085630.2.1	Homeobox-leucine zip	8.36	5.24	20.83	5.26	9.92	0.25	0	0	0.08	0.01	0.005919
Solyc01g111880.2.1	Tyrosine-protein kinas	3.16	2.91	7.45	5.63	4.79	1.04	0.67	0.7	0.8	0.17	0.005925
Solyc04g008200.2.1	Cc-nbs-lrr, resistance p	5.4	5.95	3.34	4.15	4.71	1.41	1.72	1.38	1.5	0.32	0.005925
Solyc06g073110.2.1	Legume lectin beta dom	26.11	25.2	35.48	32.94	29.93	8.19	9.63	6.13	7.98	0.27	0.005925
Solyc10g080450.1.1	Unknown Protein (AH	103.47	103.07	78.95	91.3	94.2	32.98	40.61	21.2	31.6	0.34	0.005953
Solyc10g047250.1.1	Unknown Protein (AH	39.58	33.92	67.45	93.32	58.57	7.11	8.36	4.37	6.61	0.11	0.00596
Solyc03g111690.2.1	Pectate lyase (AHRD V	31.19	35.02	32.56	18.25	29.26	2522.97	828.98	2938.43	2096.79	71.67	0.005964
Solyc05g047530.2.1	Cytochrome P450	0.37	0.55	0.29	0.35	0.39	4.94	7.22	6.56	6.24	16	0.006025
Solyc11g008960.1.1	Receptor like kinase, R	8.07	8.64	8.75	8.02	8.37	35.33	16.08	35.28	28.9	3.45	0.00604
Solyc09g064270.2.1	Receptor-like kinase (A	7.38	10.06	11.57	11.09	10.03	1.21	3.95	1.37	2.18	0.22	0.006041
Solyc00g009110.2.1	Inositol-1 4 5-trisphosp	65.36	58.35	126.1	32.58	70.6	5.88	7.33	5.31	6.17	0.09	0.006072
Solyc02g077790.1.1	Unknown Protein (AH	0	0	2.73	0	0.68	36.47	16.03	121.77	58.09	85.11	0.006072
Solyc03g006240.2.1	GDSL esterase/lipase A	3.4	4.61	1.48	1.01	2.62	0.29	0	0	0.1	0.04	0.006075
Solyc01g108190.2.1	Calmodulin-like protei	7.8	10.42	9.21	4.01	7.86	20.92	17.63	13.73	17.43	2.22	0.006103
Solyc02g094320.2.1	Actin (AHRD V1 ***-	33.32	36.73	19.36	27.99	29.35	6.42	12.54	4.87	7.94	0.27	0.006108
Solyc04g078090.2.1	Acyl-CoA-binding dom	26.27	31.26	24.55	23.17	26.31	88.35	79.73	40.72	69.6	2.65	0.00613
Solyc06g009050.2.1	Universal stress protei	57.83	104.74	30.27	30.53	55.84	2133.92	882.35	1126.13	1380.8	24.73	0.00614
Solyc10g006920.2.1	CBS domain containin	2.16	0.89	0.78	0.94	1.19	0	0	0	0	0.01	0.006144
Solyc08g007990.2.1	RNA-binding protein N	77.15	67.39	104.83	77.35	81.68	24.29	29.6	30.55	28.15	0.34	0.006145
Solyc01g097340.2.1	NAD-dependent epime	100.79	112.44	126.35	111.38	112.74	68.28	31.94	37.69	45.97	0.41	0.006184
Solyc04g006930.2.1	Receptor-like protein k	12.77	9.97	16.62	10.14	12.38	4.06	4.5	2.21	3.59	0.29	0.006203
Solyc03g116660.2.1	Unknown Protein (AH	16.27	14.48	24.52	15.34	17.65	38.7	38.29	36.33	37.77	2.14	0.006204
Solyc06g082010.2.1	Zinc finger CCCH dom	43.16	35.82	48.37	44.39	42.94	8.91	16.82	11.79	12.51	0.29	0.006207
Solyc03g095490.2.1	Receptor like kinase, R	4.36	5.54	3.92	3.81	4.41	0.92	1.61	0.72	1.08	0.25	0.00622
Solyc07g065860.2.1	Receptor like kinase, R	5.12	8.02	9.7	9.74	8.14	21.26	14.05	23.52	19.61	2.41	0.006225
Solyc11g011020.1.1	Receptor like kinase, R	12.93	18.26	10.53	19.01	15.18	4.06	7.44	3.26	4.92	0.32	0.006226
Solyc08g080640.1.1	Osmotin-like protein (I	0.27	1.66	1.26	0.75	0.99	229.96	953.33	24.03	402.44	408.57	0.006226
Solyc09g090050.2.1	Thyroid hormone recep	38.53	26.34	30.55	30.09	31.38	8.97	11.56	6.43	8.99	0.29	0.006303
Solyc02g070800.2.1	Ycf23 protein (AHRD	126.36	105.38	65.41	113.38	102.63	11.81	56.38	44.37	37.52	0.37	0.006314
Solyc12g099450.1.1	Genomic DNA chromo	1.93	1.55	1.37	2.18	1.76	0	0.16	0	0.05	0.03	0.006314
Solyc10g085270.1.1	Unknown Protein (AH	6.34	3.39	5.99	1.79	4.38	31.77	26.88	15.11	24.59	5.62	0.006341
Solyc12g013890.1.1	Genomic DNA chromo	29.18	26.39	27.47	30.96	28.5	7.27	10.25	8.47	8.66	0.3	0.006387
Solyc08g076590.2.1	Heat stress transcriptio	2.02	2.15	1.27	0.57	1.5	0.11	0.11	0.4	0.21	0.14	0.006395
Solyc08g059800.2.1	Os02g0200800 protein	21.8	19.66	22.33	29.62	23.35	3.82	9.7	4.41	5.98	0.26	0.006414
Solyc02g065420.2.1	UPF0497 membrane pr	79.08	112.54	59.37	59.22	77.55	26.31	26.88	26.86	26.68	0.34	0.006416
Solyc12g008350.1.1	Ethylene-responsive tr	27.93	21.14	45.92	55.68	37.67	5.15	6.27	1.31	4.24	0.11	0.006419
Solyc01g097650.2.1	Serine incorporator 1 (	0.81	1.15	0.96	0.69	0.9	2.88	3.43	1.45	2.59	2.87	0.006488
Solyc02g090120.1.1	Unknown Protein (AH	9.1	12.82	5.37	13.94	10.31	19.6	51.29	44.41	38.43	3.73	0.006505
Solyc06g068650.2.1	4-coumarate CoA ligas	2.67	2.16	2.29	4.97	3.02	0.78	0.2	0.72	0.57	0.19	0.006509
Solyc12g056600.2.1	Short chain alcohol de	1.17	2.6	3.1	0.41	1.82	254.47	49.24	255.55	186.42	102.43	0.006509
Solyc05g047590.2.1	Pectinesterase (AHRD	1.49	1.93	0.54	0.32	1.07	0	0	0	0	0.01	0.006523
Solyc06g075360.2.1	Senescence-associated	99.53	126.43	164.88	181.75	143.15	37.51	62.37	40.61	46.83	0.33	0.006532
Solyc01g006520.2.1	Receptor-like protein k	2.52	2.96	2.01	3.79	2.82	0.39	0.6	0.73	0.57	0.2	0.006533
Solyc03g119830.2.1	HAT family dimerisati	1.54	1.63	1.48	1.63	1.57	2.49	4.39	2.96	3.28	2.09	0.00657
Solyc12g044720.1.1	60S ribosomal protein	2787.58	2095.55	2998.65	3133.72	2753.88	924.49	1129.89	891.75	982.04	0.36	0.006587
Solyc01g108570.2.1	Gibberellin receptor G	5.3	10.01	4.5	4.8	6.15	48.5	20.22	78.96	49.23	8	0.006603
Solyc08g078550.1.1	Matrix metalloproteina	0	0	0	0.26	0.07	0.9	1.96	1.65	1.5	23.13	0.00663
Solyc12g044840.1.1	Receptor-like kinase (A	0.44	0.47	0.84	0.88	0.66	0	0	0	0	0.02	0.00663
Solyc03g121070.2.1	Hexokinase (AHRD V	30.41	31.06	31.04	45.26	34.44	11.22	13.14	10.5	11.62	0.34	0.006641
Solyc07g056430.2.1	Glutathione S-transfera	11.7	17.91	16.56	9.93	14.03	131.17	69.39	174.62	125.06	8.92	0.00666
Solyc07g007050.1.1	Unknown Protein (AH	3.85	0.49	5.84	6.22	4.1	11.55	10.75	5.46	9.25	2.26	0.006661
Solyc10g086530.1.1	GRAS family transcrip	0.43	0.62	0.71	1.47	0.81	0	0	0	0	0.01	0.006661
Solyc04g077860.2.1	Hydrolase alpha/beta f	11.08	14.46	16.55	11.14	13.31	86.51	74.82	42.27	67.87	5.1	0.00667
Solyc03g114720.2.1	Transcription factor BI	86.93	80.11	74.2	79.24	80.12	23.83	39.09	33.15	32.02	0.4	0.006671
Solyc01g100370.2.1	Universal stress protei	100.34	108.99	231.17	221.28	165.44	942.14	642.84	785.39	790.12	4.78	0.006706
Solyc10g074790.1.1	Auxin efflux carrier fa	376.89	455.21	289.17	238.45	339.93	115	153.68	102.61	123.76	0.36	0.006789
Solyc03g119960.2.1	Phototropic-responsive	6.14	4.51	3.26	3.69	4.4	0.5	0.38	0.46	0.45	0.1	0.006814
Solyc01g009680.2.1	Lipoxygenase (AHRD	0.15	0.79	1.75	0.42	0.78	3.12	2.42	4.87	3.47	4.46	0.00683
Solyc02g088090.1.1	Calmodulin-like protei	16.67	9.68	8.55	10.77	11.42	2.34	2.66	3.96	2.99	0.26	0.00691
Solyc07g005100.2.1	Chitinase-like protein (	1.22	0.47	0.21	2.72	1.15	3.68	5.28	1.74	3.57	3.09	0.00691
Solyc11g068440.1.1	Glucan endo-1 3-beta-g	33.59	37.76	38.41	22.53	33.07	62.81	46.98	98.31	69.37	2.1	0.006912
Solyc12g005040.1.1	Calcium-dependent pro	13.32	9.6	8.23	11.66	10.7	0	1.03	0.63	0.55	0.05	0.006951
Solyc02g083760.2.1	Thaumatin-like protein	50.44	41.84	39.99	32.53	41.2	13.37	22.03	8.61	14.67	0.36	0.007035
Solyc10g084340.1.1	AP2-like ethylene-resp	2.56	1.9	2.52	2.82	2.45	0	0.81	0.14	0.32	0.13	0.007053
Solyc03g117100.2.1	PPPDE peptidase dom	3.79	8.05	3.1	8.67	5.9	0.24	0.95	1.16	0.78	0.13	0.007053
Solyc05g005690.1.1	30S ribosomal protein	151.56	138.45	155.66	143.76	147.36	38.17	50.13	31.86	40.05	0.27	0.007071
Solyc01g091570.2.1	Thioesterase family pro	30.94	25.87	25.06	27.94	27.45	5.72	10.93	5.18	7.28	0.27	0.007077
Solyc12g096540.1.1	40S ribosomal protein	546.36	425.92	519.88	506.28	499.61	177.56	203.74	181.61	187.64	0.38	0.007084
Solyc04g071030.1.1	U-box domain-contain	4.47	6.17	5.27	3.71	4.91	8.59	11.81	12.24	10.88	2.22	0.007121

Solyc11g008380.1.1	Glycerol-3-phosphate	1.78	2.89	1.91	2.52	2.27	6.02	5.15	7.56	6.24	2.74	0.007121
Solyc08g082480.2.1	Phosphatidylinositol 3-	7.46	10.15	8.93	15.34	10.47	3.65	2.67	4.26	3.53	0.34	0.007139
Solyc03g025200.2.1	Multidrug resistance p	15.11	14.19	15.37	18.04	15.68	4.7	5.31	2.75	4.25	0.27	0.00714
Solyc09g075400.2.1	Unknown Protein (AH	1.15	2.28	0.91	1.45	1.45	0.1	0.21	0	0.1	0.07	0.007148
Solyc01g010710.2.1	Serine carboxypeptidas	2.42	1.67	1.74	0.76	1.65	0	0	0	0	0.01	0.007204
Solyc05g055480.2.1	NAC-domain transcrip	23.7	22.61	29.56	29.66	26.38	6.06	10	5.08	7.05	0.27	0.007221
Solyc01g111890.2.1	F-box/LRR-repeat prot	18.26	24.2	27.24	21.62	22.83	3.81	8.95	4.79	5.85	0.26	0.007229
Solyc11g068820.1.1	50S ribosomal protein	232.33	219.26	228.98	246.14	231.68	75.18	114.79	117.99	102.65	0.44	0.007232
Solyc08g068700.1.1	N-acetyltransferase (A	107.37	77.49	33.72	38.54	64.28	3.67	7.18	3.98	4.94	0.08	0.007263
Solyc09g092480.1.1	UDP-glucosyltransfera	0.84	0.38	1.16	0.4	0.69	2.5	2.75	2.51	2.59	3.72	0.007266
Solyc08g079150.1.1	Auxin responsive SAU	3.24	1.8	6.21	3.43	3.67	0.33	0	0	0.11	0.03	0.007281
Solyc12g042080.1.1	40S ribosomal protein	545.09	489.89	517.58	521.99	518.64	168.89	203.61	168.62	180.37	0.35	0.007299
Solyc08g062970.1.1	Glutaredoxin (AHRD	13.19	17.19	10.01	10	12.6	1.9	3.84	0.94	2.23	0.18	0.0073
Solyc09g090390.1.1	Unknown Protein (AH	1.86	1.03	2.74	0.99	1.66	0	0	0	0	0.01	0.007316
Solyc05g007150.2.1	UDP-galactose transpo	8.11	5.54	3.78	10.13	6.89	11.73	14.74	15.34	13.94	2.02	0.007317
Solyc01g105710.2.1	Peptidyl-prolyl cis-tran	499.16	398.01	458.04	622.66	494.47	201.87	194.57	162.84	186.43	0.38	0.007323
Solyc09g063150.2.1	Glutathione S-transfera	16.72	25.5	25.71	30.4	24.58	3.62	13.14	2.67	6.48	0.26	0.007338
Solyc04g077650.2.1	Serine carboxypeptidas	27.72	35.59	40.19	30.83	33.58	349.88	205.38	312.51	289.26	8.61	0.007346
Solyc06g068550.2.1	Aspartic proteinase ne	0.84	1.12	0.82	1.18	0.99	0	0	0	0	0.01	0.007365
Solyc10g006530.2.1	Oxygen evolving enha	5.83	9.59	1.06	4.44	5.23	0	0	0	0	0	0.007375
Solyc06g069440.2.1	Zinc finger CCCH don	14.92	14.93	28.67	23.31	20.46	50.81	32.76	41.21	41.59	2.03	0.007387
Solyc02g092530.2.1	Formamidase (AHRD	34.63	25.24	35.31	33.03	32.05	9.75	15.32	14.15	13.07	0.41	0.007412
Solyc06g060830.2.1	Homeobox-leucine zip	6.62	5.5	7.29	6.55	6.49	0.71	2.34	1.97	1.67	0.26	0.007427
Solyc08g077770.2.1	Calcineurin B-like cal	49.32	52.4	37.1	37.94	44.19	16.44	16.83	10.71	14.66	0.33	0.007453
Solyc05g006020.2.1	Nucleobase ascorbate	2.47	2.2	1.31	1.05	1.76	0.2	0.1	0.12	0.14	0.08	0.007478
Solyc12g008650.1.1	Inositol oxygenase (AI	3.94	16.8	11.99	2.64	8.84	0.5	0	0.41	0.3	0.03	0.007478
Solyc10g047950.1.1	Inorganic pyrophospha	69.1	70.66	82.05	80.35	75.54	23.49	32.44	24.86	26.93	0.36	0.007503
Solyc01g007130.2.1	Receptor like kinase, R	6.57	8.95	10.42	8.42	8.59	1.71	3.29	1.97	2.32	0.27	0.007517
Solyc07g008100.2.1	Genomic DNA chrom	4.51	6.62	3.6	6.01	5.18	1.18	0.76	1.19	1.04	0.2	0.007532
Solyc02g081810.2.1	tRNA pseudouridine s	88.81	75.7	85.77	70.38	80.16	27.18	33.8	24.64	28.54	0.36	0.00754
Solyc01g010130.2.1	Transcription factor (A	4.17	6.22	6.08	3.64	5.03	1.49	0.9	1.22	1.2	0.24	0.007556
Solyc06g071910.2.1	3-oxoacyl-reductase (A	87.48	88.25	65.26	79.99	80.25	22.01	35.73	20.13	25.96	0.32	0.007559
Solyc01g108000.2.1	Receptor-like kinase (A	0.89	0.7	0.81	1.11	0.88	0	0.08	0.2	0.09	0.11	0.007567
Solyc06g083000.1.1	Calmodulin-like protei	0	0	0	0	0	3.55	6.09	0.44	3.36	336	0.007583
Solyc07g064240.2.1	CT099 (Fragment) (AI	10.24	12.06	7.82	5.54	8.92	1.7	3.19	0.3	1.73	0.19	0.007602
Solyc08g076790.2.1	Cinnamoyl-CoA reduc	5.22	10.03	2.96	0	4.55	137.63	102.24	99.26	113.04	24.83	0.007602
Solyc01g008910.2.1	Scarecrow transcriptio	1.38	1.35	0.82	1.37	1.23	2.35	5.07	3.71	3.71	3.02	0.007605
Solyc07g042430.1.1	Dimethylaniline mono	0	0	0	0	0	0.73	0.31	0.13	0.39	39	0.007605
Solyc01g108860.2.1	1-aminocyclopropane-	2.25	3	0.44	1.33	1.76	0.15	0	0	0.05	0.03	0.007621
Solyc08g005220.2.1	Plastid lipid-associate	6.3	4.06	4.46	10.47	6.32	0.76	0.77	0.94	0.82	0.13	0.007684
Solyc00g136260.1.1	Ring H2 finger protein	148.31	164.57	289.74	238.4	210.25	46.68	63.96	26.77	45.8	0.22	0.007708
Solyc04g082460.2.1	Catalase (AHRD V1 *	19.54	23.22	21.79	16.66	20.3	51.78	45.8	57.91	51.83	2.55	0.007708
Solyc01g106700.2.1	MADS-box transcripti	0	0.27	0.36	0	0.16	20.17	7.03	16.52	14.57	92.53	0.007709
Solyc09g014490.2.1	En/Spm-like transposo	5.29	4.27	5.52	11.84	6.73	0.3	0.8	0	0.37	0.05	0.007713
Solyc11g013130.1.1	Unknown Protein (AH	150.89	84.95	251.71	241.23	182.19	18.71	49.18	10.45	26.11	0.14	0.00772
Solyc02g093020.2.1	Os06g0524700 protein	6.55	5.61	7.15	15.78	8.77	0.1	0.69	0.36	0.38	0.04	0.007743
Solyc11g008510.1.1	60S ribosomal protein	941.5	983.04	588.19	617.22	782.49	349.49	330.23	260.99	313.57	0.4	0.007757
Solyc01g007870.2.1	Cyclin B-like protein (	10.39	5.09	7.97	7.35	7.7	0.42	1.69	0.52	0.88	0.11	0.007777
Solyc12g096600.1.1	Poly(ADP-ribose) glyc	4.37	8.76	6.88	3.61	5.9	0.88	0.3	1.09	0.76	0.13	0.007777
Solyc08g016770.2.1	Decarboxylase family	1.45	2.06	0.68	0.61	1.2	0	0	0.14	0.05	0.04	0.007782
Solyc01g008920.2.1	NAD dependent epime	11.7	10.41	9.52	7.42	9.76	1.96	4.27	2.2	2.81	0.29	0.007802
Solyc04g054760.2.1	Os10g0422600 protein	62.61	69.56	66.93	66.57	66.42	36.32	20.2	23.36	26.63	0.4	0.007825
Solyc11g022590.1.1	Kunitz trypsin inhibito	14.3	15.07	12.05	28.9	17.58	0.47	2.14	3.77	2.13	0.12	0.007836
Solyc04g076610.1.1	Receptor expression-e	17.69	25.73	32.62	25.02	25.26	8.28	11.78	7.11	9.06	0.36	0.007867
Solyc09g089740.2.1	1-aminocyclopropane-	2.97	2.23	1.62	0	1.7	6.09	5.39	2.95	4.81	2.82	0.007867
Solyc08g081140.2.1	Myc-like anthocyanin	28.41	30.81	50.52	33.04	35.7	15.47	13.12	7.31	11.97	0.34	0.007888
Solyc04g078110.1.1	Subtilisin-like proteas	0.34	0.92	0.91	0.12	0.57	0.97	2.17	1.11	1.42	2.47	0.007897
Solyc04g051460.2.1	Cell division protein k	4.04	1.99	1.65	4.14	2.96	0.56	0.57	0.28	0.47	0.16	0.007922
Solyc10g017970.1.1	Chitinase (AHRD V1 *	9.73	3.85	25.52	41.3	20.1	375.71	863.65	113.79	451.05	22.44	0.00794
Solyc09g005720.2.1	60S ribosomal protein	1801.78	1543.72	1363.58	1319.75	1507.21	608.78	683.14	445.19	579.04	0.38	0.00802
Solyc07g008240.2.1	Non-symbiotic hemogl	15.52	21.11	3.56	12.2	13.1	1.74	1.76	0.86	1.45	0.11	0.008075
Solyc05g005790.2.1	RING finger protein (A	2.93	3.1	1.32	1.97	2.33	5.63	5.57	3.88	5.03	2.16	0.008092
Solyc08g077480.2.1	Os04g0585900 protein	103.12	147.09	135.18	129.17	128.64	43.2	50.65	28.95	40.93	0.32	0.008092
Solyc09g008280.1.1	S-adenosylmethionine	935.57	675.89	961.85	1475.63	1012.24	210.33	327.08	241.66	259.69	0.26	0.008098
Solyc04g009200.2.1	Glutamate-1-semialde	96.16	77.19	77.99	96.02	86.84	26.37	41.74	28.88	32.33	0.37	0.008117
Solyc08g077230.2.1	Two-component respo	4.82	4.19	2.77	2.99	3.69	103.56	32.39	54.86	63.6	17.23	0.00812
Solyc04g009370.2.1	Homology to unknown	10.54	9.63	6.7	6.5	8.34	14.85	25.78	15.3	18.64	2.23	0.00814
Solyc09g083320.1.1	Prenylated rab accepto	10.15	9.03	11.97	15.46	11.65	23.97	29.26	22.86	25.36	2.18	0.008147
Solyc06g074820.2.1	Aquaporin-like protein	216.31	188.54	465.44	739.09	402.35	10.4	39.84	8.09	19.44	0.05	0.008189
Solyc07g062300.2.1	NHL repeat-containing	20.34	21.9	23.84	18.08	21.04	7	9.18	3.14	6.44	0.31	0.008207
Solyc12g038920.1.1	Serine/threonine protei	32.99	36.87	33.82	53.32	39.25	9.99	17.6	6.43	11.34	0.29	0.008264
Solyc02g077840.1.1	Ethylene responsive tr	5.53	3.41	4.52	3.61	4.27	0.69	0	0.42	0.37	0.09	0.008265
Solyc02g086760.1.1	Unknown Protein (AH	1.78	2.9	2.09	1.26	2.01	0	0	0	0	0	0.008267
Solyc05g005910.2.1	Peptide transporter (AI	5.32	4.12	4.43	7.16	5.26	1.13	2.7	1.47	1.77	0.34	0.008268
Solyc01g095490.2.1	Ubiquitin-conjugating	57.12	63.05	48.58	40.08	52.21	135.93	128.8	110.31	125.01	2.39	0.008282
Solyc06g050510.2.1	DNA repair protein (A	20.44	24.21	33.6	29.56	26.95	7.57	9.43	7.32	8.11	0.3	0.008306
Solyc01g096410.2.1	Phosphatidylinositol-4	0.59	1.51	1.3	1.2	1.15	0.07	0.28	0	0.12	0.1	0.008363
Solyc08g005430.2.1	Growth-regulating fact	5.36	4.61	6.76	7.58	6.08	1.64	1.36	0.92	1.31	0.22	0.00837
Solyc09g092300.2.1	Cc-nbs, resistance prot	3.69	2.74	2.42	4.06	3.23	0.5	0.33	0	0.28	0.09	0.00838
Solyc04g009020.2.1	Pre-mRNA-splicing fa	8.32	9.99	12.33	9.44	10.02	4.38	3.47	1.25	3.03	0.3	0.008396
Solyc05g055430.2.1	(RAP Annotation relea	9.39	15.72	9.12	13.67	11.97	2.45	4.95	2.47	3.29	0.27	0.008396
Solyc02g080060.1.1	Cysteine-rich receptor-	0.18	0	0	0.25	0.11	1.3	1.31	1.07	1.23	11.41	0.008405

Solyc11g070140.1.1	Cell division protein k	4.7	3.18	5.8	3.8	4.37	0.36	1.09	0.89	0.78	0.18	0.008431
Solyc05g007090.2.1	Zinc knuckle (CCHC-t	13.8	14.83	13.3	11.66	13.4	5.98	4.53	1	3.84	0.29	0.008464
Solyc10g079280.1.1	Organic anion transpo	12.3	15.1	7.51	9.6	11.13	3.08	3.28	2.32	2.89	0.26	0.008465
Solyc04g064940.2.1	Receptor like kinase, F	0.79	0.16	0.43	0.86	0.56	0.05	0	0	0.02	0.03	0.008465
Solyc01g094550.2.1	Acyl-CoA thioesterase	49.98	54.74	62.05	48.29	53.76	19.63	23.43	17.71	20.26	0.38	0.008468
Solyc05g008440.1.1	Unknown Protein (AH	154.73	135.93	122.64	115.09	132.1	55.4	55.06	48.62	53.03	0.4	0.008468
Solyc03g078720.2.1	Glucosyltransferase-2 (	36.62	32.85	25.72	22.2	29.35	7.51	12.79	4.9	8.4	0.29	0.008509
Solyc07g066350.1.1	Unknown Protein (AH	4.11	3.07	2.33	2.33	2.96	0.53	0.27	0.16	0.32	0.11	0.008509
Solyc02g064640.2.1	ADP-RIBOSYLATION	47.78	59.03	37.86	31.27	43.99	17.29	16.56	10.26	14.7	0.33	0.008515
Solyc01g107460.2.1	Unknown Protein (AH	0	0.42	0	0	0.1	7.61	5.38	0	4.33	41.24	0.008528
Solyc03g046470.2.1	Auxin-independent gro	19.52	26.79	20.65	25.17	23.03	8.08	9.8	6.68	8.19	0.36	0.008528
Solyc04g008400.1.1	Serine/threonine-prote	0.56	0.54	0.42	0.79	0.58	0	0.04	0	0.01	0.02	0.008548
Solyc03g121970.1.1	Unknown Protein (AH	3.39	5.46	3.81	6.62	4.82	1.56	0.39	0.8	0.92	0.19	0.00856
Solyc04g015630.2.1	Nbs-lrr, resistance prot	1.9	2.42	2.41	3.46	2.55	0.22	0.22	0.14	0.19	0.08	0.00856
Solyc08g006760.2.1	Unknown Protein (AH	18.91	26.55	13.88	13.67	18.25	3.73	7.19	2.5	4.47	0.25	0.00856
Solyc08g063040.2.1	Zinc finger protein (Al	2.26	2.38	3.83	0.8	2.32	39.97	23.89	26.86	30.24	13.05	0.00856
Solyc09g011920.2.1	Mitochondrial uncoupl	50.28	43.22	41.32	46.81	45.41	105.21	83.35	93.64	94.07	2.07	0.008571
Solyc03g116670.2.1	TPR domain protein (A	346.35	282.98	334.73	305.93	317.5	69.32	150.21	72.53	97.35	0.31	0.008583
Solyc01g095460.2.1	G-box binding factor 3	52.27	43.3	56.84	31.4	45.95	11.19	19.63	28.13	19.65	0.43	0.008627
Solyc11g011890.1.1	Zinc finger protein 7 (A	6.14	2.61	1.58	2.27	3.15	0.22	0	0	0.07	0.02	0.008644
Solyc10g074470.1.1	Bzip transcription fact	6.32	7.01	8.23	6.5	7.02	1.46	2.34	0.57	1.46	0.21	0.0087
Solyc03g115710.1.1	Pto-like, Serine/threon	0	0	0	0.11	0.03	0.44	0.57	2.39	1.13	41.21	0.008722
Solyc09g075750.1.1	Unknown Protein (AH	1.79	0.88	1.17	1.69	1.38	0	0	0	0	0.01	0.008743
Solyc06g073990.1.1	Unknown Protein (AH	9.35	6.85	4.54	1.56	5.57	16.88	17.02	1.64	11.85	2.12	0.008759
Solyc02g070540.2.1	Os01g0611000 protein	29.79	28.16	50.03	88.79	49.19	1.9	4.53	0.84	2.42	0.05	0.008817
Solyc07g062280.2.1	Gamma-glutamyl hydr	1.95	3.3	2.07	4.14	2.87	0.79	0.16	0.39	0.45	0.16	0.008822
Solyc01g079350.2.1	PAPA-1-like conserved	47.03	44.91	44.54	36.98	43.36	17.67	16.72	12.73	15.71	0.36	0.008877
Solyc01g087790.2.1	Subtilisin-like protease	7.95	9.11	15.27	19.86	13.05	1.16	3.38	1.26	1.93	0.15	0.008877
Solyc05g050980.2.1	3-phosphoshikimate 1-	29.62	23.76	29.69	33.08	29.04	13.66	10.98	10.72	11.79	0.41	0.008886
Solyc08g013720.2.1	Genomic DNA chromo	16.55	9.88	12.6	13.33	13.09	2.88	5.63	2.08	3.53	0.27	0.008904
Solyc05g056390.2.1	chaperonin (AHRD V1	210.7	129.41	125.48	123.79	147.34	226.25	449.26	245.89	307.13	2.08	0.008917
Solyc05g054710.2.1	Beta-hexosaminidase b	21.95	31.75	33.08	29.87	29.16	10.31	11.86	9.71	10.63	0.36	0.008953
Solyc10g080200.1.1	AN1-type zinc finger p	8.46	5.38	5.42	4.79	6.01	9.57	14.37	13.91	12.62	2.1	0.008976
Solyc01g007970.1.1	RLK, Receptor like pro	2.11	1.16	2.49	1.71	1.87	0.24	0.41	0.2	0.28	0.15	0.008977
Solyc06g061090.2.1	LOC555512 protein (F	9.3	12.04	5.32	3.19	7.46	0.46	1.61	0.28	0.78	0.1	0.008984
Solyc03g117510.2.1	Formamidopyrimidine-	21.55	16.42	16.72	17.33	18	5.6	5.77	3.22	4.86	0.27	0.008986
Solyc02g063140.2.1	Fatty acid elongase 3-k	1.19	1.42	0.47	0.38	0.86	0	0	0	0	0.01	0.008993
Solyc02g077570.1.1	MLO-like protein 6 (A	0.7	1.09	2.07	0.99	1.21	0	0	0	0	0.01	0.009018
Solyc04g079530.2.1	Solute carrier family 1	0	0	0	0	0	1.56	2.22	1.58	1.79	178.67	0.009027
Solyc11g032090.1.1	Os05g0272200 protein	0.67	1.79	1.42	0.57	1.11	0.11	0	0	0.04	0.03	0.009027
Solyc03g078520.2.1	Receptor like kinase, F	17.79	17.96	17.54	12.89	16.55	7.89	6.81	7	7.23	0.44	0.009049
Solyc11g071290.1.1	Alcohol dehydrogenase	3.15	2.02	4.54	0	2.43	0	0	0	0	0	0.00909
Solyc04g076380.2.1	Cytochrome P450 NA	147.11	148.96	177.3	184.32	164.42	46.16	73.55	57.86	59.19	0.36	0.009094
Solyc09g064530.2.1	Auxin responsive prot	9.25	13.54	8.24	9.3	10.08	4.48	2.85	2.45	3.26	0.32	0.009101
Solyc08g078020.1.1	Methionine rich arabin	8.19	5.76	12.21	5.49	7.91	1.39	1.05	0.86	1.1	0.14	0.009126
Solyc02g069460.2.1	Photosystem I reaction	341.09	371.77	206.65	193.56	278.27	97.05	114.32	68.34	93.24	0.34	0.009166
Solyc01g110920.2.1	Auxin-induced SAUR-	29.57	31.72	19.68	19.31	25.07	6.13	7.41	5.27	6.27	0.25	0.009193
Solyc06g019200.2.1	Peptidase M50 family	15.75	17.28	11.12	10.94	13.77	4.32	6.19	4.61	5.04	0.37	0.009193
Solyc08g067990.1.1	Subtilisin-like protease	0.49	1.1	0.77	0.35	0.68	0	0.07	0	0.02	0.03	0.009204
Solyc09g083200.2.1	Nod factor receptor pro	5.34	3.07	4.67	5.12	4.55	0.28	0.98	0.34	0.53	0.12	0.00924
Solyc03g116800.2.1	Unknown Protein (AH	49.6	34.15	50.78	50.96	46.37	13.99	20.97	11.61	15.52	0.33	0.009263
Solyc01g102810.2.1	DNA polymerase III ga	4.09	4.42	5.25	7.18	5.23	0.67	1.31	0.36	0.78	0.15	0.009302
Solyc09g011160.2.1	Regulator of chromoso	0.56	0.38	0.17	0.4	0.38	1.25	2.99	5.88	3.37	8.94	0.00931
Solyc06g035570.1.1	Transcription factor M	11.99	5.34	15.57	8.48	10.34	0	0.98	0	0.33	0.03	0.00931
Solyc12g011410.1.1	Unknown Protein (AH	57.42	154.4	30.27	19.01	65.28	0	1	0	0.33	0.01	0.009329
Solyc04g025530.2.1	Glutamate decarboxyla	1.6	1.78	1.1	0.57	1.26	0.11	0	0.13	0.08	0.06	0.009352
Solyc05g012530.1.1	Unknown Protein (AH	9.83	4.37	6.62	0.99	5.45	0	0	0.7	0.23	0.04	0.009352
Solyc06g075450.1.1	Peptide transporter 1 (A	1.09	0.75	0.57	0.85	0.82	0	0	0	0	0.01	0.009353
Solyc03g111770.2.1	Abhydrolase domain-c	68.45	59.98	61.09	64.47	63.5	22.49	27.89	14.08	21.49	0.34	0.009366
Solyc03g119750.2.1	Family with sequence s	14.39	14.5	16.86	15.55	15.32	4.68	5.46	3.17	4.44	0.29	0.009398
Solyc09g007150.2.1	Glutathione S-transfera	18.22	27.27	41.68	64.03	37.8	2.01	6.3	3.01	3.77	0.1	0.009436
Solyc11g068720.1.1	Os04g0625000 protein	3.84	4.81	3.19	7.64	4.87	1.51	0.59	0.62	0.91	0.19	0.009475
Solyc06g065920.2.1	Phosphatase 2C family	15.31	24.74	18.47	15.68	18.55	5.12	8.6	5.41	6.38	0.34	0.00948
Solyc11g069660.1.1	Nbs-lrr, resistance prot	2.11	1.81	0.8	2.02	1.69	0.3	0.25	0.15	0.23	0.14	0.009481
Solyc10g007040.2.1	Beta-1,3-galactosyl-O-	0	0	0	0	0	2.57	0.86	3.75	2.39	239.33	0.009493
Solyc08g081160.2.1	(related) (AHRD V1 *	10.93	12.97	11.46	14.88	12.56	4.64	5.28	1.93	3.95	0.31	0.009497
Solyc06g063300.2.1	Kelch-domain-contain	10.73	14.03	12.53	10.77	12.02	3.29	4.81	2.66	3.59	0.3	0.009571
Solyc07g005900.2.1	Calcineurin subunit B	14.62	20.35	2.21	5.3	10.62	0.3	0.31	0.37	0.33	0.03	0.009589
Solyc08g081420.2.1	Glycosyltransferase-lik	23.04	28.91	21.39	21.37	23.68	7.53	9.64	4.12	7.1	0.3	0.009629
Solyc11g013880.1.1	Receptor-like protein k	8.58	9.32	9.75	16.81	11.11	1.65	2.05	0.94	1.55	0.14	0.009683
Solyc04g071680.1.1	Receptor like kinase, F	0.86	1.21	0.93	1.42	1.1	0.06	0.18	0.07	0.1	0.09	0.009699
Solyc08g082680.2.1	Ring H2 finger protein	7.14	11.18	6.74	5.25	7.58	1.85	1.63	0.85	1.44	0.19	0.009712
Solyc08g068460.1.1	CK25 (AHRD V1 ***-	3.32	3.64	0.3	2.17	2.36	0.21	0	0.25	0.15	0.07	0.009718
Solyc02g005530.1.1	Helitron helicase-like	0.16	0.58	1.34	1.15	0.81	2.23	2.78	1.45	2.15	2.67	0.00973
Solyc05g005480.2.1	Oxidoreductase zinc-bi	81.4	105.2	65.23	40.06	72.97	21.79	26.12	18.52	22.14	0.3	0.00975
Solyc01g008790.2.1	Phosphoesterase famil	42.26	56.75	65.67	119.77	71.11	10.83	13.68	9.5	11.34	0.16	0.009751
Solyc05g055730.2.1	Methylenetetrahydrofo	1.55	1.18	0.39	2.34	1.36	0	0	0	0	0.01	0.009751
Solyc02g088000.2.1	Glycogen synthase (AH	27.97	24.51	27.83	29.97	27.57	7.76	14.05	7.49	9.77	0.35	0.009802
Solyc11g010980.1.1	Alcohol dehydrogenase	1.47	4.24	3.03	0.52	2.31	0	0	0.18	0.06	0.03	0.009802
Solyc03g058430.1.1	Omega-6 fatty acid des	1.39	0.46	0.82	2.22	1.22	0	0	0	0	0.01	0.009805
Solyc03g111000.2.1	Glyceraldehyde 3-phos	41.47	43.28	57.82	49.59	48.04	20.41	13.21	20.58	18.07	0.38	0.009819
Solyc04g077670.2.1	Serine carboxypeptidas	20.46	22.42	13.74	18.2	18.71	5.69	9.27	4.71	6.56	0.35	0.009822

Solyc11g069140.1.1	Unknown Protein (AH	15.83	5.87	21.79	9.33	13.2	31.98	32.25	39.29	34.51	2.61	0.009836
Solyc12g008420.1.1	RNA-dependent RNA	15.36	21.96	17.53	15.32	17.54	4.27	5.33	2.5	4.03	0.23	0.009869
Solyc11g010320.1.1	AT2G46550 protein (F	1.06	0.63	1.67	0	0.84	4.58	7.51	2.82	4.97	5.92	0.009873
Solyc08g066070.1.1	Exocyst complex prote	31	37.81	36.45	35.88	35.29	9.02	17.01	9.84	11.96	0.34	0.009882
Solyc07g042130.2.1	Pentatricopeptide repe	3.09	4.04	2.14	4.24	3.38	7.12	5.7	8.12	6.98	2.07	0.009897
Solyc02g082170.1.1	Purine permease (AHR	0.96	1.71	0.76	1.59	1.25	2.46	8.35	1.11	3.97	3.17	0.009916
Solyc03g095770.2.1	WRKY transcription fa	74.88	39.86	20.45	30.31	41.38	0.78	2.94	9.32	4.35	0.11	0.009929
Solyc01g107200.2.1	Os02g0794300 protein	4.22	4.12	5.95	6.84	5.28	1.25	2.1	0.82	1.39	0.26	0.009929
Solyc03g119060.2.1	Alpha-dioxygenase 2 (	43.25	41	26.44	21.52	33.05	11.03	10.53	8.59	10.05	0.3	0.009954
Solyc08g014030.1.1	GRAS family transcrip	0.94	1.68	0.19	1.34	1.04	0	0	0	0	0.01	0.009962
Solyc02g078500.2.1	Aluminum-induced pro	8.76	7.34	7.3	6.2	7.4	1.04	2.31	2.56	1.97	0.27	0.009973
Solyc10g085280.1.1	UDP-glucosyltransfera	21.34	23.31	24.04	27.25	23.98	154.86	150.66	149.54	151.69	6.32	0.009973
Solyc12g007060.1.1	Unknown Protein (AH	22.89	29.96	72.27	29.52	38.66	2.72	6.03	6.01	4.92	0.13	0.009973
Solyc02g063490.2.1	Malate dehydrogenase	38.12	44.74	37.48	39.65	40	120.34	82.84	120.58	107.92	2.7	0.009991
Solyc03g098210.2.1	Cyclic nucleotide gate	26.68	24.31	38.97	20.41	27.59	8.94	10.94	11.08	10.32	0.37	0.009991
Solyc03g111820.2.1	Sieve element-occludi	0.82	0.97	0.86	0.9	0.89	0	0.15	0	0.05	0.06	0.010089
Solyc08g079080.2.1	Acid beta-fructofurano	3.54	3.78	1.67	2.72	2.93	0.33	0.74	0	0.36	0.12	0.010089
Solyc02g065610.2.1	Leaf senescence protei	12.01	12.1	11.78	7.37	10.81	3.62	3.65	2.01	3.09	0.29	0.010097
Solyc01g109850.2.1	Diaminopimelate deca	122.75	130.98	125.08	133.67	128.12	47.2	57.11	53.91	52.74	0.41	0.010103
Solyc08g005450.1.1	CAX-interacting prote	295.26	220	277.16	246.95	259.84	93.43	103.09	101.22	99.25	0.38	0.01013
Solyc09g065270.2.1	Ribosome recycling fa	62.52	54.33	49.95	58.84	56.41	20.68	27.88	22.23	23.6	0.42	0.010165
Solyc11g005650.1.1	Ubiquitin (AHRD V1	9.84	15.45	10.24	10.64	11.54	25.25	22.16	36.76	28.06	2.43	0.010254
Solyc05g056300.2.1	Thioredoxin-like prote	71.38	74.76	65.92	49.97	65.51	30	28.2	15.39	24.53	0.37	0.010265
Solyc06g068400.2.1	60S ribosomal protein	51.55	45.88	51.05	39.29	46.94	10.57	21.11	13.79	15.16	0.32	0.010271
Solyc01g009010.2.1	4-diphosphocytidyl-2-(	22.21	24.45	22.51	17.92	21.77	84.67	55.23	71.2	70.37	3.23	0.010303
Solyc07g045170.2.1	184 (Fragment) (AHRI	5.45	5.45	8.25	7.37	6.63	1.01	2.67	2.25	1.98	0.3	0.010371
Solyc11g068380.1.1	Unknown Protein (AH	0.4	0.35	0	0.56	0.33	16.92	12.88	2.35	10.72	32.72	0.010376
Solyc09g075160.2.1	60S ribosomal protein	119.38	109.04	98.22	79.99	101.66	37.22	38.4	29.62	35.08	0.35	0.010403
Solyc10g049970.1.1	Kynurenine formamida	112.74	120.49	93.51	87.05	103.45	39.18	52.96	25.9	39.35	0.38	0.010425
Solyc09g082370.2.1	Inositol 1 4 5-trisphosp	1.16	1.97	0.75	2.09	1.49	0.17	0.17	0.42	0.25	0.17	0.010472
Solyc11g067070.1.1	Serine/threonine protei	2.6	2.24	3.72	4.01	3.14	5.09	5.33	8.84	6.42	2.04	0.010584
Solyc01g105690.2.1	Transcription factor/ tr	23.61	22.58	17.18	14.98	19.59	3.92	8.81	5.69	6.14	0.31	0.010588
Solyc02g085660.1.1	UDP-glucosyltransfera	0	0.12	0.16	0	0.07	11.29	2.96	14.54	9.6	137.1	0.010602
Solyc12g014310.1.1	Pathogenesis-related p	2.81	2.25	1.32	0.79	1.79	0	0	0.28	0.09	0.05	0.010655
Solyc05g020020.2.1	CONSTANS-like zinc	34.83	39.57	38.06	38.6	37.77	12.58	13.73	12.59	12.97	0.34	0.010666
Solyc09g098360.2.1	Ribosome biogenesis p	380.16	357.68	322.96	324.64	346.36	127.43	151.39	112.15	130.32	0.38	0.010674
Solyc08g006540.2.1	Peptidyl-prolyl cis-tran	30.99	22.24	11.2	18.03	20.61	2.42	6.52	2.23	3.72	0.18	0.010684
Solyc03g097560.2.1	MtN3-like protein (AH	2.67	1.79	1.84	0.63	1.73	10.8	4.36	7.74	7.63	4.41	0.010714
Solyc08g081150.2.1	GMP synthase (AHRD	21.13	17.49	33.77	19.46	22.96	3.97	6.11	5.37	5.15	0.22	0.010731
Solyc11g021360.1.1	Unknown Protein (AH	81.07	80.64	88.15	71.95	80.45	17.34	40.8	21.3	26.48	0.33	0.010799
Solyc02g090310.1.1	Dof zinc finger protein	2.53	4.95	7.46	5.01	4.99	20.63	8.86	13.3	14.26	2.86	0.010832
Solyc07g053600.2.1	Receptor like kinase, F	3.81	3.86	3.86	5.19	4.18	1.73	0.65	0.53	0.97	0.23	0.010884
Solyc03g120020.2.1	AE family transporter	0	0	0	0	0	0.76	0.15	0.47	0.46	46	0.010955
Solyc04g082030.1.1	Ornithine decarboxylas	11	8.43	8.83	8.86	9.28	2.71	3.24	1.36	2.44	0.26	0.01096
Solyc03g031940.2.1	Acyl-CoA synthetase/A	0.75	1.62	0.5	0.3	0.79	0	0	0	0	0.01	0.010966
Solyc01g010580.2.1	Ribosomal protein (AH	914.6	722.36	1014.17	898.75	887.47	257.07	361.91	347.58	322.19	0.36	0.010991
Solyc03g124030.2.1	TPR repeat protein (AI	33.95	34.17	36.9	32.29	34.33	11.38	12.93	13.83	12.71	0.37	0.010991
Solyc05g014250.2.1	Ring finger protein 14	14.66	18.64	18.53	6.67	14.62	4.23	5.12	1.04	3.46	0.24	0.010997
Solyc03g006080.2.1	Receptor like kinase, F	0.67	1.24	0.36	0.43	0.68	0.1	0	0	0.03	0.05	0.011013
Solyc01g060140.2.1	Calmodulin-binding tra	71.6	71.9	102.42	59.09	76.25	31.12	33.32	27.84	30.76	0.4	0.011016
Solyc01g005560.2.1	Isocitrate dehydrogena	65.81	54.62	57.81	57.87	59.03	322.61	183.5	269.17	258.43	4.38	0.011056
Solyc04g073990.2.1	Annexin (AHRD V1 *	1321.94	1280.9	1082.98	1340.19	1256.5	466.49	593.89	379.59	479.99	0.38	0.011067
Solyc04g064720.2.1	Maltose excess protein	13.07	15.07	11.8	7.99	11.98	3.78	5.52	2.4	3.9	0.33	0.011077
Solyc04g011510.2.1	Triosephosphate isome	531.83	521.88	491.84	530.11	518.91	201.3	237.98	178.82	206.03	0.4	0.011078
Solyc07g020870.1.1	U-box domain-contain	2.61	3.86	2.24	1.85	2.64	0.43	1.02	0.12	0.52	0.2	0.011099
Solyc03g080100.2.1	Copper-translocating F	420.14	589.34	966.13	214.34	547.49	70.03	62.38	67.94	66.78	0.12	0.011114
Solyc12g005030.1.1	Calcium-dependent pro	4.93	7.16	4.31	3.88	5.07	0.59	0.89	0.73	0.74	0.15	0.011114
Solyc01g110060.2.1	Purple acid phosphatas	5.24	2.77	1.67	2.2	2.97	0.11	0.58	0	0.23	0.08	0.011143
Solyc12g055940.1.1	Unknown Protein (AH	23.58	20.62	21.94	21.16	21.82	7.81	9.31	7.53	8.22	0.38	0.011157
Solyc01g110670.2.1	Auxin-induced SAUR-	17.54	18.58	8.87	9.45	13.61	2.7	2.72	2.49	2.64	0.19	0.011176
Solyc07g053710.2.1	Tyrosine aminotransfe	25.6	27.54	22.42	21.77	24.33	7.61	10.37	6.24	8.07	0.33	0.01118
Solyc09g011420.2.1	50S ribosomal protein	185.64	160.67	216.6	166.38	182.32	76.44	74.18	69.04	73.22	0.4	0.011211
Solyc10g044520.1.1	Ferredoxin I (AHRD V	798.48	916.75	460.01	329.5	626.18	161.37	169.43	58.26	129.69	0.21	0.011211
Solyc06g068050.2.1	Nucleic acid binding p	46.9	65.59	40.74	26.97	45.05	15.82	15.96	10.74	14.17	0.31	0.011233
Solyc11g067190.1.1	Fatty acyl coA reducta	0	0.12	0	0	0.03	10.05	2.62	1.99	4.89	162.89	0.011248
Solyc06g007580.1.1	Plant-specific domain	10.78	11.94	14.27	17.11	13.52	1.24	5.91	0.87	2.67	0.2	0.011248
Solyc02g067060.2.1	Glycosyltransferase (A	6.05	9.02	6.85	6.99	7.23	1.1	2.01	0.74	1.28	0.18	0.011265
Solyc01g080770.2.1	Receptor like kinase, F	3.43	5.38	3.48	2.14	3.61	0.94	1.45	1.36	1.25	0.35	0.011293
Solyc02g078720.2.1	Unknown Protein (AH	4.55	3.24	5.37	4.5	4.42	0	0.37	0.9	0.42	0.1	0.011294
Solyc03g031600.2.1	Peroxisomal membran	68.45	92.17	76.16	72.24	77.25	26.08	36.13	14.79	25.67	0.33	0.011315
Solyc11g064920.1.1	Dihydropyrimidinase (	20.84	22.94	20.66	23.02	21.86	7.57	9.45	4.16	7.06	0.32	0.011336
Solyc12g008410.1.1	RNA-dependent RNA	8.26	9.47	7.87	8.53	8.53	3.46	2.46	2.21	2.71	0.32	0.011336
Solyc03g096380.2.1	Amino acid permease-	7.5	11.61	6.54	4.03	7.42	20.35	13.68	14.44	16.16	2.18	0.011369
Solyc08g077970.2.1	Unknown Protein (AH	16.93	21.63	14.34	18.42	17.83	1.75	4.24	3.02	3	0.17	0.011407
Solyc09g066360.1.1	Ethylene-responsive tr	42.17	30.57	26.08	4.81	25.91	0.27	2.49	0.34	1.03	0.04	0.011434
Solyc06g072530.2.1	UPF0497 membrane p	0	0.63	0.83	0	0.36	45.03	12.94	22.41	26.79	73.41	0.011438
Solyc10g085070.1.1	UPF0503 protein At3g	1.98	1.76	1.42	2.79	1.99	0.27	0.54	0	0.27	0.14	0.01146
Solyc03g043950.2.1	Amino-acid N-acetyltr	9.28	15.84	16.49	13.29	13.72	3.71	6.14	4.88	4.91	0.36	0.011472
Solyc07g065460.1.1	UPA24 (AHRD V1 *-*	17.78	15.83	19.63	13.79	16.76	4.63	2.34	2.28	3.08	0.18	0.011474
Solyc12g049390.1.1	Transmembrane protei	14.02	19.46	20.03	24.01	19.38	5.4	8.17	2.94	5.5	0.28	0.011536
Solyc01g096880.2.1	Nitrate transporter (AH	3.9	2.45	0.95	1.46	2.19	0.19	0.28	0	0.16	0.07	0.011549
Solyc07g006920.2.1	Os06g0207500 protein	9.04	9.47	5.55	9.63	8.42	2.5	3.13	1.72	2.45	0.29	0.011562

Solyc12g015770.1.1	Cellulose synthase (AH	10.88	12.71	2.91	3.37	7.47	0.76	0.55	0.25	0.52	0.07	0.011716
Solyc11g018540.1.1	Beta-galactosidase (AH	36.87	24.18	6.87	16.47	21.1	0	3.16	0	1.05	0.05	0.01172
Solyc03g026220.2.1	BCL-2 binding anthano	164.16	156.4	208.46	228.11	189.28	63.58	81.58	93.05	79.4	0.42	0.011751
Solyc01g016970.1.1	Unknown Protein (AH	0	0.46	1.21	0.72	0.6	4.13	5	2.54	3.89	6.51	0.011794
Solyc07g048110.2.1	Uncharacterized memb	2.15	1.62	1.56	2.34	1.92	0.2	0.81	0.08	0.36	0.19	0.011819
Solyc09g030450.2.1	Receptor like kinase, R	8.95	11.11	18.05	11.04	12.29	5.04	4.07	4.23	4.45	0.36	0.011822
Solyc07g008930.1.1	Unknown Protein (AH	0	0	0	0	0	1.07	1.88	0.65	1.2	120	0.011873
Solyc04g050730.2.1	GDSL esterase/lipase A	2.51	1.91	0.63	0.76	1.45	0.14	0	0	0.05	0.03	0.011935
Solyc01g006870.2.1	cDNA clone J023119C	38.23	45.66	31.97	34.69	37.64	17.91	15.88	13.61	15.8	0.42	0.011947
Solyc11g006070.1.1	Peptidyl-prolyl cis-tran	187.2	173.85	162.23	126.64	162.48	70.26	66.67	45.92	60.95	0.38	0.012089
Solyc06g074390.2.1	Fatty acyl coA reducta	1.47	2.27	0.79	0.38	1.23	0.11	0	0	0.04	0.03	0.012113
Solyc04g015560.2.1	Beta-D-glucosidase (A	14.44	17.07	14.92	22.06	17.12	6.05	7.3	7.95	7.1	0.41	0.012117
Solyc08g081380.2.1	At5g63850-like protei	7.85	8.45	10.72	14.75	10.44	3.75	2.44	1.64	2.61	0.25	0.01215
Solyc05g046310.2.1	Genomic DNA chromo	5.03	11.32	5.94	4.12	6.6	13.49	37.13	104.14	51.59	7.81	0.012152
Solyc07g021630.2.1	Branched-chain-amino	0	0	0	0	0	1.11	0.7	1.19	1	100	0.012157
Solyc05g014400.2.1	Genomic DNA chromo	5.55	5.07	6.54	9.7	6.71	1.53	1.9	1.3	1.58	0.23	0.012161
Solyc02g080880.2.1	Aspartic proteinase (A	15.29	17.56	22.99	10.64	16.62	41.54	30.95	37.45	36.65	2.2	0.012241
Solyc12g099260.1.1	ATP citrate lyase a-sub	23.72	25.26	22.88	48.11	29.99	5.08	9.27	4.09	6.15	0.2	0.012246
Solyc02g083130.2.1	IST1 homolog (AHRD	15.89	14.57	34.48	26.07	22.75	5.49	3.76	4.69	4.65	0.2	0.012255
Solyc04g009230.2.1	Mitosis protein dim1 (	89.96	89.92	67.49	77	81.09	243.76	225.18	207.89	225.61	2.78	0.012255
Solyc05g054200.2.1	Transcriptional adapte	14.55	18.13	19.74	18.35	17.69	5.19	6.62	7.82	6.54	0.37	0.012273
Solyc07g005660.2.1	Purine permease famil	17.65	23.74	24.13	14.18	19.92	162.16	115.52	108.08	128.59	6.45	0.012287
Solyc03g113520.2.1	Beta-1 3-galactosyltran	6.21	5.53	5	6.56	5.83	1.27	2.01	1.76	1.68	0.29	0.012311
Solyc07g053550.1.1	Glutaredoxin (AHRD V	7.08	20.88	19.33	11.9	14.8	212.84	108.24	196.01	172.36	11.65	0.012326
Solyc09g090400.2.1	Translation initiation f	10.25	8.06	7.26	9.19	8.69	1.8	3.48	1.19	2.16	0.25	0.012367
Solyc02g063380.1.1	Unknown Protein (AH	3.14	6.15	8.15	2.67	5.03	0	0	0	0	0	0.012401
Solyc06g060520.1.1	Unknown Protein (AH	1.1	4.1	1.55	0.93	1.92	0	0	0	0	0.01	0.012417
Solyc06g064630.2.1	Ribosomal protein L15	449.89	318.77	430.92	404.67	401.06	149.5	172.29	159.71	160.5	0.4	0.012417
Solyc02g063220.2.1	Mannose-6-phosphate	16.34	15.35	13.21	15.62	15.13	4.16	4.69	10.66	6.5	0.43	0.01243
Solyc08g079650.2.1	ACT domain containin	8.25	9.31	9.03	4.58	7.79	2.14	2.76	2.78	2.56	0.33	0.012436
Solyc05g032760.2.1	PHD finger family pro	37.02	31.75	46.06	32.37	36.8	7.61	13.71	4.68	8.67	0.24	0.01247
Solyc11g010220.1.1	Pentatricopeptide repe	1.48	1.12	0.67	2.42	1.42	0	0.19	0.23	0.14	0.1	0.012472
Solyc05g007140.2.1	Receptor-like protein k	19.9	11.58	12.76	20.17	16.1	3.29	4.09	7.62	5	0.31	0.012521
Solyc12g005660.1.1	CONSTANS-like zinc	3.25	1.16	4.6	0.92	2.48	0	0	0	0	0	0.012585
Solyc12g088160.1.1	Phosphoenolpyruvate c	0.2	0.27	0.24	0.57	0.32	1.23	1.48	0.9	1.2	3.76	0.012601
Solyc01g067890.2.1	1-deoxy-D-xylulose 5-	49.02	52.76	49.83	52.75	51.09	137.24	94.45	154.43	128.71	2.52	0.012604
Solyc04g077440.2.1	Squalene monooxygen	79.39	77.91	125.18	199.4	120.47	9.93	16.97	16.18	14.36	0.12	0.012604
Solyc05g050500.1.1	ATP synthase F1 delta	125.13	95.35	99.06	56.3	93.96	33.04	42.65	23	32.9	0.35	0.012638
Solyc06g076040.2.1	U-box domain-contain	68.06	66.87	90.88	152.24	94.51	6.52	14.86	28.98	16.79	0.18	0.012768
Solyc10g054670.1.1	Unknown Protein (AH	2.36	0	2.78	0	1.29	28.55	9.6	16.37	18.17	14.14	0.012808
Solyc04g055160.2.1	cDNA clone J033025P	15.7	11.33	14.27	12.9	13.55	3.94	6.22	2.53	4.23	0.31	0.012824
Solyc10g078500.1.1	Small basic intrinsic p	4.4	8.39	6.67	3.55	5.75	0.51	0	0	0.17	0.03	0.012858
Solyc04g025990.2.1	Potassium transporter	22.02	25.11	23.87	24.23	23.81	8.02	9.66	4.85	7.51	0.32	0.012892
Solyc05g007780.2.1	Oxygen evolving enha	11.56	8.78	7.62	9.14	9.28	0.82	3.05	1.01	1.63	0.18	0.012934
Solyc05g054350.2.1	Epoxide hydrolase (AH	385.99	391.3	304.32	315.88	349.37	128.12	202.51	150.69	160.44	0.46	0.012985
Solyc04g077450.2.1	AGAP001695-PA (Fra	15.96	19.39	19.19	22.57	19.28	5.7	6.5	5.79	6	0.31	0.01301
Solyc03g118740.2.1	Auxin efflux carrier (A	1.83	2.4	2.54	1.37	2.04	0.26	0.35	0.53	0.38	0.19	0.01303
Solyc08g008020.1.1	Phosphoribosylanthran	1.38	1.53	1.71	1.58	1.55	0.27	0.38	0.2	0.28	0.18	0.013049
Solyc04g016480.2.1	Calmodulin binding pr	14.13	11.57	9.28	6.13	10.28	2.2	4.45	1.91	2.85	0.28	0.013054
Solyc02g071000.1.1	Chlorophyll a/b bindin	9.67	5.52	5.85	4.91	6.49	2.6	0.4	0.25	1.08	0.17	0.013137
Solyc08g082660.2.1	Cellulose synthase (AH	0.49	1.31	0.72	0.52	0.76	0	0	0	0	0.01	0.013188
Solyc07g045100.1.1	Aspartic proteinase ne	0.48	0.75	0.42	1.02	0.67	2.42	1.17	6.19	3.26	4.88	0.0132
Solyc03g006880.2.1	Gibberellin 20-oxidase	1.04	1.86	0.41	0.98	1.07	0	0	0	0	0.01	0.01321
Solyc04g015530.1.1	Polygalacturonase A (	0	0	0	0	0	1.08	0.96	0.33	0.79	79	0.013259
Solyc10g012410.2.1	HAD superfamily hydr	16.19	16.59	17.94	14.57	16.32	5.94	3.4	4.14	4.49	0.28	0.013288
Solyc04g076710.2.1	Unknown Protein (AH	11.51	13.65	5.43	16.28	11.72	115.89	64.38	55.58	78.62	6.71	0.013367
Solyc03g082370.1.1	Avr9/Cf-9 rapidly elici	1.47	1.3	0.86	2.07	1.42	10.66	18.52	289.58	106.25	74.56	0.013399
Solyc09g065610.1.1	Transmembrane BAX	31.31	39.81	18.47	44.29	33.47	40.65	81.09	135.4	85.71	2.56	0.013421
Solyc06g071300.1.1	Genomic DNA chromo	4.53	3.59	0.59	0.71	2.36	6.51	16.41	6.5	9.81	4.16	0.013448
Solyc11g021060.1.1	Proteinase inhibitor (A	197.62	338.13	6.25	22.5	141.12	0	0.96	1.46	0.81	0.01	0.013462
Solyc08g016440.2.1	Unknown Protein (AH	9.04	19.3	9.6	7.67	11.4	113.9	53.76	111.23	92.96	8.15	0.013467
Solyc06g069470.2.1	Glycerophosphoryl die	65.47	60.36	43.88	57.61	56.83	20.67	28.39	14.2	21.09	0.37	0.013511
Solyc12g013670.1.1	Mannan endo-1 4-beta	8.52	11.54	7.87	8.39	9.08	2.4	1.51	2.58	2.16	0.24	0.013511
Solyc01g098110.2.1	Hydrolase alpha/beta f	6.22	6.92	9.17	4.95	6.82	141.45	74.66	110.91	109.01	16	0.013524
Solyc07g006270.2.1	Subunit VIb of cytochr	131.1	131.73	141.69	144.76	137.32	345.07	282.57	325.73	317.79	2.31	0.013562
Solyc11g008480.1.1	Photosystem II oxygen	1.99	2.76	2.61	1.88	2.31	0	0.54	0.22	0.25	0.11	0.013562
Solyc06g071420.2.1	Ring finger protein 12	8.18	7.75	6.5	5.79	7.05	12.93	15.65	15	14.53	2.06	0.013564
Solyc02g061990.2.1	Basic leucine zipper tr	6.27	5.58	0.43	2.09	3.59	0	0	0	0	0	0.013573
Solyc03g113980.2.1	Calmodulin-binding pr	3.22	3.3	1.75	4.55	3.21	0.5	0.4	1.97	0.96	0.3	0.013606
Solyc05g006570.1.1	Receptor like kinase, R	1.94	2.21	1.27	1.99	1.85	0.17	0.35	0.21	0.24	0.13	0.013685
Solyc11g039470.1.1	Unknown Protein (AH	9	9.79	1.18	4.24	6.05	0	0	0	0	0	0.013685
Solyc03g123430.2.1	AP2-like ethylene-resp	2.44	4.45	3.63	6.72	4.31	0.73	1.05	0.38	0.72	0.17	0.013727
Solyc01g107070.2.1	N-hydroxycinnamoyl/b	18.13	15.11	19.57	14.73	16.88	4.68	4.4	2.3	3.79	0.22	0.013743
Solyc12g013970.1.1	Enhanced disease resis	3.26	2.9	4.06	3.85	3.52	0.44	1.03	0.9	0.79	0.22	0.013828
Solyc08g005640.2.1	Ent-kaurene synthase-l	0.59	1.14	0.23	0.42	0.59	0	0	0	0	0.02	0.013887
Solyc09g082780.2.1	Asparagine synthetase	227.5	285.18	220.22	236.99	242.47	103.85	119.67	69.64	97.72	0.4	0.01389
Solyc09g007560.2.1	50S ribosomal protein	147.81	140.38	130.5	119.63	134.58	54.1	65.88	70.41	63.46	0.47	0.01389
Solyc01g103390.2.1	Flavin oxidoreductase/	4.08	3.3	2.62	3.41	3.35	0.45	0.6	0.92	0.66	0.2	0.013894
Solyc01g079470.2.1	CP12 (AHRD V1 ***-	16.36	22.08	29.89	23.89	23.05	3.41	9.46	7.33	6.73	0.29	0.013902
Solyc03g112630.2.1	Fas-associated factor 1	3.54	4.52	3.99	4.79	4.21	0.75	1.13	0.46	0.78	0.19	0.013912
Solyc03g033330.2.1	RING finger protein 44	7.63	14.56	6.75	7.33	9.07	2.2	0.22	0.27	0.9	0.1	0.014055
Solyc03g083300.2.1	Mediator of RNA poly	86.43	72.83	62.07	62.01	70.83	27.66	28.92	19.48	25.35	0.36	0.014061

Solyc04g015470.2.1	Phosphatidylinositol-4-	6.51	5.09	5.5	8.09	6.3	1.49	2.08	1.05	1.54	0.24	0.014064
Solyc11g007420.1.1	Receptor-like protein k	3.04	2.26	4.79	2.87	3.24	0	0	0	0	0	0.014071
Solyc04g058150.2.1	Type 2 metallothionein	670.56	803.5	356.11	379.65	552.46	205.16	136.97	130.07	157.4	0.28	0.014145
Solyc05g012800.1.1	UDP-N-acetylmuramo	4.37	3.77	3.13	4.87	4.04	0.75	0.97	0.79	0.84	0.21	0.014183
Solyc11g069510.1.1	GATA transcription fa	32.28	30.34	29.99	27.97	30.14	12.39	14.3	9.01	11.9	0.39	0.014185
Solyc05g009300.1.1	80C09_19 related (AH	140.96	167.39	100.07	97.95	126.59	59.92	58.77	30.5	49.73	0.39	0.014231
Solyc04g054340.1.1	Unknown Protein (AH	24.35	19.57	38.92	35.55	29.6	0.63	14.08	5.46	6.72	0.23	0.014268
Solyc09g065840.2.1	Single-stranded DNA b	13.85	12.33	9.6	12.44	12.05	1.6	5.55	4.58	3.91	0.32	0.014317
Solyc10g079110.1.1	1-acyl-sn-glycerol-3-pl	11.2	10.88	9.35	10.23	10.41	2.23	4.91	1.03	2.72	0.26	0.014362
Solyc06g062840.2.1	Threonine synthase (A	25.23	20.21	31.4	40.32	29.29	6.83	9.15	8.64	8.21	0.28	0.014373
Solyc07g014730.2.1	Phospholipase A2 (AH	2.94	4.49	1.98	0.59	2.5	0	0	0.42	0.14	0.06	0.014379
Solyc07g047760.1.1	F-box family protein (/	12.62	13.89	9.93	8.45	11.22	29.72	23.78	20.35	24.62	2.19	0.014395
Solyc05g006630.2.1	Tir-nbs-lrr, resistance p	3.96	4.56	5.75	5.12	4.85	0.94	1.77	1.15	1.29	0.27	0.014415
Solyc05g009320.2.1	LOB domain protein 4:	2.59	4.14	5.8	1.1	3.41	0.21	0.42	0.26	0.3	0.09	0.014415
Solyc01g008530.2.1	Cinnamoyl CoA reduct	3.21	3.75	3.08	2.55	3.15	7.13	12.09	4.78	8	2.54	0.014416
Solyc08g076780.1.1	Cinnamoyl-CoA reduc	0.53	0	0.62	0	0.29	25.16	12.47	28.81	22.15	77.03	0.014547
Solyc10g079050.1.1	BHLH transcription fac	0.69	0.46	0.41	0	0.39	5.04	5.36	5.67	5.36	13.74	0.014722
Solyc05g006890.1.1	Eukaryotic translation	32.16	25.32	19.46	22.75	24.92	5	9.74	8.19	7.64	0.31	0.014761
Solyc03g098660.2.1	FIP1 (AHRD V1 **-- I	4.86	3.79	2.51	3.87	3.76	0.25	0.25	0.6	0.37	0.1	0.01482
Solyc09g074410.2.1	Short-chain dehydroge	17.61	15.27	20.51	9.77	15.79	5.22	6.72	3.32	5.09	0.32	0.01482
Solyc01g005830.2.1	Glucan endo-1 3-beta-g	19.69	21.91	22.94	24.03	22.14	4.77	9.83	2.2	5.6	0.25	0.014851
Solyc04g078370.2.1	Cytochrome P450	0.52	2.08	2.6	0.37	1.39	0	0	0	0	0.01	0.014895
Solyc08g006410.2.1	UDP-glucose glucosylt	4.02	7.29	5.26	4.47	5.26	11.61	9.37	12.7	11.23	2.13	0.014895
Solyc09g010090.2.1	Beta-fructofuranosidas	2.49	2.82	2	2.72	2.51	0.18	0.92	0.22	0.44	0.18	0.014929
Solyc07g042160.2.1	Polygalacturonase (AH	8.13	8.3	6.76	11.12	8.58	1.18	4.56	1.98	2.57	0.3	0.01496
Solyc08g068770.1.1	N-acetyltransferase (A	29.59	21.75	9.93	11.9	18.29	2.19	3.54	1.89	2.54	0.14	0.014974
Solyc06g008790.2.1	Cc-nbs-lrr, resistance p	0.9	1.36	1.12	0.9	1.07	0.09	0.26	0.05	0.13	0.12	0.014976
Solyc09g056170.2.1	Fimbrin-like potential	1.79	1.85	1.17	1.96	1.69	0.24	0.16	0.2	0.2	0.12	0.015022
Solyc12g006200.1.1	Unknown Protein (AH	3.65	3.45	2.56	1.94	2.9	0.28	0.93	0.11	0.44	0.15	0.015064
Solyc07g052340.2.1	Exosome complex exo	5.74	6.63	4.84	5.06	5.57	1.57	2.61	0.59	1.59	0.29	0.01508
Solyc12g005410.1.1	Bile acid sodium symp	10	8.9	5.9	4.33	7.28	33.75	22.08	21.93	25.92	3.56	0.01508
Solyc03g113450.2.1	Receptor like kinase, F	3.84	3.9	3.88	7.13	4.69	0.8	1.25	1.52	1.19	0.25	0.015104
Solyc12g014490.1.1	Microtubule-associated	35.42	28.53	28.36	35.16	31.87	2.72	15.81	2.77	7.1	0.22	0.015113
Solyc03g120430.2.1	Glycerate kinase (AHR	29.71	25.14	21.13	27.6	25.9	11.18	11.39	11.13	11.23	0.43	0.015123
Solyc12g005400.1.1	Cyclic nucleotide gated	0.38	0.51	2.49	1.49	1.22	0	0	0.1	0.03	0.03	0.015124
Solyc01g008830.1.1	L-ascorbate oxidase (A	0.12	0.31	0.14	0.65	0.31	1.49	0.94	1.14	1.19	3.9	0.015124
Solyc02g082160.1.1	Pentatricopeptide repea	1.96	2.16	1.54	0.79	1.61	0.23	0.3	0.09	0.21	0.13	0.01519
Solyc02g091690.2.1	BHLH transcription fac	25.39	37.72	21.7	31.39	29.05	7.27	11.4	5.95	8.21	0.28	0.01519
Solyc02g083450.2.1	Aspartic proteinase-lik	0.76	2.47	1.34	1.61	1.54	0	0.1	0.13	0.08	0.05	0.015194
Solyc12g009500.1.1	UPF0405 protein C3or	16.3	16.81	20.14	16.83	17.52	5.43	5.06	7.19	5.89	0.34	0.015227
Solyc04g007780.2.1	Major latex-like protei	19.92	22.07	9.92	15.03	16.73	6.8	3.25	8.35	6.13	0.37	0.015258
Solyc06g008870.2.1	GID1-like gibberellin r	9.91	12.56	19.57	8.36	12.6	48.52	37.91	65.1	50.51	4.01	0.015258
Solyc08g079600.2.1	Os02g0448600 protein	3.61	1.38	9.12	6.56	5.17	0	0.42	0	0.14	0.03	0.015258
Solyc11g013240.1.1	Serine/threonine-prote	1.16	1.66	1.99	1.63	1.61	0.29	0.22	0	0.17	0.11	0.015258
Solyc03g116910.2.1	Cinnamoyl-CoA reduc	7.53	4.94	3.27	3.64	4.84	21.61	9.36	10.03	13.67	2.82	0.015258
Solyc09g007640.2.1	Serine carboxypeptidas	6.15	9.32	6.95	7.96	7.6	2.01	3.41	2.6	2.67	0.35	0.015263
Solyc01g102950.2.1	FAD dependent oxidor	2.49	3.18	4.47	2.14	3.07	1.31	0.53	0.64	0.83	0.27	0.015282
Solyc03g071720.2.1	Phosphatidate cytidyly	11.3	12.62	7.27	8.72	9.98	2.99	3.85	3.67	3.5	0.35	0.015288
Solyc11g065750.1.1	Armadillo repeat-conta	56.1	57.84	85.33	78.52	69.45	24.32	26.52	26.29	25.71	0.37	0.015297
Solyc11g012370.1.1	Unknown Protein (AH	0.98	3.91	10.95	5.53	5.34	0	0	0	0	0	0.015304
Solyc02g086180.2.1	Sterol C-5 desaturase (	32.02	36.7	12.88	121.1	50.67	0	0.2	0.72	0.31	0.01	0.015304
Solyc04g009550.2.1	FGFR1 oncogene partr	26.03	22.48	18.24	26.61	23.34	4.37	10.5	3.07	5.98	0.26	0.015378
Solyc04g079710.2.1	Receptor-like kinase (/	3.01	5.01	3.89	7	4.73	0.78	1.5	0.29	0.86	0.18	0.015403
Solyc01g095430.2.1	Threonine endopeptida	386.95	350.22	225.76	174.08	284.25	98.49	110.34	70.08	92.97	0.33	0.015411
Solyc04g014610.2.1	Armadillo repeat-conta	4.2	4.32	6.49	6.4	5.35	0.78	0.26	0.32	0.45	0.08	0.015411
Solyc10g045480.1.1	Calmodulin-binding pr	1.45	4.06	1.22	0.88	1.9	6.87	9.47	4.53	6.96	3.66	0.015443
Solyc01g098010.2.1	Acetyltransferase GNA	4.6	1.02	1.36	2.71	2.42	0	0	0	0	0	0.015467
Solyc01g088400.2.1	CER1 (AHRD V1 **--	11.33	16.99	11.63	8.16	12.03	1104.95	210.67	1859.94	1058.52	88.01	0.01553
Solyc08g082780.1.1	Unknown Protein (AH	3.34	5.2	14.78	4.72	7.01	0	0	0	0	0	0.015559
Solyc06g072680.2.1	Receptor expression-er	0	0.86	0	0	0.21	3.53	3.16	2.89	3.19	14.85	0.015565
Solyc04g054730.2.1	High affinity sulfate tr	2.1	4	2.82	2.3	2.8	0.77	1.09	1.33	1.06	0.38	0.015581
Solyc12g076350.1.1	Thyroid receptor-intera	6.63	12.1	12.12	9.85	10.18	2.41	2.7	1.97	2.36	0.23	0.015581
Solyc03g031840.2.1	Expansin (AHRD V1 *	261.18	278.45	248.6	292.48	270.18	100.23	132.19	97.73	110.05	0.41	0.01564
Solyc03g111670.2.1	Receptor like kinase, F	13.09	15.83	18.28	24.74	17.98	5.81	7.09	5.95	6.28	0.35	0.01564
Solyc02g061790.2.1	Activated RNA polyme	60.9	58.15	34.43	33.2	46.67	86.61	150.42	88.15	108.39	2.32	0.015663
Solyc01g067640.2.1	Cell division protein ki	16.69	17.08	15.67	17.67	16.78	6.24	7.86	4.73	6.28	0.37	0.01567
Solyc10g005640.2.1	Phosphatase 2C family	3.91	5.69	4.75	4.19	4.63	0.86	1.93	0.94	1.24	0.27	0.015759
Solyc11g067270.1.1	Acyltransferase-like pr	0.77	0.41	1.26	1.52	0.99	0	0	0	0	0.01	0.015759
Solyc06g008450.2.1	Cc-nbs-lrr, resistance p	2.58	4.36	4.91	5.14	4.25	1.11	1.33	0.37	0.94	0.22	0.015822
Solyc12g009020.1.1	Protein kinase (AHRD	56.39	44.12	48.92	42.49	47.98	19.95	15.32	15.73	17	0.35	0.015825
Solyc03g119570.1.1	Glucose-repressible alc	4.72	4.76	5.02	2.45	4.24	1.65	0.64	0.94	1.08	0.25	0.015889
Solyc04g078980.2.1	Glycogenin-like protei	1.52	2.08	0.96	0.99	1.39	0	0.1	0	0.03	0.02	0.015917
Solyc06g067890.2.1	Hydrolase alpha/beta f	4.68	6.2	7.72	8.23	6.71	2.1	1.86	2.58	2.18	0.33	0.015934
Solyc06g036060.2.1	Zinc finger family prot	9.7	11.42	8.27	13.27	10.66	1.92	4.48	3.32	3.24	0.3	0.015958
Solyc07g051930.1.1	Protein serine/threonin	1.96	2.06	3.15	0.75	1.98	10.63	6.96	7.77	8.45	4.27	0.015995
Solyc06g068980.2.1	Serine/threonine-prote	8.53	9.13	11.16	11.5	10.08	3.12	3.86	3.11	3.36	0.33	0.016035
Solyc08g006330.2.1	UDP-glucose salicylic	4.76	8.22	6.13	6.33	6.36	33.01	19.17	25.79	25.99	4.09	0.016035
Solyc09g074470.2.1	Kinase interacting fam	1.62	2.06	0.55	1.31	1.39	0	0	0	0	0.01	0.016066
Solyc11g008270.1.1	Genomic DNA chromo	225.25	240.67	344.28	290.04	275.06	123.88	115.75	116.22	118.62	0.43	0.016079
Solyc05g007050.2.1	ATP binding / serine-tl	22.45	23.78	29.74	25.52	25.37	7.68	11.78	6.15	8.54	0.34	0.016079
Solyc11g030720.1.1	Alcohol dehydrogenase	13	14.81	9.02	8.11	11.23	3.86	4.83	3.23	3.97	0.35	0.016107
Solyc12g035710.1.1	Cbs domain containing	1.87	1.54	1.89	1.89	1.8	0.32	0.33	0.13	0.26	0.14	0.016123

Solyc08g077340.2.1	F-box/LRR-repeat prot	3.4	5.9	2.29	2.52	3.53	1.18	0.53	0.32	0.68	0.19	0.016131
Solyc01g067740.2.1	Superoxide dismutase	1693.69	1367.69	1025.04	1061.85	1287.07	2922.71	2508.69	2351.37	2594.26	2.02	0.016207
Solyc12g097060.1.1	Genomic DNA chromo	4.69	6	0	3.32	3.5	0	0.24	0	0.08	0.02	0.016324
Solyc08g079510.2.1	Unknown Protein (AH	4.87	6.07	2.79	2.36	4.02	6.41	8.85	13.4	9.55	2.37	0.016399
Solyc09g057630.2.1	Glucan endo-1 3-beta-g	11.07	10.23	4.69	14.05	10.01	2.52	2.2	0.56	1.76	0.18	0.016411
Solyc08g065800.1.1	Unknown Protein (AH	5.51	6.58	10.09	9.43	7.9	1.87	2.01	1.73	1.87	0.24	0.016436
Solyc07g049390.1.1	Armadillo/beta-catenin	13.77	10.61	11.33	17.52	13.31	2.9	5.19	2.87	3.65	0.27	0.016527
Solyc08g082370.1.1	Unknown Protein (AH	43.76	45.92	18.49	12.93	30.27	0.53	6.39	0.65	2.52	0.08	0.016528
Solyc03g006250.2.1	GDSL esterase/lipase A	0.35	0.46	0	0	0.2	1.26	1.69	0	0.98	4.86	0.01653
Solyc07g041310.2.1	Ribosomal protein (AH	441.18	338.09	356.73	316.5	363.12	143.46	168.22	138.6	150.09	0.41	0.01654
Solyc10g045610.1.1	Receptor-like kinase (A	9.02	8.03	10.08	14.1	10.31	1.15	1.55	1.41	1.37	0.13	0.016552
Solyc05g006650.2.1	Transcription factor (A	84.34	92.69	139.13	155.53	117.92	26.94	44.31	23.99	31.75	0.27	0.016603
Solyc09g005860.2.1	Ankyrin repeat family	15.46	20.47	13.93	22.21	18.02	2.36	5.55	2.9	3.6	0.2	0.016681
Solyc02g092920.2.1	Cadmium-transporting	38.84	39.79	40.72	35.87	38.8	14.26	18.18	12.83	15.09	0.39	0.016767
Solyc04g081290.2.1	Lysine decarboxylase-l	26.56	35.85	56.14	34.68	38.31	11.56	9.28	9.85	10.23	0.27	0.016773
Solyc07g006760.2.1	Serine/threonine protei	12.66	14.41	16.37	9.56	13.25	4.43	5.26	3.81	4.5	0.34	0.016836
Solyc12g009000.1.1	Os07g0175100 protein	0.41	0.36	0.32	4.04	1.28	6.37	8.2	56.84	23.8	18.56	0.016841
Solyc11g008850.1.1	Serine protease (AHRI	10.07	8.57	13.84	14.4	11.72	2.59	4.06	3.08	3.24	0.28	0.016843
Solyc01g101100.2.1	Receptor-like protein k	29.03	25.95	26.61	39.05	30.16	11.2	11.93	13.51	12.21	0.4	0.016893
Solyc07g055640.1.1	Receptor-like kinase (A	0.32	0.07	0	0.57	0.24	0.65	0.72	2.07	1.15	4.78	0.016905
Solyc04g016490.2.1	Icc family phosphohyd	1.27	2.1	0.86	2.31	1.64	0	0.15	0	0.05	0.03	0.016928
Solyc05g013850.2.1	Sieve element-occludi	10.03	10.62	7.33	3.4	7.84	0.87	3.44	0.41	1.57	0.2	0.016994
Solyc05g051640.2.1	Receptor like kinase, R	0.65	0.46	0.31	0.09	0.38	0	0	0	0	0.03	0.017004
Solyc12g076320.1.1	Thyroid receptor-intera	12.12	9.99	14.83	19.68	14.15	3.63	3.29	3.56	3.49	0.25	0.017032
Solyc06g009150.1.1	POT family domain co	0	0	0	0	0	2.33	1.68	0.82	1.61	161	0.017045
Solyc12g005910.1.1	B-cell receptor-associa	44.68	56.05	53.07	52.6	51.6	17.2	19.06	27.38	21.21	0.41	0.017104
Solyc03g025230.2.1	Multidrug resistance p	8.43	9.84	8.39	10.43	9.27	1.91	3.86	1.18	2.32	0.25	0.017224
Solyc04g005560.2.1	Guanine nucleotide ex	0.91	1.22	1.08	1.94	1.29	0.07	0.52	0.23	0.27	0.21	0.017224
Solyc07g065060.1.1	Wax synthase isoform	2.49	3.23	4.74	1.62	3.02	0	0.78	0.57	0.45	0.15	0.017224
Solyc01g006450.2.1	Enoyl reductase (AHR	42.13	43.03	39.02	50.58	43.69	20.21	17.37	25.99	21.19	0.49	0.017242
Solyc10g005130.2.1	Ribonuclease 3-like pr	6.57	7.65	8.28	8.89	7.85	2.74	2.79	3.33	2.95	0.38	0.01725
Solyc01g006690.2.1	Unknown Protein (AH	3.36	3.09	6.65	6.28	4.84	2.01	0.35	1.29	1.22	0.25	0.017252
Solyc06g076630.2.1	Peroxidase (AHRD V1	72.82	68.13	54.34	88.03	70.83	20.95	32.96	18.94	24.28	0.34	0.017255
Solyc10g009110.1.1	Ethylene-responsive tr	38.16	43.71	55.15	38.92	43.98	5.26	26.04	15.56	15.62	0.36	0.017255
Solyc01g079360.2.1	WRKY transcription fa	19.16	19.98	25.19	15.88	20.05	8.4	6.69	2.17	5.75	0.29	0.017261
Solyc06g073550.2.1	La related protein-like	32.88	32.4	45.29	35.79	36.59	10.64	14.32	18.69	14.55	0.4	0.017261
Solyc11g045240.1.1	Glutamate carboxypep	14.48	17.18	22.78	19.35	18.45	3.9	3.93	4.79	4.21	0.23	0.017321
Solyc10g083400.1.1	Cytochrome P450	3.71	4.72	4.69	1.31	3.61	50.84	28.71	46.15	41.9	11.61	0.017436
Solyc09g065470.2.1	Vicilin (Fragment) (AH	0.98	3.37	8.28	2.18	3.7	0	0.11	0	0.04	0.01	0.017436
Solyc04g082020.2.1	DNL zinc finger family	43.34	49.64	39.86	31.56	41.1	106.34	96.61	66.75	89.9	2.19	0.017446
Solyc10g078370.1.1	Auxin efflux carrier (A	1.88	1.78	1.05	0.94	1.41	0.27	0.09	0.11	0.16	0.11	0.017481
Solyc09g059950.2.1	Unknown Protein (AH	0.89	2.37	0.35	0.42	1.01	3.11	5.54	4.4	4.35	4.32	0.017594
Solyc09g065670.2.1	Zinc finger family prot	19.47	17.95	25.12	28.93	22.87	8.37	9.47	3.75	7.2	0.31	0.017645
Solyc02g086460.2.1	Unknown Protein (AH	15.34	20.05	43.71	18.65	24.44	48.22	47.67	67.15	54.35	2.22	0.017659
Solyc07g063270.2.1	Nucleolar GTP-binding	101.32	99.6	161.23	106.67	117.2	39.28	49.08	44.2	44.19	0.38	0.01767
Solyc10g074960.1.1	Unknown Protein (AH	32.99	17.35	37.15	27.57	28.77	32.71	89.19	68.46	63.45	2.21	0.017709
Solyc03g111760.2.1	Argonaute 4-like prote	9.57	9.22	9	8.65	9.11	2.79	4.34	2.36	3.16	0.35	0.017739
Solyc03g082640.1.1	26S protease regulator	5.2	6.08	4.73	2.1	4.53	0.36	1.09	0.44	0.63	0.14	0.017749
Solyc09g061250.2.1	Phototropic-responsive	0.65	0.48	0.9	0.61	0.66	0	0	0	0	0.02	0.017751
Solyc02g082490.2.1	Serine/threonine phosp	7.37	7.93	6.06	5.33	6.67	2.22	2.23	1.02	1.82	0.27	0.017808
Solyc04g050950.2.1	Major latex-like protei	12.48	14.28	6.84	2.52	9.03	1.08	1.09	0.44	0.87	0.1	0.017825
Solyc02g080620.2.1	Prephenate dehydratas	2.96	3.95	4.66	5.82	4.35	0.4	1.61	0.82	0.94	0.22	0.017837
Solyc01g107840.2.1	Immediate-early salicy	1.35	2.4	0.79	1.9	1.61	81.04	21.39	54.79	52.41	32.55	0.01786
Solyc02g068530.2.1	UDP-N-acetylglucosar	69.91	76.23	66.78	84.48	74.35	25.88	34.84	21.96	27.56	0.37	0.017863
Solyc04g005550.1.1	Cc-nbs-lrr, resistance p	1.29	0.92	1.27	1.6	1.27	0.12	0.25	0.1	0.16	0.12	0.017882
Solyc03g034070.1.1	P-166-4_1 (Fragment)	3.22	6.21	7.59	4.55	5.39	0	0.44	0	0.15	0.03	0.017882
Solyc03g120890.2.1	GATA transcription fa	16.73	11.71	17.96	25.52	17.98	3.8	6.74	1.68	4.07	0.23	0.017931
Solyc09g092490.2.1	UDP-glucosyltransfera	9.34	13.14	6.4	5.54	8.61	99.54	69.47	36.48	68.5	7.96	0.017931
Solyc10g006970.2.1	Thioredoxin m (AHRD	22.46	30.29	45.13	31.77	32.41	166.7	104.94	119.47	130.37	4.02	0.017952
Solyc11g067100.1.1	60s acidic ribosomal p	822.9	753.5	921.01	883.23	845.16	349.25	336.22	328.56	338.01	0.4	0.017952
Solyc12g041880.1.1	Homology to unknown	54.6	50.38	52.06	40.67	49.43	16.03	25.24	11.95	17.74	0.36	0.01796
Solyc02g063420.2.1	Hydroxyproline-rich gl	163.07	150.92	93.18	61.77	117.23	25.9	40.51	50.26	38.89	0.33	0.018044
Solyc09g072810.2.1	Receptor like kinase, R	0.32	0.29	0.19	0.84	0.41	0	0	0	0	0.02	0.018044
Solyc07g005210.2.1	Outer membrane lipop	29.79	24.94	31.8	28.6	28.78	73.07	156.65	62.31	97.34	3.38	0.018089
Solyc01g100010.2.1	F-box protein PP2-B1 (	8.99	4	4.46	7.02	6.12	0.76	1.93	1.41	1.37	0.22	0.018116
Solyc04g071860.2.1	Palmitoyltransferase A	24.49	22.9	32.94	44.78	31.28	6.93	9.02	14.86	10.27	0.33	0.01813
Solyc06g005470.2.1	Metallothionein-like p	625.26	435.14	90.8	199.07	337.57	26.06	24.49	13.82	21.46	0.06	0.01813
Solyc10g084890.1.1	UDP-glucosyltransfera	4.19	1.29	2.28	3.19	2.74	30.75	34.7	24.5	29.98	10.95	0.018176
Solyc08g065140.1.1	Harpin-induced 1 (AH	24.15	17.28	44.06	19.37	26.22	1.01	8.72	0.74	3.49	0.13	0.01819
Solyc07g064400.1.1	Serine/threonine-prote	1.15	1.79	1.24	1.49	1.42	0.23	0.08	0.19	0.17	0.12	0.018239
Solyc10g080540.1.1	Homeobox-leucine zip	2.23	3.14	1.32	0.26	1.74	0	0.15	0	0.05	0.03	0.018239
Solyc12g056580.1.1	Cellulose synthase (AH	24.4	28.49	28.5	38.82	30.05	5.61	9.42	14.08	9.7	0.32	0.018241
Solyc08g061250.2.1	ATP binding / serine-th	7.38	8.85	4.09	2.34	5.67	1.22	0.98	0.9	1.03	0.18	0.018252
Solyc08g006680.2.1	Folate/biopterin transp	8.21	8.34	8.18	10.89	8.91	3.42	2.51	1.53	2.49	0.28	0.018338
Solyc04g040200.2.1	Cellular retinaldehyde-	91.2	90.99	81.55	80.3	86.01	31.85	41.98	23.22	32.35	0.38	0.018344
Solyc05g041450.2.1	Unknown Protein (AH	4.28	5.34	6.06	4.85	5.13	0	0	0.85	0.28	0.06	0.018355
Solyc07g007120.2.1	Homeobox protein kno	229.98	210.39	239.08	225.08	226.13	88.21	111.99	103.98	101.39	0.45	0.01836
Solyc07g064230.2.1	TBC1 domain family m	6.81	5.51	5.3	4.38	5.5	1.13	2.27	0.61	1.34	0.24	0.018377
Solyc05g014160.2.1	Genomic DNA chromo	10.9	11.3	12.05	12.49	11.69	3.9	4.05	4.93	4.29	0.37	0.018418
Solyc03g063560.2.1	Glutamate synthase (Fe	47.26	53.57	63.77	57.07	55.42	18.37	24	21.56	21.31	0.38	0.018489
Solyc04g074410.1.1	Os06g0220000 protein	15.18	15.94	9.7	26.24	16.76	1.36	2.75	11.09	5.07	0.3	0.018522
Solyc11g006270.1.1	3-oxo-5-alpha-steroid 4	8.89	18.47	12.24	5.24	11.21	85.64	34.23	41.69	53.85	4.8	0.018522

Solyc08g079850.1.1	Subtilisin-like protease	0.53	0.55	0.52	0.5	0.53	0	0	0	0	0.02	0.018563
Solyc03g034170.2.1	Uncharacterized conse	37.37	39.01	23.4	20.56	30.09	12.67	12.97	5.95	10.53	0.35	0.018568
Solyc06g060680.1.1	Receptor-like protein k	6.08	3.01	2.39	7.64	4.78	11.1	6.61	32.41	16.71	3.5	0.018662
Solyc06g076320.1.1	DVL13 (AHRD V1 *-*	2.7	4.33	1.28	2.29	2.65	441.68	146.73	127.21	238.54	90.02	0.018693
Solyc03g119660.1.1	Cell number regulator	1.43	2.26	0.94	1.57	1.55	0	0.13	0	0.04	0.03	0.018779
Solyc07g052670.2.1	Transcription factor (F	8.89	10.54	8.58	6.86	8.72	1.09	3.51	0.27	1.62	0.19	0.018779
Solyc09g098490.2.1	Epsin 2 (AHRD V1 *-*	1.18	1.05	0.15	0.74	0.78	0.05	0.11	0	0.05	0.07	0.018789
Solyc02g091390.2.1	Little protein 1 (AHRD	11.14	9.15	1.01	3.64	6.24	0	0	0	0	0	0.018829
Solyc02g077720.2.1	Unknown Protein (AH	17.74	13.36	6.44	5.15	10.67	50.36	30.77	32.52	37.88	3.55	0.018866
Solyc01g102940.2.1	Methionine s-methyltra	10.1	10.83	14	13.69	12.15	3.88	3.87	2.96	3.57	0.29	0.018889
Solyc07g045540.2.1	Glucose-6-phosphate 1	21.12	23.87	17.51	15.82	19.58	4.43	10.32	4.64	6.46	0.33	0.019056
Solyc10g047030.1.1	Beta-xylosidase 4 (AH	0	0.23	0	0.24	0.12	10.37	3.42	14.52	9.44	80.31	0.019056
Solyc01g099820.2.1	Hypoxia induced prote	6.53	7.56	9.25	8.32	7.92	1.06	0	0.65	0.57	0.07	0.019086
Solyc10g006040.1.1	Serine acetyltransferas	7.75	11.55	5.74	5.86	7.72	3.35	2.64	0.72	2.24	0.29	0.019086
Solyc01g103470.2.1	Unknown Protein (AH	5.64	8.16	5.41	17.47	9.17	0.86	0	0.7	0.52	0.06	0.019119
Solyc08g008380.2.1	Auxin response factor	15.05	16.4	19.23	17.46	17.04	6.31	6.99	7.18	6.83	0.4	0.019156
Solyc05g018880.1.1	Polyamine oxidase (AH	1.16	0.57	0.91	0.36	0.75	0	0	0	0	0.01	0.019159
Solyc03g116390.2.1	Late embryogenesis ab	18.41	22.19	12.22	13.02	16.46	2.48	4.38	7.99	4.95	0.3	0.019159
Solyc07g042150.2.1	Ubiquinone biosynthes	28.09	20.64	24.6	31	26.08	8.6	13.87	6.34	9.6	0.37	0.019159
Solyc12g099140.1.1	MYB transcription fac	0	0	0	0.31	0.08	0.89	1.25	1.31	1.15	14.84	0.019159
Solyc01g110680.2.1	Auxin-induced SAUR-	14.14	14.68	4.63	4.44	9.47	0.63	1.28	2.34	1.42	0.15	0.019208
Solyc04g072660.2.1	Ribosomal protein L30	414.34	452.72	260.12	239.84	341.75	156.16	101.86	110.86	122.96	0.36	0.019208
Solyc03g120620.2.1	Homeobox-leucine zip	14.96	14.38	17.65	17.8	16.2	6.8	5.47	5.91	6.06	0.37	0.019208
Solyc11g066380.1.1	Auxin-regulated protei	15.1	17.63	15.77	18.21	16.68	5.6	7.67	5.41	6.23	0.37	0.01922
Solyc01g014250.2.1	Unknown Protein (AH	9.96	33.23	7.34	3.52	13.51	79.44	39.56	48.19	55.73	4.12	0.019242
Solyc02g092940.2.1	Receptor like kinase, R	19.56	19.77	23.38	29.49	23.05	9.29	7.34	8.09	8.24	0.36	0.019242
Solyc02g078700.2.1	C20orf11 homolog (AI	2.42	7.38	7.33	7.33	6.12	0.84	1.41	0	0.75	0.12	0.019243
Solyc05g009350.2.1	Zinc finger CCCH dom	13.15	13.18	22.7	14.23	15.82	4.8	4.78	4.32	4.63	0.29	0.019243
Solyc12g008950.1.1	At1g17490/F1L3_4 (A	14.81	11.98	19.46	14.28	15.13	4.62	3.57	1.34	3.18	0.21	0.019243
Solyc08g068600.2.1	Decarboxylase family	27.12	10.08	4.95	18.98	15.28	1.58	0.46	1.39	1.14	0.07	0.019305
Solyc08g068320.2.1	Transcription factor m	2.98	2.94	2.62	3.32	2.96	0.56	1.14	0.63	0.78	0.26	0.01935
Solyc09g061840.2.1	3-ketoacyl CoA thiolas	104.37	83.12	172.01	50.84	102.59	8.86	32	10.46	17.11	0.17	0.01935
Solyc06g060170.2.1	Glycoside hydrolase fa	1.48	1.08	1.11	1.14	1.2	4.03	2.86	5.09	3.99	3.32	0.019373
Solyc11g006130.1.1	Methyltransferase-like	4.35	8.26	4.89	5.03	5.63	23.78	15.61	11.18	16.86	2.99	0.019389
Solyc08g075920.2.1	Protein BREVIS RAD	9.92	7.79	9.87	22.57	12.54	0.63	1.74	1.55	1.31	0.1	0.019509
Solyc05g007510.2.1	RNA-dependent RNA	35.5	46.29	61.43	34.06	44.32	10.42	15.33	13.21	12.99	0.29	0.019528
Solyc05g024430.2.1	Unknown Protein (AH	41.52	62.26	44.95	38.61	46.84	17	20.85	3.95	13.93	0.3	0.019532
Solyc11g069260.1.1	Cobalt import ATP-bin	13.14	17.77	15.51	13.23	14.91	6.13	4.74	4.77	5.21	0.35	0.019541
Solyc12g095980.1.1	Harpin-induced 1 (AH	13.43	7.76	13.62	30.33	16.28	0.38	2.5	0	0.96	0.06	0.019651
Solyc01g109860.2.1	Phosphatidylinositol/pl	2.75	0.82	3.52	1.46	2.14	0.28	0.28	0.11	0.22	0.1	0.019667
Solyc03g006750.1.1	Nbs, resistance protein	2.57	4.32	5.05	5.25	4.3	0.23	0.7	0.85	0.59	0.14	0.019679
Solyc08g074240.2.1	40S ribosomal protein	663.41	570.23	696.75	672.25	650.66	230.89	271.15	253.29	251.78	0.39	0.019707
Solyc04g070970.2.1	ABC transporter G fan	8.58	12.51	1.18	4	6.57	0.15	0.3	0.18	0.21	0.03	0.01977
Solyc07g008920.2.1	Oxysterol-binding prot	0.17	0	0	0.24	0.1	0.69	1.54	0.17	0.8	7.8	0.019806
Solyc01g096190.2.1	Calcium-transporting A	1.7	3.02	3.71	4.36	3.2	0.76	0.51	0.69	0.65	0.2	0.019848
Solyc10g044890.1.1	DMI1 protein (Fragme	8.58	8.81	5.06	5.48	6.98	1.93	3.16	1.47	2.19	0.31	0.019873
Solyc01g056990.2.1	Genomic DNA chromo	4.71	3.7	3.92	3.92	4.06	0	0.23	0.28	0.17	0.04	0.01994
Solyc04g016160.2.1	F-box/LRR-repeat prot	2.78	2.69	3.86	3.08	3.1	0.29	1.09	0.24	0.54	0.17	0.01994
Solyc01g103250.2.1	Histone-lysine N-meth	86.82	89.6	78.3	78.95	83.42	22.92	33.87	12.83	23.21	0.28	0.020066
Solyc05g023770.2.1	Unknown Protein (AH	24.53	19.18	26.01	25.79	23.88	5.36	12.02	4.44	7.27	0.3	0.020087
Solyc03g006020.2.1	Ubiquitin domain cont	14.14	16.78	6.25	9.16	11.58	29.03	23.04	18.71	23.59	2.04	0.020122
Solyc01g010220.2.1	Unknown Protein (AH	22.85	21.98	10.87	15.64	17.84	2.68	8.11	2.56	4.45	0.25	0.020139
Solyc07g043460.2.1	Cytochrome P450 (AH	16.33	22.37	31.31	83.39	38.35	0.21	1.66	0.25	0.71	0.02	0.020146
Solyc11g006750.1.1	Unknown Protein (AH	83.41	56.69	55.47	40.76	59.08	237.09	321.31	180.64	246.35	4.17	0.020166
Solyc03g032210.2.1	Acyl-CoA synthetase/A	4.26	4.98	7.27	3.96	5.12	1.18	2.19	0.44	1.27	0.25	0.020168
Solyc08g080630.2.1	Chymotrypsin inhibito	0	0.49	0	0	0.12	25.32	119.16	3.27	49.25	402.04	0.020168
Solyc12g010230.1.1	Os06g0183700 protein	7.91	7.7	2.92	4.89	5.86	2	1.41	0.49	1.3	0.22	0.020223
Solyc02g071350.2.1	Lipid A export ATP-bi	0.1	0.19	0.43	0.59	0.33	0	0	0	0	0.03	0.020391
Solyc03g123990.2.1	Mitochondrial carrier p	10.58	7.82	8.67	7.35	8.61	2.32	2.92	3.02	2.75	0.32	0.020462
Solyc03g093360.2.1	Wound/stress protein (	778.46	1190.91	181.61	396.07	636.76	58.33	58.83	27.28	48.15	0.08	0.020553
Solyc04g026020.2.1	Sieve element-occludi	1.09	1.56	0.69	0.24	0.9	0	0.07	0	0.02	0.03	0.02062
Solyc01g073750.2.1	Glucan synthase like 3	28.86	43.02	48.76	44.61	41.31	13.66	16.08	17.03	15.59	0.38	0.02069
Solyc06g060450.2.1	Transmembrane emp24	0.31	0	0	0	0.08	16.29	8.46	6.37	10.37	133.85	0.020712
Solyc12g044810.1.1	Multidrug resistance p	1.99	2.12	2.81	7.87	3.7	0	0	0	0	0	0.020722
Solyc11g071970.1.1	Serine/threonine-prote	4.16	4.01	5.72	7.23	5.28	1.12	2.47	0.34	1.31	0.25	0.020765
Solyc02g024070.2.1	Class III homeodomain	1.01	1.79	1.1	0.77	1.17	0.19	0.32	0	0.17	0.15	0.02087
Solyc11g069710.1.1	ABC transporter G fan	8.84	9.49	10.11	7.35	8.95	1.62	3.34	1.09	2.02	0.23	0.020897
Solyc05g018060.1.1	Unknown Protein (AH	9.04	8.85	9.6	15.34	10.71	28.47	16.2	27.81	24.16	2.26	0.020907
Solyc10g085080.1.1	Unknown Protein (AH	9.95	8.45	4.37	4.58	6.84	1.12	2.64	0.92	1.56	0.23	0.02094
Solyc08g083410.1.1	cytochrome P450	0.63	2.36	3.73	1.97	2.17	0.2	0.41	0.13	0.25	0.11	0.021076
Solyc10g055620.1.1	Heparan-alpha-glucosa	4.62	5.94	2.73	1.82	3.78	0.21	0.84	0.51	0.52	0.14	0.021171
Solyc11g018690.1.1	ABC transporter G fan	7.5	8.22	8.17	5.71	7.4	1.55	2.66	2.29	2.17	0.29	0.021178
Solyc04g015480.2.1	cDNA clone 006-204-I	23.52	19.45	16.45	17.66	19.27	38.5	40.36	43.17	40.68	2.11	0.021266
Solyc05g007130.2.1	Calmodulin binding pr	18.52	15.85	15.51	15.72	16.4	5.41	8.08	6.05	6.51	0.4	0.02129
Solyc12g014420.1.1	Glucan endo-1 3-beta-g	5.62	6.84	3.07	1.94	4.37	1	0.89	0.41	0.77	0.18	0.02131
Solyc12g009070.1.1	Unknown Protein (AH	6.94	3.09	3.07	7.37	5.12	0	0	0	0	0	0.021426
Solyc10g086760.1.1	Tubulin beta chain (AI	59.36	49.43	29.82	66.92	51.38	10.92	21.55	6.85	13.11	0.26	0.02146
Solyc01g102840.2.1	Tir-nbs, resistance prot	3.36	3.28	2.56	6.75	3.99	7.36	5.31	15.08	9.25	2.32	0.021468
Solyc02g063330.2.1	Serine/threonine prote	1.26	2.81	1.04	1.61	1.68	0.2	0.31	0.25	0.25	0.15	0.021476
Solyc12g099970.1.1	5-amp-activated protei	58.24	60.54	78.37	85.59	70.69	22.61	38.37	23.93	28.3	0.4	0.02153
Solyc05g052500.2.1	Dehydration-responsiv	9.63	8.65	8.17	10.16	9.15	2.25	4.89	2.35	3.16	0.35	0.021558
Solyc06g072380.2.1	Dihydroxyacetone/glyc	28.61	29.01	34.14	27.76	29.88	10.84	14.37	13.1	12.77	0.43	0.0216

Solyc11g062060.1.1	Zinc finger protein (AH	0.76	2.02	1.19	0.36	1.08	6.92	3.49	7	5.8	5.36	0.0216
Solyc10g082030.1.1	Peroxiredoxin (AHRD	161.97	149.63	129.82	103.76	136.3	58.67	67.4	55.71	60.59	0.44	0.021657
Solyc10g074820.1.1	Unknown Protein (AH	13.53	13.34	6.63	17.07	12.64	3.87	4.75	2.48	3.7	0.29	0.021675
Solyc10g044840.1.1	PIN domain containing	24.49	21.4	16.92	20.91	20.93	8.56	9.35	2.19	6.7	0.32	0.021683
Solyc11g011880.1.1	RLK, Receptor like pro	4.1	1.83	3.27	6.53	3.93	0.17	1	0.41	0.53	0.13	0.021716
Solyc09g059470.2.1	Os01g0917200 protein	4.23	3.44	2.99	5.3	3.99	0.39	1.38	0.84	0.87	0.22	0.02175
Solyc10g079420.1.1	Calmodulin (AHRD V	89.99	45.32	35.14	72.93	60.84	8.8	16.96	18.44	14.73	0.24	0.021826
Solyc07g065340.1.1	Serine acetyltransferas	11.53	13.26	12.19	7.31	11.07	103.87	43.56	94.67	80.7	7.29	0.021855
Solyc01g094830.2.1	Receptor-like protein k	4.61	5.75	3.5	3.59	4.36	1.71	2.16	1.16	1.68	0.38	0.021889
Solyc09g011750.2.1	Receptor-like protein k	1.21	1.31	1.27	0.76	1.14	0	0.22	0	0.07	0.06	0.021898
Solyc10g044510.1.1	MLO-like protein 4 (A	2.83	4.41	2.23	4.01	3.37	1.14	0.58	1.05	0.92	0.27	0.021922
Solyc08g067310.1.1	CBL-interacting protei	0	0.26	0.17	0	0.11	2.47	3.43	1.15	2.35	21.86	0.021925
Solyc08g061910.2.1	Unknown Protein (AH	5.78	9.36	8.99	8.55	8.17	1.7	2.57	1.04	1.77	0.22	0.021939
Solyc05g042120.2.1	Unknown Protein (AH	18.26	18.98	15.57	12.5	16.33	4.19	7.58	3.86	5.21	0.32	0.02201
Solyc08g082130.2.1	Unknown Protein (AH	2.24	2.65	2.64	5.8	3.33	0.3	0.3	0	0.2	0.06	0.022104
Solyc11g069930.1.1	Disease resistance prot	36.32	42.88	34.11	30.41	35.93	14.37	9.66	7.36	10.46	0.29	0.022106
Solyc02g081360.2.1	Long-chain-fatty-acid--	4.35	1.43	2.57	9.23	4.39	0.19	0	0	0.06	0.01	0.02213
Solyc04g053130.2.1	LHC-related protein (A	500.17	542.21	476.52	363.27	470.54	1113.56	1048.82	677.21	946.53	2.01	0.022144
Solyc00g044350.2.1	EPIDERMAL PATTE	5.77	8.07	2.92	1.17	4.48	0	0	0	0	0	0.022271
Solyc08g014340.2.1	Cysteine synthase (AH	155.81	154.9	145.61	173.13	157.36	42.56	86.54	55.84	61.65	0.39	0.022529
Solyc01g100970.2.1	Pyrrolidone-carboxylat	585.04	509.48	558.62	525.11	544.56	222.4	238.73	239.31	233.48	0.43	0.022545
Solyc08g067320.1.1	Chlorophyll a/b bindin	20.08	18.51	5.92	13.19	14.43	4.06	4.09	1.42	3.19	0.22	0.022556
Solyc03g096850.2.1	Genomic DNA chromo	185.14	152.78	144.78	112.38	148.77	64	80.35	51.17	65.17	0.44	0.022565
Solyc08g082860.2.1	Alpha alpha-trehalase	4.99	7.16	6.15	4.17	5.62	1.74	2.49	1.24	1.82	0.32	0.022692
Solyc09g009190.2.1	1 4-alpha-glucan branc	35.73	29.99	16.86	17.34	24.98	6.44	8.64	4.55	6.54	0.26	0.022732
Solyc02g065350.2.1	B3 domain-containing	33.18	35.37	23.21	27.83	29.9	10.6	11.33	9.86	10.6	0.35	0.022758
Solyc01g018020.1.1	Transketolase-like prot	0	0	0	0	0	0.27	0.2	0.17	0.21	21.33	0.022798
Solyc09g065260.1.1	Blue copper protein (A	3.7	6.89	20.65	4.28	8.88	0.27	0	0	0.09	0.01	0.022811
Solyc03g112200.1.1	Unknown Protein (AH	7.85	10.95	14.83	8.52	10.54	25.59	18.13	20.53	21.42	2.03	0.022844
Solyc04g071470.2.1	Chromosome 15 contig	14.44	18.88	9.89	10.09	13.32	3.89	6.1	1.95	3.98	0.3	0.022926
Solyc09g005880.1.1	Unknown Protein (AH	0.87	1.43	0.52	2.27	1.27	0.12	0	0	0.04	0.03	0.023063
Solyc10g079600.1.1	Response regulator 6 (	17.07	19.06	22.19	24.56	20.72	9.12	6.37	5.46	6.98	0.34	0.023086
Solyc02g032800.1.1	Arabidopsis thaliana g	18.02	17.94	45.76	25.49	26.8	4.19	6.22	2.43	4.28	0.16	0.0231
Solyc04g049840.2.1	CCR4-NOT transcripti	4.67	5.86	4.51	4.01	4.76	1.92	1.37	0.91	1.4	0.29	0.023112
Solyc01g058720.2.1	NaCl-inducible Ca2+-b	5.5	1.63	1.62	1.94	2.67	0	0	0	0	0	0.023118
Solyc09g084450.2.1	Chymotrypsin inhibito	26.51	35.12	2.08	5	17.18	0.48	0	0	0.16	0.01	0.023159
Solyc11g007160.1.1	RNA binding protein (	7.9	5.86	5.24	4.65	5.91	1.59	2.55	0.49	1.54	0.26	0.023245
Solyc05g007560.2.1	60S ribosomal protein	381.65	361.99	427.11	354.75	381.38	176.63	143.5	169.93	163.35	0.43	0.02326
Solyc08g006880.2.1	ABC transporter C fam	12.16	14.33	14.02	8.95	12.37	4.46	4.82	4.51	4.6	0.37	0.023279
Solyc06g063050.2.1	COP1-interacting prote	1.4	0.71	0.71	1.98	1.2	2.42	2.52	2.77	2.57	2.14	0.023384
Solyc03g119740.2.1	MYB transcription fac	48.82	39.14	45.4	30.79	41.04	116.55	91.57	81.85	96.66	2.36	0.023387
Solyc10g074550.1.1	Unknown Protein (AH	36.18	43.56	35.15	18.06	33.24	3.44	6.94	4.22	4.87	0.15	0.023397
Solyc02g077970.2.1	Late embryo abundanc	0.95	0.42	1.12	0.34	0.71	2.31	4.27	0.95	2.51	3.55	0.023511
Solyc03g006050.2.1	Leucine rich repeat far	9.42	6.17	6.54	7.06	7.3	1.34	2.71	0.28	1.44	0.2	0.023511
Solyc10g074890.1.1	Receptor like kinase, R	2.26	3.5	3.09	1.96	2.7	0.94	0.77	0.14	0.62	0.23	0.023521
Solyc06g072230.1.1	Kunitz trypsin inhibito	1.97	3.8	0.39	1.39	1.89	0	0	0	0	0.01	0.023582
Solyc06g071880.2.1	60S ribosomal protein	598.52	489.28	505.34	484.16	519.32	206.24	254.37	208.31	222.97	0.43	0.023612
Solyc10g007110.2.1	Tyrosine aminotransfer	15.04	15.54	14.31	15.1	15	276.81	123.85	246.04	215.57	14.37	0.023707
Solyc03g083250.2.1	ATP-dependent Clp pr	24.03	26.94	21.67	23.04	23.92	95.68	60.63	84.98	80.43	3.36	0.023797
Solyc03g111930.2.1	Cytochrome P450	0	0	0	0	0	2.25	0.54	1.32	1.37	137	0.023895
Solyc01g095940.2.1	O-acyltransferase WSI	90.53	89.14	84.65	26.56	72.72	952.76	424.57	722.69	700.01	9.63	0.023924
Solyc12g087980.1.1	Hydroxycinnamoyl Co	20.71	26.2	15.71	9.83	18.11	7.29	5.3	3.96	5.52	0.3	0.023924
Solyc12g088750.1.1	Receptor-like protein k	6.87	6.12	8.27	9.35	7.65	1.17	2.8	1.97	1.98	0.26	0.023946
Solyc12g096930.1.1	Caleosin (AHRD V1 *	1.07	1.67	2.53	0.76	1.51	0	0	0	0	0.01	0.023956
Solyc10g086690.1.1	Phosphatidylcholine:c	2.69	3.99	4.77	3.81	3.81	6.35	7.86	19.6	11.27	2.95	0.02397
Solyc02g022930.2.1	3-hydroxyisobutyrate d	14.34	19.4	12.86	8.11	13.68	3.86	5.3	2.09	3.75	0.27	0.024011
Solyc10g078770.1.1	Seed maturation protei	1.48	1.98	0	0	0.86	14.97	29.6	21.34	21.97	25.4	0.024019
Solyc08g079680.2.1	Ycf49 protein (AHRD	4.99	6.03	3.79	4.04	4.71	0.29	1.16	0.35	0.6	0.13	0.024042
Solyc08g067240.2.1	DNA topoisomerase 2-	6.16	6.55	5.84	9.27	6.96	1.84	2.18	2.52	2.18	0.31	0.024078
Solyc07g063620.2.1	Uncharacterized secret	13.86	15.1	22.84	16.91	17.18	33.6	19.87	57.65	37.04	2.16	0.024092
Solyc02g092260.2.1	Amino-acid N-acetyltr	10.87	10.66	12.3	9.1	10.73	3.58	4.25	3.63	3.82	0.36	0.024154
Solyc05g050630.2.1	Protein TIF31 homolog	3.73	3.84	4.05	3.59	3.8	1.16	1.52	0.84	1.17	0.31	0.024155
Solyc10g011950.1.1	Unknown Protein (AH	2.67	2.04	6.75	1.08	3.14	0	0	0	0	0	0.024155
Solyc02g089780.2.1	cDNA clone 001-043-A	3.24	3.37	5.74	6.88	4.81	65.09	55.52	35.42	52.01	10.82	0.024164
Solyc02g067790.2.1	Nitrate transporter (AH	0	0.25	0	0	0.06	3.01	1.52	2.41	2.31	37.01	0.024271
Solyc05g012620.2.1	Hemimethylated DNA	32.5	36.76	35.34	24.92	32.38	14.87	13.08	12.24	13.4	0.41	0.02431
Solyc10g017980.1.1	Chitinase (AHRD V1 *	4.12	0	3.65	10.21	4.5	90.77	275.51	28.65	131.64	29.29	0.024314
Solyc01g005290.2.1	SEC14 cytosolic factor	24.2	25.08	12.91	8.91	17.77	63.48	66.99	29.29	53.25	3	0.024464
Solyc03g006980.2.1	Alpha-L-fucosidase 1 (	1.63	1.81	1.6	1.15	1.55	0.33	0.22	0	0.18	0.12	0.024484
Solyc06g071310.2.1	LIM domain protein (A	49.48	44.69	31.13	46.14	42.86	16.58	19.11	10.19	15.29	0.36	0.024532
Solyc03g117180.2.1	Serine/threonine-prote	8.26	9.46	10.8	11.27	9.95	3.58	2.89	1.9	2.79	0.28	0.024635
Solyc07g018290.2.1	AP2-like ethylene-resp	0.84	1.13	0.33	0.8	0.77	0	0	0	0	0.01	0.02467
Solyc03g096900.2.1	GTP pyrophosphokinas	7.09	7.11	5.87	3.31	5.84	2.13	1.97	0.82	1.64	0.28	0.024685
Solyc08g078850.2.1	L-lactate dehydrogenas	2.63	3.18	4.21	8.24	4.56	0.3	0.77	0	0.36	0.08	0.024722
Solyc04g077340.2.1	Serine/threonine-prote	0.37	0.33	0.11	0	0.2	2.53	1.5	0.55	1.53	7.54	0.024784
Solyc06g082140.2.1	Unknown Protein (AH	3001.68	3540.9	2352.92	2652.32	2886.95	1338.61	1321.2	891.57	1183.79	0.41	0.024788
Solyc12g098710.1.1	15-cis-zeta-carotene is	9.98	14.44	11.57	16.14	13.03	668.1	163.89	1471.23	767.74	58.91	0.024795
Solyc08g006740.2.1	Decarboxylase family	1.27	0.88	0.33	1.6	1.02	0	0	0	0	0.01	0.024837
Solyc10g054800.1.1	Aquaporin (AHRD V1	1.81	1.25	0.47	0.85	1.09	0	0	0	0	0.01	0.024906
Solyc12g098490.1.1	Serine hydroxymethylt	7.27	5.22	7.92	11.27	7.92	2.48	3.53	0.14	2.05	0.26	0.024938
Solyc07g047770.2.1	Histidine kinase 1 (AH	14.36	18.48	27.46	22.88	20.8	6.64	7.92	9.17	7.91	0.38	0.024974
Solyc01g005330.2.1	microtubule associated	49.88	46.09	49.6	66.35	52.98	15.23	25.11	10.93	17.09	0.32	0.025047

Solyc02g071110.2.1	Purine permease famil	9.55	10.97	5.94	7.37	8.46	134.93	67.2	55.66	85.93	10.16	0.025047
Solyc08g065220.2.1	Glycine dehydrogenase	61.96	58.2	84.29	82.7	71.79	22.46	32.4	25.95	26.94	0.38	0.025047
Solyc06g008770.1.1	Cc-nbs-lrr, resistance p	1.4	1.72	2.53	3.42	2.27	0.48	0.7	0.27	0.48	0.21	0.025056
Solyc02g085010.1.1	Unknown Protein (AH	7.57	12.51	3.19	10.71	8.5	1.75	0.88	2.68	1.77	0.21	0.025154
Solyc06g069530.2.1	Acetyl-CoA carboxylas	54.64	46.84	51.69	68.38	55.39	19.33	25.44	21.49	22.09	0.4	0.02517
Solyc08g075880.2.1	Metal ion binding prote	13.56	15.98	1.73	0.52	7.95	0	0	0	0	0	0.025178
Solyc06g036180.2.1	Unknown Protein (AH	8.65	12.51	5.1	9.18	8.86	0	0	1.07	0.36	0.04	0.025293
Solyc02g090020.2.1	Stress regulated protei	18.55	22.17	16.57	17.61	18.73	4.07	9.72	3.42	5.74	0.31	0.025367
Solyc01g111330.2.1	Thaumatin-like protein	0.74	0.66	1.09	1.83	1.08	0	0	0	0	0.01	0.02539
Solyc01g058160.2.1	Agenet domain-contain	11.28	8.87	11.09	8.24	9.87	1.82	3.52	2.05	2.46	0.25	0.025392
Solyc04g074130.2.1	Mitochondrial carrier p	6.54	7.71	7.53	5.81	6.9	2.09	1.86	1.21	1.72	0.25	0.025485
Solyc02g077220.2.1	Isoamyl acetate-hydrol	16.15	13.16	11.3	9.29	12.47	3.98	4.91	2.17	3.69	0.3	0.025495
Solyc06g053980.2.1	Chlorophyllase 2 (AHI	12.22	13.51	9.95	20.27	13.99	3.24	5.67	3.14	4.02	0.29	0.02559
Solyc02g091600.1.1	Os08g0119500 protein	4.28	2.8	1.35	4.85	3.32	0.46	0.23	1.7	0.8	0.24	0.025593
Solyc07g007750.2.1	Defensin protein (AHR	4965.82	7740.33	931.96	1723.31	3840.36	308.72	175.93	90.99	191.88	0.05	0.025769
Solyc11g020610.1.1	Neutral invertase like p	9.11	10.19	13.94	10.97	11.05	2.98	3.61	3.3	3.3	0.3	0.025807
Solyc05g042030.2.1	BAH-PHD domain-con	145.88	146.1	155.66	130.99	144.66	58.3	66.38	52.88	59.19	0.41	0.025818
Solyc01g066040.2.1	Unknown Protein (AH	0	0.52	0	0	0.13	1.4	2.83	0	1.41	10.85	0.025842
Solyc10g006300.2.1	Purple acid phosphatas	1.35	1.74	2.3	0.85	1.56	0.24	0.24	1.04	0.51	0.32	0.025842
Solyc02g067530.2.1	Prolyl 4-hydroxylase a	19.92	18.09	14.44	28.76	20.3	5.53	8.96	8.24	7.58	0.37	0.025846
Solyc06g074010.2.1	Transcription factor (E	7.47	7.2	9.27	6.27	7.55	2.89	2.41	3.24	2.85	0.38	0.025856
Solyc01g006160.2.1	Unknown Protein (AH	1088.52	922.08	1106.45	1126.15	1060.8	413.67	495.42	328.16	412.42	0.39	0.025867
Solyc03g121330.2.1	60S ribosomal protein	739.43	579.39	778.32	751.71	712.21	283.89	325.16	286.48	298.51	0.42	0.025875
Solyc05g009500.2.1	Peptide transporter (AI	1.84	2.86	3.25	0.98	2.23	0.65	0.37	0.11	0.38	0.17	0.025878
Solyc01g108580.2.1	Gibberellin receptor G	75.24	61.53	56.94	80.2	68.48	149.51	105.63	161.72	138.95	2.03	0.025886
Solyc12g049300.1.1	MYB transcription fac	0	0	0	0	0	0.91	1.07	0.74	0.91	90.67	0.025913
Solyc08g005790.2.1	Pectinacetylesterase (F	0	0	0	0	0	1.71	0.29	1.05	1.02	101.67	0.02593
Solyc07g018350.2.1	DNA mismatch repair	14.79	20.03	13.8	12.07	15.17	5.25	7.28	3.95	5.49	0.36	0.02596
Solyc05g009880.2.1	Transcription factor bH	1.32	3.51	1.03	1.24	1.78	0	0.18	0	0.06	0.03	0.026016
Solyc04g083160.1.1	cytochrome P450	0.9	0.8	1.22	0.73	0.91	0	0	0.13	0.04	0.05	0.02607
Solyc09g011580.2.1	Glutathione S-transfera	86.55	78.91	62.12	26.97	63.64	180.68	83.84	293.47	186	2.92	0.02616
Solyc01g058390.2.1	Galactokinase (AHRD	28.71	34.22	32.93	23.1	29.74	10.43	14.81	4.97	10.07	0.34	0.026188
Solyc05g006740.2.1	Glutathione S-transfera	71.49	72.37	46.7	64.67	63.81	14.13	22	16.45	17.53	0.27	0.026188
Solyc04g073970.2.1	cDNA clone J033025P	72.84	69.32	50.27	100.44	73.22	23.5	33.79	20.01	25.77	0.35	0.026188
Solyc08g083060.2.1	Oligopeptide transport	45.01	55.62	19.81	19.53	34.99	6.98	8.91	3.25	6.38	0.18	0.026188
Solyc08g067050.2.1	Arginine N-methyltran	10.46	7.42	5.23	2.76	6.47	0.43	1.01	0.35	0.6	0.09	0.02637
Solyc03g006260.2.1	Sodium/calcium excha	3.23	3.29	1.46	1.07	2.26	0.23	0.54	0.19	0.32	0.14	0.026382
Solyc01g091220.2.1	40S ribosomal protein	145.4	145.63	150.03	141.82	145.72	54.7	67.55	65.48	62.58	0.43	0.026428
Solyc02g072050.1.1	Os05g0272200 protein	0.79	1.05	0.78	0.19	0.7	0	0	0	0	0.01	0.026433
Solyc04g074760.2.1	SKIP interacting protei	4.01	6.78	8.28	3.12	5.55	1.62	1.31	0.8	1.24	0.22	0.026468
Solyc06g053810.2.1	At1g04280-like protein	6.69	7.52	7.74	3.21	6.29	0.71	2.78	1.63	1.71	0.27	0.026477
Solyc05g053010.1.1	Receptor like protein k	3.15	2.97	2.7	1.62	2.61	0.54	0.62	0.76	0.64	0.25	0.026488
Solyc05g042010.1.1	Unknown Protein (AH	22.17	16.44	11.21	4.48	13.57	0	3.01	0	1	0.07	0.026497
Solyc02g092390.2.1	Unknown Protein (AH	16.49	14.68	27.99	15.96	18.78	6.31	6.84	7.18	6.78	0.36	0.026525
Solyc02g083740.1.1	Oligopeptide transport	0.27	0.88	0.64	0.76	0.64	0.07	0	0	0.02	0.04	0.02653
Solyc09g010480.1.1	Thioredoxin-related (A	2.01	7.17	4.16	1.42	3.69	0.41	0	1	0.47	0.13	0.026557
Solyc02g089160.2.1	Cytochrome P450	2.84	2.9	2.51	1.2	2.36	138.69	42.89	155.04	112.21	47.49	0.026589
Solyc03g120520.1.1	Peroxisome assembly f	2.61	6.63	3.08	2.64	3.74	0	0.3	0	0.1	0.03	0.026589
Solyc06g068960.1.1	Calmodulin (AHRD V	30.22	22.73	30.13	45.75	32.21	9.97	9.02	11.83	10.27	0.32	0.026589
Solyc01g090310.2.1	Ethylene responsive tra	6.56	2.92	0.86	4.64	3.75	0	0	0	0	0	0.026835
Solyc06g071790.2.1	Elongation factor Tu (A	120.97	115.87	126.5	108.42	117.94	48.93	57.45	50.67	52.35	0.44	0.026849
Solyc06g083640.2.1	Leucine-rich repeat far	17.28	23.45	20.89	17.29	19.73	7.83	7.81	7.01	7.55	0.38	0.026849
Solyc12g009790.1.1	Synaptotagmin-7 (AHI	17.02	18.26	9.35	15.17	14.95	2.92	5.98	3.12	4.01	0.27	0.026952
Solyc06g076160.2.1	Cytochrome P450	0.13	0.47	0.31	0	0.23	1.48	1.38	0.65	1.17	5.14	0.026965
Solyc07g062670.1.1	Unknown Protein (AH	0	0	0	0	0	313.8	20.54	181.73	172.02	17202.33	0.027093
Solyc09g083150.2.1	NAD(P)H-quinone oxi	39.22	28.89	32.79	24.98	31.47	12.59	15.45	8.25	12.1	0.38	0.027128
Solyc07g041970.2.1	Subtilisin-like protease	32.86	33.01	45.8	61.4	43.27	5.59	13.74	6.78	8.7	0.2	0.027134
Solyc06g062890.2.1	Unknown Protein (AH	65.25	74.67	80.37	49.7	67.5	23.75	26.29	22.06	24.03	0.36	0.027161
Solyc02g085350.2.1	Succinate dehydrogena	227.65	203.73	228.92	233.14	223.36	730.11	579.39	639.65	649.72	2.91	0.027202
Solyc01g090840.2.1	Cys2/His2 zinc-finger	2.85	3.26	1.92	2.59	2.65	0.82	0.33	0	0.38	0.14	0.027249
Solyc01g103460.2.1	3&apos-5&apos exonu	37.92	31.28	47.28	44.48	40.24	14.69	21.1	8.26	14.68	0.36	0.027323
Solyc03g093120.2.1	Xyloglucan endotransg	1.38	1.84	1.08	5.2	2.38	4.27	7.49	26.92	12.89	5.43	0.02741
Solyc08g061490.2.1	MTD1 (AHRD V1 ***	17.72	30.67	12	16.71	19.27	5.3	8.02	2.93	5.42	0.28	0.027432
Solyc01g097380.1.1	Aldo/keto reductase fa	3.36	0.88	1.86	2.24	2.08	0	0.48	0	0.16	0.08	0.0275
Solyc05g006960.2.1	Homeobox-leucine zip	21.12	21.12	22.53	20.46	21.31	4.91	9.2	5.74	6.62	0.31	0.027636
Solyc09g082760.2.1	Aspartic proteinase 2 (	0.53	0.71	4.38	2.25	1.97	0	0	0.26	0.09	0.04	0.027646
Solyc09g072620.2.1	Phytochelatin synthase	15.59	15.59	14.97	20.17	16.58	4.88	7.19	4.44	5.5	0.33	0.027668
Solyc12g088460.1.1	Cytochrome P450	5.56	6.33	2.59	1.28	3.94	48.07	35.63	29.4	37.7	9.57	0.027734
Solyc01g009230.2.1	Xanthine dehydrogena	3.83	4.4	5.43	5.07	4.68	1.25	1.18	0.63	1.02	0.22	0.027754
Solyc07g062720.1.1	RING-finger protein-li	1.45	1.13	1.3	1.2	1.27	0.21	0.35	0.08	0.21	0.17	0.027779
Solyc01g105350.1.1	UDP-glucuronosyltran	5.53	8.64	4.62	2.86	5.41	2.29	0.99	0.8	1.36	0.25	0.027789
Solyc09g005140.1.1	Unknown Protein (AH	1.36	1.14	1.04	1.24	1.2	0	0.46	0	0.15	0.13	0.027816
Solyc02g063200.1.1	Lipase-like protein (AI	0.38	0.67	0.89	0.27	0.55	2.45	2.63	0.75	1.94	3.52	0.027818
Solyc10g085250.1.1	Unknown Protein (AH	12.22	19.57	34.59	10.37	19.19	47.38	51.77	70.33	56.49	2.94	0.027818
Solyc01g107560.2.1	UDP-glucosyltransfera	6.89	4.33	6.54	4.59	5.59	1.31	1.76	1.48	1.52	0.27	0.027849
Solyc01g056580.2.1	L-aspartate oxidase (A	4.26	2.12	7.64	4.58	4.65	1.1	0.97	1.18	1.08	0.23	0.028001
Solyc05g051740.2.1	Unknown Protein (AH	3.38	9.03	4.99	5.98	5.84	0	0.69	0	0.23	0.04	0.028001
Solyc06g074510.2.1	2,3-bisphosphoglycerat	10.74	9.21	7.94	5.86	8.44	2.3	3.9	1.28	2.49	0.3	0.028001
Solyc08g061130.2.1	Transcription factor H	61.41	56.5	55.31	36.97	52.55	485.05	267.1	258.63	336.93	6.41	0.028041
Solyc07g065670.1.1	Unknown Protein (AH	3.37	2.4	0.79	1.9	2.11	19.58	14.81	6.01	13.47	6.37	0.028059
Solyc07g065870.2.1	Regulatory protein Rec	13.58	17.18	15.27	10.8	14.21	5.48	7.09	3.79	5.45	0.38	0.028072
Solyc01g109980.2.1	BEL1-like homeodoma	26.14	28.45	40.48	25.57	30.16	14.11	11.48	12.77	12.79	0.42	0.028144

Solyc09g065760.2.1	Plant-specific domain	60.52	67.06	108.89	25.9	65.59	8.31	13.81	7.96	10.03	0.15	0.028265
Solyc06g036050.2.1	60S ribosomal protein	737.55	755.26	446.27	465.24	601.08	282.21	298.57	181.37	254.05	0.42	0.028331
Solyc01g105090.2.1	Chloroplastic group IIA	13.69	15.93	22.56	10.42	15.65	5.95	6.15	5.75	5.95	0.38	0.028551
Solyc08g075480.2.1	Carotenoid cleavage di	0.57	0.51	0.94	0.48	0.62	0	0	0	0	0.02	0.028566
Solyc03g119400.2.1	Os05g0264200 protein	44.2	54.13	50.01	48.89	49.31	23.22	23.88	21.13	22.74	0.46	0.028578
Solyc04g082840.2.1	Cell division protein ki	1.67	2.04	2.22	3.84	2.44	0.17	0.34	0	0.17	0.07	0.028579
Solyc02g070650.2.1	60S ribosomal protein	541.41	416.25	419.16	446.25	455.77	167.64	228.24	171.94	189.27	0.42	0.028608
Solyc11g072310.1.1	Gibberellin 20-oxidase	0.52	2.32	3.48	1.72	2.01	108.56	52.77	89.26	83.53	41.56	0.028719
Solyc11g044800.1.1	PHP domain-containin	11.53	11.09	11.97	14.57	12.29	4.22	5.01	2.59	3.94	0.32	0.028781
Solyc10g079870.1.1	Cytokinin oxidase/deh	0	0.12	0.31	0	0.11	4.93	0.87	1.98	2.59	24.12	0.02888
Solyc03g062790.2.1	cDNA clone 002-130-0	21.62	25.39	9.2	17.65	18.47	7.2	6.36	6.86	6.81	0.37	0.028881
Solyc09g059430.2.1	RPM1 interacting prot	35.44	26.71	51.58	45.01	39.68	10.24	11.38	3.34	8.32	0.21	0.028882
Solyc07g040680.2.1	Heat stress transcriptio	28.09	29.6	37.06	15.42	27.54	79.13	67.16	30.26	58.85	2.14	0.028972
Solyc09g059510.2.1	Ethylene-responsive tra	55.66	49.85	52.7	52.98	52.8	22.76	15.12	15.69	17.86	0.34	0.029081
Solyc08g068610.2.1	Decarboxylase family	15.65	6.72	1.98	10.87	8.8	0.9	0.23	0.83	0.65	0.07	0.029137
Solyc06g072460.1.1	CHP-rich zinc finger p	6.05	6.81	6.5	10.06	7.36	0.86	1.88	1.77	1.5	0.2	0.029157
Solyc00g160780.2.1	Unknown Protein (AH	4.26	10.73	2.51	2.01	4.88	0	0	0	0	0	0.029271
Solyc01g100640.2.1	Catalase (AHRD V1 **	5	6.48	5.37	6.44	5.82	16.54	8.53	16.71	13.93	2.39	0.029374
Solyc02g080590.2.1	Autophagy-related pro	40.43	49.24	33.89	25.59	37.29	110.47	79.34	60.2	83.34	2.23	0.029375
Solyc10g078430.1.1	Transcription factor Dp	11.89	11.25	12.5	12.09	11.93	57.65	35.13	55.34	49.37	4.14	0.029441
Solyc05g014750.2.1	DNA topoisomerase (A	1.94	2.37	1.43	2.06	1.95	0	0	0	0	0.01	0.029494
Solyc06g074460.2.1	Peroxisomal membran	17.96	21.04	24	22.03	21.26	6.96	9.71	5.82	7.5	0.35	0.029494
Solyc11g007690.1.1	Pyruvate kinase (AHR	21.5	22.12	19.97	19.96	20.89	9.44	9.42	10.27	9.71	0.46	0.029538
Solyc11g065020.1.1	GDP-mannose transpo	5.85	8.68	6.52	1.84	5.72	0.79	1.06	1.61	1.15	0.2	0.029636
Solyc04g018110.1.1	Calmodulin-like protei	71.13	41.56	42.6	49.54	51.21	53.3	104.64	172.19	110.04	2.15	0.029644
Solyc01g073680.2.1	Non-specific lipid-tran	7.44	8.73	6.79	1.44	6.1	0.82	1.38	0.67	0.96	0.16	0.029645
Solyc03g033720.2.1	Genomic DNA chromo	3.5	4.4	3.27	2.56	3.43	0.78	1.08	0.96	0.94	0.27	0.029645
Solyc01g079770.2.1	TLC domain-containin	6.67	8.8	3.25	4.55	5.82	15.97	14.98	6.16	12.37	2.13	0.029774
Solyc07g054760.1.1	Wound induced protei	29.06	51.72	19.46	10	27.56	41.88	124.79	181.64	116.1	4.21	0.029799
Solyc01g005250.2.1	Aspartate-semialdehyd	60.1	48.57	48.08	55.21	52.99	19.95	20.97	30	23.64	0.45	0.029864
Solyc05g006130.2.1	ATP-dependent RNA h	6.01	7.55	7.64	6.67	6.97	2.28	2.78	1.05	2.04	0.29	0.029893
Solyc03g123620.2.1	Pectinesterase (AHRD	11.27	7.96	5.89	19.55	11.17	0.38	0.66	4.03	1.69	0.15	0.029893
Solyc11g011620.1.1	Fatty acid-binding prot	11.05	8.19	15.57	15.62	12.61	3.72	3	3.35	3.36	0.27	0.02992
Solyc01g099920.2.1	Photoassimilate-respon	105.94	59.93	44.89	46.08	64.21	261.18	183.13	213.02	219.11	3.41	0.029937
Solyc01g005130.2.1	Zinc finger protein 7 (A	18.14	14.27	19.85	29	20.31	5.95	9.85	11.22	9.01	0.44	0.029966
Solyc01g097350.2.1	Calcium-binding EF ha	89.91	111.52	103.7	134.28	109.85	50.54	40.65	55.7	48.96	0.45	0.029983
Solyc01g099830.2.1	60S ribosomal protein	1366.54	1168.69	1271.87	1288.65	1273.94	571.9	561.47	506.71	546.69	0.43	0.030054
Solyc02g069250.2.1	Cinnamyl alcohol dehy	1.64	2.76	1.29	1.55	1.81	0.3	0	0	0.1	0.06	0.030247
Solyc05g009670.2.1	Choline transporter-lik	7.43	8.59	6	5.72	6.93	1.69	2.97	1.29	1.98	0.29	0.030276
Solyc02g084440.2.1	Fructose-bisphosphate	1099.35	776.73	1092.02	893.74	965.46	177.66	374.52	345.41	299.2	0.31	0.030369
Solyc06g076680.2.1	Acyl-coenzyme A thio	8.3	9.6	3.92	8.8	7.66	1.01	1.01	0.41	0.81	0.11	0.030482
Solyc01g111640.2.1	Skp1 (AHRD V1 ***-	11.2	10.71	16.64	12.91	12.86	2.35	4.4	3.29	3.35	0.26	0.030599
Solyc01g106780.2.1	Pyruvate kinase (AHR	50.05	47.3	27.02	18.47	35.71	8.98	12.13	7.39	9.5	0.27	0.03064
Solyc12g010020.1.1	Leucyl aminopeptidase	42.98	26.32	78.26	11.01	39.64	0.39	6.44	0.83	2.55	0.06	0.030658
Solyc01g079710.2.1	Uncharacterized conse	53.18	51.56	59.94	34.9	49.9	18.75	24.12	17.05	19.97	0.4	0.030658
Solyc09g011070.1.1	Receptor like protein k	1.74	1.89	1.26	1.64	1.63	0.23	0.08	0.19	0.17	0.1	0.030852
Solyc08g067250.2.1	DNA cross-link repair	2.82	2.97	4.04	1.94	2.94	0.69	1.19	0.08	0.65	0.22	0.030874
Solyc02g082720.2.1	Serine protease Do (AI	0.94	2.09	1.29	1.11	1.36	3.66	3.06	3.72	3.48	2.56	0.030888
Solyc07g056240.2.1	Unknown Protein (AH	7.96	11.81	9.84	5.9	8.88	1.84	3.4	1.51	2.25	0.25	0.030944
Solyc02g092410.2.1	Adenylyl-sulfate kinase	7.07	5.53	6.57	7.27	6.61	1.38	2.62	1.28	1.76	0.27	0.030987
Solyc08g082430.2.1	Nucleoside diphosphat	121.18	90.4	113	122.6	111.8	18.73	61.63	23.01	34.46	0.31	0.030997
Solyc01g095560.2.1	Unknown Protein (AH	1.78	0.53	2.1	0.84	1.31	3.84	8.72	3.54	5.37	4.09	0.031037
Solyc02g068580.1.1	Histone acetyltransfera	14.91	11.02	11.28	8.95	11.54	5.23	4.58	4.89	4.9	0.42	0.031037
Solyc04g064630.2.1	RAG1-activating prote	3.68	4.91	2.48	0.74	2.95	0.42	0.21	0	0.21	0.07	0.031037
Solyc08g082070.2.1	TOX high mobility gro	0.92	0.94	0.47	0.19	0.63	3.41	3.33	1.96	2.9	4.6	0.031037
Solyc12g019210.1.1	Unknown Protein (AH	106.57	173.88	116.75	150.74	136.99	41	49.62	78.07	56.23	0.41	0.031089
Solyc09g010210.2.1	Endoglucanase 1 (AHF	2.42	2.76	1.43	1.71	2.08	341.22	95.67	695.31	377.4	181.44	0.031188
Solyc03g093590.1.1	Unknown Protein (AH	20.44	13.23	25.21	17.09	18.99	2.25	3.03	2.77	2.68	0.14	0.03121
Solyc03g115840.2.1	DnaJ homolog subfam	8.33	9.61	13.17	13.98	11.27	2.81	5.29	4.27	4.12	0.37	0.031249
Solyc07g053980.2.1	Glucan synthase like 1	1.96	2.66	3.97	5.24	3.46	0.52	0.65	0.64	0.6	0.17	0.031283
Solyc01g095070.2.1	Heavy metal-associate	283.14	278.68	253.61	121	234.11	98.95	101.86	62.35	87.72	0.37	0.031314
Solyc07g045190.1.1	RING-H2 finger protei	95.17	111.65	119.3	84.14	102.56	41.07	38.34	33.28	37.56	0.37	0.031314
Solyc01g104590.2.1	Ribosomal protein L3 (	769.32	638.63	807.86	744.13	739.99	274.15	319.79	314.48	302.81	0.41	0.031351
Solyc01g090270.2.1	Bzip-like transcription	30.69	32.2	53.34	58.84	43.77	9.36	13.49	9.93	10.93	0.25	0.031411
Solyc06g048970.2.1	At2g40630/T2P4.2 (AI	11.37	10.95	14.52	12.43	12.32	3.69	4.58	4.07	4.11	0.33	0.03147
Solyc07g007220.2.1	Serine/threonine phosp	10.82	10.86	5.68	7.53	8.72	3.05	3.64	1.19	2.63	0.3	0.031505
Solyc12g013500.1.1	Unknown Protein (AH	27.83	25.69	23.11	27.7	26.08	26.65	54.6	102.32	61.19	2.35	0.031563
Solyc03g113550.2.1	Transcription factor bH	20.71	20.14	19.32	4.77	16.23	1.95	6.67	2.39	3.67	0.23	0.031589
Solyc01g095610.2.1	Leaf senescence protei	3.64	3.71	5.88	1.71	3.74	0.54	1.21	0.27	0.67	0.18	0.031698
Solyc12g010840.1.1	Ketol-acid reductoison	101.41	99.08	90.45	95.05	96.5	28.63	46.22	41.04	38.63	0.4	0.031814
Solyc04g025650.2.1	Monooxygenase FAD-	2	1.64	2.17	2.36	2.04	0	0.54	0.33	0.29	0.14	0.031873
Solyc10g080190.1.1	Atozi1 (Fragment) (AH	305.01	265.36	247.65	341.75	289.94	575.9	732.36	445.62	584.63	2.02	0.031909
Solyc02g005110.2.1	Unknown Protein (AH	32.36	37.67	41.12	36.97	37.03	7.04	11.16	3.71	7.3	0.2	0.031927
Solyc03g044000.1.1	Ribosomal protein L26	1065.41	934.63	1035.11	996	1007.79	438.72	440.3	423.18	434.07	0.43	0.031931
Solyc07g047750.2.1	Unknown Protein (AH	101.16	104.38	84.75	70.51	90.2	216.75	169.64	194.99	193.79	2.15	0.031936
Solyc03g034020.2.1	U-box domain-contain	7.49	6	5.19	11.78	7.62	1.36	1.3	1.67	1.44	0.19	0.031949
Solyc03g033260.2.1	Maltose excess protein	17.84	22.53	17.62	9.39	16.85	4.69	8.79	4.12	5.87	0.35	0.031976
Solyc01g056810.2.1	Syntaxin (AHRD V1 *	17.4	15.72	16.24	15.8	16.29	6.92	5.71	3.61	5.41	0.33	0.031977
Solyc04g024960.1.1	Unknown Protein (AH	14.38	10.54	32.93	16.75	18.65	3.42	2.07	0.84	2.11	0.11	0.031984
Solyc07g006380.2.1	Defensin-like protein (	11.2	89.73	6.61	22.89	32.61	0	0	0	0	0	0.031984
Solyc03g098090.1.1	UPF0497 membrane p	0	0	0	0	0	0.59	1.79	0	0.79	79.33	0.032062
Solyc02g069260.2.1	ARGONAUTE 1 (AHI	20.56	23.98	31.27	37.58	28.35	4.24	7.16	11.43	7.61	0.27	0.03212

Solyc09g008480.2.1	Phosphatidylinositol-4-	21.23	28.64	29.83	19.49	24.8	11.98	9.25	9.89	10.37	0.42	0.032138
Solyc11g005090.1.1	Cyclin A1 (AHRD V1	0.94	0.96	0.48	0.38	0.69	0	0	0.13	0.04	0.06	0.03221
Solyc11g010560.1.1	Kinesin-like protein (A	15.81	19.9	23.49	19.1	19.58	7.48	9.17	6.65	7.77	0.4	0.032212
Solyc12g009310.1.1	ATP binding / serine-th	0.88	1.41	0.83	0.5	0.91	0	0	0	0	0.01	0.032212
Solyc03g093700.1.1	Histone-lysine N-meth	1.81	1.19	1.67	1.44	1.53	0.13	0.32	0	0.15	0.1	0.032296
Solyc08g082280.2.1	Long-chain-fatty-acid-(	26.63	27.98	34.49	22.94	28.01	7.28	12.15	9.34	9.59	0.34	0.032329
Solyc01g110770.2.1	Auxin-induced SAUR-	6.38	7.95	2.01	2.41	4.69	0.69	0.69	0	0.46	0.1	0.032365
Solyc02g070000.2.1	Receptor like kinase, R	3.28	6.53	5.39	5.64	5.21	1.72	2.05	1.34	1.7	0.33	0.032409
Solyc03g007540.2.1	Unknown Protein (AH	8.85	10.74	9.97	9.1	9.67	22.75	30.16	17.17	23.36	2.42	0.032575
Solyc01g086710.2.1	DNA-directed RNA po	12.87	13.27	13.48	10.89	12.63	4.28	5.24	5.31	4.94	0.39	0.032575
Solyc10g075100.1.1	Non-specific lipid-tran	1306.23	1495.93	451.43	613.45	966.76	3013.92	2703.77	1543.1	2420.26	2.5	0.032576
Solyc03g007530.2.1	Multidrug resistance pr	14.07	19.68	27.52	13.6	18.72	5.16	7.29	4.06	5.5	0.29	0.032653
Solyc10g007830.1.1	LRR receptor-like seri	0.6	1.46	0.35	0.63	0.76	0	0	0	0	0.01	0.032692
Solyc08g007580.2.1	Uncharacterized conse	2.38	4.54	3.61	2.41	3.23	17.31	7.2	10.46	11.66	3.6	0.032709
Solyc06g054410.2.1	Iaa-amino acid hydrola	12.28	10.39	11.45	8.58	10.68	2.94	5.19	1.51	3.21	0.3	0.032809
Solyc07g054780.1.1	Wound induced protei	58.12	94.36	56.52	74.43	70.86	92.64	96.64	268.94	152.74	2.16	0.032812
Solyc01g108390.2.1	Proteinase inhibitor (A	22.3	29.77	2.19	2.63	14.22	0	0	0	0	0	0.032822
Solyc06g064460.2.1	Ribosomal protein L7a	502.81	453.62	460.98	454.69	468.03	189.94	232.05	173.44	198.48	0.42	0.033096
Solyc01g103240.2.1	Protein serine/threonin	0.73	0.82	0.43	2.07	1.01	0	0	0	0	0.01	0.033098
Solyc01g095930.2.1	O-acyltransferase WSI	88.35	88.89	74.57	22.19	68.5	946.33	414.27	636.05	665.55	9.72	0.033152
Solyc07g064610.2.1	Calcium-dependent pro	53.84	51.63	46.67	69.18	55.33	14.5	24.41	27.85	22.25	0.4	0.033178
Solyc02g087790.1.1	Aldose-1-epimerase-lik	1.86	0.9	0.8	0.95	1.13	0	0.14	0.17	0.1	0.09	0.033183
Solyc06g053820.2.1	30S ribosomal protein	98.52	90.77	97.8	92.7	94.95	39.62	37.49	35.76	37.62	0.4	0.033242
Solyc04g008690.2.1	cDNA clone J013057D	26.17	29.45	27.82	21.11	26.14	10.14	12.34	6.87	9.78	0.37	0.033272
Solyc05g051280.2.1	Calmodulin-binding ho	18.54	18.04	25.94	26.23	22.19	6.97	11.01	7.99	8.66	0.39	0.033272
Solyc08g076480.2.1	Plastid lipid-associated	185.87	167.8	201.24	165.26	180.04	57.93	101.28	71.75	76.99	0.43	0.033272
Solyc07g062950.2.1	BHLH transcription fac	9.56	12.83	13.5	8.87	11.19	3.67	3.06	2.33	3.02	0.27	0.033311
Solyc00g187050.2.1	Leucyl aminopeptidase	35.25	22.38	69.88	10.27	34.45	0.21	5.28	0.64	2.04	0.06	0.033434
Solyc01g104490.2.1	Separin (AHRD V1 *--	0.62	0.99	0.91	0.75	0.82	0.13	0.2	0.12	0.15	0.18	0.033501
Solyc09g010670.2.1	DP-2 transcription fact	6.73	12.28	11.91	7.14	9.51	2.99	1.65	2.34	2.33	0.24	0.033606
Solyc06g072470.2.1	50S ribosomal protein	108.35	91.45	102.83	130.08	108.18	35.53	58.97	42.46	45.65	0.42	0.033638
Solyc01g095280.2.1	Protein ABIL1 (AHRD	40.1	33.58	34.85	37.2	36.43	11.84	16.87	11.13	13.28	0.36	0.033737
Solyc10g080870.2.1	Cytochrome P450	0	0	0	0	0	2.5	1.05	1.41	1.65	165.33	0.033737
Solyc05g005710.2.1	Spermidine synthase (A	554.7	509.94	469.71	436.38	492.68	219.41	243.87	186.52	216.6	0.44	0.033749
Solyc04g080230.2.1	BAC19.4 (AHRD V1 *	154.15	166.69	116.18	170.24	151.81	51.48	86.54	40.89	59.64	0.39	0.033754
Solyc04g071420.2.1	Thioredoxin family pro	53.23	47.88	30.06	36.85	42	12.58	24.23	9.27	15.36	0.37	0.033755
Solyc06g082480.2.1	Os12g0114200 protein	3.08	2.6	2.71	2.13	2.63	0.96	0.84	1.88	1.23	0.47	0.033794
Solyc10g061860.1.1	Calcium-dependent pro	9.83	7.5	14.9	11.91	11.04	1.89	2.67	0.46	1.67	0.15	0.033794
Solyc07g006480.2.1	LRR receptor-like seri	1.82	0.99	1.07	3.85	1.93	0	0.08	0.1	0.06	0.03	0.033953
Solyc07g042460.1.1	Respiratory burst oxida	0.97	0.87	1.15	0.63	0.9	6.22	2.99	2.15	3.79	4.18	0.033955
Solyc05g053240.2.1	Squamosa promoter bi	26.83	33.04	36.05	43.4	34.83	12.55	16.41	13.54	14.17	0.41	0.033972
Solyc08g066610.2.1	EPIDERMAL PATTE	9.14	10.46	1.54	5.54	6.67	0.53	1.6	0	0.71	0.11	0.033972
Solyc10g081570.1.1	KED (AHRD V1 *-*-	53.17	77.55	56.92	145.31	83.24	112.03	143.09	354.13	203.08	2.44	0.033972
Solyc01g005020.2.1	Sodium/hydrogen exch	23.14	27.32	29.46	23.9	25.95	6.06	12.23	8.07	8.79	0.34	0.03405
Solyc12g087840.1.1	RING finger protein (A	0	0	0	0	0	3.54	1.53	1.66	2.24	224.33	0.034106
Solyc09g082570.2.1	Neurogenic locus notcl	9.51	11.74	5.07	4.77	7.77	15.37	14.5	28.32	19.4	2.5	0.034219
Solyc09g075570.1.1	Binding protein (AHRI	36.91	50.5	28.85	30.02	36.57	8.95	20.68	5.95	11.86	0.32	0.034318
Solyc07g054920.1.1	2-oxoglutarate-iron(II)	0.55	1.94	3.43	0.51	1.61	0	0	0	0	0.01	0.034352
Solyc05g054830.2.1	Ankyrin-repeat protein	68.68	51.99	61.6	91.56	68.46	17.58	27.02	35.15	26.58	0.39	0.034393
Solyc09g074970.2.1	Thiamin pyrophosphok	5.35	7.93	5.11	4.32	5.68	1.03	2.08	0.51	1.21	0.21	0.034393
Solyc03g112360.1.1	60S ribosomal protein	591.08	380.66	851.45	816.04	659.81	130.56	216.83	199.08	182.16	0.28	0.034496
Solyc10g012120.1.1	Non-specific lipid-tran	1.75	3.64	6.2	0.83	3.1	0	0	0	0	0	0.034496
Solyc06g072760.2.1	Syntaxin (AHRD V1 *	0	0	1.1	0	0.28	4.13	3.03	2.54	3.23	11.76	0.034571
Solyc03g019750.2.1	Alpha-1 4-glucan-prote	0.38	0.84	2	0.8	1	0	0	0	0	0.01	0.034679
Solyc02g071870.2.1	Receptor like kinase, R	2.84	3.5	3.76	4.31	3.6	0.93	1.05	1.28	1.09	0.3	0.034709
Solyc09g090670.2.1	Oxidation resistance 1-	7.44	6.97	4.85	9.41	7.17	1.42	2.23	1.55	1.73	0.24	0.034713
Solyc07g041400.2.1	Unknown Protein (AH	2.13	1.26	2.09	2.01	1.87	0	0	0.35	0.12	0.06	0.034726
Solyc09g064500.2.1	Photosystem II reactior	83.1	98.29	50.31	46.4	69.52	31.8	25.25	10.85	22.63	0.33	0.034778
Solyc04g074450.1.1	Phi-1 protein (Fragmer	14.66	12.86	9.64	23.4	15.14	0.68	2.22	14.34	5.75	0.38	0.034778
Solyc09g018430.2.1	Gamma-interferon-ind	3.18	3.14	0.83	2	2.29	0.29	0	0	0.1	0.04	0.034805
Solyc06g008830.1.1	Calcium-dependent pro	23.7	17.1	21.16	9.97	17.98	3.62	9.39	0.64	4.55	0.25	0.034859
Solyc01g112230.2.1	PGPS/NH21 (AHRD V	1.59	10.61	16.88	6.75	8.96	0.64	0	0	0.21	0.02	0.034966
Solyc09g008370.1.1	Nodulin-like protein (F	1.82	3.24	5.05	3.94	3.51	11.59	5.85	10.74	9.39	2.67	0.034995
Solyc07g049550.2.1	1-aminocyclopropane-	313	160.03	450.79	187.22	277.76	1876.04	882.68	1688.08	1482.27	5.34	0.035067
Solyc03g082960.2.1	Serine/threonine phosp	53.81	58.12	46.58	31.11	47.41	21	22.81	16.87	20.23	0.43	0.035126
Solyc06g084300.1.1	Unknown Protein (AH	5.15	10.09	6.08	7.29	7.15	26.65	21	18.42	22.02	3.08	0.035191
Solyc07g043490.1.1	UDP-glucosyltransfera	16.36	14.19	18.65	58.8	27	1.1	1.11	1.08	1.1	0.04	0.035264
Solyc07g043420.2.1	2-oxoglutarate-depend	207.91	260.7	223.51	797.4	372.38	16.16	28.37	7.45	17.33	0.05	0.035299
Solyc00g231780.1.1	NAD(P)H-quinone oxi	0.61	0.54	0	0	0.29	4.44	3.48	4.24	4.05	14.1	0.035311
Solyc07g009410.2.1	Kelch-like protein 7 (A	2.21	1.97	2.09	0.42	1.67	0.12	0.24	0.15	0.17	0.1	0.035342
Solyc05g055840.2.1	Glucosyltransferase (A	11.88	12.19	12.86	11.07	12	2.82	7.06	1.11	3.66	0.31	0.035342
Solyc10g050080.1.1	Calcium-dependent pro	26.3	18.07	19.05	10.44	18.46	2.24	10.53	1.37	4.71	0.26	0.035342
Solyc03g007050.2.1	Receptor like kinase, R	0.61	0.72	0.56	0.48	0.59	0	0	0	0	0.02	0.035346
Solyc02g069500.1.1	Pentatricopeptide repea	1	1.79	1.55	1.86	1.55	0.06	0.57	0.31	0.31	0.2	0.035367
Solyc06g072340.2.1	Kinase family protein (	2.39	2.81	2.05	2.62	2.47	0.84	0.98	0.59	0.8	0.33	0.035367
Solyc07g008080.2.1	WD repeat-containing	10.83	10.45	13.24	16.61	12.78	4.74	5.37	5.25	5.12	0.4	0.035367
Solyc09g008790.2.1	Serine/threonine protei	17.35	20.21	35.62	16.81	22.5	3.27	8.33	4.37	5.32	0.24	0.035389
Solyc01g081370.2.1	Aspartic proteinase ne	0.9	1.6	0.53	0.85	0.97	0	0.12	0	0.04	0.04	0.035408
Solyc05g055230.1.1	40S ribosomal protein	278.44	226.76	270	260	258.8	96.67	131.24	96.64	108.18	0.42	0.035458
Solyc08g076080.2.1	Xyloglucan endotransg	0	0	0	0.25	0.06	0.86	0.86	0.88	0.87	13.87	0.035476
Solyc07g043160.1.1	UDP-glucosyltransfera	43.98	55.12	44.83	53.32	49.31	29.49	17.68	22.72	23.3	0.47	0.03552
Solyc02g077090.2.1	Chloroplast unusual po	3.68	3.64	4.34	3.76	3.85	1.4	1.33	1.12	1.28	0.33	0.035587

Solyc08g005210.2.1	Dolichol phosphate-ma	39.91	76.84	36.51	38.02	47.82	119.76	130.74	57.4	102.63	2.15	0.035627
Solyc02g020890.2.1	Transmembrane protei	2.34	3.61	3.5	2.87	3.08	5.67	6.48	10.84	7.66	2.49	0.035774
Solyc03g115860.2.1	Endoplasmic reticulum	40.86	59.01	29.24	32.21	40.33	128.51	79.68	80.44	96.21	2.39	0.035832
Solyc01g058170.2.1	Agenet domain-contai	15.99	13.73	17.18	10.5	14.35	3	4.65	2.55	3.4	0.24	0.035873
Solyc02g086930.2.1	Homeobox-leucine zip	237.59	211.66	243.84	210.45	225.88	82	97.37	148.37	109.25	0.48	0.035904
Solyc01g094760.2.1	Origin recognition com	4.64	4.35	5.62	6.05	5.17	1.58	0.9	0.49	0.99	0.19	0.035909
Solyc09g007380.2.1	Hydrolase alpha/beta f	0.38	0.78	0.74	0.71	0.65	0	0	0	0	0.02	0.035933
Solyc09g090080.1.1	Inorganic phosphate tr	0	0.11	0	0	0.03	0.2	2.61	0.61	1.14	41.45	0.035944
Solyc11g066590.1.1	Lysosomal Pro-X carb	0.65	1.51	0.92	0.92	1	0	0	0	0	0.01	0.035972
Solyc01g099200.2.1	Lipoxygenase (AHRD	1.72	1.46	1.39	1.44	1.5	18.42	8.56	46.27	24.42	16.25	0.035984
Solyc04g056330.1.1	Protein containing AIC	7.29	6.14	5.43	9.77	7.16	1.55	1.88	1.52	1.65	0.23	0.036132
Solyc09g075210.2.1	Late embryogenesis ab	50166.3	30622.91	27260.25	29637.3	34421.69	8128.03	13667.59	6508.8	9434.81	0.27	0.036132
Solyc01g068470.2.1	CM0216.540.nc protei	10.17	9.6	6	5.67	7.86	2.99	3.52	2.29	2.93	0.37	0.036263
Solyc09g014890.2.1	Mitochondrial interme	6.18	5.84	5.16	3.9	5.27	1.15	2.48	1.32	1.65	0.31	0.036369
Solyc01g095620.2.1	UDP-glucosyltransfera	70.67	80.99	121.89	71.21	86.19	4.47	52.97	3.02	20.15	0.23	0.036376
Solyc10g081810.1.1	Phosphatidylglycerol/p	2.54	2.63	0.5	2.99	2.17	0	0	0	0	0	0.03642
Solyc05g006390.1.1	Unknown Protein (AH	6.21	7.6	3.66	7.68	6.29	9.41	17.08	32.36	19.62	3.12	0.036427
Solyc04g026110.2.1	Cc-nbs, resistance prot	3.28	2.78	1.76	1.9	2.43	0.24	0.85	0.89	0.66	0.27	0.036436
Solyc05g010530.2.1	Serine/threonine protei	4.68	6.01	4.29	3.18	4.54	0.98	1.27	0.86	1.04	0.23	0.036441
Solyc03g097430.1.1	Unknown Protein (AH	2.14	1.63	1.44	1.73	1.73	5.68	4.73	3.03	4.48	2.58	0.036471
Solyc02g014310.2.1	Polyadenylate-binding	165.55	141.3	228.32	178.66	178.46	66.54	83.7	97.19	82.48	0.46	0.036549
Solyc02g077690.2.1	Unknown Protein (AH	14.14	10.78	10.59	13.97	12.37	3.63	5.49	0.45	3.19	0.26	0.036549
Solyc03g119040.2.1	Guanine nucleotide-bi	181.98	130.71	230.88	226.3	192.47	54.77	75.13	76.1	68.67	0.36	0.036549
Solyc09g008520.2.1	Chromodomain helicas	13.46	11.92	11.54	14.03	12.74	3.72	6.12	3.66	4.5	0.35	0.036552
Solyc10g006770.2.1	FACT complex subuni	6.76	7.63	13.45	6.67	8.63	11.05	14.8	32.08	19.31	2.24	0.036589
Solyc01g087840.2.1	Subtilisin-like proteas	0.43	0.46	1.12	1.22	0.81	0	0.14	0	0.05	0.06	0.036638
Solyc11g010390.1.1	Unknown Protein (AH	33.93	23.07	89.51	136.64	70.79	2.28	6.14	14.97	7.8	0.11	0.03665
Solyc02g093550.2.1	Methyltransferase type	21.61	20.25	11.93	9.75	15.88	5.94	6.37	5.48	5.93	0.37	0.036757
Solyc04g081160.2.1	Cell growth defect fact	37.7	50.33	28.49	32.49	37.25	10.47	16.32	4.09	10.29	0.28	0.036809
Solyc12g017960.1.1	Unknown Protein (AH	6.75	11.34	1.77	4.24	6.03	0	0	0	0	0	0.036809
Solyc01g102730.2.1	Cinnamoyl-CoA reduc	12.51	7.54	19.28	16.27	13.9	3.59	5.1	7.41	5.37	0.39	0.036871
Solyc09g009340.1.1	DNA double-strand br	5.05	6.66	5.95	3.31	5.24	2.04	2.28	1.16	1.83	0.35	0.036891
Solyc09g018750.2.1	Inosine-5&apos-mono	357.18	359.1	294.03	334.62	336.23	123.56	204.44	89.96	139.32	0.41	0.036926
Solyc06g084280.1.1	Unknown Protein (AH	0	0.37	0	0	0.09	5.66	3.36	8.19	5.74	62.02	0.036934
Solyc01g090880.2.1	Holliday junction reso	72.45	51.64	48.64	54.99	56.93	17.37	30.48	20.17	22.67	0.4	0.036949
Solyc09g011830.2.1	Transcription antiterm	48.01	45.69	53.65	50.8	49.54	22.39	23.54	26.15	24.03	0.49	0.037032
Solyc12g042430.1.1	Inositol hexakisphosph	15.24	15.64	15.02	19.02	16.23	4.34	8.76	4.98	6.03	0.37	0.037148
Solyc11g062130.1.1	Mitochondrial ADP/AT	1098.67	1055.33	1301.62	1251.45	1176.77	457.79	499.38	590.36	515.84	0.44	0.037213
Solyc03g031980.2.1	Receptor-like kinase (	0.78	0.78	3.09	1.1	1.44	0	0.08	0.1	0.06	0.04	0.037272
Solyc06g008620.1.1	Protein tolB (AHRD V	1	1.07	1.88	1.55	1.38	0.24	0.24	0.3	0.26	0.19	0.037377
Solyc01g067460.1.1	Glutaredoxin family pr	16.65	32.49	6.8	2.72	14.66	1.03	0	0	0.34	0.02	0.037427
Solyc02g088580.1.1	Genomic DNA chrom	2.34	2.08	3.31	3.7	2.86	2.64	5.94	9.37	5.98	2.09	0.037455
Solyc09g090530.2.1	At1g04030 (Fragment)	0	0.59	0.26	0.31	0.29	5.72	3.97	1.54	3.74	12.91	0.037563
Solyc10g050860.1.1	Polyadenylate-binding	149.78	107.71	136.98	127.9	130.59	43.98	64.66	42.74	50.46	0.39	0.037563
Solyc11g068960.1.1	Uncharacterized basic	24.66	24.66	68.8	21.85	34.99	2.98	5.43	1.69	3.37	0.1	0.037563
Solyc01g108730.1.1	Hydrolase alpha/beta f	0	0	0.69	0	0.17	6.66	2.88	3.51	4.35	25.22	0.037602
Solyc08g075090.2.1	60S ribosomal protein	111.73	108.96	110.79	101.05	108.13	40.43	56.66	42.24	46.44	0.43	0.03773
Solyc04g014900.2.1	Receptor like kinase, R	3.34	2.76	3.52	10.12	4.93	0	0.1	0.12	0.07	0.01	0.037771
Solyc05g054530.1.1	Genomic DNA chrom	2.4	3.31	1.8	1.54	2.26	0.35	0	0.43	0.26	0.11	0.037816
Solyc05g054860.1.1	Pto-like, Serine/threon	0.89	1.81	2.2	2.41	1.83	3.09	5.83	33.39	14.1	7.72	0.037818
Solyc06g060800.2.1	Gibberellin 2-beta-dio	2.55	3.78	3	2.1	2.86	5.14	6.91	6.32	6.12	2.14	0.037833
Solyc09g065880.2.1	Leucine Rich Repeat f	9.46	9.9	7.22	13.53	10.03	1.35	3.08	0.77	1.73	0.17	0.037833
Solyc04g016400.2.1	Calpain-2 catalytic sub	20.36	20.54	23.06	18.05	20.5	7.68	7.52	5.39	6.86	0.33	0.037866
Solyc06g075190.1.1	Unknown Protein (AH	143.62	169.41	137.83	130.25	145.28	65.51	74.48	53.2	64.4	0.44	0.038244
Solyc02g067140.1.1	Mutator-like transposa	0.16	0	0	0	0.04	1.03	0.78	0.95	0.92	23	0.038278
Solyc04g051470.1.1	Unknown Protein (AH	14.02	28.62	10.7	9.33	15.67	5.33	1.34	0	2.22	0.14	0.038344
Solyc05g012950.1.1	Sulfotransferase family	0.56	0.99	0	0.79	0.58	3.15	3.18	4.42	3.58	6.13	0.038347
Solyc07g054170.2.1	Expansin B1 (AHRD V	2.22	1.31	0.87	3.48	1.97	0.2	0.2	0	0.13	0.07	0.038426
Solyc05g010610.2.1	Transcription factor (A	6.24	5.21	5.71	5.94	5.78	1.85	1.97	1.14	1.65	0.29	0.038484
Solyc07g025510.2.1	Senescence-associated	19.79	23.92	25.66	34.91	26.07	8.29	10.35	7.52	8.72	0.33	0.038505
Solyc10g075070.1.1	Non-specific lipid-tran	3479.26	3283.38	1532.27	1717.01	2502.98	883.83	822.26	286.98	664.36	0.27	0.038505
Solyc10g085390.1.1	D-alanine--D-alanine l	7.73	7.58	6.21	7.29	7.2	0.9	2.83	1.56	1.76	0.24	0.038505
Solyc02g021360.2.1	WD-40 repeat protein	10.16	9.92	9.06	12.03	10.29	4.8	4.58	3.93	4.44	0.43	0.038666
Solyc07g045080.2.1	Alcohol dehydrogenase	8.08	6.61	5.41	11.12	7.8	77.65	39.16	32.52	49.78	6.38	0.038709
Solyc04g051870.1.1	Kelch repeat-containin	16.58	16.56	21.09	20.07	18.58	7.74	7.81	5.49	7.01	0.38	0.038713
Solyc04g008310.2.1	Glucosyltransferase (A	46.92	51.23	61.8	30.11	47.52	17.43	25.11	11.82	18.12	0.38	0.038918
Solyc06g069580.2.1	Heparanase-like protei	6.35	6.52	4.9	3.97	5.43	2.37	1.79	0.61	1.59	0.29	0.038964
Solyc03g113870.1.1	U-box domain-contain	3.49	2.33	3.68	4.23	3.43	0.81	0.81	0.62	0.75	0.22	0.039005
Solyc12g096940.1.1	CCT motif family prot	1.08	1.45	0	1.02	0.89	0	0	0	0	0.01	0.039072
Solyc08g006250.1.1	Copper transporter (A	36.37	44.41	30.93	27.52	34.81	12.98	17.92	7.56	12.82	0.37	0.039102
Solyc01g067630.2.1	MRNA complete cds c	32.32	30.84	27.12	31.58	30.46	8.34	16.29	6.95	10.53	0.35	0.039133
Solyc01g095550.2.1	Receptor-interacting s	0.84	0.75	0.44	1.33	0.84	0.15	0.08	0.28	0.17	0.2	0.03916
Solyc01g059930.2.1	Universal stress protei	221.65	231.99	162.78	179.23	198.91	82.55	95.71	51.11	76.46	0.38	0.039231
Solyc04g010210.1.1	ABC transporter G fam	0.09	0	0	0.13	0.06	1.04	0.75	0.92	0.9	16.42	0.039303
Solyc07g062520.2.1	Cytochrome P450	29.43	38.39	27.54	24.05	29.85	13.12	13.96	8.69	11.92	0.4	0.039318
Solyc03g123700.2.1	NUMOD3 motif famil	7.26	7.51	7.05	8.76	7.64	3.19	2.35	2.65	2.73	0.36	0.039396
Solyc05g051480.2.1	Unknown Protein (AH	6.87	12.23	4.86	11.66	8.91	1.11	0	0	0.37	0.04	0.039506
Solyc12g044450.1.1	Os06g0207500 protein	3.42	3.38	0.9	3.5	2.8	0.61	0.31	0.19	0.37	0.13	0.039512
Solyc02g077420.2.1	Lipase-like (AHRD V1	1.47	0.44	1.93	0.23	1.02	0	0	0	0	0.01	0.039527
Solyc06g060360.2.1	Universal stress protei	0.87	2.83	0.34	1.64	1.42	0	0	0.29	0.1	0.07	0.039555
Solyc10g054280.1.1	Haloacid dehalogenase	15.76	18.07	14.5	14.36	15.67	5.61	7.4	5.04	6.02	0.38	0.039555
Solyc06g062440.2.1	Cc-nbs-lrr, resistance p	10.96	15.83	13.27	11.43	12.87	6.22	5.09	4.87	5.39	0.42	0.039637

Solyc06g068260.1.1	1-aminocyclopropane-	0	0.5	0.66	1.58	0.69	4.52	5.01	2.77	4.1	5.99	0.039642
Solyc04g072100.1.1	Unknown Protein (AH	1.06	0.94	4.36	3.73	2.52	34.11	17.2	11	20.77	8.23	0.039722
Solyc06g076110.1.1	Fasciclin-like arabinog	9.1	11.47	13.87	15.55	12.5	2.76	2.16	4.14	3.02	0.24	0.039727
Solyc09g092290.1.1	Cc-nbs-lrr, resistance p	0.78	0.83	0.73	0.99	0.83	0.13	0	0.23	0.12	0.14	0.039841
Solyc03g005900.2.1	GDSL esterase/lipase A	0.72	0.48	1.06	1.53	0.95	0	0	0	0	0.01	0.040073
Solyc06g073680.2.1	Notchless protein hom	3.57	3.64	2.47	2.37	3.01	0.25	1.37	1.25	0.96	0.32	0.040077
Solyc06g062330.1.1	UDP-glucosyltransfera	3.27	3.49	6.68	6.16	4.9	1.23	0.18	1.73	1.05	0.21	0.040094
Solyc02g079570.2.1	Serine/threonine-prote	1.17	0.49	0.09	0.44	0.55	0	0	0.23	0.08	0.14	0.04022
Solyc02g079430.2.1	CONSTANS-like zinc	3.19	1.89	1.26	0.5	1.71	0	0	0	0	0.01	0.040261
Solyc07g062130.2.1	Dtdp-glucose 4 6-dehy	55.81	52.45	67.57	114.74	72.64	23.45	22.06	17.75	21.09	0.29	0.040352
Solyc02g082120.2.1	DNA-3-methyladenine	6.16	4	4.72	4.24	4.78	1.75	1.49	0.66	1.3	0.27	0.040362
Solyc12g095850.1.1	Tryptophanyl-tRNA sy	7.69	7.84	6.99	8.38	7.72	1.68	2.35	1.11	1.71	0.22	0.040404
Solyc04g010200.1.1	ABC transporter G fam	0.28	0.08	0.22	0.26	0.21	9.41	3.36	7.1	6.62	31.54	0.040407
Solyc01g087500.2.1	DNA topoisomerase 2	0.18	0.4	0.37	0.44	0.35	0	0.04	0	0.01	0.04	0.040454
Solyc11g005660.1.1	Serine/threonine-prote	1.88	4.81	4.15	4.65	3.87	8.35	7.08	12.12	9.18	2.37	0.040675
Solyc03g006490.2.1	Aluminum-induced pro	7.01	37.22	78.44	54.37	44.26	3.36	6.14	3.61	4.37	0.1	0.040702
Solyc01g087920.1.1	Serine carboxypeptidas	0	0.34	0.3	0	0.16	5.23	1.76	3.27	3.42	21.38	0.040864
Solyc09g075070.2.1	Beta-glucosidase (AHI	0.38	1.24	0.89	0.72	0.81	0.1	0	0.13	0.08	0.09	0.040885
Solyc04g076970.2.1	BZIP transcription fact	2.97	2.41	0.32	1.15	1.71	0	0	0	0	0.01	0.040903
Solyc11g012220.1.1	Wax synthase isoform	0.93	1.65	3.5	0.26	1.58	0	0	0	0	0.01	0.040904
Solyc01g098140.2.1	Neutral cholesterol est	0	0.09	0	0	0.02	6.24	1.91	1.31	3.15	140.15	0.040968
Solyc01g105520.2.1	DNA replication licens	4.39	3.71	4.66	3.73	4.12	1.06	1.34	1.42	1.27	0.31	0.041025
Solyc08g061970.2.1	Spermidine synthase (A	0.19	2.01	1.33	0.53	1.01	0	0	0	0	0.01	0.04107
Solyc03g006960.2.1	Protein phosphatase 2C	4.55	4.36	3.92	2.72	3.89	766.25	154.29	225.97	382.17	98.31	0.041145
Solyc04g078340.2.1	Cytochrome P450	0.25	0.68	0.45	0.72	0.53	1.53	1.96	1.13	1.54	2.93	0.041191
Solyc01g108140.2.1	B3 domain-containing	7.04	6	6.57	4.56	6.04	0.71	1.43	0.58	0.91	0.15	0.041198
Solyc07g007980.2.1	ATP binding / serine-tl	1.94	1.07	0.71	1.48	1.3	0.12	0.24	0	0.12	0.09	0.041297
Solyc03g097390.2.1	Acyl-ACP thioesterase	0.89	1.42	0.84	0.25	0.85	0	0	0	0	0.01	0.041336
Solyc04g076040.2.1	Cyclin d2 (AHRD V1	18.16	28.7	14.21	14.15	18.8	3.74	8.91	2.02	4.89	0.26	0.041418
Solyc08g068640.2.1	Decarboxylase family	1.77	0.86	0.57	2.73	1.48	0.13	0	0.32	0.15	0.1	0.041512
Solyc10g079160.1.1	Os01g0617600 protein	5.79	5.16	4.56	3.41	4.73	1.04	1.84	1.6	1.49	0.32	0.041512
Solyc01g008130.2.1	DEP domain-containin	14.85	14.65	15.21	17.59	15.57	4.37	6.57	5.03	5.32	0.34	0.041596
Solyc05g010580.2.1	Unknown Protein (AH	8.13	15.28	3.2	1.28	6.97	0	0	0	0	0	0.041596
Solyc12g019020.1.1	Unknown Protein (AH	0	0	0	0	0	1.45	0.63	0.76	0.95	94.67	0.041596
Solyc06g065690.2.1	Serine/threonine-prote	15.47	16.71	20.12	24.12	19.11	5.34	8.4	4.33	6.02	0.32	0.041615
Solyc08g043140.2.1	Arf-GAP with GTPase	1.33	1.33	0.98	1.41	1.26	0	0.14	0	0.05	0.04	0.041615
Solyc02g067710.1.1	HEAT repeat family pr	2.41	3.36	1.22	2.43	2.35	0.42	0.7	0.34	0.49	0.21	0.041754
Solyc09g008730.2.1	GTP-binding protein (A	27.67	32.12	24.81	25.68	27.57	9.12	12.99	8.13	10.08	0.37	0.04177
Solyc03g119010.2.1	Pre-mRNA-splicing fa	106.48	87.95	129.5	125.45	112.34	46.66	50.19	51.62	49.49	0.44	0.041792
Solyc03g034240.2.1	Chromosome 18 contig	0	0	0	0	0	5.52	1.7	5.9	4.37	437.33	0.041794
Solyc09g074530.2.1	MTN3 (AHRD V1 **-	1.84	4.5	12.47	2.93	5.44	0.19	0.19	0.23	0.2	0.04	0.041817
Solyc11g012520.1.1	Unknown Protein (AH	4.16	3.7	1.23	0.73	2.46	0	0	0	0	0	0.041827
Solyc10g012110.1.1	Non-specific lipid-tran	2.29	4.6	16.24	4.06	6.8	0	0	0	0	0	0.041841
Solyc06g060150.2.1	Replication factor C su	35.93	34.11	27.18	23.98	30.3	11.17	15.62	10.06	12.28	0.41	0.04185
Solyc07g040740.2.1	Diacylglycerol acyltran	3.33	3.19	1.66	3.99	3.04	0.31	0.63	0.89	0.61	0.2	0.04185
Solyc08g008500.2.1	Dof zinc finger protein	4.3	3.82	1.09	1.3	2.63	0.25	0	0.3	0.18	0.07	0.04185
Solyc02g067680.1.1	RING finger protein (A	1.82	1.94	0.43	2.06	1.56	0.29	0.15	0.36	0.27	0.17	0.041894
Solyc12g010030.1.1	Leucyl aminopeptidase	41.1	30.8	84.21	6.12	40.56	0.87	4.41	0	1.76	0.04	0.04201
Solyc03g120230.2.1	MAR-binding filament	6.71	10.22	10.08	6.89	8.47	2.6	3.82	3.19	3.2	0.38	0.042083
Solyc08g075910.1.1	RING finger protein 13	32.33	26.44	21.39	29.35	27.38	10.06	13.35	4.99	9.47	0.35	0.042108
Solyc03g115460.1.1	Unknown Protein (AH	17.51	16.26	12.27	15.07	15.28	6.97	3.31	6.04	5.44	0.36	0.042275
Solyc03g114580.2.1	Uridine kinase (AHRD	22.03	24.43	20.26	25.09	22.95	10.22	8.1	14.13	10.82	0.47	0.042361
Solyc02g082420.2.1	Ring finger protein (Al	28.12	33.45	35.41	37.48	33.62	15.73	11.46	7.78	11.66	0.35	0.042372
Solyc02g079290.2.1	SELF PRUNING 2G	25.06	27.22	41.31	15.12	27.18	5.36	12.91	4.02	7.43	0.27	0.042444
Solyc09g009130.2.1	Unknown Protein (AH	73.22	69.68	58.16	54.35	63.85	18.16	36.04	15.83	23.34	0.37	0.042475
Solyc12g011020.1.1	Had superfamily (Subf	6.53	7.32	8.95	5.07	6.97	1.36	3.09	1.88	2.11	0.3	0.042526
Solyc08g076190.2.1	Genomic DNA chromo	0.57	0.68	1.34	0.27	0.71	0	0.08	0	0.03	0.04	0.042666
Solyc04g014720.2.1	60S ribosomal protein	828.65	889.13	1010.12	981.84	927.44	416.66	442.92	415.38	424.99	0.46	0.042706
Solyc00g072100.2.1	LTRGag-pol-polymera	53.64	65.83	28.3	26.86	43.66	13.19	14.66	5.95	11.27	0.26	0.042748
Solyc04g055170.2.1	Annexin 2 (AHRD V1	33.51	32.6	30.45	37.97	33.63	11.6	15.26	11.57	12.81	0.38	0.042762
Solyc08g080890.2.1	Copper-exporting ATP	0.81	0.53	0.09	0.21	0.41	0	0	0	0	0.02	0.042843
Solyc12g021340.1.1	Rhodanese-like family	54.05	37.25	50.03	45.48	46.7	13.21	21.46	26.14	20.27	0.43	0.042876
Solyc03g044090.2.1	Phototropic-responsive	0.31	1.4	0.99	0.89	0.9	0.08	0.17	0	0.08	0.09	0.042923
Solyc09g008640.1.1	Receptor-like protein k	1.47	1.31	3.59	0.89	1.81	0.25	0.17	0	0.14	0.08	0.042978
Solyc01g010540.2.1	Ribosomal protein (AH	546.43	451.03	551.11	507.11	513.92	172.01	257.11	242.09	223.74	0.44	0.043025
Solyc03g005320.2.1	Fatty acid elongase 3-k	2.03	1.24	1.65	1.26	1.54	0.31	0	0.13	0.15	0.09	0.043218
Solyc01g014120.2.1	Unknown Protein (AH	3.07	3.75	1.81	1.63	2.57	0.46	0.78	0.38	0.54	0.21	0.043253
Solyc02g065470.1.1	Pathogenesis-related p	242.38	271.84	135.45	420.69	267.59	80.39	87.32	35.82	67.84	0.25	0.043253
Solyc12g005730.1.1	Glycosyltransferase-lik	2.47	6.12	3.57	0.58	3.19	12.33	10.19	9.96	10.83	3.4	0.043334
Solyc06g064500.2.1	O-methyltransferase (A	29.01	44.41	17.22	33.46	31.02	73.45	79.81	67.68	73.65	2.37	0.043341
Solyc04g080450.1.1	Fatty acid elongase 3-k	18.08	19.95	24.64	28.28	22.74	5.35	10.9	4.8	7.02	0.31	0.043386
Solyc08g062180.1.1	Unknown Protein (AH	1.36	2.27	0.4	2.65	1.67	0.14	0.28	0	0.14	0.08	0.043426
Solyc02g062800.2.1	F-box family protein (A	21.11	20.7	24.71	33.83	25.09	8.93	6.99	11.79	9.24	0.37	0.043552
Solyc09g090030.1.1	SET and zf-MYND do	8.16	12.36	9.22	6.38	9.03	2.81	3.82	2.07	2.9	0.32	0.043552
Solyc03g007760.2.1	Cell division protease	29.75	26.13	25.41	11.85	23.28	11.41	7.15	8.31	8.96	0.38	0.043624
Solyc06g076640.2.1	Tubulin beta chain (AI	131.21	117.56	112.37	196.93	139.52	31.88	65.03	27.03	41.31	0.3	0.043697
Solyc07g041020.2.1	Cell growth-regulating	28.57	24.12	31.23	27.34	27.82	9.51	13.01	10.64	11.05	0.4	0.043751
Solyc06g073130.2.1	ProFAR isomerase asso	9.9	13.56	11.67	13.55	12.17	4.7	4.35	5.92	4.99	0.41	0.04377
Solyc01g068530.2.1	30S ribosomal protein	422.48	345.28	438.13	444.95	412.71	167.31	180.85	168.69	172.28	0.42	0.043811
Solyc05g006770.2.1	Kinase family protein (	9.16	14.52	8.43	8.81	10.23	2.96	4.48	2	3.15	0.31	0.043853
Solyc02g063460.1.1	Os08g0119500 protein	19.53	17.11	12.22	19.67	17.13	28.2	58.58	25.55	37.44	2.19	0.044016
Solyc07g052780.2.1	Tir, resistance protein	3.72	3.31	3.41	7.6	4.51	0.67	0.67	0.41	0.58	0.13	0.044061

Solyc02g030130.2.1	Subtilisin-like serine p	0.19	0.75	0.44	1.06	0.61	0	0	0.09	0.03	0.05	0.044106
Solyc05g051560.2.1	Mitochondrial carrier p	11.61	13.23	6.76	9.64	10.31	3.38	5.43	1.23	3.35	0.32	0.044109
Solyc03g078430.1.1	Cinnamyl alcohol dehy	1.58	1.4	1.49	1.79	1.56	0	0	0	0	0.01	0.044161
Solyc01g014200.1.1	Unknown Protein (AH	20.47	12.15	10.74	16.09	14.86	0.92	4.63	2.26	2.6	0.18	0.044431
Solyc10g084600.1.1	Plant-specific domain	0	0	0	0	0	71.23	5.86	107.15	61.41	6141.33	0.044432
Solyc08g065440.2.1	Os01g0600300 protein	4.72	5.52	4.77	3.63	4.66	1.63	1.98	1.21	1.61	0.34	0.04448
Solyc06g062670.2.1	Zinc finger family prot	31.15	29.36	26.1	22.59	27.3	4.65	16.21	2.6	7.82	0.29	0.044487
Solyc04g074510.2.1	14-3-3 protein beta/alp	289.39	235.73	276.9	332.68	283.68	86.39	149.68	79.84	105.3	0.37	0.044602
Solyc09g072710.2.1	Phosphatidylcholine-st	13.88	12.24	14.19	15.52	13.96	5.02	6.57	3.81	5.13	0.37	0.044615
Solyc08g080340.2.1	Lysophospholipid acyl	9.38	9.49	9.23	16.09	11.05	2.18	4.29	2.54	3	0.27	0.044653
Solyc05g055990.2.1	Aquaporin 2 (AHRD V	0.45	1.42	1.34	1.29	1.12	0	0	0	0	0.01	0.044699
Solyc06g073320.2.1	GDP-L-galactose phos	630.34	637.01	1129.1	683.04	769.87	334.88	345.99	344.6	341.82	0.44	0.044757
Solyc05g014240.2.1	Pto-like, Serine/threon	6.97	9.31	6.63	3.62	6.63	2.99	2.73	1.27	2.33	0.35	0.044829
Solyc05g052100.2.1	Glutamate dehydrogen	20.87	14.47	22.18	18.48	19	4.38	8.31	3.8	5.5	0.29	0.045012
Solyc10g076720.1.1	Unknown Protein (AH	1.31	4.09	0.77	1.86	2.01	0.27	0	0	0.09	0.04	0.045275
Solyc12g005330.1.1	50S ribosomal protein	396.08	324.39	391.55	351.45	365.87	128.45	170.34	142.76	147.18	0.4	0.045275
Solyc01g109230.2.1	CHP-rich zinc finger p	2.38	3.07	2.81	0.37	2.16	0.43	0	0.53	0.32	0.15	0.045302
Solyc01g079880.2.1	Asparagine synthetase	777.74	912.91	1115.26	496.11	825.5	358.92	413.73	325.48	366.04	0.44	0.045313
Solyc04g072740.2.1	High affinity sulfate tr	2.22	2.87	1.55	0.29	1.73	0.41	0.08	0	0.16	0.09	0.045313
Solyc06g072810.2.1	Unknown Protein (AH	0.47	0.58	0.11	0.66	0.45	0	0	0	0	0.02	0.045313
Solyc03g079980.1.1	DUF599 family protein	0	0	0	0	0	1.14	0.92	1.12	1.06	106	0.045394
Solyc02g085760.2.1	Rhomboid family prote	29.9	21.26	13.23	8.27	18.16	1.97	6.36	5.32	4.55	0.25	0.045444
Solyc08g005780.2.1	Beta-amylase (AHRD	11.47	13.91	13.65	17.07	14.03	5.51	5.56	6.87	5.98	0.43	0.045444
Solyc08g080130.2.1	Phospholipase D (AHR	10.28	10.81	10.29	16.31	11.92	2.01	5.01	2.4	3.14	0.26	0.045518
Solyc05g032690.2.1	3-hydroxyisobutyryl-C	40.32	61.98	14.7	26.96	35.99	10.07	6.57	10.91	9.18	0.26	0.045564
Solyc02g080460.1.1	Kinesin heavy chain (F	4.66	4.14	4.58	6.59	4.99	0	0.63	0.77	0.47	0.09	0.045626
Solyc02g081690.1.1	Glucosyltransferase (A	3.97	3.53	1.45	2.32	2.82	1.22	0.45	0.95	0.87	0.31	0.045644
Solyc04g077840.2.1	F-box/kelch-repeat pro	8.55	13.05	6.49	12.1	10.05	21.71	20.91	18.19	20.27	2.02	0.045997
Solyc07g051950.2.1	C4-dicarboxylate trans	0	0	0	0	0	11.91	1.95	11.52	8.46	846	0.046041
Solyc03g007220.2.1	Membrane receptor-lik	36.97	52.91	15.4	18.46	30.94	6.44	7.09	4.32	5.95	0.19	0.046137
Solyc02g085250.2.1	FAD dependent oxidor	21.52	19.68	17.66	16.38	18.81	4.82	9.82	7.43	7.36	0.39	0.046178
Solyc06g016790.1.1	Unknown Protein (AH	27.69	27.91	15.37	54.15	31.28	1.65	6.97	0.81	3.14	0.1	0.046178
Solyc01g095950.1.1	DNA-directed RNA po	0	0	0	0	0	5.05	1.39	2.82	3.09	308.67	0.046227
Solyc02g071580.2.1	Subtilisin-like protease	1.36	0.83	1.4	0.84	1.11	3.37	2.15	2.7	2.74	2.47	0.046352
Solyc03g093490.2.1	WD-repeat protein (AI	4.16	2.96	1.72	2.35	2.8	0.67	0.68	0	0.45	0.16	0.046597
Solyc01g104110.2.1	Legumin 11S-globulin	0	0	0	0	0	0.88	0.59	0.54	0.67	67	0.046602
Solyc02g080780.2.1	Unknown Protein (AH	14.05	21.62	10.4	8.09	13.54	4.04	5.43	4.73	4.73	0.35	0.046602
Solyc03g095300.2.1	Proline-rich family pro	15.47	10.93	4.83	3.86	8.77	1.1	1.85	0	0.98	0.11	0.046602
Solyc00g007100.2.1	Serine/threonine protei	26.3	24.19	25.14	23.07	24.67	11.51	11.49	9.19	10.73	0.43	0.046602
Solyc12g008430.1.1	Malic enzyme (AHRD	0.22	0.1	0.4	0.16	0.22	2.26	2.64	8.43	4.44	20.2	0.046602
Solyc01g109840.2.1	Diaminopimelate deca	31.05	48.35	45.78	24.7	37.47	6.27	3.16	7.7	5.71	0.15	0.046719
Solyc12g098640.1.1	Cycloeucalenol cyclois	35.63	51.76	32.27	37.72	39.34	13.19	19.67	13.23	15.36	0.39	0.046761
Solyc06g068130.2.1	TPR domain protein (A	48.73	22.17	0.65	0.77	18.08	0	0	0.27	0.09	0	0.046802
Solyc08g068780.1.1	N-acetyltransferase (A	6.32	12.23	3.57	3.89	6.5	0.67	0.45	1.64	0.92	0.14	0.046876
Solyc03g059160.1.1	CASTOR protein (Frag	14.12	18.36	20.18	12.67	16.33	6.36	5.97	5.66	6	0.37	0.047129
Solyc03g082580.2.1	Glucosamine-6-phosph	78.38	84.85	81.25	98.58	85.77	37.59	41.46	30.59	36.55	0.43	0.047129
Solyc08g080510.2.1	Replication factor C su	20.27	19.45	16.64	15.46	17.95	6.58	9.23	5.52	7.11	0.4	0.047129
Solyc08g079480.2.1	Unknown Protein (AH	1.2	2.14	1.77	0.85	1.49	11.14	4.15	13.99	9.76	6.55	0.04713
Solyc08g067190.2.1	Anaphase promoting c	1.98	1.94	1.61	1.25	1.69	0.39	0.61	0.74	0.58	0.34	0.04746
Solyc08g066860.2.1	Os02g0658033 protein	1.9	1.69	1.12	1.12	1.46	0.13	0.26	0.31	0.23	0.16	0.047465
Solyc10g005870.2.1	Peptidyl-prolyl cis-tran	19.73	19.75	17.46	18.31	18.81	4.23	9.29	3.98	5.83	0.31	0.047598
Solyc11g013890.1.1	F-box family protein (A	11.51	14.83	5.27	4.37	8.99	1.94	2.38	1.19	1.84	0.2	0.047734
Solyc10g074610.1.1	Pentatricopeptide repe	17.59	11.74	16.14	18.66	16.03	4.34	4.78	4.37	4.5	0.28	0.047867
Solyc01g098670.1.1	Kinesin 1-like (AHRD	0	0	0	0	0	0	1.04	0.32	0.45	45.33	0.047887
Solyc04g008570.2.1	Pentatricopeptide repe	2.92	6.45	5.23	1.71	4.08	0.9	0.98	0.6	0.83	0.2	0.047902
Solyc04g064930.2.1	Alkyl hydroperoxide re	21.21	19.57	22.28	21.96	21.26	5.64	12.65	7.7	8.66	0.41	0.048021
Solyc10g084970.1.1	Unknown Protein (AH	6.56	6.59	10.13	7.14	7.61	2.04	2.61	1.5	2.05	0.27	0.048027
Solyc03g031850.1.1	Unknown Protein (AH	4.02	5.53	1.94	3.36	3.71	61.27	34.25	21.22	38.91	10.48	0.048073
Solyc09g074640.2.1	Zinc finger CCCH dom	35.86	39.05	46.06	32.96	38.48	18.46	20.47	13.37	17.43	0.45	0.048075
Solyc08g005570.2.1	DUF647 domain-conta	6.85	8.22	8.59	7.69	7.84	2.72	2.83	3.67	3.07	0.39	0.048099
Solyc08g023490.2.1	Pentatricopeptide repe	6.23	6.1	5.32	6	5.91	2.11	2.5	1.34	1.98	0.34	0.048099
Solyc01g096660.2.1	Genomic DNA chromo	117.04	101.71	85.97	64.42	92.29	259.13	237.07	150.14	215.45	2.33	0.048239
Solyc01g086910.2.1	Unknown Protein (AH	2.38	4.24	13.13	26.98	11.68	0	0	0	0	0	0.048252
Solyc11g066660.1.1	Magnesium transporter	28.13	34.86	25.02	15	25.75	11.18	13.32	7.89	10.8	0.42	0.048471
Solyc06g068890.1.1	Unknown Protein (AH	0	0	0	0	0	3.9	3.28	2.4	3.19	319.33	0.048619
Solyc03g078090.2.1	Pectinesterase family p	2.38	2.94	1.09	0.75	1.79	0.11	0.54	0.13	0.26	0.15	0.048743
Solyc06g071960.2.1	Nucleoside diphosphat	73.01	64.45	48.77	73.82	65.01	23.93	42.53	29.39	31.95	0.49	0.048886
Solyc06g076850.2.1	Binding protein (AHRI	46.37	24.46	12.55	9.47	23.21	1.26	2.34	1.66	1.75	0.08	0.048937
Solyc03g111120.2.1	Malate synthase (AHR	0.27	0.96	1.27	0.38	0.72	1.31	4.17	2.41	2.63	3.65	0.048983
Solyc06g066800.1.1	Glycosyl transferase fa	38.62	48.93	16.14	33.76	34.36	12.13	14.64	7.19	11.32	0.33	0.048983
Solyc03g078400.2.1	Actin (AHRD V1 ***-	1676.33	1569.74	2172.91	1894.71	1828.42	672.15	869.79	753.05	765	0.42	0.048995
Solyc01g079950.2.1	Xylanase inhibitor (Fra	0.62	1.51	1.09	2.83	1.51	0.12	0	0	0.04	0.03	0.049083
Solyc07g047840.2.1	Unknown Protein (AH	35.76	50.48	30.55	23.55	35.09	10.46	16.58	3.67	10.24	0.29	0.049166
Solyc02g091140.2.1	S-adenosyl-L-methioni	4.08	4.42	3.77	4.01	4.07	5.16	13.58	6.34	8.36	2.05	0.049267
Solyc01g089970.2.1	Nucleoside diphosphat	1106.9	891.35	1292.85	1305.8	1149.22	481.83	497.89	566.93	515.55	0.45	0.049366
Solyc03g059150.2.1	DMI1 protein (Fragme	15.4	14.9	14.22	9.47	13.5	4.06	3	5.65	4.24	0.31	0.049366
Solyc10g008340.2.1	Os05g0169400 protein	32.26	27.21	50.03	37.26	36.69	14.64	16.93	17.03	16.2	0.44	0.049367
Solyc07g065970.1.1	Chaperone protein dna	1.6	1.42	0.94	3.96	1.98	1.94	9.12	13.1	8.05	4.07	0.049458
Solyc04g056580.2.1	Homology to unknown	101.58	111.74	56.56	59.09	82.24	28.95	43.9	19.94	30.93	0.38	0.049461
Solyc03g120800.2.1	Peroxidase (AHRD V1	0.48	1.55	0.75	1.12	0.98	0	0.13	0	0.04	0.04	0.049461
Solyc04g079900.2.1	GP63 leishmanolysin (	18.23	23.17	24.15	41.63	26.8	3.68	9.06	3.22	5.32	0.2	0.049498
Solyc12g044320.1.1	WD-40 repeat family p	0.44	1.16	0.69	0.41	0.68	0	0	0	0	0.01	0.04966

Solyc01g060240.2.1	Unknown Protein (AH	207.49	155.31	97.92	111.38	143.03	58.46	61.56	39.08	53.03	0.37	0.049764
Solyc04g077480.2.1	Transcription factor (A	7.58	7.97	7.11	10.23	8.22	1.95	4.35	1.71	2.67	0.32	0.049773
Solyc09g018510.2.1	Phosphatidate cytidyly	42.41	49.93	49.77	47.61	47.43	21.75	22.86	14.92	19.84	0.42	0.049773
Solyc03g119700.2.1	ATP-dependent Clp pr	195.85	179.44	166.85	134.72	169.22	89.61	84.67	72.03	82.1	0.49	0.049778
Solyc10g080000.1.1	Transcription initiation	19.79	25.29	23.35	11.49	19.98	10.25	5.79	5.54	7.19	0.36	0.049828
Solyc02g081250.1.1	Pentatricopeptide repe	0.41	0.96	0.71	0.85	0.73	0.13	0.1	0.31	0.18	0.25	0.049858
Solyc05g053670.2.1	60S ribosomal protein	497.81	418.37	613.26	701.9	557.84	221.43	223.6	232.19	225.74	0.4	0.049906
Solyc07g056420.2.1	Glutathione S-transfera	80.9	85.81	66.91	76	77.41	540.7	281.92	800.6	541.07	6.99	0.049912
Solyc02g078620.1.1	Dof zinc finger protein	2.55	1.44	0.55	0.98	1.38	0	0	0	0	0.01	0.049985

Supplemental Table 3. Expression of carotenoid biosynthesis genes expression in AC and hp1 at various fruit developmental stages.

Gene ID	Gene name	Annotation	IG				MG				BK				BK7			
			AC	*hp1*	ratio *hp1* /AC	adjust p	AC	*hp1*	ratio *hp1* /AC	adjust p	AC	*hp1*	ratio *hp1* /AC	adjust p	AC	*hp1*	ratio *hp1* /AC	adjust p
Solyc01g067890	DXS	1-deoxy-D-xylulo	51.09	45.71	0.89	1	44.84	57.73	1.29	0.506132	128.71	149.55	1.16	1	96.59	119.32	1.24	1
Solyc03g114340	DXR	1-deoxy-D-xylulo	74.51	108.54	1.46	0.111554	70.37	90.15	1.28	0.481875	49.26	53.52	1.09	1	19.78	32.14	1.62	0.929803
Solyc09g008920	GGPS1	Geranylgeranyl py	107.28	161.93	1.51	0.048442	97.76	180.42	1.85	0.001459	140.96	163.12	1.16	1	85.71	165.37	1.93	0.272528
Solyc04g079960	GGPS2	Geranylgeranyl py	3.05	4.81	1.58	0.333448	1.98	1.62	0.82	1	90.05	81.46	0.9	1	4.23	7.26	1.72	1
Solyc03g031860	PSY1	Phytoene synthase	143.75	240.25	1.67	0.011295	139.97	185.14	1.32	0.281439	5794.56	6729.6	1.16	1	5128.62	5261.89	1.03	1
Solyc02g081330	PSY2	Phytoene synthase	21.17	47.33	2.24	0.000107	19.92	33.94	1.7	0.019315	30.5	34.11	1.12	1	18.42	51.41	2.79	0.213629
Solyc03g123760	PDS	Phytoene desatura	42.48	61.79	1.45	0.12196	43.93	74.99	1.71	0.010587	91.2	87.55	0.96	1	53.34	78.19	1.47	1
Solyc01g097810	ZDS	Zeta-carotene des	39.8	49.1	1.23	0.553086	34.9	46.68	1.34	0.324188	53.79	61.54	1.14	1	54.99	75.1	1.37	1
Solyc12g098710	ZISO	15-cis-zeta-carote	13.03	16.12	1.24	0.615761	17.17	18.87	1.1	1	767.74	600.56	0.78	1	1016.81	919.83	0.9	1
Solyc10g081650	CRTISO	Carotenoid isome	22.18	29.44	1.33	0.364156	33.47	34.65	1.04	1	79.97	96.25	1.2	1	121.58	128.69	1.06	1
Solyc06g074240	CYCB (NXS, Beta)	Chromoplast-spec	1.91	1.6	0.84	1	2.37	2.57	1.09	1	3.67	1.86	0.51	1	1.18	1.01	0.86	1
Solyc04g040190	LCYB1 (CrtLB1)	Lycopene beta-cyc	12.96	22.58	1.74	0.034472	10.9	10.6	0.97	1	1.17	2.08	1.78	1	0.85	3.41	4.03	0.217463
Solyc10g079480	LCYB2	Lycopene beta-cyc	0.69	1.22	1.77	0.674762	0.51	0.68	1.33	1	0.04	0	0.27	1	0.03	0	0.3	1
Solyc12g008980	LCYE (CrtLE)	Lycopene epsilon	5.91	14.53	2.46	0.000287	4.24	5.46	1.29	0.834961	0.61	0.24	0.4	1	0.06	0	0.16	1
Solyc06g036260	CrtR-B1 (CHYB1)	Beta-carotene hyd	46.32	104.62	2.26	0.000458	113.19	208.43	1.84	0.001457	86.37	104.58	1.21	1	89.41	175.85	1.97	0.700205
Solyc03g007960	CrtR-B2 (CHYB2, BC	Beta-carotene hyd	51.04	28.63	0.56	0.544847	29.03	8.54	0.29	0.000179	30.87	22.55	0.73	1	33.89	36.34	1.07	1
Solyc02g090890	ZEP	Zeaxanthin epoxi	20.76	35.41	1.71	0.061562	26.2	45.19	1.72	0.012269	13.53	17.04	1.26	1	17.02	27.61	1.62	0.826874
Solyc04g050930	VDE1	Violaxanthin de-e	33.5	76.83	2.29	1.45E-05	30.87	61.84	2	0.000482	33.13	43.7	1.32	1	3.52	25.88	7.36	0.000869

Supplemental Table 4. Significantly changed pathways in hp1 at various developmental stages.

IG	MG	BK	BK7
Oxygenic photosynthesis	Oxygenic photosynthesis	Oxygenic photosynthesis	Oxygenic photosynthesis
Calvin-Benson-Bassham cycle	Calvin-Benson-Bassham cycle	Calvin-Benson-Bassham cycle	Calvin-Benson-Bassham cycle
Photosynthesis light reactions	Photosynthesis light reactions	Photosynthesis light reactions	
Gluconeogenesis	Gluconeogenesis	Gluconeogenesis	Gluconeogenesis
Photorespiration	Photorespiration	Photorespiration	
Formaldehyde assimilation III (dihydroxyacetone cycle)	Formaldehyde assimilation III (dihydroxyacetone cycle)	Formaldehyde assimilation III (dihydroxyacetone cycle)	Formaldehyde assimilation III (dihydroxyacetone cycle)
Glycolysis IV (plant cytosol)	Glycolysis IV (plant cytosol)	Glycolysis IV (plant cytosol)	Glycolysis IV (plant cytosol)
Glycolysis I	Glycolysis I	Glycolysis I	Glycolysis I
Superpathway of glycolysis, pyruvate dehydrogenase, TCA, and glyoxylate bypass	Superpathway of glycolysis, pyruvate dehydrogenase, TCA, and glyoxylate bypass	Superpathway of glycolysis, pyruvate dehydrogenase, TCA, and glyoxylate bypass	Superpathway of glycolysis, pyruvate dehydrogenase, TCA, and glyoxylate bypass
Glycolysis III	Glycolysis III	Glycolysis III	Glycolysis III
Rubisco shunt		Rubisco shunt	
Luteolin biosynthesis	Choline degradation I		S-adenosyl-L-methionine cycle
Glycine cleavage complex	Glycine betaine biosynthesis I (Gram-negative bacteria)		C4 photosynthetic carbon assimilation cycle
Chlorophyllide a biosynthesis I			S-adenosylmethionine biosynthesis
removal of superoxide radicals			Methionine degradation I (to homocysteine)
heme biosynthesis from uroporphyrinogen I			2'-deoxymugineic acid phytosiderophore biosynthesis
			Methionine and methyl-donor-molecule biosynthesis
			TCA cycle variation IV

Supplemental Table 5. List of primers used in this study.

Gene name	Gene ID	Primer name	Primer sequence	Usage
TIP41	Solyc10g049850	C196-TIP41-F	GCTGCGTTTCTGGCTTAGG	qRT-PCR primers, internal control
		C197-TIP41-R	ATGGAGTTTTTGAGTCTTCTGC	
RIN	Solyc05g012020	C206-RIN-F	AACATCATGGCATTGTGGTG	qRT-PCR primers
		C207-RIN-R	GTGTTGATGGTGCTGCATTT	
FUL1	Solyc06g069430	C208-FUL1-F	GTTTTGCCACAACAACTGGACTC	qRT-PCR primers
		C209-FUL1-R	CTTGCTGCTGTGAAGAACTACC	
FUL2	Solyc03g114830	C226-FUL2-F	CGATGCTGAAGTTGGACTCA	qRT-PCR primers
		C227-FUL2-R	TCCTTTCCATGCAAGAGTCA	

Supplemental Dataset 1. Expression of tomato genes (RPKM) in *hp1* fruit bottom part tissues compared to AC fruit bottom part tissues at IG, MG, BK and BK7 stages.

GeneID	Annotation	AC_IG_B					*hp1* _IG_B					ratio	adjust p	AC_MG_B				*hp1* _MG_B				ratio	adjust p	AC_BK_B				*hp1* _BK_B				ratio	adjust p	AC_BK7_B				*hp1* _BK7_B			ratio	adjust p
		rep1	rep2	rep3	rep4	mean	rep1	rep2	rep3	rep4	mean			rep1	rep2	rep3	mean	rep1	rep2	rep3	mean			rep1	rep2	rep3	mean	rep1	rep2	rep3	mean			rep1	rep2	rep3	mean	rep1	rep2	mean		
Solyc00g005000.2.1	Aspartic proteinase nepenthe	0	0	0	0	0	0	0	0	0	0	1	NA	0	0.12	0	0.04	0	0	0.11	0.04	0.92	1	0	0	0	0	0	0	0	0	1	NA	0.2	0	0	0.07	0	0	0	0.15	1
Solyc00g005020.1.1	Unknown Protein (AHRD V1)	0	0	0	0	0	0	0	0	0	0	1	NA	0	0	0	0	0	0	0	0	1	NA	0	0	0	0	0	0	0	0	1	NA	0	0.3	0	0.1	0	0	0	0.1	1
Solyc00g005040.2.1	Potassium channel (AHRD V1	3.33	3.95	5.23	3.92	4.11	3.7	3.06	2.55	3.58	3.22	0.78	1	5.67	7.23	7.02	6.64	6.54	9.19	5.66	7.13	1.07	1	3.58	4.07	3.85	3.83	2.91	2.94	4.04	3.3	0.86	1	7.88	6.85	11.08	8.6	6.6	11.18	8.89	1.03	1
Solyc00g005050.2.1	Arabinogalactan protein (AHI	107.03	125.82	90.94	83.79	101.9	100.64	114.53	131.83	116.23	115.81	1.14	0.887153	107.08	118.21	79.89	101.73	86.38	101.78	106.33	98.16	0.96	1	105.24	133.3	84.53	107.69	98.84	106.64	106.94	104.14	0.97	1	80.88	119.3	80.74	93.64	128.18	108.31	118.25	1.26	1
Solyc00g005060.1.1	Unknown Protein (AHRD V1)	0	0	0	0	0	0	0	0	0	0	1	NA	0	0	0	0	0	0	0	0	1	NA	0	0	0	0	0	0	0	0	1	NA	0	0	0	0	0	0	0	1	NA
Solyc00g005070.1.1	Unknown Protein (AHRD V1)	0	0	0	0	0	0	0	0	0	0	1	NA	0	0	0	0	0	0	0	0	1	NA	0	0	0	0	0	0	0	0	1	NA	0	0	0	0	0	0	0	1	NA
Solyc00g005080.1.1	Ycf2 (AHRD V1 ***- A6YA23_	0	0	0	0.94	0.23	0	0	0	0	0	0.04	1	0	0	0	0	0	0	0	0	1	NA	0	0.54	0	0.18	0	0	0.49	0.16	0.91	1	0.95	0	0	0.32	0.61	0	0.3	0.96	1
Solyc00g005090.1.1	Unknown Protein (AHRD V1)	0	0	0	0	0	0	0	0	0	0	1	NA	0	0	0	0	0	0	0	0	1	NA	0	0	0	0	0	0	0	0	1	NA	0	0	0	0	0	0	0	1	NA
Solyc00g005100.1.1	Unknown Protein (AHRD V1)	0	0	0	0	0	0	0	0	0	0	1	NA	0	0	0	0	0	0	0	0	1	NA	0	0	0	0	0	0	0	0	1	NA	0	0	0	0	0	0	0	1	NA
Solyc00g005110.1.1	Unknown Protein (AHRD V1)	0	0	0	0	0	0	0	0	0	0	1	NA	0	0	0	0	0	0.24	0	0.08	8	1	0	0	0	0	0	0	0	0	1	NA	0.65	0	0	0.22	0	0	0	0.05	1
Solyc00g005120.1.1	Unknown Protein (AHRD V1)	0	0	0	0	0	0	0	0	0	0	1	NA	0	0	0.9	0.3	0	0	0	0	0.03	1	0	0	0	0	0	0	0	0	1	NA	0	1.55	0	0.52	0	2.8	1.4	2.71	1
Solyc00g005130.1.1	Ulp1 protease family C-termi	0	0	0	0	0	0	0	0	0	0	1	NA	0	0	0	0	0	0	0	0	1	NA	0	0	0	0	0	0	0	0	1	1	0	0.79	0	0.26	0	0.47	0.23	0.89	1
Solyc00g005140.1.1	Unknown Protein (AHRD V1)	0	0	0	0	0	0	0	0	0	0	1	NA	0	0	0	0	0	0	0	0	1	NA	0	0	0.38	0.13	0	0	0	0	0.08	NA	0.55	0.28	1.19	0.67	0	0	0	0.01	1
Solyc00g005150.1.1	HAT dimerisation domain-cor	1.56	1.23	1.63	0.73	1.29	0.96	0.55	1.91	1.12	1.14	0.88	1	1.13	1.81	2.63	1.86	1.08	1.97	2.86	1.97	1.06	1	0.98	0.85	0.52	0.78	1.14	0.92	1.14	1.07	1.36	1	0.49	1.63	1.33	1.15	1.9	1.52	1.71	1.49	1
Solyc00g005160.1.1	Retrovirus-related Pol polypr	0	0	0	0	0	0	0	0	0	0	1	NA	0	0	0	0	0	0	0	0	1	NA	0	0	0	0	0	0	0	0	1	NA	0	0	0	0	0	0	0	1	NA
Solyc00g005170.1.1	Unknown Protein (AHRD V1)	0	0	0	0	0	0	0	0	0	0	1	NA	0	0	0	0	0	0	0	0	1	NA	0	0	0	0	0	0	0	0	1	NA	0	0	0	0	0	0	0	1	NA
Solyc00g005180.1.1	Unknown Protein (AHRD V1)	0	0	0	0	0	0	0	0	0	0	1	NA	0	0	0	0	0	0	0	0	1	NA	0	0	0	0	0	0	0	0	1	NA	0	0	0	0	0	0	0	1	NA
Solyc00g005280.1.1	Unknown Protein (AHRD V1)	0	0	0	0	0	0	0	0	0	0	1	NA	0	0	0	0	0	0	0	0	1	NA	0	0	0	0	0	0	0	0	1	NA	0	0	0	0	0	0	0	1	NA
Solyc00g005420.1.1	Unknown Protein (AHRD V1)	0	0	0	0	0	0	0	0	0	0	1	NA	0	0	0	0	0	0	0	0	1	NA	0	0	0	0	0	0	0	0	1	NA	0	0	0	0	0	0	0	1	NA
Solyc00g005430.1.1	Unknown Protein (AHRD V1)	0	0	0	0	0	0	0	0	0	0	1	NA	0	0	0	0	0	0	0	0	1	NA	0	0	0	0	0	0	0	0	1	NA	0	0	0	0	0	0	0	1	NA
Solyc00g005440.1.1	40S ribosomal protein S9 (AH	6.6	11.05	10.99	9.33	9.49	2.16	3.37	2.85	9.19	4.39	0.46	0.252293	11.19	7.76	9.51	9.49	4.84	4.62	4.57	4.68	0.49	0.297988	8.47	1.26	6.16	5.3	2.04	3.53	3.39	2.99	0.56	1	1.1	0.56	0.6	0.75	2.84	2.38	2.61	3.46	0.929803
Solyc00g005450.1.1	Unknown Protein (AHRD V1)	0	0	0	0	0	0	0	0	0	0	1	NA	0	0	0	0	0	0	0	0	1	NA	0	0	0	0	0	0	0	0	1	NA	0	0	0	0	0	0	0	1	NA
Solyc00g005460.1.1	Unknown Protein (AHRD V1);	0	0	0	0	0	0	0	0	0	0	1	NA	0	0	0	0	0	0	0	0	1	NA	0	0	0	0	0	0	0	0	1	NA	0	0	0	0	0	1.11	0.56	55.5	1
Solyc00g005470.1.1	40S ribosomal protein S9 (AH	0	0	0	0	0	0	0	0	0	0	1	NA	0	0	0	0	0	0	0	0	1	NA	0	0	0	0	0	0	0	0	1	NA	0	0	0	0	0	0	0	1	NA
Solyc00g005480.1.1	Ubiquitin carrier protein (AHI	0	0	0	0	0	0	0	0	0	0	1	NA	0	0	0	0	0	0	0	0	1	NA	0	0	0	0	0	0	0	0	1	NA	0	0	0	0	0	0	0	1	NA
Solyc00g005490.1.1	Unknown Protein (AHRD V1)	0	0	0	0	0	0	0	0	0	0	1	NA	0	0	0	0	0	0	0	0	1	NA	0	0	0	0	0	0	0	0	1	NA	0	0	0	0	0	0	0	1	NA
Solyc00g005840.2.1	Thioredoxin family protein (A	31.71	34.52	47.1	34.44	36.94	79.63	61.47	57.53	55.09	63.43	1.72	0.018573	32.66	33.23	28.46	31.45	41.63	48.08	52.73	47.48	1.51	0.143915	15.78	17.53	8.71	14.01	17.67	8.8	13.62	13.36	0.95	1	2.42	2.06	0.87	1.78	1.3	5.21	3.25	1.83	1
Solyc00g005850.1.1	Unknown Protein (AHRD V1)	0	0	0	0	0	0	0	0	0	0	1	NA	0	0	0	0	0	0	0	0	1	NA	0	0	0	0	0	0	0	0	1	NA	0	0	0	0	0	0	0	1	NA
Solyc00g005860.1.1	Unknown Protein (AHRD V1)	1.27	1.13	0.75	0	0.79	0.71	1	2.33	0	1.01	1.28	1	1.18	0.55	0.54	0.76	0	1.31	1.99	1.1	1.45	1	1.02	0	1.26	0.76	0	0	0	0	0.01	1	1.8	1.84	0	1.21	1.74	0	0.87	0.72	1
Solyc00g005870.1.1	Mutator-like transposase (AH	0.37	0.67	0.66	0.26	0.49	0	0	0	0	0	0.02	0.198448	0.35	0.65	0.47	0.49	0.26	0.29	1.03	0.53	1.07	1	0.3	0.15	0.19	0.21	0.25	0.57	0.54	0.45	2.13	1	0	0.14	0	0.05	0	0.33	0.17	3.54	1
Solyc00g005880.1.1	Unknown Protein (AHRD V1)	0	0	0	0	0	0	0	0.33	0	0.08	8.25	1	0	0	0	0	0	0.37	0.28	0.22	21.67	1	0	0	0	0	0	0	0	0	1	NA	0	0	0	0	0	0	0	1	NA
Solyc00g005890.2.1	Unknown Protein (AHRD V1)	2.87	0	0	0	0.72	0	0	0	0	0	0.01	1	0	0	0	0	0	0	0	0	1	NA	0	0	0	0	0	0	0	0	1	NA	0	0	0	0	0	0	0	1	NA
Solyc00g005900.1.1	Oxygen-evolving enhancer pr	0	0	0	0	0	0	0	0	0	0	1	NA	0	0	0	0	0	0	0	0	1	NA	0	0	0	0	0	0	0	0	1	NA	0	0	0	0	0	0	0	1	NA
Solyc00g005980.1.1	Zinc finger CCHC-type (AHRD	0	0	0	0	0	0	0	0	0	0	1	NA	0	0	0	0	0	0	0	0	1	NA	0	0	0	0	0	0	0	0	1	NA	0	0	0	0	0	0	0	1	NA
Solyc00g006010.1.1	Unknown Protein (AHRD V1)	0	0	0	0	0	0	0	0	0	0	1	NA	0	0	0	0	0	0	0	0	1	NA	0	0	0	0	0	0	0	0	1	NA	0	0	0	0	0	0	0	1	NA
Solyc00g006020.1.1	Endonuclease/exonuclease/p	0	0	0	0	0	0	0	0	0	0	1	NA	0	0	0	0	0	0	0	0	1	NA	0	0	0	0	0	0	0	0	1	NA	0	0	0	0	0	0	0	1	NA
Solyc00g006050.1.1	Unknown Protein (AHRD V1);	0	0	0	0	0	0	0	0	0	0	1	NA	0	0	0	0	0	0	0	0	1	NA	0	0	0	0	0	0	0	0	1	1	0	0	0	0	0	0	0	1	NA
Solyc00g006090.1.1	Endonuclease/exonuclease/p	0	0	0	0	0	0	0	0	0	0	1	NA	0	0	0	0	0	0	0	0	1	NA	0	0	0	0	0	0	0	0	1	NA	0	0	0	0	0	0	0	1	NA
Solyc00g006170.1.1	Zinc finger CCHC-type (AHRD	0	0	0	0	0	0	0	0	0	0	1	NA	0	0	0	0	0	0	0	0	1	NA	0	0	0	0	0	0	0	0	1	NA	0	0	0	0	0	0	0	1	NA
Solyc00g006250.1.1	Unknown Protein (AHRD V1)	0	0	0	0	0	0	0	0	0	0	1	NA	0	0	0	0	0	0	0	0	1	NA	0	0	0	0	0	0	0	0	1	NA	0	0	0	0	0	0	0	1	NA
Solyc00g006470.1.1	Unknown Protein (AHRD V1)	0	0	0.42	0	0.1	0	0	0	0	0	0.1	1	0.33	0	0	0.11	0.24	0	0	0.08	0.73	1	0	0.29	0.35	0.21	0	0	0	0	0.05	1	0.5	0.26	0	0.25	0	0	0	0.04	1
Solyc00g006480.1.1	Glucosyltransferase-12 (AHRI	0	0	0	0	0	0	0	0	0	0	1	NA	0	0	0	0	0	0	0	0	1	NA	0	0	0	0	0	0	0	0	1	NA	0	0	0	0	0	0	0	1	NA
Solyc00g006490.2.1	Serine/threonine-protein pho	13.77	11.91	13.96	13.02	13.16	10.49	10.93	11.8	12.22	11.36	0.86	1	11.65	12.77	17.18	13.87	13.89	13.45	15.74	14.36	1.04	1	14.62	14.62	16.74	15.33	14.33	16	15.72	15.35	1	1	20.23	15.67	20.81	18.9	15.08	17.16	16.12	0.85	1
Solyc00g006500.1.1	Unknown Protein (AHRD V1)	0	0	0	0	0	0	0	0	0	0	1	NA	0	0	0	0	0	0	0	0	1	NA	0	0	0	0	0	0	0	0	1	NA	0	0	0	0	0	0	0	1	NA
Solyc00g006510.1.1	Unknown Protein (AHRD V1)	0	0	0	0	0	0	0	0	0	0	1	NA	0	0	0	0	0	0	0	0	1	NA	0	0	0	0	0	0	0	0	1	NA	0	0	0	0	0	0	0	1	NA
Solyc00g006520.1.1	Unknown Protein (AHRD V1)	0	0	0	0	0	0	0	0	0	0	1	NA	0	0	0	0	0	0	0	0	1	NA	0	0	0	0	0	0	0	0	1	NA	0	0	0	0	0	0	0	1	NA
Solyc00g006530.1.1	Unknown Protein (AHRD V1)	0	0	0	0	0	0	0	0	0	0	1	NA	0	0	0	0	0	0	0	0	1	NA	0	0	0	0	0	0	0	0	1	NA	0	0	0	0	0	0	0	1	NA
Solyc00g006540.1.1	Unknown Protein (AHRD V1)	0	0.12	0	0	0.03	0.15	0	0	0	0.04	1.25	1	0	0	0	0	0	0	0.11	0.04	3.67	1	0	0	0	0	0	0	0	0	1	NA	0	0	0	0	0	0	0	1	NA
Solyc00g006550.1.1	UDP-glucosyltransferase fami	0	0	0	0	0	0	0	0	0	0	1	NA	0	0	0	0	0	0	0	0	1	NA	0	0	0	0	0	0	0	0	1	NA	0	0	0	0	0	0	0	1	NA
Solyc00g006560.1.1	Glucosyltransferase-12 (AHRI	0	0	0	0	0	0	0	0	0	0	1	NA	0	0	0	0	0	0	0	0	1	NA	0	0	0	0	0	0	0	0	1	NA	0	0	0	0	0	0	0	1	NA
Solyc00g006570.1.1	Unknown Protein (AHRD V1)	0	0	0	0	0	0	0	0	0	0	1	NA	0	0	0	0	0	0	0	0	1	NA	0	0	0	0	0	0	0	0	1	NA	0	0	0	0	0	0	0	1	NA
Solyc00g006590.1.1	Unknown Protein (AHRD V1)	0	0	0	0	0	0	0	0	0	0	1	NA	0	0	0	0	0	0	0	0	1	NA	0	0	0	0	0	0	0	0	1	NA	0	0	0	0	0	0	0	1	NA
Solyc00g006600.1.1	HAT family dimerisation dom	0.52	0.31	0.41	0.25	0.37	0	0.28	0.96	0.38	0.41	1.09	1	0.16	1.37	1.03	0.85	0.48	0.81	0.55	0.61	0.72	1	0.71	0	0.17	0.29	0.8	0.53	0	0.44	1.51	1	0.5	0.51	0.54	0.52	0.16	0.92	0.54	1.05	1
Solyc00g006630.1.1	Unknown Protein (AHRD V1)	0	0	0	0	0	0	0	0	0	0	1	NA	0	0	0	0	0	0	0	0	1	NA	0	0	0	0	0	0	0	0	1	NA	0	0	0	0	0	0	0	1	NA
Solyc00g006650.1.1	Unknown Protein (AHRD V1)	0	0	0	0	0	0	0	0	0	0	1	NA	0	0	0	0	0	0	0	0	1	NA	0	0	0	0	0	0	0	0	1	NA	0	0	0	0	0	0	0	1	NA
Solyc00g006660.1.1	Unknown Protein (AHRD V1)	0	0	0	0	0	0	0	0	0	0	1	NA	0	0	0	0	0	0	0	0	1	NA	0	0	0	0	0	0	0	0	1	NA	0	0	0	0	0	0	0	1	NA
Solyc00g006670.2.1	Unknown Protein (AHRD V1)	0	0	0	0	0	0	0	0	0	0	1	NA	0	0	0	0	0	0	0	0	1	NA	0	0	0	0	0	0	0	0	1	NA	0	0	0	0	0	0	0	1	NA
Solyc00g006680.1.1	Plant senescence-associated	0	0	0	0	0	0	0	0	0	0	1	NA	0	0	0	0	0	0	0	0	1	NA	0	0	0	0	0	0	0	0	1	NA	0	0	0	0	0	0	0	1	NA
Solyc00g006690.2.1	Unknown Protein (AHRD V1)	0	0	0	0	0	0	0	0	0	0	1	NA	0	0	0	0	0	0	0	0	1	NA	0	0	0	0	0	0	0	0	1	NA	0	0	0	0	0	0	0	1	NA
Solyc00g006700.1.1	Unknown Protein (AHRD V1)	0	0	0	0	0	0	0	0	0	0	1	NA	0	0	0	0	0	0	0	0	1	NA	0	0	0	0	0	0	0	0	1	NA	0	0	0	0	0	0	0	1	NA
Solyc00g006800.2.1	Transaldolase (AHRD V1 ***-	135.86	134.32	164.37	155.1	147.41	115.69	143.64	91.26	132.68	120.82	0.82	0.893438	129.45	116.53	118.57	121.52	126.2	123.14	113.04	120.79	0.99	1	100.51	128.75	125.58	118.28	95.38	96.12	98.78	96.76	0.82	1	106.86	99.77	88.24	98.29	152.35	150.81	151.58	1.54	0.743098
Solyc00g006810.2.1	Peptidyl-prolyl cis-trans isom	47.71	44.82	39.26	47.49	44.82	70.71	75.8	73.92	71.68	73.03	1.63	0.031572	49.04	46.59	55.47	50.37	73.54	73.5	65.73	70.92	1.41	0.227231	76.38	88.32	71.91	78.87	96.54	94.87	90.78	94.06	1.19	1	20.94	49.2	33.06	34.4	61.74	53.71	57.73	1.68	0.859839
Solyc00g006820.2.1	Peptide transporter-like prote	12.51	13.74	10.35	9.13	11.43	8.75	6.45	9.46	8.91	8.39	0.73	0.483921	7.51	5.79	5.62	6.31	2.31	2.72	2.7	2.58	0.41	0.008374	1.3	0.99	1	1.1	0.13	0.38	0.37	0.29	0.27	1	0	0.15	0.47	0.21	0.09	0.18	0.14	0.65	1
Solyc00g006830.2.1	No exine formation-1 (AHRD	8.23	8.5	9.92	10.2	9.21	7.81	7.16	7.51	8.71	7.8	0.85	0.988634	10.51	11.33	13.25	11.7	8.38	9.95	10.62	9.65	0.83	0.929856	6.89	9.45	12.35	9.56	4.93	5.97	9.81	6.9	0.72	1	7.35	5.06	6.28	6.23	6.38	10.43	8.4	1.35	0.983925
Solyc00g006840.2.1	Unknown Protein (AHRD V1)	0	0	0	0	0	0	0	0	0	0	1	NA	0	0	0	0	0	0	0	0	1	NA	0	0	0	0	0	0	0	0	1	NA	0	0	0	0	0	0	0	1	NA
Solyc00g006850.1.1	Unknown Protein (AHRD V1)	0	0	0	0	0	0	0	0	0	0	1	NA	0	0	0	0	0	0	0	0	1	NA	0	0	0	0	0	0	0	0	1	NA	0	0	0	0	0	0	0	1	NA
Solyc00g006860.1.1	Unknown Protein (AHRD V1)	0	0	0	0	0	0	0	0	0	0	1	NA	0	0	0	0	0	0	0	0	1	NA	0	0	0	0	0	0	0	0	1	NA	0	0	0	0	0	0	0	1	NA
Solyc00g006870.1.1	Unknown Protein (AHRD V1)	0	0	0	0	0	0	0	0	0	0	1	NA	0	0	0	0	0	0	0	0	1	NA	0	0	0	0	0	0	0	0	1	NA	0	0	0	0	0	0	0	1	NA
Solyc00g006880.1.1	Unknown Protein (AHRD V1)	0	0	0	0	0	0	0	0	0	0	1	NA	0	0	0	0	0	0	0	0	1	NA	0	0	0	0	0	0	0	0	1	NA	0	0	0	0	0	0	0	1	NA
Solyc00g006890.1.1	Unknown Protein (AHRD V1)	0	0	0	0	0	0	0	0	0	0	1	NA	0	0	0	0	0	0	0	0	1	NA	0	0	0	0	0	0	0	0	1	NA	0	0	0	0	0	0	0	1	NA
Solyc00g006900.1.1	Unknown Protein (AHRD V1)	1.22	1.81	2.88	0	1.48	0.91	2.57	4.49	1.75	2.43	1.64	0.90511	2.65	2.83	3.1	2.86	0.85	2.74	2.88	2.16	0.75	1	3.29	3.65	1.62	2.85	3.21	3.09	3.26	3.19	1.12	1	2.32	7.1	3.76	4.39	3.73	3.21	3.47	0.79	1
Solyc00g007000.1.1	Unknown Protein (AHRD V1)	0	0	0	0	0	0	0	0	0	0	1	NA	0	0	0	0	0	0	0	0	1	NA	0	0	0	0	0	0	0	0	1	NA	0	0	0	0	0	0	0	1	NA
Solyc00g007010.2.1	Phosphatidylinositol phospha	7.24	9.06	7.61	6.08	7.5	8.3	10.36	10.4	8.77	9.46	1.26	0.678841	7.47	5.71	7.17	6.78	4.87	7.21	6.4	6.16	0.91	1	10.7	10.79	9.88	10.46	8.99	7.03	7.26	7.76	0.74	1	4.99	6.9	6.96	6.28	6.84	7.24	7.04	1.12	1
Solyc00g007020.1.1	Tetratricopeptide repeat prot	0	0	0	0	0	0	0	0	0	0	1	NA	0	0	0	0	0	0	0	0	1	NA	0	0	0	0	0	0	0	0	1	NA	0	0	0	0	0	0	0	1	NA
Solyc00g007030.1.1	Glutathione S-transferase (AH	0	0.24	0.97	0	0.3	0	0.22	0	0	0.06	0.18	0.963658	0	0	0	0	0.19	0	0	0.06	6.33	1	0	0.22	0	0.07	0	0	0	0	0.14	1	0	0	0.42	0.14	0.25	0	0.12	0.89	1
Solyc00g007040.1.1	Glutathione-S-transferase (AH	0	0	0.33	0	0.08	0	0.22	0	0	0.06	0.67	1	0	0	0	0	0	0	0	0	1	NA	0	0	0	0	0	0	0	0	1	NA	0	0	0	0	0	0	0	1	NA
Solyc00g007050.1.1	Unknown Protein (AHRD V1)	0	0	0	0	0	0	0	0	0	0	1	NA	0	0	0	0	0	0	0	0	1	NA	0	0	0	0	0	0	0	0	1	NA	0	0	0	0	0	0	0	1	NA
Solyc00g007060.2.1	Serine/threonine kinase-like p	23.63	24.13	24.88	28.94	25.39	14.63	15.02	19.1	13.32	15.52	0.61	0.132233	20.8	27.71	29.47	25.99	13.86	12.4	18.07	14.78	0.57	0.039996	6.66	11.37	6.3	8.11	10	6.85	6.47	7.77	0.96	1	19.17	23.61	22.44	21.74	10.89	9.46	10.18	0.47	0.272348
Solyc00g007070.2.1	Heterogeneous nuclear ribon	105.3	94.16	103.38	73.95	94.2	86.34	80.97	87.67	84.18	84.79	0.9	1	101.19	106.04	86.42	97.88	84.09	87.91	83.54	85.18	0.87	1	77.23	95.34	91.92	88.16	92.56	73.21	83.99	83.25	0.94	1	145.83	137.6	142.79	142.07	110.53	119.4	114.97	0.81	1
Solyc00g007080.2.1	Unknown Protein (AHRD V1);	3.47	2.06	1.82	7.09	3.61	3	7.92	4.97	3.6	4.87	1.35	0.829736	5.02	1.01	1.3	2.44	0	1.6	3.64	1.75	0.71	1	0	0.63	0.38	0.34	0	0.58	0.56	0.38	1.13	1	0	0.28	0.59	0.29	0	0	0	0.03	1
Solyc00g007090.2.1	Multi-pass transmembrane pi	14.26	21.26	12.62	13.62	15.44	20.22	21.11	11.8	18.94	18.02	1.17	0.941902	16.25	16.43	9.34	14.01	20.53	14.4	10.93	15.29	1.09	1	8.93	13.66	8.49	10.36	5.39	10.81	10.65	8.95	0.86	1	5.07	11.4	17.55	11.34	8.49	9.07	8.78	0.77	1
Solyc00g007100.2.1	Serine/threonine protein kina	26.3	24.19	25.14	23.07	24.67	21.07	23.55	24.99	23.31	23.23	0.94	1	29.25	24.39	21.72	25.12	20.08	18.87	20.09	19.68	0.78	0.816675	11.51	11.49	9.19	10.73	8.78	10.13	8.14	9.02	0.84	1	29.87	23.69	26.89	26.82	14.66	24.73	19.7	0.73	1
Solyc00g007110.2.1	Epidermal growth factor rece	3.98	3.41	4.17	6.67	4.56	3.27	5	1.9	4.44	3.65	0.8	1	4.66	4.1	4.1	4.29	3.47	3.28	2.43	3.06	0.71	0.896625	4.04	4.32	1.9	3.42	1.84	1.79	2.25	1.96	0.57	1	1.67	0.64	0.68	1	1.75	2.58	2.17	2.17	0.868616
Solyc00g007120.2.1	Calcium-binding EF hand fam	3.95	4.14	6.01	9.32	5.86	3.61	6.73	3.8	3.66	4.45	0.76	1	5.31	4.81	7.49	5.87	3.59	3.18	3.66	3.48	0.59	0.217609	3.19	4.94	3.57	3.9	2.71	2.2	2.37	2.43	0.62	1	2.25	2.17	1.89	2.1	1.45	2.39	1.92	0.91	1
Solyc00g007130.2.1	Amino acid transporter family	0.78	0.56	0.18	0.22	0.43	0.7	1.11	1.01	0.67	0.87	2.01	0.792227	1.16	0.54	0.4	0.7	0.33	1.13	0.74	0.73	1.05	1	0.88	0.89	0.16	0.64	0.82	0.12	1.25	0.73	1.13	1	0	0	0	0	0.14	0.27	0.21	20.5	1
Solyc00g007140.1.1	Unknown Protein (AHRD V1)	0	0.54	0	0	0.14	0	0	0	0	0	0.07	1	0	0	0	0	0	0	0	0	1	NA	0	0	0	0	0	0	0	0	1	NA	0	0	0	0	0	0	0	1	NA
Solyc00g007150.2.1	Unknown Protein (AHRD V1)	7.96	4.05	6.71	9.65	7.09	7.59	1.8	2.09	8.17	4.91	0.69	1	7.41	12.85	19.21	13.16	5.52	14.13	13.41	11.02	0.84	1	3.68	2.78	9.03	5.16	2.24	2.59	2.49	2.44	0.47	1	11.32	2.48	1.75	5.18	9.37	11.97	10.67	2.06	1
Solyc00g007160.1.1	Transposase (AHRD V1 **-- Q	0	0	0	0	0	0	0	0.36	0	0.09	9	1	0.37	0	0	0.12	0	0	0.31	0.1	0.84	1	0.32	0.32	0	0.21	0	0	0	0	0.05	1	0.56	0	0	0.19	0	0	0	0.05	1
Solyc00g007170.1.1	Unknown Protein (AHRD V1)	0	0	0	0	0	0	0	0	0	0	1	NA	0	0	0	0	0	0	0	0	1	NA	0	0	0.7	0.23	0	0	0	0	0.04	NA	0	0.52	0	0.17	0.65	0	0.33	1.88	1
Solyc00g007180.1.1	Gibberellin 3-beta-hydroxylas	0	0	0	0	0	0	0	0	0	0	1	NA	0	0	0	0	0	0	0	0	1	NA	0	0	0	0	0	0	0	0	1	NA	0	0	0	0	0	0	0	1	NA
Solyc00g007190.2.1	Serine/threonine phosphatas	1.52	0.15	0.6	0.48	0.69	0	0.8	0.78	0.73	0.58	0.84	1	0.79	0.59	1.14	0.84	0.47	0.96	0.53	0.65	0.78	1	0.14	0.69	0.67	0.5	0.11	0.13	0.25	0.16	0.33	1	0.24	1.11	0.52	0.62	0.77	0.89	0.83	1.33	1
Solyc00g007200.2.1	MLO-like protein 3 (AHRD V1	0.24	0	0	0.17	0.1	0	0.09	0	0.09	0.04	0.44	1	0	0.41	0.7	0.37	0.08	0.12	0.09	0.1	0.26	0.809136	0	0.1	0.12	0.07	0.16	0	0	0.05	0.73	1	0	0.09	0	0.03	0	0	0	0.33	1
Solyc00g007210.1.1	Unknown Protein (AHRD V1)	0	0	0	0	0	0	0	0	0	0	1	NA	0	0	0	0	0	0	0	0	1	NA	0	0	0	0	0	0	0	0	1	NA	0	0	0	0	0	0	0	1	NA
Solyc00g007220.2.1	Ring finger protein (AHRD V1	4.3	6.06	10.57	8.11	7.26	7.17	4.81	3.63	3.35	4.74	0.65	0.706929	6	10.29	8.78	8.36	9.95	10.21	5.92	8.69	1.04	1	58.8	40.61	37.72	45.71	30.15	33.42	32.66	32.08	0.7	1	16.31	19.01	13.78	16.37	22.32	14.78	18.55	1.13	1
Solyc00g007230.1.1	Pol polyprotein (AHRD V1 ***	0	0	0	0	0	0	0.24	0	0	0.06	6	1	0	0	0	0	0	0	0	0	1	NA	0	0	0.3	0.1	0.4	0	0	0.13	1.33	1	0	0	0	0	0	0	0	1	NA
Solyc00g007240.1.1	Retrovirus-related Pol polypr	0	0	0	1.01	0.25	0	0	0	0	0	0.04	1	0	0	0	0	0	0	0	0	1	NA	0	0	0	0	0	0	0	0	1	NA	0	0	0	0	0	0	0	1	NA
Solyc00g007250.1.1	Pol polyprotein (AHRD V1 *-*	0	0	0	0	0	0	0	0	0	0	1	NA	0	0	0	0	0	0	0	0	1	NA	0	0	0	0	0	0	0	0	1	NA	0	0	0	0	0	0	0	1	NA
Solyc00g007260.2.1	Sentrin-specific protease 7 (A	9.73	10.83	9.12	8.7	9.59	11.14	10.28	12.5	8.32	10.56	1.1	1	7.99	7.46	6.65	7.37	5.56	8.08	8.21	7.28	0.99	1	3.47	5.13	2.56	3.72	4.14	2.82	5.11	4.02	1.08	1	5.29	4.05	1.98	3.77	4.19	5.4	4.79	1.27	1
Solyc00g007270.2.1	Translation initiation factor (	490.49	425.8	472.69	383.88	443.22	479.34	339.81	346.18	438.01	400.83	0.9	1	474.46	430.34	426.06	443.62	567.3	476.96	720.14	588.13	1.33	0.355784	167.7	209.35	161.04	179.36	192.03	193.7	204.22	196.65	1.1	1	297.79	252.04	253.2	267.68	346.39	451.45	398.92	1.49	0.764868
Solyc00g007290.1.1	Ulp1 protease family C-termi	0	0	0	0	0	0	0	0	0	0	1	NA	0	0	0	0	0	0	0	0	1	NA	0	0	0	0	0	0	0	0	1	NA	0	0	0	0	0	0	0	1	NA
Solyc00g007300.2.1	Lrr, resistance protein fragm	5.69	5.17	6.58	5.59	5.76	5.42	4.68	6.41	5.75	5.56	0.97	1	7.35	3.53	8.93	6.6	5.88	8.18	5.11	6.39	0.97	1	9.48	15.99	34.7	20.06	21.97	21.66	24.8	22.81	1.14	1	17.34	25.39	25	22.58	28.71	22.51	25.61	1.13	1
Solyc00g007310.1.1	Unknown Protein (AHRD V1)	0	0	0	0	0	0	0	0	0	0	1	NA	0	0	0	0	0	0	0	0	1	NA	0	0	0	0	0	0	0	0	1	NA	0	0	0	0	0	0	0	1	NA
Solyc00g007320.1.1	Unknown Protein (AHRD V1)	0	0	0	0	0	0	0	0	0	0	1	NA	0	0	0	0	0	0	0	0	1	NA	0	0	0	0	0	0	0	0	1	NA	0	0	0	0	0	0	0	1	NA

Solyc00g007330.1.1	Unknown Protein (AHRD V1)	0	0	0	0	0	0	0	0	0	0	1	NA	0	0	0	0	0	0	0	0	1	NA	0	0	0	0	0	0	0	0	1	NA	0	0	0	0	0	0	0	1	NA
Solyc00g007340.1.1	Unknown Protein (AHRD V1)	0	0	0	0	0	0	0	0	0	0	1	NA	0	0	0	0	0	0	0	0	1	NA	0	0	0	0	0	0	0	0	1	NA	0	0	0	0	0	0	0	1	NA
Solyc00g007350.1.1	Zinc finger CCCH domain-con	0	0	0	0	0	0	0	0	0	0	1	NA	0	0	0	0	0	0	0	0	1	NA	0	0	0	0	0	0	0	0	1	NA	0	0	0	0	0	0	0	1	NA
Solyc00g007450.1.1	Zinc finger CCCH domain-con	0	0	0	0	0	0	0	0	0	0	1	NA	0	0	0	0	0	0	0	0	1	NA	0	0	0	0	0.19	0	0	0.06	6.33	1	0	0	0	0	0	0	0	1	NA
Solyc00g007550.1.1	Unknown Protein (AHRD V1)	0	0	0	0	0	0	0	0	0	0	1	NA	0	0	0	0	0	0.92	0	0.31	30.67	1	1.44	0	0	0.48	0	1.35	0	0.45	0.94	1	0	0	2.74	0.91	0	0	0	0.01	1
Solyc00g007560.1.1	Serine/threonine-protein pho	0	0	0	0	0	0	0	0	0	0	1	NA	0	0	0	0	0.15	0.33	0.17	0.22	21.67	0.923054	0	0.17	0	0.06	0	0	0.16	0.05	0.94	1	0	0	0.33	0.11	0	0	0	0.09	1
Solyc00g007570.1.1	Polygalacturonase (AHRD V1	0	0	0	0	0	0	0	0	0	0	1	NA	0	0	0	0	0	0	0	0	1	NA	0	0	0	0	0	0	0	0	1	NA	0	0	0	0	0	0	0	1	NA
Solyc00g007580.1.1	Mutator-like transposase (AH	0	0	0	0	0	0	0	0	0	0	1	NA	0	0	0	0	0	0	0	0	1	NA	0	0	0	0	0	0	0	0	1	NA	0	0	0	0	0	0	0	1	NA
Solyc00g007590.1.1	Unknown Protein (AHRD V1)	0	0	0	0	0	0	0	0	0	0	1	NA	0	0	0	0	0	0	0	0	1	NA	0	0	0	0	0	0	0	0	1	NA	0	0	0	0	0	0	0	1	NA
Solyc00g007600.1.1	Meiotic recombination protei	1.19	0.35	0	0	0.39	0.44	0.31	0	0.57	0.33	0.86	1	0	0.69	0	0.23	0	0.2	0	0.07	0.29	1	0	0	0	0	0	0	0.29	0.1	9.67	1	0	0	0	0	0	0	0	1	NA
Solyc00g007610.1.1	Unknown Protein (AHRD V1)	0	0	0	0	0	0	0	0	0	0	1	NA	0	0	0	0	0	0	0	0	1	NA	0	0	0	0	0	0	0	0	1	NA	0	0	0	0	0	0	0	1	NA
Solyc00g007910.1.1	Unknown Protein (AHRD V1)	0	0	0	0	0	0	0	0	0	0	1	NA	0	0	0	0	0	0	0	0	1	NA	0	0	0	0	0	0	0	0	1	NA	0	0	0	0	0	0	0	1	NA
Solyc00g007920.1.1	Unknown Protein (AHRD V1)	0	0	0	0	0	0	0	0	0	0	1	NA	0	0	0	0	0	0	0	0	1	NA	0	0	0	0	0	0	0	0	1	NA	0	0	0	0	0	0	0	1	NA
Solyc00g007930.1.1	Unknown Protein (AHRD V1)	0	0	0	0	0	0	0	0	0	0	1	NA	0	0	0	0	0	0	0	0	1	NA	0	0	0	0	0	0	0	0	1	NA	0	0	0	0	0	0	0	1	NA
Solyc00g007940.1.1	Unknown Protein (AHRD V1)	0	0	0	0	0	0	0	0	0	0	1	NA	0	0	0	0	0	0	0	0	1	NA	0	0	0	0	0	0	0	0	1	NA	0	0	0	0	0	0	0	1	NA
Solyc00g007950.1.1	Unknown Protein (AHRD V1)	0	0	0	0	0	0	0	0	0	0	1	NA	0	0	0	0	0	0	0	0	1	NA	0	0	0	0	0	0	0	0	1	NA	0	0	0	0	0	0	0	1	NA
Solyc00g007960.1.1	Unknown Protein (AHRD V1)	0	0	0	0	0	0	0	0	0	0	1	NA	0	0	0	0	0	0	0	0	1	NA	0	0	0	0	0	0	0	0	1	NA	0	0	0	0	0	0	0	1	NA
Solyc00g007970.1.1	Unknown Protein (AHRD V1)	0	0	0	0	0	0	0	0	0	0	1	NA	0	0	0	0	0	0	0	0	1	NA	0	0	0	0	0	0	0	0	1	NA	0	0	0	0	0	0	0	1	NA
Solyc00g007980.1.1	Unknown Protein (AHRD V1)	0	0	0	0	0	0	0	0	0	0	1	NA	0	0	0	0	0	0	0	0	1	NA	0	0	0	0	0	0	0	0	1	NA	0	0	0	0	0	0	0	1	NA
Solyc00g007990.1.1	Unknown Protein (AHRD V1)	0	0	0	0	0	0	0	0	0	0	1	NA	0	0	0	0	0	0	0	0	1	NA	0	0	0	0	0	0	0	0	1	NA	0	0	0	0	0	0	0	1	NA
Solyc00g008000.1.1	Unknown Protein (AHRD V1)	0	0	0	0	0	0	0	0	0	0	1	NA	0	0	0	0	0	0	0	0	1	NA	0	0	0	0	0	0	0	0	1	NA	0	0	0	0	0	0	0	1	NA
Solyc00g008010.1.1	Unknown Protein (AHRD V1)	0	0	0	0	0	0	0	0	0	0	1	NA	0	0	0	0	0	0	0	0	1	NA	0	0	0	0	0	0	0	0	1	NA	0	0	0	0	0	0	0	1	NA
Solyc00g008020.1.1	Unknown Protein (AHRD V1)	0	0	0	0	0	0	0	0	0	0	1	NA	0	0	0	0	0	0	0	0	1	NA	0	0	0	0	0	0	0	0	1	NA	0	0	0	0	0	0	0	1	NA
Solyc00g008030.1.1	Unknown Protein (AHRD V1)	0	0	0	0	0	0	0	0	0	0	1	NA	0	0	0	0	0	0	0	0	1	NA	0	0	0	0	0	0	0	0	1	NA	0	0	0	0	0	0	0	1	NA
Solyc00g008040.1.1	Unknown Protein (AHRD V1)	0	0	0	0	0	0	0	0	0	0	1	NA	0	0	0	0	0	0	0	0	1	NA	0	0	0	0	0	0	0	0	1	NA	0	0	0	0	0	0	0	1	NA
Solyc00g008050.1.1	Unknown Protein (AHRD V1)	0	0	0	0	0	0	0	0	0	0	1	NA	0	0	0	0	0	0	0	0	1	NA	0	0	0	0	0	0	0	0	1	NA	0	0	0	0	0	0	0	1	NA
Solyc00g008060.1.1	Unknown Protein (AHRD V1)	0	0	0	0	0	0	0	0	0	0	1	NA	0	0	0	0	0	0	0	0	1	NA	0	0	0	0	0	0	0	0	1	NA	0	0	0	0	0	0	0	1	NA
Solyc00g008070.1.1	Unknown Protein (AHRD V1)	0	0	0	0	0	0	0	0	0	0	1	NA	0	0	0	0	0	0	0	0	1	NA	0	0	0	0	0	0	0	0	1	NA	0	0	0	0	0	0	0	1	NA
Solyc00g008080.1.1	Unknown Protein (AHRD V1)	0	0	0	0	0	0	0	0	0	0	1	NA	0	0	0	0	0	0	0	0	1	NA	0	0	0	0	0	0	0	0	1	NA	0	0	0	0	0	0	0	1	NA
Solyc00g008090.1.1	Unknown Protein (AHRD V1)	0	0	0	0	0	0	0	0	0	0	1	NA	0	0	0	0	0	0	0	0	1	NA	0	0	0	0	0	0	0	0	1	NA	0	0	0	0	0	0	0	1	NA
Solyc00g008100.1.1	Unknown Protein (AHRD V1)	0	0	0	0	0	0	0	0	0	0	1	NA	0	0	0	0	0	0	0	0	1	NA	0	0	0	0	0	0	0	0	1	NA	0	0	0	0	0	0	0	1	NA
Solyc00g008110.1.1	Unknown Protein (AHRD V1)	0	0	0	0	0	0	0	0	0	0	1	NA	0	0	0	0	0	0	0	0	1	NA	0	0	0	0	0	0	0	0	1	NA	0	0	0	0	0	0	0	1	NA
Solyc00g008120.1.1	Unknown Protein (AHRD V1)	0	0	0	0	0	0	0	0	0	0	1	NA	0	0	0	0	0	0	0	0	1	NA	0	0	0	0	0	0	0	0	1	NA	0	0	0	0	0	0	0	1	NA
Solyc00g008130.1.1	Unknown Protein (AHRD V1)	0	0	0	0	0	0	0	0	0	0	1	NA	0	0	0	0	0	0	0	0	1	NA	0	0	0	0	0	0	0	0	1	NA	0	0	0	0	0	0	0	1	NA
Solyc00g008140.1.1	Unknown Protein (AHRD V1)	0	0	0	0	0	0	0	0	0	0	1	NA	0	0	0	0	0	0	0	0	1	NA	0	0	0	0	0	0	0	0	1	NA	0	0	0	0	0	0	0	1	NA
Solyc00g008150.1.1	Unknown Protein (AHRD V1)	0	0	0	0	0	0	0	0	0	0	1	NA	0	0	0	0	0	0	0	0	1	NA	0	0	0	0	0	0	0	0	1	NA	0	0	0	0	0	0	0	1	NA
Solyc00g008160.1.1	Unknown Protein (AHRD V1)	0	0	0	0	0	0	0	0	0	0	1	NA	0	0	0	0	0	0	0	0	1	NA	0	0	0	0	0	0	0	0	1	NA	0	0	0	0	0	0	0	1	NA
Solyc00g008260.1.1	Unknown Protein (AHRD V1)	0	0	0	0	0	0	0	0	0	0	1	NA	0	0	0	0	0	0	0	0	1	NA	0	0	0	0	0	0	0	0	1	NA	0	0	0	0	0	0	0	1	NA
Solyc00g008270.1.1	Unknown Protein (AHRD V1)	0	0	0	0	0	0	0	0	0	0	1	NA	0	0	0	0	0	0	0	0	1	NA	0	0	0	0	0	0	0	0	1	NA	0	0	0	0	0	0	0	1	NA
Solyc00g008570.2.1	Unknown Protein (AHRD V1)	0	0	0	0	0	0	0	0	0	0	1	NA	0	0	0	0	0	0.92	0	0.31	30.67	1	0	0	0	0	0	0	0	0	1	NA	0	0	0	0	0	0	0	1	NA
Solyc00g008580.1.1	Unknown Protein (AHRD V1)	0	0	0	0	0	0	0	0	0	0	1	NA	0	0	0	0	0	0	0	0	1	NA	0	0	0	0	0	0	0	0	1	NA	0	0	0	0	0	0	0	1	NA
Solyc00g008680.2.1	Calcium binding protein-like (	116.04	114.63	109.57	100.26	110.12	121.78	119.2	109.22	117.14	116.84	1.06	1	100.27	93.87	111.61	101.92	95.19	101.76	106.93	101.29	0.99	1	118.97	121.75	107.97	116.23	121.12	116.22	108.45	115.26	0.99	1	96.03	117.36	101.14	104.84	122.4	101.22	111.81	1.07	1
Solyc00g008690.1.1	Unknown Protein (AHRD V1)	0	0	0	0	0	0	0	0	0	0	1	NA	0	0	0	0	0	0	0	0	1	NA	0	0	0	0	0	0	0	0	1	NA	0	0	0	0	0	0	0	1	NA
Solyc00g008800.1.1	Unknown Protein (AHRD V1)	0	0	0	0	0	0	0	0	0	0	1	NA	0	0	0	0	0	0	0	0	1	NA	0	0	0	0	0	0	0	0	1	NA	0	0	0	0	0	0	0	1	NA
Solyc00g008900.1.1	Unknown Protein (AHRD V1)	0	0	0	0	0	0	0	0	0	0	1	NA	0	0	0	0	0	0	0	0	1	NA	0	0	0	0	0	0	0	0	1	NA	0	0	0	0	0	0	0	1	NA
Solyc00g008910.1.1	Unknown Protein (AHRD V1)	0	0	0	0	0	0	0	0	0	0	1	NA	0	0	0	0	0	0	0	0	1	NA	0	0	0	0	0	0	0	0	1	NA	0	0	0	0	0	0	0	1	NA
Solyc00g008920.1.1	Unknown Protein (AHRD V1)	0	0	0	0	0	0	0	0	0	0	1	NA	0	0	0	0	0	0	0	0	1	NA	0	0	0	0	0	0	0	0	1	NA	0	0	0	0	0	0	0	1	NA
Solyc00g009020.2.1	Mitochondrial ATP synthase (	564.15	630.39	634.83	655.51	621.22	586.66	594.13	731.62	670.67	645.77	1.04	1	523.75	517.31	448.98	496.68	425.74	411.44	448.47	428.55	0.86	0.991848	436.48	428.51	349.24	404.74	367.22	383.14	421.78	390.71	0.97	1	287.95	448.19	379.49	371.88	398.88	358.24	378.56	1.02	1
Solyc00g009030.1.1	Solute carrier family 2, facilit	0	0	0	0	0	0	0	0	0	0	1	NA	0	0	0.14	0.05	0	0.09	0	0.03	0.64	1	0.13	0.14	0	0.09	0	0	0	0	0.11	1	0	0	0	0	0	0	0	1	NA
Solyc00g009040.2.1	Polyadenylate-binding protei	0.41	1.08	0.72	0.29	0.62	0.56	0.72	0.19	0.22	0.42	0.68	0.928144	0	0	0.26	0.09	0.07	0.21	0	0.09	1.08	1	0.08	0	0	0.03	0.07	0	0	0.02	0.88	1	0	0	0.16	0.05	0	0	0	0.19	1
Solyc00g009050.1.1	UPF0544 protein C5orf45 (AH	0.9	0.97	0.85	0.51	0.81	0.4	0.86	1	0.91	0.79	0.98	1	0.67	0.94	0.61	0.74	0.75	1.22	1.42	1.13	1.53	0.985959	0	0.29	0	0.1	0	0	0.53	0.18	1.83	1	0.51	0	0	0.17	0.5	0	0.25	1.47	1
Solyc00g009060.2.1	Hypoxanthine phosphoribosy	40.9	49.03	24.13	32.66	36.68	45.83	46.35	52.47	44.76	47.35	1.29	0.599955	38.35	51.9	30.37	40.21	42.1	45.26	74.14	53.83	1.34	0.813994	43.44	60.48	31.1	45.01	33.59	49	56.25	46.28	1.03	1	26.73	51.27	30.94	36.31	27.78	33.55	30.66	0.84	1
Solyc00g009070.1.1	Unknown Protein (AHRD V1)	0	0	0	0	0	0	0	0	0	0	1	NA	0	0	0	0	0	0	0	0	1	NA	0	0	0	0	0	0	0	0	1	NA	0	0	0	0	0	0	0	1	NA
Solyc00g009080.2.1	Unknown Protein (AHRD V1)	74.73	92.49	43.39	29.73	60.09	99.94	116.61	100.91	95.14	103.15	1.72	0.095375	83.09	63.94	32.54	59.86	58.71	84.03	100.94	81.23	1.36	0.771546	57.54	88.96	28.97	58.49	53.69	51.77	87.21	64.22	1.1	1	26.56	44.1	30.52	33.73	37.94	30.71	34.33	1.02	1
Solyc00g009090.2.1	Receptor like kinase, RLK	0	0	0	0	0	0	0	0	0	0	1	NA	0	0	0	0	0	0	0	0	1	NA	0	0	0	0	0	0	0	0	1	NA	0	0	0	0	0	0	0	1	NA
Solyc00g009100.2.1	30S ribosomal protein S1-like	15.4	13.82	10.15	12.35	12.93	21.12	19.05	19	21.1	20.07	1.55	0.114983	12.89	11.83	10.73	11.82	15.04	18.41	17.95	17.13	1.45	0.229832	12.96	13.49	10.57	12.34	11.38	9.83	14.5	11.9	0.96	1	3.65	12.49	6.91	7.68	13.04	8.89	10.96	1.43	1
Solyc00g009110.2.1	Inositol-1 4 5-trisphosphate 5	65.36	58.35	126.1	32.58	70.6	57.16	28.15	106.91	82.68	68.72	0.97	1	76.59	29.95	38.91	48.48	29.57	28.93	30.81	29.77	0.61	0.837708	5.88	7.33	5.31	6.17	7.17	4.98	3.9	5.35	0.87	1	8.91	16.22	13.83	12.99	8.14	8.5	8.32	0.64	0.774482
Solyc00g009120.2.1	Short-chain dehydrogenase/r	90.52	89.57	183.9	44.27	102.06	96.87	33.91	106.08	100.23	84.27	0.83	1	106.18	42.94	45.61	64.91	22.56	25.74	23.63	23.98	0.37	0.444498	42.39	27	25.89	31.76	27.41	20.7	12.97	20.36	0.64	1	10.47	26.97	21.23	19.56	31.46	21.52	26.49	1.35	1
Solyc00g009130.2.1	Dehydrogenase/reductase SD	60.34	115.28	134.78	47.8	89.55	126.1	80.03	116.13	107.43	107.42	1.2	0.949671	92.8	55.95	44.17	64.31	41.85	39.99	48.05	43.3	0.67	0.811508	222.29	117.35	105.41	148.35	92.49	111.59	91.46	98.51	0.66	1	11.44	33.31	17.32	20.69	114.15	67.01	90.58	4.38	0.136675
Solyc00g009140.2.1	Short-chain dehydrogenase/r	92.97	98.04	147.7	75.52	103.56	158.6	101.26	136.83	134.6	132.82	1.28	0.533503	90.19	62.95	54.42	69.19	49.8	44.64	50.88	48.44	0.7	0.592296	288.06	182.28	142.54	204.29	128.81	146.05	137.29	137.38	0.67	1	20.5	76.44	41.99	46.31	160.67	83.94	122.3	2.64	0.78425
Solyc00g009240.1.1	Unknown Protein (AHRD V1)	0	0	0	0	0	0	0	0	0	0	1	NA	0	0	0	0	0	0	0	0	1	NA	0	0	0	0	0	0	0	0	1	NA	0	0	0	0	0	0	0	1	NA
Solyc00g009440.1.1	Unknown Protein (AHRD V1)	0	0	0	0	0	0	0	0	0	0	1	NA	0	0	0	0	0	0	0	0	1	NA	0	0	0	0	0	0	0	0	1	NA	0	0	0	0	0	0	0	1	NA
Solyc00g009540.1.1	Helicase-like protein (AHRD V	0	0	0	0	0	0	0	0	0	0	1	NA	0	0	0	0	0	0	0	0	1	NA	0	0	0	0	0	0	0	0	1	NA	0	0	0	0	0	0	0	1	NA
Solyc00g009650.1.1	Unknown Protein (AHRD V1)	0	0	0	0	0	0	0	0	0	0	1	NA	0	0	0	0	0	0	0	0	1	NA	0	0	0	0	0	0	0	0	1	NA	0	0	0	0	0	0	0	1	NA
Solyc00g009660.1.1	Unknown Protein (AHRD V1)	0	0	0	0	0	0	0	0	0	0	1	NA	0	0	0	0	0	0	0	0	1	NA	0	0	0	0	0	0	0	0	1	NA	0	0	0	0	0	0	0	1	NA
Solyc00g009760.2.1	Cytochrome P450 monooxyge	0	0	0	0	0	0	0	0	0	0	1	NA	0	0	0	0	0	0	0	0	1	NA	0	0	0	0	0	0	0	0	1	NA	0	0	0	0	0	0	0	1	NA
Solyc00g009770.1.1	Unknown Protein (AHRD V1)	0	0	0	0	0	0	0	0	0	0	1	NA	0	0	0	0	0	0	0	0	1	NA	0	0	0	0	0	0	0	0	1	NA	0	0	0	0	0	0	0	1	NA
Solyc00g009780.1.1	Unknown Protein (AHRD V1)	0	0	0	0	0	0	0	0	0	0	1	NA	0	0	0	0	0	0	0	0	1	NA	0	0	0	0	0	0	0	0	1	NA	0	0	0	0	0	0	0	1	NA
Solyc00g009790.1.1	Unknown Protein (AHRD V1)	0	0	0	0	0	0	0	0	0	0	1	NA	0	0	0	0	0	0	0	0	1	NA	0	0	0	0	0	0	0	0	1	NA	0	0	0	0	0	0	0	1	NA
Solyc00g009800.1.1	Unknown Protein (AHRD V1)	0	0	0	0	0	0	0	0	0	0	1	NA	0	0	0	0	0	0	0	0	1	NA	0	0	0	0	0	0	0	0	1	NA	0	0	0	0	0	0	0	1	NA
Solyc00g010100.1.1	Unknown Protein (AHRD V1)	0	0	0	0	0	0	0	0	0	0	1	NA	0	0	0	0	0	0	0	0	1	NA	0	0	0	0	0	0	0	0	1	NA	0	0	0	0	0	0	0	1	NA
Solyc00g010110.1.1	Unknown Protein (AHRD V1)	0	0	0	0	0	0	0	0	0	0	1	NA	0	0	0	0	0	0	0	0	1	NA	0	0	0	0	0	0	0	0	1	NA	0	0	0	0	0	0	0	1	NA
Solyc00g010210.1.1	Polyprotein (AHRD V1 ***- Q	0	0	0	0	0	0	0	0	0	0	1	NA	0	0	0	0	0	0	0	0	1	NA	0	0	0	0	0	0	0	0	1	NA	0	0	0	0	0	0	0	1	NA
Solyc00g010410.1.1	Unknown Protein (AHRD V1)	0	0	0	0	0	0	0	0	0	0	1	NA	0	0	0	0	0	0	0	0	1	NA	0	0	0	0	0	0	0	0	1	NA	0	0	0	0	0	0	0	1	NA
Solyc00g010420.1.1	Unknown Protein (AHRD V1)	0	0	0	0	0	0	0	0	0	0	1	NA	0	0	0	0	0	0	0	0	1	NA	0	0	0	0	0	0	0	0	1	NA	0	0	0	0	0	0	0	1	NA
Solyc00g010520.1.1	Unknown Protein (AHRD V1)	0	0	0	0	0	0	0	0	0	0	1	NA	0	0	0	0	0	0	0	0	1	NA	0	0	0	0	0	0	0	0	1	NA	0	0	0	0	0	0	0	1	NA
Solyc00g010530.1.1	Unknown Protein (AHRD V1)	0	0	0	0	0	0	0	0	0	0	1	NA	0	0	0	0	0	0	0	0	1	NA	0	0	0	0	0	0	0	0	1	NA	0	0	0	0	0	0	0	1	NA
Solyc00g010540.1.1	Pol polyprotein (AHRD V1 ***	0	0	0	0.27	0.07	0	0	0	0	0	0.15	1	0	0	0	0	0	0	0	0	1	NA	0	0	0.38	0.13	0.25	0	0	0.08	0.66	1	0	0	0	0	0	0.34	0.17	17	1
Solyc00g010550.1.1	Unknown Protein (AHRD V1)	0	0	0	0	0	0	0	0	0	0	1	NA	0	0	0	0	0	0	0	0	1	NA	0	0	0	0	0	0	0	0	1	NA	0	0	0	0	0	0	0	1	NA
Solyc00g010750.1.1	Guanylate binding protein 2 (	1.49	0.79	1.75	1.68	1.43	0.33	0.47	0.27	0.64	0.43	0.3	0.568246	0.83	1.55	2.01	1.46	1.03	0.77	1.17	0.99	0.68	1	1.2	0.48	1.77	1.15	0.39	1.13	0.43	0.65	0.57	1	3.38	1.08	0.91	1.79	1.9	2.61	2.25	1.26	1
Solyc00g010760.1.1	Helicase-like protein (AHRD V	0	0	0	0	0	0	0	0	0	0	1	NA	0	0	0	0	0	0	0	0	1	NA	0	0	0	0	0	0	0	0	1	NA	0	0	0	0	0	0	0	1	NA
Solyc00g010770.1.1	Unknown Protein (AHRD V1)	0	0	0	0.39	0.1	0	0	0	0.2	0.05	0.51	1	0.26	0.48	0	0.25	0	0.43	0	0.14	0.58	1	0	0	0	0	0	0	0.41	0.14	13.67	1	0	0.81	0.86	0.56	0	0	0	0.02	1
Solyc00g010780.1.1	Unknown Protein (AHRD V1)	0	0	0	0	0	0	0	0	0	0	1	NA	0	0	0	0	0	0	0	0	1	NA	0	0	0	0	0	0	0	0	1	NA	0	0	0	0	0	0	0	1	NA
Solyc00g010790.1.1	Unknown Protein (AHRD V1)	0	0	0	0	0	0	0	0	0	0	1	NA	0	0	0	0	0	0	0	0	1	NA	0	0	0	0	0	0	0	0	1	NA	0	0	0	0	0	0	0	1	NA
Solyc00g010810.1.1	Unknown Protein (AHRD V1)	0	0	0	0	0	0	0	0	0	0	1	NA	0	0	0	0	0	0	0	0	1	NA	0	0	0	0	0	0	0	0	1	NA	0	0	0	0	0	0	0	1	NA
Solyc00g010930.1.1	Unknown Protein (AHRD V1)	0	0	0	0	0	0	0	0	0	0	1	NA	0	0	0	0	0	0	0	0	1	NA	0	0	0	0	0	0	0	0	1	NA	0	0	0	0	0	0	0	1	NA
Solyc00g011130.1.1	Unknown Protein (AHRD V1)	0	0	0	0	0	0	0	0	0	0	1	NA	0	0	0	0	0	0	0	0	1	NA	0	0	0	0	0	0	0	0	1	NA	0	0	0	0	0	0	0	1	NA
Solyc00g011140.1.1	Unknown Protein (AHRD V1)	0	0	0	0	0	0	0	0	0	0	1	NA	0	0	0	0	0	0	0	0	1	NA	0	0	0	0	0	0	0	0	1	NA	0	0	0	0	0	0	0	1	NA
Solyc00g011150.1.1	Unknown Protein (AHRD V1)	0	0	0	0	0	0	0	0	0	0	1	NA	0	0	0	0	0	0	0	0	1	NA	0	0	0	0	0	0	0	0	1	NA	0	0	0	0	0	0	0	1	NA
Solyc00g011160.1.1	Unknown Protein (AHRD V1)	0	0	0	0	0	0	0	0	0	0	1	NA	0	0	0	0	0	0	0	0	1	NA	0	0	0	0	0	0	0	0	1	NA	0	0	0	0	0	0	0	1	NA
Solyc00g011170.1.1	Unknown Protein (AHRD V1)	0	0	0	0	0	0	0	0	0	0	1	NA	0	0	0	0	0	0	0	0	1	NA	0	0	0	0	0	0	0	0	1	NA	0	0	0	0	0	0	0	1	NA
Solyc00g011180.1.1	Unknown Protein (AHRD V1)	0	0	0	0	0	0	0	0	0	0	1	NA	0	0	0	0	0	0	0	0	1	NA	0	0	0	0	0	0	0	0	1	NA	0	0	0	0	0	0	0	1	NA
Solyc00g011310.1.1	Unknown Protein (AHRD V1)	0	0	0	0	0	0	0	0	0	0	1	NA	0	0	0	0	0	0	0	0	1	NA	0	0	0	0	0	0	0	0	1	NA	0	0	0	0	0	0	0	1	NA
Solyc00g011550.1.1	B3 domain-containing protei	0	0	0	0	0	0	0	0	0	0	1	NA	0	0	0	0	0	0	0	0	1	NA	0	0	0	0	0	0	0	0	1	NA	0	0	0	0	0	0	0	1	NA
Solyc00g011560.1.1	E3 ubiquitin-protein ligase SI	0.35	0	0	0	0.09	0	0	0	0	0	0.11	1	0.33	0	0.3	0.21	0	0.18	0.28	0.15	0.73	1	0	0	0	0	0	0	0	0	1	1	1.01	0	0	0.34	0	0	0	0.03	1
Solyc00g011660.1.1	B3 domain-containing protei	0	0	0	0	0	0	0	0	0	0	1	NA	0	0	0	0	0	0	0	0	1	NA	0	0	0	0	0	0	0	0	1	NA	0	0	0	0	0	0	0	1	NA
Solyc00g011670.1.1	Unknown Protein (AHRD V1)	0	0	0	0	0	0	0	0	0	0	1	NA	0	0	0	0	0	0	0	0	1	NA	0	0	0	0	0	0	0	0	1	NA	0	0	0	0	0	0	0	1	NA
Solyc00g011870.1.1	Phosphatase 1G (AHRD V1 **	0	0	0	0	0	0	0	0	0	0	1	NA	0	0	0	0	0	0	0	0	1	NA	0	0.44	0	0.15	0	0	0	0	0.07	1	0	0	0	0	0	0	0	1	NA
Solyc00g011880.2.1	AIG2-like protein (AHRD V1 *	19.75	30.67	17.85	14.26	20.63	28.02	31.18	33.23	34.09	31.63	1.53	0.281866	26.19	23.38	14.91	21.49	25.36	31.98	28.4	28.58	1.33	0.567763	25.12	36.29	19.6	27	24.57	25.8	38.28	29.55	1.09	1	2.39	3.97	8.4	4.92	11.92	9.21	10.57	2.15	0.715266
Solyc00g011890.2.1	Galactokinase like protein (AH	26.34	29.33	32.2	25.65	28.38	26.45	21.4	20.41	23.38	22.91	0.81	0.734913	33.43	44.52	42.34	40.1	35.78	35.8	33.7	35.09	0.88	1	90.66	77.29	103.24	90.4	61.06	63.84	66.43	63.78	0.71	1	57.35	71.85	62.9	64.03	38.9	33.58	36.24	0.57	0.40715
Solyc00g012090.1.1	Unknown Protein (AHRD V1)	0	0	0	0	0	0	0	0	0	0	1	NA	0	0	0	0	0	0	0	0	1	NA	0	0	0	0	0	0	0	0	1	NA	0	0	0	0	0	0	0	1	NA
Solyc00g012310.1.1	Retrovirus-related Pol polypr	0	0	0	0	0	0	0	0	0	0	1	NA	0	0	0	0	0	0	0	0	1	NA	0	0	0	0	0	0	0	0	1	NA	0	0	0	0	0	0	0	1	NA
Solyc00g012430.1.1	Unknown Protein (AHRD V1)	0	0	0	0	0	0	0	0	0	0	1	NA	0	0	0	0	0	0	0	0	1	NA	0	0	0	0	0	0	0	0	1	NA	0	0	0	0	0	0	0	1	NA
Solyc00g012440.1.1	TO54-2 (Fragment) (AHRD V1	0	0	0	0	0	0	0	0	0	0	1	NA	0	0	0	0	0	0	0	0	1	NA	0	0	0	0	0	0	0	0	1	NA	0	0	0	0	0	0	0	1	NA
Solyc00g012540.1.1	Zinc finger CCCH domain-con	0	0	0	0	0	0	0	0	0	0	1	NA	0	0	0	0	0	0	0	0	1	NA	0	0	0	0	0	0	0	0	1	NA	0	0	0	0	0	0	0	1	NA
Solyc00g012640.1.1	DNA (cytosine-5)-methyltran	0	0	0	0	0	0	0	0	0	0	1	NA	0	0	0	0	0	0	0	0	1	NA	0	0	0	0	0	0	0	0	1	NA	0	0	0	0	0	0	0	1	NA

Solyc00g012650.1.1	Unknown Protein (AHRD V1)	0	0	0	0	0	0	0	0	0	0	1	NA	0	0	0	0	0	0	0	0	1	NA	0	0	0	0	0	0	0	0	1	NA	0	0	0	0	0	0	0	1	NA
Solyc00g012660.1.1	F-box domain containing pro	0	0	0	0	0	0	0	0	0	0	1	NA	0	0	0	0	0	0	0	0	1	NA	0	0	0	0	0	0	0	0	1	NA	0	0	0	0	0	0	0	1	NA
Solyc00g012670.1.1	Unknown Protein (AHRD V1)	0	0	0	0	0	0	0	0	0	0	1	NA	0	0	0	0	0	0	0	0	1	NA	0	0	0	0	0	0	0	0	1	NA	0	0	0	0	0	0	0	1	NA
Solyc00g012680.1.1	D111/G-patch Zinc finger C2H	0	0	0	0	0	0	0	0	0	0	1	NA	0	0	0	0	0	0	0	0	1	NA	0	0	0	0	0	0	0	0	1	NA	0	0	0	0	0	0	0	1	NA
Solyc00g012690.1.1	Ovarian tumour otubain (AHR	0	0	0	0	0	0	0	0	0	0	1	NA	0	0	0	0	0	0	0	0	1	NA	0	0	0	0	0	0	0	0	1	NA	0	0	0	0	0	0	0	1	NA
Solyc00g012700.1.1	Ovarian tumour otubain (AHR	0	0	0	0	0	0	0	0	0	0	1	NA	0	0	0	0	0	0	0	0	1	NA	0	0	0	0	0	0	0	0	1	NA	0	0	0	0	0	0	0	1	NA
Solyc00g012800.1.1	Unknown Protein (AHRD V1)	0	0	0	0	0	0	0	0	0	0	1	NA	0	0	0	0	0	0	0	0	1	NA	0	0	0	0	0	0	0	0	1	NA	0	0	0	0	0	0	0	1	NA
Solyc00g012900.1.1	Unknown Protein (AHRD V1)	0	0	0	0	0	0	0	0	0	0	1	NA	0	0	0	0	0	0	0	0	1	NA	0	0	0	0	0	0	0	0	1	NA	0	0	0	0	0	0	0	1	NA
Solyc00g013000.1.1	Unknown Protein (AHRD V1)	0	0	0	0	0	0	0	0	0	0	1	NA	0	0	0	0	0	0	0	0	1	NA	0	0	0	0	0	0	0	0	1	NA	0	0	0	0	0	0	0	1	NA
Solyc00g013100.1.1	Unknown Protein (AHRD V1)	0	0	0	0	0	0	0	0	0	0	1	NA	0	0.73	0.71	0.48	0	0	0	0	0.02	1	0	0	0	0	0	0	0	0	1	NA	0	0	0	0	0	0	0	1	NA
Solyc00g013110.1.1	Unknown Protein (AHRD V1)	0	0	0	0	0	0	0	0	0	0	1	NA	0	0	0	0	0	0	0	0	1	NA	0	0	0	0	0	0	0	0	1	NA	0	0	0	0	0	0	0	1	NA
Solyc00g013120.2.1	Uncharacterized mitochondri	1.41	3.77	5	3.33	3.38	1.57	0.37	0.87	0.68	0.87	0.26	0.2123	0.88	2.05	4.78	2.57	1.96	1.46	1.11	1.51	0.59	0.987071	0.76	2.3	2.81	1.96	1.86	1.07	1.03	1.32	0.67	1	4.02	1.71	0.72	2.15	2.16	2.07	2.12	0.98	1
Solyc00g013130.2.1	NADH-quinone oxidoreductas	2.85	3.32	14.74	14.88	8.95	4.63	2.42	2.42	2.52	3	0.34	0.542618	4.69	4.76	22.03	10.49	5.02	2.38	1.72	3.04	0.29	0.754473	3.36	8.39	15.01	8.92	4.03	4.15	3.99	4.06	0.45	1	3.74	3.02	0.34	2.37	4.41	2.88	3.65	1.54	1
Solyc00g013140.1.1	NADH dehydrogenase subuni	0.43	0.51	2.36	2.02	1.33	1.75	0.23	0.13	0.82	0.73	0.55	0.944449	0.66	0.37	1.09	0.71	0.79	1.18	0.22	0.73	1.03	1	1.15	0.93	1.42	1.17	1.31	0.97	0.31	0.86	0.74	1	0.81	1.14	0.88	0.94	0.78	0	0.39	0.41	1
Solyc00g013150.1.1	Unknown Protein (AHRD V1)	0	0	0	0	0	0	0	0	0	0	1	NA	0	0	0	0	0	0	0	0	1	NA	0	0	0	0	0.44	0.51	0	0.32	31.67	1	0	0	0	0	0	0	0	1	NA
Solyc00g013160.1.1	Unknown Protein (AHRD V1)	0	0.69	1.82	1.64	1.04	1.29	0.3	0.35	0	0.48	0.47	0.939233	1.08	1.01	1.3	1.13	0.8	0.4	0	0.4	0.35	0.912327	1.87	2.2	1.53	1.87	0.76	0.29	0.56	0.54	0.29	1	1.1	0.28	0	0.46	0	1.35	0.68	1.47	1
Solyc00g013170.1.1	Unknown Protein (AHRD V1)	0	0	0	0	0	0	0	0	0	0	1	NA	0	0	0	0	0	0	0	0	1	NA	0	0	0	0	0	0	0	0	1	NA	0	0	0	0	0	0	0	1	NA
Solyc00g013180.1.1	NADH-ubiquinone oxidoredu	0	0	0	0	0	0.41	0.29	1.37	0.54	0.65	65.25	0.513478	0.69	0	0	0.23	0.26	0.58	0.88	0.57	2.49	1	0.3	0	0	0.1	0.24	0.28	0.27	0.26	2.63	1	0	0.54	0	0.18	0.68	0.33	0.51	2.81	1
Solyc00g013190.1.1	Unknown Protein (AHRD V1)	0	0	0	0	0	0.72	0	0	0.46	0.29	29.5	1	0	0	0	0	0	0	0	0	1	NA	0	0.53	0.64	0.39	0.42	0	0	0.14	0.36	1	0.92	0.47	0	0.46	0	0	0	0.02	1
Solyc00g013320.1.1	Unknown Protein (AHRD V1)	0	0	0	0	0	0	0	0	0	0	1	NA	0	0	0	0	0	0	0	0	1	NA	0	0	0	0	0	0	0	0	1	NA	0	0	0	0	0	0	0	1	NA
Solyc00g013330.1.1	Unknown Protein (AHRD V1)	0	0	0	0	0	0	0	0	0	0	1	NA	0	0	0	0	0	0	0	0	1	NA	0	0	0	0	0	0	0	0	1	NA	0	0	0	0	0	0	0	1	NA
Solyc00g013770.1.1	Unknown Protein (AHRD V1)	0	0	0	0	0	0	0	0	0	0	1	NA	0	0	0	0	0	0	0	0	1	NA	0	0	0	0	0	0	0	0	1	NA	0	0	0	0	0	0	0	1	NA
Solyc00g014170.1.1	Unknown Protein (AHRD V1)	0	0	0	0	0	0	0	0	0	0	1	NA	0	0	0	0	0	0	0	0	1	NA	0	0	0	0	0	0	0	0	1	NA	0	0	0	0	0	0	0	1	NA
Solyc00g014180.1.1	Unknown Protein (AHRD V1)	0	0	0	0	0	0	0	0	0	0	1	NA	0	0	0	0	0	0	0	0	1	NA	0	0	0	0	0	0	0	0	1	NA	0	0	0	0	0	0	0	1	NA
Solyc00g014190.1.1	Unknown Protein (AHRD V1)	0	0	0	0	0	0	0	0	0	0	1	NA	0	0	0	0	0	0	0	0	1	NA	0	0	0	0	0	0	0	0	1	NA	0	0	0	0	0	0	0	1	NA
Solyc00g014290.1.1	Unknown Protein (AHRD V1)	0	0	0	0	0	0	0	0	0	0	1	NA	0	0	0	0	0	0	0	0	1	NA	0	0	0	0	0	0	0	0	1	NA	0	0	0	0	0	0	0	1	NA
Solyc00g014390.1.1	F-box family protein (AHRD V	0	0	0	0	0	0	0	0	0	0	1	NA	0	0	0	0	0	0	0	0	1	NA	0	0	0	0	0	0	0	0	1	NA	0	0	0	0	0	0	0	1	NA
Solyc00g014790.2.1	Unknown Protein (AHRD V1)	1.83	0.54	1.44	0.86	1.17	0	0	0	0.44	0.11	0.09	0.638915	1.7	0	2.06	1.25	0.42	0.32	0	0.25	0.2	0.855973	0.99	1.49	1.21	1.23	0	0.46	0.45	0.3	0.25	1	3.47	1.77	2.82	2.69	0.56	3.21	1.89	0.7	1
Solyc00g014800.1.1	Zinc-finger protein 1 (AHRD V	0	1.07	0.47	0	0.39	0	0.32	0	0	0.08	0.21	0.966465	0.75	0	0.34	0.36	0	0	0.32	0.11	0.29	1	0.32	0.33	0	0.22	0.26	0.61	0.29	0.39	1.78	1	0	0	0	0	0	0	0	1	NA
Solyc00g014810.1.1	Unknown Protein (AHRD V1)	0	0	0	0	0	0	0	0	0	0	1	NA	0	0	0	0	0	0	0	0	1	NA	0	0	0	0	0	0	0	0	1	NA	0	0	0	0	0	0	0	1	NA
Solyc00g014820.2.1	Unknown Protein (AHRD V1)	0.38	0	0	1.09	0.37	0	0	0.35	0	0.09	0.24	1	0.36	0	0.65	0.34	0	0	0	0	0.03	0.962977	0	0	0.38	0.13	0	0	0	0	0.08	1	0.55	0	0	0.18	0	0	0	0.05	1
Solyc00g014830.2.1	NADH-quinone oxidoreductas	3.34	3.12	5.12	5.67	4.31	2.6	1.85	2	2.76	2.3	0.53	0.384736	3.11	3.77	6.77	4.55	3.48	1.64	1.97	2.36	0.52	0.515868	2.02	4.76	5.97	4.25	3.51	3.92	2.43	3.29	0.77	1	4.75	3.76	5.65	4.72	3.98	3.81	3.9	0.83	1
Solyc00g014840.1.1	Unknown Protein (AHRD V1)	0	0	0	0	0	0	0	0	0	0	1	NA	0	0	0	0	0	0	0	0	1	NA	0	0	0	0	0	0	0	0	1	NA	0	0	0	0	0	0	0	1	NA
Solyc00g014850.1.1	ORF86 (AHRD V1 ***- B6VJY4	0	0	0	0	0	0.71	0	0.58	0.46	0.44	43.75	0.995762	0	0	0.54	0.18	0.44	0	0	0.15	0.81	1	0.51	0	0	0.17	0.83	0.96	0	0.6	3.51	1	0.9	1.38	1.95	1.41	0	1.67	0.83	0.59	1
Solyc00g014860.1.1	Unknown Protein (AHRD V1)	0	0	0	0	0	0	0	0	0	0	1	NA	0	0	0	0	0	0	0	0	1	NA	0	0	0	0	0	0	0	0	1	NA	0	0	0	0	0	0	0	1	NA
Solyc00g014960.1.1	F-box family protein (AHRD V	0	0	0	0	0	0	0	0	0	0	1	NA	0	0	0	0	0	0	0	0	1	NA	0	0	0	0	0	0	0	0	1	NA	0	0	0	0	0	0	0	1	NA
Solyc00g014970.1.1	Helicase-like protein (AHRD V	0	0	0	0	0	0	0	0	0	0	1	NA	0	0	0	0	0	0	0	0	1	NA	0	0	0	0	0	0	0	0	1	NA	0	0	0	0	0	0	0	1	NA
Solyc00g014980.1.1	Dihydroflavonol-4-reductase (	0	0	0	0	0	0	0	0	0	0	1	NA	0	0	0	0	0	0	0	0	1	NA	0	0	0	0	0	0	0	0	1	NA	0	0	0	0	0	0	0	1	NA
Solyc00g014990.1.1	Unknown Protein (AHRD V1);	0	0	0	0	0	0	0	0	0	0	1	NA	0	0	0	0	0	0	0	0	1	NA	0	0	0	0	0	0	0	0	1	NA	0	0	0	0	0	0	0	1	NA
Solyc00g015000.1.1	DNA (Cytosine-5-)-methyltran	0	0	0	0	0	0	0	0	0	0	1	NA	0	0	0	0	0	0	0	0	1	NA	0	0	0	0	0	0	0	0	1	NA	0	0	0	0	0	0	0	1	NA
Solyc00g015600.1.1	Unknown Protein (AHRD V1)	0	0	0	0	0	0	0	0	0	0	1	NA	0	0	0	0	0	0	0	0	1	NA	0	0	0	0	0	0	0	0	1	NA	0	0	0	0	0	0	0	1	NA
Solyc00g015610.1.1	Unknown Protein (AHRD V1)	0	0	0	0	0	0	0	0	0	0	1	NA	0	0	0	0	0	0	0	0	1	NA	0	0	0	0	0	0	0	0	1	NA	0	0	0	0	0	0	0	1	NA
Solyc00g015720.1.1	Glycosyltransferase-like prote	0	0	0	0	0	0	0	0	0	0	1	NA	0	0	0	0	0	0	0	0	1	NA	0	0	0	0	0	0	0	0	1	NA	0	0	0	0	0	0	0	1	NA
Solyc00g015730.1.1	RAMOSA1 C2H2 zinc-finger tr	0	0	0	0	0	0	0	0	0	0	1	NA	0	0	0	0	0	0	0	0	1	NA	0	0	0	0	0	0	0	0	1	NA	0	0	0	0	0	0	0	1	NA
Solyc00g015740.1.1	Reductase 2 (AHRD V1 ***- C	0	0	0	0	0	0	0	0	0	0	1	NA	0	0	0	0	0	0	0	0	1	NA	0	0	0	0	0	0	0	0	1	NA	0	0	0	0	0	0	0	1	NA
Solyc00g015750.1.1	Reductase 2 (AHRD V1 ***- C	0	0	0	0	0	0	0	0	0	0	1	NA	0	0	0	0	0	0	0	0	1	NA	0	0	0	0	0	0	0	0	1	NA	0	0	0	0	0	0	0	1	NA
Solyc00g015760.1.1	CHP-rich zinc finger protein-li	0	0	0	0	0	0	0	0	0	0	1	NA	0	0	0	0	0	0	0	0	1	NA	0	0	0	0	0	0	0	0	1	NA	0	0	0	0	0	0	0	1	NA
Solyc00g015770.1.1	Aldo-keto reductase (AHRD V	0	0	0	0	0	0	0	0	0	0	1	NA	0	0	0	0	0	0	0	0	1	NA	0	0	0	0	0	0	0	0	1	NA	0	0	0	0	0	0	0	1	NA
Solyc00g016470.1.1	Unknown Protein (AHRD V1)	0	0	0	0	0	0	0	0	0	0	1	NA	0	0	0	0	0	0	0	0	1	NA	0	0	0	0	0	0	0	0	1	NA	0	0	0	0	0	0	0	1	NA
Solyc00g016480.1.1	HAT family dimerisation dom	0	0	0	0	0	0	0	0	0	0	1	NA	0	0	0	0	0	0	0	0	1	NA	0	0	0	0	0	0	0	0	1	NA	0	0	0	0	0	0	0	1	NA
Solyc00g016490.1.1	F-box family protein (AHRD V	0	0	0	0	0	0	0	0	0	0	1	NA	0	0	0	0	0	0	0	0	1	NA	0	0	0	0	0	0	0	0	1	NA	0	0	0	0	0	0	0	1	NA
Solyc00g016590.1.1	Unknown Protein (AHRD V1)	0	0	0	0	0	0	0	0	0	0	1	NA	0	0	0	0	0	0	0	0	1	NA	0	0	0	0	0	0	0	0	1	NA	0	0	0	0	0	0	0	1	NA
Solyc00g016790.1.1	Unknown Protein (AHRD V1)	0	0	0	0	0	0	0	0	0	0	1	NA	0	0	0	0	0	0	0	0	1	NA	0	0	0	0	0	0	0	0	1	NA	0	0	0	0	0	0	0	1	NA
Solyc00g016890.1.1	Unknown Protein (AHRD V1)	0	0	0	0	0	0	0	0	0	0	1	NA	0	0	0	0	0	0	0	0	1	NA	0	0	0	0	0	0	0	0	1	NA	0	0	0	0	0	0	0	1	NA
Solyc00g016900.1.1	Unknown Protein (AHRD V1)	0	0	0	0	0	0	0	0	0	0	1	NA	0	0	0	0	0	0	0	0	1	NA	0	0	0	0	0	0	0	0	1	NA	0	0	0	0	0	0	0	1	NA
Solyc00g016910.1.1	Unknown Protein (AHRD V1)	0	0	0	0	0	0	0	0	0	0	1	NA	0	0	0	0	0	0	0	0	1	NA	0	0	0	0	0	0	0	0	1	NA	0	0	0	0	0	0	0	1	NA
Solyc00g017210.1.1	Actin (AHRD V1 ***- P93775_	0	0	0	0	0	0	0	0	0	0	1	NA	0	0	0	0	0	0	0	0	1	NA	0	0	0	0	0	0	0	0	1	NA	0	0	0	0	0	0	0	1	NA
Solyc00g017220.1.1	Phantastica transcription fact	0	0	0	0	0	0	0	0	0	0	1	NA	0	0	0	0	0	0	0	0	1	NA	0	0	0	0	0	0	0	0	1	NA	0	0	0	0	0	0	0	1	NA
Solyc00g017230.1.1	Expansin (AHRD V1 ***- A4G	0	0	0	0	0	0	0	0	0	0	1	NA	0	0	0	0	0	0	0	0	1	NA	0	0	0	0	0	0	0	0	1	NA	0	0	0	0	0	0	0	1	NA
Solyc00g017530.1.1	Gag-Pol polyprotein (AHRD V	0	0	0	0	0	0	0	0	0	0	1	NA	0	0	0	0	0	0	0	0	1	NA	0	0	0	0	0	0	0	0	1	NA	0	0	0	0	0	0	0	1	NA
Solyc00g017540.1.1	Signal recognition particle 72	0	0	0	0	0	0	0	0	0	0	1	NA	0	0	0	0	0	0	0	0	1	NA	0	0	0	0	0	0	0	0	1	NA	0	0	0	0	0	0	0	1	NA
Solyc00g017550.1.1	Signal recognition particle 72	0	0	0	0	0	0	0	0	0	0	1	NA	0	0	0	0	0	0	0	0	1	NA	0	0	0	0	0	0	0	0	1	NA	0	0	0	0	0	0	0	1	NA
Solyc00g017750.1.1	Unknown Protein (AHRD V1)	0	0	0	0	0	0	0	0	0	0	1	NA	0	0	0	0	0	0	0	0	1	NA	0	0	0	0	0	0	0	0	1	NA	0	0	0	0	0	0	0	1	NA
Solyc00g017760.1.1	Unknown Protein (AHRD V1)	0	0	0	0	0	0	0	0	0	0	1	NA	0	0	0	0	0	0	0	0	1	NA	0	0	0	0	0	0	0	0	1	NA	0	0	0	0	0	0	0	1	NA
Solyc00g017860.1.1	Pyridine nucleotide-disulfide (	0	0	0	0	0	0	0	0	0	0	1	NA	0	0	0	0	0	0	0	0	1	NA	0	0	0	0	0	0	0	0	1	NA	0	0	0	0	0	0	0	1	NA
Solyc00g017870.1.1	TonB family protein (AHRD V	0	0	0	0	0	0	0	0	0	0	1	NA	0	0	0	0	0	0	0	0	1	NA	0	0	0	0	0	0	0	0	1	NA	0	0	0	0	0	0	0	1	NA
Solyc00g017880.1.1	DNA mismatch repair protein	0	0	0	0	0	0	0	0	0	0	1	NA	0	0	0	0	0	0	0	0	1	NA	0	0	0	0	0	0	0	0	1	NA	0	0	0	0	0	0	0	1	NA
Solyc00g017890.1.1	Conserved predicted inner me	0	0	0	0	0	0	0	0	0	0	1	NA	0	0	0	0	0	0	0	0	1	NA	0	0	0	0	0	0	0	0	1	NA	0	0	0	0	0	0	0	1	NA
Solyc00g017900.1.1	tRNA/rRNA methyltransferas	0	0	0	0	0	0	0	0	0	0	1	NA	0	0	0	0	0	0	0	0	1	NA	0	0	0	0	0	0	0	0	1	NA	0	0	0	0	0	0	0	1	NA
Solyc00g017910.1.1	ATP-NAD/AcoX kinase (AHRD	0	0	0	0	0	0	0	0	0	0	1	NA	0	0	0	0	0	0	0	0	1	NA	0	0	0	0	0	0	0	0	1	NA	0	0	0	0	0	0	0	1	NA
Solyc00g017920.1.1	Fructose-bisphosphate aldola	0	0	0	0	0	0	0	0	0	0	1	NA	0	0	0	0	0	0	0	0	1	NA	0	0	0	0	0	0	0	0	1	NA	0	0	0	0	0	0	0	1	NA
Solyc00g017930.1.1	Acetyl-coenzyme A carboxyla	0	0	0	0	0	0	0	0	0	0	1	NA	0	0	0	0	0	0	0	0	1	NA	0	0	0	0	0	0	0	0	1	NA	0	0	0	0	0	0	0	1	NA
Solyc00g018760.1.1	Unknown Protein (AHRD V1)	0	0	0	0	0	0	0	0	0	0	1	NA	0	0	0	0	0	0	0	0	1	NA	0	0	0	0	0	0	0	0	1	NA	0	0	0	0	0	0	0	1	NA
Solyc00g018770.1.1	F-box family protein (AHRD V	0	0	0	0	0	0	0	0	0	0	1	NA	0	0	0	0	0	0	0	0	1	NA	0	0	0	0	0	0	0	0	1	NA	0	0	0	0	0	0	0	1	NA
Solyc00g018970.1.1	Unknown Protein (AHRD V1)	0	0	0	0	0	0	0	0	0	0	1	NA	0	0	0	0	0	0	0	0	1	NA	0	0	0	0	0	0	0	0	1	NA	0	0	0	0	0	0	0	1	NA
Solyc00g019610.1.1	Unknown Protein (AHRD V1)	0	0	0	0	0	0	0	0	0	0	1	NA	0	0	0	0	0	0	0	0	1	NA	0	0	0	0	0	0	0	0	1	NA	0	0	0	0	0	0	0	1	NA
Solyc00g019620.1.1	PDZ domain containing prote	0	0	0	0	0	0	0	0	0	0	1	NA	0	0	0	0	0	0	0	0	1	NA	0	0	0	0	0	0	0	0	1	NA	0	0	0	0	0	0	0	1	NA
Solyc00g019630.1.1	Unknown Protein (AHRD V1)	0	0	0	0	0	0	0	0	0	0	1	NA	0	0	0	0	0	0	0	0	1	NA	0	0	0	0	0	0	0	0	1	NA	0	0	0	0	0	0	0	1	NA
Solyc00g019730.1.1	Cytochrome c oxidase subuni	1.35	0.4	2.65	1.9	1.58	0	0.35	1.65	0.64	0.66	0.42	0.90221	0.42	0.78	0.76	0.65	0.62	0.7	0.35	0.56	0.85	1	0	0.37	0	0.12	0.29	0.34	0.33	0.32	2.59	1	0	0.33	0.69	0.34	0.41	1.18	0.79	2.34	1
Solyc00g019740.1.1	ATPase subunit 8 (AHRD V1 *	0.42	1.87	4.96	0	1.81	0.47	0.33	1.93	2.11	1.21	0.67	1	0.78	1.1	4.26	2.05	1.75	2.39	0.99	1.71	0.84	1	0	0.34	1.25	0.53	1.1	1.27	2.14	1.5	2.84	1	2.39	3.66	0.65	2.23	1.92	1.84	1.88	0.84	1
Solyc00g019750.1.1	Unknown Protein (AHRD V1)	1.88	0	1.11	0	0.75	1.05	0	0	0	0.26	0.35	1	0	0.82	0.8	0.54	0	0.98	1.48	0.82	1.52	1	0.76	0	0.94	0.57	0.62	0	0.69	0.44	0.77	1	1.34	1.37	0	0.9	0.86	1.65	1.25	1.39	1
Solyc00g019950.1.1	NADH-quinone oxidoreductas	0	0.61	0.41	0.49	0.38	0	0	0.32	0.25	0.14	0.38	1	0.32	0.3	0	0.21	0.72	0.18	0	0.3	1.45	1	0	0.56	0	0.19	0	0	0.25	0.08	0.45	1	0.98	1.25	0.53	0.92	0	0.3	0.15	0.16	1
Solyc00g019960.1.1	Unknown Protein (AHRD V1)	0	0	0	0	0	0	0	0	0	0	1	NA	0	0	0	0	0	0	0	0	1	NA	0	0	0	0	0	0	0	0	1	NA	0	0	0	0	0	0	0	1	NA
Solyc00g019970.2.1	Unknown Protein (AHRD V1)	0	0	0	0	0	0	0	0	0	0	1	NA	0	0	0	0	0	0	0	0	1	NA	0	0	0	0	0	0	0	0	1	NA	0	1.13	0	0.38	0	0	0	0.03	1
Solyc00g019980.2.1	Unknown Protein (AHRD V1)	0	0	0	0	0	0	0	0	0	0	1	NA	0	0	0	0	0	0	0	0	1	NA	0	0	0.37	0.12	0	0	0	0	0.08	NA	0	0.27	1.73	0.67	0	0	0	0.02	1
Solyc00g019990.1.1	Unknown Protein (AHRD V1)	0	0	0	0	0	0	0	0	0	0	1	NA	0	0	0	0	0	0	0	0	1	NA	0	0.75	0	0.25	0	0.69	0	0.23	0.92	1	0	0	0	0	0	0	0	1	NA
Solyc00g020000.1.1	Unknown Protein (AHRD V1)	0	0	0	0	0	0	0	0	0	0	1	NA	0	0	0	0	0	0	0	0	1	NA	0	0	0	0	0	0	0	0	1	NA	0	0	0	0	0	0	0	1	NA
Solyc00g020010.1.1	Unknown Protein (AHRD V1)	0	0.77	0	1.23	0.5	0	0	2.39	0	0.6	1.2	1	1.61	0.75	0	0.79	0.6	0	0	0.2	0.25	1	0	0.71	0.86	0.52	0.57	0.66	0	0.41	0.78	1	0	0	0	0	0.79	1.52	1.16	115.5	1
Solyc00g020020.2.1	Unknown Protein (AHRD V1)	0	0	0	0.56	0.14	0	0	0	0	0	0.07	1	0	0	0	0	0	0.2	0	0.07	6.67	1	0	0	0	0	0	0	0	0	1	1	0	0	0	0	0	0	0	1	NA
Solyc00g020030.1.1	Unknown Protein (AHRD V1)	0	0	0	0	0	1.09	0	0	0.71	0.45	45	1	0	0	0	0	0	0	0	0	1	NA	0	0	0.98	0.33	0.65	0.75	0	0.47	1.43	1	0	0	1.51	0.5	1.8	0.86	1.33	2.64	1
Solyc00g020040.1.1	Cytochrome c biogenesis (Fra	0	0	0	0	0	0	0	0	0	0	1	NA	0	0	0	0	0	0	0	0	1	NA	0	0	0	0	0	0	0	0	1	1	0	0.46	0	0.15	0	0	0	0.07	1
Solyc00g020440.1.1	Unknown Protein (AHRD V1)	0	0	0	0	0	0	0	0	0	0	1	NA	0	0	0	0	0	0	0	0	1	NA	0	0	0	0	0	0	0	0	1	NA	0	0	0	0	0	0	0	1	NA
Solyc00g020540.1.1	Aminotransferase-like protein	0	0	0.11	0	0.03	0	0.07	0	0	0.02	0.64	1	0	0	0	0	0	0	0	0	1	NA	0	0	0	0	0	0	0	0	1	NA	0	0	0	0	0	0	0	1	NA
Solyc00g020840.2.1	Ferric reductase (AHRD V1 **	0	0	0	0	0	0.33	0.23	0	0	0.14	14	1	0	0	0	0	0	0.15	0	0.05	5	1	0	0	0	0	0	0	0	0	1	1	0	0.21	0	0.07	0	0.26	0.13	1.86	1
Solyc00g020940.1.1	Cyclin-L1 (AHRD V1 *-*- D6RK	0	0	0	0	0	0	0	0	0	0	1	NA	0	0	0	0	0	0	0	0	1	NA	0	0	0	0	0	0	0	0	1	NA	0	0	0	0	0	0	0	1	NA
Solyc00g020950.1.1	Unknown Protein (AHRD V1)	0	0	0	0	0	0	0	0	0	0	1	NA	0	0	0	0	0	0	0	0	1	NA	0	0	0	0	0	0	0	0	1	NA	0	0	0	0	0	0	0	1	NA
Solyc00g020960.1.1	Unknown Protein (AHRD V1)	0	0	0	0	0	0	0	0	0	0	1	NA	0	0	0	0	0	0	0	0	1	NA	0	0	0	0	0	0	0	0	1	NA	0	0	0	0	0	0	0	1	NA
Solyc00g020970.1.1	RING finger protein 13 (AHRD	0	0	0	0	0	0	0	0	0	0	1	NA	0	0	0	0	0	0	0	0	1	NA	0	0	0	0	0	0	0	0	1	NA	0	0	0	0	0	0	0	1	NA
Solyc00g020980.1.1	Unknown Protein (AHRD V1)	0	0	0	0	0	0	0	0	0	0	1	NA	0	0	0	0	0	0	0	0	1	NA	0	0	0	0	0	0	0	0	1	NA	0	0	0	0	0	0	0	1	NA
Solyc00g020990.1.1	ADP-ribosylation factor-like 8	0	0	0	0	0	0	0	0	0	0	1	NA	0	0	0	0	0	0	0	0	1	NA	0	0	0	0	0	0	0	0	1	NA	0	0	0	0	0	0	0	1	NA
Solyc00g021490.1.1	Unknown Protein (AHRD V1)	0	0	0	0	0	0	0	0	0	0	1	NA	0	0	0	0	0	0	0	0	1	NA	0	0	0	0	0	0	0	0	1	NA	0	0	0	0	0	0	0	1	NA
Solyc00g021500.1.1	DNA alkylation repair enzyme	0	0	0	0	0	0	0	0	0	0	1	NA	0	0	0	0	0	0	0	0	1	NA	0	0	0	0	0	0	0	0	1	NA	0	0	0	0	0	0	0	1	NA
Solyc00g021510.1.1	Sodium/hydrogen exchanger	0	0	0	0	0	0	0	0	0	0	1	NA	0	0	0	0	0	0	0	0	1	NA	0	0	0	0	0	0	0	0	1	NA	0	0	0	0	0	0	0	1	NA
Solyc00g021520.1.1	GTP-binding protein HflX (AH	0	0	0	0	0	0	0	0	0	0	1	NA	0	0	0	0	0	0	0	0	1	NA	0	0	0	0	0	0	0	0	1	NA	0	0	0	0	0	0	0	1	NA
Solyc00g021530.1.1	Calcineurin-like phosphoester	0	0	0	0	0	0	0	0	0	0	1	NA	0	0	0	0	0	0	0	0	1	NA	0	0	0	0	0	0	0	0	1	NA	0	0	0	0	0	0	0	1	NA
Solyc00g021630.1.1	NADH-ubiquinone oxidoredu	0.3	0.27	0.71	0	0.32	1.68	0	0.83	0	0.63	1.96	1	0.28	0	1.02	0.43	0.21	0.16	0.24	0.2	0.47	1	0.49	0.25	0.6	0.45	0	0	0.44	0.15	0.33	1	0	1.1	0	0.37	0.55	0.53	0.54	1.47	1

Solyc00g021640.2.1	Ribosomal protein S4 (AHRD	0.16	0.14	0.76	0.23	0.32	0.36	0.51	0.15	0.35	0.34	1.06	1	0.6	0.14	0.82	0.52	0.45	0.42	0.13	0.33	0.64	1	0	0.13	0.96	0.36	0.11	0.61	0.24	0.32	0.88	1	1.84	2.11	1.74	1.9	0.59	0.99	0.79	0.42	0.944946
Solyc00g021650.1.1	Unknown Protein (AHRD V1)	0	0	0	0	0	0	0	0	0	0	1	NA	0	0	0	0	0	0	0	0	1	NA	0	0	0	0	0	0	0	0	1	NA	0	0	0	0	0	0	0	1	NA
Solyc00g021660.1.1	Neutral invertase-like protein	0	0	0	0	0	0	0	0	0	0	1	NA	0	0	0	0	0	0	0	0	1	NA	0	0	0	0	0	0	0	0	1	NA	0	0	0	0	0	0	0	1	NA
Solyc00g022070.1.1	Unknown Protein (AHRD V1)	0	0	0	0	0	0	0	0	0	0	1	NA	0	0	0	0	0	0	0	0	1	NA	0	0	0	0	0	0	0	0	1	NA	0	0	0	0	0	0	0	1	NA
Solyc00g022080.1.1	Unknown Protein (AHRD V1)	0	0	0	0	0	2.07	0	0	0	0.52	51.75	1	0	0	0	0	0	0	0	0	1	NA	0	0	0	0	0	0	0	0	1	NA	0	0	0	0	0	0	0	1	NA
Solyc00g022090.1.1	Unknown Protein (AHRD V1)	0	0	0	0	0	0	0	0	0	0	1	NA	0	0	0	0	0	0	0	0	1	NA	0	0	0	0	0	0	0	0	1	NA	0	0	0	0	0	0	0	1	NA
Solyc00g022100.1.1	Ribosomal protein L10 (Fragn	0.82	0	0	0.58	0.35	0.92	0.33	0	0.89	0.54	1.53	1	0	1.08	1.74	0.94	0	0.64	1.3	0.65	0.69	1	0.33	1.01	0.41	0.58	0.27	0.94	1.8	1	1.72	1	4.1	2.99	1.27	2.79	2.26	2.89	2.58	0.92	1
Solyc00g022110.2.1	Ribosomal protein L2 (AHRD	3.51	2.9	5.62	6.39	4.61	2.79	2.97	1.85	3.96	2.89	0.63	0.809307	2.8	4.14	8.05	5	3.65	2.47	1.58	2.57	0.51	0.719715	5.07	5.11	14.44	8.21	5.6	5.51	4.39	5.17	0.63	1	23.18	18.58	11.96	17.91	15.61	11	13.3	0.74	1
Solyc00g022920.1.1	Phosphoribosylaminoimidazo	0	0	0	0	0	0	0	0	0	0	1	NA	0	0	0	0	0	0	0	0	1	NA	0	0	0	0	0	0	0	0	1	NA	0	0	0	0	0	0	0	1	NA
Solyc00g023020.1.1	Phosphoribosylaminoimidazo	0	0	0	0	0	0	0	0	0	0	1	NA	0	0	0	0	0	0	0	0	1	NA	0	0	0	0	0	0	0	0	1	NA	0	0	0	0	0	0	0	1	NA
Solyc00g023030.1.1	CER1-like protein (AHRD V1 *	0	0	0	0	0	0	0	0	0	0	1	NA	0	0	0	0	0	0	0	0	1	NA	0	0	0	0	0	0	0	0	1	NA	0	0	0	0	0	0	0	1	NA
Solyc00g023040.1.1	CER1-like protein (AHRD V1 *	0	0	0	0	0	0	0	0	0	0	1	NA	0	0	0	0	0	0	0	0	1	NA	0	0	0	0	0	0	0	0	1	NA	0	0	0	0	0	0	0	1	NA
Solyc00g023050.1.1	CER1-like protein (AHRD V1 *	0	0	0	0	0	0	0	0	0	0	1	NA	0	0	0	0	0	0	0	0	1	NA	0	0	0	0	0	0	0	0	1	NA	0	0	0	0	0	0	0	1	NA
Solyc00g023250.1.1	Short-chain dehydrogenase/r	0	0	0	0	0	0	0	0	0	0	1	NA	0	0	0	0	0	0	0	0	1	NA	0	0	0	0	0	0	0	0	1	NA	0	0	0	0	0	0	0	1	NA
Solyc00g023260.1.1	ADP-ribosylation factor GTPa	0	0	0	0	0	0	0	0	0	0	1	NA	0	0	0	0	0	0	0	0	1	NA	0	0	0	0	0	0	0	0	1	NA	0	0	0	0	0	0	0	1	NA
Solyc00g023270.1.1	Origin recognition complex su	0	0	0	0	0	0	0	0	0	0	1	NA	0	0	0	0	0	0	0	0	1	NA	0	0	0	0	0	0	0	0	1	NA	0	0	0	0	0	0	0	1	NA
Solyc00g023470.1.1	Pectinesterase (AHRD V1 ***	0	0	0	0	0	0	0	0	0	0	1	NA	0	0	0	0	0	0	0	0	1	NA	0	0	0	0	0	0	0	0	1	NA	0	0	0	0	0	0	0	1	NA
Solyc00g023570.1.1	Unknown Protein (AHRD V1)	0	0	0	0	0	0	0	0	0	0	1	NA	0	0	0	0	0	0	0	0	1	NA	0	0	0.6	0.2	0	0.45	0	0.15	0.75	1	0	0.87	0	0.29	0	0.53	0.27	0.91	1
Solyc00g023580.1.1	Unknown Protein (AHRD V1)	0	0	0	0	0	0	0	0	0	0	1	NA	0	0	0	0	0	0	0	0	1	NA	0	0	0	0	0	0	0	0	1	NA	0	0	0	0	0	0	0	1	NA
Solyc00g023590.2.1	Unknown Protein (AHRD V1)	0	0.26	0	0	0.07	0	0	0	0	0	0.15	1	0	0.26	0	0.09	0	0	0	0	0.12	1	0	0	0.29	0.1	0.39	0.22	0	0.2	2.1	1	0	0	0	0	0	0	0	1	NA
Solyc00g023600.1.1	Unknown Protein (AHRD V1)	0	0	0	0	0	0	0	0	0	0	1	NA	0	0	0	0	0	0	0	0	1	NA	0	0	0	0	0	0	0	0	1	NA	0	0	0	0	0	0	0	1	NA
Solyc00g023700.2.1	Unknown Protein (AHRD V1)	0	0	0	0	0	0.61	1.72	0.5	0.39	0.81	80.5	0.60848	0.51	0	0.46	0.32	0	0	0	0	0.03	1	0	0.44	0	0.15	0	0	0	0	0.07	1	0	0	0.84	0.28	0	0	0	0.04	1
Solyc00g023710.1.1	Unknown Protein (AHRD V1)	0	0	0	0	0	0	0	0	0	0	1	NA	0	0	0	0	0	0	0	0	1	NA	0	0	0	0	0.37	0	0	0.12	12.33	1	0	0	0	0	0	0	0	1	NA
Solyc00g024010.1.1	FeS assembly SUF system pro	0	0	0	0	0	0	0	0	0	0	1	NA	0	0	0	0	0	0	0	0	1	NA	0	0	0	0	0	0	0	0	1	NA	0	0	0	0	0	0	0	1	NA
Solyc00g024020.1.1	Hydroxymethylglutaryl-CoA l	0	0	0	0	0	0	0	0	0	0	1	NA	0	0	0	0	0	0	0	0	1	NA	0	0	0	0	0	0	0	0	1	NA	0	0	0	0	0	0	0	1	NA
Solyc00g024030.1.1	Peptidase T (AHRD V1 ***- C	0	0	0	0	0	0	0	0	0	0	1	NA	0	0	0	0	0	0	0	0	1	NA	0	0	0	0	0	0	0	0	1	NA	0	0	0	0	0	0	0	1	NA
Solyc00g024040.1.1	Thiamine-monophosphate kir	0	0	0	0	0	0	0	0	0	0	1	NA	0	0	0	0	0	0	0	0	1	NA	0	0	0	0	0	0	0	0	1	NA	0	0	0	0	0	0	0	1	NA
Solyc00g024050.1.1	Pyridine nucleotide-disulphid	0	0	0	0	0	0	0	0	0	0	1	NA	0	0	0	0	0	0	0	0	1	NA	0	0	0	0	0	0	0	0	1	NA	0	0	0	0	0	0	0	1	NA
Solyc00g024150.2.1	Pterin-4-alpha-carbinolamine	80.35	78.19	70.82	58.38	71.94	227.64	140.7	155.81	162.96	171.78	2.39	0.000203	70.96	63.6	55.98	63.51	123.61	118.82	133.67	125.37	1.97	0.00073	132.36	128.41	79.14	113.3	95.92	127.49	116.2	113.2	1	1	27.56	72.67	45.19	48.47	82.71	66.07	74.39	1.53	1
Solyc00g024160.1.1	ATP binding protein (AHRD V	0	0	0	0	0	0	0	0	0	0	1	NA	0	0	0	0	0	0	0	0	1	NA	0	0	0	0	0	0	0	0	1	NA	0	0	0	0	0	0	0	1	NA
Solyc00g024170.1.1	Unknown Protein (AHRD V1)	0	0	0	0	0	0	0	0	0	0	1	NA	0	0	0	0	0	0	0	0	1	NA	0	0	0	0	0	0	0	0	1	NA	0	0	0	0	0	0	0	1	NA
Solyc00g024370.1.1	Unknown Protein (AHRD V1)	0	0	0	0	0	0	0	0	0	0	1	NA	0	0	0	0	0	0	0	0	1	NA	0	0	0	0	0	0	0	0	1	NA	0	0	0	0	0	0	0	1	NA
Solyc00g024570.1.1	F-box family protein (AHRD V	0	0	0	0	0	0	0	0	0	0	1	NA	0	0	0	0	0	0	0	0	1	NA	0	0	0	0	0	0	0	0	1	NA	0	0	0	0	0	0	0	1	NA
Solyc00g024670.1.1	Beta-1-3-galactosyl-o-glycosy	0	0	0	0	0	0	0	0	0	0	1	NA	0	0	0	0	0	0	0	0	1	NA	0	0	0	0	0	0	0	0	1	NA	0	0	0	0	0	0	0	1	NA
Solyc00g024680.1.1	Dof zinc finger protein (AHRD	0	0	0	0	0	0	0	0	0	0	1	NA	0	0	0	0	0	0	0	0	1	NA	0	0	0	0	0	0	0	0	1	NA	0	0	0	0	0	0	0	1	NA
Solyc00g024690.1.1	Dof zinc finger protein DOF2.	0	0	0	0	0	0	0	0	0	0	1	NA	0	0	0	0	0	0	0	0	1	NA	0	0	0	0	0	0	0	0	1	NA	0	0	0	0	0	0	0	1	NA
Solyc00g025290.1.1	Hypothetical protein (nad5 in	2.44	1.09	1.44	3.46	2.11	1.36	0.96	0	1.17	0.87	0.41	0.741309	1.52	1.06	1.72	1.43	1.13	2.32	1.6	1.68	1.17	1	2.3	5.31	4.04	3.88	3.75	4.32	1.48	3.18	0.82	1	2.89	3.55	2.5	2.98	3.36	1.79	2.58	0.86	1
Solyc00g025300.1.1	Unknown Protein (AHRD V1)	0	0	0	0	0	0	0	0	0	0	1	NA	0	0	0	0	0	0	0	0	1	NA	0	0	0	0	0	0	0	0	1	NA	0	0	0	0	0	0	0	1	NA
Solyc00g025400.2.1	Unknown Protein (AHRD V1)	0	0	0	0	0	0	0	0	0	0	1	NA	0	0	0	0	0	0	0	0	1	NA	0	0	0	0	0	0	0	0	1	1	1.19	0.61	2.58	1.46	0.38	0	0.19	0.13	1
Solyc00g025500.1.1	Transcriptional regulator Gnt	0	0	0	0	0	0	0	0	0	0	1	NA	0	0	0	0	0	0	0	0	1	NA	0	0	0	0	0	0	0	0	1	NA	0	0	0	0	0	0	0	1	NA
Solyc00g025510.1.1	D-isomer specific 2-hydroxya	0	0	0	0	0	0	0	0	0	0	1	NA	0	0	0	0	0	0	0	0	1	NA	0	0	0	0	0	0	0	0	1	NA	0	0	0	0	0	0	0	1	NA
Solyc00g025520.1.1	Unknown Protein (AHRD V1)	0	0	0	0	0	0	0	0	0	0	1	NA	0	0	0	0	0	0	0	0	1	NA	0	0	0	0	0	0	0	0	1	NA	0	0	0	0	0	0	0	1	NA
Solyc00g025530.1.1	Class II aldolase/adducin fami	0	0	0	0	0	0	0	0	0	0	1	NA	0	0	0	0	0	0	0	0	1	NA	0	0	0	0	0	0	0	0	1	NA	0	0	0	0	0	0	0	1	NA
Solyc00g025540.1.1	Transcriptional regulator Lys	0	0	0	0	0	0	0	0	0	0	1	NA	0	0	0	0	0	0	0	0	1	NA	0	0	0	0	0	0	0	0	1	NA	0	0	0	0	0	0	0	1	NA
Solyc00g025650.1.1	Gag/pol polyprotein (AHRD V	0	0	0	0	0	0	0	0	0	0	1	NA	0	0	0	0	0	0	0	0	1	NA	0	0	0	0	0	0	0	0	1	NA	0	0	0	0	0	0	0	1	NA
Solyc00g025660.1.1	Unknown Protein (AHRD V1)	0	0	0	0	0	0	0	0	0	0	1	NA	0	0	0	0	0	0	0	0	1	NA	0	0	0	0	0	0	0	0	1	NA	0	0	0	0	0	0	0	1	NA
Solyc00g026160.2.1	Ferric reductase oxidase (AH	0	0	0	0	0	0	0	0	0	0	1	NA	0.14	0	0	0.05	0	0	0	0	0.21	1	0.12	0	0	0.04	0	0	0	0	0.25	1	0	0	0	0	0	0.26	0.13	13	1
Solyc00g026860.1.1	Glutamate 5-kinase (AHRD V1	0	0	0	0	0	0	0	0	0	0	1	NA	0	0	0	0	0	0	0	0	1	NA	0	0	0	0	0	0	0	0	1	NA	0	0	0	0	0	0	0	1	NA
Solyc00g026870.1.1	GTPase obg (AHRD V1 *-*- C	0	0	0	0	0	0	0	0	0	0	1	NA	0	0	0	0	0	0	0	0	1	NA	0	0	0	0	0	0	0	0	1	NA	0	0	0	0	0	0	0	1	NA
Solyc00g026880.1.1	50S ribosomal protein L21 (A	0	0	0	0	0	0	0	0	0	0	1	NA	0	0	0	0	0	0	0	0	1	NA	0	0	0	0	0	0	0	0	1	NA	0	0	0	0	0	0	0	1	NA
Solyc00g026890.1.1	Unknown Protein (AHRD V1)	0	0	0	0	0	0	0	0	0	0	1	NA	0	0	0	0	0	0	0	0	1	NA	0	0	0	0	0	0	0	0	1	NA	0	0	0	0	0	0	0	1	NA
Solyc00g026900.1.1	Unknown Protein (AHRD V1)	0	0	0	0	0	0	0	0	0	0	1	NA	0	0	0	0	0	0	0	0	1	NA	0	0	0	0	0	0	0	0	1	NA	0	0	0	0	0	0	0	1	NA
Solyc00g027120.1.1	Mutator-like transposase (AH	0	0	0	0	0	0	0	0	0	0	1	NA	0	0	0	0	0	0	0	0	1	NA	0	0	0	0	0	0	0	0	1	NA	0	0	0	0	0	0	0	1	NA
Solyc00g027130.1.1	Sentrin-specific protease 2 (A	0.36	0	0	0	0.09	0.4	0.43	0.17	0.13	0.28	3.14	0.972324	0.17	0.31	0.15	0.21	0	0	0	0	0.05	0.833891	0	0	0	0	0.12	0	0	0.04	4	1	0.51	0	0	0.17	0	0	0	0.06	1
Solyc00g027140.1.1	Ulp1 protease family C-termi	0	0	0.4	0	0.1	0	0.09	0.1	0.08	0.07	0.68	1	0.21	0	0.1	0.1	0.08	0	0.09	0.06	0.55	1	0	0	0.11	0.04	0	0	0	0	0.27	1	0.16	0.25	0.17	0.19	0	0.1	0.05	0.26	1
Solyc00g027640.1.1	C17orf39 homolog (AHRD V1	0	0	0	0	0	0	0	0	0	0	1	NA	0	0	0	0	0	0	0	0	1	NA	0	0	0	0	0	0	0	0	1	NA	0	0	0	0	0	0	0	1	NA
Solyc00g027650.1.1	FHY1-3 (AHRD V1 ***- Q8S4	0	0	0	0	0	0	0	0	0	0	1	NA	0	0	0	0	0	0	0	0	1	NA	0	0	0	0	0	0	0	0	1	NA	0	0	0	0	0	0	0	1	NA
Solyc00g027660.1.1	WD-40 repeat family protein	0	0	0	0	0	0	0	0	0	0	1	NA	0	0	0	0	0	0	0	0	1	NA	0	0	0	0	0	0	0	0	1	NA	0	0	0	0	0	0	0	1	NA
Solyc00g027670.1.1	WD-40 repeat family protein	0	0	0	0	0	0	0	0	0	0	1	NA	0	0	0	0	0	0	0	0	1	NA	0	0	0	0	0	0	0	0	1	NA	0	0	0	0	0	0	0	1	NA
Solyc00g027770.2.1	Pectinesterase (AHRD V1 ***	49.37	48.37	69.02	62.5	57.31	50.27	48.15	38.53	44.98	45.48	0.79	0.802393	62.33	65.28	73.94	67.18	80.43	59.94	65.87	68.75	1.02	1	41.9	47.33	44.27	44.5	40.2	41.04	44.75	42	0.94	1	137.45	75.34	84.86	99.22	62.29	82.94	72.61	0.73	1
Solyc00g027870.1.1	Unknown Protein (AHRD V1)	0	0	0	0	0	0	0	0	0	0	1	NA	0	0	0	0	0	0	0	0	1	NA	0	0	0	0	0	0	0	0	1	NA	0	0	0	0	0	0	0	1	NA
Solyc00g027970.1.1	Abhydrolase domain-containi	0	0	0	0	0	0	0	0	0	0	1	NA	0	0	0	0	0	0	0	0	1	NA	0	0	0	0	0	0	0	0	1	NA	0	0	0	0	0	0	0	1	NA
Solyc00g027980.1.1	Esterase/lipase/thioesterase (	0	0	0	0	0	0	0	0	0	0	1	NA	0	0	0	0	0	0	0	0	1	NA	0	0	0	0	0	0	0	0	1	NA	0	0	0	0	0	0	0	1	NA
Solyc00g027990.1.1	Cellulose synthase-like protei	0	0	0	0	0	0	0	0	0	0	1	NA	0	0	0	0	0	0	0	0	1	NA	0	0	0	0	0	0	0	0	1	NA	0	0	0	0	0	0	0	1	NA
Solyc00g028090.1.1	Type VI secretion-associated	0	0	0	0	0	0	0	0	0	0	1	NA	0	0	0	0	0	0	0	0	1	NA	0	0	0	0	0	0	0	0	1	NA	0	0	0	0	0	0	0	1	NA
Solyc00g028100.1.1	Unknown Protein (AHRD V1)	0	0	0	0	0	0	0	0	0	0	1	NA	0	0	0	0	0	0	0	0	1	NA	0	0	0	0	0	0	0	0	1	NA	0	0	0	0	0	0	0	1	NA
Solyc00g028110.1.1	Rhs element Vgr protein (AH	0	0	0	0	0	0	0	0	0	0	1	NA	0	0	0	0	0	0	0	0	1	NA	0	0	0	0	0	0	0	0	1	NA	0	0	0	0	0	0	0	1	NA
Solyc00g028120.1.1	Rhs element Vgr protein (AH	0	0	0	0	0	0	0	0	0	0	1	NA	0	0	0	0	0	0	0	0	1	NA	0	0	0	0	0	0	0	0	1	NA	0	0	0	0	0	0	0	1	NA
Solyc00g028420.1.1	Unknown Protein (AHRD V1)	0	0	0	0	0	0	0	0	0	0	1	NA	0	0	0	0	0	0	0	0	1	NA	0	0	0	0	0	0	0	0	1	NA	0	0	0	0	0	0	0	1	NA
Solyc00g028520.1.1	Unknown Protein (AHRD V1)	0	0	0	0	0	0	0	0	0	0	1	NA	0	0	0	0	0	0	0	0	1	NA	0	0	0	0	0	0	0	0	1	NA	0	0	0	0	0	0	0	1	NA
Solyc00g028530.1.1	Unknown Protein (AHRD V1)	0	0	0	0	0	0	0	0	0	0	1	NA	0	0	0	0	0	0	0	0	1	NA	0	0	0	0	0	0	0	0	1	NA	0	0	0	0	0	0	0	1	NA
Solyc00g028540.1.1	CoA ligase (AHRD V1 ***- D3	0	0	0	0	0	0	0	0	0	0	1	NA	0	0	0	0	0	0	0	0	1	NA	0	0	0	0	0	0	0	0	1	NA	0	0	0	0	0	0	0	1	NA
Solyc00g028550.1.1	Transcriptional regulator Ma	0	0	0	0	0	0	0	0	0	0	1	NA	0	0	0	0	0	0	0	0	1	NA	0	0	0	0	0	0	0	0	1	NA	0	0	0	0	0	0	0	1	NA
Solyc00g028560.1.1	Extra-cytoplasmic Solute Rec	0	0	0	0	0	0	0	0	0	0	1	NA	0	0	0	0	0	0	0	0	1	NA	0	0	0	0	0	0	0	0	1	NA	0	0	0	0	0	0	0	1	NA
Solyc00g028760.1.1	Unknown Protein (AHRD V1)	0	0	0	0	0	0	0	0	0	0	1	NA	0	0	0	0	0	0	0	0	1	NA	0	0	0	0	0	0	0	0	1	NA	0	0	0	0	0	0	0	1	NA
Solyc00g028960.1.1	Glutaredoxin family protein (	0	0	0	0	0	0	0	0	0	0	1	NA	0	0	0	0	0	0	0	0	1	NA	0	0	0	0	0	0	0	0	1	NA	0	0	0	0	0	0	0	1	NA
Solyc00g029060.1.1	Unknown Protein (AHRD V1)	0	0	0	0	0	0	0	0	0	0	1	NA	0	0	0	0	0	0	0	0	1	NA	0	0	0	0	0	0	0	0	1	NA	0	0	0	0	0	0	0	1	NA
Solyc00g029070.1.1	Unknown Protein (AHRD V1)	0	0	0	0	0	0	0	0	0	0	1	NA	0	0	0	0	0	0	0	0	1	NA	0	0	0	0	0	0	0	0	1	NA	0	0	0	0	0	0	0	1	NA
Solyc00g029080.1.1	DNA replication helicase Dna	0	0	0	0	0	0	0	0	0	0	1	NA	0	0	0	0	0	0	0	0	1	NA	0	0	0	0	0	0	0	0	1	NA	0	0	0	0	0	0	0	1	NA
Solyc00g029090.1.1	Unknown Protein (AHRD V1)	0	0	0	0	0	0	0	0	0	0	1	NA	0	0	0	0	0	0	0	0	1	NA	0	0	0	0	0	0	0	0	1	NA	0	0	0	0	0	0	0	1	NA
Solyc00g029190.2.1	S-adenosyl-L-methionine sali	0	0	0	0	0	0.24	0.34	0	0.15	0.18	18.25	0.911811	0	0.18	0	0.06	0.3	0.22	0.33	0.28	4.72	1	0	0	0	0	0	0	0	0	1	NA	0	0	0	0	0	0	0	1	NA
Solyc00g029290.1.1	High mobility group protein (	0	0	0	0	0	0	0	0	0	0	1	NA	0	0	0	0	0	0	0	0	1	NA	0	0	0	0	0	0	0	0	1	NA	0	0	0	0	0	0	0	1	NA
Solyc00g029300.1.1	Vacuolar sorting-associated p	0	0	0	0	0	0	0	0	0	0	1	NA	0	0	0	0	0	0	0	0	1	NA	0	0	0	0	0	0	0	0	1	NA	0	0	0	0	0	0	0	1	NA
Solyc00g029500.1.1	Unknown Protein (AHRD V1)	0	0	0	0	0	0	0	0	0	0	1	NA	0	0	0	0	0	0	0	0	1	NA	0	0	0	0	0	0	0	0	1	NA	0	0	0	0	0	0	0	1	NA
Solyc00g029800.1.1	Unknown Protein (AHRD V1);	0	0	0	0	0	0	0	0	0	0	1	NA	0	0	0	0	0	0	0	0	1	NA	0	0	0	0	0	0	0	0	1	NA	0	0	0	0	0	0	0	1	NA
Solyc00g030000.1.1	Cellulose synthase-4 (AHRD V	0	0	0	0	0	0	0	0	0	0	1	NA	0	0	0	0	0	0	0	0	1	NA	0.21	0	0.13	0.11	0	0	0	0	0.09	1	0	0	0	0	0	0	0	1	NA
Solyc00g030100.1.1	Unknown Protein (AHRD V1)	0	0	0	0	0	0	0	0	0	0	1	NA	0	0	0	0	0	0	0	0	1	NA	0	0	0	0	0	0	0	0	1	NA	0	0	0	0	0	0	0	1	NA
Solyc00g030110.1.1	Unknown Protein (AHRD V1)	0	0	0	0	0	0	0	0	0	0	1	NA	0	0	0	0	0	0	0	0	1	NA	0	0	0	0	0	0	0	0	1	NA	0	0	0	0	0	0	0	1	NA
Solyc00g030210.1.1	Ribosomal RNA small subunit	0	0	0	0	0	0	0	0	0	0	1	NA	0	0	0	0	0	0	0	0	1	NA	0	0	0	0	0	0	0	0	1	NA	0	0	0	0	0	0	0	1	NA
Solyc00g030510.2.1	Polygalacturonase (AHRD V1	0	0	1.54	0	0.39	0	0	0	0	0	0.03	0.859566	0	0	0	0	0	0	0	0	1	NA	0	0	0	0	0	0	0	0	1	NA	0	0	0	0	0	0	0	1	NA
Solyc00g030520.2.1	Endoplasmic reticulum-Golgi	10.36	4.61	8.68	5.4	7.26	12.42	12.27	6.77	7.24	9.67	1.33	0.764994	8.88	5.69	8.75	7.77	5.11	8.19	6.43	6.58	0.85	1	8.15	8.89	7.31	8.12	6.09	7.44	8.54	7.36	0.91	1	3.1	5.94	6.71	5.25	6.49	5.5	6	1.14	1
Solyc00g030820.1.1	Unknown Protein (AHRD V1)	0.58	0.52	0	0.83	0.48	0	0	0.54	0.42	0.24	0.5	1	0	2.03	1.97	1.33	0.81	0.6	1.38	0.93	0.7	1	0	0	0.58	0.19	0.77	0.44	0	0.4	2.09	1	0	0	0	0	0	0.51	0.26	25.5	1
Solyc00g031020.1.1	Unknown Protein (AHRD V1)	0	0	0	0	0	0	0	0	0	0	1	NA	0	0	0	0	0	0	0	0	1	NA	0	0	0	0	0	0	0	0	1	NA	0	0	0	0	0	0	0	1	NA
Solyc00g031030.1.1	2OG-Fe(II) oxygenase (AHRD	0	0	0	0	0	0	0	0	0	0	1	NA	0	0	0	0	0	0	0	0	1	NA	0	0	0	0	0	0	0	0	1	NA	0	0	0	0	0	0	0	1	NA
Solyc00g031040.1.1	Adenosine deaminase (AHRD	0	0	0	0	0	0	0	0	0	0	1	NA	0	0	0	0	0	0	0	0	1	NA	0	0	0	0	0	0	0	0	1	NA	0	0	0	0	0	0	0	1	NA
Solyc00g031050.1.1	Major facilitator family trans	0	0	0	0	0	0	0	0	0	0	1	NA	0	0	0	0	0	0	0	0	1	NA	0	0	0	0	0	0	0	0	1	NA	0	0	0	0	0	0	0	1	NA
Solyc00g031060.1.1	Unknown Protein (AHRD V1)	0	0	0	0	0	0	0	0	0	0	1	NA	0	0	0	0	0	0	0	0	1	NA	0	0	0	0	0	0	0	0	1	NA	0	0	0	0	0	0	0	1	NA
Solyc00g031360.1.1	D-serine dehydratase (AHRD	0	0	0	0	0	0	0	0	0	0	1	NA	0	0	0	0	0	0	0	0	1	NA	0	0	0	0	0	0	0	0	1	NA	0	0	0	0	0	0	0	1	NA
Solyc00g031370.1.1	Transcriptional regulator (AH	0	0	0	0	0	0	0	0	0	0	1	NA	0	0	0	0	0	0	0	0	1	NA	0	0	0	0	0	0	0	0	1	NA	0	0	0	0	0	0	0	1	NA
Solyc00g031380.1.1	Unknown Protein (AHRD V1)	0	0	0	0	0	0	0	0	0	0	1	NA	0	0	0	0	0	0	0	0	1	NA	0	0	0	0	0	0	0	0	1	NA	0	0	0	0	0	0	0	1	NA
Solyc00g031390.1.1	Unknown Protein (AHRD V1)	0	0	0	0	0	0	0	0	0	0	1	NA	0	0	0	0	0	0	0	0	1	NA	0	0	0	0	0	0	0	0	1	NA	0	0	0	0	0	0	0	1	NA
Solyc00g031490.1.1	Unknown Protein (AHRD V1)	0	0	0	0	0	0	0	0	0	0	1	NA	0	0	0	0	0	0	0	0	1	NA	0	0	0	0	0	0	0	0	1	NA	0	0	0	0	0	0	0	1	NA
Solyc00g031500.1.1	Unknown Protein (AHRD V1)	0	0	0	0	0	0	0	0	0	0	1	NA	0	0	0	0	0	0	0	0	1	NA	0	0	0	0	0	0	0	0	1	NA	0	0	0	0	0	0	0	1	NA
Solyc00g031700.1.1	Aldo/keto reductase (AHRD V	0	0	0	0	0	0	0	0	0	0	1	NA	0	0	0	0	0	0	0	0	1	NA	0	0	0	0	0	0	0	0	1	NA	0	0	0	0	0	0	0	1	NA
Solyc00g031710.1.1	Unknown Protein (AHRD V1)	0	0	0	0	0	0	0	0	0	0	1	NA	0	0	0	0	0	0	0	0	1	NA	0	0	0	0	0	0	0	0	1	NA	0	0	0	0	0	0	0	1	NA
Solyc00g031720.1.1	Copper-translocating P-type	0	0	0	0	0	0	0	0	0	0	1	NA	0	0	0	0	0	0	0	0	1	NA	0	0	0	0	0	0	0	0	1	NA	0	0	0	0	0	0	0	1	NA

Solyc00g031730.1.1	Copper-exporting P-type ATP	0	0	0	0	0	0	0	0	0	0	1	NA	0	0	0	0	0	0	0	0	1	NA	0	0	0	0	0	0	0	0	1	NA	0	0	0	0	0	0	0	1	NA
Solyc00g032030.1.1	E3 ubiquitin-protein ligase SI	2.67	1.59	2.1	0.63	1.75	0.5	1.06	0.82	0.64	0.76	0.43	0.785598	0.83	1.16	0.38	0.79	0.93	1.85	1.4	1.39	1.76	1	0	0	0.44	0.15	0.59	0.34	0	0.31	2.11	1	1.9	0.97	0.69	1.19	1.22	0.39	0.8	0.68	1
Solyc00g032230.1.1	Unknown Protein (AHRD V1)	0	0	0	0	0	0	0	0	0	0	1	NA	0	0	0	0	0	0	0	0	1	NA	0	0	0	0	0	0	0	0	1	NA	0	0	0	0	0	0	0	1	NA
Solyc00g032940.1.1	Ulp1 protease family C-termi	0	0	0	0	0	0	0	0	0	0	1	NA	0	0	0	0	0	0	0	0	1	NA	0	0	0	0	0	0	0	0	1	NA	0	0	0	0	0	0	0	1	NA
Solyc00g033460.2.1	S-adenosyl-L-methionine salic	1.37	1.22	0	0.65	0.81	0	1.45	0.84	0	0.57	0.71	1	0.43	0	0	0.14	0	0	0	0	0.07	1	0	0	0	0	0.6	0.35	0.33	0.43	42.67	1	0	0	0	0	0	0	0	1	NA
Solyc00g033860.1.1	Ketohexokinase (AHRD V1 **	0	0	0	0	0	0	0	0	0	0	1	NA	0	0	0	0	0	0	0	0	1	NA	0	0	0	0	0	0	0	0	1	NA	0	0	0	0	0	0	0	1	NA
Solyc00g033870.1.1	Predicted CDS Pa_1_1990 (AH	0	0	0	0	0	0	0	0	0	0	1	NA	0	0	0	0	0	0	0	0	1	NA	0	0	0	0	0	0	0	0	1	NA	0	0	0	0	0	0	0	1	NA
Solyc00g033880.1.1	Oxidoreductase NAD-binding	0	0	0	0	0	0	0	0	0	0	1	NA	0	0	0	0	0	0	0	0	1	NA	0	0	0	0	0	0	0	0	1	NA	0	0	0	0	0	0	0	1	NA
Solyc00g033890.1.1	Unknown Protein (AHRD V1)	0	0	0	0	0	0	0	0	0	0	1	NA	0	0	0	0	0	0	0	0	1	NA	0	0	0	0	0	0	0	0	1	NA	0	0	0	0	0	0	0	1	NA
Solyc00g033990.1.1	Acetyltransferase (AHRD V1 *	0	0	0	0	0	0	0	0	0	0	1	NA	0	0	0	0	0	0	0	0	1	NA	0	0	0	0	0	0	0	0	1	NA	0	0	0	0	0	0	0	1	NA
Solyc00g034000.1.1	Permease of the major facilit	0	0	0	0	0	0	0	0	0	0	1	NA	0	0	0	0	0	0	0	0	1	NA	0	0	0	0	0	0	0	0	1	NA	0	0	0	0	0	0	0	1	NA
Solyc00g034010.1.1	Unknown Protein (AHRD V1)	0	0	0	0	0	0	0	0	0	0	1	NA	0	0	0	0	0	0	0	0	1	NA	0	0	0	0	0	0	0	0	1	NA	0	0	0	0	0	0	0	1	NA
Solyc00g034810.1.1	Calcineurin B-like calcium bin	0	0	0	0	0	0	0	0	0	0	1	NA	0	0	0	0	0	0	0	0	1	NA	0	0	0	0	0	0	0	0	1	NA	0	0	0	0	0	0	0	1	NA
Solyc00g035010.1.1	C2H2 finger domain-containi	0	0	0	0	0	0	0	0	0	0	1	NA	0	0	0	0	0	0	0	0	1	NA	0	0	0	0	0	0	0	0	1	NA	0	0	0	0	0	0	0	1	NA
Solyc00g035020.1.1	Unknown Protein (AHRD V1)	0	0	0	0	0	0	0	0	0	0	1	NA	0	0	0	0	0	0	0	0	1	NA	0	0	0	0	0	0	0	0	1	NA	0	0	0	0	0	0	0	1	NA
Solyc00g036320.1.1	ARID/BRIGHT DNA binding do	0	0	0	0.9	0.23	0	0	0.58	0	0.14	0.64	1	0	0	0	0	0	0	0	0	1	NA	0	0	0	0	0	0	0	0	1	NA	0.9	0	0	0.3	0.58	0	0.29	0.97	1
Solyc00g036520.1.1	Unknown Protein (AHRD V1)	0	0	0	0	0	0	0	0	0	0	1	NA	0	0	0	0	0	0	0	0	1	NA	0	0	0.35	0.12	0	0	0	0	0.09	1	0.5	0.26	0	0.25	0	0	0	0.04	1
Solyc00g036530.1.1	Unknown Protein (AHRD V1)	0	0	0	0	0	0	0	0	0	0	1	NA	0	0	0	0	0	0	0	0	1	NA	0	0	0	0	0	0	0	0	1	NA	0	0	0	0	0	0	0	1	NA
Solyc00g036540.1.1	50S ribosomal protein L2 chlo	5.61	1.87	2.48	2.98	3.24	0	4.99	1.29	1.51	1.95	0.6	1	1.31	3.66	5.33	3.43	4.87	3.63	2.21	3.57	1.04	1	1.13	2.29	2.79	2.07	3.23	1.6	2.05	2.29	1.11	1	24.95	7.14	7.55	13.21	5.14	9.85	7.49	0.57	1
Solyc00g036740.1.1	Unknown Protein (AHRD V1)	0	0	0	0	0	0	0	0	0	0	1	NA	0	0	0	0	0	0	0	0	1	NA	0	0	0	0	0	0	0	0	1	NA	0	0	0	0	0	0	0	1	NA
Solyc00g036750.1.1	Unknown Protein (AHRD V1)	0	0	0	0	0	0	0	0	0	0	1	NA	0	0	0	0	0	0	0	0	1	NA	0	0	0	0	0	0	0	0	1	NA	0	0	0	0	0	0	0	1	NA
Solyc00g036760.1.1	Proprotein convertase subtilis	0	0	0	0	0	0	0	0	0	0	1	NA	0	0	0	0	0	0	0	0	1	NA	0	0	0	0	0	0	0	0	1	NA	0	0	0	0	0	0	0	1	NA
Solyc00g036960.1.1	Unknown Protein (AHRD V1)	0	0	0	0	0	0	0	0.38	0	0.1	9.5	1	0	0	0	0	0	0	0	0	1	NA	0	0	0	0	0	0	0	0	1	NA	0	0	0	0	0	0	0	1	NA
Solyc00g038390.1.1	Unknown Protein (AHRD V1)	0	0	0	0	0	0	0	0	0	0	1	NA	0	0	0	0	0	0	0	0	1	NA	0	0	0	0	0	0	0	0	1	NA	0	0	0	0	0	0	0	1	NA
Solyc00g039290.1.1	Zinc-binding protein (AHRD V	0	0	0	0	0	0	0	0	0	0	1	NA	0	0	0	0	0	0	0	0	1	NA	0	0	0	0	0	0	0	0	1	NA	0	0	0	0	0	0	0	1	NA
Solyc00g039990.2.1	TRNA nucleotidyltransferase	5.94	6.12	5.53	3.54	5.28	3.82	4.44	6.04	3.59	4.47	0.85	1	4.65	4.89	6.34	5.29	4.34	4.21	4.67	4.41	0.83	1	2.78	4.84	6.52	4.71	4.11	3.08	3.19	3.46	0.73	1	4.89	10.67	11.54	9.03	7.16	5.48	6.32	0.7	1
Solyc00g040290.1.1	Transferase family protein (A	0	0	0	0	0	0	0	0	0	0	1	NA	0	0	0	0	0	0	0	0	1	NA	0	0	0	0	0	0	0	0	1	NA	0	0	0	0	0	0	0	1	NA
Solyc00g040390.1.1	Hydroxycinnamoyl CoA quina	0	0	0	0	0	0	0	0	0	0	1	NA	0	0	0	0	0	0	0	0	1	NA	0	0	0	0	0	0	0	0	1	NA	0	0	0	0	0	0	0	1	NA
Solyc00g040490.1.1	Unknown Protein (AHRD V1)	0	0	0	0	0	0	0	0	0	0	1	NA	0	0	0	0	0	0	0	0	1	NA	0	0	0	0	0	0	0	0	1	NA	0	0	0	0	0	0	0	1	NA
Solyc00g041090.1.1	Vacuolar-processing enzyme (	0	0	0	0	0	0	0	0	0	0	1	NA	0	0	0	0	0	0	0	0	1	NA	0	0	0	0	0	0	0	0	1	NA	0	0	0	0	0	0	0	1	NA
Solyc00g041190.1.1	Acyl carrier protein (AHRD V1	0	0	0	0	0	0	0	0	0	0	1	NA	0	0	0	0	0	0	0	0	1	NA	0	0	0	0	0	0	0	0	1	NA	0	0	0	0	0	0	0	1	NA
Solyc00g041200.1.1	Unknown Protein (AHRD V1)	0	0	0	0	0	0	0	0	0	0	1	NA	0	0	0	0	0	0	0	0	1	NA	0	0	0	0	0	0	0	0	1	NA	0	0	0	0	0	0	0	1	NA
Solyc00g041210.1.1	Tetratricopeptide TPR_2 repe	0	0	0	0	0	0	0	0	0	0	1	NA	0	0	0	0	0	0	0	0	1	NA	0	0	0	0	0	0	0	0	1	NA	0	0	0	0	0	0	0	1	NA
Solyc00g041220.1.1	UvrABC system protein B (AH	0	0	0	0	0	0	0	0	0	0	1	NA	0	0	0	0	0	0	0	0	1	NA	0	0	0	0	0	0	0	0	1	NA	0	0	0	0	0	0	0	1	NA
Solyc00g042120.1.1	Methyl-accepting chemotaxis	0	0	0	0	0	0	0	0	0	0	1	NA	0	0	0	0	0	0	0	0	1	NA	0	0	0	0	0	0	0	0	1	NA	0	0	0	0	0	0	0	1	NA
Solyc00g042130.1.1	ATP synthase subunit alpha (	0	0	0	0	0	0	0	0	0	0	1	NA	0	0	0	0	0	0	0	0	1	NA	0	0	0	0	0	0	0	0	1	NA	0	0	0	0	0	0	0	1	NA
Solyc00g042140.1.1	ATP synthase subunit beta (A	0	0	0	0	0	0	0	0	0	0	1	NA	0	0	0	0	0	0	0	0	1	NA	0	0	0	0	0	0	0	0	1	NA	0	0	0	0	0	0	0	1	NA
Solyc00g042640.1.1	Helicase-like protein (AHRD V	0	0	0.1	0.12	0.06	0.1	0	0.08	0.19	0.09	1.68	1	0	0	0	0	0	0	0	0	1	NA	0	0	0	0	0	0	0	0	1	NA	0	0	0	0	0	0	0	1	NA
Solyc00g042840.1.1	F-box domain containing prot	0.7	0.46	0.82	0.49	0.62	0.97	0.55	0.48	0.12	0.53	0.86	1	0.65	0.61	0.59	0.62	0.24	0.18	0.96	0.46	0.75	1	0.14	0.28	0.17	0.2	0	0	0.25	0.08	0.42	1	0	0	0	0	0	0	0	1	NA
Solyc00g042850.1.1	GDSL esterase/lipase 7 (AHRD	0	0	0.37	0	0.09	0	0	0.29	0	0.07	0.78	1	0	0	0	0	0	0.16	0	0.05	5.33	1	0	0	0	0	0	0	0	0	1	1	0	0	0	0	0.29	0	0.14	14.5	1
Solyc00g044350.2.1	EPIDERMAL PATTERNING FAC	5.77	8.07	2.92	1.17	4.48	5.5	9.77	10.61	9.47	8.84	1.97	0.574323	0	1.43	6.27	2.57	2.29	1.28	2.59	2.05	0.8	1	0	0	0	0	0	1.25	0	0.42	41.67	NA	0	0	0	0	0.75	0	0.38	37.5	1
Solyc00g044550.1.1	Unknown Protein (AHRD V1)	0	0	0	0	0	0	0	0	0	0	1	NA	0	0	0	0	0	0	0	0	1	NA	0	0	0	0	0	0	0	0	1	NA	0	0	0	0	0	0	0	1	NA
Solyc00g044750.1.1	U1 small nuclear ribonucleop	0.33	0	1.17	0	0.38	0.37	0.26	0	0	0.16	0.42	1	0	0	0	0	0.69	0.85	0	0.51	51.33	0.673688	0.8	0.81	0	0.54	0.65	1.25	1.44	1.11	2.07	1	1.88	0.48	0.51	0.96	2.11	1.74	1.92	2.01	1
Solyc00g045570.1.1	Unknown Protein (AHRD V1)	0	0.22	0.57	0	0.2	0.27	0	0	0.17	0.11	0.56	1	0.22	0.21	0.41	0.28	0.17	0.13	0	0.1	0.36	1	0	0.39	0.24	0.21	0	0	0.53	0.18	0.84	1	2.41	0.88	1.11	1.47	0.66	1.06	0.86	0.59	1
Solyc00g046570.1.1	Unknown Protein (AHRD V1)	0.36	0.32	1.71	0.51	0.72	0.4	1.14	0.67	1.3	0.88	1.21	1	0	0.95	0.92	0.62	1.01	0.75	0.57	0.78	1.25	1	0.59	1.77	0.72	1.03	0.24	1.1	0.53	0.62	0.61	1	0.52	0	0	0.17	1.33	0.64	0.99	5.68	1
Solyc00g046580.1.1	Serine/threonine-protein pho	0	0	0	0	0	0	0.12	0	0	0.03	3	1	0	0	0	0	0	0.08	0.35	0.14	14.33	0.978434	0	0	0	0	0	0	0	0	1	NA	0	0	0	0	0.14	0	0.07	7	1
Solyc00g046980.1.1	Unknown Protein (AHRD V1)	0	0	0	0	0	0	0	0	0	0	1	NA	0	0	0	0	0	0	0	0	1	NA	0	0	0	0	0	0	0	0	1	NA	0	0	0	0	0	0	0	1	NA
Solyc00g047180.1.1	Unknown Protein (AHRD V1)	0	0	0	0	0	0	0	0	0	0	1	NA	0	0	0	0	0	0	0	0	1	NA	0	0	0	0	0	0	0	0	1	NA	0	0	0	0	0	0	0	1	NA
Solyc00g047190.1.1	Unknown Protein (AHRD V1)	0	0	0	0	0	0	0	0	0	0	1	NA	0	0	0	0	0	0	0	0	1	NA	0	0	0	0	0	0	0	0	1	NA	0	0	0	0	0	0	0	1	NA
Solyc00g047200.1.1	Cytochrome b (AHRD V1 ***-	0.85	1.51	1	0	0.84	1.88	0.67	0	1.82	1.09	1.3	1	2.36	0.74	0	1.03	0	0	0	0	0.01	0.830731	0	0	0.84	0.28	1.11	0.64	0	0.58	2.08	1	2.41	1.84	0	1.42	0	0	0	0.01	1
Solyc00g047700.2.1	Unknown Protein (AHRD V1)	229.89	197.39	222.04	164.88	203.55	141.27	172.22	151.08	167.37	157.99	0.78	0.627694	234.92	183.3	184.25	200.82	135.3	135.79	105.28	125.46	0.62	0.121358	130.14	172.16	159.09	153.8	180.8	138.04	135.37	151.4	0.98	1	80.56	166.67	168.04	138.42	114.11	121.01	117.56	0.85	1
Solyc00g047810.1.1	Unknown Protein (AHRD V1)	0	0	0	0	0	0	0	0	0.39	0.1	9.75	1	0	0	0	0	0	0	0	0	1	NA	0	0.44	0	0.15	0	0	0	0	0.07	1	1.55	0	0	0.52	0	0.48	0.24	0.46	1
Solyc00g048410.1.1	Unknown Protein (AHRD V1)	0	0	0	0	0	0	0	0	0	0	1	NA	0	0	0	0	0.16	0	0	0.05	5.33	1	0	0	0	0	0	0	0	0	1	1	0	0	0	0	0	0	0	1	NA
Solyc00g048510.2.1	Phloem lectin (AHRD V1 ***-	0	0	0	0	0	0.44	0	0.37	0.29	0.28	27.5	0.995762	0.37	0	0	0.12	0	0	0	0	0.08	1	0	0.65	0	0.22	0.53	0.91	0.29	0.58	2.66	1	19.9	2.03	3.69	8.54	0.73	0.35	0.54	0.06	0.994665
Solyc00g049210.1.1	Cytochrome c-type biogenesi	0.11	0.5	0.13	0.63	0.34	0	0.09	0	0.24	0.08	0.24	0.702069	0.21	0.1	0.19	0.17	0.08	0.12	0.09	0.1	0.58	1	0	0.46	0	0.15	0.07	0.42	0.16	0.22	1.41	1	0.16	0.16	0.17	0.16	0.1	0.1	0.1	0.61	1
Solyc00g049220.1.1	Unknown Protein (AHRD V1)	0	0	0	0	0	0	0	0	0	0	1	NA	0	0	0	0	0	0	0	0	1	NA	0	0	0	0	0	0	0	0	1	NA	0	0	0	0	0	0.84	0.42	42	1
Solyc00g049520.2.1	Unknown Protein (AHRD V1)	484.07	468.19	292.12	319.94	391.08	485.71	522.86	828.71	496.38	583.41	1.49	0.294964	392.9	264.39	184.53	280.61	155.69	427.19	362.24	315.04	1.12	1	74.06	57.25	47.62	59.64	154.78	153.9	142.35	150.34	2.52	0.13107	85.27	111.39	118.57	105.08	241.56	164.51	203.03	1.93	0.372693
Solyc00g049620.1.1	Unknown Protein (AHRD V1)	0	0	0	0	0	0	0	0	0	0	1	NA	0	0	0	0	0	0	0	0	1	NA	0	0	0	0	0	0	0	0	1	NA	0	0	0	0	0	0	0	1	NA
Solyc00g049920.1.1	3-oxoacyl-reductase (AHRD V	0	0	0	0	0	0	0	0	0	0	1	NA	0	0	0	0	0	0	0	0	1	NA	0	0	0	0	0	0	0	0	1	NA	0	0	0	0	0	0	0	1	NA
Solyc00g050020.1.1	Ribonuclease II (AHRD V1 ***	0	0	0	0	0	0	0	0	0	0	1	NA	0	0	0	0	0	0	0	0	1	NA	0	0	0	0	0	0	0	0	1	NA	0	0	0	0	0	0	0	1	NA
Solyc00g050030.1.1	Shikimate 5-dehydrogenase (	0	0	0	0	0	0	0	0	0	0	1	NA	0	0	0	0	0	0	0	0	1	NA	0	0	0	0	0	0	0	0	1	NA	0	0	0	0	0	0	0	1	NA
Solyc00g050130.1.1	UDP-glucose glucosyltransfer	6.87	6.11	5.74	4.66	5.84	6.52	9.94	6.71	9.76	8.23	1.41	0.415497	7.59	6.34	6.77	6.9	7.34	6.74	7.09	7.06	1.02	1	3.24	5.01	4.12	4.12	4.14	4.12	4.59	4.28	1.04	1	3.46	4.47	3.3	3.74	6.42	7.15	6.79	1.81	0.860853
Solyc00g050430.1.1	BHLH transcription factor (AH	0.77	0.68	0.45	1.09	0.75	0.64	0.15	0.35	0.41	0.39	0.52	0.914991	0	0.17	0	0.06	0	0	0	0	0.18	1	0.15	0	0	0.05	0	0	0.14	0.05	0.93	1	0	0	0	0	0.88	1.68	1.28	128	0.256139
Solyc00g052430.2.1	Cytochrome c biogenesis FC (	0	0.31	0.61	0.74	0.41	0.19	0.14	0.16	0.12	0.15	0.37	0.929815	0	0.15	0.59	0.25	0.48	0.09	0.55	0.37	1.51	1	0.14	0.14	0.86	0.38	0.23	0.53	0.25	0.34	0.89	1	0	0.38	0.53	0.3	0.64	0.15	0.4	1.3	1
Solyc00g052530.1.1	Unknown Protein (AHRD V1)	0	1.03	0	0	0.26	0	0	0	0	0	0.04	1	0	0	0	0	0	0	0	0	1	NA	0.94	0	0	0.31	1.52	0	0	0.51	1.62	1	0	1.68	0	0.56	2.12	0	1.06	1.89	1
Solyc00g052540.1.1	Unknown Protein (AHRD V1)	0	0	0	0	0	0	0	0	0	0	1	NA	0	0	0	0	0	0	0	0	1	NA	0	0	0	0	0	0	0	0	1	NA	0	0	0	0	0	0.54	0.27	27	1
Solyc00g052940.2.1	Nodulin-like protein (AHRD V	0	0	0	0.33	0.08	0	0.18	0	0	0.04	0.55	1	0.22	0	0	0.07	0	0	0	0	0.14	1	0	0	0	0	0	0	0	0	1	NA	0	0	0	0	0	0	0	1	NA
Solyc00g053440.2.1	Transmembrane BAX inhibito	18.07	20.94	15.86	35.07	22.48	18.68	14.93	16.61	9.65	14.97	0.67	0.658256	24.23	32.51	27.33	28.02	34.1	27.85	18.82	26.92	0.96	1	40.06	65.74	62.15	55.98	49.41	54.14	51.44	51.66	0.92	1	40.63	56.46	44.58	47.22	61.16	50.84	56	1.19	1
Solyc00g053740.1.1	ARID/BRIGHT DNA-binding do	0	0	0	0	0	0	0	0	0	0	1	NA	0	0	0	0	0	1.27	0	0.42	42.33	1	0	0	0	0	0.4	0.93	0	0.44	44.33	1	0	0	0	0	0	0	0	1	NA
Solyc00g055940.1.1	Secreted repeat protein (AHR	0	0	0	0	0	0	0	0	0	0	1	NA	0	0	0	0	0	0	0	0	1	NA	0	0	0	0	0	0	0	0	1	NA	0	0	0	0	0	0	0	1	NA
Solyc00g055950.1.1	Response regulator receiver T	0	0	0	0	0	0	0	0	0	0	1	NA	0	0	0	0	0	0	0	0	1	NA	0	0	0	0	0	0	0	0	1	NA	0	0	0	0	0	0	0	1	NA
Solyc00g055960.1.1	Sensor histidine kinase PhoR	0	0	0	0	0	0	0	0	0	0	1	NA	0	0	0	0	0	0	0	0	1	NA	0	0	0	0	0	0	0	0	1	NA	0	0	0	0	0	0	0	1	NA
Solyc00g055970.1.1	Unknown Protein (AHRD V1)	0	0	0	0	0	0	0	0	0	0	1	NA	0	0	0	0	0	0	0	0	1	NA	0	0	0	0	0	0	0	0	1	NA	0	0	0	0	0	0	0	1	NA
Solyc00g055980.1.1	Unknown Protein (AHRD V1)	0	0	0	0	0	0	0	0	0	0	1	NA	0	0	0	0	0	0	0	0	1	NA	0	0	0	0	0	0	0	0	1	NA	0	0	0	0	0	0	0	1	NA
Solyc00g056180.1.1	Unknown Protein (AHRD V1)	0	0	0	0	0	0	0	0	0	0	1	NA	0	0	0	0	0	0	0	0	1	NA	0	0	0	0	0	0	0	0	1	NA	0	0	0	0	0	0	0	1	NA
Solyc00g056680.1.1	Lipase-like protein (AHRD V1	0.98	0.65	0.72	0.69	0.76	1.5	1.06	1.35	1.32	1.31	1.72	0.664595	0.46	0.21	0.72	0.46	0.77	1.02	2.02	1.27	2.74	0.414124	0.4	0.9	0.37	0.56	0.24	0.93	1.43	0.87	1.56	1	0.17	1.6	1.13	0.97	2.02	0.97	1.5	1.55	1
Solyc00g057280.1.1	B3 domain-containing protei	0	0	0	0	0	0	0	0	0	0	1	NA	0	0	0	0	0	0	0	0	1	NA	0	0	0	0	0	0	0	0	1	NA	0	0	0	0	0	0	0	1	NA
Solyc00g057880.2.1	Unknown Protein (AHRD V1)	71.97	56.93	47.17	79.18	63.81	80	69.44	79.04	77.5	76.5	1.2	0.854547	61.37	99.05	69.23	76.55	70.71	89.02	106.83	88.85	1.16	0.997395	45.22	40.73	17.86	34.6	40.71	39.4	43.7	41.27	1.19	1	31.27	29.04	33.82	31.38	38.43	63.1	50.77	1.62	1
Solyc00g058280.1.1	Unknown Protein (AHRD V1)	0	0	0.28	0	0.07	0	0	0	0	0	0.14	1	0	0	0.2	0.07	0.16	0	0.56	0.24	3.6	1	0	0	0	0	0.16	0	0	0.05	5.33	1	0	0.17	0	0.06	0	0	0	0.18	1
Solyc00g058480.1.1	Paired amphipathic helix prot	0	0	0	0	0	0	0	0	0	0	1	NA	0	0	0	0	0	0	0	0	1	NA	0	0	0	0	0.51	0	0	0.17	17	1	0	0	0	0	0	0	0	1	NA
Solyc00g058490.1.1	Ulp1 protease family C-termi	0	0	0	0	0	0.26	0.18	0.85	0.17	0.36	36.5	0.595668	0.21	0	0.39	0.2	0.16	0.12	0.18	0.15	0.77	1	0.19	0.56	0	0.25	0.15	0.17	0.17	0.16	0.65	1	0	0.34	0	0.11	0	0.4	0.2	1.76	1
Solyc00g058890.1.1	Unknown Protein (AHRD V1)	0	0	0	0	0	0	0	0	0	0	1	NA	0	0	0	0	0	0	0	0	1	NA	0	0	0	0	0	0	0	0	1	NA	0	0	0	0	0	0	0	1	NA
Solyc00g058900.1.1	GDSL esterase/lipase At2g31	0	0	0	0	0	0	0	0	0	0	1	NA	0	0	0	0	0	0	0	0	1	NA	0	0	0	0	0	0	0	0	1	NA	0	0	0	0	0	0	0	1	NA
Solyc00g059100.2.1	WD-repeat protein-like (AHR	3.04	3.42	5.01	3.15	3.65	2.7	3.2	4.09	3.49	3.37	0.92	1	3.76	1.58	3.93	3.09	5.05	5.24	3.98	4.76	1.54	0.59019	3.27	4.12	4.22	3.87	3.59	3.83	2.8	3.41	0.88	1	1.73	4.26	2.18	2.72	1.67	2.84	2.25	0.83	1
Solyc00g059500.1.1	CDP-alcohol phosphatidyltrar	14.96	21.19	11.23	13.47	15.21	12.1	12.89	21.89	9.28	14.04	0.92	1	20.88	19.51	17.81	19.4	17.93	16.55	18.17	17.55	0.9	1	17.58	19.95	20.25	19.26	21.89	17.53	20.82	20.08	1.04	1	24.17	22.72	17.78	21.56	19.92	21.47	20.7	0.96	1
Solyc00g060700.1.1	Mg chelatase subunit ChlI (AH	0	0	0	0	0	0	0	0	0	0	1	NA	0	0	0	0	0	0	0	0	1	NA	0	0	0	0	0	0	0	0	1	NA	0	0	0	0	0	0	0	1	NA
Solyc00g060710.1.1	Mg chelatase subunit ChlI (AH	0	0	0	0	0	0	0	0	0	0	1	NA	0	0	0	0	0	0	0	0	1	NA	0	0	0	0	0	0	0	0	1	NA	0	0	0	0	0	0	0	1	NA
Solyc00g060810.2.1	Major latex-like protein (AHR	0	0.4	1.06	0	0.36	1.5	7.09	7.43	0	4	10.97	0.288836	0.42	0	1.52	0.65	16.5	26.72	2.12	15.11	23.37	0.095528	170.77	78.26	60.14	103.06	277.43	285.04	575.25	379.24	3.68	0.029692	474.09	1629.44	559.68	887.74	165.15	144.41	154.78	0.17	0.510527
Solyc00g062710.1.1	Unknown Protein (AHRD V1)	0	0	0	0	0	0	0	0	0	0	1	NA	0	0	0	0	0	0	0	0	1	NA	0	0	0	0	0	0	0	0	1	NA	0	0	0	0	0	0	0	1	NA
Solyc00g063110.2.1	Non-specific lipid-transfer pro	0	0	0	0	0	0	0	0	0	0	1	NA	0	0	0	0	0	0	0	0	1	NA	0	0	0	0	0	0	0	0	1	NA	0	0	0	0	0	0	0	1	NA
Solyc00g063510.1.1	Fasciculation and elongation	0	0	0	0	0	0	0	0	0	0	1	NA	0	0	0	0	0	0	0	0	1	NA	0	0	0	0	0	0	0	0	1	NA	0	0	0	0	0	0	0	1	NA
Solyc00g065510.1.1	Palmitoyltransferase swf1 (AI	0.27	0	0	0	0.07	0	0	0	0	0	0.15	1	0	0	0	0	0	0	0	0	1	NA	0	0	0	0	0	0	0	0	1	NA	0	0	0	0	0	0	0	1	NA
Solyc00g065610.1.1	Unknown Protein (AHRD V1)	0	0	0	0	0	0	0	0	0	0	1	NA	0	0	0	0	0	0	0	0	1	NA	0	0	0	0	0	0	0	0	1	NA	0	0	0	0	0	0	0	1	NA
Solyc00g065620.1.1	Unknown Protein (AHRD V1)	0	0	0	0	0	0	0	0	0	0	1	NA	0	0	0	0	0	0	0	0	1	NA	0	0	0	0	0	0	0	0	1	NA	0	0	0	0	0	0	0	1	NA
Solyc00g065630.1.1	Pre-mRNA-processing protein	0	0	0	0	0	0	0	0	0	0	1	NA	0	0	0	0	0	0	0	0	1	NA	0	0	0	0	0	0	0	0	1	NA	0	0	0	0	0	0	0	1	NA
Solyc00g065930.1.1	Peptidase family S46 dipeptic	0	0	0	0	0	0	0	0	0	0	1	NA	0	0	0	0	0	0	0	0	1	NA	0	0	0	0	0	0	0	0	1	NA	0	0	0	0	0	0	0	1	NA
Solyc00g065940.1.1	Unknown Protein (AHRD V1)	0	0	0	0	0	0	0	0	0	0	1	NA	0	0	0	0	0	0	0	0	1	NA	0	0	0	0	0	0	0	0	1	NA	0	0	0	0	0	0	0	1	NA
Solyc00g066140.1.1	YdiU protein (AHRD V1 ***- (	0	0	0	0	0	0	0	0	0	0	1	NA	0	0	0	0	0	0	0	0	1	NA	0	0	0	0	0	0	0	0	1	NA	0	0	0	0	0	0	0	1	NA
Solyc00g066150.1.1	Penicillin-binding protein 1A	0	0	0	0	0	0	0	0	0	0	1	NA	0	0	0	0	0	0	0	0	1	NA	0	0	0	0	0	0	0	0	1	NA	0	0	0	0	0	0	0	1	NA
Solyc00g066350.1.1	Serine/threonine-protein pho	0	0	0	0	0	0	0	0	0	0	1	NA	0	0	0	0	0	0	0	0	1	NA	0	0	0	0	0	0	0	0	1	NA	0	0	0	0	0	0	0	1	NA
Solyc00g066360.1.1	Serine/threonine-protein pho	0	0	0.27	0	0.07	0	0	0	0	0	0.15	1	0	0	0.2	0.07	0	0	0	0	0.15	1	0	0	0	0	0	0	0	0	1	NA	0	0	0	0	0	0	0	1	NA
Solyc00g066370.1.1	Unknown Protein (AHRD V1)	0	0	0	0	0	0	0	0	0	0	1	NA	0	0	0	0	0	0	0	0	1	NA	0	0	0	0	0	0	0	0	1	NA	0	0	0	0	0	0	0	1	NA
Solyc00g067470.1.1	Unknown Protein (AHRD V1)	3.6	3.2	7.08	11.88	6.44	2.67	1.89	5.51	2.58	3.16	0.49	0.868387	2.23	4.17	5.07	3.82	5.82	3.73	0.94	3.5	0.91	1	0.97	1.95	4.76	2.56	2.36	0.91	2.62	1.96	0.77	1	1.71	0	0	0.57	1.1	0	0.55	0.96	1
Solyc00g068970.2.1	Cytochrome P450 like_TBP (A	0	0	0	0	0	0	0	0	0	0	1	NA	0	0	0	0	0	0	0	0	1	NA	0	0	0	0	0	0	0	0	1	NA	0	0	0	0	0	0	0	1	NA
Solyc00g068980.1.1	Unknown Protein (AHRD V1)	0	0	1.42	0	0.35	0	0	1.1	0	0.28	0.77	1	0	0	2.03	0.68	0.83	0.62	0	0.48	0.71	1	0	0	0	0	1.58	0.91	0	0.83	83	1	1.71	0	0	0.57	0	0	0	0.02	1
Solyc00g069680.1.1	Unknown Protein (AHRD V1)	0	0	0	0	0	0	0	0	0	0	1	NA	0	0	0	0	0	0	0	0	1	NA	0	0	0	0	0	0	0	0	1	NA	0	0	0	0	0	0	0	1	NA

Solyc00g069880.2.1	Unknown Protein (AHRD V1)	0	0	0.34	0	0.09	0	0	0	0	0	0.12	1	0	0.5	0.24	0.25	0	0	0.23	0.08	0.31	1	0.23	0	0.29	0.17	0.19	0	0.21	0.13	0.77	1	0.41	0.21	0	0.21	0	0.76	0.38	1.84	1
Solyc00g070680.1.1	Unknown Protein (AHRD V1)	0	0	0	0	0	0	0	0	0	0	1	NA	0	0	0	0	0	0	0	0	1	NA	0	0	0	0	0	0	0	0	1	NA	0	0	0	0	0	0	0	1	NA
Solyc00g071180.2.1	Cysteine proteinase inhibitor	0.19	0	0	0	0.05	0.21	0	0	0	0.05	1.11	1	2.97	0	0	0.99	0	0.1	0	0.03	0.03	0.916547	0	0	0	0	0	0	0	0	1	NA	0	0	0	0	0	0	0	1	NA
Solyc00g071580.1.1	Unknown Protein (AHRD V1)	0	0	0	0	0	0	0	0	0	0	1	NA	0	0	0	0	0	0	0	0	1	NA	0	0	0	0	0	0	0	0	1	NA	0	0	0	0	0	0	0	1	NA
Solyc00g071590.1.1	TBC1 domain family member	0	0	0	0	0	0	0	0	0	0	1	NA	0	0	0	0	0	0	0	0	1	NA	0	0	0	0	0	0	0	0	1	NA	0	0	0	0	0	0	0	1	NA
Solyc00g071600.1.1	GTPase activating protein (AH	0	0	0	0	0	0	0	0	0	0	1	NA	0	0	0	0	0	0	0	0	1	NA	0	0	0	0	0	0	0	0	1	NA	0	0	0	0	0	0	0	1	NA
Solyc00g072100.2.1	LTRGag-pol-polymerase 3 (AH	53.64	65.83	28.3	26.86	43.66	57.41	55.5	80.57	48.56	60.51	1.39	0.688361	55.17	40.55	25.33	40.35	38.36	52.44	69.13	53.31	1.32	0.817649	13.19	14.66	5.95	11.27	16.85	10.86	14.81	14.17	1.26	1	8.05	10.89	7.69	8.88	4.58	6.43	5.5	0.62	1
Solyc00g072400.2.1	Peroxidase 1 (AHRD V1 ***- A	0	0	0	0	0	0	0	0	0	0	1	NA	0	0	0	0	0	0	0	0	1	NA	0	0	0	0	0	0	0	0	1	NA	0	0	0	0	0	0	0	1	NA
Solyc00g072800.2.1	Unknown Protein (AHRD V1)	0	0	0	0	0	0	0	0	0	0	1	NA	0	0	0	0	0	0	0	0	1	NA	0	0	0	0	0	0	0	0	1	NA	1.53	0	0.82	0.78	0.49	0	0.24	0.31	1
Solyc00g073200.1.1	Transposon protein-like (AHR	0	0	0	0	0	0	0	0	0	0	1	NA	0	0	0	0	0	0	0	0	1	NA	0	0	0	0	0	0	0	0	1	NA	0	0	0	0	0	0	0	1	NA
Solyc00g073900.1.1	Unknown Protein (AHRD V1)	0	0	0	0	0	0	0	0	0	0	1	NA	0	0	0	0	0	0	0	0	1	NA	0	0	0	0	0	0	0	0	1	NA	0	0	0	0	0	0	0	1	NA
Solyc00g073910.1.1	Unknown Protein (AHRD V1)	0	0	0	0	0	0	0	0	0	0	1	NA	0	0	0	0	0	0	0	0	1	NA	0	0	0	0	0	0	0	0	1	NA	0	0	0	0	0	0	0	1	NA
Solyc00g074210.1.1	Mitochondrial import inner m	0	0	0	0	0	0	1.4	0	0.95	0.59	58.75	0.632426	0	0	0	0	3.69	2.06	3.13	2.96	296	0.007197	0.36	0	0.44	0.27	1.45	0.34	1.94	1.24	4.66	1	0.63	0.96	0	0.53	3.24	1.55	2.4	4.52	1
Solyc00g074810.1.1	DNA processing protein DprA	0	0	0	0	0	0	0	0	0	0	1	NA	0	0	0	0	0	0	0	0	1	NA	0	0	0	0	0	0	0	0	1	NA	0	0	0	0	0	0	0	1	NA
Solyc00g074820.1.1	Unknown Protein (AHRD V1)	0	0	0	0	0	0	0	0	0	0	1	NA	0	0	0	0	0	0	0	0	1	NA	0	0	0	0	0	0	0	0	1	NA	0	0	0	0	0	0	0	1	NA
Solyc00g074830.1.1	FeS assembly protein SufD (A	0	0	0	0	0	0	0	0.42	0	0.1	10.5	1	0	0	0	0	0	0	0	0	1	NA	0	0	0	0	0	0	0	0	1	NA	0	0	0	0	0	0	0	1	NA
Solyc00g075030.2.1	Gag polyprotein (AHRD V1 *-	0	0	0	0	0	0	0	0	0	0	1	NA	0	0	0	0	0	0	0	0	1	NA	0	0	0	0	0	0	0	0	1	NA	0	0	0	0	0	0	0	1	NA
Solyc00g075040.1.1	Transposon Ty1-A Gag-Pol po	0	0	0	0	0	0	0	0	0	0	1	NA	0	0	0	0	0	0	0	0	1	NA	0	0	0	0	0	0	0	0	1	NA	0	0	0	0	0	0	0	1	NA
Solyc00g075440.1.1	Unknown Protein (AHRD V1)	0	0	0	0	0	0	0	0	0	0	1	NA	0	0	0	0	0	0	0	0	1	NA	0	0	0	0	0	0	0	0	1	NA	0	0	0	0	0	0	0	1	NA
Solyc00g075450.2.1	Unknown Protein (AHRD V1)	1.01	0	1.2	0	0.55	0	0	0	0.73	0.18	0.33	1	0	0	0.86	0.29	0	0	0	0	0.03	1	0	0	0	0	0	0	0	0	1	NA	0	0	0	0	0.93	0	0.47	46.5	1
Solyc00g076250.1.1	Unknown Protein (AHRD V1)	0	0	0	0	0	0	0	0	0	0	1	NA	0	0	0	0	0	0	0	0	1	NA	0	0	0	0	0	0	0	0	1	NA	0	0	0	0	0	0	0	1	NA
Solyc00g079050.1.1	Unknown Protein (AHRD V1)	0	0	0	0	0	0	0	0	0	0	1	NA	0	0	0	0	0	0	0	0	1	NA	0	0	0	0	0	0	0	0	1	NA	0	0	0	0	0	0	0	1	NA
Solyc00g080550.1.1	Unknown Protein (AHRD V1)	0	0	0	0	0	0	0	0	0	0	1	NA	0	0	0	0	0	0	0	0	1	NA	0	0	0	0	0	0	0	0	1	NA	0	0	0	0	0	0	0	1	NA
Solyc00g080750.2.1	Unknown Protein (AHRD V1)	0.28	0.25	0.33	0.39	0.31	0.62	0.22	0.51	0.2	0.39	1.24	1	0.26	0	0.24	0.17	0	0	0	0	0.06	1	0	0.23	0	0.08	0.18	0	0	0.06	0.78	1	0	0.2	0	0.07	0	0	0	0.15	1
Solyc00g082150.2.1	Kinesin light chain (AHRD V1	85.12	76.78	80.54	113.46	88.97	49.86	84.78	57.8	62.52	63.74	0.72	0.537431	82.23	101.75	112.41	98.8	86.74	90.95	82.72	86.8	0.88	1	69.04	104.05	66.46	79.85	84.31	59.68	90.42	78.14	0.98	1	117.07	74.79	83.83	91.9	85.72	104.92	95.32	1.04	1
Solyc00g082550.1.1	Unknown Protein (AHRD V1)	0	0.69	0.46	0	0.29	0	0	0	0	0	0.03	0.920738	0	0	0	0	0	0	0	0	1	NA	0	0	0	0	0	0	0	0	1	NA	0	0	0	0	0	0	0	1	NA
Solyc00g084650.1.1	Unknown Protein (AHRD V1)	0	0	0	0	0	0	0	0	0	0	1	NA	0	0	0	0	0	0	0	0	1	NA	0	0	0	0	0	0	0	0	1	NA	0	0	0	0	0	0	0	1	NA
Solyc00g084750.2.1	Pentatricopeptide repeat-con	1.4	1	1.32	0	0.93	2.18	0.44	0.26	1	0.97	1.04	1	0.52	0.97	1.42	0.97	0.39	1.45	1.54	1.13	1.16	1	0.68	1.37	1.11	1.05	0.55	0.64	1.22	0.8	0.76	1	0.79	3.45	1.29	1.84	1.54	0.25	0.9	0.49	1
Solyc00g084760.1.1	RNase H family protein (AHR	0.86	1.28	0.68	0.81	0.91	1.6	1.14	2.38	1.03	1.54	1.69	0.924458	1.07	1.5	1.46	1.34	1.4	2.83	3.62	2.62	1.95	0.697335	1.63	0.7	0.57	0.97	0.95	0.66	0.63	0.75	0.77	1	0.41	0.42	0.89	0.57	0	0.51	0.26	0.44	1
Solyc00g084770.1.1	Transposase (AHRD V1 **-- A	1.33	1.19	0.79	0.94	1.06	1.48	1.05	0.61	0	0.79	0.74	1	1.24	1.16	0	0.8	0	1.04	2.09	1.04	1.3	1	1.62	0	0.66	0.76	0.44	0	1.94	0.79	1.04	1	0	0.48	0	0.16	0	0	0	0.06	1
Solyc00g084870.2.1	TCP family transcription facto	1.24	1.1	1.95	2.33	1.66	0.92	0.33	0.76	2.07	1.02	0.62	1	1.15	1.43	1.04	1.21	1.43	1.92	1.3	1.55	1.28	1	0	0.34	0	0.11	1.08	0	0.3	0.46	4.06	1	0	0.3	0	0.1	0.38	0	0.19	1.9	1
Solyc00g085070.2.1	Squalene monooxygenase (AH	20.33	18.1	28.68	33.59	25.18	22.28	30.87	22.75	22.15	24.51	0.97	1	18.65	19.4	22.46	20.17	22.19	21.5	20.11	21.27	1.05	1	22.4	27.69	29.21	26.43	29.14	26.83	27.56	27.84	1.05	1	54.38	30.36	35.2	39.98	45.94	39.52	42.73	1.07	1
Solyc00g085370.1.1	Unknown Protein (AHRD V1)	0	0	0	0	0	0	0	0	0	0	1	NA	0	0	0	0	0	0	0	0	1	NA	0	0	0	0	0	0	0	0	1	NA	0	0	0	0	0	0	0	1	NA
Solyc00g085770.1.1	Unknown Protein (AHRD V1)	0	0	0	0	0	0	0	0	0	0	1	NA	0	0	0	0	0	0	0	0	1	NA	0	0	0	0	0	0	0	0	1	NA	0	0	0	0	0	0	0	1	NA
Solyc00g085780.1.1	Unknown Protein (AHRD V1)	0	0	0	0	0	0	0	0	0	0	1	NA	0	0	0	0	0	0	0	0	1	NA	0	0	0	0	0	0	0	0	1	NA	0	0	0	0	0	0	0	1	NA
Solyc00g088080.1.1	Adenine phosphoribosyltrans	0	0	0	0	0	0	0	0	0	0	1	NA	0	0	0	0	0	0	0	0	1	NA	0	0	0	0	0	0	0	0	1	NA	0	0	0	0	0	0	0	1	NA
Solyc00g088090.1.1	Conserved hypothetical mem	0	0	0	0	0	0	0	0	0	0	1	NA	0	0	0	0	0	0	0	0	1	NA	0	0	0	0	0	0	0	0	1	NA	0	0	0	0	0	0	0	1	NA
Solyc00g088190.1.1	Phosphoribosylaminoimidazo	0	0	0	0	0	0	0	0	0	0	1	NA	0	0	0	0	0	0	0	0	1	NA	0	0	0	0	0	0	0	0	1	NA	0	0	0	0	0	0	0	1	NA
Solyc00g088200.1.1	Unknown Protein (AHRD V1)	0	0	0	0	0	0	0	0	0	0	1	NA	0	0	0	0	0	0	0	0	1	NA	0	0	0	0	0	0	0	0	1	NA	0	0	0	0	0	0	0	1	NA
Solyc00g088210.1.1	Major facilitator superfamily	0	0	0	0	0	0	0	0	0	0	1	NA	0	0	0	0	0	0	0	0	1	NA	0	0	0	0	0	0	0	0	1	NA	0	0	0	0	0	0	0	1	NA
Solyc00g090110.1.1	UDP-3-O- (AHRD V1 ***- C6B	0	0	0	0	0	0	0	0	0	0	1	NA	0	0	0	0	0	0	0	0	1	NA	0	0	0	0	0	0	0	0	1	NA	0	0	0	0	0	0	0	1	NA
Solyc00g090120.1.1	Unknown Protein (AHRD V1);	0	0	0	0	0	0	0	0	0	0	1	NA	0	0	0	0	0	0	0	0	1	NA	0	0	0	0	0	0	0	0	1	NA	0	0	0	0	0	0	0	1	NA
Solyc00g090130.1.1	Protein translocase subunit se	0	0	0	0	0	0	0	0	0	0	1	NA	0	0	0	0	0	0	0	0	1	NA	0	0	0	0	0	0	0	0	1	NA	0	0	0	0	0	0	0	1	NA
Solyc00g090430.2.1	Os08g0100600 protein (Fragr	0	0	0	0	0	0	0	0	0	0	1	NA	0	0	0.16	0.05	0	0	0	0	0.19	1	0	0	0	0	0	0	0	0	1	1	0	0	0	0	0	0.16	0.08	8	1
Solyc00g090630.1.1	Membrane transporter (AHRD	0	0	0	0	0	0	0	0	0	0	1	NA	0	0	0	0	0	0	0	0	1	NA	0	0	0	0	0	0	0	0	1	NA	0	0	0	0	0	0	0	1	NA
Solyc00g090640.1.1	Membrane transporter (AHRD	0	0	0	0	0	0	0	0	0	0	1	NA	0	0	0	0	0	0	0	0	1	NA	0	0	0	0	0	0	0	0	1	NA	0	0	0	0	0	0	0	1	NA
Solyc00g090650.1.1	Membrane transporter (AHRD	0	0	0	0	0	0	0	0	0	0	1	NA	0	0	0	0	0	0	0	0	1	NA	0	0	0	0	0	0	0	0	1	NA	0	0	0	0	0	0	0	1	NA
Solyc00g090660.1.1	Unknown Protein (AHRD V1)	0	0	0	0	0	0	0	0	0	0	1	NA	0	0	0	0	0	0	0	0	1	NA	0	0	0	0	0	0	0	0	1	NA	0	0	0	0	0	0	0	1	NA
Solyc00g090860.1.1	Nitrate transporter (AHRD V1	0	0	0	0	0	0	0	0	0	0	1	NA	0	0	0	0	0	0	0	0	1	NA	0	0	0	0	0	0	0	0	1	NA	0	0	0	0	0	0	0	1	NA
Solyc00g090870.2.1	5-AMP-activated protein kina	14.48	13.84	8.86	15.17	13.09	21.46	12.7	21.7	15.4	17.82	1.36	0.707126	19.46	16.32	12.68	16.15	15.62	16.11	16.86	16.2	1	1	14.73	18.79	5.86	13.13	14.45	15.85	16.42	15.57	1.19	1	21.35	9.35	4.95	11.88	12.27	18.34	15.3	1.29	1
Solyc00g091170.2.1	ATP binding protein (AHRD V	8.07	12.02	5.38	10.18	8.91	9.17	8.73	15	6.68	9.89	1.11	1	7.02	9.61	6.37	7.67	3.65	5.54	11.31	6.83	0.89	1	2.69	5	1.74	3.14	1.96	0.93	2.68	1.86	0.59	1	1.5	3.95	2.43	2.63	1.93	1.23	1.58	0.6	1
Solyc00g092870.1.1	Unknown Protein (AHRD V1)	0	0	0	0	0	0	0	0	0	0	1	NA	0	0	0	0	0	0	0	0	1	NA	0	0	0	0	0	0	0	0	1	NA	0	0	0	0	0	0	0	1	NA
Solyc00g092880.1.1	Unknown Protein (AHRD V1)	0	0	0	0	0	0	0	0	0	0	1	NA	0	0	0	0	0	0	0	0	1	NA	0	0	0	0	0	0	0	0	1	NA	0	0	0	0	0	0	0	1	NA
Solyc00g092890.1.1	Unknown Protein (AHRD V1)	0	0	0	0	0	0	0	0	0	0	1	NA	0	0	0	0	0	0	0	0	1	NA	0	0	0	0	0	0	0	0	1	NA	0	0	0	0	0	0	0	1	NA
Solyc00g092900.1.1	Unknown Protein (AHRD V1)	0	0.16	0	0	0.04	0	0.29	0	0	0.07	1.81	1	0	0.16	0	0.05	0	0.1	0.15	0.08	1.56	1	0	0	0.18	0.06	0	0.28	0	0.09	1.56	NA	0	0	0.57	0.19	0	0	0	0.05	1
Solyc00g093000.1.1	Unknown Protein (AHRD V1)	3.14	11.18	11.12	17.77	10.8	10.48	14.88	5.78	4.51	8.91	0.83	1	10.23	12.29	9.29	10.6	5.45	8.13	9.88	7.82	0.74	1	3.81	16.64	9.35	9.93	12.38	8.33	6.87	9.19	0.93	1	20.1	15.97	9.66	15.24	12.94	13.77	13.36	0.88	1
Solyc00g093100.1.1	Multidrug and toxin extrusion	0	0	0	0	0	0	0	0	0	0	1	NA	0	0	0	0	0	0	0	0	1	NA	0	0	0	0	0	0	0	0	1	NA	0	0	0	0	0	0	0	1	NA
Solyc00g093300.1.1	Transposase (Fragment) (AHR	0	0	0	0.37	0.09	0	0	0	0	0	0.11	1	0	0	0	0	0	0	0	0	1	NA	0	0	0	0	0	0	0	0	1	NA	0	0	0	0	0	0	0	1	NA
Solyc00g093310.1.1	Unknown Protein (AHRD V1)	0	1.25	0	0.99	0.56	0.78	0.55	0	2.52	0.96	1.72	1	1.96	0	0	0.65	0.97	0.73	1.1	0.93	1.43	1	0.57	0	0	0.19	1.84	0.53	0	0.79	4.16	1	2	0.51	1.08	1.2	0	0.62	0.31	0.26	1
Solyc00g094010.1.1	Virulence VirF2 protein (AHRD	0	0	0	0	0	0	0	0	0	0	1	NA	0	0	0	0	0	0	0	0	1	NA	0	0	0	0	0	0	0	0	1	NA	0	0	0	0	0	0	0	1	NA
Solyc00g094020.1.1	Replication protein C (AHRD V	0	0	0	0	0	0	0	0	0	0	1	NA	0	0	0	0	0	0	0	0	1	NA	0	0	0	0	0	0	0	0	1	NA	0	0	0	0	0	0	0	1	NA
Solyc00g094520.1.1	Unknown Protein (AHRD V1)	0	0.95	0	4.52	1.37	0	0	0	0	0	0.01	0.854351	0	0	1.8	0.6	0.74	0.55	0	0.43	0.72	1	0	0.87	0	0.29	0.7	0	0	0.23	0.8	1	3.03	0.77	0	1.27	0	0.93	0.47	0.37	1
Solyc00g094530.1.1	NADH-ubiquinone oxidoredu	0	0	0	0.72	0.18	0	0	0	0.36	0.09	0.5	1	0	0	0	0	0	0.26	0	0.09	8.67	1	0	0.41	0	0.14	0	0	0	0	0.07	1	0	0.37	1.56	0.64	0	0	0	0.02	1
Solyc00g094540.1.1	Unknown Protein (AHRD V1)	0	0	0	0	0	0	0	0	0	0	1	NA	0	0	0	0	0	0	0	0	1	NA	0	0	0	0	0	0	0	0	1	NA	0	0	0	0	0	0	0	1	NA
Solyc00g094550.1.1	Unknown Protein (AHRD V1)	0	0	0	0	0	0	0	0	0	0	1	NA	0	0	0.71	0.24	0	0	0	0	0.04	1	0	0	0.84	0.28	0	0	0	0	0.04	NA	0	0	0	0	0	0	0	1	NA
Solyc00g094850.1.1	Unknown Protein (AHRD V1)	0	0	0	0	0	0	0	0	0	0	1	NA	0	0	0	0	0	0	0	0	1	NA	0	0	0	0	0	0	0	0	1	NA	0	0	0	0	0	0	0	1	NA
Solyc00g095450.1.1	Ankyrin repeat-containing pro	0	0	0	0	0	0	0	0	0	0	1	NA	0	0	0	0	0	0	0	0	1	NA	0	0	0	0	0	0	0	0	1	NA	0	0	0	0	0	0	0	1	NA
Solyc00g095460.1.1	Isoflavone reductase family p	0	0	0	0	0	0	0	0	0	0	1	NA	0	0	0	0	0	0	0	0	1	NA	0	0	0	0	0	0	0	0	1	NA	0	0	0	0	0	0	0	1	NA
Solyc00g095760.1.1	1-aminocyclopropane-1-carbo	0.4	0.53	0.93	0.28	0.54	0.22	0.62	0.36	0	0.3	0.56	1	0.55	1.2	1	0.92	0.82	0.82	0.47	0.7	0.77	1	4.63	7.56	3.92	5.37	2.34	0.9	1.15	1.46	0.27	1	0.28	0.72	0.3	0.43	0	0.69	0.34	0.8	1
Solyc00g095860.1.1	1-aminocyclopropane-1-carbo	10.6	10.48	11.12	12.08	11.07	7.2	6.28	5.14	5.07	5.92	0.54	0.196566	7.67	18.43	15.92	14.01	11.85	8.08	8.8	9.58	0.68	0.791307	81.85	92.88	76.01	83.58	24.18	28.35	31.98	28.17	0.34	0.348475	6.7	26.95	16.3	16.65	28.58	18.08	23.33	1.4	1
Solyc00g096060.1.1	Ectonucleoside triphosphate (	0	0	0	0	0	0	0	0	0	0	1	NA	0	0	0	0	0	0	0	0	1	NA	0	0	0	0	0	0	0	0	1	NA	0	0	0	0	0	0	0	1	NA
Solyc00g096460.1.1	AT4G08310 protein (Fragmer	11.59	15.08	8.41	7.57	10.66	5.95	6.33	9.02	12.8	8.53	0.8	1	12.44	14.72	9.79	12.32	10.51	10.15	8.41	9.69	0.79	1	7.2	9.44	3.54	6.73	5.86	9.46	10.39	8.57	1.27	1	3.8	37.56	24.68	22.01	13.87	21.89	17.88	0.81	1
Solyc00g097260.1.1	Unknown Protein (AHRD V1)	0	0	0	0	0	0	0	0	0	0	1	NA	0	0	0	0	0	0	0	0	1	NA	0	0	0	0	0	0	0	0	1	NA	0	0	0	0	0	0	0	1	NA
Solyc00g097760.1.1	Ulp1 protease family C-termi	0	0	0	0	0	0	0	0	0	0	1	NA	0	0	0	0	0	0	0	0	1	NA	0	0	0	0	0	0	0	0	1	NA	0	0	0	0	0	0	0	1	NA
Solyc00g097960.1.1	Unknown Protein (AHRD V1)	4.49	6.79	2.12	1.27	3.67	3.99	4.25	7.01	3.54	4.7	1.28	1	3.34	5.46	2.65	3.82	4.05	3.25	1.41	2.9	0.76	1	2.54	5.12	2.23	3.3	0.88	2.72	1.64	1.75	0.53	1	5.1	6.19	0.69	3.99	3.29	3.15	3.22	0.81	1
Solyc00g098560.2.1	GDSL esterase/lipase At5g03	18.24	32.7	19.93	17.07	21.98	29.81	21.38	34.27	17.33	25.7	1.17	1	19.21	30.07	19.03	22.77	26.04	26.94	28.03	27	1.19	0.863933	17.33	16.61	9.85	14.6	9.34	8.13	8.99	8.82	0.6	1	14.11	7.79	6.6	9.5	3.19	6.58	4.88	0.51	0.999777
Solyc00g099160.1.1	Unknown Protein (AHRD V1)	0	0	0	0	0	0	0	0	0	0	1	NA	0	0	0	0	0	0	0	0	1	NA	0	0	0	0	0	0	0	0	1	NA	0	0	0	0	0	0	0	1	NA
Solyc00g099460.1.1	Unknown Protein (AHRD V1)	0	0	0	0	0	0	0	0	0	0	1	NA	0	0	0	0	0	0	0	0	1	NA	0	0	0	0	0	0	0	0	1	NA	0	0	0	0	0	0	0	1	NA
Solyc00g099470.1.1	Predicted CDS Pa_2_8820 (AH	0	0	0	0	0	0	0	0	0	0	1	NA	0	0	0	0	0	0	0	0	1	NA	0	0	0	0	0	0	0	0	1	NA	0	0	0	0	0	0	0	1	NA
Solyc00g099570.1.1	Oligoendopeptidase F homolo	0	0	0	0	0	0	0	0	0	0	1	NA	0	0	0	0	0	0	0	0	1	NA	0	0	0	0	0	0	0	0	1	NA	0	0	0	0	0	0	0	1	NA
Solyc00g099580.1.1	Gamma-glutamyltranspeptida	0.14	0	0	0.2	0.09	0.16	0.23	0.26	0.1	0.19	2.21	1	0	0	0	0	0	0	0	0	1	NA	0	0	0	0	0	0	0	0	1	1	0	0	0	0	0	0	0	1	NA
Solyc00g099880.1.1	Unknown Protein (AHRD V1)	0	0	0	0	0	0	0	0	0	0	1	NA	0	0	0	0	0	0	0	0	1	NA	0	0	0	0	0	0	0	0	1	NA	0	0	0	0	0	0	0	1	NA
Solyc00g100880.2.1	GTP binding protein (AHRD V	0	0	0	0	0	0	0	0	0	0	1	NA	0	0	0	0	0	0	0	0	1	NA	0	0	0	0	0	0	0	0	1	NA	0	0	0	0	0	0	0	1	NA
Solyc00g101700.1.1	Protein kinase (AHRD V1 ***-	0	0	0	0	0	0	0	0	0	0	1	NA	0	0	0	0	0	0	0	0	1	NA	0	0	0	0	0	0	0	0	1	NA	0	0	0	0	0	0	0	1	NA
Solyc00g102000.1.1	Unknown Protein (AHRD V1)	0	0	0	0	0	0	0	0	0	0	1	NA	0	0	0	0	0	0	0	0	1	NA	0	0	0	0	0	0	0	0	1	NA	0	0	0	0	0	0	0	1	NA
Solyc00g102400.2.1	Cc-nbs-lrr, resistance protein	1.06	1.53	2.11	1.97	1.67	1.03	0.63	1.52	1.24	1.1	0.66	0.719699	1.85	1.5	1.17	1.51	0.6	1.27	2.03	1.3	0.86	1	0.59	0.49	0.53	0.54	0.7	0.15	0.1	0.32	0.59	1	0.56	0.43	0.2	0.4	0.24	0.41	0.32	0.82	1
Solyc00g104200.1.1	Nucleoid occlusion protein (A	0	0	0	0	0	0	0	0	0	0	1	NA	0	0	0	0	0	0	0	0	1	NA	0	0	0	0	0	0	0	0	1	NA	0	0	0	0	0	0	0	1	NA
Solyc00g104210.1.1	Possible replication protein A	0	0	0	0	0	0	0	0	0	0	1	NA	0	0	0	0	0	0	0	0	1	NA	0	0	0	0	0	0	0	0	1	NA	0	0	0	0	0	0	0	1	NA
Solyc00g104310.1.1	Unknown Protein (AHRD V1)	0	0	0	0	0	0	0	0	0	0	1	NA	0	0	0	0	0	0	0	0	1	NA	0	0	0	0	0	0	0	0	1	NA	0	0	0	0	0	0	0	1	NA
Solyc00g104320.1.1	Unknown Protein (AHRD V1)	0	0	0	0	0	0	0	0	0	0	1	NA	0	0	0	0	0	0	0	0	1	NA	0	0	0	0	0	0	0	0	1	NA	0	0	0	0	0	0	0	1	NA
Solyc00g104330.1.1	Unknown Protein (AHRD V1)	0	0	0	0	0	0	0	0	0	0	1	NA	0	0	0	0	0	0	0	0	1	NA	0	0	0	0	0	0	0	0	1	NA	0	0	0	0	0	0	0	1	NA
Solyc00g104630.1.1	D-xylulose kinase (AHRD V1 *	0	0	0	0	0	0	0	0	0	0	1	NA	0	0	0	0	0	0	0	0	1	NA	0	0	0	0	0	0	0	0	1	NA	0	0	0	0	0	0	0	1	NA
Solyc00g104730.1.1	Endopolygalacturonase (Frag	0	0	0	0	0	0	0	0	0	0	1	NA	0	0	0	0	0	0	0	0	1	NA	0	0	0	0	0	0	0	0	1	NA	0	0	0	0	0	0	0	1	NA
Solyc00g104740.1.1	Essential cytoplasmic protein	0	0	0	0	0	0	0	0	0	0	1	NA	0	0	0	0	0	0	0	0	1	NA	0	0	0	0	0	0	0	0	1	NA	0	0	0	0	0	0	0	1	NA
Solyc00g104940.1.1	Bax inhibitor-1 like (AHRD V1	0	0	0	0	0	0	0	0	0	0	1	NA	0	0	0	0	0	0	0	0	1	NA	0	0	0	0	0	0	0	0	1	NA	0	0	0	0	0	0	0	1	NA
Solyc00g105340.1.1	Genomic DNA chromosome 5	0	0	0	0	0	0	0	0	0	0	1	NA	0	0	0.36	0.12	0	0	0	0	0.08	1	0	0	0	0	0	0	0	0	1	NA	0.3	0.15	0.32	0.26	0.39	0	0.2	0.76	1
Solyc00g105350.1.1	Mutator-like transposase-like	0	0	0	0	0	0	0	0	0	0	1	NA	0	0.1	0	0.03	0.16	0.06	0	0.07	2.2	1	0	0.09	0	0.03	0	0	0.25	0.08	2.78	1	0.16	0.17	0	0.11	0	0.2	0.1	0.91	1
Solyc00g105750.1.1	Mutator-like transposase (AH	0	0.12	0	0	0.03	0	0	0	0	0	0.33	1	0.12	0	0	0.04	0.09	0.07	0.1	0.09	2.17	1	0	0	0	0	0	0	0	0	1	NA	0	0	0	0	0.12	0	0.06	6	1
Solyc00g106650.1.1	BZIP transcription factor fami	6.16	11.74	17.64	11.2	11.68	25.42	18.75	21.84	10.74	19.19	1.64	0.487187	9.82	10.7	14.12	11.55	14.64	10.02	11.06	11.91	1.03	1	17.06	24.37	12.22	17.88	10.4	18.67	11.54	13.54	0.76	1	11.26	27.47	20.29	19.67	20.13	26.22	23.17	1.18	1
Solyc00g107050.1.1	Nuclear transcription factor Y	0	0	1.07	0	0.27	0	0	0	0	0	0.04	1	0	0	0	0	0	0	0	0	1	NA	0	0	0	0	0	0	0	0	1	NA	0	0	0	0	0	0	0	1	NA
Solyc00g107550.1.1	Unknown Protein (AHRD V1)	0	0	0	0	0	0	0	0	0	0	1	NA	0	0	0	0	0	0	0	0	1	NA	0	0	0	0	0	0	0	0	1	NA	0	0	0	0	0	0	0	1	NA
Solyc00g108550.1.1	Unknown Protein (AHRD V1)	0	0	0	0	0	0	0	0	0	0	1	NA	0	0	0	0	0	0	0	0	1	NA	0	0	0	0	0	0	0.3	0.1	10	1	0	0	0	0	0	0	0	1	NA
Solyc00g109450.1.1	Unknown Protein (AHRD V1)	0	0	0	0	0	0	0	0	0	0	1	NA	0	0	0	0	0	0	0	0	1	NA	0	0	0	0	0	0	0	0	1	NA	0	0	0	0	0	0	0	1	NA
Solyc00g109850.1.1	Unknown Protein (AHRD V1)	0	0	0	0	0	0	0	0	0	0	1	NA	0	0	0	0	0	0	0	0	1	NA	0	0	0	0	0	0	0	0	1	NA	0	0	0	0	0	0	0	1	NA
Solyc00g109860.1.1	Unknown Protein (AHRD V1)	0	0	0	0	0	0	0	0	0	0	1	NA	0	0	0	0	0	0	0	0	1	NA	0	0	0	0	0	0	0	0	1	NA	0	0	0	0	0	0	0	1	NA
Solyc00g110060.1.1	Unknown Protein (AHRD V1)	0	0	0	0	0	0	0	0	0	0	1	NA	0	0	0	0	0	0	0	0	1	NA	0	0	0	0	0	0	0	0	1	NA	0	0	0	0	0	0	0	1	NA

Solyc00g110070.1.1	C2H2 finger domain-containi	0	0	0	0	0	0	0	0	0	0	1	NA	0	0	0	0	0	0	0	0	1	NA	0	0	0	0	0	0	0	0	1	NA	0	0	0	0	0	0	0	1	NA
Solyc00g110370.1.1	Unknown Protein (AHRD V1);	0	0	0.68	0	0.17	0	0	0	0	0	0.06	1	0	0	0	0	0.4	0	0.45	0.28	28.33	1	0	0	0	0	0	0	1.25	0.42	41.67	1	0	0	0	0	0	0	0	1	NA
Solyc00g110870.2.1	Receptor like kinase, RLK	3.77	4.16	2.67	2.13	3.18	3.18	2.14	4.02	3.57	3.23	1.01	1	2.66	0.92	1.78	1.79	0.52	0.62	0.59	0.58	0.32	0.256371	0.12	0.25	0.45	0.27	0.2	0.23	0.11	0.18	0.66	1	0	0	0	0	0	0.13	0.07	6.5	1
Solyc00g111170.1.1	Unknown Protein (AHRD V1);	0	0	0	0	0	0	0	0	0	0	1	NA	0	0	0	0	0	0	0	0	1	NA	0	0	0	0	0	0	0	0	1	NA	0	0	0	0	0	0	0	1	NA
Solyc00g111270.1.1	Unknown Protein (AHRD V1)	0	0	0	0	0	0	0	0	0	0	1	NA	0	0	0	0	0	0	0	0	1	NA	0	0	0	0	0	0	0	0	1	NA	0	0	0	0	0	0	0	1	NA
Solyc00g111370.1.1	Unknown Protein (AHRD V1)	0	0	0	0	0	0	0	0	0	0	1	NA	0	0	0	0	0	0	0	0	1	NA	0	0	0	0	0	0	0	0	1	NA	0	0	0	0	0	0	0	1	NA
Solyc00g111770.1.1	Unknown Protein (AHRD V1)	0	0	0	0	0	0	0	0	0	0	1	NA	0	0	0	0	0	0	0	0	1	NA	0	0	0	0	0	0	0	0	1	NA	0	0	0	0	0	0	0	1	NA
Solyc00g111780.1.1	Unknown Protein (AHRD V1)	0	0	0	0	0	0	0	0	0	0	1	NA	0	0	0	0	0	0	0	0	1	NA	0	0	0	0	0	0	0	0	1	NA	0	0	0	0	0	0	0	1	NA
Solyc00g112180.1.1	Unknown Protein (AHRD V1)	0	0	0	0	0	0	0	1.19	0	0.3	29.75	1	0	0	0	0	0	0	0	0	1	NA	0	0	0	0	0	0	0	0	1	NA	0	0	0	0	0	0	0	1	NA
Solyc00g112190.2.1	Unknown Protein (AHRD V1)	0	0	0	0.54	0.14	0.42	0	0.35	0	0.19	1.43	1	0	0.33	0	0.11	0	0	0	0	0.09	1	0	0	0	0	0	0	0	0	1	NA	0.54	0	0.59	0.38	0	0	0	0.03	1
Solyc00g112490.1.1	Hemagglutinin-related autotr	0	0	0	0	0	0	0	0	0	0	1	NA	0	0	0	0	0	0	0	0	1	NA	0	0	0	0	0	0	0	0	1	NA	0	0	0	0	0	0	0	1	NA
Solyc00g112500.1.1	Outer membrane autotransp	0	0	0	0	0	0	0	0	0	0	1	NA	0	0	0	0	0	0	0	0	1	NA	0	0	0	0	0	0	0	0	1	NA	0	0	0	0	0	0	0	1	NA
Solyc00g112600.1.1	Sugar transport protein 2 (AH	0	0	0	0	0	0	0	0	0	0	1	NA	0	0	0	0	0	0	0	0	1	NA	0	0	0	0	0	0	0	0	1	NA	0	0	0	0	0	0	0	1	NA
Solyc00g112700.1.1	Unknown Protein (AHRD V1)	0	0	0	0	0	0	0	0	0	0	1	NA	0	0	0	0	0	0	0	0	1	NA	0	0	0	0	0	0	0	0	1	NA	0	0	0	0	0	0	0	1	NA
Solyc00g112710.1.1	Unknown Protein (AHRD V1)	0	0	0	0	0	0	0	0	0	0	1	NA	0	0	0	0	0	0	0	0	1	NA	0	0	0	0	0	0	0	0	1	NA	0	0	0	0	0	0	0	1	NA
Solyc00g112810.1.1	V-type H(+)-translocating pyr	0	0	0	0	0	0	0	0	0	0	1	NA	0	0	0	0	0	0	0	0	1	NA	0	0	0	0	0	0	0	0	1	NA	0	0	0	0	0	0	0	1	NA
Solyc00g112820.1.1	Unknown Protein (AHRD V1)	0	0	0	0	0	0	0	0	0	0	1	NA	0	0	0	0	0	0	0	0	1	NA	0	0	0	0	0	0	0	0	1	NA	0	0	0	0	0	0	0	1	NA
Solyc00g112830.1.1	Short-chain dehydrogenase/r	0	0	0	0	0	0	0	0	0	0	1	NA	0	0	0	0	0	0	0	0	1	NA	0	0	0	0	0	0	0	0	1	NA	0	0	0	0	0	0	0	1	NA
Solyc00g112930.2.1	Alpha-N-acetylglucosaminida	0	0	0	0	0	0	0	0	0	0	1	NA	0	0.75	0.73	0.49	2.41	0.45	2.05	1.64	3.32	0.994324	0	0.71	0	0.24	0	0	0	0	0.04	1	0	0	0	0	0	0	0	1	NA
Solyc00g113230.1.1	Unknown Protein (AHRD V1)	0	0	0	0	0	0	0	0	0	0	1	NA	0	0	0	0	0	0	0	0	1	NA	0	0	0	0	0	0	0	0	1	NA	0	0	0	0	0	0	0	1	NA
Solyc00g113330.1.1	Unknown Protein (AHRD V1)	0	0	0	0	0	0	0	0	0	0	1	NA	0	0	0	0	0	0	0	0	1	NA	0	0	0	0	0	0	0	0	1	NA	0	0	0	0	0	0	0	1	NA
Solyc00g113730.1.1	Type VI secretion lipoprotein	0	0	0	0	0	0	0	0	0	0	1	NA	0	0	0	0	0	0	0	0	1	NA	0	0	0	0	0	0	0	0	1	NA	0	0	0	0	0	0	0	1	NA
Solyc00g113740.1.1	Unknown Protein (AHRD V1)	0	0	0	0	0	0	0	0	0	0	1	NA	0	0	0	0	0	0	0	0	1	NA	0	0	0	0	0	0	0	0	1	NA	0	0	0	0	0	0	0	1	NA
Solyc00g113750.1.1	ImcF domain protein (AHRD V	0	0	0	0	0	0	0	0	0	0	1	NA	0	0	0	0	0	0	0	0	1	NA	0	0	0	0	0	0	0	0	1	NA	0	0	0	0	0	0	0	1	NA
Solyc00g114550.1.1	At1g32160/F3C3_6 (AHRD V1	0	0	0	0	0	0	0	0	0	0	1	NA	0	0	0	0	0	0	0	0	1	NA	0	0	0	0	0	0	0	0	1	NA	0	0	0	0	0	0	0	1	NA
Solyc00g116150.1.1	Unknown Protein (AHRD V1)	0	0	0	0	0	0	0	0	0	0	1	NA	0	0	0	0	0	0	0	0	1	NA	0	0	0	0	0	0	0	0	1	NA	0	0	0	0	0	0	0	1	NA
Solyc00g117450.2.1	Short internode related seque	0	0	0	0	0	0	0	0	0	0	1	NA	0	0	0	0	0	0	0	0	1	NA	0	0	0	0	0.23	0	0	0.08	7.67	1	0	0	0	0	0	0	0	1	NA
Solyc00g117650.1.1	RabGAP/TBC domain-contain	0	0	0	0	0	0	0	0	0	0	1	NA	0	0	0	0	0	0	0	0	1	NA	0	0	0	0	0	0	0	0	1	NA	0	0	0	0	0	0	0	1	NA
Solyc00g118680.2.1	Unknown Protein (AHRD V1)	0	0	0	0	0	0	0	0	0	0	1	NA	0	0	1.69	0.56	0	0	0	0	0.02	1	0	0	0	0	0	1.52	0	0.51	50.67	NA	0	0	0	0	0	0	0	1	NA
Solyc00g118690.1.1	NADH-ubiquinone oxidoredu	0.76	0	0	2.15	0.73	0.84	0	0	0	0.21	0.29	1	0	0	1.28	0.43	0.53	0.39	1.19	0.7	1.65	1	0	0	0	0	0	0.57	0	0.19	19	1	0	0.55	1.17	0.57	0	0	0	0.02	1
Solyc00g120120.1.1	Unknown Protein (AHRD V1)	0	0	0	0	0	0	0	0	0	0	1	NA	0	0	0	0	0	0	0	0	1	NA	0	0	0	0	0	0	0	0	1	NA	0	0	0	0	0	0	0	1	NA
Solyc00g120820.1.1	L-fucose H+ symporter perme	0	0	0	0	0	0	0	0	0	0	1	NA	0	0	0	0	0	0	0	0	1	NA	0	0	0	0	0	0	0	0	1	NA	0	0	0	0	0	0	0	1	NA
Solyc00g120830.1.1	Xylose isomerase domain pro	0	0	0	0	0	0	0	0	0	0	1	NA	0	0	0	0	0	0	0	0	1	NA	0	0	0	0	0	0	0	0	1	NA	0	0	0	0	0	0	0	1	NA
Solyc00g120930.1.1	Calmodulin (AHRD V1 ***- B6	0	0	0	0	0	0	0	0	0	0	1	NA	0	0	0	0	0	0	0	0	1	NA	0	0	0	0	0	0	0	0	1	NA	0	0	0	0	0	0	0	1	NA
Solyc00g121230.1.1	Cation/multidrug efflux syste	0	0	0	0	0	0	0	0	0	0	1	NA	0	0	0	0	0	0	0	0	1	NA	0	0	0	0	0	0	0	0	1	NA	0	0	0	0	0	0	0	1	NA
Solyc00g121730.1.1	&aposchromo&apos domain	0	0	0	0	0	0	0	0	0	0	1	NA	0	0	0	0	0	0	0	0	1	NA	0	0	0	0	0	0	0	0	1	NA	0	0	0	0	0	0	0	1	NA
Solyc00g122530.1.1	Calcium/proton antiporter (A	0	0	0	0	0	0	0	0	0	0	1	NA	0	0	0	0	0	0	0	0	1	NA	0	0	0	0	0	0	0	0	1	NA	0	0	0	0	0	0	0	1	NA
Solyc00g123030.1.1	Unknown Protein (AHRD V1)	0	0	0	0	0	0	0	0	0	0	1	NA	0	0	0	0	0	0	0	0	1	NA	0	0	0	0	0	0	0	0	1	NA	0	0	0	0	0	0	0	1	NA
Solyc00g123040.1.1	Unknown Protein (AHRD V1)	0	0	0	0	0	0	0	0	0	0	1	NA	0	0	0	0	0	0	0	0	1	NA	0	0	0	0	0	0	0	0	1	NA	0	0	0	0	0	0	0	1	NA
Solyc00g123240.1.1	Unknown Protein (AHRD V1)	0	0	0	0	0	0	0	0	0	0	1	NA	0	0	0	0	0	0	0	0	1	NA	0	0	0	0	0	0	0	0	1	NA	0	0	0	0	0	0	0	1	NA
Solyc00g124840.1.1	P2 family phage major capsid	0	0	0	0	0	0	0	0	0	0	1	NA	0	0	0	0	0	0	0	0	1	NA	0	0	0	0	0	0	0	0	1	NA	0	0	0	0	0	0	0	1	NA
Solyc00g124850.1.1	Phage capsid scaffolding prot	0	0	0	0	0	0	0	0	0	0	1	NA	0	0	0	0	0	0	0	0	1	NA	0	0	0	0	0	0	0	0	1	NA	0	0	0	0	0	0	0	1	NA
Solyc00g124860.1.1	Terminase ATPase subunit (A	0	0	0	0	0	0	0	0	0	0	1	NA	0	0	0	0	0	0	0	0	1	NA	0	0	0	0	0	0	0	0	1	NA	0	0	0	0	0	0	0	1	NA
Solyc00g125260.1.1	CoA-binding domain protein (	0	0	0	0	0	0	0	0	0	0	1	NA	0	0	0	0	0	0	0	0	1	NA	0	0	0	0	0	0	0	0	1	NA	0	0	0	0	0	0	0	1	NA
Solyc00g125270.1.1	Intracellular protease PfpI far	0	0	0	0	0	0	0	0	0	0	1	NA	0	0	0	0	0	0	0	0	1	NA	0	0	0	0	0	0	0	0	1	NA	0	0	0	0	0	0	0	1	NA
Solyc00g125580.1.1	Ulp1 protease family C-termi	0	0	0	0	0	0.68	0	0	0	0.17	17	1	0	0.27	0.52	0.26	0.21	0	0	0.07	0.27	1	0.25	0	0	0.08	0	0	0	0	0.12	1	0	0	0	0	0	0.27	0.14	13.5	1
Solyc00g125980.1.1	NADH-ubiquinone oxidoredu	1.94	1.73	10.3	8.23	5.55	1.08	0	0.89	2.09	1.02	0.18	0.408286	1.8	1.69	20.49	7.99	2.02	1.51	0.76	1.43	0.18	0.892896	1.57	6.32	11.56	6.48	3.19	1.47	4.24	2.97	0.46	1	1.38	1.41	0	0.93	0.89	0.85	0.87	0.94	1
Solyc00g125990.1.1	NADH-quinone oxidoreductas	0.31	0.27	0	0.87	0.36	0.34	0.48	0.56	0.66	0.51	1.41	1	0	0.53	0.78	0.44	0.21	0.16	0	0.12	0.28	1	1.24	0.75	0.91	0.97	0	0.7	0.89	0.53	0.55	1	0.44	0.67	0	0.37	0	1.35	0.68	1.82	1
Solyc00g126000.1.1	NADH-ubiquinone oxidoredu	0	1.15	4.58	1.83	1.89	1.44	3.06	0	1.86	1.59	0.84	1	1.2	3.37	3.28	2.62	0.9	1.34	1.02	1.09	0.42	1	3.14	1.05	2.57	2.25	2.55	3.92	2.83	3.1	1.38	1	1.84	3.76	1.99	2.53	3.55	2.27	2.91	1.15	1
Solyc00g126010.1.1	Uncharacterized mitochondri	0	3.04	5.37	8.04	4.11	2.53	0.9	1.05	0.82	1.33	0.32	0.718867	2.12	2.97	3.84	2.98	1.58	1.18	0.89	1.22	0.41	0.974398	3.68	1.85	3.39	2.97	2.99	2.59	4.14	3.24	1.09	1	4.85	3.3	0	2.72	3.12	5.98	4.55	1.67	1
Solyc00g126420.1.1	Unknown Protein (AHRD V1)	0	0	0	0	0	0	0	0	0	0	1	NA	0	0	0	0	0	0	0	0	1	NA	0	0	0	0	0	0	0	0	1	NA	0	0	0	0	0	0	0	1	NA
Solyc00g126430.1.1	Methylthioadenosine phosph	0	0	0	0	0	0	0	0	0	0	1	NA	0	0	0	0	0	0	0	0	1	NA	0	0	0	0	0	0	0	0	1	NA	0	0	0	0	0	0	0	1	NA
Solyc00g126440.1.1	Unknown Protein (AHRD V1)	0	0	0	0	0	0	0	0	0	0	1	NA	0	0	0	0	0	0	0	0	1	NA	0	0	0	0	0	0	0	0	1	NA	0	0	0	0	0	0	0	1	NA
Solyc00g127540.1.1	Ribonucleoside-diphosphate r	0	0	0	0	0	0	0	0	0	0	1	NA	0	0	0	0	0	0	0	0	1	NA	0	0	0	0	0	0	0	0	1	NA	0	0	0	0	0	0	0	1	NA
Solyc00g127550.1.1	Ribonucleoside-diphosphate r	0	0	0	0	0	0	0	0	0	0	1	NA	0	0	0	0	0	0	0	0	1	NA	0	0	0	0	0	0	0	0	1	NA	0	0	0	0	0	0	0	1	NA
Solyc00g127560.1.1	Ribonucleoside-diphosphate r	0	0	0	0	0	0	0	0	0	0	1	NA	0	0	0	0	0	0	0	0	1	NA	0	0	0	0	0	0	0	0	1	NA	0	0	0	0	0	0	0	1	NA
Solyc00g127660.2.1	Unknown Protein (AHRD V1)	93.83	134.39	31.13	33.18	73.13	143.4	76.39	113.22	70.52	100.88	1.38	0.9965	103.64	81.56	50.77	78.66	36.6	76.66	127.88	80.38	1.02	1	137.39	52.56	64.03	84.66	52.97	75.56	84.4	70.98	0.84	1	47.94	38.33	54.1	46.79	42.94	33.42	38.18	0.82	1
Solyc00g127670.1.1	Membrane protein (AHRD V1	5.39	8.49	7.34	5.87	6.77	11.99	6.55	6.49	7.15	8.04	1.19	1	5.79	3.25	6.31	5.12	6.04	9.45	7.17	7.55	1.48	0.730819	5.03	4.06	0.82	3.3	4.36	2.83	4.54	3.91	1.18	1	0	0.6	0	0.2	0.38	2.55	1.46	7.33	1
Solyc00g127870.2.1	Phosphatidylinositol N-acetyl	9.76	7.99	8.75	13.8	10.07	8.68	14.79	8.97	11.49	10.98	1.09	0.967871	14.89	14.25	11.87	13.67	4.6	7.48	9.2	7.09	0.52	0.228079	4.42	8.91	5.04	6.12	5.13	3.85	4.55	4.51	0.74	1	0.56	2.55	1.8	1.64	1.07	1.03	1.05	0.64	1
Solyc00g128870.2.1	Nudix hydrolase 1 (AHRD V1	54.83	68.25	57.11	68.47	62.16	67.14	45.32	46.48	57.51	54.11	0.87	0.987146	53	48.04	53.91	51.65	60.31	48.13	55.55	54.66	1.06	1	30.8	35.95	35.29	34.01	31.52	38.64	30.59	33.58	0.99	1	27.41	25.82	19.76	24.33	28.63	39.44	34.03	1.4	1
Solyc00g129670.1.1	Unknown Protein (AHRD V1)	0	0	0	0	0	0	0	0	0	0	1	NA	0	0	0	0	0	0	0	0	1	NA	0	0	0	0	0	0	0	0	1	NA	0	0	0	0	0	0	0	1	NA
Solyc00g129680.1.1	Unknown Protein (AHRD V1)	0	0	0	0	0	0	0	0	0	0	1	NA	0	0	0	0	0	0	0	0	1	NA	0	0	0	0	0	0	0	0	1	NA	0	0	0	0	0	0	0	1	NA
Solyc00g129880.1.1	Unknown Protein (AHRD V1)	0	0	0	0	0	0	0	0	0	0	1	NA	0	0	0	0	0	0	0	0	1	NA	0	0	0	0	0	0	0	0	1	NA	0	0	0	0	0	0	0	1	NA
Solyc00g129980.1.1	Unknown Protein (AHRD V1)	0	0	0	0	0	0	0	0	0	0	1	NA	0	0	0	0	0	0	0	0	1	NA	0	0	0	0	0	0	0	0	1	NA	0	0	0	0	0	0	0	1	NA
Solyc00g130180.1.1	Hypothetical membrane span	0	0	0	0	0	0	0	0	0	0	1	NA	0	0	0	0	0	0	0	0	1	NA	0	0	0	0	0	0	0	0	1	NA	0	0	0	0	0	0	0	1	NA
Solyc00g130190.1.1	Nicotinate phosphoribosyltra	0	0	0	0	0	0	0	0	0	0	1	NA	0	0	0	0	0	0	0	0	1	NA	0	0	0	0	0	0	0	0	1	NA	0	0	0	0	0	0	0	1	NA
Solyc00g130290.1.1	Urea ABC transporter permea	0	0	0	0	0	0	0	0	0	0	1	NA	0	0	0	0	0	0	0	0	1	NA	0	0	0	0	0	0	0	0	1	NA	0	0	0	0	0	0	0	1	NA
Solyc00g130690.1.1	Membrane protein involved i	0	0	0	0	0	0	0	0	0	0	1	NA	0	0	0	0	0	0	0	0	1	NA	0	0	0	0	0	0	0	0	1	NA	0	0	0	0	0	0	0	1	NA
Solyc00g130700.1.1	Unknown Protein (AHRD V1)	0	0	0	0	0	0	0	0	0	0	1	NA	0	0	0	0	0	0	0	0	1	NA	0	0	0	0	0	0	0	0	1	NA	0	0	0	0	0	0	0	1	NA
Solyc00g130800.1.1	Peptidase C14 caspase cataly	0	0	0	0	0	0	0	0	0	0	1	NA	0	0	0	0	0	0	0	0	1	NA	0	0	0	0	0	0	0	0	1	NA	0	0	0	0	0	0	0	1	NA
Solyc00g130810.1.1	Cationic outer membrane pro	0	0	0	0	0	0	0	0	0	0	1	NA	0	0	0	0	0	0	0	0	1	NA	0	0	0	0	0	0	0	0	1	NA	0	0	0	0	0	0	0	1	NA
Solyc00g131610.1.1	Disease resistance response (	0	0	0	0	0	0	0	0	0	0	1	NA	0	0	0	0	0	0	0	0	1	NA	0	0	0	0	0	0	0	0	1	NA	0	0	0	0	0	0	0	1	NA
Solyc00g131710.1.1	Pol polyprotein (AHRD V1 *-*	0	0	0	0	0	0	0	0	0	0	1	NA	0	0	0	0	0	0	0	0	1	NA	0	0	0	0	0	0	0	0	1	NA	0	0	0	0	0	0	0	1	NA
Solyc00g133110.1.1	Peptidase M14 carboxypeptid	0	0	0	0	0	0	0	0	0	0	1	NA	0	0	0	0	0	0	0	0	1	NA	0	0	0	0	0	0	0	0	1	NA	0	0	0	0	0	0	0	1	NA
Solyc00g133710.1.1	Unknown Protein (AHRD V1)	0	0	0	0	0	0	0	0	0	0	1	NA	0	0	0	0	0	0	0	0	1	NA	0	0	0	0	0	0	0	0	1	NA	0	0	0	0	0	0	0	1	NA
Solyc00g133720.1.1	Unknown Protein (AHRD V1)	0	0	0	0	0	0	0	0.22	0	0.06	5.5	1	0.23	0	0	0.08	0	0	0	0	0.13	1	0	0	0	0	0	0	0	0	1	NA	0	0	0	0	0	0	0	1	NA
Solyc00g134620.1.1	Transferase family protein (A	0	0.2	0	0	0.05	0	0	0	0	0	0.2	1	0	0	0	0	0	0	0	0	1	NA	0	0	0	0	0	0	0	0	1	NA	0	0	0	0	0	0	0	1	NA
Solyc00g134820.1.1	Type IV prepilin leader peptid	0	0	0	0	0	0	0	0	0	0	1	NA	0	0	0	0	0	0	0	0	1	NA	0	0	0	0	0	0	0	0	1	NA	0	0	0	0	0	0	0	1	NA
Solyc00g134830.1.1	Dephospho-CoA kinase (AHRD	0	0	0	0	0	0	0	0	0	0	1	NA	0	0	0	0	0	0	0	0	1	NA	0	0	0	0	0	0	0	0	1	NA	0	0	0	0	0	0	0	1	NA
Solyc00g134840.1.1	Oligopeptide transporter OPT	0	0	0	0	0	0	0	0	0	0	1	NA	0	0	0	0	0	0	0	0	1	NA	0	0	0	0	0	0	0	0	1	NA	0	0	0	0	0	0	0	1	NA
Solyc00g135040.1.1	Transcriptional regulator Ars	0	0	0	0	0	0	0	0	0	0	1	NA	0	0	0	0	0	0	0	0	1	NA	0	0	0	0	0	0	0	0	1	NA	0	0	0	0	0	0	0	1	NA
Solyc00g135050.1.1	Transcriptional regulator Lys	0	0	0	0	0	0	0	0	0	0	1	NA	0	0	0	0	0	0	0	0	1	NA	0	0	0	0	0	0	0	0	1	NA	0	0	0	0	0	0	0	1	NA
Solyc00g135060.1.1	Zinc-binding alcohol dehydro	0	0	0	0	0	0	0	0	0	0	1	NA	0	0	0	0	0	0	0	0	1	NA	0	0	0	0	0	0	0	0	1	NA	0	0	0	0	0	0	0	1	NA
Solyc00g135260.1.1	Transferase family protein (A	0	0	0	0	0	0	0	0	0	0	1	NA	0	0	0	0	0	0	0	0	1	NA	0	0	0	0	0	0	0	0	1	NA	0	0	0	0	0	0	0	1	NA
Solyc00g136260.1.1	Ring H2 finger protein (AHRD	148.31	164.57	289.74	238.4	210.25	170.22	202.25	289.11	165.6	206.8	0.98	1	181.41	182.09	228.17	197.22	108.55	149.6	120.34	126.16	0.64	0.166516	46.68	63.96	26.77	45.8	44.92	19.34	46.32	36.86	0.8	1	8.22	3.5	2.22	4.65	2.2	3.38	2.79	0.6	1
Solyc00g136560.2.1	Undecaprenyl pyrophosphate	3.58	1.77	5.16	1.12	2.91	4.42	2.2	4.38	4.28	3.82	1.31	0.976183	5.18	9.33	5.37	6.63	22.6	10.29	13.12	15.34	2.31	0.299503	18.3	30.44	14.99	21.24	47.52	24.4	22.3	31.41	1.48	1	5.09	13.85	6.72	8.55	13.82	4.18	9	1.05	1
Solyc00g138060.2.1	2-oxoglutarate-dependent di	0	0	0	0	0	0	0	0	0	0	1	NA	0	0	0	0	0	0	0	0	1	NA	0	0	0	0	0	0	0	0	1	NA	0	0	0	0	0	0	0	1	NA
Solyc00g139760.1.1	Filamentous hemagglutinin fa	0	0	0	0	0	0	0	0	0	0	1	NA	0	0	0	0	0	0	0	0	1	NA	0	0	0	0	0	0	0	0	1	NA	0	0	0	0	0	0	0	1	NA
Solyc00g140060.2.1	Tripeptidyl peptidase II (AHRD	32.99	37.63	33.96	37.33	35.48	19	25.09	21.78	35.74	25.4	0.72	0.604664	38.77	48.75	60.28	49.27	52.83	52.17	39.12	48.04	0.98	1	24.95	28.59	22.62	25.39	23.05	18.18	30.16	23.8	0.94	1	23.88	16.99	22.59	21.15	38.7	42.33	40.52	1.92	0.349274
Solyc00g141360.1.1	Lrr, resistance protein fragm	0	0	0	0	0	0	0.68	0.53	0.82	0.51	50.75	0.462739	0	0	0	0	0	0	0.23	0.08	7.67	1	0	0	0	0	0	0	0	0	1	NA	0	0	0	0	0	0	0	1	NA
Solyc00g141560.1.1	Unknown Protein (AHRD V1);	0	0	0	0	0	0	0	0	0	0	1	NA	0.79	0.37	0	0.39	0	0.66	0	0.22	0.57	1	0	0	0	0	0.56	0.32	0	0.29	29.33	1	0	0	0	0	0	0	0	1	NA
Solyc00g142160.1.1	Unknown Protein (AHRD V1)	0	0	0	0	0	0	0	0	0	0	1	NA	0	0	0.53	0.18	0	0.32	0.49	0.27	1.53	1	0	0	0	0	0	0	0.91	0.3	30.33	1	0	0	0	0	0	0	0	1	NA
Solyc00g142170.2.1	RNA-dependent RNA polyme	0	0	0	0	0	0	0	0	0	0	1	NA	0	0	0	0	0	0	0	0	1	NA	0.35	0.35	0	0.23	0	0.33	0.47	0.27	1.14	1	1.23	0.47	0.33	0.68	0.2	0	0.1	0.15	1
Solyc00g143770.1.1	Unknown Protein (AHRD V1)	12.69	21.83	13.97	26.32	18.7	7.52	6.68	8.55	10.32	8.27	0.44	0.191096	14.95	9.56	15	13.17	7.63	10.07	10.63	9.44	0.72	0.97732	14.35	6.2	14.27	11.61	7.78	10.26	8.01	8.68	0.75	1	8.42	9.83	11.71	9.99	10.84	9.64	10.24	1.03	1
Solyc00g144470.1.1	Serine/threonine-protein pho	0	0	0	0	0	0	0	0	0	0	1	NA	0	0	0	0	0	0	0	0	1	NA	0	0	0	0	0	0	0	0	1	NA	0	0	0	0	0	0	0	1	NA
Solyc00g145170.1.1	Proteinase inhibitor II (AHRD	740.44	586.32	374.46	351.73	513.24	781.52	1043.48	330.43	603.46	689.72	1.34	0.860671	554.43	358.52	292.24	401.73	190.06	233.13	134.14	185.78	0.46	0.25447	8.95	13.53	5.95	9.48	17.58	9.79	22.19	16.52	1.74	1	2.62	1.68	2.13	2.14	0.42	0	0.21	0.1	0.854307
Solyc00g147570.2.1	Gelsolin (Fragment) (AHRD V	0	0	0	0	0	0	0	0	0	0	1	NA	0	0	0	0	0	0	0	0	1	NA	0	0	0	0	0	0	0	0	1	NA	0	0	0	0	0	0	0	1	NA
Solyc00g147770.1.1	HAT family dimerisation dom	2.18	2.53	2.77	2.13	2.4	2.42	2.78	2.16	2.64	2.5	1.04	1	2.8	5.68	4.95	4.48	3.48	3.21	5.26	3.98	0.89	1	1.89	1.36	1.5	1.58	1.76	1.52	1.71	1.66	1.05	1	1.9	4.5	3.86	3.42	2.3	3.67	2.98	0.87	1
Solyc00g149970.2.1	Pho1-like protein (AHRD V1 *	1.74	4.64	2.05	0	2.11	0.77	0	4.47	1.74	1.75	0.83	1	2.91	0.91	2.93	2.25	0.72	0.72	2.46	1.3	0.58	0.900689	2.81	1.13	1.38	1.77	1.14	0.53	1.52	1.06	0.6	1	0	4.03	3.2	2.41	0.64	0.91	0.78	0.32	1
Solyc00g153680.1.1	Serine/threonine protein kina	1.1	2.21	2.61	0.78	1.68	2.15	1.53	2.54	1.19	1.85	1.11	1	2.57	0.96	0.47	1.33	0.38	0.86	0.22	0.49	0.36	0.911351	0.45	1.12	0.27	0.61	0	0.21	0.2	0.14	0.22	1	0.39	0.2	0	0.2	0.25	0.48	0.36	1.86	1
Solyc00g153980.1.1	Unknown Protein (AHRD V1)	0	0	0	0	0	0	0	0	0	0	1	NA	0	0	0	0	0	0	0	0	1	NA	0	0	0	0	0	0	0	0	1	NA	1.67	0	0	0.56	0	0	0	0.02	1
Solyc00g154480.1.1	Cellulose synthase-like protei	0	0	0	0	0	0	0	0	0	0	1	NA	0	0	0	0	0	0	0	0	1	NA	0	0	0	0	0	0	0	0	1	NA	0	0	0	0	0	0	0	1	NA
Solyc00g154980.1.1	Ethylene insensitive 3 class tr	0	0	0	0	0	0	0	0	0	0	1	NA	0	0	0	0	0	0	0	0	1	NA	0	0	0	0	0	0	0	0	1	NA	0	0	0	0	0	0	0	1	NA
Solyc00g156980.2.1	Choline dehydrogenase (AHR	37.93	57.61	13.13	21.58	32.56	31.62	20.83	52.32	37.3	35.52	1.09	1	23.4	22.58	17.76	21.25	2.86	4.91	7.45	5.07	0.24	1.47E-05	0	0	0	0	0	0	0	0	1	NA	0	0	0	0	0	0	0	1	NA
Solyc00g157080.1.1	Pto kinase interactor-like pro	0	0	0	0	0	0	0	0	0	0	1	NA	0	0	0	0	0	0	0	0	1	NA	0	0	0	0	0	0	0	0	1	NA	0	0	0	0	0	0	0	1	NA
Solyc00g157180.2.1	cDNA clone J033046E14 full i	6.33	3.68	8.33	9.64	7	5.95	3.84	5.15	2.97	4.48	0.64	0.625941	5.89	7.62	4.11	5.87	3.71	0.63	1.15	1.83	0.31	0.060697	5.51	8.93	6.04	6.83	2.08	2.58	1.77	2.14	0.31	0.626059	11.42	6.54	10.48	9.48	2.45	1.28	1.87	0.2	0.05255

Solyc00g160780.2.1	Unknown Protein (AHRD V1)	4.26	10.73	2.51	2.01	4.88	8.67	6.16	8.48	4.58	6.97	1.43	1	1.98	3.08	0.6	1.89	0.49	2.2	3.34	2.01	1.07	1	0	0	0	0	0	0	0	0	1	NA	0	0	0	0	0	0	0	1	NA
Solyc00g163580.1.1	O-methyltransferase (AHRD V	0	0	0	0	0	0	0	0	0	0	1	NA	0	0	0	0	0	0	0	0	1	NA	0	0	0	0	0	0	0	0	1	NA	0	0	0	0	0	0	0	1	NA
Solyc00g164580.1.1	Unknown Protein (AHRD V1)	0	0	0	0	0	0	0.29	0	0	0.07	7.25	1	0	0.32	0	0.11	0	0	0	0	0.09	1	0.6	0	1.1	0.57	0	0.28	0	0.09	0.16	1	0	0.54	0.57	0.37	0.34	0	0.17	0.46	1
Solyc00g164680.1.1	ABC transporter G family mer	12.5	7.29	14.58	12.16	11.63	14.19	17.29	12.72	12.44	14.16	1.22	0.609756	12.87	14.6	15.21	14.23	29.26	20.66	12.35	20.76	1.46	0.704874	12.05	20.25	12.54	14.95	21.36	13.24	16.84	17.15	1.15	1	20.06	20.98	18.6	19.88	22.02	21.09	21.55	1.08	1
Solyc00g165080.1.1	Unknown Protein (AHRD V1)	0	0	0	0	0	0	0	0	0	0	1	NA	0	0	0	0	0	0	0	0	1	NA	0	0	0	0	0	0	0	0	1	NA	0	0	0	0	0	0	0	1	NA
Solyc00g165090.1.1	Unknown Protein (AHRD V1)	0	0	0	0	0	0	0	0	0	0	1	NA	0	0	0	0	0	0	0	0	1	NA	0	0	0	0	0	0	0	0	1	NA	0	0	0	0	0	0	0	1	NA
Solyc00g165190.1.1	Unknown Protein (AHRD V1)	0	0	0	0	0	0	0	0	0	0	1	NA	0	0	0	0	0	0	0	0	1	NA	0	0	0	0	0	0	0	0	1	NA	0	0	0	0	0	0	0	1	NA
Solyc00g166190.1.1	Unknown Protein (AHRD V1)	0	0	0	0	0	0	0	0	0	0	1	NA	0	0	0	0	0	0	0	0	1	NA	0	0	0	0	0	0	0	0	1	NA	0	0	0	0	0	0	0	1	NA
Solyc00g166490.1.1	Ulp1 protease family C-termi	0.17	0.16	0.21	0	0.14	0.78	0.55	0.16	0	0.37	2.76	0.951897	0	0.15	0.59	0.25	0	0	0	0	0.04	0.772021	0	0	0	0	0.11	0	0	0.04	3.67	1	0	0.25	0.27	0.17	0	0	0	0.06	1
Solyc00g166690.1.1	Unknown Protein (AHRD V1)	0	0	0	0	0	0	0	0	0	0	1	NA	0	0	0	0	0	0	0.27	0.09	9	1	0	0	0	0	0	0	0	0	1	NA	0	0	0	0	0	0	0	1	NA
Solyc00g167290.1.1	Single-stranded-DNA-specific	0	0	0	0	0	0	0	0	0	0	1	NA	0	0	0	0	0	0	0	0	1	NA	0	0	0	0	0	0	0	0	1	NA	0	0	0	0	0	0	0	1	NA
Solyc00g167300.1.1	GH3 auxin-responsive promot	0	0	0	0	0	0	0	0	0	0	1	NA	0	0	0	0	0	0	0	0	1	NA	0	0	0	0	0	0	0	0	1	NA	0	0	0	0	0	0	0	1	NA
Solyc00g167800.1.1	Unknown Protein (AHRD V1)	0	0	0	0	0	0	0	0	0	0	1	NA	0	0	0	0	0	0	0	0	1	NA	0	0	0	0	0	0	0	0	1	NA	0	0	0	0	0	0	0	1	NA
Solyc00g168200.1.1	Unknown Protein (AHRD V1)	11.47	25.53	33.84	20.29	22.78	15.94	11.32	31.65	12.36	17.82	0.78	1	16.01	7.48	19.38	14.29	19.89	19.31	22.54	20.58	1.44	1	2.32	7.01	5.69	5.01	3.77	0	4.18	2.65	0.53	1	12.23	2.08	8.82	7.71	2.63	2.51	2.57	0.33	1
Solyc00g170200.1.1	Hydrolase alpha/beta fold far	0	0	0	0	0	1.15	0	0	0	0.29	28.75	1	0	0	0	0	0	0.27	0	0.09	9	1	0.83	0.42	0	0.42	0	0.39	0.38	0.26	0.62	1	0	0	0	0	0	0	0	1	NA
Solyc00g170210.2.1	Unknown Protein (AHRD V1)	79.5	51.19	87.81	78.96	74.36	58.29	32.05	74.65	57.08	55.52	0.75	0.804881	83.39	58.82	64.29	68.83	49.28	50.8	63.81	54.63	0.79	0.972234	58.77	39.97	83.95	60.9	42.23	53.85	40.68	45.59	0.75	1	72.15	34.4	49.42	51.99	44.91	53.39	49.15	0.95	1
Solyc00g170510.1.1	Pectinesterase (AHRD V1 ***	0	0.2	1.87	0.32	0.6	0	0.36	0	0	0.09	0.15	0.862525	0	0	0	0	0	0	0	0	1	NA	0	0	0	0	0	0	0	0	1	NA	0	0	0	0	0	0	0	1	NA
Solyc00g171710.1.1	Unknown Protein (AHRD V1);	0	0	0	0	0	0	0	0	0	0	1	NA	0	0	0	0	0	0	0	0	1	NA	0	0	0	0	0	0	0	0	1	NA	0	0	0	0	0	0	0	1	NA
Solyc00g171810.2.1	ATP synthase subunit a (AHR	1.73	1.88	4.54	3.26	2.85	4.49	0.61	2.12	1.8	2.26	0.79	1	1.25	4.18	5.2	3.54	5.74	4.78	4.08	4.87	1.37	0.875932	2.49	2.82	5.54	3.62	3.03	1.31	3.22	2.52	0.7	1	12.03	10.2	10.06	10.76	5.11	8.77	6.94	0.64	1
Solyc00g172410.1.1	DNA binding protein-like (AH	0	0	0	0	0	0	0	0	0	0	1	NA	0	0	0	0	0	0	0	0	1	NA	0	0	0	0	0	0	0	0	1	NA	0	0	0	0	0	0	0	1	NA
Solyc00g173430.1.1	Nucleic acid binding protein (	0	0	0	0	0	0	0	0	0	0	1	NA	0	0	0	0	0	0	0	0	1	NA	0	0	0	0	0	0	0	0	1	NA	0	0	0	0	0	0	0	1	NA
Solyc00g174330.2.1	Pathogenesis related protein	3.93	0.7	13.9	175.52	48.51	6.11	19.22	10.83	9.59	11.44	0.24	0.982067	8.04	6.14	9.95	8.04	221.15	24.8	5.55	83.83	10.42	0.794054	21.57	7.68	9.35	12.87	40.24	6.55	16.02	20.94	1.63	1	0	1.14	0	0.38	0	5.51	2.75	7.25	1
Solyc00g174340.1.1	Pathogenesis-related protein	2.06	0.73	7.78	113.15	30.93	1.83	10.09	9.48	5.33	6.68	0.22	0.974223	3.84	4.3	8.01	5.38	150.42	14.94	2.59	55.98	10.4	0.816675	10.99	4.37	4.91	6.76	23.29	3.13	10.22	12.21	1.81	1	0	0.3	0	0.1	0	2.89	1.45	14.45	1
Solyc00g175240.1.1	Ulp1 protease family C-termi	0	0	0	0	0	0	0	0	0	0	1	NA	0	0	0	0	0	0	0	0	1	NA	0	0	0	0	0	0	0	0	1	NA	0	0	0	0	0	0	0	1	NA
Solyc00g178340.1.1	Branched-chain-amino-acid a	0	0	0	0	0	0	0	0	0	0	1	NA	0.3	0	0.27	0.19	0	0.33	0	0.11	0.58	1	0	0	0	0	0	0	0	0	1	NA	0	0	0	0	0	0	0	1	NA
Solyc00g179240.1.1	MADS-box transcription facto	0	0	0	0	0	0	0.95	0	0	0.24	23.75	1	0	0	0	0	0	0	0	0	1	NA	0	0	0	0	0	0	0	0	1	NA	0	0	0	0	0	0	0	1	NA
Solyc00g179640.1.1	Unknown Protein (AHRD V1)	0	0	0	0	0	0	0	0	0	0	1	NA	0	0	0	0	0.64	0	0	0.21	21.33	1	0	0	0	0	0	0	0	0	1	NA	0	0	0	0	0	0	0	1	NA
Solyc00g179840.1.1	Unknown Protein (AHRD V1)	0	0	0	0	0	0	0	0	0	0	1	NA	0	0	0	0	0	0	0	0	1	NA	0	0	0	0	0	0	0	0	1	NA	0	0	0	0	0	0	0	1	NA
Solyc00g180440.1.1	50S ribosomal protein L20 ch	0	0	0	0	0	0	0	0	0	0	1	NA	0	0	0	0	0	0	0	0	1	NA	0	0	0	0	0	0	0	0	1	NA	0	0	0	0	0	0	0	1	NA
Solyc00g180840.1.1	Unknown Protein (AHRD V1)	0	0.25	0.33	0	0.15	0	0	0	0.2	0.05	0.34	1	0	0.25	0	0.08	0	0	0	0	0.12	1	0.23	0.46	0	0.23	0	0	0	0	0.04	1	0	0	0	0	0	0	0	1	NA
Solyc00g181240.1.1	Unknown Protein (AHRD V1)	0	0.98	0	0	0.24	0	0	0	0	0	0.04	1	0	0	0	0	0	0	0	0	1	NA	0	0	0	0	0	0	0	0	1	1	0	0	0	0	0	0	0	1	NA
Solyc00g181250.2.1	Unknown Protein (AHRD V1)	0	0	0	0	0	0	0	0	0	0	1	NA	0	0	0	0	0	0	0	0	1	NA	0	0	0	0	0	0	0	0	1	1	0.65	0	0	0.22	0	0	0	0.05	1
Solyc00g183050.1.1	FYVE zinc finger containing pr	0	0	0	0	0	1.83	1.3	4.55	0	1.92	192	0.790538	0	0	0	0	2.29	0	1.3	1.2	119.67	1	2.66	0	0	0.89	0	0	0	0	0.01	1	0	1.2	7.61	2.94	0	0	0	0	1
Solyc00g183550.1.1	Unknown Protein (AHRD V1)	0	0	0	0	0	0	0	0	0	0	1	NA	0	0	0	0	0	0	0	0	1	NA	0	0	0	0	0	0	0	0	1	NA	0	0	0	0	0	0	0	1	NA
Solyc00g184350.2.1	Genomic DNA chromosome 5	13.83	14.2	15.06	13.55	14.16	11.83	12.18	9.29	9.93	10.81	0.76	0.904262	8.91	20.81	13.03	14.25	12.55	15.43	14.22	14.07	0.99	1	15.04	12.57	16.37	14.66	8.74	11.29	10.08	10.04	0.68	1	11.35	12.37	10.64	11.45	12.18	17.26	14.72	1.29	1
Solyc00g185750.1.1	Dual specificity phosphatase (	0	0	0	0	0	0	0	0	0	0	1	NA	0	0	0	0	0	0	0	0	1	NA	0	0	0	0	0	0	0	0	1	NA	0	0	0	0	0	0	0	1	NA
Solyc00g185850.1.1	Histone-lysine N-methyltrans	0	0	0	0	0	0	0	0	0	0	1	NA	0	0	0	0	0	0	0	0	1	NA	0	0	0	0	0	0	0	0	1	NA	0	0	0	0	0	0	0	1	NA
Solyc00g186050.1.1	Unknown Protein (AHRD V1)	0	0.33	0	0	0.08	0.41	0	0	0	0.1	1.24	1	0	0	0	0	0	0	0	0	1	NA	0	0	0	0	0	0	0	0	1	NA	0	0	0	0	0	0	0	1	NA
Solyc00g187050.2.1	Leucyl aminopeptidase (AHRD	35.25	22.38	69.88	10.27	34.45	29.25	15.25	17.04	37.78	24.83	0.72	1	24.23	2.7	5.69	10.87	3.06	1.01	1.22	1.76	0.16	0.698555	0.21	5.28	0.64	2.04	2.04	0.1	0.94	1.03	0.5	1	0.18	0.09	0	0.09	0	0	0	0.11	1
Solyc00g188250.1.1	Mutator-like transposase-like	0.49	0.87	2.88	0	1.06	0.54	0.77	0.45	0.35	0.53	0.5	1	0.45	2.12	0.41	0.99	1.02	1.01	1.15	1.06	1.07	1	0	0.4	0.49	0.3	0.96	0	0.36	0.44	1.48	1	0	0.35	0.75	0.37	0.45	0	0.23	0.61	1
Solyc00g188750.1.1	Unknown Protein (AHRD V1)	0	0	0	0	0	0	0	0	0	0	1	NA	0	0	0	0	0	0	0	0	1	NA	0	0	0	0	0	0	0	0	1	NA	0	0	0	0	0	0	0	1	NA
Solyc00g188850.2.1	Glycosyltransferase CAZy fam	0	0	0	0	0	0	0	0	0	0	1	NA	0	0	0	0	0	0	0	0	1	NA	0	0	0	0	0	0	0	0	1	NA	0	0	0	0	0	0	0	1	NA
Solyc00g192150.2.1	Mutator-like transposase (AH	0	0	0	0	0	0	0	0	0	0	1	NA	0	0	0	0	0	0	0	0	1	NA	0	0	0.12	0.04	0	0	0	0	0.25	NA	0	0	0	0	0	0	0	1	NA
Solyc00g195350.1.1	Serine/threonine-protein pho	0	0	0	0	0	0	0	0	0	0	1	NA	0	0	0	0	0	0	0	0	1	NA	0	0	0	0	0	0	0	0	1	NA	0	0	0	0	0	0	0	1	NA
Solyc00g195360.1.1	Serine/threonine-protein pho	0	0	0	0	0	0	0	0	0	0	1	NA	0	0	0	0	0	0	0	0	1	NA	0	0	0	0	0	0	0	0	1	NA	0	0	0	0	0	0	0	1	NA
Solyc00g196060.2.1	Auxin response factor 6 (AHR	64.85	60.04	69.26	66.43	65.14	41.64	58.26	30.16	58.33	47.1	0.72	0.530724	75.41	67.07	66.58	69.69	60.75	54.81	57.56	57.71	0.83	0.913934	57.06	63.63	53.64	58.11	49.34	51.84	54.86	52.01	0.9	1	44.25	25.72	25.22	31.73	38.55	46.89	42.72	1.35	0.962516
Solyc00g196460.1.1	Unknown Protein (AHRD V1)	0	0	0	0	0	0	0	0	0	0	1	NA	0	0	0	0	0	0.29	0	0.1	9.67	1	0	0	0	0	0.37	0	0	0.12	12.33	1	0	0	0	0	0	0	0	1	NA
Solyc00g198360.1.1	Zinc finger CCCH domain-con	0	0	0	0	0	0	0	0	0	0	1	NA	0	0	0	0	0	0	0	0	1	NA	0	0	0	0	0	0	0	0	1	NA	0	0	0	0	0	0	0	1	NA
Solyc00g199960.1.1	Unknown Protein (AHRD V1)	0	0	0	0	0	0	0	0	0	0	1	NA	0	0	0	0	0	0	0	0	1	NA	0	0	0	0	0	0	0	0	1	NA	0	0	0	0	0	0	0	1	NA
Solyc00g201060.1.1	Unknown Protein (AHRD V1)	0	0	0	0	0	0	0	0	0	0	1	NA	0	0	0	0	0	0	0	0	1	NA	0	0	0	0	0	0	0	0	1	NA	0	0	0	0	0	0	0	1	NA
Solyc00g201160.2.1	Receptor protein kinase (AHR	0.25	0.67	0	0	0.23	0	0	0.23	0	0.06	0.25	0.967585	0	0.22	0	0.07	0.17	0.26	0.2	0.21	2.86	1	0	0.2	0.5	0.23	0.33	0	0	0.11	0.47	1	0	0.73	0	0.24	0.23	0.22	0.23	0.92	1
Solyc00g201460.1.1	Ulp1 protease family C-termi	0	0	0	0	0	0	0	0	0	0	1	NA	0	0	0	0	0	0	0	0	1	NA	0	0	0	0	0	0	0	0	1	NA	0	0	0	0	0	0	0	1	NA
Solyc00g201760.1.1	Unknown Protein (AHRD V1)	0	0	0	0	0	0	0	0	0	0	1	NA	0	0	0	0	0	0	0	0	1	NA	0	0	0	0	0	0	0	0	1	NA	0	0	0	0	0	0	0	1	NA
Solyc00g202560.1.1	Beta-1 3-glucanase (AHRD V1	0	0	0	0	0	0	0	0	0	0	1	NA	0	0	0	0	0	0	0	0	1	NA	0	0	0	0	0	0	0	0	1	NA	0	0	0	0	0	0	0	1	NA
Solyc00g203660.1.1	Ribulose bisphosphate carbox	0	0	0	0	0	0	0	0	0	0	1	NA	0	0	0	0	0	0	0	0	1	NA	0	0	0	0	0	0	0	0	1	NA	0	0	0	0	0	0	0	1	NA
Solyc00g206460.1.1	Os06g0220000 protein (Frag	12.32	12.11	7.27	18.52	12.55	9.7	12.36	5.19	5.34	8.15	0.65	0.705166	2.39	4.46	12.79	6.55	2.31	0.93	2.02	1.75	0.27	0.587671	0.41	1.46	6.88	2.92	5.4	1.75	0.37	2.51	0.86	1	58.03	36.54	62.76	52.44	17.39	24.98	21.19	0.4	0.231048
Solyc00g207960.1.1	Unknown Protein (AHRD V1);	0	0	0.52	0	0.13	0.49	0	0.41	0	0.22	1.73	1	0	0.38	0.37	0.25	0.31	0	0.35	0.22	0.88	1	0	0	0	0	0.29	0.34	1.29	0.64	64	1	0	0	0	0	0	0	0	1	NA
Solyc00g209460.1.1	GDSL esterase/lipase At1g71	1.19	2.12	2.1	1.68	1.77	0	1.88	2.73	2.13	1.68	0.95	1	2.76	1.03	1.51	1.77	3.3	2.46	3.27	3.01	1.7	0.964678	0.96	1.45	0.59	1	1.95	1.8	0	1.25	1.25	1	0.85	0.86	0	0.57	0	1.04	0.52	0.91	1
Solyc00g209560.1.1	Ulp1 protease family C-termi	0	0	0	0	0	0	0	0	0	0	1	NA	0	0	0	0	0	0	0	0	1	NA	0	0	0	0	0	0	0	0	1	NA	0	0	0	0	0	0	0	1	NA
Solyc00g210860.1.1	Unknown Protein (AHRD V1)	0	0	0	0	0	0	0	0	0	0	1	NA	0	0	0	0	0	0	0	0	1	NA	0	0	0	0	0	0	0	0	1	NA	0	0	0	0	0	0	0	1	NA
Solyc00g212260.1.1	GH3 family protein (AHRD V1	0.09	0	0	0.13	0.06	0.1	0.15	0	0.13	0.1	1.73	1	0.09	0	0.16	0.08	0.13	0.05	0.14	0.11	1.28	1	0	0	0	0	0	0	0	0	1	1	0.13	0	0	0.04	0.08	0	0.04	0.92	1
Solyc00g213960.1.1	Unknown Protein (AHRD V1)	0	0	0	0	0	0	0	0	0	0	1	NA	0	0	0	0	0	0	0	0	1	NA	0	0	0	0	0	0	0	0	1	NA	0	0	0	0	0	0	0	1	NA
Solyc00g215560.1.1	Unknown Protein (AHRD V1)	0.67	1.19	0	0	0.46	0	0.53	0	0	0.13	0.28	1	2.48	1.16	0.56	1.4	1.85	1.38	1.05	1.43	1.02	1	2.15	1.09	1.98	1.74	2.19	0	1.46	1.22	0.7	1	3.79	2.9	1.02	2.57	3.05	0.58	1.81	0.71	1
Solyc00g217560.1.1	Transcription factor CYCLOIDE	0	0	0	0	0	0	0	0	0	0	1	NA	0	0	0	0	0	0	0	0	1	NA	0	0	0	0	0	0	0	0	1	NA	0	0	0	0	0	0	0	1	NA
Solyc00g217960.1.1	Ring H2 finger protein (AHRD	12.93	9.12	13.67	11.35	11.77	13.38	13.02	3.69	4.8	8.72	0.74	0.878254	3.73	8.52	6.78	6.34	15.77	12.23	11.56	13.19	2.08	0.133977	17.65	31.96	20.35	23.32	15.52	18.24	20.79	18.18	0.78	1	36.12	19.1	15.77	23.66	7.34	4.3	5.82	0.25	0.477859
Solyc00g218560.1.1	Unknown Protein (AHRD V1)	2.54	2.26	5.99	5.98	4.19	0	0.67	0.78	4.86	1.58	0.38	0.803484	5.51	0.74	5.71	3.99	1.17	3.07	4.65	2.96	0.74	1	0.68	4.82	11.75	5.75	8.33	8.33	6.78	7.81	1.36	1	6.01	4.3	6.5	5.6	10.84	4.45	7.64	1.36	1
Solyc00g220360.1.1	Unknown Protein (AHRD V1)	0	0	0	0	0	0	0	0	0	0	1	NA	0	0	0	0	0	0	0	0	1	NA	0	0	0	0	0	0	0	0	1	NA	0	0	0	0	0	0	0	1	NA
Solyc00g222060.1.1	Unknown Protein (AHRD V1)	0	0	0	0	0	0	0	0	0	0	1	NA	0	0	0	0	0	0	0	0	1	NA	0	0	0	0	0	0	0	0	1	NA	0	0	0	0	0	0	0	1	NA
Solyc00g222460.1.1	Gag-Pol polyprotein (AHRD V	0	0	0	0	0	0	0	0	0	0	1	NA	0	0	0	0	0	0	0	0	1	NA	0	0	0	0	0	0	0	0	1	NA	0	0	0	0	0	0	0	1	NA
Solyc00g226760.1.1	Steroid sulfotransferase 3 (AH	0	0.89	0.47	0	0.34	0	0	0	0	0	0.03	0.510997	0	0	0	0	0	0	0	0	1	NA	0	0	0	0	0	0	0	0	1	NA	0	0	0	0	0	0	0	1	NA
Solyc00g227860.1.1	UDP-glucosyltransferase (AH	1.26	1.87	1.65	1.58	1.59	2.8	1.66	2.83	2.41	2.42	1.53	0.67298	1.04	1.46	1.77	1.42	1.94	2.39	2.64	2.32	1.63	0.67425	0	0.23	0.14	0.12	0.09	0	0.2	0.1	0.78	1	5.38	1.42	0.22	2.34	0.77	0.61	0.69	0.29	1
Solyc00g227960.1.1	Sulfotransferase family prote	0	0.96	0.25	0	0.3	0	0	0	0	0	0.03	0.680258	0	0	0	0	0	0	0	0	1	NA	0	0	0	0	0	0	0	0	1	NA	0	0	0	0	0	0	0	1	NA
Solyc00g228260.1.1	Unknown Protein (AHRD V1)	0	0	0	0	0	0	0	0	0	0	1	NA	0	0	0	0	0	0	0	0	1	NA	0	0	0	0	0	0	0	0	1	NA	0	0	0	0	0	0	0	1	NA
Solyc00g229260.1.1	TdcA1-ORF2 protein (Fragme	0	0	0	0	0	0	0.28	0	0	0.07	7	1	0	0	0	0	0	0	0	0	1	NA	0	0	0	0	0	0	0	0	1	NA	0	0	0	0	0	0	0	1	NA
Solyc00g230060.1.1	RNase H family protein (AHR	0.45	0	0	0	0.11	0	0	0.21	0	0.05	0.47	1	0.21	0.39	0	0.2	0.78	0.35	0	0.38	1.88	1	0	0	0	0	0	0	0	0	1	1	0.64	0	0	0.21	0	0	0	0.05	1
Solyc00g230070.1.1	Photosystem II CP43 chloroph	7.5	4.67	4.42	0	4.15	1.67	2.96	2.07	3.23	2.48	0.6	0.890742	4.18	4.56	2.53	3.76	4.68	2.33	2.36	3.12	0.83	1	1.21	3.67	2.23	2.37	2.95	1.14	2.73	2.27	0.96	1	9.59	2.18	1.15	4.31	2.06	3.94	3	0.7	1
Solyc00g230080.1.1	Photosystem II D2 protein (A	3.88	3.45	0.65	0	2	6.16	2.19	3.57	3.58	3.88	1.94	0.84369	0.52	1.93	1.4	1.28	1.92	0.57	3.05	1.85	1.44	1	1.34	1.36	1.1	1.27	2.55	2.94	2.02	2.5	1.98	1	3.15	2.42	1.7	2.42	1.01	0.97	0.99	0.41	1
Solyc00g230280.1.1	Unknown Protein (AHRD V1)	0	0	0	0	0	0	0	0	0	0	1	NA	0	0	0	0	0	0	0	0	1	NA	0	0	0	0	0	0	0	0	1	NA	0	0	0	0	0	0	0	1	NA
Solyc00g231680.1.1	Mannan endo-1 4-beta-mann	0	0	0	0	0	0	0	0	0	0	1	NA	0	0	0	0	0	0.09	0	0.03	3	1	0	0	0	0	0	0	0	0	1	NA	0	0	0	0	0	0	0	1	NA
Solyc00g231780.1.1	NAD(P)H-quinone oxidoredu	0.61	0.54	0	0	0.29	0	0	0.56	0	0.14	0.49	1	0.57	0.53	1.55	0.88	0.85	0.95	0	0.6	0.68	1	4.44	3.48	4.24	4.05	2.81	0.93	2.67	2.14	0.53	1	1.74	1.33	0.94	1.34	0	0	0	0.01	1
Solyc00g233480.1.1	ABC transporter-like protein (	0	0.64	0	0	0.16	0	0	0	0	0	0.06	1	0	0	0	0	0	0	0	0	1	NA	0	0	0	0	0	0	0	0	1	NA	0	0.52	0	0.17	0	0	0	0.06	1
Solyc00g237680.1.1	Unknown Protein (AHRD V1)	0	0	0	0	0	0	0	0	0	0	1	NA	0	0	0	0	0	0	0	0	1	NA	0	0	0	0	0	0	0	0	1	NA	0	0	0	0	0	0	0	1	NA
Solyc00g240580.1.1	Unknown Protein (AHRD V1)	0	0	0	0	0	0	0	0	0	0	1	NA	0	0	0	0	0	0	0	0	1	NA	0	0	0	0	0	0	0	0	1	NA	0	0	0	0	0	0	0	1	NA
Solyc00g244280.1.1	Unknown Protein (AHRD V1)	0	0	0	0	0	0	0	0	0	0	1	NA	0	0	0	0	0	0	0	0	1	NA	0	0	1.07	0.36	0	0	0	0	0.03	NA	0	0	0	0	0	0	0	1	NA
Solyc00g244290.1.1	NADH dehydrogenase subuni	0.8	2.15	1.9	2.84	1.92	1.34	0.95	1.85	0.87	1.25	0.65	1	0.75	3.5	1.02	1.76	0.84	1.04	1.26	1.05	0.6	1	2.6	2.62	2	2.41	1.59	0.91	2.05	1.52	0.63	1	2.86	2.05	0	1.64	2.21	1.06	1.64	1	1
Solyc00g244300.1.1	Unknown Protein (AHRD V1)	0.77	0	7.24	8.68	4.17	3.41	0.61	0.71	0	1.18	0.28	0.773437	2.14	2.67	5.83	3.55	3.72	1.19	0.6	1.84	0.52	0.944042	2.48	5	6.09	4.52	2.02	2.91	1.12	2.02	0.45	1	2.18	1.11	0	1.1	1.4	3.36	2.38	2.17	1
Solyc00g245200.1.1	Photosystem I P700 chloroph	0	0	0	0	0	0	0	0	0	0	1	NA	0	0	0	0	0	0	0	0	1	NA	0	0	0	0	0	0	0	0	1	NA	0	0	0	0	0	0	0	1	NA
Solyc00g246800.1.1	Unknown Protein (AHRD V1)	0	0	0	0	0	0	0	0	0	0	1	NA	0	0	0	0	0	0	0	0	1	NA	0	0	0	0	0	0	0	0	1	NA	0	0	0	0	0	0	0	1	NA
Solyc00g247300.2.1	Cytochrome P450	3.21	4.28	2.05	3.21	3.19	0.59	0.53	0.86	0.77	0.69	0.22	0.000615	4.72	13.81	8.01	8.85	6.21	3.94	6.72	5.62	0.64	0.819867	0	0.11	0	0.04	0.18	0	0	0.06	1.64	1	0.38	0.1	0.21	0.23	0	0	0	0.04	1
Solyc00g249200.1.1	Ulp1 protease family C-termi	0	0	0	0	0	0	0	0	0	0	1	NA	0	0	0	0	0	0	0	0	1	NA	0	0	0	0	0	0	0	0	1	NA	0	0	0	0	0	0	0	1	NA
Solyc00g249900.2.1	Adaptin N terminal region fa	51.86	43.44	41.39	49.62	46.58	46.63	38.23	44.88	44.62	43.59	0.94	1	50.37	47.4	59.26	52.34	40.47	40.87	55.74	45.69	0.87	1	32.35	32.32	29.15	31.27	28.06	36.13	34.46	32.88	1.05	1	23.31	18.56	20.52	20.8	36.3	34.44	35.37	1.7	0.698088
Solyc00g249910.1.1	Adaptin N terminal region fa	20.71	12.29	8.15	21.7	15.71	5.97	10.9	4.94	11.01	8.21	0.52	0.501337	5.71	16.67	16.85	13.08	9.58	7.15	9.65	8.79	0.67	0.84893	5.58	9.38	6.85	7.27	9.57	6.98	8.38	8.31	1.14	1	8.73	10.59	2.36	7.23	11.24	16.81	14.02	1.94	1
Solyc00g254110.1.1	Unknown Protein (AHRD V1)	0	0	0	0	0	0	0	0	0	0	1	NA	0	0	0	0	0	0	0	0	1	NA	0	0	0	0	0	0	0	0	1	NA	0	0	0	0	0	0	0	1	NA
Solyc00g254910.1.1	Indole-3-acetic acid-amido sy	0.25	0.22	0.3	0	0.19	0.28	0.4	0	0	0.17	0.88	1	0.94	1.09	0.64	0.89	1.22	1.3	0.59	1.04	1.16	1	0	0.21	0.25	0.15	0	0.38	0.18	0.19	1.22	1	0.72	0.18	0.77	0.56	0	0.66	0.33	0.59	1
Solyc00g255510.1.1	Unknown Protein (AHRD V1);	0	0	0	0	0	0	0	0	0	0	1	NA	0	0	0	0	0	0	0	0	1	NA	0	0	0	0	0	0	0	0	1	NA	0	0	0	0	0	0	0	1	NA
Solyc00g256710.1.1	Unknown Protein (AHRD V1)	27.82	29	27.19	52.84	34.21	16.79	43.93	24.12	21.68	26.63	0.78	1	34.01	38	54.38	42.13	43	32.51	19.99	31.83	0.76	0.944042	25.69	35.62	18.93	26.75	32.9	25.3	25.49	27.9	1.04	1	21.47	22.51	20.78	21.59	24.74	38.33	31.53	1.46	1
Solyc00g257110.2.1	H-ATPase (AHRD V1 **** Q8	0.12	0.1	2.17	0	0.6	0.64	0.18	0	0.66	0.37	0.62	1	0	0	0.1	0.03	0.32	0.06	0.09	0.16	4.7	1	0	0.09	0	0.03	0.23	0.09	0.17	0.16	5.44	1	0.49	0	0.71	0.4	0.11	0.4	0.26	0.64	1
Solyc00g263210.1.1	Unknown Protein (AHRD V1)	0	0	0	0	0	0	0	0	0	0	1	NA	0	0	0	0	0	0	0	0	1	NA	0	0	0	0	0	0	0	0	1	NA	0	0	0	0	0	0	0	1	NA
Solyc00g265110.2.1	RNA-binding protein Nova-1 (	7.58	4.72	4.47	11.8	7.14	3.37	7.78	4.18	5.99	5.33	0.75	1	9.17	6.59	6.4	7.39	4.21	5.1	3.58	4.3	0.58	0.896625	7.96	8.65	6.77	7.79	7.97	5.17	6.63	6.59	0.85	1	0	0.55	0	0.18	1.39	0	0.7	3.79	1
Solyc00g265510.1.1	Unknown Protein (AHRD V1)	0	0	1.56	0	0.39	0	0	0	0	0	0.03	1	0	0	0	0	0	0	0	0	1	NA	0	0	0	0	0	0	0	0	1	NA	0	0	0	0	0	0	0	1	NA
Solyc00g266110.1.1	Indole-3-acetic acid-amido sy	0	0	0	0	0	0	0	0	0	0	1	NA	0	0	0	0	0	0	0.52	0.17	17.33	1	0	0	0	0	0	0	0	0	1	NA	0	0	0	0	0	0	0	1	NA
Solyc00g268310.1.1	Major facilitator superfamily	0	0	0	0	0	0	0	0	0	0	1	NA	0	0	0	0	0	0	0	0	1	NA	0	0	0	0	0	0	0	0	1	NA	0	0	0	0	0	0	0	1	NA
Solyc00g269810.1.1	F-box family protein (AHRD V	0	0	0	0	0	0	0	0	0	0	1	NA	0	0	0	0	0.12	0	0	0.04	4	1	0	0	0	0	0	0	0.13	0.04	4.33	1	0	0	0	0	0	0	0	1	NA
Solyc00g270510.1.1	Nuclear transcription factor Y	0	0	0.42	0	0.1	0	0	0	0	0	0.1	1	0	0	0	0	0	0	0	0	1	NA	0	0	0	0	0	0	0	0	1	NA	0	0	0	0	0	0	0	1	NA
Solyc00g272810.1.1	N-acetyltransferase (AHRD V1	0.27	0	0	0	0.07	0	0	0	0	0	0.15	1	0	0	0	0	0	0	0	0	1	NA	0	0.44	0	0.15	0.18	0	0	0.06	0.41	1	0	0.59	0	0.2	0	0	0	0.05	1
Solyc00g273110.1.1	Unknown Protein (AHRD V1)	6.28	15.84	2.47	1.48	6.52	4.66	9.09	11.55	8.27	8.39	1.29	1	8.77	8.19	2.65	6.54	5.08	10.84	4.11	6.68	1.02	1	3.38	5.12	5.2	4.57	1.38	3.17	2.29	2.28	0.5	1	1.49	4.56	1.61	2.55	1.92	1.84	1.88	0.74	1

Solyc00g275310.1.1	Unknown Protein (AHRD V1);	0	0	0	0	0	0	0	0	0	0	1	NA	0	0	0	0	0	0	0	0	1	NA	0	0	0	0	0	0	0.12	0.04	4	1	0	0	0	0	0	0	0	1	NA
Solyc00g277510.1.1	Photosystem Q(B) protein (A	47.26	63.97	41.82	36.21	47.31	37.21	41.2	49.79	84.12	53.08	1.12	0.996578	38.47	29.96	34.1	34.18	86.73	74.94	42.56	68.08	1.99	0.134436	30.23	30.49	37.14	32.62	75.03	55.97	55.97	62.32	1.91	0.089495	39.2	37.9	51.48	42.86	28.84	63.02	45.93	1.07	1
Solyc00g278110.2.1	CER1 (AHRD V1 **-- B6TFH3_	0	0	0	0	0	0	0	0	0	0	1	NA	0	0	0	0	0	0	0	0	1	NA	0	0	0.92	0.31	0	0	0	0	0.03	1	0	2.7	1.43	1.38	0	0	0	0.01	1
Solyc00g280110.1.1	Plant-specific domain TIGR01	0	0	0	0	0	0	0	0	0	0	1	NA	0	0	0	0	0.92	1.37	0	0.76	76.33	1	0	0	0	0	0	1	0	0.33	33.33	NA	0	0	0	0	0	0	0	1	NA
Solyc00g281110.1.1	Unknown Protein (AHRD V1)	0	0	0	0	0	0	0	0	0	0	1	NA	0	0	0	0	0	0	0	0	1	NA	0	0	0	0	0	0	0.39	0.13	13	1	0	0	0	0	0	0	0	1	NA
Solyc00g282510.1.1	Phenylalanine ammonia-lyase	3.34	2.18	1.71	0.95	2.04	3.59	2.46	3.38	2	2.86	1.4	0.824752	1.45	0.58	0.66	0.9	1.24	1.67	2.8	1.9	2.12	0.423138	1.44	3.63	1.11	2.06	0.88	0.84	1.14	0.95	0.46	1	1.43	0.97	0.86	1.09	1.63	1.86	1.74	1.61	1
Solyc00g283010.1.1	Multidrug resistance protein	0	0	0	0	0	0	0	0	0	0	1	NA	0	0	0	0	0	0	0	0	1	NA	0	0	0	0	0	0	0	0	1	NA	0	0	0	0	0	0	0	1	NA
Solyc00g283110.1.1	Unknown Protein (AHRD V1)	0	0	0	0	0	0	0	0	0	0	1	NA	0	0	0	0	0	0	0	0	1	NA	0	0	0	0	0	0	0	0	1	NA	0	0	0	0	0	0	0	1	NA
Solyc00g284510.1.1	Unknown Protein (AHRD V1)	0	0	0	0	0	0	0	0	0	0	1	NA	0	0	0	0	0	0	0	0	1	NA	0	0	0	0	0	0	0	0	1	NA	0	0	0	0	0	0	0	1	NA
Solyc00g286010.1.1	Plasmid partitioning protein	0	0	0	0	0	0	0	0	0	0	1	NA	0	0	0	0	0	0	0	0	1	NA	0	0	0	0	0	0	0	0	1	NA	0	0	0	0	0	0	0	1	NA
Solyc00g286020.1.1	Plasmid partitioning protein	0	0	0	0	0	0	0	0	0	0	1	NA	0	0	0	0	0	0	0	0	1	NA	0	0	0	0	0	0	0	0	1	NA	0	0	0	0	0	0	0	1	NA
Solyc00g286030.1.1	Replication protein C (AHRD V	0	0	0	0	0	0	0	0	0	0	1	NA	0	0	0	0	0	0	0	0	1	NA	0	0	0	0	0	0	0	0	1	NA	0	0	0	0	0	0	0	1	NA
Solyc00g287330.1.1	Unknown Protein (AHRD V1);	0	0	0	0	0	0	0	0	0	0	1	NA	0	0	0	0	0	0	0	0	1	NA	0	0	0	0	0	0	0	0	1	NA	0	0	0	0	0	0	0	1	NA
Solyc00g289230.1.1	Receptor protein kinase (AHR	1.32	0.84	0	0.8	0.74	1.05	1.19	0.52	0.95	0.93	1.25	1	0.7	0.82	0.48	0.67	1.7	0.98	1.04	1.24	1.86	0.84953	0.46	0.46	0.19	0.37	1.24	0.72	0.69	0.88	2.39	1	0.27	1.51	0	0.59	0.17	0	0.09	0.14	1
Solyc00g294230.1.1	Tir-nbs, resistance protein fra	0.66	0.29	1.17	0.47	0.65	0	0.78	0.61	0.24	0.41	0.63	1	0.31	0.86	0.84	0.67	0	0.17	0	0.06	0.08	0.70488	0	0	0	0	0.22	0	0.24	0.15	15.33	1	0	0.24	0	0.08	0	0	0	0.12	1
Solyc00g294630.1.1	Mutator-like transposase (AH	0	0	0	0	0	0.49	0	0	0	0.12	12.25	1	0	0	0	0	0	0	0	0	1	NA	0	0	0	0	0	0	0	0	1	NA	0	0	0	0	0	0	0	1	NA
Solyc00g297330.1.1	Unknown Protein (AHRD V1)	0	0	0	0	0	0	0	0	0	0	1	NA	0	0	0	0	0	0	0	0	1	NA	0	0	0	0	0	0	0	0	1	NA	0	0	0	0	0	0	0	1	NA
Solyc00g300430.1.1	Unknown Protein (AHRD V1)	0	0	0	0	0	0	0	0	0	0	1	NA	0	0	0	0	0	0	0	0	1	NA	0	0	0	0	0	0	0	0	1	NA	0	0	0	0	0	0	0	1	NA
Solyc00g300730.1.1	E3 ubiquitin-protein ligase sir	0	0	0	0	0	0	0	0	0	0	1	NA	0	0	0	0	0	0	0	0	1	NA	0	0	0	0	0	0	0	0	1	NA	0	0	0	0	0	0	0	1	NA
Solyc00g303630.1.1	Unknown Protein (AHRD V1)	0	0	0	0	0	0	0	0	0	0	1	NA	0	0	0	0	0	0	0	0	1	NA	0	0	0	0	0	0	0	0	1	NA	0	0	0	0	0	0	0	1	NA
Solyc00g304030.1.1	Macrolide export ATP-binding	0	0	0	0	0	0	0	0	0	0	1	NA	0	0	0	0	0	0	0	0	1	NA	0	0	0	0	0	0	0	0	1	NA	0	0	0	0	0	0	0	1	NA
Solyc00g304630.1.1	Ulp1 protease family C-termi	0	0	0	0	0	0	0	0	0	0	1	NA	0	0	0	0	0	0	0	0	1	NA	0	0	0	0	0	0	0	0	1	NA	0	0	0	0	0	0	0	1	NA
Solyc00g306730.1.1	Mutator-like transposase (AH	0	0	0	0	0	0	0	0	0	0	1	NA	0	0	0	0	0	0	0	0	1	NA	0	0	0	0	0	0	0	0	1	NA	0	0	0	0	0	0	0	1	NA
Solyc00g306830.1.1	Glucose-methanol-choline ox	0	0	0	0	0	0	0	0	0	0	1	NA	0	0	0	0	0	0	0	0	1	NA	0	0	0	0	0	0	0	0	1	NA	0	0	0	0	0	0	0	1	NA
Solyc00g308530.1.1	Unknown Protein (AHRD V1)	0	0	0	0	0	0	0	0	0	0	1	NA	0	0	0	0	0	0	0	0	1	NA	0	0	0	0	0	0	0	0	1	NA	0	0	0	0	0	0	0	1	NA
Solyc00g308930.1.1	Lysine-specific histone demet	0	0	0	0	0	0	0	0	0	0	1	NA	0	0	0	0	0	0	0	0	1	NA	0	0	0	0	0	0	0	0	1	NA	0	0	0	0	0	0	0	1	NA
Solyc00g309530.1.1	Unknown Protein (AHRD V1)	0	0	0	0	0	0	0	0	0	0	1	NA	0	0	0	0	0	0	0	0	1	NA	1.01	0	0	0.34	0	0	0	0	0.03	1	0	0	0	0	0	0	0	1	NA
Solyc00g311230.2.1	Unknown Protein (AHRD V1)	0.9	1.2	1.06	4.44	1.9	2	3.9	2.89	1.61	2.6	1.37	0.920872	0.42	0	0.38	0.27	0.31	1.16	1.76	1.08	4.04	0.832635	1.09	0.37	0	0.49	0.59	0.34	0	0.31	0.64	1	0.64	0	0	0.21	0	0	0	0.05	1
Solyc00g313030.1.1	Unknown Protein (AHRD V1)	0	0	0	0	0	0	0	0	0	0	1	NA	0	0	0	0	0	0	0	0	1	NA	0	0	0	0	0	0	0	0	1	NA	0	0	0	0	0	0	0	1	NA
Solyc00g313530.1.1	Unknown Protein (AHRD V1)	0	0	0	0	0	0	0	0.99	0	0.25	24.75	1	0	0	0	0	0	0	0	0	1	NA	0	0	0	0	0	0	0	0	1	NA	0	0	0	0	0	0	0	1	NA
Solyc00g313930.1.1	Unknown Protein (AHRD V1)	0	0	0	0	0	0	0	0	0	0	1	NA	0	0	0	0	0	0	0	0	1	NA	0	0	0	0	0	0	0	0	1	NA	0	0	0	0	0	0	0	1	NA
Solyc00g316030.1.1	Mutator-like transposase (AH	0	0	0	0	0	0	0	0	0	0	1	NA	0	0	0	0	0	0	0	0	1	NA	0	0	0	0	0	0	0	0	1	NA	0	0	0	0	0	0	0	1	NA
Solyc00g316530.1.1	Unknown Protein (AHRD V1)	0	0.74	0.99	0	0.43	0	0	0	0.6	0.15	0.35	1	0	0	0	0	0	0.43	0	0.14	14.33	1	0	0	0	0	0	0	0	0	1	NA	0	0	0	0	0	0	0	1	NA
Solyc00g316730.1.1	Mutator-like transposase (AH	0	0	0	0	0	0	0	0	0	0	1	NA	0	0	0	0	0	0	0	0	1	NA	0	0	0	0	0	0	0	0	1	NA	0	0	0	0	0	0	0	1	NA
Solyc00g318030.1.1	Unknown Protein (AHRD V1)	0	0	0	0	0	0	0	0	0	0	1	NA	0	0	0	0	0	0	0	0	1	NA	0	0	0	0	0	0	0	0	1	NA	0	0	0	0	0	0	0	1	NA
Solyc00g318130.1.1	Paired amphipathic helix prot	0	0.21	0.28	0	0.12	0	0	0	0	0	0.08	1	0	0	0	0	0	0	0	0	1	NA	0	0	0	0	0	0	0	0	1	NA	0	0	0	0	0	0	0	1	NA
Solyc00g318830.1.1	Os01g0126600 protein (Fragi	0.35	0.63	1.67	0	0.66	0.39	0.56	0.65	1.27	0.72	1.08	1	0.33	0.92	0.3	0.52	0.25	0.55	0.28	0.36	0.7	1	2.01	0.29	0.7	1	0.93	0.54	0.52	0.66	0.66	1	1.51	4.64	3.82	3.32	1.3	1.55	1.43	0.43	0.985795
Solyc00g320430.1.1	Unknown Protein (AHRD V1)	0	0	0	0	0	0	0	0	0	0	1	NA	0	0	0	0	0	0	0	0	1	NA	0	0	0	0	0	0	0	0	1	NA	0	0.8	0	0.27	0	0	0	0.04	1
Solyc00g322630.1.1	Indole-3-acetic acid-amido sy	0	0	0	0	0	0	0	0	0	0	1	NA	0	0	0	0	0	0	0.52	0.17	17.33	1	0	0	0	0	0	0	0	0	1	NA	0	0	0	0	0	0	0	1	NA
Solyc00g322830.1.1	Unknown Protein (AHRD V1)	0	0	0	0	0	0	0	0	0	0	1	NA	0	0	0	0	0	0	0	0	1	NA	0	0	0	0	0	0	0	0	1	NA	0	0	0	0	0	0	0	1	NA
Solyc00g323130.2.1	Major latex-like protein (AHR	0	0	0	0	0	0	0	0	0	0	1	NA	0.29	0	0	0.1	0	0	0	0	0.1	1	0	0	0	0	0	0	0	0	1	NA	0	0	0	0	0	0	0	1	NA
Solyc00g324530.1.1	Ulp1 protease family C-termi	0	0	0	0	0	0	0	0	0.34	0.09	8.5	1	0	0	0	0	0	0	0	0	1	NA	0	0	0	0	0	0	0	0	1	NA	0	0	0	0	0	0	0	1	NA
Solyc01g005000.2.1	Glutamate decarboxylase (AH	44.48	42.86	18.94	20.2	31.62	47.33	36.62	55.28	44.04	45.82	1.45	0.499958	27.84	8.63	11.83	16.1	5	7.82	6.73	6.52	0.4	0.673688	3.3	2.66	1.76	2.57	0.8	0.52	0.99	0.77	0.3	0.22529	0.97	0.89	0.42	0.76	2.24	1.67	1.96	2.57	0.868616
Solyc01g005010.2.1	Protein XRI1 (AHRD V1 *-*- X	13.4	18.25	16.12	10.04	14.45	23.96	17.43	20.78	19.44	20.4	1.41	0.470627	14.91	11.2	16.87	14.33	13.49	19.19	12.39	15.02	1.05	1	2.97	2.14	1.57	2.23	5.87	1.59	2.87	3.44	1.55	1	0	0.57	0.4	0.32	1.2	0.92	1.06	3.28	1
Solyc01g005020.2.1	Sodium/hydrogen exchanger	23.14	27.32	29.46	23.9	25.95	20.82	16.95	25.69	22.74	21.55	0.83	0.838948	24.18	23.7	28.49	25.46	22	22.8	25.97	23.59	0.93	1	6.06	12.23	8.07	8.79	9.74	5.99	12.98	9.57	1.09	1	6.03	8.69	9.51	8.08	12.06	15.46	13.76	1.7	0.625159
Solyc01g005030.2.1	Serine/threonine-protein kina	8.47	12.17	12.56	9.06	10.57	10.43	7.63	11.42	10.8	10.07	0.95	1	13.25	19.45	21.7	18.13	21.31	21.12	23.97	22.13	1.22	0.699602	24.92	26.39	19.76	23.69	24.47	18.26	30.48	24.4	1.03	1	17	17.95	19.53	18.16	13.72	18.51	16.12	0.89	1
Solyc01g005040.2.1	Unknown Protein (AHRD V1);	0	0	0	0	0	0	0	0	0	0	1	NA	0	0	0	0	0	0	0	0	1	NA	0	0	0	0	0	0	0	0	1	NA	0	0	0	0	0	0	0	1	NA
Solyc01g005050.2.1	Unknown Protein (AHRD V1)	0	0	0.69	0	0.17	0	0	0	0	0	0.06	1	0	0	0	0	0	0	0	0	1	NA	0	0	0	0	0	0	0	0	1	NA	0	0	0	0	0	0	0	1	NA
Solyc01g005060.2.1	Zinc finger protein (AHRD V1	6.79	9.15	8.93	9.33	8.55	4.1	6.13	9.1	7.38	6.68	0.78	0.924458	6.86	7.93	4.1	6.3	4.44	6.13	6.4	5.66	0.9	1	3.76	2.53	2.31	2.87	3.31	2.79	2.83	2.98	1.04	1	1.66	2.96	3.58	2.73	1.6	1.19	1.4	0.51	1
Solyc01g005070.2.1	Clathrin assembly protein (AH	0	0.15	0	0	0.04	0	0	0	0	0	0.27	1	0	0	0	0	0	0	0	0	1	NA	0	0	0	0	0	0	0	0	1	NA	0	0	0	0	0	0	0	1	NA
Solyc01g005080.2.1	Microtubule-associated prote	5.07	8.07	9.73	3.67	6.63	15.19	5.77	16.46	11.25	12.17	1.83	0.364652	5.92	8.19	6.17	6.76	12.34	8.97	6.85	9.39	1.39	0.610056	5.62	4.7	1.64	3.99	4.72	2.68	3.52	3.64	0.91	1	0.84	2.31	2.17	1.77	4.64	9.61	7.12	4.02	0.179982
Solyc01g005090.2.1	Inositol 1 4 5-trisphosphate 5	0	0	0	0	0	0	0	0	0	0	1	NA	0	0	0	0	0	0	0	0	1	NA	0	0	0	0	0	0	0	0	1	NA	0	0	0	0	0	0	0	1	NA
Solyc01g005100.2.1	Prolyl 3-hydroxylase 1 (AHRD	14.08	13.4	11.39	8.8	11.92	18.92	21.84	16.86	14.57	18.05	1.51	0.226309	13.4	14.51	13.96	13.96	12.94	14.41	12.99	13.45	0.96	1	9.79	9.74	5.85	8.46	7.64	6.82	8.11	7.52	0.89	1	6.52	6.9	8.06	7.16	8.84	9.9	9.37	1.31	1
Solyc01g005110.2.1	cDNA clone J065210E11 full i	77.23	88.79	70.24	75.11	77.84	82.27	69.87	115.39	64.69	83.05	1.07	1	80.82	75.53	54.36	70.24	54.68	65.82	67.02	62.51	0.89	1	189.79	121.94	102.22	137.98	79.28	103.66	124.29	102.41	0.74	1	43.47	80.64	81.65	68.59	91.32	57.84	74.58	1.09	1
Solyc01g005120.2.1	Xyloglucan endotransglucosyl	4.71	6.82	6.49	6.39	6.1	3.93	8.06	7.76	6.63	6.59	1.08	1	14.06	18.77	32.5	21.78	25.06	26.23	16.82	22.7	1.04	1	8.72	10.88	8.77	9.46	22.19	14.43	14.16	16.93	1.79	0.678237	6.7	13.69	12.38	10.92	16	20.83	18.41	1.69	0.854307
Solyc01g005130.2.1	Zinc finger protein 7 (AHRD V	18.14	14.27	19.85	29	20.31	9.35	17.64	6.77	13.21	11.74	0.58	0.514386	13.94	20.33	19.98	18.08	22.6	18.51	15.49	18.87	1.04	1	5.95	9.85	11.22	9.01	8.63	6.77	9	8.13	0.9	1	56.05	33.22	26.68	38.65	24.3	28.81	26.55	0.69	1
Solyc01g005140.2.1	Cytochrome b561 (AHRD V1 *	0	0.79	1.05	0.42	0.57	0.33	0.94	2.19	0	0.86	1.53	1	2.49	2.84	4.27	3.2	11.13	9.39	11.68	10.73	3.35	0.001804	9.6	9.2	6.49	8.43	6.44	6.98	7.58	7	0.83	1	42.67	57.63	47.98	49.43	12.24	13.81	13.03	0.26	0.002351
Solyc01g005150.2.1	Cytochrome b561 (AHRD V1 *	0.88	0.78	0.69	0.41	0.69	0.33	0.46	1.08	0.21	0.52	0.75	1	0	0	0	0	1.42	0.46	0.69	0.86	85.67	0.270372	0.24	0.48	0	0.24	0.19	0	0	0.06	0.26	1	0	0	0	0	0.27	0	0.14	13.5	1
Solyc01g005160.2.1	U-box domain-containing pro	0.15	0	0	0	0.04	0	0.23	0	0	0.06	1.53	1	0.14	0	0.12	0.09	0.1	0.08	0	0.06	0.69	1	0.24	0.48	0.44	0.39	0.29	0	0	0.1	0.25	1	1.04	4.78	7.87	4.56	0.67	2.31	1.49	0.33	0.993806
Solyc01g005170.1.1	Clathrin assembly protein (AH	0.48	0.42	0.19	0	0.27	0.53	0.5	0.73	0.57	0.58	2.14	0.90221	0.59	0.55	0.67	0.6	0.77	0.49	1.87	1.04	1.73	0.962977	0.64	0.26	0.63	0.51	0.42	0.72	0.35	0.5	0.97	1	0	0.58	1.47	0.68	0.87	0.98	0.93	1.35	1
Solyc01g005180.1.1	Unknown Protein (AHRD V1)	0	1.91	0.85	0	0.69	0	0	0	0	0	0.01	0.790538	0	0	0	0	0	0	0	0	1	NA	0	0	0	0	0	0	0	0	1	NA	0	0	0	0	0	0	0	1	NA
Solyc01g005190.1.1	Zinc finger family protein (AH	0	0.61	0.27	0.32	0.3	0	0.18	0.63	0.16	0.24	0.81	1	0	0.6	0.19	0.26	0.32	0.47	0.9	0.56	2.14	1	0.37	0.37	0.23	0.32	0.15	0.17	0	0.11	0.33	1	0.98	0.67	0.35	0.67	0.21	0.4	0.3	0.46	1
Solyc01g005200.1.1	Zinc finger family protein (AH	0.22	0	0.52	0	0.18	0.25	0	0	0.16	0.1	0.55	1	0	0	0.19	0.06	0.15	0.23	0.35	0.24	3.84	1	0	0	0	0	0	0	0	0	1	1	0	0.32	0	0.11	0	0.19	0.1	0.89	1
Solyc01g005210.2.1	Alpha alpha-trehalose-phosph	15.38	17.18	25.31	21.97	19.96	15.38	15.54	12.23	11.04	13.55	0.68	0.308648	11.37	19.05	24.16	18.19	17.28	13.81	14.5	15.2	0.84	1	26.34	37.22	28.47	30.68	18.69	21.5	19.89	20.03	0.65	1	30.72	28.09	24.59	27.8	12.74	10.79	11.77	0.42	0.097162
Solyc01g005220.2.1	Necrotic spotted lesions 1 (Fr	8.84	10.13	7.95	19.85	11.69	9.46	16.75	10.57	7.7	11.12	0.95	1	11.41	27.67	24.64	21.24	16.63	11.9	14.59	14.37	0.68	0.748348	7.86	7.92	15.14	10.31	6.97	7.96	6.36	7.1	0.69	1	31.58	85.48	77.83	64.96	19.42	22.77	21.09	0.32	0.277633
Solyc01g005230.2.1	S-adenosyl-L-methionine salic	0.58	0.17	0	0.27	0.26	2.57	1.98	1.95	1.52	2	7.86	0.010076	1.26	1.17	0.81	1.08	1.2	1.7	2.73	1.88	1.74	0.804182	0	0	0	0	0.13	0.15	0	0.09	9.33	1	0	0.42	0.3	0.24	0	0	0	0.04	1
Solyc01g005240.2.1	Aspartokinase (AHRD V1 ***-	29.02	32.78	26.95	28.52	29.32	34.98	30.83	33.33	31.8	32.73	1.12	0.919033	33.94	27.05	24.91	28.63	25.63	29.27	29.13	28.01	0.98	1	25.14	20.04	21.98	22.39	19.98	25.97	26.67	24.21	1.08	1	10.44	19.13	20.79	16.79	13.23	14.82	14.03	0.84	1
Solyc01g005250.2.1	Aspartate-semialdehyde dehy	60.1	48.57	48.08	55.21	52.99	63.16	61.08	55.89	48.23	57.09	1.08	0.979125	41.93	30.78	37.78	36.83	39.65	37.64	43.29	40.19	1.09	1	19.95	20.97	30	23.64	21.33	27.75	27.06	25.38	1.07	1	19.64	28.85	22.04	23.51	44.42	43.61	44.02	1.87	0.5173
Solyc01g005260.2.1	SEC14 cytosolic factor family	23.27	23.63	17.76	21.68	21.59	17.65	18.37	42.28	24.17	25.62	1.19	0.929978	16.3	19.51	13.64	16.48	10.82	13.46	15.27	13.18	0.8	0.960024	17.47	17.62	8.42	14.5	7.37	6.85	10.37	8.2	0.57	1	3.11	5.17	4.63	4.3	3.01	1.2	2.1	0.49	0.94024
Solyc01g005270.2.1	SEC14 cytosolic factor family	15.88	23.88	11.95	13.16	16.22	22.82	22.91	31.96	15.72	23.35	1.44	0.52542	24.19	27.13	20.58	23.97	17.09	31.75	28.83	25.89	1.08	1	23.66	31.9	10.87	22.14	20.86	21.78	13.36	18.67	0.84	1	0.78	3.18	8	3.99	4.26	2.16	3.21	0.81	1
Solyc01g005280.2.1	SEC14 cytosolic factor (AHRD	28.47	38.49	16.79	15.49	24.81	35.91	29.82	52.1	37.34	38.79	1.56	0.383728	29.79	20.23	14.33	21.45	7.97	16.02	12.48	12.16	0.57	0.635027	78.29	62.01	26.36	55.55	19.78	18.67	21.54	20	0.36	1	3.89	6.16	8.42	6.16	4.76	1.2	2.98	0.48	0.874234
Solyc01g005290.2.1	SEC14 cytosolic factor family	24.2	25.08	12.91	8.91	17.77	26.16	24.34	49.99	22.61	30.78	1.73	0.526242	18.19	10.95	8.12	12.42	5.52	9.27	8.86	7.88	0.63	0.83287	63.48	66.99	29.29	53.25	30.27	37.94	40.1	36.1	0.68	1	34.41	38.77	43.33	38.84	30.95	28.78	29.87	0.77	1
Solyc01g005300.2.1	Flavin-binding kelch domain F	59.35	52.53	55.17	73.98	60.26	44.02	55.68	45.39	55.93	50.26	0.83	0.963372	46.93	46.22	57.74	50.3	68.33	59.49	61.14	62.99	1.25	0.558445	49.35	58	57.19	54.85	45.9	47.53	52.26	48.56	0.89	1	67.11	63.91	52.56	61.19	78.87	89.52	84.19	1.38	0.98833
Solyc01g005310.2.1	Dynamin like protein (AHRD V	0	0	0.12	0	0.03	0	0.08	0	0.08	0.04	1.33	1	0	0	0	0	0	0	0	0	1	NA	0	0	0	0	0	0	0	0	1	NA	0	0	0	0	0	0	0	1	NA
Solyc01g005320.2.1	Conserved membrane protein	0	0.18	0	0	0.04	0	0	0	0	0	0.22	1	0.38	0	0.17	0.18	0.14	0.11	0.32	0.19	1.04	1	0.17	0	0	0.06	0	0	0.15	0.05	0.88	1	0	0	0	0	0	0.18	0.09	9	1
Solyc01g005330.2.1	microtubule associated prote	49.88	46.09	49.6	66.35	52.98	38.14	41.97	40.7	47.67	42.12	0.8	0.831651	40.78	40.5	51.25	44.18	38.85	44.68	36.28	39.94	0.9	1	15.23	25.11	10.93	17.09	26.71	15.97	21.22	21.3	1.25	1	31.17	28.69	39.24	33.03	44.69	56.08	50.38	1.53	0.698088
Solyc01g005340.2.1	Ankyrin repeat family protein	13.72	15.24	24.06	19.23	18.06	12.32	12.03	15.63	18.08	14.52	0.8	0.924458	22.13	24.3	28.92	25.12	23.4	25.62	21.87	23.63	0.94	1	20.55	27.8	17.37	21.91	19.27	16.6	17.43	17.77	0.81	1	41.53	35.39	35.43	37.45	30.77	33.01	31.89	0.85	1
Solyc01g005350.2.1	S-adenosyl-L-methionine salic	0	0	0	0	0	0	0	0	0	0	1	NA	0.19	0	0	0.06	0.14	0.1	0	0.08	1.26	1	0	0	0	0	0	0	0	0	1	NA	0	0	0	0	0	0	0	1	NA
Solyc01g005360.2.1	S-adenosyl-L-methionine salic	0	0	0	0	0	0	0	0	0	0	1	NA	0	0	0	0	0	0	0	0	1	NA	0	0	0	0	0	0	0	0	1	NA	0	0	0	0	0	0	0	1	NA
Solyc01g005370.2.1	Calmodulin-2 (AHRD V1 ***-	17.54	19.68	15.3	17.8	17.58	26.28	16.86	28.05	19.71	22.73	1.29	0.709246	16.67	23.2	16.43	18.77	33.06	36.33	35.06	34.82	1.86	0.028236	61.92	70.23	60.94	64.36	82.42	59.54	90.59	77.52	1.2	1	39.04	35.17	31.08	35.1	33.51	32.77	33.14	0.94	1
Solyc01g005380.2.1	SET domain containing protei	9.22	7.34	10.88	7.51	8.74	9.94	9.27	8.22	8.83	9.06	1.04	1	6.24	6.32	8.38	6.98	7.76	6.51	6.15	6.81	0.98	1	4.74	7.63	8.05	6.81	6.52	6.78	5.7	6.33	0.93	1	9.14	8.43	4.94	7.5	12.15	10.05	11.1	1.48	1
Solyc01g005390.2.1	Nudix hydrolase 4 (AHRD V1	0	0.36	0	0.58	0.23	0	0.32	0.75	0	0.27	1.14	1	0	0	0	0	0	0.21	0	0.07	7	1	0	0	0.41	0.14	0	0	0	0	0.07	1	35.54	5.36	14.49	18.46	1.5	3.23	2.37	0.13	0.889522
Solyc01g005400.2.1	Calcium binding protein Cale	0.66	0.29	3.1	0.93	1.24	1.46	1.04	0.3	0.94	0.93	0.75	1	0.61	0.29	0.55	0.48	0.46	1.02	0	0.49	1.02	1	0.53	1.34	1.63	1.17	1.29	1.99	1.91	1.73	1.48	1	3.27	9.77	5.55	6.2	1.5	0.86	1.18	0.19	0.209383
Solyc01g005410.2.1	Calcium binding protein Cale	20.62	18.96	8.51	10.21	14.57	23.3	20.62	31.59	17.02	23.13	1.59	0.40381	24.61	20.91	11.9	19.14	29.31	26.51	31.86	29.23	1.53	0.230476	0.56	0	0	0.19	1.35	1.04	0.5	0.96	5.16	1	0.49	0.5	0	0.33	0	1.81	0.91	2.74	1
Solyc01g005420.2.1	SRC2-like protein (AHRD V1 *	18.35	21.89	8.75	24.3	18.32	15.62	23.73	28	15.97	20.83	1.14	0.969891	11.98	27.49	19.45	19.64	20.85	23.04	20.25	21.38	1.09	1	0.32	1.59	0.77	0.89	2.05	0.59	0	0.88	0.99	1	0	0.85	0	0.28	0	0	0	0.04	1
Solyc01g005430.2.1	UBX domain protein 6 (AHRD	5.92	6.94	6.98	6.13	6.49	4.81	3.42	7.17	4.56	4.99	0.77	0.800128	6.45	6.53	7.19	6.72	7.31	6.13	6.24	6.56	0.98	1	3.85	5.76	5.59	5.07	9.1	6.56	5.79	7.15	1.41	1	26.07	19.4	27.08	24.18	8.72	11.14	9.93	0.41	0.189985
Solyc01g005440.2.1	Jasmonate ZIM-domain prote	363.91	331.26	572.19	353.4	405.19	368.08	352.48	297.41	309.08	331.76	0.82	0.868752	342.85	233.27	272.85	282.99	226.57	205.39	175.1	202.35	0.72	0.484544	63.44	86.53	46.68	65.55	40.54	40.32	36.6	39.15	0.6	1	66.57	44.05	37.96	49.53	58.64	46.65	52.64	1.06	1
Solyc01g005450.2.1	F-box protein family-like prot	8.57	9.53	8.34	3.03	7.37	10.24	8.96	16.54	8	10.94	1.48	0.648245	8.37	6.33	5.07	6.59	4.75	7.54	10.77	7.69	1.17	1	1.9	4.01	0.64	2.18	2.39	1.62	2.34	2.12	0.97	1	0.91	0.47	0	0.46	0.59	1.13	0.86	1.87	1
Solyc01g005460.2.1	Dynein light chain 1 cytoplasi	9.06	8.96	2.38	5.7	6.53	11.2	12.32	16.21	5.42	11.29	1.73	0.534926	3.28	0.88	2.55	2.24	0	0.52	0.4	0.31	0.14	0.322402	0	0	0	0	0	0	0	0	1	NA	0	0	0	0	0	0	0	1	NA
Solyc01g005470.2.1	Cell number regulator 10 (AH	3.6	1.78	3.77	2.26	2.85	0.44	1.58	3.31	0.29	1.41	0.49	0.689959	1.86	2.43	0	1.43	1.11	1.24	0.31	0.89	0.62	1	0	0	0	0	0.79	0.3	0	0.36	36.33	1	5.12	11.33	19.06	11.84	5.12	2.45	3.79	0.32	0.796983
Solyc01g005480.2.1	Unknown Protein (AHRD V1);	8.92	7.01	14.45	17.57	11.99	6.61	7.46	5.79	7.41	6.82	0.57	0.521196	7.81	9.43	14.19	10.48	7.04	6.8	8.11	7.32	0.7	0.601741	4.24	3.14	4.69	4.02	4.37	1.86	2.68	2.97	0.74	1	9.21	3.43	5.92	6.19	4.97	6.14	5.55	0.9	1
Solyc01g005490.2.1	Iaa-amino acid hydrolase 4 (A	22.94	20.15	18.57	21.63	20.82	16.83	23.08	14.61	21.64	19.04	0.91	1	24.13	24.24	24.95	24.44	23.92	25.93	22.39	24.08	0.99	1	21.44	35.55	21.13	26.04	22.85	19.09	30.92	24.29	0.93	1	5.33	7.84	5.3	6.16	8.65	6.57	7.61	1.24	1
Solyc01g005500.2.1	3-phenylpropionate-dihydrod	0	0	0	0	0	0	0	0	0	0	1	NA	0	0	0	0	0	0	0	0	1	NA	0	0	0	0	0	0	0	0	1	NA	0	0	0	0	0.21	0	0.1	10.5	1
Solyc01g005510.2.1	Laccase-2 (AHRD V1 **-- LAC	0	0.11	1.95	0	0.52	0	0	0	0	0	0.02	0.72385	0.11	0	0	0.04	0	0.06	0	0.02	0.55	1	0	0	0	0	0	0	0	0	1	1	0	0	0	0	0.11	0.21	0.16	16	1
Solyc01g005520.2.1	Tetratricopeptide TPR_2 repe	44.45	43.1	38.56	27.91	38.51	126.95	95.98	134.87	106.46	116.06	3.01	1.41E-09	36.15	38.98	28.79	34.64	68.43	79.46	84.89	77.59	2.24	2.93E-05	16.42	26.64	9.69	17.58	31.82	21.75	30.06	27.88	1.59	0.974113	11.05	25.77	15.02	17.28	50.54	45.96	48.25	2.79	0.068532
Solyc01g005530.2.1	Genomic DNA chromosome 3	23.04	24.23	13.59	13.33	18.55	27.35	25.63	19.26	24.43	24.17	1.3	0.772659	19.97	19.57	19.02	19.52	15.62	24.4	26.33	22.12	1.13	1	15.23	16.64	6.76	12.88	10.32	13.49	19.08	14.3	1.11	1	5.21	11.79	4.83	7.28	6.23	11.02	8.62	1.19	1
Solyc01g005540.2.1	Lysyl-tRNA synthetase (AHRD	11.95	12.14	10.51	9.89	11.12	19.18	23.06	19.39	15.54	19.29	1.73	0.025438	9.55	11.18	6.38	9.04	12.75	12.61	13.03	12.8	1.42	0.325294	6.01	6.71	5.93	6.22	5.48	5.47	7.07	6.01	0.97	1	4.33	5.9	5.03	5.09	9.08	7.81	8.45	1.66	0.88831
Solyc01g005550.2.1	Carboxyl-terminal peptidase (	60.41	81.13	74.73	46.11	65.59	72.3	52.57	85.09	63.78	68.44	1.04	1	48.57	50.36	42.93	47.29	49.94	59.12	59.37	56.14	1.19	0.729891	16.23	18.88	11.81	15.64	13.8	11.83	14.75	13.46	0.86	1	10.32	23.45	15.21	16.33	12.87	23.57	18.22	1.12	1
Solyc01g005560.2.1	Isocitrate dehydrogenase (AH	65.81	54.62	57.81	57.87	59.03	52.88	62.85	54.97	58.18	57.22	0.97	1	79.36	40.39	40.05	53.27	33.22	31.45	35.64	33.44	0.63	0.751092	322.61	183.5	269.17	258.43	251.59	186.18	178.89	205.55	0.8	1	15.56	26.38	15.12	19.02	14.23	15.29	14.76	0.78	1
Solyc01g005570.2.1	Unknown Protein (AHRD V1);	43.05	67.37	41.78	47.15	49.84	59.44	40.02	71.51	47.86	54.71	1.1	1	44.57	49.5	50.44	48.17	38.05	46.02	61.12	48.4	1	1	41.52	49.8	23.44	38.25	35.13	35.26	36.94	35.78	0.94	1	109.6	50.44	46.99	69.01	36.87	31.66	34.27	0.5	1
Solyc01g005580.2.1	Unknown Protein (AHRD V1);	81.79	96.92	67.17	84.37	82.56	95.93	84.9	141.25	73.64	98.93	1.2	0.795461	84.48	94.27	84.34	87.7	62.68	73.23	95.52	77.14	0.88	1	121.2	125.19	88.36	111.58	117.55	99.32	117.88	111.58	1	1	110.5	186.74	166.76	154.67	71.56	61.8	66.68	0.43	0.08054
Solyc01g005590.2.1	Unknown Protein (AHRD V1)	181.95	144.89	123.02	179.11	157.24	247.2	174.73	256.32	144.38	205.66	1.31	0.539908	188.05	103.6	146.49	146.05	45.02	78.23	56.86	60.04	0.41	0.059283	1.72	3.47	3.17	2.79	1.4	1.61	6.98	3.33	1.19	1	0	0.77	0	0.26	1.95	0.93	1.44	5.61	1
Solyc01g005600.2.1	Adenosine 5&apos-phosphos	46.03	59.49	58.18	54.87	54.64	60.91	48.8	60.45	53.18	55.84	1.02	1	50.36	55.63	56.46	54.15	59.59	54.92	52.19	55.57	1.03	1	65.52	65.9	65.7	65.71	41.32	63.12	56.22	53.55	0.82	1	122.15	75.61	71.11	89.62	55.37	58.8	57.08	0.64	0.991189
Solyc01g005610.2.1	GDSL esterase/lipase At1g09	0	0	0	0	0	0	0	0	0	0	1	NA	0	0	0	0	0	0	0	0	1	NA	0	0	0	0	0	0	0	0	1	NA	0	0	0	0	0	0	0	1	NA
Solyc01g005620.2.1	Mitochondrial 2-oxoglutarate	372.8	351.1	434.61	481.61	410.03	469.03	405.83	371.88	428.33	418.77	1.02	1	273.26	255.75	295.81	274.94	241.99	250.11	276.97	256.36	0.93	1	225.36	215.39	256	232.25	241.56	272.32	222.62	245.5	1.06	1	81.84	117.05	102.13	100.34	218.46	192.16	205.31	2.05	0.221439
Solyc01g005630.1.1	Ethylene responsive transcrip	0	0	0	0	0	0	0	0	0	0	1	NA	0	0	0	0	0	0	0	0	1	NA	0	0	0	0	0	0	0	0	1	NA	0	0	0	0	0	0	0	1	NA

Solyc01g005640.2.1	Uncharacterized plant-specifi	27.52	35.77	34.79	16.07	28.54	26.71	24.49	35.55	31.52	29.57	1.04	1	32.31	24.69	21.03	26.01	20.15	28.73	27.64	25.51	0.98	1	64.24	41.48	40.33	48.68	51.66	45.86	45.21	47.58	0.98	1	15.84	29.03	26.2	23.69	36.19	29.69	32.94	1.39	1
Solyc01g005650.1.1	Ariadne-like ubiquitin ligase (	0	0	0	0	0	0	0	0	0	0	1	NA	0	0	0	0	0	0	0	0	1	NA	0	0	0	0	0	0	0	0	1	NA	0	0	0	0	0	0	0	1	NA
Solyc01g005660.1.1	Myb transcription factor (AHI	0.21	0	0.25	0	0.11	0	0	0	0.15	0.04	0.33	1	0	0.37	0.9	0.42	0.44	0.33	0.5	0.42	1	1	0	0	0	0	0	0.16	0	0.05	5.33	1	0	0	0	0	0	0	0	1	NA
Solyc01g005670.2.1	Disease resistance protein (A	5.69	9.31	5.9	8.37	7.32	11.38	8.08	11.5	6.04	9.25	1.26	0.843588	10.79	6.92	8.65	8.79	10.26	8.36	12.34	10.32	1.17	0.988859	5.51	6.3	4.52	5.44	5.83	6.55	5.3	5.89	1.08	1	4.85	9.09	7.7	7.21	5.83	3.99	4.91	0.68	1
Solyc01g005680.2.1	Cytokinin riboside 5'-m	0	0	0	0	0	0	0	0	0	0	1	NA	0	0	0	0	0	0	0	0	1	NA	0	0	0	0	0	0	0	0	1	NA	0	0	0	0	0	0	0	1	NA
Solyc01g005690.2.1	Unknown Protein (AHRD V1)	40.12	43.77	41.72	45.61	42.8	44.43	38.01	50.27	40.86	43.39	1.01	1	48.24	42.88	43.3	44.81	27.82	41.08	44.02	37.64	0.84	0.973558	20.29	27.36	18.29	21.98	24.99	17.57	22.09	21.55	0.98	1	22.18	42.84	38.16	34.39	21.35	26.32	23.84	0.69	0.857888
Solyc01g005700.2.1	Mutator-like transposase-like	6.68	6.33	9.33	5.84	7.04	4.2	4.79	4.93	7.69	5.4	0.77	0.837173	6.95	5.42	7.12	6.5	6.82	6.32	6.49	6.54	1.01	1	3.05	3.94	4.01	3.67	2.83	3.6	3.39	3.27	0.89	1	3.12	4.08	3.78	3.66	3.46	2.7	3.08	0.84	1
Solyc01g005710.2.1	LRR receptor-like serine/thre	0	0	0.45	0	0.11	0	0.3	1.05	0	0.34	3	1	0.71	1.98	0.32	1	0.26	0.2	0	0.15	0.15	0.669164	0	0	0	0	0	0	0	0	1	1	0	0	0	0	0	0	0	1	NA
Solyc01g005720.2.1	LRR receptor-like serine/thre	6.23	6.05	4.79	5.47	5.63	3.78	4.62	6.33	5.15	4.97	0.88	1	4.83	10.42	6.38	7.21	5.04	2.25	4.9	4.06	0.56	0.516484	7.17	4.31	9.84	7.11	8.06	12.59	12.72	11.12	1.57	0.867269	0.67	4.11	3.48	2.75	4.32	2.65	3.49	1.27	1
Solyc01g005730.2.1	LRR receptor-like serine/thre	5.09	4.39	2.91	3.26	3.91	5.57	7.73	8.12	6.63	7.01	1.79	0.046591	4.44	5.05	2.42	3.97	2.04	1.98	3.56	2.53	0.64	0.665104	3.15	1.62	3.95	2.91	4.34	5.61	4.87	4.94	1.7	1	0.23	3.58	3.3	2.37	1.68	1.47	1.58	0.66	1
Solyc01g005740.2.1	Unknown Protein (AHRD V1)	2.64	4.7	0	3.73	2.77	2.93	0	0	1.89	1.21	0.44	1	7.36	9.18	0	5.51	9.15	1.37	0	3.51	0.64	1	108.73	60.21	83.82	84.25	72.81	122	211.53	135.45	1.61	1	0	7.67	20.29	9.32	2.42	0	1.21	0.13	1
Solyc01g005750.1.1	Lipoxygenase (AHRD V1 ***-	0.71	0	2.51	0	0.8	0.79	1.68	3.26	0	1.43	1.78	1	0	0	0.6	0.2	1.48	0.37	0	0.62	3.08	1	0	0	0	0	0	0	0	0	1	NA	0	0	1.09	0.36	0	0	0	0.03	1
Solyc01g005760.2.1	LRR receptor-like serine/thre	56.93	46.54	33.36	38.79	43.9	35.64	37.26	74.08	49.03	49	1.12	1	33.27	82.93	42.67	52.96	36.49	24.56	45.83	35.63	0.67	0.861151	67.84	45.45	80.36	64.55	95.51	114.82	129.19	113.17	1.75	0.315611	7.25	56.22	48.7	37.39	34.65	25.94	30.3	0.81	1
Solyc01g005770.2.1	Unknown Protein (AHRD V1)	2.79	0.83	0	2.63	1.56	3.1	2.93	0.85	2	2.22	1.42	1	0.86	0	2.35	1.07	1.29	0.48	0.73	0.83	0.78	1	0.75	0.76	0	0.5	1.22	0.7	0	0.64	1.27	1	1.32	0.67	0	0.66	0.85	0	0.42	0.64	1
Solyc01g005780.1.1	LRR receptor-like serine/thre	2.32	2.21	4.66	3.03	3.05	0.76	0.61	1.1	2.09	1.14	0.37	0.12391	1.2	5.44	2.1	2.91	0.89	0.62	1.28	0.93	0.32	0.590698	0.28	0.28	0.43	0.33	0.68	0.59	1.25	0.84	2.55	1	0.12	1.25	0.92	0.76	0	0.23	0.12	0.15	0.698088
Solyc01g005790.1.1	Pentatricopeptide repeat-con	4.89	4.22	3.73	2.24	3.77	3.83	4.2	3.44	4.33	3.95	1.05	1	4.41	3.62	2.91	3.65	5.48	4.99	3.84	4.77	1.31	0.789611	4.18	3.75	3.28	3.74	3.21	3.16	4.29	3.55	0.95	1	4.9	1.88	3.76	3.51	2.11	3.28	2.7	0.77	1
Solyc01g005800.2.1	Calmodulin binding protein (A	0.6	0.62	0.82	0.42	0.61	0.33	0.63	0.18	0.14	0.32	0.52	0.786173	0.28	0.35	0.34	0.32	0	0.15	0.16	0.1	0.32	0.843006	0.4	0.08	0.2	0.23	0.13	0	0	0.04	0.19	1	0.14	0.22	0	0.12	0	0	0	0.08	1
Solyc01g005810.2.1	Mak16 protein (AHRD V1 *-*-	143.26	150.76	135.63	108.71	134.59	138.68	112.76	192.58	124.89	142.23	1.06	1	147.03	168.07	155.94	157.01	156.9	194.69	198.65	183.41	1.17	0.765494	61.21	82.68	44.09	62.66	128.01	86.97	90.91	101.96	1.63	1	76.77	92.03	92.41	87.07	87.9	108.05	97.97	1.13	1
Solyc01g005820.2.1	Splicing factor arginine/serine	222.95	189.83	214.11	231.63	214.63	182.61	181.77	156.08	171.85	173.08	0.81	0.786006	207.86	235.51	244.47	229.28	163.49	166.6	165.17	165.09	0.72	0.399332	108.49	128.98	128.12	121.86	128.47	121.74	113.06	121.09	0.99	1	210.95	134.47	149.96	165.13	137.49	169.64	153.56	0.93	1
Solyc01g005830.2.1	Glucan endo-1 3-beta-glucosi	19.69	21.91	22.94	24.03	22.14	26.54	37.51	24.44	23.86	28.09	1.27	0.524971	19	16.48	20.79	18.76	12.63	17.21	15.86	15.23	0.81	0.980282	4.77	9.83	2.2	5.6	3.07	1.12	5.02	3.07	0.55	1	1.05	0.72	2.65	1.47	2.25	3.24	2.75	1.86	0.999777
Solyc01g005840.2.1	Ubiquitin-conjugating enzyme	21.39	25.81	20.53	16.92	21.16	24.98	16.6	23.66	22.38	21.9	1.04	1	20.23	18.59	15.92	18.25	15.59	18.96	19.66	18.07	0.99	1	24.89	24.52	14.03	21.15	11.91	21.98	21.66	18.52	0.88	1	14.43	14.22	13.38	14.01	23.23	14.94	19.09	1.36	1
Solyc01g005850.1.1	Extensin-like protein Dif54 (A	0.44	0.79	0	1.26	0.62	0.99	0.88	0.2	1.12	0.8	1.28	1	0.21	0.19	0.56	0.32	1.69	1.15	0.7	1.18	3.69	0.466702	0	0.36	0.22	0.19	0.15	0.17	0.65	0.32	1.67	1	1.58	0.48	0	0.69	0.81	0.58	0.7	1.01	1
Solyc01g005860.2.1	Unknown Protein (AHRD V1)	0	0	0	0	0	0.85	0	0	0	0.21	21.25	1	0	0	0	0	0	0	0	0	1	NA	0	0	0	0	0	0	0	0	1	NA	0	0	0	0	0	0	0	1	NA
Solyc01g005870.1.1	LRR receptor-like serine/thre	5.3	3.61	6.54	2.11	4.39	2.21	2.03	4.34	3.45	3.01	0.69	0.773327	3.08	5.47	3.64	4.06	2.41	1.16	2.41	1.99	0.49	0.136513	2.61	2.9	4.77	3.43	4.3	5.52	5.91	5.24	1.53	1	0.12	1.2	1.65	0.99	1.06	1.81	1.44	1.45	1
Solyc01g005880.1.1	Extensin-like protein Dif10 (F	0.19	0	0	0	0.05	0	0	0	0	0	0.21	1	0	0	0	0	0	0	0.15	0.05	5	1	0	0	0	0	0.12	0	0	0.04	4	1	0	0	0	0	0	0	0	1	NA
Solyc01g005890.1.1	Unknown Protein (AHRD V1)	0	0	0	0	0	0	0	0	0	0	1	NA	0	0	0	0	0	0	0	0	1	NA	0	0	0	0	0	0	0	0	1	NA	0	0	0	0	0	0	0	1	NA
Solyc01g005900.2.1	Transferase family protein (A	5.04	9.18	3.68	3.73	5.41	12.53	9.66	11.25	7.75	10.3	1.9	0.165596	9.15	4.17	5.47	6.26	4.83	9.69	7.54	7.35	1.17	1	6.98	7.82	5	6.6	3.94	4	6.47	4.8	0.73	1	1.36	2.96	0.37	1.56	2.42	2.1	2.26	1.45	1
Solyc01g005910.2.1	Acid phosphatase/vanadium-	20.26	26.39	17.2	14.68	19.63	32.68	29.85	30.44	20.93	28.48	1.45	0.363904	14.71	22.12	16.9	17.91	17.82	17.91	25.05	20.26	1.13	1	15.37	18.12	12.02	15.17	10.71	12.36	17.29	13.45	0.89	1	5.62	17.23	18.62	13.82	15.38	18.41	16.89	1.22	1
Solyc01g005920.2.1	Amino acid permease (Fragm	0.83	0.74	0	0.2	0.44	1.08	0.66	1.15	0.4	0.82	1.86	0.914991	0.9	0.36	1.17	0.81	0.67	0.72	0.65	0.68	0.84	1	0.56	0.45	0.14	0.38	0.27	0.21	0.2	0.23	0.59	1	0	0.3	0.64	0.31	0.25	0	0.12	0.4	1
Solyc01g005930.2.1	Unknown Protein (AHRD V1)	4.39	6.8	7.48	3.45	5.53	5.06	4.11	8.52	5.37	5.76	1.04	1	7.71	7.91	6.72	7.45	5.63	8.08	11.75	8.49	1.14	1	13.52	12.71	9.68	11.97	8.33	12.19	11.25	10.59	0.88	1	3.93	11.21	9.74	8.29	7.44	8.55	8	0.96	1
Solyc01g005940.2.1	Phytoene synthase 3	0	0	0	0	0	0	0	0	0	0	1	NA	0	0	0	0	0	0	0.13	0.04	4.33	1	0	0	0.17	0.06	0	0	0	0	0.18	NA	0	0	0	0	0	0	0	1	NA
Solyc01g005950.2.1	Serine/threonine-protein pho	52.31	61.33	53.54	56.17	55.84	49.04	60.21	57.76	52.19	54.8	0.98	1	51.59	54.43	59.45	55.16	47.21	53.63	53.66	51.5	0.93	1	67.56	77.55	60.06	68.39	60.04	66.56	80.84	69.15	1.01	1	39.73	38.91	42.32	40.32	43.47	49.55	46.51	1.15	1
Solyc01g005960.2.1	ATP dependent RNA helicase	25.63	21.84	23.54	25.14	24.04	22.42	22.3	15.14	21.37	20.31	0.84	0.951897	19.58	18.96	23.85	20.8	19.21	23.26	19.97	20.81	1	1	21.94	24.79	26.3	24.34	19.2	20.66	24	21.29	0.87	1	14.11	15.92	12.58	14.2	21.66	20.94	21.3	1.5	0.930886
Solyc01g005970.2.1	Kelch-like protein 3 (AHRD V1	4.52	6.84	6.57	7.24	6.29	3.01	3.09	4.71	3.68	3.62	0.58	0.295028	4.76	4.58	6.11	5.15	3.87	4.29	5.8	4.65	0.9	1	2.92	3.31	3.74	3.32	2.47	2.17	2.85	2.5	0.75	1	4.71	3.5	4.4	4.2	5.1	4.36	4.73	1.13	1
Solyc01g005980.2.1	POT family domain containing	9.39	10.44	16.3	12.91	12.26	10.43	9.26	18.7	10.67	12.27	1	1	10.91	12.24	14.98	12.71	11.03	11.75	10.45	11.08	0.87	1	3.37	3.82	3.62	3.6	2.4	0.99	2.66	2.02	0.56	1	0	0.38	0	0.13	0.48	0.91	0.7	5.49	1
Solyc01g005990.2.1	Non-specific lipid-transfer pro	46.93	42.98	34.5	31.27	38.92	52.16	44.03	45.02	60.79	50.5	1.3	0.518791	45.55	33.7	36.19	38.48	15.57	14.44	16.57	15.53	0.4	0.000745	3.57	3.6	1.35	2.84	0.89	0	0.74	0.54	0.19	0.928032	0	0.25	0	0.08	0	0	0	0.12	1
Solyc01g006000.2.1	GPI mannosyltransferase 2 (A	9.35	15.16	13.15	10.69	12.09	20.22	16.28	17.31	12.23	16.51	1.37	0.455311	15.13	13.92	16.66	15.24	11.11	14.59	15.6	13.77	0.9	1	7.14	7.52	9.28	7.98	7.99	6.31	6.35	6.88	0.86	1	5.65	4.37	4.14	4.72	5.28	9.32	7.3	1.55	0.939829
Solyc01g006010.2.1	Branchpoint-bridging protein	9.03	6.35	11.81	9.52	9.18	7.66	10.88	5.28	7.72	7.88	0.86	1	8.78	8.13	11.85	9.59	11.27	10.41	10.19	10.62	1.11	0.9964	9.68	10.97	9.85	10.17	9.54	8.71	9.57	9.27	0.91	1	18.9	8.52	10.85	12.76	15.85	17.41	16.63	1.3	1
Solyc01g006020.2.1	At3g32930-like protein (Frag	27.71	30.77	24.91	32.1	28.87	38.72	42.5	36.15	48.12	41.37	1.43	0.19065	17.92	24.77	23.63	22.11	56.19	39.76	41.27	45.74	2.07	0.002657	18.97	29.67	15.19	21.28	18.72	23.8	38.07	26.86	1.26	1	7.13	11.12	7.3	8.52	17.15	18.28	17.71	2.08	0.618249
Solyc01g006030.2.1	E3 ubiquitin-protein ligase br	12.22	12.78	11.22	9.06	11.32	8.63	10.57	9.63	11.14	9.99	0.88	1	13.18	12.82	13.93	13.31	12.92	15.87	12.2	13.66	1.03	1	15.68	13.12	15.21	14.67	15.04	15.15	14	14.73	1	1	13.79	14.94	16.71	15.15	15.45	11.06	13.25	0.88	1
Solyc01g006040.2.1	E3 ubiquitin-protein ligase BR	7.95	7.81	8.54	6.38	7.67	6.33	7.77	9.27	6.99	7.59	0.99	1	9.05	7.12	8.72	8.3	10.04	9.58	9.7	9.77	1.18	0.895788	10.64	10.06	9.78	10.16	9.59	12.32	11.59	11.17	1.1	1	10.46	11.03	9.85	10.45	10.64	10.51	10.57	1.01	1
Solyc01g006050.2.1	Plant viral-response family pr	0	0	0	0	0	0	0	0	0	0	1	NA	0	0	0	0	0	0	0.33	0.11	11	1	0	0	0.21	0.07	0	0	0.15	0.05	0.71	1	0	0.15	0	0.05	0.19	0.18	0.18	3.7	1
Solyc01g006060.2.1	Disease resistance response p	0.68	0	0	0	0.17	0	0	0	0	0	0.06	1	0	0	0	0	0	0	0	0	1	NA	0	0	0	0	0	0	0	0	1	NA	0	0	0	0	0	0	0	1	NA
Solyc01g006070.2.1	Plant viral-response family pr	0	0	0	0	0	0	0	0	0	0	1	NA	0	0	0	0	0	0	0	0	1	NA	0	0	0	0	0	0	0	0	1	NA	0	0	0	0	0	0	0	1	NA
Solyc01g006080.1.1	Protein involved in mRNA tur	0	0	0	0.35	0.09	0	0	0	0	0	0.11	1	0	0	0	0	0	0	0	0	1	NA	0	0	0	0	0	0	0.18	0.06	6	1	0	0	0	0	0	0	0	1	NA
Solyc01g006090.2.1	rRNA processing protein Ebp2	14.22	12.24	10.18	8.9	11.38	3.37	7.73	7.5	7.03	6.41	0.56	0.204083	9.54	10.54	8.86	9.65	4.85	10.38	10.08	8.44	0.87	1	3.2	6.84	5.09	5.04	6.13	4.42	6.46	5.67	1.12	1	10.94	19.13	22.94	17.67	16	21.05	18.52	1.05	1
Solyc01g006100.2.1	Cytosine-specific methyltrans	1.47	1.16	0.96	0.92	1.13	0.91	2.32	1.87	1.7	1.7	1.51	0.576732	1.37	1.49	1.86	1.57	1.53	1.18	0.64	1.12	0.71	0.955554	0.33	0.53	0.16	0.34	0.05	0.06	0.3	0.14	0.4	1	0.12	0.06	0.13	0.1	0.37	0.14	0.26	2.47	1
Solyc01g006110.2.1	PP2A regulatory subunit TAP4	50.37	48.16	47.93	48.04	48.62	41.9	45.41	51.84	43.4	45.64	0.94	1	45.95	50.57	46.94	47.82	41.59	41.06	44.82	42.49	0.89	1	51.46	55.74	51.77	52.99	64.15	61.95	49.02	58.37	1.1	1	84.79	96.54	99.2	93.51	61.28	65.96	63.62	0.68	0.875062
Solyc01g006120.2.1	Casein kinase-like protein (AH	66.26	66.36	72.82	77.33	70.69	45.52	47.81	49.73	57.9	50.24	0.71	0.400961	80.23	89.59	104.1	91.31	81.46	76.47	67.94	75.29	0.82	0.892092	66.02	63.52	81.4	70.31	84.58	71.09	60.11	71.93	1.02	1	132.11	132.94	135.91	133.65	100.44	102.81	101.62	0.76	1
Solyc01g006130.2.1	Homology to unknown gene (	165.07	161.53	110.46	105.54	135.65	402.53	381.64	359.17	322.23	366.39	2.7	2.2E-08	117.1	129.72	116.31	121.04	239.77	219.71	355.22	271.57	2.24	0.005806	43.52	49.3	33.46	42.09	68.7	64.85	91.11	74.89	1.78	0.824187	28.67	54.4	38.1	40.39	73.05	77.29	75.17	1.86	0.587552
Solyc01g006140.2.1	DNA replication factor Cdt1 (	1.04	0.31	0.68	0.49	0.63	0.64	0.55	2.01	1.9	1.27	2.02	0.525044	0.75	0.7	0.49	0.65	0.16	0.24	0.81	0.4	0.62	0.979267	0.09	1.22	0.46	0.59	0	0	0.08	0.03	0.05	1	0	0.25	0	0.08	1.37	1.11	1.24	14.88	0.291835
Solyc01g006150.2.1	Cation-efflux pump fieF (AHR	15.76	22.7	15.91	15.17	17.39	18.78	19.63	17.48	20.41	19.07	1.1	0.993052	12.09	12.71	10.84	11.88	7.89	10.6	11.75	10.08	0.85	1	9.32	15.62	7.58	10.84	5.98	7.27	11.49	8.25	0.76	1	0.69	1.06	1	0.92	11.16	7.69	9.43	10.28	7.24E-05
Solyc01g006160.2.1	Unknown Protein (AHRD V1)	1088.52	922.08	1106.45	1126.15	1060.8	1407.47	1348.23	1369.59	1016.68	1285.49	1.21	0.555626	949.07	927.98	861.14	912.73	921.36	1055.78	946.76	974.63	1.07	0.991848	413.67	495.42	328.16	412.42	501.84	388.65	481.29	457.26	1.11	1	246.22	273.33	338.92	286.16	475.58	561.99	518.78	1.81	0.340536
Solyc01g006170.2.1	rRNA processing protein ebna	34.93	23.57	18.04	20.34	24.22	8.63	19.28	16.58	17.21	15.43	0.64	0.464268	28.03	27.78	28.34	28.05	15.99	26.59	26.19	22.92	0.82	0.962977	12.36	25.86	23.79	20.67	23.39	15.23	18.3	18.97	0.92	1	46.41	90.86	119	85.42	73.56	96.27	84.91	0.99	1
Solyc01g006180.2.1	GTP-binding protein-like (AHI	11.64	6.15	6.86	8.85	8.38	4.27	7.45	6.06	8.83	6.65	0.79	0.980122	9.8	9.26	8.72	9.26	7.31	8.24	9.58	8.38	0.9	1	4.79	5.9	5.23	5.31	5.12	3.91	5.12	4.72	0.89	1	5.15	4.78	4.22	4.72	4.12	3.27	3.7	0.78	1
Solyc01g006190.2.1	E3 ubiquitin-protein ligase sir	71.95	74.48	90.43	90.6	81.87	66.21	63.38	70.67	65.3	66.39	0.81	0.835634	89.76	111.17	81.33	94.09	55.38	72.74	84.06	70.73	0.75	0.588213	86.76	83.68	77.56	82.67	83.59	76.54	77.48	79.2	0.96	1	108.06	81.1	87.66	92.27	72.12	68.89	70.5	0.76	1
Solyc01g006200.2.1	Unknown Protein (AHRD V1)	55.82	79.04	41.91	43.07	54.96	76.15	54.09	79.32	43.72	63.32	1.15	1	56.64	61.76	36.43	51.61	45.76	55.18	101.7	67.55	1.31	0.96542	94.3	84.77	90.67	89.91	76.68	67.31	103.54	82.51	0.92	1	64.94	125.32	206.8	132.35	109.17	88.99	99.08	0.75	1
Solyc01g006210.2.1	Non-structural maintenance c	1.9	3.38	3.23	1.79	2.58	0.7	2.66	2.91	2.27	2.14	0.83	1	2.74	1.47	2.67	2.29	1.02	1.75	1.82	1.53	0.67	0.971575	0.51	0.52	0.21	0.41	0.14	0.48	0.61	0.41	0.99	1	0.3	1.07	0.65	0.67	0.77	0.55	0.66	0.98	1
Solyc01g006220.2.1	Histone-lysine N-methyltrans	4.52	4.42	5.17	4.95	4.76	4.06	4.26	4.43	4.19	4.24	0.89	1	4.34	2.92	3.7	3.65	2.94	3.55	2.52	3	0.82	1	1.06	1.55	2.61	1.74	2.01	1	1.81	1.61	0.92	1	0.83	0.74	0.45	0.67	0.53	1.28	0.91	1.34	1
Solyc01g006230.2.1	Cysteine protease (AHRD V1 *	20.36	24.41	24.22	26.42	23.85	26.19	21.26	24.76	21.26	23.37	0.98	1	25.67	26.77	23.31	25.25	28.95	24.49	22.88	25.44	1.01	1	62	69.39	63.48	64.96	42.9	47.71	64.02	51.54	0.79	1	45.94	52.32	47.1	48.45	45.76	33.5	39.63	0.82	1
Solyc01g006240.2.1	Myrosinase-binding protein-li	2.65	2.7	0.45	0.54	1.58	3.79	4.79	12.9	3.81	6.32	3.99	0.303781	1.06	0	1.28	0.78	1.84	0.79	0.3	0.98	1.25	1	0.31	0.31	0	0.21	0	0	0.55	0.18	0.89	1	0	0	0	0	0	0	0	1	NA
Solyc01g006250.1.1	Unknown Protein (AHRD V1);	10.56	4.7	3.11	4.98	5.84	9.78	5.56	3.23	6.95	6.38	1.09	1	5.73	5.35	3.71	4.93	10.98	9.11	7.6	9.23	1.87	0.652011	2.84	9.32	0.87	4.34	4.04	10	8.97	7.67	1.77	1	5	4.47	2.71	4.06	0	7.71	3.85	0.95	1
Solyc01g006260.2.1	Ribonuclease P/MRP protein	11.37	9.72	15.03	13.51	12.41	12.64	15.8	11.71	14.37	13.63	1.1	0.981941	6.35	9.89	11.91	9.38	17.36	13.9	13.94	15.07	1.61	0.447338	4.78	7.41	4.97	5.72	7.47	3.45	2.98	4.63	0.81	1	3.88	1.65	4.2	3.24	7.91	5.58	6.75	2.08	0.862434
Solyc01g006270.2.1	Unknown Protein (AHRD V1)	0	0	0	0	0	0	0	2.43	0	0.61	60.75	1	0	0	2.23	0.74	0	0	0	0	0.01	1	0	0	0	0	0	0	0	0	1	NA	0	0	0	0	0	0	0	1	NA
Solyc01g006280.2.1	Formate-tetrahydrofolate liga	96	93.66	148.8	166.06	126.13	89.27	93.67	79.28	94.58	89.2	0.71	0.631966	93.55	79.83	121.09	98.16	86.4	81.23	68.99	78.87	0.8	0.820371	86.37	83.56	159.43	109.79	50.71	46.3	74.95	57.32	0.52	1	27.92	43.46	29.08	33.49	94.25	40.17	67.21	2.01	0.968537
Solyc01g006290.2.1	Peroxidase (AHRD V1 **** Q	0	0	0	0	0	0	0	0	0	0	1	NA	0	0	0	0	0	0	0	0	1	NA	0.32	3.28	0.2	1.27	0	0	0.29	0.1	0.08	1	0.29	0	0	0.1	0	0.18	0.09	0.93	1
Solyc01g006300.2.1	Peroxidase (AHRD V1 **** Q	0	0	0	0	0	0	0	0	0	0	1	NA	0	0	0	0	0	0	0	0	1	NA	0.82	2.8	0	1.21	0	0	0.44	0.15	0.12	1	0	0	0	0	0	0	0	1	NA
Solyc01g006310.2.1	Peroxidase (AHRD V1 ***- D7	0	0	0	0	0	0	0	0	0	0	1	NA	0	0	0	0	0	0	0	0	1	NA	0	0	0	0	0	0	0	0	1	NA	0	0	0	0	0	0	0	1	NA
Solyc01g006320.2.1	Non-race specific disease resi	26.52	13.28	9.39	25.79	18.74	5.16	9.42	7.62	5.95	7.04	0.38	0.179757	3.7	20.46	12.6	12.25	4.14	3.09	2.87	3.37	0.27	0.410746	0.8	0.27	0.33	0.47	1.52	0.75	0.24	0.84	1.79	1	0	0.96	0.51	0.49	0	0.29	0.14	0.3	1
Solyc01g006330.2.1	PAP fibrillin family protein (A	0.66	0.59	1.17	0.23	0.66	0.55	0.13	1.52	0.83	0.76	1.14	1	0.46	0.43	0.42	0.44	0.46	0.68	1.3	0.81	1.86	0.948858	1.2	0.27	0	0.49	0.22	0	0.36	0.19	0.39	1	0	0.36	0.25	0.2	0.3	0.58	0.44	2.16	1
Solyc01g006340.2.1	ATP dependent RNA helicase	6.15	7.18	8.16	7.43	7.23	6.27	7.38	7.42	6.16	6.81	0.94	1	7.03	7.35	6.49	6.96	7.82	7.16	6.04	7.01	1.01	1	7.04	8.56	4.7	6.77	6.23	7.86	8.31	7.47	1.1	1	5.65	6.14	3.74	5.18	6.8	7.41	7.11	1.37	1
Solyc01g006350.2.1	Glucan synthase like 7 (AHRD	18.31	26.59	23.47	20.67	22.26	22.12	17.47	29.09	24.14	23.21	1.04	1	22.77	26.58	24.34	24.56	18.73	23.95	27.91	23.53	0.96	1	23	20.18	21.61	21.6	18.7	18.46	19.55	18.9	0.88	1	25.29	20.89	27.35	24.51	18.86	21.87	20.37	0.83	1
Solyc01g006360.2.1	Glucan synthase like 7 (AHRD	10.88	16.6	16.71	15.76	14.99	7.58	10.16	9.37	13.39	10.12	0.68	0.389899	19.2	18.77	20.49	19.49	17.61	16.99	15	16.53	0.85	1	14.16	16.75	15.6	15.5	13.67	10.93	14.09	12.9	0.83	1	13.63	10.98	13.94	12.85	14.31	20.06	17.18	1.34	0.98833
Solyc01g006370.2.1	Glucan synthase like 3 (AHRD	25.93	29.16	28.11	33.32	29.13	18.44	25.6	18.36	24.3	21.68	0.74	0.571426	19.17	20.54	26.02	21.91	12.98	18.17	17.4	16.18	0.74	0.528578	7.03	7.25	6.79	7.02	5.38	5.11	6.24	5.58	0.79	1	2.26	2.73	3.23	2.74	4.4	5.1	4.75	1.73	0.623006
Solyc01g006380.2.1	U-box domain-containing pro	0.29	0.13	1.21	0	0.41	0	0	0.14	0.21	0.09	0.21	0.863123	0.14	0.13	0.25	0.17	0	0.08	0	0.03	0.15	1	0	0	0	0	0	0	0	0	1	NA	0	0	0	0	0	0	0	1	NA
Solyc01g006390.2.1	Cysteine-rich extensin-like pro	0	0	0	0	0	0	0	0	0	0	1	NA	0.44	0	0	0.15	0	0	0	0	0.07	1	0	0	0	0	0	0	0	0	1	NA	0	0	0	0	0	0	0	1	NA
Solyc01g006400.2.1	Cysteine-rich extensin-like pro	50.26	45.9	80.92	47.77	56.21	38.12	32.04	14.92	32.89	29.49	0.52	0.154837	1.46	7.74	14.37	7.86	1.45	4.34	1.44	2.41	0.31	0.735043	1.27	1.71	0.78	1.25	0.52	0.4	1.34	0.75	0.6	1	0	0.57	0.81	0.46	0	0	0	0.02	1
Solyc01g006410.2.1	Cysteine-rich extensin-like pro	0	0	0	0	0	0	0	0	0	0	1	NA	0	0	0	0	0	0	0	0	1	NA	0	0	0	0	0	0	0	0	1	NA	0	0	0	0	0	0	0	1	NA
Solyc01g006420.2.1	Unknown Protein (AHRD V1)	0	0	0	0	0	0	0	0	0	0	1	NA	0	0	0	0	0	0	0	0	1	NA	0	0	0	0	0	0	0	0	1	NA	0	0	0	0	0	0	0	1	NA
Solyc01g006430.2.1	Omega-6 fatty acid desaturas	163.04	143.27	234.51	218.47	189.82	95.68	133.19	68.24	112.61	102.43	0.54	0.086288	236.99	219.83	299.21	252.01	229.26	141.17	138.79	169.74	0.67	0.401266	40.25	59.92	53.03	51.07	49.43	34.9	31.3	38.54	0.75	1	1796.81	977.52	1145.5	1306.61	1599.72	2369.29	1984.51	1.52	0.738499
Solyc01g006440.1.1	Peptide transporter-like prote	0	0	0	0.16	0.04	0	0	0	0	0	0.25	1	0.1	0	0	0.03	0	0	0.09	0.03	0.9	1	0	0	0	0	0	0	0	0	1	NA	0	0	0	0	0	0	0	1	NA
Solyc01g006450.2.1	Enoyl reductase (AHRD V1 **	42.13	43.03	39.02	50.58	43.69	50.01	71.03	37.81	45.68	51.13	1.17	0.724374	26.7	25.39	25.8	25.96	24.57	25.55	27.84	25.99	1	1	20.21	17.37	25.99	21.19	14.67	18.7	22.14	18.5	0.87	1	6.92	8.66	5.42	7	24.28	18.4	21.34	3.05	0.047635
Solyc01g006460.1.1	Non-structural maintenance c	0	0	0	0	0	0	0	0	0	0	1	NA	0	0	0	0	0	0	0	0	1	NA	0	0	0	0	0	0	0	0	1	NA	0	0	0	0	0	0	0	1	NA
Solyc01g006470.1.1	Bromodomain protein (AHRD	0	0.2	0	0	0.05	0	0	0	0	0	0.2	1	0	0	0	0	0	0	0	0	1	NA	0	0	0	0	0	0	0	0	1	NA	0	0	0	0	0	0	0	1	NA
Solyc01g006480.2.1	Alpha/beta hydrolase (AHRD	19.49	21.8	13.46	14.17	17.23	16.09	13.63	16.64	16.79	15.79	0.92	1	19.16	16.7	12.23	16.03	19.89	20.61	19.25	19.92	1.24	0.755862	25.64	22	20.45	22.7	18.83	17.72	26.57	21.04	0.93	1	13.06	15.97	23.54	17.52	19.37	19.77	19.57	1.12	1
Solyc01g006490.2.1	Protein YdiU (AHRD V1 ***- E	7.34	6.43	9.34	9.25	8.09	6.12	4.44	5.27	7	5.71	0.71	0.636919	9.28	9.57	9.88	9.58	11.38	10.34	11.09	10.94	1.14	0.962977	11.49	11.97	14.58	12.68	9.27	11.74	12.46	11.16	0.88	1	12.07	13.25	11.64	12.32	14.91	15.39	15.15	1.23	1
Solyc01g006500.2.1	Pentatricopeptide repeat-con	5.4	7.08	10.66	5.92	7.27	7.2	6.28	7.81	7.26	7.14	0.98	1	7.03	6.57	6.72	6.77	8.89	7.75	8.48	8.37	1.24	0.832635	5.78	5.94	5.09	5.6	5.58	4.09	4.52	4.73	0.84	1	9.02	6.47	6.02	7.17	5.19	6.39	5.79	0.81	1
Solyc01g006510.2.1	L-threonine 3-dehydrogenase	75.61	95	76.96	60.29	76.97	124.21	90.56	118.59	98.45	107.95	1.4	0.263348	81.36	107.93	80.92	90.07	126.6	131.35	143.59	133.85	1.49	0.088385	65.13	69.31	50.22	61.55	69.88	54.36	80.08	68.11	1.11	1	40.84	49.4	31.06	40.43	35.45	44.03	39.74	0.98	1
Solyc01g006520.2.1	Receptor-like protein kinase (	2.52	2.96	2.01	3.79	2.82	3.25	2.95	3.14	4.14	3.37	1.2	0.813369	2.12	3.88	3.29	3.1	4.51	3.83	4.98	4.44	1.43	0.557625	0.39	0.6	0.73	0.57	1.17	0.25	0.59	0.67	1.17	1	0.12	0.59	0.37	0.36	0.37	0.36	0.36	1.01	1
Solyc01g006530.1.1	Receptor-like protein kinase (	0	0	0	0	0	0	0	0	0	0	1	NA	0	0	0	0	0	0	0	0	1	NA	0	0	0	0	0	0	0	0	1	NA	0	0	0	0	0	0	0	1	NA
Solyc01g006540.2.1	Lipoxygenase (AHRD V1 ****	0.29	0.79	0.17	0.42	0.42	1.96	1.16	1.28	0.37	1.19	2.86	0.350446	0.89	2.05	1.12	1.35	16.53	7.39	2.2	8.71	6.43	0.378601	208.62	266.33	205.06	226.67	342.65	521.98	418.68	427.77	1.89	1	13.7	90.86	125.88	76.81	260.01	277.48	268.75	3.5	0.002541
Solyc01g006550.2.1	LRR receptor-like serine/thre	1.24	1.38	0.55	0.88	1.01	1.38	1.66	1.57	1.17	1.44	1.43	0.79626	0.51	2.16	1.51	1.39	3.67	1.61	1.9	2.39	1.72	0.821464	0.75	0.51	0.39	0.55	1.69	1.24	1.64	1.52	2.77	1	0.33	1.19	3.47	1.66	2.85	3.34	3.09	1.86	0.951723
Solyc01g006560.2.1	Lipoxygenase (AHRD V1 ****	0.95	1.04	3.1	0.62	1.43	6.25	4.04	3.36	2.52	4.04	2.83	0.116167	0	0.32	0.19	0.17	1.32	1.4	1.03	1.25	7.35	0.000869	1.18	1.01	0.8	1	1.54	2.77	1.54	1.95	1.96	1	0	0.53	0.79	0.44	0.6	1.02	0.81	1.84	1
Solyc01g006570.1.1	Lipoxygenase (AHRD V1 ***-	0	0	0	0	0	0	0	0	0	0	1	NA	0	0	0	0	0	0	0	0	1	NA	0	0	0	0	0	0	0	0	1	NA	0	0	0	0	0	0	0	1	NA
Solyc01g006580.2.1	2-oxoglutarate-dependent di	0	0.19	0	0	0.05	0	0	0	0	0	0.21	1	0	0	0	0	0	0	0	0	1	NA	0.17	0	0	0.06	0	0	0.15	0.05	0.88	1	0	0	0	0	0	0	0	1	NA
Solyc01g006590.1.1	Receptor-like protein kinase (	0	0.11	0	0	0.03	0	0	0	0	0	0.36	1	0	0	0	0	0	0	0	0	1	NA	0	0	0	0	0	0	0	0	1	NA	0	0	0	0	0	0	0	1	NA
Solyc01g006600.1.1	Non-ribosomal peptide synth	0	0	0	0	0	0	0	0	0	0	1	NA	0	0	0	0	0	0	0	0	1	NA	0	0	0	0	0	0	0	0	1	NA	0	0	0	0	0	0	0	1	NA
Solyc01g006610.1.1	2-oxoglutarate-dependent di	0	0	0.25	0.29	0.14	0	0	0	0.3	0.07	0.56	1	0	0	0	0	0	0	0	0	1	NA	0	0	0	0	0	0	0	0	1	NA	0	0	0	0	0	0	0	1	NA
Solyc01g006620.2.1	Phosphoribosylanthranilate t	0	0	0	0	0	0	0	0	0	0	1	NA	0.08	0	0	0.03	0	0	0	0	0.38	1	0	0	0	0	0	0	0	0	1	NA	0	0	0	0	0	0	0	1	NA
Solyc01g006630.2.1	Oxidoreductase family protei	19.06	23.16	17.08	22.29	20.4	18.13	25.75	17.52	20.79	20.55	1.01	1	18.92	20.07	17.95	18.98	16.9	14.71	18.43	16.68	0.88	1	12.29	22.85	11.1	15.41	13.12	15.83	17.09	15.35	1	1	1.3	4.53	2.82	2.88	8.05	7.23	7.64	2.65	0.521798
Solyc01g006640.1.1	AMP-dependent synthetase a	0	0	0	0	0	0	0	0	0	0	1	NA	0	0	0	0	0	0	0	0	1	NA	0	0	0	0	0	0	0	0	1	NA	0	0	0	0	0	0	0	1	NA
Solyc01g006650.1.1	Ethylene insensitive 3 class tr	0.9	0.09	0.59	0.28	0.47	0.11	1.5	0.55	0.29	0.61	1.32	1	0.37	0.17	0.59	0.38	0.48	0.26	0.47	0.4	1.07	1	0.24	0.41	0.4	0.35	0.2	0.38	0.15	0.24	0.7	1	0.57	0.36	0.61	0.51	0.46	0.53	0.49	0.96	1
Solyc01g006660.1.1	Subtilisin-like serine protease	0.1	0	0	0.14	0.06	0	0.08	0	0	0.02	0.33	1	0	0	0	0	0	0	0	0	1	NA	0	0	0.1	0.03	0.06	0	0	0.02	0.6	1	0.28	0	0	0.09	0	0	0	0.11	1
Solyc01g006670.2.1	UDP-glucosyltransferase HvU	1.9	2.65	1.6	1.53	1.92	3.31	1.28	2.74	1.56	2.22	1.16	1	5.92	3.65	1.83	3.8	8.64	9.12	7.03	8.26	2.17	0.013719	6.57	6.73	4.3	5.87	7.56	5.96	8.29	7.27	1.24	1	2.12	9.74	5.42	5.76	10.54	8.67	9.61	1.67	1
Solyc01g006680.2.1	Transcription factor jumonji j	16.53	16.19	12.48	20.11	16.33	18.2	15.53	10.95	11.04	13.93	0.85	0.962949	12	9.49	15.08	12.19	18.46	19.09	16.11	17.89	1.47	0.24647	22.04	30.31	29.38	27.24	22.05	25.69	31.69	26.48	0.97	1	15.75	20.9	21.1	19.25	30.27	36.53	33.4	1.74	0.618759
Solyc01g006690.2.1	Unknown Protein (AHRD V1)	3.36	3.09	6.65	6.28	4.84	3.49	1.97	3.09	2.88	2.86	0.59	0.498543	2.22	1.41	2.29	1.97	1.5	2.13	2.64	2.09	1.06	1	2.01	0.35	1.29	1.22	1.49	1.56	0.24	1.1	0.9	1	0	0	0	0	0	0.09	0.04	4.5	1

Solyc01g006700.2.1	Downstream neighbor of SON	13.28	12.83	13.58	12.32	13	13.26	11.22	11.73	13.11	12.33	0.95	1	17.17	14.7	17.79	16.55	13.31	17.24	16.06	15.54	0.94	1	12.07	14.71	8.96	11.91	8.65	9.75	9.98	9.46	0.79	1	18.29	18.61	14.83	17.24	15.1	16.37	15.73	0.91	1
Solyc01g006710.2.1	ATP-dependent RNA helicase	6.97	8.21	7.88	8.49	7.89	8.12	8.94	8.75	7.48	8.32	1.06	1	8.01	7.88	8.7	8.2	7.27	7.45	7.3	7.34	0.9	1	5.22	5.86	5.23	5.44	4.43	4.42	4.13	4.33	0.8	1	7.73	11.64	11.15	10.17	5.86	6.6	6.23	0.61	0.698227
Solyc01g006720.2.1	ABC transporter G family mer	3.73	6.07	3.76	0.51	3.52	2.63	2.23	3.68	5.03	3.39	0.96	1	2.62	3.48	3	3.03	1.26	1.18	1.43	1.29	0.43	0.061273	1.47	1.56	0.99	1.34	0.48	0.28	0.33	0.36	0.27	0.63661	0	0.07	0.28	0.12	0	0.08	0.04	0.34	1
Solyc01g006730.2.1	Calcium dependent protein ki	0.34	1.01	0.4	1.12	0.72	0.13	0.18	0.21	0.81	0.33	0.46	0.786661	0.11	0.49	0.48	0.36	0.31	0.29	0.18	0.26	0.72	1	0	0	0	0	0	0	0.08	0.03	2.67	1	0	0	0	0	0	0	0	1	NA
Solyc01g006740.2.1	Sucrose phosphate phosphata	4.84	7	7.14	4.28	5.82	8.24	6.33	4.45	7.17	6.55	1.13	1	5.21	5.26	5.11	5.19	4.09	4.54	5.23	4.62	0.89	1	4.03	2.34	2.1	2.82	1.89	1.26	1.87	1.67	0.59	1	0.22	0.99	0.47	0.56	0.42	0.66	0.54	0.96	1
Solyc01g006750.2.1	Unknown Protein (AHRD V1)	0	0	0	0	0	0	0	0	0	0	1	NA	0	0	0	0	0	0	0	0	1	NA	0	0	0	0	0	0	0	0	1	NA	0	0	0	0	0	0	0	1	NA
Solyc01g006760.2.1	Unknown Protein (AHRD V1)	0	0	0	0	0	0	0	0	0	0	1	NA	0	0	0	0	0	0	0	0	1	NA	0	0	0	0	0	0	0	0	1	NA	0	0	0	0	0	0	0	1	NA
Solyc01g006770.1.1	Glycoprotein-N-acetylgalacto	0	0	0	0	0	0	0	0.12	0	0.03	3	1	0	0	0	0	0	0	0	0	1	NA	0	0	0	0	0	0	0	0	1	NA	0	0	0	0	0	0	0	1	NA
Solyc01g006780.2.1	Unknown Protein (AHRD V1)	0	0	0	0	0	1.08	0.77	0.89	0	0.69	68.5	0.993291	0	0	0	0	0	0	0	0	1	NA	0	0	0	0	0	0	0	0	1	NA	0	0	0	0	0	0	0	1	NA
Solyc01g006790.2.1	Unknown Protein (AHRD V1)	1.24	1.1	3.41	0.58	1.58	5.5	1.95	4.93	5.03	4.35	2.75	0.233926	0.38	0.36	1.74	0.83	2.86	3.2	3.24	3.1	3.75	0.277889	0	0	0	0	0.27	0.63	0.6	0.5	50	1	0	0	0	0	0.75	0.36	0.55	55.5	1
Solyc01g006800.2.1	Methionine aminopeptidase (	81.87	78.29	86.41	69.39	78.99	77.28	66.37	75.28	81.85	75.19	0.95	1	82.45	82.34	86.3	83.7	89.64	91.44	87.07	89.38	1.07	1	50.7	56.79	46.51	51.33	62.34	49.49	57.44	56.42	1.1	1	100.29	84.9	85.58	90.26	95.39	94.18	94.78	1.05	1
Solyc01g006810.2.1	RING-H2 zinc finger protein (F	37.52	33.03	32.11	37.91	35.14	33.23	48.99	32.98	33.89	37.27	1.06	0.9771	27.81	20.97	18.46	22.41	17.87	20.28	18.63	18.93	0.84	1	53.96	56.28	32.54	47.59	30.34	26.41	39.36	32.04	0.67	1	1.47	3.89	4.12	3.16	4.53	5.42	4.97	1.57	1
Solyc01g006820.2.1	Unknown Protein (AHRD V1)	0	1.15	4.58	3.66	2.35	4.31	1.02	0	1.86	1.8	0.77	1	1.2	2.25	3.28	2.24	0	0.67	0	0.22	0.1	0.816675	3.14	1.05	0	1.4	0	0	0.94	0.31	0.22	1	1.84	4.7	5.97	4.17	0	0	0	0	0.848276
Solyc01g006830.2.1	Os02g0200800 protein (Fragr	81.09	73.62	79.58	91.43	81.43	76.13	91.29	87.92	80.99	84.08	1.03	1	68.26	54.9	57.35	60.17	45.24	57.72	49.86	50.94	0.85	1	44.56	40.59	43.56	42.9	49.86	43.48	45.81	46.38	1.08	1	58.6	72.36	66.8	65.92	81.83	80.59	81.21	1.23	1
Solyc01g006840.2.1	Calcium dependent protein ki	0	0	0	0	0	0	0	0	0	0	1	NA	0	0	0	0	0	0	0	0	1	NA	0	0	0	0	0	0	0	0	1	NA	0	0	0	0	0	0	0	1	NA
Solyc01g006850.1.1	Pentatricopeptide repeat-con	0.24	0.59	0.28	0.59	0.42	0.33	0.52	0.72	0.39	0.49	1.15	1	0.73	0.73	0.91	0.79	0.79	0.37	0.52	0.56	0.71	0.961798	0.73	0.69	0.3	0.57	0.36	0.46	0.18	0.33	0.58	1	0.68	0.7	0.83	0.74	0.83	0.95	0.89	1.21	1
Solyc01g006860.2.1	Co-chaperone HscB-like prote	22.89	27.39	19.17	20.89	22.59	35.3	31.87	34.18	24.39	31.43	1.39	0.400569	25.19	29.1	22.66	25.65	24.07	27.28	22.44	24.6	0.96	1	30.43	37.11	23.7	30.41	27.33	25.37	28.7	27.13	0.89	1	16.45	26.46	20.82	21.24	18.7	18.56	18.63	0.88	1
Solyc01g006870.2.1	cDNA clone J023119C16 full i	38.23	45.66	31.97	34.69	37.64	32.74	32.21	34.27	34.68	33.48	0.89	0.98862	28.59	34.07	28.04	30.23	13.52	19.8	20.87	18.06	0.6	0.074083	17.91	15.88	13.61	15.8	13.58	11.36	12.1	12.35	0.78	1	4.72	4.59	5.43	4.91	3.73	6.01	4.87	0.99	1
Solyc01g006880.2.1	Histone-lysine N-methyltrans	6.07	5.66	7.41	5.55	6.17	5.74	5.48	6.8	7.65	6.42	1.04	1	7.26	7.97	10.33	8.52	8.31	8.58	9.54	8.81	1.03	1	5.94	6.66	7.26	6.62	6.4	6.58	7.01	6.66	1.01	1	13.8	8.52	12.64	11.65	7.64	10.29	8.96	0.77	1
Solyc01g006890.2.1	Genomic DNA chromosome 5	8.1	9.89	6.4	23.63	12	5.65	9.04	6.73	6.62	7.01	0.58	0.870142	6.97	14.97	14.55	12.16	6.63	5.31	4.2	5.38	0.44	0.152787	1.67	1.82	3.5	2.33	2.72	0.91	1.38	1.67	0.72	1	0.37	0.69	1.32	0.79	1.26	1.21	1.23	1.56	1
Solyc01g006900.2.1	Phosphatidylglycerol/phosph	432.94	528.43	427.1	787.35	543.95	492.24	492.51	466.29	588.48	509.88	0.94	1	591.79	562.36	457.96	537.37	608.56	652.41	562.84	607.94	1.13	0.822448	333.78	338.34	207.5	293.21	266.8	293.13	322.1	294.01	1	1	111.97	192.56	128.08	144.2	232.13	224.14	228.13	1.58	0.868616
Solyc01g006910.2.1	Ring finger protein (AHRD V1	0	0	0	0	0	0	0	0	0	0	1	NA	0	0	0	0	0	0	0	0	1	NA	0	0	0	0	0	0	0	0	1	NA	0	0	0	0	0	0	0	1	NA
Solyc01g006920.2.1	Pentatricopeptide repeat-con	0.62	0.62	0.18	0.55	0.49	1.04	0.8	0.79	0.56	0.8	1.62	0.804781	0.65	0.81	1.25	0.9	0.97	0.93	0.92	0.94	1.04	1	0.44	0.51	0.39	0.45	0.15	0.35	0.45	0.32	0.71	1	0.33	0.34	1.31	0.66	0.43	0.55	0.49	0.74	1
Solyc01g006930.2.1	Nuclear transcription factor Y	9.3	7.9	12.22	7.48	9.23	10.58	10.35	8.17	10.63	9.93	1.08	1	20.85	16.73	11.61	16.4	13.79	5.8	6.65	8.75	0.53	0.418673	2.73	4.31	2.52	3.19	5.97	4.97	2.93	4.62	1.45	1	1.2	4.45	5.2	3.62	3.87	5.19	4.53	1.25	1
Solyc01g006940.2.1	Poly(U)-binding-splicing facto	65.77	63.53	40.93	50.49	55.18	115.46	104.48	106.19	106.96	108.27	1.96	0.000729	58.75	70.59	55.21	61.52	116.82	121.99	128.27	122.36	1.99	0.000349	31.1	44.12	26.87	34.03	41.89	34.65	51	42.51	1.25	1	2.28	12.96	4.93	6.72	14.68	11.6	13.14	1.95	1
Solyc01g006950.2.1	Syntaxin (AHRD V1 ***- Q6X9	0.66	0.2	0.26	2.17	0.82	0	0	0.2	0	0.05	0.06	0.513059	0.82	2.86	2.96	2.21	0.3	0.34	0.52	0.39	0.17	0.082965	0.71	1.61	1.74	1.35	0	0	0.16	0.05	0.04	1	1.56	7.16	7.41	5.38	0	0.77	0.39	0.07	0.179982
Solyc01g006960.2.1	Transport protein particle sub	10.09	11.34	9.81	9.78	10.26	9.07	10.34	10.47	10.08	9.99	0.97	1	11.22	10.44	11.5	11.05	9.99	9.56	12.01	10.52	0.95	1	7.56	8.73	8	8.1	8.2	7.64	8.52	8.12	1	1	10.13	7.1	9.11	8.78	9.23	10.99	10.11	1.15	1
Solyc01g006970.2.1	ATP-dependent RNA helicase	11.95	15.3	16.14	14.83	14.55	11.58	8.58	11.46	12.9	11.13	0.76	0.640499	13.62	18.62	22.46	18.23	25.85	21.45	20.09	22.46	1.23	0.652982	11.31	13.96	14.66	13.31	16.71	15.02	14.11	15.28	1.15	1	27.08	19.5	25.36	23.98	24.4	31.39	27.89	1.16	1
Solyc01g006980.2.1	Malonyl CoA-acyl carrier prot	50.76	45.5	54.05	52.1	50.6	66.81	78.7	54.25	67.1	66.72	1.32	0.291359	38.86	36.64	39.78	38.43	42.45	42.13	41.76	42.11	1.1	0.983436	26.95	28.37	23.83	26.38	29.98	27.22	28.44	28.55	1.08	1	1.82	9.58	3.95	5.12	16.78	16.55	16.66	3.26	0.130708
Solyc01g006990.2.1	F-box family protein (AHRD V	18.3	18.64	13.92	17.71	17.14	21.01	17.01	25.46	19.79	20.82	1.21	0.702069	21.05	23.76	22.37	22.39	21.79	26.37	27.34	25.17	1.12	0.938793	22.18	26.88	19.24	22.77	28.71	20.89	26.76	25.45	1.12	1	40.57	30.08	30.36	33.67	25.35	26.49	25.92	0.77	1
Solyc01g007000.2.1	U-box domain-containing pro	0	0	0	0	0	0	0	0	0	0	1	NA	0	0	0	0	0.11	0	0	0.04	3.67	1	0	0	0	0	0.11	0	0	0.04	3.67	1	0	0	0	0	0	0	0	1	NA
Solyc01g007010.2.1	U-box domain-containing pro	0	0	0	0	0	0	0	0	0	0	1	NA	0	0	0	0	0	0	0	0	1	NA	0	0	0	0	0	0	0	0	1	NA	0.69	0	0.25	0.31	0	0	0	0.03	1
Solyc01g007020.2.1	U-box domain-containing pro	0	0	0	0	0	0	0	0.15	0	0.04	3.75	1	0	0	0.14	0.05	0	0	0	0	0.21	1	0	0	0.32	0.11	0	0	0	0	0.09	NA	1.15	0.47	0.25	0.62	0.3	0	0.15	0.24	1
Solyc01g007030.2.1	U-box domain-containing pro	0	0	0	0	0	0	0	0	0	0	1	NA	0	0	0	0	0.22	0	0	0.07	7.33	1	0	0	0.62	0.21	0.41	0	0	0.14	0.66	1	0.44	0.23	0.48	0.38	0	0	0	0.03	1
Solyc01g007040.2.1	U-box domain-containing pro	0	0	0	0	0	0	0	0	0	0	1	NA	0	0	0	0	0	0	0	0	1	NA	0	0	0.73	0.24	0.24	0	0	0.08	0.33	1	0.52	0.27	0	0.26	0	0	0	0.04	1
Solyc01g007050.1.1	U-box domain-containing pro	0	0	0	0	0	0	0	0	0	0	1	NA	0	0	0	0	0	0	0	0	1	NA	0	0	0	0	0	0	0	0	1	NA	0	0	0	0	0	0	0	1	NA
Solyc01g007060.2.1	At5g63850 (Fragment) (AHRD	5.09	5.33	7.94	9.1	6.87	5.82	6.85	5.78	6.01	6.12	0.89	1	5.71	7.41	6.7	6.61	5.29	6.12	6.47	5.96	0.9	1	11.24	8.78	9.8	9.94	7.76	7.6	8.07	7.81	0.79	1	6.17	3.59	3.22	4.33	4.93	4.46	4.7	1.09	1
Solyc01g007070.2.1	BEL1-like homeodomain prot	216.36	183.93	299.34	251.86	237.87	173.97	215.91	234.32	231.95	214.04	0.9	1	266.56	293.94	328.6	296.37	281.19	217.27	228.01	242.16	0.82	0.815134	184.91	196.17	129.1	170.06	189.43	119.9	155.47	154.93	0.91	1	171.81	213.33	162.23	182.46	162.91	152.73	157.82	0.86	1
Solyc01g007080.2.1	Aluminum-activated malate t	0	0	0	0	0	0	0	0	0	0	1	NA	0	0	0	0	0	0	0	0	1	NA	0	0	0	0	0	0	0	0	1	NA	0	0	0	0	0	0	0	1	NA
Solyc01g007090.1.1	Aluminum-activated malate t	0	0	0	0	0	0	0	0	0	0	1	NA	0	0	0	0	0	0	0	0	1	NA	0	0	0	0	0	0	0	0	1	NA	0	0	0	0	0	0	0	1	NA
Solyc01g007100.2.1	Digalactosyldiacylglycerol syn	13.84	14.66	16.33	8.65	13.37	17.18	11.26	19.59	17.43	16.37	1.22	0.713814	12.79	8.93	8.61	10.11	8.13	6.95	9.41	8.16	0.81	0.896625	6.04	2.77	3.12	3.98	2.23	1.35	2.47	2.02	0.51	1	0.48	0.68	0.39	0.52	0.7	0.74	0.72	1.39	1
Solyc01g007110.2.1	NC domain-containing protei	90.93	90.22	84.74	118.9	96.2	49.55	53.29	37.71	75.89	54.11	0.56	0.04237	118.94	160.97	114.24	131.38	100.35	93.24	91.68	95.09	0.72	0.447972	103.3	111.04	122.37	112.24	114.96	143.83	82.04	113.61	1.01	1	201.72	150.41	137.87	163.33	181.4	161.69	171.55	1.05	1
Solyc01g007120.2.1	Serine/threonine protein kina	17.57	20.9	19.03	12.84	17.59	17.13	13.65	18.28	17.79	16.71	0.95	1	22.51	16.65	16.91	18.69	17.98	23.94	25.58	22.5	1.2	0.745982	14.31	17.96	14.01	15.43	18.93	18.12	15.43	17.49	1.13	1	13.52	23.23	21.93	19.56	21.62	20.96	21.29	1.09	1
Solyc01g007130.2.1	Receptor like kinase, RLK	6.57	8.95	10.42	8.42	8.59	9.43	5.94	9.82	9.19	8.59	1	1	11.33	7.56	8.44	9.11	5.93	8.4	7.85	7.39	0.81	0.962977	1.71	3.29	1.97	2.32	2.52	2.07	3.19	2.59	1.12	1	0.1	0.35	0.21	0.22	0.88	1.2	1.04	4.73	0.477071
Solyc01g007140.2.1	Pre-rRNA-processing protein	3.07	3	2.47	1.8	2.58	2	2.25	3.1	3.23	2.65	1.02	1	3.41	1.69	2.28	2.46	1.92	2.87	3.47	2.75	1.12	1	2.24	2.2	1.93	2.12	1.33	1.31	2.29	1.64	0.77	1	0.96	1.09	1.96	1.34	1.71	1.97	1.84	1.38	1
Solyc01g007150.2.1	Coatomer subunit beta-1 (AH	41.18	42.46	47.93	50.98	45.64	35.82	48.96	34.93	40.08	39.95	0.88	1	45.41	39.06	44.93	43.13	35.83	32.83	41.53	36.73	0.85	0.952965	28.78	38.31	31.36	32.82	30.66	34.37	34.77	33.27	1.01	1	30.9	37.68	34.81	34.46	40.24	50.17	45.2	1.31	1
Solyc01g007160.2.1	N-acetyltransferase ESCO1 (A	0.37	0.82	0.87	0.78	0.71	0.2	0.87	0.51	1.59	0.79	1.12	1	1.71	0.32	0.47	0.83	0.26	0.48	0.58	0.44	0.53	1	0.15	0.75	0	0.3	0.12	0	0.4	0.17	0.58	1	0	0.27	0	0.09	0	0.16	0.08	0.89	1
Solyc01g007170.2.1	Protein unc-13 homolog C (AH	0	0	0.08	0	0.02	0.15	0.05	0	0	0.05	2.5	1	0	0	0.06	0.02	0	0	0	0	0.5	1	0	0	0	0	0	0	0.05	0.02	1.67	1	0	0	0	0	0	0	0	1	NA
Solyc01g007180.2.1	Unknown Protein (AHRD V1)	7.54	11.32	16.68	6	10.38	8.9	7.07	13.43	5.07	8.62	0.83	1	8.33	6.96	8.76	8.02	8.17	7.32	10	8.5	1.06	1	7.99	10.75	10.76	9.83	5.57	7.5	6.52	6.53	0.66	1	15.41	11.29	14.49	13.73	14.23	15.29	14.76	1.08	1
Solyc01g007190.2.1	Unknown Protein (AHRD V1)	0	0.96	0.95	0.76	0.67	1.5	0	0.74	0.97	0.8	1.2	1	0.75	1.17	2.05	1.32	3.17	2.79	2.33	2.76	2.09	0.568106	1.31	1.97	5.88	3.05	1.77	2.24	2.94	2.32	0.76	1	1.53	1.56	1.24	1.44	1.73	1.18	1.46	1.01	1
Solyc01g007200.2.1	Unknown Protein (AHRD V1)	0.59	0.78	1.73	0.83	0.98	0.98	1.85	0.27	0.42	0.88	0.9	1	1.36	3.31	0.99	1.89	1.83	1.06	0.92	1.27	0.67	1	0.47	1.91	0.58	0.99	1.35	0.44	0.21	0.67	0.68	1	0	0.21	0.45	0.22	0	0.26	0.13	0.59	1
Solyc01g007210.2.1	Unknown Protein (AHRD V1)	0	0	0	0	0	0	0	0	0	0	1	NA	0	0	0	0	0	0	0	0	1	NA	0	0	0	0	0	0	0	0	1	NA	0	0	0	0	0	0	0	1	NA
Solyc01g007220.2.1	NHL1 (Fragment) (AHRD V1 *	368.44	325.42	491.96	354.78	385.15	345.21	260.91	248.63	336.23	297.75	0.77	0.62535	402.64	645.7	655.51	567.95	892.68	836.38	591.81	773.62	1.36	0.461433	137.77	368.19	139	214.99	266.16	155.19	286.63	235.99	1.1	1	684.9	323.43	283.76	430.7	317.05	438.46	377.75	0.88	1
Solyc01g007230.2.1	Harpin-induced protein-like (	11.76	12	13.2	11.77	12.18	10.2	13.36	6.07	10.09	9.93	0.82	0.981333	17.88	17.7	10.42	15.33	12.53	11.44	11.27	11.75	0.77	0.906212	18.08	17.06	16.51	17.22	10.74	16.3	13.17	13.4	0.78	1	11.01	10.62	6.18	9.27	8.4	7.54	7.97	0.86	1
Solyc01g007240.2.1	NHL3 (Fragment) (AHRD V1 *	0	0	0	0	0	0	0	0	0	0	1	NA	0	0	0	0	0	0	0	0	1	NA	0	0	0	0	0	0	0	0	1	NA	0	0	0	0	0	0	0	1	NA
Solyc01g007250.2.1	U3 small nucleolar ribonuclec	23.56	25.76	21.97	16.5	21.95	16.96	19.49	24.35	20.14	20.23	0.92	1	27.97	21.07	15.54	21.53	16.03	15.57	24.51	18.7	0.87	1	30.09	16.64	16.26	21	14.59	18.2	18.64	17.14	0.82	1	19.78	15.48	9.66	14.97	20.95	16.53	18.74	1.25	1
Solyc01g007260.2.1	Os04g0416500 protein (Fragr	0	0.21	0.55	0.11	0.22	0.61	0.37	0.29	0.56	0.46	2.1	0.661671	0.43	0.2	0.33	0.32	0.54	0.08	0.06	0.23	0.71	1	0.31	0.51	0.08	0.3	0.1	0.24	0.23	0.19	0.63	1	0.11	0.23	0.12	0.15	0.14	0.14	0.14	0.91	1
Solyc01g007270.1.1	Cytokinin riboside 5'-m	0.93	0	0	0	0.23	0	0	1.71	0	0.43	1.84	1	0	0	0	0	0	0	0	0	1	NA	0	0.76	0	0.25	0	0	0	0	0.04	1	0	0	0	0	0	0	0	1	NA
Solyc01g007280.2.1	30S ribosomal protein S4 chlo	0.98	0.29	3.47	0.46	1.3	1.45	0.26	1.5	0.23	0.86	0.66	1	0.91	0.57	1.93	1.14	1.36	0.34	0.77	0.82	0.72	1	0.26	0.53	0.65	0.48	0.86	0	0.48	0.45	0.93	1	4.18	3.08	2.01	3.09	1.2	2.29	1.75	0.56	1
Solyc01g007290.1.1	NAD(P)H-quinone oxidoreduc	0	0.37	0.49	0	0.21	0	0.33	0.76	0.3	0.35	1.62	1	0	0.36	1.05	0.47	0	0.21	0	0.07	0.15	1	0	0.34	1.65	0.66	0.55	0.31	0.3	0.39	0.58	1	4.72	0.3	0	1.67	0.38	1.09	0.74	0.44	1
Solyc01g007300.1.1	NAD(P)H-quinone oxidoreduc	1.75	0.26	0.69	0.41	0.78	1.62	1.15	1.34	1.05	1.29	1.66	0.959699	0.27	0.51	1.48	0.75	0.61	0.45	0.69	0.58	0.77	1	0.47	0.71	0.58	0.59	0.38	0.44	0.43	0.42	0.71	1	5.81	1.91	0	2.57	0	1.02	0.51	0.2	1
Solyc01g007310.2.1	NAD(P)H-quinone oxidoreduc	0	0.97	1.29	0.77	0.76	0	0.43	0	0.39	0.21	0.27	0.983202	2.03	0.47	1.38	1.29	0	0.28	0.43	0.24	0.18	0.763865	0.44	0	0	0.15	0.72	0	1.19	0.64	4.34	1	3.1	2.38	0.84	2.11	0.5	0.96	0.73	0.35	1
Solyc01g007320.2.1	ATP synthase subunit beta ch	82.63	82.01	280.96	202.39	162	116.37	76.25	105.5	117.86	104	0.64	0.902661	59.25	76.24	208.63	114.71	81.09	58.4	70.29	69.93	0.61	0.927266	28.22	57.02	90.49	58.58	38.37	29.84	29	32.4	0.55	1	95.64	6.91	7.15	36.57	48.41	35.75	42.08	1.15	1
Solyc01g007330.2.1	Ribulose bisphosphate carbox	6.76	4.05	6.19	7.03	6.01	8.59	3.92	10.66	9.12	8.07	1.34	0.593504	1.8	3.6	3.73	3.04	5.55	4.14	2.71	4.13	1.36	0.84953	1.9	2.47	3.97	2.78	5.53	4.18	4.02	4.58	1.65	1	68.29	52.13	23.35	47.92	22.11	26.14	24.12	0.5	0.916983
Solyc01g007340.2.1	Acetyl-coenzyme A carboxyla	0.26	0	0.15	0	0.1	0.72	0.21	0.6	0	0.38	3.73	0.753905	0.12	0.11	0	0.08	0.18	0.2	0.51	0.3	3.87	0.890119	0.1	0	0.39	0.16	0.26	0.1	0.66	0.34	2.08	1	11.63	5.94	3.2	6.92	0.95	1.02	0.98	0.14	0.529136
Solyc01g007350.2.1	Photosystem I reaction cente	0	0.8	3.2	1.28	1.32	1	1.43	0.83	1.3	1.14	0.86	1	1.68	0	3.05	1.58	3.13	0.47	0.71	1.44	0.91	1	0.73	0	0.9	0.54	1.19	0	0	0.4	0.73	1	12.85	2.63	2.78	6.09	2.48	1.58	2.03	0.33	1
Solyc01g007360.2.1	Photosystem I assembly prote	0	0	0.42	0	0.1	0	0	0.66	0	0.17	1.57	1	0.66	0	0.3	0.32	0	0	0.84	0.28	0.87	1	0.29	0.29	0	0.19	0	0	0	0	0.05	1	2.54	0.52	1.1	1.39	0	0	0	0.01	0.796711
Solyc01g007370.2.1	Chloroplast envelope membra	0	0.77	1.69	0.81	0.82	0	0	0.79	0.21	0.25	0.31	0.779774	0.27	0	0.73	0.33	0.2	0.3	0.23	0.24	0.73	1	0.23	0.47	1.71	0.8	0.57	0.22	0.21	0.33	0.41	1	3.26	1.67	0.44	1.79	0.26	1.01	0.64	0.35	1
Solyc01g007380.1.1	Apocytochrome f (AHRD V1 *	0	0.55	0.97	0.29	0.45	0.69	0.32	0.57	0.44	0.51	1.12	1	0.38	0.36	0.87	0.54	0.29	0.11	0	0.13	0.25	0.810492	0.17	0.67	0.82	0.55	0.14	0.47	0.3	0.3	0.55	1	6.43	2.69	1.58	3.57	0.75	0.54	0.65	0.18	0.826874
Solyc01g007390.1.1	Unknown Protein (AHRD V1)	0	0	0	0	0	2.44	1.74	0	0	1.04	104.5	1	2.05	0	1.86	1.3	0	0	1.73	0.58	0.44	1	0	0	0	0	1.44	0	0	0.48	48	1	0	0	0	0	0	0	0	1	NA
Solyc01g007400.1.1	Unknown Protein (AHRD V1);	6.28	1.4	7.41	2.22	4.33	10.48	4.96	4.33	9.02	7.2	1.66	0.920872	8.77	10.92	6.63	8.77	8.72	7.32	4.94	6.99	0.8	1	17.77	7.68	17.15	14.2	7.22	4.76	4.58	5.52	0.39	1	8.93	12.55	33.82	18.43	24.44	20.66	22.55	1.22	1
Solyc01g007410.2.1	Cytochrome b559 subunit alp	14.84	23.49	33.08	30.33	25.43	20.17	9.11	21.23	24.87	18.85	0.74	0.98105	16.88	31.54	37.61	28.68	22.88	22.2	10.37	18.48	0.64	0.853144	19.99	37.63	29.47	29.03	21.67	28.75	21.63	24.02	0.83	1	37.52	33.54	43.11	38.06	42.27	49.17	45.72	1.2	1
Solyc01g007420.2.1	Cytochrome b6-f complex sub	0	0	0	0	0	1.13	0	1.87	0.73	0.93	93.25	0.904728	0.94	0	0	0.31	0	0.53	0	0.18	0.56	1	0	0	1.01	0.34	0	0	0.74	0.25	0.73	1	0	0	0	0	0	0	0	1	NA
Solyc01g007430.2.1	Cytochrome b6-f complex sub	1.4	2.5	0	1.99	1.47	4.68	1.11	5.16	3.02	3.49	2.37	0.935492	1.31	0	0	0.44	0	1.45	1.1	0.85	1.95	1	2.27	1.14	0	1.14	0	0	3.07	1.02	0.9	1	11.97	7.14	2.16	7.09	0	0	0	0	0.487801
Solyc01g007440.1.1	Photosystem I reaction cente	4.3	14.04	10.15	4.06	8.14	25.51	12.45	17.14	7.21	15.58	1.91	0.678841	6.67	3.74	1.21	3.87	11.94	8.17	7.89	9.33	2.41	0.628165	10.43	7.01	7.12	8.19	2.83	2.17	10.45	5.15	0.63	1	2.04	16.67	11.03	9.91	3.94	7.54	5.74	0.58	1
Solyc01g007450.1.1	50S ribosomal protein L33 ch	0.98	0	1.16	0	0.53	3.28	1.55	3.62	2.12	2.64	4.94	0.624333	0	1.71	3.33	1.68	2.73	1.53	0	1.42	0.85	1	1.59	3.21	1.95	2.25	0	0.75	1.44	0.73	0.32	1	9.8	3.58	3.03	5.47	0	5.18	2.59	0.47	1
Solyc01g007460.2.1	30S ribosomal protein S18 ch	0.65	0.58	0.76	0.91	0.72	1.44	0.51	3.57	2.79	2.08	2.87	0.710137	0	1.12	1.09	0.74	1.35	0	0.51	0.62	0.84	1	1.05	0.53	1.93	1.17	1.27	0.49	0	0.59	0.5	1	5.52	4.23	1.99	3.91	0	0	0	0	0.32208
Solyc01g007470.1.1	50S ribosomal protein L20 ch	1.53	0.46	1.21	0	0.8	1.14	2.02	1.41	1.47	1.51	1.89	0.957692	0	0.89	0.43	0.44	1.42	1.32	0.8	1.18	2.68	0.960515	1.24	0.83	1.02	1.03	1.34	0	1.86	1.07	1.04	1	9.45	5.2	2.36	5.67	2.34	2.24	2.29	0.4	1
Solyc01g007480.1.1	Ribosomal protein S12 (Fragn	0	0	0	0	0	0	0	0.75	0	0.19	18.75	1	0	0	0	0	0	0.42	0	0.14	14	1	0	0	0	0	0	0	0	0	1	1	0	0.59	1.25	0.61	0	0	0	0.02	1
Solyc01g007490.2.1	ATP-dependent Clp protease	13.39	10.78	25.19	17.13	16.62	18.42	16.35	27.25	8.47	17.62	1.06	1	9.19	9.42	19.92	12.84	11.49	13.53	8.77	11.26	0.88	1	6.69	9.35	15.82	10.62	4.82	6.28	6.97	6.02	0.57	1	37.16	13.89	7.84	19.63	8.75	10.62	9.68	0.49	1
Solyc01g007500.2.1	Photosystem II CP47 chloropl	2.74	4.35	0.84	1.35	2.32	3.57	2.63	4.05	4.96	3.8	1.64	0.5855	1.33	1.35	2.21	1.63	2.64	2.9	2.53	2.69	1.65	0.516408	0.77	2.33	2.01	1.7	3.29	2.26	2.6	2.72	1.59	1	2.2	2.16	2.75	2.37	1.53	2.09	1.81	0.76	1
Solyc01g007510.1.1	PsbN (AHRD V1 ***- Q7HIW7	0	0	0	0	0	0	0	2.02	0	0.51	50.5	1	2.05	0	0	0.68	0	0	0	0	0.01	1	0	0	0	0	0	0	0	0	1	NA	3.13	3.19	10.14	5.49	0	0	0	0	1
Solyc01g007520.2.1	Photosystem II reaction cente	0	0	1.05	1.26	0.58	0	0.7	1.64	0.64	0.74	1.29	1	0.83	0	0.75	0.53	1.85	0.92	0.7	1.16	2.2	1	1.44	0	0	0.48	1.76	1.35	0.65	1.25	2.61	1	1.27	1.94	1.37	1.53	0	0.78	0.39	0.26	1
Solyc01g007530.2.1	Cytochrome b6 (AHRD V1 ***	0.92	1.65	1.09	0.87	1.13	1.03	0.73	1.7	1.55	1.25	1.11	1	0.29	0.27	0.78	0.45	1.07	0.32	1.94	1.11	2.49	0.931682	1	0.5	1.22	0.91	1.42	0.93	0.67	1.01	1.11	1	8.77	6.05	9.96	8.26	1.98	2.97	2.47	0.3	0.41931
Solyc01g007540.2.1	Cytochrome b6-f complex sub	0.47	0.84	1.11	0	0.6	1.05	0	1.3	1.35	0.93	1.53	1	0.88	0.82	1.59	1.1	1.63	0.73	1.85	1.4	1.28	1	0.38	1.15	0	0.51	0.31	0.36	0.69	0.45	0.89	1	5.36	3.42	2.17	3.65	1.73	0	0.86	0.24	0.824749
Solyc01g007550.1.1	DNA-directed RNA polymeras	0	1.17	1.56	0	0.68	5.87	1.04	7.28	0.95	3.79	5.55	0.613943	0	0	0	0	0.92	2.05	0	0.99	99	1	0	1.08	0	0.36	0.87	1	0.96	0.94	2.62	1	5.63	0.96	0	2.2	1.21	0	0.6	0.28	1
Solyc01g007560.2.1	DNA-directed RNA polymeras	0.49	0.44	0	1.05	0.49	1.37	0.2	1.59	1.06	1.06	2.13	0.784107	0	0.86	0.83	0.56	0	0.13	0.78	0.3	0.54	1	0.2	0.6	0.49	0.43	0.49	0	0.18	0.22	0.52	1	4.22	2.33	2.66	3.07	0	0.43	0.21	0.07	0.155827
Solyc01g007570.2.1	30S ribosomal protein S11 ch	0.77	1.71	0.45	1.63	1.14	3.84	0.91	2.12	2.2	2.27	1.99	0.776968	0.71	2	1.94	1.55	0.27	0.99	1.81	1.02	0.66	1	2.48	0.63	1.52	1.54	1.01	1.16	0.84	1	0.65	1	11.45	7.52	6.49	8.49	0.7	2.35	1.52	0.18	0.146309
Solyc01g007580.2.1	30S ribosomal protein S8 (AH	0.98	1.74	1.15	0.69	1.14	4.89	2.7	1.8	2.1	2.87	2.52	0.62535	3.18	1.7	1.65	2.18	1.36	3.29	2.3	2.32	1.06	1	3.55	1.99	2.91	2.82	2.57	1.11	0.71	1.46	0.52	1	25.71	10.65	8.27	14.88	3.13	5.57	4.35	0.29	0.924156
Solyc01g007590.2.1	50S ribosomal protein L14 ch	2.68	0.95	3.8	0.76	2.05	3.58	2.12	2.96	1.93	2.65	1.29	1	2.99	2.8	2.27	2.69	2.6	3.05	1.26	2.3	0.86	1	1.73	3.06	3.73	2.84	2.11	0.81	1.56	1.49	0.53	1	30.5	10.13	9.9	16.84	3.93	5.64	4.79	0.28	0.986172
Solyc01g007600.2.1	50S ribosomal protein L16 ch	5.54	3.95	7.85	3.14	5.12	2.46	1.31	5.1	1.59	2.61	0.51	0.594601	3.09	3.86	4.68	3.88	2.31	2.01	2.61	2.31	0.6	0.943912	1.34	2.71	4.95	3	1.82	1.68	4.04	2.51	0.84	1	37.83	9.26	11.08	19.39	8.12	7.29	7.71	0.4	1
Solyc01g007610.2.1	30S ribosomal protein S3 chlo	3.18	2.99	5.51	5.55	4.31	8.73	3.25	6.19	5.37	5.89	1.37	0.71488	2.43	4.55	4.58	3.85	2.2	4.74	3.52	3.49	0.9	1	1.36	2.59	2.23	2.06	2.95	2.12	2.18	2.42	1.17	1	31.89	10.04	7.47	16.47	5.82	5.08	5.45	0.33	1
Solyc01g007620.2.1	30S ribosomal protein S19 ch	5.28	2.35	1.56	1.87	2.77	1.47	1.04	0	1.89	1.1	0.4	0.933869	0	4.59	2.23	2.27	1.83	0.68	3.11	1.87	0.82	1	1.07	3.23	1.31	1.87	0.87	1	1.92	1.26	0.68	1	60.03	6.71	2.03	22.92	4.83	2.31	3.57	0.16	1
Solyc01g007630.2.1	50S ribosomal protein L2 chlo	15.51	12.81	19.84	19.56	16.93	17.66	14.16	14.61	16.77	15.8	0.93	1	10.6	14.78	20.68	15.35	17.89	18	13.66	16.52	1.08	1	6.64	9.14	8.72	8.17	8.84	8.07	5.86	7.59	0.93	1	98.05	39.09	25.58	54.24	24.98	29	26.99	0.5	1
Solyc01g007640.2.1	Ycf2 (AHRD V1 ***- D2KLP5_	0	0.15	0.24	0.29	0.17	0.13	0.07	0.43	0.04	0.17	0.99	1	0.13	0.08	0.34	0.18	0.02	0.03	0.16	0.07	0.38	0.774176	0.07	0.12	0.17	0.12	0.13	0.07	0.04	0.08	0.67	1	1.28	0.74	0.45	0.82	0.24	0.23	0.23	0.29	0.682649
Solyc01g007650.2.1	Ycf15 (AHRD V1 ***- D2KLP6	0	0.83	2.19	1.31	1.08	0	0.73	2.56	1.33	1.16	1.07	1	0.86	0	0	0.29	0	0.48	0	0.16	0.56	1	0	0.76	0	0.25	0	0	0	0	0.04	1	6.61	3.37	0	3.33	0	0	0	0	0.893097
Solyc01g007660.2.1	NAD(P)H-quinone oxidoreduc	1.93	1.71	3.03	1.82	2.12	2.28	1.52	2.48	1.75	2.01	0.95	1	0.84	1.67	3.79	2.1	2.14	0.73	1.71	1.53	0.73	1	1.24	1.46	2.93	1.88	0.93	0.68	1.03	0.88	0.47	1	3.47	2.61	1.38	2.49	1.53	2.14	1.83	0.74	1
Solyc01g007670.2.1	30S ribosomal protein S7 chlo	6.34	3.76	3.49	4.79	4.59	15.51	7.68	24.5	3.64	12.83	2.79	0.452454	2.36	7.72	5.71	5.26	5.87	5.69	2.33	4.63	0.88	1	1.71	2.41	8.81	4.31	2.78	1.28	3.7	2.59	0.6	1	7.82	8.6	7.15	7.86	3.87	6.3	5.08	0.65	1
Solyc01g007680.1.1	Orf146 protein (AHRD V1 ***	0	0	0	0	0	0	0	0.46	0	0.12	11.5	1	0	0	0	0	0	0	0	0	1	NA	0.4	0	0	0.13	0	0.38	0	0.13	0.95	1	0	0.36	0	0.12	0	0	0	0.08	1
Solyc01g007690.2.1	Unknown Protein (AHRD V1)	0	0	2.36	0	0.59	0	0	0	0	0	0.02	1	0	0	0	0	2.77	0	0	0.92	92.33	1	0	1.63	0	0.54	1.31	3.03	1.46	1.93	3.56	1	11.37	0	3.07	4.81	0	0	0	0	1
Solyc01g007700.2.1	Conserved protein (AHRD V1	0	0	0	0	0	0	0	0	0	0	1	NA	0	0	0	0	0	0	0	0	1	NA	0	0	0	0	0	0	0	0	1	NA	0	0	0	0	0	0	0	1	NA
Solyc01g007710.2.1	Unknown Protein (AHRD V1)	0.8	0	0	0	0.2	0	0	0	0.58	0.14	0.72	1	0	0	0	0	0.56	0	0	0.19	18.67	1	0	0	0	0	0	0	0	0	1	1	12.58	1.17	2.47	5.41	0.74	0	0.37	0.07	1
Solyc01g007720.2.1	ORF42f (AHRD V1 *-*- Q3298	27.94	20.03	32.05	31.84	27.96	17.25	27.57	42.1	31.76	29.67	1.06	0.997255	6.5	7.42	19.01	10.98	19.92	20.09	12.2	17.4	1.59	0.690324	8.78	13.91	10.01	10.9	22.94	15.29	27.15	21.79	2	0.968845	1441.17	275.65	208.86	641.89	85.26	65.33	75.3	0.12	1
Solyc01g007730.2.1	ORF91 (AHRD V1 *--- C1BRN8	0	0	0	0	0	0	0	0	0	0	1	NA	0	0	0	0	0	0	0	0	1	NA	0	0	0	0	0	0	0	0	1	NA	0	0	0	0	0	0	0	1	NA
Solyc01g007740.2.1	Peroxiredoxin (AHRD V1 **--	98.22	117.87	76.67	86.35	94.78	142.83	116.41	141.91	111.51	128.16	1.35	0.3563	95.02	100.14	74.23	89.8	94.41	126.43	140.06	120.3	1.34	0.300117	76.37	88.06	82.59	82.34	80.69	102.06	83.77	88.84	1.08	1	72.1	118	112.8	100.97	153.91	151.3	152.61	1.51	0.868588
Solyc01g007750.2.1	Mitochondrial transcription t	7.26	10.52	15.42	16.43	12.41	23.06	14.41	20.41	13.55	17.86	1.44	0.388995	5.98	7.75	10.51	8.08	22.01	16.86	14.67	17.85	2.21	0.006311	4.69	7.78	4.94	5.8	6.27	4.4	6.8	5.82	1	1	13.57	6.48	3.83	7.96	4.94	3.64	4.29	0.54	1

Solyc01g007760.2.1	Transcription factor E2F (AHR	14.71	15.66	18	19.83	17.05	12.53	15.95	15.67	14.8	14.74	0.86	1	17.39	11.83	15.32	14.85	9.72	9.89	13.39	11	0.74	0.665429	21.76	19.38	22.1	21.08	20.13	19.58	19.83	19.85	0.94	1	23.45	23.56	28.32	25.11	25.54	21.57	23.55	0.94	1
Solyc01g007770.2.1	Genomic DNA chromosome 5	14.33	14.09	23.57	30.39	20.59	9.64	10.42	6.93	10.01	9.25	0.45	0.185181	20.34	24.58	24.84	23.25	16.21	11.91	7.41	11.84	0.51	0.085947	6.09	12.29	11.6	9.99	8.42	8.29	6.32	7.68	0.77	1	19.83	11.23	15.07	15.38	21.05	14.21	17.63	1.15	1
Solyc01g007780.2.1	Receptor expression-enhancii	0.41	1.09	0.97	1.16	0.91	0	0.65	1.13	1.47	0.81	0.9	1	0.76	1.42	0.69	0.96	0.85	0	0	0.28	0.3	0.88771	0.33	0.33	0.41	0.36	0.81	0.62	0.3	0.58	1.62	1	0	3.87	5.04	2.97	1.5	0	0.75	0.25	1
Solyc01g007790.2.1	UPF0554 protein C2orf43 hor	17.8	25.35	17.54	12.44	18.28	23.28	27.78	28.11	18.19	24.34	1.33	0.571147	16.36	24.03	16.8	19.06	15.54	16.16	19.59	17.1	0.9	1	19.46	12.97	16.21	16.21	13.9	18.41	14.19	15.5	0.96	1	5.66	14.75	17.71	12.71	17.45	13.22	15.34	1.21	1
Solyc01g007800.2.1	Plant-specific domain TIGR01	0	0	0	0	0	0	0	0	0	0	1	NA	0	0	0	0	0	0	0	0	1	NA	0	0	0	0	0	0	0	0	1	NA	0	0	0	0	0	0	0	1	NA
Solyc01g007810.1.1	Plant-specific domain TIGR01	0	0	0	0	0	0.49	0	0	0	0.12	12.25	1	0	0	0	0	0	0	0	0	1	NA	0	0	0	0	0	0	0	0	1	NA	0	0	0	0	0	0	0	1	NA
Solyc01g007820.1.1	Unknown Protein (AHRD V1)	2.64	3.91	3.11	1.24	2.73	4.89	2.08	2.43	1.26	2.67	0.98	1	3.27	6.12	2.23	3.87	0.61	1.82	2.07	1.5	0.39	0.817649	2.84	3.58	5.24	3.89	2.89	2	1.28	2.06	0.53	1	7.5	10.86	4.06	7.47	3.22	2.31	2.77	0.37	0.92381
Solyc01g007830.1.1	Unknown Protein (AHRD V1)	0	0	0.71	0	0.18	0	0	0.56	0	0.14	0.79	1	0	0	0.51	0.17	0	0.31	0	0.1	0.61	1	0	0.49	0	0.16	0	0	0	0	0.06	1	0	0	0	0	0	0	0	1	NA
Solyc01g007840.2.1	CCR4-NOT transcription comp	18.22	15.6	18.23	12.07	16.03	21.54	15.66	15.48	13.58	16.57	1.03	1	14.16	13.23	20.26	15.88	19.52	19.35	17.04	18.64	1.17	0.904652	16.03	18.61	14.42	16.35	16.97	20.98	18.99	18.98	1.16	1	10.17	9.38	11.35	10.3	15.84	13.96	14.9	1.45	1
Solyc01g007850.2.1	Pentatricopeptide repeat-con	2.46	2.59	1.91	2.38	2.33	2.81	2.45	2.74	3.16	2.79	1.19	0.880122	2.71	2.65	1.97	2.44	3.64	4.46	4.12	4.07	1.67	0.142369	3.35	2.69	2.89	2.98	3.1	2.31	2.41	2.61	0.88	1	3.04	4.18	4.58	3.93	3.14	3.46	3.3	0.84	1
Solyc01g007860.2.1	Ubiquitin-conjugating enzym	543.92	486.49	667.66	646.86	586.23	494.87	442.88	434.5	425.29	449.38	0.77	0.615588	521.81	513.39	543.94	526.38	442.93	419.65	377.02	413.2	0.78	0.735043	417.69	443.59	469.07	443.45	396.83	416	353.52	388.78	0.88	1	230.35	246.42	195.3	224.02	238.28	219.57	228.93	1.02	1
Solyc01g007870.2.1	Cyclin B-like protein (AHRD V	10.39	5.09	7.97	7.35	7.7	3.46	2.87	1.43	3.36	2.78	0.36	0.156272	15.46	8.58	3.95	9.33	4.68	2.15	4.9	3.91	0.42	0.647547	0.42	1.69	0.52	0.88	1.36	0	0	0.45	0.52	1	0	0	0.8	0.27	0	0	0	0.04	1
Solyc01g007880.2.1	MORN repeat protein (AHRD	6.16	8.33	7.56	7.04	7.27	7.78	6.66	6.67	5.51	6.65	0.92	1	5.81	7.85	8.12	7.26	9.36	7.73	8.22	8.44	1.16	0.93656	3.88	5.84	4.94	4.89	4.43	4.28	6.64	5.12	1.05	1	5.52	8.64	5.97	6.71	6.49	6.51	6.5	0.97	1
Solyc01g007890.1.1	Unknown Protein (AHRD V1)	1.22	0.77	1.44	0.49	0.98	0.97	1.37	1.12	0.75	1.05	1.07	1	0.97	0.61	0.59	0.72	0.97	0.72	0.14	0.61	0.84	1	0.14	0	0.17	0.1	0.34	0.13	0	0.16	1.52	1	1.48	1.64	0.8	1.31	0.16	0.61	0.39	0.29	0.901125
Solyc01g007900.2.1	Tobamovirus multiplication p	17.16	17.26	12.88	13.24	15.14	19.08	14.43	21.72	15.04	17.57	1.16	0.944697	23.01	17.44	16.38	18.94	14.69	19.73	18.57	17.66	0.93	1	33.31	21.43	25.44	26.73	22.99	27.7	32.16	27.62	1.03	1	32.64	43.71	49.35	41.9	30.6	40.65	35.62	0.85	1
Solyc01g007910.2.1	Succinyl-CoA ligase (AHRD V1	83.6	73.7	98.87	108.17	91.09	51.75	66.32	76.14	73.96	67.04	0.74	0.599894	61.73	76.46	73.12	70.44	53.04	44.72	49.82	49.19	0.7	0.322067	47.06	68.13	62.73	59.31	60.39	48.5	53.27	54.05	0.91	1	63.1	80.43	73.72	72.42	80.52	80.08	80.3	1.11	1
Solyc01g007920.2.1	Isochorismatase family protei	63.07	62.44	55.43	68.28	62.3	71.54	66.56	68.94	70.93	69.49	1.12	0.883084	77.83	114.14	88.88	93.62	166.95	119.3	107.99	131.41	1.4	0.449726	73.84	125.87	65.16	88.29	70.82	64.64	98.26	77.91	0.88	1	76.41	56.1	43.55	58.69	52.73	45.99	49.36	0.84	1
Solyc01g007930.2.1	Sel1 domain protein repeat-c	15.66	12.46	13.26	7.82	12.3	9.63	4.66	7.96	7.41	7.42	0.6	0.151338	7.2	9.77	9.65	8.87	11.5	9.92	9.27	10.23	1.15	0.978902	6.85	5.86	4.39	5.7	5.33	7.96	6.31	6.53	1.15	1	29.87	12.05	14.45	18.79	10.12	13.9	12.01	0.64	1
Solyc01g007940.2.1	Alanine aminotransferase 2 (	0	0.87	1.48	0.2	0.64	0	0	0	0.1	0.03	0.04	0.341654	0.13	0	0	0.04	0	0	0	0	0.23	1	0	0	0	0	0	0	0	0	1	NA	0	0	0.21	0.07	0	0.24	0.12	1.71	1
Solyc01g007950.2.1	Peroxidase 1 (AHRD V1 *-*- B	5.88	6.22	10.97	3.37	6.61	6.2	2.7	6.91	6.57	5.6	0.85	1	7.54	2.78	2.95	4.42	4.85	3.23	3.1	3.73	0.84	1	1.68	2.91	1.26	1.95	1.81	0.9	2.17	1.63	0.83	1	0	0.05	0	0.02	0	0.07	0.04	2.1	1
Solyc01g007960.2.1	RLK, Receptor like protein, pu	0	0	0.12	0.29	0.1	0	0.08	0.19	0	0.07	0.66	1	0	0	0.17	0.06	0.14	0.16	0.24	0.18	3.18	1	0	0	0	0	0.07	0	0.07	0.05	4.67	1	0	0.07	0	0.02	0.09	0	0.04	1.93	1
Solyc01g007970.1.1	RLK, Receptor like protein, pu	2.11	1.16	2.49	1.71	1.87	2.12	1.91	1.76	1.73	1.88	1.01	1	1.31	2.19	2.63	2.04	1.88	1.46	1.58	1.64	0.8	1	0.24	0.41	0.2	0.28	0.59	0.15	0.37	0.37	1.31	1	1.14	0.95	0.77	0.95	0.55	0.18	0.36	0.38	0.973622
Solyc01g007980.2.1	RLK, Receptor like protein, pu	44.29	37.4	56.39	42.5	45.14	43.04	38.47	61.32	42.69	46.38	1.03	1	43.21	62.64	60.87	55.57	44.97	33.03	41.38	39.79	0.72	0.332016	5.09	5.55	2.77	4.47	9.9	3.29	3.99	5.73	1.28	1	14.53	11.85	9.68	12.02	5.67	7.15	6.41	0.53	0.578392
Solyc01g007990.2.1	RLK, Receptor like protein, pu	5.99	7.99	8.04	13.14	8.79	7	5.46	7.59	6.89	6.74	0.77	0.894057	5.09	22.71	17.96	15.25	12.53	8.02	7.63	9.39	0.62	0.888081	2.34	4.12	3.69	3.38	7.6	4.54	4.36	5.5	1.63	1	14.39	29.47	46.52	30.13	12.85	20.1	16.48	0.55	1
Solyc01g008000.2.1	ADP-ribosylation factor (AHR	2155.68	1728.99	1622.98	1686.79	1798.61	1395.4	1375.73	1231.03	1765.71	1441.97	0.8	0.724374	2334.21	2217.43	1820.48	2124.04	2585.66	2591.91	2647.05	2608.21	1.23	0.542235	1430.18	1571.26	1162.78	1388.07	1945.69	1976.73	1718.3	1880.24	1.35	1	1282.35	1202.9	1379.19	1288.15	1699.27	2010.99	1855.13	1.44	0.819748
Solyc01g008010.2.1	Os09g0327550 protein (Fragr	14.78	19.83	15.67	13.63	15.98	28.09	24.52	38.6	21.37	28.15	1.76	0.039841	14.74	17.69	10.67	14.37	13.37	11.76	13.3	12.81	0.89	1	13.15	13.44	10.42	12.34	8.72	11.36	10.15	10.08	0.82	1	0.3	2.33	0.33	0.99	1.57	0.38	0.97	0.99	1
Solyc01g008020.2.1	Equilibrative nucleoside trans	3.96	5.5	3.55	3.36	4.09	5.82	3.63	4.08	5.01	4.63	1.13	1	1.18	1.65	2.68	1.84	3.96	1.72	1.99	2.56	1.39	0.983162	0.13	0.13	0.16	0.14	0	0	0.23	0.08	0.55	1	0.23	0.23	0	0.15	0.15	0.28	0.22	1.4	1
Solyc01g008030.2.1	Unknown Protein (AHRD V1)	0	0	0	0	0	1	0	0	0	0.25	25	1	0	0	0	0	1.25	0	0	0.42	41.67	1	0	0	0	0	0	0	0	0	1	NA	0	0	0	0	0	0	0	1	NA
Solyc01g008040.1.1	S-locus F-box-like protein a (F	0	0	0	0	0	0	0	0	0	0	1	NA	0	0	0	0	0.12	0.09	0	0.07	7	1	0	0	0	0	0.34	0	0	0.11	11.33	1	0	0	0	0	0.16	0	0.08	8	1
Solyc01g008050.2.1	Leaf senescence protein-like (	0	0	0	0	0	0	0	0	0.11	0.03	2.75	1	0	0	0	0	0	0	0	0	1	NA	0	0	0	0	0.1	0	0	0.03	3.33	1	0	0	0	0	0	0.14	0.07	7	1
Solyc01g008060.2.1	Phosphoglycerate mutase (AH	63.58	65.28	55.81	50.34	58.75	63.68	57.05	64.25	64.03	62.25	1.06	1	59.14	47.16	48.57	51.62	46.96	42.81	46.05	45.27	0.88	1	41.55	34.48	32.53	36.19	33.22	37.99	34.32	35.18	0.97	1	31.47	35.62	28.03	31.71	28.8	26.26	27.53	0.87	1
Solyc01g008070.2.1	Alpha/beta superfamily hydro	49.21	45.02	56.44	43.32	48.5	52.5	43.42	47.63	48.89	48.11	0.99	1	50.31	70.86	69.88	63.68	103.45	73.99	80.46	85.97	1.35	0.347236	99.21	103.79	120.53	107.84	128.51	157.16	126.01	137.23	1.27	1	199.51	188.45	125.81	171.26	176.01	133.05	154.53	0.9	1
Solyc01g008080.2.1	Ribosomal protein S27 (AHRD	4956.17	3781.99	7622.17	7809.57	6042.48	5116.38	4122.92	4066.06	4640.96	4486.58	0.74	0.817178	5041.81	4635.24	7022.1	5566.38	4469.9	4635.22	4928.83	4677.98	0.84	0.907488	2413.14	2903.53	3000.14	2772.27	2767.19	2523.64	2360.05	2550.29	0.92	1	1256.02	974.27	1171.88	1134.06	1787.42	1952.69	1870.06	1.65	0.465623
Solyc01g008090.2.1	Ribosomal protein S27 (AHRD	1402.83	1297.11	2756.33	2685.78	2035.51	1396.68	1217.06	1172.69	1382.76	1292.3	0.63	0.619017	1430.44	1285.37	2222.43	1646.08	1252.77	1010.2	1322.94	1195.3	0.73	0.676342	859.52	962.69	1269	1030.4	864.28	855.77	821.68	847.24	0.82	1	567.1	364.6	384.8	438.83	783.69	827.08	805.38	1.84	0.254842
Solyc01g008100.2.1	Transferase transferring glyco	37.83	33.13	35.35	52.22	39.63	45.41	55.19	32	28.24	40.21	1.01	1	37.72	31.83	36.04	35.2	26.24	31.65	31.39	29.76	0.85	1	30.07	32.06	24.93	29.02	26.44	19.73	18.52	21.56	0.74	1	6.88	12.09	12.1	10.36	11.63	18.04	14.84	1.43	1
Solyc01g008110.2.1	Cytochrome P450	138.57	127.41	172.89	202.31	160.3	112.7	130.21	114.85	163.44	130.3	0.81	0.943718	117.07	106.82	103.22	109.04	89.45	90.22	99.23	92.97	0.85	0.971733	172.78	153.89	190.14	172.27	212.61	255.03	185.89	217.84	1.26	1	251.6	311.25	296.86	286.57	338.83	327.5	333.16	1.16	1
Solyc01g008120.2.1	Histone acetyltransferase (AH	11.68	9.98	14.93	13.76	12.59	7.82	12.98	9.48	11.64	10.48	0.83	0.996253	14.17	12.97	17.65	14.93	15.88	13.55	14.06	14.5	0.97	1	12.5	13.14	12.06	12.57	12.44	11.96	10.87	11.76	0.94	1	18.6	12.34	13.75	14.9	11.69	15.41	13.55	0.91	1
Solyc01g008130.2.1	DEP domain-containing prote	14.85	14.65	15.21	17.59	15.57	10.37	7.45	11.54	8.02	9.34	0.6	0.1071	16.28	22.42	18.48	19.06	21.56	19.19	18.73	19.83	1.04	1	4.37	6.57	5.03	5.32	3.33	2.88	5.12	3.78	0.71	1	0.98	1.17	0.89	1.01	0.53	1.72	1.12	1.11	1
Solyc01g008140.2.1	Receptor kinase (AHRD V1 **	4.16	4.19	7.56	5.88	5.45	3.03	2.04	3.2	3.96	3.06	0.56	0.227783	3.42	4.27	6.56	4.75	4.92	5.29	4.28	4.83	1.02	1	7.16	6.78	8.86	7.6	6.13	5.11	5.46	5.57	0.73	1	8.72	7.38	6.71	7.6	6.36	6.63	6.5	0.85	1
Solyc01g008150.1.1	Unknown Protein (AHRD V1)	0	0	0	0	0	1.47	0	0	0	0.37	36.75	1	1.23	0	0	0.41	0.92	0.68	0	0.53	1.3	1	0	0	0	0	0	0	0	0	1	NA	0	1.92	2.03	1.32	0	0	0	0.01	1
Solyc01g008160.2.1	Tetratricopeptide repeat-con	30.83	22.68	33.7	36.05	30.82	33.35	43	34.2	42.79	38.34	1.24	0.428784	32.31	24.3	29.34	28.65	32.36	36.71	30.69	33.25	1.16	0.826278	52.3	58.95	77.31	62.85	62.65	49.38	59.91	57.31	0.91	1	22.5	27.32	18.33	22.72	30.1	24.15	27.12	1.19	1
Solyc01g008170.2.1	Zinc finger CCCH domain-con	18.48	17.77	24.55	22.44	20.81	15.97	17.39	15.36	17.55	16.57	0.8	0.840135	21.35	25.71	25.14	24.07	25.31	21.74	21.56	22.87	0.95	1	16.43	23.95	21.86	20.75	21.76	16.27	19.38	19.14	0.92	1	69.52	48.46	69.45	62.48	30.07	43.85	36.96	0.59	0.738613
Solyc01g008180.2.1	DNA-binding protein (Fragme	8.58	9.09	8.19	15.52	10.34	5.89	8.56	6.26	9.15	7.46	0.72	0.851655	11.03	11.84	11.93	11.6	14.09	14.3	13.98	14.12	1.22	0.77615	3.16	3.49	3.76	3.47	5.88	3.73	4.6	4.74	1.37	1	23.49	16.31	21.34	20.38	15.7	23.45	19.57	0.96	1
Solyc01g008190.2.1	Os05g0304100 protein (Fragr	6.96	6.73	11.07	8.56	8.33	5.05	5.97	4.73	5.87	5.41	0.65	0.678344	6.76	7.63	9.71	8.03	7.77	5.17	5.23	6.06	0.75	0.944042	7.58	12.33	12.02	10.64	8.95	9.86	9.04	9.28	0.87	1	17.64	9.67	9.77	12.36	9.14	9.82	9.48	0.77	1
Solyc01g008200.2.1	Os05g0304100 protein (Fragr	13.32	11.85	16.42	17.12	14.68	20.86	8.6	18.36	16.51	16.08	1.1	1	7.88	12.63	19.43	13.31	16.37	14.1	11.89	14.12	1.06	1	13.69	18.74	25.23	19.22	18.29	20.18	21.61	20.03	1.04	1	32.7	28.13	29.78	30.2	16.07	13.8	14.94	0.49	0.69201
Solyc01g008210.2.1	Unknown Protein (AHRD V1)	9.91	5.48	5.24	14.51	8.79	4.18	8.64	2.83	7.36	5.75	0.65	0.951656	15.9	7.13	16.46	13.16	5.93	5.66	3.76	5.12	0.39	0.173782	1.66	4.18	5.09	3.64	8.31	4.66	1.49	4.82	1.32	1	5.35	1.24	0.53	2.37	0.63	3	1.81	0.76	1
Solyc01g008220.2.1	Arabinogalactan protein (AHF	2.72	3.63	2.81	5.29	3.61	1.13	3.49	3.13	1.71	2.37	0.65	0.925999	5.38	4.14	5.46	4.99	1.42	0.7	2.14	1.42	0.28	0.054329	1.1	0.28	1.01	0.8	0.89	1.03	0.5	0.81	1.01	1	0.48	0.49	0	0.32	1.25	0.6	0.93	2.86	1
Solyc01g008230.2.1	TCP family transcription facto	150.15	164.87	212.33	185.51	178.22	149.3	103.33	163.77	136.59	138.25	0.78	0.626712	165.44	219.07	248.29	210.93	217.98	200.57	195.14	204.56	0.97	1	40.08	65.58	66.79	57.48	70.38	50.2	63.84	61.47	1.07	1	86.29	104.07	85.62	91.99	116.19	145.76	130.97	1.42	0.975227
Solyc01g008240.2.1	Solute carrier family 2, facilit	0	0	0.31	0	0.08	0	0	0	0	0	0.13	1	0	0	0	0	0	0	0	0	1	NA	0	0	0	0	0	0	0	0	1	NA	0	0	0	0	0	0	0	1	NA
Solyc01g008250.2.1	Thioredoxin X (AHRD V1 ***-	48.67	54.86	30.62	37.73	42.97	98.98	69.73	100.1	89.81	89.66	2.09	0.000787	45.6	45.13	31.97	40.9	53.26	50.77	64.59	56.21	1.37	0.376583	14.56	17.33	10.73	14.21	17.52	20.49	21.02	19.68	1.39	1	2.56	11.26	6.65	6.82	17.82	12.64	15.23	2.23	0.773191
Solyc01g008260.2.1	E2F-associated phosphoprote	31.59	32.38	17.92	22.06	25.99	27.11	29.67	28.31	35.31	30.1	1.16	0.916716	33.1	35.8	36.81	35.24	58.79	50.3	54.04	54.38	1.54	0.148375	25.52	48.22	25.8	33.18	26.79	29.09	39.33	31.74	0.96	1	19.33	29.33	18.44	22.37	32.57	32.6	32.59	1.46	1
Solyc01g008270.2.1	Prefoldin subunit 5 (AHRD V1	168.05	182.49	177.75	196.72	181.25	156.57	163.64	189.76	148.81	164.69	0.91	1	175.39	181.34	176.2	177.64	179.62	148.63	171.3	166.52	0.94	1	113.8	130.76	99.55	114.7	112.45	110.14	108.49	110.36	0.96	1	89.36	113.31	89.79	97.49	133.02	155.55	144.29	1.48	0.938102
Solyc01g008280.2.1	Serine/threonine-protein pho	28.05	34.06	40.04	41.39	35.89	28.49	27.2	34.55	31.7	30.48	0.85	0.990806	43.81	36.12	38.9	39.61	48.28	36.4	39.33	41.34	1.04	1	20.98	30.62	18.05	23.22	26.84	24.8	25.49	25.71	1.11	1	23.88	27.42	21.3	24.2	30.75	40.54	35.64	1.47	0.953653
Solyc01g008290.2.1	Dihydroorotate dehydrogena	5.33	4.62	4.93	4.07	4.74	2.56	3.87	3.05	3.41	3.22	0.68	0.666312	5.49	5.38	5.23	5.37	2.9	4.7	5.66	4.42	0.82	1	1.75	4.23	2.29	2.76	1.99	2.29	2.41	2.23	0.81	1	2.87	3.45	5.09	3.8	5.8	6.95	6.38	1.68	0.86823
Solyc01g008300.1.1	Hydroxycinnamoyl CoA shikin	4.8	4.7	3.11	3.39	4	4	4.17	4.63	2.58	3.85	0.96	1	5.58	6.05	3.44	5.02	3.49	5.59	4.71	4.6	0.92	1	6.59	5.47	3.1	5.05	2.99	4	3.85	3.61	0.72	1	0.34	1.74	0.74	0.94	0.66	0.21	0.43	0.46	1
Solyc01g008310.2.1	Phenylalanyl-tRNA synthetas	48.13	58	62.86	52.88	55.47	55.28	46.13	59.29	49.74	52.61	0.95	1	54.35	49	57.07	53.47	52.97	47.18	57.86	52.67	0.98	1	51.73	42.02	49.82	47.86	38.08	44.72	42.83	41.88	0.88	1	48.28	46.34	42.55	45.72	51.49	55.44	53.47	1.17	1
Solyc01g008320.2.1	DDHD domain containing 1 (	15.64	17.65	19.04	14.58	16.73	18.25	17.79	19.41	17.71	18.29	1.09	0.954522	17.19	24.35	28.64	23.39	26.23	25.91	26.31	26.15	1.12	0.956426	39	42	45.58	42.19	44.51	41	41.44	42.32	1	1	48.52	55.09	64.06	55.89	40.48	39.64	40.06	0.72	0.987573
Solyc01g008330.2.1	Acetyl-CoA carboxylase biotir	77.76	68.01	68.85	68.81	70.86	67.6	86.71	53.54	92.87	75.18	1.06	0.933538	51.99	42.93	44	46.31	32.55	38.8	35.24	35.53	0.77	0.725355	40.49	42.85	41.22	41.52	35.99	44.23	42.7	40.97	0.99	1	10.31	25.07	17.76	17.71	55.22	38.99	47.11	2.66	0.147765
Solyc01g008340.2.1	50S ribosomal protein L19 (A	26.55	30.88	24.19	29	27.66	28.05	35.28	27.79	28.68	29.95	1.08	0.980705	30.07	22.39	29.75	27.4	22.6	23.95	27.47	24.67	0.9	1	28.24	32.34	34.7	31.76	31.25	27.29	25.66	28.07	0.88	1	16.44	34.17	30.72	27.11	29.83	33.42	31.62	1.17	1
Solyc01g008350.2.1	DNAJ heat shock N-terminal d	76	70.35	67.79	81.28	73.85	73.33	83.28	109.01	71.15	84.19	1.14	0.804781	78.05	94.87	69.59	80.84	101.21	75.4	80.75	85.79	1.06	1	99.47	94.63	68.2	87.43	91.47	91.71	100.58	94.59	1.08	1	82.99	106.87	82.28	90.71	90.17	85.03	87.6	0.97	1
Solyc01g008360.2.1	Pre-mRNA branch site p14-lik	143.11	134.95	127.42	160.29	141.44	149.03	160.45	222.57	133.68	166.43	1.18	0.703053	146.48	175.28	132.11	151.29	183.39	136.91	158.04	159.45	1.05	1	188.26	176.01	134.13	166.13	172.31	175.81	191.91	180.01	1.08	1	155.07	202.48	169.35	175.63	170.94	159.53	165.24	0.94	1
Solyc01g008370.2.1	26S proteasome regulatory su	125.58	118.37	122.38	111.62	119.49	112.47	101.65	105.51	101.12	105.19	0.88	0.982055	124.51	121.89	125.06	123.82	118.73	103.24	124.69	115.55	0.93	1	123.56	108.73	120.44	117.58	114.97	132.32	118.41	121.9	1.04	1	120.64	104	103.4	109.35	123.89	133.17	128.53	1.18	1
Solyc01g008380.1.1	Pentatricopeptide repeat-con	3.23	2.87	2.18	2.61	2.72	2.56	2.19	5.94	4.64	3.83	1.41	0.925051	4.29	1.2	2.34	2.61	6.08	5.73	5.08	5.63	2.16	0.408543	0.37	1.5	0.46	0.78	2.42	0.35	1.34	1.37	1.76	1	1.97	3.02	3.55	2.85	2.53	2.83	2.68	0.94	1
Solyc01g008390.1.1	LRR receptor-like serine/thre	0	0	0	0	0	0	0	0	0	0	1	NA	0	0	0	0	0	0	0	0	1	NA	0	0	0	0	0	0	0	0	1	NA	0	0	0	0	0	0	0	1	NA
Solyc01g008400.2.1	Unknown Protein (AHRD V1)	1.03	0	1.22	0	0.56	1.15	0	3.79	2.22	1.79	3.18	0.916716	0	0.9	3.48	1.46	1.43	2.13	4.05	2.54	1.74	1	0.83	0	1.02	0.62	0	2.34	0	0.78	1.26	1	4.4	0	0	1.47	0	0	0	0.01	1
Solyc01g008410.1.1	LRR receptor-like serine/thre	0	0	0	0	0	0	0	0	0	0	1	NA	0	0	0	0	0	0	0	0	1	NA	0	0	0	0	0	0	0	0	1	1	0	0	0	0	0	0	0	1	NA
Solyc01g008420.2.1	Mate efflux family protein (A	4.42	4.37	3.62	3.82	4.06	10.92	5.82	6.44	3.44	6.66	1.64	0.622222	5.71	4.49	3.84	4.68	8.69	5.53	4.54	6.25	1.34	0.81196	0.3	0.4	0.12	0.27	0.89	0.56	0.18	0.54	1.99	1	0	1.07	0.19	0.42	0.34	0.86	0.6	1.43	1
Solyc01g008430.1.1	Unknown Protein (AHRD V1)	0	0	0.41	0	0.1	0	0.14	0	0	0.04	0.34	1	0	0	0	0	0	0	0	0	1	NA	0	0	0	0	0	0	0	0	1	NA	0	0	0	0	0	0	0	1	NA
Solyc01g008440.2.1	Calcium-dependent protein k	9.88	10.56	10.35	8.91	9.93	11.12	7.51	11.02	11.35	10.25	1.03	1	7.93	6.01	7.62	7.19	7.8	11.51	9.03	9.45	1.31	0.561965	13.87	11.88	12.02	12.59	4.54	4.49	8.37	5.8	0.46	0.599247	0.35	0.72	0.57	0.55	1.47	1.08	1.27	2.33	1
Solyc01g008450.1.1	Unknown Protein (AHRD V1)	0	0.21	0	0	0.05	0	0	0	0	0	0.19	1	0	0	0	0	0	0	0	0	1	NA	0	0	0	0	0	0	0.17	0.06	5.67	1	0.67	0	0	0.22	0	0.21	0.1	0.47	1
Solyc01g008460.2.1	2Fe-2S ferredoxin (AHRD V1 *	19.66	24.28	8.23	12.56	16.18	24.33	18.03	25.66	20.04	22.02	1.36	0.678841	19.77	21.23	12.32	17.77	20.02	20.53	27.42	22.66	1.27	0.749201	21.27	17.83	12.28	17.13	16.46	20.67	21.26	19.46	1.14	1	11.27	21.88	22.44	18.53	22.07	22.25	22.16	1.2	1
Solyc01g008470.2.1	Non-selective cation channel-	7.15	6.69	7.57	6.74	7.04	6.93	5.5	7.25	6.05	6.43	0.91	1	9.2	7.01	6.97	7.73	5.34	5.5	4.61	5.15	0.67	0.674862	6.66	8.81	6	7.16	4.33	5.14	4.01	4.49	0.63	0.960051	1.56	3.19	3.38	2.71	3.02	1.29	2.16	0.8	1
Solyc01g008480.2.1	Pentatricopeptide repeat-con	4.46	4.66	6.05	3.94	4.78	5.08	6.33	5.53	5.6	5.63	1.18	0.854325	5.7	5.23	5.93	5.62	5.33	5.14	4.47	4.98	0.89	1	5.67	5.18	5.97	5.61	4.47	3.29	3.25	3.67	0.65	1	8.4	7.28	4.28	6.65	5.3	5.67	5.48	0.82	1
Solyc01g008490.2.1	Nuclear transcription factor Y	15.39	17.45	16.41	13.42	15.67	8.9	10.32	12.79	15.89	11.97	0.76	0.780921	14.7	16.86	15.13	15.56	23.1	21.28	18.88	21.09	1.35	0.473913	21.46	21.3	16.53	19.76	19.52	24.12	25.65	23.1	1.17	1	23.07	17.76	14.58	18.47	29.71	22.73	26.22	1.42	0.989607
Solyc01g008500.2.1	Receptor serine/threonine kir	0.31	0	0	0.22	0.13	0	0.37	0.57	0.45	0.35	2.62	0.843786	0.44	0.27	0	0.24	0	0.08	0.12	0.07	0.28	1	0.13	0.13	0.31	0.19	0.1	0	0.34	0.15	0.77	1	0	0	0	0	0.14	0	0.07	7	1
Solyc01g008510.2.1	Photosystem II 5 kDa protein,	0	2.02	2.01	0	1.01	3.16	1.35	2.09	3.27	2.47	2.45	0.749359	1.06	3.46	0.96	1.83	12.62	3.83	5.36	7.27	3.98	0.421824	13.78	61.17	25.4	33.45	74.72	60.78	128.89	88.13	2.63	1	6.47	4.96	4.37	5.27	5.21	2.49	3.85	0.73	1
Solyc01g008520.2.1	DNA repair protein XRCC2 (A	1.56	2.42	2.3	3.03	2.33	3.03	1.23	3.04	2.79	2.52	1.08	1	2.35	2.37	4.27	3	6.07	2.82	3.52	4.14	1.38	0.950467	1.1	1.74	0.97	1.27	1.41	1.03	1.28	1.24	0.98	1	3.6	1.84	2.09	2.51	1.07	1.71	1.39	0.55	1
Solyc01g008530.2.1	Cinnamoyl CoA reductase-like	3.21	3.75	3.08	2.55	3.15	2.67	3.48	6.27	4.32	4.19	1.33	0.821423	1.31	2.09	2.2	1.87	1.67	1.35	1.73	1.58	0.85	1	7.13	12.09	4.78	8	0.92	0.76	2.34	1.34	0.17	0.512731	0.29	1.02	0.31	0.54	0.55	0	0.28	0.51	1
Solyc01g008540.2.1	Cinnamoyl CoA reductase-like	0.61	0.72	1.2	0.29	0.71	0	0.16	0.19	0.15	0.12	0.18	0.440159	0	0	0.17	0.06	0	0.11	0	0.04	0.65	1	7.24	12.61	4.85	8.23	0.67	0.62	2.97	1.42	0.17	0.891453	0	0.59	0.31	0.3	0	0	0	0.03	1
Solyc01g008550.2.1	Cinnamoyl CoA reductase-like	52.82	55.6	35.89	38.37	45.67	67.62	67.01	54.8	46.92	59.09	1.29	0.539721	13.19	6.61	11.63	10.48	5.56	6.49	8.24	6.76	0.65	0.604902	3.82	3.35	2.45	3.21	2.16	2.8	1.8	2.25	0.7	1	2.63	2.39	3.16	2.73	2.07	1.98	2.02	0.74	1
Solyc01g008560.2.1	NAD kinase 1 (AHRD V1 ****	13.76	14.57	12.73	29.64	17.68	12.27	14.59	12.66	9.17	12.17	0.69	0.844985	14.19	21.56	26.18	20.64	14.19	14.77	16.76	15.24	0.74	0.663727	10.72	10.61	21.17	14.17	14.01	15.32	6.42	11.92	0.84	1	15.16	29.45	29.56	24.72	22.7	20.77	21.73	0.88	1
Solyc01g008570.1.1	Receptor-like protein kinase (	0	0	0	0	0	0	0.2	0	0	0.05	5	1	0.24	0	0	0.08	0	0	0	0	0.12	1	0	0	0	0	0	0	0	0	1	NA	0	0	0	0	0	0	0	1	NA
Solyc01g008580.1.1	Zinc transporter protein (AHR	0	0	0	0	0	0	0	0	0	0	1	NA	0	0	0	0	0	0	0	0	1	NA	0	0	0	0	0	0	0	0	1	NA	0	0	0	0	0	0	0	1	NA
Solyc01g008590.1.1	Zinc transporter protein (AHR	0	0	0	0	0	0	0	0	0	0	1	NA	0	0	0	0	0	0	0	0	1	NA	0	0	0	0	0	0	0	0	1	NA	0	0	0	0	0	0	0	1	NA
Solyc01g008600.2.1	Zinc finger CCCH domain-con	10.3	10.7	6.08	10.29	9.34	13.81	17.07	11.05	10.73	13.17	1.41	0.304244	7.52	5.62	6.49	6.54	6.45	8.58	9.53	8.19	1.25	0.741271	6.53	5.76	3.61	5.3	4.84	3.98	5.67	4.83	0.91	1	6.03	4.92	6.68	5.88	4.81	4.87	4.84	0.82	1
Solyc01g008610.2.1	Beta-1 3-glucanase (AHRD V1	0	0	0	0	0	0	0	0	0	0	1	NA	0	0	0	0	0	0	0	0	1	NA	0	0	0	0	0	0	0	0	1	NA	0	0	0	0	0	0	0	1	NA
Solyc01g008620.2.1	Beta-1 3-glucanase (AHRD V1	0	0	0	0	0	0	0	0.18	0	0.04	4.5	1	0	0	0	0	0	0	0	0	1	NA	0	0	0	0	0	0	0	0	1	NA	0	0	0	0	0	0	0	1	NA
Solyc01g008640.2.1	Cytochrome P450	0	0	0	0	0	0	0	0	0	0	1	NA	0	0	0	0	0	0	0	0	1	NA	0	0	0	0	0	0	0	0	1	NA	0	0	0	0	0	0	0	1	NA
Solyc01g008650.2.1	Cytochrome P450	0	0	0	0	0	0	0	0	0	0	1	NA	0	0	0	0	0	0	0	0	1	NA	0	0	0	0	0	0	0	0	1	NA	0	0	0	0	0	0	0	1	NA
Solyc01g008660.2.1	F-box family protein (AHRD V	6.5	7.83	6.99	7.03	7.09	5.31	3.93	6.51	6.45	5.55	0.78	0.90221	8.18	6.65	6.94	7.26	7.29	9.8	8.42	8.5	1.17	0.943066	1.85	2.34	2.66	2.28	3.14	2.61	4.6	3.45	1.51	1	6.25	4.03	5	5.09	5.6	8.22	6.91	1.36	1
Solyc01g008670.2.1	Cytochrome P450	0	0	0	0	0	0	0.11	0	0	0.03	2.75	1	0	0	0	0	0	0.07	0.1	0.06	5.67	1	0	0	0	0	0	0	0	0	1	NA	0	0.1	0.41	0.17	0	0	0	0.06	1
Solyc01g008680.2.1	S2 self-incompatibility locus-l	0	0	0	0.48	0.12	0	0	0	0	0	0.08	1	0	0.89	0	0.3	0	0.35	1.07	0.47	1.6	1	1.1	1.11	1.7	1.3	0.9	4.15	3.49	2.85	2.18	1	8.26	12.41	13.67	11.45	15.33	12.89	14.11	1.23	1
Solyc01g008690.2.1	GDP-mannose transporter (A	0	0	0	0.27	0.07	0	0	0.35	0	0.09	1.3	1	0.18	0	0	0.06	0.13	0	0	0.04	0.72	1	0	0	0	0	0.13	0	0	0.04	4.33	1	0	0	0	0	0	0	0	1	NA
Solyc01g008700.2.1	cDNA clone 001-102-C05 full	11.65	9	10.38	7.47	9.62	18.82	14.76	20.02	13.1	16.67	1.73	0.099675	10.23	11.47	13.74	11.81	16.93	13.78	20.91	17.21	1.46	0.428444	185.12	185.82	116.56	162.5	143.16	154	184.6	160.59	0.99	1	10.32	21.4	20.63	17.45	16.51	10.99	13.75	0.79	1
Solyc01g008710.2.1	Mannan endo-1 4-beta-mann	0	0.44	0.19	1.87	0.62	4.4	0.26	4.4	0.36	2.35	3.77	0.623277	0	0	0.28	0.09	0.34	0.51	0.91	0.59	6.29	0.516491	1113.54	1171.51	1004.82	1096.62	811.51	1102.66	1287.78	1067.32	0.97	1	2.58	36.66	63.91	34.38	39.56	12.58	26.07	0.76	1
Solyc01g008720.1.1	Mannan endo-1 4-beta-mann	0	0	0.19	0.23	0.11	0.18	0	0.3	0	0.12	1.14	1	0	0	0	0	0	0	0	0	1	NA	26.91	20.59	33.07	26.86	21.99	28.86	31.81	27.55	1.03	1	0	0.24	1.01	0.42	0.75	0.58	0.67	1.6	1
Solyc01g008730.2.1	BZIP transcription factor (AHF	22.12	18.44	25.86	19.91	21.58	19.23	18.9	25.52	18.92	20.64	0.96	1	19.46	31.65	28.71	26.61	24.13	26.76	25.13	25.34	0.95	1	30.06	28.03	29.35	29.15	30.39	29.42	31.66	30.49	1.05	1	52.05	41.93	42.99	45.66	28.72	25.75	27.23	0.6	0.728205
Solyc01g008740.1.1	Calcium-dependent protein k	0	0	0	0	0	0	0	0	0	0	1	NA	0	0	0	0	0	0	0	0	1	NA	0	0	0	0	0	0	0	0	1	NA	0	0	0	0	0	0	0	1	NA
Solyc01g008750.1.1	cDNA clone J013074B07 full i	0	0.07	0.09	0	0.04	0	0	0	0	0	0.25	1	0	0.07	0	0.02	0.05	0.04	0.06	0.05	2.14	1	0	0.06	0	0.02	0.1	0	0.11	0.07	3.5	1	0	0.23	0.24	0.16	0.14	0.07	0.11	0.67	1
Solyc01g008760.2.1	Genomic DNA chromosome 5	12.82	11.41	10.33	10.61	11.29	8.34	8.89	8.62	7.63	8.37	0.74	0.778948	9.6	10.06	8.71	9.46	9.11	8.58	7.86	8.52	0.9	1	8.34	10.7	7.14	8.73	4.72	7.35	9.11	7.06	0.81	1	9.34	4.54	3.85	5.91	5.72	6.58	6.15	1.04	1
Solyc01g008770.2.1	Rho GTPase activating protei	24.55	25.23	23.04	24.3	24.28	19.18	24.42	20.61	24.17	22.1	0.91	1	21.56	17.18	19.71	19.48	13.65	14.48	17.08	15.07	0.77	0.720252	11.91	12.08	7.77	10.59	9.74	10.02	8.92	9.56	0.9	1	7.02	7.95	8.41	7.79	7.58	6.66	7.12	0.91	1
Solyc01g008780.2.1	Phospholipase A22 (AHRD V1	64.05	62.61	83.97	48.4	64.76	91.28	54.89	60.66	102.98	77.45	1.2	0.81595	72.82	64.96	64.97	67.58	108.79	85.18	74.43	89.47	1.32	0.360941	79.39	96.14	77.17	84.23	84.88	98.34	122.68	101.97	1.21	1	39.7	52.09	34.94	42.24	94.47	83.04	88.75	2.1	0.270273
Solyc01g008790.2.1	Phosphoesterase family prote	42.26	56.75	65.67	119.77	71.11	50.58	58.59	39.13	58.15	51.61	0.73	0.972223	33.7	27.52	41.42	34.21	34.83	28.59	17.01	26.81	0.78	0.965119	10.83	13.68	9.5	11.34	3.39	2.23	6.98	4.2	0.37	0.147309	0.84	0.53	0.45	0.61	1.75	1.29	1.52	2.51	0.944946
Solyc01g008800.1.1	Tir-nbs-lrr, resistance protein	1.78	1.71	1.05	1.82	1.59	1.21	1.13	1.14	1.21	1.17	0.74	0.799769	0.83	2.36	1.25	1.48	1.06	1.69	1.71	1.49	1	1	0.52	1.09	0.49	0.7	0.65	0.82	0.5	0.66	0.94	1	0.84	1.47	1.37	1.23	0.81	1.17	0.99	0.81	1
Solyc01g008810.2.1	Zinc finger family protein (AH	29.26	30.95	29.63	36.31	31.54	34.06	19.83	25.89	32.1	27.97	0.89	1	27.47	19.92	22.61	23.33	23.55	25.15	27.55	25.42	1.09	1	19.62	20.47	17.53	19.21	25.02	28.03	24.14	25.73	1.34	1	41.99	25.66	28.44	32.03	34.11	30.25	32.18	1	1
Solyc01g008820.2.1	Signal peptide peptidase fam	37.95	39.14	19.74	18.72	28.89	42.4	38.1	44.37	30.04	38.73	1.34	0.654151	34.94	29.6	19.4	27.98	16.74	25.69	28.75	23.73	0.85	1	60.69	42.5	43.65	48.95	38.48	44.69	42.97	42.05	0.86	1	12.45	26.71	22.14	20.43	28.15	24.06	26.1	1.28	1

Solyc01g008830.1.1	L-ascorbate oxidase (AHRD V	0.12	0.31	0.14	0.65	0.31	0.13	0.09	0.21	0.17	0.15	0.49	1	0.43	0.7	0.1	0.41	0.4	0.36	0.54	0.43	1.06	1	1.49	0.94	1.14	1.19	0.53	0.79	0.84	0.72	0.61	1	0.49	1	0.89	0.79	0.53	0.5	0.52	0.65	1
Solyc01g008840.2.1	COP9 signalosome complex s	48.43	55.51	38.21	34.36	44.13	44	47.7	52.52	52.31	49.13	1.11	0.947105	57.46	57.87	47.11	54.15	60.52	66.06	67.16	64.58	1.19	0.70488	61.05	69.4	55.21	61.89	84.31	66.48	76.04	75.61	1.22	1	72.91	109.22	81.62	87.92	68.9	69.02	68.96	0.78	1
Solyc01g008850.2.1	CBL-interacting protein kinas	69.43	85.6	104.43	110.11	92.39	56.79	63.95	57.62	67.54	61.48	0.67	0.262268	103.3	132.59	140.13	125.34	67.54	98.12	94.24	86.63	0.69	0.288906	154.68	218.95	323.09	232.24	206.72	192.94	131.96	177.21	0.76	1	194.22	245.03	254.43	231.23	246.93	222.28	234.61	1.01	1
Solyc01g008860.1.1	ATP binding / serine-threonin	0	0	0	0	0	0	0	0	0	0	1	NA	0	0	0	0	0	0	0	0	1	NA	0	0	0	0	0	0	0	0	1	NA	0	0	0	0	0	0	0	1	NA
Solyc01g008870.1.1	ATP binding / serine-threonin	0	0	0	0	0	0	0	0	0	0	1	NA	0	0	0	0	0	0	0	0	1	NA	0	0	0	0	0	0	0	0	1	NA	0	0	0	0	0	0	0	1	NA
Solyc01g008880.1.1	Ethylene responsive transcrip	0	0	0	0	0	0	0.26	0	0	0.07	6.5	1	0	0	0	0	0	0.17	0	0.06	5.67	1	0	0.54	0	0.18	0	0	0.48	0.16	0.89	1	0	0.72	0	0.24	0	0.29	0.14	0.6	1
Solyc01g008890.2.1	Ethylene responsive transcrip	17.14	16.8	19.71	15.96	17.4	21.23	11.82	18.76	19.63	17.86	1.03	1	16.35	16.79	16.68	16.61	15.05	14.94	10.91	13.63	0.82	1	9.29	7.78	8.62	8.56	11.55	7.4	9.49	9.48	1.11	1	18.51	20.96	20.36	19.94	9.14	10.66	9.9	0.5	0.473634
Solyc01g008900.2.1	Genomic DNA chromosome 3	5.06	5.96	4.74	4.63	5.1	4.97	5.41	6.16	5.35	5.47	1.07	1	4.29	4.4	5.79	4.83	5.58	5.4	5.5	5.49	1.14	1	2.53	4.49	3.25	3.42	3.72	4.4	4.88	4.33	1.27	1	3.81	4.87	3.43	4.04	4.36	5.22	4.79	1.19	1
Solyc01g008910.2.1	Scarecrow transcription facto	1.38	1.35	0.82	1.37	1.23	0.46	0.55	0.64	0.99	0.66	0.54	0.732414	1.67	1.56	2.34	1.86	0.77	1.58	1.3	1.22	0.66	0.930556	2.35	5.07	3.71	3.71	7.27	5.87	3.93	5.69	1.53	1	16.91	7.33	10.42	11.55	6.2	6.55	6.38	0.55	1
Solyc01g008920.2.1	NAD dependent epimerase/d	11.7	10.41	9.52	7.42	9.76	21.08	16.09	19.48	24.19	20.21	2.07	0.004815	10.88	11.57	10.56	11	27.15	22.56	24.73	24.81	2.26	0.000927	1.96	4.27	2.2	2.81	3.58	2.6	2.5	2.89	1.03	1	6.02	7.33	3.41	5.59	11.45	8.84	10.14	1.82	0.901157
Solyc01g008930.2.1	Phycoerythrobilin ferredoxin	11.92	13.01	9.53	10.61	11.27	21.38	20.04	21.75	15.19	19.59	1.74	0.061108	12.52	10.37	11.37	11.42	12.67	16.43	17.38	15.49	1.36	0.515567	11.34	13.48	7.45	10.76	8.84	12.25	10.93	10.67	0.99	1	1.64	7.54	5.32	4.83	10.92	10.96	10.94	2.26	0.624572
Solyc01g008940.2.1	Anther protein (AHRD V1 ***	2.27	0.67	1.79	2.15	1.72	0	0	0.7	0	0.17	0.1	0.533277	0	0	0	0	0	0	0	0	1	NA	0	0	0	0	0	0	0	0	1	NA	0	0	0	0	0	0	0	1	NA
Solyc01g008950.2.1	Calmodulin 5/6/7/8-like prot	429.69	412.95	394.87	403.12	410.16	365.19	353.12	498.59	370.06	396.74	0.97	1	389.87	394.17	382.24	388.76	344.7	347.95	416.14	369.6	0.95	1	501.72	469.48	378.49	449.9	409.69	453.35	459.59	440.88	0.98	1	235.12	393.54	318.54	315.73	306.78	374.43	340.61	1.08	1
Solyc01g008960.2.1	Argonaute 4-like protein (AH	153.47	158.27	207.67	172.79	173.05	125.71	121.39	125.05	133.92	126.52	0.73	0.441162	147.01	150.43	211.19	169.54	164.52	136.33	137.3	146.05	0.86	0.968769	67	94.64	69.66	77.1	83.82	68.35	85.37	79.18	1.03	1	114.62	70.03	88.85	91.17	101.34	128.78	115.06	1.26	0.994665
Solyc01g008970.2.1	Heterogeneous nuclear ribon	288.73	244.98	321.86	245.29	275.22	186.73	184.98	182.77	249	200.87	0.73	0.448154	234.88	244.3	320.14	266.44	221.01	237.29	191.84	216.71	0.81	0.859116	153.68	151.29	167.61	157.53	258.65	161.94	148.18	189.59	1.2	1	292.34	294.23	350.35	312.31	233.39	266.05	249.72	0.8	1
Solyc01g008980.2.1	BZIP transcription factor (AH	6.73	12.38	16.94	12.06	12.03	11.97	6.73	12.79	14.5	11.5	0.96	1	10.85	19.9	16.68	15.81	18.36	21.61	17.63	19.2	1.21	0.902821	17.77	18.28	12.47	16.17	18.87	15.31	18.97	17.72	1.1	1	24.89	34.88	33.13	30.97	18.9	18.49	18.7	0.6	0.828552
Solyc01g008990.2.1	THO complex subunit 7 homo	26.71	31.38	24.26	28.33	27.67	24.35	28.26	18.66	22.82	23.52	0.85	0.963653	26.58	26.7	28.88	27.39	25.19	27.52	20.99	24.57	0.9	1	30.21	30.25	28.1	29.52	36.67	24.7	32.7	31.36	1.06	1	43.29	45.39	44.36	44.35	30.32	30.21	30.27	0.68	0.945151
Solyc01g009000.2.1	RING finger protein 6 (AHRD	51.08	44.54	72.46	69.62	59.42	39.77	35.1	29.28	41.72	36.47	0.61	0.157555	45.21	45.12	57.99	49.44	51.78	40.26	39.33	43.79	0.89	1	32.58	40.28	40.44	37.77	28.67	32.6	34.06	31.78	0.84	1	138.79	43.86	51.84	78.16	50.74	46.96	48.85	0.62	1
Solyc01g009010.2.1	4-diphosphocytidyl-2-C-meth	22.21	24.45	22.51	17.92	21.77	33.65	25.46	27.98	23.62	27.68	1.27	0.625191	20.51	19.45	19.73	19.9	21.79	27.17	27.54	25.5	1.28	0.56117	84.67	55.23	71.2	70.37	42.15	69.08	60.3	57.18	0.81	1	9.36	32.38	21	20.91	23.79	17.02	20.41	0.98	1
Solyc01g009020.2.1	Cysteine proteinase inhibitor	624.99	572.72	406.91	496.87	525.37	363.44	469.2	511.85	618.62	490.78	0.93	1	622.26	583.53	531.3	579.03	525.79	476.41	460.24	487.48	0.84	0.956854	432	396.11	284.91	371.01	323.52	306.59	315.43	315.18	0.85	1	59.24	74.39	67.63	67.09	84.76	112.64	98.7	1.47	0.954323
Solyc01g009030.2.1	Nuclear control of ATPase pro	14.28	16.85	18.15	18.79	17.02	14.89	18.35	18.22	12.71	16.04	0.94	1	17.5	17.13	19.07	17.9	15.05	14.44	17.66	15.72	0.88	1	17.97	24.62	23.51	22.03	20.29	19.46	18.71	19.49	0.88	1	16.21	25.81	20.6	20.87	20.97	24.26	22.62	1.08	1
Solyc01g009040.2.1	Cyclin B1 (AHRD V1 **** Q9L	0.61	0.54	0	0.21	0.34	0.17	0.24	0	0.33	0.18	0.54	1	0.42	0	0.13	0.18	0.52	0.08	0.36	0.32	1.75	1	0.12	0	0	0.04	0	0	0	0	0.25	1	0	0.22	0.23	0.15	0	0.13	0.07	0.43	1
Solyc01g009050.2.1	Molybdenum cofactor biosyn	24.19	21.09	18.33	23.22	21.71	20.22	24.06	18.44	22.38	21.27	0.98	1	18.38	25.98	27.9	24.09	28.71	27.69	26.66	27.69	1.15	0.882225	15.73	21.79	18.74	18.75	16.99	20.19	17.34	18.17	0.97	1	38.72	36.35	37.04	37.37	52.38	49.83	51.11	1.37	0.976223
Solyc01g009060.2.1	BTB/POZ domain containing p	20.47	21.4	35.27	28.96	26.52	19.15	19.5	21.21	16.33	19.05	0.72	0.538103	28.11	25.99	31.02	28.37	24.46	24.99	23.75	24.4	0.86	1	27.83	27.14	31.29	28.75	28.39	20.32	21.55	23.42	0.81	1	36.73	26.32	26.24	29.76	26.33	27.5	26.91	0.9	1
Solyc01g009070.2.1	MYB transcription factor (AH	10.3	11.46	17.5	11.79	12.76	13.49	11.23	17.02	10.92	13.16	1.03	1	11.06	10.87	13.15	11.69	9.44	10.22	12.05	10.57	0.9	1	4.66	5.4	4.02	4.69	3.95	2.88	4.11	3.65	0.78	1	5.06	5.17	7.35	5.86	2.92	5.59	4.25	0.73	1
Solyc01g009080.2.1	FHA domain containing prote	41.92	40.78	36.42	18.85	34.49	84.53	64.65	58.86	75.13	70.79	2.05	0.001535	53.2	46.05	31.29	43.51	56.12	58.22	55.16	56.5	1.3	0.475047	8.66	6.89	5.81	7.12	11.96	14.53	12.55	13.01	1.83	0.772591	0	0.47	1.5	0.66	2.97	2.28	2.62	4	0.77715
Solyc01g009090.2.1	Serrate RNA effector molecul	27.74	23.83	45.51	39.49	34.14	18.31	19.19	18.87	20.23	19.15	0.56	0.155736	25.56	33.31	39.13	32.67	31.83	24.4	22.01	26.08	0.8	0.832635	26.4	27.99	39.69	31.36	28.82	23.7	24.33	25.62	0.82	1	72.51	58.6	71.11	67.41	55.55	58.77	57.16	0.85	1
Solyc01g009100.2.1	Ribosomal protein L30 (AHRD	248.12	230.18	333.37	309.68	280.34	240.11	191.28	188.41	217.95	209.44	0.75	0.568086	225.37	180.67	198.72	201.59	108.92	118.49	111.49	112.97	0.56	0.026703	185.84	181.73	235.85	201.14	153.41	147.35	168.9	156.55	0.78	1	63.09	66.57	52.97	60.88	143.76	100.33	122.04	2	0.343226
Solyc01g009110.2.1	Histone deacetylase family pr	12.4	9.71	7.61	8.42	9.54	13.23	11.55	13.68	11.75	12.55	1.32	0.682085	13.38	14.23	11.52	13.04	16.34	14.77	17.93	16.35	1.25	0.810624	7.81	7.68	8.86	8.12	8.8	8.46	11.93	9.73	1.2	1	32.44	25.04	21.74	26.41	27.24	31.53	29.38	1.11	1
Solyc01g009120.2.1	Histone deacetylase family pr	10.62	7.45	7.21	6.37	7.91	8.59	8.38	12.13	7.86	9.24	1.17	0.992201	10.18	9.23	5.98	8.46	15.4	12.33	17.45	15.06	1.78	0.168998	5.98	8.92	4.79	6.56	7.19	11.95	7.27	8.8	1.34	1	16.93	16.13	13.36	15.47	18.56	25.39	21.98	1.42	1
Solyc01g009130.1.1	Unknown Protein (AHRD V1)	0	0	0	0	0	0	0	0	0	0	1	NA	0	0	0	0	0	0	0	0	1	NA	0	0	0	0	0	0	0	0	1	NA	0	0	0	0	0	0	0	1	NA
Solyc01g009140.1.1	Dicer-like protein (AHRD V1 *	0	0	0	0	0	0	0	0	0	0	1	NA	0	0	0	0	0	0	0.46	0.15	15.33	1	0	0	0	0	0	0	0	0	1	NA	0	0	0	0	0	0	0	1	NA
Solyc01g009150.2.1	Glycosyl hydrolase (Fragment	33.27	33.22	34.14	32.74	33.34	26.05	31.52	24.21	31.57	28.34	0.85	0.994469	40.37	33.96	21.02	31.78	33.72	24.42	34.79	30.98	0.97	1	22.91	26.17	24.7	24.59	23.38	19.96	21.72	21.69	0.88	1	13.99	17.86	16.02	15.96	23.04	20.3	21.67	1.36	1
Solyc01g009160.2.1	Harpin-induced protein-like (	0.25	0.89	0	2.82	0.99	1.66	2.16	1.14	0.54	1.38	1.39	0.988924	1.16	1.3	4.63	2.36	0.35	1.42	1.17	0.98	0.41	0.869314	0.6	1.42	5.19	2.4	1.8	1.32	0.36	1.16	0.48	1	40	18.99	47.47	35.49	22.79	41.47	32.13	0.91	1
Solyc01g009170.2.1	Ethylene insensitive 3 class tr	42.48	43.15	78.97	71.31	58.98	20.15	21.85	18.94	28.49	22.36	0.38	0.016283	88.8	61.26	79.73	76.6	118.22	85.87	86.58	96.89	1.26	0.595088	68.12	71.76	81.77	73.88	71.24	56.91	66.52	64.89	0.88	1	19.06	36.62	42.56	32.75	27.3	46.18	36.74	1.12	1
Solyc01g009180.2.1	5-methyltetrahydropteroyltri	10.65	10.77	14.18	9.7	11.32	10.4	10.25	9.34	12.97	10.74	0.95	1	12.38	9.81	10.63	10.94	15.49	12.78	14.26	14.18	1.3	0.538389	14.53	14.66	18.89	16.03	14.78	19.74	14.1	16.21	1.01	1	14.79	21.67	19.35	18.6	26.73	23.97	25.35	1.36	1
Solyc01g009190.1.1	Double-stranded RNA binding	0	0	0	0	0	0	0	0	0	0	1	NA	0	0	0	0	0	0	0	0	1	NA	0	0	0	0	0	0	0	0	1	NA	0	0	0.23	0.08	0	0.13	0.07	0.85	1
Solyc01g009200.2.1	Restricted tev movement 2 (A	2.27	5.8	2.67	0.8	2.89	5.35	6.04	5.21	3.05	4.91	1.7	0.644958	1.58	1.97	2.39	1.98	5.89	0.73	1.11	2.58	1.3	1	0.23	1.38	0.56	0.72	0.74	1.07	2.68	1.5	2.07	1	0	1.03	0.44	0.49	0.52	0	0.26	0.53	1
Solyc01g009210.1.1	Unknown Protein (AHRD V1)	0	0	0	0	0	0	0	0	0	0	1	NA	0	0	0	0	0	0	0	0	1	NA	0	0	0	0	0	0	0	0	1	NA	0	0	0	0	0	0	0	1	NA
Solyc01g009220.2.1	Restricted tev movement 2 (A	0.31	2.74	0.73	0.44	1.06	0.34	1.46	1.98	1.77	1.39	1.32	1	0.57	0.54	0.26	0.46	0.43	0	0.97	0.47	1.02	1	0	0.25	0	0.08	0.61	0	1.12	0.58	6.92	1	0	0	0	0	0	0	0	1	NA
Solyc01g009230.2.1	Xanthine dehydrogenase/oxid	3.83	4.4	5.43	5.07	4.68	3.23	4.13	3.74	4.24	3.83	0.82	0.966427	4.01	3.87	4.34	4.07	5.44	4.71	3.69	4.61	1.13	0.950755	1.25	1.18	0.63	1.02	1.34	0.81	1.06	1.07	1.05	1	0.69	1.34	0.97	1	0.93	0.76	0.84	0.84	1
Solyc01g009240.2.1	Beta-D-glucosidase (AHRD V1	0.1	0	0.12	0	0.06	0	0.08	0	0	0.02	0.36	1	0	0	0	0	0	0.05	0	0.02	1.67	1	0	0	0	0	0	0	0	0	1	NA	0	0	0	0	0	0	0	1	NA
Solyc01g009250.2.1	Unknown Protein (AHRD V1)	0.42	1.5	1.98	1.19	1.27	3.27	1.33	3.09	2.11	2.45	1.93	0.778711	1.56	0.73	1.06	1.12	1.46	3.7	1.65	2.27	2.03	0.778175	1.36	1.37	0.42	1.05	1.1	0.64	0.92	0.89	0.84	1	3.58	3.05	1.29	2.64	1.15	0.74	0.94	0.36	1
Solyc01g009260.1.1	60S ribosomal protein L5, mit	0	0	0	0	0	0	0	0	0	0	1	NA	0	0	0	0	0	0	0	0	1	NA	0	0	0	0	0	0	0	0	1	NA	0	0.1	0	0.03	0	0	0	0.3	1
Solyc01g009270.1.1	Unknown Protein (AHRD V1)	0	0	0	0	0	0	0	0	0	0	1	NA	0.13	0	0	0.04	0	0	0	0	0.23	1	0	0	0	0	0	0.11	0	0.04	3.67	NA	0.4	0	0	0.13	0	0	0	0.07	1
Solyc01g009280.2.1	Serine/threonine-protein pho	16.38	15.94	17.35	16.92	16.65	11.07	14.51	8.91	14.35	12.21	0.73	0.570342	16.72	16.85	26.03	19.87	20.4	17.75	16.65	18.27	0.92	1	12.8	16.61	14.93	14.78	15.15	14.54	16.12	15.27	1.03	1	21.57	16.94	22.61	20.37	17.74	23.56	20.65	1.01	1
Solyc01g009290.2.1	Polyribonucleotide 5'-h	12.29	14.41	14.86	14	13.89	12.5	11.25	7.44	11.09	10.57	0.76	0.715469	14.09	14.47	12.54	13.7	14.14	10.25	11.55	11.98	0.87	1	12.6	12.34	12.8	12.58	13.89	13.64	10.6	12.71	1.01	1	20.46	15.9	17.06	17.81	17.02	15.64	16.33	0.92	1
Solyc01g009300.2.1	Serine/threonine kinase (AHR	22.21	22.94	49.6	27.49	30.56	17.93	18.68	15.92	18.37	17.73	0.58	0.465262	38.61	9.19	12.51	20.1	8.76	10.13	11.58	10.16	0.51	0.928531	7	10.61	5.31	7.64	5.21	4.16	6.31	5.23	0.68	1	3.48	2.21	2.64	2.78	2.78	3.65	3.21	1.16	1
Solyc01g009310.2.1	Sterol reductase (AHRD V1 **	238.56	273.05	239.58	160.03	227.81	184.76	103.93	227.29	188.9	176.22	0.77	0.564896	201.02	211.99	154.99	189.33	128.85	110.64	138.13	125.87	0.66	0.150829	49.75	59.2	43.96	50.97	55.89	46.32	49.18	50.46	0.99	1	117.52	131.3	107.74	118.85	79.27	101.99	90.63	0.76	1
Solyc01g009320.1.1	U-box domain-containing pro	0	0	0	0	0	0	0	0	0	0	1	NA	0	0	0	0	0	0	0	0	1	NA	0	0	0	0	0	0	0	0	1	NA	0	0	0	0	0	0	0	1	NA
Solyc01g009330.1.1	U-box domain-containing pro	0	0	0	0	0	0	0	0	0	0	1	NA	0	0	0	0	0	0	0	0	1	NA	0	0	0	0	0	0	0	0	1	NA	0	0	0	0	0	0	0	1	NA
Solyc01g009340.2.1	Calmodulin-binding protein fa	0.33	1.45	0.58	0.46	0.7	1.27	0.39	0.9	0.47	0.76	1.07	1	1.22	0.28	0.41	0.64	0.68	0.25	0.38	0.44	0.69	1	0	0	0.32	0.11	0.21	0	0.12	0.11	1.03	1	0	0.83	0.75	0.53	0	0	0	0.02	0.811923
Solyc01g009350.2.1	CCR4-NOT transcription comp	0	0	0	0	0	0	0	0	0	0	1	NA	0	0	0	0	0	0	0	0	1	NA	0	0	0	0	0	0	0	0	1	NA	0	0	0	0	0	0	0	1	NA
Solyc01g009360.1.1	CCR4-NOT transcription comp	0	0	0	0	0	0	0	0	0	0	1	NA	0	0	0	0	0	0	0	0	1	NA	0	0	0	0	0	0	0	0	1	NA	0	0	0	0	0	0	0	1	NA
Solyc01g009370.1.1	Cytochrome P450	0	0	0	0	0	0	0	0	0	0	1	NA	0	0	0	0	0	0	0	0	1	NA	0	0	0	0	0	0	0	0	1	1	0	0	0	0	0	0	0	1	NA
Solyc01g009380.2.1	Nicalin (AHRD V1 ***- B4FZF	5.13	6.02	4.54	5.61	5.32	6.87	6.44	4.39	5.1	5.7	1.07	1	7.59	5.37	7.38	6.78	4.45	6.7	7.14	6.1	0.9	1	6.4	6.55	7.29	6.75	4.98	4.24	6.37	5.2	0.77	1	5.97	5.33	6.27	5.86	6.83	7.26	7.04	1.2	1
Solyc01g009390.2.1	MRNA decapping enzyme (AH	10.19	9.23	11.39	8.85	9.92	12.12	10.44	10.85	13.99	11.85	1.2	0.789364	12.47	10.41	11.78	11.55	13.27	10.92	15.17	13.12	1.14	1	13.29	15.3	14.37	14.32	11.74	11.65	12.12	11.84	0.83	1	18.56	9.09	9.62	12.42	13.26	13.95	13.61	1.1	1
Solyc01g009400.2.1	Peroxidase 57 (AHRD V1 ***-	0	0	0	0	0	0	0	0	0	0	1	NA	0	0	0	0	0	0	0	0	1	NA	0	0	0	0	0	0	0	0	1	NA	0	0	0	0	0	0	0	1	NA
Solyc01g009410.2.1	Peroxidase 57 (AHRD V1 ****	0	0	0	0	0	0	0	0	0	0	1	NA	0	0	0	0	0	0	0	0	1	NA	0	0	0	0	0	0	0	0	1	NA	0	0.29	0.31	0.2	0	0	0	0.05	1
Solyc01g009420.2.1	Bifunctional polymyxin resist	516.01	426.93	633.31	604.02	545.07	353.21	414.91	235.42	350.14	338.42	0.62	0.100066	364.7	446.63	518.04	443.12	401.64	302.48	329.82	344.65	0.78	0.610638	240.6	332.13	284.94	285.89	281.64	280.5	272.17	278.1	0.97	1	545.82	384.93	404.48	445.08	480.78	517.3	499.04	1.12	1
Solyc01g009430.2.1	Os02g0448600 protein (Frag	242.82	224.71	828.85	289.72	396.53	204.25	185.43	229.6	159.44	194.68	0.49	0.701696	351.8	76.56	114.65	181	157.39	99.07	114.55	123.67	0.68	1	250.87	183.51	140.91	191.76	184.25	175.92	147.49	169.22	0.88	1	420.86	259.64	235.29	305.26	286.78	197.15	241.96	0.79	1
Solyc01g009440.1.1	Ethylene-responsive transcrip	0	0	0	0	0	0	0	0	0	0	1	NA	0	0	0	0	0	0	0	0	1	NA	0	0	0	0	0	0	0	0	1	NA	0	0	0	0	0	0	0	1	NA
Solyc01g009450.2.1	Mitochondrial inner membra	195.4	173.91	282.99	287.05	234.84	190.5	169.27	159.88	189.45	177.27	0.75	0.678841	220.11	212.87	301.62	244.87	303.88	249.13	218.09	257.03	1.05	1	171.77	189.03	222.07	194.29	214.34	190.22	185.24	196.6	1.01	1	287	161.71	157.13	201.95	250.42	294.56	272.49	1.35	0.975227
Solyc01g009460.2.1	Unknown Protein (AHRD V1)	0	0	0	4.44	1.11	0	2.48	2.89	0	1.34	1.21	1	0	5.46	2.65	2.7	0	0	0	0	0	0.964678	2.54	0	0	0.85	0	0	0	0	0.01	1	0	0	0	0	0	0	0	1	NA
Solyc01g009470.1.1	Poly (AHRD V1 ***- B9SCR8_	0	0	0	0	0	0	0	0	0	0	1	NA	0	0	0.07	0.02	0	0	0	0	0.43	1	0	0	0	0	0	0	0	0	1	1	0	0	0	0	0	0	0	1	NA
Solyc01g009480.2.1	Hydrolase alpha/beta fold far	0	0	0	0	0	0	0	0	0	0	1	NA	0	0	0	0	0	0	0	0	1	NA	0	0	0	0	0	0	0	0	1	NA	0	0	0	0	0	0	0	1	NA
Solyc01g009490.1.1	cDNA clone J100026I16 full in	0	0	0	0	0	0	0	0	0	0	1	NA	0	0	0	0	0	0	0	0	1	NA	0	0	0	0	0	0	0	0	1	NA	0	0	0	0	0	0	0	1	NA
Solyc01g009500.2.1	Hydrolase alpha/beta fold far	9.94	9.02	7.44	7.3	8.43	10.2	12.98	10.02	8.37	10.39	1.23	0.806083	10.67	8.64	8.56	9.29	13	10.99	14.28	12.76	1.37	0.576823	6.03	9.82	4.37	6.74	5.4	5.8	7.39	6.2	0.92	1	7.07	5	5.29	5.79	5.25	7.38	6.31	1.09	1
Solyc01g009510.1.1	Unknown Protein (AHRD V1);	0	1.23	0.41	0.49	0.53	0	0	0.32	0.25	0.14	0.27	0.907712	0	0	0	0	0	0	0	0	1	NA	0	0	0	0	0	0	0	0	1	NA	0	0	0	0	0	0	0	1	NA
Solyc01g009520.2.1	Ribosomal protein (AHRD V1	409.2	341.73	373.38	365.95	372.56	325.77	305.54	277.56	357.07	316.48	0.85	0.927109	347.26	286.21	298.63	310.7	235.74	266.81	307.48	270.01	0.87	1	158.91	175.15	156.61	163.56	171.96	167.06	170.81	169.94	1.04	1	99.85	107.97	133.24	113.69	191.75	227.91	209.83	1.85	0.299701
Solyc01g009530.1.1	Genomic DNA chromosome 3	0	0.27	0	0	0.07	0	0	0	0	0	0.15	1	0	0	0	0	0	0.16	0	0.05	5.33	1	0.25	0	0	0.08	0	0	0	0	0.12	1	0	0	0	0	0	0	0	1	NA
Solyc01g009540.1.1	Genomic DNA chromosome 3	0	0	0	0	0	0	0	0	0	0	1	NA	0	0	0	0	0	0	0	0	1	NA	0	0	0	0	0	0	0	0	1	NA	0	0	0	0	0	0	0	1	NA
Solyc01g009550.1.1	Hypothetical chloroplast RF1	0	0	0	0	0	0	0	0	0	0	1	NA	0	0	0	0	0	0	0	0	1	NA	0	0	0.74	0.25	0	0	0	0	0.04	NA	0	0	0	0	0	0	0	1	NA
Solyc01g009560.1.1	Terminal flower 1 (Fragment)	0	0	0	0	0	0	0	0	0	0	1	NA	0	0	0	0	0	0	0	0	1	NA	0	0	0	0	0	0	0	0	1	NA	0	0	0	0	0	0	0	1	NA
Solyc01g009570.2.1	Unknown Protein (AHRD V1)	3.96	3.52	0	0	1.87	2.93	2.08	3.64	2.84	2.87	1.54	1	1.23	0	0	0.41	0	0	1.04	0.35	0.85	1	0	0	0	0	0	0	0	0	1	NA	0	0	0	0	0	0	0	1	NA
Solyc01g009580.1.1	Terminal flower 1 (Fragment)	0	0	0	0	0	0	0	0	0	0	1	NA	0	0	0	0	0	0	0	0	1	NA	0	0	0	0	0	0	0	0	1	NA	0	0	0	0	0	0	0	1	NA
Solyc01g009590.2.1	A9-like protein (Fragment) (A	0	0	0	0	0	0	0	0	0	0	1	NA	0	0	0	0	0	0	0	0	1	NA	0	0	0	0	0	0	0	0	1	NA	0	0	0	0	0	0	0	1	NA
Solyc01g009600.2.1	Dehydration-responsive famil	0	0	0	0	0	0	0	0	0	0	1	NA	0	0	0	0	0	0	0	0	1	NA	0	0	0	0	0	0	0	0	1	NA	0	0	0	0	0	0	0	1	NA
Solyc01g009610.2.1	Unknown Protein (AHRD V1)	168.66	145.68	125.56	143.5	145.85	150.81	227.49	143.63	157.73	169.92	1.16	0.727093	128.52	108.74	78.85	105.37	83.31	115.04	106.62	101.66	0.96	1	62.35	54.26	34.59	50.4	44.16	42.93	45.81	44.3	0.88	1	36.28	40.23	29.67	35.39	51.27	41.47	46.37	1.31	1
Solyc01g009620.2.1	Cleavage and polyadenylation	41.03	35.93	34.46	36.21	36.91	39.77	40.42	35.31	42.41	39.48	1.07	0.997392	29.31	27.39	32.16	29.62	30.28	32.29	35.59	32.72	1.1	1	26.78	31.83	36.16	31.59	28.46	39.3	35.64	34.47	1.09	1	30.8	28.84	26.75	28.8	45.97	40.58	43.27	1.5	0.910144
Solyc01g009630.2.1	Cleavage and polyadenylation	53.17	37.39	38.34	33.89	40.7	59.83	71.52	64.58	53.96	62.47	1.54	0.111378	41.83	35.95	29.66	35.81	36.42	47.41	51.59	45.14	1.26	0.622656	42.16	48.41	55.06	48.54	54.98	74.63	52.38	60.66	1.25	1	51.33	54.83	52.49	52.88	73.61	70.22	71.91	1.36	1
Solyc01g009640.1.1	Polygalacturonase (AHRD V1	0	0	0	0	0	0	0	0	0	0	1	NA	0	0	0	0	0	0	0	0	1	NA	0	0	0	0	0	0	0	0	1	NA	0	0	0	0	0	0	0	1	NA
Solyc01g009650.1.1	MYB transcription factor (AH	0	0	0	0	0	0	0	0	0	0	1	NA	0	0	0	0	0	0	0	0	1	NA	0	0	0	0	0	0	0	0	1	NA	0	0	0	0	0	0	0	1	NA
Solyc01g009660.1.1	Low-temperature-induced 65	0	0	0	0	0	0	0	0	0	0	1	NA	0.32	0	0	0.11	0	0	0	0	0.09	1	0	0	0	0	0	0	0	0	1	NA	0	0	0	0	0	0	0	1	NA
Solyc01g009670.2.1	CCA-adding enzyme (AHRD V	1.79	1.37	0.91	0.91	1.25	1.99	1.52	1.88	1.66	1.76	1.42	0.876237	0.71	0.89	0.97	0.86	0.98	1.13	1.71	1.27	1.49	0.955554	0.41	0.42	0.25	0.36	0	0.1	0.47	0.19	0.53	1	0	0.19	0	0.06	0.35	0.22	0.28	4.5	1
Solyc01g009680.2.1	Lipoxygenase (AHRD V1 ***-	0.15	0.79	1.75	0.42	0.78	4.79	2.46	2.05	1.39	2.67	3.44	0.241245	0	0	0	0	1.03	0.62	0.82	0.82	82.33	0.022203	3.12	2.42	4.87	3.47	4.29	8	6.17	6.15	1.77	1	0	0.76	1.83	0.86	3.26	3.26	3.26	3.78	0.275183
Solyc01g009690.1.1	LRR receptor-like serine/thre	1.07	0.88	0.54	0.75	0.81	0.59	1.2	1.47	1.09	1.09	1.34	0.865129	0.43	1.52	0.77	0.91	1.43	0.47	1.14	1.01	1.12	1	0.55	0.31	0.23	0.36	0.85	1.1	1.39	1.11	3.06	1	0.11	0.83	0.7	0.55	0.49	0.6	0.54	1	1
Solyc01g009700.1.1	LRR receptor-like serine/thre	0.59	0.52	0.12	0.41	0.41	0.33	0.69	0.63	0.63	0.57	1.39	0.989925	0.09	2.04	0.91	1.01	1.35	0.71	0.61	0.89	0.88	1	0.79	0.08	0.29	0.39	0.58	0.59	0.43	0.53	1.38	1	0	0.71	1.05	0.59	0.45	0.34	0.4	0.67	1
Solyc01g009710.1.1	Unknown Protein (AHRD V1)	0	0	0	0	0	0	0	0	0	0	1	NA	0	0	0	0	0	0.3	0	0.1	10	1	0	0	0	0	0	0	0	0	1	NA	0	0	0	0	0	0	0	1	NA
Solyc01g009720.1.1	Zinc finger family protein (AH	0.2	0.17	0	0	0.09	0	0	0	0	0	0.11	1	0.18	0	0	0.06	0	0	0	0	0.17	1	0	0	0	0	0	0	0.14	0.05	4.67	1	0	0	0	0	0	0	0	1	NA
Solyc01g009730.1.1	Unknow protein (AHRD V1 *-	0	0	0	0	0	0	0	0	0	0	1	NA	0	0	0	0	0	0	0	0	1	NA	0	0	0	0	0	0	0	0	1	NA	0	0	0.3	0.1	0	0	0	0.1	1
Solyc01g009740.1.1	Unknown Protein (AHRD V1);	0	0	0	0	0	0	0	0	0	0	1	NA	0	0	0	0	0	0	0	0	1	NA	0	0	0	0	0	0	0	0	1	NA	0	0	0	0	0	0	0	1	NA
Solyc01g009750.2.1	Unknown Protein (AHRD V1)	260.94	236.43	254.34	221.63	243.34	266.48	223.68	249.12	271.06	252.58	1.04	1	286.55	318.99	304.47	303.34	398.73	409.89	328.66	379.09	1.25	0.494761	218.9	227.51	237.37	227.93	281.7	312.06	243.8	279.19	1.22	1	146.56	163.42	197.46	169.15	260.43	311.94	286.19	1.69	0.522113
Solyc01g009760.2.1	GRAM-containing/ABA-respo	0.84	1	1.32	1.59	1.19	0	1.11	0	0	0.28	0.23	0.514166	0.78	3.17	2.61	2.19	0.58	2.33	1.54	1.48	0.68	1	0.45	2.52	1.11	1.36	0.92	1.06	0.41	0.8	0.59	1	5.19	1.43	2.59	3.07	2.57	2.46	2.51	0.82	1
Solyc01g009770.2.1	Major ampullate spidroin 2-2	3.75	5.01	2.85	4.17	3.94	6.86	2.33	2.47	2.7	3.59	0.91	1	3.99	3.03	1.59	2.87	4.65	2.22	2.32	3.06	1.07	1	15.82	7.43	13.04	12.1	15.15	23.17	21.11	19.81	1.64	1	5.72	6.62	5.77	6.04	4.17	2.82	3.5	0.58	1
Solyc01g009780.2.1	LITAF-domain-containing pro	65.54	74.45	72.74	81.73	73.62	81	56.51	78.1	67.17	70.69	0.96	1	89.44	100.83	88.86	93.04	90.02	75.01	61.67	75.57	0.81	0.906866	77.68	72.73	91.59	80.67	80.73	92.81	74.15	82.56	1.02	1	281.39	147.5	123.99	184.29	144.86	117.2	131.03	0.71	1
Solyc01g009790.1.1	Unknown Protein (AHRD V1)	0	0	0	0	0	0	0	0	0	0	1	NA	0	0	0	0	0	0	0	0	1	NA	0	0	0	0	0	0	0	0	1	NA	0	0	0	0	0	0	0	1	NA
Solyc01g009800.1.1	Mutator-like transposase 538	0	0	0	0	0	0	0	0	0	0	1	NA	0	0	0	0	0	0	0	0	1	NA	0	0	0	0	0	0	0	0	1	NA	0	0	0	0	0	0	0	1	NA
Solyc01g009810.2.1	LRR receptor-like serine/thre	0	0	0	0	0	0	0.26	0	0	0.07	6.5	1	0	0	0	0	0	0	0	0	1	NA	0	0	0	0	0	0	0	0	1	NA	0	0	0	0	0	0	0	1	NA
Solyc01g009820.2.1	Hydroxyproline-rich glycopro	16.68	15.4	20.04	29.93	20.51	13.79	15.39	12.36	11.82	13.34	0.65	0.518183	16	35.88	38.64	30.17	26.67	21.48	22.44	23.53	0.78	0.962778	17.85	17.24	20.48	18.52	24.03	18.64	17.01	19.89	1.07	1	104.17	50.34	56.09	70.2	41.88	40.94	41.41	0.59	1
Solyc01g009830.2.1	UDP-glucuronic acid/UDP-N-a	6.81	5.72	2.76	4.68	4.99	3.89	3.07	1.97	4.75	3.42	0.69	0.694053	10.86	22.33	13.31	15.5	18.49	20.76	27.53	22.26	1.44	0.561621	1.42	5.07	0.58	2.36	1.53	0	1.42	0.98	0.42	1	0	0	0	0	0	0	0	1	NA
Solyc01g009840.1.1	GAI-like protein 1 (Fragment)	0	0	0	0	0	0	0	0	0	0	1	NA	0.24	0	0.22	0.15	0	0.14	0	0.05	0.3	1	0	0	0	0	0	0	0	0	1	NA	0	0	0	0	0	0	0	1	NA
Solyc01g009850.2.1	Unknown Protein (AHRD V1)	150.17	130.56	115.72	114.19	127.66	145.7	115.82	157.23	114.67	133.35	1.04	1	146.95	116.2	92.38	118.51	85.49	88.09	88	87.19	0.74	0.674862	147.97	152.08	130.1	143.38	123.17	146.06	132.84	134.02	0.93	1	58.01	82.59	74.75	71.78	119.19	85.24	102.22	1.42	1
Solyc01g009860.2.1	NAC domain transcription fac	15.22	23.01	12.54	23.24	18.5	21.49	17.36	26.22	21.17	21.56	1.17	0.854533	24.5	81.92	52.25	52.89	93.86	44.17	65.52	67.85	1.28	1	21.28	46.47	14.63	27.46	88.58	31.14	49.31	56.34	2.05	0.467274	42.95	83.37	72.46	66.26	25.88	45.34	35.61	0.54	0.425599
Solyc01g009870.2.1	Glycerophosphodiester phosp	11.55	22.2	11.46	26.53	17.94	32.39	26.44	27.86	20.68	26.84	1.5	0.30198	15.42	33.91	29.42	26.25	31.82	40.57	36.05	36.15	1.38	0.440282	0.54	1.91	3.82	2.09	4.29	2.16	0.73	2.39	1.15	1	2.14	4.74	6.44	4.44	2.15	1.61	1.88	0.42	0.74351
Solyc01g009880.2.1	Exocyst complex protein EXO	0	0	0	0	0	0	0	0	0	0	1	NA	0	0	0	0	0	0	0	0	1	NA	0	0	0	0	0	0	0	0	1	NA	0	0	0	0	0	0	0	1	NA

Solyc01g009890.1.1	Glutaredoxin (AHRD V1 ***-	0	0	0	0	0	0	0	0	0	0	1	NA	0	0	0	0	0	0	0	0	1	NA	0	0	0	0	0	0	0	0	1	NA	0	0	0	0	0	0	0	1	NA
Solyc01g009900.2.1	Glutaredoxin (AHRD V1 ***-	0	0	0	0	0	0	0	0	0	0	1	NA	0	0	0	0	0	0	0	0	1	NA	0	0	0	0	0	0	0	0	1	NA	0	0	0	0	0	0	0	1	NA
Solyc01g009910.1.1	Glutaredoxin (AHRD V1 ***-	0	0	0	0	0	0	0	0	0	0	1	NA	0	0	0	0	0	0	0	0	1	NA	0	0	0	0	0	0	0	0	1	NA	0	0	0	0	0	0	0	1	NA
Solyc01g009920.1.1	MORC family CW-type zinc fi	0	0	0	0	0	0	0	0	0	0	1	NA	0	0	0	0	0	0	0	0	1	NA	0	0	0	0	0	0	0	0	1	NA	0	0	0	0	0	0	0	1	NA
Solyc01g009930.1.1	LRR receptor-like serine/thre	0	0	0	0.16	0.04	0	0	0	0	0	0.25	1	0	0.1	0	0.03	0	0	0	0	0.3	1	0	0	0	0	0	0	0	0	1	NA	0	0	0	0	0	0.1	0.05	5	1
Solyc01g009940.1.1	5-methylthioadenosine/S-ade	0	0	0	0	0	0	0	0	0	0	1	NA	0	0	0	0	0	0	0	0	1	NA	0	0	0	0	0	0	0	0	1	1	0	0	0	0	0	0	0	1	NA
Solyc01g009950.1.1	Polyprotein-like related (AHR	0	0	0	0	0	0	0	0	0	0	1	NA	0	0	0	0	0	0	0	0	1	NA	0	0	0	0	0	0	0	0	1	NA	0	0	0	0	0	0	0	1	NA
Solyc01g009960.2.1	Stearoyl-acyl-carrier protein	0	0	0	0	0	0	0	0	0	0	1	NA	0	0	0	0	0	0	0	0	1	NA	0	0	0	0	0	0	0	0	1	NA	0	0	0	0	0	0	0	1	NA
Solyc01g009970.2.1	Amidohydrolase 2 (AHRD V1	21.16	25.28	14.4	16.45	19.32	32	32.21	31.2	26.56	30.49	1.58	0.126851	17.56	18.73	16.27	17.52	19.44	24.78	25.02	23.08	1.32	0.511422	36.35	37.6	28.77	34.24	21.17	29.19	33.35	27.9	0.81	1	5.69	12.19	8.21	8.7	17.1	16.05	16.58	1.91	0.737405
Solyc01g009980.1.1	Retrotransposon like protein	0	0	0	0	0	0	0	0	0	0	1	NA	0	0	0	0	0	0	0	0	1	NA	0	0	0	0	0	0	0	0	1	NA	0	0	0	0	0	0	0	1	NA
Solyc01g009990.2.1	Peptidyl-prolyl cis-trans isom	173.27	161.52	192.86	224.86	188.13	234.72	285.72	182.94	258.12	240.38	1.28	0.283396	164.13	152.24	154.85	157.07	192.76	204.67	164.07	187.17	1.19	0.654599	90.33	143.79	135.44	123.19	131.41	115.47	142.1	129.66	1.05	1	62.91	115.07	91.26	89.75	170.01	160.29	165.15	1.84	0.507916
Solyc01g010000.2.1	ACT domain-containing prote	8.65	7.7	9.17	3.53	7.26	6.36	4.51	7.41	6.84	6.28	0.86	0.977607	6.82	6.12	6.81	6.58	8.34	8.27	6.68	7.76	1.18	0.913909	13.27	12.42	10.19	11.96	10.88	10.67	11.11	10.89	0.91	1	11.05	18.63	13.08	14.25	14.76	12.85	13.8	0.97	1
Solyc01g010010.1.1	Mutator-like transposase (AH	0	0	0	0	0	0	0	0	0	0	1	NA	0	0	0	0	0	0	0	0	1	NA	0	0	0	0	0	0	0	0	1	NA	0	0	0	0	0	0	0	1	NA
Solyc01g010020.2.1	Calmodulin (AHRD V1 ***- B	1.63	2.54	0.96	4.61	2.44	1.36	0.96	1.12	2.63	1.52	0.62	1	1.14	1.77	3.78	2.23	3.95	3.37	1.92	3.08	1.38	1	0.66	2.65	1.62	1.64	1.61	1.85	0.59	1.35	0.82	1	2.89	1.48	3.13	2.5	1.86	4.64	3.25	1.3	1
Solyc01g010030.2.1	Receptor-like protein kinase	3.25	4.02	5.22	5.11	4.4	4.21	4.56	3.98	3.3	4.01	0.91	1	3.11	2.12	3.51	2.91	2.57	3.08	2.2	2.62	0.9	1	2.77	2.35	2.42	2.51	3.38	3.08	3.16	3.21	1.28	1	1.41	1.84	2.08	1.78	3.22	3.09	3.16	1.78	0.90597
Solyc01g010040.2.1	S-adenosylmethionine decarb	77.77	100.32	78.86	81.57	84.63	94.7	93.82	102.43	80.3	92.81	1.1	0.97992	76.4	75.96	74.54	75.63	80.6	65.15	114.33	86.69	1.15	0.983162	77.65	67.29	53.77	66.24	42.76	56.29	59.22	52.76	0.8	1	101.25	56.8	46.36	68.14	36.39	42.14	39.27	0.58	1
Solyc01g010050.2.1	S-adenosylmethionine decarb	32.17	41.5	32.63	33.75	35.01	39.18	38.81	42.38	33.22	38.4	1.1	0.97992	31.61	31.42	30.84	31.29	33.35	26.95	47.3	35.87	1.15	0.983162	32.13	27.84	22.25	27.41	17.69	23.29	24.5	21.83	0.8	1	41.89	23.5	19.18	28.19	15.06	17.43	16.25	0.58	1
Solyc01g010060.2.1	Unknown Protein (AHRD V1)	6.14	1.73	0.38	2.29	2.63	0	0.26	0.3	0.93	0.37	0.14	0.420546	0.3	1.12	0.55	0.66	0.22	1	1.27	0.83	1.26	1	0.78	1.58	2.25	1.54	0.42	0.98	0	0.47	0.3	1	2.3	11.04	5.97	6.44	2.07	1.98	2.02	0.31	0.755623
Solyc01g010070.1.1	Unknown Protein (AHRD V1)	0	0	0	0	0	0	0	0	0	0	1	NA	0	0	0	0	0	0	0	0	1	NA	0	0	0	0	0	0	0	0	1	NA	0	0	0	0	0	0	0	1	NA
Solyc01g010080.2.1	Potassium/sodium hyperpola	0	0	0.12	0	0.03	0	0	0	0	0	0.33	1	0	0	0	0	0	0	0	0	1	NA	0	0	0	0	0	0	0	0	1	NA	0	0	0	0	0	0	0	1	NA
Solyc01g010090.1.1	Unknown Protein (AHRD V1)	0	0	0	0	0	0	0	0	0	0	1	NA	0	0	0	0	0	0	0	0	1	NA	0	0	0	0	0	0	0	0	1	NA	0	0	0	0	0	0	0	1	NA
Solyc01g010100.1.1	Ulp1 protease family C-termi	0	0	0	0	0	0	0	0	0	0	1	NA	0	0	0	0	0	0	0	0	1	NA	0	0	0	0	0	0	0	0	1	NA	0	0	0	0	0	0	0	1	NA
Solyc01g010110.1.1	Mutator-like transposase (AH	0	0	0	0	0	0	0	0	0	0	1	NA	0	0.71	0.23	0.31	0.76	1.55	0.86	1.06	3.37	0.660359	0	0.67	0.27	0.31	0.18	0	0.6	0.26	0.83	1	3.1	1.78	2.52	2.47	0.25	2.63	1.44	0.58	1
Solyc01g010120.1.1	Unknown Protein (AHRD V1)	0	0	0	0	0	0	0	0	0	0	1	NA	0	0	0	0	0	0	0	0	1	NA	0	0	0	0	0	0	0	0	1	NA	0	0	0	0	0	0	0	1	NA
Solyc01g010130.2.1	Transcription factor (AHRD V	4.17	6.22	6.08	3.64	5.03	3.95	2.61	3.04	3.61	3.3	0.66	0.452852	3.76	2.66	4.45	3.62	3.66	3.49	2.99	3.38	0.93	1	1.49	0.9	1.22	1.2	0.81	1.02	0.8	0.88	0.73	1	1.05	1.78	1.7	1.51	1.23	1.61	1.42	0.94	1
Solyc01g010140.1.1	Unknown Protein (AHRD V1)	0	0	0.16	0	0.04	0	0	0	0	0	0.25	1	0	0	0	0	0	0	0	0	1	NA	0	0	0	0	0	0	0	0	1	NA	0	0	0	0	0	0	0	1	NA
Solyc01g010150.2.1	Unknown protein DS12 from	1.18	2.35	2.43	1.66	1.91	1.14	1.51	1.89	2.11	1.66	0.87	1	3.55	1.66	1.49	2.23	2.85	3.42	3.58	3.28	1.47	0.682402	3.21	4.79	2.92	3.64	3.47	5.12	4.93	4.51	1.24	1	3.97	8.32	6.1	6.13	4.44	4.9	4.67	0.76	1
Solyc01g010160.2.1	Beta-glucosidase (AHRD V1 *	0	0	0	0	0	0	0	0	0	0	1	NA	0	0	0	0	0	0	0	0	1	NA	0.57	0	1.39	0.65	0	0	0	0	0.02	1	14.97	41.8	21.58	26.12	6.42	4.31	5.37	0.21	0.214066
Solyc01g010170.1.1	Unknown Protein (AHRD V1)	0	0	0	0	0	0	0	0	0	0	1	NA	0	0	0	0	0	0	0	0	1	NA	0	0	0	0	0	0	1.5	0.5	50	1	5.86	20.96	3.17	10	9.44	3.62	6.53	0.65	1
Solyc01g010180.2.1	Unknown Protein (AHRD V1)	96.68	81.66	67.54	57.92	75.95	174.9	122.13	232.17	181.01	177.55	2.34	0.000421	39.5	29	12.49	27	33.13	42.4	60.19	45.24	1.68	0.494783	5.51	4.02	2.26	3.93	4.23	3.45	2.76	3.48	0.89	1	0	0.83	1.75	0.86	0.35	0	0.17	0.2	1
Solyc01g010190.2.1	Unknown Protein (AHRD V1)	38.61	42.48	42.32	47.3	42.68	28.05	26.16	35.78	42.12	33.03	0.77	0.783572	49.77	57.35	43.14	50.09	31.42	57.61	29.63	39.55	0.79	1	14.25	10.4	8.75	11.13	17.97	13.83	13.74	15.18	1.36	1	72.19	47.25	49.09	56.18	43.69	48.25	45.97	0.82	1
Solyc01g010200.2.1	LTRGag-pol-polymerase 3 (AH	656.86	810.4	542.42	419.92	607.4	722	562.94	1182.8	603.72	767.87	1.26	0.892017	581.07	663.03	320.91	521.67	453.89	791.53	734.15	659.86	1.26	0.821464	111.53	105.52	66.69	94.58	133.23	150.62	113.36	132.4	1.4	1	90.32	159.13	152.8	134.08	114.44	181.38	147.91	1.1	1
Solyc01g010210.1.1	Unknown Protein (AHRD V1)	0	0	0	0	0	0	0	0	0	0	1	NA	0	0	0	0	0	0	0	0	1	NA	0	0	0.35	0.12	0	0	0	0	0.09	NA	0	0	0	0	0	0	0	1	NA
Solyc01g010220.2.1	Unknown Protein (AHRD V1)	22.85	21.98	10.87	15.64	17.84	11.88	11.35	16.6	15.61	13.86	0.78	0.788276	15.08	16.34	20.55	17.32	19.68	20.04	16.22	18.65	1.08	1	2.68	8.11	2.56	4.45	7.75	6.7	6.98	7.14	1.61	1	2.62	9.9	10.77	7.76	8.43	5.82	7.12	0.92	1
Solyc01g010230.2.1	Unknown Protein (AHRD V1)	66.53	71.65	58.04	43.49	59.93	58.42	41.05	92.01	50.97	60.61	1.01	1	68.65	86.02	38.72	64.46	65.92	95.53	68.54	76.66	1.19	0.903895	20.33	18.68	4.44	14.48	17.26	19.07	13.85	16.73	1.15	1	46.9	88.93	77.37	71.07	35.82	59.31	47.56	0.67	0.854433
Solyc01g010240.2.1	CRABS CLAW (Fragment) (AH	0	0	0	0	0	0	0	0	0	0	1	NA	0	0	0	0	0	0	0	0	1	NA	0	0	0	0	0	0	0	0	1	NA	0	0	0	0	0	0	0	1	NA
Solyc01g010250.2.1	6-phosphogluconolactonase (	18.78	14.07	18.07	13.98	16.23	13.18	18.34	12.27	9.05	13.21	0.81	0.907108	28.27	26.63	22.75	25.88	37.36	30.06	26.21	31.21	1.21	0.772021	39.33	38.46	31.39	36.39	58.76	43.63	52.04	51.48	1.41	1	26	53.48	39.13	39.54	20.81	22.75	21.78	0.55	0.414921
Solyc01g010260.2.1	Cytochrome P450	0	0	0	0	0	0	0	0.12	0	0.03	3	1	0	0	0	0	0	0	0	0	1	NA	0	0	0	0	0	0	0	0	1	NA	0	0	0	0	0	0	0	1	NA
Solyc01g010270.2.1	Nitrilase associated protein-li	564.21	564.21	441.05	532.34	525.45	594.34	634.91	485.83	667.13	595.55	1.13	0.790281	536.51	436.39	385.8	452.9	315.5	434.94	377.23	375.89	0.83	0.963902	147.21	196.6	109.76	151.19	119.7	135.24	136.44	130.46	0.86	1	182.24	155.61	123.67	153.84	203.6	212.64	208.12	1.35	1
Solyc01g010280.2.1	Unknown Protein (AHRD V1)	0	0	0	0	0	0.17	0	0	0	0.04	4.25	1	0	0	0	0	0	0	0.12	0.04	4	1	0	0	0	0	0	0	0	0	1	NA	0	0	0	0	0	0	0	1	NA
Solyc01g010290.2.1	Ureide permease 2 (AHRD V1	26.36	26.19	22.89	20.58	24	28.4	14.81	37.32	23.8	26.08	1.09	1	26.77	20.8	15.02	20.86	14.92	17.41	18.81	17.05	0.82	0.98388	16.07	13.57	10.43	13.36	11.58	7.35	10.02	9.65	0.72	1	0.69	2.23	1.24	1.39	2.66	1.13	1.9	1.37	1
Solyc01g010300.1.1	MADS-box protein-like (AHRD	0	0	0	0	0	0	0	0	0	0	1	NA	0	0	0	0	0	0	0	0	1	NA	0	0	0	0	0	0	0	0	1	NA	0	0	0	0	0	0	0	1	NA
Solyc01g010310.2.1	Glucan endo-1 3-beta-glucosi	86.46	67.06	82.75	67.29	75.89	84.14	58.39	52.36	73.79	67.17	0.89	1	42.96	26.86	34.61	34.81	14.69	24.45	24.01	21.05	0.6	0.313149	14.31	20.37	9.65	14.78	11.4	7.89	13.16	10.82	0.73	1	0.99	0.5	1.07	0.85	0	1.52	0.76	0.89	1
Solyc01g010320.1.1	Mutator-like transposase (AH	0.33	0.3	0	0.47	0.28	0	0.26	0	0	0.07	0.24	1	0	0.29	0.56	0.28	0.46	0.17	0	0.21	0.74	1	0	0.27	0	0.09	0	0	0	0	0.11	1	0	0.24	0	0.08	0	0	0	0.12	1
Solyc01g010330.1.1	Unknown Protein (AHRD V1)	0	0	0	0	0	0	0	0	0	0	1	NA	0	0	0	0	0	0	0	0	1	NA	0	0	0	0	0	0	0	0	1	NA	0	0	0	0	0	0	0	1	NA
Solyc01g010340.1.1	Serine/threonine-protein pho	0.14	0	0	0.19	0.08	0.15	0	0.38	0.1	0.16	1.91	1	0.13	0.12	0.23	0.16	0	0.21	0.11	0.11	0.67	1	0.11	0.11	0.68	0.3	0.09	0.21	0.3	0.2	0.67	1	0.2	0.6	0	0.27	0.38	0.24	0.31	1.16	1
Solyc01g010350.2.1	Glucose-6-phosphate/phosph	0.16	0.14	0	0	0.08	0.18	0.25	0	0.12	0.14	1.83	1	0	0	0	0	0	0	0	0	1	NA	0	0	0	0	0	0	0	0	1	NA	0	0	0	0	0	0	0	1	NA
Solyc01g010360.2.1	14-3-3-like protein GF14 epsi	5.52	6.19	3.96	3.73	4.85	1.87	2.08	1.76	3.44	2.29	0.47	0.241561	2.45	2.92	2.03	2.47	0.17	1.74	1.89	1.27	0.51	0.741102	0.97	3.52	1.43	1.97	0.16	0.36	0.52	0.35	0.18	1	0	0.52	1.11	0.54	0	0	0	0.02	1
Solyc01g010370.2.1	Dehydration-responsive prote	3.1	4.66	2.65	5.29	3.92	3.21	3.38	1.77	2.53	2.72	0.69	0.676281	2.39	2.04	2.71	2.38	1.26	1.49	1.51	1.42	0.6	0.638843	2.68	2.44	1.8	2.31	1.4	0.73	1.25	1.13	0.49	1	0	0.08	0.33	0.14	0.1	0.09	0.1	0.7	1
Solyc01g010380.2.1	Unknown Protein (AHRD V1)	0	0	0	0	0	0	0	0	0	0	1	NA	0	0	0	0	0	0	0	0	1	NA	0	0	0	0	0	0	0	0	1	NA	0	0	0	0	0	0	0	1	NA
Solyc01g010390.2.1	Beta-glucosidase (AHRD V1 *	2.47	3.35	3.22	4.22	3.32	3.9	5.13	3.1	3.54	3.92	1.18	0.888406	4.35	4.06	4.28	4.23	6.12	2.62	3.37	4.04	0.95	1	0.31	0.85	0.13	0.43	0.85	1.08	1.42	1.12	2.6	1	0.37	2.08	1.4	1.28	0.12	0.34	0.23	0.18	0.567611
Solyc01g010400.1.1	Ripening-related protein 3 (A	0	0	0	0	0	0	0	0	0	0	1	NA	0	0	0	0	0	0	0	0	1	NA	0	0	0	0	0	0	0	0	1	NA	0	0	0	0	0	0	0	1	NA
Solyc01g010410.2.1	Cys2/His2 zinc-finger transcri	1.26	0.81	1.75	2.1	1.48	0.76	0.9	1.36	0.9	0.98	0.66	0.924458	1.59	2.38	1.54	1.84	1.9	2.36	1.35	1.87	1.02	1	3.14	3.72	1.7	2.85	3.45	1.64	2.91	2.67	0.93	1	1.3	2.32	4.21	2.61	0.94	1	0.97	0.37	0.851215
Solyc01g010420.2.1	NPH3 domain containing prot	0	0	0	0	0	0.36	0	0	0.23	0.15	14.75	1	0	0	0.27	0.09	0.23	0	0	0.08	0.85	1	0	0	0	0	0	0	0	0	1	NA	0	0	0	0	0	0.28	0.14	14	1
Solyc01g010430.2.1	cDNA clone J023121M11 full	18.01	17.38	16.9	21.49	18.45	8.93	9.25	14.57	15.42	12.04	0.65	0.408318	32.3	23.96	23.83	26.7	27.24	26.29	12.62	22.05	0.83	1	18.23	20.87	17.02	18.71	17.39	14.31	14.23	15.31	0.82	1	48.13	123.41	152.17	107.9	62.97	90	76.48	0.71	1
Solyc01g010440.2.1	Unknown Protein (AHRD V1)	60.61	56.2	72.39	55.61	61.2	44.86	47.5	35.74	50.46	44.64	0.73	0.482348	51.39	71.59	78.24	67.07	134.17	89.98	89.41	104.52	1.56	0.27134	30.2	44.54	32.52	35.75	45.69	38.38	45.69	43.25	1.21	1	84.9	65.11	76.71	75.57	81.38	97.14	89.26	1.18	1
Solyc01g010450.1.1	Ulp1 protease family C-termi	0	0	0	0	0	0	0	0	0	0	1	NA	0	0	0	0	0	0	0	0	1	NA	0	0	0	0	0	0	0	0	1	NA	0	0	0	0	0	0	0	1	NA
Solyc01g010460.2.1	Unknown Protein (AHRD V1)	3.46	3.24	3.68	3.18	3.39	4.23	4.1	4.3	4.1	4.18	1.23	0.90174	3.06	2.86	3.36	3.09	2.28	3.77	3.81	3.29	1.06	1	2.94	2.68	1.2	2.27	3.41	2.23	3.03	2.89	1.27	1	1.72	1.51	2.66	1.96	2.38	1.97	2.17	1.11	1
Solyc01g010470.2.1	Isoamylase N-terminal domai	6.03	13.89	8.37	10.03	9.58	14.59	12.04	16.31	8.4	12.83	1.34	0.759724	11.88	8.63	9.89	10.13	5.9	7.35	8.36	7.2	0.71	0.863933	6.3	5.78	6.69	6.26	6.29	4.84	6.46	5.86	0.94	1	3.53	6.7	7.64	5.96	6.82	6.84	6.83	1.15	1
Solyc01g010480.2.1	Potassium voltage-gated cha	32.99	38.79	30.25	16.44	29.62	39.14	44.8	42.9	47.85	43.67	1.47	0.18782	68.68	41.3	41.86	50.61	65.2	86.72	81.26	77.73	1.54	0.101276	54.13	50.97	35.46	46.85	54.9	46.63	53.18	51.57	1.1	1	3.64	12.41	7.62	7.89	20.81	20.08	20.45	2.59	0.223754
Solyc01g010490.2.1	Cytochrome P450	0.91	0.93	2.31	0.74	1.22	1.02	0.83	1.68	1.31	1.21	0.99	1	6.44	18.74	10.26	11.81	21.56	23.81	24.74	23.37	1.98	0.021041	52.77	65.47	38.38	52.21	62.91	46.04	87.39	65.45	1.25	1	6.32	41.94	20.29	22.85	29.06	12.83	20.95	0.92	1
Solyc01g010500.1.1	Ein3-binding f-box protein 3 (	0	0	0	0	0	0	0	0	0.11	0.03	2.75	1	0	0	0	0	0	0	0	0	1	NA	0	0	0	0	0	0	0	0	1	NA	0	0	0	0	0	0	0	1	NA
Solyc01g010510.2.1	Pentatricopeptide repeat-con	0.66	1.08	0.26	0.47	0.62	0.61	1.05	1.22	0.48	0.84	1.36	1	1.13	0.86	1.31	1.1	0.92	0.97	1.22	1.04	0.94	1	0.54	0.36	0.22	0.37	0.65	0.5	1.05	0.73	1.96	1	0.31	0.56	1.36	0.74	1.32	0.78	1.05	1.41	1
Solyc01g010520.2.1	Uncharacterized plant-specifi	9.92	13.08	11.27	12.22	11.62	21.86	17.12	15.04	19.13	18.29	1.57	0.139722	7.86	7.19	6.83	7.29	4.08	7.61	5.78	5.82	0.8	1	0.89	2.55	0.36	1.27	0.85	0.7	1.07	0.87	0.69	1	0	0	0.85	0.28	0.5	0.32	0.41	1.45	1
Solyc01g010530.1.1	Solute carrier family 2, facilit	0	0	0	0	0	0	0	0	0	0	1	NA	0	0	0	0	0	0	0	0	1	NA	0	0	0	0	0	0	0	0	1	NA	0	0	0	0	0	0	0	1	NA
Solyc01g010540.2.1	Ribosomal protein (AHRD V1	546.43	451.03	551.11	507.11	513.92	295.18	323.81	265.6	370.05	313.66	0.61	0.063475	494.64	464.28	459.56	472.83	504.24	442.5	363.7	436.81	0.92	1	172.01	257.11	242.09	223.74	260.25	247.23	233.57	247.02	1.1	1	202.28	253.13	327.4	260.94	322.08	411.33	366.7	1.41	0.893994
Solyc01g010550.1.1	Unknown Protein (AHRD V1)	0	0	0	0	0	0	0	0	0	0	1	NA	0	0	0	0	0	0	0	0	1	NA	0	0	0	0	0	0	0	0	1	NA	0	0	0	0	0	0	0	1	NA
Solyc01g010570.2.1	Mucin-like protein (Fragment	52.36	47.16	53.86	46.81	50.05	35.62	27.12	37.36	38.19	34.57	0.69	0.313823	39.35	44.06	56.25	46.55	66.24	53.35	55.79	58.46	1.26	0.61209	23.86	30.04	27.23	27.04	41.27	26.83	47.62	38.57	1.43	0.952815	107.79	67.84	82.77	86.13	55.98	62.62	59.3	0.69	1
Solyc01g010580.2.1	Ribosomal protein (AHRD V1	914.6	722.36	1014.17	898.75	887.47	569.99	550.55	450.94	604.23	543.93	0.61	0.063138	827.19	771.29	852.7	817.06	889.93	824.09	734.21	816.08	1	1	257.07	361.91	347.58	322.19	452.43	383.85	362.98	399.75	1.24	1	442.93	396.63	532.34	457.3	586.52	761.01	673.76	1.47	0.732269
Solyc01g010590.2.1	Peptidyl-prolyl cis-trans isom	47.25	53.07	41.47	53.38	48.79	46.43	55.95	63.57	54.39	55.08	1.13	0.845735	57.88	61.35	48.42	55.88	56.75	67.12	64.98	62.95	1.13	0.908433	28.16	38.73	27.45	31.45	30.66	24.45	40.1	31.74	1.01	1	36.86	39.55	32.34	36.25	34.28	34.86	34.57	0.95	1
Solyc01g010600.2.1	Homeobox-leucine zipper-like	0	0	0	0	0	0	0.21	0	0	0.05	5.25	1	0	0	0	0	0	0	0	0	1	NA	0	0	0	0	0	0	0	0	1	NA	0	0	0	0	0	0	0	1	NA
Solyc01g010610.1.1	Unknown Protein (AHRD V1);	1.55	1.38	1.83	3.29	2.01	0.86	0.61	0.71	1.67	0.96	0.48	1	1.44	2.02	5.9	3.12	0.54	1.61	2.44	1.53	0.49	0.94069	1.25	0	0	0.42	0	0	1.13	0.38	0.9	1	1.1	0.56	0	0.55	0.71	0	0.35	0.64	1
Solyc01g010630.1.1	Ulp1 protease family C-termi	0	0	0	0	0	0	0	0.44	0.46	0.23	22.5	0.625191	0	0	0	0	0.33	0.42	0.38	0.38	37.67	0.408252	0	0	0	0	1.37	1.34	1.17	1.29	129.33	0.273664	0	0	0.25	0.08	2.64	1.41	2.02	24.3	0.049385
Solyc01g010640.2.1	Uncharacterized membrane p	21.14	30.5	53.65	23.1	32.1	51.97	30.09	42.11	37.02	40.3	1.26	0.839697	33.07	31.18	35.98	33.41	56.42	41.29	36.49	44.73	1.34	0.458089	81.24	52.97	64.85	66.35	61.85	56.07	49.24	55.72	0.84	1	18.21	14.89	18.22	17.11	33.71	27.8	30.76	1.8	0.583641
Solyc01g010650.2.1	UDP-galactose transporter 3	54.12	46.06	48.47	56.16	51.2	36.23	32.12	23.79	32.9	31.26	0.61	0.097161	48.5	48.93	63.39	53.61	95.76	58.29	51.69	68.58	1.28	0.873941	26.49	49.25	35.29	37.01	31.14	29.49	39.15	33.26	0.9	1	26.68	26.11	18.22	23.67	41.95	52.13	47.04	1.99	0.340058
Solyc01g010660.2.1	Receptor-like protein kinase	11.62	13.14	8.9	12.44	11.52	9.43	9.18	7.08	7.1	8.2	0.71	0.523805	10.08	12.56	15.39	12.68	8.72	8.38	6.54	7.88	0.62	0.299027	10.15	9.6	14.5	11.42	11.97	9.17	7.44	9.53	0.83	1	17.2	30.8	27.78	25.26	28.9	25.07	26.98	1.07	1
Solyc01g010670.1.1	Unknown Protein (AHRD V1)	0	0	0	0	0	0	0	0	0	0	1	NA	0	0	0	0	0	0	0	0	1	NA	0	0	0	0	0	0	0	0	1	NA	0	0	0	0	0	0	0	1	NA
Solyc01g010680.1.1	Genomic DNA chromosome 5	0	0	0	0	0	0	0	0	0	0	1	NA	0.2	0	0	0.07	0	0	0	0	0.15	1	0	0	0	0	0	0	0	0	1	NA	0	0	0	0	0	0	0	1	NA
Solyc01g010690.1.1	Polyvinylalcohol dehydrogena	0	0	0	0	0	0	0	0	0	0	1	NA	0	0	0	0	0	0	0	0	1	NA	0	0	0	0	0	0	0	0	1	NA	0	0	0	0	0	0	0	1	NA
Solyc01g010700.2.1	AKIN gamma (AHRD V1 **--	19.42	20.71	23.26	16.99	20.09	25.21	22.59	20.85	21.71	22.59	1.12	0.919129	21.1	17.53	15.65	18.09	21.39	18.35	18.64	19.46	1.08	1	17.01	19.93	16.33	17.76	11.78	14.61	16.42	14.27	0.8	1	11.8	16.69	13.91	14.13	16.42	15.47	15.95	1.13	1
Solyc01g010710.2.1	Serine carboxypeptidase 1 (A	2.42	1.67	1.74	0.76	1.65	1.79	0.64	1.73	1.25	1.35	0.82	0.989253	3.75	1.52	1.59	2.29	0.19	0.83	0.95	0.66	0.29	0.361823	0	0	0	0	0	0	0	0	1	1	0	0	0	0	0	0	0	1	NA
Solyc01g010720.2.1	Serine carboxypeptidase K10	0	0	0	0	0	0	0	0	0	0	1	NA	0	0	0	0	0	0	0	0	1	NA	0	0	0	0	0	0	0	0	1	NA	0	0	0	0	0	0	0	1	NA
Solyc01g010730.1.1	Serine carboxypeptidase (AH	0	0	0	0	0	0	0	0	0	0	1	NA	0	0	0	0	0	0	0	0	1	NA	0	0	0	0	0	0	0	0	1	NA	0	0	0	0	0	0	0	1	NA
Solyc01g010740.2.1	Pectate lyase family protein (	0	0	0	0	0	0	0	0	0	0	1	NA	0	0	0	0	0	0	0	0	1	NA	0	0	0	0	0	0	0	0	1	NA	0	0.11	0	0.04	0	0	0	0.27	1
Solyc01g010750.2.1	Stress responsive protein (AH	426.6	380.75	608.66	728.32	536.08	356.15	473.98	278.87	402.87	377.97	0.71	0.754371	489.98	482.38	678.84	550.4	599.57	411.14	319.13	443.28	0.81	0.970881	368.38	463.33	554.6	462.1	430.61	455.5	399.98	428.7	0.93	1	478.71	391.5	342.69	404.3	449.8	485.67	467.74	1.16	1
Solyc01g010760.2.1	Porin/voltage-dependent ani	65.26	51.08	46.92	35.37	49.66	39.18	28.96	35.91	52.32	39.09	0.79	0.749797	40.98	48.44	34.6	41.34	42.78	28.61	34.63	35.34	0.85	0.98388	97.36	98.01	88.89	94.75	91.21	126.36	69.77	95.78	1.01	1	77.54	90.99	80.57	83.03	150	117.15	133.57	1.61	0.718052
Solyc01g010770.2.1	Spfh domain / band 7 family	0.72	0.65	2	0	0.84	0.54	0.76	2.22	1.21	1.18	1.4	1	1.12	4.62	3.88	3.21	5.36	7.38	8.93	7.22	2.25	0.086719	0.59	0.98	0.96	0.84	0.16	0.18	1.41	0.58	0.69	1	0.34	0	0	0.11	0.22	0	0.11	0.97	1
Solyc01g010780.2.1	ATP binding / serine-threonin	31.89	31.92	36.98	33.69	33.62	32.41	34.69	24.16	34.29	31.39	0.93	1	36.16	36.65	44.16	38.99	42.62	43.44	38.18	41.41	1.06	1	32.95	35.24	35.05	34.41	34.89	33.4	34.81	34.37	1	1	36.58	27.64	27.78	30.67	27.31	25.68	26.49	0.86	1
Solyc01g010790.1.1	F-box protein At2g21930 (AH	0	0	0	0	0	0	0	0	0	0	1	NA	0	0	0	0	0	0	0	0	1	NA	0	0	0	0	0	0	0	0	1	NA	0	0	0	0	0	0	0	1	NA
Solyc01g010800.1.1	Unknown Protein (AHRD V1)	0	0	0	0	0	0	0	0	0	0	1	NA	0	0	0	0	0	0	0	0	1	NA	0	0	0	0	0	0	0	0	1	NA	0	0	0	0	0	0	0	1	NA
Solyc01g010810.2.1	RAN guanine nucleotide relea	16.82	19.86	19.46	19.67	18.95	17.25	18.64	12.19	17.88	16.49	0.87	1	17.75	21.08	17.21	18.68	20.86	19.26	17.28	19.13	1.02	1	16.72	17.66	12.84	15.74	11.9	14.46	12.96	13.11	0.83	1	16.09	16.91	13.43	15.48	14.21	16.73	15.47	1	1
Solyc01g010820.2.1	Cullin C (AHRD V1 ***- D3BH	9.96	12.3	18.32	13.84	13.61	10.58	9.35	9.82	10.37	10.03	0.74	0.615993	14.86	13.03	13.95	13.95	12.39	12.32	11.71	12.14	0.87	1	14.07	14.26	16.48	14.94	12.56	11.91	12.17	12.21	0.82	1	13.27	16.23	13.11	14.2	12.82	14.09	13.46	0.95	1
Solyc01g010830.2.1	HAD-superfamily hydrolase (	18.59	26.98	20.74	27.02	23.33	31.99	32.58	33.02	26.88	31.12	1.33	0.431003	25.83	24.13	21.94	23.97	28.27	27.83	29.03	28.38	1.18	0.844156	31.49	36.12	41.72	36.44	32.13	32.82	38.79	34.58	0.95	1	18.47	29.6	22.33	23.47	35.44	30.37	32.91	1.4	1
Solyc01g010840.1.1	Unknown Protein (AHRD V1)	0	0	0	0	0	0	0	0	0	0	1	NA	0	0	0	0	0	0	0	0	1	NA	0	0	0	0	0	0	0	0	1	NA	0	0	0	0	0	0	0	1	NA
Solyc01g010850.1.1	Unknown Protein (AHRD V1)	0	0	0	0	0	0	0	0	0	0	1	NA	0	0	0	0	0	0	0	0	1	NA	0	0	0	0	0	0	0	0	1	NA	0	0	0	0	0	0	0	1	NA
Solyc01g010860.2.1	Sphingosine hydroxylase (AH	6.92	9.24	12.83	6.29	8.82	14.56	8.39	13.86	12.95	12.44	1.41	0.51281	9.42	10.09	6.89	8.8	9.77	12.15	13.59	11.84	1.35	0.672688	12.38	10.67	10.55	11.2	8.28	8.24	7.92	8.15	0.73	1	17.21	15.97	18.62	17.27	12.21	8.23	10.22	0.59	0.868202
Solyc01g010870.2.1	Dehydration-responsive famil	15.73	19.6	24.45	20.26	20.01	16.89	14.39	20.25	18.54	17.52	0.88	1	31.29	33.96	36.57	33.94	36.65	37.53	32.23	35.47	1.05	1	136.93	103.62	114.16	118.24	106.49	103.37	113.86	107.91	0.91	1	59.09	97.56	91.94	82.86	66.15	73.92	70.03	0.85	1
Solyc01g010880.2.1	E3 ubiquitin-protein ligase M	10.17	8.47	9.44	12.24	10.08	5.05	7.34	5.77	7.92	6.52	0.65	0.563568	8.85	8.27	10.41	9.18	11.25	10.75	10.03	10.68	1.16	0.96542	6.29	7.76	7.51	7.19	6.39	6.23	6.46	6.36	0.88	1	8.3	12.1	12.31	10.9	7.72	9.1	8.41	0.77	1
Solyc01g010890.2.1	Expansin 2 (AHRD V1 ***- B1	0	0	0	0	0	0	0	0.23	0	0.06	5.75	1	0	0	0	0	0	0	0	0	1	NA	0	0	0	0	0	0	0	0	1	NA	0	0	0	0	0	0	0	1	NA
Solyc01g010900.2.1	Cytochrome P450	0	0	0	0	0	0	0	0	0	0	1	NA	0	0	0	0	0	0	0	0	1	NA	0	0	0	0	0	0	0	0	1	NA	0	0	0	0	0	0	0	1	NA
Solyc01g010910.1.1	MYB transcription factor (AH	0	0	0	0	0	0	0	0	0	0	1	NA	0	0	0	0	0	0	0	0	1	NA	0	0	0	0	0	0	0	0	1	NA	0	0	0	0	0	0	0	1	NA
Solyc01g010920.1.1	Os03g0169000 protein (Frag	3.09	3.43	5.01	2.73	3.56	4.29	2.44	3.55	1.38	2.92	0.82	1	1.08	2.01	1.63	1.57	1.34	1	0.91	1.08	0.69	1	1.56	2.51	0.77	1.61	0.76	0.29	0.56	0.54	0.33	1	1.65	0.56	0	0.74	0	0	0	0.01	1
Solyc01g010930.2.1	Os03g0169000 protein (Frag	1.48	2.19	3.19	0.7	1.89	2.74	0.97	4.53	2.3	2.63	1.39	0.964813	3.43	1.07	3.53	2.68	1.88	1.02	1.16	1.35	0.51	0.676496	0.8	1.2	1.47	1.16	0.49	0.56	1.08	0.71	0.61	1	0	0.54	0	0.18	0.45	0	0.23	1.25	1
Solyc01g010940.2.1	Transmembrane emp24 dom	35.27	35.99	33.36	30.96	33.9	35.82	38.4	46.68	37.98	39.72	1.17	0.807767	34.22	35.41	26.45	32.03	26.78	28.81	38.47	31.35	0.98	1	68.53	71.35	66.99	68.96	39.74	53.69	85.74	59.72	0.87	1	19.88	36.21	28.98	28.36	40.63	25.06	32.84	1.16	1
Solyc01g010950.2.1	Kinase family protein (AHRD	11.17	15.12	10.65	12.34	12.32	15.82	15.71	15.48	15.27	15.57	1.26	0.635547	15.09	14.5	12.41	14	15.61	11.81	14.8	14.07	1.01	1	17.31	17.84	12.91	16.02	15.38	17.98	15.95	16.44	1.03	1	28.73	21.46	21.65	23.95	18.5	15.3	16.9	0.71	1
Solyc01g010960.1.1	60S ribosomal protein L35 (A	0	0.57	0	0	0.14	0	0.51	0	0	0.13	0.89	1	0	0	0.54	0.18	0	0	0.5	0.17	0.93	1	0.52	0	0	0.17	0	0	0	0	0.06	1	0	0	0	0	0	0	0	1	NA

Solyc01g010970.2.1	ARGONAUTE 1 (AHRD V1 ***	0	0.18	0	0.09	0.07	0	0.05	0	0.09	0.04	0.52	1	0.12	0.06	0.06	0.08	0.37	0.31	0.16	0.28	3.5	0.592175	0.21	0.21	0.07	0.16	0.13	0.1	0.1	0.11	0.67	1	0.19	0.38	0.2	0.26	0.42	0.17	0.29	1.15	1
Solyc01g010980.1.1	Unknown Protein (AHRD V1)	0	0	0	0	0	0	0	0	0	0	1	NA	0	0	0	0	0	0	0	0	1	NA	0	0	0	0	0	0	0	0	1	NA	0	0	0	0	0	0	0	1	NA
Solyc01g010990.2.1	Unknown Protein (AHRD V1)	5.26	7.9	4.28	5.13	5.64	7.86	5.58	9.66	6.38	7.37	1.31	0.799849	4.72	7.09	6.89	6.23	3.77	5.44	10.54	6.58	1.06	1	10.4	6.35	4.68	7.14	5.24	4.53	7.4	5.72	0.8	1	2.83	4.34	5.85	4.34	5.81	3.97	4.89	1.13	1
Solyc01g011000.2.1	Eukaryotic translation initiati	345.11	323.29	345.87	264.2	319.62	324.04	265.3	278.64	355.51	305.87	0.96	1	335.59	307.88	265.02	302.83	352.61	315.1	360	342.57	1.13	0.890701	371.76	365.55	314.31	350.54	393.3	523.45	437.17	451.31	1.29	1	334.15	241.37	219.38	264.97	490.67	519.5	505.09	1.91	0.226491
Solyc01g011010.2.1	Unknown Protein (AHRD V1)	3.47	12.36	8.19	2.46	6.62	11.58	5.48	20.75	13.71	12.88	1.95	0.737127	9.69	10.56	8.8	9.68	9.63	12.58	31.38	17.86	1.84	0.85658	7.01	7.07	6.89	6.99	1.14	7.89	5.06	4.7	0.67	1	17.28	13.87	24.03	18.39	11.12	10.66	10.89	0.59	1
Solyc01g011020.1.1	Unknown Protein (AHRD V1)	0	0	0	0	0	0	0	0	0	0	1	NA	0	0	0	0	0	0	0	0	1	NA	0	0	0	0	0	0	0	0	1	NA	0	0	0	0	0	0	0	1	NA
Solyc01g011030.2.1	RING zinc finger protein-like (	23.39	23.88	30.78	34.12	28.04	30.1	22.54	25.8	24.39	25.71	0.92	1	26.56	28.89	36.38	30.61	46.44	36.96	35.21	39.54	1.29	0.568519	36.79	49.14	40.32	42.08	40.27	31.34	35.16	35.59	0.85	1	28	23.96	19.68	23.88	32	39.07	35.53	1.49	0.916983
Solyc01g011040.2.1	LRR receptor-like serine/thre	61.4	68.97	37.98	52.28	55.16	52.35	68.24	60.11	61.49	60.55	1.1	0.959699	70.79	65.64	37.26	57.9	35.28	40.12	58.56	44.65	0.77	0.940618	70.64	66.39	44.58	60.54	39.03	55.72	51.11	48.62	0.8	1	28.53	42.29	51.33	40.72	42.56	48.61	45.59	1.12	1
Solyc01g011050.2.1	LRR receptor-like serine/thre	0	0	0.44	0	0.11	0	0	0	0	0	0.09	1	0	0	0	0	0	0	0	0	1	NA	0	0	0	0	0	0	0	0	1	NA	0	0	0	0	0	0	0	1	NA
Solyc01g011060.1.1	Maturase (Fragment) (AHRD	0	0.66	0	0	0.17	0	0	0	0	0	0.06	1	0	0	0	0	0.51	0	0	0.17	17	1	0	0	0	0	0	0	0	0	1	NA	0	0	0	0	0	0	0	1	NA
Solyc01g011070.1.1	Unknown Protein (AHRD V1)	0	0	0	0	0	0	0	0	0	0	1	NA	0	0	0	0	0	0	0	0	1	NA	0	0	0	0	0	0	0	0	1	NA	0	0	0	0	0	0	0	1	NA
Solyc01g011080.2.1	Unknown Protein (AHRD V1)	0	0	0	0	0	0	0	1.08	0	0.27	27	1	0	0	0	0	0	0	0.93	0.31	31	1	0	0	0	0	0	0	0	0	1	NA	0	0	0	0	0	0	0	1	NA
Solyc01g011090.2.1	Phospholipid-transporting AT	15.57	14.56	11.21	17.54	14.72	7.92	16.87	11.89	14.97	12.91	0.88	1	15.95	17.89	14.49	16.11	12.81	17.22	14.72	14.92	0.93	1	19.4	14.62	17.55	17.19	20.46	17	14.23	17.23	1	1	31.14	23.19	27.59	27.31	24.64	28.92	26.78	0.98	1
Solyc01g011100.2.1	Phospholipid-transporting AT	13.51	15.76	13.38	16.97	14.9	11.88	17.65	14.29	14.31	14.53	0.98	1	14.21	17.67	16.23	16.04	14.01	16.13	16.54	15.56	0.97	1	17.22	15.87	17.77	16.95	15.42	15.76	15.67	15.62	0.92	1	29.37	29.77	32.27	30.47	23.23	27.31	25.27	0.83	1
Solyc01g011110.1.1	Unknown Protein (AHRD V1)	0	0	0	0	0	0	0	0	0	0	1	NA	0	0	0	0	0	0	0	0	1	NA	0	0	0	0	0	0	0	0	1	NA	0	0	0	0	0	0	0	1	NA
Solyc01g011130.1.1	Ulp1 protease family C-termi	0	0	0	0	0	0	0	0	0.08	0.02	2	1	0	0	0	0	0	0	0	0	1	NA	0	0	0	0	0	0	0	0	1	NA	0	0	0	0	0	0	0	1	NA
Solyc01g011150.1.1	Unknown Protein (AHRD V1);	0	0	0	0	0	0	0	0	0	0	1	NA	0	0	0	0	0	0	0	0	1	NA	0	0	0	0	0	0	0	0	1	NA	0	0	0	0	0	0	0	1	NA
Solyc01g011170.1.1	Unknown Protein (AHRD V1)	0	0	0	0	0	0	0	0	0	0	1	NA	0	0	0	0	0	0	0	0	1	NA	0	0	0	0	0	0	0	0	1	NA	0	0	0	0	0	0	0	1	NA
Solyc01g011180.1.1	Unknown Protein (AHRD V1)	3.09	0.92	3.65	4.37	3.01	0	1.63	2.84	0.74	1.3	0.43	0.95184	2.88	2.69	4.35	3.31	2.86	2.13	3.24	2.74	0.83	1	3.33	2.52	2.05	2.63	1.35	0.78	0	0.71	0.27	1	1.47	0	0	0.49	1.89	1.81	1.85	3.78	1
Solyc01g011190.1.1	Unknown Protein (AHRD V1)	2.06	2.75	0	2.92	1.93	3.44	1.63	0.95	0	1.51	0.78	1	2.88	1.79	2.61	2.43	2.14	1.6	0.81	1.52	0.63	1	0	0	0	0	0	3.13	1.5	1.54	154.33	1	0	0.75	0	0.25	0	0.9	0.45	1.8	1
Solyc01g011200.2.1	Aspartyl protease family prot	0.35	1.55	1.24	0	0.78	2.72	0.28	1.6	1.75	1.59	2.02	0.90496	1.3	0.3	0.29	0.63	0	0.72	1.37	0.7	1.11	1	0.56	1.71	0.35	0.87	0	0.79	0	0.26	0.3	1	0	1.01	0.54	0.52	0.64	0.61	0.62	1.21	1
Solyc01g011220.1.1	Aspartyl protease family prot	0.9	0.53	0.35	0.84	0.66	0.33	0.71	1.37	0.21	0.66	1	1	1.11	0.26	0.5	0.62	1.45	0.93	0	0.79	1.27	1	0	0.73	0	0.24	0.2	0	0.44	0.21	0.88	1	0.42	0.87	0.46	0.58	1.09	0.79	0.94	1.61	1
Solyc01g011230.2.1	Aspartyl protease family prot	0.69	2.45	0	0.97	1.03	0.76	0	0	0.99	0.44	0.43	0.995198	0.64	0	0.58	0.41	0	0.71	0	0.24	0.58	1	0.56	0.56	0	0.37	0.9	0	0.5	0.47	1.25	1	0.98	1.5	0	0.83	2.52	0	1.26	1.52	1
Solyc01g011240.1.1	Unknown Protein (AHRD V1)	0	0	0	0	0	0	0	0	0	0	1	NA	0	0	0	0	0	0	0	0	1	NA	0	0	0	0	0	0	0	0	1	NA	0	0	0	0	0	0	0	1	NA
Solyc01g011250.1.1	Unknown Protein (AHRD V1);	0	0	0	0	0	0	0	0	0	0	1	NA	0	0	0	0	0	0	0	0	1	NA	0	0	0	0	0	0	0	0	1	NA	0	0	0	0	0	0	0	1	NA
Solyc01g011260.1.1	Aspartyl protease family prot	0	0	0	0	0	0	0	0	0	0	1	NA	0	0	0	0	0	0	0	0	1	NA	0	0	0	0	0	0	0	0	1	NA	0	0	0	0	0	0	0	1	NA
Solyc01g011270.1.1	Aspartyl protease family prot	0	0	0	0.83	0.21	0	0	0	0	0	0.05	1	0	0	0	0	0.4	0	0	0.13	13.33	1	0	0	0	0	0	0	0	0	1	NA	0	0	0	0	0	0.51	0.26	25.5	1
Solyc01g011280.1.1	Unknown Protein (AHRD V1)	0	0	0	0	0	0	0	0	0	0	1	NA	0	0	0	0	0	0	0	0	1	NA	0	0	0	0	0	0	0	0	1	NA	0	0	0	0	0	0	0	1	NA
Solyc01g011290.1.1	HAT family dimerisation dom	0	0	0	0	0	0	0	0	0	0	1	NA	0	0	0	0	0	0	0	0	1	NA	0	0	0	0	0	0	0	0	1	NA	0	0	0	0	0	0	0	1	NA
Solyc01g011300.1.1	Aspartyl protease family prot	0	0	0	0	0	0	0	0	0	0	1	NA	0	0	0	0	0	0	0	0	1	NA	0	0	0	0	0	0	0	0	1	NA	0	0	0	0	0	0	0	1	NA
Solyc01g011310.1.1	Aspartyl protease family prot	0	0	0	0	0	0	0.19	0	0.17	0.09	9	1	0	0	0	0	0	0	0	0	1	NA	0	0	0	0	0	0	0	0	1	NA	0	0	0.37	0.12	0	0.21	0.1	0.85	1
Solyc01g011320.1.1	Aspartyl protease family prot	0	0	0	0	0	0	0.36	0	0.33	0.17	17.25	1	0	0	0	0	0	0	0	0	1	NA	0	0	0	0	0	0	0	0	1	NA	0	0	0.71	0.24	0	0.4	0.2	0.85	1
Solyc01g011330.1.1	Aspartyl protease family prot	0	0	0	0	0	0	0	0	0	0	1	NA	0	0	0	0	0	0	0	0	1	NA	0	0	0	0	0	0	0	0	1	1	0	0	0	0	0	0	0	1	NA
Solyc01g011340.2.1	Serine/threonine-protein pho	61.03	72.87	63.13	71.43	67.12	61.39	84.32	69.74	63.25	69.67	1.04	1	74.56	82	72.78	76.45	51.57	56.41	68.06	58.68	0.77	0.657415	80.03	86.5	69.96	78.83	71.01	66.94	72.35	70.1	0.89	1	50.41	82.1	80.63	71.05	57.05	62.18	59.61	0.84	1
Solyc01g011350.2.1	cDNA clone J013057D02 full	228.31	252.51	196.87	193.34	217.76	318.73	365.09	301.89	307.86	323.39	1.49	0.084244	235.03	213.46	199.76	216.08	175.4	201.82	240.25	205.82	0.95	1	58.52	62.72	51.36	57.53	71.95	61.77	57.5	63.74	1.11	1	139.78	120.1	120.34	126.74	75.19	84.68	79.94	0.63	0.769881
Solyc01g011370.1.1	Unknown Protein (AHRD V1)	0	0	0	0	0	0	0	1.12	0.44	0.39	39	1	0	0	0	0	0.42	0	0	0.14	14	1	0	0	0	0	0.4	0	0	0.13	13.33	1	0	0	0	0	0	0	0	1	NA
Solyc01g011390.2.1	Unknown Protein (AHRD V1)	0	0	0	0	0	0	0	0	0	0	1	NA	0	0	0	0	0	0	0	0	1	NA	0	0	0	0	0	0	0	0	1	NA	0	0	0	0	0	0	0	1	NA
Solyc01g011400.1.1	Unknown Protein (AHRD V1)	0	0	0	0	0	0	0	0	0	0	1	NA	0	0	0	0	0	0	0	0	1	NA	0	0	0	0	0	0	0	0	1	NA	0	0	0	0	0	0	0	1	NA
Solyc01g011410.1.1	NADH-quinone oxidoreducta	0	0	0	1.26	0.32	0	0	0	0	0	0.03	1	0	0	0.75	0.25	0.62	0	0.7	0.44	1.76	1	0	2.18	0.88	1.02	0	0.68	0	0.23	0.22	1	3.8	0.65	5.48	3.31	0.82	0.78	0.8	0.24	1
Solyc01g011420.1.1	Unknown Protein (AHRD V1)	5.5	2.45	3.24	0	2.8	0	0	10.11	1.97	3.02	1.08	1	5.11	14.34	2.32	7.26	3.81	4.27	0	2.69	0.37	0.974398	2.22	0	5.46	2.56	1.81	0	8.01	3.27	1.28	1	0	9.98	0	3.33	5.03	2.41	3.72	1.12	1
Solyc01g011430.2.1	Diacylglycerol O-acyltransfera	0.13	0.46	0.15	0.18	0.23	0.14	0.21	0	0.19	0.14	0.59	1	0.48	0.45	0.22	0.38	0.09	0.07	0.1	0.09	0.23	0.714654	0.53	0.32	0	0.28	0	0.1	0.09	0.06	0.22	1	0	0.28	0	0.09	0	0	0	0.11	1
Solyc01g011450.1.1	Unknown Protein (AHRD V1)	0	0	0	0	0	0	0	0	0	0	1	NA	0.42	0	0	0.14	0	0	0	0	0.07	1	0	0	0	0	0.3	0	0	0.1	10	1	0	0	0	0	0	0.4	0.2	20	1
Solyc01g011460.1.1	Unknown Protein (AHRD V1)	0	0	0	0.62	0.15	0	0.69	0	0	0.17	1.11	1	0	0	0	0	0	0	0	0	1	NA	0	0.36	0	0.12	0	0	0	0	0.08	1	0	0	0	0	0	0	0	1	NA
Solyc01g011470.1.1	Major latex-like protein (AHR	0	0	0	0	0	0	0	0	0	0	1	NA	0	0	0	0	0	0	0	0	1	NA	0	0	0	0	0	0	0	0	1	NA	0	0	0	0	0	0	0	1	NA
Solyc01g011480.1.1	Photosystem II CP43 chloroph	0	0	0	0	0	0	0	0	0.55	0.14	13.75	1	0	0	0	0	0	0	0	0	1	NA	0	0	0	0	0	0	0	0	1	NA	0	0	0	0	0	0	0	1	NA
Solyc01g011490.1.1	O-acyltransferase WSD1 (AH	0	0	0	0	0	0	0	0	0	0	1	NA	0	0	0	0	0	0	0	0	1	NA	0	0	0	0	0	0	0	0	1	NA	0	0	0	0	0	0	0	1	NA
Solyc01g011500.1.1	Unknown Protein (AHRD V1)	0	0	0	0	0	0	0	0	0	0	1	NA	0	0	0	0	0	0	0	0	1	NA	0	0	0	0	0	0	0	0	1	NA	0	0	0	0	0	0	0	1	NA
Solyc01g011510.2.1	Aldehyde dehydrogenase (AH	16.2	18.99	9.48	5.59	12.57	17.56	8.94	20.58	16.64	15.93	1.27	0.980439	11.39	8.81	5.56	8.59	4.66	7.36	8.49	6.84	0.8	1	11.38	7.3	10.85	9.84	3.29	2.79	3.17	3.08	0.31	0.765013	5.8	18.94	16.2	13.65	8.2	5.43	6.81	0.5	0.823424
Solyc01g011520.1.1	Dicer-like protein (AHRD V1 *	0	0	0	0	0	0	0	0	0	0	1	NA	0	0	0	0	0	0	0	0	1	NA	0	0	0	0	0	0	0	0	1	NA	0	0	0	0	0	0	0	1	NA
Solyc01g011530.1.1	Unknown Protein (AHRD V1)	0	0	0	0	0	0	0	0	0	0	1	NA	0	0	0	0	0	0	0	0	1	NA	0	0	0	0	0	0	0	0	1	NA	0	0	0	0	0	0	0	1	NA
Solyc01g011540.1.1	Dicer-like protein (AHRD V1 *	0	0	0	0	0	0	0	0	0	0	1	NA	0	0	0	0	0	0	0	0	1	NA	0	0	0	0	0	0	0	0	1	NA	0	0	0	0	0	0	0	1	NA
Solyc01g012540.1.1	Dicer-like protein (AHRD V1 *	0	0	0	0	0	0	0	0	0	0	1	NA	0	0	0	0	0	0	0	0	1	NA	0	0	0	0	0	0	0	0	1	NA	0	0	0	0	0	0	0	1	NA
Solyc01g012550.1.1	Endoribonuclease Dicer (AHR	0	0	0	0	0	0	0	0	0	0	1	NA	0	0	0	0	0	0	0	0	1	NA	0	0	0	0	0	0	0	0	1	NA	0	0	0	0	0	0	0	1	NA
Solyc01g012560.1.1	Unknown Protein (AHRD V1)	0	0	0	0	0	0	0	0	0	0	1	NA	0	0	0	0	0	0	0	0	1	NA	0	0	0	0	0	0	0	0	1	NA	0	0	0	0	0	0	0	1	NA
Solyc01g012570.1.1	Dicer-like protein (AHRD V1 *	0	0	0	0	0	0	0	0	0	0	1	NA	0	0	0	0	0	0	0	0	1	NA	0	0	0	0	0	0	0	0	1	NA	0	0	0	0	0	0	0	1	NA
Solyc01g012580.1.1	Endoribonuclease Dicer homo	0	0	0	0	0	0	0	0	0	0	1	NA	0	0	0	0	0	0	0	0	1	NA	0	0	0	0	0	0	0	0	1	NA	0	0	0	0	0	0	0	1	NA
Solyc01g012590.1.1	Dicer-like protein (AHRD V1 *	0	0	0	0	0	0	0	0	0	0	1	NA	0	0	0	0	0	0	0	0	1	NA	0	0	0	0	0	0	0	0	1	NA	0	0	0	0	0	0	0	1	NA
Solyc01g012600.1.1	Dicer-like protein (AHRD V1 *	0	0	0	0	0	0	0	0	0	0	1	NA	0	0	0	0	0	0	0	0	1	NA	0	0	0	0	0	0	0	0	1	NA	0	0	0	0	0	0	0	1	NA
Solyc01g012610.1.1	Dicer-like protein (AHRD V1 *	0	0	0	0	0	0	0	0	0	0	1	NA	0	0	0	0	0	0	0	0	1	NA	0	0	0	0	0	0	0	0	1	NA	0	0	0	0	0	0	0	1	NA
Solyc01g012620.1.1	Ulp1 protease family C-termi	0	0	0	0	0	0	0	0	0	0	1	NA	0	0	0	0	0.73	0	0	0.24	24.33	1	0	0	0	0	0	0	1.54	0.51	51.33	1	0	0	0	0	0.48	0	0.24	24	1
Solyc01g012630.1.1	Dicer-like protein (AHRD V1 *	0	0	0	0	0	0	0	0	0	0	1	NA	0	0	0	0	0	0	0	0	1	NA	0	0	0	0	0	0	0	0	1	NA	0	0	0	0	0	0	0	1	NA
Solyc01g012640.1.1	Dicer-like protein (AHRD V1 *	0	0	0	0	0	0	0	0	0	0	1	NA	0	0	0	0	0	0	0	0	1	NA	0	0	0	0	0	0	0	0	1	NA	0	0	0	0	0	0	0	1	NA
Solyc01g012650.1.1	Dicer-like protein 2 (AHRD V1	0	0	0	0	0	0	0	0	0	0	1	NA	0	0	0	0	0	0	0	0	1	NA	0	0	0	0	0	0	0	0	1	NA	0	0	0	0	0	0	0	1	NA
Solyc01g012660.1.1	Dicer-like protein (AHRD V1 *	0	0	0	0	0	0	0	0	0	0	1	NA	0	0	0	0	0	0	0	0	1	NA	0	0	0	0	0	0	0	0	1	NA	0	0	0	0	0	0	0	1	NA
Solyc01g012670.1.1	Dicer-like protein (AHRD V1 *	0	0	0	0	0	0	0	0	0	0	1	NA	0	0	0	0	0	0	0	0	1	NA	0	0	0	0	0	0	0	0	1	NA	0	0	0	0	0	0	0	1	NA
Solyc01g012680.1.1	Endoribonuclease Dicer homo	0	0	0	0	0	0	0	0	0	0	1	NA	0	0	0	0	0	0	0	0	1	NA	0	0	0	0	0	0	0	0	1	NA	0	0	0	0	0	0	0	1	NA
Solyc01g012690.1.1	LRR receptor-like serine/thre	0	0	0	0	0	0	0	0	0	0	1	NA	0	0	0	0	0	0	0	0	1	NA	0	0	0	0	0	0	0	0	1	NA	0	0	0	0	0	0	0	1	NA
Solyc01g012700.2.1	Aldehyde dehydrogenase (AH	1.82	2.54	0.61	0	1.24	1.44	0.62	2.39	1.12	1.39	1.12	1	0.72	1.35	0.22	0.76	0.18	0.67	0.82	0.56	0.73	1	1.26	0.63	0.77	0.89	0.17	0.2	0	0.12	0.14	1	0	1.89	0.8	0.9	0.71	0.23	0.47	0.52	1
Solyc01g012710.1.1	Dicer-like protein (AHRD V1 *	0	0	0	0	0	0	0	0	0	0	1	NA	0	0	0	0	0	0	0	0	1	NA	0	0	0	0	0	0	0	0	1	NA	0	0	0	0	0	0	0	1	NA
Solyc01g012720.1.1	Dicer-like protein (AHRD V1 *	0	0	0	0	0	0	0	0	0	0	1	NA	0	0	0	0	0	0	0	0	1	NA	0	0	0	0	0	0	0	0	1	NA	0	0	0	0	0	0	0	1	NA
Solyc01g012730.1.1	Dicel-like 2 (Fragment) (AHRD	0	0	0	0	0	0	0	0	0	0	1	NA	0	0	0	0	0	0	0	0	1	NA	0	0	0	0	0	0	0	0	1	NA	0	0	0	0	0	0	0	1	NA
Solyc01g012740.1.1	Dicer-like protein (AHRD V1 *	0	0	0	0	0	0	0	0	0	0	1	NA	0	0	0	0	0	0	0	0	1	NA	0	0	0	0	0	0	0	0	1	NA	0	0	0	0	0	0	0	1	NA
Solyc01g012750.1.1	Aldehyde dehydrogenase fam	8.02	8.78	0.73	0.87	4.6	3.43	3.89	3.97	6.2	4.37	0.95	1	5.74	2.68	1.56	3.33	2.99	3.19	1.94	2.71	0.81	1	7.47	5.02	1.84	4.78	1.22	1.4	1.35	1.32	0.28	1	1.75	11.2	12.33	8.43	2.26	3.78	3.02	0.36	0.930886
Solyc01g012760.1.1	Aldehyde dehydrogenase (AH	0.32	0.56	0	0	0.22	0.35	0	0.87	0.91	0.53	2.42	1	0	0.28	0.27	0.18	0.22	0	1	0.41	2.22	1	0.77	0.26	0.31	0.45	0	0	0.46	0.15	0.34	1	0.9	0	0	0.3	0.29	0	0.14	0.48	1
Solyc01g013760.1.1	Unknown Protein (AHRD V1)	0	0	0	0	0	0	0	0	0	0	1	NA	0	0	0	0	0	0	0	0	1	NA	0	0	0	0	0	0	0	0	1	NA	0	0	0	0	0	0	0	1	NA
Solyc01g013770.1.1	Unknown Protein (AHRD V1)	0	0	0	0	0	0	0	0	0	0	1	NA	0	0	0	0	0	0	0	0	1	NA	0	0	0	0	0	0	0	0	1	NA	0	0	0	0	0	0	0	1	NA
Solyc01g013780.1.1	Dicer-like protein (AHRD V1 *	0	0	0	0	0	0	0	0	0	0	1	NA	0	0	0	0	0	0	0	0	1	NA	0	0	0	0	0	0	0	0	1	NA	0	0	0	0	0	0	0	1	NA
Solyc01g013790.1.1	Dicer-like protein (AHRD V1 *	0	0	0	0	0	0	0	0	0	0	1	NA	0	0	0	0	0	0	0	0	1	NA	0	0	0	0	0	0	0	0	1	NA	0	0	0	0	0	0	0	1	NA
Solyc01g013800.1.1	Dicer-like protein (AHRD V1 *	0	0	0	0	0	0	0	0	0	0	1	NA	0	0	0	0	0	0	0	0	1	NA	0	0	0	0	0	0	0	0	1	NA	0	0	0	0	0	0	0	1	NA
Solyc01g013810.1.1	Dicel-like 2 (Fragment) (AHRD	0	0	0	0	0	0	0	0	0	0	1	NA	0	0	0	0	0	0	0	0	1	NA	0	0	0	0	0	0	0	0	1	NA	0	0	0	0	0	0	0	1	NA
Solyc01g013820.1.1	Unknown Protein (AHRD V1)	0	0	0	0	0	0	0	0	0	0	1	NA	0	0	0	0	0	0	0	0	1	NA	0	0	0	0	0	0	0	0	1	NA	0	0	0	0	0	0	0	1	NA
Solyc01g013830.1.1	Dicer-like protein (AHRD V1 *	0	0	0	0	0	0	0	0	0	0	1	NA	0	0	0	0	0	0	0	0	1	NA	0	0	0	0	0	0	0	0	1	NA	0	0	0	0	0	0	0	1	NA
Solyc01g013840.1.1	Dicer-like protein (AHRD V1 *	0	0	0	0	0	0	0	0	0	0	1	NA	0	0	0	0	0	0	0	0	1	NA	0	0	0	0	0	0	0	0	1	NA	0	0	0	0	0	0	0	1	NA
Solyc01g013850.1.1	Dicer-like protein (AHRD V1 *	0	0	0	0	0	0	0	0	0	0	1	NA	0	0	0	0	0	0	0	0	1	NA	0	0	0	0	0	0	0	0	1	NA	0	0	0	0	0	0	0	1	NA
Solyc01g013860.1.1	Dicer-like protein (AHRD V1 *	0	0	0	0	0	0	0	0	0	0	1	NA	0	0	0	0	0	0	0	0	1	NA	0	0	0	0	0	0	0	0	1	NA	0	0	0	0	0	0	0	1	NA
Solyc01g013870.1.1	Hcr9-4E (AHRD V1 ***- O500	0	0	0	0	0	0	0	0	0	0	1	NA	0	0	0	0	0	0	0	0	1	NA	0	0	0	0	0	0	0	0	1	NA	0	0	0	0	0	0	0	1	NA
Solyc01g013880.1.1	LRR receptor-like serine/thre	0	0	0	0	0	0	0	0	0	0	1	NA	0	0	0	0	0	0	0	0	1	NA	0	0	0	0	0	0	0	0	1	NA	0	0	0	0	0	0	0	1	NA
Solyc01g013890.1.1	Aldehyde dehydrogenase (AH	4.27	6.97	1.68	1.34	3.56	3.17	2.25	3.27	5.45	3.54	0.99	1	4.19	2.68	1.2	2.69	1.81	1.84	2.98	2.21	0.82	1	3.64	2.51	2.59	2.91	0.78	0.54	1.73	1.02	0.35	1	1.01	5.17	7.66	4.61	1.96	2.5	2.23	0.48	1
Solyc01g013900.2.1	Aldehyde dehydrogenase (AH	3.22	4.3	3.8	4.55	3.97	1.79	3.81	5.92	9.82	5.33	1.34	0.931714	3.74	2.1	0.68	2.17	1.67	2.08	1.26	1.67	0.77	1	1.3	3.28	3.99	2.86	0	0	0.59	0.2	0.07	1	1.14	8.76	6.19	5.36	1.47	0	0.73	0.14	0.620606
Solyc01g013910.1.1	Unknown Protein (AHRD V1)	0	0	0	0	0	0	0	0	0	0	1	NA	0	0	0	0	0	0	0	0	1	NA	0	0	0	0	0	0	0	0	1	NA	0	0	0	0	0	0	0	1	NA
Solyc01g013920.1.1	Endoribonuclease Dicer homo	0	0	0	0	0	0	0	0	0	0	1	NA	0	0	0	0	0	0	0	0	1	NA	0	0	0	0	0	0	0	0	1	NA	0	0	0	0	0	0	0	1	NA
Solyc01g013930.1.1	Dicer-like protein (AHRD V1 *	0	0	0	0	0	0	0	0	0	0	1	NA	0	0	0	0	0	0	0	0	1	NA	0	0	0	0	0	0	0	0	1	NA	0	0	0	0	0	0	0	1	NA
Solyc01g013940.1.1	Dicel-like 2 (Fragment) (AHRD	0	0	0	0	0	0	0	0	0	0	1	NA	0	0	0	0	0	0	0	0	1	NA	0	0	0	0	0	0	0	0	1	NA	0	0	0	0	0	0	0	1	NA
Solyc01g013950.1.1	Dicer-like protein (AHRD V1 *	0	0	0	0	0	0	0	0	0	0	1	NA	0	0	0	0	0	0	0	0	1	NA	0	0	0	0	0	0	0	0	1	NA	0	0	0	0	0	0	0	1	NA
Solyc01g013960.1.1	LRR receptor-like serine/thre	0	0	0	0	0	0	0	0	0	0	1	NA	0	0	0	0	0	0	0	0	1	NA	0	0	0	0	0	0	0	0	1	NA	0	0	0	0	0	0	0	1	NA
Solyc01g013970.1.1	Unknown Protein (AHRD V1)	0	0	0	0	0	0	0	0	0	0	1	NA	0	0	0	0	0	0	0	0	1	NA	0	0	0	0	0	0	0	0	1	NA	0	0	0	0	0	0	0	1	NA
Solyc01g013980.1.1	Vacuolar protein sorting-asso	0	0	0	0	0	0	0	0	0	0	1	NA	0	0	0	0	0	0	0	0	1	NA	0	0	0	0	0	0	0	0	1	NA	0	0	0	0	0	0	0	1	NA
Solyc01g013990.1.1	Unknown Protein (AHRD V1)	0	0	0	0	0	0	0	0	0	0	1	NA	0	0	0	0	0	0	0	0	1	NA	0	0	0	0	0	0	0	0	1	NA	0	0	0	0	0	0	0	1	NA
Solyc01g014000.1.1	Unknown Protein (AHRD V1)	0	0	0	0	0	0	0	0	0	0	1	NA	0	0	0	0	0	0	0	0	1	NA	0	0	0	0	0	0	0	0	1	NA	0	0	0	0	0	0	0	1	NA
Solyc01g014010.1.1	Unknown Protein (AHRD V1)	0	0	0	0	0	0	0	0	0	0	1	NA	0	0	0	0	0	0	0	0	1	NA	0	0	0	0	0	0	0	0	1	NA	0	0	0	0	0	0	0	1	NA
Solyc01g014020.1.1	LRR receptor-like serine/thre	0	0	0	0	0	0	0	0	0	0	1	NA	0	0	0	0	0	0	0	0	1	NA	0	0	0	0	0	0	0	0	1	NA	0	0	0	0	0	0	0	1	NA
Solyc01g014030.1.1	Unknown Protein (AHRD V1)	0	0	0	0	0	0	0	0	0	0	1	NA	0	0	0	0	0	0	0	0	1	NA	0	0	0	0	0	0	0	0	1	NA	0	0	0	0	0	0	0	1	NA
Solyc01g014040.1.1	Unknown Protein (AHRD V1)	0	0	0	0	0	0	0	0	0	0	1	NA	0	0	0	0	0	0	0	0	1	NA	0	0	0	0	0	0	0	0	1	NA	0	0	0	0	0	0	0	1	NA
Solyc01g014050.1.1	Unknown Protein (AHRD V1)	0	0	0	0	0	0	0	0	0	0	1	NA	0	0	0	0	0	0	0	0	1	NA	0	0	0	0	0	0	0	0	1	NA	0	0	0	0	0	0	0	1	NA
Solyc01g014060.1.1	Blue copper protein (AHRD V	0	0	0	0	0	0	0	0	0	0	1	NA	0	0	0	0	0	0	0	0	1	NA	0	0	0	0	0	0	0	0	1	NA	0	0	0	0	0	0	0	1	NA
Solyc01g014070.1.1	Blue copper protein (AHRD V	0	0	0	0	0	0	0	0	0	0	1	NA	0	0	0	0	0	0	0	0	1	NA	0	0	0	0	0	0	0	0	1	NA	0	0	0	0	0	0	0	1	NA

Solyc01g014080.1.1	Gag-Pol polyprotein (AHRD V	0	0	0	0	0	0	0	0	0	0	1	NA	0	0	0	0	0	0	0	0	1	NA	0	0	0.62	0.21	0	0	0	0	0.05	NA	0	0	0	0	0	0	0	1	NA
Solyc01g014090.1.1	Plant synaptotagmin (AHRD V	0	0	0	0	0	0	0	0	0	0	1	NA	0	0	0	0	0	0	0	0	1	NA	0	0	0	0	0	0	0	0	1	NA	0	0	0	0	0	0	0	1	NA
Solyc01g014100.2.1	Unknown Protein (AHRD V1)	5.21	7.73	7.17	7.37	6.87	19.3	17.82	16.76	17.45	17.83	2.6	0.059174	2.42	9.05	5.87	5.78	2.41	3.6	1.36	2.46	0.43	0.749637	0	0	0	0	0	0	0	0	1	NA	0	0	0	0	0	0	0	1	NA
Solyc01g014110.2.1	Unknown Protein (AHRD V1)	3.43	5.34	1.01	1.21	2.75	10.48	2.71	3.15	4.92	5.31	1.93	0.899951	3.19	5.96	5.07	4.74	4.75	7.1	10.1	7.32	1.54	0.945807	0	1.4	0.85	0.75	1.69	0	0.62	0.77	1.03	1	0	0	0	0	0	1.5	0.75	75	1
Solyc01g014120.2.1	Unknown Protein (AHRD V1)	3.07	3.75	1.81	1.63	2.57	4.69	2.42	4.23	3.3	3.66	1.43	0.851655	4.28	4.17	2.43	3.63	3.99	5.26	9.19	6.15	1.69	0.627841	0.46	0.78	0.38	0.54	0.63	0.58	0.84	0.68	1.27	1	0	0.28	0.29	0.19	0.35	0.67	0.51	2.68	1
Solyc01g014130.1.1	LRR receptor-like serine/thre	0	0	0	0	0	0	0	0	0	0	1	NA	0	0	0	0	0	0	0	0	1	NA	0	0	0	0	0	0	0	0	1	NA	0	0	0	0	0	0	0	1	NA
Solyc01g014140.2.1	Hcr9-9D (AHRD V1 ***- O500	1.12	1.99	0	1.58	1.17	0	0	1.03	0	0.26	0.22	0.962949	1.04	0	0	0.35	0.78	1.74	0	0.84	2.42	1	0	0	0	0	0	0	0	0	1	NA	0	0	0	0	0	0	0	1	NA
Solyc01g014150.1.1	LRR receptor-like serine/thre	0	0	0	0	0	0	0	0	0	0	1	NA	0.28	0	0	0.09	0	0	0.24	0.08	0.86	1	0	0	0	0	0	0	0	0	1	NA	0	0	0	0	0	0	0	1	NA
Solyc01g014160.1.1	LRR receptor-like serine/thre	0	0	0	0	0	0	0	0	0	0	1	NA	0	0	0	0	0	0	0	0	1	NA	0	0	0	0	0	0	0	0	1	NA	0	0	0	0	0	0	0	1	NA
Solyc01g014170.1.1	Hcr9-9A (AHRD V1 ***- O500	0	0	0	0	0	0	0	0	0	0	1	NA	0	0	0	0	0	0	0	0	1	NA	0	0	0	0	0	0	0	0	1	NA	0	0	0	0	0	0	0	1	NA
Solyc01g014180.2.1	Zinc finger A20 and AN1 dom	607.55	612.74	533.96	517.58	567.96	462.16	387.9	567.06	568.62	496.43	0.87	0.959678	739.59	855.21	686.47	760.42	1003.44	826.53	958.53	929.5	1.22	0.618518	818.38	830.43	705.84	784.88	762.96	848.86	797.41	803.08	1.02	1	1438.59	1214.87	1532.32	1395.26	973.62	1324.66	1149.14	0.82	1
Solyc01g014190.1.1	Unknown Protein (AHRD V1)	0	0	0	0	0	0	0	0	0	0	1	NA	0	0	0	0	0	0	0	0	1	NA	0	0	0	0	0	0	0	0	1	NA	0	0	0	0	0	0	0	1	NA
Solyc01g014200.1.1	Unknown Protein (AHRD V1)	20.47	12.15	10.74	16.09	14.86	7.59	6.29	3.14	12.25	7.32	0.49	0.509856	12.7	13.84	10.57	12.37	7.89	2.36	5.36	5.2	0.42	0.410456	0.92	4.63	2.26	2.6	0.75	2.59	6.63	3.32	1.28	1	0	0.83	1.75	0.86	1.04	0	0.52	0.6	1
Solyc01g014210.1.1	ADP ATP carrier protein-like (	0	0	0	0	0	0	0	0	0	0	1	NA	0	0	0	0	0	0	0	0	1	NA	0	0	0	0	0	0	0	0	1	NA	0	0	0	0	0	0	0	1	NA
Solyc01g014220.1.1	cDNA clone J075118M20 full	2.41	1.67	0	1.9	1.5	3.58	3.81	1.73	3.08	3.05	2.04	0.453471	2.25	0.7	0.45	1.13	1.3	1.94	0.63	1.29	1.14	1	0.43	0.87	0.27	0.52	0.18	0	0.2	0.13	0.24	1	0	0.39	0	0.13	0.25	0.24	0.24	1.88	1
Solyc01g014230.2.1	U-box domain-containing pro	4.14	6.87	8.15	11.49	7.66	5.15	4.87	4.48	5.07	4.89	0.64	0.630371	8.09	8.76	10.77	9.21	8.17	5.76	6.7	6.88	0.75	0.662803	4.79	6.42	8.47	6.56	4.86	5.3	3.79	4.65	0.71	1	17.67	13.93	12.24	14.61	7.66	8.62	8.14	0.56	0.601719
Solyc01g014240.1.1	TMV resistance protein N (AH	0	0	0	0	0	0	0	0	0	0	1	NA	0	0	0	0	0	0	0	0	1	NA	0	0	0	0	0	0	0	0	1	NA	0	0	0	0	0	0	0	1	NA
Solyc01g014250.2.1	Unknown Protein (AHRD V1)	9.96	33.23	7.34	3.52	13.51	48.43	22.6	61.8	19.66	38.12	2.82	0.427165	19.68	40.03	16.82	25.51	15.54	39.31	52.82	35.89	1.41	0.967182	79.44	39.56	48.19	55.73	43.34	47.17	68.03	52.85	0.95	1	37.17	133.8	160.78	110.58	12.53	20.74	16.63	0.15	0.284696
Solyc01g014260.1.1	Unknown Protein (AHRD V1)	0	0	0	0	0	0	0	0	0	0	1	NA	0	0	0	0	0	0	0	0	1	NA	0	0	0	0	0	0	1.02	0.34	34	1	0	0	0	0	0	0	0	1	NA
Solyc01g014270.2.1	Unknown Protein (AHRD V1)	115.1	154.9	101.84	74.45	111.57	205.17	156.8	261.98	147.63	192.9	1.73	0.132624	148.19	137.88	72.91	119.66	125.1	151.17	148.92	141.73	1.18	0.834961	77.11	83.5	36.92	65.84	52.56	65.96	92.57	70.36	1.07	1	21.95	57.6	32.38	37.31	64.25	62.15	63.2	1.69	0.969829
Solyc01g014280.2.1	Unknown Protein (AHRD V1)	0	0	0	0	0	0	0	0	0	0	1	NA	0	0	0	0	0	0	0	0	1	NA	0	0	0	0	0	0	0	0	1	NA	0	0	0	0	0	0	0	1	NA
Solyc01g014290.1.1	Unknown Protein (AHRD V1)	0	0	0	0	0	0	0	0	0	0	1	NA	0	0	0	0	0	0	0	0	1	NA	0	0	0	0	0	0	0	0	1	NA	0	0	0	0	0	0	0	1	NA
Solyc01g014300.1.1	PR-protein (Fragment) (AHRD	0	0	0	0	0	0	0	0	0	0	1	NA	0	0	0	0	0	0	0	0	1	NA	0	0	0	0	0	0	0	0	1	NA	0	0	0	0	0	0	0	1	NA
Solyc01g014310.1.1	LRR receptor-like serine/thre	0	0	0	0	0	0	0	0	0	0	1	NA	0	0	0	0	0	0	0	0	1	NA	0	0	0	0	0	0	0	0	1	NA	0	0	0	0	0	0	0	1	NA
Solyc01g014320.2.1	S-adenosyl-L-methionine sali	0	0	0	0	0	0	0	0.63	0.24	0.22	21.75	1	0	0	0	0	0.47	0.18	0	0.22	21.67	1	0	0	0	0	0	0	0	0	1	NA	0	0	0	0	0	0	0	1	NA
Solyc01g014330.2.1	S-adenosyl-L-methionine carb	0	0	0	0	0	0	0	0	0.52	0.13	13	1	0	0	0	0	0	0	0	0	1	NA	0	0	0	0	0	0	0	0	1	NA	0	0	0	0	0	0	0	1	NA
Solyc01g014340.1.1	Unknown Protein (AHRD V1)	0	0	0	0	0	1.07	0	0	0	0.27	26.75	1	0	0	0	0	0	0	0	0	1	NA	0	0	0	0	0	0	0	0	1	NA	0	0	0	0	0	0	0	1	NA
Solyc01g014350.1.1	Kinase family protein (AHRD	0	0	0	0	0	0	0	0	0	0	1	NA	0	0	0	0	0	0	0	0	1	NA	0	0	0	0	0	0	0	0	1	NA	0	0	0	0	0	0	0	1	NA
Solyc01g014360.1.1	Unknown Protein (AHRD V1)	0	0	0	0	0	0	0	0	0	0	1	NA	0	0	0	0	0	0	0	0	1	NA	0	0	0	0	0	0	0	0	1	NA	0	0	0	0	0	0	0	1	NA
Solyc01g014370.1.1	Plant synaptotagmin integral	0	0	0	0	0	0	0	0	0	0	1	NA	0	0	0	0	0	0	0	0	1	NA	0	0	0	0	0	0	0	0	1	NA	0	0	0	0	0	0	0	1	NA
Solyc01g014380.2.1	Unknown Protein (AHRD V1)	4.1	4.03	3.31	2.44	3.47	5.75	4.08	5.55	7.58	5.74	1.65	0.397837	3.81	5.43	4.73	4.66	5.53	7.59	11.18	8.1	1.74	0.325233	1.22	1.05	0.21	0.83	1.27	2.29	0.63	1.4	1.69	1	0	0.31	0	0.1	0.39	1.13	0.76	7.35	1
Solyc01g014390.2.1	Unknown Protein (AHRD V1)	0	0	0	0	0	0	0	0	0	0	1	NA	0	0	0	0	0	0.57	0.86	0.48	47.67	1	0	0	0	0	0	0	0	0	1	NA	0	0	0	0	0	0	0	1	NA
Solyc01g014400.1.1	Dicer-like protein (AHRD V1 *	0	0	0	0	0	0	0	0	0	0	1	NA	0	0	0	0	0	0	0	0	1	NA	0	0	0	0	0	0	0	0	1	NA	0	0	0	0	0	0	0	1	NA
Solyc01g014410.1.1	Dicer-like protein (AHRD V1 *	0	0	0	0	0	0	0	0	0	0	1	NA	0	0	0	0	0	0	0	0	1	NA	0	0	0	0	0	0	0	0	1	NA	0	0	0	0	0	0	0	1	NA
Solyc01g014420.1.1	Dicer-like protein (AHRD V1 *	0	0	0	0	0	0	0	0	0	0	1	NA	0	0	0	0	0	0	0	0	1	NA	0	0	0	0	0	0	0	0	1	NA	0	0	0	0	0	0	0	1	NA
Solyc01g014430.1.1	Dicer-like protein (AHRD V1 *	0	0	0	0	0	0	0	0	0	0	1	NA	0	0	0	0	0	0	0	0	1	NA	0	0	0	0	0	0	0	0	1	NA	0	0	0	0	0	0	0	1	NA
Solyc01g014440.1.1	Dicer-like protein B (Fragmen	0	0	0	0	0	0	0	0	0	0	1	NA	0	0	0	0	0	0	0	0	1	NA	0	0	0	0	0	0	0	0	1	NA	0	0	0	0	0	0	0	1	NA
Solyc01g014450.1.1	Dicer-like protein (AHRD V1 *	0	0	0	0	0	0	0	0	0	0	1	NA	0	0	0	0	0	0	0	0	1	NA	0	0	0	0	0	0	0	0	1	NA	0	0	0	0	0	0	0	1	NA
Solyc01g014460.1.1	Endoribonuclease Dicer homo	0	0	0	0	0	0	0	0	0	0	1	NA	0	0	0	0	0	0	0	0	1	NA	0	0	0	0	0	0	0	0	1	NA	0	0	0	0	0	0	0	1	NA
Solyc01g014470.1.1	Unknown Protein (AHRD V1)	0.46	0	0	0	0.12	0	0	0.42	0	0.1	0.91	1	0	0	0	0	0	0	0	0	1	NA	0	0	0	0	0	0	0	0	1	NA	0	0	0	0	0	0	0	1	NA
Solyc01g014480.2.1	Ethylene insensitive 3 class tr	8	7.9	10.25	6.76	8.23	8.46	7.4	7.09	5.75	7.18	0.87	0.990806	8.35	7.89	8.98	8.41	7.85	8.44	9.05	8.45	1	1	12.14	11.05	12.01	11.73	12.69	12.8	12.52	12.67	1.08	1	13.46	14.46	16.36	14.76	13.49	12.75	13.12	0.89	1
Solyc01g014490.2.1	Type II DNA topoisomerase V	14.31	16.4	14.11	13.73	14.64	12.97	18.19	18.66	16.33	16.54	1.13	0.863251	15.41	16.36	15.65	15.81	15.64	15.58	16.95	16.06	1.02	1	14.97	15.82	10.99	13.93	15.39	14.04	14.07	14.5	1.04	1	17.14	15.66	14.32	15.71	13.1	14.78	13.94	0.89	1
Solyc01g014500.1.1	Unknown Protein (AHRD V1);	0	0	0	0	0	0	0	0	0	0	1	NA	0	0	0	0	0	0	0	0	1	NA	0	0	0	0	0	0	0	0	1	NA	0	0	0	0	0	0	0	1	NA
Solyc01g014510.1.1	S-locus-specific glycoprotein S	0	0	0	0	0	0	0	0	0	0	1	NA	0	0	0	0	0	0	0	0	1	NA	0	0	0	0	0	0	0	0	1	NA	0	0	0	0	0	0	0	1	NA
Solyc01g014520.1.1	Receptor-like protein kinase (	0	0	0	0	0	0	0	0	0	0	1	NA	0	0	0	0	0	0	0	0	1	NA	0	0	0	0	0	0	0	0	1	NA	0	0	0	0	0	0	0	1	NA
Solyc01g014530.2.1	Receptor like kinase (Fragmen	0	0.33	0	0	0.08	0.41	0	0.34	0.27	0.26	3.09	1	0.34	0.32	0	0.22	0.77	1.54	0.87	1.06	4.82	0.661485	3.89	4.23	1.84	3.32	3.17	3.09	7.02	4.43	1.33	1	0	0	0	0	0.34	0	0.17	17	1
Solyc01g014540.1.1	Unknown Protein (AHRD V1);	0	0	0	0	0	0	0	0	0	0	1	NA	0	0	0	0	0	0	0	0	1	NA	0	0	0	0	0	0	0	0	1	NA	0	0	0	0	0	0	0	1	NA
Solyc01g014550.1.1	LRR receptor-like serine/thre	0	0	0	0	0	0	0	0	0	0	1	NA	0	0	0	0	0	0	0	0	1	NA	0	0	0	0	0	0	0	0	1	NA	0	0	0	0	0	0	0	1	NA
Solyc01g014560.2.1	D-mannose binding lectin fan	0	0	0	0	0	0.35	0	0.29	0.23	0.22	21.75	0.68343	0	0	0	0	0.22	0.25	0.75	0.41	40.67	0.378205	22.86	12.57	10.1	15.18	6.68	10.36	22.7	13.25	0.87	1	0	0.46	0	0.15	0.29	0	0.14	0.95	1
Solyc01g014570.1.1	Unknown Protein (AHRD V1)	0	0	0	0	0	0	0	0	0	0	1	NA	0	0	0	0	0	0	0	0	1	NA	0	0	0	0	0	0	0	0	1	NA	0	0	0	0	0	0	0	1	NA
Solyc01g014580.1.1	Unknown Protein (AHRD V1)	0	0	0	0	0	0	0	0	0	0	1	NA	0	0	0	0	0	0	0	0	1	NA	0	0	0	0	0	0	0	0	1	NA	0	0	0	0	0	0	0	1	NA
Solyc01g014600.1.1	Unknown Protein (AHRD V1)	0	0	0	0	0	0	0	0	0	0	1	NA	0	0	0	0	0	0	0	0	1	NA	0	0	0	0	0	0	0	0	1	NA	0	0	0	0	0	0	0	1	NA
Solyc01g014610.1.1	Unknown Protein (AHRD V1)	0	0	0	0	0	0	0	0	0.68	0.17	17	1	0	0	0	0	0	2.93	2.96	1.96	196.33	0.494809	0	0	0	0	0	0	0	0	1	1	0	0.68	0	0.23	0.86	0	0.43	1.9	1
Solyc01g014620.1.1	Unknown Protein (AHRD V1)	0.53	0	0	0	0.13	0	0	0	0	0	0.08	1	0	0	0	0	0	0.82	0	0.27	27.33	1	0	0	0	0	0	0.4	0	0.13	13.33	NA	0	0	0	0	0	0	0	1	NA
Solyc01g014630.1.1	Unknown Protein (AHRD V1)	0	0	0	0	0	0	0	0	0	0	1	NA	0	0	0	0	0	0	0	0	1	NA	0	0	0	0	0	0	0	0	1	NA	0	0	0	0	0	0	0	1	NA
Solyc01g014640.1.1	CCHC-type integrase (AHRD V	0	0	0	0	0	0	0	0	0	0	1	NA	0	0	0	0	0	0	0	0	1	NA	0	0	0	0	0	0	0	0	1	NA	0	0	0	0	0	0	0	1	NA
Solyc01g014650.1.1	Receptor-like protein kinase (	0	0	0	0	0	0	0	0	0	0	1	NA	0	0	0	0	0	0	0	0	1	NA	0	0	0	0	0	0	0	0	1	NA	0	0	0	0	0	0	0	1	NA
Solyc01g014660.1.1	Receptor like kinase (Fragmen	0	0	0	0	0	0	0	0	0	0	1	NA	0	0	0	0	0	0	0	0	1	NA	0	0	0	0	0	0	0	0	1	NA	0	0	0	0	0	0	0	1	NA
Solyc01g014670.1.1	Unknown Protein (AHRD V1)	0	0	0	0	0	0	0	0	0	0	1	NA	0	0	0	0	0	0	0	0	1	NA	0	0	0	0	0	0	0	0	1	NA	0	0	0	0	0	0	0	1	NA
Solyc01g014680.1.1	CCHC-type integrase (AHRD V	0	0	0	0	0	0	0	0	0	0	1	NA	0	0	0	0	0	0	0	0	1	NA	0	0	0	0	0	0	0	0	1	NA	0	0	0	0	0	0	0	1	NA
Solyc01g014690.2.1	Receptor-like protein kinase (	0	0	0	0	0	0	0	0	0	0	1	NA	0	0	0	0	0	0	0	0	1	NA	0	0	0	0	0	0	0	0	1	NA	0	0	0	0	0	0	0	1	NA
Solyc01g014700.2.1	D-mannose binding lectin fan	0	0	0	0	0	0	0	0	0	0	1	NA	0	0	0	0	0.22	1	1.52	0.91	91.33	0.282403	1.04	0.26	1.28	0.86	1.69	1.95	4.69	2.78	3.23	1	0	0	0	0	0	0	0	1	NA
Solyc01g014710.1.1	Unknown Protein (AHRD V1);	11.13	2.83	13.13	13.49	10.15	4.42	1.88	2.92	8.56	4.45	0.44	0.546528	10.35	16.58	15.44	14.12	9.92	6.58	10.62	9.04	0.64	0.752529	17.34	13.6	24.46	18.47	16.19	15.06	12.16	14.47	0.78	1	6.78	21.36	11	13.05	22.56	25.09	23.82	1.83	1
Solyc01g014720.1.1	Ethylene-responsive transcrip	0	0	0	0	0	0	0.3	0	0	0.07	7.5	1	0	0	0	0	0.26	0	0	0.09	8.67	1	0	0	0	0	0.25	0	0	0.08	8.33	1	0	0	0	0	0.35	0	0.17	17.5	1
Solyc01g014730.1.1	Unknown Protein (AHRD V1)	0	2.26	0	0	0.56	2.82	0	4.67	1.82	2.33	4.12	1	0	0	0	0	0	0	0	0	1	NA	2.05	0	0	0.68	0	0	0	0	0.01	1	0	1.84	0	0.61	2.32	0	1.16	1.89	1
Solyc01g014740.1.1	Unknown Protein (AHRD V1)	0	0	0	0	0	0	0	0	0	0	1	NA	0	0	0.63	0.21	0	0	0	0	0.05	1	0	0	0.74	0.25	0	0	0	0	0.04	NA	0	0	0	0	0	0	0	1	NA
Solyc01g014760.1.1	Unknown Protein (AHRD V1)	0	0	0	0	0	0	0	0	0	0	1	NA	0	0	0	0	0	0	0	0	1	NA	0	0	0	0	0	0	0	0	1	NA	0	0	0	0	0	0	0	1	NA
Solyc01g014770.1.1	Ethylene receptor (AHRD V1 *	0	0	0	0	0	0	0	0	0	0	1	NA	0	0	0	0	0	0	0	0	1	NA	0	0	0	0	0	0	0.36	0.12	12	1	0	0	0	0	0	0	0	1	NA
Solyc01g014780.1.1	Dicer-like protein (AHRD V1 *	0	0	0	0	0	0	0	0	0	0	1	NA	0	0	0	0	0	0	0	0	1	NA	0	0	0	0	0	0	0	0	1	NA	0	0	0	0	0	0	0	1	NA
Solyc01g014790.1.1	Dicer-like protein (AHRD V1 *	0	0	0	0	0	0	0	0	0	0	1	NA	0	0	0	0	0	0	0	0	1	NA	0	0	0	0	0	0	0	0	1	NA	0	0	0	0	0	0	0	1	NA
Solyc01g014800.1.1	Dicer-like protein (AHRD V1 *	0	0	0	0	0	0	0	0	0	0	1	NA	0	0	0	0	0	0	0	0	1	NA	0	0	0	0	0	0	0	0	1	NA	0	0	0	0	0	0	0	1	NA
Solyc01g014820.1.1	Inosine-uridine preferring nuc	0	0	0	0	0	0	0	0	0	0	1	NA	0	0	0	0	0	0	0	0	1	NA	0	0	0	0	0	0	0	0	1	NA	0	0	0	0	0	0	0	1	NA
Solyc01g014830.1.1	Extensin-like protein Dif10 (F	0	0	0	0	0	0	0	0	0	0	1	NA	0	0	0	0	0	0	0	0	1	NA	0	0	0	0	0	0	0	0	1	NA	0	0	0	0	0	0	0	1	NA
Solyc01g014840.2.1	Tir-nbs-lrr, resistance protein	0.2	0.53	1.41	2.74	1.22	0.39	0.75	0.32	0.75	0.55	0.45	0.929884	0.51	2.16	1.64	1.44	0.66	0.57	0.66	0.63	0.44	0.55479	0.2	0.49	1.73	0.81	1.21	0.72	0.22	0.72	0.89	1	0.07	1.23	0.38	0.56	0.55	1.31	0.93	1.66	1
Solyc01g014850.2.1	Zinc finger CCCH domain-con	52.3	48.42	48.99	62.09	52.95	42.95	58.1	43.98	39.81	46.21	0.87	1	59.65	48.89	64.16	57.57	52.87	43	45.85	47.24	0.82	0.900797	39.92	49.26	39.13	42.77	46.96	50.2	41.37	46.18	1.08	1	67.64	37.03	50.12	51.6	58.23	55.31	56.77	1.1	1
Solyc01g014860.2.1	Zinc finger CCCH domain-con	25.07	15.27	21.79	29.86	23	14.67	16.67	16.98	18.94	16.82	0.73	0.9707	34.36	21.79	21.17	25.77	29.28	23.23	15.55	22.69	0.88	1	26.65	12.9	23.57	21.04	31.2	31	14.42	25.54	1.21	1	9.38	11.5	12.17	11.02	14.49	28.92	21.71	1.97	0.987148
Solyc01g014870.1.1	Ulp1 protease family C-termi	0	0	0	0	0	0	0	0	0	0	1	NA	0	0	0	0	0	0	0	0	1	NA	0	0	0	0	0	0	0	0	1	NA	0	0	0	0	0	0	0	1	NA
Solyc01g014880.2.1	Unknown Protein (AHRD V1)	0	0	0	0	0	0	0	0	0	0	1	NA	0	0	0	0	0	0	0	0	1	NA	0	0	0	0	0	0	0	0	1	NA	0	0	0	0	0	0	0	1	NA
Solyc01g014890.1.1	Unknown Protein (AHRD V1)	0	0	0	0	0	0	0	0	0	0	1	NA	0	0	0	0	0	0	0	0	1	NA	0	0	0	0	0	0	0	0	1	NA	0	0	0	0	0	0	0	1	NA
Solyc01g014900.1.1	Unknown Protein (AHRD V1)	0	0	0	0	0	0	0	0	0	0	1	NA	0	0	0	0	0	0	0	0	1	NA	0	0	0	0	0	0	0	0	1	NA	0	0	0	0	0	0	0	1	NA
Solyc01g014910.1.1	Transcription factor bHLH95 (	0	0	0	0	0	0	0	0	0	0	1	NA	0	0	0	0	0	0	0	0	1	NA	0	0	0	0	0	0	0	0	1	NA	0	0	0	0	0	0	0	1	NA
Solyc01g014920.1.1	Unknown Protein (AHRD V1)	0	0	0	0	0	0	0	0	0	0	1	NA	0	0	0	0	0	0	0	0	1	NA	0	0	0	0	0	0	0	0	1	NA	0	0	0	0	0.93	0	0.47	46.5	1
Solyc01g014930.1.1	LRR receptor-like serine/thre	0	0	0	0	0	0	0	0	0	0	1	NA	0	0	0	0	0	0	0	0	1	NA	0	0	0	0	0	0	0	0	1	NA	0	0	0	0	0	0	0	1	NA
Solyc01g014940.1.1	LRR receptor-like serine/thre	0	0	0	0	0	0	0	0	0	0	1	NA	0	0	0	0	0	0	0	0	1	NA	0	0	0	0	0	0	0	0	1	NA	0	0	0	0	0	0	0	1	NA
Solyc01g014950.1.1	Unknown Protein (AHRD V1)	0	0.9	0	0	0.23	0.56	0	0.47	0	0.26	1.14	1	1.42	0.88	1.71	1.34	1.76	3.15	1.2	2.04	1.52	1	0	0	0.5	0.17	0	0.38	1.11	0.5	2.98	1	0.72	0	0	0.24	0	0.44	0.22	0.92	1
Solyc01g014960.1.1	Transcription factor bHLH95 (	0.26	0	0	0	0.07	0	0	0	0	0	0.15	1	0.49	0	0	0.16	0.18	0.14	0.21	0.18	1.08	1	0	0	0	0	0	0	0	0	1	NA	0	0	0	0	0	0	0	1	NA
Solyc01g014970.1.1	Unknown Protein (AHRD V1);	0	0	0	0	0	0	0	0	0	0	1	NA	0	0	0	0	0	0	0	0	1	NA	0	0	0	0	0	0	0	0	1	NA	0	0	0	0	0	0	0	1	NA
Solyc01g014980.2.1	Unknown Protein (AHRD V1)	0	0	0	0	0	0	0	0	0	0	1	NA	0	0	0	0	0	0	0	0	1	NA	0.52	0	0	0.17	0	0	0	0	0.06	1	0	0	0	0	0	0	0	1	NA
Solyc01g014990.1.1	Unknown Protein (AHRD V1)	0	0	0	0	0	0	0	0	0	0	1	NA	0	0	0	0	0	0	0	0	1	NA	0	0	0	0	0	0	0	0	1	NA	0	0	0	0	0	0	0	1	NA
Solyc01g015000.2.1	Nitrogen regulatory protein P	76.19	87.38	58.1	65.28	71.74	65.07	99.28	81.73	114.29	90.09	1.26	0.480705	48.98	54.92	55.46	53.12	73.47	74.89	65.23	71.2	1.34	0.355812	47.29	55.52	43.65	48.82	74.67	80.05	68.16	74.29	1.52	0.974113	12.33	25.87	20.48	19.56	32.04	30.96	31.5	1.61	0.945561
Solyc01g015010.1.1	Unknown Protein (AHRD V1)	0	0	0	0	0	0	0	0	0	0	1	NA	0	0	0	0	0	0	0	0	1	NA	0.92	0	0	0.31	0	0	0	0	0.03	1	0	0	0	0	0	0	0	1	NA
Solyc01g015020.2.1	PRLI-interacting factor A (Fra	61.24	57.87	50.4	53.35	55.71	42.38	39.7	58.92	54.45	48.86	0.88	0.989302	65.14	75.91	75.47	72.17	67.51	81.12	70.86	73.16	1.01	1	62.36	72.59	54.75	63.23	81.79	57.79	64.03	67.87	1.07	1	75.73	109.87	105.4	97	60.51	70.64	65.58	0.68	0.813587
Solyc01g015030.1.1	Unknown Protein (AHRD V1)	0	0	0	0	0	0	0	0	0	0	1	NA	0	0	0	0	0	0	0	0	1	NA	0	0	0	0	0	0	0	0	1	NA	0	0	0	0	0	0	0	1	NA
Solyc01g015040.1.1	Unknown Protein (AHRD V1)	0	0	0	0	0	0	0	0	0	0	1	NA	0	0	0	0	0	0	0	0	1	NA	0	0	0	0	0	0	0	0	1	NA	0	0	0	0	0	0	0	1	NA
Solyc01g015050.1.1	Unknown Protein (AHRD V1)	0	0	0	0	0	0	0	0	0	0	1	NA	0	0	0	0	0	0	0	0	1	NA	0	0	0	0	0	0	0	0	1	NA	0	0	0	0	0	0	0	1	NA
Solyc01g015060.1.1	X1 (Fragment) (AHRD V1 *---	0	0	0	0	0	0	0	0	0	0	1	NA	0	0	0	0	0	0	0	0	1	NA	0	0	0	0	0	0	0	0	1	NA	0	0	0	0	0	0	0	1	NA
Solyc01g015070.2.1	Unknown Protein (AHRD V1)	0	0	0	0	0	0	0	0	0	0	1	NA	0	0	0	0	0	0	0	0	1	NA	0	0	0	0	0	0	0	0	1	NA	0	0	0	0	0	0	0	1	NA
Solyc01g015080.2.1	Peroxidase (AHRD V1 ***- D7	0	0	0	0	0	0	0	0	0	0	1	NA	0	0	0	0	0	0	0	0	1	NA	0	0	0	0	0	0	0	0	1	NA	0	0	0	0	0	0	0	1	NA
Solyc01g015090.1.1	Gag-Pol polyprotein (AHRD V	0	0	0	0	0	0	0	0	0	0	1	NA	0	0	0	0	0	0	0	0	1	NA	0	0	0	0	0	0	0	0	1	NA	0.31	0.16	0	0.16	0.2	0	0.1	0.64	1
Solyc01g015100.1.1	Transposon Ty1-A Gag-Pol po	0	0	0	0	0	0	0	0	0	0	1	NA	0	0	0	0	0	0	0	0	1	NA	0	0	0	0	0	0	0	0	1	1	0	0	0	0	0	0	0	1	NA
Solyc01g015110.1.1	Ulp1 protease family C-termi	0	0	0	0	0	0	0	0	0.15	0.04	3.75	1	0	0	0	0	0	0	0	0	1	NA	0	0	0	0	0.14	0	0	0.05	4.67	1	0	0	0	0	0	0	0	1	NA
Solyc01g015120.1.1	Ulp1 protease family C-termi	0	0	0	0	0	0	0	0	0	0	1	NA	0	0	0	0	0	0	0	0	1	NA	0	0	0	0	0	0	0	0	1	NA	0	0.42	0	0.14	0	0	0	0.07	1
Solyc01g015130.1.1	F-box family protein (AHRD V	0	0	0	0	0	0	0	0	0	0	1	NA	0	0	0	0	0	0	0	0	1	NA	0	0	0	0	0	0	0	0	1	NA	0	0	0	0	0	0	0	1	NA
Solyc01g015140.1.1	Cytochrome b (AHRD V1 ***-	0	0	0	0	0	0.83	0	0	0	0.21	20.75	1	0	0.65	0	0.22	0	0	0	0	0.05	1	0	0	0	0	0	0	0	0	1	NA	0	0	0	0	0	0	0	1	NA
Solyc01g015150.1.1	Polyprotein (AHRD V1 ***- A	0	0	0	0	0	0	0	0	0	0	1	NA	0	0	0	0	0	0	0	0	1	NA	0	0	0	0	0	0	0	0	1	NA	0	0	0	0	0	0	0	1	NA
Solyc01g015160.1.1	Unknown Protein (AHRD V1)	0	0	0	0	0	0	0	0	0	0	1	NA	0	0	0	0	0	0	0	0	1	NA	0	0	0	0	0	0	0	0	1	NA	0	0	0	0	0	0	0	1	NA

Solyc01g015170.1.1	Unknown Protein (AHRD V1)	0	0	0	0	0	0	0	0	0	0	1	NA	0	0	0	0	0	0	0	0	1	NA	0	0	0	0	0	0	0	0	1	NA	0	0	0	0	0	0	0	1	NA
Solyc01g015180.1.1	Unknown Protein (AHRD V1)	0	0	0	0	0	0	0	0	0	0	1	NA	0	0	0	0	0	0	0	0	1	NA	0	0	0	0	0	0	0	0	1	NA	0	0	0	0	0	0	0	1	NA
Solyc01g015190.1.1	Unknown Protein (AHRD V1)	0	0	0	0	0	0	0	0	0	0	1	NA	0	0	0	0	0	0	0	0	1	NA	0	0	0	0	0	0	0	0	1	NA	0	0	0	0	0	0	0	1	NA
Solyc01g015200.1.1	Unknown Protein (AHRD V1)	0	0	0	0	0	0	0	0	0	0	1	NA	0	0	0	0	0	0	0	0	1	NA	0	0	0	0	0	0	0	0	1	NA	0	0	0	0	0	0	0	1	NA
Solyc01g015210.1.1	B3 domain-containing protei	0	0	0	0	0	0	0	0	0	0	1	NA	0	0	0	0	0	0	0	0	1	NA	0	0	0	0	0	0	0	0	1	NA	0	0	0	0	0	0	0	1	NA
Solyc01g015220.1.1	Polyprotein (Fragment) (AHR	0	0	0	0	0	0	0	0	0	0	1	NA	0	0	0	0	0	0	0	0	1	NA	0	0	0	0	0	0	0	0	1	NA	0	0	0	0	0	0	0	1	NA
Solyc01g015230.1.1	Unknown Protein (AHRD V1)	0	0	0	0	0	0	0	0	0	0	1	NA	0	0	0	0	0	0	0	0	1	NA	0	0	0	0	0	0	0	0	1	NA	0	0	0	0	0	0	0	1	NA
Solyc01g015240.1.1	Unknown Protein (AHRD V1)	0	0	0	0	0	0	0	0	0	0	1	NA	0	0	0	0	0	0	0	0	1	NA	0	0	0	0	0	0	0	0	1	NA	0	0	0	0	0	0	0	1	NA
Solyc01g015250.1.1	Unknown Protein (AHRD V1)	0	0	0	0	0	0	0	0	0	0	1	NA	0	0	0	0	0	0	0	0	1	NA	0	0	0	0	0	0	0	0	1	NA	0	0	0	0	0	0	0	1	NA
Solyc01g015260.1.1	Unknown Protein (AHRD V1)	0	0	0	0	0	0	0	0	0	0	1	NA	0	0	0	0	0	0	0	0	1	NA	0	0	0	0	0	0	0	0	1	NA	0	0	0	0	0	0	0	1	NA
Solyc01g015270.1.1	Unknown Protein (AHRD V1)	0	0	0	0	0	0	0	0	0	0	1	NA	0	0	0	0	0	0	0	0	1	NA	0	0	0	0	0	0	0	0	1	NA	0	0	0	0	0	0	0	1	NA
Solyc01g015280.1.1	Unknown Protein (AHRD V1)	0	0	0	0	0	0	0	0	0	0	1	NA	0	0	0	0	0	0	0	0	1	NA	0	0	0	0	0	0	0	0	1	NA	0	0	0	0	0	0	0	1	NA
Solyc01g015290.1.1	Glycine-rich protein 2 (AHRD	0	0	0	0	0	0	0	0	0	0	1	NA	0	0	0	0	0	0	0	0	1	NA	0	0	0	0	0	0	0	0	1	NA	0	0	0	0	0	0	0	1	NA
Solyc01g015300.1.1	Unknown Protein (AHRD V1)	0	0	0	0	0	0	0	0	0	0	1	NA	0	0	0	0	0	0	0	0	1	NA	0	0	0	0	0	0	0	0	1	NA	0	0	0	0	0	0	0	1	NA
Solyc01g016300.1.1	Unknown Protein (AHRD V1)	0	0	0	0	0	0	0	0	0	0	1	NA	0	0	0	0	0	0	0	0	1	NA	0	0	0	0	0	0	0	0	1	NA	0	0	0	0	0	0	0	1	NA
Solyc01g016310.1.1	Unknown Protein (AHRD V1)	0	0	0	0	0	0	0	0	0	0	1	NA	0	0	0	0	0	0	0	0	1	NA	0	0	0	0	0	0	0	0	1	NA	0	0	0	0	0	0	0	1	NA
Solyc01g016320.1.1	Unknown Protein (AHRD V1)	0	0	0	0	0	0	0	0	0	0	1	NA	0	0	0	0	0	0	0	0	1	NA	0	0	0	0	0	0	0	0	1	NA	0	0	0	0	0	0	0	1	NA
Solyc01g016330.1.1	Gag-pol polyprotein (AHRD V	0	0	0	0	0	0	0	0	0	0	1	NA	0	0	0	0	0	0	0	0	1	NA	0	0	0	0	0	0	0	0	1	NA	0.32	0	0	0.11	0	0	0	0.09	1
Solyc01g016340.1.1	Gag-pol polyprotein (AHRD V	0	0	0	0	0	0	0	0	0	0	1	NA	0	0	0	0	0	0	0	0	1	NA	0	0	0	0	0	0	0	0	1	NA	0	0	0	0	0	0	0	1	NA
Solyc01g016350.1.1	Unknown Protein (AHRD V1)	0	0	0	0	0	0	0	0	0	0	1	NA	0	0	0	0	0	0	0	0	1	NA	0	0	0	0	0	0	0	0	1	NA	0	0	0	0	0	0	0	1	NA
Solyc01g016360.1.1	LRR receptor-like serine/thre	0.43	0	0	0	0.11	0	0	0	0.31	0.08	0.72	1	0	0	1.09	0.36	0.3	0.22	0	0.17	0.48	1	0	0	0	0	0.28	0	0.31	0.2	19.67	1	0	0.94	0.66	0.53	0.39	0.38	0.39	0.72	1
Solyc01g016370.1.1	LRR receptor-like serine/thre	0.85	0.51	0.45	0.67	0.62	0.63	0.82	0.96	0.54	0.74	1.19	1	0.35	1.15	1.04	0.85	1.05	0.54	0.89	0.83	0.98	1	1	0.77	1.51	1.09	1.49	2.73	2.69	2.3	2.11	0.847995	0	0.07	0.29	0.12	0.87	0.91	0.89	7.42	0.405925
Solyc01g016380.2.1	Os06g0207500 protein (Frag	0	0	0	0.38	0.1	0	0	0	0	0	0.11	1	0	0	0	0	0	0	0	0	1	NA	0	0	0	0	0	0	0	0	1	NA	0	0	0	0	0	0	0	1	NA
Solyc01g016390.2.1	Os06g0207500 protein (Frag	0	0	0.17	0	0.04	0	0.11	0	0	0.03	0.65	1	0	0	0	0	0	0	0	0	1	NA	0	0	0	0	0	0	0	0	1	NA	0	0	0	0	0	0	0	1	NA
Solyc01g016400.1.1	Unknown Protein (AHRD V1)	0	0	0	0	0	0.91	0.64	0.75	0.58	0.72	72	0.907563	0	1.42	2.06	1.16	1.69	2.11	3.84	2.55	2.2	0.979177	6.58	0.66	6.47	4.57	4.28	4.32	5.94	4.85	1.06	1	0	8.28	7.51	5.26	3.73	3.57	3.65	0.69	1
Solyc01g016410.2.1	RING finger protein 43 (AHRD	11.92	10.61	6.87	11.37	10.19	9.55	10.94	8.92	10.95	10.09	0.99	1	15.21	14.22	15.45	14.96	26.34	19.09	17.64	21.02	1.41	0.454837	25.08	27.56	22.84	25.16	32.96	36.35	32.12	33.81	1.34	1	34.29	35.03	36.23	35.18	38.06	34.03	36.05	1.02	1
Solyc01g016430.1.1	Unknown Protein (AHRD V1)	0	0	0	0	0	0	0	0	0	0	1	NA	0	0	0	0	0	0	0	0	1	NA	0	0	0	0	0	0	0	0	1	NA	0	0	0	0	0	0	0	1	NA
Solyc01g016450.1.1	Unknown Protein (AHRD V1)	0	0	0	0	0	0	0	0	0	0	1	NA	0	0	0	0	0	0	0	0	1	NA	0	0	0	0	0	0	0	0	1	NA	0	0	0	0	0	0	0	1	NA
Solyc01g016460.2.1	Unknown Protein (AHRD V1)	57.17	76.33	57.08	27.99	54.64	119.78	86.8	127.38	69.46	100.85	1.85	0.180295	65.46	80.29	61.29	69.01	33.55	80.84	105.42	73.27	1.06	1	40.86	30.46	24.01	31.78	18.78	18.33	28.84	21.98	0.69	1	25.01	20.76	23.67	23.15	18.12	32.78	25.45	1.1	1
Solyc01g016470.2.1	Unknown Protein (AHRD V1);	178.88	204.37	267.92	281.74	233.23	236.92	223.35	344.11	197.65	250.51	1.07	0.950907	230.12	175.35	185.38	196.95	121.43	161.59	146.56	143.19	0.73	0.644344	110.7	136.46	141.04	129.4	109.18	104.81	110.94	108.31	0.84	1	59.53	70.03	81.94	70.5	126.59	88.99	107.79	1.53	0.927575
Solyc01g016480.1.1	Ulp1 protease family C-termi	0	0	0	0	0	0	0	0	0	0	1	NA	0	0	0	0	0	0	0	0	1	NA	0	0	0	0	0	0	0	0	1	NA	0	0	0	0	0	0	0	1	NA
Solyc01g016510.1.1	AT4g03420/F9H3_4 (AHRD V	0	0	0	0	0	0	0	0	0	0	1	NA	0	0	0	0	0	0	0	0	1	NA	0	0	0	0	0	0	0	0	1	NA	0	0	0	0	0	0	0	1	NA
Solyc01g016520.1.1	DNA-directed RNA polymeras	0	0	0	0	0	0	0	0	0	0	1	NA	0	0	0	0	0	0	0	0	1	NA	0	0	0	0	0	0	0	0	1	NA	0	0	0	0	0	0	0	1	NA
Solyc01g016530.1.1	Unknown Protein (AHRD V1);	0	0	0	0	0	0	0	0	0	0	1	NA	0	0	0	0	0	0	0	0	1	NA	0	0	0	0	0	0	0	0	1	NA	0	0	0	0	0	0	0	1	NA
Solyc01g016540.1.1	Os08g0100600 protein (Frag	0	0	0	0	0	0	0	0	0	0	1	NA	0	0	0	0	0	0	0	0	1	NA	0	0	0	0	0	0	0	0	1	NA	0	0	0	0	0	0	0	1	NA
Solyc01g016550.1.1	Unknown Protein (AHRD V1)	0	0	0	0	0	0	0	0	0	0	1	NA	0	0	0	0	0	0	0	0	1	NA	0	0	0	0	0	0	0	0	1	NA	0	0	0	0	0	0	0	1	NA
Solyc01g016560.1.1	Unknown Protein (AHRD V1)	0	0.47	0	0	0.12	0	0	0	0	0	0.09	1	0.49	0	0	0.16	0	0	0	0	0.06	1	0	0	0	0	0	0	0	0	1	NA	0	0	0	0	0	0	0	1	NA
Solyc01g016570.1.1	Pol polyprotein (AHRD V1 ***	1.75	3.11	5.35	3.95	3.54	6.21	2.2	6.42	2.76	4.4	1.24	0.997374	1.62	0.91	2.95	1.83	0.24	1.81	2.47	1.51	0.82	1	1.97	1.14	2.08	1.73	0.92	1.85	0.25	1.01	0.58	1	3.47	6.59	5.37	5.14	0.64	1.53	1.08	0.21	0.343054
Solyc01g016580.1.1	Unknown Protein (AHRD V1)	0	0	0	0	0	0	0	0	0	0	1	NA	0	0	0	0	0	0	0	0	1	NA	0	0	0	0	0	0	0	0	1	NA	0	0	0	0	0	0	0	1	NA
Solyc01g016590.1.1	Unknown Protein (AHRD V1)	0	0	0	0	0	0	0	0	0	0	1	NA	0	0	0	0	0	0	0	0	1	NA	0	0	0	0	0	0	0	0	1	NA	0	0	0	0	0	0	0	1	NA
Solyc01g016600.1.1	Unknown Protein (AHRD V1);	0	0	0	0	0	0	0	0	0	0	1	NA	0	0	0	0	0	0	0	0	1	NA	0	0	0	0	0	0	0	0	1	NA	0	0	0	0	0	0	0	1	NA
Solyc01g016610.1.1	Branched-chain alpha keto-ac	0	0	0	0	0	0	0	0	0	0	1	NA	0	0	0	0	0	0	0	0	1	NA	0	0	0	0	0	0	0	0	1	NA	0	0	0	0	0	0	0	1	NA
Solyc01g016620.1.1	Unknown Protein (AHRD V1)	0	0	0	0	0	0	0	0	0	0	1	NA	0	0	0	0	0	0	0	0	1	NA	0	0	0	0	0	0	0	0	1	NA	0	0	0	0	0	0	0	1	NA
Solyc01g016630.1.1	Unknown Protein (AHRD V1)	0	0	0	0	0	0	0	0	0	0	1	NA	0	0	0	0	0	0	0	0	1	NA	0	0	0	0	0	0	0	0	1	NA	0	0	0	0	0	0	0	1	NA
Solyc01g016640.1.1	1-aminocyclopropane-1-carb	0	0	0	0	0	0	0	0	0	0	1	NA	0	0	0	0	0	0	0	0	1	NA	0	0	0	0	0	0	0	0	1	NA	0	0	0	0	0	0	0	1	NA
Solyc01g016650.1.1	Unknown Protein (AHRD V1)	0	0	0	0	0	0	0	0	0	0	1	NA	0	0	0	0	0	0	0	0	1	NA	0	0	0	0	0	0	0	0	1	NA	0	0	0	0	0	0	0	1	NA
Solyc01g016660.1.1	Granule-bound starch syntha	0	0	0	0	0	0	0	0	0	0	1	NA	0	0	0	0	0.8	0	0	0.27	26.67	1	0	0	0	0	0	0	0	0	1	NA	0	0	0	0	0	0	0	1	NA
Solyc01g016670.1.1	Unknown Protein (AHRD V1)	0	0	0	0	0	0	0	0	0	0	1	NA	0	0	0	0	0	0	0	0	1	NA	0	0	0	0	0	0	0	0	1	NA	0	0	0	0	0	0	0	1	NA
Solyc01g016680.1.1	1-aminocyclopropane-1-carb	0	0	0	0	0	0	0	0	0	0	1	NA	0	0	0	0	0	0	0	0	1	NA	0	0	0	0	0	0	0	0	1	NA	0	0	0	0	0	0	0	1	NA
Solyc01g016690.1.1	Unknown Protein (AHRD V1)	0	0	0	0	0	0	0	0	0	0	1	NA	0	0	0	0	0	0	0	0	1	NA	0	0	0	0	0	0	0	0	1	NA	0	0	0	0	0	0	0	1	NA
Solyc01g016700.2.1	Unknown Protein (AHRD V1)	0	4.3	0	0	1.07	0	0	2.96	1.16	1.03	0.96	1	1.5	1.4	0	0.97	0	0	5.06	1.69	1.74	1	5.2	0	1.6	2.27	2.11	2.44	2.35	2.3	1.01	1	0	1.17	2.47	1.21	1.47	0	0.73	0.61	1
Solyc01g016710.1.1	Polyprotein (Fragment) (AHR	0	0	0	0	0	0	0.27	0	0	0.07	6.75	1	0	0	0	0	0	0.36	0	0.12	12	1	0	0	0	0	0	0	0	0	1	1	0.49	0	0	0.16	0	0	0	0.06	1
Solyc01g016720.2.1	Unknown Protein (AHRD V1)	0	0	0	0	0	0	0	0	0	0	1	NA	0	0	0	0	0	0	0	0	1	NA	0	0	0	0	0	0	0	0	1	NA	0	0	0	0	0	0	0	1	NA
Solyc01g016730.1.1	Unknown Protein (AHRD V1)	0	0	0	0	0	0	0	0	0	0	1	NA	0	0	0	0	0	0	0	0	1	NA	0	0	0	0	0	0	0	0	1	NA	0	0	0	0	0	0	0	1	NA
Solyc01g016740.1.1	Helicase-like protein (AHRD V	0	0	0	0	0	0	0	0	0	0	1	NA	0	0	0	0	0	0	0	0	1	NA	0	0	0	0	0	0	0	0	1	NA	0	0	0	0	0	0	0	1	NA
Solyc01g016750.1.1	Helicase-like protein (AHRD V	0	0	0	0	0	0	0	0	0	0	1	NA	0	0	0	0	0	0	0	0	1	NA	0	0	0	0	0	0	0	0	1	NA	0	0	0	0	0	0	0	1	NA
Solyc01g016760.1.1	Unknown Protein (AHRD V1)	0	0	0	0	0	0	0	0	0	0	1	NA	0	0	0	0	0	0	0	0	1	NA	0	0	0	0	0	0	0	0	1	NA	0	0	0	0	0	0	0	1	NA
Solyc01g016770.1.1	Myb-related transcription fac	0	0	0	0	0	0	0	0	0	0	1	NA	0	0	0	0	0	0	0	0	1	NA	0	0	0	0	0	0	0	0	1	NA	0	0	0	0	0	0	0	1	NA
Solyc01g016780.1.1	Unknown Protein (AHRD V1)	0	0	0	0	0	0	0	0	0	0	1	NA	0	0	0	0	0	0	0	0	1	NA	0	0	0	0	0	0	0	0	1	NA	0	0	0	0	0	0	0	1	NA
Solyc01g016790.1.1	Unknown Protein (AHRD V1)	0	0	0	0	0	0	0	0	0	0	1	NA	0	0	0	0	0	0	0	0	1	NA	0	0	0	0	0	0	0	0	1	NA	0	0	0	0	0	0	0	1	NA
Solyc01g016800.1.1	Unknown Protein (AHRD V1)	0	0	0	0	0	0	0	0	0	0	1	NA	0	0	0	0	0	0	0	0	1	NA	0	0	0	0	0	0	0	0	1	NA	0	0	0	0	0	0	0	1	NA
Solyc01g016810.1.1	Unknown Protein (AHRD V1)	0	0	0	0	0	0	0	0	0	0	1	NA	0	0	0	0	0	0	0	0	1	NA	0	0	0	0	0	0	0	0	1	NA	0	0	0	0	0	0	0	1	NA
Solyc01g016820.1.1	Unknown Protein (AHRD V1)	0	0	0	0	0	0	0	0	0	0	1	NA	0	0	0	0	0	0	0	0	1	NA	0	0	0	0	0	0	0	0	1	1	0	0	0	0	0	0	0	1	NA
Solyc01g016830.1.1	Unknown Protein (AHRD V1)	0	0	0	0	0	0	0	0	0	0	1	NA	0	0	0	0	0	0	0	0	1	NA	0	0	0	0	0	0	0	0	1	NA	0	0	0	0	0	0	0	1	NA
Solyc01g016840.1.1	Unknown Protein (AHRD V1)	0	0	0	0	0	0	0	0	0	0	1	NA	0	0	0	0	0	0	0	0	1	NA	0	0	0	0	0	0	0	0	1	NA	0	0	0	0	0	0	0	1	NA
Solyc01g016850.1.1	Unknown Protein (AHRD V1)	0	0	0	0	0	0	0	0	0	0	1	NA	0	0	0	0	0	0	0	0	1	NA	0	0	0	0	0	0	0	0	1	NA	0	0	0	0	0	0	0	1	NA
Solyc01g016860.1.1	Transposase family tnp2 prot	0.22	0	0	0	0.06	0	0	0.2	0	0.05	0.91	1	0	0.19	0	0.06	0	0	0	0	0.16	1	0	0	0	0	0.14	0	0.16	0.1	10	1	0	0	0	0	0	0.19	0.1	9.5	1
Solyc01g016870.1.1	Unknown Protein (AHRD V1)	0	0	0	0	0	0	0	0	0	0	1	NA	0	0	0	0	0	0	0	0	1	NA	0	0	0	0	0	0	0	0	1	NA	0	0	0	0	0	0	0	1	NA
Solyc01g016880.1.1	Unknown Protein (AHRD V1)	0	0	0	0	0	0	0	0	0	0	1	NA	0	0	0	0	0	0	0	0	1	NA	0	0	0	0	0	0	0	0	1	NA	0	0	0	0	0	0	0	1	NA
Solyc01g016890.1.1	Unknown Protein (AHRD V1)	0	0	0	0	0	0	0	0	0	0	1	NA	0	0	0	0	0	0	0	0	1	NA	0	0	0	0	0	0	0	0	1	NA	0	0	0	0	0	0	0	1	NA
Solyc01g016900.1.1	Vacuolar protein sorting-asso	0	0	0	0	0	0	0	0	0	0	1	NA	0	0	0	0	0	0	0	0	1	NA	0	0	0	0	0	0	0	0	1	NA	0	0	0	0	0	0	0	1	NA
Solyc01g016910.1.1	Unknown Protein (AHRD V1)	0	0	0	0	0	0	0	0	0	0	1	NA	0	0	0	0	0	0	0	0	1	NA	0	0	0	0	0	0	0	0	1	NA	0	0	0	0	0	0	0	1	NA
Solyc01g016920.1.1	Unknown Protein (AHRD V1);	0	0	0	0	0	0	0	0	0	0	1	NA	0	0	0	0	0	0	0	0	1	NA	0	0	0	0	0	0	0	0	1	NA	0	0	0	0	0	0	0	1	NA
Solyc01g016930.1.1	Unknown Protein (AHRD V1);	0	0	0	0	0	0	0	0	0	0	1	NA	0	0	0	0	0	0	0	0	1	NA	0	0	0	0	0	0	0	0	1	NA	0	0	0	0	0	0	0	1	NA
Solyc01g016940.1.1	Unknown Protein (AHRD V1)	0	0	0	0	0	0	0	0	0	0	1	NA	0	0	0	0	0	0	0	0	1	NA	0	0	0	0	0	0	0	0	1	NA	0	0	0	0	0	0	0	1	NA
Solyc01g016950.1.1	Transposon Ty1-PL Gag-Pol p	0	0	0	0	0	0	0	0	0	0	1	NA	0	0	0	0	0	0.12	0	0.04	4	1	0.18	0	0	0.06	0	0	0	0	0.17	1	0	0	0	0	0	0	0	1	NA
Solyc01g016960.1.1	Nbs, resistance protein fragm	0	0	0	0	0	0	0	0	0	0	1	NA	0	0	0	0	0	0	0	0	1	NA	0	0	0	0	0	0	0	0	1	NA	0	0	0	0	0	0	0	1	NA
Solyc01g016970.1.1	Unknown Protein (AHRD V1)	0	0.46	1.21	0.72	0.6	0	0.81	1.41	1.47	0.92	1.54	1	3.33	5.33	0.43	3.03	3.9	3.18	1.61	2.9	0.96	1	4.13	5	2.54	3.89	3.36	2.33	11.55	5.75	1.48	1	0	1.11	1.57	0.89	2.34	0	1.17	1.31	1
Solyc01g016980.1.1	Unknown Protein (AHRD V1)	0	0	0	0	0	0	0	0	0	0	1	NA	0	0	0	0	0	0	0	0	1	NA	0	0	0	0	0	0	0	0	1	NA	0	0	0	0	0	0	0	1	NA
Solyc01g016990.1.1	Unknown Protein (AHRD V1)	0	0	0	0	0	0	0	0	0	0	1	NA	0	0	0	0	0	0	0	0	1	NA	0	0	0	0	0	0	0	0	1	NA	0	0	0	0	0	0	0	1	NA
Solyc01g017000.1.1	Unknown Protein (AHRD V1)	0	0	0	0	0	0	0	0	0	0	1	NA	0	0	0	0	0	0	0	0	1	NA	0	0	0	0	0	0	0	0	1	NA	0	0	0	0	0	0	0	1	NA
Solyc01g017010.1.1	Unknown Protein (AHRD V1)	0	0	0	0	0	0	0	0	0	0	1	NA	0	0	0	0	0	0	0	0	1	NA	0	0	0	0	0	0	0	0	1	NA	0	0	0	0	0	0	0	1	NA
Solyc01g017020.1.1	Unknown Protein (AHRD V1)	0	0	0	0	0	0	0	0	0	0	1	NA	0	0	0	0	0	0	0	0	1	NA	0	0	0	0	0	0	0	0	1	NA	0	0	0	0	0	0	0	1	NA
Solyc01g017030.1.1	ARID/BRIGHT DNA-binding d	0	0	0	0	0	0	0	0	0	0	1	NA	0	0	0	0	0	0.67	0	0.22	22.33	1	0	0	0	0	0.28	0.32	0	0.2	20	1	0	0	0	0	0	0	0	1	NA
Solyc01g017040.1.1	Unknown Protein (AHRD V1)	0	0	0	0	0	0	0	0	0	0	1	NA	0	0	0	0	0	0	0	0	1	NA	0	0	0	0	0	0	0	0	1	NA	0	0	0	0	0	0	0	1	NA
Solyc01g017050.1.1	PG1 protein (Fragment) (AHR	0	0	0	0	0	0	0	0	0	0	1	NA	0	0	0	0	0	0	0	0	1	NA	0	0	0	0	0	0	0	0	1	NA	0	0	0	0	0	0	0	1	NA
Solyc01g017060.1.1	Ycf2 (AHRD V1 ***- D2KLP5_	0	0	0	0	0	0	0	0	0	0	1	NA	0	0	0	0	0	0	0	0	1	NA	0	0	0	0	0	0	0	0	1	NA	0	0	0	0	0	0.25	0.12	12.5	1
Solyc01g017070.1.1	Ycf2 (AHRD V1 *-*- A6Y9W3_	0	0	0	0	0	0	0	0	0	0	1	NA	0	0	0	0	0.45	0	0	0.15	15	1	0	0	0	0	0	0	0	0	1	NA	0	0	0	0	0	0	0	1	NA
Solyc01g017080.1.1	Ycf2 (AHRD V1 *-*- A6YA57_	0	0	0	0	0	0	0	0	0	0	1	NA	0	0	0	0	0	0	0	0	1	NA	0	0	0	0	0	0	0	0	1	NA	0	0	0	0	0	0	0	1	NA
Solyc01g017090.2.1	NADH-quinone oxidoreducta	0	0	0	0	0	0	0	0	0.24	0.06	6	1	0	0.29	0.28	0.19	0	0	0	0	0.05	1	0	0	0	0	0.22	0	0	0.07	7.33	1	0	0	0	0	0	0	0	1	NA
Solyc01g017100.1.1	NADH-quinone oxidoreducta	0	0	0	0	0	0	0	0	0	0	1	NA	0	0	0	0	0.37	0	0	0.12	12.33	1	0	0	0	0	0	0	0	0	1	NA	0	0	0	0	0	0	0	1	NA
Solyc01g017110.1.1	NADH dehydrogenase subuni	0	0	0	0	0	0	0	0	0	0	1	NA	0	0	0	0	0	0	0	0	1	NA	0	0	0.17	0.06	0	0	0	0	0.18	1	0	0	0	0	0	0	0	1	NA
Solyc01g017120.1.1	Ycf2 (AHRD V1 ***- A6Y9Y0_	0	0	0	0	0	0	0	0	0	0	1	NA	0	0	0	0	0	0	0	0	1	NA	0	0	0	0	0	0	0	0	1	NA	0	0	0	0	0	0	0	1	NA
Solyc01g017130.1.1	Ycf2 (AHRD V1 ***- D2SXY2_	0	0	0	0	0	0	0	0	0	0	1	NA	0	0	0	0	0	0	0	0	1	NA	0	0.7	0	0.23	0	0	0	0	0.04	1	0	0	0	0	0	0	0	1	NA
Solyc01g017140.1.1	Ycf2 (AHRD V1 ***- A6YA48_	0	0	0	0	0	0	0	0	0	0	1	NA	0	0	0	0	0	0	0	0	1	NA	0	0	0	0	0	0	0	0	1	NA	0	0	0	0	0	0	0	1	NA
Solyc01g017150.1.1	Ycf15 (AHRD V1 ***- D2KLP6	0	0	0	0	0	0	0	0	0	0	1	NA	0	0	0	0	0	0	0	0	1	NA	0	0	0	0	0	0	0	0	1	NA	0	0	0	0	0	0	0	1	NA
Solyc01g017160.1.1	NAD(P)H-quinone oxidoreduc	0	0	0	0	0	0	0	0	0	0	1	NA	0	0	0	0	0	0	0	0	1	NA	0	0	0	0	0	0	0	0	1	NA	0	0	0	0	0	0	0	1	NA
Solyc01g017170.1.1	NAD(P)H-quinone oxidoreduc	0	0	0	0	0	0.53	0	0	0	0.13	13.25	1	0	0.42	0	0.14	0.33	0	0.38	0.24	1.69	1	0	0	0.95	0.32	0	0	0	0	0.03	1	0.68	0	0.74	0.47	0	0	0	0.02	1
Solyc01g017180.1.1	NAD(P)H-quinone oxidoreduc	0	0	0	0	0	0	0	0	0	0	1	NA	0	0	0	0	0	0	0	0	1	NA	0	0	0	0	0	0	0	0	1	NA	0	0	0	0	0	0	0	1	NA
Solyc01g017190.1.1	NAD(P)H-quinone oxidoreduc	0	0	0	0	0	0	0	0	0	0	1	NA	0	0	0	0	0	0	0	0	1	NA	0	0	0	0	0	0	0	0	1	NA	0	0	0	0	0	0	0	1	NA
Solyc01g017200.1.1	Acetyl-coenzyme A carboxyla	0	0	0	0	0	0	0	0	0	0	1	NA	0	0	0	0	0	0	0	0	1	NA	0	0	0	0	0	0	0	0	1	NA	0	0	0	0	0	0	0	1	NA
Solyc01g017210.1.1	NAD(P)H-quinone oxidoreduc	0	0	0	0	0	0	0	0	0	0	1	NA	0	0	0	0	0	0	0	0	1	NA	0	0	0	0	0	0	0	0	1	NA	0	0	0	0	0	0	0	1	NA
Solyc01g017220.1.1	ATP synthase epsilon chain c	8.16	18.76	10.43	8.66	11.5	13.61	5.91	11.88	22.46	13.46	1.17	1	6.33	10.05	12.06	9.48	5.66	6.34	18.17	10.06	1.06	1	6.04	8.87	7.43	7.45	3.13	7.22	2.97	4.44	0.6	1	7.74	0.99	1.05	3.26	3.74	3.58	3.66	1.12	1
Solyc01g017230.1.1	NADH dehydrogenase subuni	0	0	0	0	0	0	0	0	0	0	1	NA	0	0	0	0	0	0	0	0	1	NA	0	0	0	0	0	0	0	0	1	NA	0	0	0	0	0	0	0	1	NA
Solyc01g017240.1.1	Ycf1 (Fragment) (AHRD V1 **	0	0	0	0	0	0	0	0.51	0	0.13	12.75	1	0	0	0	0	0	0	0	0	1	NA	0	0	0	0	0	0	0	0	1	NA	0	0	0	0	0	0	0	1	NA
Solyc01g017250.1.1	Ycf1 (AHRD V1 *--- D2KLR1_	0	0	0	0	0	0	0	0	0	0	1	NA	0	0	0	0	0	0	0	0	1	NA	0	0	0	0	0	0	0	0	1	NA	0	0	0	0	0	0	0	1	NA

Solyc01g017260.1.1	NAD(P)H-quinone oxidoreduc	0	0	0	0	0	0	0	0	0	0	1	NA	0	0	0	0	0	0	0	0	1	NA	0	0	0	0	0	0	0	0	1	NA	0	0	0	0	0	0	0	1	NA
Solyc01g017270.1.1	Ycf15 (AHRD V1 ***- A6YA40	0	0	0	0	0	0	0	0	0	0	1	NA	0	0	0	0	0	0	0	0	1	NA	0	0	0	0	0	0	0	0	1	NA	0	0	0	0	0	0	0	1	NA
Solyc01g017280.1.1	Ycf2 (AHRD V1 ***- C0LEB7_	0	0	0	0	0	0	0	0	0	0	1	NA	0	0	0	0	0	0	0	0	1	NA	0	0	0	0	0	0	0	0	1	NA	0	0	0	0	0	0	0	1	NA
Solyc01g017290.1.1	Ycf2 (AHRD V1 ***- A6YA16_	0	0	0	0	0	0	0	0	0	0	1	NA	0	0	0	0	0	0	0	0	1	NA	0	0	0	0	0	0	0	0	1	NA	0	0	0	0	0	0	0	1	NA
Solyc01g017300.1.1	Photosystem I P700 chloroph	0	0	0	0	0	0	0	0	0	0	1	NA	0	0	0	0	0.16	0	0	0.05	5.33	1	0.19	0.19	0.23	0.2	0	0	0.17	0.06	0.28	1	1.01	0.17	0	0.39	0.43	0	0.21	0.55	1
Solyc01g017310.1.1	Photosystem I P700 chloroph	0	0	0	0	0	0	0	0	0	0	1	NA	0	0	0	0	0	0	0	0	1	NA	0	0	0	0	0	0	0	0	1	NA	0	0	0	0	0	0	0	1	NA
Solyc01g017320.1.1	Photosystem I P700 chloroph	0	0	0	0	0	0	0	0	0	0	1	NA	0	0	0	0	0	0	0	0	1	NA	0	0	0	0	0	0	0	0	1	NA	0	0	0	0	0	0	0	1	NA
Solyc01g017330.1.1	Photosystem I P700 chloroph	0	0	0	0	0	1.06	0	0	0.34	0.35	35	0.988051	0	0	0	0	0	0	0	0	1	NA	0	0	0	0	0	0	0.35	0.12	11.67	1	0	0	0	0	0	0	0	1	NA
Solyc01g017340.1.1	Cytochrome c biogenesis prot	0	0	0	0	0	0	0	0.52	0	0.13	13	1	0	0	0.24	0.08	0	0	0	0	0.12	1	0	0	0.28	0.09	0	0	0	0	0.11	1	0	0.41	0	0.14	0	0	0	0.07	1
Solyc01g017350.1.1	DNA-directed RNA polymeras	0	0	0	0	0	0	0	0	0	0	1	NA	0	0	0	0	0	0	0	0	1	NA	0	0	0	0	0	0	0	0	1	NA	0	0	0	0	0	0	0	1	NA
Solyc01g017360.1.1	DNA-directed RNA polymeras	0	0	0	0	0	0	0	0	0	0	1	NA	0	0	0	0	0	0	0	0	1	NA	0	0	0	0	0	0	0	0	1	NA	1	0	0	0.33	0.64	0	0.32	0.96	1
Solyc01g017370.1.1	DNA-directed RNA polymeras	0	0	0	0	0	0	0	0	0	0	1	NA	0	0	0	0	0	0	0	0	1	NA	0	0	0	0	0	0	0	0	1	NA	0	0.87	0	0.29	0	0	0	0.03	1
Solyc01g017380.1.1	DNA-directed RNA polymeras	0	0	0	0	0	0	0	0	0	0	1	NA	0	0	0	0	0	0	0	0	1	NA	0	0	0.76	0.25	0.5	0	0	0.17	0.66	1	1.09	0	0	0.36	0.7	0	0.35	0.96	1
Solyc01g017390.1.1	DNA-directed RNA polymeras	0	0	0	0	0	0	0	0	0	0	1	NA	0	0	0	0	0	0	0	0	1	NA	0	0	0	0	0	0	0	0	1	NA	0	0	0	0	0	0	0	1	NA
Solyc01g017400.1.1	30S ribosomal protein S7 chlo	0	0	0	0	0	0	0	0	0	0	1	NA	0	0	0	0	0	0	0	0	1	NA	0	0	0	0	0	0	0	0	1	NA	0	0	0	0	0	0.27	0.14	13.5	1
Solyc01g017410.1.1	Ycf2 (AHRD V1 ***- D2KLP5_	0	0	0	0	0	0	0	0	0	0	1	NA	0	0	0	0	0	0	0	0	1	NA	0	0	0	0	0.26	0	0	0.09	8.67	1	0	0	0	0	0	0	0	1	NA
Solyc01g017420.1.1	DNA-directed RNA polymeras	0	0	0	0	0	0	0	0	0.1	0.03	2.5	1	0	0	0.12	0.04	0	0	0	0	0.25	1	0	0	0	0	0	0	0	0	1	1	0.39	0	0.21	0.2	0.13	0	0.07	0.33	1
Solyc01g017430.1.1	Cytochrome b6-f complex sub	0	0	0	0	0	0	0	0	0	0	1	NA	0	0	0	0	0	0	0	0	1	NA	0	0	0	0	0	0	0	0	1	NA	0	0	0	0	0	0	0	1	NA
Solyc01g017440.1.1	DNA-directed RNA polymeras	0	0	0	0	0	0	0	0.3	0	0.07	7.5	1	0	0	0	0	0	0.17	0	0.06	5.67	1	0	0	0	0	0	0	0	0	1	NA	0	0.24	0	0.08	0	0	0	0.12	1
Solyc01g017450.1.1	30S ribosomal protein S11 ch	0	0	0	0	0	0	0	0	0	0	1	NA	0	0	0	0	0	0	0	0	1	NA	0	0	0	0	0	0	0	0	1	NA	0	0	0	0	0	0	0	1	NA
Solyc01g017460.1.1	DNA-directed RNA polymeras	0	0	0	0	0	0	0	0	0	0	1	NA	0	0	0	0	0	0	0	0	1	NA	0	0	0	0	0.83	0	0	0.28	27.67	1	1.8	0	1.95	1.25	1.16	0	0.58	0.46	1
Solyc01g017470.1.1	DNA-directed RNA polymeras	0	0	0	0	0	0	0	0	0	0	1	NA	0	0	0	0	0	0	0	0	1	NA	0	0	0	0	0.31	0	0	0.1	10.33	1	0	0	0	0	0	0	0	1	NA
Solyc01g017480.1.1	Ycf1 (Fragment) (AHRD V1 **	0	0	0	0	0	0	0	0	0	0	1	NA	0	0	0	0	0	0	0	0	1	NA	0	0	0	0	0	0	0	0	1	NA	0	0	0	0	0	0	0	1	NA
Solyc01g017490.1.1	Conserved protein (AHRD V1	0	0	0	0	0	0	0	0	0	0	1	NA	0	0	0	0	0	0	0	0	1	NA	0	0	0	0	0	0	0	0	1	NA	0	0	0	0	0	0	0	1	NA
Solyc01g017500.1.1	Unknown Protein (AHRD V1)	0	0	0	0	0	0	0	0	0	0	1	NA	0	0	0	0	0	0	0	0	1	NA	0	0	0	0	0	0	0	0	1	NA	0	0	0	0	0	0	0	1	NA
Solyc01g017510.1.1	DNA-directed RNA polymeras	0	0	0	0	0	0	0	0	0	0	1	NA	0	0	0	0	0	0	0	0	1	NA	0	0	0	0	0	0	0	0	1	1	0.63	0	0	0.21	0	0	0	0.05	1
Solyc01g017520.1.1	ATP synthase subunit a chloro	0	0	0	0	0	0	0	0	0	0	1	NA	0	0	0	0	0	0	0	0	1	NA	0	0	0	0	0	0	0	0	1	NA	0	0	0	0	0	0	0	1	NA
Solyc01g017530.1.1	30S ribosomal protein S2 (AH	0	0	0.39	0	0.1	0	0.26	0	0.24	0.12	1.28	1	0.31	0.29	0.83	0.48	0.23	0.17	0	0.13	0.28	1	0	0.27	0	0.09	0.22	0	0.24	0.15	1.7	1	0.47	0.24	0	0.24	0.3	1.44	0.87	3.68	1
Solyc01g017540.1.1	Dirigent-like protein 1 (AHRD	0	0	0	0	0	0	0	0	0	0	1	NA	0	0	0	0	0	0	0	0	1	NA	0	0	0	0	0	0	0	0	1	NA	0	0	0	0	0	0	0	1	NA
Solyc01g017550.1.1	Unknown Protein (AHRD V1)	0	0	0	0	0	0	0	0	0	0	1	NA	0	0	0	0	0	0	0	0	1	NA	0	0	0	0	0	0	0	0	1	NA	0	0	0	0	0	0	0	1	NA
Solyc01g017560.1.1	Unknown Protein (AHRD V1)	0	0	0	0	0	0	0	0	0	0	1	NA	0	0	0	0	0	0	0	0	1	NA	0	0	0	0	0	0	0	0	1	NA	0	0	0	0	0	0	0	1	NA
Solyc01g017570.1.1	Unknown Protein (AHRD V1)	0	0	0	0	0	0	0	0	0	0	1	NA	0	0	0	0	0	1.49	0.75	0.75	74.67	1	0	0.78	0	0.26	0	1.45	0	0.48	1.86	1	0	0	0	0	0	0	0	1	NA
Solyc01g017580.1.1	Unknown Protein (AHRD V1)	0	0	0	0	0	0	0	0	0	0	1	NA	0	0	0	0	0	0	0	0	1	NA	0	0	0	0	0	0	0	0	1	NA	0	0	0	0	0	0	0	1	NA
Solyc01g017590.1.1	Unknown Protein (AHRD V1)	0	0	0	0	0	0	0	0	0	0	1	NA	0	2.61	0	0.87	0	3.11	0	1.04	1.19	1	0	7.33	0	2.44	0	0	0	0	0	1	8.53	2.18	23.06	11.26	0	0	0	0	0.904864
Solyc01g017600.2.1	Plant viral-response family pr	0	0	0	0.6	0.15	0.23	0	0	0	0.06	0.38	1	0	0	0	0	0.15	0	0	0.05	5	1	0	0	0	0	0	0	0.15	0.05	5	1	0	0	0	0	0	0	0	1	NA
Solyc01g017610.1.1	Alliinase (Fragment) (AHRD V	0	0	0	0	0	0	0	0	0	0	1	NA	0	0	0	0	0	0	0	0	1	NA	0	0	0	0	0	0	0	0	1	NA	0	0	0	0	0	0	0	1	NA
Solyc01g017620.1.1	Unknown Protein (AHRD V1)	0	0	0	0	0	0	0	0	0	0	1	NA	0	0	0	0	0	0	0	0	1	NA	0	0	0	0	0	0	0	0	1	NA	0	0	0	0	0	0	0	1	NA
Solyc01g017630.1.1	Ribosomal protein S4 (AHRD	0	0	0	0	0	0	0	0	0	0	1	NA	0	0	0	0	0	0	0	0	1	NA	0	0	0	0	0	0	0	0	1	NA	0	0	0	0	0	0	0	1	NA
Solyc01g017640.1.1	Unknown Protein (AHRD V1)	0	0	0	0	0	0	0	0	0	0	1	NA	0	0	0	0	0	0	0	0	1	NA	0	0	0	0	0	0	0	0	1	NA	0	0	0	0	0	0	0	1	NA
Solyc01g017650.1.1	Unknown Protein (AHRD V1)	0	0	0	0	0	0	0	0	0	0	1	NA	0	0	0	0	0	0	0	0	1	NA	0	0	0	0	0	0	0	0	1	NA	0	0	0	0	0	0	0	1	NA
Solyc01g017660.1.1	Unknown Protein (AHRD V1)	0	0	0	0	0	0	0	0	0	0	1	NA	0	0	0	0	0	0	0	0	1	NA	0	0	0	0	0	0	0	0	1	NA	0	0	0	0	0	0	0	1	NA
Solyc01g017670.1.1	Unknown Protein (AHRD V1)	0	0	0	0	0	0	0	0	0	0	1	NA	0	0	0	0	0	0	0	0	1	NA	0	0	0	0	0	0	0	0	1	NA	0	0	0	0	0	0	0	1	NA
Solyc01g017680.1.1	Unknown Protein (AHRD V1)	0	0	0	0	0	0	0	0	0	0	1	NA	0	0	0	0	0	0	0	0	1	NA	0	0	0	0	0	0	0	0	1	NA	0	0	0	0	0	0	0	1	NA
Solyc01g017690.1.1	Nbs, resistance protein fragm	0	0	0	0	0	0	0	0.62	0	0.15	15.5	1	0	0	0	0	0	0	0	0	1	NA	0	0	0	0	0	0	0	0	1	NA	0	0	0	0	0	0	0	1	NA
Solyc01g017700.1.1	Resistance protein PSH-RGH7	0	0	0	0	0	0	0	0	0	0	1	NA	0	0	0	0	0	0	0	0	1	NA	0	0	0	0	0	0	0	0	1	NA	0	0	0	0	0	0	0	1	NA
Solyc01g017710.1.1	Unknown Protein (AHRD V1)	0	0	1.5	0	0.38	0	1	2.33	0	0.83	2.22	1	2.36	2.21	2.14	2.24	1.76	5.91	1.99	3.22	1.44	1	1.02	0	0	0.34	0.83	0.96	0	0.6	1.75	1	0	0	1.95	0.65	0	0	0	0.02	1
Solyc01g017720.1.1	Unknown Protein (AHRD V1)	0	0	0	1.46	0.36	1.15	0	0	2.22	0.84	2.31	1	2.88	0.9	2.61	2.13	1.43	1.6	0.81	1.28	0.6	1	1.67	0.84	1.02	1.18	2.03	0	0.75	0.93	0.79	1	0	0	0	0	0	0	0	1	NA
Solyc01g017730.1.1	Unknown Protein (AHRD V1)	0	0	0	0	0	0	0	0	0	0	1	NA	0	0	0	0	0	0	0	0	1	NA	0	0	0	0	0	0	0	0	1	NA	0	0	0	0	0	0	0	1	NA
Solyc01g017740.1.1	Cytochrome b6 (AHRD V1 *-*	0	0.6	0.79	0	0.35	0	0	0.62	0.48	0.28	0.79	1	0	0	0	0	0.93	0.35	1.06	0.78	78	0.900689	0	0.55	0.67	0.41	0.88	0	0	0.29	0.72	1	2.87	0.49	1.04	1.47	1.23	0.59	0.91	0.62	1
Solyc01g017750.1.1	Unknown Protein (AHRD V1)	0	0	0	0	0	0	0	0	0	0	1	NA	0	0	0	0	0	0	0	0	1	NA	0	0	0	0	0	0	0	0	1	NA	0	0	0	0	0	0	0	1	NA
Solyc01g017760.1.1	Unknown Protein (AHRD V1)	0	0	0	0	0	0	0	0	0	0	1	NA	0	0	0	0	0	0	0	0	1	NA	0	0	0	0	0	0	0	0	1	NA	0	0	0	0	0	0	0	1	NA
Solyc01g017770.1.1	F-box family protein (AHRD V	0	0	0	0	0	0	0	0	0	0	1	NA	0	0	0	0	0	0	0	0	1	NA	0	0	0	0	0	0	0	0	1	NA	0	0	0	0	0	0	0	1	NA
Solyc01g017780.1.1	Transposon Ty1-BR Gag-Pol p	0	0	0	0	0	0	0	0	0	0	1	NA	0	0	0	0	0	0	0	0	1	NA	0	0	0	0	0	0	0	0	1	NA	0	0	0	0	0	0	0	1	NA
Solyc01g017790.1.1	ARID/BRIGHT DNA-binding do	0	0	0	0	0	0	0	0	0	0	1	NA	0	0	0	0	0	0.79	0	0.26	26.33	1	0	0	0	0	0.33	0.38	0	0.24	23.67	1	0	0	0	0	0	0	0	1	NA
Solyc01g017800.1.1	Unknown Protein (AHRD V1)	0	0	0	0	0	0	0	0	0	0	1	NA	0	0	0	0	0	0	0	0	1	NA	0	0	0	0	0	0	0	0	1	NA	0	0	0	0	0	0	0	1	NA
Solyc01g017810.1.1	Transposase family tnp2 prot	0	0	0	0	0	0	0	0	0	0	1	NA	0	0	0	0	0	0	0	0	1	NA	0	0	0	0	0	0	0	0	1	NA	0	0	0	0	0	0	0	1	NA
Solyc01g017820.1.1	Unknown Protein (AHRD V1)	0	0	0	0	0	0	0	0	0	0	1	NA	0	0	0	0	0	0	0	0	1	NA	0	0	0	0	0	0	0	0	1	NA	0	0	0	0	0	0	0	1	NA
Solyc01g017830.1.1	Transposase family tnp2 prot	0.22	0	0	0	0.06	0	0	0.2	0	0.05	0.91	1	0	0.19	0	0.06	0	0	0	0	0.16	1	0	0	0	0	0.14	0	0.16	0.1	10	1	0	0	0	0	0	0.19	0.1	9.5	1
Solyc01g017840.1.1	subunit of replication protein	0.17	0.74	0.2	1.41	0.63	0.19	0.13	0.31	0.84	0.37	0.58	1	0.15	0.58	0.56	0.43	0.23	0.17	0.39	0.26	0.61	1	0	0.14	0.17	0.1	0	0.13	0	0.04	0.42	1	0	0	0	0	0	0.15	0.07	7.5	1
Solyc01g017850.1.1	Helicase-like protein (AHRD V	0	0	0	0	0	0	0	0	0.06	0.01	1.5	1	0	0	0	0	0	0	0	0	1	NA	0	0	0	0	0	0	0	0	1	NA	0	0	0	0	0	0	0	1	NA
Solyc01g017860.2.1	Unknown Protein (AHRD V1)	10.15	7.53	12.97	7.18	9.46	3.76	5.34	4.67	14.57	7.08	0.75	1	11.01	16.17	2.14	9.77	9.39	8.76	12.63	10.26	1.05	1	11.62	6.89	4.2	7.57	10.56	13.46	9.24	11.09	1.46	1	3.61	5.53	3.9	4.35	8.52	11.12	9.82	2.26	1
Solyc01g017870.1.1	Unknown Protein (AHRD V1)	0	0.66	0	0	0.17	0	0	0	0	0	0.06	1	0	0	0	0	0	0.38	0	0.13	12.67	1	0	0	0	0	0	0	0	0	1	1	0	0	0	0	0	0	0	1	NA
Solyc01g017880.1.1	Unknown Protein (AHRD V1)	0	0	0	0	0	0	0	0	0	0	1	NA	0	0	0	0	0	0	0	0	1	NA	0	0	0	0	0	0	0	0	1	NA	0	0	0	0	0	0	0	1	NA
Solyc01g017890.1.1	Unknown Protein (AHRD V1)	0	0	0	0	0	0	0	0	0	0	1	NA	0	0	0	0	0	0	0	0	1	NA	0	0	0	0	0	0	0	0	1	NA	0	0	0	0	0	0	0	1	NA
Solyc01g017900.1.1	Unknown Protein (AHRD V1)	0	0	0	0	0	0	0	0	0	0	1	NA	0	0	0	0	0	0	0	0	1	NA	0	0	0	0	0	0	0	0	1	NA	0	0	0	0	0	0	0	1	NA
Solyc01g017910.1.1	Unknown Protein (AHRD V1)	0	0	0	0	0	0	0	0	0	0	1	NA	0	0	0	0	0	0	0	0	1	NA	0	0	0	0	0	0	0	0	1	NA	0	0	0	0	0	0	0	1	NA
Solyc01g017920.1.1	Unknown Protein (AHRD V1)	0	0	0	0	0	0	0	0	0	0	1	NA	0	0	0	0	0	0	0	0	1	NA	0	0	0	0	0	0	0	0	1	NA	0	0	0	0	0	0	0	1	NA
Solyc01g017930.1.1	Unknown Protein (AHRD V1)	0	0	0	0	0	0	0	0	0	0	1	NA	0	0	0	0	0	0	0	0	1	NA	0	0	0	0	0	0	0	0	1	NA	0	0	0	0	0	0	0	1	NA
Solyc01g017940.1.1	Unknown Protein (AHRD V1)	0	0	0	0	0	0	0	0	0	0	1	NA	0	0	0	0	0	0	0	0	1	NA	0	0	0	0	0	0	0	0	1	NA	0	0	0	0	0	0	0	1	NA
Solyc01g017950.1.1	Unknown Protein (AHRD V1)	0	0	0	0	0	0	0	0	0	0	1	NA	0	0	0	0	0	0	0	0	1	NA	0	0	0	0	0	0	0	0	1	NA	0	0	0	0	0	0	0	1	NA
Solyc01g017960.1.1	Unknown Protein (AHRD V1)	0	0	0	0	0	0	0	0	0	0	1	NA	0	0	0	0	0	0	0	0	1	NA	0	0	0	0	0	0	0	0	1	NA	0	0	0	0	0	0	0	1	NA
Solyc01g017970.1.1	Unknown Protein (AHRD V1)	0	0	0	0	0	0	0	0	0	0	1	NA	0	0	0	0	0	0	0	0	1	NA	0	0	0	0	0	0	0	0	1	NA	0	0	0	0	0	0	0	1	NA
Solyc01g017980.1.1	Transposase (AHRD V1 **-- Q	0	0	0	0	0	0	0	0	0	0	1	NA	0	0	0	0	0	0	0	0	1	NA	0	0	0	0	0	0	0	0	1	NA	0	0	0	0	0	0	0	1	NA
Solyc01g017990.1.1	Unknown Protein (AHRD V1)	0	0	0	0	0	0	0	0	0	0	1	NA	0	0	0	0	0	0	0	0	1	NA	0	0	0	0	0	0	0	0	1	NA	0	0	0	0	0	0	0	1	NA
Solyc01g018000.1.1	Unknown Protein (AHRD V1)	0	0	0	0	0	0	0	0	0	0	1	NA	0	0	0	0	0	0	0	0	1	NA	0	0	0	0	0	0	0	0	1	NA	0	0	0	0	0	0	0	1	NA
Solyc01g018010.1.1	Unknown Protein (AHRD V1)	2.44	2.17	0	0	1.15	2.72	3.86	2.25	3.51	3.08	2.68	1	4.55	6.37	4.13	5.02	6.78	2.53	5.76	5.02	1	1	5.92	0	0	1.97	1.61	0	3.56	1.72	0.87	1	0	0	0	0	0	0	0	1	NA
Solyc01g018020.1.1	Transketolase-like protein (AH	0	0	0	0	0	0	0	0	0.06	0.01	1.5	1	0.16	0.07	0.14	0.12	0.06	0	0	0.02	0.16	0.911351	0.27	0.2	0.17	0.21	0.49	0.19	0.24	0.31	1.44	1	0	0.18	0.13	0.1	0	0	0	0.1	1
Solyc01g018040.1.1	Protein FAR1-RELATED SEQU	0	0	0	0	0	0	0	0	0	0	1	NA	0	0	0	0	0	0	0	0	1	NA	0	0	0	0	0	0	0	0	1	NA	0	0	0	0	0	0	0	1	NA
Solyc01g018050.1.1	Unknown Protein (AHRD V1)	0	0	0	0	0	0	0	0	0	0	1	NA	0	0	0	0	0	0	0	0	1	NA	0	0	0	0	0	0	0	0	1	NA	0	0	0	0	0	0	0	1	NA
Solyc01g018060.1.1	30S ribosomal protein S7 chlo	0	0	0	0	0	0	0	0	0	0	1	NA	0	0	0	0	0	0	0	0	1	NA	0	0	0	0	0	0	0	0	1	NA	0	0	0	0	0	0	0	1	NA
Solyc01g018070.1.1	ARID/BRIGHT DNA-binding do	0	0	0	0	0	0	0	0	0	0	1	NA	0	0	0	0	0	0	0	0	1	NA	0	0	0	0	0	0	0	0	1	NA	0	0	0	0	0	0	0	1	NA
Solyc01g018080.1.1	Unknown Protein (AHRD V1)	0	0	0	0	0	0	0	0	0	0	1	NA	0	0	0	0	0	0	0	0	1	NA	0	0	0	0	0	0	0	0	1	NA	0	0	0	0	0	0	0	1	NA
Solyc01g018090.1.1	Photosystem I P700 chloroph	0	0.59	0	0	0.15	0	0	0	0	0	0.07	1	0	0	0	0	0	0	0	0	1	NA	0	0	0	0	0	0	0	0	1	NA	0.94	0.96	0	0.63	0	0.58	0.29	0.46	1
Solyc01g018100.1.1	Unknown Protein (AHRD V1)	0	0	0	0	0	0	0	0	0	0	1	NA	0	0	0	0	0	0	0	0	1	NA	0	0	0	0	0	0	0	0	1	NA	0	0	0	0	0	0	0	1	NA
Solyc01g019100.1.1	Unknown Protein (AHRD V1)	0	0	0	0	0	0	0	0	0	0	1	NA	0	0	0	0	0	0	0	0	1	NA	0	0	0	0	0	0	0	0	1	NA	0	0	0	0	0	0	0	1	NA
Solyc01g020100.1.1	Unknown Protein (AHRD V1)	0	0	0	0	0	0	0	0	0	0	1	NA	0	0	0	0	0	0	0	0	1	NA	0	0	0	0	0	0	0	0	1	NA	0	0	0	0	0	0	0	1	NA
Solyc01g020110.1.1	Zinc finger family protein (AH	0	0	0	0	0	0	0	0	0	0	1	NA	0	0	0	0	0	0	0	0	1	NA	0	0	0	0	0	0	0	0	1	1	0	0	0	0	0	0	0	1	NA
Solyc01g020120.1.1	DNA replication licensing fact	0	0.39	0	0	0.1	0.49	0	0	0	0.12	1.26	1	0	0	0.37	0.12	0	0	0	0	0.08	1	0	0.36	0	0.12	0.29	0.33	0.64	0.42	3.5	1	0	0	0	0	0	0	0	1	NA
Solyc01g020130.2.1	Glycosyltransferase (AHRD V1	7.93	6.21	9.35	5.83	7.33	5.29	4.51	2.04	5.69	4.38	0.6	0.540005	9.74	5.79	12.05	9.19	11	12.15	9.72	10.96	1.19	0.954908	8.46	13.44	11.02	10.97	10.42	7.93	9.01	9.12	0.83	1	16.69	17.97	14.63	16.43	13.94	15.57	14.75	0.9	1
Solyc01g020140.2.1	Glycosyltransferase (AHRD V1	22.46	22.49	24.84	17.87	21.92	12.48	16.62	7.74	19.15	14	0.64	0.77875	26.11	13.42	33.2	24.24	36.99	41.43	28.68	35.7	1.47	0.637682	22.68	38.89	32.04	31.2	37.81	29.79	31.71	33.1	1.06	1	53.88	69.32	53.96	59.05	41.12	50.46	45.79	0.78	1
Solyc01g020150.2.1	Glycosyltransferase (AHRD V1	14.9	20.83	22.6	36.12	23.61	28.39	21.84	25.44	25.97	25.41	1.08	1	47.51	20.35	28.76	32.21	32.47	47.38	43.48	41.11	1.28	0.922676	18.91	34.68	40.13	31.24	44.74	24.19	40.32	36.42	1.17	1	33.28	57.19	55.63	48.7	68.18	70.9	69.54	1.43	1
Solyc01g020160.1.1	Unknown Protein (AHRD V1)	0	0.76	1.01	0	0.44	0	0	0	0	0	0.02	1	0	0.74	0.72	0.49	0	0	1.35	0.45	0.92	1	0	0.7	0	0.23	0.56	0	1.25	0.6	2.59	1	1.22	0	1.32	0.85	0	0	0	0.01	1
Solyc01g020170.1.1	Basic helix-loop-helix family p	0	0	0.26	0	0.07	0	0	0	0	0	0.15	1	0	0	0	0	0	0	0	0	1	NA	0	0	0	0	0	0	0	0	1	NA	0	0	0	0	0	0	0	1	NA
Solyc01g020180.1.1	Transcription factor bHLH95 (	0	0	0	0	0	0	0	0	0	0	1	NA	0	0	0	0	0	0	0	0	1	NA	0	0	0	0	0	0	0	0	1	NA	0	0	0	0	0	0	0	1	NA
Solyc01g020190.2.1	Membrane-associated ring fin	11.52	11.18	24.09	12.59	14.84	8.73	9.92	6.26	9.02	8.48	0.57	0.472931	15.1	18.21	24.76	19.36	23.24	11.93	11.52	15.56	0.8	1	6.35	12.37	23.91	14.21	9.97	13.1	6.1	9.72	0.68	1	33.5	20.15	15.3	22.98	25.88	29.38	27.63	1.2	1
Solyc01g020200.1.1	Hypothetical chloroplast RF1	0	0	0	0	0	0	0	0.39	0.3	0.17	17.25	1	0	0	0	0	0	0	0.33	0.11	11	1	0	0	0	0	0	0	0.31	0.1	10.33	1	0.6	0.31	1.3	0.74	0	0	0	0.01	1
Solyc01g020210.1.1	Mutator-like transposase (AH	0	0	0.59	0	0.15	0	0.39	0	0	0.1	0.66	1	0	0	0	0	0	0	0	0	1	NA	0.4	0	0	0.13	0	0	0	0	0.07	1	0	0	0	0	0	0	0	1	NA
Solyc01g020220.1.1	Replication factor C subunit 5	5.88	9.88	7.96	8.32	8.01	9.92	8.59	9.81	7.82	9.04	1.13	0.999281	8.1	7.57	8.28	7.98	8.76	9.02	9.58	9.12	1.14	1	4.93	4.79	1.73	3.82	4.29	3.63	4.28	4.07	1.07	1	6.5	3.32	4.69	4.84	3.99	6.87	5.43	1.12	1
Solyc01g020230.2.1	Unknown Protein (AHRD V1)	6	7.12	4.72	2.83	5.17	13.33	3.16	11.03	0	6.88	1.33	1	1.86	5.21	3.38	3.48	1.39	5.18	6.28	4.28	1.23	1	6.46	4.89	3.97	5.11	2.63	3.03	1.46	2.37	0.46	1	11.37	5.81	6.15	7.78	0	3.51	1.75	0.23	1
Solyc01g020240.2.1	T31J12.1 protein (Fragment)	25.31	24.93	20.8	22.6	23.41	34.13	27.84	31.66	26.36	30	1.28	0.576217	24.31	28.85	25.93	26.36	21.73	27.07	22.99	23.93	0.91	1	21.96	21.3	25.33	22.86	25.76	24.06	23.73	24.52	1.07	1	28.32	37.22	29.35	31.63	32.28	38.93	35.61	1.13	1
Solyc01g020250.1.1	Pol polyprotein (AHRD V1 ***	0	0	0.46	0	0.12	0	0.31	0	0	0.08	0.67	1	0	0	0	0	0.27	0	0	0.09	9	1	0.31	0	0.39	0.23	0	0	0	0	0.04	1	0	0	0	0	0	0	0	1	NA
Solyc01g020260.1.1	Unknown Protein (AHRD V1)	0	0	0	0	0	0	0	0	0	0	1	NA	0	0	0	0	0	0	0	0	1	NA	0	0	0	0	0	0	0	0	1	NA	0	0	0	0	0	0	0	1	NA
Solyc01g020270.1.1	Ulp1 protease family C-termi	0	0	0	0	0	0	0	0	0	0	1	NA	0	0	0	0	0	0	0	0	1	NA	0	0	0	0	0	0	0	0	1	NA	0	0	0	0	0	0	0	1	NA
Solyc01g020280.1.1	Unknown Protein (AHRD V1)	0	0	0	0	0	0	0	0	0	0	1	NA	0	0	0	0	0	0	0	0	1	NA	0	0	0	0	0	0	0	0	1	NA	0	0	0	0	0	0	0	1	NA
Solyc01g020290.1.1	60S ribosomal protein L7A-lik	3.6	4.27	1.42	3.39	3.17	6.67	0	4.41	5.17	4.06	1.28	1	1.12	1.04	0	0.72	1.66	1.86	5.66	3.06	4.25	0.822356	0.97	0.98	2.38	1.44	3.15	0	3.5	2.22	1.54	1	0	0	0	0	2.2	0	1.1	110	1
Solyc01g020300.2.1	Unknown Protein (AHRD V1)	0	0	0	0	0	0	0	0	0	0	1	NA	0	0	0	0	0	1.1	0	0.37	36.67	1	0	0	0	0	0	0	0	0	1	NA	0	0	0	0	0	0	0	1	NA

Solyc01g020310.1.1	Pol polyprotein (AHRD V1 ***	0	0	0	0	0	0	0	0	0	0	1	NA	0	0	0	0	0	0	0	0	1	NA	0	0	0	0	0	0	0	0	1	NA	0	0	0	0	0	0	0	1	NA
Solyc01g020320.2.1	Unknown Protein (AHRD V1)	56.12	78.87	40.66	34.82	52.62	49.25	45.86	69.71	59.38	56.05	1.07	1	75.1	76.18	41.58	64.29	62.83	61.69	54.17	59.56	0.93	1	79.55	76.22	38.12	64.63	55.63	57.46	72.47	61.85	0.96	1	30.8	60.79	42.4	44.66	38.76	35.4	37.08	0.83	1
Solyc01g020330.2.1	Unknown Protein (AHRD V1)	0	0	0	0	0	0	0	0	0	0	1	NA	0	0	0	0	0	0	0	0	1	NA	0	0	0	0	0	0	0	0	1	NA	0	0	0	0	0	0	0	1	NA
Solyc01g020340.1.1	Pentatricopeptide repeat-con	0.15	0.78	0.34	0.21	0.37	0.16	0.34	1.07	0.63	0.55	1.49	1	0.54	1.01	0.37	0.64	0.6	0.45	1.03	0.69	1.08	1	0.12	0.47	0.29	0.29	0.19	0.33	0.11	0.21	0.72	1	0	0.32	0.45	0.26	0	0.25	0.12	0.49	1
Solyc01g020350.1.1	Unknown Protein (AHRD V1)	0	0	0	0	0	0	0	0	0	0	1	NA	0	0	0	0	0	0	0	0	1	NA	0	0	0	0	0	0	0	0	1	NA	0	0	0	0	0	0	0	1	NA
Solyc01g020360.1.1	Unknown Protein (AHRD V1)	0	0	0	0	0	0	0	0	0	0	1	NA	0	0	0	0	0	0	0	0	1	NA	0	0	0	0	0	0	0	0	1	NA	0	0	0	0	0	0	0	1	NA
Solyc01g020370.1.1	Unknown Protein (AHRD V1)	0	0	0	0	0	0	0	0	0	0	1	NA	0	0	0	0	0	0	0	0	1	NA	0	0	0	0	0	0	0	0	1	NA	0	0	0	0	0	0	0	1	NA
Solyc01g020380.1.1	Ulp1 protease family C-termi	0	0.18	0	0	0.04	0.46	0.16	0.19	0	0.2	4.5	1	0	0.18	0	0.06	0	0	0	0	0.17	1	0.17	0	0	0.06	0.14	0	0.15	0.1	1.71	1	0	0.15	0	0.05	0.19	0	0.1	1.9	1
Solyc01g020390.1.1	Unknown Protein (AHRD V1)	0	0	0	0	0	0	0	0	0	0	1	NA	0	0	0	0	0	0	0	0	1	NA	0	0	0	0	0	0	0	0	1	NA	0	0	0	0	0	0	0	1	NA
Solyc01g020400.1.1	Unknown Protein (AHRD V1)	0	0	0	0	0	0	0	0	0	0	1	NA	0	0	0	0	0	0	0	0	1	NA	0	0	0	0	0	0	0	0	1	NA	0	0	0	0	0	0	0	1	NA
Solyc01g020410.1.1	Unknown Protein (AHRD V1)	0	0	0	0	0	0	0	0	0	0	1	NA	0	0	0	0	0	0	0	0	1	NA	0	0	0	0	0	0	0	0	1	NA	0	0	0	0	0	0	0	1	NA
Solyc01g020430.1.1	Thiosulfate cyanide sulfurtrar	0	0	0	0	0	0	0	0	0	0	1	NA	0	0	0	0	0	0	0	0	1	NA	0	0	0	0	0	0	0	0	1	NA	0	0	0	0	0	0	0	1	NA
Solyc01g020440.2.1	Replication factor C subunit 5	22.67	24.15	19.95	16.82	20.9	27.06	24.79	35.03	27.88	28.69	1.37	0.383737	23.16	23.59	22.29	23.01	24.88	21.46	28.33	24.89	1.08	1	6.16	11.51	4.43	7.37	8.18	5.92	10.7	8.27	1.12	1	12.42	12.55	13.43	12.8	17.52	20.37	18.95	1.48	0.942876
Solyc01g020450.1.1	Unknown Protein (AHRD V1)	0	0	0	0	0	0	0	0	0	0	1	NA	0	0	0	0	0	0	0	0	1	NA	0	0	0	0	0	0	0	0	1	NA	0	0	0	0	0	0	0	1	NA
Solyc01g020460.1.1	ORF65d (AHRD V1 *-*- A4QM	0	0	0	0	0	0	0	0	0	0	1	NA	0	0	0	0	0	0	0	0	1	NA	0	0.2	0	0.07	0	0	0	0	0.15	1	0	0	0	0	0	0	0	1	NA
Solyc01g020470.1.1	NADH-ubiquinone oxidoredu	0	0	0	0	0	0	0	0	0	0	1	NA	0	0	0	0	0	0	0	0	1	NA	0	0	0	0	0	0	0	0	1	NA	0	0	0	0	0	0	0	1	NA
Solyc01g020480.1.1	HAT family dimerisation dom	0	0	0	0	0	0	0	0	0	0	1	NA	0	0	0	0	0	0	0	0	1	NA	0	0	0	0	0	0	0	0	1	NA	0	0	0	0	0	0	0	1	NA
Solyc01g020490.1.1	Unknown Protein (AHRD V1)	0	0	0	0	0	0	0	0	0	0	1	NA	0	0	0	0	0	0	0	0	1	NA	0	0	0	0	0	0	0	0	1	NA	0	0	0	0	0	0	0	1	NA
Solyc01g020500.1.1	Unknown Protein (AHRD V1)	1.43	6.38	3.38	6.09	4.32	9.57	5.66	21.1	14.41	12.68	2.94	0.254994	8	3.74	6.06	5.93	0.99	7.43	5.64	4.69	0.79	1	6.95	4.67	8.54	6.72	3.77	2.17	6.27	4.07	0.61	1	2.04	0	6.62	2.89	3.94	1.26	2.6	0.9	1
Solyc01g020510.1.1	Ycf2 (AHRD V1 *-*- A6Y9X1_	0	0	0	0	0	0	0	0	0	0	1	NA	0	0	0	0	0	0	0	0	1	NA	0	0	0	0	0	0	0	0	1	NA	0	0	0	0	0	0	0	1	NA
Solyc01g020520.2.1	Membrane-bound transcripti	3.51	2.17	4.32	2.38	3.09	3.06	3.38	2.95	3.29	3.17	1.02	1	3.55	2.92	2.71	3.06	2.33	2.21	3.6	2.71	0.89	1	1.73	1.24	1.82	1.6	0.8	1.04	1.78	1.21	0.76	1	1.95	2.22	1.88	2.02	2.38	2.95	2.67	1.32	1
Solyc01g020530.1.1	Unknown Protein (AHRD V1)	0	0	0	0	0	0	0	0	0	0	1	NA	0.37	0	0	0.12	0	0	0	0	0.08	1	0	0.32	0	0.11	0	0	0	0	0.09	1	0	0	0	0	0	0	0	1	NA
Solyc01g020550.1.1	Unknown Protein (AHRD V1)	0	0	0	0	0	0	0	0	0	0	1	NA	0	0	0	0	0	0	0	0	1	NA	0	0	0	0	0	0	0	0	1	NA	0	0	0	0	0	0	0	1	NA
Solyc01g020560.1.1	Unknown Protein (AHRD V1)	0	0	0	0	0	0	0	0	0	0	1	NA	0	0	0	0	0	0	0	0	1	NA	0	0	0	0	0	0	0	0	1	NA	0	0	0	0	0	0	0	1	NA
Solyc01g020570.1.1	Calcium-transporting ATPase	0.49	0	0	0	0.12	0	0	0	0	0	0.08	1	0	0	0	0	0	0.25	0	0.08	8.33	1	0	0	0	0	0	0	0	0	1	NA	0	0	0	0	0	0	0	1	NA
Solyc01g020580.1.1	Unknown Protein (AHRD V1)	0	0	0	0	0	0	0	0	0	0	1	NA	0	0	0	0	0	0	0	0	1	NA	0	0	0	0	0	0	0	0	1	NA	0	0	0	0	0	0	0	1	NA
Solyc01g020590.1.1	Unknown Protein (AHRD V1)	0	0	0	0	0	0	0	0	0	0	1	NA	0	0	0	0	0	0	0	0	1	NA	0	0	0	0	0	0	0	0	1	NA	0	0	0	0	0	0	0	1	NA
Solyc01g020600.1.1	Os11g0256400 protein (Fragr	0	0	0	0	0	0	0	0	0	0	1	NA	0	0.19	0	0.06	0.15	0.11	0.17	0.14	2.26	1	0	0	0	0	0.14	0	0.47	0.2	20.33	1	0	0	0	0	0	0	0	1	NA
Solyc01g021600.2.1	Disease resistance response p	0.29	0	0	0	0.07	0.32	0	0	0	0.08	1.1	1	0	0	0	0	0	0.3	0	0.1	10	1	0	0.23	0	0.08	0.19	0	0	0.06	0.83	1	0	0	0	0	0	0.25	0.12	12.5	1
Solyc01g021610.1.1	Unknown Protein (AHRD V1)	0	0	0	0	0	0	0	0	0	0	1	NA	0	0	0	0	0	0	0	0	1	NA	0	0	0	0	0	0	0	0	1	NA	0	0	0	0	0	0	0	1	NA
Solyc01g021620.1.1	Unknown Protein (AHRD V1)	0	0	0	0	0	0	0	0	0	0	1	NA	0	0	0	0	0	0	0	0	1	NA	0	0	0	0	0	0	0	0	1	NA	0	0	0	0	0	0	0	1	NA
Solyc01g021630.1.1	Ulp1 protease family C-termi	0	0	0	0	0	0	0	0	0	0	1	NA	0	0	0	0	0	0	0	0	1	NA	0	0	0	0	0	0	0	0	1	NA	0	0	0	0	0	0	0	1	NA
Solyc01g021640.2.1	DDB1- and CUL4-associated f	11.55	12.05	11.12	8.64	10.84	9.74	9.75	13.42	9.53	10.61	0.98	1	13.46	11.54	9.87	11.62	8.01	8.32	8.97	8.43	0.73	0.686586	8.9	7.46	8.96	8.44	7.85	9.26	8.71	8.61	1.02	1	19.44	15.43	17.15	17.34	12.15	11.64	11.89	0.69	1
Solyc01g021650.1.1	Unknown Protein (AHRD V1);	0	0	0	0	0	0	0	0	0	0	1	NA	0	0	0	0	0	0	0	0	1	NA	0	0	0	0	0	0	0	0	1	NA	0	0	0	0	0	0	0	1	NA
Solyc01g021660.1.1	Ulp1 protease family C-termi	0	0	0	0	0	0	0	0	0	0	1	NA	0	0	0	0	0	0	0	0	1	NA	0	0	0	0	0	0	0	0	1	NA	0	0	0	0	0	0	0	1	NA
Solyc01g021670.2.1	POT family domain containin	112.83	129.77	71.2	98.09	102.97	87.92	86.41	99.26	123.25	99.21	0.96	1	208.15	171.06	98.14	159.12	185.88	177.77	187.94	183.86	1.16	0.911565	106.9	68.42	55.06	76.79	66.98	65.34	70.75	67.69	0.88	1	26.11	58.8	64.56	49.82	47.69	59.49	53.59	1.08	1
Solyc01g021680.1.1	HAT family dimerisation dom	0	0	0	0	0	0	0	0	0	0	1	NA	0	0	0	0	0	0	0	0	1	NA	0	0	0	0	0	0	0	0	1	NA	0	0	0	0	0	0	0	1	NA
Solyc01g021690.1.1	Unknown Protein (AHRD V1)	0	0	0	0	0	0	0	0	0	0	1	NA	0	0	0	0	0	0	0	0	1	NA	0	0	0	0	0	0	0	0	1	NA	0	0	0	0	0	0	0	1	NA
Solyc01g021700.1.1	Unknown Protein (AHRD V1)	0	0	0	0	0	0	0	0	0	0	1	NA	0	0	0	0	0	0	0	0	1	NA	0	0	0	0	0	0	0	0	1	NA	0	0	0	0	0	0	0	1	NA
Solyc01g021710.1.1	HAT family dimerisation dom	0	0	0	0	0	0	0	0	0	0	1	NA	0	0	0	0	0	0	0	0	1	NA	0	0	0	0	0	0	0	0	1	NA	0	0	0	0	0	0	0	1	NA
Solyc01g021720.1.1	Unknown Protein (AHRD V1)	0	0	0	0	0	0	0	0	0	0	1	NA	0.71	0.66	0	0.46	0.53	0	0	0.18	0.39	1	0	0	0	0	0	0	0	0	1	1	3.23	0.55	3.5	2.43	0	0.66	0.33	0.14	1
Solyc01g021730.2.1	NAC domain protein IPR0034	0.25	0	0	0	0.06	0	0	0	0	0	0.16	1	0.46	0.43	0.21	0.37	0.52	0.13	0.19	0.28	0.76	1	0.2	0.4	0.25	0.28	0.33	0.38	0.54	0.42	1.47	1	1.76	0.54	0	0.77	0	0.22	0.11	0.14	1
Solyc01g021740.1.1	Mitochondrial 2-oxoglutarate	0	0	0	0	0	0	0.44	0	0.4	0.21	21	1	0	0	0.47	0.16	0.39	0	0	0.13	0.83	1	0	0	0	0	0	0	0.41	0.14	13.67	1	0	0	0	0	0	0	0	1	NA
Solyc01g022740.2.1	Kelch-like protein 21 (AHRD V	4.22	3.35	4.8	5.33	4.43	3.01	2.38	3.32	2.81	2.88	0.65	0.639388	5.18	3.01	3.18	3.79	4.28	4.45	2.96	3.9	1.03	1	3.29	3.93	2.84	3.35	3.56	2.74	4.61	3.64	1.08	1	2.36	3.61	4.63	3.53	1.93	3.3	2.62	0.74	1
Solyc01g022750.2.1	NADH dehydrogenase (AHRD	110.12	94.03	108.85	131.2	111.05	119.65	155.07	122.22	128.91	131.46	1.18	0.597807	102.43	100.03	80.86	94.44	86.7	93.23	81.08	87	0.92	1	181.13	196.84	180.2	186.06	140.12	195.49	156.15	163.92	0.88	1	63.47	96.91	86.19	82.19	148.48	143.95	146.21	1.78	0.583641
Solyc01g022760.1.1	Unknown Protein (AHRD V1)	0	0	0	0	0	0	0	0	0	0	1	NA	0	0	0	0	0	0	0	0	1	NA	0	0	0	0	0	0	0	0	1	NA	0	0	0	0	0	0	0	1	NA
Solyc01g022770.1.1	Unknown Protein (AHRD V1)	0	0	0	0	0	0	0	0	0	0	1	NA	0	0	0	0	0	0	0	0	1	NA	0	0	0	0	0	1.39	0	0.46	46.33	NA	2.61	0	0	0.87	0	0	0	0.01	1
Solyc01g022780.1.1	Unknown Protein (AHRD V1)	0	0	0	0	0	0	0	0	0	0	1	NA	0	0	0	0	0	0	0	0	1	NA	0	0	0	0	0	0	0	0	1	NA	0	0	0	0	0	0	0	1	NA
Solyc01g022790.1.1	Unknown Protein (AHRD V1)	0	0	0	0	0	0	0	0	0	0	1	NA	0	0	0	0	0	0	0	0	1	NA	0	0	0	0	0	0	0	0	1	1	0	0	0	0	0	0	0	1	NA
Solyc01g028800.1.1	Pumilio-family RNA binding p	0.71	1.03	1.78	0.88	1.1	0.59	0.84	0.41	0.83	0.67	0.61	0.799975	0.58	0.23	0.08	0.3	0.12	0	0.21	0.11	0.37	0.884464	0.22	0	0.53	0.25	0	0	0	0	0.04	1	0	0	0	0	0	0	0	1	NA
Solyc01g028810.2.1	chaperonin (AHRD V1 ***- B4	474.11	391.43	361.27	353.98	395.2	448.3	535.24	400.03	504.09	471.91	1.19	0.600071	336.48	362.91	429.05	376.15	635.71	600.94	578.93	605.19	1.61	0.016688	231.23	488.57	423.45	381.08	565.86	500.26	581.21	549.11	1.44	0.971257	552	849.69	809.71	737.13	1041.74	976.29	1009.01	1.37	1
Solyc01g028830.2.1	ATP binding / serine-threonin	29.66	31.41	27.96	31.35	30.09	23.88	22.47	22.57	20.93	22.46	0.75	0.553086	28.38	36.75	31.53	32.22	24.59	25.51	29.47	26.52	0.82	0.909851	11.71	14.59	13.03	13.11	12.21	8.4	11.8	10.8	0.82	1	15.03	22.29	19.4	18.91	14.2	17.49	15.84	0.84	1
Solyc01g028840.1.1	Helicase-like protein (AHRD V	0	0	0	0	0	0	0	0	0	0	1	NA	0	0	0	0	0	0	0	0	1	NA	0	0	0	0	0	0	0	0	1	NA	0	0	0	0	0	0	0	1	NA
Solyc01g028850.1.1	Unknown Protein (AHRD V1);	0	0	0	0	0	0	0	0	0	0	1	NA	0	0.23	0	0.08	0	0.14	0.21	0.12	1.52	1	0	0.22	0.27	0.16	0	0	0	0	0.06	1	0	0	0	0	0.24	0	0.12	12	1
Solyc01g028860.2.1	YTH domain family 2 (Predict	189.33	173.4	314.19	259.62	234.14	128.51	122.66	114.02	150.8	129	0.55	0.110888	202.93	186.8	272.14	220.62	246.97	212.48	221.97	227.14	1.03	1	145.5	132.68	168.85	149.01	161.77	139.75	135.04	145.52	0.98	1	329.42	296.57	346.53	324.17	344.58	419.79	382.19	1.18	1
Solyc01g028870.2.1	N utilization substance protei	34.57	41.02	39.45	36.75	37.95	61.28	54.4	58.37	44.44	54.62	1.44	0.20436	31.31	38.49	47.71	39.17	60.33	50.42	61.43	57.39	1.47	0.190327	26.47	30.38	24	26.95	28.5	33.05	36.06	32.54	1.21	1	56.54	52.84	57.32	55.57	55.63	50.12	52.88	0.95	1
Solyc01g028880.1.1	Unknown Protein (AHRD V1)	0	0	0	0	0	0	0	0	0	0	1	NA	0	0	0	0	0	0	0	0	1	NA	0	0	0	0	0	0	0	0	1	NA	0	0	0	0	0	0	0	1	NA
Solyc01g028890.1.1	Unknown Protein (AHRD V1)	0	0	0	0	0	0.31	0	0	0	0.08	7.75	1	0	0	0	0	0	0	0	0	1	NA	0	0	0	0	0	0	0	0	1	NA	0	0	0	0	0	0	0	1	NA
Solyc01g028900.2.1	1-deoxy-D-xylulose-5-phosph	106.03	122.66	120.76	113.33	115.69	107.04	89.95	104.08	107.26	102.08	0.88	0.9965	118.46	116.45	112.84	115.92	106.81	101.71	123.33	110.62	0.95	1	122.63	107.81	116.19	115.54	91.63	108.22	99.36	99.74	0.86	1	171.66	190.19	180.29	180.71	130.54	150	140.27	0.78	1
Solyc01g028910.1.1	Protein kinase-like protein (Fr	0	0	0	0	0	0	0	0	0	0	1	NA	0	0	0	0	0	0	0	0	1	NA	0	0	0	0	0	0	0	0	1	NA	0	0	0	0	0	0	0	1	NA
Solyc01g028920.1.1	Unknown Protein (AHRD V1)	0	0	0	0	0	0	0	0	0	0	1	NA	0	0	0	0	0	0	0	0	1	NA	0	0	0	0	0	0	0	0	1	NA	0	0	0	0	0	0	0	1	NA
Solyc01g028930.2.1	TATA-box binding protein (AH	38.16	35.11	35.48	31.56	35.08	39.9	39.06	39.55	30.88	37.35	1.06	1	35.8	47.51	39.88	41.06	31.4	37.17	33.55	34.04	0.83	0.979744	44.42	44.01	32.74	40.39	36.33	36.28	33.7	35.44	0.88	1	43.22	59.19	42.27	48.23	38.48	33.74	36.11	0.75	1
Solyc01g028940.1.1	Unknown Protein (AHRD V1)	0	1.43	0	0	0.36	0	0	0	1.16	0.29	0.81	1	1.5	0	0	0.5	0	0	1.26	0.42	0.84	1	0	0	0	0	0	0	0	0	1	NA	0	0	0	0	0	0	0	1	NA
Solyc01g028950.1.1	Unknown Protein (AHRD V1)	0	0	0	0	0	0	0	0	0	0	1	NA	0	0	0	0	0	0	0	0	1	NA	0	0	0	0	0	0	0	0	1	NA	0	0	0	0	0	0	0	1	NA
Solyc01g028960.1.1	Unknown Protein (AHRD V1)	0	0	0	0	0	0	0	0	0	0	1	NA	0	0	0	0	0	0	0	0	1	NA	0	0	0	0	0	0	0	0	1	NA	0	0	0	0	0	0	0	1	NA
Solyc01g028970.1.1	Retrotransposon gag protein	0.12	0.1	0.14	0	0.09	0.13	0	0	0.08	0.05	0.58	1	0.11	0.1	0.3	0.17	0	0.06	0	0.02	0.12	0.913866	0	0	0	0	0	0	0	0	1	NA	0	0	0	0	0	0	0	1	NA
Solyc01g028980.1.1	Unknown Protein (AHRD V1)	0	0	0	0	0	0	0	0	0	0	1	NA	0	0	0	0	0	0	0	0	1	NA	0	0	0	0	0	0	0	0	1	NA	0	0	0	0	0	0	0	1	NA
Solyc01g033980.2.1	Unknown Protein (AHRD V1)	9.96	11.08	17.62	29.93	17.15	17.99	17.69	17.17	23.23	19.02	1.11	0.967597	11.58	25.97	30.49	22.68	25.04	19.33	10.76	18.38	0.81	1	6.03	26.37	27.18	19.86	22.9	19.81	14.51	19.07	0.96	1	24.78	9.04	15.31	16.38	25.07	40.38	32.73	2	0.700272
Solyc01g033990.1.1	Genomic DNA chromosome 5	0	0	0	0	0	0	0	0	0	0	1	NA	0	0	0	0	0	0	0	0	1	NA	0	0	0	0	0	0	0	0	1	NA	0	0	0	0	0	0	0	1	NA
Solyc01g034000.1.1	Mutator-like transposase (AH	0	0	0	0	0	0.1	0	0	0	0.03	2.5	1	0	0	0	0	0	0	0	0	1	NA	0	0	0	0	0	0	0	0	1	NA	0	0	0	0	0	0	0	1	NA
Solyc01g034010.1.1	Serine/threonine-protein pho	0	0.55	0	0.35	0.23	0.42	0.69	0.11	0.71	0.48	2.14	0.804781	0	0.43	0	0.14	0.6	0.32	0.2	0.37	2.6	0.905031	0	0.2	0.37	0.19	0.16	0.19	0.45	0.27	1.4	1	0.18	0	0.19	0.12	0.57	0.33	0.45	3.65	1
Solyc01g034020.2.1	Serine/threonine-protein pho	74.14	77.65	65.41	86.63	75.96	73.57	64.81	57.3	65.1	65.19	0.86	0.955733	73.96	68.74	65.7	69.47	58.87	62.53	67.89	63.1	0.91	1	74.48	89.3	85.75	83.18	63.95	66.94	74.07	68.32	0.82	1	66.3	48.54	48.58	54.47	63.54	63.68	63.61	1.17	1
Solyc01g034030.1.1	Unknown Protein (AHRD V1)	0	0	0	0	0	0	0	0	0	0	1	NA	0	0	0	0	0	0	0	0	1	NA	0	0	0	0	0	0	0	0	1	NA	0	0	0	0	0	0	0	1	NA
Solyc01g034040.1.1	HGWP repeat containing prot	0	0.08	0	0.13	0.05	0	0	0	0	0	0.19	1	0	0	0	0	0	0	0	0	1	NA	0	0	0	0	0	0	0	0	1	NA	0	0	0	0	0	0	0	1	NA
Solyc01g034050.1.1	Histone-lysine N-methyltrans	0	0	0.52	0	0.13	0	0	0	0	0	0.08	1	0	0.77	0	0.26	0	0.23	0	0.08	0.3	1	0	0	0	0	0	0	0	0	1	NA	0.63	0	0	0.21	0	0	0	0.05	1
Solyc01g034060.1.1	Unknown Protein (AHRD V1)	0	0	0	0	0	0	0	0	0	0	1	NA	0	0	0	0	0	0	0	0	1	NA	0	0	0	0	0	0	0	0	1	NA	0	0	0	0	0	0	0	1	NA
Solyc01g034070.1.1	Unknown Protein (AHRD V1);	0	0	0	0	0	0	0	0	0	0	1	NA	0	0	0	0	0	0	0	0	1	NA	0	0	0	0	0	0	0	0	1	NA	0	0	0	0	0	0	0	1	NA
Solyc01g034080.1.1	Amino acid permease (Fragm	0.56	0.62	0.33	0	0.38	0.47	0.77	0.77	0.7	0.68	1.79	0.918106	0.52	0.12	0.83	0.49	0.1	0.51	0.77	0.46	0.94	1	0.34	0.23	0	0.19	0.28	0.21	0.31	0.27	1.4	1	0	0	0	0	0	0	0	1	NA
Solyc01g034090.1.1	Gag and Pol (AHRD V1 ***- Q	0	0	0	0	0	0	0	0	0	0	1	NA	0	0	0	0	0	0	0	0	1	NA	0	0	0	0	0	0	0	0	1	NA	0	0	0	0	0	0	0	1	NA
Solyc01g034100.1.1	Ulp1 protease family C-termi	0.28	0.49	0	0	0.19	0.62	0.22	0.89	0.2	0.48	2.51	0.912648	0.26	0	0.35	0.2	0.1	0.43	0.44	0.32	1.59	1	0.34	0.11	0.14	0.2	0.27	0.32	0.4	0.33	1.68	1	0.99	0.91	1.07	0.99	0.25	0.37	0.31	0.31	0.994216
Solyc01g034110.1.1	MuDR family transposase cor	0	0	0	0	0	0	0	0	0	0	1	NA	0	0	0	0	0	0	0	0	1	NA	0	0	0	0	0	0	0	0	1	NA	0	0	0	0	0	0	0	1	NA
Solyc01g034120.2.1	Mutator-like transposase (AH	0	0	0	0	0	0	0	0	0	0	1	NA	0	0	0	0	0	0	0	0	1	NA	0	0	0	0	0	0	0	0	1	NA	0	0	0	0	0	0	0	1	NA
Solyc01g034130.1.1	Ulp1 protease family C-termi	0.24	0.32	0.14	0	0.18	0.13	0	0.11	0.17	0.1	0.59	1	0	0	0	0	0	0	0.09	0.03	3	1	0	0	0.12	0.04	0	0	0	0	0.25	1	0.17	0	0	0.06	0	0.11	0.06	0.97	1
Solyc01g034140.1.1	Unknown Protein (AHRD V1)	0	0	0	0	0	0	0	0	0	0	1	NA	0	0	0	0	0	0	0	0	1	NA	0	0	0	0	0	0	0	0	1	NA	0	0	0	0	0	0	0	1	NA
Solyc01g034150.2.1	DNA polymerase IV (AHRD V1	5.08	5.51	6	4.71	5.33	5.75	3.65	5.18	5.04	4.91	0.92	1	5.41	5.38	7.02	5.94	5.32	6.17	6.32	5.94	1	1	4.48	5.57	4.95	5	4.49	3.78	4.11	4.13	0.83	1	11.3	9.6	15.63	12.18	7.78	10.29	9.04	0.74	1
Solyc01g034160.1.1	Pol polyprotein (AHRD V1 ***	0	0	0	0	0	0	0	0	0.16	0.04	4	1	0	0	0	0	0	0	0	0	1	NA	0	0	0	0	0	0	0	0	1	NA	0	0	0	0	0	0	0	1	NA
Solyc01g034170.1.1	Unknown Protein (AHRD V1)	0	0	0	0	0	0	0	0	0	0	1	NA	0	0	0	0	0	0	0	0	1	NA	0	0	0	0	0	0	0	0	1	NA	0	0	0	0	0	0	0	1	NA
Solyc01g034180.1.1	Unknown Protein (AHRD V1);	0	0	0	0	0	0	0	0	0	0	1	NA	0	0	0	0	0	0	0	0	1	NA	0	0	0	0	0	0	0	0	1	NA	0.09	0	0	0.03	0	0	0	0.33	1
Solyc01g034190.1.1	Unknown Protein (AHRD V1);	0	0	0	0	0	0	0	0	0	0	1	NA	0	0	0	0	0	0	0	0	1	NA	0	0	0	0	0	0	0	0	1	NA	0	0	0	0	0	0	0	1	NA
Solyc01g034200.1.1	Uncharacterized mitochondri	0	0	0	0	0	0	0	0	0	0	1	NA	0	0	0	0	0	0	0	0	1	NA	0	0	0	0	0	0	0	0	1	NA	0	0	0	0	0	0	0	1	NA
Solyc01g034210.1.1	Polyprotein (AHRD V1 ***- Q	0	0	0	0	0	0	0	0	0	0	1	NA	0	0	0	0	0	0	0	0	1	NA	0	0	0	0	0	0	0	0	1	NA	0	0	0	0	0	0	0	1	NA
Solyc01g034220.1.1	Unknown Protein (AHRD V1);	0	0	0	0	0	0	0.06	0	0	0.01	1.5	1	0	0	0	0	0	0	0	0	1	NA	0	0	0	0	0	0	0	0	1	NA	0	0	0	0	0	0	0	1	NA
Solyc01g035220.1.1	NAD(P)H-quinone oxidoreduc	2.13	0	0	0	0.53	0	0	0	0	0	0.02	1	0	0	0	0	0	0	1.67	0.56	55.67	1	0	0	0	0	0	0	1.55	0.52	51.67	1	0	0	0	0	0.97	0	0.48	48.5	1
Solyc01g038220.1.1	Unknown Protein (AHRD V1)	0.6	0	0	0	0.15	0.33	0	0	0	0.08	0.55	1	0.56	0	0.25	0.27	0.21	0.16	0	0.12	0.46	1	0	0	0.3	0.1	0	0	0	0	0.1	1	0	0.22	0	0.07	0	0	0	0.14	1
Solyc01g038230.1.1	Unknown Protein (AHRD V1)	0	0	0	0	0	0	0	0	0	0	1	NA	0	0	0	0	0	0	0	0	1	NA	0	0	0	0	0	0	0	0	1	NA	0	0	0	0	0	0	0	1	NA
Solyc01g044240.2.1	Unknown Protein (AHRD V1)	464.87	599.43	546.63	427.52	509.61	665.1	658.91	835.08	577.16	684.06	1.34	0.347264	720.68	1637.73	1337.3	1231.9	1819.61	2325.1	3163.74	2436.15	1.98	0.103573	780.89	950.13	776.65	835.89	1380.78	1113.99	746.82	1080.53	1.29	1	1834.51	1827.46	1439.13	1700.37	532.28	817.87	675.08	0.4	0.03644
Solyc01g044250.1.1	Photosystem II CP43 chloroph	2.67	6.35	2.1	2.52	3.41	1.98	6.33	6.56	7.04	5.48	1.61	0.89726	1.66	3.1	4.52	3.09	6.18	4.15	1.4	3.91	1.26	1	2.16	1.45	0	1.2	0.59	0	0.65	0.41	0.34	1	5.07	7.77	4.11	5.65	0.82	3.13	1.97	0.35	1
Solyc01g044270.2.1	Chaperone protein dnaJ 49 (A	6.94	6.41	7.89	6.59	6.96	5.46	4.55	6.31	5.78	5.53	0.79	0.839472	6.07	7.73	9.44	7.75	9.28	9.69	8.05	9.01	1.16	0.895243	6.02	9.66	13.11	9.6	11.07	8.92	12.65	10.88	1.13	1	12.52	14.09	14.72	13.78	11.16	12.4	11.78	0.86	1
Solyc01g044290.1.1	Ulp1 protease family C-termi	0.08	0	0	0	0.02	0	0.13	0	0	0.03	1.62	1	0	0	0	0	0	0.09	0.13	0.07	7.33	0.990265	0	0	0	0	0	0	0	0	1	NA	0	0	0	0	0	0	0	1	NA
Solyc01g044300.1.1	Unknown Protein (AHRD V1)	0	0	0	0	0	0	0	0	0	0	1	NA	0	0	0	0	0	0	0	0	1	NA	0	0	0	0	0	0	0	0	1	NA	0	0	0	0	0	0	0	1	NA
Solyc01g044320.1.1	Ulp1 protease family C-termi	0.73	0.43	1.15	0.34	0.66	1.08	1.15	0.9	0.17	0.82	1.25	1	0.91	1.06	0	0.66	0.17	0.38	0.38	0.31	0.47	1	0	0	0	0	0	0	0.35	0.12	11.67	1	0.35	0.53	0.37	0.42	0.67	0.43	0.55	1.32	1
Solyc01g044330.1.1	Ulp1 protease family C-termi	0	0	0	0	0	0.14	0.1	0.12	0.18	0.14	13.5	0.804781	0	0	0	0	0	0.13	0.1	0.08	7.67	1	0	0	0	0	0	0	0	0	1	1	0	0	0	0	0	0	0	1	NA
Solyc01g044340.2.1	Os09g0513800 protein (Fragr	55.45	61.27	99.83	87.91	76.12	47.29	46.04	59.78	62.12	53.81	0.71	0.588036	81.38	98.07	113.86	97.77	86.21	96.04	90.16	90.8	0.93	1	61.02	82.19	68.33	70.51	72.86	56.16	66.54	65.19	0.92	1	128.46	130.9	133.06	130.81	99.34	93.47	96.41	0.74	1
Solyc01g044350.2.1	Zinc finger (Ran-binding) fam	17.49	17.54	34.74	32.88	25.66	16.24	14.86	15.88	18.6	16.39	0.64	0.586137	26.15	29.64	41.7	32.5	26.87	27.62	27.84	27.44	0.84	0.982244	17.89	27.06	22.19	22.38	20.94	17.28	18.23	18.82	0.84	1	45.32	43.25	43.23	43.93	33.44	30.47	31.95	0.73	1
Solyc01g044360.2.1	Importin beta-3 (AHRD V1 **	69.29	64.36	114.02	84.34	83	47.15	56.58	46.36	60.44	52.63	0.63	0.289873	74.94	75.96	93.1	81.33	93.69	79.43	70.31	81.14	1	1	38.82	57.63	57	51.15	61.38	49.78	54.65	55.27	1.08	1	122.81	108.8	126.26	119.29	126.32	165.47	145.89	1.22	1
Solyc01g044370.1.1	MOSC domain containing pro	4.18	3.45	2.11	0.42	2.54	2.32	5.89	5.76	4.07	4.51	1.78	0.556258	1.94	3.11	4.29	3.11	1.66	2.63	3.28	2.52	0.81	1	0.96	1.46	0.3	0.91	1.18	1.58	0.87	1.21	1.33	1	0	0.43	3.67	1.37	0.82	2.09	1.45	1.06	1

Solyc01g044380.1.1	Unknown Protein (AHRD V1)	0	0	0	0	0	0	0	0	0	0	1	NA	0	0	0.32	0.11	0	0	0	0	0.09	1	0	0	0	0	0	0	0	0	1	NA	0	0.28	0	0.09	0	0.67	0.34	3.59	1
Solyc01g044390.1.1	Unknown Protein (AHRD V1)	0	0	0	0	0	0	0	0	0	0	1	NA	0	0	0	0	0	0	0	0	1	NA	0	0	0	0	0	0	0	0	1	NA	0	0	0	0	0	0	0	1	NA
Solyc01g044400.1.1	Unknown Protein (AHRD V1)	0	0	0	0	0	0	0	0	0	0	1	NA	0	0	0	0	0	0	0	0	1	NA	0	0	0	0	0	0	0	0	1	NA	0	0	0	0	0	0	0	1	NA
Solyc01g044410.1.1	Unknown Protein (AHRD V1)	0	0	0	0	0	0	0	0	0	0	1	NA	0	0	0	0	0	0	0	0	1	NA	0	0	0	0	0	0	0	0	1	NA	0	0	0	0	0	0	0	1	NA
Solyc01g044420.2.1	Calmodulin binding protein (A	0.46	0	0	0	0.12	0	0	0	0	0	0.09	0.942294	0	0	0	0	0	0	0	0	1	NA	0	0	0	0	0	0	0	0	1	NA	0	0	0	0	0	0	0	1	NA
Solyc01g044430.1.1	Ulp1 protease family C-termi	0	0	0	0	0	0	0	0	0	0	1	NA	0	0	0	0	0	0	0	0	1	NA	0	0	0	0	0	0.12	0	0.04	4	NA	0	0	0	0	0	0	0	1	NA
Solyc01g044440.1.1	Mutator-like transposase (AH	0	0	0	0	0	0	0	0	0	0	1	NA	0	0	0	0	0	0	0	0	1	NA	0	0	0	0	0	0	0	0	1	NA	0	0	0	0	0	0	0	1	NA
Solyc01g044460.1.1	Unknown Protein (AHRD V1)	0	0	0	0	0	0	0	0	0	0	1	NA	0	0	0	0	0	0	0	0	1	NA	0	0	0	0	0	0	0	0	1	NA	0	0	0	0	0	0	0	1	NA
Solyc01g044470.1.1	Unknown Protein (AHRD V1)	0	0	0	0	0	0	0	0	0	0	1	NA	0	0	0	0	0	0	0	0	1	NA	0	0	0	0	0	0	0	0	1	NA	0	0	0	0	0	0	0	1	NA
Solyc01g044480.2.1	Elongation factor P (AHRD V1	27.4	35.29	28.07	27.03	29.45	24.44	31.59	30.16	30.28	29.12	0.99	1	27.83	27.89	28.01	27.91	24.75	31.92	32.01	29.56	1.06	1	44.27	49.06	41.51	44.95	44.05	42.35	49.91	45.44	1.01	1	34.34	44.25	44.35	40.98	40.92	38.25	39.59	0.97	1
Solyc01g044490.2.1	Elongation factor P family pro	111.64	158.08	102.98	93.32	116.51	127.48	153.84	162.37	131.89	143.89	1.24	0.71533	113.29	115.58	92.58	107.15	95.74	130.32	142.77	122.94	1.15	0.910379	181.22	190.22	163.2	178.21	174.02	180.77	193.04	182.61	1.02	1	121.22	221.88	213.82	185.64	154.23	145.94	150.08	0.81	1
Solyc01g044500.1.1	Unknown Protein (AHRD V1)	0	0	0	0	0	0	0	0	0	0	1	NA	0	0	0	0	0	0	0	0	1	NA	0	0	0	0	0	0	0	0	1	NA	0	0	0	0	0	0	0	1	NA
Solyc01g044510.1.1	Unknown Protein (AHRD V1)	0	0	0	0	0	0	0	0	0	0	1	NA	0	0	0	0	0	0	0	0	1	NA	0	0	0	0	0	0	0	0	1	NA	0	0	0	0	0	0	0	1	NA
Solyc01g044520.1.1	LOB domain protein 1 (AHRD	0	0	0	0	0	0	0	0	0	0	1	NA	0	0	0	0	0	0	0	0	1	NA	0	0	0	0	0	0	0	0	1	NA	0	0	0	0	0	0	0	1	NA
Solyc01g044540.1.1	Unknown Protein (AHRD V1)	5.17	3.45	1.53	1.83	3	1.44	3.06	1.19	2.79	2.12	0.71	1	4.81	2.25	2.19	3.08	0.9	1.34	3.05	1.76	0.57	1	0	3.16	0	1.05	0.85	0	3.77	1.54	1.46	1	1.84	2.82	0	1.55	1.18	3.4	2.29	1.47	1
Solyc01g044550.1.1	Anthocyanidin synthase (AHR	0	0	0	0	0	0	0	0	0	0	1	NA	0	0	0	0	0	0	0	0	1	NA	0	0	0	0	0	0	0	0	1	NA	0	0	0	0	0	0	0	1	NA
Solyc01g044560.1.1	1-AMINOCYCLOPROPANE-1-C	0	0	0	0	0	0	0	0	0	0	1	NA	0	0	0	0	0	0	0	0	1	NA	0	0	0	0	0	0	0	0	1	NA	0	0	0	0	0	0	0	1	NA
Solyc01g045570.1.1	Ulp1 protease family C-termi	0.61	0	0	0.86	0.37	0.68	0.48	1.12	0.44	0.68	1.85	1	1.14	0.53	0	0.56	0.42	0	0	0.14	0.25	1	0	0	0	0	0	0	0	0	1	1	0	0	0	0	0	0	0	1	NA
Solyc01g045580.1.1	Ulp1 protease family C-termi	0	0	0	0	0	0	0	0	0	0	1	NA	0	0	0	0	0	0	0	0	1	NA	0	0	0	0	0	0	0	0	1	NA	0	0	0	0	0	0	0	1	NA
Solyc01g047580.1.1	Unknown Protein (AHRD V1);	0	0	0	0	0	0	0	0	0	0	1	NA	0	0	0	0	0.24	0	0	0.08	8	1	0	0	0	0	0	0	0	0	1	NA	0	0	0	0	0	0	0	1	NA
Solyc01g047590.1.1	Signal recognition particle pro	1.95	1.74	1.15	1.61	1.61	1.09	1.29	1.35	0.7	1.11	0.69	0.902661	1.06	1.27	1.65	1.33	0.68	0.59	0.9	0.72	0.55	0.817649	1.05	1.59	2.1	1.58	0.64	0.99	1.07	0.9	0.57	1	0.93	0.83	1.5	1.09	1.04	1	1.02	0.94	1
Solyc01g048590.1.1	Photosystem Q(B) protein (AH	62.04	43.34	63.93	72.21	60.38	65.48	41.54	48.38	60.33	53.93	0.89	1	31.41	37.55	49.09	39.35	83.34	56.52	47.52	62.46	1.59	0.401947	35.53	40.32	45.21	40.35	66.56	45.24	54.94	55.58	1.38	0.967387	69.23	18.25	12.08	33.19	35.95	60.6	48.28	1.45	1
Solyc01g048600.1.1	Unknown Protein (AHRD V1)	0	0	0	0	0	0	0	0	0	0	1	NA	0	0	0	0	0	0	0	0	1	NA	0	0	0	0	0	0	0	0	1	NA	0	0	0	0	0	0	0	1	NA
Solyc01g048610.1.1	Unknown Protein (AHRD V1)	0	0	0	0	0	0	0	0	0	0	1	NA	0	0	0	0	0	0	0	0	1	NA	0	0	0	0	0	0	0	0	1	NA	0	0	0	0	0	0	0	1	NA
Solyc01g049610.1.1	Unknown Protein (AHRD V1)	0	0	0	0	0	0	0	0.46	0	0.12	11.5	1	0	0	0	0	0	0	0	0	1	NA	0	0	0	0	0	0	0	0	1	NA	0	0	0	0	0	0	0	1	NA
Solyc01g049630.1.1	Unknown Protein (AHRD V1)	0	0	0	0	0	0	0	0	0	0	1	NA	0	0	0	0	0	0	0	0	1	NA	0	0	0	0	0	0	0	0	1	NA	0	0	0	0	0	0	0	1	NA
Solyc01g049640.1.1	Polynucleotidyl transferase R	0	0	0	0	0	0	0	0	0	0	1	NA	0	0	0	0	0	0	0	0	1	NA	0	0	0	0	0	0	0	0	1	NA	0	0	0	0	0	0	0	1	NA
Solyc01g049650.2.1	Pyruvate kinase (AHRD V1 **	105.97	129.8	132.45	140.99	127.3	87.04	87.7	75.37	98.87	87.25	0.69	0.277797	90.89	90.98	104.13	95.33	68.41	73.48	76.68	72.86	0.76	0.624558	18.33	33.02	17.05	22.8	13.7	13.92	18.25	15.29	0.67	1	7.65	6	5.04	6.23	9.6	15.06	12.33	1.98	0.450081
Solyc01g049660.1.1	S-locus F-box-like protein b (A	0	0	0	0	0	0	0	0	0	0	1	NA	0	0	0	0	0	0	0	0	1	NA	0	0	0	0	0	0	0	0	1	NA	0	0	0	0	0	0	0	1	NA
Solyc01g049670.1.1	Unknown Protein (AHRD V1)	0	0	0	0	0	0	0	0	0	0	1	NA	0	0	0	0	0	0	0	0	1	NA	0	0	0	0	0	0	0	0	1	NA	0	0	0	0	0	0	0	1	NA
Solyc01g049680.2.1	Transcriptional activator prot	126.08	128.52	140.1	115.61	127.58	144.22	111.4	151.98	114.72	130.58	1.02	1	111.82	108.32	103.7	107.95	74.48	90.71	92.74	85.98	0.8	0.83225	122.29	104.23	117.87	114.8	97.75	85	91.61	91.45	0.8	1	136.01	177.55	168.51	160.69	93.27	83.55	88.41	0.55	0.40203
Solyc01g049690.2.1	Transcriptional activator prot	79.05	85.03	79.17	83.66	81.73	52.47	52.08	60.66	62.06	56.82	0.7	0.410664	80.94	93.44	57.16	77.18	49.7	51.83	52.74	51.42	0.67	0.357154	73.52	73.22	71.69	72.81	56.04	61.64	56.36	58.01	0.8	1	44.47	87.16	62.09	64.57	46.85	43.38	45.12	0.7	0.887712
Solyc01g049700.2.1	Retrotransposon gag protein	0	0	0	0	0	0	0	0	0	0	1	NA	0	0	0	0	0.19	0	0	0.06	6.33	1	0	0	0	0	0	0	0	0	1	NA	0	0.2	0	0.07	0	0	0	0.15	1
Solyc01g049710.1.1	Mutator-like transposase (AH	0	0	0	0	0	0	0	0	0	0	1	NA	0	0	0	0	0.16	0.24	0.18	0.19	19.33	0.994791	0	0.19	0	0.06	0	0.18	0	0.06	0.95	1	0.33	0.85	1.08	0.75	0	0.41	0.2	0.27	1
Solyc01g049720.1.1	Unknown Protein (AHRD V1)	0	0	0	0	0	0	0	0	0	0	1	NA	0	0	0	0	0	0	0	0	1	NA	0	0	0	0	0	0	0	0	1	NA	0	0	0	0	0	0	0	1	NA
Solyc01g049740.1.1	DNA repair and recombinatio	0	0	0	0	0	0	0	0	0	0	1	NA	0	0	0	0	0	0	0	0	1	NA	0	0	0	0	0	0	0	0	1	NA	0	0	0	0	0	0	0	1	NA
Solyc01g049750.1.1	Unknown Protein (AHRD V1)	0	0	0	0	0	0	0	0	0	0	1	NA	0	0	0	0	0	0	0	0	1	NA	0	0	0	0	0	0	0	0	1	NA	0	0	0	0	0	0	0	1	NA
Solyc01g049760.1.1	UDP-glucosyltransferase fami	0	0	0	0	0	0	0	0	0	0	1	NA	0	0	0	0	0	0	0	0	1	NA	0	0	0	0	0	0	0	0	1	NA	0	0	0	0	0	0	0	1	NA
Solyc01g049770.2.1	Unknown Protein (AHRD V1)	10.46	8.59	13.29	9.1	10.36	24.15	7.62	16.27	10.97	14.75	1.42	0.83242	11.23	12.59	21.07	14.96	8.93	9.58	6.95	8.49	0.57	0.628752	4.55	13.11	12.78	10.15	7.93	6.1	1.76	5.26	0.52	1	10.29	11.69	9.9	10.63	0.74	2.82	1.78	0.17	0.32208
Solyc01g049780.1.1	Unknown Protein (AHRD V1)	0	0	0	0	0	0	0	0	0	0	1	NA	0	0	0	0	0	0	0	0	1	NA	0	0	0	0	0	0	0	0	1	NA	0	0	0	0	0	0	0	1	NA
Solyc01g049790.1.1	UDP-glucose glucosyltransfer	0	0	0	0	0	0	0	0	0	0	1	NA	0	0	0	0	0	0	0	0	1	NA	0	0	0	0	0	0	0	0	1	NA	0	0	0	0	0	0	0	1	NA
Solyc01g049800.1.1	Unknown Protein (AHRD V1)	0	0	0	0	0	0	0	0	0	0	1	NA	0	0	0	0	0	0	0	0	1	NA	0	0	0	0	0	0	0	0	1	NA	0	0	0	0	0	0	0	1	NA
Solyc01g049810.2.1	Pentatricopeptide repeat-con	0.23	0.63	0.41	0	0.32	0.39	0.65	0.65	0.34	0.51	1.6	1	0.33	0.31	0.1	0.25	0.16	0.24	0.83	0.41	1.66	1	0.28	0.29	0.12	0.23	0.15	0	0.51	0.22	0.96	1	0.17	0.51	0	0.23	0.21	0.51	0.36	1.59	1
Solyc01g049820.2.1	Unknown Protein (AHRD V1)	0	0	0	0	0	0	0	0	0	0	1	NA	0	0	0	0	0	0	0	0	1	NA	0	0	0	0	0	0	0	0	1	1	0	0	0	0	0	0	0	1	NA
Solyc01g049830.2.1	BY-inesin-like protein 5 (AHRI	0	0	0	0	0	0	0	0	0	0	1	NA	0	0	0	0	0	0	0	0	1	NA	0	0	0	0	0	0	0	0	1	NA	0	0	0	0	0	0	0	1	NA
Solyc01g049840.1.1	BY-inesin-like protein 5 (AHRI	0	0	0	0	0	0	0	0	0	0	1	NA	0	0	0	0	0	0	0	0	1	NA	0	0	0	0	0	0	0	0	1	NA	0	0	0	0	0	0	0	1	NA
Solyc01g049850.1.1	Ulp1 protease family C-termi	0	0	0	0	0	0	0	0	0	0	1	NA	0	0	0	0	0	0	0	0	1	NA	0	0	0	0	0	0	0	0	1	NA	0	0	0	0	0	0	0	1	NA
Solyc01g049860.1.1	Mutator-like transposase (AH	0	0	0	0	0	0	0	0	0	0	1	NA	0	0	0	0	0	0	0	0	1	NA	0	0	0	0	0	0	0	0	1	NA	0	0	0	0	0	0	0	1	NA
Solyc01g049880.2.1	Unknown Protein (AHRD V1)	51.54	65.14	65.67	81.65	66	72.19	84.63	88.14	68.82	78.44	1.19	0.751742	54.65	53.76	57.46	55.29	69.35	76.32	61.57	69.08	1.25	0.751893	24.15	40.32	40.93	35.13	20.31	13.28	17.28	16.96	0.48	1	17.59	20.96	15.85	18.13	16.98	37.96	27.47	1.51	1
Solyc01g049890.2.1	Unknown Protein (AHRD V1)	446.85	532.78	472.46	533.65	496.43	535.09	501.09	467.48	427.19	482.71	0.97	1	537.9	469.53	448.81	485.41	396.26	414.29	509.94	440.16	0.91	1	411.61	381.34	352.73	381.89	293.89	345.71	344.41	328	0.86	1	380.69	376.77	345.55	367.67	374.85	438.34	406.6	1.11	1
Solyc01g049900.2.1	Membrane-bound transcripti	2.8	2.63	3.12	1.1	2.41	2.77	2.21	2.43	4.02	2.86	1.18	0.999887	3.47	3.92	2.89	3.43	4.1	3.71	4.52	4.11	1.2	1	1.89	2.03	2.16	2.03	1.02	1.53	1.81	1.45	0.72	1	2.65	3.39	4.07	3.37	2.99	3.27	3.13	0.93	1
Solyc01g049910.2.1	Unknown Protein (AHRD V1)	0	3.09	0	0	0.77	1.93	0	1.6	1.25	1.2	1.55	1	0	4.53	4.4	2.98	3.61	1.8	1.36	2.26	0.76	1	1.4	0	0	0.47	2.28	5.26	1.27	2.94	6.29	1	0	0	0	0	0	3.04	1.52	152	1
Solyc01g049920.1.1	Unknown Protein (AHRD V1)	0	1.59	5.26	0	1.71	4.95	0.7	9.02	1.28	3.99	2.33	0.917205	5.8	3.1	1.51	3.47	1.85	0.92	4.9	2.56	0.74	1	2.88	2.18	1.77	2.28	4.69	2.03	2.6	3.11	1.36	1	0	0	0	0	0	0	0	1	NA
Solyc01g049930.2.1	Unknown Protein (AHRD V1)	0	0	0	0	0	0	0	0.65	0	0.16	16.25	1	0	0	0	0	0	0	0	0	1	NA	0	0	0	0	0	0	0	0	1	NA	3.99	1.53	1.08	2.2	3.85	1.23	2.54	1.15	1
Solyc01g049950.1.1	Ribosomal protein L5 (AHRD \	0	0	0	0	0	0	0	0	0	0	1	NA	0	0	0	0	0	0	0	0	1	NA	0	0	0	0	0	0	0	0	1	NA	0	0	0	0	0	0	0	1	NA
Solyc01g049960.2.1	Unknown Protein (AHRD V1)	144.06	131.81	178.7	174.25	157.2	193.81	166.35	171.45	153.69	171.32	1.09	0.917319	122.73	122.3	141.58	128.87	134	144.3	124.85	134.38	1.04	1	97.35	120.68	103.57	107.2	132.23	111.23	116.26	119.91	1.12	1	133.04	108.05	124.22	121.77	163.92	180.02	171.97	1.41	0.906678
Solyc01g049970.1.1	Ulp1 protease family C-termi	0	0	0	0	0	0	0	0	0	0	1	NA	0	0	0	0	0	0	0	0	1	NA	0	0	0	0	0	0	0	0	1	NA	0	0	0	0	0	0	0	1	NA
Solyc01g049980.2.1	Unknown Protein (AHRD V1)	0	0	0	0	0	0	0	0	0	0	1	NA	0	0	0	0	0	0	0	0	1	NA	0	0	0	0	0	0	0	0	1	NA	0	0	0	0	0	0	0	1	NA
Solyc01g049990.1.1	Unknown Protein (AHRD V1)	0	0	0	0	0	0	0	0	0	0	1	NA	0	0	0	0	0	0	0	0	1	NA	0	0	0	0	0	0	0	0	1	NA	0	0	0	0	0	0	0	1	NA
Solyc01g050000.2.1	HAT family dimerisation dom	5.43	7.6	3.2	5.49	5.43	5.61	6.13	6.42	4.18	5.58	1.03	1	5.05	6.75	5.24	5.68	6.46	5.63	10.67	7.59	1.34	0.957174	4.08	6.01	3.47	4.52	2.55	1.47	3.68	2.57	0.57	1	2.76	5.64	4.77	4.39	1.78	2.38	2.08	0.47	0.999777
Solyc01g050010.2.1	HAT family dimerisation dom	7.59	10.56	5.6	5.37	7.28	5.8	5.62	10.91	6.47	7.2	0.99	1	8.83	6.6	10.02	8.48	9.55	10.81	10.44	10.27	1.21	0.9964	4.22	6.19	8.01	6.14	2.18	3.24	4.84	3.42	0.56	1	6.07	8.96	8.76	7.93	6.08	5.41	5.75	0.72	1
Solyc01g050020.1.1	Photosystem II CP47 chloroph	0	0	0.82	0	0.2	0.77	0	0	0	0.19	0.94	1	0	0.6	0	0.2	0	0	0	0	0.05	1	0	0.57	0	0.19	0.46	0	0	0.15	0.81	1	0	0	0	0	0	0	0	1	NA
Solyc01g050040.2.1	RING finger protein 13 (AHRD	11.26	11.22	12.48	10.83	11.45	11.51	6.93	9.73	11.15	9.83	0.86	1	10.68	10.57	9.51	10.25	11.56	9.44	15.04	12.01	1.17	0.991848	9.82	9.36	11.17	10.12	10.8	12.8	13.62	12.41	1.23	1	10.88	10.47	7.62	9.66	10.72	11.25	10.98	1.14	1
Solyc01g050050.1.1	Unknown Protein (AHRD V1)	0	0	0	0	0	0	0	0	0	0	1	NA	0	0	0	0	0	0	0	0	1	NA	0	0	0	0	0	0	0	0	1	NA	0	0	0	0	0	0	0	1	NA
Solyc01g050060.1.1	Unknown Protein (AHRD V1)	0	0	0.82	0	0.2	0	0	0	0	0	0.05	1	0	0	0	0	0	0	0	0	1	NA	0	0	0	0	0	0	0	0	1	NA	0	0	0	0	0	0	0	1	NA
Solyc01g050070.2.1	Genomic DNA chromosome 3	14.33	22.05	15.5	22.8	18.67	19.25	12.96	20.86	18.86	17.98	0.96	1	18.6	16.87	16.64	17.37	16.15	15.92	18.06	16.71	0.96	1	15.19	9	8.59	10.93	8.63	7.24	12.62	9.5	0.87	1	7.22	7.15	11.02	8.46	8.74	12.82	10.78	1.27	1
Solyc01g050080.1.1	Unknown Protein (AHRD V1)	0	0	0	0	0	0	0	0	0	0	1	NA	0	0	0	0	0	0	0	0	1	NA	0	0	0	0	0	0	0	0	1	NA	0	0	0	0	0	0	0	1	NA
Solyc01g055160.1.1	F-box (AHRD V1 ***- Q84KQ8	0	0	0	0	0	0	0	0	0	0	1	NA	0	0	0	0	0	0	0	0	1	NA	0	0	0	0	0	0	0	0	1	NA	0	0	0	0	0	0	0	1	NA
Solyc01g055170.1.1	Unknown Protein (AHRD V1)	0	0	0	0	0	0	0	0	0	0	1	NA	0	0	0	0	0	0	0	0	1	NA	0	0	0	0	0	0	0	0	1	NA	0	0	0	0	0	0	0	1	NA
Solyc01g055200.1.1	S8 self-incompatibility ribonu	0	0	0	0	0	0	0	0	0	0	1	NA	0	0	0	0	0	0	0	0	1	NA	0	0	0	0	0	0	0	0	1	NA	0	0	0	0	0	0	0	1	NA
Solyc01g056200.1.1	Unknown Protein (AHRD V1)	0	1.7	0	0	0.42	2.13	0.75	0.88	0.69	1.11	2.62	1	1.78	0	3.23	1.67	0	1.49	0.75	0.75	0.45	1	0	0	2.85	0.95	0	0	2.09	0.7	0.73	1	1.36	2.08	0	1.15	0.88	0.84	0.86	0.75	1
Solyc01g056210.1.1	Unknown Protein (AHRD V1);	0	0	0	0	0	0	0	0	0	0	1	NA	0	0	0	0	0	0.07	0	0.02	2.33	1	0.22	0.11	0	0.11	0.09	0.21	0.2	0.17	1.52	1	0	0.2	0.21	0.14	0.25	0.12	0.18	1.35	1
Solyc01g056220.2.1	F-box family protein (AHRD V	0	0	0	0	0	0	0	0	0	0	1	NA	0	0	0	0	0	0	0	0	1	NA	0	0	0	0	0	0	0	0	1	NA	0	0	0	0	0	0	0	1	NA
Solyc01g056230.1.1	Unknown Protein (AHRD V1);	0	0	0	0	0	0	0	0	0	0	1	NA	0	0	0	0	0	0	0	0	1	NA	0	0	0	0	0	0	0	0	1	NA	0	0	0	0	0	0	0	1	NA
Solyc01g056240.1.1	S-locus F-box-like protein c (A	0	0	0	0	0	0	0	0	0	0	1	NA	0	0	0	0	0	0	0	0	1	NA	0	0	0	0	0	0	0	0	1	NA	0	0	0	0	0	0	0	1	NA
Solyc01g056250.2.1	S-locus F-box-like protein d (A	0	0	0	0	0	0	0	0	0	0	1	NA	0	0	0	0	0	0	0	0	1	NA	0	0	0	0	0	0	0	0	1	NA	0	0	0	0	0	0	0	1	NA
Solyc01g056260.1.1	Unknown Protein (AHRD V1)	0	1.65	1.1	0	0.69	0	0.73	0	0.67	0.35	0.51	1	0.86	0	0	0.29	0	0.48	0.73	0.4	1.41	1	0	0	0	0	0	0	0	0	1	NA	0	0	0	0	0	0	0	1	NA
Solyc01g056270.2.1	Unknown Protein (AHRD V1)	5.65	0.56	2.22	6.22	3.66	0.7	4.46	0	2.26	1.85	0.51	0.980282	0.58	2.73	2.65	1.99	3.05	2.6	2.47	2.71	1.36	1	0	0.51	0	0.17	0	0	0	0	0.06	1	0	0	0	0	0	0	0	1	NA
Solyc01g056280.2.1	F-box (AHRD V1 ***- Q84KQ9	0	0	0	0	0	0	0	0	0	0	1	NA	0	0	0	0	0	0	0	0	1	NA	0	0	0	0	0	0	0	0	1	NA	0	0	0	0	0	0	0	1	NA
Solyc01g056290.2.1	Unknown Protein (AHRD V1)	0	0	0	0	0	0	0	0	0	0	1	NA	0	0	0	0	0	0	0	0	1	NA	0	0	0	0	0	0	0	0	1	NA	0	0	0	0	0	0	0	1	NA
Solyc01g056300.1.1	Unknown Protein (AHRD V1)	0	0	0	0	0	0	0	0	0	0	1	NA	0	0	0	0	0	0	0	0	1	NA	0	0	0	0	0	0	0	0	1	NA	0	0	0	0	0	0	0	1	NA
Solyc01g056310.2.1	Laccase-2 (AHRD V1 **-- LAC	0	0	1.68	0	0.42	0	0	0	0	0	0.02	0.804781	0	0	0	0	0	0	0	0	1	NA	0	0	0	0	0	0	0	0	1	NA	0	0	0	0	0	0	0	1	NA
Solyc01g056320.1.1	Unknown Protein (AHRD V1)	4.63	1.03	1.37	0	1.76	0	1.83	0	3.32	1.29	0.73	1	2.15	6.04	2.93	3.71	3.21	4.19	3.64	3.68	0.99	1	1.87	2.83	2.3	2.33	3.8	0.88	0.84	1.84	0.79	1	1.65	1.68	3.56	2.3	3.18	3.04	3.11	1.35	1
Solyc01g056330.1.1	DNA-directed RNA polymeras	0	0	0	0	0	0	0	0	0.37	0.09	9.25	1	0.48	0	0	0.16	0.36	0	0	0.12	0.75	1	0.42	0	0	0.14	0	0	0	0	0.07	1	0.73	0.75	0.79	0.76	0	0	0	0.01	1
Solyc01g056340.2.1	DET1-like protein (AHRD V1 *	6.67	8.4	6.68	7.12	7.22	9.24	10.54	10.65	7.5	9.48	1.31	0.58866	10.07	8.54	8.51	9.04	10.74	8.41	10.39	9.85	1.09	1	10.78	12.82	14.62	12.74	9.68	10.11	8.53	9.44	0.74	1	15.22	9.88	12	12.37	10.03	13.14	11.59	0.94	1
Solyc01g056350.1.1	Unknown Protein (AHRD V1)	0	0	0	0	0	0	0	0	0	0	1	NA	0	0	1.01	0.34	0	0	0	0	0.03	1	0	0	0	0	0	0	0	0	1	NA	0	0	0	0	0	0	0	1	NA
Solyc01g056360.2.1	Unknown Protein (AHRD V1)	0	0	0	0	0	0	0	0	0	0	1	NA	0	0	0	0	0	0	0	0	1	NA	0	0	0	0	0	0	0	0	1	NA	0	0	0	0	0	0	0	1	NA
Solyc01g056370.2.1	Unknown Protein (AHRD V1)	0	0	1.28	0	0.32	1.2	0	0	0	0.3	0.94	1	0	0	0	0	0	0	0.85	0.28	28.33	1	0	0	0	0	0	0	0	0	1	NA	0	0	0	0	0	0	0	1	NA
Solyc01g056380.1.1	Nodulin / glutamate-ammoni	0	0	0	0	0	0	0	0	0	0	1	NA	0	0	0	0	0	0	0	0	1	NA	0	0	0	0	0	0	0	0	1	NA	0	0	0	0	0	0	0	1	NA
Solyc01g056390.1.1	Nodulin / glutamate-ammoni	0	0	0	0	0	0	0	0	0	0	1	NA	0	0	0	0	0	0	0	0	1	NA	0	0	0	0	0	0	0	0	1	NA	0	0	0	0	0	0	0	1	NA
Solyc01g056400.1.1	Developmental protein FluG (	0	0	0	0	0	0	0	0	0	0	1	NA	0	0	0	0	0	0	0	0	1	NA	0	0	0	0	0	0	0	0	1	NA	0	0	0	0	0	0	0	1	NA
Solyc01g056410.1.1	Unknown Protein (AHRD V1)	0	0	0	0	0	0	0	0	0	0	1	NA	0	0	0	0	0	0	0	0	1	NA	0	0	0	0	0	0	0	0	1	NA	0	0	0	0	0	0	0	1	NA
Solyc01g056420.1.1	Unknown Protein (AHRD V1);	0	0	0	0	0	0	0	0	0	0	1	NA	0	0	0	0	0	0	0	0	1	NA	0	0	0	0	0	0	0	0	1	NA	0	0	0	0	0	0	0	1	NA
Solyc01g056430.1.1	Amidohydrolase family (AHRD	0	0	0	0	0	0	0	0.18	0	0.04	4.5	1	0	0	0	0	0	0	0	0	1	NA	0	0	0	0	0	0	0	0	1	NA	0	0	0	0	0	0	0	1	NA
Solyc01g056440.1.1	Nodulin / glutamate-ammoni	0	0	0	0	0	0	0	0	0	0	1	NA	0	0	0	0	0	0	0	0	1	NA	0	0	0	0	0	0	0	0	1	NA	0	0	0	0	0	0	0	1	NA
Solyc01g056450.1.1	Glutamine synthetase I (AHRD	0	0	0	0	0	0	0	0	0	0	1	NA	0	0	0	0	0	0	0	0	1	NA	0	0	0	0	0	0	0	0	1	NA	0	0	0	0	0	0	0	1	NA
Solyc01g056460.1.1	Ycf2 (AHRD V1 *-*- D2KLP5_(	0	0	0	0	0	0	0	0	0	0	1	NA	0	0	0	0	0	0	0	0	1	NA	0	0	0	0	0	0	0	0	1	NA	0	0	0	0	0	0	0	1	NA
Solyc01g056470.1.1	Photosystem II CP43 chloroph	0	0	0	0	0	0	0	0	0	0	1	NA	0	0	0	0	0	0	0	0	1	NA	0	0	0	0	0	0	0	0	1	NA	0.51	0	0	0.17	0	0.31	0.15	0.91	1
Solyc01g056480.2.1	Unknown Protein (AHRD V1)	0.67	4.74	2.36	2.83	2.65	10.37	3.16	1.84	3.83	4.8	1.81	0.924375	2.48	4.63	6.19	4.43	4.16	3.11	3.14	3.47	0.78	1	6.46	8.15	2.65	5.75	3.06	4.04	4.37	3.82	0.66	1	3.79	4.84	5.12	4.58	3.66	4.09	3.88	0.85	1
Solyc01g056490.1.1	Ulp1 protease family C-termi	0	0	0.29	0.09	0.1	0.21	0.1	0.29	0.13	0.18	1.92	0.917319	0.12	0.27	0.31	0.23	0	0.03	0	0.01	0.04	0.201413	0	0.1	0.06	0.05	0.04	0	0	0.01	0.25	1	0.09	0.18	0.19	0.15	0	0.27	0.14	0.88	1
Solyc01g056510.1.1	HAT family dimerisation dom	0	0	0	0	0	0	0	0	0	0	1	NA	0	0	0	0	0	0	0	0	1	NA	0	0	0	0	0	0	0	0	1	NA	0	0	0	0	0	0	0	1	NA
Solyc01g056520.2.1	RAN binding protein 16-like (	8.27	10.04	11.6	8.51	9.6	7.94	10.19	10.2	8.95	9.32	0.97	1	9.62	10.35	12.86	10.94	9.04	9.28	9.95	9.42	0.86	1	10.93	12	11.63	11.52	8.89	10.16	12.65	10.57	0.92	1	25.39	20.34	26.49	24.07	19.96	21.15	20.55	0.85	1
Solyc01g056530.1.1	Oligopeptide transporter 9 (A	0	0	0	0	0	0	0	0.26	0	0.07	6.5	1	0.26	0.25	0.24	0.25	0	0	0.22	0.07	0.29	1	0	0	0	0	0	0	0	0	1	NA	0	0	0	0	0	0	0	1	NA
Solyc01g056550.1.1	Ulp1 protease family C-termi	0.26	0	0.31	0	0.14	0	0.42	0	0	0.1	0.74	1	0	0.23	0	0.08	0.18	0.27	0.21	0.22	2.87	1	0	0	0	0	0	0	0	0	1	1	0	0	0	0	0	0	0	1	NA
Solyc01g056560.1.1	Ulp1 protease family C-termi	0.17	0	0	0.25	0.11	0	0	0.16	0.25	0.1	0.98	1	0.16	0.15	0.15	0.15	0.12	0	0.14	0.09	0.57	1	0	0	0	0	0	0	0	0	1	1	0.25	0	0	0.08	0	0.15	0.07	0.9	1
Solyc01g056570.2.1	Regulator of chromosome co	10.14	10.34	19.08	15.39	13.74	9.98	10.14	9.5	9.62	9.81	0.71	0.639388	12.01	10.96	16.72	13.23	12.62	10.95	11.98	11.85	0.9	1	15.1	15.78	17.76	16.21	13.81	12.16	12.41	12.79	0.79	1	16.87	12.24	11.91	13.67	14.8	15.09	14.95	1.09	1

Solyc01g056580.2.1	L-aspartate oxidase (AHRD V1	4.26	2.12	7.64	4.58	4.65	2.27	2.42	2.66	2.33	2.42	0.52	0.488448	1.9	2.96	2.88	2.58	3.19	2.21	3.08	2.83	1.1	1	1.1	0.97	1.18	1.08	1.68	0.26	0.25	0.73	0.67	1	2.91	2.97	3.15	3.01	3.43	3.29	3.36	1.12	1
Solyc01g056590.1.1	Genomic DNA chromosome 5	1.42	0.42	1.12	2.01	1.24	0	1.5	1.31	2.04	1.21	0.98	1	0.44	0.41	3.21	1.35	0.33	1.72	1.86	1.3	0.96	1	2.3	1.55	0.47	1.44	0.94	0.36	0.35	0.55	0.38	1	3.37	1.03	0	1.47	1.3	0	0.65	0.44	1
Solyc01g056600.2.1	Ribonuclease 3 (AHRD V1 *-*	13.61	10.72	10.5	12.59	11.86	7.57	15.71	14.92	12.03	12.56	1.06	1	17.53	16.38	12.38	15.43	22.88	16.54	10.7	16.71	1.08	1	11.84	14.51	6.76	11.04	11.01	11.91	14.88	12.6	1.14	1	11.91	19.01	14.49	15.14	17.73	22.49	20.11	1.33	1
Solyc01g056610.1.1	Unknown Protein (AHRD V1)	8.68	3.86	4.1	14.73	7.84	5.79	2.06	6.38	11.84	6.52	0.83	1	4.04	5.28	6.6	5.31	7.83	8.54	6.82	7.73	1.46	0.956426	7.71	7.78	4.31	6.6	9.69	7.89	5.06	7.55	1.14	1	14.81	5.67	2.67	7.72	10.33	11.42	10.88	1.41	1
Solyc01g056620.2.1	DsRNA-binding protein (AHRD	18.26	20.72	19.66	18.73	19.34	20.3	25.23	23.72	20.16	22.35	1.16	0.850063	18.69	22.22	19.67	20.19	22.17	26.24	28.34	25.58	1.27	0.665212	17.52	17.3	16.99	17.27	17.85	15.23	17.8	16.96	0.98	1	27.59	20.39	24.92	24.3	18.6	25.82	22.21	0.91	1
Solyc01g056640.1.1	Unknown Protein (AHRD V1)	0	0	0	0	0	0	0	0	0	0	1	NA	0	0	0	0	0	0	0	0	1	NA	0	0	0	0	0	0	0	0	1	NA	0	0	0	0	0	0	0	1	NA
Solyc01g056650.1.1	Starch synthase (AHRD V1 **	0	0	0	0	0	0	0	0	0	0	1	NA	0	0	0	0	0	0	0	0	1	NA	0	0	0	0	0	0	0	0	1	NA	0	0	0	0	0	0	0	1	NA
Solyc01g056660.1.1	F-box family protein (AHRD V	0	0	0	0	0	0	0	0	0	0	1	NA	0	0	0	0	0	0	0	0	1	NA	0	0	0	0	0	0	0	0	1	NA	0	0	0	0	0	0	0	1	NA
Solyc01g056670.1.1	NADH-quinone oxidoreductas	0	0	0	0	0	0	0	0	0	0	1	NA	0	0	0	0	0	0	0	0	1	NA	0	0	0	0	0	0	0	0	1	NA	0	0.84	0	0.28	0	0	0	0.04	1
Solyc01g056680.1.1	Unknown Protein (AHRD V1)	0	0	0	0	0	0	0	0	0	0	1	NA	0	0	0	0	0	0	0	0	1	NA	0	0	0	0	0	0	0.52	0.17	17.33	1	0	0	0	0	0	0	0	1	NA
Solyc01g056690.2.1	Kinase family protein (AHRD V	13.21	14.36	16.66	14.98	14.8	14.13	14.26	17.25	15.57	15.3	1.03	1	14.73	15.52	16.1	15.45	15.74	13.32	12.53	13.86	0.9	1	9.78	9.37	8.51	9.22	10.87	9.71	8.97	9.85	1.07	1	18.21	12.45	11.79	14.15	13.57	13.27	13.42	0.95	1
Solyc01g056700.1.1	Unknown Protein (AHRD V1)	0	0	0	0	0	0	0	0	0	0	1	NA	0	0	0	0	0	0	0	0	1	NA	0	0	0	0	0	0	0	0	1	NA	0	0	0	0	0	0	0	1	NA
Solyc01g056710.1.1	Unknown Protein (AHRD V1)	0	0	0	0	0	0	0	0	0	0	1	NA	0	0	0	0	0	0	0	0	1	NA	0	0	0	0	0	0	0	0	1	NA	0	0	0	0	0	0	0	1	NA
Solyc01g056720.2.1	Aquaporin SIP12 (AHRD V1 **	32.23	40.04	35.87	37.41	36.39	43.26	34.56	65.97	29.03	43.2	1.19	0.90221	29.69	26.43	21.06	25.73	18.34	22.35	27.48	22.72	0.88	1	23.82	24.53	26.55	24.97	18.17	16.13	18.83	17.71	0.71	1	3.89	7.73	6.54	6.05	6.12	6.93	6.53	1.08	1
Solyc01g056730.1.1	Unknown Protein (AHRD V1)	8.33	7.42	17.2	6.88	9.96	6.95	8.77	16.6	12.96	11.32	1.14	1	9.69	10.26	9.97	9.97	11.56	7.19	9.82	9.52	0.95	1	2.81	4.53	4.14	3.83	10.49	3.16	4.05	5.9	1.54	1	4.94	6.56	7.48	6.33	2.54	4.87	3.71	0.59	1
Solyc01g056740.1.1	HAT dimerisation domain-con	6.1	8.15	6.92	6.31	6.87	4.18	5.38	10.79	6.07	6.6	0.96	1	6.33	11.84	13.29	10.49	7.98	7.17	12.73	9.29	0.89	1	2.28	3.06	3.73	3.02	5.24	3.38	3.93	4.18	1.38	1	4.67	4.77	3.97	4.47	3.01	3.91	3.46	0.77	1
Solyc01g056750.1.1	Os11g0573200 protein (Fragr	4.38	7.79	4.06	3.54	4.94	4.52	5.43	6.9	8.98	6.46	1.31	0.862399	7.56	6.25	7.39	7.07	5.2	6.31	5.65	5.72	0.81	1	2.53	2.55	1.55	2.21	2.05	1.42	2.28	1.92	0.87	1	2.67	7.27	3.37	4.44	4.29	4.39	4.34	0.98	1
Solyc01g056760.2.1	DNA-binding protein HEXBP (	4.6	9.06	6.58	4.64	6.22	6.57	4.66	10.86	5.66	6.94	1.12	1	7.33	6.28	5.82	6.48	2.96	3.06	4.9	3.64	0.56	0.543578	2.65	2.14	1.95	2.25	1.94	1.99	2.87	2.27	1.01	1	5.13	3.34	3.53	4	1.8	4.32	3.06	0.77	1
Solyc01g056770.1.1	Unknown Protein (AHRD V1)	4.78	6.81	7.9	2.7	5.55	4.25	1.51	7.03	4.12	4.23	0.76	1	5.34	7.48	9.69	7.5	4.64	4.46	6.01	5.04	0.67	0.994791	4.63	4.67	1.9	3.73	1.88	2.17	1.39	1.81	0.49	1	1.36	3.47	7.35	4.06	1.75	6.71	4.23	1.04	1
Solyc01g056780.2.1	50S ribosomal protein L34 (A	62.19	50.49	30.74	38.63	45.51	113.97	98.19	70.31	106.19	97.17	2.13	0.001594	34.79	41.28	32.65	36.24	85.68	63.31	68.03	72.34	2	0.002974	30.55	30.82	25.44	28.94	30.92	39.81	34.91	35.21	1.22	1	4.18	9.77	6.46	6.8	18.08	12.53	15.3	2.25	0.741711
Solyc01g056790.2.1	Exostosin-like (AHRD V1 ***-	4.3	3.83	4.23	1.52	3.47	5.58	4.25	4.29	3.6	4.43	1.28	1	2.67	3.74	1.21	2.54	2.24	1.86	2.25	2.12	0.83	1	0.87	1.46	0	0.78	0.47	0.54	0.78	0.6	0.77	1	0	0	0.55	0.18	0	0	0	0.05	1
Solyc01g056800.1.1	Exostosin-like (AHRD V1 ***-	0.8	2.51	2.37	0.57	1.56	2.24	1.91	1.85	3.47	2.37	1.52	0.949909	1.87	0.35	2.38	1.53	0.56	0.62	2.53	1.24	0.81	1	0.65	0	1.2	0.62	0	0.3	0.59	0.3	0.48	1	0	0	0	0	0	0.35	0.17	17.5	1
Solyc01g056810.2.1	Syntaxin (AHRD V1 ***- Q6X9	17.4	15.72	16.24	15.8	16.29	15.3	15.38	21.49	17.9	17.52	1.08	1	12.08	17.38	14.7	14.72	14.77	12.91	18.57	15.42	1.05	1	6.92	5.71	3.61	5.41	9.38	5.71	10.79	8.63	1.59	1	2.95	2.45	2.4	2.6	1.9	3.87	2.88	1.11	1
Solyc01g056820.1.1	Methionine aminopeptidase (	0	0	0	0	0	0	0	0	0	0	1	NA	0.31	0	0.29	0.2	0	0	0	0	0.05	1	0	0.28	0	0.09	0	0	0	0	0.11	1	0	0.25	0	0.08	0	0	0	0.12	1
Solyc01g056830.2.1	Syntaxin (Fragment) (AHRD V	14.34	9.57	13.54	2.03	9.87	8.77	6.79	10.55	9.27	8.84	0.9	1	10.01	11.22	9.69	10.31	7.96	10.02	15.78	11.25	1.09	1	2.32	2.92	1.42	2.22	4.24	3.8	4.18	4.07	1.83	1	0	2.6	1.1	1.23	2.63	2.51	2.57	2.08	1
Solyc01g056840.1.1	DNA-directed RNA polymeras	0	0	0	0	0	0	0	0.66	0	0.17	16.5	1	0	0	0	0	0	0	0	0	1	NA	0	0.58	0	0.19	0	0	0	0	0.05	1	1.02	0	0	0.34	0	1.26	0.63	1.85	1
Solyc01g056850.2.1	CTP synthase (AHRD V1 ***-	23.59	44.97	44.35	19.98	33.22	31.54	18.9	22.13	23.55	24.03	0.72	0.650703	19.04	11.03	14.06	14.71	4.48	6.7	7.53	6.24	0.42	0.03792	2.81	2.93	2.73	2.82	1.73	1.27	0.61	1.2	0.43	1	0.34	0.96	0.55	0.62	0.55	1.57	1.06	1.72	1
Solyc01g056860.1.1	Pol polyprotein (AHRD V1 ***	0	0	0	0	0	0	0	0	0	0	1	NA	0	0	0	0	0	0	0	0	1	NA	0	0	0	0	0	0	0	0	1	NA	0	0.26	0	0.09	0	0	0	0.12	1
Solyc01g056870.1.1	Ycf2 (AHRD V1 ***- D2KLP5_(	0	0	0	0	0	0	0	0	0	0	1	NA	0	0	0.57	0.19	0.47	0	0.53	0.33	1.75	1	0	0	0	0	0	0	0	0	1	NA	2.87	0.49	1.04	1.47	0	0	0	0.01	1
Solyc01g056880.1.1	Soul heme-binding family pro	1.86	5.79	4.38	0	3.01	1.03	5.87	9.4	6.67	5.74	1.91	0.776784	3.46	3.23	5.49	4.06	10.31	5.77	3.65	6.58	1.62	0.926621	6.01	7.57	4.61	6.06	4.27	7.75	8.13	6.72	1.11	1	13.21	9.45	2.86	8.51	7.66	8.15	7.91	0.93	1
Solyc01g056890.1.1	Unknown Protein (AHRD V1)	0.49	3.04	0	0	0.88	1.09	1.54	0.9	0.7	1.06	1.2	1	0.45	1.27	0	0.57	0.68	1.01	1.92	1.2	2.1	1	1.18	1.99	0	1.06	0.96	0.37	1.78	1.04	0.98	1	1.39	4.97	7.51	4.62	0.89	0.86	0.88	0.19	0.692109
Solyc01g056900.1.1	Unknown Protein (AHRD V1)	0	0	0	0	0	0	0	0	0	0	1	NA	0	0	0	0	0.67	0	0	0.22	22.33	1	0	0	0	0	0	0.74	0	0.25	24.67	NA	0	0	0	0	0	0	0	1	NA
Solyc01g056910.1.1	Protease Do-like 7 (AHRD V1	0	0	0	0	0	0	0	0	0	0	1	NA	0	0	0	0	0	0	0	0	1	NA	0	0	0	0	0	0	0	0	1	NA	0	0	0	0	0	0	0	1	NA
Solyc01g056930.1.1	Hypothetical chloroplast RF1	0	0	0	0	0	0	0	0	0	0	1	NA	0	0	0	0	0	0	0	0	1	NA	0	0	0	0	0	0	0	0	1	NA	0	0	0	0	0	0	0	1	NA
Solyc01g056940.2.1	Ubiquitin (AHRD V1 ***- D3P	892.92	656.32	971.16	993.78	878.55	496.98	685.04	509.59	695.05	596.66	0.68	0.313823	759.44	684.88	701.3	715.21	571.22	502.36	417.07	496.88	0.69	0.298879	421.97	473.89	420.84	438.9	408.27	401.92	400.82	403.67	0.92	1	157.72	253.61	261.05	224.13	354.27	426.27	390.27	1.74	0.522005
Solyc01g056950.1.1	Orf141 protein (AHRD V1 *-*	0	0	0	0	0	0	0	0	0	0	1	NA	0	0	0	0	0	0	0	0	1	NA	0	0	0	0	0	0	0	0	1	NA	0	0	0	0	0	0	0	1	NA
Solyc01g056960.1.1	Unknown Protein (AHRD V1)	0	0	0	0	0	0	0	0	0	0	1	NA	0	0	0	0	0	0	0	0	1	NA	0	0	0	0	0	0	0	0	1	NA	0	0	0	0	0	0	0	1	NA
Solyc01g056970.1.1	Unknown Protein (AHRD V1)	0	0	0	0	0	0	0	0	0	0	1	NA	0	0	0	0	0	0	0	0	1	NA	0	0	0	0	0	0	0	0	1	NA	0	0	0	0	0	0	0	1	NA
Solyc01g056980.2.1	Unknown Protein (AHRD V1)	245.89	290.9	318.4	407.2	315.6	359.99	378.78	529.36	305.7	393.46	1.25	0.547209	382.14	508.31	392.58	427.68	307.81	371.08	374.7	351.2	0.82	0.950993	356.13	339.65	276.81	324.2	376.26	263.64	290.63	310.18	0.96	1	865.5	579.34	627.13	690.66	378.81	433.82	406.31	0.59	0.742285
Solyc01g056990.2.1	Genomic DNA chromosome 5	4.71	3.7	3.92	3.92	4.06	3.08	2.41	6.63	3.78	3.98	0.98	1	2.58	3.37	2.81	2.92	1.15	2.01	2.18	1.78	0.61	0.894674	0	0.23	0.28	0.17	0.36	0.42	1.21	0.66	3.9	1	3.15	0.6	2.13	1.96	1.01	1.22	1.11	0.57	1
Solyc01g057000.2.1	Universal stress protein famil	471.33	518.58	475.09	464.42	482.36	406.51	384.45	497.52	386.56	418.76	0.87	0.930102	657.28	930.48	760.44	782.73	777.33	680.19	645.21	700.91	0.9	1	2963.15	3084.38	2563.65	2870.39	1981.55	2283.31	3212.34	2492.4	0.87	1	146.93	341.22	245.7	244.62	305.09	177.21	241.15	0.99	1
Solyc01g057010.1.1	F-box family protein (AHRD V	0	0	0	0	0	0	0	0	0	0	1	NA	0	0	0	0	0	0	0	0	1	NA	0	0	0	0	0	0	0	0	1	NA	0	0	0	0	0	0	0	1	NA
Solyc01g057020.2.1	PDE320 (AHRD V1 ***- D7L6	10.12	8.01	14.59	10.6	10.83	12.5	13.02	13.79	11.84	12.79	1.18	0.884698	13.25	11.73	19	14.66	12.48	13.2	11.49	12.39	0.85	1	6.06	12.22	13.02	10.43	6.89	8.81	8.19	7.96	0.76	1	37.31	15.52	23.06	25.3	14.41	16.43	15.42	0.61	1
Solyc01g057030.1.1	Unknown Protein (AHRD V1)	0	0	0	0	0	0	0	0	0	0	1	NA	0	0	0	0	0	0	0	0	1	NA	0	0	0	0	0	0	0	0	1	NA	0	0	0	0	0	0	0	1	NA
Solyc01g057040.2.1	PDE320 (AHRD V1 ***- D7L6	5.71	8.66	9.73	3.89	7	12.69	10.18	11.08	7.59	10.38	1.48	0.482633	7.08	10.48	6.97	8.18	9.39	8.65	10.14	9.39	1.15	1	6.49	6.72	10.28	7.83	3.47	7.05	5.08	5.2	0.66	1	14.43	11.21	11.06	12.23	8.9	9.08	8.99	0.73	1
Solyc01g057060.1.1	Arabidopsis protein targeted	0	0	0	0	0	0	0	0	0	0	1	NA	0	0	0	0	0	0	0	0	1	NA	0	0	0	0	0	0	0	0	1	NA	0	0	0	0	0	0	0	1	NA
Solyc01g057070.1.1	Arabidopsis protein targeted	0	0.67	0.88	1.06	0.65	0.83	0.59	1.38	0.54	0.83	1.28	1	0	0.65	1.27	0.64	0	0.78	0	0.26	0.41	1	0.61	1.22	0.74	0.86	0	2.27	0.55	0.94	1.1	1	1.07	0	0	0.36	0	1.31	0.66	1.84	1
Solyc01g057080.1.1	Ethylene-responsive transcrip	0	0	0	0	0	0	0	0	0	0	1	NA	0	0	0	0	0	0	0	0	1	NA	0	0	0	0	0	0	0	0	1	NA	0	0	0	0	0	0	0	1	NA
Solyc01g057090.2.1	Rhodopsin-like receptor (AHR	10.82	14.91	16.34	10.82	13.22	14.96	9.37	12.98	9.09	11.6	0.88	0.956676	11.29	14.22	11.48	12.33	13.64	15.92	16.69	15.42	1.25	0.694315	13.11	9.57	10.08	10.92	10.66	10.1	12.11	10.96	1	1	25.33	15.33	16.23	18.96	17.03	18.74	17.88	0.94	1
Solyc01g057100.1.1	DNA-directed RNA polymeras	0	0	0	0	0	0	0	0	0	0	1	NA	0	0	0	0	0	0	0	0	1	NA	0	0	0	0	0	0	0	0	1	NA	1.03	0	0	0.34	0.66	0	0.33	0.96	1
Solyc01g057110.1.1	Unknown Protein (AHRD V1)	0	0	0	0	0	0	0	0	0	0	1	NA	0	0	0	0	0	0	0	0	1	NA	0	0	0	0	0	0	0	0	1	NA	0	0	0	0	0	0	0	1	NA
Solyc01g057120.1.1	Unknown Protein (AHRD V1)	0	0	0	0	0	0	0	0	0	0	1	NA	0	0	0	0	0	0	0	0	1	NA	0	0	0	0	0	0	0	0	1	NA	0	0	0	0	0	0	0	1	NA
Solyc01g057130.1.1	Unknown Protein (AHRD V1)	0	0	0	0	0	0	0	0	0	0	1	NA	0	0	0	0	0	0	0	0	1	NA	0	0	0	0	0	0	0	0	1	NA	3.54	0	0	1.18	0	0	0	0.01	1
Solyc01g057140.1.1	Unknown Protein (AHRD V1)	0	0	0	0	0	0	0	0	0	0	1	NA	0	0	0	0	0	0	0	0	1	NA	0	0	0	0	0	0	0	0	1	NA	0	0	0	0	0	0	0	1	NA
Solyc01g057150.1.1	Unknown Protein (AHRD V1)	0	0	0	0	0	1.58	0	0	0	0.4	39.5	1	0	0	0	0	0	0	0	0	1	NA	0	0	0	0	0	0	0	0	1	NA	0	0	0	0	0.65	0	0.33	32.5	1
Solyc01g057160.1.1	Unknown Protein (AHRD V1)	0	0	0	0	0	0	0	0	0	0	1	NA	0	0	0	0	0	0	0	0	1	NA	0	0	0	0	0	0	0	0	1	NA	0	0	0	0	0	0	0	1	NA
Solyc01g057170.2.1	Unknown Protein (AHRD V1)	26.99	40.03	10.61	0	19.41	66.67	30.78	93.74	36.6	56.95	2.93	0.341929	44.63	20.85	5.07	23.52	20.8	23.29	47.13	30.41	1.29	1	2.42	7.33	0	3.25	5.91	4.55	17.48	9.31	2.87	1	0	8.71	4.61	4.44	2.75	5.26	4	0.9	1
Solyc01g057180.1.1	Unknown Protein (AHRD V1)	0	0	0	0	0	0	0	0	0	0	1	NA	0	0	0	0	0	0	0	0	1	NA	0	0	0	0	0	0	0	0	1	NA	0	0	0	0	0	0	0	1	NA
Solyc01g057190.1.1	F-box (AHRD V1 ***- Q84KQ9	0	0	0	0	0	0	0	0	0	0	1	NA	0	0	0	0	0	0	0	0	1	NA	0	0	0	0	0	0	0	0	1	NA	0	0	0	0	0	0	0	1	NA
Solyc01g057200.2.1	Pentatricopeptide repeat-con	7.84	7.11	8.76	7.33	7.76	16.35	9.51	9.4	10.06	11.33	1.46	0.364844	5.6	6.45	7.57	6.54	15.35	13.56	9.91	12.94	1.98	0.011749	2.72	6.39	4.45	4.52	5.61	4.99	6.02	5.54	1.23	1	5.18	6.51	5.6	5.76	7.31	6.26	6.79	1.18	1
Solyc01g057210.2.1	Pyruvate dehydrogenase kina	53.94	68.47	43.61	35.27	50.32	78.64	62.69	86.51	62.22	72.51	1.44	0.256806	64.16	45.97	36.75	48.96	35.57	46.79	52.96	45.11	0.92	1	35.29	27.24	19.14	27.22	26.96	23.19	31.71	27.29	1	1	9.81	18.11	15.77	14.56	20.72	21.25	20.98	1.44	1
Solyc01g057220.2.1	Pectinesterase (AHRD V1 ***	0	0	0	0	0	0	0	0	0	0	1	NA	0	0	0	0	0	0	0	0	1	NA	0	0	0	0	0	0	0	0	1	NA	0	0	0	0	0	0	0	1	NA
Solyc01g057240.1.1	50S ribosomal protein L3-2 (A	4.85	0.86	2.29	0	2	1.08	2.3	1.78	2.79	1.99	0.99	1	1.8	2.53	4.92	3.08	2.69	2.01	1.52	2.07	0.67	1	1.57	0	1.93	1.17	4.46	5.88	4.24	4.86	4.17	1	4.14	0.7	4.48	3.11	2.66	1.7	2.18	0.7	1
Solyc01g057250.1.1	50S ribosomal protein L3-2 (A	0	0	0	0	0	0	0	0	0	0	1	NA	0	0	0	0	0	0	0	0	1	NA	0	0	0	0	0	0	0	0	1	NA	0	0	0	0	0	0	0	1	NA
Solyc01g057260.2.1	Os06g0524700 protein (Fragr	0.75	1.33	0.35	0	0.61	0.67	0.95	0.69	0.54	0.71	1.17	1	0.28	1.04	0.51	0.61	0.42	0.47	1.3	0.73	1.2	1	0.73	0	0	0.24	0	0	0	0	0.04	1	0.21	0.22	1.15	0.53	0.14	0	0.07	0.13	1
Solyc01g057270.2.1	Calmodulin-binding transcript	0.14	0.25	0.08	0	0.12	0	0	0.13	0	0.03	0.28	0.811762	0.06	0	0.06	0.04	0.1	0.04	0.05	0.06	1.58	1	0.17	0.11	0.14	0.14	0	0	0.15	0.05	0.36	1	0	0.05	0.11	0.05	0	0.12	0.06	1.12	1
Solyc01g057280.1.1	26S proteasome non-ATPase	0.94	2.23	0.74	0.88	1.2	2.09	1.48	1.72	0.45	1.44	1.2	1	0.87	0.82	0.79	0.83	0.22	0.81	1.72	0.92	1.11	1	1.01	1.27	0.93	1.07	0.82	0.47	1.14	0.81	0.76	1	1.33	1.36	1.44	1.38	0	1.1	0.55	0.4	1
Solyc01g057300.1.1	Transcription factor bHLH95 (	0	0.19	0	0	0.05	0	0.17	0	0	0.04	0.89	1	0	0	0	0	0	0	0	0	1	NA	0	0	0	0	0.14	0	0	0.05	4.67	1	0	0	0	0	0	0	0	1	NA
Solyc01g057310.2.1	Kinesin (Centromeric protein)	1.23	1.22	1.92	1.68	1.51	0.69	0.9	1.09	1.77	1.11	0.74	0.931534	0.82	0.77	1.19	0.93	0.46	0.71	0.55	0.57	0.62	0.743246	0.11	0.25	0.04	0.13	0.12	0	0.22	0.11	0.85	1	0	0.1	0.07	0.06	0	0.04	0.02	0.35	1
Solyc01g057320.1.1	Kinesin-like protein (AHRD V1	0.45	0.8	0.53	1.02	0.7	0.6	0.28	0.83	0.65	0.59	0.84	1	0.42	0.39	0.61	0.47	0.56	0.56	0.35	0.49	1.04	1	0	0.15	0.09	0.08	0	0.07	0.07	0.05	0.58	1	0.13	0.07	0	0.07	0.16	0.16	0.16	2.4	1
Solyc01g057330.1.1	Yellow stripe-like protein 1.3	2.05	1.37	2.41	0.72	1.64	1.71	1.21	0.94	1.47	1.33	0.81	1	2.85	2.67	3.46	2.99	0.71	1.06	0.4	0.72	0.24	0.41109	0	0.42	0	0.14	0	0	0	0	0.07	1	0	0	0	0	0	0	0	1	NA
Solyc01g057340.2.1	Unknown Protein (AHRD V1)	18.52	25.75	5.46	9.82	14.89	64.33	25.58	58.53	14.13	40.64	2.73	0.456992	26.91	20.12	16.62	21.22	8.83	26.97	35.47	23.76	1.12	1	1.87	3.77	10.34	5.33	6.84	12.28	10.12	9.75	1.83	1	3.29	12.61	21.36	12.42	5.3	4.06	4.68	0.38	1
Solyc01g057350.1.1	Unknown Protein (AHRD V1)	0	0	0	0	0	0	0	0	0	0	1	NA	0	0	0	0	0	0	0	0	1	NA	0	0	0	0	0	0	0	0	1	NA	0	0	0	0	0	0	0	1	NA
Solyc01g057360.1.1	Poly polymerase catalytic dor	0	0	0	0	0	0	0	0	0	0	1	NA	0	0	0	0	0	0	0	0	1	NA	0	0	0	0	0	0	0	0	1	NA	0	0	0	0	0	0	0	1	NA
Solyc01g057370.1.1	Poly polymerase catalytic dor	0	0	0	0	0	0	0	0	0	0	1	NA	0	0	0	0	0	0	0	0	1	NA	0	0	0	0	0	0	0	0	1	NA	0	0	0	0	0	0	0	1	NA
Solyc01g057380.1.1	Unknown Protein (AHRD V1)	0	0	0	0	0	0	0	0	0	0	1	NA	0	0	0	0	0	0	0	0	1	NA	0	0	0	0	0	0	0	0	1	NA	0	0	4.23	1.41	0	0	0	0.01	1
Solyc01g057390.2.1	Unknown Protein (AHRD V1)	0	0	0	0	0	0	0	0	0	0	1	NA	0	0	0	0	0	0	0	0	1	NA	0	0	0	0	0	0	0	0	1	1	0	0.32	0	0.11	0	0	0	0.09	1
Solyc01g057400.1.1	Helicase-like protein (AHRD V	0	0	0	0	0	0	0	0	0	0	1	NA	0	0	0	0	0	0	0	0	1	NA	0	0	0	0	0	0	0	0	1	NA	0	0	0	0	0	0	0	1	NA
Solyc01g057410.1.1	Unknown Protein (AHRD V1)	0	0	0	0	0	0	0	0	0	0	1	NA	0	0	0	0	0	0	0	0	1	NA	0	0	0	0	0	0	0	0	1	NA	0	0	0	0	0	0	0	1	NA
Solyc01g057420.1.1	Unknown Protein (AHRD V1)	0	0	0	0	0	0	0	0	0	0	1	NA	0	0	0	0	0	0	0	0	1	NA	0	0	0	0	0	0	0	0	1	NA	0	0	0	0	0	0	0	1	NA
Solyc01g057430.2.1	Chloroplast carboxyltransfera	3.96	3.52	0	0	1.87	5.87	4.17	2.43	0	3.12	1.67	1	1.23	4.59	0	1.94	0.92	0.68	2.07	1.22	0.63	1	2.13	2.15	0	1.43	1.73	0	1.92	1.22	0.85	1	1.88	5.75	0	2.54	0	1.16	0.58	0.23	1
Solyc01g057440.2.1	Unknown Protein (AHRD V1)	2.31	1.03	1.37	0	1.18	1.29	1.83	1.06	0.83	1.25	1.06	1	0	1.01	0	0.34	0	1.8	0	0.6	1.78	1	0	0	1.15	0.38	0	2.63	0	0.88	2.29	1	0	0	0	0	0	0	0	1	NA
Solyc01g057450.1.1	Unknown Protein (AHRD V1)	0	0	0	0	0	0	0	0	0	0	1	NA	0	0	0	0	0	0	0	0	1	NA	0	0	0	0	0	0	0	0	1	NA	0	0	0	0	0	0	0	1	NA
Solyc01g057460.2.1	CTV.22 (AHRD V1 ***- Q8H6(	77.9	91.82	57.96	67.51	73.8	74.89	64.83	121.96	61.47	80.79	1.09	1	71.81	66.5	37.34	58.55	42.84	50.15	66.18	53.06	0.91	1	58.4	48.04	32.04	46.16	28.58	23.94	41.43	31.32	0.68	1	16.96	86.65	67.99	57.2	23.13	31.38	27.25	0.48	0.938951
Solyc01g057470.1.1	Unknown Protein (AHRD V1)	0	0	0	0	0	0	0	0	0	0	1	NA	0	0	0	0	0	0	0	0	1	NA	0	0	0	0	0	0	0	0	1	NA	1.25	0	0	0.42	0	0	0	0.02	1
Solyc01g057480.1.1	Helicase-like protein (AHRD V	0	0.14	0	0.22	0.09	0	0.24	0.14	0	0.1	1.06	1	0	0	0	0	0	0	0	0	1	NA	0.13	0	0.15	0.09	0	0.12	0	0.04	0.43	1	0.22	0	0	0.07	0	0	0	0.14	1
Solyc01g057490.2.1	Unknown Protein (AHRD V1)	3.88	9.5	10.3	4.12	6.95	7.55	6.89	4.46	8.36	6.81	0.98	1	5.41	9.28	7.37	7.35	4.04	4.02	5.34	4.47	0.61	0.941359	10.19	4.74	5.78	6.9	4.46	5.88	4.24	4.86	0.7	1	5.52	4.93	8.95	6.47	6.22	2.55	4.38	0.68	1
Solyc01g057500.1.1	Unknown Protein (AHRD V1)	0	0	0	0	0	0	0	0	0	0	1	NA	0	0	0	0	0	0	0	0	1	NA	0	0	0	0	0	0	0	0	1	NA	0	0	0	0	0	0	0	1	NA
Solyc01g057510.1.1	Bax inhibitor 1 (AHRD V1 ***	0	0	0	0	0	0	0	0	0	0	1	NA	0	0	0	0	0	0	0	0	1	NA	0	0	0	0	0	0	0	0	1	NA	0	0	0	0	0	0	0	1	NA
Solyc01g057520.2.1	Genomic DNA chromosome 3	10.58	10.34	12.63	12.9	11.61	9.53	8.88	10.23	9.52	9.54	0.82	0.907712	11.48	11.63	14.02	12.38	8.52	6.52	7.31	7.45	0.6	0.082965	4.32	8.32	6.16	6.27	2.48	3.69	3.63	3.27	0.52	0.676183	5.27	5.43	5.61	5.44	13.91	13.33	13.62	2.51	0.065025
Solyc01g057530.1.1	Unknown Protein (AHRD V1)	0	0	0	0	0	0	0	0	0	0	1	NA	0	0	0	0	0	0	0	0	1	NA	0	0	0	0	0	0	0	0	1	NA	0	0	0	0	0	0	0	1	NA
Solyc01g057540.1.1	Unknown Protein (AHRD V1)	0	0	0	0	0	0	0	0	0	0	1	NA	0	0	0	0	0	0	0	0	1	NA	0	0	0	0	0	0	0	0	1	NA	0	0	0	0	0	0	0	1	NA
Solyc01g057550.1.1	Retrovirus-related Pol polypr	0	0	0	0	0	0	0	0	0	0	1	NA	0	0	0	0	0	0	0	0	1	NA	0	0	0	0	0	0	0	0	1	NA	0	0	0	0	0	0	0	1	NA
Solyc01g057560.1.1	LRR receptor-like serine/thre	0	0	0	0	0	0	0	0	0	0	1	NA	0	0	0	0	0	0	0	0	1	NA	0	0	0	0	0	0	0	0	1	NA	0	0	0	0	0	0	0	1	NA
Solyc01g057570.2.1	Alkylated DNA repair protein	18.88	22.19	22.58	17.45	20.27	24.35	23.26	27.32	23.14	24.52	1.21	0.766383	17.8	21.01	24.88	21.23	24.28	25.42	30.67	26.79	1.26	0.724034	13.02	14.36	9	12.13	12.9	12.79	16.15	13.95	1.15	1	13.96	11.7	6.97	10.88	13.6	13.25	13.43	1.23	1
Solyc01g057580.1.1	Kinase family protein (AHRD V	1.32	1.84	1.33	1.86	1.59	1.25	3.26	1.04	1.08	1.66	1.04	1	1.4	1.96	0.48	1.28	1.3	1.46	2.22	1.66	1.3	1	1.06	1.23	0.56	0.95	1.11	1.42	1.1	1.21	1.27	1	2.14	0.82	0.58	1.18	1.03	2.14	1.58	1.34	1
Solyc01g057590.1.1	Unknown Protein (AHRD V1)	0	0	0	0	0	0	0	0	0	0	1	NA	0	0	0	0	0	0	0	0	1	NA	0	0	0	0	0	0	0	0	1	NA	0	0	0	0	0	0	0	1	NA
Solyc01g057600.2.1	Unknown Protein (AHRD V1)	4.28	4.58	6.06	3.64	4.64	7.62	4.06	6.3	4.31	5.57	1.2	1	0.8	4.47	5.07	3.45	2.97	2.22	0.67	1.95	0.57	1	0	0.7	0.85	0.52	0.56	0.65	0	0.4	0.78	1	0	0	0	0	0	0	0	1	NA
Solyc01g057610.1.1	Unknown Protein (AHRD V1)	0	0	0	0	0	0	0	0	0	0	1	NA	0	0	0	0	0	0	0	0	1	NA	0	0	0	0	0	0	0	0	1	NA	0	0	0	0	0	0	0	1	NA
Solyc01g057620.1.1	Unknown Protein (AHRD V1)	0	0	0	0	0	0	0	0	0	0	1	NA	0	0	0	0	0	0	0	0	1	NA	0	0	0	0	0	0	0	0	1	NA	0	0	0	0	0	0	0	1	NA
Solyc01g057630.1.1	Unknown Protein (AHRD V1)	0	0	0	0	0	0	0	0	0	0	1	NA	0	0	0	0	0	0	0	0	1	NA	0	0	0	0	0	0	0	0	1	NA	0	0	0	0	0	0	0	1	NA
Solyc01g057640.1.1	Unknown Protein (AHRD V1)	0	0	0	0	0	0	0	0	0	0	1	NA	0	0	0	0	0	0	0	0	1	NA	0	0	0	0	0	0	0	0	1	1	0	0	0	0	0	0	0	1	NA
Solyc01g057650.1.1	Heat shock protein DnaJ N-te	0	0	0	0	0	0	0	0	0	0	1	NA	0	0	0	0	0	0	0	0	1	NA	0	0	0	0	0	0	0	0	1	NA	0	0	0	0	0	0	0	1	NA
Solyc01g057660.1.1	Unknown Protein (AHRD V1)	0	0	0	0	0	0	0	0	0	0	1	NA	0	0	0	0	0	0	0	0	1	NA	0	0	0	0	0	0	0	0	1	NA	0	0	0	0	0	0	0	1	NA
Solyc01g057670.1.1	DNAJ heat shock N-terminal d	0	0	0	0	0	0	0	0	0	0	1	NA	0	0	0	0	0	0	0	0	1	NA	0	0	0	0	0	0	0	0	1	NA	0	0	0	0	0	0	0	1	NA
Solyc01g057680.2.1	Hcr2-p4.1 (AHRD V1 ***- Q40	1.05	0	0	1.48	0.63	0	0	0	0.75	0.19	0.3	1	0.97	0	0.88	0.62	0	0	0	0	0.02	1	13.54	12.8	10.39	12.24	6.19	5.56	11.45	7.73	0.63	1	1.49	13.69	4.83	6.67	0.96	0	0.48	0.07	0.754855

Solyc01g057690.1.1	Unknown Protein (AHRD V1)	0	0	0	0	0	0	0	0	0	0	1	NA	0	0	0	0	0	0	0	0	1	NA	0	0	0	0	0	0	0	0	1	NA	0	0	0	0	0	0	0	1	NA
Solyc01g057700.1.1	Calcium-dependent protein ki	0	0	0	0	0	0	0	0	0	0	1	NA	0	0	0	0	0	0	0	0	1	NA	0	0	0	0	0	0	0	0	1	NA	0	0	0	0	0	0	0	1	NA
Solyc01g057710.1.1	Helitron helicase-like protein	0	0	0	0	0	0	0	0	0	0	1	NA	0	0	0	0	0	0	0	0	1	NA	0	0	0	0	0	0	0	0	1	NA	0	0	0	0	0	0	0	1	NA
Solyc01g057720.1.1	Helitron helicase-like protein	0	0	0	0	0	0	0	0	0	0	1	NA	0	0	0	0	0	0	0	0	1	NA	0	0	0	0	0	0	0	0	1	NA	0	0	0	0	0	0	0	1	NA
Solyc01g057730.1.1	Unknown Protein (AHRD V1)	6.14	8.19	9.05	8.68	8.02	8.53	3.63	7.05	4.41	5.91	0.74	1	12.84	14.67	22.03	16.51	15.96	19.86	13.26	16.36	0.99	1	9.92	7.5	10.66	9.36	10.08	9.3	5.59	8.32	0.89	1	26.18	33.43	21.23	26.95	7.02	10.76	8.89	0.33	0.570472
Solyc01g057740.1.1	RNase H family protein (AHRI	0	0	0	0	0	0	0	0	0	0	1	NA	0	0	0	0	0	0	0	0	1	NA	0	0	0	0	0	0	0	0	1	NA	0	0	0	0	0	0	0	1	NA
Solyc01g057750.1.1	DNAJ heat shock N-terminal d	0	0	0	0	0	0	0	0	0	0	1	NA	0	0	0	0	0	0	0	0	1	NA	0	0	0	0	0	0	0	0	1	NA	0	0	0	0	0	0	0	1	NA
Solyc01g057760.2.1	ATP dependent RNA helicase	21.32	17.63	26.11	26.96	23.01	13.74	18.33	13.15	15.5	15.18	0.66	0.275421	20.84	24.26	27.09	24.06	22.58	19.26	15.91	19.25	0.8	0.819112	18.45	23.23	23.9	21.86	22.91	18.7	22.03	21.21	0.97	1	38.38	28.39	34.95	33.91	27.53	31.67	29.6	0.87	1
Solyc01g057770.2.1	AE family transporter anion e	6.65	6.17	5.01	17.51	8.84	5.24	10.43	4.67	4.84	6.29	0.71	1	4.3	14.3	32.31	16.97	8.72	11.72	5.01	8.48	0.5	0.937299	2.31	11.67	40.54	18.17	13.35	20.38	5.52	13.08	0.72	1	5.91	38.79	41.35	28.68	49.48	59.79	54.63	1.9	0.853779
Solyc01g057780.2.1	Zinc finger protein VAR3, chlc	16.44	14.96	17.26	14.18	15.71	17.93	23.57	15.6	20.03	19.28	1.23	0.542955	19.08	17.17	22.99	19.75	29.49	23.54	21.75	24.93	1.26	0.552585	10.96	12.29	13.47	12.24	17.88	13.26	16.21	15.78	1.29	1	24.55	26.61	24.7	25.29	31.96	29.48	30.72	1.21	1
Solyc01g057790.1.1	Unknown Protein (AHRD V1)	0	0	0	0	0	0	0	0	0	0	1	NA	0	0	0	0	0	0	0	0	1	NA	0	0	0	0	0	0	0	0	1	NA	0	0	0	0	0	0	0	1	NA
Solyc01g057800.1.1	Unknown Protein (AHRD V1)	0	0	0	0	0	0	0	0	0	0	1	NA	0	0	0	0	0	0	0	0	1	NA	0	0	0	0	0	0	0	0	1	NA	0	0	0	0	0	0	0	1	NA
Solyc01g057810.1.1	Unknown Protein (AHRD V1)	0	0	0	0	0	0	0	0	0	0	1	NA	0	0	0	0	0	0	0	0	1	NA	0	0	0	0	0	0	0	0	1	NA	0	0	0	0	0	0	0	1	NA
Solyc01g057820.1.1	Ribosomal protein-like (AHRD	0	0	0	0	0	0	0	0	0	0	1	NA	0	0	0	0	0	0	0	0	1	NA	0	0	0	0	0	0	0	0	1	NA	0	0	0	0	0	0	0	1	NA
Solyc01g057830.2.1	30S ribosomal protein S1 (AH	101.91	90.27	71.42	78.86	85.62	189.81	209.47	131.01	229.24	189.88	2.22	0.000226	69.69	91.09	81.36	80.71	186.46	158.22	170.97	171.88	2.13	3.88E-05	29.55	81.22	41.07	50.61	64.01	69.49	99.29	77.6	1.53	0.876677	14.76	41.53	23.34	26.54	74.57	60.64	67.6	2.55	0.147272
Solyc01g057840.2.1	Serologically defined colon ca	14.36	11.36	16.13	14.51	14.09	12.03	10.25	15.29	12.35	12.48	0.89	1	12.08	11.79	14.15	12.67	13.59	12.51	16.21	14.1	1.11	1	7.82	9.47	10.86	9.38	12.2	7.34	10.05	9.86	1.05	1	21.22	22.67	24.35	22.75	15.75	17.38	16.56	0.73	1
Solyc01g057850.2.1	Serologically defined colon ca	9.97	9.67	10.57	11.57	10.45	5.23	6.63	8.34	11.18	7.84	0.75	0.800128	10.31	10.52	11.45	10.76	11.11	10.32	12.41	11.28	1.05	1	7.06	9.4	8.23	8.23	7.65	7.47	10.37	8.5	1.03	1	12.9	13.91	12.75	13.19	14.76	15.91	15.34	1.16	1
Solyc01g057860.1.1	Unknown Protein (AHRD V1)	0	0	0	0	0	0	0	0	0	0	1	NA	0	0	0	0	0	0	0	0	1	NA	0	0	0	0	0	0	0	0	1	NA	0	0	0	0	0	0	0	1	NA
Solyc01g057870.1.1	Unknown Protein (AHRD V1)	0	0	0	0	0	0	0	0	0.61	0.15	15.25	1	0	0	0	0	0.59	0	0.66	0.42	41.67	1	0	0.69	0	0.23	0	0.64	0	0.21	0.93	1	0	0	0	0	0	0	0	1	NA
Solyc01g057880.1.1	Ulp1 protease family C-termi	0	0.1	0	0	0.03	0	0	0	0	0	0.4	1	0	0	0	0	0	0	0	0	1	NA	0	0	0	0	0	0	0	0	1	NA	0	0	0	0	0	0	0	1	NA
Solyc01g057890.1.1	Mutator-like transposase (AH	0	0	0	0	0	0	0	0	0	0	1	NA	0	0	0	0	0	0	0	0	1	NA	0	0	0	0	0	0	0	0	1	NA	0	0	0	0	0	0	0	1	NA
Solyc01g057900.2.1	Ubiquitin-protein ligase (AHR	12.99	17.82	14.72	12.85	14.6	15.01	13.79	16.47	15.85	15.28	1.05	1	16.19	16.02	14.43	15.55	15.27	14.94	20.16	16.79	1.08	1	17.73	17.1	16.96	17.26	13.24	16.78	17.25	15.76	0.91	1	12.55	15.06	15.65	14.42	16.28	16.77	16.52	1.15	1
Solyc01g057910.2.1	MYB transcription factor (AHI	10.08	6.52	7.84	5.83	7.57	8.4	12.84	6.95	6.74	8.73	1.15	0.956053	8.1	12.35	10.45	10.3	13.82	11.86	10.26	11.98	1.16	0.963902	8.7	10.45	3.18	7.44	5.42	2.43	6.18	4.68	0.63	1	3.58	1.33	2.82	2.58	1.05	1.81	1.43	0.55	1
Solyc01g057920.1.1	Unknown Protein (AHRD V1)	0	0	0	0	0	0	0	0	0	0	1	NA	0	0	0	0	0	0	0	0	1	NA	0	0	0	0	0	0	0	0	1	NA	0	0	0	0	0	0	0	1	NA
Solyc01g057930.1.1	Transposase family protein (A	0	0	0	0	0	0	0	0	0	0	1	NA	0	0	0	0	0	0	0	0	1	NA	0	0	0	0	0	0	0	0	1	NA	0	0	0	0	0	0	0	1	NA
Solyc01g057940.1.1	Unknown Protein (AHRD V1);	0	0	0	0	0	0	0	0	0	0	1	NA	0	0	0	0	0	0	0	0	1	NA	0	0	0	0	0	0	0	0	1	NA	0	0	0	0	0	0	0	1	NA
Solyc01g057960.1.1	Class III homeodomain-leucin	0	0	0	0	0	0	0	0	0	0	1	NA	0	0	0	0	0	0	0	0	1	NA	0	0	0	0	0	0	0	0	1	NA	0	0	0	0	0	0	0	1	NA
Solyc01g057970.1.1	Unknown Protein (AHRD V1)	0	0	0	0	0	0	0	0	0	0	1	NA	0	0	0	0	0	0	0	0	1	NA	0	0	0	0	0	0	0	0	1	NA	0	0	0	0	0	0	0	1	NA
Solyc01g057980.2.1	Unknown Protein (AHRD V1)	0	0	0	0	0	0	0	0	0	0	1	NA	0	0	0	0	0	0	0	0	1	NA	0	0	0	0	0	0	0	0	1	NA	0	0	0	0	0	0	0	1	NA
Solyc01g057990.1.1	Unknown Protein (AHRD V1)	0	0	0	0	0	0	0	0	0	0	1	NA	0	0	0	0	0	0	0	0	1	NA	0	0	0	0	0	0	0	0	1	NA	0	0	0	0	0	0	0	1	NA
Solyc01g058000.2.1	HR7 protein (AHRD V1 ***- Q	0	0	0	0	0	0	0	0	0	0	1	NA	0	0	0	0	0	0	0	0	1	NA	0	0	0	0	0	0	0	0	1	NA	0	0	0	0	0	0	0	1	NA
Solyc01g058010.2.1	Neutral invertase (AHRD V1 *	9.63	7.18	6.45	8.47	7.93	7.52	7.5	9.81	6.82	7.91	1	1	7.26	9.05	6.26	7.52	5.41	6.06	9.1	6.86	0.91	1	5.89	7.63	7.88	7.13	9.57	5.23	6.07	6.96	0.98	1	7.03	15.4	11.41	11.28	6.67	8.1	7.38	0.65	0.821561
Solyc01g058020.2.1	Neutral invertase (AHRD V1 *	16.38	12.49	15.46	15.88	15.05	13.52	12.19	15.49	10.75	12.99	0.86	1	15.67	14.64	12.25	14.19	7.46	9.69	9.19	8.78	0.62	0.613683	8.32	12.58	14.86	11.92	14.75	4.96	8.86	9.52	0.8	1	10.64	16.99	14.39	14.01	6	9.03	7.51	0.54	0.848276
Solyc01g058030.1.1	Gibberellin 2-oxidase (AHRD \	0	0	0	0	0	0	0	0	0	0	1	NA	0	0	0	0	0	0	0	0	1	NA	0	0	0	0	0	0	0	0	1	NA	0	0	0	0	0	0	0	1	NA
Solyc01g058040.1.1	Gibberellin 2-oxidase (AHRD \	0	0	0	0	0	0	0	0	0	0	1	NA	0	0	0	0	0	0	0	0	1	NA	0	0	0	0	0	0	0	0	1	NA	0	0	0	0	0	0	0	1	NA
Solyc01g058050.1.1	Unknown Protein (AHRD V1)	0	0	0	0	0	0	0	0	0	0	1	NA	0	0	0	0	0	0	0	0	1	NA	0	0	0	0	0	0	0	0	1	NA	0	0	0	0	0	0	0	1	NA
Solyc01g058060.1.1	Unknown Protein (AHRD V1)	0	0	0	0	0	0	0	0	0	0	1	NA	0	0	1.57	0.52	0	0	0	0	0.02	1	0	0	0	0	0	0.7	0	0.23	23.33	NA	0	0	0	0	0	0	0	1	NA
Solyc01g058070.1.1	Unknown Protein (AHRD V1)	0	0	0	0	0	0	0	0	0	0	1	NA	0	0	0	0	0	0	0	0	1	NA	0	0	0	0	0	0	0	0	1	NA	0	0	0	0	0	0	0	1	NA
Solyc01g058080.1.1	Unknown Protein (AHRD V1)	0	0	0	0	0	0	0	0	0	0	1	NA	0	0	0	0	0	0	0	0	1	NA	0	0	0	0	0	0	0	0	1	NA	0	0	0	0	0	0	0	1	NA
Solyc01g058090.1.1	Unknown Protein (AHRD V1)	0	0	0	0	0	0	0	0	0	0	1	NA	0	0	0	0	0	0	0	0	1	NA	0	0	0	0	0	0	0	0	1	NA	0	0	0	0	0	0	0	1	NA
Solyc01g058100.2.1	Unknown Protein (AHRD V1)	0	0	0	0	0	0	0	0	0	0	1	NA	0	0	0	0	0	0	0	0	1	NA	0	0	0	0	0	0	0	0	1	NA	0	0	0	0	0	0	0	1	NA
Solyc01g058120.1.1	Unknown Protein (AHRD V1)	0	0	0	0	0	0	0	0	0	0	1	NA	0	0	0	0	0	0	0	0	1	NA	0	0	0	0	0	0	0	0	1	NA	0	0	0	0	0	0	0	1	NA
Solyc01g058130.1.1	Unknown Protein (AHRD V1);	0	0	0	0	0	0	0	0	0	0	1	NA	0	0	0	0	0	0	0	0	1	NA	0	0	0	0	0	0	0	0	1	NA	0	0	0	0	0	0	0	1	NA
Solyc01g058140.2.1	Beta-glucosidase A (AHRD V1	36.01	27.58	38.13	34.98	34.17	34.1	32.56	27.07	29.91	30.91	0.9	1	32.73	31.22	34.84	32.93	35.01	34.32	30.56	33.3	1.01	1	14.17	16.64	17.82	16.21	19.88	21.07	16.9	19.28	1.19	1	41.28	31.07	37.08	36.48	34.56	38.41	36.48	1	1
Solyc01g058150.1.1	Ulp1 protease family C-termi	0	0	0	0	0	0	0	0	0	0	1	NA	0	0	0	0	0	0	0	0	1	NA	0	0	0	0	0	0	0	0	1	NA	0	0	0	0	0	0	0	1	NA
Solyc01g058160.2.1	Agenet domain-containing pr	11.28	8.87	11.09	8.24	9.87	14	10.54	11.58	12.82	12.23	1.24	0.747964	10.14	6.7	9.52	8.79	11.6	11	8.57	10.39	1.18	0.903176	1.82	3.52	2.05	2.46	3.33	1.28	3.01	2.54	1.03	1	1.6	0.68	0.58	0.95	1.38	1.48	1.43	1.5	1
Solyc01g058170.2.1	Agenet domain-containing pr	15.99	13.73	17.18	10.5	14.35	20.63	18.26	23.11	22.14	21.04	1.47	0.317992	16.47	10.43	13.51	13.47	13.67	16.12	13.92	14.57	1.08	1	3	4.65	2.55	3.4	3.56	1.73	3.12	2.8	0.82	1	0.81	1.66	0.44	0.97	2.35	2.75	2.55	2.63	1
Solyc01g058190.2.1	30S ribosomal protein S6 (AH	45.71	41.06	25.18	19.21	32.79	52.72	55.83	58.87	61.29	57.18	1.74	0.021868	36.7	35.8	22.4	31.63	55.77	56.93	56.42	56.37	1.78	0.007682	45.98	52.88	34.67	44.51	74.78	70.75	83.89	76.47	1.72	1	57.01	103.82	84.87	81.9	100.26	80.72	90.49	1.1	1
Solyc01g058200.2.1	Unknown Protein (AHRD V1)	0	0	3.24	0	0.81	0	0	5.05	0	1.26	1.56	1	0	0	2.32	0.77	0	0	0	0	0.01	1	0	0	0	0	0	0	0	0	1	1	0	0	0	0	0	0	0	1	NA
Solyc01g058210.1.1	Gelsolin (AHRD V1 **-- Q9DG	0	0	0	0	0	0	0	0.19	0	0.05	4.75	1	0	0	0	0	0	0	0	0	1	NA	0	0	0	0	0	0	0	0	1	NA	0	0	0.32	0.11	0	0	0	0.09	1
Solyc01g058220.1.1	At2g42220/T24P15.13 (Fragr	17.42	7.99	11.21	11.2	11.96	9.97	6.25	11.65	10.23	9.52	0.8	0.978072	6.87	6.42	11.59	8.29	9.52	9.29	12.44	10.42	1.26	1	2.98	3.01	5.24	3.74	3.81	3.2	5	4	1.07	1	1.5	3.45	6.49	3.81	4.35	5.09	4.72	1.24	1
Solyc01g058230.1.1	Unknown Protein (AHRD V1)	0	0	0	0	0	0	0	0	0	0	1	NA	0	0	0	0	0	0	0	0	1	NA	0	0	0	0	0	0	0	0	1	NA	0	0	0	0	0	0	0	1	NA
Solyc01g058240.1.1	Unknown Protein (AHRD V1)	0	0	0	0	0	0	0	0	0	0	1	NA	0	0	0	0	0	0	0	0	1	NA	0.9	1.82	0	0.91	0	0	0	0	0.01	1	4.77	0.81	0	1.86	1.02	0	0.51	0.27	1
Solyc01g058250.1.1	Gibberellin 3-beta-hydroxylas	0	0	0	0	0	0	0	0	0	0	1	NA	0	0	0	0	0	0	0	0	1	NA	0	0	0	0	0	0	0	0	1	NA	0	0	0	0	0	0	0	1	NA
Solyc01g058260.2.1	Poly(A) polymerase (AHRD V1	80.54	89.42	142.69	69.6	95.56	111.37	84.42	128.77	97.68	105.56	1.1	0.974245	103.43	87.22	88.69	93.11	69.02	70.02	67.18	68.74	0.74	0.536202	72.95	73.8	80.49	75.75	55.62	50	68.61	58.08	0.77	1	67.03	70.87	54.1	64	46.68	45.67	46.17	0.72	0.94508
Solyc01g058270.2.1	CAS1 domain containing 1 (A	14.11	18.14	8.96	10.92	13.03	22.25	18.47	19.96	15.5	19.05	1.46	0.301702	10.99	9.33	7.33	9.22	3.26	6.31	8.91	6.16	0.67	0.698814	0.97	1.87	0.84	1.23	0.24	0.37	0.44	0.35	0.29	0.985231	0	0	0.19	0.06	0	0	0	0.16	1
Solyc01g058280.2.1	Transmembrane protein 93 (	146.53	172.75	127.23	112.16	139.67	172.05	189.8	155.14	154.84	167.96	1.2	0.739052	164.03	160.46	98.58	141.02	112.63	137.94	137.09	129.22	0.92	1	137.86	140.08	83.74	120.56	93.34	105.77	120.65	106.59	0.88	1	69.45	83.85	117.05	90.12	105.68	140.16	122.92	1.36	0.986172
Solyc01g058290.1.1	Unknown Protein (AHRD V1)	0	0	0	0	0	0	0	0	0	0	1	NA	0	0	0	0	0	0	0	0	1	NA	0	0	0	0	0	0	0	0	1	NA	0	0	0	0	0	0	0	1	NA
Solyc01g058300.1.1	Unknown Protein (AHRD V1)	0	0	0	0	0	0	0	0	0	0	1	NA	0	0	0	0	0	0	0	0	1	NA	0	0	0	0	0	0	0	0	1	NA	0	0	0	0	0	0	0	1	NA
Solyc01g058310.1.1	Unknown Protein (AHRD V1)	0	0	0	0	0	0	0	0	0	0	1	NA	0	0	0	0	0	0	0	0	1	NA	0	0	0	0	0	0	0	0	1	NA	0	0	0	0	0	0	0	1	NA
Solyc01g058320.2.1	T6A9.7 protein (AHRD V1 *-*	278.96	264.63	218.49	224.96	246.76	238.85	291.85	303.93	183.7	254.58	1.03	1	181.25	92.57	83.78	119.2	32.03	56.48	88.14	58.88	0.49	0.587995	2.28	4.03	3.74	3.35	2.63	0.89	3.78	2.43	0.73	1	4.35	1.2	3.99	3.18	3.67	7.44	5.55	1.75	0.814112
Solyc01g058330.1.1	Unknown Protein (AHRD V1)	0	0	0	0	0	0	0	0	0	0	1	NA	0	0	0	0	0	0	0	0	1	NA	0	0	0	0	0	0	0	0	1	NA	0	0	0	0	0	0	0	1	NA
Solyc01g058340.1.1	Unknown Protein (AHRD V1)	0	0	0	0	0	0.52	0	0	1.01	0.38	38.25	0.917205	0	0.81	0	0.27	0.32	0.24	0	0.19	0.69	1	0	0	0	0	1.23	0.71	2.73	1.56	155.67	0.744388	0.67	0.68	0	0.45	2.14	0.82	1.48	3.29	1
Solyc01g058350.1.1	Unknown Protein (AHRD V1)	0	2.3	0	0	0.57	1.44	3.06	2.38	0	1.72	2.99	1	1.2	0	3.28	1.49	0	2.01	1.02	1.01	0.68	1	1.05	0	0	0.35	0	0	0	0	0.03	1	1.84	0	0	0.61	0	0	0	0.02	1
Solyc01g058360.1.1	Major facilitator superfamily	0	0	0.22	0.27	0.12	0	0	0.17	0	0.04	0.35	1	0	0	0	0	0	0.1	0	0.03	3.33	1	0	0	0.56	0.19	0.25	0.86	0.28	0.46	2.48	1	0.81	0.28	0	0.36	0.87	0.17	0.52	1.43	1
Solyc01g058370.1.1	Helitron helicase-like protein	0	0	0	0	0	0	0	0	0	0	1	NA	0	0	0	0	0	0	0	0	1	NA	0	0	0	0	0	0	0	0	1	NA	0	0	0	0	0	0	0	1	NA
Solyc01g058380.1.1	Coatomer subunit gamma (AH	5.45	2.96	4.64	7.7	5.19	2.02	4.78	1.67	3.26	2.93	0.57	0.687659	4.79	3.42	4.35	4.19	2.1	1.72	2.14	1.99	0.47	0.482313	1.96	5.18	1.5	2.88	2.19	2.52	3.53	2.75	0.95	1	4.3	1.98	4.19	3.49	3.05	4.25	3.65	1.05	1
Solyc01g058390.2.1	Galactokinase (AHRD V1 ****	28.71	34.22	32.93	23.1	29.74	31.03	18.82	26.76	24.96	25.39	0.85	0.85048	27.8	28.16	19.8	25.25	18.81	16.91	21.94	19.22	0.76	0.67425	10.43	14.81	4.97	10.07	6.06	3.49	9.98	6.51	0.65	1	8.42	8.03	5.47	7.31	7.84	7.97	7.9	1.08	1
Solyc01g058400.1.1	Unknown Protein (AHRD V1)	0	0	0	0	0	0	0	3.79	1.48	1.32	131.75	0.729692	0	0	0	0	2.86	4.27	1.62	2.92	291.67	0.25447	0	0	0	0	3.39	0	2.25	1.88	188	1	0	0.75	0	0.25	0.94	2.71	1.82	7.3	1
Solyc01g058410.1.1	Unknown Protein (AHRD V1)	0	0	0	0	0	0.34	0	0.28	0	0.16	15.5	1	0	0	0	0	0	0	0	0	1	NA	0	0	0	0	0	0	0	0	1	NA	0	0	0	0	0	0	0	1	NA
Solyc01g058420.1.1	Ribosomal protein S3 (Fragm	0	0	0	0	0	0	0	0	0	0	1	NA	0	0	0	0	0	0	0	0	1	NA	0	0	0	0	0	0	0	0	1	NA	0	0	0	0	0	0	0	1	NA
Solyc01g058430.1.1	Mutator-like transposase (AH	0	0	0	0	0	0	0	0.13	0	0.03	3.25	1	0	0	0	0	0	0	0	0	1	NA	0	0	0	0	0	0	0	0	1	NA	0	0.1	0	0.03	0	0	0	0.3	1
Solyc01g058440.1.1	Unknown Protein (AHRD V1)	0	0	0	0	0	0	0	0	0	0	1	NA	0	0	0	0	0.19	0	0	0.06	6.33	1	0	0	0	0	0	0	0	0	1	NA	0	0	0	0	0	0	0	1	NA
Solyc01g058450.2.1	RNA binding protein (AHRD V	16.32	13.1	15.59	18.19	15.8	13.11	20.44	9.29	13.94	14.19	0.9	1	16.41	17.88	18.95	17.75	16.79	16.39	14.22	15.8	0.89	1	15.9	15.68	13.99	15.19	13.92	13.31	14.6	13.94	0.92	1	20.68	17.2	18.41	18.76	20.05	21.3	20.68	1.1	1
Solyc01g058460.1.1	Unknown Protein (AHRD V1)	0	0	0	0	0	0	0	0	0	0	1	NA	0	0	0	0	0	0	0	0	1	NA	0	0	0	0	0	0	0	0	1	NA	0	0	0	0	0	0	0	1	NA
Solyc01g058470.1.1	Unknown Protein (AHRD V1)	2.03	4.52	0	1.44	2	5.64	5.61	14.93	5.83	8	4.01	0.214714	2.83	8.82	2.57	4.74	2.82	7.88	6.38	5.69	1.2	1	4.1	0.83	4.03	2.99	0.67	0.77	1.48	0.97	0.33	1	0	9.58	7.8	5.79	1.86	0.89	1.38	0.24	0.96512
Solyc01g058480.1.1	Receptor protein kinase-like p	0	0	0	0	0	0	0	0	0	0	1	NA	0	0	0	0	0	0	0	0	1	NA	0	0	0	0	0	0	0	0	1	NA	0	0	0	0	0	0	0	1	NA
Solyc01g058490.1.1	Unknown Protein (AHRD V1)	0	0	0	0	0	0	0	0	0	0	1	NA	0	0	0	0	0	0	0	0	1	NA	0	0	0	0	0	0	0	0	1	NA	0	0	0	0	0	0	0	1	NA
Solyc01g058500.2.1	Unknown Protein (AHRD V1)	0	0	0	0	0	0	0	0	0	0	1	NA	0	0	0	0	0	0	0	0	1	NA	0	0	0	0	0	0	0	0	1	NA	0	0	0	0	0	0	0	1	NA
Solyc01g058510.1.1	Unknown Protein (AHRD V1)	0	0	0	0	0	0	0	0	0	0	1	NA	0	0	0	0	0	0	0	0	1	NA	0	0	0	0	0	0.57	0	0.19	19	NA	0	0	0	0	0.69	0	0.34	34.5	1
Solyc01g058520.2.1	Peroxidase 40 (AHRD V1 ***-	0.99	1.94	1.4	1.68	1.5	0.88	0.63	1.82	0.85	1.04	0.7	0.963372	0.74	0.86	1	0.87	0.14	0.92	0.31	0.46	0.53	1	0.32	0.48	1.38	0.73	0.39	0	0.72	0.37	0.51	1	0.28	0.29	0.61	0.39	0.54	0.52	0.53	1.35	1
Solyc01g058530.2.1	30S ribosomal protein S18 fa	10.9	10.68	6.43	6.17	8.54	4.24	7.32	7.02	6.65	6.31	0.74	0.91296	8.62	7.58	4.61	6.94	3.4	6.21	9.85	6.49	0.94	1	3.96	3.11	4.33	3.8	2.87	1.65	2.38	2.3	0.61	1	3.1	1.58	3.35	2.68	1.5	3.82	2.66	0.99	1
Solyc01g058540.2.1	WRKY transcription factor 31	4.06	5.6	3.59	2.3	3.89	4.06	3.04	6.16	2.48	3.94	1.01	1	3.21	4.59	6	4.6	3.38	2.42	3.67	3.16	0.69	0.792247	0	0	0	0	0	0	0.3	0.1	10	1	0	0	0.31	0.1	0	0.18	0.09	0.87	1
Solyc01g058550.1.1	Unknown Protein (AHRD V1)	0	0	0	0	0	0	0	0	0	0	1	NA	0	0	0	0	0	0	0	0	1	NA	0	0	0	0	0	0	0	0	1	NA	0	0	0	0	0	0	0	1	NA
Solyc01g058560.1.1	Unknown Protein (AHRD V1)	0	0	0	0	0	0	0	0	0	0	1	NA	0	0	0	0	0	0	0	0	1	NA	0	0	0	0	0	0	0	0	1	NA	0	0	0	0	0	0	0	1	NA
Solyc01g058570.1.1	Ulp1 protease family C-termi	0	0	0	0	0	0	0	0	0	0	1	NA	0	0	0	0	0	0	0	0	1	NA	0	0	0	0	0	0	0	0	1	NA	0	0	0	0	0	0	0	1	NA
Solyc01g058580.1.1	Retrovirus-related Pol polypro	0	0	0	0	0	0	0	0	0	0	1	NA	0	0	0	0	0	0	0	0	1	NA	0	0	0	0	0	0	0	0	1	NA	0	0	0	0	0	0	0	1	NA
Solyc01g058590.1.1	Ubiquitin-like domain contair	1.5	0.44	0	0.71	0.66	0	0.79	0	0.72	0.38	0.57	1	1.86	1.74	0.42	1.34	0	0	0.39	0.13	0.1	0.487861	0	0.81	0	0.27	0	0	0.73	0.24	0.9	1	1.42	0.73	0	0.72	0	0.88	0.44	0.61	1
Solyc01g058600.1.1	Unknown Protein (AHRD V1)	0	0	0	0	0	0	0	0	0	0	1	NA	0	0	0	0	0	0	0	0	1	NA	0	0	0	0	0	0	0	0	1	NA	0	0	0	0	0	0	0	1	NA
Solyc01g058610.1.1	MuDR family transposase cor	0	0	0	0	0	0	0	0	0	0	1	NA	0	0	0	0	0	0	0	0	1	NA	0	0	0	0	0	0	0	0	1	NA	0	0	0	0	0	0	0	1	NA
Solyc01g058620.1.1	Unknown Protein (AHRD V1)	0	0	0	0	0	0	0	0	0	0	1	NA	0	0	0	0	0	0	0	0	1	NA	0	0	0	0	0	0	0	0	1	NA	0	0	0	0	0	0	0	1	NA
Solyc01g058630.1.1	Unknown Protein (AHRD V1)	0	0	0	0	0	0	0	0	0	0	1	NA	0	0	0	0	0	0	0	0	1	NA	0	0	0	0	0	0	0	0	1	NA	0	0	0	0	0	0	0	1	NA
Solyc01g058640.1.1	Unknown Protein (AHRD V1)	0	0	0	0	0	0	0	0	0	0	1	NA	0	0	0	0	0	0	0	0	1	NA	0	0	0	0	0	0	0	0	1	NA	0	0	0	0	0	0	0	1	NA
Solyc01g058650.2.1	Prefoldin subunit 3 (AHRD V1	0	0	0	0	0	0	0	0	0	0	1	NA	0	0	0	0	0	0	0	0	1	NA	0	0	0	0	0	0	0	0	1	NA	0	0	0	0	0	0	0	1	NA
Solyc01g058660.1.1	Unknown Protein (AHRD V1)	0	0	0	2.33	0.58	0	0	0	0	0	0.02	1	0	0	0	0	0	0.85	0	0.28	28.33	1	0	0	0	0	0	0	0	0	1	NA	0	0	0	0	0	0	0	1	NA
Solyc01g058670.2.1	Transcription factor (AHRD V	139.88	151.05	174.4	212.57	169.48	200.83	164.46	205.38	167.64	184.58	1.09	0.878681	121.2	131.83	172.25	141.76	66.26	100.49	93.58	86.78	0.61	0.086719	32.73	34.8	48.91	38.81	46.22	44.19	39.3	43.24	1.11	1	65.78	67	55.99	62.92	45.36	44.16	44.76	0.71	0.974111
Solyc01g058680.1.1	Unknown Protein (AHRD V1)	0	0	0	0	0	0	0	0	0	0	1	NA	0	0	0	0	0	0	0	0	1	NA	0	0	0	0	0	0	0	0	1	NA	0	0	0	0	0	0	0	1	NA
Solyc01g058690.1.1	Zinc finger CCCH domain-cont	0	0	0	0	0	0	0.39	0	0	0.1	9.75	1	0	0	0	0	0	0	0	0	1	NA	0	0	0	0	0	0	0	0	1	NA	0	0	0	0	0	0	0	1	NA
Solyc01g058700.1.1	Unknown Protein (AHRD V1)	0	0	0	0	0	0	0	0	0	0	1	NA	0	0	0	0	0	0	0	0	1	NA	0	0	0	0	0	0	0	0	1	NA	0	0	0	0	0	0	0	1	NA
Solyc01g058710.1.1	Unknown Protein (AHRD V1)	0	0	0	0	0	0	0	0	0	0	1	NA	0	0	0	0	0	0	0	0	1	NA	0	0	0	0	0	0	0	0	1	NA	0	0	0	0	0	0	0	1	NA
Solyc01g058720.2.1	NaCl-inducible Ca2+-binding p	5.5	1.63	1.62	1.94	2.67	4.58	12.3	9.27	4.93	7.77	2.91	0.159764	4.26	5.18	2.71	4.05	4.45	3.8	2.16	3.47	0.86	1	0	0	0	0	0.3	0	0	0.1	10	1	0.65	0.67	0	0.44	0	1.61	0.8	1.83	1
Solyc01g058730.2.1	Os03g0310500 protein (Fragr	38	36	54.65	52.25	45.23	27.92	32.96	30.97	35.54	31.85	0.7	0.452085	45.21	50.68	67.15	54.35	55.54	51.34	41.27	49.38	0.91	1	27.28	40.4	37.56	35.08	30.43	28.18	31.61	30.07	0.86	1	46.15	35.24	39.88	40.42	38.67	51.26	44.97	1.11	1
Solyc01g058740.2.1	Unknown Protein (AHRD V1)	0	0	0	0	0	0	0	0	0	0	1	NA	0	0	0	0	0	0	0	0	1	NA	0	0	0	0	0	0	0	0	1	NA	0	0.67	0	0.22	0	0	0	0.04	1
Solyc01g059740.1.1	Unknown Protein (AHRD V1)	0	0	0	0	0	0	0	0	0	0	1	NA	0	0	0	0	0	0	0	0	1	NA	0	0	0	0	0	0	0	0	1	NA	0	0	0	0	0	0	0	1	NA
Solyc01g059750.1.1	Unknown Protein (AHRD V1)	0	0	0.53	0	0.13	2.49	1.42	0.83	0	1.19	8.94	0.495183	0.83	0	0.76	0.53	1.25	1.16	0.35	0.92	1.74	1	0	0	0.45	0.15	0.59	1.36	0	0.65	4.33	1	1.91	0.33	0.69	0.98	1.23	1.18	1.21	1.23	1
Solyc01g059760.1.1	Ulp1 protease family C-termi	0	0.08	0	0	0.02	0.29	0	0	0	0.07	3.62	1	0.16	0.08	0.15	0.13	0.12	0	0	0.04	0.31	1	0.07	0	0	0.02	0	0	0	0	0.43	1	0	0	0	0	0	0.08	0.04	4	1

Solyc01g059780.1.1	Unknown Protein (AHRD V1)	0	0	0	0	0	0	0	0	0	0	1	NA	0	0	0	0	0	0	0	0	1	NA	0	0	0	0	0	0	0	0	1	NA	0	0	0	0	0	0	0	1	NA
Solyc01g059790.1.1	Unknown Protein (AHRD V1)	0	0	0	0	0	0	0	0	0	0	1	NA	0	0	0	0	0	0	0	0	1	NA	0	0	0	0	0	0	0	0	1	NA	0	0	0	0	0	0	0	1	NA
Solyc01g059800.1.1	Unknown Protein (AHRD V1)	0	0	0	0	0	0	0	0	0	0	1	NA	0	0	0	0	0	0	0	0	1	NA	0	0	0	0	0	0	0	0	1	1	0	0	0	0	0	0	0	1	NA
Solyc01g059810.1.1	Unknown Protein (AHRD V1)	0	0	0	0	0	0	0	0	0	0	1	NA	0	0	0	0	0	0	0	0	1	NA	0	0	0	0	0	0	0	0	1	NA	0	0	0	0	0	0	0	1	NA
Solyc01g059820.1.1	Unknown Protein (AHRD V1)	0	0	0	0	0	0	0	0	0	0	1	NA	0	0	0	0	0	0	0	0	1	NA	0	0	0	0	0	0	0	0	1	NA	0	0	0	0	0	0	0	1	NA
Solyc01g059830.2.1	Enoyl-CoA hydratase (AHRD V	46.87	44.15	56.76	65.95	53.43	45.76	49.54	47.2	51.64	48.53	0.91	1	42.91	57.56	57.61	52.69	62.61	57.66	51.65	57.31	1.09	1	82.75	104.69	90.1	92.51	99.87	68.24	97.96	88.69	0.96	1	86.39	53.5	43.86	61.25	65.61	55.23	60.42	0.99	1
Solyc01g059840.1.1	GDSL esterase/lipase At4g162	0	0	0	0	0	0	0	0.17	0	0.04	4.25	1	0	0	0	0	0	0	0	0	1	NA	0	0	0	0	0	0	0	0	1	NA	0	0	0	0	0	0	0	1	NA
Solyc01g059850.2.1	Unknown Protein (AHRD V1);	0	0	2.36	0	0.59	0	0	0	0	0	0.02	1	0	0	0	0	0	0	0	0	1	NA	0	0	0	0	0	0	0	0	1	NA	0	0	0	0	0	0	0	1	NA
Solyc01g059860.2.1	Kinase family protein (AHRD	19.16	20.29	22.91	16.8	19.79	13.68	15.13	14.35	18.24	15.35	0.78	0.696818	21.69	20.65	20.14	20.83	28.74	23.3	25.68	25.91	1.24	0.622228	8.96	12.43	9.72	10.37	6.44	5.72	11.43	7.86	0.76	1	1.36	3.21	2.53	2.37	2.94	2.74	2.84	1.2	1
Solyc01g059870.2.1	Phytochrome B1	12.3	17.27	22.21	19.62	17.85	15.94	19.23	16.66	16.61	17.11	0.96	1	12.41	13.12	14.03	13.19	10.95	11.44	12.92	11.77	0.89	1	3.11	4.89	3.18	3.73	2.68	2.08	2.85	2.54	0.68	1	2.73	3.17	2.24	2.71	3.36	3.58	3.47	1.28	1
Solyc01g059880.2.1	ATP citrate lyase a-subunit (A	59.47	54.57	42.94	48.73	51.43	51.9	44.56	46.81	54.59	49.47	0.96	1	44.94	37.1	33.49	38.51	19.08	20.98	30.73	23.6	0.61	0.088531	30.19	33.9	26.56	30.22	17.79	12.73	21.08	17.2	0.57	1	0	1.81	1.17	0.99	2.97	4.94	3.95	3.98	0.206424
Solyc01g059890.1.1	Unknown Protein (AHRD V1)	0	0	0	0	0	0	0	0.71	0	0.18	17.75	1	0	0	0	0	0	0	0	0	1	NA	0	0	0	0	0	0	0	0	1	NA	0	0	0	0	0	0	0	1	NA
Solyc01g059900.2.1	Dirigent-like protein (AHRD V	0	0	0	0	0	0.42	0	0	0	0.1	10.5	1	0	0	0	0	0	0.2	0	0.07	6.67	1	0	0	0	0	0	0	0	0	1	NA	0	0	0	0	0	0	0	1	NA
Solyc01g059910.2.1	Pto-like, Serine/threonine kin	0	0	0	0	0	0	0	0	0	0	1	NA	0	0	0	0	0	0	0	0	1	NA	0	0	0	0	0	0	0	0	1	NA	0	0	0	0	0	0	0	1	NA
Solyc01g059920.2.1	Genomic DNA chromosome 3	2.08	3	1.76	2.95	2.45	1.65	2.27	2.65	1.78	2.09	0.85	1	1.48	1.72	1.26	1.49	1.24	1.34	1.87	1.48	1	1	0.72	0.65	0.39	0.59	0.59	0.3	0.36	0.42	0.71	1	0.99	1.22	1.37	1.19	1.09	1.13	1.11	0.93	1
Solyc01g059930.2.1	Universal stress protein (AHR	221.65	231.99	162.78	179.23	198.91	186.01	153.39	213.04	202.45	188.72	0.95	1	205.8	206.66	143.04	185.17	162.38	190.78	193.47	182.21	0.98	1	82.55	95.71	51.11	76.46	70.56	55.18	87.65	71.13	0.93	1	43.47	85.02	74.23	67.57	53.39	60.66	57.02	0.84	1
Solyc01g059940.2.1	Pectinesterase inhibitor (AHR	0	0	0	0	0	0	0	0	0	0	1	NA	0	0	0	0	0	0	0	0	1	NA	0	0	0	0	0	0	0	0	1	NA	0	0	0	0	0	0	0	1	NA
Solyc01g059950.1.1	GAI-like protein 1 (Fragment)	0.94	1.04	0.28	0	0.56	0.26	0.19	0.65	0	0.28	0.49	0.884698	0.66	1.43	2.38	1.49	0.98	2.31	3.51	2.27	1.52	0.974663	0	0	0.23	0.08	0.31	0.18	0.68	0.39	5.09	1	0	0	0	0	0	0	0	1	NA
Solyc01g059960.1.1	GAI-like protein 1 (Fragment)	0	0	0	0	0	0	0	0	0	0	1	NA	0	0	0	0	0	0	0	0	1	NA	0	0	0	0	0	0	0	0	1	NA	0	0	0	0	0	0	0	1	NA
Solyc01g059970.1.1	Mutator-like transposase (AH	0	0.15	0	0	0.04	0	0	0	0	0	0.27	1	0	0	0	0	0	0	0.14	0.05	4.67	1	0	0	0	0	0.11	0	0	0.04	3.67	1	0	0	0	0	0	0	0	1	NA
Solyc01g059980.2.1	Beta-glucanase (AHRD V1 ***	0.37	0.49	0.43	0	0.32	0.2	0.72	0.5	0	0.35	1.1	1	3.4	0.32	0.46	1.39	0.13	0.28	0	0.14	0.1	0.811508	9.15	64.03	1.63	24.94	0.36	0.28	0.27	0.3	0.01	0.927298	61.84	136.98	210.2	136.34	180.17	71.78	125.97	0.92	1
Solyc01g059990.2.1	Serine/threonine-protein pho	0	0	0	0	0	0	0	0	0	0	1	NA	0	0	0	0	0.08	0	0	0.03	2.67	1	0	0	0	0	0	0	0	0	1	NA	0	0.09	0	0.03	0	0	0	0.33	1
Solyc01g060000.1.1	Mutator-like transposase (AH	0	0	0	0	0	0	0	0	0	0	1	NA	0	0	0	0	0	0	0	0	1	NA	0	0	0	0	0	0	0	0	1	NA	0	0	0	0	0	0	0	1	NA
Solyc01g060010.1.1	Beta-glucanase (AHRD V1 ***	0	0	0	0	0	0	0	0	0	0	1	NA	0	0	0	0	0	0	0	0	1	NA	0	0	0	0	0	0	0	0	1	NA	0	0	0	0	0	0	0	1	NA
Solyc01g060020.2.1	Beta-glucanase (AHRD V1 ***	0.37	0.49	0.43	0	0.32	0.2	0.72	0.5	0	0.35	1.1	1	3.4	0.32	0.46	1.39	0	0.28	0	0.09	0.07	0.762944	9.15	64.03	1.63	24.94	0.36	0.28	0.27	0.3	0.01	0.927298	61.84	137.11	210.48	136.48	180.17	71.78	125.97	0.92	1
Solyc01g060030.2.1	Zinc finger CCHC domain-con	133.43	134.42	137.52	108.75	128.53	87.94	70.49	122	112.71	98.28	0.76	0.546606	114.42	129.79	143.91	129.37	177.18	174.9	159.43	170.5	1.32	0.337249	92.57	117.62	76.56	95.58	164.56	110.53	122.17	132.42	1.39	1	318.07	386.92	439.35	381.45	202.97	218.54	210.75	0.55	0.415136
Solyc01g060040.2.1	Bromodomain and WD repea	4.46	4.72	6.25	5.67	5.28	4.52	4.85	3.8	4.27	4.36	0.83	0.972152	4.57	4.66	6	5.08	3.63	4.75	4.06	4.15	0.82	0.987356	6	5.53	7.25	6.26	6.2	4.02	4.43	4.88	0.78	1	6.43	8.4	7.85	7.56	5.56	6.29	5.93	0.78	1
Solyc01g060050.1.1	WD-40 repeat protein-like (A	2.71	3.18	2.68	3.22	2.95	1.08	2.74	1.79	3.42	2.26	0.77	0.992609	2.22	2.73	3.48	2.81	2.1	2.41	4.09	2.87	1.02	1	3.24	2.65	1.94	2.61	2.7	2.79	3.08	2.86	1.09	1	3.7	2.99	2.83	3.17	4.96	3.23	4.09	1.29	1
Solyc01g060070.2.1	Pore protein homolog (AHRD	34.12	23.96	20.13	23.6	25.45	28.66	27.24	29.98	26.4	28.07	1.1	1	27.16	26.7	19.85	24.57	16.04	16.69	17.28	16.67	0.68	0.577419	14.7	13.59	9.41	12.57	10.46	8.33	9.95	9.58	0.76	1	1.08	1.65	1.17	1.3	2.78	1.66	2.22	1.71	1
Solyc01g060080.2.1	Serine/threonine-protein pho	8.94	12.31	8.66	7.06	9.24	14.12	9.81	15.14	10.13	12.3	1.33	0.575913	11.73	12.6	11.53	11.95	9.01	8.97	13.16	10.38	0.87	1	14.98	12.27	14.39	13.88	13.11	13.62	12.41	13.05	0.94	1	23.01	17.16	23.44	21.2	17.23	17.82	17.52	0.83	1
Solyc01g060090.1.1	Serine/threonine-protein pho	6.22	4.06	5.38	7.04	5.67	4.61	6.88	3.05	7.74	5.57	0.98	1	10.81	12.26	7.01	10.03	10.07	8.81	6.52	8.47	0.84	1	11.4	9.8	11.12	10.77	11.45	11.95	10.28	11.23	1.04	1	16.52	18.99	29.99	21.83	14.43	17.83	16.13	0.74	1
Solyc01g060100.2.1	Unknown Protein (AHRD V1)	6.18	5.5	4.86	11.66	7.05	4.58	8.14	5.69	5.92	6.08	0.86	1	3.84	8.96	8.71	7.17	2.86	10.67	4.86	6.13	0.85	1	14.99	13.44	4.09	10.84	14.9	7.81	15.02	12.58	1.16	1	5.86	7.49	25.36	12.9	16.98	14.46	15.72	1.22	1
Solyc01g060110.1.1	Mutator-like transposase (AH	0	0	0	0	0	0	0	0	0	0	1	NA	0	0	0	0	0	0	0	0	1	NA	0	0	0	0	0	0	0	0	1	NA	0	0	0	0	0	0	0	1	NA
Solyc01g060120.1.1	Ulp1 protease family C-termi	0	0	0	0	0	0	0	0	0	0	1	NA	0	0	0	0	0	0	0	0	1	NA	0	0	0	0	0	0	0	0	1	NA	0	0	0	0	0	0	0	1	NA
Solyc01g060130.2.1	ADP-ribosylation factor-like p	52.03	44.49	34.9	44.25	43.92	39.69	48.32	35.33	36.62	39.99	0.91	1	43.97	35.18	27	35.38	31.13	36.62	44.36	37.37	1.06	1	66.49	78.14	56.71	67.11	57.41	50.26	61.21	56.29	0.84	1	12.09	25.69	15.16	17.65	18.68	23.26	20.97	1.19	1
Solyc01g060140.2.1	Calmodulin-binding transcrip	71.6	71.9	102.42	59.09	76.25	91.12	57.38	112.14	100.82	90.36	1.19	0.781291	81.39	65.85	58.81	68.68	46.24	37.94	49.13	44.44	0.65	0.118773	31.12	33.32	27.84	30.76	29.58	26.65	27.71	27.98	0.91	1	54.2	50.64	58.32	54.39	37.61	47.62	42.61	0.78	1
Solyc01g060150.2.1	Unknown Protein (AHRD V1)	59.12	56.05	101.08	63.02	69.82	71.43	50.05	81.93	77.19	70.15	1	1	76.91	58.09	68.75	67.92	50.21	37.26	48.14	45.2	0.67	0.281481	29.07	35.95	22.96	29.33	25.05	22.73	24.04	23.94	0.82	1	51.77	40.45	32.94	41.72	25.49	41.32	33.41	0.8	1
Solyc01g060160.1.1	Unknown Protein (AHRD V1)	0	0	0	0	0	0	0	0	0	0	1	NA	0	0.17	0	0.06	0	0	0	0	0.18	1	0	0	0	0	0	0	0	0	1	NA	0	0	0	0	0	0	0	1	NA
Solyc01g060170.2.1	Calmodulin-binding transcrip	49.89	63.12	80.26	38.49	57.94	64.17	41.34	106.91	78.44	72.72	1.26	0.846866	74.02	55.56	52.24	60.61	28.03	32.24	51.52	37.26	0.61	0.273544	22.32	26.21	18.01	22.18	26.82	16.88	20.73	21.48	0.97	1	24.04	19.76	21.56	21.79	24.91	30.01	27.46	1.26	1
Solyc01g060180.2.1	AMP-dependent synthetase a	24.74	19.85	30.01	29.41	26	31.11	19.62	20.93	26.6	24.56	0.94	1	23.98	21.61	24.03	23.21	32.95	22.67	23.64	26.42	1.14	0.918247	4.22	4.92	2.54	3.89	5.04	3.35	3.72	4.04	1.04	1	2.48	4.3	3.39	3.39	8.72	9.27	9	2.65	0.212958
Solyc01g060190.1.1	Mutator-like transposase (AH	0.41	0.73	0.72	0.29	0.54	0	0	0	0	0	0.02	0.198448	0.38	0.71	0.52	0.54	0.14	0.32	1.12	0.53	0.98	1	0.33	0.17	0.2	0.23	0.27	0.62	0.6	0.5	2.13	1	0	0.15	0	0.05	0	0.36	0.18	3.6	1
Solyc01g060200.1.1	3&apos-5&apos exonuclease	1.27	1.13	2.4	0.36	1.29	1.7	2.41	1.87	1.65	1.91	1.48	0.917213	0.47	1.11	1.72	1.1	0.18	1.06	1.8	1.01	0.92	1	0.62	0.83	0.76	0.74	0.33	0.77	0.37	0.49	0.67	1	1.81	0.74	0.78	1.11	0.47	1.79	1.13	1.02	1
Solyc01g060210.2.1	X1 (Fragment) (AHRD V1 ***	3.82	5.25	4.51	5.4	4.75	4.25	4.93	7.66	4.24	5.27	1.11	1	4.84	4.23	4.11	4.39	2.65	5.93	3.55	4.04	0.92	1	3.37	3.96	1.72	3.02	1.82	0.53	4.55	2.3	0.76	1	0.99	1.51	2.14	1.55	2.22	0.61	1.42	0.91	1
Solyc01g060220.1.1	Unknown Protein (AHRD V1)	0	0	0	0	0	0	0	0	0	0	1	NA	0	0	0	0	0	0	0	0	1	NA	0	0	0	0	0	0.53	0	0.18	17.67	NA	0	0	0	0	0	0	0	1	NA
Solyc01g060230.1.1	Unknown Protein (AHRD V1)	0	0	0	0	0	0	0	0	0	0	1	NA	0	0	0	0	0	0	0	0	1	NA	0	0	0	0	0	0	0	0	1	NA	0	0	0	0	0	0	0	1	NA
Solyc01g060240.2.1	Unknown Protein (AHRD V1);	207.49	155.31	97.92	111.38	143.03	379.67	322.58	299.37	255.91	314.38	2.2	0.000541	95.02	61.05	59.32	71.8	39.85	61.15	76.93	59.31	0.83	1	58.46	61.56	39.08	53.03	55.22	64.52	37.22	52.32	0.99	1	18.15	20.87	31.09	23.37	11.69	11.2	11.45	0.49	0.938102
Solyc01g060250.2.1	Unknown Protein (AHRD V1)	28.96	30.79	22.78	18.21	25.19	20.57	23.5	28.85	30.03	25.74	1.02	1	31.43	24.48	24.46	26.79	22.32	32.91	32.88	29.37	1.1	1	18.85	14.42	4.79	12.69	16.38	12.81	17.59	15.59	1.23	1	22.88	19.87	33.4	25.38	19.88	28.92	24.4	0.96	1
Solyc01g060260.2.1	Unknown Protein (AHRD V1)	0	0	0	0	0	0	0	0	0	0	1	NA	0	0	0	0	0.22	0.17	0	0.13	13	1	0.26	0.26	0	0.17	0	0.24	0	0.08	0.46	1	0	0	0	0	0	0	0	1	NA
Solyc01g060270.1.1	Os06g0207500 protein (Frag	0	0	0	0	0	0	0	0	0	0	1	NA	0.13	0	0	0.04	0	0	0	0	0.23	1	0	0	0	0	0	0	0	0	1	NA	0	0	0	0	0	0	0	1	NA
Solyc01g060280.2.1	STAM binding protein (AHRD	8.82	15.82	15.99	13.2	13.46	14.64	14.23	11.76	12.85	13.37	0.99	1	14.27	15.34	14.58	14.73	12.77	13.17	17.28	14.41	0.98	1	15.6	16.77	13.83	15.4	15.54	11.92	12.95	13.47	0.87	1	16.36	21.45	21.63	19.81	14.28	16.03	15.16	0.76	1
Solyc01g060290.1.1	Unknown Protein (AHRD V1)	0	0	0	0	0	0	0	0	0	0	1	NA	0	0	0	0	0	0	0	0	1	NA	0	0	0	0	0	0	0	0	1	NA	0	0	0	0	0	0	0	1	NA
Solyc01g060300.1.1	MADS-box transcription facto	0	0	0	0	0	0	0	0	0	0	1	NA	0	0	0	0	0	0	0	0	1	NA	0	0	0	0	0	0	0	0	1	NA	0	0	0	0	0	0	0	1	NA
Solyc01g060310.1.1	MADS-box transcription facto	0	0	0	0	0	0	0	0	0	0	1	NA	0	0	0	0	0	0	0	0	1	NA	0	0	0	0	0	0	0	0	1	NA	0	0	0	0	0	0	0	1	NA
Solyc01g060320.1.1	Unknown Protein (AHRD V1)	0.97	0	0	0	0.24	0	0	0	0	0	0.04	1	0	0	0	0	0	0	0	0	1	NA	0	0	0	0	0	0	0	0	1	NA	0	0	0	0	0	0	0	1	NA
Solyc01g060330.1.1	Ulp1 protease family C-termi	0.45	0	0.18	0.21	0.21	0.17	0.48	0	0	0.16	0.77	1	0.14	0.13	0	0.09	0.21	0.31	0.24	0.25	2.81	1	0	0	0.15	0.05	0	0.11	0	0.04	0.73	1	0	0	0	0	0	0	0	1	NA
Solyc01g060340.1.1	Endo-1 4-beta-glucanase (AH	0	0	0	0	0	0	0	0	0	0	1	NA	0	0	0	0	0	0	0	0	1	NA	0	0	0	0	0	0	0	0	1	NA	0	0	0	0	0	0	0	1	NA
Solyc01g060350.1.1	Unknown Protein (AHRD V1)	0	0	0	0	0	0	0	0	0	0	1	NA	0	0	0	0	0	0	0	0	1	NA	0	0	0	0	0	0	0	0	1	NA	0	0	0	0	0	0	0	1	NA
Solyc01g060360.1.1	Unknown Protein (AHRD V1)	0	0	0	0	0	0	0	0	0	0	1	NA	0	0	0	0	0	0	0	0	1	NA	0	0	0	0	0	0	0	0	1	NA	0	0	0	0	0	0	0	1	NA
Solyc01g060370.1.1	B3 domain-containing protei	0	0	0	0	0	0	0	0	0	0	1	NA	0	0	0	0	0	0	0	0	1	NA	0	0	0	0	0	0	0	0	1	NA	0	0	0	0	0	0	0	1	NA
Solyc01g060380.1.1	cDNA clone J065202E18 full i	0.4	0	0.47	1.14	0.5	0.45	0.32	0.37	0.29	0.36	0.71	1	1.12	0	0.68	0.6	0	0.62	0.63	0.42	0.69	1	0	0.66	0	0.22	0.26	0.3	0.59	0.38	1.74	1	0.57	0.29	0	0.29	0.37	0.71	0.54	1.88	1
Solyc01g060390.1.1	Unknown Protein (AHRD V1)	0.52	1.85	1.23	0.73	1.08	1.15	0	1.91	1.12	1.04	0.97	1	1.45	3.16	0.44	1.68	1.8	1.08	1.22	1.37	0.81	1	2.52	0.85	0.52	1.3	1.71	1.18	0.76	1.22	0.94	1	1.48	1.51	1.6	1.53	1.43	1.37	1.4	0.92	1
Solyc01g060400.1.1	Heat shock protein (AHRD V1	0	0	0	0	0	0	0	0	0	0	1	NA	0	0	0	0	0	0	0	0	1	NA	0	0	0	0	0	0	0	0	1	NA	0	0	0	0	0	0	0	1	NA
Solyc01g060410.2.1	Unknown Protein (AHRD V1)	0.36	0.45	0.26	0.2	0.32	0	0.34	0.13	0.26	0.18	0.57	0.966035	0.2	0.19	0.12	0.17	0.25	0.22	0.11	0.19	1.14	1	0.06	0.06	0	0.04	0	0	0.16	0.05	1.33	1	0	0	0	0	0	0	0	1	NA
Solyc01g060420.2.1	Zinc finger protein 6 (AHRD V	0	0	0	0	0	0	0	0	0	0	1	NA	0	0	0	0	0	0	0	0	1	NA	0	0	0	0	0	0	0	0	1	NA	0	0	0	0	0	0	0	1	NA
Solyc01g060430.1.1	Unknown Protein (AHRD V1);	0	0	0	0	0	0	0	0	0	0	1	NA	0	0	0	0	0	0	0	0	1	NA	0	0	0	0	0	0	0	0	1	NA	0	0	0	0	0	0	0	1	NA
Solyc01g060440.1.1	Unknown Protein (AHRD V1)	0	0	0	0	0	0	0	0	0	0	1	NA	0	0	0	0	0	0	0	0	1	NA	0	0	0	0	0	0	0	0	1	NA	0	0	0	0	0	0	0	1	NA
Solyc01g060450.2.1	Unknown Protein (AHRD V1);	0.51	0.23	0.3	0.36	0.35	0	0.4	0.23	0.55	0.3	0.84	1	0.24	0	0.21	0.15	0.7	0	0	0.23	1.56	1	0	0.21	0	0.07	0	0	0	0	0.14	1	0	0	0	0	0	0	0	1	NA
Solyc01g060460.1.1	DNA repair protein (AHRD V1	0	0.15	0.1	0	0.06	0	0	0	0.06	0.01	0.24	1	0	0.23	0.51	0.25	0.12	0.09	0.14	0.12	0.47	0.990265	0.91	1.48	0.43	0.94	1.14	0.52	1.58	1.08	1.15	1	0	0	0	0	0.08	0	0.04	4	1
Solyc01g060470.2.1	Importin alpha-1b subunit (A	178.28	165.97	246.25	223.01	203.38	158.26	163.11	160.49	180.17	165.51	0.81	0.873795	189.76	187.49	231.07	202.77	192.24	181.34	189.61	187.73	0.93	1	157.79	170.49	221.48	183.25	179.39	181.46	172.38	177.74	0.97	1	249.6	196.17	180.73	208.83	254.07	264.16	259.12	1.24	1
Solyc01g060480.1.1	TNP2 (AHRD V1 **-- C7FDI5_	0	0	0	0	0	0	0	0	0	0	1	NA	0	0	0	0	0	0	0	0	1	NA	0	0	0	0	0	0	0	0	1	NA	0	0	0	0	0	0	0	1	NA
Solyc01g060490.2.1	GATA transcription factor 22	0.77	0.68	1.81	2.17	1.36	0.85	1.21	1.76	1.38	1.3	0.96	1	2.14	0.67	0.32	1.04	0.53	0.4	0.3	0.41	0.39	0.973882	0.31	0	0	0.1	0	0	0	0	0.1	1	0	0	0	0	0	0	0	1	NA
Solyc01g065490.2.1	Aldo/keto reductase (AHRD V	20	20.55	25.78	13.12	19.86	30.25	21.32	30.14	25.9	26.9	1.35	0.452454	21.67	19.71	15.32	18.9	20.3	19.43	21.06	20.26	1.07	1	16.99	16.46	11.25	14.9	12.32	12.97	13.82	13.04	0.87	1	9.97	10.78	16.8	12.52	11.51	13.56	12.54	1	1
Solyc01g065500.2.1	Phosphoribosylanthranilate t	7.64	6.62	8.23	12.12	8.65	2.36	3.62	3.85	4.63	3.62	0.42	0.020649	10.01	7.97	9.48	9.15	5.35	5.06	4.91	5.11	0.56	0.059684	0.86	0.7	0.86	0.81	0.96	0.6	0.53	0.7	0.86	1	0.57	0.53	0.51	0.54	0.97	0.93	0.95	1.77	1
Solyc01g065510.2.1	Unknown Protein (AHRD V1)	6.03	4.03	11.12	11.73	8.23	13.83	12.2	10.4	8.93	11.34	1.38	0.541435	2.45	2.95	7.32	4.24	1.83	1.17	2.37	1.79	0.42	0.595368	0	0.61	0	0.2	0.99	0.29	0.82	0.7	3.44	1	0	0.27	0.58	0.28	0	0	0	0.04	1
Solyc01g065520.1.1	Unknown Protein (AHRD V1)	0	0	0	0	0	0	0	0	0	0	1	NA	0	0	0	0	0	0	0	0	1	NA	0	0.17	0	0.06	0	0	0.16	0.05	0.94	1	0	0	0	0	0	0.19	0.1	9.5	1
Solyc01g065530.2.1	COBRA-like protein (AHRD V1	8.74	6.06	9.59	13.23	9.41	11.3	14.28	6.54	8.32	10.11	1.07	0.986994	9.08	9.37	18.37	12.27	9.8	9.89	8.47	9.39	0.76	0.970506	31.95	29.74	83.04	48.24	34.19	43.14	35.7	37.68	0.78	1	186.73	113.4	133.33	144.49	91.84	110.61	101.22	0.7	1
Solyc01g065540.2.1	Response regulator 8 (AHRD	6.81	9.27	10.1	8.4	8.64	6.38	7.99	6.62	6.64	6.91	0.8	0.866037	6.61	9.22	10.68	8.84	13.09	11.08	10.32	11.5	1.3	0.592296	4.4	7.45	3.96	5.27	6.84	4.72	6.74	6.1	1.16	1	2.91	6.43	5.84	5.06	6.06	8.36	7.21	1.42	1
Solyc01g065550.2.1	Protein DEHYDRATION-INDU	22.7	29.63	24.28	28.68	26.32	28.93	23.89	31.44	24.98	27.31	1.04	1	20.27	24.73	29.39	24.8	27.5	35.57	30.44	31.17	1.26	0.67364	21.03	21.21	23.43	21.89	18.29	20.64	14.78	17.9	0.82	1	8.61	29.23	22.8	20.21	14.96	19.37	17.16	0.85	1
Solyc01g065560.2.1	Os08g0100600 protein (Frag	9.26	5.99	8.93	8.09	8.07	5.61	6.78	5.73	7.97	6.52	0.81	0.991112	6.57	7.75	10.23	8.18	8.52	9.24	8.07	8.61	1.05	1	8.29	11.66	9.02	9.66	8.73	6.38	9.57	8.23	0.85	1	11.25	6.48	5.43	7.72	7.09	6.49	6.79	0.88	1
Solyc01g065570.2.1	Chaperone protein dnaJ 49 (A	0	0	0	0	0	0	0	0	0	0	1	NA	0	0	0	0	0	0	0	0	1	NA	0	0	0	0	0	0	0	0	1	NA	0	0	0	0	0	0	0	1	NA
Solyc01g065580.2.1	Pumilio domain-containing p	30.06	29.22	32.21	19.68	27.79	21.96	22.48	22.66	24.48	22.89	0.82	0.807096	32.81	23.92	23.51	26.75	26.08	23.71	26.88	25.56	0.96	1	16.82	17.57	14.24	16.21	17.31	12.64	16.41	15.45	0.95	1	16.31	16.51	18.13	16.98	16.43	21.33	18.88	1.11	1
Solyc01g065590.1.1	Lipoprotein (AHRD V1 **-- C7	0	0	0	0	0	0	0	0	0	0	1	NA	0	0	0	0	0	0	0	0	1	NA	0	0	0	0	0	0	0	0	1	NA	0	0	0	0	0	0	0	1	NA
Solyc01g065600.1.1	NAD(P)H-quinone oxidoreduc	0	0	0	0	0	0	0	0.28	0	0.07	7	1	0	0	0	0	0	0	0	0	1	NA	0	0	0	0	0	0	0	0	1	NA	0	0	0.47	0.16	0	0	0	0.06	1
Solyc01g065610.1.1	NAD(P)H-quinone oxidoreduc	0	0	0	0	0	0	0	0	0	0	1	NA	0	0	0	0	0	0	0	0	1	NA	0	0	0	0	0	0	0	0	1	NA	0	0	0	0	0	0	0	1	NA
Solyc01g065620.1.1	NAD(P)H-quinone oxidoreduc	0	0	0	0	0	0	0	0.5	0	0.12	12.5	1	0	0	0	0	0	0	0	0	1	NA	0	0	0	0	0	0	0	0	1	NA	0	0	0	0	0	0	0	1	NA
Solyc01g065630.1.1	NAD(P)H-quinone oxidoreduc	0	0	0	0	0	0	0	0	0	0	1	NA	0	0	0	0	0	0	0	0	1	NA	0	0	0	0	0	0	0	0	1	NA	0	0	0	0	0	0	0	1	NA
Solyc01g065640.1.1	NAD(P)H-quinone oxidoreduc	0	0	0	0	0	0	0	0	0	0	1	NA	0	0	0	0	0	0	0	0	1	NA	0	0	0	0	0	0	0	0	1	NA	0	0	0	0	0	0	0	1	NA
Solyc01g065650.1.1	WD-repeat protein-like (AHR	0	0	0	0	0	0	0	0	0	0	1	NA	0	0	0	0	0	0	0	0	1	NA	0	0	0	0	0	0	0	0	1	NA	0	0	0	0	0	0	0	1	NA
Solyc01g065660.1.1	Unknown Protein (AHRD V1)	0	0	0	0	0	0	0	0	0	0	1	NA	0	0	0	0	0	0	0	0	1	NA	0	0	0	0	0	0	0	0	1	NA	0	0	0	0	0	0	0	1	NA
Solyc01g065670.1.1	WD-repeat protein-like (AHR	0	0	0	0	0	0	0	0	0	0	1	NA	0	0	0	0	0	0	0	0	1	NA	0	0	0	0	0	0	0	0	1	NA	0	0	0	0	0	0	0	1	NA
Solyc01g065680.2.1	Unknown Protein (AHRD V1)	2.64	7.05	6.23	13.06	7.25	11.73	19.79	9.7	9.47	12.67	1.75	0.575913	2.45	9.18	8.92	6.85	4.58	14.35	14.52	11.15	1.63	0.881367	0	2.15	1.31	1.15	1.73	0	2.88	1.54	1.33	1	0	0.96	0	0.32	0	0	0	0.03	1
Solyc01g065690.2.1	Heavy-metal associated dom	8.47	7.39	11.34	4.84	8.01	7.24	5.92	2.55	6.78	5.62	0.7	0.72385	7.27	7.65	8.81	7.91	8.36	4.22	1.79	4.79	0.61	0.724976	6.32	4.78	3.88	4.99	3.75	3.46	2.73	3.31	0.66	1	11.81	6.03	6.26	8.03	4.32	6.43	5.38	0.67	1
Solyc01g065700.2.1	Integrin-linked kinase-associa	0	0	0	0	0	0.2	0	0	0	0.05	5	1	0.17	0	0	0.06	0	0	0	0	0.18	1	0.15	0	0	0.05	0	0	0	0	0.2	1	0	0.13	0	0.04	0	0.16	0.08	1.85	1
Solyc01g065710.2.1	Unknown Protein (AHRD V1)	16.35	21.08	10.64	11.96	15.01	20.68	14.69	19.7	10.93	16.5	1.1	1	19.41	17.16	15.72	17.43	16.43	12.26	19.05	15.91	0.91	1	11.39	7.81	6.72	8.64	4.07	5.13	8.63	5.94	0.69	1	0	1.64	1.73	1.12	2.06	1.48	1.77	1.58	1
Solyc01g065720.2.1	Phospholipase D (AHRD V1 **	10.79	14.43	11.95	11.97	12.29	10.4	14.21	11.67	10.57	11.71	0.95	1	13.47	14.09	15.05	14.2	13.85	13.76	14.8	14.14	1	1	23.64	20.4	22.54	22.19	19.73	19.96	20.71	20.13	0.91	1	32.2	27.31	30.15	29.89	25.64	26.23	25.94	0.87	1
Solyc01g065740.2.1	F-box family protein (AHRD V	0.65	2.43	0.77	0.18	1.01	2.31	0.92	2.75	0.56	1.64	1.62	1	1.45	1.47	1.32	1.41	1.44	1.75	2.86	2.02	1.43	0.898535	0.42	0.74	0.52	0.56	0.43	0.3	0.85	0.53	0.94	1	0.18	0.75	0.8	0.58	0.36	0.23	0.29	0.51	1
Solyc01g065750.2.1	Unknown Protein (AHRD V1)	11.52	13.98	13.59	29.62	17.18	31.43	27.28	32.74	33.83	31.32	1.82	0.140871	21.43	12.74	9.73	14.63	9.44	14.1	16.46	13.33	0.91	1	19.46	15.36	16.63	17.15	21.33	15.08	37.39	24.6	1.43	1	5.96	2.28	0	2.75	1.92	1.84	1.88	0.68	1
Solyc01g065760.1.1	NAD(P)H-quinone oxidoreduc	0	0	0	0	0	0	0	0	0	0	1	NA	0	0	0	0	0	0	0	0	1	NA	0	0	0	0	0	0	0	0	1	NA	1.56	0	0	0.52	0	0	0	0.02	1
Solyc01g065770.1.1	NADH-quinone oxidoreducta	0	0	0	0	0	0	0	0	0.37	0.09	9.25	1	0	0.45	0	0.15	0.72	0.27	0	0.33	2.2	1	0.42	0	0	0.14	0	0.39	0	0.13	0.93	1	1.48	0.38	0	0.62	0	0.46	0.23	0.37	1
Solyc01g065780.1.1	NADH-quinone oxidoreducta	0	0	0	0	0	1.11	0	0	0.72	0.46	45.75	1	0	0	0	0	0	0	0	0	1	NA	0	0	0	0	0	0	0	0	1	NA	0	0	0	0	0	0	0	1	NA
Solyc01g065790.1.1	Unknown Protein (AHRD V1)	0	0	0	0	0	0	0	0	0	0	1	NA	0	0	0	0	0	0	0	0	1	NA	0	0	0	0	0	0	0	0	1	NA	0	0	0	0	0	0	0	1	NA
Solyc01g065800.1.1	Unknown Protein (AHRD V1)	0	0	0	0	0	0	0	0	0	0	1	NA	0	0	0	0	0	0	0	0	1	NA	0	0	0	0	0	0	0	0	1	NA	0	0	0	0	0	0	0	1	NA
Solyc01g065810.1.1	NADH-quinone oxidoreducta	0	0	0	0	0	0.56	0	0	0	0.14	14	1	0	0	0.43	0.14	0	0	0	0	0.07	1	0	0	0	0	0.33	0	0.37	0.23	23.33	1	0	0	0.78	0.26	0	0	0	0.04	1
Solyc01g065820.1.1	Unknown Protein (AHRD V1);	0	0	0	0	0	0	0	0	0	0	1	NA	0	1.45	0.71	0.72	1.16	0.43	0	0.53	0.74	1	2.7	2.04	2.49	2.41	0.55	1.9	1.22	1.22	0.51	1	1.19	2.43	2.57	2.06	6.12	4.39	5.25	2.55	1
Solyc01g065830.1.1	Unknown Protein (AHRD V1)	0	0	0	0	0	0	0	0	0	0	1	NA	0	0	0	0	0	0	0	0	1	NA	0.31	0	0	0.1	0	0	0	0	0.1	1	0	0	0	0	0	0	0	1	NA
Solyc01g065840.2.1	F-box/LRR-repeat protein At3	10.13	13.53	8.42	4.4	9.12	13.05	9.63	17.47	8.6	12.19	1.34	0.876331	14.99	11.21	11.47	12.56	7.03	9.54	12.49	9.69	0.77	0.805882	6.6	6.85	5.71	6.39	4.46	5.15	6.96	5.52	0.86	1	8.51	11.21	11.33	10.35	6.32	7.77	7.04	0.68	0.984911

Solyc01g065850.1.1	Unknown Protein (AHRD V1)	0	0	0	0	0	0	0	0	0	0	1	NA	0	0	0	0	0	0	0	0	1	NA	0	0	0	0	0	0	0	0	1	NA	0	0	0	0	0	0	0	1	NA
Solyc01g065860.1.1	Helitron helicase-like protein	0	0	0	0	0	0	0	0	0	0	1	NA	0	0	0	0	0	0	0	0	1	NA	0	0	0	0	0	0	0	0	1	NA	0	0	0	0	0	0	0	1	NA
Solyc01g065870.1.1	Helitron helicase-like protein	0	0	0	0	0	0	0	0	0	0	1	NA	0	0	0	0	0	0	0	0	1	NA	0	0	0	0	0	0	0	0	1	NA	0	0	0	0	0	0	0	1	NA
Solyc01g065880.1.1	Helitron helicase-like protein	0	0	0	0	0	0	0	0	0	0	1	NA	0	0	0	0	0	0	0	0	1	NA	0	0	0	0	0	0	0	0	1	NA	0	0	0	0	0	0	0	1	NA
Solyc01g065890.1.1	Gt-2-related-like (AHRD V1 **	0	0	0	0	0	0	0	0	0	0	1	NA	0	0	0	0	0	0	0	0	1	NA	0	0	0	0	0	0.42	0	0.14	14	NA	0	0	0	0	0	0	0	1	NA
Solyc01g065900.1.1	Gt-2-related-like (AHRD V1 *-	0	0	0	0	0	0	0	0	0	0	1	NA	0	0	0	0	0	0	0	0	1	NA	0	0	0	0	0	0	0	0	1	NA	0	0	0	0	0	0	0	1	NA
Solyc01g065910.1.1	Periaxin-like protein (AHRD V	0	0	0	0	0	0	0	0	0	0	1	NA	0	0	0	0	0	0	0	0	1	NA	0	0	0	0	0	0	0	0	1	NA	0	0	0	0	0	0	0	1	NA
Solyc01g065920.1.1	Unknown Protein (AHRD V1)	0	0	0	0	0	0	0	0.7	0	0.17	17.5	1	0	0	0	0	0	0	0	0	1	NA	0	0	0	0	0	0	0	0	1	NA	1.08	0	0	0.36	0	0	0	0.03	1
Solyc01g065930.1.1	Unknown Protein (AHRD V1)	0	0	0	0	0	0.62	1.09	0.51	0.8	0.75	75.5	0.213143	0.52	0.48	0	0.33	0.96	0.86	0.44	0.75	2.26	0.955159	0.22	0.45	0.28	0.32	2.91	2.73	3.43	3.02	9.55	0.267943	0.39	1.81	1.7	1.3	2.79	3.4	3.09	2.38	1
Solyc01g065940.1.1	Unknown Protein (AHRD V1)	0	0	0	0	0	0	0	1.68	0	0.42	42	1	1.7	0	0	0.57	0	0.47	3.6	1.36	2.39	1	0	0	0	0	5.42	2.08	3.34	3.61	361.33	0.652224	0	0	1.41	0.47	1.68	9.64	5.66	12.04	0.707581
Solyc01g065950.1.1	Unknown Protein (AHRD V1)	0	0	0	0	0	0	0.72	0.84	0	0.39	39	1	0.85	0	0	0.28	1.27	0.47	0	0.58	2.05	1	0	0	0	0	2.41	1.39	2.67	2.16	215.67	1	0	0	0	0	0.84	6.43	3.63	363.5	0.76924
Solyc01g065960.1.1	Unknown Protein (AHRD V1);	1.32	0.59	2.33	0.93	1.29	0.73	0	3.03	1.89	1.41	1.09	1	1.84	2.29	1.67	1.93	1.37	0.34	0.52	0.74	0.38	0.911506	0	0	0.65	0.22	0.43	1	0.48	0.64	2.94	1	0.94	0.48	0	0.47	0	1.74	0.87	1.84	1
Solyc01g065970.1.1	Unknown Protein (AHRD V1)	0	0	0	0	0	0	0	0	0	0	1	NA	0	0	0	0	0	0	0	0	1	NA	0	0	0	0	0	0	0	0	1	NA	0	0	0	0	0	0	0	1	NA
Solyc01g065980.2.1	Ethylene responsive transcrip	427.13	478	826.24	294.95	506.58	275.5	169.52	254.76	302	250.44	0.49	0.070948	656.49	279.54	293.46	409.83	178	261.34	245.81	228.38	0.56	0.790845	1026.68	1272.47	1075.19	1124.78	1294.72	1231.8	1334.95	1287.16	1.14	1	731.25	1036.85	980.25	916.12	1012.67	1073.83	1043.25	1.14	1
Solyc01g065990.1.1	Unknown Protein (AHRD V1)	0	0	0	0	0	0	0	0	0	0	1	NA	0	0	0	0	0	0	0	0	1	NA	0	0	0	0	0	0	0	0	1	NA	0	0	0	0	0	0	0	1	NA
Solyc01g066000.1.1	Unknown Protein (AHRD V1);	0	0.8	0	0	0.2	0	0	0	0	0	0.05	1	0	0.39	0	0.13	0	0	0	0	0.08	1	0	0	0	0	0.3	0	0	0.1	10	1	0	0	0.69	0.23	0	0	0	0.04	1
Solyc01g066010.1.1	Zinc finger CCCH domain-cont	1.43	6.66	7.15	3.03	4.57	1.59	1.13	4.59	2.82	2.53	0.55	0.73004	2.99	2.17	4.82	3.33	4.2	3.51	4.48	4.06	1.22	1	3.75	1.74	1.77	2.42	4.92	3.51	3.9	4.11	1.7	1	8.11	3.11	6.03	5.75	5.22	3.44	4.33	0.75	1
Solyc01g066020.1.1	Tir-nbs-lrr, resistance protein	0.08	0.29	0.09	0.11	0.14	0.36	0.44	0.3	0.23	0.33	2.33	0.796773	0.07	0	0.2	0.09	0.11	0.08	0.06	0.08	0.93	1	0	0	0	0	0	0	0	0	1	NA	0	0	0	0	0	0	0	1	NA
Solyc01g066030.1.1	Mutator-like transposase (AH	0	0	0	0	0	0	0	0	0	0	1	NA	0	0.13	0	0.04	0	0	0.11	0.04	0.85	1	0	0	0	0	0	0	0	0	1	NA	0.21	0	0	0.07	0	0	0	0.14	1
Solyc01g066040.2.1	Unknown Protein (AHRD V1)	0	0.52	0	0	0.13	1.29	0	0	0	0.32	2.48	1	0	0	0	0	0	0	0	0	1	NA	1.4	2.83	0	1.41	2.66	2.19	2.11	2.32	1.65	1	0	0	0	0	0	2.03	1.01	101.5	1
Solyc01g066050.2.1	Peptidyl-prolyl cis-trans isom	42.25	40.79	46.79	63.08	48.23	32.68	42.13	41.01	44.26	40.02	0.83	0.982067	45.74	48.33	49.25	47.77	41.3	44.07	45.63	43.67	0.91	1	63.58	54.56	68.06	62.07	72.23	58.15	72.2	67.53	1.09	1	51.82	73.49	77.13	67.48	56.88	59.58	58.23	0.86	1
Solyc01g066060.2.1	DAG protein (AHRD V1 ***- E	19.79	18.97	19.46	19.38	19.4	21.44	25.44	21.46	23.32	22.91	1.18	0.773437	19.59	20.95	23.36	21.3	28.86	24.7	23.13	25.56	1.2	0.832635	22.34	30.81	24.93	26.03	30.67	26.73	23.3	26.9	1.03	1	25.97	26.91	24.58	25.82	39.49	41.82	40.66	1.57	0.823724
Solyc01g066070.2.1	Polygalacturonase (AHRD V1	0	0	0.38	0	0.1	0	0	0	0	0	0.11	1	0	0	0	0	0	0	0	0	1	NA	0	0	0	0	0	0	0	0	1	NA	0	0	0	0	0	0	0	1	NA
Solyc01g066080.1.1	O-glucosyltransferase 3 (AHR	0	0	0	0	0	0	0	0	0	0	1	NA	0	0	0	0	0	0	0.31	0.1	10.33	1	0	0	0	0	0	0	0	0	1	NA	0	0	0	0	0	0	0	1	NA
Solyc01g066090.1.1	Glucosyltransferase (AHRD V1	0	0	0	0	0	0	0	0	0	0	1	NA	0	0	0	0	0	0	0	0	1	NA	0	0	0	0	0	0	0	0	1	NA	0	0	0	0	0	0	0	1	NA
Solyc01g066100.1.1	UDP-glucosyltransferase fami	0	0	0	0	0	0	0	0	0	0	1	NA	0	0	0	0	0.1	0	0	0.03	3.33	1	0	0	0	0	0	0	0	0	1	NA	0	0	0	0	0	0	0	1	NA
Solyc01g066110.1.1	Glucosyltransferase (AHRD V1	0	0	0	0	0	0	0	0	0	0	1	NA	0	0	0	0	0	0	0	0	1	NA	0	0	0	0	0	0	0	0	1	NA	0	0	0	0	0	0	0	1	NA
Solyc01g066120.1.1	Glucosyltransferase (AHRD V1	0	0	0	0	0	0	0	0	0	0	1	NA	0	0	0	0	0	0	0	0	1	NA	0	0	0	0	0	0	0	0	1	NA	0	0	0	0	0	0	0	1	NA
Solyc01g066130.1.1	Unknown Protein (AHRD V1)	0	0	0	0	0	0	0	0	0	0	1	NA	0	0	0	0	0	0	0	0	1	NA	0	0	0	0	0	0	0	0	1	NA	0	0	0	0	0	0	0	1	NA
Solyc01g066140.1.1	UDP-glucosyltransferase fami	0	0	0	0	0	0	0	0	0	0	1	NA	0	0	0	0	0	0	0	0	1	NA	0	0	0	0	0	0	0	0	1	1	0	0	0	0	0	0	0	1	NA
Solyc01g066150.1.1	Glucosyltransferase (AHRD V1	0	0	0	0	0	0	0	0	0	0	1	NA	0	0	0	0	0	0.26	0	0.09	8.67	1	0	0	0	0	0.33	0	0	0.11	11	1	0	0	0	0	0	0	0	1	NA
Solyc01g066160.1.1	Unknown Protein (AHRD V1)	0	0	0	0	0	0.76	0	0	0	0.19	19	1	0	0	0	0	0	0	0	0	1	NA	0.56	1.68	0	0.75	1.81	1.56	2	1.79	2.4	1	0	0	0	0	0	0.6	0.3	30	1
Solyc01g066170.1.1	Ulp1 protease family C-termi	0	0	0	0	0	0.21	0.15	0.86	0.4	0.41	40.5	0.366325	0	0	0	0	0.65	0.58	0.73	0.65	65.33	0.156913	0.15	0	0	0.05	1.96	1.7	1.63	1.76	35.27	0.025819	0	0	0	0	2.05	2.13	2.09	209	0.03684
Solyc01g066180.1.1	Mutator-like transposase (AH	0	0	0	0	0	0.26	0	0.88	0	0.29	28.5	0.81921	0.22	0	0	0.07	0	0	0	0	0.14	1	0	0	0	0	0	0.18	0	0.06	6	1	0	0	0	0	0	0.21	0.1	10.5	1
Solyc01g066190.1.1	Unknown Protein (AHRD V1)	0	0.56	0.74	0	0.33	0.7	0	0	0.45	0.29	0.88	1	1.17	0	0.53	0.57	0.87	0.33	0	0.4	0.71	1	0	0	0	0	0	0	0	0	1	NA	0	0.46	0	0.15	0	0	0	0.07	1
Solyc01g066200.1.1	Unknown Protein (AHRD V1)	0.73	0.65	1.73	2.07	1.29	0	0	0	2.1	0.53	0.41	1	2.05	0.64	0.62	1.1	1.02	0.38	0	0.47	0.42	1	0	0.6	0.73	0.44	0	1.11	0	0.37	0.83	1	1.04	4.26	0	1.77	0	0	0	0.01	1
Solyc01g066210.1.1	Unknown Protein (AHRD V1)	0	0	0	0	0	0	0.45	0	0	0.11	11.25	1	0	0	0	0	0	0	0.45	0.15	15	1	0	0	0	0	0.75	0	0.41	0.39	38.67	1	0	0	0.87	0.29	0	0.5	0.25	0.86	1
Solyc01g066220.1.1	Unknown Protein (AHRD V1)	0	0	0	0	0	0	0	0	0	0	1	NA	0	0	0	0	0	0	0	0	1	NA	0	0	0	0	0	0	0	0	1	NA	0	0	0	0	0	0	0	1	NA
Solyc01g066230.1.1	Unknown Protein (AHRD V1)	0	0.33	0	0	0.08	0	0	0	0.8	0.2	2.42	1	0	0.64	0	0.21	0	0	0	0	0.05	1	0	0.6	0	0.2	0.49	0.56	2.16	1.07	5.35	1	0.53	0.27	0.57	0.46	1.02	0.65	0.83	1.83	1
Solyc01g066240.1.1	Unknown Protein (AHRD V1)	0	0	0	0	0	0	0	0	0	0	1	NA	0	0	0	0	0	0	0	0	1	NA	0	0	0	0	0	0	0	0	1	NA	0	0.68	0	0.23	1.73	0	0.86	3.82	1
Solyc01g066250.1.1	Unknown Protein (AHRD V1)	0	0	0	0	0	0	0	0	0	0	1	NA	0	0	0	0	0	0	0	0	1	NA	0	0	0	0	0	0	0	0	1	NA	0	0	0	0	0	0.66	0.33	33	1
Solyc01g066260.2.1	Unknown Protein (AHRD V1)	0.7	1.25	0	0	0.49	0	0	0	1.51	0.38	0.77	1	0.65	0.61	0	0.42	1.95	0.36	0	0.77	1.83	1	0	0	0	0	0	0	0.51	0.17	17	1	0	0	0	0	0	0	0	1	NA
Solyc01g066270.2.1	Unknown Protein (AHRD V1)	3.51	1.25	4.14	0	2.22	0	1.11	3.23	5.54	2.47	1.11	1	2.61	1.22	0	1.28	4.38	1.09	0	1.82	1.43	1	2.27	3.43	1.39	2.36	0	0.53	0.51	0.35	0.15	1	0	0	0	0	0	0	0	1	NA
Solyc01g066280.1.1	Unknown Protein (AHRD V1)	0	0	0	0	0	0	0	0	0	0	1	NA	0	0	0	0	0	0	0	0	1	NA	0	0	0	0	0	0	0	0	1	NA	0	0	0	0	0	0	0	1	NA
Solyc01g066290.2.1	Unknown Protein (AHRD V1)	6.32	11.24	18.22	2.98	9.69	7.8	3.32	11.62	12.6	8.83	0.91	1	11.75	1.83	1.78	5.12	10.22	2.54	0.55	4.44	0.87	1	5.1	6.86	13.24	8.4	0.92	0	1.53	0.82	0.1	0.994232	1	4.08	0	1.69	0	0.62	0.31	0.18	1
Solyc01g066300.1.1	Unknown Protein (AHRD V1)	0	0	0	0	0	0	0	0	0	0	1	NA	0	0	0	0	0	0	0	0	1	NA	0	0	0	0	0	0	0	0	1	NA	0	0	0	0	0	0	0	1	NA
Solyc01g066310.2.1	Acyl-CoA dehydrogenase (AH	38.91	40.65	46.94	36.7	40.8	33.44	31.04	40.97	32.51	34.49	0.85	0.889629	43.01	57.14	53.17	51.11	44.43	43.84	45.9	44.72	0.88	1	31.82	34.43	30.73	32.33	29.69	26.18	30.5	28.79	0.89	1	52.27	42.08	46.5	46.95	29.14	37.4	33.27	0.71	1
Solyc01g066320.1.1	Unknown Protein (AHRD V1)	0	0	0	0	0	0	0	0	0	0	1	NA	0	0	0	0	0	0	0	0	1	NA	0	0	0	0	0	0	0	0	1	NA	0	0	0	0	0	0	0	1	NA
Solyc01g066330.1.1	Unknown Protein (AHRD V1)	0	0	0	0	0	0	0	0	0	0	1	NA	0	0	0	0	0	0	0	0	1	NA	0	0	0	0	0	0	0	0	1	NA	0	0	0	0	0	0	0	1	NA
Solyc01g066340.2.1	Unknown Protein (AHRD V1)	0	0	0.89	0	0.22	0	0	0	0	0	0.04	1	0	0	0	0	0	0	0	0	1	NA	0	0	0	0	0	0	0	0	1	NA	1.08	0	0	0.36	0	0	0	0.03	1
Solyc01g066350.1.1	Unknown Protein (AHRD V1)	0	0	0	0	0	0	0	0	0	0	1	NA	0	0	0	0	0	0	0	0	1	NA	0	0	0	0	0	0	0	0	1	NA	0	0	0	0	0	0	0	1	NA
Solyc01g066360.2.1	Pectinesterase (AHRD V1 ***	0	0	0.28	0	0.07	0	0	0	0	0	0.14	1	0	0	0	0	0	0	0	0	1	NA	0	0	0	0	0	0	0	0	1	NA	0	0	0	0	0	0	0	1	NA
Solyc01g066370.2.1	Pectinesterase (AHRD V1 ***	0	0	5.44	0	1.36	0	0	0	0	0	0.01	0.83153	0	0	0	0	0	0	0	0	1	NA	0	0	0	0	0	0	0	0	1	NA	0	0	0	0	0	0	0	1	NA
Solyc01g066380.2.1	Unknown Protein (AHRD V1)	0	0	0	0	0	0	0	0	0	0	1	NA	0	0	0	0	0	0	0	0	1	NA	0	0	0	0	0	0	0	0	1	NA	0	0	0	0	0	0	0	1	NA
Solyc01g066390.2.1	Unknown Protein (AHRD V1)	0	0	6.92	0	1.73	0	0	0	0	0	0.01	0.859566	0	0	0	0	0	0	0	0	1	NA	0	0	0	0	0	0	0	0	1	NA	0	0	0	0	0	0	0	1	NA
Solyc01g066400.1.1	Pectinesterase (AHRD V1 ***	0	0	0	0	0	0	0	0	0	0	1	NA	0	0	0	0	0	0	0	0	1	NA	0	0	0	0	0	0	0	0	1	NA	0	0	0	0	0	0	0	1	NA
Solyc01g066410.1.1	Pectinesterase (AHRD V1 ***	0	0	0	0	0	0	0	0	0	0	1	NA	0	0	0	0	0	0	0	0	1	NA	0	0	0	0	0	0	0	0	1	NA	0	0	0	0	0	0	0	1	NA
Solyc01g066420.2.1	Pectinesterase (AHRD V1 ***	0	0	2.25	0	0.56	0	0	0	0	0	0.02	0.767873	0	0	0	0	0	0	0	0	1	NA	0	0	0	0	0	0	0	0	1	NA	0	0	0	0	0	0	0	1	NA
Solyc01g066430.2.1	Ring H2 finger protein (AHRD	8.77	3.9	5.95	23.25	10.47	2.68	5.19	2.42	1.26	2.89	0.28	0.557633	4.69	11.62	21.66	12.66	5.02	6.58	3.1	4.9	0.39	0.652011	4.96	8.93	24.15	12.68	10.94	8.47	3.35	7.59	0.6	1	135.87	88.35	100.1	108.11	49.16	54.58	51.87	0.48	0.305404
Solyc01g066440.2.1	Unknown Protein (AHRD V1)	38.16	34.91	27.77	43.88	36.18	51.13	78.55	58.36	35.07	55.78	1.54	0.281932	11.28	17.05	14.16	14.16	11.38	15.51	15.69	14.19	1	1	4.9	6.1	2.83	4.61	6.09	2.16	3.12	3.79	0.82	1	0	0	0	0	0	0.31	0.15	15.5	1
Solyc01g066450.2.1	Epoxide hydrolase (AHRD V1	0	0	0	0	0	0	0	0	0	0	1	NA	0	0	0	0	0.27	0.1	0	0.12	12.33	1	0	0	0	0	0	0	0.29	0.1	9.67	1	0	0	0	0	0	0	0	1	NA
Solyc01g066460.2.1	Epoxide hydrolase (AHRD V1	0	0.3	0.4	0	0.17	0	0.27	0	0.73	0.25	1.43	1	0	5.88	1.71	2.53	3.05	0.18	0.27	1.17	0.46	1	12.03	8.55	1.34	7.31	0.67	0.26	0.25	0.39	0.05	1	0	0.25	0	0.08	0	0	0	0.12	1
Solyc01g066470.2.1	Epoxide hydrolase (AHRD V1	0	0	0	0	0	0	0	0	0.24	0.06	6	1	0	0.29	0.29	0.19	0.23	0	0	0.08	0.4	1	1.37	0.28	0	0.55	0.22	0	0	0.07	0.13	1	0	0.25	0	0.08	0	0	0	0.12	1
Solyc01g066480.2.1	Fumarylacetoacetate hydrola	48.97	42.45	48.2	58.14	49.44	45.97	57.89	38.49	51.01	48.34	0.98	1	40.36	34.19	41.15	38.57	32.56	38.89	35.29	35.58	0.92	1	36.08	45.69	46.09	42.62	25	29.62	35.87	30.16	0.71	1	19.48	24.14	21.07	21.56	28.8	31.81	30.3	1.41	1
Solyc01g066490.2.1	Unknown Protein (AHRD V1)	4.26	11.36	7.53	9.03	8.04	33.12	10.08	31.31	19.86	23.59	2.93	0.20787	0	0	1.8	0.6	4.43	1.1	0	1.84	3.07	1	0	0	0	0	0	0	0	0	1	NA	0	0	0	0	0	0	0	1	NA
Solyc01g066500.1.1	MADS-box transcription facto	0.43	0	0	0.3	0.18	0	0	0	0.15	0.04	0.21	1	0.59	0.18	0.18	0.32	0	0	0	0	0.03	0.698378	0	0	0	0	0.14	0	0	0.05	4.67	1	0	0	0	0	0	0	0	1	NA
Solyc01g066510.1.1	Genomic DNA chromosome 5	0.29	0.58	0.63	0.58	0.52	1.31	0.93	0.76	1.27	1.07	2.05	0.229182	0.49	0.46	0.2	0.38	0.37	0.34	0.51	0.41	1.06	1	0	0.14	0.12	0.09	0	0	0.04	0.01	0.15	1	0.17	0	0	0.06	0.05	0	0.03	0.44	1
Solyc01g066520.2.1	Dihydrolipoyllysine-residue a	31.81	30.17	37.22	22.87	30.52	25.22	25.63	29.85	29.2	27.47	0.9	1	33.59	23.23	24.67	27.16	33.1	22.48	23.67	26.42	0.97	1	32.55	50.78	37.14	40.16	35.63	36.46	33.25	35.11	0.87	1	57.84	68.27	65.36	63.82	50.13	46.64	48.39	0.76	1
Solyc01g066530.1.1	Unknown Protein (AHRD V1)	0	0	0	0	0	0	0	0	0	0	1	NA	0	0	0	0	0	0	0	0	1	NA	0	0	0	0	0	0	0	0	1	NA	0	0	0	0	0	0	0	1	NA
Solyc01g066560.2.1	Multidrug resistance protein	0	0.12	0	0	0.03	0	0.11	0	0	0.03	0.92	1	0.12	0	0.11	0.08	0.09	0	0.1	0.06	0.83	1	0	0	0	0	0	0	0	0	1	NA	0	0	0	0	0	0	0	1	NA
Solyc01g066570.2.1	Genomic DNA chromosome 5	388.07	437.33	262.68	296.42	346.12	507.39	392.92	518.7	354.17	443.3	1.28	0.555292	273.89	388.36	299.5	320.58	285.64	294.61	399.89	326.71	1.02	1	103.46	239.93	67.89	137.09	63.73	54.05	117.71	78.5	0.57	1	123.45	81.03	112.64	105.71	95.03	101.22	98.12	0.93	1
Solyc01g066580.1.1	Unknown Protein (AHRD V1)	0	0	0	0	0	0	0	0	0.95	0.24	23.75	1	0	4.59	3.34	2.64	4.58	4.78	2.07	3.81	1.44	1	2.13	1.08	5.24	2.82	0	2	5.77	2.59	0.92	1	1.88	0	6.09	2.66	0	1.16	0.58	0.22	1
Solyc01g066590.2.1	Dynein light chain 2 cytoplasm	57.72	63.61	119.99	94.87	84.05	78.22	63.8	75.82	58.41	69.06	0.82	0.981195	64.43	75.99	103.55	81.32	87.32	75.43	53.14	71.96	0.88	1	29.76	38.08	27.83	31.89	35.75	25.42	23.24	28.14	0.88	1	81.29	54.7	61.71	65.9	49.32	73.27	61.3	0.93	1
Solyc01g066600.2.1	Unknown Protein (AHRD V1)	0	0	0	0	0	0	0	0	0	0	1	NA	0	0	0	0	0	0	0	0	1	NA	0	0	0	0	0	0	0	0	1	NA	0	0	0	0	0	0	0	1	NA
Solyc01g066610.2.1	Fanconi anemia group I prote	0.83	0.53	0.6	1.04	0.75	0.51	0.84	0.85	0.79	0.75	1	1	0.94	1.04	0.86	0.95	0.77	0.62	0.58	0.66	0.69	0.892119	0.15	0.45	0.14	0.25	0.12	0.03	0.2	0.12	0.47	1	0.39	0.17	0.28	0.28	0.72	0.36	0.54	1.93	1
Solyc01g066620.2.1	Fatty acid oxidation complex	1.11	1.81	1.64	0.91	1.37	1.44	1.31	2.97	1.13	1.71	1.25	0.98421	0.86	1.29	0.78	0.98	1.35	1.82	2.03	1.73	1.77	0.486767	0.37	0.75	0.46	0.53	0.42	0.56	0.61	0.53	1.01	1	0	0.2	0.14	0.11	0.17	0.16	0.17	1.46	1
Solyc01g066630.1.1	Unknown Protein (AHRD V1)	0	0	0	0	0	0	0	0	0	0	1	NA	0	0	0	0	0	0	0	0	1	NA	0	0	0	0	0	0	0	0	1	NA	0	0	0	0	0	0	0	1	NA
Solyc01g066640.2.1	Os04g0405500 protein (Fragr	19.41	24.46	21.48	35.72	25.27	12.99	20.48	17.22	13.07	15.94	0.63	0.394916	15.5	25.54	25.39	22.14	23.12	25.35	31.32	26.6	1.2	0.830983	2.22	2.52	0.68	1.81	2.6	0.13	1.75	1.49	0.83	1	0.49	0	0	0.16	0	0	0	0.06	1
Solyc01g066650.1.1	Protein FAR1-RELATED SEQU	0	0	0	0	0	0	0	0	0	0	1	NA	0	0	0	0	0	0	0	0	1	NA	0	0	0	0	0	0	0	0	1	NA	0	0	0	0	0	0	0	1	NA
Solyc01g066660.1.1	Protein FAR1-RELATED SEQU	0	0	0	0	0	0	0	0	0	0	1	NA	0	0	0	0	0	0	0	0	1	NA	0	0	0	0	0	0	0	0	1	NA	0	0	0	0	0	0	0	1	NA
Solyc01g066670.1.1	Transposon-like element Lyt2	0	0	0	0	0	0	0	0	0	0	1	NA	0	0	0	0	0	0	0	0	1	NA	0	0	0	0	0	0	0	0	1	NA	0	0	0	0	0	0	0	1	NA
Solyc01g066680.2.1	Phylloplanin (AHRD V1 *-*- Q	0	0	0	0	0	0	0	0	0	0	1	NA	0.78	0	0	0.26	0	0	0	0	0.04	1	0	0	0	0	0	0	0	0	1	NA	0	0	0	0	0	0	0	1	NA
Solyc01g066690.2.1	Translation initiation factor 2	40.58	37.98	44.48	42.4	41.36	35.21	41.34	30.33	32.89	34.94	0.84	0.945168	40.18	35.05	36.04	37.09	42.03	43.51	40.53	42.02	1.13	0.877888	34.58	31.25	32.61	32.81	36.8	35.72	33.77	35.43	1.08	1	35.73	49.15	41.87	42.25	57.39	58.42	57.91	1.37	1
Solyc01g066700.2.1	Bifunctional polymyxin resista	1.21	2.51	1.42	1.71	1.71	2.68	1.59	2.22	2.89	2.35	1.37	1	8.23	5.94	3.74	5.97	8.09	7.91	7.27	7.76	1.3	0.911351	4.55	4.92	2.79	4.09	1.32	1.22	1.17	1.24	0.3	1	1.14	1.75	1.24	1.38	0.37	0.71	0.54	0.39	1
Solyc01g066710.2.1	Unknown Protein (AHRD V1)	1.56	0.46	2.45	2.2	1.67	0.58	1.23	0.96	1.12	0.97	0.58	1	6.28	2.71	2.19	3.73	4.32	6.99	5.72	5.68	1.52	0.825568	4.62	4.66	4.12	4.47	1.02	0	2.27	1.1	0.25	1	0.74	0.75	0	0.5	0.48	0.46	0.47	0.95	1
Solyc01g066720.2.1	Hypoxia induced protein cons	435.54	349.89	460.64	475.15	430.31	232.72	324.72	243.24	318.49	279.79	0.65	0.214286	389.48	593.95	394.03	459.15	501.92	385.49	325.88	404.43	0.88	1	1420.03	1125.07	1097.16	1214.09	1485.89	2066.39	1148.38	1566.89	1.29	1	3035.02	1229.17	1734.95	1999.71	2574.61	2063.45	2319.03	1.16	1
Solyc01g066730.2.1	MADS-box protein (AHRD V1	0	0.59	0.78	0	0.34	1.95	0.87	1.21	0.94	1.24	3.63	0.408262	1.02	0.57	0.37	0.65	0.91	0.34	0.69	0.65	0.99	1	0.71	0	0.65	0.45	0.72	0.5	0.8	0.67	1.49	1	0.31	0.16	1.01	0.49	0	0.38	0.19	0.39	1
Solyc01g066740.2.1	Transducin family protein (Fr	36.48	42.41	44.98	38.75	40.65	30.9	54.96	34.96	45.07	41.47	1.02	1	39.22	44.25	45.69	43.05	26.79	31.86	38.9	32.52	0.76	0.544577	1.37	0.98	0.69	1.01	0.52	0.26	0.58	0.45	0.45	1	0.99	0.58	0.77	0.78	0.41	0.66	0.54	0.69	1
Solyc01g066750.1.1	Unknown Protein (AHRD V1)	0	0	0	0	0	0	0	0	0	0	1	NA	0	0	0	0	0	0	0	0	1	NA	0	0	0	0	0	0	0	0	1	NA	0	0	0	0	0	0	0	1	NA
Solyc01g066760.2.1	Unknown Protein (AHRD V1)	0	0	0	1.37	0.34	0	0	0	0.7	0.17	0.51	1	0	0	0	0	0	0	0	0	1	NA	0	0	0	0	0	0	0	0	1	NA	0	0	0	0	0	0	0	1	NA
Solyc01g066770.2.1	Chaperone protein dnaJ 49 (A	26.06	26.44	25.8	23.46	25.44	28.16	19.42	33.85	34.28	28.93	1.14	0.866856	25.94	26.61	26.32	26.29	35.52	38.4	34.8	36.24	1.38	0.294721	11.93	16.04	11.58	13.18	12.93	11.33	15.27	13.18	1	1	23.84	26.6	31.39	27.28	23.86	28.92	26.39	0.97	1
Solyc01g066790.1.1	Unknown Protein (AHRD V1)	0	0	0	0	0	1.13	0	0	0	0.28	28.25	1	0	0	0	0	0	0	0	0	1	NA	0	0	0	0	0	0	0	0	1	NA	0	0	0	0	0	0	0	1	NA
Solyc01g066800.1.1	Actin (AHRD V1 ***- ACT_ME	0	0	0	0	0	0	0	0	0	0	1	NA	0	0	0	0	0	0	0	0	1	NA	0	0	0	0	0	0	0	0	1	NA	0	0	0	0	0	0	0	1	NA
Solyc01g066810.2.1	Universal stress protein (AHR	0	0	0.49	0	0.12	0	0	0	0	0	0.08	1	0.39	0	0	0.13	0	0.21	0	0.07	0.54	1	0	0	0	0	0	0	0	0	1	NA	0	0	0	0	0	0	0	1	NA
Solyc01g066820.2.1	Kinesin like protein (AHRD V1	1.18	0.9	1.39	2.02	1.37	0.75	0.99	0.77	1.75	1.06	0.78	1	1.17	0.58	1.06	0.94	0.81	0.61	0.4	0.61	0.65	0.950871	0.34	1.16	0.42	0.64	0.28	0.32	0.55	0.38	0.6	1	0.12	0.06	0	0.06	0.08	0.22	0.15	2.5	1
Solyc01g066830.2.1	Sentrin-specific protease 2 (A	6.14	5.69	6.79	5.97	6.15	7.82	7.67	7.76	4.77	7	1.14	0.960119	7.02	7.78	6.05	6.95	8.6	8.74	8.74	8.69	1.25	0.775103	6.09	7.08	5.96	6.38	8.73	5.33	6.06	6.71	1.05	1	8	12.07	7.08	9.05	6.55	6.84	6.7	0.74	1
Solyc01g066840.2.1	40S ribosomal protein S21 (A	736.81	712.35	666.73	662.21	694.52	657.34	645.07	684.03	635.68	655.53	0.94	1	683.89	556.89	457.85	566.21	375.42	400.43	416.63	397.49	0.7	0.352658	599.13	486.41	463.9	516.48	425.55	510.86	451.78	462.73	0.9	1	186.47	219.95	256.67	221.03	371.07	392.35	381.71	1.73	0.498249
Solyc01g066850.2.1	Nodulin MtN21 family protei	0	0	0	0	0	0	0	0	0.12	0.03	3	1	0	0.29	0	0.1	0	0	0	0	0.1	1	0	0	0.17	0.06	0	0	0	0	0.18	NA	0	0	0	0	0	0	0	1	NA
Solyc01g066860.2.1	Unknown Protein (AHRD V1)	23.68	28.23	14.47	11.96	19.59	12.22	21.37	19.44	24.29	19.33	0.99	1	8.26	12.5	19.29	13.35	21.7	5.26	4.32	10.43	0.78	1	2.73	4.48	1.68	2.96	4.17	3.21	6.16	4.51	1.52	1	0.6	0.61	0	0.4	0	0.37	0.18	0.46	1
Solyc01g066870.2.1	Protein phosphatase 2C conta	11.2	13.11	8.32	8.22	10.21	15.45	14.41	12.78	11.92	13.64	1.34	0.638773	9.84	7.93	6.66	8.14	6.91	9.99	12.23	9.71	1.19	0.934769	5.53	6.25	2.68	4.82	4.91	3.62	5.59	4.71	0.98	1	6.78	3.92	3.19	4.63	3.99	7.28	5.63	1.22	1
Solyc01g066880.2.1	Copper chaperone (AHRD V1	4.4	6.52	6.42	1.48	4.71	2.79	1.98	2.5	3.16	2.61	0.55	0.425198	3.31	7.1	4.25	4.89	3.05	3.69	3.46	3.4	0.7	0.869608	5.08	5.8	4.99	5.29	6.33	5.08	3.36	4.92	0.93	1	11.32	6.39	5.8	7.84	4.03	2.57	3.3	0.42	0.741711
Solyc01g066890.1.1	Unknown Protein (AHRD V1)	0	0	0	0	0	0	0	0	0	0	1	NA	0	0	0	0	0	0	0	0	1	NA	0	0	0	0	0.15	0	0	0.05	5	1	0	0	0	0	0	0	0	1	NA
Solyc01g066900.2.1	Os12g0541400 protein (Fragr	21.25	24.83	19.33	20.67	21.52	25.1	24.82	31.75	20.34	25.5	1.19	0.905344	21.83	21.55	17.2	20.19	10.13	14.9	14.61	13.21	0.65	0.603713	7.15	9.02	4.83	7	5.53	9.06	9.36	7.98	1.14	1	3.78	9.97	6.13	6.63	5.67	5.43	5.55	0.84	1
Solyc01g066910.2.1	PVR3-like protein (AHRD V1 *	1.94	2.88	0	3.66	2.12	12.22	4.6	2.38	2.79	5.5	2.59	0.73004	3.01	0.56	2.19	1.92	1.35	3.35	5.08	3.26	1.7	0.965503	0	0	0.64	0.21	0	0	0	0	0.05	NA	0	0	0	0	0	0	0	1	NA
Solyc01g066930.1.1	Ulp1 protease family C-termi	0.07	0	0.08	0	0.04	0.08	0	0.06	0.05	0.05	1.27	1	0	0.06	0.17	0.08	0	0	0	0	0.13	0.838837	0	0	0.07	0.02	0.09	0	0	0.03	1.29	1	0	0.1	0.11	0.07	0	0	0	0.14	1
Solyc01g066940.2.1	Vesicle-associated membrane	20.8	27.24	19.28	33.21	25.13	13.21	18.3	21.31	19.2	18	0.72	0.639803	31.51	35.65	30.12	32.43	36.69	38.16	29.43	34.76	1.07	1	18.49	19.37	23.01	20.29	22.45	19.14	19.27	20.29	1	1	37.18	44.68	36.1	39.32	26.93	39.08	33	0.84	1

Solyc01g066950.1.1	Flowering promoting factor-li	0.62	0	0	0	0.15	0	0.49	0	0.44	0.23	1.5	1	0	0.54	1.04	0.53	0	0	0.48	0.16	0.3	1	0.5	1	0	0.5	0	0	0	0	0.02	1	0	0	0	0	0	0	0	1	NA
Solyc01g066960.1.1	Unknown Protein (AHRD V1)	0	0	0	0	0	0	0	0	0	0	1	NA	0	0	0	0	0	0	0	0	1	NA	0	0	0	0	0	0	0	0	1	NA	0	0	0	0	0	0	0	1	NA
Solyc01g066970.2.1	Flowering promoting factor-li	3.08	7.13	1.45	0	2.92	0.69	1.95	1.13	4.43	2.05	0.7	1	2.87	8.58	5.73	5.73	0.86	1.28	1.94	1.36	0.24	0.130059	2.49	4.02	1.22	2.58	0	0.47	0.45	0.31	0.12	0.578131	0.88	3.58	4.74	3.07	1.13	1.08	1.1	0.36	1
Solyc01g066980.2.1	Flowering promoting factor-li	3.73	5.54	0.73	0	2.5	1.38	2.46	0	2.23	1.52	0.61	0.965954	5.79	2.16	1.58	3.18	0.43	0.32	1.47	0.74	0.23	0.513361	3.52	8.62	0.62	4.25	1.64	2.83	0.45	1.64	0.39	1	1.77	5.42	5.74	4.31	1.71	4.37	3.04	0.71	1
Solyc01g066990.1.1	Unknown Protein (AHRD V1)	0.96	0	0	1.35	0.58	1.06	0	1.76	1.37	1.05	1.81	1	0	0.83	0	0.28	0	0.5	0	0.17	0.6	1	0	0	0.95	0.32	0.63	0	0	0.21	0.66	1	2.72	0	0	0.91	0	0	0	0.01	1
Solyc01g067000.2.1	SRC2 homolog (Fragment) (A	24.71	21.25	24.21	34.96	26.28	28.87	15.38	19.64	16.39	20.07	0.76	0.760084	23.77	49.15	52.89	41.94	57.23	41.42	36.7	45.12	1.08	1	12.86	26.11	22.45	20.47	30.54	20.32	26.25	25.7	1.26	1	81.29	32.7	36.07	50.02	33.17	40.21	36.69	0.73	1
Solyc01g067010.2.1	F-box family protein (AHRD V	3.55	6.94	4.6	2.01	4.27	7.1	4.2	13.37	8.53	8.3	1.94	0.427937	7.09	4.32	1.95	4.45	5.41	8.26	6.97	6.88	1.54	0.572708	13.61	8.38	7.39	9.79	8.39	9.68	12.41	10.16	1.04	1	18.91	23.57	26.45	22.98	6.33	10.11	8.22	0.36	0.071118
Solyc01g067020.2.1	Receptor like kinase, RLK	3.37	1.94	2.95	2.31	2.64	0.61	1.29	0.2	1.88	0.99	0.38	0.087012	2.23	4.45	3.77	3.48	5.44	3.04	2.14	3.54	1.02	1	1.23	2.4	4.65	2.76	3.86	2.23	1.43	2.51	0.91	1	4.49	13.76	14.73	10.99	5.58	5.54	5.56	0.51	0.865423
Solyc01g067030.2.1	F-box family protein (AHRD V	2.5	3.02	2.1	1.51	2.28	2.38	1.41	3.44	1.79	2.25	0.99	1	2.32	1.08	1.51	1.64	1.61	2.77	3.22	2.53	1.55	0.773559	2.74	1.89	0.53	1.72	2.34	1.35	1.56	1.75	1.02	1	2.28	2.33	3.56	2.72	1.31	1.88	1.59	0.59	1
Solyc01g067040.1.1	PWWP domain-containing pr	0.1	0.43	1.03	0.14	0.43	0.11	0	0.18	0.42	0.18	0.42	0.914991	0	0.25	0	0.08	0	0	0.08	0.03	0.32	1	0	0.08	0	0.03	0	0	0	0	0.38	1	0	0	0	0	0	0	0	1	NA
Solyc01g067050.1.1	Gag-Pol polyprotein (AHRD V	0	0	0	0	0	0	0	0	0	0	1	NA	0	0	0	0	0	0	0	0	1	NA	0.09	0	0	0.03	0.15	0	0	0.05	1.67	1	0	0.17	0.18	0.12	0	0.2	0.1	0.86	1
Solyc01g067060.1.1	HAT family dimerisation dom	0	0	0	0	0	0	0	0	0	0	1	NA	0	0	0	0	0	0	0	0	1	NA	0	0	0	0	0	0	0	0	1	NA	0	0	0	0	0	0	0	1	NA
Solyc01g067070.2.1	Mitochondrial deoxynucleoti	21.06	19.45	24.85	20.24	21.4	15.68	11.3	10.78	13.98	12.93	0.6	0.153262	13.68	21.07	21.15	18.63	26.19	23.77	20.93	23.63	1.27	0.657915	8.99	13.93	10.06	10.99	11.49	9.64	11.58	10.9	0.99	1	24.01	24.25	29.95	26.07	27.28	37.46	32.37	1.24	1
Solyc01g067080.2.1	Genomic DNA chromosome 3	25.24	32.68	29.78	23.53	27.81	29.33	36.68	29.54	30.48	31.51	1.13	0.959524	23.48	30.92	28.1	27.5	29.44	30.89	23.44	27.92	1.02	1	107.06	58.9	72.32	79.43	68.59	104.79	94.48	89.29	1.12	1	44.04	141.25	89.98	91.76	171.19	112.67	141.93	1.55	1
Solyc01g067090.2.1	Homeodomain-like (AHRD V1	2.89	3.74	4.44	4.21	3.82	2.24	1.8	2.17	2.26	2.12	0.55	0.229182	3.59	2.89	3.48	3.32	2.73	3.45	3.03	3.07	0.92	1	7.15	3.57	30.87	13.86	13.81	35.59	7.85	19.08	1.38	0.760824	63.78	66.75	75.45	68.66	72.58	28.81	50.7	0.74	1
Solyc01g067100.2.1	Cullin 1-like protein C (AHRD	60.64	72.86	80.77	68.2	70.62	53.89	56.43	62.78	62.09	58.8	0.83	0.878114	70.86	85.6	92.54	83	98.72	75.03	79.06	84.27	1.02	1	66.07	84.65	83.28	78	82.36	76.52	82.04	80.31	1.03	1	200.6	151.87	145.14	165.87	127.12	147.29	137.2	0.83	1
Solyc01g067110.1.1	Unknown Protein (AHRD V1);	0	0	0	0	0	0	0	0	0	0	1	NA	0	0	0	0	0	0	0	0	1	NA	0	0	0	0	0	0	0	0	1	NA	0	0	0	0	0	0	0	1	NA
Solyc01g067120.2.1	Cullin 1-like protein C (AHRD	1.95	2.78	0.46	1.66	1.71	0	0.31	3.23	1.12	1.17	0.68	1	2.18	4.07	1.32	2.52	4.6	2.43	4.6	3.88	1.54	0.934769	0.63	0.95	0.39	0.66	0.51	0	0.85	0.45	0.69	1	0	0	0	0	0.36	0	0.18	18	1
Solyc01g067130.2.1	Nuclear transcription factor Y	0	0	0	0	0	0	0	0.44	0	0.11	11	1	0	0.83	0	0.28	0.66	0	0	0.22	0.8	1	0	0.39	0	0.13	0.31	0	0	0.1	0.79	1	0	0	0.73	0.24	0.87	0.83	0.85	3.49	1
Solyc01g067140.1.1	Cullin 1-like protein C (AHRD	1.47	2.03	1.35	0.23	1.27	1.09	2.19	1.5	2.92	1.93	1.52	0.795461	1.82	2.41	1.24	1.82	1.58	0.84	3.46	1.96	1.07	1	0.79	1.86	0.65	1.1	1.28	1.11	1.19	1.19	1.08	1	6.72	1.3	1.75	3.26	1.64	1.14	1.39	0.43	1
Solyc01g067150.1.1	Unknown Protein (AHRD V1)	0	0	0	0	0	0	0	0	0	0	1	NA	0	0	0	0	0	0	0	0	1	NA	0	0.31	0	0.1	0	0	0	0	0.1	1	0	0	0	0	0	0	0	1	NA
Solyc01g067160.2.1	Unknown Protein (AHRD V1)	3.74	3.03	4.81	1.44	3.25	3.4	1.07	2.19	1.71	2.09	0.64	0.786173	0.95	2.36	3.16	2.16	2.36	1.58	0.8	1.58	0.73	1	0.27	1.39	0.68	0.78	0.89	0.77	0.5	0.72	0.92	1	0.48	0.74	1.05	0.76	2.8	0.6	1.7	2.25	1
Solyc01g067170.1.1	Cullin 1-like protein C (AHRD	0.49	0.87	5.77	2.07	2.3	1.63	2.7	2.25	3.16	2.44	1.06	1	1.82	1.7	5.37	2.96	4.07	2.02	2.69	2.93	0.99	1	1.97	4.78	5.82	4.19	3.85	3.7	4.63	4.06	0.97	1	6.95	0.35	2.25	3.18	8.95	11.14	10.04	3.16	0.277633
Solyc01g067180.2.1	Cullin 1-like protein C (AHRD	7.33	11.54	9.31	7.98	9.04	8.15	14.24	14	8.5	11.22	1.24	0.929942	6.29	11.76	6.19	8.08	8.21	10.51	10.19	9.64	1.19	1	2.28	4.14	2.8	3.07	2.22	5.13	5.75	4.37	1.42	1	2.41	4.1	4.34	3.62	5.68	1.48	3.58	0.99	1
Solyc01g067190.1.1	Unknown Protein (AHRD V1)	0	0	0	0	0	0	0	0	0	0	1	NA	0	0	0	0	0	0	0	0	1	NA	0	0	0	0	0	0	0	0	1	NA	0	0	0	0	0	0	0	1	NA
Solyc01g067200.1.1	Cullin 1-like protein C (AHRD	0	0	0.4	0	0.1	0.76	0	0.63	0	0.35	3.48	1	0.64	0.3	0.29	0.41	0	0.18	0	0.06	0.15	1	0	0	0	0	0.22	0.26	0.75	0.41	41	1	0.49	0.25	0	0.25	0	0.3	0.15	0.61	1
Solyc01g067210.2.1	Cullin 1-like protein C (AHRD	4.28	4.84	7.24	7.37	5.93	5.79	5.67	5.21	4.49	5.29	0.89	1	4.2	6.74	7.72	6.22	6.74	4.61	5.55	5.63	0.91	1	3.46	7.73	5.97	5.72	6.46	5.09	4.05	5.2	0.91	1	18.27	13.37	14.77	15.47	9.54	9.74	9.64	0.62	0.889823
Solyc01g067220.1.1	Unknown Protein (AHRD V1)	3.61	4.02	5.33	1.28	3.56	5.02	2.85	3.32	2.6	3.45	0.97	1	7.57	1.57	3.05	4.06	1.88	1.87	4.97	2.91	0.72	1	2.19	1.47	0.9	1.52	1.78	0	1.32	1.03	0.68	1	3.85	0.66	2.78	2.43	1.65	1.58	1.61	0.66	1
Solyc01g067230.2.1	Cullin 1-like protein C (AHRD	3.88	6.22	6.41	7.68	6.05	6.04	6.13	9.28	7.24	7.17	1.19	1	4.33	8.1	10.49	7.64	3.23	4.42	3.05	3.57	0.47	0.645111	5.64	2.53	9.24	5.8	5.1	3.53	3.96	4.2	0.72	1	23.17	4.51	9.55	12.41	7.82	9.53	8.68	0.7	1
Solyc01g067240.2.1	Cullin-like protein1 (AHRD V1	7.24	8.59	7.59	5.69	7.28	4.47	12.07	14.79	5.78	9.28	1.27	0.9771	5.99	8.39	8.15	7.51	4.46	7.08	6.32	5.95	0.79	1	6.5	3.93	3.99	4.81	4.23	1.22	2.35	2.6	0.54	1	2.29	3.51	8.66	4.82	4.42	3.53	3.98	0.82	1
Solyc01g067250.2.1	Cullin 1-like protein C (AHRD	6.18	5.5	3.65	5.83	5.29	4.58	10.58	9.48	5.18	7.46	1.41	0.942525	2.88	4.48	8.71	5.36	2.86	4.8	2.43	3.36	0.63	1	5	2.52	5.12	4.21	2.03	0.78	0.75	1.19	0.28	1	2.93	2.99	3.17	3.03	2.83	0.9	1.86	0.62	1
Solyc01g067260.2.1	Cullin 1-like protein C (AHRD	5.03	6.43	3.71	2.22	4.35	4.54	9.42	7.51	5.41	6.72	1.55	0.625191	5.26	5.73	3.98	4.99	4.58	3.09	6.67	4.78	0.96	1	5.58	6.4	4.37	5.45	4.54	4.29	3.2	4.01	0.74	1	4.91	6.16	6.76	5.94	4.6	4.68	4.64	0.78	1
Solyc01g067270.2.1	Cullin 1-like protein C (AHRD	5.45	9.19	6.09	4.46	6.3	8.29	10.87	11.6	7.83	9.65	1.53	0.498548	8	7.98	8.24	8.07	6.96	6.24	7.89	7.03	0.87	1	6.26	7.01	7.97	7.08	4.71	5.43	6.06	5.4	0.76	1	6.93	7.08	11.47	8.49	6.04	7.04	6.54	0.77	1
Solyc01g067280.2.1	F-box domain containing prot	26.6	29.59	24.48	16.56	24.31	21.88	17.22	29.35	24.83	23.32	0.96	1	27.96	27.52	22.92	26.13	25.46	29.2	20.91	25.19	0.96	1	24.72	30.35	11.09	22.05	15.03	12.1	20.35	15.83	0.72	1	18.91	13.72	12.68	15.1	16.07	17.96	17.02	1.13	1
Solyc01g067290.2.1	NAD-dependent epimerase/d	22.25	33.47	14.05	13.74	20.88	65.67	51.59	60.08	47.7	56.26	2.69	1.61E-06	20.7	15.26	13.37	16.44	23.37	29.53	33.37	28.76	1.75	0.017705	7.34	13.15	4.51	8.33	7.51	6.89	11.99	8.8	1.06	1	0.22	1.14	0	0.45	4.16	3.71	3.94	8.68	0.07458
Solyc01g067300.2.1	UPF0497 membrane protein	1.34	1.79	7.51	5.21	3.96	0.37	0	0.62	0.48	0.37	0.09	0.111144	10.59	21.25	15.84	15.89	8.13	2.77	1.32	4.07	0.26	0.021487	4.6	17.19	9.31	10.37	6.82	3.05	2.2	4.02	0.39	1	3.81	4.86	8.24	5.64	3.07	9.1	6.08	1.08	1
Solyc01g067310.1.1	Unknown Protein (AHRD V1)	0	0	0	0	0	0	0	0	0	0	1	NA	0	0	0	0	0	0	0	0	1	NA	0	0	0	0	0	0	0	0	1	NA	0	0	0	0	0	0	0	1	NA
Solyc01g067320.1.1	Receptor protein kinase-like (	0	0	0	0	0	0	0	0	0	0	1	NA	0	0	0	0	0	0	0	0	1	NA	0	0	0	0	0	0	0	0	1	NA	0	0	0	0	0	0	0	1	NA
Solyc01g067330.2.1	UPF0497 membrane protein	1.38	4	3.26	2.93	2.89	3.46	1.64	2.22	2.73	2.51	0.87	1	0.96	0.9	1.46	1.11	1.44	0	0.27	0.57	0.52	0.979744	0.84	2.25	0.34	1.14	0.23	1.05	0.25	0.51	0.45	1	0	0	0	0	0	0	0	1	NA
Solyc01g067340.1.1	Unknown Protein (AHRD V1);	0	0	0.36	0	0.09	0	0	0	0	0	0.11	1	0	0	0	0	0	0.16	0	0.05	5.33	1	0	0	0	0	0	0	0	0	1	NA	0	0	0	0	0	0	0	1	NA
Solyc01g067350.2.1	UDP-glucosyltransferase (AH	0	0	0	0	0	0	0	0	0	0	1	NA	0	0	0	0	0	0	0	0	1	NA	0	0	0	0	0	0	0	0	1	NA	0	0	0	0	0	0	0	1	NA
Solyc01g067360.2.1	CHY zinc finger family protein	0	0	0	0	0	0.27	0.58	0.45	0.35	0.41	41.25	0.521092	0.23	0.64	0.21	0.36	0.68	0.38	0.97	0.68	1.88	1	0	0.4	0.24	0.21	0	0.37	0.18	0.18	0.86	1	0.35	0.18	0.76	0.43	0	0	0	0.02	1
Solyc01g067370.2.1	Pollen-specific lysine-rich pro	0.28	0.25	0.66	0	0.3	0	0	0.26	0.2	0.12	0.39	1	0.52	0	0	0.17	0	0	0	0	0.06	1	0	0	0	0	0	0	0	0	1	NA	0	0	0	0	0	0	0	1	NA
Solyc01g067380.2.1	Lipase (AHRD V1 ***- B6V3M	21.01	31.1	29.98	24.88	26.74	27.43	18.71	19.99	26.14	23.07	0.86	0.975918	27.73	40.57	42.31	36.87	64.06	64.39	62.21	63.55	1.72	0.017086	40.86	34.84	29.59	35.1	24.56	28.33	34.01	28.97	0.83	1	60.1	37.27	36.82	44.73	32.21	30.85	31.53	0.7	1
Solyc01g067390.2.1	Chromodomain-helicase-DNA	50.56	48.47	59.43	46.92	51.34	31.92	30.39	41.25	43.5	36.77	0.72	0.383737	51.18	58.58	62.99	57.58	55.24	52.55	49.36	52.38	0.91	1	40.65	46.34	39.01	42	51.75	41.23	40.09	44.36	1.06	1	108.84	86.53	101.73	99.03	67.08	82.97	75.03	0.76	1
Solyc01g067400.2.1	Serine/threonine-protein kina	27.45	30.16	33.75	44.65	34	19.91	28.58	19.67	28.21	24.09	0.71	0.570216	30.8	26.1	32.21	29.7	24.75	28.89	25.21	26.28	0.88	1	24.57	28.14	23.4	25.37	26.49	24.9	27.42	26.27	1.04	1	28.95	22.65	19.81	23.8	23.47	27.45	25.46	1.07	1
Solyc01g067410.1.1	Pectinesterase (AHRD V1 ***	0	0	0.32	0	0.08	0	0.64	0	0	0.16	2	1	0	0	0	0	0	0	0	0	1	NA	0	0	0	0	0	0	0	0	1	NA	0	0	0	0	0	0	0	1	NA
Solyc01g067420.2.1	Pectinesterase (AHRD V1 ***	0	0	0	0	0	0	0.38	0	0	0.1	9.5	1	0	0	0	0	0	0	0	0	1	NA	0	0	0	0	0	0	0	0	1	NA	0	0	0	0	0	0	0	1	NA
Solyc01g067430.1.1	Unknown Protein (AHRD V1)	0	0	0	0	0	0	0	0	0	0	1	NA	0	0	0	0	0	0	0	0	1	NA	0	0	0	0	0	0	0	0	1	NA	0	0	0	0	0	0	0	1	NA
Solyc01g067440.2.1	Glutaredoxin (AHRD V1 ***-	7.05	22.23	3.02	0	8.08	5.7	6.57	8.83	14.25	8.84	1.09	1	4.77	0.56	0	1.78	0	0	0	0	0.01	0.80519	18.63	1.04	7.63	9.1	1.26	1.46	0	0.91	0.1	1	0.91	0.93	1.97	1.27	0	0	0	0.01	1
Solyc01g067450.1.1	Mutator-like transposase-like	0	0	0	0.18	0.04	0.14	0.1	0	0.09	0.08	1.83	1	0	0	0	0	0	0	0	0	1	NA	0	0	0	0	0	0	0	0	1	NA	0	0	0	0	0	0	0	1	NA
Solyc01g067460.1.1	Glutaredoxin family protein (	16.65	32.49	6.8	2.72	14.66	23.49	3.03	15.31	14.25	14.02	0.96	1	6.55	1.11	0	2.55	0	0	0.5	0.17	0.07	0.812162	1.03	0	0	0.34	0	0	0	0	0.03	1	0	0.47	0	0.16	0	0	0	0.06	1
Solyc01g067480.2.1	Kelch-domain-containing prot	12.23	10.71	10.53	14.32	11.95	13.6	15.75	13.18	12.6	13.78	1.15	0.773437	10.54	10.46	13.04	11.35	12.52	13.24	11.11	12.29	1.08	1	0.97	3.84	0.9	1.9	2.77	2.28	3	2.68	1.41	1	0.14	0.36	1.23	0.58	1.19	0.88	1.03	1.79	1
Solyc01g067490.2.1	Anaphase-promoting comple	17.14	16.51	14.68	11.29	14.91	15.92	13.44	14.68	17.78	15.46	1.04	1	14.74	10.31	12.59	12.55	14.17	12.76	15.22	14.05	1.12	0.98209	9.48	11.96	7.81	9.75	7.56	8.99	10.69	9.08	0.93	1	10.18	8.27	10.11	9.52	9.35	13.48	11.41	1.2	1
Solyc01g067500.2.1	Serine/threonine-protein pho	38.43	47.8	38.67	50.26	43.79	42.56	41.43	38.62	37.97	40.15	0.92	1	47.02	50.88	51.77	49.89	50.91	39.79	49.03	46.58	0.93	1	36.5	50.95	34.86	40.77	39.45	35.07	38.14	37.55	0.92	1	44.84	30.41	26.47	33.91	30.64	30.43	30.54	0.9	1
Solyc01g067510.2.1	Receptor-like kinase (AHRD V	13.09	9.6	8.14	16.01	11.71	14.16	24.99	16.26	12.95	17.09	1.46	0.401706	6.91	0.46	1.79	3.05	0.12	1.01	2.08	1.07	0.35	0.953787	0	0.14	0	0.05	0.12	0	0	0.04	0.86	1	0	0.26	0.27	0.18	0	0	0	0.06	1
Solyc01g067520.2.1	Cellulose synthase D-like prot	0	0	0	0	0	0	0	0	0	0	1	NA	0	0	0	0	0	0	0	0	1	NA	0	0	0	0	0	0	0	0	1	NA	0	0	0	0	0	0	0	1	NA
Solyc01g067530.2.1	GTPase obg (AHRD V1 ***- D	19.73	17.04	16.45	14.58	16.95	26.19	24.1	20.85	30.38	25.38	1.5	0.108885	17.72	22.96	20.81	20.5	44.33	39.39	31.09	38.27	1.87	0.00203	23.87	15.76	45.8	28.48	25.6	30.28	31.55	29.14	1.02	1	30.01	39.5	37.74	35.75	37.92	36.07	37	1.03	1
Solyc01g067540.1.1	Ethylene responsive transcrip	0	0.18	0	0	0.04	0	0	0	0	0	0.22	1	0	0	0	0	0	0	0	0	1	NA	0	0	0	0	0	0	0	0	1	NA	0	0	0	0	0	0	0	1	NA
Solyc01g067550.2.1	Zinc finger CCCH domain-cont	36.24	36.62	48.7	56.33	44.47	36.15	49.57	29.17	38.6	38.37	0.86	1	36.79	32.89	39.8	36.49	34.25	34.43	28.69	32.46	0.89	1	43.22	48.46	48.09	46.59	38.89	36.72	41.79	39.13	0.84	1	16.99	16.1	17.96	17.02	22.36	21.18	21.77	1.28	1
Solyc01g067560.2.1	Always early protein 3 (AHRD	9.87	10.38	13.19	10.8	11.06	10.85	8.87	10.13	11.86	10.43	0.94	1	9.84	10.24	11.47	10.52	10.55	10.64	9	10.06	0.96	1	3.74	5.95	7.25	5.65	5.95	5.45	5.96	5.79	1.02	1	16.12	11.92	13.41	13.82	9.68	9.18	9.43	0.68	0.987573
Solyc01g067570.2.1	Unknown Protein (AHRD V1)	21.69	26.18	34.36	19.34	25.39	34.35	26.98	30.05	25.17	29.14	1.15	0.919524	36.76	39.26	38.15	38.06	34.01	35.85	36.9	35.59	0.94	1	16.81	22.04	17.99	18.95	21.28	18.92	19.06	19.75	1.04	1	37.18	39.07	27.42	34.56	19.59	27.36	23.48	0.68	0.929819
Solyc01g067580.2.1	Unknown Protein (AHRD V1)	15.42	19.83	21.73	22.42	19.85	18.57	17.59	28.75	17.84	20.69	1.04	1	15.54	14.15	13.75	14.48	10.7	13.09	14.14	12.64	0.87	1	12.11	10.47	10.21	10.93	8.72	7.14	10.93	8.93	0.82	1	15.23	19.29	19.1	17.87	15.29	13.52	14.4	0.81	1
Solyc01g067590.2.1	5&apos-AMP-activated prote	24.18	21.15	22.88	28.59	24.2	19.89	24.37	21.6	27.36	23.31	0.96	1	25.79	23.15	23.3	24.08	22.26	23.48	25.44	23.73	0.99	1	21.41	21.59	24.4	22.47	23.33	20.6	20.6	21.51	0.96	1	21.94	15.28	14.28	17.17	18.25	20.36	19.3	1.12	1
Solyc01g067600.2.1	CW-type Zinc Finger family pr	8.35	8.68	13.91	10.4	10.34	4.4	6.85	7.16	9.12	6.88	0.67	0.501337	10.04	12.12	14.78	12.31	12.61	12.5	12.18	12.43	1.01	1	9.56	9.18	9.56	9.43	12.1	9.48	10.51	10.7	1.13	1	19.49	17.56	19.97	19.01	15.07	16.85	15.96	0.84	1
Solyc01g067610.2.1	Unknown Protein (AHRD V1)	12.69	24.84	35.92	37.09	27.64	26.32	18.7	28	17.61	22.66	0.82	1	28.32	33.82	26.43	29.52	29.33	35.03	28.58	30.98	1.05	1	27.33	15.85	39.46	27.55	20.56	28.21	20.96	23.24	0.84	1	50.51	46.07	75.43	57.34	37.16	44.49	40.83	0.71	1
Solyc01g067620.2.1	1-aminocyclopropane-1-carb	0.36	1.29	1.28	0.51	0.86	0.81	0.57	1.17	1.95	1.12	1.31	1	3.37	3.78	4.75	3.97	4.78	4.41	3.99	4.39	1.11	1	0.73	1.18	0.36	0.76	1.31	1.79	1.72	1.61	2.12	1	3.87	1.18	3.34	2.8	1.16	1.27	1.21	0.43	1
Solyc01g067630.2.1	MRNA complete cds clone RA	32.32	30.84	27.12	31.58	30.46	35.18	54.19	29.1	49.04	41.88	1.37	0.281526	28.82	28.38	26.45	27.88	47.14	42.09	43.73	44.32	1.59	0.103573	8.34	16.29	6.95	10.53	9.41	10.35	17.48	12.41	1.18	1	1.89	2.42	1.54	1.95	9.46	9.64	9.55	4.9	0.114627
Solyc01g067640.2.1	Cell division protein kinase 12	16.69	17.08	15.67	17.67	16.78	14.26	19.63	14.72	18.67	16.82	1	1	12.57	11.65	11.32	11.85	10.95	12.35	12.67	11.99	1.01	1	6.24	7.86	4.73	6.28	6.71	7.23	8.44	7.46	1.19	1	1.45	2.72	2.62	2.26	2.6	2.19	2.4	1.06	1
Solyc01g067650.1.1	Unknown Protein (AHRD V1)	0	0	0	0	0	0	0	0	0	0	1	NA	0	0	0	0	0	0	0	0	1	NA	0	0	0	0	0	0	0	0	1	NA	0	0	0	0	0	0	0	1	NA
Solyc01g067660.2.1	Beta-amylase (AHRD V1 ****	271.15	291.82	548.6	261.67	343.31	258.71	175.88	238.55	206.15	219.82	0.64	0.522117	216.95	241.27	277.98	245.4	307.32	211.76	304.88	274.65	1.12	0.969707	388.8	480.91	569.43	479.71	376.3	458.13	404.32	412.92	0.86	1	834.73	734.82	577.26	715.6	668.35	435.01	551.68	0.77	1
Solyc01g067670.2.1	Unknown Protein (AHRD V1)	0	1.01	5.37	4.83	2.8	0	0.9	4.18	0	1.27	0.45	1	0	0	0.96	0.32	0	0	0.89	0.3	0.93	1	117.62	38	232.57	129.4	41.1	92.24	65.48	66.27	0.51	1	27.49	304.84	234.38	188.9	191.58	69.81	130.69	0.69	1
Solyc01g067680.2.1	Tubby-like F-box protein 6 (A	63.86	72.57	83.44	83.61	75.87	58.67	54.79	51.92	53.05	54.61	0.72	0.455371	54.74	71.57	82.91	69.74	87.3	63.4	71.13	73.94	1.06	1	127.28	118.7	186.76	144.25	97.77	110.6	103.46	103.94	0.72	1	93.05	94.68	91.71	93.15	87.45	95.56	91.5	0.98	1
Solyc01g067690.2.1	Unknown Protein (AHRD V1)	3.95	6.52	6.65	9.57	6.67	9.4	5.79	4.67	6.07	6.48	0.97	1	5.77	2.94	3.33	4.01	2.74	2.34	3.99	3.02	0.75	1	1.37	2.76	3.92	2.68	2.22	2.56	3.7	2.83	1.05	1	0	0.82	0.87	0.56	0.52	1.48	1	1.78	1
Solyc01g067700.2.1	Unknown Protein (AHRD V1)	4.3	5.11	4.65	4.06	4.53	4.78	4.81	8.41	5.41	5.85	1.29	0.789446	3.34	3.12	3.79	3.42	2.36	3.25	2.68	2.76	0.81	1	1.88	2.63	1.96	2.16	0.94	0.82	1.31	1.02	0.47	1	0.76	0.39	0.55	0.57	0.98	1.1	1.04	1.84	1
Solyc01g067710.2.1	Sodium/hydrogen exchanger	5.89	7.42	8.82	5.72	6.96	10.9	7.74	9.13	8.72	9.12	1.31	0.607735	9.92	8.21	7.97	8.7	8.93	10.35	8.38	9.22	1.06	1	16.35	19.98	23.25	19.86	15.55	21	23.86	20.14	1.01	1	8.72	11.22	13.2	11.05	14.26	14.51	14.38	1.3	1
Solyc01g067720.2.1	Vesicle-associated membrane	30.68	40.04	24.94	24.51	30.04	29.86	28.61	33.47	32.75	31.17	1.04	1	34.6	34.27	26.45	31.77	24.7	23.95	34.48	27.71	0.87	1	30.44	27.98	18.78	25.73	19.42	17.35	22.82	19.86	0.77	1	9.72	13.51	10.27	11.17	11.5	10.59	11.04	0.99	1
Solyc01g067730.2.1	Acyl carrier protein (AHRD V1	944.3	917.99	643.11	632.1	784.38	838.23	953.84	998.39	967.99	939.61	1.2	0.674762	711.74	598.59	435.6	581.98	450.88	549.01	633.11	544.33	0.94	1	660.98	725.14	499.48	628.53	642.17	684.33	740.76	689.09	1.1	1	171.16	264.06	269.54	234.92	327.52	423.9	375.71	1.6	0.715254
Solyc01g067740.2.1	Superoxide dismutase (AHRD	1693.69	1367.69	1025.04	1061.85	1287.07	1142.16	1205.39	1028.38	1326.45	1175.6	0.91	1	1293.48	1155.57	854.02	1101.02	1097.85	1030.98	1084.67	1071.17	0.97	1	2922.71	2508.69	2351.37	2594.26	2535.44	2993.23	2541.95	2690.21	1.04	1	1335.85	1804.18	1574.08	1571.37	1944.34	1884.23	1914.28	1.22	1
Solyc01g067750.2.1	Shikimate dehydrogenase (AH	26.1	28.71	28.37	26.49	27.42	34.38	29.19	25.66	27.67	29.23	1.07	1	23.6	21.74	28.27	24.54	22.04	23.71	24.5	23.42	0.95	1	41.58	35.44	38.74	38.59	25.96	28.57	30.64	28.39	0.74	1	18.21	20.8	17.28	18.76	20.57	20.45	20.51	1.09	1
Solyc01g067760.1.1	Photosystem I reaction cente	0.75	4.67	3.54	0	2.24	8.33	1.18	3.45	2.15	3.78	1.69	1	0.7	0.65	0.63	0.66	4.68	1.94	2.95	3.19	4.83	0.494547	2.42	1.22	1.49	1.71	0.49	1.14	2.73	1.45	0.85	1	0	2.18	0	0.73	1.37	1.97	1.67	2.3	1
Solyc01g067770.1.1	DNAJ heat shock N-terminal	0	0	0	0	0	0	0	0	0	0	1	NA	0	0	0	0	0	0	0	0	1	NA	0	0	0	0	0	0	0	0	1	NA	0	0	0	0	0	0	0	1	NA
Solyc01g067780.1.1	Chaperone protein dnaJ 49 (A	0.07	0.19	0.34	0	0.15	0	0	0.07	0.31	0.1	0.63	1	0.34	0.06	0.25	0.22	0	0.08	0.34	0.14	0.65	1	0.06	0	0	0.02	0.1	0	0.05	0.05	2.5	1	0	0.27	0	0.09	0	0.06	0.03	0.33	1
Solyc01g067790.2.1	Mediator of DNA damage che	1.64	1.81	1.74	1.28	1.62	1.76	2.01	1.51	1.87	1.79	1.11	1	1	0.79	1.29	1.03	0.82	0.82	1.02	0.89	0.86	1	0.46	0.55	0.11	0.37	0.22	0.21	0.21	0.21	0.57	1	0.08	0.29	0	0.12	0.36	0.4	0.38	3.08	1
Solyc01g067800.2.1	E3 ubiquitin-protein ligase M	63.4	57.65	61.66	56.47	59.79	50.67	35.44	48.68	35.18	42.49	0.71	0.296819	44.79	70.75	67.15	60.9	45.04	46.93	53.73	48.57	0.8	0.768835	10.66	19.55	12.59	14.27	16.77	15.33	14.73	15.61	1.09	1	18.76	32.61	35.57	28.98	22.27	29.45	25.86	0.89	1
Solyc01g067810.1.1	Unknown Protein (AHRD V1)	0	0	0	1.04	0.26	0	1.16	0	0.53	0.42	1.62	1	0.68	0	0	0.23	0	0	0.58	0.19	0.85	1	0.59	0	0	0.2	0	0	0	0	0.05	1	1.04	0	0	0.35	0	0	0	0.03	1
Solyc01g067820.2.1	Unknown Protein (AHRD V1)	32.99	46.48	36.75	33.7	37.48	30.56	37.62	48.86	41.44	39.62	1.06	1	28.13	46.2	34.05	36.13	24.78	33.68	35.28	31.25	0.86	1	22.21	31.36	25.47	26.35	26.48	18.06	18.03	20.86	0.79	1	23.45	54.57	47.91	41.98	32.71	35.35	34.03	0.81	1
Solyc01g067830.2.1	U1 small nuclear ribonucleop	13.52	17.32	14.01	18.2	15.76	24.2	22.4	20.02	16.81	20.86	1.32	0.583242	13.19	21.22	30.93	21.78	45.07	24.42	27.74	32.41	1.49	0.77852	33.31	56.71	55.66	48.56	42.04	50	47.11	46.38	0.96	1	37.52	21.32	25.36	28.07	38.65	33.84	36.25	1.29	1
Solyc01g067840.2.1	Unknown Protein (AHRD V1)	1.42	0.42	0	0	0.46	1.06	1.5	1.31	0.68	1.14	2.47	0.944397	0	0	0	0	0	0	0	0	1	NA	0	0	0	0	0	0	0	0	1	NA	0	0	0	0	0	0	0	1	NA
Solyc01g067850.2.1	Peroxidase (AHRD V1 **** Q	0	0	0	0	0	0	0	0	0	0	1	NA	0	0	0	0	0	0	0	0	1	NA	0	0	0	0	0	0	0	0	1	NA	0	0	0	0	0	0	0	1	NA
Solyc01g067860.2.1	Peroxidase 24 (AHRD V1 ****	0	0	0	0	0	0	0	0	0	0	1	NA	0	0	0	0	0	0	0	0	1	NA	0	0	0	0	0	0	0	0	1	NA	0	0	0	0	0	0	0	1	NA
Solyc01g067870.2.1	Peroxidase 24 (AHRD V1 ****	0	0	0	0	0	0	0	0	0	0	1	NA	0	0	0	0	0	0	0	0	1	NA	0	0	0	0	0	0	0	0	1	NA	0	0	0	0	0	0	0	1	NA
Solyc01g067880.1.1	Peroxidase 27 (AHRD V1 ****	0	0	0	0	0	0	0	0	0	0	1	NA	0	0	0	0	0	0	0	0	1	NA	0	0	0	0	0	0	0	0	1	NA	0	0	0	0	0	0	0	1	NA
Solyc01g067890.2.1	1-deoxy-D-xylulose 5-phosph	49.02	52.76	49.83	52.75	51.09	46.55	44.27	42.04	49.99	45.71	0.89	1	41.08	44.6	48.83	44.84	71.56	48.34	53.28	57.73	1.29	0.506132	137.24	94.45	154.43	128.71	113.64	182.23	152.77	149.55	1.16	1	85.33	121.58	82.85	96.59	159.11	79.53	119.32	1.24	1
Solyc01g067900.2.1	Rapid alkalinization factor 2 (	156.68	136.42	254.1	323.86	217.77	192.5	169.65	148.07	150.1	165.08	0.76	0.951177	148	103.31	208.54	153.28	91.18	84.89	70.14	82.07	0.54	0.283006	286.09	353.38	401.56	347.01	173.99	193.39	321.67	229.68	0.66	1	30.35	75.4	38.04	47.93	95.47	74.43	84.95	1.77	0.916098
Solyc01g067910.2.1	Unknown Protein (AHRD V1)	0	2.26	5.99	3.59	2.96	11.28	2	9.33	5.46	7.02	2.37	0.876187	7.08	13.23	4.29	8.2	10.56	10.51	13.96	11.68	1.42	1	266.49	153	181.34	200.28	110.01	96.16	299.54	168.57	0.84	1	0	53.44	50.72	34.72	4.65	4.45	4.55	0.13	0.791287
Solyc01g067920.1.1	Protein FAR1-RELATED SEQU	0	0	0	0	0	0	0	0	0	0	1	NA	0	0	0	0	0	0	0	0	1	NA	0	0	0	0	0	0	0	0	1	NA	0	0	0	0	0	0	0	1	NA
Solyc01g067930.2.1	Alpha-1 6-xylosyltransferase	95.05	99.34	115.09	133.56	110.76	63.6	102.44	72.44	83.95	80.61	0.73	0.578787	105.86	75.56	101.41	94.28	50.74	90.01	64.38	68.38	0.73	0.750352	69.21	65.73	77.9	70.95	72.5	53.56	58.78	61.61	0.87	1	66.68	118.54	115.87	100.36	80.14	98.69	89.41	0.89	1
Solyc01g067940.2.1	Unknown Protein (AHRD V1)	0	0	0.57	0	0.14	1.08	0.77	0.45	0	0.58	4.04	0.981941	0	0	0	0	0	0	0	0	1	NA	0	0	0	0	0.32	0	0	0.11	10.67	1	0	0	0	0	0	0	0	1	NA
Solyc01g067950.1.1	Unknown Protein (AHRD V1)	0	0	0	0	0	0	0	0	0	0	1	NA	0	0	0	0	0	0	0	0	1	NA	0	0	0	0	0	0	0	0	1	NA	0	0	0	0	0	0	0	1	NA
Solyc01g067960.1.1	Unknown Protein (AHRD V1)	0	0	0	0	0	0	0	0	0	0	1	NA	0	0	0	0	0	0	0	0	1	NA	0	0	0	0	0	0	0	0	1	NA	0	0	0	0	0	0	0	1	NA
Solyc01g067970.1.1	Unknown Protein (AHRD V1)	0	0	0	0	0	0	0	0	0	0	1	NA	0	0	0	0	0	0	0	0	1	NA	0	0	0	0	0	0	0	0	1	NA	0	0	0	0	0	0	0	1	NA
Solyc01g067980.1.1	Protein phosphatase-2C (AH	0	0	0	0	0	0	0	0	0	0	1	NA	0.3	0	0	0.1	0	0	0	0	0.1	1	0	0	0	0	0	0	0	0	1	1	0	0	0	0	0	0.28	0.14	14	1
Solyc01g067990.1.1	Transducin family protein (AH	0	0	0	0	0	0	0.38	0	0	0.1	9.5	1	0	0	0	0	0	0	0	0	1	NA	0	0	0	0	0	0	0	0	1	NA	0	0	0.74	0.25	0	0	0	0.04	1
Solyc01g068000.2.1	Serine/threonine phosphatas	17.57	23.12	16.54	24.85	20.52	20.77	16.08	20.1	21.47	19.6	0.96	1	23.82	31.03	32.67	29.17	28.91	26.22	28.79	27.97	0.96	1	32.31	42.64	53.24	42.73	39.53	52.13	45.35	45.67	1.07	1	47.83	63.12	54.6	55.18	65.01	70.62	67.81	1.23	1
Solyc01g068010.1.1	Unknown Protein (AHRD V1)	0	0	0	0	0	0	0	0	0	0	1	NA	0	0	0	0	0	0	0	0	1	NA	0	0	0	0	0	0	0	0	1	NA	0	0	0	0	0	0	0	1	NA

Solyc01g068020.2.1	Cyclase/dehydrase (AHRD V1	16.08	11.84	13.41	10.19	12.88	14.79	19.7	12.23	18.71	16.36	1.27	0.6496	18.31	12.05	15.92	15.43	18.65	14.35	18.52	17.17	1.11	1	9.85	12.65	7.7	10.07	6.74	6.93	9.7	7.79	0.77	1	1.97	3.42	1.28	2.22	2.54	1.46	2	0.9	1
Solyc01g068030.2.1	Cyclase/dehydrase (AHRD V1	26.06	19	23.22	20.39	22.17	49.61	47.27	29.56	38.81	41.31	1.86	0.008083	17.53	19.52	18.26	18.44	39.22	31.29	29.63	33.38	1.81	0.024362	27.99	38.4	37.69	34.69	41.15	45.38	49.08	45.2	1.3	0.968845	8.28	13.49	7.25	9.67	25.63	14.83	20.23	2.09	0.737193
Solyc01g068040.1.1	3-5 exonuclease/ nucleic acid	0	0	0	0	0	0	0	0	0	0	1	NA	0	0	0	0	0	0	0	0	1	NA	0	0	0	0	0	0	0	0	1	NA	0	0	0	0	0	0	0	1	NA
Solyc01g068050.2.1	tRNA guanosine-2'-O-m	9.89	11.82	8.36	9.39	9.87	11.16	10.14	13.43	8.26	10.75	1.09	1	9.88	7.7	6.98	8.19	5.94	7.41	8.35	7.23	0.88	1	4.65	5.05	4.1	4.6	4.85	4.25	3.33	4.14	0.9	1	4.41	4.29	4.31	4.34	4.05	5.82	4.94	1.14	1
Solyc01g068060.2.1	Autophagy-related protein 8	50.03	85.62	73.94	62.21	67.95	111.22	68.58	119.8	78.15	94.44	1.39	0.41451	57.79	69.77	66.4	64.65	57.96	60.91	89	69.29	1.07	1	55.96	44.8	55.11	51.96	29.61	42.92	44.47	39	0.75	1	26.58	59.49	36.35	40.81	37.74	22.66	30.2	0.74	1
Solyc01g068070.2.1	Wd-repeat protein (AHRD V1	4.25	3.82	3.55	3.78	3.85	3.23	3.2	4.65	3.7	3.7	0.96	1	3.64	4.89	4.15	4.23	4.76	5.04	5.02	4.94	1.17	0.895788	1.46	1.98	1.99	1.81	1.63	1.77	1.95	1.78	0.99	1	4.95	4.85	5.65	5.15	3.8	5.1	4.45	0.86	1
Solyc01g068080.2.1	Dihydroflavonol 4-reductase	19.11	24.69	13.09	15.99	18.22	18.28	16.71	26.08	17.56	19.66	1.08	1	13.38	14.47	11.11	12.99	10.98	8.3	12.44	10.57	0.81	0.945783	70.57	31.65	61.4	54.54	56.16	54.67	34.15	48.33	0.89	1	2.34	11.94	2.84	5.71	15.8	4.14	9.97	1.75	1
Solyc01g068090.1.1	Unknown Protein (AHRD V1)	0	0	0	0	0	0	0	0	0	0	1	NA	0	0	0	0	0	0	0	0	1	NA	0	0	0	0	0	0	0	0	1	NA	0	0	0	0	0	0	0	1	NA
Solyc01g068100.2.1	Squamosa promoter binding	62.46	70.04	91.6	109.45	83.39	49.16	60.56	50.84	57.17	54.43	0.65	0.32343	66.3	94.81	121.75	94.29	78.04	83.83	78.31	80.06	0.85	1	84.74	80.29	106.02	90.35	98.57	90.1	72.62	87.1	0.96	1	146.4	137.73	157.22	147.12	99.78	114.99	107.38	0.73	1
Solyc01g068110.2.1	Unknown Protein (AHRD V1)	0	0	1.02	0	0.26	0	0	0	0	0	0.04	1	0	0	0	0	0	0	0	0	1	NA	0	0	0	0	0	0	0	0	1	NA	0	0	0	0	0	0	0	1	NA
Solyc01g068120.2.1	Pectinesterase (AHRD V1 ***	0	0	2.27	0	0.57	0	0	0	0	0	0.02	0.985318	0	0	0	0	0	0	0	0	1	NA	0	0	0	0	0	0	0	0	1	NA	0	0	0	0	0	0	0	1	NA
Solyc01g068130.2.1	Os05g0272200 protein (Fragr	33.66	38.9	19.38	43.78	33.93	22.48	27.76	19.47	24.95	23.67	0.7	0.552588	29.42	36.58	37.61	34.54	13.18	21.44	19.95	18.19	0.53	0.013462	2.96	4.42	4.18	3.85	2.85	2.37	1.98	2.4	0.62	1	5.21	4.54	5.84	5.2	7.21	11.07	9.14	1.76	0.698088
Solyc01g068140.2.1	10-deacetylbaccatin III-10-O-	0	0	0	0	0	0	0	0	0	0	1	NA	0	0	0	0	0	0.07	0	0.02	2.33	1	0	0	0	0	0	0.11	0	0.04	3.67	1	0	0	0	0	0	0	0	1	NA
Solyc01g068150.2.1	Chromosome 11 contig 1 DNA	17.25	10.5	13.21	15.84	14.2	16.48	20.31	18.92	15.43	17.79	1.25	0.664595	12.1	18.41	10.99	13.83	12.38	15.36	14.03	13.92	1.01	1	8.56	16.03	9.91	11.5	11.13	11.7	11.25	11.36	0.99	1	14.63	17.36	11.63	14.54	11.91	11.94	11.93	0.82	1
Solyc01g068160.2.1	Cysteine desulfurase like (AH	23.92	23.74	20.7	25.84	23.55	22.4	20.95	18.93	24.25	21.63	0.92	1	27.11	24.83	24.98	25.64	32.68	26.05	24.61	27.78	1.08	1	18.04	17.96	17.27	17.76	22.48	19.67	19.44	20.53	1.16	1	21.44	22.64	22.07	22.05	28.67	26.06	27.37	1.24	1
Solyc01g068170.2.1	Oxidoreductase/ transition m	6.86	5.83	9.57	5.96	7.06	4.33	4.31	5.31	5.6	4.89	0.69	0.648245	7.25	6.1	6.72	6.69	7.46	7.75	6.62	7.28	1.09	1	4.41	5.21	3.25	4.29	4.71	2.72	3.41	3.61	0.84	1	4.66	4.53	4.32	4.5	2.14	2.87	2.5	0.56	0.951723
Solyc01g068180.2.1	Pentatricopeptide repeat-con	3.46	3.45	2.92	3.73	3.39	4.03	3.51	3.56	3.49	3.65	1.08	1	4.44	3.87	3.83	4.05	4.57	4.9	4.85	4.77	1.18	0.913866	3.06	3.76	2.37	3.06	2.71	3.43	3.72	3.29	1.07	1	4.92	6.64	5.32	5.63	4.15	5.27	4.71	0.84	1
Solyc01g068190.2.1	Unknown Protein (AHRD V1)	25.94	35.62	23.61	30.41	28.89	56.03	49.16	40.21	39.91	46.33	1.6	0.219899	23.44	45.75	38.19	35.79	53.47	47.97	38.45	46.63	1.3	0.692054	38.93	48.93	30.9	39.59	36.03	44.38	41.59	40.67	1.03	1	14.75	19.92	10.26	14.98	25.11	21.45	23.28	1.55	1
Solyc01g068200.2.1	Abscisic acid insensitive 8 hor	22.95	30.58	27.67	32.47	28.42	23.14	21.39	25.48	28	24.5	0.86	1	26.6	27.26	25.85	26.57	21.14	23.7	23.06	22.63	0.85	1	17.33	17.99	15.93	17.08	18.21	13.5	16.09	15.93	0.93	1	14.27	10.29	13.11	12.56	12.97	14.3	13.64	1.09	1
Solyc01g068210.2.1	Glutamate dehydrogenase (A	23.9	23.49	24.51	26.45	24.59	21.71	21.41	25.22	19.91	22.06	0.9	1	22.52	25.92	26.24	24.89	25.51	26.2	20.74	24.15	0.97	1	19.3	18.71	19.49	19.17	19.45	18.9	16.66	18.34	0.96	1	35.75	29.96	33.39	33.03	25.11	23.77	24.44	0.74	1
Solyc01g068230.1.1	Ulp1 protease family C-termi	0	0	0	0	0	0	0	0	0	0	1	NA	0	0	0	0	0	0	0	0	1	NA	0	0	0	0	0	0	0	0	1	NA	0	0	0	0	0	0	0	1	NA
Solyc01g068240.2.1	Ubiquitin carboxyl-terminal h	20.01	20.89	22.3	21.14	21.09	25.27	22.63	22.04	21.11	22.76	1.08	0.982081	24.48	19.33	18.36	20.72	19.79	18.74	23.11	20.55	0.99	1	21.76	21.85	20.23	21.28	18.34	20.69	22.96	20.66	0.97	1	12.12	18.57	15.2	15.3	16.85	16.79	16.82	1.1	1
Solyc01g068250.2.1	WD-40 repeat family protein	0	0.28	0.56	0.22	0.27	0.18	0.25	0.15	0.23	0.2	0.76	1	0.15	0.28	0.4	0.28	0.33	0.33	0.25	0.3	1.1	1	1.41	0.77	0.16	0.78	0.21	0.12	0.35	0.23	0.29	1	0.45	0.92	0.49	0.62	0.14	0.69	0.41	0.67	1
Solyc01g068260.2.1	BRCT domain-containing prot	3.29	3.04	2.61	3.03	2.99	2.61	1.96	3.27	2.94	2.69	0.9	1	2.99	3.5	3.62	3.37	2.09	1.98	2.27	2.11	0.63	0.412911	3.9	4.97	5.26	4.71	2.6	3.76	2.49	2.95	0.63	0.876677	4.19	6.23	4.12	4.85	5.83	4.47	5.15	1.06	1
Solyc01g068270.2.1	Receptor-like kinase (AHRD V	58.16	61.25	85.75	102.15	76.83	40.85	68.48	37	45.32	47.91	0.62	0.459044	34.78	62.7	65.96	54.48	36.64	38.91	41.09	38.88	0.71	0.640284	21.38	28.15	18.33	22.62	25.35	21.66	22.83	23.28	1.03	1	7.84	5.01	7.35	6.73	6.14	5.8	5.97	0.89	1
Solyc01g068280.2.1	Chromodomain-helicase-DNA	3.74	4.15	3.58	4.22	3.92	3.5	3.64	4.49	4.13	3.94	1	1	3.73	3.91	4.49	4.04	3.76	5.84	4.95	4.85	1.2	0.811508	2.02	2.65	1.35	2.01	1.64	1.69	2.1	1.81	0.9	1	0.77	2.13	0.67	1.19	1.14	1.09	1.12	0.94	1
Solyc01g068290.2.1	tRNA uridine 5-carboxymethy	5.12	6.05	5.61	5.67	5.61	7.35	8.39	7.2	7.09	7.51	1.34	0.485191	6.41	6.64	8.03	7.03	9.56	6.66	6.59	7.6	1.08	1	5.19	6.76	7.95	6.63	7.41	7.49	7.81	7.57	1.14	1	9.54	6.56	6.45	7.52	9.55	9.31	9.43	1.25	1
Solyc01g068300.1.1	DNA repair protein (AHRD V1	0	0	0	0	0	0	0.13	0	0	0.03	3.25	1	0	0.15	0	0.05	0	0	0	0	0.2	1	0	0	0	0	0	0	0	0	1	NA	0	0	0	0	0	0	0	1	NA
Solyc01g068320.1.1	DNA repair protein (AHRD V1	0	0.09	0	0	0.02	0	0.23	0	0	0.06	2.56	1	0	0.26	0.25	0.17	0.21	0	0.23	0.15	0.86	1	0	0.08	0.1	0.06	0.06	0.15	0.07	0.09	1.56	1	0.14	0.07	0	0.07	0	0	0	0.14	1
Solyc01g068330.2.1	Cell division protease ftsH ho	12.6	12.5	14.39	10.67	12.54	9.19	7.41	9.37	9.33	8.82	0.7	0.391503	12.84	13.05	14.24	13.38	13.25	12.88	12.74	12.96	0.97	1	9.08	10.4	10.52	10	9.86	8.15	9.53	9.18	0.92	1	26.59	23.61	27.15	25.78	16.82	22.03	19.43	0.75	1
Solyc01g068340.2.1	Crossover junction endonucle	2.05	2.73	1.81	1.09	1.92	1.14	2.22	2.47	1.65	1.87	0.97	1	2.38	2.78	1.94	2.37	1.06	1.99	1.31	1.45	0.61	0.808014	0.62	0.73	0.25	0.53	0.34	0.48	0.65	0.49	0.92	1	0.91	0.19	0.59	0.56	0.35	1.35	0.85	1.51	1
Solyc01g068350.2.1	Crossover junction endonucle	3.07	1.09	2.17	2.6	2.23	3.07	3.88	1.97	1.98	2.73	1.22	1	3.14	1.6	1.55	2.1	1.92	1.27	2.89	2.03	0.97	1	0.5	1	0.61	0.7	1.21	0.23	0.22	0.55	0.79	1	0.44	0.22	1.42	0.69	0.56	1.35	0.96	1.38	1
Solyc01g068360.2.1	Receptor like kinase, RLK	0.67	0.9	0.5	0.36	0.61	0.19	0.6	0.93	0.54	0.57	0.93	1	0.7	0.51	0.64	0.62	0.64	0.13	0.07	0.28	0.45	0.699602	0.2	0.27	0.08	0.18	0.17	0.06	0.31	0.18	0.98	1	0	0.12	0	0.04	0.08	0.07	0.08	1.88	1
Solyc01g068370.2.1	Histone-lysine N-methyltrans	2.7	2.75	4.55	3.55	3.39	3.86	2.13	0.89	2.91	2.45	0.72	0.91554	1.97	4.7	2.93	3.2	4.15	3.4	2.12	3.22	1.01	1	1.56	3.93	3.06	2.85	3.55	2.05	2.81	2.8	0.98	1	3.29	2.8	4.75	3.61	2.83	2.2	2.52	0.7	1
Solyc01g068380.2.1	Purple acid phosphatase (AH	0.1	0	0	0	0.03	0	0.16	0	0	0.04	1.6	1	0.1	0.54	0	0.21	0.14	0.05	0.16	0.12	0.55	1	0.58	0.59	0.51	0.56	0.34	0.31	0.3	0.32	0.57	1	0.59	0.67	2.06	1.11	0	0	0	0.01	0.279778
Solyc01g068390.2.1	Zinc finger family protein (AH	17.02	14.14	21.4	22.37	18.73	11.33	10.59	11.3	11.36	11.14	0.59	0.097584	15.76	17.66	19.44	17.62	18.72	13.93	14.02	15.56	0.88	1	17.62	18.82	20.02	18.82	17.38	18.98	16.94	17.77	0.94	1	46.15	28.86	31.59	35.53	25.39	31.48	28.44	0.8	1
Solyc01g068400.2.1	Tubulin-specific chaperone A	245.89	261.54	312.34	324.79	286.14	220.95	225.24	264.68	209.75	230.16	0.8	0.813312	261.4	310.57	345.18	305.72	253.74	262.17	230.27	248.73	0.81	0.883675	157.13	180.82	183.69	173.88	155.34	142.86	139.23	145.81	0.84	1	546.95	340.38	305.66	397.66	329.4	346.3	337.85	0.85	1
Solyc01g068410.2.1	Auxin Efflux Carrier (AHRD V1	0	0.98	0.43	0	0.35	0.2	0.29	0	0	0.12	0.35	0.889107	0	0	0	0	0	0	0	0	1	NA	0	0	0	0	0	0	0	0	1	NA	0	0	0.28	0.09	0	0	0	0.11	1
Solyc01g068420.2.1	Os06g0207500 protein (Fragr	0	0	0	0	0	0	0	0	0	0	1	NA	0	0	0	0	0	0	0	0	1	NA	0	0	0	0	0	0	0	0	1	NA	0	0	0	0	0	0	0	1	NA
Solyc01g068430.1.1	Os06g0207500 protein (Fragr	0.19	2.85	5.78	0	2.21	0.21	4.76	0	0	1.24	0.56	1	0	0.16	0.16	0.11	0	0	0	0	0.09	1	0.15	0.31	0	0.15	0	0	0.27	0.09	0.59	1	0	0	0.29	0.1	0	0	0	0.1	1
Solyc01g068440.1.1	Os06g0207500 protein (Fragr	0	0	0.2	0	0.05	0	0	0	0	0	0.2	1	0	0	0	0	0	0	0	0	1	NA	0	0	0	0	0	0	0	0	1	1	0	0.24	0	0.08	0.46	0	0.23	2.88	1
Solyc01g068450.2.1	Genomic DNA chromosome 3	29.9	28.34	23	20.23	25.37	21.31	20.53	30.18	23.33	23.84	0.94	1	29.02	33.05	26.35	29.47	57.03	43.08	40.1	46.74	1.59	0.096659	28.88	33.37	27.42	29.89	42.48	39.16	33.15	38.26	1.28	1	89.64	82.85	81.96	84.82	79.73	99.16	89.44	1.05	1
Solyc01g068460.2.1	Pathogen-induced calmodulin	77.37	78.19	108.55	85.2	87.33	55.02	43.48	61.96	59.31	54.94	0.63	0.090781	75.91	81.57	115.87	91.12	105.3	96.79	80.94	94.34	1.04	1	13.31	17.35	12.06	14.24	19.29	13.62	11.82	14.91	1.05	1	16.56	19.94	14.97	17.16	11.58	14.59	13.09	0.76	1
Solyc01g068470.2.1	CM0216.540.nc protein (Frag	10.17	9.6	6	5.67	7.86	12.16	12.53	9.78	13.17	11.91	1.52	0.285056	5.59	7.77	6.9	6.75	11.76	10.85	7.75	10.12	1.5	0.307563	2.99	3.52	2.29	2.93	4.86	2.8	4.16	3.94	1.34	1	2.41	3.69	3.56	3.22	4.23	2.7	3.47	1.08	1
Solyc01g068480.2.1	Embryo defective 2765 (Fragr	18.06	18.07	33.26	28.42	24.45	16.94	15.21	15.38	17.97	16.38	0.67	0.51304	21.9	27	40.61	29.84	42.88	31.06	29.36	34.43	1.15	1	13.62	16.61	17.68	15.97	16.99	13.75	16.82	15.85	0.99	1	27.68	15.62	22.54	21.95	21.85	28.28	25.07	1.14	1
Solyc01g068490.2.1	U-box domain-containing pro	0.69	0.34	0.81	0.54	0.59	0.17	0.48	0.21	0.33	0.3	0.5	0.759724	0.43	2.46	1.29	1.39	1.11	0.52	0.48	0.7	0.5	0.872742	2.35	1.56	2.13	2.01	2.11	1.45	1.23	1.6	0.79	1	0.54	5.73	4.47	3.58	0.7	0.81	0.76	0.21	0.724055
Solyc01g068500.2.1	Early-responsive to dehydrati	0.28	0.58	0.55	0.53	0.49	0.62	0.07	0.26	0.13	0.27	0.56	0.814512	0.26	0.24	0.32	0.27	0.32	0.34	0.07	0.24	0.89	1	3.7	1.45	3.43	2.86	0.92	1.56	1.7	1.39	0.49	1	0.66	1.49	0.86	1	2.31	1.97	2.14	2.13	0.94508
Solyc01g068510.1.1	Unknown Protein (AHRD V1)	0	0	0	0	0	0	0	0	0	0	1	NA	0	0	0	0	0	0	0	0	1	NA	0	0	0	0	0	0	0	0	1	NA	0	0	0	0	0	0	0	1	NA
Solyc01g068520.1.1	Unknown Protein (AHRD V1)	0	0	0	0	0	0	0	0	0	0	1	NA	0	0	0	0	0	0	0	0	1	NA	0	0	0	0	0.65	0	0	0.22	21.67	1	0	0	0	0	0	0	0	1	NA
Solyc01g068530.2.1	30S ribosomal protein S9 (AH	422.48	345.28	438.13	444.95	412.71	276.82	225.23	180.36	314.6	249.25	0.6	0.062212	386.48	358.52	318.09	354.36	331.2	279.82	303.52	304.85	0.86	0.988213	167.31	180.85	168.69	172.28	192.01	182.12	180.2	184.78	1.07	1	151.57	151.04	170.65	157.75	299.95	383.06	341.5	2.16	0.100166
Solyc01g068540.2.1	Unknown Protein (AHRD V1);	3.63	2.83	2.14	3.42	3	2.02	4.9	3.06	3.69	3.42	1.14	0.962949	1.13	1.97	2.43	1.84	1.99	2.51	1.78	2.09	1.14	1	0.86	1.48	0.75	1.03	1.09	0.69	0.88	0.89	0.86	1	1.08	1.76	0.7	1.18	1.25	0.93	1.09	0.92	1
Solyc01g068550.2.1	O-methyltransferase (AHRD V	0.19	0.17	0.22	0.26	0.21	0	0.3	0.34	0	0.16	0.76	1	0.35	0.49	0.16	0.33	0.65	0.68	0.59	0.64	1.92	0.973796	0	0.3	0	0.1	0	0	0	0	0.1	1	0	0	0	0	0	0	0	1	NA
Solyc01g068560.2.1	Unknown Protein (AHRD V1)	320.43	364.87	441.97	269.95	349.31	673.08	431.54	615.23	482.08	550.48	1.58	0.087453	348.47	178.19	97.51	208.06	109.49	96.37	118.5	108.12	0.52	0.808452	102.79	67.2	56.13	75.37	32.5	26.79	39.49	32.93	0.44	0.395673	117.25	147.17	101.45	121.96	114.31	128.08	121.2	0.99	1
Solyc01g068580.2.1	C9orf78 (AHRD V1 **-- C3KG	36.52	29.46	28.82	24.54	29.84	24.11	22.89	31.27	29.99	27.06	0.91	1	32.06	31.55	25.64	29.75	22.01	23.05	32.65	25.9	0.87	1	34.86	31.99	23.79	30.21	39.89	30.1	30.94	33.64	1.11	1	41.87	71.29	61.43	58.2	41.37	37.03	39.2	0.67	0.808535
Solyc01g068590.2.1	Unknown Protein (AHRD V1)	16.49	33.94	36.48	45.2	33.03	28.65	31.74	28.43	28.86	29.42	0.89	1	20.14	46.59	58.33	41.69	45.04	41.1	47.79	44.64	1.07	1	24.15	22.68	28.65	25.16	29.12	15.63	24.79	23.18	0.92	1	231.56	74.12	80.84	128.84	68.88	75.92	72.4	0.56	1
Solyc01g068600.2.1	Paladin-like protein (AHRD V	9.99	16.43	17.49	19.26	15.79	12.11	15.77	16.69	14.77	14.84	0.94	1	12.1	19.73	26.84	19.56	23.09	17.55	20.93	20.52	1.05	1	11.25	10.36	13.82	11.81	14.51	8.03	12.79	11.78	1	1	111.87	32.75	40.02	61.55	36.84	35.02	35.93	0.58	1
Solyc01g068610.2.1	Metal ion binding protein (AH	12.17	13.9	14.72	18.96	14.94	9.43	12.18	12.55	13.11	11.82	0.79	0.856227	13.27	18.28	20.15	17.23	16.12	14.52	14.45	15.03	0.87	1	9.79	11.02	12.88	11.23	11.92	10.25	9.95	10.71	0.95	1	99.57	31.57	34.51	55.22	28.31	30.03	29.17	0.53	1
Solyc01g068620.2.1	Cyclase/dehydrase (AHRD V1	24.48	21.39	19.26	23.09	22.06	25.96	25.77	25.85	26.69	26.07	1.18	0.765955	18.35	18.92	17.62	18.3	25.47	23.59	19.6	22.89	1.25	0.687038	23.81	27.34	20.25	23.8	25.62	27.49	28.75	27.29	1.15	1	6.77	9.88	4.18	6.94	19.51	16.5	18.01	2.59	0.277633
Solyc01g068630.2.1	Pyrimidine 5'-nucleotid	3.39	2.55	4	1.84	2.94	7.25	4.53	5.99	6.74	6.13	2.08	0.168534	1.94	2.49	2.42	2.28	0.54	1.22	1.02	0.93	0.41	0.567763	4	4.25	1.29	3.18	1.54	1.78	1.9	1.74	0.55	0.967597	0	0.76	0	0.25	0.48	0.91	0.7	2.74	1
Solyc01g068640.2.1	Uncharacterized aarF domain	8.83	6.7	7.72	6.94	7.55	9.33	8.52	9.22	8.3	8.84	1.17	0.849483	9.23	5.88	7.64	7.58	3.93	5.25	7.11	5.43	0.72	0.690491	3.61	3.47	2.6	3.23	3.94	2.89	3.5	3.44	1.07	1	1.86	2.77	2.01	2.21	1.2	2.29	1.75	0.79	1
Solyc01g073640.2.1	Uncharacterized oxidoreducta	199.69	346.86	403.83	1226.03	544.1	393.74	825.51	338.97	444.66	500.72	0.92	1	33.99	26.25	29.15	29.8	5.46	7.23	10.17	7.62	0.26	3.3E-08	0.41	2.48	3.27	2.05	1.33	0.38	0.18	0.63	0.31	1	3.61	2.58	3.9	3.36	2.09	2.22	2.16	0.64	1
Solyc01g073650.2.1	Serine/threonine-protein pho	106.65	125.49	108.17	115.68	114	95.59	103.33	112.79	89.13	100.21	0.88	0.982298	109.2	119.64	103.43	110.76	82.01	91.45	104.68	92.71	0.84	0.929648	105.92	103.22	85.78	98.31	84.32	65.82	80.33	76.82	0.78	1	81.81	67.05	85.24	78.03	63.09	70.04	66.56	0.85	1
Solyc01g073660.2.1	Uncharacterized MFS-type tra	1.74	1.55	1.71	0.82	1.46	1.13	0.57	3.33	1.66	1.67	1.15	1	1.75	1.89	1.71	1.78	0.7	2.02	2.39	1.7	0.96	1	10.52	6.96	5.74	7.74	2.09	2.52	2	2.2	0.28	0.629657	0.62	1.68	1.33	1.21	1.06	1.27	1.17	0.96	1
Solyc01g073670.2.1	Uncharacterized MFS-type tra	0	0	0	0	0	0	0	0	0	0	1	NA	0	0	0.12	0.04	0	0	0	0	0.25	1	0.24	0.12	0.29	0.22	0	0	0	0	0.05	1	0	0	0	0	0	0	0	1	NA
Solyc01g073680.2.1	Non-specific lipid-transfer pro	7.44	8.73	6.79	1.44	6.1	18.8	13.62	12.13	8.5	13.26	2.17	0.200703	5.66	2.65	0.86	3.06	1.41	1.4	2.66	1.82	0.6	1	0.82	1.38	0.67	0.96	0	0.26	0	0.09	0.09	1	0	0.25	0	0.08	0	0	0	0.12	1
Solyc01g073690.2.1	V-type ATP synthase subunit	201.39	200.39	201.99	231.59	208.84	218.21	201.37	204.56	213.99	209.53	1	1	171.92	164.84	167.39	168.05	142.46	140.78	145.25	142.83	0.85	0.969905	177.59	201.32	199.1	192.67	165.11	166	178.28	169.8	0.88	1	113.92	177.31	149.09	146.77	182.64	150.81	166.72	1.14	1
Solyc01g073700.2.1	26S protease regulatory subu	2.88	2.9	1.18	1.6	2.14	2.37	2.08	2.08	2.7	2.31	1.08	1	0.93	1.64	0.95	1.17	0.87	0.65	0.2	0.57	0.49	0.73259	0	0.1	0	0.03	0.25	0.19	0	0.15	4.4	1	0	0	0	0	0.23	0	0.12	11.5	1
Solyc01g073710.2.1	Calcium-binding EF hand fam	25.92	31.87	18.9	21.33	24.5	25.67	27.53	35.09	26.39	28.67	1.17	0.933869	13.15	18.02	17.51	16.23	16.67	14.39	15.92	15.66	0.97	1	6.85	7.68	5.61	6.71	5.57	3.57	8.93	6.02	0.9	1	6.7	3.42	2.17	4.1	4.74	2.48	3.61	0.88	1
Solyc01g073720.2.1	Unknown Protein (AHRD V1)	0	0	0	0	0	0	0	0	0	0	1	NA	0	0	0	0	0	0	0	0	1	NA	0	0.6	0	0.2	0	0	0	0	0.05	1	0	0	0	0	0	0	0	1	NA
Solyc01g073730.2.1	Molybdenum cofactor biosyn	16.41	19.47	18.77	14.3	17.24	17.5	14.13	22.71	14.04	17.09	0.99	1	16.5	13.4	13.72	14.54	15.87	13.82	14.33	14.67	1.01	1	9.37	8.51	9.38	9.09	8.93	7.41	6.64	7.66	0.84	1	7.54	9.63	6.88	8.02	6.37	5.81	6.09	0.76	1
Solyc01g073740.2.1	Citrate synthase (AHRD V1 **	48.33	62.17	52.43	57.36	55.07	41.84	39.47	43.03	43.57	41.98	0.76	0.569997	54	42.74	40.12	45.62	28.8	33.29	37.66	33.25	0.73	0.519339	24.46	31.8	22.2	26.15	20.26	18.53	24.8	21.2	0.81	1	31	32.78	32.68	32.15	32.67	28.01	30.34	0.94	1
Solyc01g073750.2.1	Glucan synthase like 3 (AHRD	28.86	43.02	48.76	44.61	41.31	32.57	24.26	41.94	40.75	34.88	0.84	0.944449	39.94	54.92	54.51	49.79	33.17	33.42	36.14	34.24	0.69	0.223171	13.66	16.08	17.03	15.59	15.88	14.62	12.27	14.26	0.91	1	48.7	34.09	42.86	41.88	30.18	35.66	32.92	0.79	1
Solyc01g073760.2.1	Callose synthase 1 (AHRD V1	30.88	92.44	69.55	55.59	62.12	88.93	29.92	95.5	53.41	66.94	1.08	1	49.61	71.99	73.5	65.03	32.13	37.06	55.15	41.45	0.64	0.440282	22.68	16.01	23.69	20.79	8.3	17.02	6.14	10.49	0.5	0.958652	41.91	61.17	62.59	55.22	20.56	24.61	22.59	0.41	0.448699
Solyc01g073770.2.1	PRLI-interacting factor A (AH	15.06	8.51	14.38	13.52	12.87	11.69	10.19	12.31	8.58	10.69	0.83	0.980705	15.79	16.83	18.37	17	22.05	21.41	14.09	19.18	1.13	0.953691	19.7	36.03	22.54	26.09	33.29	19.57	25.6	26.15	1	1	33.3	42.36	38.59	38.08	19.91	22.63	21.27	0.56	0.54909
Solyc01g073780.2.1	CHP-rich zinc finger protein-li	0	0	0	0	0	0	0	0	0	0	1	NA	0	0	0	0	0	0	0	0	1	NA	0	0	0	0	0	0	0	0	1	NA	0	0	0	0	0	0	0	1	NA
Solyc01g073800.2.1	CHP-rich zinc finger protein-li	0	0.24	0	0	0.06	0	0	0	0	0	0.17	1	0	0	0	0	0	0	0	0	1	NA	0	0	0	0	0	0	0	0	1	NA	0	0	0.41	0.14	0	0	0	0.07	1
Solyc01g073810.2.1	DC1 domain containing prote	0	0	0	0	0	0	0	0	0	0	1	NA	0.25	0	0	0.08	0.18	0	0	0.06	0.72	1	0	0	0	0	0	0	0	0	1	NA	0	0.19	0	0.06	0	0	0	0.16	1
Solyc01g073820.2.1	CHP-rich zinc finger protein-li	0	0.23	0	0.36	0.15	0.28	0	0	0	0.07	0.47	1	0.24	0.88	0	0.37	0.88	0	0	0.29	0.79	1	0	0	0	0	0	0	0	0	1	NA	0.72	0.18	0.78	0.56	0	0.44	0.22	0.39	1
Solyc01g073830.1.1	CHP-rich zinc finger protein-li	0	0	0	0	0	0	0	0	0	0	1	NA	0	0	0	0	0	0	0	0	1	NA	0	0	0	0	0	0	0	0	1	NA	0	0	0	0	0	0	0	1	NA
Solyc01g073840.1.1	CHP-rich zinc finger protein-li	0	0	0	0	0	0	0	0	0	0	1	NA	0	0	0	0	0	0	0	0	1	NA	0	0	0	0	0	0	0	0	1	NA	0	0	0	0	0	0	0	1	NA
Solyc01g073850.1.1	DC1 domain containing prote	0	0	0	0	0	0	0	0	0	0	1	NA	0	0	0	0	0	0	0	0	1	NA	0	0	0	0	0	0	0	0	1	NA	0	0	0	0	0	0	0	1	NA
Solyc01g073860.2.1	CHP-rich zinc finger protein (A	0	0	0	0	0	0	0	0	0	0	1	NA	0	0	0	0	0	0	0	0	1	NA	0	0	0	0	0	0	0	0	1	NA	0	0	0	0	0	0	0	1	NA
Solyc01g073870.2.1	ATP synthase subunit-like pro	31.74	29.28	27.45	27.27	28.93	26.04	29.78	34.53	27.92	29.57	1.02	1	30.76	37.85	35.51	34.71	33.65	39.58	33.56	35.6	1.03	1	30.61	29.93	28.04	29.53	34.28	27.08	31.85	31.07	1.05	1	72.3	80.98	93.53	82.27	54	62.21	58.11	0.71	0.977509
Solyc01g073880.2.1	CHP-rich zinc finger protein-li	0.34	0	0	0	0.09	0	0	0.31	0.24	0.14	1.62	1	0.32	0.59	0.29	0.4	0	0	0.53	0.18	0.44	1	0	0	0	0	0	0	0	0	1	NA	0	0	1.05	0.35	0	0	0	0.03	1
Solyc01g073890.2.1	CHP-rich zinc finger protein-li	0	0	0	0.49	0.12	0	0	0.16	0	0.04	0.33	1	0	0.75	0.58	0.44	1.2	0.18	0.14	0.51	1.14	1	0.14	0.42	0.34	0.3	0.11	0.13	0	0.08	0.27	1	2.46	7.9	12.22	7.53	0.47	1.51	0.99	0.13	0.43465
Solyc01g073900.2.1	RING finger protein 5 (AHRD	55.2	50.33	51.23	49.99	51.69	40.03	38.8	41.47	40.35	40.16	0.78	0.703375	52.6	59.98	48.61	53.73	48.56	46.53	53.96	49.68	0.92	1	58.19	49.37	45.77	51.11	57.49	56.38	49.06	54.31	1.06	1	62.21	116.85	106.62	95.23	61.01	66.7	63.86	0.67	0.796983
Solyc01g073910.2.1	Homeobox-leucine zipper pro	0.18	0.16	0.21	1.27	0.45	0.4	0.43	0.17	0.13	0.28	0.62	1	0.34	0.47	0.91	0.57	0.38	0	0	0.13	0.22	0.630791	0	0	0	0	0	0	0	0	1	NA	0.26	0.39	0.83	0.49	0	0	0	0.02	0.975227
Solyc01g073920.2.1	Unknown Protein (AHRD V1)	7.5	6.5	13.61	11.7	9.83	4.49	7.9	4.77	6.77	5.98	0.61	0.564117	9.48	10.37	13.97	11.27	10	8.17	5.59	7.92	0.7	0.693469	4.2	5.33	6.68	5.4	7.45	6.27	4.63	6.12	1.13	1	13.95	8.94	8.28	10.39	18.66	17.37	18.02	1.73	0.654344
Solyc01g073930.2.1	N-terminal asparagine amido	5.15	7.52	6.5	5.72	6.22	5.72	5.95	7.94	7.92	6.88	1.11	1	8.2	5.27	6.98	6.82	5.35	4.95	6.79	5.7	0.84	1	6.24	7.04	5.29	6.19	4.95	4.74	6.29	5.33	0.86	1	5.23	4	3.39	4.21	6.22	5.32	5.77	1.37	1
Solyc01g073940.2.1	Zinc finger CCCH domain-con	17.97	15.79	16.37	17.7	16.96	12.9	17.42	16.94	20.9	17.04	1	1	14.6	20.76	21.71	19.02	21.93	20.73	24.13	22.26	1.17	0.929939	23.16	26.14	22.58	23.96	28.39	24.83	25.03	26.08	1.09	1	19.73	20.32	13.99	18.01	31.86	35.9	33.88	1.88	0.537808
Solyc01g073950.2.1	Bromodomain protein (AHRD	11.56	10.99	13.13	17.23	13.23	7.11	9.48	10.55	8.05	8.8	0.67	0.375725	14.83	29.4	27.16	23.8	13.31	14.11	13.77	13.73	0.58	0.274002	17.98	29.06	20.96	22.67	32.46	17.53	23.43	24.47	1.08	1	20.3	43.52	44.46	36.09	21.57	27.19	24.38	0.68	0.934906
Solyc01g073960.2.1	ENT domain containing prote	39.15	38.96	34.55	34.45	36.78	35.47	40.04	42.66	39.06	39.31	1.07	0.996253	36.7	42.34	42.18	40.41	49.82	44.05	43.37	45.75	1.13	0.911351	42.34	43.96	31.06	39.12	42.43	44.39	41.45	42.76	1.09	1	61.58	57.43	56.17	58.39	43.04	37.71	40.38	0.69	0.938951
Solyc01g073970.2.1	Histone H3 (AHRD V1 ***- D8	16.37	6.43	7.39	17.71	11.98	5.89	12.17	2.66	6.91	6.91	0.58	0.804781	15.23	12.98	6.1	11.44	2.67	2.99	2.27	2.64	0.23	0.034389	2.33	7.85	0.48	3.55	0.95	0.36	2.11	1.14	0.32	0.692516	1.37	0.35	0.74	0.82	2.2	0.84	1.52	1.85	1
Solyc01g073980.1.1	Unknown Protein (AHRD V1)	0.19	0	0	0	0.05	0	0	0	0	0	0.21	1	0.18	0	0	0.06	0	0	0	0	0.17	1	0	0	0	0	0	0	0.14	0.05	4.67	1	0	0	0	0	0	0	0	1	NA
Solyc01g073990.2.1	Unknown Protein (AHRD V1)	7.33	5.44	2.88	1.73	4.34	2.72	4.82	5.62	3.51	4.17	0.96	1	9.09	7.43	6.19	7.57	7.63	2.53	12.48	7.55	1	1	0.99	1	0	0.66	0	0	0	0	0.02	1	0	0.89	0	0.3	0	1.07	0.54	1.8	1
Solyc01g074000.2.1	Histone H3 (AHRD V1 ***- D8	36.12	27	36.36	66.07	41.39	26.76	48.66	24.79	39.07	34.82	0.84	1	43.45	57.35	59.38	53.39	19.71	25.68	18.16	21.18	0.4	0.000881	14.78	38.45	2.87	18.7	3.8	1.46	9.12	4.79	0.26	0.577095	7.53	3.85	2.96	4.78	26.45	13.93	20.19	4.22	0.179664
Solyc01g074010.2.1	Protein kinase (AHRD V1 *-*-	34.22	29.1	32.41	48.67	36.1	26.1	42.85	29.1	30.57	32.16	0.89	1	33.78	33.05	41.43	36.09	25.38	25.3	26.3	25.66	0.71	0.393815	29.49	36.87	33.97	33.44	30.35	29.61	29.51	29.82	0.89	1	109.48	45.91	58.91	71.43	49.25	51.34	50.3	0.7	1
Solyc01g074020.2.1	Box C/D snoRNA (AHRD V1 *-	8.29	7.38	9.16	7.24	8.02	6.27	5.43	9.08	7.34	7.03	0.88	1	9.84	9.35	8.34	9.18	8.93	6.85	8.04	7.94	0.87	1	10.12	8.91	8.93	9.32	9.39	9.49	9.13	9.34	1	1	12.29	9.61	8.95	10.28	10.01	10.52	10.27	1	1
Solyc01g074030.2.1	Beta-glucosidase 01 (AHRD V	20.38	35.02	16.08	16.03	21.88	59.18	40.32	48.95	31.64	45.02	2.06	0.066379	16.23	15.28	10.33	13.95	9.72	14.64	16.31	13.56	0.97	1	0.21	1.04	0.51	0.59	0.33	0	0.56	0.3	0.51	1	0	0	0	0	0.12	0	0.06	6	1
Solyc01g074040.1.1	Beta-glucosidase 01 (AHRD V	0	0	0	0	0	0	0	0	0	0	1	NA	0	0	0	0	0	0	0	0	1	NA	0	0	0	0	0	0	0	0	1	NA	0	0	0	0	0	0	0	1	NA
Solyc01g074050.1.1	Beta-glucosidase (AHRD V1 *	0	0	0	0	0	0	0	0	0	0	1	NA	0	0	0	0	0	0	0	0	1	NA	0	0	0	0	0	0	0	0	1	NA	0	0	0	0	0	0	0	1	NA
Solyc01g074060.1.1	Beta-glucosidase 01 (AHRD V	0	0	0	0	0	0	0	0	0	0	1	NA	0	0	0	0	0	0	0	0	1	NA	0	0	0	0	0	0	0	0	1	NA	0	0	0	0	0	0	0	1	NA
Solyc01g079060.2.1	Charged multivesicular body	2.5	3.9	2.95	2.21	2.89	3.82	2.96	2.3	1.8	2.72	0.94	1	3.2	2.72	2.64	2.85	3.04	2.43	1.97	2.48	0.87	1	2.53	2.8	1.55	2.29	1.03	1.9	0.46	1.13	0.49	1	1.33	2.04	2.4	1.92	3.43	3.29	3.36	1.75	1
Solyc01g079070.2.1	SNF7 family protein (AHRD V	10.59	10.15	9.29	10.37	10.1	8.15	9.64	9.73	11.5	9.75	0.97	1	9.09	12.51	10.09	10.56	10.36	11.95	10.88	11.06	1.05	1	5.04	7.52	5.93	6.16	6.96	7.2	8.9	7.69	1.25	1	10.42	9.66	10.02	10.03	10.19	12.85	11.52	1.15	1
Solyc01g079080.2.1	Coiled-coil protein (AHRD V1	0.25	0.37	0.3	1.19	0.53	0.19	0.13	0.08	0.3	0.17	0.33	0.720542	0.24	0.59	0.71	0.51	0	0.13	0.13	0.09	0.17	0.208187	0.07	0	0.08	0.05	0	0	0	0	0.2	1	0.24	0.24	0.13	0.2	0.08	0.07	0.08	0.37	1
Solyc01g079090.2.1	Protoporphyrinogen oxidase	30.92	34.66	33	33.39	32.99	52.74	56.93	42.97	51.26	50.97	1.55	0.048323	32.71	36.01	41.57	36.76	79.56	56.34	56.02	63.97	1.74	0.031022	13.83	28.27	25.89	22.66	27.68	26.66	35.95	30.1	1.33	0.702525	43.96	54	51.72	49.89	66.01	72.85	69.43	1.39	0.987061

Solyc01g079100.2.1	Palmitoyltransferase erf2 (AH	0.38	0.34	0	0.54	0.32	0	0.2	0	0	0.05	0.16	0.616307	0.12	0.11	0	0.08	0.09	0	0.1	0.06	0.83	1	0	0	0	0	0	0	0	0	1	NA	0	0.09	0	0.03	0	0.11	0.06	1.83	1
Solyc01g079110.2.1	Histone H3 (AHRD V1 ***- D8	25.52	30	19.32	41.55	29.1	18.73	25.47	18.15	21.09	20.86	0.72	0.717334	21.95	30.14	24.4	25.5	9.02	14.96	15.89	13.29	0.52	0.126756	5.06	18.44	1.43	8.31	2.53	0	3.51	2.01	0.24	0.867269	2.05	0.35	0	0.8	5.29	3.38	4.33	5.42	0.566555
Solyc01g079120.2.1	Unknown Protein (AHRD V1)	0	0	0	0	0	0	0	0	0	0	1	NA	0	0	0	0	0	0	0	0	1	NA	0	0	0	0	0	0	0	0	1	NA	0	0	0	0	0	0	0	1	NA
Solyc01g079130.1.1	Polygalacturonase (AHRD V1	0	0	0	0	0	0	0	0	0	0	1	NA	0	0	0	0	0	0	0	0	1	NA	0	0	0	0	0	0	0	0	1	NA	0	0	0	0	0	0	0	1	NA
Solyc01g079140.2.1	Unknown Protein (AHRD V1)	1.87	1.25	0.55	1.32	1.25	0	0	0	0	0	0.01	0.195768	0	1.22	0.79	0.67	0.32	0.24	0	0.19	0.28	1	0	0.38	0	0.13	0	0	0	0	0.08	1	0	0.34	0	0.11	0.43	0.41	0.42	3.71	1
Solyc01g079150.2.1	AE family transporter anion e	2.84	3.66	2.27	2.07	2.71	1.53	2.24	1.93	2.1	1.95	0.72	0.740223	2.04	1.59	0.7	1.44	0.7	1.04	0.72	0.82	0.57	0.80116	0.59	0.6	0.36	0.52	0.48	0.49	0.47	0.48	0.93	1	0.26	0.53	0.7	0.5	0.42	0.72	0.57	1.15	1
Solyc01g079160.2.1	GDSL esterase/lipase At1g09	0	0	0	0	0	0	0	0	0	0	1	NA	0	0	0	0	0	0	0	0	1	NA	0	0	0	0	0	0	0	0	1	NA	0	0	0	0	0	0	0	1	NA
Solyc01g079170.2.1	Galactinol synthase (AHRD V1	0.95	1.35	0.89	1.07	1.06	1.26	0.45	2.44	4.22	2.09	1.96	0.689726	4.23	6.76	3.52	4.84	18.28	13.64	11.62	14.51	3	0.000761	1.68	1.24	0.56	1.16	0.75	1.44	1.11	1.1	0.95	1	0.54	0.14	0.58	0.42	1.04	0.17	0.6	1.44	1
Solyc01g079180.2.1	Pectinesterase (AHRD V1 ***	0	0	0	0	0	0	0	0	0	0	1	NA	0	0	0	0	0	0	0	0	1	NA	0	0	0	0	0	0	0	0	1	NA	0	0	0	0	0	0	0	1	NA
Solyc01g079190.2.1	Unknown Protein (AHRD V1)	0	0	0	0	0	0	0	0	0	0	1	NA	0	0	0	0	0	0	0	0	1	NA	0	0	0	0	0	0	0	0	1	NA	0	0	0	0	0	0	0	1	NA
Solyc01g079200.2.1	Gibberellin 2-oxidase (AHRD	0	0	0	0	0	0	0	0	0	0	1	NA	0.18	0	0	0.06	0	0	0	0	0.17	1	0	0	0	0	0	0	0	0	1	NA	0	0	0	0	0.18	0	0.09	9	1
Solyc01g079210.2.1	SWR1-complex protein 4 (AH	14.74	13.51	13.78	14.04	14.02	13.3	12.33	13.96	14.36	13.49	0.96	1	16.02	15.22	15.29	15.51	15.59	16.25	18.01	16.62	1.07	1	12.15	14.27	8.4	11.61	10.93	13.27	11.81	12	1.03	1	11	7.95	7.86	8.94	8.68	10.37	9.52	1.07	1
Solyc01g079220.2.1	NifU like protein (AHRD V1 *	26.81	26.01	22.75	25.99	25.39	40.52	45.9	34.62	36.77	39.45	1.55	0.113739	21.86	27.76	23.92	24.51	38.03	32.91	32.2	34.38	1.4	0.365267	34.8	38.78	37.67	37.08	30.28	41.55	34.24	35.36	0.95	1	11.14	22.1	14.82	16.02	35.57	25.62	30.59	1.91	0.698751
Solyc01g079230.2.1	Agenet domain-containing pr	26.7	27.07	29.73	24.24	26.93	24.6	28.13	34.73	26.35	28.45	1.06	1	29.16	31.4	37.33	32.63	27.14	31.31	31.42	29.96	0.92	1	30.98	25.19	29.41	28.53	31.57	24.95	24.4	26.97	0.95	1	56.03	41.64	51.6	49.76	28.63	30.67	29.65	0.6	0.707581
Solyc01g079240.2.1	Long-chain-fatty-acid--CoA lig	19.57	18.75	14.07	19.11	17.88	10.49	11.14	13.06	13.34	12.01	0.67	0.291359	15.06	8.46	10.15	11.22	2.21	4.37	4.92	3.83	0.34	0.026414	11.72	10.04	9.37	10.38	1.37	2.26	1.38	1.67	0.16	0.012663	2.4	2.31	4.43	3.05	0.73	0.44	0.58	0.19	0.088832
Solyc01g079250.2.1	mRNA splicing factor ATP-dep	119.46	115.97	159.32	135.08	132.46	118.4	111.36	105.81	110.1	111.42	0.84	0.919033	125.36	113.11	127.76	122.08	112.3	114.83	120.02	115.72	0.95	1	72.81	81.01	78.14	77.32	72.79	69.34	70.72	70.95	0.92	1	121.65	88.68	119.24	109.86	87	103.54	95.27	0.87	1
Solyc01g079260.2.1	WRKY transcription factor 4 (	17.06	15	11.4	11.63	13.77	4.8	6.17	7.56	5.75	6.07	0.44	0.006986	15.1	17.69	8.33	13.71	13.68	12.34	11.63	12.55	0.92	1	89.5	95.62	99.35	94.82	119.08	93.62	121.91	111.54	1.18	1	26.01	91.05	116.62	77.89	121.72	118.39	120.06	1.54	1
Solyc01g079270.1.1	Unknown Protein (AHRD V1)	0	0	0	0	0	0	0	0	0	0	1	NA	0	0	0	0	0	0	0	0	1	NA	0	0	0	0	0	0	0	0	1	NA	0	0	0	0	0	0	0	1	NA
Solyc01g079280.1.1	Unknown Protein (AHRD V1)	0	0	0	0	0	0	0	0	0	0	1	NA	0	0	0	0	0	0	0	0	1	NA	0	0	0	0	0	0	0	0	1	NA	0	0	0	0	0	0	0	1	NA
Solyc01g079290.1.1	Ubiquitin-conjugating enzym	0.23	0	0	0.06	0	0.18	0	0	0	0.04	0.78	1	0	0.6	0	0.2	0	0	0	0	0.05	1	0	0.19	0.23	0.14	0	0	0	0	0.07	1	0	0	0	0	0	0	0	1	NA
Solyc01g079300.2.1	Stachyose synthase (AHRD V1	0.08	0.07	0	0	0.04	0	0	0	0.05	0.01	0.33	1	0.07	0	0	0.02	0.11	0.04	0.06	0.07	3	1	0.06	0	0	0.02	0.1	0.06	0.06	0.07	3.67	1	0	0	0	0	0	0	0	1	NA
Solyc01g079310.2.1	Unknown Protein (AHRD V1)	12.79	14.64	10.35	12.92	12.68	13.81	13.42	10.59	13.12	12.73	1	1	15.3	8.1	8.95	10.78	7.86	11.26	9.48	9.53	0.88	1	6.35	9.68	7.07	7.7	3.24	5.26	5.33	4.61	0.6	1	0	1.33	0	0.44	1.84	0.8	1.32	2.98	1
Solyc01g079320.2.1	Pentatricopeptide repeat-con	7.87	6.89	8.17	9.79	8.18	6.04	7.07	6.74	4.68	6.13	0.75	0.77742	6.19	4.96	4.93	5.36	6.5	8.93	8.21	7.88	1.47	0.367857	2.3	4.54	0.67	2.5	2.59	2.16	4.65	3.13	1.25	1	5.6	3.75	5.22	4.86	6.96	7.14	7.05	1.45	0.982269
Solyc01g079330.2.1	ATP dependent RNA helicase	14.98	14.92	16.47	15.53	15.47	9.08	8.6	13.23	14.91	11.46	0.74	0.61659	17.58	14.06	17.83	16.49	18.3	17.01	18.26	17.86	1.08	1	12.04	14.91	13.69	13.55	12.75	10.84	14.14	12.58	0.93	1	24.33	17.68	18.59	20.2	15.43	19.26	17.34	0.86	1
Solyc01g079340.2.1	Receptor-like kinase (AHRD V	22.59	23.15	26.33	34.37	26.61	17.51	21.1	17.71	20.68	19.25	0.72	0.566195	26.37	23.71	29.8	26.63	18.98	18.5	19.95	19.14	0.72	0.453644	13.45	17.16	15.28	15.3	16.5	15.42	13.84	15.25	1	1	16.12	11.7	14.67	14.16	16.72	16.97	16.84	1.19	1
Solyc01g079350.2.1	PAPA-1-like conserved region	47.03	44.91	44.54	36.98	43.36	39.65	33.85	43.36	36.14	38.25	0.88	0.96538	38.3	41.84	51.9	44.01	48.3	48.83	52.52	49.88	1.13	0.910379	17.67	16.72	12.73	15.71	25.92	18.7	16.56	20.39	1.3	1	112.21	121.74	153.67	129.21	79.51	109.58	94.55	0.73	1
Solyc01g079360.2.1	WRKY transcription factor 3 (	19.16	19.98	25.19	15.88	20.05	16.74	19.23	24.41	15.13	18.88	0.94	1	17.82	11.66	6.47	11.98	11.77	13.46	19.15	14.79	1.23	0.975922	8.4	6.69	2.17	5.75	5.75	3.32	6.78	5.28	0.92	1	1.17	0.99	0.42	0.86	0.25	0.48	0.36	0.42	1
Solyc01g079370.2.1	GRAS transcription factor (Fr	0	0.07	0	0	0.02	0	0	0	0	0	0.57	1	0	0	0	0	0	0	0	0	1	NA	0.06	0	0.15	0.07	0.05	0	0.11	0.05	0.76	1	0	0	0	0	0	0	0	1	NA
Solyc01g079380.1.1	GRAS transcription factor (Fr	0	0	0	0	0	0	0	0	0	0	1	NA	0.12	0	0	0.04	0.09	0	0	0.03	0.75	1	0	0	0	0	0	0	0	0	1	NA	0	0.1	0	0.03	0	0.12	0.06	1.8	1
Solyc01g079390.2.1	Histone-lysine N-methyltrans	29.01	30.21	42.85	30.02	33.02	22.18	22.97	25.21	28.19	24.64	0.75	0.569392	30.61	34.13	41.15	35.3	32.8	35.15	31.61	33.19	0.94	1	19.07	25	18.62	20.9	21.25	16.81	21.95	20	0.96	1	70.07	42.32	46.32	52.9	37.4	42.65	40.02	0.76	1
Solyc01g079400.2.1	Os06g0207500 protein (Frag	13.15	18.52	10.77	9.12	12.89	17.32	11.15	17.46	10.66	14.15	1.1	1	7.02	7.09	9.25	7.79	8.19	9.45	11	9.55	1.23	0.830731	2.56	4.56	1.45	2.86	2.48	2.21	2.93	2.54	0.89	1	2.25	3.18	1.12	2.18	1.11	1.39	1.25	0.57	0.971976
Solyc01g079410.2.1	SMP-30/Gluconolaconase/LR	54.88	67.87	34.27	45.94	50.74	48.22	40.14	42.11	60.25	47.68	0.94	1	41.47	21.22	23.34	28.68	5.74	9.82	10.31	8.62	0.3	0.061677	0.98	0.49	0.6	0.69	0.4	0.31	0.59	0.43	0.63	1	0	0.15	0	0.05	0.18	0.18	0.18	3.6	1
Solyc01g079420.2.1	Cytochrome c oxidase subuni	379.09	445.61	250.34	250.7	331.44	522.08	433.76	668.58	429.44	513.47	1.55	0.166728	337.97	308.99	200.84	282.6	197.37	255.93	348.6	267.3	0.95	1	396.95	271.19	244.83	304.32	325.36	345.16	369.17	346.56	1.14	1	126	215.62	214.25	185.29	208.21	165.33	186.77	1.01	1
Solyc01g079430.2.1	A_IG002N01.30 protein (Frag	2.82	2.51	3.86	2.52	2.93	2.15	1.64	3.55	2.24	2.4	0.82	1	2.9	2.97	2.64	2.84	1.85	3	2.22	2.36	0.83	1	1.56	1.45	2.21	1.74	1.56	1.91	1.84	1.77	1.02	1	2.75	5.4	3.43	3.86	1.9	3.91	2.91	0.75	1
Solyc01g079440.1.1	Unknown Protein (AHRD V1)	0	0	1.14	1.37	0.63	0	0	0	0	0	0.02	1	0	0	0.82	0.27	0.67	0	0	0.22	0.82	1	0	0	0.96	0.32	0	0	0	0	0.03	NA	0	0.7	0	0.23	0.89	0	0.45	1.91	1
Solyc01g079450.2.1	Peptidase S9 prolyl oligopept	45.42	43.42	56.68	50.8	49.08	49.96	54.96	46.72	49.83	50.37	1.03	1	41.93	47.92	54.13	47.99	58.42	44.69	44.22	49.11	1.02	1	39.96	48.49	56.25	48.23	54.49	50.72	49.35	51.52	1.07	1	96.67	73.32	75.96	81.98	86.3	79.11	82.7	1.01	1
Solyc01g079460.2.1	Unknown Protein (AHRD V1)	0	0.54	0	0	0.14	0	0.95	0.28	0.43	0.41	3.07	0.986666	0	0.52	0.25	0.26	0.21	0.31	0	0.17	0.68	1	0.24	0	0	0.08	0	0	0.44	0.15	1.83	1	0	0	0	0	0	0	0	1	NA
Solyc01g079470.2.1	CP12 (AHRD V1 ***- O24136	16.36	22.08	29.89	23.89	23.05	290.98	344.58	219.82	290.62	286.5	12.43	4.29E-30	19.15	29.36	27.19	25.23	266.48	175.16	153.05	198.23	7.86	2.66E-06	3.41	9.46	7.33	6.73	11.09	7.2	48.46	22.25	3.3	1	0.75	1.53	0	0.76	0	0.46	0.23	0.3	1
Solyc01g079480.2.1	BZIP transcription factor (AH	198.37	147.39	142.78	101.04	147.39	128.96	154.81	117.13	104.85	126.44	0.86	0.916407	143.04	104.41	128.35	125.27	77.31	89.99	96.9	88.07	0.7	0.412187	195.55	159.05	183.8	179.47	147.65	140	119.02	135.56	0.76	1	401.07	134.49	185.4	240.32	210.33	248.12	229.23	0.95	1
Solyc01g079490.2.1	Protein fat-free homolog (AH	8.14	7.09	6.6	11.03	8.21	5.28	9.77	7.17	6.45	7.17	0.87	1	6.39	8.11	7.59	7.36	6.12	4.79	5.73	5.55	0.75	0.733821	3.63	5.04	5.72	4.8	4.01	3.92	3.4	3.78	0.79	1	9.52	12.13	17.99	13.21	6.21	10.56	8.38	0.63	0.943939
Solyc01g079500.2.1	DNA replication licensing fact	5.78	4.49	5.09	3.76	4.78	2.75	5.14	5.74	6.72	5.09	1.06	1	5.46	2.39	1.7	3.18	0.76	1.33	3.46	1.85	0.58	0.953391	2.67	5.53	3.19	3.8	1.21	1.81	2.21	1.74	0.46	0.201892	1.04	1.27	1.69	1.33	5.46	4.67	5.06	3.8	0.041759
Solyc01g079510.2.1	WD-40 repeat protein-like (A	9.1	10.84	11.29	9.45	10.17	8.35	9.09	9.67	9.91	9.25	0.91	1	11.85	13.86	15.53	13.75	12.04	11.47	13.02	12.18	0.89	1	9.71	9.8	9.81	9.77	8.78	9.77	9.45	9.33	0.95	1	16.42	12.84	12.83	14.03	11.33	13.63	12.48	0.89	1
Solyc01g079520.2.1	DNA mismatch repair protein	2.11	1.3	1.42	1.21	1.51	1.06	1.63	1.85	1.91	1.61	1.07	1	1.31	0.96	1.19	1.15	0.94	0.94	1.11	1	0.86	1	0.53	1.19	0.55	0.76	0.5	0.46	0.4	0.45	0.6	1	1.07	0.66	0.62	0.78	0.74	1.1	0.92	1.17	1
Solyc01g079530.2.1	E3 ubiquitin-protein ligase M	0.3	1.59	1.75	0.84	1.12	0.99	0.7	1.91	0.43	1.01	0.9	1	0.55	0.26	0.5	0.44	1.03	0.92	0.47	0.81	1.85	1	1.2	0.97	2.36	1.51	1.17	0.9	1.08	1.05	0.7	1	10.56	9.93	5.94	8.81	1.09	1.04	1.06	0.12	0.011518
Solyc01g079540.2.1	ADP-ribosylation factor-like p	222.4	241.82	196.59	203.95	216.19	212.81	205.38	227.93	216.79	215.73	1	1	204.34	189.19	145.91	179.81	121.47	139.98	172.1	144.52	0.8	0.816479	319.52	241.35	202.18	254.35	188.32	224.23	227.98	213.51	0.84	1	188.08	179.31	163.67	177.02	206.7	197.08	201.89	1.14	1
Solyc01g079550.2.1	Multidrug resistance protein	0	0.3	0.4	0.47	0.29	0	0.79	0	0	0.2	0.68	1	1.56	0.58	0.57	0.9	0	0.17	0	0.06	0.06	0.492317	0.27	0.82	0	0.36	0	0	0.24	0.08	0.22	1	0.48	0.73	0	0.4	0.61	0	0.3	0.76	1
Solyc01g079560.2.1	B3 domain-containing protei	0	0	0	0	0	0	0	0	0	0	1	NA	0	0	0	0	0	0	0	0	1	NA	0	0	0	0	0	0	0	0	1	NA	0	0	0	0	0	0	0	1	NA
Solyc01g079570.2.1	Beta xylosidase (AHRD V1 **	0.09	0	0.1	0	0.05	0.09	0.07	0.08	0.18	0.1	2.21	1	0.08	0	0	0.03	0	0.13	0.07	0.07	2.5	1	0	0	0	0	0	0	0	0	1	NA	0	0	0	0	0	0	0	1	NA
Solyc01g079580.2.1	DNAJ heat shock N-terminal	19.67	22.17	30.27	40.38	28.12	26.5	23.72	18.78	22.11	22.78	0.81	0.992657	8.65	9.11	13.82	10.53	6.53	8.36	7.31	7.4	0.7	0.580074	0.52	0.87	0.53	0.64	0.7	0.32	0.08	0.37	0.57	1	0.15	0.31	0.82	0.43	0.2	0.19	0.2	0.46	1
Solyc01g079590.2.1	Transmembrane protein 205	128.86	144.04	120.02	127.76	130.17	134.67	102.46	118.48	82.99	109.65	0.84	0.79626	109.83	129.7	143.47	127.67	101.13	106.94	113.38	107.15	0.84	0.939393	186.05	157.5	186.96	176.84	127.38	141.59	120.86	129.94	0.73	1	235.81	122.25	116.14	158.07	128.12	121.63	124.88	0.79	1
Solyc01g079600.2.1	Lipase (AHRD V1 *-*- C1FIH0	13.7	15.08	15.84	19.5	16.03	10.12	16.29	9.61	10.29	11.58	0.72	0.576735	18.86	18.57	20.63	19.35	15	15.54	13.74	14.76	0.76	0.724976	35.9	57.58	33.81	42.43	37.06	33.54	48.99	39.86	0.94	1	5.89	38.03	18.02	20.65	30.2	16.04	23.12	1.12	1
Solyc01g079610.2.1	DNAJ chaperone (AHRD V1 *	21.16	19.68	16.2	29.67	21.68	16.74	32.06	22.79	23.54	23.78	1.1	0.89692	18.62	21.71	15.3	18.54	11.51	14.71	14.68	13.63	0.74	0.71782	36.05	69.61	56.59	54.08	43.84	25.72	55.99	41.85	0.77	1	6.24	17.31	13.49	12.35	15.88	15.05	15.46	1.25	1
Solyc01g079620.2.1	Myb 12 transcription factor (	31.72	36.03	36.51	28.08	33.08	47.16	30.73	62.98	42.19	45.77	1.38	0.364896	40.73	30.28	24.82	31.94	15.66	30.13	24.62	23.47	0.73	0.906866	56.13	58.2	35.16	49.83	23.78	19.91	16.73	20.14	0.4	1	1.66	3.25	4.19	3.03	3.92	3.24	3.58	1.18	1
Solyc01g079630.2.1	Unknown Protein (AHRD V1)	4.39	5.93	6	4.22	5.13	10.34	7.48	8.71	5.79	8.08	1.57	0.359598	4.9	4.27	5.78	4.98	4.5	5.9	9.24	6.55	1.31	0.856505	6.95	7.58	5.22	6.58	4.15	4.52	5.75	4.81	0.73	1	2.99	4.46	4.86	4.1	5.78	4.15	4.96	1.21	1
Solyc01g079640.2.1	Unknown Protein (AHRD V1)	8.07	12.46	10.08	11.41	10.5	10.29	11.05	9.38	15.5	11.55	1.1	0.962949	10.82	10.73	11.22	10.92	21.23	14.25	14.55	16.68	1.53	0.294465	6.14	11.02	5.89	7.68	5.46	10.61	9.51	8.53	1.11	1	5.74	5.17	6.2	5.7	9.34	10.61	9.97	1.75	0.839299
Solyc01g079650.2.1	CTP synthase-like protein (AH	13.59	16.69	15.76	13.43	14.87	12.37	11.19	12.61	11.69	11.96	0.8	0.790747	15.37	19.25	19.89	18.17	27.23	19.23	18.42	21.63	1.19	0.819942	12.21	12.22	10.12	11.52	14.32	12.61	12.81	13.25	1.15	1	38.15	33.02	29.91	33.69	26.71	30.21	28.46	0.84	1
Solyc01g079660.2.1	Long cell-linked locus protein	0.35	0.93	0	0.99	0.57	0	0	0.64	0	0.16	0.28	0.903675	0.32	0	2.95	1.09	0	0	0	0	0.01	0.756893	0	0	10.05	3.35	0.92	0.79	0	0.57	0.17	1	60.05	21.04	27.37	36.15	33.23	24.18	28.7	0.79	1
Solyc01g079670.2.1	Long cell-linked locus protein	0	0	0	0	0	0	0	0	0	0	1	NA	0	0	0.36	0.12	0	0	0	0	0.08	1	0	0	0	0	0	0	0	0	1	NA	0	0	1.3	0.43	0	0	0	0.02	1
Solyc01g079680.2.1	Ran GTPase activating protei	17.96	16.3	18.5	20.15	18.23	13.97	11.25	13.1	14.18	13.12	0.72	0.52188	14.14	14.88	17.8	15.61	11.04	9.79	11.76	10.86	0.7	0.433962	6.48	8.98	7.01	7.49	7.94	6.44	6.98	7.12	0.95	1	11.41	11.22	12.15	11.59	8.11	11.86	9.98	0.86	1
Solyc01g079690.2.1	ATP-dependent DNA helicase	5.56	5.25	8.36	6.84	6.5	3.9	4.44	4.99	5.56	4.72	0.73	0.687917	4.87	6.05	6.99	5.97	6.71	6.34	7.03	6.69	1.12	1	4.98	4.75	6.25	5.33	6.18	5.12	5.45	5.58	1.05	1	16.01	11.16	11	12.72	8.85	7.61	8.23	0.65	0.90901
Solyc01g079700.2.1	Chromatin remodeling compl	4.77	4.64	6.14	3.68	4.81	0.96	3.43	2.39	4.36	2.79	0.58	0.794927	4.44	7.17	4.03	5.21	5.72	4.94	4.43	5.03	0.96	1	4.56	3.89	5.6	4.68	2	2.63	5.06	3.23	0.69	1	6.79	6.62	10.68	8.03	5.96	4.57	5.27	0.66	1
Solyc01g079710.2.1	Uncharacterized conserved p	53.18	51.56	59.94	34.9	49.9	41.03	30.59	33.44	43.3	37.09	0.74	0.468587	45.39	52.42	50.01	49.27	62.11	45.7	51.7	53.17	1.08	1	18.75	24.12	17.05	19.97	23.17	23.41	22.51	23.03	1.15	1	45.99	39.69	37.38	41.02	40.48	53.52	47	1.15	1
Solyc01g079720.2.1	Kinase-associated protein ph	29.46	26.71	27.59	29.02	28.2	19.38	26.65	28.91	29.7	26.16	0.93	1	32.32	39.03	40.44	37.26	53.71	51.41	43.78	49.63	1.33	0.322402	36.63	40.36	30.88	35.96	38.85	35.95	36.9	37.23	1.04	1	33.72	56.65	51.06	47.14	40.19	37.24	38.72	0.82	1
Solyc01g079730.1.1	Unknown Protein (AHRD V1)	0	0	0	0	0	0	0	0	0	0	1	NA	0	0	0	0	0	0	0	0	1	NA	0	0	0	0	0	0	0	0	1	NA	0	0	0	0	0	0	0	1	NA
Solyc01g079740.2.1	Unknown Protein (AHRD V1)	0.42	0	0.25	0.3	0.24	0	0.83	0	0.15	0.24	1.01	1	0.2	0.18	0.35	0.24	0.15	0	0	0.05	0.21	1	0	0	0	0	0	0	0	0	1	NA	0	0	0	0	0	0.18	0.09	9	1
Solyc01g079750.2.1	Protein serine/threonine kina	2.68	4.09	3.05	3.93	3.44	1.81	2.04	2.91	2.82	2.4	0.7	0.707597	2.49	2.16	2.26	2.3	1.59	2.08	2.03	1.9	0.82	1	0.77	3.04	1.33	1.71	1.01	1.16	0.98	1.05	0.61	1	0.54	0.7	0.59	0.61	0.35	0.34	0.34	0.57	1
Solyc01g079760.2.1	Mitochondrial carrier protein	19.52	20.49	22.31	20.06	20.59	21.02	18.33	23.05	17.12	19.88	0.97	1	17.4	18.04	22.74	19.39	16.68	14.58	17.44	16.23	0.84	0.972894	29.72	26.63	30.4	28.92	16.47	22.27	23.06	20.6	0.71	1	24.84	32.09	30.97	29.3	17.5	16.58	17.04	0.58	0.652613
Solyc01g079770.2.1	TLC domain-containing prote	6.67	8.8	3.25	4.55	5.82	10.73	7.98	10.99	8.42	9.53	1.64	0.365616	6.63	7.99	6.21	6.94	11.64	9.05	11.56	10.75	1.55	0.403427	15.97	14.98	6.16	12.37	9.36	10.45	13.9	11.24	0.91	1	0.98	2	2.83	1.94	2.31	2.62	2.46	1.27	1
Solyc01g079780.2.1	Uncharacterized GPI-anchore	125.85	104.78	89.81	113.41	108.46	119.59	159.45	122.87	142.82	136.18	1.26	0.440309	141.3	112.34	90.87	114.84	108.41	122.27	110.07	113.58	0.99	1	158.53	238.74	132.64	176.64	173.13	159.24	198.21	176.86	1	1	57.24	71.5	52.54	60.43	147.72	140.6	144.16	2.39	0.094412
Solyc01g079790.2.1	Glucose-1-phosphate adenyly	12.02	9.33	9.65	9.22	10.05	37.95	33.11	23.86	38	33.23	3.3	4.28E-09	7.73	11	9.61	9.45	26.16	17.54	17.88	20.53	2.17	0.010138	0.93	1.56	0.63	1.04	2.69	1.74	2.05	2.16	2.08	1	0	0.56	0.59	0.38	0.94	1.12	1.03	2.69	1
Solyc01g079800.2.1	ATP-dependent RNA helicase	3.3	4.6	2.37	1.62	2.97	4.78	3.28	2.9	5.66	4.16	1.4	0.741039	3.6	2.99	2.66	3.08	4.87	4.53	4.96	4.79	1.55	0.483493	3.13	4.44	3.42	3.66	2.26	3.8	4.39	3.48	0.95	1	2.04	2.81	3.53	2.79	3.41	4.9	4.16	1.49	1
Solyc01g079810.2.1	RING-H2 finger protein (Frag	24.41	21.4	15.39	30.58	22.95	19.68	18.1	14.22	19.93	17.98	0.78	0.826254	18.03	35.48	34.94	29.48	25.33	23.45	15.67	21.48	0.73	0.869314	5.27	11.39	16.83	11.16	12.24	11.44	7.88	10.52	0.94	1	23.85	41.55	47.28	37.56	39.24	32.19	35.72	0.95	1
Solyc01g079820.2.1	Redoxin domain protein (AHR	129.87	152.47	62.35	71.33	104.01	228.44	169.88	295.98	152.25	211.64	2.03	0.110234	105.06	89.47	51.93	82.15	73.78	114.81	149.56	112.72	1.37	0.774218	70.88	56.29	51.77	59.65	58.99	73.04	72.24	68.09	1.14	1	37.32	66.48	50.99	51.6	49.32	55.42	52.37	1.01	1
Solyc01g079830.2.1	Unknown Protein (AHRD V1)	0	0	0	0	0	0	0	0	0	0	1	NA	0	0	0	0	0	0	0	0	1	NA	0	0	0	0	0	0	0	0	1	NA	0	0	0	0	0	0	0	1	NA
Solyc01g079840.2.1	NEDD8-activating enzyme E1	40.11	41.85	33.63	37.2	38.2	44.42	37.58	32.96	38.08	38.26	1	1	41.96	39.08	33.01	38.02	43.51	44.58	43.18	43.76	1.15	0.832635	43.33	45.02	44.19	44.18	41.41	47.66	46.9	45.32	1.03	1	62.04	77.16	64.17	67.79	59.18	61.97	60.58	0.89	1
Solyc01g079850.2.1	Os06g0207500 protein (Frag	0	0	0	0.62	0.15	0.16	0.35	0.27	0.1	0.22	1.42	1	0.14	0.13	0.25	0.17	0.2	0.08	0.11	0.13	0.75	1	0.83	0.95	0.58	0.79	0.77	0.88	0.53	0.73	0.92	1	0	0.11	0.22	0.11	0.13	0.51	0.32	2.91	1
Solyc01g079860.2.1	Carbohydrate kinase YjeF rela	32.88	39.99	30.36	28.89	33.03	34.74	33.54	42.82	38.42	37.38	1.13	0.916246	38.19	41.72	39.5	39.8	41.65	44.42	37.72	41.26	1.04	1	82.01	74.73	62.04	72.93	70.71	72.6	67.42	70.24	0.96	1	44.72	57.11	51.19	51.01	60.95	52.29	56.62	1.11	1
Solyc01g079870.2.1	Nuclear transcription factor Y	208.61	187.02	237.7	178.34	202.92	156.57	137.35	151.06	172.63	154.4	0.76	0.556586	196.42	228.95	231.49	218.95	229.49	173.82	178.35	193.89	0.89	1	239.74	280	246.76	255.5	271.24	296.53	266.54	278.1	1.09	1	415.75	317.83	265.17	332.92	296.36	311.1	303.73	0.91	1
Solyc01g079880.2.1	Asparagine synthetase (AHRD	777.74	912.91	1115.26	496.11	825.5	1345.97	706.74	1017.59	914.67	996.24	1.21	0.919033	1159.46	871.34	812.07	947.62	999.88	1019.54	850.74	956.72	1.01	1	358.92	413.73	325.48	366.04	538.9	573.65	390.47	501.01	1.37	1	4999.77	1411.98	2077.73	2829.83	1785.21	1737.72	1761.47	0.62	1
Solyc01g079890.2.1	Aquaporin (AHRD V1 ***- B3	0	0	0	0	0	0	0	0	0	0	1	NA	0	0	0	0	0	0	0	0	1	NA	0	0	0	0	0	0	0	0	1	NA	0	0	0	0	0	0	0	1	NA
Solyc01g079900.1.1	Xylanase inhibitor (Fragment	0	0	0	0	0	0	0	0	0	0	1	NA	0	0	0	0	0	0	0	0	1	NA	0	0	0	0	0	0	0	0	1	NA	0	0	0	0	0	0	0	1	NA
Solyc01g079910.1.1	Xyloglucan specific endogluca	0	0	0	0	0	0	0	0	0	0	1	NA	0	0	0	0	0	0	0	0	1	NA	0	0	0	0	0	0	0	0	1	NA	0	0	0	0	0	0	0	1	NA
Solyc01g079920.2.1	Xylanase inhibitor (Fragment	0	0	0	0	0	0	0	0	0	0	1	NA	0	0	0	0	0	0	0	0	1	NA	0	0	0	0	0	0	0	0	1	NA	0	0	0	0	0	0	0	1	NA
Solyc01g079930.1.1	Xylanase inhibitor (Fragment	0	0	0	0.22	0.06	0	0	0	0	0	0.18	1	0	0	0	0	0	0	0	0	1	NA	0	0	0	0	0	0	0	0	1	NA	0	0	0	0	0	0	0	1	NA
Solyc01g079940.2.1	Xylanase inhibitor (Fragment	0	0.14	0.18	0.43	0.19	0.17	0.24	0	0.11	0.13	0.69	1	0.14	0	0.13	0.09	0.11	0	0	0.04	0.41	1	0	0	0.3	0.1	0	0	0	0	0.1	NA	4.57	0.22	0.94	1.91	0	0	0	0.01	0.885103
Solyc01g079950.2.1	Xylanase inhibitor (Fragment	0.62	1.51	1.09	2.83	1.51	1.2	1.09	0.71	0.55	0.89	0.59	0.85	1.57	1.07	1.69	1.44	0.32	0.72	1.33	0.79	0.55	0.780439	0.12	0	0	0.04	0.4	0.35	0	0.25	6.25	1	8.31	0.56	3.31	4.06	2.67	5.12	3.9	0.96	1
Solyc01g079960.2.1	Xylanase inhibitor (Fragment	0	0	0	0	0	0	0	0	0	0	1	NA	0	0	0	0	0	0	0	0	1	NA	0	0	0	0	0	0	0	0	1	NA	0.21	0	0	0.07	0	0	0	0.14	1
Solyc01g079970.2.1	Xylanase inhibitor (Fragment	0	0	0	0	0	0	0	0	0	0	1	NA	0	0	0	0	0	0	0	0	1	NA	0	0	0	0	0	0	0	0	1	NA	0	0	0	0	0	0	0	1	NA
Solyc01g079980.2.1	Xylanase inhibitor (Fragment	0	0	0	0	0	0	0	0.14	0	0.04	3.5	1	0	0	0	0	0	0	0	0	1	NA	0	0	0	0	0	0	0	0	1	NA	0	0	0	0	0	0	0	1	NA
Solyc01g079990.1.1	Xylanase inhibitor (Fragment	0	0	0	0	0	0	0	0	0	0	1	NA	0	0	0	0	0	0	0	0	1	NA	0	0	0	0	0	0	0	0	1	NA	0	0	0	0	0	0	0	1	NA
Solyc01g080000.1.1	Xylanase inhibitor (Fragment	0	0.26	0.17	0	0.11	0.33	0.23	0	0	0.14	1.3	1	0	0.13	0.12	0.08	0.1	0.08	0	0.06	0.72	1	0	0	0	0	0	0	0	0	1	NA	0	0	0	0	0	0	0	1	NA
Solyc01g080010.2.1	Xylanase inhibitor (Fragment	12.47	11.1	8.86	19.56	13	25.89	31.91	19.48	17.8	23.77	1.83	0.077878	23.48	13.72	9.14	15.45	14.38	22.56	17.83	18.26	1.18	0.973367	10.93	15.43	9.99	12.12	5.23	7.75	8.65	7.21	0.6	1	10.68	0.11	1.16	3.98	0.69	0.53	0.61	0.15	1
Solyc01g080020.2.1	Xylanase inhibitor (Fragment	31.88	52.81	36.5	36.39	39.39	61.4	39.12	30.47	28.89	39.97	1.01	1	13.51	14.68	11.46	13.22	23.64	22.47	31.38	25.83	1.95	0.005417	6.5	9.9	3.13	6.51	3.73	3.11	6.44	4.43	0.68	1	32.54	5.27	7.52	15.11	1.16	2.35	1.75	0.12	0.98046
Solyc01g080030.1.1	Oxidoreductase zinc-binding	0	0	0	0	0	0	0	0	0	0	1	NA	0	0	0	0	0	0	0	0	1	NA	0	0	0	0	0	0	0	0	1	NA	0.35	0	0	0.12	0	0	0	0.09	1
Solyc01g080040.2.1	Dual specificity protein phosp	13.19	20.22	16	22.81	18.05	24.44	23.44	23.59	16.05	21.88	1.21	0.7992	15	17.84	12.69	15.18	14.74	19.36	18.72	17.61	1.16	0.962977	21.62	23.89	21.46	22.32	12.04	18.33	20.3	16.89	0.76	1	8.86	9.58	5.07	7.84	11.41	7.39	9.4	1.2	1
Solyc01g080050.2.1	Transcription factor (Fragmen	0	0	0	0	0	0	0	0	0	0	1	NA	0	0	0	0	0	0	0	0	1	NA	0	0	0	0	0	0	0	0	1	NA	0	0	0	0	0	0	0	1	NA
Solyc01g080060.1.1	Pol polyprotein (AHRD V1 ***	0	0	0	0	0	0	0	0	0	0	1	NA	0	0	0	0	0	0	0	0	1	NA	0	0	0	0	0	0	0	0	1	NA	0	0	0	0	0	0	0	1	NA
Solyc01g080070.2.1	Copper chaperone (AHRD V1	55.91	46.86	53.35	73.38	57.38	19.11	37.66	24.78	43.92	31.37	0.55	0.152329	48.69	41.33	48.13	46.05	31.69	41.45	33.83	35.66	0.77	0.789291	1.5	8.08	1.38	3.65	5.09	1.76	5.64	4.16	1.14	1	0.88	0.56	1.19	0.88	0.99	2.85	1.92	2.19	0.911074
Solyc01g080080.2.1	RING finger protein B (AHRD	42.44	59.46	58.99	36.85	49.44	61.19	40.14	54.86	45.25	50.36	1.02	1	60.58	58.08	50.75	56.47	64.7	57.44	65.05	62.4	1.1	0.9964	38.36	38.35	48.8	41.84	35.33	46.18	43.17	41.56	0.99	1	64.52	56.77	64.62	61.97	45	47.53	46.27	0.75	1
Solyc01g080090.2.1	Zinc finger RING-type protein	21.1	16.04	23.08	11.39	17.9	22.6	12.11	19.04	13.77	16.88	0.94	1	19.62	22.67	23.65	21.98	32.45	19.06	21.7	24.4	1.11	1	14.25	16.56	15.61	15.47	18.9	18.31	17.61	18.27	1.18	1	26.18	26.19	37.16	29.84	23.52	22.87	23.2	0.78	1
Solyc01g080100.2.1	Unknown Protein (AHRD V1)	0.87	0	0	0	0.22	0.49	0.69	2.01	0.63	0.95	4.39	0.77456	1.22	0.76	0.37	0.78	0	0	0	0	0.01	0.569875	0	0	0	0	0	0	0	0	1	NA	0	0	0	0	0	0.38	0.19	19	1
Solyc01g080110.2.1	SNARE associated Golgi prote	30.95	38	24.06	35.97	32.25	36.8	40.72	38.38	33.41	37.33	1.16	0.829736	27.22	35.24	28.16	30.21	23.79	26.7	33.56	28.02	0.93	1	31.2	28.54	31.19	30.31	25.69	25.64	26.4	25.91	0.85	1	19.1	31.01	34.31	28.14	25.25	27.97	26.61	0.95	1
Solyc01g080120.2.1	Unknown Protein (AHRD V1)	0.23	0.1	0.14	0.16	0.16	0	0	0	0	0	0.06	0.630555	0	0	0	0	0	0	0.18	0.06	6	1	0	0	0	0	0	0	0	0	1	NA	0	0	0	0	0	0	0	1	NA
Solyc01g080130.2.1	Unknown Protein (AHRD V1)	13.28	18.78	15.67	17.57	16.33	14.52	14.04	16.44	18.45	15.86	0.97	1	13.05	13.03	14.2	13.43	8.25	11.87	12.71	10.94	0.82	0.942579	1.73	2.44	1.17	1.78	1.83	1.87	1.79	1.83	1.03	1	2.59	3.5	3.29	3.13	4.31	8.92	6.62	2.12	0.545288
Solyc01g080140.2.1	Dolichol-phosphate mannosy	18.27	19.34	12.48	15.51	16.4	17.96	20.2	21.93	17.12	19.3	1.18	0.855706	17.89	17.22	13.81	16.31	12.01	14.24	15.72	13.99	0.86	1	27.81	24.45	23.1	25.12	18.45	25.07	28.03	23.85	0.95	1	11.49	17.46	14.2	14.38	20.6	15.85	18.23	1.27	1
Solyc01g080150.2.1	tRNA dimethylallyltransferas	1.2	3.74	2.36	0.85	2.04	1.33	1.26	3.68	3.73	2.5	1.23	1	4.83	3.82	1.69	3.45	0.69	4.14	5.03	3.29	0.95	1	1.78	1.95	0.2	1.31	2.36	2.42	0	1.59	1.22	1	0.28	0.73	2.46	1.16	1.1	0.35	0.73	0.63	1

Solyc01g080160.2.1	Cbs domain containing protei	11.17	10.64	7.6	4.83	8.56	13.29	12.35	7.85	12.93	11.61	1.36	0.6064	11.37	8.8	7.1	9.09	11.03	9.66	10.53	10.41	1.14	0.955848	4.99	7.39	4.44	5.61	6.82	3.88	6.8	5.83	1.04	1	2.62	6.11	7.48	5.4	3.73	6.45	5.09	0.94	1
Solyc01g080170.2.1	Hydroxymethylglutaryl-CoA l	14.42	20.36	21.08	17.29	18.29	24.39	20.04	20.6	19.57	21.15	1.16	0.851697	18.66	21.93	22.9	21.16	24.45	21.66	26.6	24.24	1.15	0.94937	9.87	10.47	7.47	9.27	6.9	8.79	8.22	7.97	0.86	1	13.59	15.36	9.88	12.94	7.46	9.07	8.27	0.64	0.850663
Solyc01g080180.2.1	Structural constituent of ribo	14.69	9.87	9.81	10.98	11.34	10.78	12.69	9.17	8.36	10.25	0.9	1	13.67	12.29	10.77	12.24	11.34	12.2	10.67	11.4	0.93	1	11.65	15.59	11.28	12.84	20.76	12.19	16.16	16.37	1.27	1	18.52	7.85	10.23	12.2	14.46	16.53	15.5	1.27	1
Solyc01g080190.2.1	Pentatricopeptide repeat-con	3.39	3.1	3.44	4.66	3.65	3.14	4.61	4.59	3.85	4.05	1.11	0.930443	4.38	2.95	2.78	3.37	3.92	4.48	4.29	4.23	1.26	0.815134	3.04	3.76	3.92	3.57	3.59	3.35	5.21	4.05	1.13	1	1.47	3.55	6.22	3.75	4.39	3.14	3.76	1	1
Solyc01g080200.2.1	At2g23590-like protein (Frag	4.35	5.34	8.08	8.21	6.5	5.5	4.5	4.69	4.02	4.68	0.72	0.638971	4.93	6.61	7.48	6.34	11.55	6.01	8.53	8.7	1.37	0.770898	1.33	1.55	0.65	1.18	0.43	0.53	0.62	0.53	0.45	1	1.71	0.8	0.85	1.12	0.32	0.7	0.51	0.46	0.989607
Solyc01g080210.2.1	Ribonucleoside-diphosphate	3.29	2.93	2.05	2.19	2.61	7.31	4.43	5.34	3.47	5.14	1.96	0.238864	2.88	4.88	3.59	3.78	5.64	4.71	6.23	5.53	1.46	0.699602	32.51	20.34	23.62	25.49	10.93	17.89	18.75	15.86	0.62	1	6.05	8.29	6.54	6.96	5.84	4.24	5.04	0.72	1
Solyc01g080220.2.1	Dienelactone hydrolase famil	2.76	1.72	2.93	0.39	1.95	4.3	5.01	7.11	4.16	5.14	2.64	0.093547	2.82	2.16	1.87	2.28	36.38	19.87	18	24.75	10.84	0.000374	46.83	48.36	32.06	42.42	79.6	66.74	118.48	88.27	2.08	0.017393	8.63	20.45	6.37	11.82	48.51	28.07	38.29	3.24	0.474925
Solyc01g080230.2.1	Dienelactone hydrolase famil	0	0	0	0	0	0	0	0	0	0	1	NA	0	0	0	0	0	0	0	0	1	NA	0	0	0	0	0	0	0	0	1	NA	0	0	0	0	0	0	0	1	NA
Solyc01g080240.2.1	Mitogen-activated protein ki	36.37	44.29	41.12	44.68	41.62	40.42	49.97	41.79	41.42	43.4	1.04	1	39.03	39.21	41.35	39.86	40.82	39.65	40.85	40.44	1.01	1	40.96	48.82	38.87	42.88	39.74	33.57	36.95	36.75	0.86	1	35.8	29.71	29.93	31.81	26.99	32.19	29.59	0.93	1
Solyc01g080250.2.1	BTB/POZ domain containing	52.07	50.51	64.48	62.9	57.49	47.99	44.81	40.73	50.5	46.01	0.8	0.81903	59.41	61.99	63.78	61.73	59.1	60.7	57.82	59.21	0.96	1	94.06	71.94	86.19	84.06	84.23	65.2	77.77	75.73	0.9	1	58.62	36.17	35.56	43.45	51.66	55.57	53.61	1.23	1
Solyc01g080260.2.1	At4g14280-like protein (Frag	0	0.07	0.28	0	0.09	0.09	0	0.15	0.11	0.09	1	1	0.15	0.28	0.07	0.17	0.39	0	0	0.13	0.78	1	1.35	1.04	0.32	0.9	0.42	0.06	0.46	0.31	0.35	1	0.11	0.17	0.12	0.13	0.29	0.28	0.29	2.14	1
Solyc01g080270.2.1	Alpha/beta hydrolase (AHRD	22.61	23.73	27.49	19.68	23.38	26.68	20.69	23.88	22.14	23.35	1	1	25.13	27.21	24.09	25.48	24.53	28.21	26.51	26.42	1.04	1	31.47	30.71	31.3	31.16	25.82	27.24	26.61	26.56	0.85	1	47.31	35.03	36.91	39.75	32.37	29.07	30.72	0.77	1
Solyc01g080280.2.1	Glutamine synthetase (AHRD	66.28	52.61	50.87	44.4	53.54	154.79	142.3	102.82	133.99	133.48	2.49	2.78E-07	21.82	23.57	35	26.8	47.76	30.69	25.5	34.65	1.29	0.88712	5.79	9.81	12.85	9.48	11.81	4.27	8.77	8.28	0.87	1	0.65	1.44	1.64	1.24	0.14	0	0.07	0.06	0.290714
Solyc01g080290.2.1	Plasma membrane associated	2	2.14	0.94	0.57	1.41	2.22	1.58	1.47	3.44	2.18	1.54	0.964813	2.23	2.09	1.69	2	3.61	0.62	1.26	1.83	0.91	1	0	0.65	0	0.22	0.79	0.61	0.29	0.56	2.6	1	0	0	0	0	0	0	0	1	NA
Solyc01g080300.2.1	Cobalt transport protein (AH	10.77	9.13	12.3	9.43	10.41	15.2	18.35	12.89	13.74	15.04	1.45	0.272758	11.29	12.03	9.67	11	16.48	15.84	12.49	14.94	1.36	0.46798	17.67	17.13	18.49	17.76	19.54	18.78	23.79	20.7	1.17	1	3.89	6.08	4.73	4.9	9.07	6.44	7.76	1.58	1
Solyc01g080310.2.1	Pentatricopeptide repeat-con	5.27	7.35	5.4	5.63	5.91	8.74	7.38	7.87	5.29	7.32	1.24	0.804781	5.09	4.67	4.45	4.74	5.38	7.37	5.94	6.23	1.32	0.611025	5.63	5.03	6.62	5.76	4.44	5.73	6.53	5.57	0.97	1	2.69	5.93	4.9	4.51	3.37	1.57	2.47	0.55	0.718052
Solyc01g080320.1.1	Unknown Protein (AHRD V1)	0	0	1.19	0	0.3	0	0	0	0	0	0.03	1	0	0	0	0	0	0	0	0	1	NA	0	0	0	0	0	0	0	0	1	NA	0	0	0	0	0	0	0	1	NA
Solyc01g080330.2.1	Transmembrane BAX inhibito	382.74	421.22	294	312.44	352.6	369.33	361.84	358.53	347.64	359.33	1.02	1	390.8	444.15	294.85	376.6	322.7	343.23	367.74	344.56	0.91	1	402	415.26	322.11	379.79	328.99	363.98	429.55	374.17	0.99	1	246.08	381.57	283.39	303.68	300.49	294.6	297.55	0.98	1
Solyc01g080340.2.1	BSD domain containing 1 (AH	25.3	27.08	26.19	20.86	24.86	26.57	23.04	31.11	29.98	27.68	1.11	0.931315	24.83	29.15	22.94	25.64	22.18	27.25	23.06	24.16	0.94	1	14.8	15.94	10.79	13.84	17.95	13.41	15.16	15.51	1.12	1	49.44	31.79	34.13	38.45	27.85	33.34	30.6	0.8	1
Solyc01g080350.2.1	Calcium binding protein 39-li	12.77	20.49	14.15	13.68	15.27	21.72	15.58	22.41	14.86	18.64	1.22	0.83242	15.84	14.13	12.26	14.08	9.8	14.82	15.05	13.22	0.94	1	17.51	15.92	13.44	15.62	9.28	8.65	15.79	11.24	0.72	1	3.03	7.87	8.92	6.61	8.85	6.62	7.73	1.17	1
Solyc01g080360.2.1	MHD domain-containing dea	11.59	12.66	16.04	15.8	14.02	12.53	12.73	12.31	12.03	12.4	0.88	1	10	14.66	20.12	14.93	22.44	16.92	18.8	19.39	1.3	0.70102	11.49	10.99	11.61	11.36	11.08	12.54	13.82	12.48	1.1	1	16.18	13.17	17.34	15.56	16.88	19.68	18.28	1.17	1
Solyc01g080370.2.1	Glycosyltransferase (AHRD V	0	0	0	0	0	0.19	0	0	0	0.05	4.75	1	0	0	0	0	0	0	0	0	1	NA	0	0	0	0	0	0	0	0	1	1	0	0	0	0	0	0	0	1	NA
Solyc01g080380.2.1	S-adenosylmethionine decarb	0	0	0	0.28	0.07	0.22	0.16	0	0.14	0.13	1.86	1	0	0	0	0	0	0	0	0	1	NA	0	0	0	0	0	0	0	0	1	NA	0.28	0	0	0.09	0	0.17	0.09	0.91	1
Solyc01g080390.1.1	Mutator-like transposase (AH	0	0	0	0	0	0	0.09	0	0	0.02	2.25	1	0	0	0	0	0	0.06	0.09	0.05	5	1	0	0	0	0	0	0	0	0	1	1	0	0	0	0	0	0	0	1	NA
Solyc01g080400.2.1	Integrin-linked kinase-associa	12.88	17.26	15.38	13.72	14.81	15.55	15.19	17.25	18.72	16.68	1.13	0.916407	14.79	18.79	15.57	16.38	17.86	16.63	18.49	17.66	1.08	1	14	13.21	14.99	14.07	9.09	8.43	12.05	9.86	0.7	1	41.59	16.74	14.91	24.41	14.7	13.24	13.97	0.57	1
Solyc01g080410.2.1	Peptide methionine sulfoxide	12.13	13.82	13.16	17.15	14.06	17.25	19.15	17.84	11.14	16.34	1.16	0.942294	17.6	9.28	5.33	10.74	5.05	4.52	2.67	4.08	0.38	0.577539	789.68	301.2	1323.61	804.83	535.99	1526.88	853.31	972.06	1.21	1	1339.37	1878.55	1618.67	1612.2	1828.14	639.25	1233.7	0.77	1
Solyc01g080420.1.1	Unknown Protein (AHRD V1)	0	0	0	0	0	0	0	2.02	0	0.51	50.5	1	2.05	0	0	0.68	0	3.42	0	1.14	1.67	1	0	0	0	0	0	0	0	0	1	1	0	0	0	0	0	0	0	1	NA
Solyc01g080430.2.1	Baculoviral IAP repeat-contai	28.83	33.19	37.28	40.13	34.86	22.56	21.67	24.81	26.66	23.93	0.69	0.322483	35.12	37.36	43.58	38.69	37.01	41.24	41.87	40.04	1.03	1	39.45	43.92	38.92	40.76	43.14	33.1	41.41	39.22	0.96	1	88.33	71.48	78.75	79.52	52.37	56.29	54.33	0.68	0.938102
Solyc01g080440.1.1	Unknown Protein (AHRD V1)	0	0	0	0	0	0	0	0	0	0	1	NA	0	0	0	0	0	0	0	0	1	NA	0	0	0	0	0	0	0	0	1	NA	0	0	0	0	0	0	0	1	NA
Solyc01g080450.2.1	Pentatricopeptide repeat-con	4.31	5.22	2.26	4.74	4.13	3.06	4.06	3.96	4.21	3.82	0.92	1	4.34	3.75	3.34	3.81	2.99	4.09	6.12	4.4	1.15	1	4.64	4.78	3.21	4.21	3.22	2.72	4.97	3.64	0.86	1	1.19	2.26	1.66	1.7	1.53	1.57	1.55	0.91	1
Solyc01g080460.2.1	Pyruvate phosphate dikinase	282.19	269.62	283.21	218.8	263.45	319.75	246.13	313.14	397.48	319.12	1.21	0.561646	250.73	236.39	314.94	267.35	278.18	288.78	267.49	278.15	1.04	1	630.2	562.23	471.12	554.52	513.96	450.53	409.47	457.99	0.83	1	62.24	145.93	120.98	109.72	312.89	190.83	251.86	2.3	0.531734
Solyc01g080470.2.1	tRNA (Guanosine-2&apos-O)-	20.5	20.89	30.15	37.41	27.24	22.79	15.72	27.6	17.07	20.8	0.76	0.844754	20.46	24.54	31.63	25.54	31.12	25.7	21.72	26.18	1.02	1	17.29	22.52	18.29	19.37	21.67	18.02	19.49	19.73	1.02	1	68.45	41.01	42.04	50.5	32.37	46.12	39.24	0.78	1
Solyc01g080480.2.1	Endonuclease/exonuclease/p	0	0	0	0.38	0.1	0	0	0	0	0	0.11	1	0	0	0.23	0.08	0.09	0.07	0	0.05	0.7	1	0	0	0	0	0	0	0	0	1	NA	0	0	0	0	0	0	0	1	NA
Solyc01g080490.2.1	Genomic DNA chromosome 5	0	0	0.89	0	0.22	0.42	0.3	0.35	0	0.27	1.2	1	0.7	0	0.32	0.34	0	0.2	0	0.07	0.2	1	0.61	0	0	0.2	0	0.29	0	0.1	0.48	1	0.54	0.55	0	0.36	0	0.33	0.17	0.45	1
Solyc01g080500.2.1	Unknown Protein (AHRD V1)	174.37	180.83	229.48	300.98	221.42	184.19	139.87	215.29	128.52	166.97	0.75	0.73765	145.49	277.31	302.15	241.65	165.41	184.42	153.34	167.72	0.69	0.708495	72.32	106.76	139.26	106.11	115.98	76.29	69.06	87.11	0.82	1	489.68	693.76	839.2	674.21	225.97	267.22	246.6	0.37	0.017246
Solyc01g080510.2.1	Os05g0406000 protein (Frag	23.39	21.17	26.89	21.21	23.16	34.44	32.35	34.74	27.41	32.23	1.39	0.293053	21.2	23.46	29.72	24.79	35.36	23.7	29.85	29.64	1.2	0.859279	16.64	20.69	11.51	16.28	15.1	18.18	15.15	16.14	0.99	1	22.45	18.88	17.22	19.52	12.08	11.74	11.91	0.61	0.848276
Solyc01g080520.2.1	Serine/threonine protein kina	0	0	0	0	0	0	0	0	0	0	1	NA	0	0	0	0	0	0	0.11	0.04	3.67	1	0	0	0	0	0	0	0	0	1	NA	0	0	0	0	0	0	0	1	NA
Solyc01g080530.2.1	Prolyl 4-hydroxylase alpha su	36.55	39.27	32.87	35.31	36	28.74	25.16	30.33	29.28	28.38	0.79	0.717618	33.58	34.87	37.27	35.24	24.89	38.31	32.4	31.87	0.9	1	15.31	25.24	11.06	17.2	17.57	15.2	18.19	16.99	0.99	1	4.12	9.71	8.23	7.35	12.65	11.92	12.29	1.67	0.977797
Solyc01g080540.2.1	Histidine-containing phospho	20.83	20.47	23.04	12.89	19.31	20.75	13.02	21.15	20.57	18.87	0.98	1	29.07	30.94	10.63	23.55	24.99	24.94	35.13	28.35	1.2	0.995707	5.26	10.26	3.88	6.47	2.85	4.93	6.96	4.91	0.76	1	19.75	6.62	16.02	14.13	2.78	6.85	4.81	0.34	0.854307
Solyc01g080550.1.1	Inosine-uridine preferring nu	0	0	0	0	0	0	0	0	0	0	1	NA	0	0	0	0	0	0	0	0	1	NA	0	0	0	0	0	0	0	0	1	NA	0	0	0	0	0	0	0	1	NA
Solyc01g080560.1.1	Ribonucleoside hydrolase 1 (	0	0	0	0	0	0	0	0	0	0	1	NA	0	0	0	0	0	0	0	0	1	NA	0	0	0	0	0	0	0	0	1	NA	0	0.1	0	0.03	0	0	0	0.3	1
Solyc01g080570.2.1	Inosine-uridine preferring nu	0	0	0	0	0	0	0	0	0	0	1	NA	0	0	0	0	0	0	0	0	1	NA	0	0	0	0	0	0	0	0	1	NA	0	0	0	0	0	0	0	1	NA
Solyc01g080580.2.1	Inosine-uridine preferring nu	0.41	0.36	0.19	0	0.24	0	0.06	0.15	0.12	0.08	0.34	0.738544	0.3	0.21	0.41	0.31	0.06	0.08	0.19	0.11	0.36	0.792457	0.07	0	0.73	0.27	0.21	0.06	0	0.09	0.34	1	0.23	0.3	0.25	0.26	0	0.07	0.04	0.13	1
Solyc01g080590.2.1	Unknown Protein (AHRD V1)	59.9	78.96	82.31	70.79	72.99	98.62	123.32	109.46	67.5	99.72	1.37	0.370783	67.71	98.87	73.65	80.08	82.57	85.2	67.93	78.57	0.98	1	139.68	130.38	95.59	121.88	89.17	112.07	81.23	94.16	0.77	1	48.52	142.64	125.93	105.7	85.38	73.13	79.25	0.75	1
Solyc01g080600.2.1	Histone H3 (AHRD V1 ***- D	12.04	10.71	5.68	17.03	11.37	6.42	3.04	7.08	6.91	5.86	0.52	0.356898	9.41	12.14	12.2	11.25	6.01	7.98	4.16	6.05	0.54	0.499817	3.5	7.06	0.96	3.84	1.27	0	1.4	0.89	0.23	1	1.37	0.35	0	0.57	3.97	3.38	3.67	6.41	0.624572
Solyc01g080610.2.1	Unknown Protein (AHRD V1);	0	0	0	0.44	0.11	0.17	0.25	0	0	0.11	0.95	1	0.14	0.27	0.39	0.27	0.32	0.08	0.24	0.21	0.8	1	0.38	0.63	0	0.34	0.1	0.24	0	0.11	0.34	1	0	0	0.48	0.16	0.57	0	0.28	1.78	1
Solyc01g080620.2.1	Kelch-like protein 3 (AHRD V	9.14	8.79	11.39	11.66	10.25	8.34	6.16	5.94	8.05	7.12	0.7	0.459044	9.47	9.88	12.86	10.74	14.02	10.23	10.16	11.47	1.07	1	8.64	9.26	11.5	9.8	6.64	7.77	7.36	7.26	0.74	1	34.96	14.46	17.48	22.3	15.78	17.2	16.49	0.74	1
Solyc01g080630.1.1	Unknown Protein (AHRD V1)	0	0	0	0	0	0	0	0	0	0	1	NA	0	0	0	0	0	0	0	0	1	NA	0	0	0	0	0	0	0	0	1	NA	0	0	0	0	0	0	0	1	NA
Solyc01g080640.2.1	Multidrug resistance protein	18.57	27.1	46.42	37.72	32.45	17.22	16.05	19.1	23.95	19.08	0.59	0.455124	37.17	34.01	34.98	35.39	21.7	24.7	20.27	22.22	0.63	0.107731	57.39	45.72	74.57	59.23	53.43	54.77	45.12	51.11	0.86	1	63.89	89.34	81.28	78.17	71.47	58.34	64.91	0.83	1
Solyc01g080650.2.1	Unknown Protein (AHRD V1)	53.46	59.73	49.65	59.53	55.59	41.72	44	94.12	54.71	58.64	1.05	1	49.73	47.46	40.35	45.85	34.71	49.47	57.21	47.13	1.03	1	40.43	49.12	46.29	45.28	20.92	46.55	29.84	32.44	0.72	1	97.03	59.48	99.7	85.4	55.18	64.82	60	0.7	1
Solyc01g080660.2.1	Splicing factor arginine/serin	16.59	12.66	14.91	20.67	16.21	10.1	11.23	17.8	12.76	12.97	0.8	0.982081	13.96	13.05	16.02	14.34	15.34	14.11	14.59	14.68	1.02	1	12.13	15.13	11.37	12.88	8.56	9.88	10.36	9.6	0.75	1	26.4	12.62	23.08	20.7	14.46	14.2	14.33	0.69	1
Solyc01g080670.2.1	Squamosa promoter binding	27.57	29.7	36.1	32.99	31.59	26.98	20.46	30.77	23.77	25.49	0.81	0.77456	22.9	31.8	36.02	30.24	41.25	33.71	33.65	36.2	1.2	0.751893	16.67	17.52	15.66	16.62	18.39	14.59	16.55	16.51	0.99	1	52.38	38.47	46.26	45.7	26.98	35.65	31.31	0.69	0.986172
Solyc01g080680.2.1	Glucose transporter 8 (AHRD	11.53	21.78	16.26	4.38	13.49	24.86	3.55	10.73	11.01	12.54	0.93	1	4.71	1.35	2.61	2.89	0.39	1.09	1.33	0.94	0.32	0.469779	0.68	1.15	1.4	1.08	0.09	0	0.51	0.2	0.19	1	0	0	0	0	0	0	0	1	NA
Solyc01g080690.2.1	Transcription initiation factor	26.39	24.86	27.32	26.01	26.14	28.55	21.94	24.78	28.42	25.92	0.99	1	31.47	33.07	25.96	30.17	32.52	33.65	31.55	32.57	1.08	1	46.27	46.09	40.82	44.39	31.44	45.75	40.81	39.33	0.89	1	27.94	31.4	28.06	29.13	36.88	30.89	33.89	1.16	1
Solyc01g080700.2.1	Inosine-uridine preferring nu	6.91	7.54	5.53	6.4	6.6	12.16	13.25	11.12	9.86	11.6	1.76	0.027458	6.73	6.79	8.2	7.24	8.39	8.6	9.76	8.92	1.23	0.77615	1.93	3.22	1.47	2.21	2.32	2.56	3.24	2.71	1.23	1	0.35	0.66	0.38	0.46	1.13	2.31	1.72	3.71	0.44125
Solyc01g080710.1.1	Ycf2 (AHRD V1 ***- D2KLP5_	0	0	0	0	0	0	0	0	0	0	1	NA	0	0	0	0	0	0	0	0	1	NA	0	0	0	0	0	0	0	0	1	NA	0	0	0	0	0	0	0	1	NA
Solyc01g080720.2.1	Pentatricopeptide repeat-con	1.35	1.08	1.76	1.91	1.53	0.71	1.4	1.5	1.69	1.32	0.87	1	2.18	1.73	1.8	1.9	1.53	1.51	2.4	1.81	0.95	1	0.86	1.28	0.71	0.95	1.03	1.08	1.45	1.19	1.25	1	2.23	2.89	2.63	2.58	2.54	2.5	2.52	0.98	1
Solyc01g080740.1.1	Ulp1 protease family C-termi	0	0	0	0	0	0	0	0	0	0	1	NA	0	0	0	0	0	0	0	0	1	NA	0	0	0	0	0	0	0	0	1	NA	0	0	0	0	0	0	0	1	NA
Solyc01g080750.2.1	HEAT repeat family protein (	1.55	2.17	1.83	1.26	1.7	2.22	1.75	0.61	1.12	1.43	0.84	1	0.83	0.19	0.38	0.47	1.85	0.46	0.35	0.89	1.9	1	0	0	0	0	0.15	0	0.32	0.16	15.67	1	0	0	0	0	0	0	0	1	NA
Solyc01g080760.2.1	Unknown Protein (AHRD V1)	6.64	6.7	7.6	5.46	6.6	7.49	8.05	10.84	7.61	8.5	1.29	0.604664	6.73	7.41	5.77	6.64	8.86	9.03	9.11	9	1.36	0.495242	4.24	6.3	6.98	5.84	9.24	6.68	7.87	7.93	1.36	1	12.82	21.51	15.99	16.77	16.78	15.11	15.95	0.95	1
Solyc01g080770.2.1	Receptor like kinase, RLK	3.43	5.38	3.48	2.14	3.61	2.29	2.06	2.34	1.58	2.07	0.57	0.153936	2.81	3.28	4.75	3.61	2.67	1.78	2.16	2.2	0.61	0.30707	0.94	1.45	1.36	1.25	1.13	0.88	1.1	1.04	0.83	1	1.07	1.84	2.01	1.64	0.94	1.2	1.07	0.65	1
Solyc01g080780.2.1	Translocase of chloroplast 34	3.65	4.15	7.97	4.91	5.17	5.12	4.53	5.39	6.18	5.3	1.03	1	5.29	5.36	7.73	6.13	7.18	6.22	6.06	6.49	1.06	1	6.04	5.9	5.94	5.96	6.4	5.98	6.45	6.28	1.05	1	8.59	8.95	11.4	9.65	7.45	8.23	7.84	0.81	1
Solyc01g080790.2.1	PBSP domain-containing prot	0	0	0	0	0	0	0	0	0	0	1	NA	0.81	0	0	0	0.27	0	0	0	0.04	1	0	0	0	0	0	0	0	0	1	NA	0	0	0	0	0	0	0	1	NA
Solyc01g080800.2.1	PBSP domain-containing prot	0	0	0	0	0	0	0	0	0	0	1	NA	0	0	0	0	0	0	0	0	1	NA	0	0	0	0	0	0	0	0	1	NA	0	0	0	0	0	0	0	1	NA
Solyc01g080810.2.1	Isoleucyl-tRNA synthetase (A	10.81	11.73	14.15	14.61	12.82	13.99	17.11	12.45	16.24	14.95	1.17	0.637605	10.16	12.7	14.7	12.52	15.69	14.2	13.81	14.57	1.16	0.850678	6.72	7.65	7.72	7.36	7.96	8.55	8.61	8.37	1.14	1	7.35	12.94	10.68	10.32	17.34	14.42	15.88	1.54	0.922997
Solyc01g080820.1.1	Unknown Protein (AHRD V1)	0	0	0	0	0	0	0	0	0	0	1	NA	0	0	0	0	0	0	0	0	1	NA	0	0	0	0	0	0	0	0	1	NA	0	0	0	0	0	0.33	0.17	16.5	1
Solyc01g080830.2.1	cDNA clone J023109D15 full	4.37	6.81	11.61	15.98	9.69	1.62	2.01	3.02	2.62	2.32	0.24	0.104864	6.44	3.8	5.85	5.36	2.28	2.08	1.43	1.93	0.36	0.203752	13.55	11.88	9.04	11.49	4.07	6.35	2.66	4.36	0.38	1	17.1	7.15	5.6	9.95	6.67	5.11	5.89	0.59	1
Solyc01g080840.2.1	Protein translocase subunit s	37.29	32.73	37.35	27.11	33.62	51.17	51.78	48.39	58.01	52.34	1.56	0.036321	28.85	33.65	41.23	34.58	57.84	47.83	49.1	51.59	1.49	0.088324	14.96	22.16	19.21	18.78	24.67	26.2	25.19	25.35	1.35	1	18.32	25.72	20.61	21.55	32	29.29	30.64	1.42	1
Solyc01g080850.2.1	Protein transport protein SEC	23.95	24.87	36.52	39.73	31.27	21.68	24.55	15.99	23.46	21.42	0.69	0.52171	31.73	30.9	40.39	34.34	38.6	28	27.93	31.51	0.92	1	31.03	31.79	37.6	33.47	22.51	22.32	28.37	24.4	0.73	1	37.8	21.22	23.05	27.36	26.96	33.02	29.99	1.1	1
Solyc01g080860.2.1	Protoheme IX farnesyltransfe	46.97	52.06	51.36	54.8	51.3	52.21	60.7	55.02	59.61	56.89	1.11	0.839126	56.22	57.61	48.14	53.99	46.38	43.84	43.62	44.61	0.83	0.919758	62.24	64.24	59.69	62.06	57.78	68.14	56.14	60.69	0.98	1	82.52	56.06	60.5	66.36	66.55	71.48	69.02	1.04	1
Solyc01g080870.2.1	Peptide transporter-like prote	27.55	28.72	40.53	19.25	29.01	41	22.44	53.89	59.03	44.09	1.52	0.452059	70.35	20.23	20.68	37.09	26.65	25.97	21.74	24.79	0.67	1	27.31	62.61	48.7	46.21	36.06	42.43	79.43	52.64	1.14	1	12.49	3.99	4.73	7.07	5.83	5.29	5.56	0.79	1
Solyc01g080880.2.1	Receptor-like kinase (AHRD V	62.09	59.55	65.68	59.25	61.64	56.23	47.33	49.83	59.66	53.26	0.86	0.968545	71.7	53.39	60.35	61.81	50.03	44.34	47.64	47.34	0.77	0.65923	39.89	44.05	45.29	43.08	40.44	41.28	38.42	40.05	0.93	1	65.83	60.94	53.91	60.23	56.96	59.25	58.11	0.96	1
Solyc01g080890.2.1	Unknown Protein (AHRD V1)	129.53	196.19	89.22	83.07	124.5	203.9	107.34	209.34	99.35	154.98	1.24	1	128.72	112.6	72.71	104.68	57.47	76.65	131.51	88.54	0.85	1	107.9	60.31	59.09	75.77	58.14	94.52	76.21	76.29	1.01	1	261.95	1100.29	1034.26	798.83	128.15	88.88	108.52	0.14	0.198684
Solyc01g080900.2.1	Cytochrome P450	4.64	6.48	7.97	3.37	5.62	6.48	5.65	6.58	7.13	6.46	1.15	0.959699	5.18	4.72	7.94	5.95	7.9	3.64	4.89	5.48	0.92	1	2.57	2.16	1.05	1.93	2.96	2.51	4.05	3.17	1.65	1	2.07	2.31	1.22	1.87	4.61	4.88	4.75	2.54	0.537327
Solyc01g080910.2.1	Unknown Protein (AHRD V1)	190.74	217.12	265.95	154.49	207.07	237.06	150.43	213.12	218.07	204.67	0.99	1	330.56	295.05	314.29	313.3	346	449.62	386.41	394.01	1.26	0.418523	144.76	161.76	59.35	121.96	103.44	73.72	116.92	98.03	0.8	1	89.26	23.89	21.76	44.97	109.29	108.7	109	2.42	0.425749
Solyc01g080920.2.1	U-box domain-containing pro	0	0	0	0	0	0	0	0	0	0	1	NA	0	0	0	0	0	0	0	0	1	NA	0	0	0	0	0	0	0	0	1	NA	0	0	0	0	0	0	0	1	NA
Solyc01g080930.2.1	Enoyl-CoA-hydratase (AHRD V	63.05	64.83	45.4	43.74	54.26	63.59	61.98	65.55	61.92	63.26	1.17	0.831097	54.49	53.91	53.11	53.84	52.55	58.26	65.21	58.67	1.09	0.995024	92.86	81.06	82.71	85.54	79.34	85.16	80.75	81.75	0.96	1	44.46	80.98	59.71	61.72	70.3	60.7	65.5	1.06	1
Solyc01g080940.2.1	5-hydroxyisourate hydrolase	38.09	36	44.24	38.05	39.09	39.07	32.24	29.24	30.73	32.82	0.84	0.908565	30.87	47.62	46.6	41.7	54.74	45.34	40.43	46.84	1.12	0.949675	28.71	38.4	28.65	31.92	24.38	25.45	27.47	25.77	0.81	1	62.53	38.93	37.74	46.4	44.57	45.62	45.09	0.97	1
Solyc01g080950.2.1	Unknown Protein (AHRD V1);	0	0	0	0	0	0	0	0	0	0	1	NA	0	0	0	0	0	0	0	0	1	NA	0	0	0	0	0	0	0	0	1	NA	0	0	0	0	0	0	0	1	NA
Solyc01g080960.2.1	AT-hook DNA-binding protein	0	0	0	0	0	0	0	0	0	0	1	NA	0	0	0	0	0	0	0	0	1	NA	0	0	0	0	0	0	0	0	1	NA	0	0	0	0	0	0	0	1	NA
Solyc01g080970.2.1	Carboxyl methyltransferase 4	0	0	0	0	0	0	0	0	0	0	1	NA	0	0	0	0	0	0	0	0	1	NA	0	0	0	0	0	0	0	0	1	NA	0	0	0	0	0.19	0	0.1	9.5	1
Solyc01g080980.1.1	Unknown Protein (AHRD V1)	0	0	0	0	0	0	0	0	0	0	1	NA	0	0	0	0	0	0	0	0	1	NA	0	0	0	0	0	0	0	0	1	NA	0	0	0	0	0	0	0	1	NA
Solyc01g080990.2.1	Carboxyl methyltransferase 4	0	0.33	0	0	0.08	0.41	0.15	0.34	0	0.22	2.73	1	0	0.32	0	0.11	0	0	0	0	0.09	1	0	0	0	0	0	0	0	0	1	NA	0	0	0	0	0	0	0	1	NA
Solyc01g081000.2.1	Phosphatase PTC7 homolog (	3.32	2.03	0.73	2.35	2.11	3.23	0.98	3.24	2.38	2.46	1.17	1	3.67	2.34	3.33	3.11	2.88	3.01	3.1	3	0.96	1	2.35	4.06	2.68	3.03	1.23	3.14	3.78	2.72	0.9	1	0.59	1.21	1.28	1.03	1.33	0.91	1.12	1.09	1
Solyc01g081010.2.1	Nucleolar GTP-binding protei	473.63	347.15	275.82	203.5	325.02	408.85	303.83	297.8	297.76	327.06	1.01	1	242.55	501.32	652.82	465.56	328.04	411.41	337.85	359.1	0.77	0.990495	154.47	306.35	162.16	207.66	376.8	326.48	155.33	286.2	1.38	1	230.39	334.37	409.43	324.73	353.33	445.4	399.37	1.23	1
Solyc01g081020.1.1	Pentatricopeptide repeat-con	0.11	0.09	0	0.15	0.09	0.35	0.17	0.1	0.08	0.17	2	1	0.1	0	0.18	0.09	0.22	0.05	0.08	0.12	1.25	1	0	0	0	0	0	0.08	0.08	0.05	5.33	1	0	0	0	0	0	0.09	0.04	4.5	1
Solyc01g081030.2.1	Protein SCAI (AHRD V1 ***- S	19.29	20.68	20.62	18.24	19.71	19.07	15.9	21.75	19.21	18.98	0.96	1	26.37	26.4	23.4	25.39	26.09	27.7	27.89	27.23	1.07	1	23.16	23.45	23.27	23.29	22.54	22.94	26.25	23.91	1.03	1	18.03	30.42	27.04	25.16	21.37	25.32	23.34	0.93	1
Solyc01g081040.2.1	RING finger protein (AHRD V	0.21	0	0	0.3	0.13	0	0	0	0.15	0.04	0.29	1	0.2	0	0	0.07	0	0	0	0	0.15	1	0	0	0	0	0	0	0	0	1	NA	0	0	0	0	0	0	0	1	NA
Solyc01g081050.2.1	D-alanine--poly(phosphoribit	6.5	6.62	5.42	8.5	6.76	5.29	6.77	7.19	5.38	6.16	0.91	1	8.85	12.5	10.06	10.47	8.79	8.83	10.65	9.42	0.9	1	7.99	6.1	9.49	7.86	7	7.54	5.46	6.67	0.85	1	19.04	15.52	21.77	18.78	11.71	12.75	12.23	0.65	0.924156
Solyc01g081060.2.1	Xyloglucan endotransglucosyl	9.76	24.15	35.46	13.25	20.66	14.97	16.49	22.07	11.49	16.25	0.79	0.982298	35.95	56.67	43.69	45.44	74.59	52.75	51.38	59.57	1.31	0.628879	433.64	411.29	405.87	416.93	287.85	317.02	507.62	370.83	0.89	1	390.73	863.61	662.71	639.02	207.44	109.01	158.22	0.25	0.026395
Solyc01g081070.2.1	Protein transport protein Sec	16.73	16.81	16.79	20.25	17.64	15.62	18.11	16.73	16.28	16.68	0.95	1	20.46	23.76	20.25	21.49	19.62	18.22	21.48	19.77	0.92	1	42.01	35.21	37.75	38.32	32.23	33.4	34.75	33.46	0.87	1	24.77	25.62	28.38	26.26	28.34	24.88	26.61	1.01	1
Solyc01g081080.1.1	Replication factor C subunit (	0	0	0	0	0	0	0	0	0	0	1	NA	0	0	0	0	0	0	0	0	1	NA	0	0	0	0	0	0	0	0	1	NA	0	0	0	0	0	0	0	1	NA
Solyc01g081090.1.1	BHLH transcription factor (AH	0.17	0.45	0.2	0.47	0.32	0.37	0.27	0.15	0.12	0.23	0.71	1	0.16	0	0.28	0.15	0.23	0	0	0.08	0.52	1	0	0	0	0	0	0	0	0	1	NA	0	0	0.26	0.09	0	0	0	0.12	1
Solyc01g081100.1.1	BHLH transcription factor (AH	0	0	0	0	0	0	0	0	0	0	1	NA	0	0	0	0	0	0	0	0	1	NA	0	0	0	0	0	0	0	0	1	NA	0	0	0	0	0	0	0	1	NA
Solyc01g081110.2.1	Pentatricopeptide repeat-con	0.65	1.16	0.13	1.23	0.79	0.84	1.54	1.8	1.01	1.3	1.64	0.654328	0.71	0.47	1.19	0.79	1.2	1.8	0.85	1.28	1.62	0.724374	0.7	0.44	0.32	0.49	0.5	0.49	0.79	0.59	1.22	1	0.31	0.71	0.67	0.56	0.7	0.86	0.78	1.38	1
Solyc01g081120.2.1	F16G16.3 protein (Fragment)	22.15	23.14	27.27	30.65	25.8	33.19	35.74	33.21	20.74	30.72	1.19	0.804781	20.6	25.95	19.52	22.02	14.7	15.71	20.81	17.07	0.78	0.906212	10.12	11.38	9.08	10.19	5.69	10.22	9.12	8.34	0.82	1	8.9	9.09	8.89	8.96	5.29	6.33	5.81	0.65	1
Solyc01g081130.2.1	Major latex-like protein (AHR	0	0	0	0	0	0	0	0	0	0	1	NA	0	0	0	0	0	0	0	0	1	NA	0	0	0	0	0	0	0	0	1	NA	0	0	0	0	0	0	0	1	NA
Solyc01g081140.1.1	Phototropic-responsive NPH3	0	0	0	0	0	0	0	0	0	0	1	NA	0	0	0	0	0	0	0	0	1	NA	0	0	0	0	0	0	0	0	1	NA	0	0	0	0	0	0	0	1	NA
Solyc01g081150.2.1	DNA-directed RNA polymeras	87.35	95.16	81.59	81.09	86.3	81.93	72.2	104.16	91.46	87.44	1.01	1	88.87	80.29	44.58	71.25	59.96	86.69	98.68	81.78	1.15	0.983376	56.97	50.42	46.51	51.3	39.45	49.66	54.7	47.94	0.93	1	38.17	55.52	49.67	47.79	52.89	65.82	59.35	1.24	1
Solyc01g081160.2.1	Beta-glucosidase (AHRD V1 *	2.15	0.55	1.09	0.43	1.05	7.5	5.33	5.64	4.85	5.83	5.53	0.001164	2.28	4.27	4.66	3.74	10.85	8.26	8.92	9.34	2.5	0.039268	0	0.25	0.3	0.18	0	0	0.22	0.07	0.4	1	0	0.22	0.47	0.23	0.28	0	0.14	0.61	1
Solyc01g081170.1.1	Beta-glucosidase (AHRD V1 *	2.66	1.39	0.55	0.89	1.37	3.83	2.6	3.31	2.81	3.14	2.29	0.187758	1.31	2.32	1.46	1.7	4.35	3.73	5.42	4.5	2.65	0.033062	0.63	0.64	0.16	0.48	0.31	0.24	0.11	0.22	0.46	1	0	0	0	0	0.43	0.14	0.29	28.5	1
Solyc01g081180.1.1	Beta-glucosidase (AHRD V1 *	0	0	0	0	0	0	0	0	0	0	1	NA	0	0	0	0	0	0	0	0	1	NA	0	0	0	0	0	0	0	0	1	NA	0	0	0	0	0	0	0	1	NA
Solyc01g081190.2.1	Calcium-binding EF hand fam	103.1	113.56	83.89	83.26	95.95	131.3	101.67	167.08	110.32	127.59	1.33	0.435181	98.48	121.56	101.43	107.16	122.75	108.61	116.11	115.82	1.08	1	60.96	78.22	39.6	59.59	58.39	58.09	91.25	69.24	1.16	1	96.04	212.61	160.37	156.34	75.58	73.43	74.5	0.48	0.330882
Solyc01g081200.2.1	Unknown Protein (AHRD V1)	27.44	39.46	28.64	29.86	31.35	45.76	24.17	47.07	25.77	35.69	1.14	1	28.96	27.07	28.97	28.33	36.6	38.8	39.82	38.41	1.36	0.556057	27.29	24.94	18.34	23.52	20.46	16	27.31	21.26	0.9	1	7.5	18.78	13.8	13.36	22.22	16.66	19.44	1.46	1
Solyc01g081210.2.1	F-box/kelch-repeat protein At	0.17	0.15	0	0	0.08	0.56	0	0.77	0.6	0.48	6.03	0.565587	0	0.15	0.28	0.14	0.12	0.09	0.13	0.11	0.79	1	0.13	0	0	0.04	0.44	0.13	0	0.19	4.38	1	0	0.12	0	0.04	0.15	0.29	0.22	5.5	1
Solyc01g081220.1.1	Phenylalanyl-tRNA synthetas	5.82	7.77	10.3	4.12	7	6.47	5.36	3.57	4.18	4.89	0.7	0.9636	1.8	5.06	6.56	4.47	4.71	2.01	4.57	3.76	0.84	1	0.78	3.16	1.93	1.96	1.91	1.47	1.41	1.6	0.82	1	1.38	4.93	1.49	2.6	3.55	3.4	3.47	1.34	1

Solyc01g081230.2.1	Unknown Protein (AHRD V1)	12.69	14.68	16.46	16.15	14.99	14.1	13.02	15.16	12.75	13.76	0.92	1	8.26	9.93	9.64	9.28	4.4	17.08	14.96	12.15	1.31	1	3.07	2.07	12.59	5.91	9.17	4.81	11.09	8.36	1.41	1	10.82	16.59	13.66	13.69	9.29	16.69	12.99	0.95	1
Solyc01g081240.2.1	Pentatricopeptide repeat-con	1.17	2.35	0.92	2.77	1.8	1.63	2.32	2.07	2.04	2.01	1.12	1	2.82	2.89	2.15	2.62	2.31	2.13	2.08	2.17	0.83	1	0.79	0.96	0.78	0.84	1.03	1.04	1.14	1.07	1.27	1	1.25	2.84	3.61	2.57	1.52	1.37	1.45	0.56	0.963653
Solyc01g081250.2.1	Glutathione-S-transferase (AH	4.17	4.33	1.64	6.88	4.25	0.77	1.1	3.19	0.5	1.39	0.33	0.444146	0	0.6	0.59	0.4	0	0	0	0	0.03	1	0.56	0	0.69	0.42	0	0	0	0	0.02	1	0	0.5	1.07	0.52	0.64	0.61	0.62	1.19	1
Solyc01g081260.1.1	Glutathione S-transferase (AH	0	0	0	0	0	0	0	0	0	0	1	NA	0	0	0	0	0	0	0	0	1	NA	0	0	0	0	0	0	0	0	1	NA	0	0	0	0	0	0	0	1	NA
Solyc01g081270.2.1	Glutathione S-transferase (AH	8.84	4.98	6.95	2.5	5.82	14.4	5.81	12.73	4.65	9.4	1.62	0.808554	11.78	6.91	7.71	8.8	18.59	15.1	14.58	16.09	1.83	0.087582	4.28	10.08	4.68	6.35	3.68	4.24	6.44	4.79	0.75	1	2.51	17.54	10.42	10.16	14.02	9.81	11.91	1.17	1
Solyc01g081280.2.1	Unknown Protein (AHRD V1)	0	0	0	0	0	0	0	0	0	0	1	NA	0	0	0	0	0	0	0	0	1	NA	0	0	0	0	0	0	0	0	1	NA	0	0	0	0	0	0	0	1	NA
Solyc01g081290.2.1	Pentatricopeptide repeat-con	2.84	2.83	2.77	2.7	2.79	2.56	2.05	3.42	2.6	2.66	0.95	1	3.96	4.09	4.43	4.16	3.57	4.44	3.62	3.88	0.93	1	2.26	3.75	4.28	3.43	4.12	4.27	3.93	4.11	1.2	1	4.39	6.26	5.72	5.46	5.42	5.67	5.54	1.02	1
Solyc01g081300.2.1	Phosphoribosylaminoimidazo	20.16	18.09	13.95	10.13	15.58	20.37	22.88	24.35	19.73	21.83	1.4	0.385763	15.34	16.07	12.1	14.5	18.14	23.2	21.86	21.07	1.45	0.229801	22.48	22.4	20.5	21.79	32.94	33.97	30.47	32.46	1.49	1	22.74	41.62	29.2	31.19	48.65	33.45	41.05	1.32	1
Solyc01g081310.2.1	Glutathione-S-transferase (AH	54.29	54.3	31	22.3	40.47	124.6	67.75	178.48	115.26	121.52	3	0.013823	98.29	160.88	78.65	112.61	130.38	118.04	145.67	131.36	1.17	0.962977	389.13	344.43	156.75	296.77	202.69	204.65	312.06	239.8	0.81	1	82.59	194.84	199.3	158.91	367.42	309.94	338.68	2.13	0.179664
Solyc01g081320.2.1	Pentatricopeptide repeat-con	9.02	10.29	7.95	8.09	8.84	10.74	11.64	12.04	12.41	11.71	1.32	0.511333	8.63	7.73	9.66	8.67	6.44	7.63	7.59	7.22	0.83	1	3.29	3.11	2.52	2.97	1.67	2.22	1.95	1.95	0.65	1	0.72	1.75	1.56	1.34	2.21	3.45	2.83	2.11	0.915151
Solyc01g081330.2.1	Chaperone protein dnaJ (AHR	2.88	3.51	2.33	3.43	3.04	4.38	3.47	5.86	3.81	4.38	1.44	0.614698	3.39	3.03	2.95	3.12	3.58	4.95	4.53	4.35	1.39	0.709908	2.45	1.98	1.96	2.13	1.89	1.61	2.32	1.94	0.91	1	2.16	2.42	3.26	2.61	1.8	2.13	1.96	0.75	1
Solyc01g081340.2.1	Carboxyl methyltransferase 4	0	0.32	0.22	0.26	0.2	0	0.14	0.34	0.13	0.15	0.76	1	0	0	0	0	0.25	0	0.14	0.13	13	1	0	0.15	0	0.05	0	0	0	0	0.2	1	0.78	0.53	0	0.44	0.17	0	0.09	0.19	1
Solyc01g081350.1.1	Aspartic proteinase nepenthe	0	0.27	0.72	0	0.25	0	0.24	0	0.11	0.09	0.35	1	0	0	0	0	0	0	0	0	1	NA	0	0	0	0	0	0	0	0	1	NA	0	0	0	0	0	0	0	1	NA
Solyc01g081360.1.1	Unknown Protein (AHRD V1)	0	0	0	0	0	0	0	0	0	0	1	NA	0	0	0	0	0	0	0	0	1	NA	0	0	0	0	0	0	0	0	1	NA	0	0	0	0	0	0	0	1	NA
Solyc01g081370.2.1	Aspartic proteinase nepenthe	0.9	1.6	0.53	0.85	0.97	0.33	1.18	0.83	0.75	0.77	0.8	1	1.67	0	0.63	0.77	0.1	0.46	0.35	0.3	0.4	1	0	0.12	0	0.04	0	0	0	0	0.25	1	0	0	0	0	0	0	0	1	NA
Solyc01g081380.2.1	Nuclear matrix protein 1 (AH	11.13	12.03	8.44	12.65	11.06	9.06	15.53	10.96	11.7	11.81	1.07	1	13.12	10.19	10.24	11.18	10.89	9.57	10.15	10.2	0.91	1	10.6	11.33	7.49	9.81	4.96	7.38	6.81	6.38	0.65	1	5.09	7.94	6.42	6.48	6	6.79	6.39	0.99	1
Solyc01g081390.2.1	Glucose 6 phosphate/phosph	13.26	12.35	6.81	13.02	11.36	32.25	33	21.22	20.38	26.71	2.35	0.003553	12.62	9.22	10.27	10.7	12.55	14.29	18.14	14.99	1.4	0.413454	10.84	13.6	11.61	12.02	8.61	11.58	12.27	10.82	0.9	1	1.77	5.78	2.88	3.48	7.99	6.7	7.35	2.11	0.819748
Solyc01g081400.2.1	B3 domain-containing protein	7.85	10.48	6.02	7.78	8.03	11.35	12.71	17.33	10.99	13.1	1.63	0.363904	11.69	9.56	9.95	10.4	11.17	11.18	13.27	11.87	1.14	1	6.03	8.32	5.46	6.6	6.19	4.76	3.72	4.89	0.74	1	3.35	4.56	4.23	4.05	5.39	5.16	5.28	1.3	1
Solyc01g081410.2.1	Nodulin-like family protein ex	2.94	3.14	2.77	3.12	2.99	3.43	3.94	4.59	2.43	3.6	1.2	0.95382	3.55	1.92	3.85	3.11	2.14	3.65	3.93	3.24	1.04	1	2.61	2.87	1.31	2.26	1.16	0.78	1.5	1.15	0.51	1	0	0.11	0.23	0.11	0.4	0.13	0.27	2.34	1
Solyc01g081420.1.1	Os04g0218600 protein (Frag	3.6	4	3.54	4.67	3.95	3.33	2.6	2.48	3.01	2.85	0.72	0.944697	3.91	4.17	5.07	4.38	5.62	5.9	4.71	5.41	1.23	0.988213	5.81	3.67	5.36	4.95	6.89	9.09	6.99	7.66	1.55	1	6.82	6.75	10.61	8.06	11.8	6.31	9.05	1.12	1
Solyc01g081430.2.1	Expressed protein (Fragment)	5.89	8.68	7.55	6.8	7.23	6.44	4.25	6.29	7.1	6.02	0.83	0.854164	7.81	6.89	9.07	7.92	6.95	7.47	7.19	7.2	0.91	1	11.41	7.04	14.42	10.96	8.85	11.35	9.16	9.79	0.89	1	11.8	18.47	19.06	16.44	14.85	14.61	14.73	0.9	1
Solyc01g081440.2.1	Unknown Protein (AHRD V1);	10.87	8.99	11.56	14.29	11.43	7.32	10.88	7.46	9.12	8.7	0.76	0.863251	9.4	13.44	16.29	13.04	14.33	13.16	10.1	12.53	0.96	1	4.95	6.98	4.25	5.39	6.84	3.48	5.69	5.34	0.99	1	8.27	15.34	13.18	12.26	10.93	12.88	11.91	0.97	1
Solyc01g081450.2.1	Unknown Protein (AHRD V1);	133.51	145.04	119.06	212.18	152.45	69.4	120.6	92.79	136.73	104.88	0.69	0.676193	170.58	339.65	244.11	251.45	157.97	126.26	124.37	136.2	0.54	0.240396	180.52	237.75	375.74	264.67	331.49	391.08	146.23	289.6	1.09	1	460.61	577.26	581.51	539.79	579.82	572.92	576.37	1.07	1
Solyc01g081460.2.1	tRNA (guanine-N(7)-)-methyl	7.35	8.41	5.95	6.24	6.99	7.47	7.8	7.15	7.54	7.49	1.07	1	4.3	7.12	4.97	5.46	5.68	8.7	9.08	7.82	1.43	0.604902	4.75	5.99	3.75	4.83	5.52	6.21	5.36	5.7	1.18	1	2.99	3.36	3.88	3.41	5.39	3.5	4.45	1.3	1
Solyc01g081470.2.1	Ubiquinone/menaquinone bi	18.69	20.04	16.74	14.01	17.37	19.02	21.33	24.43	18.59	20.84	1.2	0.81871	18.22	17.22	14.26	16.57	16.08	21.1	21.06	19.41	1.17	0.881477	20.01	15.78	13.86	16.55	18.19	14.16	20.18	17.51	1.06	1	8.32	19.3	13.16	13.59	10.51	10.27	10.39	0.76	1
Solyc01g081480.2.1	Genomic DNA chromosome 5	15.93	15.63	14.22	10.4	14.04	25.88	28.86	26.85	20.82	25.6	1.82	0.02361	14.82	11.36	12.08	12.75	15.16	12.9	17.66	15.24	1.19	0.906212	2.97	2.5	0.81	2.09	3.62	3.56	3.27	3.48	1.66	1	0	0.74	0	0.25	0.56	0.54	0.55	2.23	1
Solyc01g081490.2.1	RNA polymerase sigma factor	14.93	9.18	11.41	10.64	11.54	19.83	20.36	10.18	20.44	17.7	1.53	0.22933	7.4	7.85	7.99	7.75	16.1	13.13	14.95	14.73	1.9	0.011074	11.55	16.11	15.14	14.27	12.28	13.27	15.66	13.74	0.96	1	2.14	7.73	4.63	4.83	19.47	11.68	15.57	3.22	0.34358
Solyc01g081500.2.1	Histone-lysine N-methyltrans	61.52	64.88	58.37	61.36	61.53	49.32	46.09	58.68	51.15	51.31	0.83	0.85397	61.37	59.35	58.23	59.65	48.5	49.02	48.47	48.66	0.82	0.891049	43.97	46.23	37.16	42.45	46.91	31.63	43.75	40.76	0.96	1	49.71	118.59	96.88	88.39	45.44	53.22	49.33	0.56	0.654344
Solyc01g081510.2.1	3-hydroxyisobutyrate dehydr	13.06	15.89	14.38	16.63	14.99	9.44	15.99	10.21	13.44	12.27	0.82	0.982067	16	22.14	23.72	20.62	27.48	26.94	19.85	24.76	1.2	0.792252	6.16	13.31	7.56	9.01	13.59	8.58	13.64	11.94	1.32	1	14.86	18.19	17.08	16.71	19.73	25.39	22.56	1.35	1
Solyc01g081520.2.1	30S ribosomal protein S2 (AH	31.03	28.2	30.47	29.16	29.71	28.1	32.38	26.94	32.25	29.92	1.01	1	30.88	28.11	26.58	28.52	24.46	30.06	27.71	27.41	0.96	1	22.88	25.2	21.43	23.17	26.87	25.82	24.2	25.63	1.11	1	30.55	25.14	23.86	26.52	29.5	37.39	33.45	1.26	1
Solyc01g081530.1.1	Annexin 3 (AHRD V1 *-*- A9X	0.52	0.23	0	0.37	0.28	0	0.41	0.24	0	0.16	0.58	1	0.24	0	0	0.08	0	0	0	0	0.12	1	0	0	0	0	0	0	0	0	1	NA	0	0	0	0	0	0	0	1	NA
Solyc01g081540.2.1	Myosin XI-F (AHRD V1 ***- B	32.56	37.86	47.95	35.53	38.48	23.99	36.21	28.47	36.34	31.25	0.81	0.862995	44.54	23.33	28.93	32.27	21.07	26.45	23.54	23.69	0.73	0.898181	20.87	17.38	20.55	19.6	20.15	18.08	23.12	20.45	1.04	1	10.89	11.21	12.55	11.55	15.14	26.56	20.85	1.81	0.375043
Solyc01g081550.1.1	Unknown Protein (AHRD V1)	0	0	0	0	0	2.72	0	0	0	0.68	68	1	2.27	0	0	0.76	0	0	0	0	0.01	1	0	0	0	0	0	0	0	0	1	NA	0	0	0	0	0	0	0	1	NA
Solyc01g081560.2.1	Pentatricopeptide repeat-con	0.32	0.57	0.82	0.45	0.54	0.42	0.35	0.29	0.59	0.41	0.76	1	1	0.66	0.91	0.86	0.35	0.62	0.65	0.54	0.63	0.88053	0.36	0.72	0.38	0.49	0.29	0.14	0.37	0.27	0.55	1	0.27	0.37	0.39	0.34	0.52	0.61	0.56	1.65	1
Solyc01g081570.2.1	Carboxyl-terminal peptidase (	25.71	35.15	23.75	29.57	28.55	42	32.64	44.99	28.91	37.13	1.3	0.511579	27.51	28.94	28.12	28.19	30.83	44.1	41.14	38.69	1.37	0.272512	11.51	21.2	8.45	13.72	16.28	10.8	17.49	14.86	1.08	1	7.71	18.56	23.81	16.69	20.56	27.7	24.13	1.45	1
Solyc01g081580.1.1	Non-specific lipid-transfer pr	0	0	0	0	0	0	0	0	0	0	1	NA	0	0	0	0	0	0	0	0	1	NA	0	0	0	0	0	0	0	0	1	NA	0	0	0	0	0	0	0	1	NA
Solyc01g081590.2.1	Non-specific lipid-transfer pr	0	0	0	0	0	0	0	0	0	0	1	NA	0	0	0	0	0	0	0	0	1	NA	0	0	0	0	0	0	0	0	1	NA	0	0	0	0	0	0	0	1	NA
Solyc01g081600.2.1	Non-specific lipid-transfer pr	0	0	0.64	0	0.16	0.6	0	0	0	0.15	0.94	1	1.01	0.47	0	0.49	0	0.84	0	0.28	0.57	1	0	0.44	0	0.15	0	0	0.39	0.13	0.89	1	0	0	0	0	0.5	0.47	0.48	48.5	1
Solyc01g081610.2.1	Beta-hexosaminidase 1 (AHR	13.29	14.37	30.13	22.03	19.95	15.02	15.91	15.8	16.28	15.75	0.79	0.976048	13.42	17.62	24.47	18.5	20.97	17.97	13.05	17.33	0.94	1	48.95	41.72	83.33	58	61.7	56.16	45.15	54.34	0.94	1	15.47	25.62	19.2	20.1	30.4	25	27.7	1.38	1
Solyc01g081620.2.1	Vacuolar sorting protein (AH	0.24	0.43	0.86	0.17	0.42	0	0.1	0	0.18	0.07	0.16	0.440309	0.23	0.11	0.1	0.15	0.08	0.06	0.1	0.08	0.55	1	0.1	0.1	0	0.07	0.08	0	0.09	0.06	0.85	1	0	0.53	0.19	0.24	0	0.11	0.06	0.23	1
Solyc01g081630.2.1	Auxin-regulated protein (AHR	0	0	0	0	0	0	0.17	0	0	0.04	4.25	1	0	0	0	0	0	0	0	0	1	NA	0	0	0	0	0	0	0	0	1	NA	0	0	0	0	0	0	0	1	NA
Solyc01g081640.2.1	Legume lectin beta domain (	3.15	3.74	2.97	4.01	3.47	3.5	3.07	3.57	2.87	3.25	0.94	1	2.35	2.47	2.75	2.52	0.66	1.74	1.65	1.35	0.54	0.449221	0	0.09	0	0.03	0	0	0	0	0.33	1	0	0.08	0	0.03	0	0.18	0.09	3.37	1
Solyc01g086640.2.1	Os03g0169000 protein (Frag	4.2	3.1	4.81	3.73	3.96	1.73	2.27	1.87	2.5	2.09	0.53	0.272326	6.36	5.84	8.41	6.87	8.57	7.89	6.88	7.78	1.13	0.985312	0.97	3.03	0.95	1.65	0.87	0.36	0.79	0.67	0.41	1	8.19	1.83	3.5	4.51	0.88	4	2.44	0.54	1
Solyc01g086650.2.1	Siroheme synthase (AHRD V1	11.37	10.12	7.43	13.61	10.63	11.48	10.22	6.92	10.8	9.86	0.93	1	10.09	8.52	10.94	9.85	12.5	12.59	11.55	12.21	1.24	0.786061	10.46	9.41	8.51	9.46	12.88	13.66	15.56	14.03	1.48	1	11.2	12.46	22.07	15.24	15.38	18.26	16.82	1.1	1
Solyc01g086660.2.1	Unknown Protein (AHRD V1)	1.14	3.04	0	0	1.04	1.26	0.9	0	0.82	0.74	0.71	1	3.17	1.98	0	1.72	2.37	1.77	0	1.38	0.8	1	0.92	0.93	0	0.62	0	0	0.83	0.28	0.45	1	0	4.13	5.25	3.13	0	1	0.5	0.16	1
Solyc01g086670.2.1	Unknown Protein (AHRD V1)	0	3.98	0	3.16	1.79	1.24	0	0	1.61	0.71	0.4	0.993713	3.12	0.97	0	1.36	5.43	1.16	3.51	3.37	2.47	0.941044	0.9	0	1.11	0.67	0	0	0.81	0.27	0.4	1	1.59	1.62	1.72	1.64	0	0	0	0.01	1
Solyc01g086680.2.1	Glutathione S-transferase (AH	0.29	0.26	0	0	0.14	0	0	0	0	0	0.07	1	0	1.53	0.25	0.59	1.63	0.76	0.69	1.03	1.73	1	0.24	0.24	0	0.16	0.58	0	0.85	0.48	2.98	1	0.83	1.92	1.35	1.37	0	0	0	0.01	0.527255
Solyc01g086690.2.1	Double-strand-break repair p	0.79	0.79	0.58	1.12	0.82	0.88	1.32	0.63	0.42	0.81	0.99	1	0.46	0.77	0.42	0.55	0.14	0.46	0.39	0.33	0.6	1	0.24	0.24	0	0.16	0.06	0.07	0.22	0.12	0.73	1	0.14	0	0.15	0.1	0.09	0	0.04	0.47	1
Solyc01g086700.2.1	Phosphoribosylanthranilate t	0	0	0	0	0	0	0	0	0	0	1	NA	0	0	0	0	0	0.04	0	0.01	1.33	1	0	0	0	0	0	0	0	0	1	NA	0	0	0	0	0	0	0	1	NA
Solyc01g086710.2.1	DNA-directed RNA polymeras	12.87	13.27	13.48	10.89	12.63	8.49	9.92	10.73	10.62	9.94	0.79	0.721887	11.97	9.04	11.17	10.73	11.35	10.24	13.28	11.62	1.08	1	4.28	5.24	5.31	4.94	5.29	4.38	4.86	4.84	0.98	1	11.71	8.69	9.43	9.94	10.96	14.97	12.96	1.3	1
Solyc01g086720.2.1	Phosphoribosylanthranilate t	0.18	0.38	0.07	0.17	0.2	0.2	0.14	0.11	0.35	0.2	1	1	0.17	0.21	0.15	0.18	0.55	0.06	0.05	0.22	1.25	1	0.05	0.25	0.06	0.12	0.04	0.05	0.13	0.07	0.61	1	0	0	0	0	0	0	0	1	NA
Solyc01g086730.2.1	DUF803 domain membrane p	0	0	0	0	0	0	0	0	0	0	1	NA	0	0	0	0	0	0.1	0.15	0.08	8.33	1	0	0	0	0	0	0.14	0.14	0.09	9.33	1	0	0.28	0.29	0.19	0	0	0	0.05	1
Solyc01g086740.2.1	Chaperone protein dnaJ (AHR	41.9	37.56	36.29	30.9	36.66	33.36	27.8	31.42	37.23	32.45	0.89	1	38.84	32.03	31.63	34.17	35.35	38.77	37.37	37.16	1.09	0.980282	27.01	44.68	44.54	38.74	41.68	42.79	43.09	42.52	1.1	1	13.1	26.76	15.77	18.54	44.2	49.24	46.72	2.52	0.11412
Solyc01g086750.2.1	Protein TIF31 homolog (AHRD	32.5	33.54	45.56	41.18	38.2	27.83	29.32	26.68	34.55	29.59	0.77	0.721808	35.4	35.46	44.64	38.5	40.9	33.29	36.98	37.06	0.96	1	27.94	28.86	31.71	29.5	31.37	27.76	30.71	29.95	1.02	1	29.6	22.76	24.88	25.75	32.76	37.44	35.1	1.36	0.93033
Solyc01g086760.2.1	Histone H3 (AHRD V1 ***- B	1.4	0	0.83	0	0.56	0	0.55	0	0.5	0.26	0.47	1	0	0	0	0	0	0	0	0	1	NA	0.57	0.57	0	0.38	0	0	0.51	0.17	0.45	1	0	0	0	0	0	0	0	1	NA
Solyc01g086770.1.1	Unknown Protein (AHRD V1)	0	0	0	0	0	0	0	0	0	0	1	NA	0	0	0	0	0	0	0	0	1	NA	0	0	0	0	0	0	0	0	1	NA	0	0	0	0	0	0	0	1	NA
Solyc01g086780.1.1	Unknown Protein (AHRD V1)	0	0	0	0	0	0	0	0	0	0	1	NA	0.36	0	0	0.12	0	0	0	0	0.08	1	0	0	0	0	0	0	0	0	1	NA	0	0	0	0	0	0	0	1	NA
Solyc01g086790.1.1	Unknown Protein (AHRD V1)	0	0	0	0	0	0	0	0	0	0	1	NA	0	0	0	0	0	0	0	0	1	NA	0	0	0	0	0	0	0	0	1	NA	0	0	0	0	0	0	0	1	NA
Solyc01g086800.2.1	Histone H3 (AHRD V1 **** Q	42.19	36.87	31.68	60.76	42.88	13.64	40.58	16.93	20.93	23.02	0.54	0.429034	35.68	42.68	25.27	34.54	10.64	9.53	13.87	11.35	0.33	0.001924	11.78	18.75	1.52	10.68	3.02	0.58	2.79	2.13	0.2	0.267943	3.27	3.34	4.72	3.78	5.62	1.35	3.49	0.92	1
Solyc01g086810.2.1	Cc-nbs-lrr, resistance protein	11.6	11.61	13.12	11.75	12.02	11.9	9.86	12.06	12.37	11.55	0.96	1	15.07	17.37	20.29	17.58	20.19	19.73	17.37	19.1	1.09	0.998855	4.99	4.92	3.95	4.62	3.88	3.69	4.2	3.92	0.85	1	6.54	8.78	5.17	6.83	5.34	6.14	5.74	0.84	1
Solyc01g086820.2.1	Histone H3 (AHRD V1 ***- D	20.22	27.43	24.43	36.1	27.05	16.06	28.51	17.71	16.59	19.72	0.73	0.778639	19.26	27.21	22.37	22.95	9.35	8.98	9.08	9.14	0.4	0.013327	9.73	21.19	5.74	12.22	2.21	0.73	5.97	2.97	0.24	0.267943	4.11	1.75	2.96	2.94	14.55	4.22	9.38	3.19	0.824404
Solyc01g086830.2.1	Protease inhibitor/seed stora	0	0	0	0	0	0	0	0	0	0	1	NA	0	0	0	0	0	0	0	0	1	NA	0	0	0	0	0	0	0	0	1	NA	0	0	0	0	0	0	0	1	NA
Solyc01g086840.1.1	Thaumatin-like protein (AHRD	0	0	0	0	0	0	0	0	0	0	1	NA	0	0	0	0	0	0	0	0	1	NA	0	0	0	0	0	0	0	0	1	NA	0	0	0	0	0	0	0	1	NA
Solyc01g086850.2.1	Ras-related protein Rab-25 (	23.35	24.16	22.04	34.27	25.96	15.57	16.82	20.4	20.96	18.44	0.71	0.627288	27.7	34.51	32.79	31.67	24.9	24.94	25	24.95	0.79	0.856505	32.07	35.68	39.41	35.72	25.89	28.76	20.63	25.09	0.7	1	56.44	61.06	70.03	62.51	67.87	70.64	69.25	1.11	1
Solyc01g086860.2.1	Unknown Protein (AHRD V1)	111.69	91.85	175.7	175.54	138.69	119.8	88.7	106.9	94.26	102.41	0.74	0.754497	118.48	136.27	176.54	143.76	190.73	143.39	109.85	147.99	1.03	1	58.05	81.43	82.99	74.16	76.81	74.26	70.45	73.84	1	1	144.88	68.31	80.35	97.85	93.87	92.2	93.03	0.95	1
Solyc01g086870.2.1	BHLH transcription factor (AH	25.87	33.67	24.04	42.54	31.53	24.08	23.62	21.41	15.44	21.14	0.67	0.356846	22.26	36.97	27.59	28.94	21.2	25.2	31.64	26.01	0.9	1	33.7	24.77	12.2	23.56	16.46	13.85	15.32	15.21	0.65	1	52.65	27.72	36.8	39.06	21.61	29.06	25.34	0.65	1
Solyc01g086880.1.1	NAD(P)H-quinone oxidoreduc	0	0	0	0	0	0	0	0	0	0	1	NA	0	0	0	0	0	0	0	0	1	NA	0	0	0	0	0	0	0	0	1	NA	0	0	0	0	0	0	0	1	NA
Solyc01g086890.2.1	Splicing factor 4-like protein (	177.49	187.59	193.51	157.1	178.92	194.77	181.33	215.08	186.19	194.34	1.09	0.958713	229.98	242.54	248.95	240.49	162.14	194.37	174.2	176.9	0.74	0.499817	160.63	162.01	166.71	163.12	170.27	145.88	128.34	148.16	0.91	1	299.25	234.08	258.97	264.1	193.22	204.84	199.03	0.75	1
Solyc01g086900.2.1	RNA methyltransferase TrmH	24.71	20.63	21.94	25.94	23.3	37.94	45.04	29.27	31.27	35.88	1.54	0.101466	20.85	16.38	18.07	18.43	21.2	22.82	21.82	21.95	1.19	0.84219	11.11	12.25	9.35	10.9	9.2	12.93	10.39	10.84	0.99	1	4.35	6.48	6.27	5.7	14.92	15.86	15.39	2.7	0.222751
Solyc01g086910.2.1	Unknown Protein (AHRD V1)	2.38	4.24	13.13	26.98	11.68	2.65	5.65	5.12	1.71	3.78	0.32	0.773437	3.7	2.76	12.76	6.41	0.55	0.41	1.25	0.74	0.11	0.363289	0	0	0	0	0	0	0	0	1	NA	0	0	0	0	0	0	0	1	NA
Solyc01g086920.2.1	LRR receptor-like serine/thre	1025.95	966.47	1281.16	2231.66	1376.31	919.98	1483.88	485.98	790.81	920.16	0.67	0.906989	629.64	767.06	1139.74	845.48	289.23	545.49	401.88	412.2	0.49	0.176517	1.29	1.3	4.37	2.32	2.89	0.61	2.04	1.85	0.8	1	7.39	2.03	5.53	4.98	0.37	0.35	0.36	0.07	0.296794
Solyc01g086930.2.1	Gamma response I protein-lik	1.55	1.1	1.1	0.59	1.09	0.69	1.63	1.62	1.56	1.38	1.27	0.951897	2.02	0.99	1.14	1.38	0.93	1.39	0.89	1.07	0.77	1	0.42	0.76	0	0.39	0.41	0.08	0.45	0.31	0.8	1	0.59	0.98	1.27	0.95	0.76	0.45	0.6	0.64	1
Solyc01g086940.2.1	Zinc finger CCCH domain-con	16.94	23.12	16.53	9.59	16.55	24.61	20.51	34.9	22.22	25.56	1.54	0.285529	17.65	14.53	11.64	14.61	13.32	11.23	14.56	13.04	0.89	1	17.34	14.36	11.89	14.53	16.33	15.93	16.13	16.13	1.11	1	9.32	15.92	15.98	13.74	12	14.06	13.03	0.95	1
Solyc01g086950.2.1	Zinc finger CCCH domain-con	38.77	37.6	31.41	21.28	32.27	47.6	38.38	46.82	48.19	45.25	1.4	0.354636	30.68	28.17	23.71	27.52	21.87	26.52	30.02	26.14	0.95	1	29.92	28.29	30.44	29.55	36.88	33.55	35.21	35.21	1.19	1	18.1	24.8	24.03	22.31	31.78	29.68	30.73	1.38	1
Solyc01g086960.2.1	Zinc finger A20 and AN1 dom	157.77	147.56	184.08	216.26	176.42	163.35	179.4	164.32	109.26	154.08	0.87	1	197.73	284.31	270.64	250.89	222	199.52	160.2	193.91	0.77	0.682402	308.79	395.64	557.12	420.52	441.18	405.83	316.42	387.81	0.92	1	1557.83	1579.68	1582.35	1573.29	910.33	983.32	946.83	0.6	0.597049
Solyc01g086970.2.1	Zinc finger A20 and AN1 dom	1049.84	1015.88	1011.26	853.94	982.73	891.59	662.09	845.7	901.59	825.24	0.84	0.818867	1365.92	1630.89	1291.6	1429.47	1770.99	1486.96	1468.06	1575.34	1.1	0.948858	1495.43	1387.25	1360.52	1414.4	1222.21	1639.26	1507.59	1456.35	1.03	1	5384.92	3290.63	4117.72	4264.42	2990.85	3460.57	3225.71	0.76	1
Solyc01g086980.2.1	Polyadenylate-binding protei	11.49	14.94	17.49	12.5	14.11	10.59	12.87	13.63	10.43	11.88	0.84	0.932009	15.71	17.24	21.72	18.22	17.98	15.25	17.44	16.89	0.93	1	14.67	13.92	16.18	14.92	13.93	12.87	14.67	13.82	0.93	1	23.45	16.47	18.72	19.55	15.46	20.83	18.14	0.93	1
Solyc01g086990.2.1	Pre-mRNA-splicing factor SYF	23.31	20.62	26.14	26.97	24.26	14.31	15.56	15.48	19.21	16.14	0.67	0.285884	21.95	27.2	32.75	27.3	31.64	29.95	25.78	29.12	1.07	1	15.64	19.58	19.99	18.4	21.5	17.07	18.61	19.06	1.04	1	31.91	29.63	35.18	32.24	25.42	32.11	28.77	0.89	1
Solyc01g087000.2.1	Unknown Protein (AHRD V1)	29.64	12.76	15.79	18.93	19.28	28.7	29.44	31.65	17.85	26.91	1.4	0.674591	21.34	25.76	16.96	21.35	27.19	23.76	25.55	25.5	1.19	1	16.99	28.83	28.47	24.76	22.61	27.54	24.39	24.85	1	1	48.94	34.03	32.35	38.44	27.13	29.34	28.23	0.73	1
Solyc01g087010.2.1	Acetyl xylan esterase A (AHR	0	0	0	0	0	0	0	0.23	0	0.06	5.75	1	0	0	0	0	0	0	0	0	1	NA	0	0	0	0	0	0	0	0	1	NA	0	0	0	0	0	0	0	1	NA
Solyc01g087020.2.1	Unknown Protein (AHRD V1)	169.64	207.83	229.79	878.35	371.4	111.74	161.21	161.75	199.97	158.67	0.43	0.856615	597.09	1016.78	1132.99	915.62	239.68	256.45	169.52	221.88	0.24	0.00036	155.67	569.15	507.23	410.68	211.88	153.97	132.01	165.95	0.4	1	61.04	54.76	49.92	55.24	408.35	700.54	554.44	10.04	9.45E-06
Solyc01g087030.2.1	Makorin RING finger protein	45.87	48.63	56.49	40.32	47.83	37.17	30.87	34.78	44.48	36.83	0.77	0.639388	55.42	68.63	59.43	61.16	89.74	84.12	89.47	87.78	1.44	0.158911	35.88	42.59	34.1	37.52	41.78	40.72	41.15	41.22	1.1	1	133.81	115.61	139.38	129.6	113.42	170	141.71	1.09	1
Solyc01g087040.2.1	Thylakoid lumenal 19 kDa pr	26.72	21.84	36.34	25.84	27.69	64.24	55.1	41.61	30.53	47.87	1.73	0.172365	18.26	20.38	19.11	19.25	46.7	33.17	30.64	36.84	1.91	0.020222	5.73	11.33	7.04	8.03	10.92	7.44	12.32	10.23	1.27	0.806294	6.59	6.34	5.03	5.99	6.99	4.3	5.64	0.94	1
Solyc01g087050.2.1	Zinc finger-like protein (AHRD	0.22	0	0	0	0.06	0	0	0.2	0.32	0.13	2.36	1	0.21	0.38	0.37	0.32	0	0	0	0	0.03	0.704134	0	0	0	0	0	0	0	0	1	NA	0	0	0	0	0	0	0	1	NA
Solyc01g087060.2.1	Ring-h2 finger protein (AHRD	1.6	0.63	0.84	0.5	0.89	0.59	1.97	0.65	0.51	0.93	1.04	1	1.32	0.62	0.9	0.95	0.25	0.37	0.56	0.39	0.42	0.77615	0.14	0.43	0	0.19	0	0.13	0	0.04	0.23	1	0	0.13	0.27	0.13	0	0	0	0.07	1
Solyc01g087070.2.1	Mitochondrial ADP/ATP carri	1.14	2.88	2.92	4.03	2.74	1.69	3.45	2.1	1.77	2.25	0.82	1	2.3	1.49	4.34	2.71	3.3	4.04	4.33	3.89	1.44	0.805301	0.92	0.77	0.57	0.75	1	0.72	0.42	0.71	0.95	1	0.81	0.55	0.58	0.65	0.17	0.17	0.17	0.26	1
Solyc01g087080.2.1	Unknown Protein (AHRD V1)	0.54	0.64	0.85	1.02	0.76	0.6	0.57	0.5	0.52	0.55	0.72	1	0	0.47	0.46	0.31	0.25	0.37	0.28	0.3	0.97	1	0.15	0	0	0.05	0.12	0	0.13	0.08	1.67	1	0	0.13	0	0.04	0.16	0.16	0.16	3.69	1
Solyc01g087090.2.1	cDNA clone J023009A03 full	100.9	125.2	80.12	74.1	95.08	212.45	115.65	219.43	91.24	159.69	1.68	0.494108	71.29	91.08	63.92	75.43	102.95	100.46	121.99	108.47	1.44	0.325001	54.87	61.66	55.85	57.46	47.8	42.65	43.83	44.76	0.78	1	597.27	83.15	190.96	290.46	133.21	129.3	131.25	0.45	1
Solyc01g087120.2.1	F1-ATP synthase delta subuni	404.9	345.43	388.19	434.31	393.21	404.24	447.48	400.33	327.39	394.86	1	1	320.38	298.44	301.36	306.73	269.39	269.84	271.75	270.33	0.88	1	306.02	337.1	279.66	307.59	296.15	275.01	299.66	290.27	0.94	1	245.44	254.55	241.78	247.26	266.72	242.7	254.71	1.03	1
Solyc01g087130.2.1	MYB transcription factor (AH	0	0	0.22	0.26	0.12	0	0	0	0	0	0.08	1	0	0	0	0	0	0	0	0	1	NA	0	0	0	0	0	0	0	0	1	NA	0	0	0	0	0	0	0	1	NA
Solyc01g087140.2.1	Harpin-induced protein (AHR	0.37	0.81	0	0.26	0.36	0.41	0.14	0.5	0.13	0.3	0.82	1	0	0	0	0	0.13	0	0	0.04	4.33	1	0	0	0	0	0	0	0	0	1	NA	0	0	0.28	0.09	0	0	0	0.11	1
Solyc01g087150.2.1	Mate efflux family protein (A	0.38	0.67	1.04	0	0.52	0	0.69	0.58	0.63	0.47	0.91	1	0.35	0	0.21	0.19	0.09	0.06	0.1	0.08	0.45	1	0	0	0	0	0	0.1	0.09	0.06	6.33	1	0	0	0	0	0	0	0	1	NA
Solyc01g087160.2.1	Unknown Protein (AHRD V1)	0.72	0.65	3.42	4.1	2.22	2.42	1.14	4.67	0	2.06	0.93	1	0.67	0.63	1.84	1.05	0	0.38	0	0.13	0.12	0.913866	0.59	0	5.04	1.88	0	0	0	0	0.01	1	1.03	0.53	1.11	0.89	0	0	0	0.01	1
Solyc01g087170.2.1	Zinc finger CCCH domain-con	79.5	84.66	110.1	134.55	102.2	70.33	99.42	65.77	79.94	78.86	0.77	0.85	119.65	140.82	143.17	134.55	109.86	89.09	101.8	100.25	0.75	0.482313	148.73	196.17	159.31	168.07	144.92	133.26	125.39	134.52	0.8	1	109.39	72.25	70.48	84.04	83.78	96.45	90.12	1.07	1
Solyc01g087180.2.1	Unknown Protein (AHRD V1)	0	0.36	0.47	0	0.21	0.45	0	1.85	0.58	0.72	3.47	0.916606	0.37	0	0.68	0.35	3.63	3.12	1.9	2.88	8.24	0.065558	0.32	0.98	0	0.43	2.91	1.22	3.22	2.45	5.65	1	0.57	0.88	1.24	0.9	0.74	0.35	0.54	0.61	1
Solyc01g087190.2.1	Chaperonin-like protein (AHR	13.67	19.57	12.27	9.67	13.8	27.75	26.51	39.07	23.25	29.15	2.11	0.006868	15.76	14.72	13.3	14.59	16.08	19.39	23.59	19.69	1.35	0.601864	16.09	11.38	6.49	11.32	16.4	11.04	12.56	13.33	1.18	1	11.41	13.81	12.34	12.52	4.9	7.3	6.1	0.49	0.664714
Solyc01g087200.2.1	Cc-nbs-lrr, resistance protein	0.22	0.93	0.71	0.64	0.62	0.67	0.47	0.14	0.22	0.38	0.6	0.772626	0.35	0.39	0.13	0.29	0.62	0.31	0.35	0.43	1.47	1	0.24	0.43	0.59	0.42	0.49	0.4	0.22	0.37	0.88	1	0.96	0.33	0.81	0.7	0.48	0.59	0.53	0.76	1
Solyc01g087210.2.1	Cellulose synthase (AHRD V1	183.06	211.67	194.8	207.46	199.25	147.61	200.84	162.38	193.77	176.15	0.88	1	209.51	169.29	192.61	190.47	121.61	162.88	158.31	147.6	0.77	0.695764	35.65	46.57	34.19	38.8	49.1	34.55	38.41	40.69	1.05	1	130.83	122.13	147.02	133.33	179	296.1	237.55	1.78	0.30764
Solyc01g087230.2.1	Unknown Protein (AHRD V1)	10.41	7.7	10.96	9.06	9.53	10.86	9.48	9.72	10.58	10.16	1.07	1	9.53	9.74	10.95	10.07	10.99	9.95	12.33	11.09	1.1	1	8.41	9.13	7.95	8.5	8.42	10.92	7.58	8.97	1.06	1	14.34	9.3	10.1	11.25	8.06	9.41	8.73	0.78	1
Solyc01g087240.2.1	Nuclear transcription factor Y	85.07	72.04	98.83	48.5	76.11	100.35	99.54	101.76	132.12	108.44	1.42	0.151026	97.9	64.14	72.77	78.27	71.19	66.63	64.46	67.43	0.86	1	19.11	24.23	16.8	20.05	24.09	30.76	21.66	25.5	1.27	1	45.97	63.83	51.72	53.84	46.09	56.13	51.11	0.95	1
Solyc01g087250.2.1	Carotenoid cleavage dioxyger	17.52	24.09	17.68	23.07	20.59	20.15	20.6	22.11	21.34	21.05	1.02	1	16.07	25.52	23.06	21.55	19.61	18.46	18.04	18.7	0.87	1	11.81	13.49	11.39	12.23	14.6	13.37	12.94	13.64	1.12	1	6.01	7.28	8.55	7.28	12.5	11.02	11.76	1.62	0.823223
Solyc01g087260.2.1	Carotenoid cleavage dioxyger	167.22	189.69	188.45	184.92	182.57	202.4	192.5	210.07	264.92	217.47	1.19	0.56498	255.58	282.43	303.2	280.4	501.64	374.73	406.21	427.53	1.52	0.050788	226.08	255.2	251.73	244.34	258.52	274.83	276.73	270.03	1.11	1	174.71	216.14	164.8	185.22	349.07	336.25	342.66	1.85	0.390182
Solyc01g087270.2.1	Carotenoid cleavage dioxyger	0	0	0	0	0	0	0	0	0	0	1	NA	0	0	0	0	0	0	0.66	0.22	22	1	0	0	0	0	0	0	0	0	1	NA	0	0	0	0	0	0	0	1	NA
Solyc01g087280.1.1	Polygalacturonase A (AHRD V	0	0	0	0	0	0	0	0	0	0	1	NA	0	0	0	0	0	0	0	0	1	NA	0	0	0	0	0	0	0	0	1	NA	0	0	0	0	0	0	0	1	NA
Solyc01g087290.2.1	TRNA-dihydrouridine synthas	26.09	27.36	29.72	32.02	28.8	50.83	42.97	45.63	34.77	43.55	1.51	0.091457	30.26	37.15	35.71	34.37	38.89	39.13	41.12	39.71	1.16	0.865391	15.63	17.1	13.1	15.28	18.12	14.09	19.34	17.18	1.12	1	29.84	18.29	22.13	23.42	26.76	33.26	30.01	1.28	1
Solyc01g087300.2.1	tRNA-dihydrouridine synthase	12.56	16.37	15.65	15.26	14.96	22.02	19.27	14.15	13.59	17.26	1.15	0.90174	16.62	13.68	11.65	13.98	14.84	10.99	14.04	13.29	0.95	1	26.01	26.37	25.46	25.95	17.54	20.78	22.68	20.33	0.78	1	11.57	11.05	8.98	10.53	16.68	12.87	14.77	1.4	1

Solyc01g087310.2.1	Histone-lysine N-methyltrans	6.56	6.81	7.18	5.13	6.42	6.23	5.77	8.74	8.05	7.2	1.12	0.930443	8.33	9.15	9.41	8.96	4.69	7.93	8.65	7.09	0.79	0.862539	8.06	4.79	6.85	6.57	7.54	4.72	6.73	6.33	0.96	1	12.05	6.75	9.57	9.46	5.51	6.65	6.08	0.64	0.999777
Solyc01g087320.2.1	tRNA-dihydrouridine synthase	243.99	275.86	289.09	257.73	266.67	359.68	328.2	336.98	307.48	333.09	1.25	0.533503	313.64	338.62	324.6	325.62	300.68	297.12	350.57	316.12	0.97	1	329.94	290.12	291.03	303.7	273.79	247.63	296.07	272.5	0.9	1	329.04	227.41	259.25	271.9	271.28	278.2	274.74	1.01	1
Solyc01g087330.2.1	PHD finger family protein (AH	1.29	8.06	6.1	7.32	5.69	2.88	9.19	3.57	4.64	5.07	0.89	1	4.81	9	5.46	6.42	6.28	4.69	1.02	4	0.62	1	2.09	5.27	14.12	7.16	3.4	1.96	6.6	3.99	0.56	1	16.55	11.27	3.98	10.6	4.74	10.21	7.48	0.71	1
Solyc01g087340.2.1	Protein binding protein (AHR	19.55	21.02	20.18	19.58	20.08	16.3	21.22	29.2	22.8	22.38	1.11	1	17.42	17.7	20.64	18.59	12.43	17.29	14.08	14.6	0.79	1	26.98	11.28	13.74	17.33	19.8	17.9	16.62	18.11	1.04	1	48.64	27.8	26.3	34.25	15.66	22.14	18.9	0.55	0.953402
Solyc01g087350.2.1	Unknown Protein (AHRD V1)	9.21	13.65	16.29	8.68	11.96	5.12	10.9	9.87	6.61	8.12	0.68	0.962949	21.41	16	18.14	18.52	5.32	10.33	14.47	10.04	0.54	0.707665	9.92	15	7.61	10.84	9.07	4.65	4.47	6.06	0.56	1	6.54	8.91	11.8	9.08	5.62	12.11	8.87	0.98	1
Solyc01g087360.1.1	PHD finger family protein (AH	0.36	0	0.43	0	0.2	0	0.86	0.34	0	0.3	1.52	1	0	0	0.31	0.1	0.51	0	0.29	0.27	2.58	1	0.29	0.3	1.09	0.56	0.24	0.28	0.27	0.26	0.47	1	2.07	3.44	1.68	2.4	0	0.96	0.48	0.2	0.785276
Solyc01g087370.2.1	PHD finger family protein (AH	12.21	12.48	15.63	13.59	13.48	22.81	17.02	18.15	14.36	18.09	1.34	0.539721	14.98	18.06	23.25	18.76	18.91	16.27	18.78	17.99	0.96	1	15.74	15.03	19.85	16.87	12.46	12.8	14.01	13.09	0.78	1	38.41	35.09	38.34	37.28	20.21	32.79	26.5	0.71	1
Solyc01g087380.2.1	Histone-lysine N-methyltrans	9.02	12.16	11.55	12.76	11.37	10.89	8.14	13.98	9.07	10.52	0.93	1	11.75	13.89	12.84	12.83	11.98	13.74	12.76	12.83	1	1	12.28	8.19	13.05	11.17	9.31	11.72	9.01	10.01	0.9	1	22.72	16.66	22.19	20.52	16.04	19.66	17.85	0.87	1
Solyc01g087390.2.1	tRNA-dihydrouridine synthase	6.56	8.07	7.74	6	7.09	6.43	5.03	4.97	6.65	5.77	0.81	0.944449	7.54	9.56	9.45	8.85	8.7	8.39	9.1	8.73	0.99	1	6.39	6.76	8.62	7.26	7.35	5.7	7.59	6.88	0.95	1	15.91	13.03	18.69	15.88	15.19	16.24	15.71	0.99	1
Solyc01g087400.2.1	PHD finger family protein (AH	20.13	24.27	24.05	17.45	21.48	21.65	21.63	29.49	19.77	23.13	1.08	1	23.8	20.09	25.56	23.15	17.29	19.97	20.51	19.26	0.83	0.962977	23.82	14.5	21.53	19.95	20.05	16.73	19.87	18.88	0.95	1	45.05	32.35	39.34	38.91	20.57	23.23	21.9	0.56	0.640847
Solyc01g087410.2.1	Zinc finger protein (AHRD V1	10.05	10.68	13.8	10.82	11.34	8.17	10.18	10.06	10.98	9.85	0.87	1	12.55	14.08	11.52	12.72	13.52	13.04	11.43	12.66	1	1	11.75	11	14.14	12.3	12.02	10.34	11.36	11.24	0.91	1	24.73	14.27	19.83	19.61	16.2	19.06	17.63	0.9	1
Solyc01g087420.2.1	PHD zinc finger-containing pr	8.73	8.34	11.17	8.06	9.08	7.35	7.18	8.95	9.17	8.16	0.9	1	7.18	7.51	11.02	8.57	8.67	7.8	6.28	7.58	0.88	1	9.13	5.02	8.12	7.42	8.27	7.52	7.5	7.76	1.05	1	8.49	9.28	8.19	8.65	5.21	6.44	5.83	0.67	0.962653
Solyc01g087430.2.1	PHD zinc finger-containing pr	10.25	11.77	13.67	12.08	11.94	7.68	9.38	11.34	9.44	9.46	0.79	0.808915	11.06	12.59	15.75	13.13	11.34	12.46	12.32	12.04	0.92	1	11.88	9.99	12.02	11.3	9.76	9.18	10.07	9.67	0.86	1	30.74	17.32	19.08	22.38	15.85	16.68	16.27	0.73	1
Solyc01g087440.2.1	Protein EFR3 homolog B (AHF	0.33	0.3	2.35	0.47	0.86	1.11	0	1.52	0.48	0.78	0.9	1	0.31	0.86	0.28	0.48	0	0	0	0	0.02	0.702706	0	0	0	0	0	0	0.24	0.08	8	1	0	0.24	0	0.08	0	0.29	0.14	1.81	1
Solyc01g087450.2.1	Cyclin-like protein (AHRD V1	1.01	0.83	0.73	1.1	0.92	1.38	0.31	1	0.84	0.88	0.96	1	0.14	0.2	0.13	0.16	0.32	0.2	0.12	0.21	1.36	1	0.31	0.38	0.62	0.44	0	0.06	0.17	0.08	0.18	1	0	0	0	0	0	0.2	0.1	10	1
Solyc01g087460.1.1	Protein phosphatase 2C (AHR	0	0	0	0	0	0	0	0	0	0	1	NA	0	0	0	0	0	0	0	0	1	NA	0	0	0	0	0	0	0	0	1	NA	0	0.13	0	0.04	0	0	0	0.23	1
Solyc01g087470.2.1	Peptidyl-prolyl cis-trans isom	28.33	23.05	26.74	26.62	26.18	23.39	15.34	26.98	22.52	22.06	0.84	0.930443	22.02	28.67	31.96	27.55	34.53	28.71	24.97	29.4	1.07	1	33.35	44.85	57.85	45.35	55.17	55.98	50.73	53.96	1.19	1	48.63	51.15	46.99	48.92	48.53	45.95	47.24	0.97	1
Solyc01g087480.1.1	Photosystem Q(B) protein (AI	0	0	0	0	0	0	0	0	0	0	1	NA	0	0	0	0	0	0	0	0	1	NA	0	0	0	0	0	0	0	0	1	1	0	0	0	0	0	0	0	1	NA
Solyc01g087490.2.1	CONSTANS interacting protei	16	17.62	24.99	28.83	21.86	10.24	10.18	11.01	11.16	10.65	0.49	0.030476	15.53	21.12	29.76	22.14	17.01	16.93	18.82	17.59	0.79	0.896309	15.63	16.79	24.68	19.03	17.85	14.75	13.84	15.48	0.81	1	75.79	49.09	54.35	59.74	33.2	36.14	34.67	0.58	0.682649
Solyc01g087500.2.1	DNA topoisomerase 2 (AHRD	0.18	0.4	0.37	0.44	0.35	0.1	0.39	0.16	0.42	0.27	0.77	1	0.37	0.04	0.08	0.16	0.34	0.12	0.07	0.18	1.08	1	0	0.04	0	0.01	0	0.03	0.07	0.03	2.5	1	0	0	0.21	0.07	0	0	0	0.14	1
Solyc01g087510.1.1	LRR receptor-like serine/thre	0	0	0	0	0	0	0	0	0	0	1	NA	0	0	0	0	0	0	0	0	1	NA	0	0	0	0	0	0	0	0	1	NA	0	0	0	0	0	0	0	1	NA
Solyc01g087520.2.1	Ferredoxin-thioredoxin reduc	111.61	120.84	101.82	96.57	107.71	248.99	233.77	204.19	211.2	224.54	2.08	0.000129	102.75	116.7	85.52	101.66	187.55	216.26	201.35	201.72	1.98	0.000345	110.01	140.33	93.27	114.54	165.79	124.42	188.1	159.44	1.39	1	44.72	53.77	37.16	45.22	93.04	63.22	78.13	1.73	0.699553
Solyc01g087530.2.1	Zinc transporter (AHRD V1 *-	12.73	15.27	15.54	18.48	15.5	12.3	10.75	13.94	11.68	12.17	0.78	0.785515	12.77	20.69	19.63	17.7	10.21	12.09	13.92	12.07	0.68	0.345213	16.9	17.59	23.18	19.22	13.89	12.58	12.99	13.15	0.68	1	31.79	14.87	18.89	21.85	13.88	10.68	12.28	0.56	1
Solyc01g087540.2.1	Os01g0611000 protein (Fragi	23.66	25.21	29.61	39.3	29.45	29.29	37.36	19.94	21.62	27.05	0.92	1	32.02	22.44	34.98	29.81	26.36	24.32	23.67	24.78	0.83	0.962977	2.32	9.5	2.67	4.83	1.88	0.41	4.83	2.37	0.49	1	0.25	0.13	0.55	0.31	1.64	3.93	2.79	8.98	0.092353
Solyc01g087550.2.1	Nitrate reductase (AHRD V1 *	43.02	41.61	39.93	36.92	40.37	34.12	30.8	40.35	43.04	37.08	0.92	1	40.29	40.39	39.73	40.14	51.13	41.43	51.84	48.13	1.2	0.817865	40.78	42.3	33.6	38.89	35.61	36.96	35.32	35.96	0.92	1	32.63	41.04	31.76	35.14	42.8	39.48	41.14	1.17	1
Solyc01g087560.2.1	24-sterol C-methyltransferase	63.31	58.38	58.32	59.7	59.93	47.76	64.99	44.92	45.59	50.82	0.85	0.939221	57.12	51.73	63.59	57.48	39.69	50.3	57.22	49.07	0.85	0.985957	159.9	131.22	136.06	142.39	146	144.82	146.72	145.85	1.02	1	168.4	137.95	147.64	151.33	182.04	180.86	181.45	1.2	1
Solyc01g087570.2.1	Unknown Protein (AHRD V1)	14.66	15	2.59	17.63	12.47	10.59	12.73	6.74	5.79	8.96	0.72	0.919033	24.55	16.57	13.62	18.25	11.69	12.14	7.49	10.44	0.57	0.587914	5.33	8.96	10.19	8.16	15.41	11.67	12.29	13.12	1.61	1	1.04	5.32	10.14	5.5	4.7	6.43	5.56	1.01	1
Solyc01g087580.2.1	Unknown Protein (AHRD V1)	0	0	0.95	1.14	0.52	0	0.64	0	0.58	0.3	0.58	1	0	0	0.68	0.23	0.56	1.25	1.9	1.24	5.46	0.949816	0	0	1.6	0.53	1.59	0	1.76	1.12	2.09	1	1.14	10.52	2.47	4.71	1.47	1.41	1.44	0.31	1
Solyc01g087590.2.1	Polyamine oxidase (AHRD V1	1.72	2.71	4.84	2.62	2.97	0.15	1.36	1.83	0.67	1	0.34	0.164078	0.99	0.69	2.01	1.23	0.55	0.62	0.1	0.42	0.34	0.524679	0	0.32	0.26	0.19	0.26	0	0.1	0.12	0.62	1	1.51	1.06	1.02	1.2	0.97	0.35	0.66	0.55	1
Solyc01g087600.2.1	Protein E03H4.4 partially con	17.71	19.39	17.2	19.52	18.45	15.92	15.28	17.96	17.57	16.68	0.9	1	17.68	14.79	16.86	16.44	10.72	13.17	14.87	12.92	0.79	0.811508	9.75	12.1	8.87	10.24	8.33	7.89	9.24	8.49	0.83	1	8.76	7.5	9.48	8.58	9.93	11.42	10.68	1.24	1
Solyc01g087610.2.1	Alpha-N-acetylglucosaminida	9.88	10.25	11.94	11.78	10.96	9.8	8.96	11.11	9.73	9.9	0.9	1	9.8	11.64	11.17	10.87	8.95	8.88	9.24	9.02	0.83	0.962977	5.67	6.85	6.24	6.25	5.58	6.19	6.01	5.93	0.95	1	5.8	6.23	6.4	6.14	6.88	7.59	7.23	1.18	1
Solyc01g087620.2.1	Unknown Protein (AHRD V1)	3402.51	3844.87	2120.33	2652.43	3005.03	2639.95	3049.21	4455.47	4497.98	3660.65	1.22	0.72385	4048.23	4047.33	2502.37	3532.64	3779.74	3736.63	3387.19	3634.52	1.03	1	4365.63	3679.95	2907.01	3650.86	4040.98	4427.4	3653.62	4040.67	1.11	1	1051.67	3834.66	3172.52	2686.28	2353.4	2070.06	2211.73	0.82	1
Solyc01g087630.1.1	Dihydroflavonol-4-reductase	0	0.43	1.14	1.37	0.74	0	0	0.89	0.35	0.31	0.42	1	0	0	1.23	0.41	0.34	0.25	0.76	0.45	1.1	1	0	1.19	0.48	0.56	0	0.37	0.35	0.24	0.43	1	0	0.35	0	0.12	0.89	0	0.45	3.81	1
Solyc01g087640.2.1	Cinnamoyl CoA reductase-like	38.3	42.3	36.67	33.19	37.61	55.5	43.69	65.27	43.91	52.09	1.38	0.315823	43.46	48.46	37.43	43.12	51.88	53.16	59.25	54.76	1.27	0.548248	94.45	79.41	70.86	81.57	71.79	78.12	89.72	79.88	0.98	1	34.78	50.97	37.62	41.12	48.28	36.57	42.42	1.03	1
Solyc01g087650.2.1	Conserved oligomeric Golgi c	26.07	21.88	25.22	27.33	25.12	23.51	25.73	18.29	18.14	21.42	0.85	0.964968	21.7	20.87	24.63	22.4	19.51	16.81	18.69	18.34	0.82	0.896625	18.66	19.38	17.59	18.54	16.15	14.04	15.08	15.09	0.81	1	35.93	13.45	18.64	22.67	19.68	28.87	24.27	1.07	1
Solyc01g087660.2.1	Unknown Protein (AHRD V1)	2.78	1.85	2.46	6.88	3.49	2.32	1.64	0.64	0.5	1.27	0.37	0.641198	0.65	1.21	0.59	0.82	0	1.08	0	0.36	0.44	1	0.56	1.7	0	0.75	0.46	1.05	2.02	1.18	1.56	1	3.95	2.52	1.07	2.51	0.64	1.22	0.93	0.37	1
Solyc01g087670.2.1	Guanine nucleotide-binding p	15.18	14.55	19.72	17.47	16.73	17.93	14.11	20.11	14.08	16.56	0.99	1	15.27	16.17	18.84	16.76	16.63	18.58	20.38	18.53	1.11	0.976346	6.69	8.11	8.8	7.87	7.78	9.53	9.27	8.86	1.13	1	18.8	13.19	16.2	16.06	14.7	18.41	16.55	1.03	1
Solyc01g087680.2.1	Uncharacterized membrane p	25.22	31.73	17.45	13.72	22.03	73.87	47.3	87.64	38.31	61.78	2.8	0.026533	22.34	23.82	18.03	21.4	28.09	42.82	52.04	40.98	1.92	0.058438	42.33	30.63	25.52	32.83	41.27	47.43	51.96	46.89	1.43	1	11.72	28.01	25.73	21.82	21.54	16.8	19.17	0.88	1
Solyc01g087690.1.1	RNA polymerase sigma factor	0.16	0.28	0.18	0	0.16	0.17	0.12	0.14	0	0.11	0.69	1	0	0	0	0	0	0	0	0	1	NA	0	0	0	0	0	0	0	0	1	1	0	0.23	0	0.08	0	0	0	0.13	1
Solyc01g087700.1.1	Unknown Protein (AHRD V1)	0.53	0	0	0	0.13	0.59	0.42	0	0	0.25	1.91	1	0	0	0.45	0.15	0.37	0.55	0.42	0.45	2.98	1	0.43	0	0	0.14	0.35	0	0.39	0.25	1.72	1	0	0	0	0	0	0	0	1	NA
Solyc01g087710.2.1	Uncharacterized membrane p	0.3	1.06	0.7	0	0.52	0	1.18	0.82	0	0.5	0.97	1	3.33	0	0	1.11	2.07	4.02	4.69	3.59	3.24	0.373668	0	0	0	0	0.2	0.68	0	0.29	29.33	1	0	0	0	0	0	0.26	0.13	13	1
Solyc01g087720.2.1	Structural maintenance of ch	13.75	15.53	20.74	16.34	16.59	13.61	8.82	14.6	13.65	12.67	0.76	0.625626	13.37	21.55	24.65	19.86	21.2	19.05	16.43	18.89	0.95	1	20.69	25.27	21.93	22.63	24.12	18.93	20.54	21.2	0.94	1	35.34	24.03	33.98	31.12	32.01	31.43	31.72	1.02	1
Solyc01g087730.2.1	50S ribosomal protein L1 (AH	149.65	142.37	136.61	109.83	134.62	209.18	193.43	202.37	204.54	202.38	1.5	0.076427	119.29	168.15	146.12	144.52	283.31	218.25	215.03	238.86	1.65	0.013966	36.16	68.51	48.48	51.05	83.1	77.58	102.25	87.64	1.72	0.095296	122.02	148.83	137.06	135.97	141.09	148.62	144.86	1.07	1
Solyc01g087740.1.1	Subtilisin-like protease (AHRD	0	0.08	0	0	0.02	0	0	0	0.06	0.01	0.75	1	0	0	0	0	0	0	0	0	1	NA	0.07	0	0	0.02	0	0	0	0	0.43	1	0	0	0	0	0	0	0	1	NA
Solyc01g087750.2.1	Homology to unknown gene (	0.17	0.46	0.2	0.24	0.27	2.12	2.6	1.43	1.99	2.04	7.61	0.00493	1.29	0.6	0.29	0.73	2.4	2.69	3.81	2.97	4.08	0.016393	0	0	0.17	0.06	0	0	0	0	0.18	NA	0	0	0	0	0	0	0	1	NA
Solyc01g087760.2.1	Subtilisin-like protease (AHRD	0	0	0	0	0	0	0	0	0	0	1	NA	0	0	0	0	0	0	0	0	1	NA	0	0	0	0	0	0	0	0	1	NA	0	0	0	0	0	0	0	1	NA
Solyc01g087770.2.1	Subtilisin-like protease (AHRD	0	0	0	0.24	0.06	0	0	0	0	0	0.17	1	0	0	0	0	0	0	0	0	1	NA	0	0.14	0	0.05	0	0	0	0	0.21	1	0	0.12	0	0.04	0	0	0	0.25	1
Solyc01g087780.2.1	Subtilisin-like protease (AHRD	1.86	1.96	5.49	6.58	3.97	1.5	2.14	1.48	2.91	2.01	0.51	0.772659	2.67	1.54	2.86	2.36	3.34	2.58	1.79	2.57	1.09	1	0.2	0.69	0.42	0.44	0.22	0.06	0.25	0.18	0.4	1	0	0.18	0.13	0.1	0	0	0	0.1	1
Solyc01g087790.2.1	Subtilisin-like protease (AHRD	7.95	9.11	15.27	19.86	13.05	6.21	10.22	9.8	10.26	9.12	0.7	0.917957	9.36	5.96	10.07	8.46	11.03	10.9	9.9	10.61	1.25	0.655817	1.16	3.38	1.26	1.93	0.83	0.45	1.66	0.98	0.51	1	0	0	0.26	0.09	0.08	0	0.04	0.46	1
Solyc01g087800.2.1	Subtilisin-like protease (AHRD	0.85	1.05	1.7	0.48	1.02	0.09	0.47	0.31	0.49	0.34	0.33	0.196453	0.79	0.74	1.64	1.06	2.7	1.62	1.4	1.91	1.8	0.556057	0.27	0.48	0.17	0.31	0	0	0.31	0.1	0.34	1	0	0.06	0	0.02	0	0	0	0.5	1
Solyc01g087810.2.1	Subtilisin-like protease (AHRD	0.34	0.38	0.2	0.12	0.26	0	0.07	0	0	0.02	0.07	0.254994	0.08	0.15	0	0.08	0.53	0.18	0.67	0.46	6	0.288833	0	0	0	0	0	0	0	0	1	1	0	0	0	0	0	0	0	1	NA
Solyc01g087820.2.1	Subtilisin-like protease (AHRD	7.55	10.94	15.71	17.75	12.99	4.81	5.42	4.37	7.91	5.63	0.43	0.126007	13.41	10.32	12.89	12.21	9.82	7.02	6.33	7.72	0.63	0.217609	1.71	6.43	1.52	3.22	0.84	0.39	2.1	1.11	0.34	1	0.12	0.12	0.39	0.21	0	0	0	0.05	1
Solyc01g087830.1.1	Subtilisin-like protease (AHRD	0	0	0.31	0	0.08	0	0	0	0	0	0.13	1	0	0	0	0	0	0	0	0	1	NA	0	0	0	0	0	0	0	0	1	NA	0	0	0	0	0	0	0	1	NA
Solyc01g087840.2.1	Subtilisin-like protease (AHRD	0.43	0.46	1.12	1.22	0.81	0.19	0.27	0.56	0.37	0.35	0.43	0.574209	0.88	0.38	0.36	0.54	0.66	0.58	0.34	0.53	0.98	1	0	0.14	0	0.05	0.11	0	0	0.04	0.79	1	0	0.06	0	0.02	0	0	0	0.5	1
Solyc01g087850.2.1	Subtilisin-like protease (AHRD	21.47	19.8	22.68	37.47	25.35	22.23	27.89	13.13	14.67	19.48	0.77	0.960368	18.76	13.17	21.64	17.86	9.43	10.04	11.36	10.28	0.58	0.080922	0.42	2.89	0.09	1.13	0.06	0.26	0.38	0.23	0.21	1	0.86	0.5	1.33	0.9	0.08	0.23	0.15	0.17	0.622578
Solyc01g087860.2.1	Unknown Protein (AHRD V1)	7.67	7.17	6.79	4.34	6.49	8.53	5.45	11.28	4.13	7.35	1.13	1	4.99	3.67	3.24	3.97	1.86	2.18	0.9	1.65	0.42	0.552886	11.47	7.81	6.47	8.58	4.54	7.85	4.47	5.62	0.65	1	2.18	5.29	1.77	3.08	3.16	6.39	4.78	1.55	1
Solyc01g087870.2.1	Glucosamine--fructose-6-pho	3.5	4.33	2.07	4.95	3.71	2.06	3.84	2.42	2.79	2.78	0.75	0.863793	2.62	4.82	4.11	3.85	2.97	3.07	4.44	3.49	0.91	1	1.57	2.62	1.35	1.85	2.11	1.7	2.34	2.05	1.11	1	1.25	2.19	2.69	2.04	1.6	3.07	2.33	1.14	1
Solyc01g087880.2.1	Unknown Protein (AHRD V1)	61.06	46.22	40.57	49.64	49.37	53.83	55.96	90.34	58.95	64.77	1.31	0.533277	63.98	48.2	34.38	48.85	39.43	47.6	58.46	48.5	0.99	1	53.87	52.61	28.56	45.01	43.34	49.47	48.07	46.96	1.04	1	14.97	50.46	41.01	35.48	48.83	35.69	42.26	1.19	1
Solyc01g087890.2.1	Choline transporter-like prote	14.9	17.05	24.97	12.67	17.4	16.31	13.97	23.17	16.4	17.46	1	1	14.48	12.56	15.33	14.12	15.54	14.27	16.02	15.28	1.08	1	7.15	10.95	7.12	8.41	7.14	6.79	8.81	7.58	0.9	1	4.78	5.78	8.27	6.28	5.54	5.89	5.71	0.91	1
Solyc01g087900.2.1	N-alpha-acetyltransferase 20,	264.93	221.26	179.42	213.15	219.69	215.36	260.96	252.2	246.53	243.76	1.11	0.878093	251.92	256.25	215.26	241.14	261.77	262.65	259.22	261.21	1.08	0.979666	207.58	265.68	173.36	215.54	232.2	276.06	216.59	241.62	1.12	1	211.29	263.28	237.06	237.21	235.52	245.07	240.3	1.01	1
Solyc01g087910.2.1	F-box/WD-40 repeat-containi	15.34	18.56	15.99	12.64	15.63	21.19	10.93	18.9	14.97	16.5	1.06	1	16.9	19.55	21.38	19.28	23.34	19.43	20.83	21.2	1.1	1	9.99	9.83	9.46	9.76	8.8	10.5	11.29	10.2	1.04	1	15.67	14.39	11.22	13.76	17.31	15.54	16.42	1.19	1
Solyc01g087920.1.1	Serine carboxypeptidase 1 (A	0	0.34	0.3	0	0.16	0	0.3	0	0	0.07	0.47	1	0	0	0	0	0	0.07	0	0.02	2.33	1	5.23	1.76	3.27	3.42	1.5	3.75	3.79	3.01	0.88	1	0	0.37	0	0.12	0	0	0	0.08	1
Solyc01g087930.1.1	Unknown Protein (AHRD V1);	4.66	0.69	7.33	4.39	4.27	4.31	0.61	2.14	1.67	2.18	0.51	0.813312	2.89	5.4	8.52	5.6	6.46	2.41	2.44	3.77	0.67	1	1.25	3.16	2.31	2.24	2.04	1.18	3.39	2.2	0.98	1	1.1	1.13	2.39	1.54	3.55	4.76	4.15	2.7	1
Solyc01g087940.1.1	Serine carboxypeptidase 1 (A	0	0	0	0	0	0	0	0	0	0	1	NA	0	0	0	0	0	0	0.11	0.04	3.67	1	6.81	3.16	8.63	6.2	4.92	4.16	5.85	4.98	0.8	1	0.38	1.17	0	0.52	0.24	0	0.12	0.23	1
Solyc01g087950.2.1	Serine carboxypeptidase 1 (A	0.13	0	0.47	0.19	0.2	0.59	1.37	0.49	0.1	0.64	3.23	0.561369	0.62	1.16	0.34	0.71	2.41	1.39	0.63	1.48	2.09	0.737828	123.68	64.44	135.75	107.96	74.28	105.48	144.51	108.09	1	1	3.81	14.19	8.23	8.74	2.69	1.76	2.23	0.25	0.460838
Solyc01g087960.1.1	Serine carboxypeptidase 1 (A	0	0	0	0	0	0	0	0	0	0	1	NA	0	0	0	0	0	0	0	0	1	NA	0	0	0	0	0	0	0	0	1	NA	0	0	0	0	0	0	0	1	NA
Solyc01g087970.2.1	Serine carboxypeptidase 1 (A	0.26	0.35	0.46	0.37	0.36	0.44	1.34	0.12	0.47	0.59	1.65	0.901356	0.12	0	0	0.04	0.27	0	0.1	0.12	3.08	1	0.11	0.21	0.26	0.19	0.17	0.2	0	0.12	0.64	1	0	0	0	0	0	0	0	1	NA
Solyc01g087980.2.1	Unknown Protein (AHRD V1)	6.24	5.34	3.68	3.73	4.75	4.8	2.08	6.62	5.17	4.67	0.98	1	3.57	3.96	5.67	4.4	2.83	4.6	4.15	3.86	0.88	1	7.56	4.3	9.05	6.97	2.05	3.27	4.2	3.17	0.46	1	359.84	8.54	14.76	127.71	10.76	5.05	7.9	0.06	1
Solyc01g087990.2.1	MADS-box transcription facto	18.92	15.33	15.45	10.29	15	13.75	13.02	15.83	10.27	13.22	0.88	0.98862	17.37	36.9	40.97	31.75	49.46	40.44	40.79	43.56	1.37	0.59286	42.72	49.61	32.98	41.77	48.6	43.94	51.26	47.93	1.15	1	70.69	75.04	74.97	73.57	53.51	56.78	55.14	0.75	1
Solyc01g088000.2.1	Methyltransferase (AHRD V1	33.86	40.85	49.42	47.9	43.01	37.36	25.94	50.51	31.15	36.24	0.84	0.926661	43.4	51.9	50	48.43	45.76	44.94	43.76	44.82	0.93	1	20.87	25.14	20.91	22.31	23.07	16.35	15.54	18.32	0.82	1	15.34	35.16	32.02	27.51	21.81	25.95	23.88	0.87	1
Solyc01g088010.1.1	Lactoylglutathione lyase fami	0.56	0.5	0	0.4	0.36	0.31	0.66	0.51	1.81	0.82	2.25	0.833584	0.26	0.24	0.24	0.25	0.58	0.58	0.44	0.53	2.16	1	0	0	0	0	0	0	0	0	1	NA	0	0	0	0	0	0	0	1	NA
Solyc01g088020.2.1	Protein transport protein sec	65.91	58.92	67.94	82.53	68.82	49.41	77.41	47.88	65.17	59.97	0.87	1	71.51	60.46	65.23	65.73	54.26	54.08	54.38	54.24	0.83	0.896625	62.92	77.22	72.53	70.89	63.91	55.38	65	61.43	0.87	1	75.37	59.84	60.6	65.27	66.24	71.31	68.78	1.05	1
Solyc01g088030.2.1	Structure-specific endonuclea	3.28	2.04	2.71	3.71	2.94	3.28	3.37	5.43	4.01	4.02	1.37	0.836333	3.05	5.14	6.93	5.04	7.97	7.14	5.93	7.01	1.39	0.830032	11.67	15.24	14.01	13.64	11.43	13.93	18.42	14.59	1.07	1	10.27	15.49	13.12	12.96	14.42	10.07	12.25	0.94	1
Solyc01g088040.2.1	Unknown Protein (AHRD V1)	1799.69	1746.15	1539.52	1793.53	1719.72	1265.71	1922.89	1415.72	1666.21	1567.63	0.91	1	1871.63	1702.07	1427.57	1667.09	1474.26	1407.97	1447.51	1443.25	0.87	1	1768.45	2020.21	1706.47	1831.71	1479.58	1555.05	1659.51	1564.71	0.85	1	1585.18	1616.66	1525.76	1575.87	1500.1	1640.42	1570.26	1	1
Solyc01g088050.2.1	Receptor-like protein kinase	2.07	1.72	1.79	1.56	1.79	1.99	2.5	1.77	1.29	1.89	1.06	1	1.67	2.04	1.74	1.82	1.72	2.42	1.73	1.96	1.08	1	3.34	5.16	2.46	3.65	2.71	3.65	3.71	3.36	0.92	1	1.76	3	1.27	2.01	2.77	2.05	2.41	1.2	1
Solyc01g088060.2.1	Nbs-lrr, resistance protein	1.23	1.78	1.36	2.18	1.64	1.37	1.4	1.7	1.6	1.52	0.93	1	1.43	1.6	1.1	1.38	2.13	2.11	2.11	2.12	1.54	0.566844	2.05	1.38	1.53	1.65	1.06	1.63	1.51	1.4	0.85	1	0.87	1.62	0.59	1.03	1.13	1.15	1.14	1.11	1
Solyc01g088070.2.1	Nbs-lrr resistance protein (AH	1.19	0.35	0.47	1.69	0.92	1.77	1.57	1.83	0.29	1.36	1.48	1	0.37	1.73	1.34	1.15	1.65	3.5	0.62	1.92	1.68	0.952994	1.28	1.94	0.39	1.2	1.83	0.6	1.45	1.29	1.07	1	0	1.15	0	0.38	1.09	0.7	0.9	2.33	1
Solyc01g088080.2.1	T-complex protein theta subu	62.44	60.12	66.34	66.63	63.88	52.63	52.08	56.85	58.29	54.96	0.86	0.980103	67.82	58.83	54.49	60.38	53.02	58.67	60.74	57.48	0.95	1	44.74	66.25	45.31	52.1	55.81	45.39	54.55	51.92	1	1	56.07	52.34	52.41	53.61	70.2	75.34	72.77	1.36	0.985446
Solyc01g088090.2.1	Hydrolase alpha/beta fold far	38.32	43.95	45.06	22.01	37.34	90.91	37.77	102.28	77.09	77.01	2.06	0.123847	57.42	42.03	28.24	42.56	66.45	59.99	79.03	68.49	1.61	0.099449	397.4	290.48	205.76	297.88	225.99	257.22	293.32	258.84	0.87	1	6.4	65.94	22.94	31.76	120.9	51.53	86.22	2.71	1
Solyc01g088100.2.1	Zinc finger CCCH domain-cont	43.08	52.06	54.87	49.08	49.77	31.76	30.38	44.99	38.05	36.3	0.73	0.432083	37.39	40.82	48.34	42.18	46.07	47.78	40.16	44.67	1.06	1	42.09	32.31	32.65	35.68	51.57	42.21	42.69	45.49	1.27	1	101.61	64.28	86.48	84.12	57.01	71.43	64.22	0.76	1
Solyc01g088110.1.1	Unknown Protein (AHRD V1)	0	0	0	0	0	0	0	0	0	0	1	NA	0	0	0	0	0	0	0	0	1	NA	0	0	0	0	0	0	0	0	1	NA	0	0	0	0	0	0	0	1	NA
Solyc01g088120.1.1	Unknown Protein (AHRD V1)	0	0	0	0	0	0	0	0	0	0	1	NA	0	0	0	0	0	0	0	0	1	NA	0	0	0	0	0	0	0	0	1	NA	0	0	0	0	0	0	0	1	NA
Solyc01g088130.2.1	CST complex subunit STN1 (A	4.4	3.91	4.72	2.26	3.82	3.56	4.1	2.57	4.02	3.56	0.93	1	2.98	3.13	5.07	3.73	4.44	4.35	4.08	4.29	1.15	1	2.26	3.58	2.38	2.74	1.84	2.12	4.08	2.68	0.98	1	1.14	2.61	0.61	1.45	1.46	4.91	3.19	2.19	1
Solyc01g088140.2.1	Unknown Protein (AHRD V1)	33.17	41.13	35.18	42.18	37.92	38.53	31.78	40.44	38.8	37.39	0.99	1	39.52	36.29	37.15	37.65	50.93	38.02	38.08	42.34	1.12	0.972437	42.16	39.48	28.86	36.83	38.93	40.96	41.83	40.57	1.1	1	42.92	66.32	52.16	53.8	43.67	42.48	43.08	0.8	1
Solyc01g088150.2.1	Glycerol-3-phosphate dehydr	13.34	13.13	10.71	15.45	13.16	6.62	11.42	7.83	9.88	8.94	0.68	0.496226	11.35	16.53	11.98	13.29	8.86	11.24	12.6	10.9	0.82	0.962778	5.16	9.71	5.63	6.83	10.25	4.52	6.82	7.2	1.05	1	6.25	17	15.49	12.91	9.22	11.44	10.33	0.8	1
Solyc01g088160.2.1	Cytokinin oxidase/dehydroge	0.12	0.33	0.58	0.35	0.34	0.41	0.29	0.57	0.44	0.43	1.24	1	0	0	0	0	0	0	0	0	1	NA	0	0	0	0	0.08	0	0	0.03	2.67	1	0	0	0	0	0	0	0	1	NA
Solyc01g088170.2.1	Aldehyde oxidase (AHRD V1 *	12.39	14.08	19.34	14.53	15.09	12.97	13.36	12.46	16.38	13.79	0.91	1	15.39	16.01	17.6	16.33	17.66	16.8	18.23	17.56	1.08	1	10.44	10.33	10.44	10.4	12.32	11.81	10.23	11.45	1.1	1	12.55	18.67	16.46	15.89	14.57	13.49	14.03	0.88	1
Solyc01g088180.1.1	Aldehyde oxidase (AHRD V1 *	0	0	0	0	0	0	0	0	0	0	1	NA	0	0	0	0	0	0	0	0	1	NA	0	0	1.01	0.34	0	0	0	0	0.03	NA	0	0	0	0	0	0	0	1	NA
Solyc01g088190.1.1	Unknown Protein (AHRD V1)	0	0	0	0	0	0	0	0	0	0	1	NA	0	0	0	0	0	0	0	0	1	NA	0	0	0	0	0	0	0	0	1	NA	0	0	0	0	0	0	0	1	NA
Solyc01g088200.2.1	Aldehyde oxidase (AHRD V1 *	2.99	5.2	5.36	2.94	4.12	2.74	5.84	3.94	4.85	4.34	1.05	1	7.73	5.87	5.81	6.47	5.04	5.11	5.51	5.22	0.81	0.990265	3.36	2.65	2.84	2.95	4.86	4.53	2.93	4.11	1.39	1	1.29	2.17	1.4	1.62	4.04	4.78	4.41	2.72	0.49975
Solyc01g088210.2.1	Xanthine dehydrogenase (Fra	8.67	15.1	15.57	8.53	11.97	7.96	16.96	11.44	14.07	12.61	1.05	1	22.44	17.04	16.88	18.79	14.64	14.83	16	15.16	0.81	0.990265	9.75	7.68	8.23	8.55	14.12	13.14	8.52	11.93	1.39	1	3.75	6.3	4.06	4.7	11.73	13.88	12.8	2.72	0.49975
Solyc01g088220.2.1	Aldehyde oxidase (AHRD V1 *	1.78	3.97	3.16	1.26	2.54	1.98	7.74	2.46	3.84	4	1.58	0.951897	9.95	5.42	3.76	6.38	3.09	2.77	4.9	3.59	0.56	0.900797	2.16	0.73	2.65	1.85	5.86	4.73	2.6	4.4	2.38	1	0	1.3	1.37	0.89	2.45	3.13	2.79	3.13	1
Solyc01g088230.2.1	Xanthine dehydrogenase/oxid	5.34	9.3	9.59	5.26	7.37	4.91	10.45	7.05	8.67	7.77	1.05	1	13.83	10.5	10.4	11.58	9.02	9.14	9.86	9.34	0.81	0.990265	6.01	4.73	5.07	5.27	8.7	8.1	5.25	7.35	1.39	1	2.31	3.88	2.5	2.9	7.23	8.55	7.89	2.72	0.49975
Solyc01g088240.2.1	F-box family protein (AHRD V	41.77	40.6	45.49	38.17	41.51	40.24	34.33	39.98	28.6	35.79	0.86	0.930443	46.82	48.78	52.46	49.35	31.94	31.72	32.32	31.99	0.65	0.201318	67.32	57.95	66.76	64.01	52.63	53.74	43.86	50.08	0.78	1	47.81	37.96	35.57	40.45	36.08	36.62	36.35	0.9	1
Solyc01g088250.2.1	IQ-domain 11 (AHRD V1 ***-	62.09	66.13	63.08	47.17	59.62	77.97	116.31	114.18	106.59	103.76	1.74	0.004807	132.62	99.2	93.96	108.59	123.91	155.98	140.4	140.1	1.29	0.33741	20.09	18.97	7.56	15.54	31.44	19.94	21.05	24.14	1.55	0.619968	12.06	16.28	10.83	13.06	13.29	18.9	16.09	1.23	1
Solyc01g088260.2.1	Germin-like protein 5 (AHRD	0	0	0	0	0	0	0	0	0	0	1	NA	0	0	0.52	0.17	0	0	0	0	0.06	1	0	0	0	0	0	0	0	0	1	NA	0	0	0	0	0	0	0	1	NA
Solyc01g088270.2.1	Germin-like protein subfamily	0	0	0	0	0	0	0	0	0	0	1	NA	0	0	0	0	0	0	0	0	1	NA	0	0	0	0	0	0	0	0	1	NA	0	0	0	0	0	0	0	1	NA
Solyc01g088280.1.1	Germin-like protein 6 (AHRD	0	0	0	0	0	0	0	0	0	0	1	NA	0	0	0	0	0	0	0	0	1	NA	0	0	0	0	0	0	0	0	1	NA	0	0	0	0	0	0	0	1	NA
Solyc01g088290.1.1	Germin-like protein 5 (AHRD	0	0	0	0	0	0	0	0	0	0	1	NA	0	0	0	0	0	0	0	0	1	NA	0	0	0	0	0	0	0	0	1	NA	0	0	0	0	0	0	0	1	NA
Solyc01g088300.2.1	Germin-like protein 6 (AHRD	0	0	0	0	0	0	0	0	0	0	1	NA	0	0	0	0	0	0	0	0	1	NA	0	0	0	0	0	0	0	0	1	NA	0	0	0	0	0	0	0	1	NA
Solyc01g088310.2.1	Geranylgeranyl reductase (AH	18.83	14.55	16.19	28.08	19.41	16.71	30.3	17.71	24.52	22.31	1.15	0.794182	17.65	17.76	22.81	19.41	18.02	21.39	22.14	20.52	1.06	1	7.19	13.92	11.73	10.95	20.14	15.27	15.95	17.12	1.56	0.720131	11.41	17.39	15.94	14.91	20.17	24.44	22.3	1.5	0.958249
Solyc01g088320.1.1	AT5g06970/MOJ9_14 (AHRD	1.2	4.27	1.42	0	1.72	1.33	0	0	1.72	0.76	0.44	0.995762	1.12	2.09	3.04	2.08	2.5	0.62	1.89	1.67	0.8	1	0.97	0	0	0.32	1.58	0	0	0.53	1.63	1	0	0	0	0	0	1.05	0.53	52.5	1
Solyc01g088330.1.1	Unknown Protein (AHRD V1)	1.35	2.4	1.59	1.9	1.81	1.5	3.19	1.24	1.93	1.96	1.09	1	2.5	1.17	2.27	1.98	0	2.79	1.06	1.28	0.65	1	1.09	0	0	0.36	2.65	0	0.98	1.21	3.33	1	0	0.98	0	0.33	1.23	0	0.61	1.88	1
Solyc01g088340.1.1	Unknown Protein (AHRD V1)	7.13	3.17	0	0	2.58	3.96	1.41	0	0	1.34	0.52	0.993713	1.66	0	1.51	1.06	0	1.85	1.4	1.08	1.03	1	0	1.45	1.77	1.07	0	0	0	0	0.01	1	0	3.89	0	1.3	0	1.56	0.78	0.6	1
Solyc01g088350.2.1	NADH dehydrogenase (AHRD	26.69	28.85	23.39	26.43	26.34	32.64	26.19	28.75	28.75	29.08	1.1	0.994784	30.15	27.84	15.78	24.59	19.31	20.34	22.18	20.61	0.84	1	33.58	27.03	21.95	27.52	16.28	28.9	23.34	22.84	0.83	1	15.18	21.05	17.01	17.75	14.66	18.72	16.69	0.94	1
Solyc01g088360.2.1	Glucosamine--fructose-6-pho	0	0.11	0.14	0.17	0.11	0	0	0.11	0.09	0.05	0.48	1	0	0	0.1	0.03	0.25	0.06	0.19	0.17	5	0.998743	0.1	0.29	0	0.13	0.24	0.09	0.26	0.2	1.51	1	0.17	0.09	0	0.09	0	0.21	0.1	1.21	1

Solyc01g088370.2.1	Eukaryotic translation initiati	95.19	90.2	108.18	97.34	97.73	61.89	69.93	78.23	82.06	73.03	0.75	0.542618	104.53	101.92	104.85	103.77	95.91	91.39	89.1	92.13	0.89	1	93.24	108.41	99.86	100.5	107.32	85.59	98.69	97.2	0.97	1	113.65	142.14	153.16	136.32	95.63	122.69	109.16	0.8	1
Solyc01g088380.1.1	DNA helicase (AHRD V1 ***-	0.65	0.78	0.49	0.72	0.66	0.41	0.84	0.72	0.93	0.72	1.1	1	0.52	0.2	0.31	0.34	0.22	0.26	0.11	0.2	0.57	0.96732	0.04	0.34	0	0.13	0.06	0	0.03	0.03	0.24	1	0	0	0.07	0.02	0	0	0	0.43	1
Solyc01g088390.2.1	Sister chromatid cohesion pro	1.82	2.65	4.49	3.51	3.12	3.31	3.92	4.26	2.73	3.56	1.14	0.977921	2.92	1.72	2.93	2.52	1.03	1.28	0.78	1.03	0.41	0.314249	10.55	2.43	11.16	8.05	1.85	2.76	2.41	2.34	0.29	1	1.88	0.84	1.27	1.33	2.88	3.33	3.1	2.33	0.715254
Solyc01g088400.2.1	CER1 (AHRD V1 **-- B6TFH3_	11.33	16.99	11.63	8.16	12.03	19.12	6.54	13.4	14.38	13.36	1.11	1	3.71	1.55	2.57	2.61	0.73	0.38	1.65	0.92	0.35	0.200073	1104.95	210.67	1859.94	1058.52	237.5	644.61	889.41	590.51	0.56	1	202.95	688.56	792.38	561.3	463.35	213.16	338.25	0.6	1
Solyc01g088410.2.1	CER1 (AHRD V1 **-- B6TFH3_	0	0	0	0	0	0	0	0	0	0	1	NA	0	0	0	0	0	0	0	0	1	NA	0.17	0	0	0.06	0	0	0	0	0.18	1	0	0.15	0	0.05	0	0	0	0.2	1
Solyc01g088420.2.1	CER1 protein (AHRD V1 **-- (	0	0	0	0	0	0	0	0	0	0	1	NA	0	0	0	0	0	0	0	0	1	NA	0	0	0	0	0	0	0	0	1	NA	0	0	0	0	0	0	0	1	NA
Solyc01g088430.2.1	CER1 (AHRD V1 **-- B6TFH3_	0	0	0	0	0	0	0.08	0	0	0.02	2	1	0	0	0.09	0.03	0	0.05	0	0.02	0.56	1	0	0	0.1	0.03	0	0.08	0	0.03	0.8	NA	0	0	0	0	0	0	0	1	NA
Solyc01g088440.2.1	Ring H2 finger protein (AHRD	2.27	5.26	10.2	4.5	5.56	4.05	4.67	2.93	3.92	3.89	0.7	0.953808	2.96	1.58	3.07	2.54	1.58	2.12	3.93	2.54	1	1	2.94	1.48	2.26	2.23	0.9	3.79	2.65	2.45	1.1	1	16.82	3.63	4.9	8.45	3.75	3.19	3.47	0.41	1
Solyc01g088450.2.1	RING finger-like (AHRD V1 *-	9.97	5.07	5.6	9.4	7.51	3.69	6	3.05	4.77	4.38	0.58	0.681782	4.41	9.49	6.01	6.64	10.86	9.83	3.73	8.14	1.23	1	2.3	5.41	5.65	4.45	4.05	2.52	2.08	2.88	0.65	1	9.45	5.86	8.03	7.78	5.21	4.99	5.1	0.66	1
Solyc01g088460.2.1	Unknown Protein (AHRD V1)	68.76	96.75	53.72	86.74	76.49	123.94	128.37	113.62	94.72	115.16	1.51	0.195902	83.84	89.66	106.73	93.41	85.71	117.87	100.77	101.45	1.09	1	39.04	57.54	35.05	43.88	32.35	41.55	53.49	42.46	0.97	1	2.64	11.47	14.29	9.47	17.86	26.88	22.37	2.36	0.747913
Solyc01g088470.2.1	50S ribosomal protein L18 far	31.18	38.61	32.52	48.58	37.72	61.78	62.43	54.01	48.98	56.8	1.51	0.144947	36.15	40.06	52.29	42.83	34.16	51.94	42.26	42.79	1	1	18.98	26.14	19.29	21.47	17.81	19.52	24.7	20.68	0.96	1	3.85	6.89	4.17	4.97	11.17	10.7	10.93	2.2	0.874234
Solyc01g088480.2.1	Adenylate kinase (AHRD V1 *	101.47	84.16	80.06	91.09	89.19	75.08	79.61	75.96	84.35	78.75	0.88	1	77.73	58.71	55.99	64.14	33.77	32.37	36.79	34.31	0.53	0.019483	21.83	27.65	18.09	22.52	20.84	11.19	18.31	16.78	0.75	1	4.47	8.21	6.28	6.32	5.75	10.74	8.25	1.3	1
Solyc01g088490.2.1	Pentatricopeptide repeat-con	0.59	1.23	2.1	0.56	1.12	1.54	1.09	1.27	1.28	1.29	1.16	1	1.29	1.89	1.67	1.62	1.92	1.84	1.4	1.72	1.06	1	1.12	1.45	1.18	1.25	1.43	0.9	1.44	1.26	1.01	1	0.28	1.43	1.21	0.97	1.63	1.04	1.33	1.37	1
Solyc01g088500.2.1	Genomic DNA chromosome 5	0	0	0.26	0	0.07	0	0	0	0.16	0.04	0.62	1	0	0.19	0	0.06	0	0	0	0	0.16	1	0	0	0.22	0.07	0	0	0	0	0.14	1	0	0.16	0	0.05	0	0.19	0.1	1.78	1
Solyc01g088510.2.1	Dynamin like protein (AHRD V	43.72	44.75	55.68	61.3	51.36	33.16	39.7	30.39	41.55	36.2	0.7	0.462768	46.89	49.75	55.18	50.61	29.7	31.29	33.55	31.51	0.62	0.09382	48.93	51.24	60.5	53.56	45.87	47.27	40.86	44.67	0.83	1	62.71	68	63.08	64.6	61.69	59.98	60.83	0.94	1
Solyc01g088520.2.1	Dynamin-related protein 1A (	13.65	18.22	13.42	20.92	16.55	15.17	12.57	17.78	13.07	14.65	0.88	1	7.41	23.73	8.65	13.26	10.26	18.85	16.09	15.07	1.14	1	19.3	12.98	19.19	17.16	18.68	15.52	19.89	18.03	1.05	1	8.09	21.48	31.48	20.35	3.12	12.96	8.04	0.4	0.941082
Solyc01g088530.2.1	Transposon protein (AHRD V1	4.48	2.85	2.52	6.34	4.05	3.32	2.02	2.36	1.53	2.31	0.57	0.535709	0.6	2.41	1.44	1.48	1.33	1.33	1.51	1.39	0.94	1	0.52	1.22	1.91	1.22	1.68	1.62	0.93	1.41	1.16	1	2.12	2.64	2.63	2.46	2.54	2.43	2.49	1.01	1
Solyc01g088540.1.1	Unknown Protein (AHRD V1);	0	0	0	0	0	0	0	0	0	0	1	NA	0	0	0	0	0	0	0	0	1	NA	0	0	0	0	0	0	0	0	1	NA	0	0	0	0	0	0	0	1	NA
Solyc01g088550.2.1	Purine permease family prote	16.27	19.62	17.49	23.27	19.16	13.86	10.42	13.46	9.34	11.77	0.61	0.167623	13.62	17.91	16.33	15.95	11.66	13.1	20.17	14.98	0.94	1	16.65	14.73	19.02	16.8	12.82	10.96	10.67	11.48	0.68	1	5.4	18.25	14.17	12.61	10.09	10.14	10.12	0.8	1
Solyc01g088560.2.1	Ras-related protein Rab-6A (A	122.47	116.87	108.74	150.91	124.75	94.03	150.52	118.12	126.68	122.34	0.98	1	126.55	130.88	145.3	134.24	93.92	104.76	99.47	99.38	0.74	0.560276	101.5	142.75	128.46	124.24	115.09	104.07	112.71	110.62	0.89	1	203.3	149.48	160.18	170.99	163.55	166.61	165.08	0.97	1
Solyc01g088570.2.1	Acyl carrier protein (AHRD V1	93.32	93.08	96.82	122.9	101.53	90.03	99.93	71.51	97.03	89.62	0.88	1	92.3	100.24	53	81.85	61.38	64.15	63.65	63.06	0.77	0.890011	122.63	137.67	95.83	118.71	103.59	102.85	113.74	106.73	0.9	1	52.62	54.54	53.61	53.59	80.52	67.72	74.12	1.38	1
Solyc01g088580.1.1	Invertase inhibitor (AHRD V1	0	0	0	0	0	0	0	0	0	0	1	NA	0	0	0	0	0	0	0	0	1	NA	0	0	0	0	0	0	0	0	1	NA	0	0	0	0	0	0	0	1	NA
Solyc01g088590.2.1	Invertase inhibitor (AHRD V1	6	11.93	5.41	2.5	6.46	3.92	2.51	3.24	1.52	2.8	0.43	0.302883	13.78	8.28	4.47	8.84	1.71	4.02	5.82	3.85	0.44	0.61118	179.28	133.1	145.32	152.57	112.17	151.61	190.5	151.43	0.99	1	12.54	53.81	51.54	39.3	39.72	16.39	28.05	0.71	1
Solyc01g088600.2.1	PDE320 (AHRD V1 ***- D7L6	11.21	12.02	10.34	13.01	11.64	20.45	15.66	11.89	13.62	15.4	1.32	0.500597	8.02	9.12	11.05	9.4	16.45	13.47	11.41	13.78	1.47	0.29066	1.28	2.93	1.14	1.78	1.89	1.53	2.93	2.12	1.19	1	1.23	3.03	3.09	2.45	2.24	6.68	4.46	1.82	1
Solyc01g088610.2.1	chaperonin (AHRD V1 ***- B5	217.32	196.86	143.07	168.79	181.51	330.53	417.04	305.96	251.44	326.24	1.8	0.008217	154.31	111.32	120.46	128.7	144	197.91	186.79	176.23	1.37	0.229965	132.07	227.68	148.3	169.35	188.65	175.01	191.94	185.2	1.09	1	44.83	138.46	120.1	101.13	166.07	156.09	161.08	1.59	0.942876
Solyc01g088620.2.1	Ferredoxin-like (AHRD V1 ***	1.42	3.28	3.34	1.6	2.41	5.04	3.8	2.08	3.25	3.54	1.47	0.83844	2.9	1.23	0.96	1.7	1.18	0.88	1.11	1.06	0.62	1	0	0.46	0	0.15	0.56	0	0.62	0.39	2.57	1	0	0	0.44	0.15	0	0	0	0.07	1
Solyc01g088630.1.1	RING finger protein (AHRD V1	0.36	0	0.21	0.25	0.2	0	0.28	0	0.13	0.1	0.5	1	0.17	0.16	0.15	0.16	0.25	0	0	0.08	0.52	1	0.14	0	0	0.05	0	0.27	0	0.09	1.93	1	0	0	0	0	0	0	0	1	NA
Solyc01g088640.2.1	Ring finger protein 12 (AHRD	10.97	14.41	12.63	10.6	12.15	12.94	8.66	11.69	10.38	10.92	0.9	1	12.95	14.66	10.06	12.56	12.53	13.44	14.72	13.56	1.08	1	11.57	10.8	13.28	11.88	9.85	12.48	9.26	10.53	0.89	1	19.98	14.77	16.67	17.14	11.51	13.38	12.45	0.73	1
Solyc01g088650.2.1	Dolichyl pyrophosphate Glc1M	14.31	14.27	21.41	15.93	16.48	14.28	13.39	12.67	12.55	13.22	0.8	0.853324	14.54	17.39	20.81	17.58	16.45	11.66	13.22	13.78	0.78	0.782586	6.21	8.74	7.36	7.44	3.66	4.32	6.27	4.75	0.64	1	2.45	2.12	1.63	2.07	1.7	2.56	2.13	1.03	1
Solyc01g088660.2.1	Os04g0585900 protein (Fragr	1143.94	1356.88	1386.19	1398.8	1321.45	1714.5	1318.73	2127.21	1114.44	1568.72	1.19	0.743631	1165.32	1421.64	1109.96	1232.31	947.61	1219.77	1320.49	1162.62	0.94	1	310.73	450.49	264.55	341.92	162.09	111.5	164.89	146.16	0.43	0.004877	111.62	205.56	198.84	172.01	160.64	166.59	163.62	0.95	1
Solyc01g088670.1.1	Pentatricopeptide repeat-con	1.52	2.45	1.88	1.64	1.87	2.18	1.6	3.07	2.13	2.25	1.2	0.960371	1.96	2.52	2.02	2.17	2.01	2.21	2.45	2.22	1.03	1	0.29	1.06	0.65	0.67	0.71	0.38	1	0.7	1.05	1	0.72	1.05	1.23	1	1.13	1.46	1.29	1.29	1
Solyc01g088680.2.1	Unknown Protein (AHRD V1)	11	11.42	4.32	12.96	9.93	8.15	8.68	12.64	9.87	9.83	0.99	1	6.82	7.17	7.74	7.24	9.53	10.91	12.24	10.89	1.5	0.875889	1.48	5.23	0.91	2.54	4.82	2.08	3.34	3.41	1.34	1	2.61	1.33	2.82	2.25	4.19	3.21	3.7	1.64	1
Solyc01g088690.2.1	ATP binding / serine-threonin	7.21	9.97	11.77	10.42	9.84	6.82	3.75	8.32	4.96	5.96	0.61	0.223944	9.85	15.47	19.05	14.79	13.41	15.49	12.66	13.85	0.94	1	18.22	19	29.39	22.2	23.28	20.7	22.02	22	0.99	1	47.99	74.21	62.76	61.65	34.97	30.94	32.95	0.53	0.301663
Solyc01g088700.2.1	Eukaryotic translation initiati	32.33	34.27	46.8	44.59	39.5	23.43	30.74	27.27	32.44	28.47	0.72	0.501617	42.07	41.34	58.96	47.46	50.27	40.15	39.62	43.35	0.91	1	39.52	50.13	51.12	46.92	47.13	34.75	41.71	41.2	0.88	1	51.94	50.59	54.21	52.25	43.84	47.59	45.72	0.87	1
Solyc01g088710.2.1	Metacaspase (AHRD V1 ***-	15.91	15.12	15.19	19.98	16.55	14.11	19.34	12.99	15.53	15.49	0.94	1	18.13	21.6	19.03	19.59	18.1	19.25	18.69	18.68	0.95	1	15.46	20.4	20.05	18.64	21.26	19.51	16.81	19.19	1.03	1	20.08	22.33	20.07	20.83	27.17	25.08	26.12	1.25	1
Solyc01g088720.2.1	Transcription initiation factor	13.17	12.68	12.51	13.79	13.04	13.81	18.77	14.57	13.38	15.13	1.16	0.773437	13.35	15.64	18.18	15.72	16.12	17.02	13.97	15.7	1	1	19.55	19.11	21.15	19.94	20.4	18.02	20.68	19.7	0.99	1	51.62	29.95	34.42	38.66	23.04	29.02	26.03	0.67	1
Solyc01g088730.2.1	Chaperone protein DnaJ (AHR	38.91	36.22	48.02	37.96	40.28	33.56	29.48	36.39	32.11	32.89	0.82	0.851655	45.35	56.76	53.27	51.79	67.94	66.23	69.77	67.98	1.31	0.432412	42.46	47.38	50.61	46.82	52.15	43.56	51.99	49.23	1.05	1	98.88	80.72	76.34	85.31	75.95	75.1	75.53	0.89	1
Solyc01g088740.2.1	Casein kinase II subunit beta-	23.68	20.05	18.98	22.11	21.21	21.46	26.31	16.7	19.47	20.98	0.99	1	19.88	19.38	21.55	20.27	19.61	16.42	20.59	18.87	0.93	1	16.9	18.92	11.86	15.89	13.44	14.46	20.1	16	1.01	1	23.53	18.53	16.26	19.44	21.25	25.19	23.22	1.19	1
Solyc01g088750.2.1	Pentatricopeptide repeat-con	4.23	3.68	4.04	3.7	3.91	3.02	3.26	4.07	3.69	3.51	0.9	1	3.93	4.82	5.36	4.7	4.33	5.11	4.83	4.76	1.01	1	2.36	3.86	3	3.07	2.58	1.45	2.94	2.32	0.76	1	3.87	4.46	4.65	4.33	4.33	3.62	3.98	0.92	1
Solyc01g088760.2.1	Cytochrome P450	6.33	7.7	10.36	5.48	7.47	11.77	9.48	15.43	10.94	11.9	1.59	0.148172	12.37	19.19	13.41	14.99	21.85	20.52	23.44	21.94	1.46	0.217948	29.41	25.67	21.53	25.54	20.35	17.81	23.05	20.4	0.8	1	14.13	27	20.45	20.53	14.77	12.34	13.55	0.66	0.766343
Solyc01g088770.2.1	Os04g0665000 protein (Fragr	64.16	70.78	61.84	42.18	59.74	60.27	43.52	104.69	60.34	67.21	1.12	1	57.16	53.42	47.32	52.63	34.47	43.52	39.06	39.02	0.74	0.836608	65.71	70.69	43.06	59.82	39.18	41.78	43.46	41.47	0.69	1	20.56	36.1	33.35	30	22.34	32.49	27.41	0.91	1
Solyc01g088780.2.1	Rho-related GTP-binding prot	0.63	1.4	0	0	0.51	0.35	0	0.29	0.45	0.27	0.54	1	0	0	0.27	0.09	0.44	0.49	0.25	0.39	4.37	1	0	0	0	0	0	0	0.23	0.08	7.67	1	0	0	0	0	0	0.28	0.14	14	1
Solyc01g088790.2.1	Regulatory protein (AHRD V1	0	0.34	0.89	0	0.31	0	0.15	0	0.14	0.07	0.24	0.926839	0.53	0	0	0.18	0.13	0	0.15	0.09	0.53	1	0	0.15	0	0.05	0	0	0	0	0.2	1	0	0	0	0	0	0	0	1	NA
Solyc01g088800.2.1	Polyadenylate-binding protei	27.65	27.64	32.41	27.6	28.82	20.33	25.07	23.41	26.88	23.92	0.83	0.919033	31.66	31.26	34.66	32.53	25.61	27.72	28.4	27.24	0.84	0.951823	26.86	28.3	26.4	27.19	27.13	21.55	23.4	24.03	0.88	1	23.76	31.96	32.43	29.38	19.86	26.31	23.09	0.79	1
Solyc01g088810.2.1	Ankyrin repeat protein (AHRD	0.3	0.93	0.88	1.06	0.79	0.33	0.36	0.41	0.32	0.35	0.45	0.755985	0.84	0.65	0.63	0.71	0.31	0.23	0.59	0.38	0.53	0.944042	0.85	0.49	0.89	0.74	0.69	0.11	0.11	0.3	0.41	1	3.62	1.52	3	2.71	1.24	1.18	1.21	0.45	0.98833
Solyc01g088820.2.1	Transcription factor (AHRD V1	9.2	11.74	13.68	16.12	12.68	8.22	6.94	7.72	8.04	7.73	0.61	0.312779	8.74	12.16	13.68	11.53	9.57	10.04	10.05	9.89	0.86	1	3.88	6.03	4.17	4.69	6.57	3.18	4.95	4.9	1.04	1	82.71	14.96	23.67	40.45	12.63	16.83	14.73	0.36	1
Solyc01g088830.2.1	Ankyrin repeat protein (AHRD	0	0	0	0	0	0	0	0	0	0	1	NA	0	0	0	0	0	0	0	0	1	NA	0	0	0	0	0	0	0	0	1	NA	0	0	0	0	0	0	0	1	NA
Solyc01g088840.1.1	Unknown Protein (AHRD V1)	0	0	0	0	0	0	0	0	0	0	1	NA	0	0	0	0	0	0	0	0	1	NA	0	0	0	0	0	0	0	0	1	NA	0	0	0	0	0	0	0	1	NA
Solyc01g089840.2.1	Glutamyl-tRNA reductase (AH	0	0	0	0	0	0	0	0	0	0	1	NA	0	0	0	0	0	0	0	0	1	NA	0	0	0	0	0	0	0	0	1	NA	0	0	0	0	0	0	0	1	NA
Solyc01g089850.2.1	Cyclin-dependent protein kina	3.51	3.97	2.26	3.61	3.34	3.54	8.81	5.27	2.29	4.98	1.49	0.786093	3.26	2.77	3.23	3.09	1.77	4.13	4.76	3.55	1.15	1	1.03	1.04	0.63	0.9	1.47	0.97	1.86	1.43	1.59	1	0	0.46	0.49	0.32	0.29	0.84	0.56	1.78	1
Solyc01g089860.2.1	Unknown Protein (AHRD V1)	60.72	35.36	46.43	51.43	48.48	31.25	47.94	34.46	46.82	40.12	0.83	0.986666	60.67	52.13	39.57	50.79	55.9	41.73	52.73	50.12	0.99	1	30.89	49.18	39.44	39.84	38.66	57.39	45.34	47.13	1.18	1	84.74	99.91	106.63	97.09	53.53	71.65	62.59	0.64	0.824749
Solyc01g089870.2.1	Pentatricopeptide repeat-con	21.87	21.51	19.39	17.6	20.09	23.64	17.65	29.33	26.02	24.16	1.2	0.722371	22.03	20.48	20.31	20.94	26.06	23.77	28.77	26.2	1.25	0.643581	16.79	19	15.47	17.09	18.81	18.22	14.97	17.33	1.01	1	9.1	16.15	14.12	13.12	19.91	19.81	19.86	1.51	0.963653
Solyc01g089880.2.1	Unknown Protein (AHRD V1);	32.62	37.51	12.97	17.63	25.18	21.59	6.66	27.63	19.21	18.77	0.75	0.826254	23.18	42.05	16.72	27.32	14.49	20.49	21.89	18.96	0.69	0.9302	0.59	0.6	0.73	0.64	0.96	0.28	0.27	0.5	0.79	1	0	0.53	1.13	0.55	0.34	0.32	0.33	0.6	1
Solyc01g089890.2.1	Unknown Protein (AHRD V1);	2.07	3.38	0.81	1.47	1.93	1.15	1.09	1.91	1.49	1.41	0.73	0.999407	0	18.62	4.38	7.67	17.49	2.32	0.81	6.87	0.9	1	0.28	0.56	0	0.28	0.23	0.52	0	0.25	0.89	1	3.44	1	3.19	2.54	0	0	0	0	0.26498
Solyc01g089900.2.1	Alpha-mannosyltransferase (A	4.49	7.05	2.65	2.99	4.29	7.63	6.77	6.79	5.12	6.58	1.53	0.471433	5.15	5.51	5.13	5.26	2.75	4.51	5.7	4.32	0.82	1	8.74	3.23	7.86	6.61	4.94	4.2	5.1	4.75	0.72	1	4.69	6.9	5.68	5.76	3.26	4.28	3.77	0.65	0.989607
Solyc01g089910.2.1	Flotillin domain protein (AHR	0	0	0	0	0	0	0	0	0	0	1	NA	0	0.12	0	0.04	0	0	0	0	0.25	1	0	0	0	0	0	0	0	0	1	1	0	0	0	0	0	0	0	1	NA
Solyc01g089920.2.1	Bax inhibitor (AHRD V1 ***-	0	0	0	0	0	0	0	0	0	0	1	NA	0	0	0	0	0	0.15	0	0.05	5	1	0	0.23	0	0.08	0	0	0	0	0.13	1	0	0	0	0	0	0	0	1	NA
Solyc01g089930.1.1	Riboflavin biosynthesis protei	0.76	0	0	1.07	0.46	0	0	1.39	0	0.35	0.76	1	0	0	0.64	0.21	0.53	1.18	0.6	0.77	3.61	1	0	0.62	0.75	0.46	0	0.57	1.11	0.56	1.23	1	0	0	0	0	0	0	0	1	NA
Solyc01g089940.1.1	Bax inhibitor (AHRD V1 *-*- C	0	0	0	0	0	0	0	0	0	0	1	NA	0	0	0	0	0	0	0	0	1	NA	0	0	0	0	0	0	0	0	1	NA	0	0	0	0	0	0	0	1	NA
Solyc01g089950.2.1	Unknown Protein (AHRD V1);	4.64	5.4	2.52	3.03	3.9	6.74	3.1	8.2	4.35	5.6	1.44	0.88285	5.64	2.79	2.11	3.51	2.72	3.14	5.04	3.63	1.03	1	1.15	2.03	1.06	1.41	1.41	2.43	2.86	2.23	1.58	1	1.52	2.33	2.74	2.2	1.31	1.25	1.28	0.58	1
Solyc01g089960.2.1	WRKY family transcription fac	0	0	0	0	0	0	0	0	0.2	0.05	5	1	0	0	0	0	0	0	0	0	1	NA	0	0	0	0	0	0	0	0	1	NA	0	0	0	0	0	0	0	1	NA
Solyc01g089970.2.1	Nucleoside diphosphate kinas	1106.9	891.35	1292.85	1305.8	1149.22	892.29	844.51	619.59	795.31	787.92	0.69	0.277926	919.65	964.13	1106.94	996.91	805.49	772.79	686.86	755.05	0.76	0.583654	481.83	497.89	566.93	515.55	543.33	485.92	446.22	491.82	0.95	1	943.64	527.39	554.21	675.08	877.08	1177.04	1027.06	1.52	0.708333
Solyc01g089980.2.1	Engulfment and cell motility	54.61	52.56	55.68	57.67	55.13	57.68	35.7	35.44	40.09	42.23	0.77	0.583895	30.34	36.51	46.33	37.73	42.67	37.61	40.78	40.35	1.07	1	14.17	16.11	18.88	16.39	14.77	12.92	14.4	14.03	0.86	1	14.05	10.41	14.82	13.09	22.62	31.63	27.12	2.07	0.26498
Solyc01g089990.1.1	Uncharacterized MFS-type tra	0	0.78	0	1.24	0.51	0	0.35	0.4	0.63	0.34	0.68	1	0.82	0.38	1.11	0.77	0	0.91	1.04	0.65	0.84	1	1.07	2.51	0.44	1.34	0.29	0.33	0	0.21	0.15	1	0	0	0	0	0	0	0	1	NA
Solyc01g090000.1.1	Bax inhibitor (AHRD V1 ***- (	0	0	0	0	0	0	0	0	0	0	1	NA	0	0	0	0	0	0	0.22	0.07	7.33	1	0	0.22	0	0.07	0	0	0	0	0.14	1	0	0	0	0	0	0	0	1	NA
Solyc01g090010.1.1	Bax inhibitor (AHRD V1 ***- (	0	0	0	0	0	0	0	0	0	0	1	NA	0	0	0	0	0	0	0	0	1	NA	0	0	0	0	0	0	0	0	1	NA	0	0	0	0	0	0	0	1	NA
Solyc01g090020.1.1	Bax inhibitor 1 (AHRD V1 --*-	0	0	0	0	0	0	0	0	0	0	1	NA	0	0	0	0	0	0	0	0	1	NA	0	0	0	0	0	0	0	0	1	NA	0	0	0	0	0	0	0	1	NA
Solyc01g090030.1.1	Bax inhibitor 1 (AHRD V1 ***	0	0	0.31	0	0.08	0.29	0	0	0	0.07	0.94	1	0	0	0	0	0	0	0	0	1	NA	0	0.22	0.26	0.16	0	0	0	0	0.06	1	0	0	0	0	0	0	0	1	NA
Solyc01g090040.2.1	Unknown Protein (AHRD V1)	1.01	4.52	2.39	1.44	2.34	1.13	2.4	2.8	2.19	2.13	0.91	1	1.89	2.65	0.86	1.8	3.52	0	0.8	1.44	0.8	1	3.28	0.83	2.01	2.04	0.67	0.77	0.74	0.73	0.36	1	1.44	0	3.12	1.52	3.72	0	1.86	1.22	1
Solyc01g090050.2.1	Unknown Protein (AHRD V1)	0	0	0	0	0	0	0	0	0	0	1	NA	0	0	0	0	0	0	0	0	1	NA	0	0	0	0	0	0	0	0	1	NA	0	0	0	0	0	0	0	1	NA
Solyc01g090060.1.1	Unknown Protein (AHRD V1)	0	0	0	0	0	0	0	0	0	0	1	NA	0	0	0	0	0	0	0	0	1	NA	0	0	0	0	0	0	0	0	1	NA	0	0	0	0	0	0	0	1	NA
Solyc01g090070.1.1	Bax inhibitor 1 (AHRD V1 ***	0	0	0	0	0	0	0	0	0	0	1	NA	0	0	0	0	0	0	0	0	1	NA	0	0	0	0	0	0	0	0	1	NA	0	0	0	0	0	0	0	1	NA
Solyc01g090080.1.1	Bax inhibitor (AHRD V1 ***-	0	0	0	0	0	0	0	0	0	0	1	NA	0	0	0	0	0	0	0	0	1	NA	0	0.22	0	0.07	0	0	0	0	0.14	1	0	0	0	0	0	0	0	1	NA
Solyc01g090090.1.1	Unknown Protein (AHRD V1)	0	0	0	0	0	0	0	0	0	0	1	NA	0	0	0	0	0	0	0	0	1	NA	0	0	0	0	0	0	0	0	1	NA	0	0	0	0	0	0	0	1	NA
Solyc01g090100.1.1	Unknown Protein (AHRD V1)	0	0	0	0	0	0	0	0.31	0	0.08	7.75	1	0	0	0	0	0	0	0	0	1	NA	0	0	0.34	0.11	0	0	0.25	0.08	0.74	1	0	0	0	0	0	0	0	1	NA
Solyc01g090110.1.1	Bax inhibitor 1 (AHRD V1 ***	0	0	0	0	0	0	0	0	0	0	1	NA	0	0	0	0	0	0	0	0	1	NA	0	0	0	0	0	0	0	0	1	NA	0	0	0	0	0	0	0	1	NA
Solyc01g090120.2.1	Blue copper protein (AHRD V	0.35	0.31	0.82	0.49	0.49	0	0	0	0	0	0.02	0.62652	0.65	0	0.29	0.31	0	0.9	0	0.3	0.96	1	0	0	0	0	0	0	0.25	0.08	8.33	1	0	0	0	0	0	0.3	0.15	15	1
Solyc01g090130.1.1	Pectinesterase (AHRD V1 ***	0	0.92	1.22	0	0.54	0	0	0.47	0.74	0.3	0.57	1	0	0.45	0	0.15	0.36	1.33	0.81	0.83	5.56	0.895788	0	0.42	0.51	0.31	0	0	0	0	0.03	1	1.47	0	0	0.49	0	0	0	0.02	1
Solyc01g090140.1.1	Naringenin,2-oxoglutarate 3-	0	0	0.35	0	0.09	0	0	0	0	0	0.11	1	0	0	0	0	0	0	0	0	1	NA	0	0	0	0	0	0	0	0	1	NA	0	0	0	0	0	0	0	1	NA
Solyc01g090150.1.1	Unknown Protein (AHRD V1)	5.74	5.11	0	0	2.71	0	0	0	2.06	0.52	0.19	0.967871	5.34	2.49	0	2.61	0	0	0	0	0	0.962977	0	0	0	0	0	0	0	0	1	NA	0	0	0	0	0	0	0	1	NA
Solyc01g090160.2.1	Unknown Protein (AHRD V1)	5.21	18.54	6.14	4.91	8.7	19.3	15.08	28.73	7.48	17.65	2.03	0.640554	11.3	4.53	11.73	9.19	12.04	16.18	16.37	14.86	1.62	0.872779	15.43	19.81	20.68	18.64	18.25	22.37	17.71	19.44	1.04	1	27.15	23.96	26.7	25.94	25.43	12.18	18.8	0.73	1
Solyc01g090170.2.1	Ras-related protein Rab-1A (A	100.57	88.36	88.9	92.86	92.67	84.84	79.66	78.5	92.17	83.79	0.9	1	87.84	92.77	76.75	85.79	84.11	74.01	80.82	79.65	0.93	1	109.99	92.23	86.67	96.3	85.83	96.33	90.26	90.81	0.94	1	65.75	64.83	49.23	59.94	81.41	79.96	80.69	1.35	1
Solyc01g090180.2.1	Extradiol ring-cleavage dioxyg	2.17	6.67	2.85	7.52	4.8	5.91	3.82	1.78	5.55	4.26	0.89	1	2.7	2.31	1.84	2.28	2.51	0.88	1.14	1.51	0.66	0.962977	2.93	1.77	1.44	2.05	0.16	0.18	0.35	0.23	0.11	1	0	0	0	0	0	0	0	1	NA
Solyc01g090190.2.1	Nuclear RNA binding protein	218.75	198.26	256.35	240.92	228.57	170.43	176.16	180.12	189.32	179.01	0.78	0.708097	203.3	201.91	249.43	218.21	179.6	184.22	152.66	172.16	0.79	0.763865	207.58	211.61	219.48	212.89	266.15	194.88	199.75	220.26	1.03	1	158.75	168.96	154.28	160.66	184.68	162.47	173.57	1.08	1
Solyc01g090200.2.1	Glycyl-tRNA synthetase beta	25.16	24.82	23.01	27.5	25.12	25.34	24.28	20.28	27.61	24.38	0.97	1	25.66	30.78	28.59	28.34	26.45	26.02	27.09	26.52	0.94	1	16.71	16.75	20.35	17.94	20.49	18.97	18.24	19.23	1.07	1	26.14	27.34	33.24	28.91	31.33	35.68	33.5	1.16	1
Solyc01g090210.2.1	Genomic DNA chromosome 5	25.52	26.91	15.29	23.88	22.9	27.94	30.07	38.99	40.88	34.47	1.51	0.218454	25.93	21.85	12.94	20.24	18.79	33.75	27.77	26.77	1.32	0.71782	11.42	9.92	4.29	8.54	6.97	5.36	6.87	6.4	0.75	1	2.23	3.42	1.21	2.29	0.72	1.72	1.22	0.53	1
Solyc01g090220.2.1	Lipase (Fragment) (AHRD V1	25.99	23.28	22.05	20.63	22.99	23.29	22.37	22.43	20.28	22.09	0.96	1	21.52	25.5	24.28	23.77	28.02	26.61	25.89	26.84	1.13	0.910379	12.87	12.5	11.15	12.17	14.65	12.45	14.9	14	1.15	1	17.52	18.45	16.89	17.62	20.26	19.7	19.98	1.13	1
Solyc01g090230.2.1	GRAM domain containing pro	21.54	18.43	24.8	23.99	22.19	15.96	15.85	15.79	17.63	16.31	0.73	0.584227	19.55	20.26	27.86	22.56	19.7	16.38	14.88	16.99	0.75	0.647547	13.45	15.09	12.5	13.68	10.05	10.09	10.16	10.1	0.74	1	11.54	9.6	13.6	11.58	9.72	14.78	12.25	1.06	1
Solyc01g090240.2.1	Protein kinase 5 (AHRD V1 **	6.21	6.28	7.99	17.44	9.48	6.59	11.14	4.07	5.81	6.9	0.73	1	6.13	8.84	11.68	8.88	4.24	5.17	4.96	4.79	0.54	0.162724	0.23	0.54	0.09	0.29	0.43	0.36	0.27	0.35	1.23	1	0.27	0.41	0.43	0.37	0.17	0.66	0.42	1.12	1
Solyc01g090250.2.1	Integrator complex subunit 4	3.26	4.2	4.42	3	3.72	4.16	3.86	4.04	4.21	4.07	1.09	1	4.24	3.12	5.23	4.2	4.86	5.06	3.71	4.54	1.08	1	2.76	1.99	1.46	2.07	2.03	1.6	1.9	1.84	0.89	1	3.01	1.54	1.25	1.93	2.53	5.14	3.83	1.98	0.828552
Solyc01g090260.2.1	HEAT repeat family protein e	3.05	3.07	3.91	2.44	3.12	2.5	3.66	3.9	2.76	3.21	1.03	1	1.97	3.22	3.13	2.77	2.11	3.09	2.6	2.6	0.94	1	2.03	1.4	0.79	1.41	1.39	1.41	0.97	1.26	0.89	1	2.83	1.83	2.65	2.44	2.3	2.9	2.6	1.07	1
Solyc01g090270.2.1	Bzip-like transcription factor-	30.69	32.2	53.34	58.84	43.77	24.25	28.03	21.92	25.17	24.84	0.57	0.291626	26.31	33.81	49.94	36.69	27.6	32.33	25.07	28.33	0.77	0.911351	9.36	13.49	9.93	10.93	12.35	7.53	10.47	10.12	0.93	1	20.86	17.1	23.94	20.63	15.77	24.6	20.19	0.98	1
Solyc01g090280.2.1	Leucine carboxyl methyltrans	31.06	32.79	27.31	30.83	30.5	32.16	30.15	34.76	21.47	29.63	0.97	1	31.4	36.05	34.21	33.89	24.88	28.46	31.99	28.44	0.84	0.964678	38.34	34.9	33.7	35.65	31.05	34.65	31.63	32.44	0.91	1	48	55.9	56.36	53.42	34.26	39.24	36.75	0.69	0.925516
Solyc01g090290.2.1	COP9 signalosome complex s	43.9	51.71	48.5	44.86	47.24	40.05	43.47	51.32	44.99	44.96	0.95	1	52.87	45.66	48.01	48.85	39.6	50.05	40.08	43.24	0.89	1	71.95	69.06	70.77	70.59	61.34	58.08	59.91	59.78	0.85	1	66.74	81.82	73.74	74.1	61.09	57.18	59.14	0.8	1
Solyc01g090300.2.1	Ethylene responsive transcrip	1.62	1.74	0	0.92	1.07	0	0.26	0	0.47	0.18	0.17	0.449781	0	0	0	0	0	0	0	0	1	NA	0	0	0.65	0.22	0	0	0	0	0.05	NA	0	0	0	0	0	0	0	1	NA
Solyc01g090310.2.1	Ethylene responsive transcrip	6.56	2.92	0.86	4.64	3.75	0	0.29	0.34	0	0.16	0.04	0.014243	0	0	0.31	0.1	0	0	0	0	0.1	1	0	0	0	0	0	0	0	0	1	1	0	0	0	0	0	0	0	1	NA
Solyc01g090320.2.1	Ethylene responsive transcrip	0	0	0	0	0	0	0	0	0	0	1	NA	0	0	0	0	0	0	0	0	1	NA	0	0	0	0	0	0	0	0	1	NA	0	0	0	0	0	0	0	1	NA
Solyc01g090330.1.1	Unknown Protein (AHRD V1)	0	0	0	0	0	0	0	0	0	0	1	NA	0	0	0	0	0	0	0	0	1	NA	0	0	0	0	0	0	0	0	1	NA	0	0	0	0	0	0	0	1	NA
Solyc01g090340.2.1	Ethylene responsive transcrip	23.99	28.29	45.99	61.93	40.05	9	24.38	46.59	64.58	36.14	0.9	1	6.69	0.78	4.81	4.09	0.21	0.93	1.18	0.77	0.19	0.409418	0	0	0.3	0.1	0	0	0	0	0.1	NA	0	0.22	0	0.07	0	0	0	0.14	1
Solyc01g090350.2.1	Non-specific lipid-transfer pro	0.55	2.43	3.86	0	1.71	0	0.43	1.5	0	0.48	0.28	0.734913	3.55	2.37	0.92	2.28	0.76	0	0	0.25	0.11	0.263746	0	0	0	0	0	0	0	0	1	NA	0	0	0	0	0	0	0	1	NA
Solyc01g090360.2.1	Non-specific lipid-transfer pro	3.98	9.62	12.75	3.22	7.39	0	0.9	3.66	0	1.14	0.15	0.074807	14.28	3.46	3.36	7.03	0	0	0.45	0.15	0.02	0.156385	0	0	1.13	0.38	0	0	0	0	0.03	NA	0	0	0	0	0	0	0	1	NA
Solyc01g090370.1.1	Ethylene responsive transcrip	0	0	0	0	0	0	0	0	0	0	1	NA	0	0	0	0	0	0	0	0	1	NA	0	0	0	0	0	0	0	0	1	NA	0	0	0	0	0	0	0	1	NA
Solyc01g090380.2.1	Os02g0655100 protein (Fragr	7.08	7.38	7.4	9.45	7.83	8.77	6.55	9.68	7.85	8.21	1.05	1	12.99	11.43	8.55	10.99	10.25	9.85	11.13	10.41	0.95	1	6.7	12.37	10.85	9.97	9.57	9.2	10.91	9.89	0.99	1	8.63	10.73	10.27	9.88	10.74	10.82	10.78	1.09	1
Solyc01g090390.1.1	Pentatricopeptide repeat-con	1.77	1.77	1.44	2.82	1.95	1.35	2.53	1.22	1.19	1.57	0.81	1	0.62	1.25	1.5	1.12	1.84	1.83	2.44	2.04	1.81	0.508115	0.27	0.81	0.88	0.65	0.65	0.76	0.97	0.79	1.21	1	0.79	0.64	1.7	1.04	1.11	2.43	1.77	1.7	0.999735
Solyc01g090400.2.1	EF hand family protein (AHRD	6.78	5.08	5.05	4.04	5.24	3.17	3.94	3.61	2.56	3.32	0.63	0.712634	2.99	3.41	2.41	2.94	4.45	3.69	3.08	3.74	1.27	1	1.73	2.32	1.42	1.82	2.11	1.08	1.82	1.67	0.92	1	2.03	1.04	2.74	1.94	3.59	2.81	3.2	1.65	1
Solyc01g090410.2.1	Palmitoyl protein thioesteras	5.31	10.54	4.34	3.18	5.84	14.76	7.26	12.77	8.8	10.9	1.87	0.393843	9.69	10.3	5.52	8.5	11.47	14.06	17.82	14.45	1.7	0.118773	10.4	13.65	6.69	10.25	11.14	10.22	17.56	12.97	1.27	1	1.16	5.79	3.45	3.47	4.86	3.94	4.4	1.27	1

Solyc01g090420.2.1	Armadillo/beta-catenin repea	11.06	13.82	12.83	12.54	12.56	10.66	10.89	7.73	11.02	10.07	0.8	0.82735	12.75	12.31	14.05	13.04	13.82	13.72	12.71	13.42	1.03	1	11.74	10.27	11.7	11.24	11.58	11.29	11.45	11.44	1.02	1	10.13	12.08	12.22	11.48	10.54	12.02	11.28	0.98	1
Solyc01g090430.2.1	Cc-nbs-lrr, resistance protein	13.28	12.15	20.05	22.04	16.88	8.91	8.03	9.55	9.43	8.98	0.53	0.101557	10.98	21.35	20.31	17.55	11.48	8.57	9.51	9.85	0.56	0.2421	4.68	7.26	9.35	7.1	12.33	4.78	4.27	7.13	1	1	23.63	23.88	19.51	22.34	6.52	5.86	6.19	0.28	0.001674
Solyc01g090440.2.1	Unknown Protein (AHRD V1)	2.82	4.88	3.33	2.85	3.47	4.39	5.09	6.6	4.52	5.15	1.48	0.369555	3.23	2.1	1.98	2.44	2.46	2.46	2.22	2.38	0.98	1	0.33	1.12	0.08	0.51	0.26	0.37	1	0.54	1.07	1	0	0	0.12	0.04	0	0.35	0.17	4.38	1
Solyc01g090450.2.1	Actin-related protein 2/3 com	28.77	47.68	31.02	30.17	34.41	44.66	40.33	48.8	38.46	43.06	1.25	0.752152	40.6	36.22	31.42	36.08	28.21	32.1	28.07	29.46	0.82	1	46.09	35.57	29.05	36.9	27.7	32.71	34.34	31.58	0.86	1	23.98	30.26	32.8	29.01	24.07	32.18	28.12	0.97	1
Solyc01g090460.2.1	Homeobox-leucine zipper pro	5.75	7.98	5.97	4.55	6.06	1.79	2	3.8	6.11	3.42	0.56	0.408318	2.99	7.39	10.68	7.02	17.86	13.21	17.7	16.26	2.32	0.015065	2.23	5.43	1.37	3.01	2.72	2.96	3.52	3.07	1.02	1	33.34	20.87	17.67	23.96	10.31	11.29	10.8	0.45	0.648665
Solyc01g090470.2.1	Bfr2 (AHRD V1 **-- B6JYE3_S	32.48	37.64	41.51	25.84	34.37	27.26	29.38	39.97	32.91	32.38	0.94	1	40.91	33.82	34.86	36.53	30.39	38.8	38.42	35.87	0.98	1	48.65	47.69	46.51	47.62	51.12	51.16	48.07	50.12	1.05	1	52.19	66.22	57.75	58.72	52.65	49.39	51.02	0.87	1
Solyc01g090480.2.1	Nucleic acid binding protein (	19.42	18.21	25.54	18.87	20.51	12.03	15.57	10.09	18.94	14.16	0.69	0.490084	19.86	19.97	25.54	21.79	17.17	14.35	16.66	16.06	0.74	0.590566	15.09	14.74	14.27	14.7	18.11	17.53	13.4	16.35	1.11	1	39.42	16.04	18.47	24.64	17.24	21.97	19.6	0.8	1
Solyc01g090490.1.1	mTERF family protein (AHRD	1.67	1.95	1.36	1.63	1.65	2.09	1.4	1.92	1.5	1.73	1.05	1	2.04	2.82	2.74	2.53	1.74	2.76	1.56	2.02	0.8	1	1.69	2.64	1.66	2	1.1	0.71	1.07	0.96	0.48	1	3.86	1.9	3.54	3.1	2.01	1.65	1.83	0.59	1
Solyc01g090500.2.1	Unknown Protein (AHRD V1)	95	109.21	126.09	139.97	117.57	102.66	110.42	115.85	88.57	104.37	0.89	1	102.48	135.91	132.62	123.67	124.91	95.3	82.95	101.05	0.82	0.906212	147.64	124.72	121.14	131.17	100.55	135.5	111.53	115.86	0.88	1	62.84	73.31	61.88	66.01	66.43	67.1	66.77	1.01	1
Solyc01g090510.2.1	Pentatricopeptide repeat-con	1.12	0.92	0.55	1.06	0.91	0.42	0.59	0.34	0.6	0.49	0.53	0.697677	1.04	1.22	1.26	1.17	0.65	0.78	0.96	0.8	0.68	0.941718	0.45	0.08	0.28	0.27	0.55	0.21	0.68	0.48	1.78	1	0.13	1.02	0.72	0.62	0.43	0.82	0.62	1	1
Solyc01g090520.2.1	Unknown Protein (AHRD V1)	3.18	1.41	1.88	0	1.62	0	2.51	0	0	0.63	0.39	0.935216	0	0	0	0	0	0	0	0	1	NA	0	0	0	0	0	0	0	0	1	NA	0	0	0	0	0	0	0	1	NA
Solyc01g090530.1.1	MYB transcription factor (AH	0.21	0.37	0.24	0.58	0.35	0.23	0.49	0.57	0.44	0.43	1.24	1	0.77	0.54	0.17	0.49	0.14	0.11	0.65	0.3	0.61	1	0.17	0.17	0	0.11	0	0	0	0	0.09	1	0	0	0	0	0	0.18	0.09	9	1
Solyc01g090540.1.1	Unknown Protein (AHRD V1)	0	0	0	0	0	0	0	0	0	0	1	NA	0	0	0	0	0	0	0	0	1	NA	0	0	0	0	0	0	0	0	1	NA	0	0	0	0	0	0	0	1	NA
Solyc01g090550.2.1	DNAJ chaperone (AHRD V1 **	84.6	81.09	84.79	76.19	81.67	79.37	55.39	68.51	78.79	70.52	0.86	0.937173	90.39	110.11	107.9	102.8	133.5	119.91	111.62	121.68	1.18	0.73259	169.93	177.72	183.81	177.15	191.4	192.01	186.53	189.98	1.07	1	181.86	281.18	267.34	243.46	250.09	197.89	223.99	0.92	1
Solyc01g090560.2.1	Ethylene-responsive transcrip	18.71	17.93	37.93	22.4	24.24	20.27	43.18	40.81	23.94	32.05	1.32	0.678531	28.34	10.43	8.71	15.83	2.66	4.84	5.84	4.45	0.28	0.507051	0.39	0.2	0.24	0.28	0	0	0.17	0.06	0.2	1	0	0	0	0	0.22	0	0.11	11	1
Solyc01g090570.1.1	Unknown Protein (AHRD V1)	0	0	0	0	0	0	0	0	0	0	1	NA	0	0	0	0	0	0	0	0	1	NA	0	0	0	0	0	0	0	0	1	NA	0	0	0	0	0	0	0	1	NA
Solyc01g090580.2.1	Unknown Protein (AHRD V1)	0	0	0	0	0	0	0	0	0	0	1	NA	0	0	0	0	0	0	0	0	1	NA	0	0	0	0	0	0	0	0	1	NA	0	0	0	0	0	0	0	1	NA
Solyc01g090590.2.1	Os11g0109966 protein (Fragı	12.09	12.31	13.23	9.48	11.78	9.39	8.28	11.78	10.56	10	0.85	0.960351	17.88	17.6	18.45	17.98	20.4	19.08	13.62	17.7	0.98	1	45.3	40.7	53.48	46.49	37.02	46.03	46.48	43.18	0.93	1	20.5	38.18	36.06	31.58	29.33	22.6	25.96	0.82	1
Solyc01g090600.2.1	Chalcone synthase 3 protein (	0	0	0	0	0	0	0	0	0	0	1	NA	0	0	0	0	0	0	0	0	1	NA	0	0	0	0	0	0	0	0	1	NA	0	0	0	0	0	0	0	1	NA
Solyc01g090610.2.1	2-oxoglutarate-dependent di	0	0	0	0	0	0	0.16	0	0	0.04	4	1	0.19	0	0	0.06	0	0	0	0	0.16	1	0	0	0	0	0	0	0	0	1	NA	0	0	0	0	0	0	0	1	NA
Solyc01g090620.1.1	Ribosomal protein-like (AHRD	0	0	0	0	0	0	0	0	0	0	1	NA	0	0	0	0	0	0	0	0	1	NA	0	0	0	0	0	0	0	0	1	NA	0	0	0	0	0	0	0	1	NA
Solyc01g090630.2.1	2-oxoglutarate-dependent di	0	0	0	0	0	0	0.16	0.19	0	0.09	8.75	1	0	0	0	0	0	0.21	0	0.07	7	1	0	0	0	0	0	0	0.15	0.05	5	1	0	0	0	0	0	0	0	1	NA
Solyc01g090640.2.1	Chromodomain helicase DNA	3.19	4.25	6.39	3.61	4.36	1.06	2.26	6.15	1.83	2.83	0.65	0.839504	1.78	5.26	3.5	3.51	1.77	1.82	3.01	2.2	0.63	0.855159	0.26	1.82	0.95	1.01	0.42	0.97	0.46	0.62	0.61	1	2.27	1.62	1.47	1.79	2.04	1.68	1.86	1.04	1
Solyc01g090650.1.1	Chromodomain-helicase-DNA	2.69	1.82	1.39	1.82	1.93	2.27	1.53	1.68	1.54	1.76	0.91	1	2.1	2.15	1.09	1.78	1.27	1.5	1.86	1.54	0.87	1	0.69	0.44	0.21	0.45	0.42	0.73	0.23	0.46	1.03	1	1.37	0.94	0.99	1.1	0.79	0.66	0.73	0.66	1
Solyc01g090660.2.1	Carotenoid cleavage dioxyger	0	0	0	0	0	0	0	0	0	0	1	NA	0	0	0	0	0	0	0	0	1	NA	0	0	0.1	0.03	0	0	0	0	0.3	NA	0	0	0	0	0	0	0	1	NA
Solyc01g090670.2.1	Nuclear pore glycoprotein p6	10.69	10.44	14.15	17.34	13.16	12.17	13.11	9.99	10.98	11.56	0.88	1	13.1	10.28	16.66	13.35	10.55	14	13.93	12.83	0.96	1	4.56	6.94	6.38	5.96	7.37	5.34	5.13	5.95	1	1	15.82	7.51	10.83	11.39	10.9	15.39	13.14	1.15	1
Solyc01g090680.2.1	Genomic DNA chromosome 5	21.66	28.14	25.85	22.18	24.46	44.55	21.23	43.95	24.13	33.47	1.37	0.687659	16.21	27.7	24.39	22.77	58.88	30.55	34.82	41.42	1.82	0.407265	5.03	6.09	2.97	4.7	4.91	3.96	7.26	5.38	1.14	1	8.49	11.93	6.13	8.85	4.1	3.06	3.58	0.4	0.378499
Solyc01g090690.2.1	Elongation factor G (AHRD V1	58.43	62.11	85.81	77.01	70.84	41.34	42.36	47.03	56.7	46.86	0.66	0.23562	73.62	69.25	80.54	74.47	92.84	81.83	77.6	84.09	1.13	0.882396	49.03	59.34	56.02	54.8	54.27	54.01	51.8	53.36	0.97	1	70.38	84.75	92.92	82.68	79.28	101.17	90.22	1.09	1
Solyc01g090700.2.1	Enoyl-CoA-hydratase (AHRD V	40.89	40.3	38.82	35.25	38.81	42.19	46.42	40.44	40.58	42.41	1.09	0.952166	50.46	50.83	43.06	48.12	49.94	57.35	51.59	52.96	1.1	0.946562	128.71	163.53	94.59	128.94	119.31	117.04	141.97	126.11	0.98	1	25.01	43.77	46.84	38.54	50.4	40.13	45.27	1.17	1
Solyc01g090710.2.1	Malate dehydrogenase, cytop	0.39	1.93	0.23	0.28	0.71	1.09	0.16	0.72	0.71	0.67	0.95	1	0.18	0.86	0.67	0.57	1.37	0.2	0.15	0.57	1.01	1	0.32	0.16	0	0.16	0.26	0.15	0.14	0.18	1.15	1	0	0	0	0	0	0	0	1	NA
Solyc01g090720.2.1	Unknown Protein (AHRD V1)	61.57	62.07	54.11	46.21	55.99	60.76	63	97.63	41.95	65.83	1.18	0.925999	52.6	49.15	48.29	50.01	40.53	41.96	64.19	48.89	0.98	1	43.15	36.35	39.29	39.6	35.5	32.86	39.37	35.91	0.91	1	76.82	53.39	58.94	63.05	43.71	47.38	45.55	0.72	1
Solyc01g090730.2.1	Squamosa promoter binding	32.99	23.88	29.32	65.63	37.95	23.47	31.25	29.32	23.21	26.81	0.71	0.943718	15.55	11.47	17.64	14.89	0.61	5.35	3.11	3.02	0.2	1.58E-05	0	0.18	0.22	0.13	0	0.17	0	0.06	0.43	1	0	0	0	0	0	0	0	1	NA
Solyc01g090740.2.1	Zinc ion binding protein (AHR	31.3	31.29	35.79	36.1	33.62	17.13	9.5	38.52	21.09	21.56	0.64	0.447005	33.59	54.42	45.96	44.66	39.08	46.87	51.09	45.68	1.02	1	26.45	69.85	15.3	37.2	33.53	16.42	24.21	24.72	0.66	1	13.01	10.49	28.88	17.46	9.7	16.04	12.87	0.74	1
Solyc01g090750.2.1	T-complex protein 1 subunit	67.54	62.37	73.13	57.77	65.2	46.74	49.7	48.32	54.65	49.85	0.76	0.595583	65.75	66.38	63.58	65.24	78.44	69.32	67.8	71.85	1.1	0.958973	72.53	89.01	70.16	77.23	81.44	76.01	84.61	80.69	1.04	1	95.69	86.35	99.96	94	120.56	146.83	133.69	1.42	0.850663
Solyc01g090760.2.1	GATA transcription factor 9 (	2.78	4.05	5.67	0.72	3.31	3.09	1.4	2.32	2.72	2.38	0.72	0.916716	0.71	0.88	1.07	0.89	0.35	0.26	0.2	0.27	0.3	0.77615	0	0	0	0	0	0	0	0	1	NA	0	0	0	0	0	0	0	1	NA
Solyc01g090770.2.1	Unknown Protein (AHRD V1);	49.17	51.24	57.43	55.92	53.44	51.36	66.71	46.57	60.13	56.19	1.05	0.9707	51.71	57.99	59.06	56.25	51.24	59.62	43.11	51.32	0.91	1	32.34	49.73	33.35	38.47	33.44	33.9	39.25	35.53	0.92	1	24.59	27.46	22.8	24.95	38	43.76	40.88	1.64	0.773191
Solyc01g090780.2.1	Ccr4-Not transcription compl	12.75	14.56	17.94	13.66	14.73	12.07	11.36	10.29	12.82	11.63	0.79	0.717678	16.56	14.5	19.54	16.87	15.54	13.72	15.73	15	0.89	1	15.51	12.86	15.45	14.61	12.07	13.62	13.49	13.06	0.89	1	29.98	16.67	22.12	22.92	19.23	22.18	20.7	0.9	1
Solyc01g090790.2.1	BHLH transcription factor (AH	13.34	13.64	24.6	12.44	16	45.26	21.62	31.7	34.32	33.23	2.08	0.047553	13.2	5.55	7.19	8.65	14.66	13.22	8.81	12.23	1.41	0.70488	1.15	0.35	0.14	0.55	0.19	0	0.31	0.17	0.3	1	0.2	0	0	0.07	0	0.25	0.12	1.88	1
Solyc01g090800.2.1	Cyclin-dependent kinase (AHF	0.6	2.39	0	0.42	0.85	2.99	1.89	6.04	2.14	3.27	3.83	0.293182	1.67	1.04	0.5	1.07	0.41	1.85	1.41	1.22	1.14	1	0	0	0	0	0	0	0	0	1	1	0	0	0	0	0	0	0	1	NA
Solyc01g090810.2.1	Expansin protein (AHRD V1 *	0	0	1.97	0	0.49	0.27	0.75	0	0	0.26	0.52	1	0	0	0	0	0.17	0.12	0	0.1	9.67	1	0	0.97	0	0.32	0	0	0.17	0.06	0.18	1	0	0.52	0	0.17	0	0	0	0.06	1
Solyc01g090820.1.1	Expansin B1 (AHRD V1 ***- C	0	0	0	0	0	0	0	0	0	0	1	NA	0	0	0	0	0	0	0	0	1	NA	0	0	0	0	0	0	0	0	1	NA	0	0	0	0	0	0	0	1	NA
Solyc01g090830.2.1	Zinc finger family protein (AH	0	0	0	0	0	0	0	0	0	0	1	NA	0	0	0	0	0	0	0	0	1	NA	0	0	0	0	0	0	0	0	1	NA	0	0	0	0	0	0	0	1	NA
Solyc01g090840.2.1	Cys2/His2 zinc-finger transcri	2.85	3.26	1.92	2.59	2.65	2.04	2.25	4.31	1.9	2.62	0.99	1	1.14	4.6	3.44	3.06	2.4	1.69	1.76	1.95	0.64	0.902624	0.82	0.33	0	0.38	0.54	0	0.45	0.33	0.86	1	0.87	2.22	1.88	1.66	0.19	1.43	0.81	0.49	1
Solyc01g090850.2.1	Unknown Protein (AHRD V1);	10.32	11.46	17.88	16.31	13.99	12.82	11.75	13.19	12.8	12.64	0.9	1	10.85	11.31	14.27	12.14	16.18	12.36	10.12	12.89	1.06	1	13.32	12.89	13.31	13.17	13.92	13.72	14.36	14	1.06	1	25.93	19.09	16.7	20.57	18.29	14.81	16.55	0.8	1
Solyc01g090860.2.1	Poly(A) RNA polymerase prot	8.62	9.53	9.32	9.19	9.16	7.32	9.73	9.96	10.8	9.45	1.03	1	11.54	13.52	11.98	12.35	12.61	15.53	15.66	14.6	1.18	0.756159	7.58	8.41	8.05	8.01	9.18	7.47	9.69	8.78	1.1	1	12.62	12.09	13.33	12.68	12.29	13.74	13.02	1.03	1
Solyc01g090870.1.1	ATOFP17/OFP17 (AHRD V1 *	0	0	0	0	0	0.42	0	0	0	0.1	10.5	1	0	0	0	0	0	0	0	0	1	NA	0	0	0	0	0	0	0	0	1	NA	0	0.27	0	0.09	0	0	0	0.11	1
Solyc01g090880.2.1	Holliday junction resolvase (A	72.45	51.64	48.64	54.99	56.93	69.4	87.42	78.26	76.33	77.85	1.37	0.26341	50.95	56.58	46.02	51.18	71.29	69.08	68.05	69.47	1.36	0.329176	17.37	30.48	20.17	22.67	38.89	30.13	36.91	35.31	1.56	1	22.19	27.81	26.72	25.57	16.98	20.92	18.95	0.74	1
Solyc01g090890.2.1	Xenotropic and polytropic ret	0.25	0	0	0	0.06	0	6.93	0	0	1.73	27.72	0.813369	0	0	0	0	2.61	0	0	0.87	87	0.896309	0	0	0	0	0	0	0.37	0.12	12.33	1	2.14	6.74	0.39	3.09	0	0	0	0	0.554601
Solyc01g090900.2.1	Unknown Protein (AHRD V1)	18.57	23.91	19.57	23.47	21.38	30.52	20.27	24.52	19.99	23.82	1.11	0.972223	22.23	23.87	23.69	23.26	28.08	19.12	24.84	24.01	1.03	1	40.85	41.2	48.23	43.43	27.9	39.37	31.81	33.03	0.76	1	28.93	27.11	20.96	25.67	31.64	24.25	27.95	1.09	1
Solyc01g090910.2.1	Vesicle-associated membrane	67.94	56.46	67.85	62.21	63.62	35.41	35.39	49.25	40.48	40.13	0.63	0.151338	41.43	47.54	46.67	45.21	30.7	22.04	27.69	26.81	0.59	0.080365	14.58	15.39	15.39	15.12	19.45	11.11	8.42	12.99	0.86	1	54.11	28.46	25.14	35.9	14.97	17.55	16.26	0.45	0.889823
Solyc01g090920.2.1	EF hand family protein (AHRD	1.86	4.59	1.7	1.75	2.47	2.75	2.12	5.5	2.07	3.11	1.26	1	3.26	5.2	4.53	4.33	3.43	4.27	3.73	3.81	0.88	1	3.66	4.54	3.68	3.96	6.64	4.06	2.1	4.27	1.08	1	9.38	9.58	7.93	8.96	9.81	8.13	8.97	1	1
Solyc01g090940.2.1	Phosphatidate cytidylyltransf	23.57	22.81	22.21	22.15	22.69	27.61	26.98	25.6	24.19	26.09	1.15	0.836356	21.93	22.97	20.04	21.65	23.15	24.9	23.13	23.73	1.1	0.985957	25.18	24.49	28.74	26.14	25.25	27.94	25.25	26.15	1	1	36.89	28.04	30.65	31.86	35.77	29.41	32.59	1.02	1
Solyc01g090950.2.1	GRAS family transcription fac	9.08	9.84	11.73	14.54	11.3	9.33	14.2	6.62	7.81	9.49	0.84	1	7.01	9.16	10.71	8.96	9.03	7.45	8.96	8.48	0.95	1	5.12	5.24	2.72	4.36	3.66	3.77	3.31	3.58	0.82	1	3.53	6.6	5.27	5.13	2.43	3.61	3.02	0.59	0.762112
Solyc01g090960.1.1	Unknown Protein (AHRD V1)	0	0	0	0	0	0	0	0	0	0	1	NA	0	0	0	0	0	0	0	0	1	NA	0	0	0	0	0	0	0	0	1	NA	0	0	0	0	0	0	0	1	NA
Solyc01g090970.2.1	Cortical cell-delineating prote	0.54	0.48	0	0	0.26	1.19	0	0	0	0.3	1.17	1	0.5	0	0	0.17	0	0	0	0	0.06	1	0	0	0	0	0	0	0	0	1	NA	0	0	0	0	0	0	0	1	NA
Solyc01g090980.1.1	Unknown Protein (AHRD V1)	0	0	0	0	0	0	0	0	0.25	0.06	6.25	1	0	0	0	0	0	0	0	0	1	NA	0	0	0	0	0	0	0	0	1	NA	0	0	0	0	0	0	0	1	NA
Solyc01g090990.2.1	Vesicle-associated membrane	24.91	25.09	20.99	26.72	24.43	24.95	30.04	33.73	29.28	29.5	1.21	0.691253	28.01	25.46	27.51	26.99	26.2	27.35	23.02	25.52	0.95	1	18.8	23.2	17.66	19.89	21.76	17.64	21.54	20.31	1.02	1	10.51	21.67	17.26	16.48	13.53	14.4	13.96	0.85	1
Solyc01g091000.2.1	Zinc ion binding protein (AHR	2.89	2.57	4.65	1.86	2.99	2.63	3.11	2.9	4.34	3.25	1.08	1	2.93	1.6	2	2.18	0.73	0.82	0.62	0.72	0.33	0.419152	0	0	0	0	0	0	0.19	0.06	6.33	1	0	0	0	0	0	0	0	1	NA
Solyc01g091010.2.1	YABBY-like transcription facto	0	0	0	0	0	0	0	0	0	0	1	NA	0	0	0	0	0	0	0	0	1	NA	0	0	0	0	0	0	0	0	1	NA	0	0	0.46	0.15	0	0	0	0.07	1
Solyc01g091020.2.1	Golgi SNAP receptor complex	43.05	35.58	47.49	53.38	44.88	37.6	51.42	41.12	46.36	44.12	0.98	1	43.68	39.85	49.35	44.29	37.61	35.75	43.28	38.88	0.88	1	34.33	41.68	38.29	38.1	29.75	26.48	29.94	28.72	0.75	1	21.46	27.61	21.06	23.38	20.47	24.51	22.49	0.96	1
Solyc01g091030.2.1	Auxin-responsive family prote	0	1.1	0.97	3.5	1.39	1.37	1.95	0.76	1.48	1.39	1	1	0.38	2.15	2.79	1.77	0.86	2.99	2.27	2.04	1.15	1	0.67	2.69	0	1.12	0	0.94	0.6	0.51	0.46	1	0	0.3	0	0.1	0.38	0	0.19	1.9	1
Solyc01g091040.2.1	Cysteine-rich repeat secretory	2.42	4.11	1.3	0.93	2.19	4.16	1.56	5.05	2.05	3.21	1.46	0.993291	2.05	2.68	1.3	2.01	1.68	3.19	3.11	2.66	1.32	0.967182	2.84	1.61	1.96	2.14	0.87	1.5	1.6	1.32	0.62	1	0.63	3.35	2.37	2.12	0.6	2.31	1.46	0.69	1
Solyc01g091050.2.1	Pectinesterase (AHRD V1 ***	0.13	0.11	0.15	0	0.1	0	0.2	0.12	0.09	0.1	1.05	1	0	0	0	0	0	0	0	0	1	NA	0	0	0	0	0	0	0	0	1	1	0.18	0	0	0.06	0	0.11	0.06	0.92	1
Solyc01g091060.1.1	Pectinesterase (AHRD V1 ***	0.41	0.37	1.31	0.78	0.72	0.31	0.11	0.51	0.5	0.36	0.5	0.869554	0.26	0.72	0.12	0.37	0.48	0.29	0.54	0.44	1.19	1	0.11	0.34	0.14	0.2	0.09	0	0.1	0.06	0.32	1	0.98	0.1	0	0.36	0	0	0	0.03	1
Solyc01g091070.2.1	Methionine aminopeptidase (	52.57	55.54	61.43	50.14	54.92	40.34	43.38	45.69	46.14	43.89	0.8	0.760084	53.95	51.01	49.42	51.46	57.17	53.12	51.58	53.96	1.05	1	35.08	35.79	36.53	35.8	35.54	36.89	38.31	36.91	1.03	1	67.27	49.76	47.46	54.83	77	101.41	89.2	1.63	0.58588
Solyc01g091080.2.1	Tubulin-specific chaperone D	8.35	7.87	9.95	8.91	8.77	7.28	8.98	7.53	7.46	7.81	0.89	1	8.07	7.54	7.95	7.85	5.62	6.48	8.94	7.01	0.89	1	4.5	6.07	4.96	5.18	3.71	4.09	5.79	4.53	0.88	1	2.56	2.97	3.78	3.1	3.07	5.24	4.16	1.34	1
Solyc01g091090.2.1	Beta-tubulin cofactor-like pro	4.93	7.07	7.1	6.2	6.33	3.35	7.67	7.55	7.27	6.46	1.02	1	8.02	5.59	8.21	7.27	4.94	4.68	7.1	5.57	0.77	0.838837	4.87	6.8	3.4	5.02	5.66	2.8	4.69	4.38	0.87	1	1.75	2.68	2.32	2.25	3.01	5.16	4.08	1.82	0.95453
Solyc01g091100.2.1	Pectinesterase (AHRD V1 ***	0	0	0	0	0	0	0	0	0	0	1	NA	0	0	0	0	0	0	0	0	1	NA	0	0	0	0	0	0	0	0	1	NA	0.91	1.39	0.98	1.09	0	0	0	0.01	0.790584
Solyc01g091110.2.1	Pectinesterase/pectinesteras	0	0	0	0	0	0	0	0	0	0	1	NA	0	0	0	0	0	0	0	0	1	NA	0	0	0	0	0	0	0	0	1	NA	0	0	0	0	0	0	0	1	NA
Solyc01g091120.2.1	Os03g0825600 protein (Fragı	45.18	45.22	56.99	58.6	51.5	47.96	44.8	49.59	40.74	45.77	0.89	1	53.22	54.09	54.53	53.95	47.27	44.36	45.95	45.86	0.85	0.975922	51.78	57.2	50.74	53.24	42.72	49.53	46.14	46.13	0.87	1	62.9	59.5	53.61	58.67	49.94	51.95	50.95	0.87	1
Solyc01g091130.2.1	Nitroreductase (AHRD V1 ***	2.35	1.43	3.15	2.57	2.38	3.09	1.85	2.75	2.99	2.67	1.12	0.981333	2.88	2.88	3.25	3	4.74	3.37	1.85	3.32	1.11	1	4.92	7.05	10.28	7.42	8.98	9.63	8.71	9.11	1.23	1	2.28	2.48	3.12	2.63	7.33	5.52	6.42	2.45	0.272623
Solyc01g091140.2.1	Nitroreductase (AHRD V1 **-	6.22	4.23	7.15	6.25	5.96	7.82	5.69	7.53	8.34	7.34	1.23	0.74464	6.24	6.4	7.05	6.56	10.45	7.03	5.02	7.5	1.14	1	8.46	12.94	15.92	12.44	13.01	14.89	14.79	14.23	1.14	1	3.49	3.69	3.78	3.65	10.34	8.61	9.47	2.59	0.248204
Solyc01g091150.2.1	Golgi SNAP receptor complex	374.69	351.71	463.97	467.49	414.46	360.25	402.5	394.56	372.92	382.56	0.92	1	423.6	402.54	442.52	422.89	375.38	344.89	374.99	365.09	0.86	0.992763	358.6	438.41	397.34	398.12	337.45	358.26	363.12	352.94	0.89	1	339.67	348.43	320.1	336.07	320.71	348.74	334.73	1	1
Solyc01g091160.2.1	Agmatinase (AHRD V1 ***- Q	44.56	46.42	50.51	25.05	41.64	51.05	35.18	42.94	43.73	43.22	1.04	1	67.52	60.05	56.05	61.21	95.16	74.56	80.14	83.29	1.36	0.286062	36	39.96	28.01	34.66	43.21	40.41	43.68	42.43	1.22	1	31.82	49.33	34.11	38.42	29.04	31.74	30.39	0.79	1
Solyc01g091170.2.1	Agmatinase (AHRD V1 ***- Q	174.95	163.15	218.11	121.94	169.54	161.16	127.06	169.26	181.9	159.84	0.94	1	371.09	125.35	120.15	205.53	186.67	187.31	185.36	186.45	0.91	1	2.04	5.87	2.12	3.34	3.45	1.77	3.97	3.06	0.92	1	1.38	1.55	0.6	1.18	1.43	2.05	1.74	1.48	1
Solyc01g091180.2.1	Peptide transporter-like prote	0.22	0.19	0.51	0.77	0.42	0	0	0	0	0	0.02	0.069996	0.61	0.28	0.18	0.36	0.08	0	0	0.03	0.07	0.313457	0.18	0.09	0	0.09	0.07	0	0	0.02	0.26	1	0	0	0	0	0	0	0	1	NA
Solyc01g091190.2.1	3-phosphoshikimate 1-carbox	57.11	46.43	56.62	55.88	54.01	47.01	52.18	37.6	57.54	48.58	0.9	1	50.06	40.95	41.39	44.13	43.56	42.81	40.8	42.39	0.96	1	89.41	92.04	80.44	87.3	81.02	76.87	65.33	74.41	0.85	1	35.11	52.97	37.77	41.95	58.54	55.51	57.02	1.36	1
Solyc01g091200.2.1	NAD dependent epimerase/d	6.51	6.6	8.39	10.92	8.11	8.91	11.71	9.46	6.41	9.12	1.13	0.88347	6.76	5	4.09	5.28	2.41	4.62	4.4	3.81	0.72	0.896625	2.57	5.18	2.4	3.38	1.99	1.26	2.09	1.78	0.53	1	0.43	0.66	1.16	0.75	0.69	1.19	0.94	1.25	1
Solyc01g091210.2.1	tRNA 2&apos-phosphotransfe	25.86	20.29	18.62	20.15	21.23	20.71	22.32	20.76	24.39	22.05	1.04	1	22.02	23.24	19.25	21.5	26.15	28.82	27.73	27.57	1.28	0.586461	26.03	30.36	23.71	26.7	34.99	31.4	30.03	32.14	1.2	1	35.52	28.34	29.32	31.06	40.53	39.01	39.77	1.28	1
Solyc01g091220.2.1	40S ribosomal protein S13 (A	145.4	145.63	150.03	141.82	145.72	127.85	121.64	126.9	119.03	123.85	0.85	0.919448	117.89	102.62	104.48	108.33	71.04	73.71	84.59	76.45	0.71	0.414577	54.7	67.55	65.48	62.58	40.49	43.42	50.92	44.94	0.72	1	34.56	58.63	65.41	52.87	52.05	58.22	55.13	1.04	1
Solyc01g091230.2.1	Receptor like kinase, RLK	0	0.34	0.11	0.54	0.25	0.11	0.38	0.18	0.14	0.2	0.82	1	0	0.08	0.24	0.11	0.07	0	0	0.02	0.22	1	0	0.08	0	0.03	0	0	0	0	0.38	1	0	0	0	0	0	0	0	1	NA
Solyc01g091240.2.1	Pentatricopeptide repeat-con	2.74	3	0.62	1.19	1.89	3.98	3	3.39	3.03	3.35	1.77	0.353546	1.27	1.74	1.07	1.36	2.12	2.13	2.65	2.3	1.69	0.516545	0.51	0.52	0.73	0.59	0.48	0.4	0.61	0.5	0.85	1	0	1.07	0.49	0.52	0.58	0.28	0.43	0.83	1
Solyc01g091250.2.1	Unknown Protein (AHRD V1);	0	0	0.36	0	0.09	0	0	0	0	0	0.11	1	0	0	0	0	0	0	0	0	1	NA	0	0	0	0	0	0	0	0	1	1	0	0	0	0	0	0	0	1	NA
Solyc01g091260.2.1	AT2G45380 protein (Fragmer	40.96	25.33	25.94	20.13	28.09	16.78	26.21	11.5	6.81	15.33	0.55	0.341162	21.66	14.62	26.59	20.96	23.03	35.27	27.79	28.7	1.37	0.494761	10.8	51.3	14.12	25.41	15.58	9.48	21.37	15.48	0.61	1	9.2	6.58	9.95	8.58	15	37.43	26.21	3.06	0.41513
Solyc01g091270.2.1	AT2G45380 protein (Fragmer	39.41	35.46	33.39	24.33	33.15	26.86	28.19	32.23	47.52	33.7	1.02	1	44.35	57.35	43.22	48.31	94.23	78.57	70.5	81.1	1.68	0.016788	195.6	292.21	217.19	235	337.55	366.02	309.07	337.55	1.44	1	373.64	571.98	514.93	486.85	478.94	405.85	442.39	0.91	1
Solyc01g091280.2.1	Palmitoyltransferase PFA4 (A	38.31	34.66	42.18	40.34	38.87	27.44	30.41	33.85	36.82	32.13	0.83	0.938146	42.76	41.44	51.95	45.38	53.43	47.71	40.81	47.32	1.04	1	45.22	52.89	45.84	47.98	48.51	42.1	53.81	48.14	1	1	98.03	23.65	37.31	53	50.84	55.79	53.31	1.01	1
Solyc01g091290.2.1	Sec1-5 syntaxin-binding prote	0	0.31	0.42	0	0.18	0.78	0	0	0	0.2	1.07	1	0	0	0	0	0	0	0	0	1	NA	0.29	0	0	0.1	0	0	0	0	0.1	1	0	0	0	0	0	0	0	1	NA
Solyc01g091300.2.1	Sec1-4 syntaxin-binding prote	0.5	0.22	0.44	0	0.29	0	0.1	0	0.36	0.11	0.4	0.947105	0.23	0.22	0	0.15	0	0	0	0	0.07	0.832635	0.3	0.72	0.75	0.59	0.16	0	0.18	0.11	0.19	1	0.18	0	0	0.06	0	0	0	0.17	1
Solyc01g091310.2.1	WD-40 repeat protein (AHRD	36.27	32.81	27.29	34.62	32.75	30.2	34.64	26.07	29.57	30.12	0.92	1	33.04	35.68	31.89	33.54	30.54	31.61	34.37	32.17	0.96	1	36.3	47.55	44	42.62	39.71	38.12	44.16	40.66	0.95	1	32.89	51.6	45.9	43.46	49.19	57.19	53.19	1.22	1
Solyc01g091320.2.1	Sterol 4-alpha-methyl-oxidase	2.19	1.95	2.33	32.86	9.83	26.31	18.69	67.31	80.41	48.18	4.9	0.07018	0	0	0.37	0.12	0	0	0	0	0.08	1	0	0.18	0.22	0.13	0	0	0	0	0.07	1	0	0	0	0	0	0	0	1	NA
Solyc01g091330.2.1	Maleylacetoacetate isomeras	3.47	5.36	4.37	4.58	4.45	5.4	6.4	8.94	6.48	6.81	1.53	0.463864	0.86	1.01	1.17	1.01	0.64	0.36	0.55	0.52	0.51	0.943066	1.87	2.07	2.3	2.08	0	0.35	0.17	0.17	0.08	0.781861	0	0.17	0	0.06	0	0	0	0.18	1
Solyc01g091340.2.1	Peptidyl-prolyl cis-trans isom	11.59	10	11	7.27	9.96	5.85	7.27	9.78	9.61	8.13	0.82	0.902802	10.01	9.25	9.59	9.62	7.21	8.35	7.61	7.72	0.8	0.962255	4.34	6.62	4.98	5.31	6.12	4.44	4.18	4.91	0.92	1	14.44	9.64	10.11	11.4	17.83	14.57	16.2	1.42	0.933683
Solyc01g091350.2.1	ATP-dependent DNA helicase	10.31	9.18	15.02	8.81	10.83	6.82	5.58	9.06	7.07	7.13	0.66	0.29637	10.8	9.69	11.62	10.7	10.12	7.89	8.4	8.8	0.82	0.945984	11.26	11.36	11.71	11.44	9.71	12.04	7.85	9.87	0.86	1	13.34	13.56	12.43	13.11	9.78	11.73	10.75	0.82	1
Solyc01g091360.2.1	Homology to unknown gene (	9.99	7.54	14.64	3.42	8.9	17.49	10.03	12.8	9.34	12.41	1.4	0.808659	7.04	2.63	2.56	4.08	2.1	1.88	2.38	2.12	0.52	0.832688	0	0.99	0.3	0.43	0.6	0.23	0.44	0.42	0.98	1	0	0	0.47	0.16	0	0	0	0.06	1
Solyc01g091370.2.1	AT-hook motif nuclear localiz	11.79	13.07	11.47	19.01	13.84	11.95	17.31	11.22	9.06	12.38	0.9	1	7.89	12.22	11.35	10.49	5.31	5.89	6.01	5.74	0.55	0.182139	1.34	1.18	0.41	0.98	0.95	0.16	0.15	0.42	0.43	1	1.18	1.5	1.59	1.42	0.95	2.54	1.75	1.23	1
Solyc01g091380.2.1	Microtubule-associated prote	2.83	2.92	3.24	2.76	2.94	2.56	2.38	2.85	3.18	2.74	0.93	1	3.21	1.77	3.22	2.73	3.32	2.15	2.78	2.75	1.01	1	0.5	1.08	0.97	0.85	0.93	0.4	0.52	0.62	0.73	1	0.63	0.13	0.41	0.39	0.49	0.39	0.44	1.13	1
Solyc01g091390.2.1	Unknown Protein (AHRD V1)	7.97	9.97	12.85	13.7	11.12	6.62	6.77	8.16	8.4	7.49	0.67	0.462717	10.04	9.91	13.8	11.25	10.56	10.65	9.12	10.11	0.9	1	9.94	11.84	12.52	11.43	9.74	11.09	7.42	9.42	0.82	1	19.51	18.32	22.03	19.95	13.76	15.12	14.44	0.72	1
Solyc01g091400.2.1	LOB domain protein 31 (AHRI	0	0	0	0	0	0	0	0	0	0	1	NA	0	0	0	0	0	0	0	0	1	NA	0	0	0	0	0	0	0	0	1	NA	0	0	0	0	0	0	0	1	NA
Solyc01g091410.2.1	Acetyl esterase (AHRD V1 *--	0	0	0	0	0	0	0	0	0	0	1	NA	0	0	0.17	0.06	0	0	0	0	0.18	1	0	0	0	0	0	0	0	0	1	NA	0	0	0	0	0	0	0	1	NA
Solyc01g091420.1.1	LOB domain protein 30 (AHRI	0	0	0.29	0.69	0.24	0	0.19	0	0	0.05	0.19	1	0.23	0	0.41	0.21	0.17	0	0	0.06	0.27	1	6.86	2.77	4.57	4.73	4.3	1.84	1.06	2.4	0.51	1	0.34	1.06	0.37	0.59	0	0.64	0.32	0.54	1
Solyc01g091430.1.1	ATP-dependent DNA helicase	1.04	1.85	0.61	2.2	1.43	2.89	0	2.87	1.86	1.9	1.34	1	2.42	0.45	1.75	1.54	0.72	1.88	0.82	1.14	0.74	1	1.68	0	0.52	0.73	0	0.39	0	0.13	0.18	1	1.48	0.38	2.4	1.42	0.95	0.46	0.7	0.5	1
Solyc01g091440.2.1	ATP-dependent DNA helicase	1.78	3.97	4.21	5.04	3.75	1.98	1.41	1.64	1.92	1.74	0.46	0.852275	3.32	3.1	0	2.14	1.24	0.46	4.2	1.97	0.92	1	0.72	0	0.88	0.53	0.59	0.68	1.3	0.86	1.61	1	0	1.3	2.74	1.35	0	0.78	0.39	0.29	1
Solyc01g091450.2.1	Pentatricopeptide repeat-con	2.7	3.95	4.12	2.02	3.2	3.88	4.63	3.94	4.44	4.22	1.32	0.788276	3.98	2.34	3.75	3.36	6.82	5.01	5.86	5.9	1.76	0.247159	3.72	4.78	2.83	3.78	5.21	4.45	5.43	5.03	1.33	1	1.8	3.34	4.15	3.1	3.92	4.17	4.04	1.31	1
Solyc01g091460.2.1	ARF guanine-nucleotide exch	12.79	12.64	13.13	19.51	14.52	8.12	14.2	8.69	10.65	10.41	0.72	0.686269	14.11	12.64	17.51	14.75	10.98	10.41	12.15	11.18	0.76	0.59019	8.87	11.51	10.12	10.17	9.5	6.38	9.49	8.46	0.83	1	18.51	12.52	13.51	14.85	11.61	15.61	13.61	0.92	1
Solyc01g091470.2.1	AT-hook DNA-binding protein	0	0	0.26	0	0.07	0	0	0	0	0	0.15	1	0.2	0.19	0.18	0.19	0	0	0.17	0.06	0.3	1	0.18	0.18	0	0.12	0.14	0	0	0.05	0.39	1	0	0.16	0	0.05	0	0	0	0.19	1
Solyc01g091480.2.1	Kinesin (AHRD V1 *-*- D2VUV	6.59	6.8	7.69	7.52	7.15	6.41	7.15	6.06	6.45	6.52	0.91	1	6.2	5.47	5.82	5.83	5.71	6.63	5.24	5.86	1.01	1	3.69	5	4.61	4.43	4.82	4.37	3.87	4.35	0.98	1	12.14	8.1	11.4	10.55	10.69	11.75	11.22	1.06	1

Solyc01g091490.2.1	Unknown Protein (AHRD V1)	0	0	0	0	0	0	0	0	0	0	1	NA	0	0	0	0	0.4	0	0	0.13	13.33	1	0	0	0	0	0	0	0	0	1	NA	0	0	0	0	0	0	0	1	NA
Solyc01g091500.2.1	Forkhead-associated domain-	5.35	4.94	5.64	5.07	5.25	4.93	4.66	4.07	4.39	4.51	0.86	1	3.79	4.16	7.25	5.07	4.59	5.97	4.37	4.98	0.98	1	2.9	3.55	2.3	2.92	4.3	2.72	3.18	3.4	1.17	1	7	6.23	7.79	7.01	4.51	6.41	5.46	0.78	1
Solyc01g091510.2.1	Ankyrin repeat and zinc finge	15	13.69	21.17	19.74	17.4	11.82	11.47	12.04	10.49	11.46	0.66	0.291626	14.66	17.91	21.33	17.97	13.76	15.14	17.46	15.45	0.86	1	10.05	9.82	9.61	9.83	9.79	9.64	8.58	9.34	0.95	1	29.15	26.13	28.17	27.82	16.86	17.31	17.09	0.61	0.759291
Solyc01g091520.2.1	Enoyl-CoA hydratase/isomera	98.82	91.96	109.09	116.55	104.11	96.89	105.84	80.14	93.01	93.97	0.9	1	97.59	102.03	125.76	108.46	144.62	109.47	91.82	115.3	1.06	1	14.98	25.04	15.25	18.42	32.55	20.68	25.87	26.37	1.43	0.967485	168.76	185.89	215.52	190.06	79.55	79.68	79.62	0.42	0.078995
Solyc01g091530.2.1	Fasciclin-like arabinogalactan	127.97	122.95	180.23	253.41	171.14	156.03	194.53	88.86	123.71	140.78	0.82	1	129.09	99.49	177.77	135.45	82.8	118.03	104.56	101.8	0.75	0.833673	5.39	8.42	4.58	6.13	10.03	5.06	4.17	6.42	1.05	1	2.71	1.5	3.42	2.54	10.77	26.2	18.48	7.27	0.079073
Solyc01g091540.1.1	Growth-regulating factor 12 (	0	0.14	0.55	0.22	0.23	0	0	0	0	0	0.04	0.62439	0	0	0	0	0	0	0	0	1	NA	0	0	0	0	0	0	0	0	1	NA	0	0.11	0	0.04	0	0	0	0.27	1
Solyc01g091550.2.1	Thioesterase family protein (A	42.51	40.12	35.46	30.07	37.04	49.3	46.01	51.89	41.57	47.19	1.27	0.625626	33.41	43.01	29.72	35.38	47.54	41.56	43.49	44.2	1.25	0.700321	36.72	38.53	22.55	32.6	25.04	26.11	28.58	26.58	0.82	1	14.07	25.29	14.65	18	18.12	22.82	20.47	1.14	1
Solyc01g091560.2.1	Thioesterase family protein (A	0	0	0	0	0	0	0	0	0	0	1	NA	0	0	0	0	0	0	0	0	1	NA	0	0	0	0	0	0	0	0	1	NA	0	0	0	0	0	0	0	1	NA
Solyc01g091570.2.1	Thioesterase family protein (A	30.94	25.87	25.06	27.94	27.45	26.52	23.83	28.79	21.14	25.07	0.91	1	26	28.19	21.09	25.09	18.1	17.18	20.21	18.5	0.74	0.751893	5.72	10.93	5.18	7.28	3.43	5.65	7.06	5.38	0.74	1	23.32	28.69	24.65	25.55	19.45	17.32	18.38	0.72	1
Solyc01g091580.2.1	Signal recognition particle-do	28.78	34.37	17.51	17.7	24.59	44.91	46.86	46.36	45.18	45.83	1.86	0.007497	26.44	26.58	19.78	24.27	32.36	46.28	47.07	41.9	1.73	0.01473	23.25	26.95	21.47	23.89	30.89	25.34	36.87	31.03	1.3	1	9.91	24.15	11	15.02	20.78	17.24	19.01	1.27	1
Solyc01g091590.2.1	SRC2 protein (AHRD V1 ***- /	0	0	1.22	2.44	0.92	0	0.82	0.32	0	0.28	0.31	0.9965	0	0.9	2.33	1.08	0	0.18	0.27	0.15	0.14	0.748186	0.56	0.28	5.83	2.22	0.91	0	0.25	0.39	0.17	1	0.98	18.06	5.31	8.12	0.95	0.91	0.93	0.11	0.895889
Solyc01g091600.2.1	RING finger family protein (A	30.43	25.69	27.8	28.61	28.13	18.77	20.77	23.83	24.95	22.08	0.78	0.796773	34.88	36.18	37.98	36.35	33.09	29.79	26.08	29.65	0.82	0.918752	16.81	30.24	19.29	22.11	22.31	14.58	17.74	18.21	0.82	1	23.17	19.54	19.32	20.68	26.06	26.86	26.46	1.28	1
Solyc01g091610.1.1	Pentatricopeptide repeat-con	1.03	1.18	1.82	1.45	1.37	1.31	0.98	0.81	0.74	0.96	0.7	0.836198	0.75	0.96	1.3	1	1.02	1.1	1.21	1.11	1.11	1	0.83	0.6	0.36	0.6	0.87	0.56	1.02	0.82	1.37	1	0.21	1.17	1.24	0.87	0.94	0.9	0.92	1.05	1
Solyc01g091620.2.1	Glycogen synthase kinase (AH	61.26	61.8	60.5	75.28	64.71	60.55	78.89	54.03	48.06	60.38	0.93	1	46.54	65.54	71.17	61.08	66.62	69.89	62.3	66.27	1.08	0.991848	59.46	58.16	54.34	57.32	54.54	52.84	52.01	53.13	0.93	1	121.58	64.62	54.99	80.4	58.23	66.71	62.47	0.78	1
Solyc01g091630.2.1	Homeobox-leucine zipper pro	25.76	25.93	28.88	43.14	30.93	22.21	29.34	21.69	19.48	23.18	0.75	0.767178	24.93	49.32	58.57	44.27	30.45	26.64	24.66	27.25	0.62	0.572617	69.18	73.05	72.57	71.6	61.9	44.83	37.66	48.13	0.67	1	13.81	31.48	25.05	23.45	21.16	22.38	21.77	0.93	1
Solyc01g091640.2.1	Dehydration responsive prote	41.01	44.72	38.79	60.19	46.18	32.88	50.78	33.87	39.07	39.15	0.85	1	42.96	37.66	37.98	39.53	19.68	24.65	29.72	24.68	0.62	0.103298	40.42	38.71	33.4	37.51	32	24.42	26.36	27.59	0.74	1	15.95	16.53	14.54	15.67	21.04	23.52	22.28	1.42	0.984877
Solyc01g091650.2.1	COP9 signalosome complex s	33.81	31.95	36.71	34.07	34.13	31.43	39.8	29.57	32.11	33.23	0.97	1	37.57	35.63	38.79	37.33	30.82	28.12	32.33	30.42	0.81	0.869472	37.83	38.15	39.05	38.34	29.58	34.01	32.05	31.88	0.83	1	43.18	40.64	44.4	42.74	36.29	35.94	36.11	0.84	1
Solyc01g091660.2.1	Alcohol dehydrogenase (Frag	0	0	0	0	0	0	0	0	0	0	1	NA	0	0	0	0	0	0	0	0	1	NA	0	0	0	0	0	0	0	0	1	1	0	0	0.39	0.13	0	0	0	0.08	1
Solyc01g091670.2.1	Progesterone 5-beta-reductas	0	0	0	0	0	0	0	0	0	0	1	NA	0	0	0	0	0	0	0	0	1	NA	0	0	0	0	0	0	0	0	1	NA	0	0	0	0	0	0	0	1	NA
Solyc01g091680.2.1	Ulp1 protease family C-termi	1.14	2.02	1.34	0	1.12	1.26	3.59	1.05	0	1.47	1.31	1	1.06	1.98	1.92	1.65	0.79	2.94	1.79	1.84	1.11	1	0.92	0	0	0.31	0	0.86	0	0.29	0.93	1	0	0	0	0	0	0	0	1	NA
Solyc01g091690.2.1	Dehydration-responsive famil	36.25	44.7	59.75	93.61	58.58	40.87	57.4	31.99	38.14	42.1	0.72	0.941851	55.01	95.79	130.95	93.92	105.09	92.82	73.41	90.44	0.96	1	175.63	386.6	394.02	318.75	262.48	196.63	454.17	304.43	0.96	1	57.28	84.77	60.2	67.42	98.12	88.63	93.38	1.39	1
Solyc01g091700.2.1	F-box protein PP2-B1 (AHRD \	2.52	3.47	4.32	2.92	3.31	4.07	3.07	1.9	1.64	2.67	0.81	1	1.28	2.99	2.71	2.33	2.54	1.42	2.7	2.22	0.95	1	1.85	1.68	1.14	1.56	1.2	1.22	0	0.81	0.52	1	1.3	1.5	0	0.93	0	0.2	0.1	0.11	0.850663
Solyc01g091710.2.1	F-box family protein (AHRD V	13.75	16.11	20.95	21.67	18.12	24.77	20.06	18.89	14.25	19.49	1.08	0.989253	15.87	15.74	19.41	17.01	16.42	19.11	15.05	16.86	0.99	1	17.86	21.42	19.52	19.6	16.92	15.44	18.39	16.92	0.86	1	13.36	9.23	13.38	11.99	11.79	12.36	12.07	1.01	1
Solyc01g091720.2.1	F-box family protein (AHRD V	20.59	21.88	22.8	17.83	20.77	28.26	32.01	32.45	23.98	29.18	1.4	0.307051	23.06	27.94	20.05	23.68	35.41	31.11	35.33	33.95	1.43	0.265246	29.03	31.67	27.32	29.34	28.02	28.34	36.74	31.03	1.06	1	24.79	30.37	25.2	26.79	30.58	27.82	29.2	1.09	1
Solyc01g091730.2.1	Peroxisomal membrane prote	311.81	283.6	397.24	366.22	339.72	389.49	486.92	382.82	361.47	405.18	1.19	0.546606	317.25	315.95	352.2	328.47	414.83	319.99	306.67	347.16	1.06	1	206.66	231.77	207.57	215.33	206.47	198.59	172.79	192.62	0.89	1	477.84	409.99	307.21	398.35	336.71	339.42	338.06	0.85	1
Solyc01g091740.2.1	Armadillo/beta-catenin repea	13.91	14.18	16.69	16.14	15.23	12.82	10.32	12.24	12.12	11.88	0.78	0.760208	21.01	15.6	19.58	18.73	11.46	15.32	13.36	13.38	0.71	0.585775	6.05	6.78	7.08	6.64	5.47	5.68	4.68	5.28	0.8	1	23.66	14.76	13.53	17.32	16.21	24.18	20.2	1.17	1
Solyc01g091750.2.1	Arginine/serine-rich splicing f	59.65	60.59	33.05	41.33	48.66	53.24	59.46	51.52	53.2	54.36	1.12	0.966074	60.8	68.08	60.81	63.23	45.13	55.99	52.32	51.15	0.81	0.905074	56.71	60.39	42.16	53.09	54.02	49.54	46.54	50.03	0.94	1	63.39	57.1	67.63	62.71	43.85	51.77	47.81	0.76	1
Solyc01g091760.2.1	Ethylene responsive transcrip	0	0	0	0	0	0	0	0	0	0	1	NA	0	0	0	0	0	0	0	0	1	NA	0	0	0	0	0	0	0	0	1	NA	0	0	0	0	0	0	0	1	NA
Solyc01g091770.2.1	Ring H2 finger protein (AHRD	3.24	5.45	3.4	7.14	4.81	5.61	7.4	2.65	3.88	4.89	1.02	1	1.68	7.83	4.57	4.69	4	5.41	6.23	5.21	1.11	1	0	0	0	0	0	0	0	0	1	1	0	0	0	0	0	0.32	0.16	16	1
Solyc01g091780.2.1	Fructosamine kinase family p	30.02	18.46	13.28	13.13	18.72	35.35	34.62	29.24	35.03	33.56	1.79	0.019864	17.45	19.75	19.02	18.74	31.23	37.33	37.87	35.48	1.89	0.005626	5.9	18.35	8.04	10.76	7.27	8.53	15.98	10.59	0.98	1	4.49	5.02	3.64	4.38	9.58	7.45	8.52	1.94	0.793391
Solyc01g091790.2.1	Unknown Protein (AHRD V1)	11.39	10.83	13.44	10.55	11.55	4.37	10.23	7.58	5.92	7.03	0.61	0.513554	5.48	15.02	10.95	10.48	18.79	14.23	10.8	14.61	1.39	0.814184	6.98	7.68	4.68	6.45	9.03	5.06	12.02	8.7	1.35	1	3.35	10.55	13.28	9.06	6.11	5.51	5.81	0.64	1
Solyc01g091800.2.1	Ribosomal RNA small subunit	13.7	12.19	13.82	17.34	14.26	47.49	43.97	31.99	37.4	40.21	2.82	9.16E-08	12.91	12.53	17.81	14.42	25.75	22.4	21.53	23.23	1.61	0.095802	3.93	7.64	4.65	5.41	6.51	3.69	9.85	6.68	1.24	1	0.77	2.09	1.39	1.42	1.49	2.84	2.17	1.53	1
Solyc01g091810.1.1	Response regulator 10 (AHRD	0	0	0	0	0	0	0	0	0	0	1	NA	0	0	0	0	0	0	0	0	1	NA	0	0	0	0	0	0	0	0	1	NA	0	0	0	0	0	0	0	1	NA
Solyc01g091820.2.1	Flap structure-specific endon	6.12	6.12	6.44	6.03	6.18	5.1	4.74	6.73	5.41	5.5	0.89	1	6.5	5.41	6.18	6.03	5.61	5.03	4.55	5.06	0.84	1	4.77	3.2	3.25	3.74	3.16	2.24	3.18	2.86	0.76	1	6.37	5.32	7.05	6.25	5.3	5.94	5.62	0.9	1
Solyc01g091830.2.1	Unknown Protein (AHRD V1)	0	0	0	0	0	0	0	0	0	0	1	NA	0	0	0	0	0	0	0	0	1	NA	0	0	0	0	0	0	0	0	1	NA	0	0	0	0	0	0	0	1	NA
Solyc01g091840.2.1	3&apos-phosphoadenosine 5	0.37	0.49	0.65	0.52	0.51	0.62	1.17	0.34	0.13	0.56	1.11	1	0.69	0.96	1.09	0.91	1.79	1.34	1.16	1.43	1.57	0.940618	1.05	1.66	1.83	1.51	1.7	1.12	1.21	1.34	0.89	1	2.36	2.82	3.69	2.96	4.4	6.81	5.61	1.9	0.840666
Solyc01g091850.2.1	Integral membrane protein (A	0.5	1.32	2.05	1.05	1.23	0.83	0.78	0.91	1.42	0.98	0.8	1	0.23	0.86	2.3	1.13	0.86	0.51	0.39	0.59	0.52	0.99168	0.4	0	0.25	0.22	0.49	0	0.72	0.4	1.86	1	0	0	0	0	0.23	0.22	0.23	22.5	1
Solyc01g091860.2.1	SET domain-containing protei	13.8	11.14	12.42	9.76	11.78	10.14	9.97	9.79	11.25	10.29	0.87	1	13.27	14.34	11.56	13.06	12.01	9.39	12.5	11.3	0.87	1	5.86	8.86	6.5	7.07	4.99	5.32	7.16	5.82	0.82	1	22.45	19.28	19.61	20.45	8.46	12.41	10.44	0.51	0.443193
Solyc01g091870.2.1	Major facilitator superfamily	0	0	0	0	0	0	0	0	0	0	1	NA	0	0	0	0	0	0	0	0	1	NA	0	0	0	0	0	0	0	0	1	NA	0	0	0	0	0	0	0	1	NA
Solyc01g091880.2.1	ATPase (AHRD V1 ***- D3E0U	26.68	25.86	27.81	23.52	25.97	19.91	22.65	20.33	18.5	20.35	0.78	0.729523	31.79	29.23	19.29	26.77	23.58	24.13	27	24.9	0.93	1	41.49	38.42	32.11	37.34	23.41	23.96	30.5	25.96	0.7	1	10.91	19.51	10.12	13.51	22.25	22.59	22.42	1.66	0.937753
Solyc01g091890.2.1	Ubiquitin-related modifier 1	100.94	125.65	94.96	84.92	101.62	88.73	81.77	109.79	82.88	90.79	0.89	0.988051	125.18	114.12	108.66	115.99	102.49	95.64	109.39	102.51	0.88	1	74.62	83.32	49.77	69.24	46.37	63.5	67.3	59.06	0.85	1	62.84	59.89	64.93	62.55	76.7	74.62	75.66	1.21	1
Solyc01g091900.2.1	Peroxisomal membrane prote	12.34	14.8	14.34	15.68	14.29	11.33	13.13	11.67	13.61	12.44	0.87	1	13.3	14.76	13.14	13.73	16	15.46	14.05	15.17	1.1	1	11.99	13.55	12.78	12.77	11.16	8.4	9.12	9.56	0.75	1	20.84	25.97	25.02	23.94	16.53	18.65	17.59	0.73	1
Solyc01g091910.2.1	Phospholipase D (AHRD V1 **	5.89	5.11	7.99	16.35	8.84	3.03	5.29	4.47	5.29	4.52	0.51	0.720001	5.27	9.98	16.54	10.6	5.72	6.21	3.82	5.25	0.5	0.627841	3.09	6.42	9.94	6.48	7.93	4.97	4.02	5.64	0.87	1	2.72	9.89	7.02	6.54	6.47	10.07	8.27	1.26	1
Solyc01g091920.2.1	Subtilisin-like protease (AHRD	0.09	0.15	0.1	0	0.08	0	0.07	0	0.12	0.05	0.56	1	0	0	0	0	0	0	0	0	1	NA	0	0	0	0	0	0	0	0	1	NA	0	0	0	0	0	0	0	1	NA
Solyc01g091930.2.1	Subtilisin-like serine protease	0.29	0.26	0.11	0.14	0.2	0.21	0.23	0.18	0.07	0.17	0.86	1	0.36	0.42	0.16	0.31	0.2	0.15	0.08	0.14	0.46	0.979744	0.16	0.08	0.1	0.11	0.19	0.29	0	0.16	1.41	1	0.27	0.35	0	0.21	0	0.34	0.17	0.82	1
Solyc01g091940.2.1	CRS2-associated factor 1, chl	4.16	5.7	6.23	7.93	6	4.27	6.19	6.92	5.17	5.64	0.94	1	6.11	6.26	6.49	6.29	6.89	7.21	6.04	6.71	1.07	1	9.06	8.48	8.58	8.71	6.21	6.67	8.28	7.05	0.81	1	10.24	10	9.36	9.87	6.45	6.6	6.53	0.66	1
Solyc01g091950.2.1	1-acylglycerophosphocholine	27.52	32.62	39.41	29.75	32.33	36.06	20.87	28.39	28.64	28.49	0.88	0.982298	29.51	36.87	43.95	36.78	65.01	44.61	46.84	52.15	1.42	0.360948	15.24	13.9	10.97	13.37	12.24	10.11	10.51	10.95	0.82	1	50.74	28.1	28.09	35.64	34.87	54.68	44.77	1.26	1
Solyc01g092950.2.1	MADS-box transcription facto	0	0	0.37	0	0.09	0	0	0.29	0	0.07	0.78	1	0	0	0	0	0.22	0	0	0.07	7.33	1	0	0	0	0	0	0	0	0	1	NA	0	0	0	0	0	0	0	1	NA
Solyc01g093960.2.1	MADS box transcription facto	0.52	0.23	0	0	0.19	0.29	0.21	0.24	0	0.18	0.99	1	0.49	0.45	0	0.31	0.18	0	0	0.06	0.19	1	0	0	0.52	0.17	0.17	0.4	0.19	0.25	1.46	1	0	0	0	0	0	0.23	0.12	11.5	1
Solyc01g093970.2.1	Glycosyltransferase (AHRD V1	16.58	14.76	16.26	23.29	17.72	17.89	21.95	15.58	16.28	17.93	1.01	1	19.85	14.52	19.88	18.08	15.65	17.55	21.95	18.38	1.02	1	24.24	29.51	25.3	26.35	25.96	25.79	33.23	28.33	1.08	1	8.67	11.78	15.75	12.07	16.74	18.5	17.62	1.46	0.92381
Solyc01g093980.2.1	Gibberellin 20-oxidase 4 (AHF	0.18	0	0	0	0.04	0	0	0	0.13	0.03	0.72	1	0	0	0	0	0.38	0	0	0.13	12.67	1	0.15	0	0	0.05	0	0	0	0	0.2	1	0	0	0	0	0	0	0	1	NA
Solyc01g093990.2.1	Glyoxylate reductase/hydroxy	24.03	25.19	23.3	23.04	23.89	44.36	33.76	33.51	33.04	36.17	1.51	0.148599	24.24	19.47	23.9	22.54	25.56	23.93	34.4	27.96	1.24	0.751893	1.65	5.97	2.22	3.28	1.07	1.39	3.26	1.91	0.58	1	0.29	0.15	0	0.15	0.56	0.36	0.46	3.14	1
Solyc01g094000.2.1	Poly(A) RNA polymerase (AHF	20.74	16.87	26.78	20.92	21.33	12.38	15.84	13.99	18.37	15.14	0.71	0.511579	21.27	20.25	25.99	22.5	27.06	23.77	22.18	24.34	1.08	1	14.9	16.49	12.15	14.51	18.21	16.04	18.83	17.69	1.22	1	24.17	22.35	20.52	22.35	25.14	27.8	26.47	1.18	1
Solyc01g094010.2.1	CXE carboxylesterase (AHRD \	154.06	175.45	79.21	91.39	125.03	124.88	104.32	118.46	127.04	118.67	0.95	1	128.87	137.63	71.95	112.82	35.26	43.2	51.84	43.43	0.38	0.030351	7.21	11.07	4.04	7.44	5.23	2.35	3.68	3.75	0.5	1	0.55	0.56	0.6	0.57	0.71	1.02	0.86	1.52	1
Solyc01g094020.2.1	Receptor kinase-like protein (	13.39	15.26	13.41	17.57	14.91	8.99	15.05	9.65	14.17	11.96	0.8	0.919033	14.6	16.07	15.78	15.48	12.96	21.44	14.61	16.34	1.06	1	6.38	7.06	2.96	5.47	7.91	4.01	6.39	6.1	1.12	1	0.14	0.42	0.15	0.24	0.53	1.18	0.85	3.61	0.926032
Solyc01g094030.2.1	Syt1 synaptotagmin Ca2+-dep	48.24	54.54	50.45	48.32	50.39	38.12	47.14	45.31	45.3	43.97	0.87	0.995038	50.94	49.07	54.07	51.36	37.26	42.26	38.52	39.35	0.77	0.698541	50.03	51	53.45	51.49	49.85	45.75	40.09	45.23	0.88	1	40.04	45.74	39.9	41.89	46.9	41.15	44.02	1.05	1
Solyc01g094040.2.1	Plastocyanin-like domain con	71.47	68.23	58.73	92.45	72.72	59.75	99.34	51.67	55.91	66.67	0.92	1	48.3	98.76	73	73.35	39.4	45.86	47.28	44.18	0.6	0.401947	28.62	42.31	31.23	34.05	43.34	35.88	41.84	40.35	1.19	1	152.42	151.29	219.33	174.35	176.42	192.27	184.34	1.06	1
Solyc01g094050.1.1	Os04g0555600 protein (Fragr	4.1	5.79	4.97	6.49	5.34	1.52	3.97	2.51	5.06	3.26	0.61	0.492591	5.44	3.99	5.45	4.96	4.74	4.3	4.64	4.56	0.92	1	4.81	5.17	3.42	4.47	3.2	2.98	4.9	3.69	0.83	1	3.5	2.06	3.28	2.95	4.6	4.6	4.6	1.56	0.766894
Solyc01g094060.2.1	Beige/BEACH domain contain	8.04	7.61	9.44	11.16	9.06	4.82	8.41	5.54	9.2	6.99	0.77	0.900519	8.06	7.18	11.19	8.81	7.72	7.28	6.64	7.21	0.82	0.904867	6.44	8.41	8.31	7.72	6.37	5.62	7.41	6.47	0.84	1	8.56	5.17	6.94	6.89	7.53	8.34	7.94	1.15	1
Solyc01g094070.2.1	Dihydroflavonol 4-reductase	0	0	0	0	0	0	0	0	0	0	1	NA	0	0	0	0	0	0	0	0	1	NA	0	0	0	0	0	0	0	0	1	NA	0	0	0	0	0	0	0	1	NA
Solyc01g094080.2.1	Cytochrome P450	40.45	44.4	40.35	37.72	40.73	69.5	60.54	65.35	50.2	61.4	1.51	0.099728	48.55	55.79	43.11	49.15	62.39	69.31	70.9	67.53	1.37	0.229832	53.5	81.83	72.07	69.13	85.5	108.51	100.15	98.05	1.42	1	55.52	138.74	96.34	96.87	146.01	111.21	128.61	1.33	1
Solyc01g094090.2.1	Enoyl-CoA-hydratase (AHRD V	0	0	0	0	0	0	0	0	0	0	1	NA	0	0	0	0	0	0	0	0	1	NA	0	0	0	0	0	0.15	0	0.05	5	NA	0	0	0	0	0	0	0	1	NA
Solyc01g094100.2.1	Cytochrome P450	0	0	0	0	0	0	0	0	0	0	1	NA	0	0	0	0	0	0	0	0	1	NA	0	0	0	0	0	0	0	0	1	NA	0	0	0	0	0	0.11	0.06	5.5	1
Solyc01g094110.1.1	Legumin 11S-globulin (AHRD	0	0	0	0	0	0	0	0	0	0	1	NA	0	0	0	0	0	0	0	0	1	NA	0	0	0	0	0	0	0.13	0.04	4.33	1	0	0	0	0	0	0.16	0.08	8	1
Solyc01g094120.2.1	Cytochrome P450	5.82	3.45	6.71	4.76	5.19	9.06	8.27	6.9	6.78	7.75	1.5	0.289207	5.17	7.76	5.03	5.99	6.1	7.23	8.44	7.26	1.21	0.896625	8.15	10.54	9.24	9.31	9.01	13.73	12.16	11.63	1.25	1	8.09	15.88	9.15	11.04	15.99	11.23	13.61	1.23	1
Solyc01g094130.2.1	Cytochrome P450	0	0	0	0	0	0	0.2	0	0.09	0.07	7.25	1	0.12	0	0.22	0.11	0.09	0.27	0.1	0.15	1.35	1	0.1	0.21	0	0.1	0	0	0.09	0.03	0.29	1	0.18	0	0	0.06	0.12	0.11	0.11	1.92	1
Solyc01g094140.2.1	Cytochrome P450	0.51	0.34	0.3	0.36	0.38	0.43	0.5	0.35	0.18	0.36	0.97	1	2.38	2.45	3.46	2.76	1.51	2.58	4.02	2.7	0.98	1	0.1	0.52	0.13	0.25	0.42	0.39	0.37	0.39	1.57	1	0.18	0.19	0	0.12	0.23	0	0.12	0.93	1
Solyc01g094150.2.1	26S protease regulatory subu	14.48	13.84	16.14	16.12	15.14	11.77	13.13	12.45	14.44	12.95	0.85	1	17.09	19	18.01	18.03	14.79	12.77	12.75	13.44	0.75	0.673688	20.47	21.63	22.36	21.49	17.26	16.26	18.57	17.36	0.81	1	20.21	21.23	20.21	20.55	12.15	14.11	13.13	0.64	0.845719
Solyc01g094160.1.1	Unknown Protein (AHRD V1)	0	0	0	0	0	0	0	0	0	0	1	NA	0	0	0	0	0	0	0	0	1	NA	0	0	0	0	0	0	0	0	1	NA	0	0	0	0	0	0	0	1	NA
Solyc01g094170.2.1	Digalactosyldiacylglycerol syn	9.07	7.93	11.99	10.39	9.85	8.34	5.18	8.91	6.85	7.32	0.74	0.71821	5.82	8.97	10.56	8.45	12.25	11.01	10.93	11.4	1.35	0.596758	6.31	13.38	16.91	12.2	16.84	15.88	12.99	15.24	1.25	1	128.25	33.84	64.91	75.67	69.84	81.69	75.77	1	1
Solyc01g094180.2.1	Unknown Protein (AHRD V1)	3.25	1.24	2.74	1.31	2.14	2.07	4.4	2.99	1	2.62	1.22	1	3.03	1.21	2.75	2.33	0	1.44	4.38	1.94	0.83	1	0	1.51	0.46	0.66	0.92	1.41	1.35	1.23	1.87	1	1.98	1.35	2.14	1.82	1.28	1.63	1.46	0.8	1
Solyc01g094190.2.1	dTDP-4-dehydrorhamnose re	11.57	12.83	10.54	12.92	11.96	11.96	17.63	13.62	15.01	14.55	1.22	0.706929	11.33	10.06	8.23	9.87	9.15	9.98	9.41	9.51	0.96	1	5.41	9.43	2.82	5.89	3.33	3.54	6.06	4.31	0.73	1	1.44	2.8	2.81	2.35	3.9	4.98	4.44	1.89	0.986782
Solyc01g094200.2.1	NAD-dependent malic enzym	59.62	60.18	53.53	60.92	58.56	52.87	60.65	47.96	56.72	54.55	0.93	1	50.25	50.77	47.76	49.59	34.05	41.08	42.2	39.11	0.79	0.778329	33.02	38.22	33.07	34.77	26.13	26.08	34.82	29.01	0.83	1	12	24.28	23.59	19.96	38.32	41.03	39.67	1.99	0.335176
Solyc01g094210.2.1	Carbonyl reductase 3 (AHRD \	7.08	9.85	7.05	3.13	6.78	7.14	9.09	8.75	5.24	7.55	1.11	1	8.65	13.66	3.93	8.75	4.61	6.42	11.13	7.39	0.84	1	0.54	0.54	0.66	0.58	0.29	0.17	1.45	0.64	1.1	1	0	0.48	0.34	0.27	0	0	0	0.04	1
Solyc01g094220.2.1	Carbonyl reductase 3 (AHRD \	45.37	56.76	42.67	30.69	43.87	95.75	61.95	112.04	71.91	85.41	1.95	0.022254	61.57	68.02	49.8	59.8	75.85	103.72	99.73	93.1	1.56	0.043692	103.94	90.19	69.4	87.84	97.76	113.29	97.11	102.72	1.17	1	22.13	59.22	43.14	41.5	111.15	85.51	98.33	2.37	0.193383
Solyc01g094230.2.1	Protein phosphatase-2C (AHR	0.65	0.67	0.63	0.3	0.56	0.24	0.17	0.1	0.23	0.18	0.33	0.483961	0.1	0.19	0.09	0.13	0.15	0.22	0.17	0.18	1.42	1	0	0.09	0.11	0.07	0.14	0.24	0.08	0.15	2.3	1	0.15	0.08	0	0.08	0.1	0.28	0.19	2.48	1
Solyc01g094240.2.1	Short-chain dehydrogenase/r	3.31	4.6	4.15	7.61	4.92	3.91	5.22	8.75	3.12	5.25	1.07	1	2.31	1.62	1.57	1.83	1	1.28	1.14	1.14	0.62	0.962977	0.17	0.67	0	0.28	0.41	0.47	0.45	0.44	1.58	1	0.29	0.15	0.32	0.25	0.38	1.63	1	3.97	0.980583
Solyc01g094250.2.1	Short-chain dehydrogenase/r	22.68	32.84	25.05	31.2	27.94	32.54	28.32	58.76	23.24	35.72	1.28	0.838948	20.14	19.35	8.88	16.12	6.43	4.91	9.07	6.8	0.42	0.187984	2.83	2.02	1.02	1.96	2.3	1.25	1.2	1.58	0.81	1	5.57	13.93	11.41	10.3	5.85	10.3	8.07	0.78	1
Solyc01g094260.2.1	Short-chain dehydrogenase/r	11.75	17.25	14.84	19.83	15.92	13.06	12.21	23.5	15.39	16.04	1.01	1	9.59	11.11	4.88	8.53	3.86	2.35	5.18	3.8	0.45	0.218098	1.67	1.51	0.41	1.2	1.9	0.94	0.45	1.1	0.92	1	2.34	5.39	3.49	3.74	3.02	6.87	4.95	1.32	1
Solyc01g094270.2.1	Carbonyl reductase 1 (AHRD \	0	0	0	0.3	0.07	0	0	0.19	0	0.05	0.63	1	0	0	0.18	0.06	0	0	0	0	0.17	1	0	0	0	0	0	0	0	0	1	NA	0	0	0	0	0	0	0	1	NA
Solyc01g094280.2.1	FolD bifunctional protein (AH	18.95	16.23	15.4	14.92	16.38	15.3	22.87	17.1	17.97	18.31	1.12	0.915979	19.62	18.65	13.14	17.14	22.2	23.6	19.95	21.92	1.28	0.567141	15.02	22	15.26	17.43	12.33	18.7	18.24	16.42	0.94	1	15.25	19.48	20.62	18.45	30.44	33.55	31.99	1.73	0.622405
Solyc01g094290.2.1	Glutathione-regulated potass	15.08	16.26	19.67	16.42	16.86	14.37	15.42	15.13	18.96	15.97	0.95	1	15.92	15.45	18.12	16.5	17.1	16.44	14.7	16.08	0.97	1	14.22	16.23	15.89	15.45	18.18	15.51	15.48	16.39	1.06	1	7.82	11.31	11.42	10.18	14.91	13.31	14.11	1.39	1
Solyc01g094300.1.1	PRP31 pre-mRNA processing	0	0	0	0	0	0	0	0	0	0	1	NA	0	0	0	0	0	0	0	0	1	NA	0	0	0	0	0	0	0	0	1	NA	0	0	0	0	0	0	0	1	NA
Solyc01g094310.1.1	U4/U6 small nuclear ribonucl	0	0	0	0	0	0	0	0	0	0	1	NA	0	0	0	0	0	0	0	0	1	NA	0	0	0	0	0	0	0	0	1	NA	0	0	0	0	0	0	0	1	NA
Solyc01g094320.2.1	Cysteine and glycine-rich prot	0.29	0	0.35	0	0.16	0	0	0.54	0	0.14	0.84	1	0	0	0	0	0.2	0	0	0.07	6.67	1	0.24	0	0	0.08	0	0	0.43	0.14	1.79	1	0.42	0.86	0.91	0.73	0	0	0	0.01	0.989607
Solyc01g094330.2.1	Expressed protein with functi	5.6	6.83	7.19	6.26	6.47	7.98	6.05	6.6	6.28	6.73	1.04	1	7.68	7.26	4.98	6.64	5.11	5.8	8.11	6.34	0.95	1	10.96	9.29	14.25	11.5	10.01	11.1	12.61	11.24	0.98	1	7.69	10.35	11.94	9.99	8.1	6.98	7.54	0.75	1
Solyc01g094340.2.1	Acetyl-coenzyme A carboxyla	10	12.21	14.26	15.04	12.88	16.06	16.14	16.63	17.35	16.55	1.28	0.397918	12.43	18.17	28.32	19.64	26.16	25.78	26.64	26.19	1.33	0.61209	4.48	10.44	6.49	7.14	8.72	8.13	9.8	8.88	1.24	1	7.75	10.56	4.89	7.73	7.07	7.49	7.28	0.94	1
Solyc01g094350.2.1	ATP-dependent RNA Helicase	25.25	28	27.3	24.5	26.26	20.72	21.71	20.91	26.48	22.46	0.86	0.972223	30.62	25.51	30.6	28.91	23.11	23	27.01	24.37	0.84	0.962977	22.96	27.69	23.62	24.76	28.05	20.44	19.75	22.75	0.92	1	31.77	28.52	33.34	31.21	27.96	31.65	29.8	0.95	1
Solyc01g094360.2.1	MYB transcription factor (AH	15.96	14.39	11.55	4.82	11.68	11.35	11.42	11.94	15.89	12.65	1.08	1	22.96	19.98	14.38	19.11	9.89	10.91	8.03	9.61	0.5	0.045867	1.72	2.08	0.42	1.41	1.12	0.48	0.31	0.64	0.45	1	0.3	0	0	0.1	0	0	0	0.1	1
Solyc01g094370.2.1	Remorin 2 (AHRD V1 ***- Q9	182.08	181.85	115.64	109.31	147.22	175.58	211.01	276.59	243.58	226.69	1.54	0.071582	126.59	125.51	88.2	113.43	84.71	121.41	105.76	103.96	0.92	1	120.61	95.23	65.48	93.77	119.61	91.15	94.23	101.66	1.08	1	120.06	117.46	94.49	110.67	115.95	107.75	111.85	1.01	1
Solyc01g094380.2.1	Glycosyltransferase CAZy fam	0	0	0	0	0	0	0	0	0	0	1	NA	0	0	0	0	0.09	0.07	0	0.05	5.33	1	0	0	0	0	0	0	0	0	1	NA	0	0.19	0	0.06	0	0	0	0.16	1
Solyc01g094390.2.1	Glycosyltransferase CAZy fam	0	0	0	0	0	0	0	0	0	0	1	NA	0	0	0	0	0	0	0	0	1	NA	0	0	0	0	0	0	0	0	1	NA	0	0	0	0	0	0	0	1	NA
Solyc01g094400.2.1	Actin depolymerizing factor 1	0	0	0	0	0	0	0	0	0	0	1	NA	0	0	0	0	0	0	0	0	1	NA	0	0	0	0	0	0	0	0	1	NA	0	0	0	0	0	0	0	1	NA
Solyc01g094410.2.1	Phosphoribosylanthranilate t	28.81	26.97	43.54	57.93	39.31	16.99	19.03	16.4	20.96	18.34	0.47	0.178055	25.94	31.68	51.75	36.46	43.81	38.38	30.03	37.41	1.03	1	16.83	17.35	59.52	31.23	20.81	22.53	19.23	20.86	0.67	1	97.51	61.58	61.37	73.49	52.44	60.76	56.6	0.77	1
Solyc01g094420.2.1	Ulp1 protease family protein	23.55	26.13	23.18	23.53	24.1	23.2	21.68	23.81	23.56	23.06	0.96	1	18.69	18.92	18.86	18.82	21.76	17.4	16.49	18.55	0.99	1	17.5	13.64	13.96	15.03	19.43	17.17	15.05	17.22	1.15	1	39.68	28.86	30.9	33.15	24.63	30.63	27.63	0.83	1
Solyc01g094430.1.1	Ripening-related protein 3 (A	0	0	0	0	0	0	0	0	0	0	1	NA	0	0	0	0	0	0	0	0	1	NA	0	0	0	0	0	0	0	0	1	NA	0	0	0	0	0	0	0	1	NA
Solyc01g094440.1.1	Ripening-related protein 3 (A	0	0	0	0	0	0	0	0	0	0	1	NA	0	0	0	0	0	0	0	0	1	NA	0	0	0	0	0	0	0	0	1	NA	0	0	0	0	0	0	0	1	NA
Solyc01g094450.1.1	Ripening-related protein 3 (A	0	0	0	0	0	0	0	0	0	0	1	NA	0	0	0	0	0	0	0	0	1	NA	0	0	0	0	0	0	0	0	1	NA	0	0	0	0	0	0	0	1	NA
Solyc01g094460.2.1	AT-hook motif nuclear localiz	14.75	20.72	11.67	23.6	17.69	28.47	35.85	18.73	25.49	27.13	1.53	0.244438	18.77	19.4	16.88	18.35	20.05	24.01	24.25	22.77	1.24	0.699327	13.95	11.23	6.36	10.51	14.4	4.71	8.2	9.1	0.87	1	4.69	1.97	1.79	2.82	0.53	0.68	0.6	0.21	0.727743
Solyc01g094470.2.1	Unknown Protein (AHRD V1)	175.39	197.62	141.43	132.01	161.61	210.16	202.61	389.09	156.53	239.6	1.48	0.608415	160.9	159.45	100.57	140.31	77.56	146.21	128.98	117.58	0.84	1	136.5	118	90.24	114.91	148.51	90.25	116.08	118.28	1.03	1	50.33	83.56	51.96	61.95	68.49	52.2	60.34	0.97	1
Solyc01g094480.2.1	Pre-mRNA-splicing factor prp	92.61	81.93	114.25	120.17	102.24	80.47	70.85	71.33	94.43	79.27	0.78	0.738961	80.04	98.78	111.9	96.91	145.32	103.75	105.08	118.05	1.22	0.72473	67.2	75.91	79.18	74.1	79.59	93.27	80.12	84.33	1.14	1	122.34	78.47	77.5	92.77	105.53	117.96	111.75	1.2	1
Solyc01g094490.2.1	NAC transcription factor (AHF	16.02	14.26	17.42	17.33	16.26	14.67	14.38	18.2	22.1	17.34	1.07	0.991503	7.31	14.2	15.39	12.3	23.75	22.93	17.53	21.4	1.74	0.097726	9.14	11.26	12.16	10.85	18.37	16.91	16.48	17.25	1.59	1	27.25	33.54	28.02	29.6	25.31	27.82	26.56	0.9	1
Solyc01g094500.2.1	Riboflavin biosynthesis protei	4.65	5.47	3.36	5.3	4.7	8	8.52	7.44	4.74	7.18	1.53	0.365997	2.79	3.52	4.18	3.5	3.33	5.2	5.3	4.61	1.32	0.824103	0.97	2.32	1.79	1.69	1.18	0.91	3.28	1.79	1.06	1	0.64	0.54	0.23	0.47	0.14	0.26	0.2	0.43	1
Solyc01g094510.1.1	Unknown Protein (AHRD V1)	1.55	0.92	0.61	0	0.77	1.15	0.41	0.95	0.37	0.72	0.94	1	1.92	0	0.44	0.79	0.36	0.53	0.81	0.57	0.72	1	0.42	0	1.53	0.65	0.34	0.39	0.38	0.37	0.57	1	0	0	0	0	0	0	0	1	NA
Solyc01g094520.2.1	Kelch-like protein 14 (AHRD \	10.23	11.6	10.12	12.13	11.02	8.07	7.81	7.28	7.93	7.77	0.71	0.574871	10.59	10.75	11	10.78	9.49	9.82	8.3	9.2	0.85	1	10.79	15.05	11.46	12.43	10.73	9.25	9.49	9.82	0.79	1	12.43	7.43	5.83	8.56	11.63	10.85	11.24	1.31	1
Solyc01g094530.2.1	Acyl-CoA thioesterase (AHRD	19.38	20.53	20.86	14.79	18.89	20	17.48	20.08	17	18.64	0.99	1	18.6	18.71	15.98	17.76	19.09	14.25	14.62	15.99	0.9	1	12.3	17.17	11.6	13.69	8.89	9.91	12.66	10.49	0.77	1	11.59	12.06	10.88	11.51	14.08	11.46	12.77	1.11	1

Solyc01g094540.2.1	Small nuclear ribonucleoprote	19.72	15.88	45.7	41.84	30.79	14.09	20.02	13.17	18.71	16.5	0.54	0.61778	19.65	19.39	37.68	25.57	28.82	17.5	15.68	20.67	0.81	1	7.97	18.75	22.37	16.36	12.03	8.01	10.61	10.22	0.62	1	39.72	10.74	13.72	21.39	22.14	22.85	22.5	1.05	1
Solyc01g094550.2.1	Acyl-CoA thioesterase family	49.98	54.74	62.05	48.29	53.76	41.71	42.49	48.92	52.55	46.42	0.86	0.98182	50.92	50.93	41.95	47.93	30.5	30.97	33.72	31.73	0.66	0.223613	19.63	23.43	17.71	20.26	19.9	18.07	14.46	17.48	0.86	1	33.45	39.31	30.74	34.5	42.37	46.79	44.58	1.29	1
Solyc01g094560.2.1	60S ribosomal protein L36 (A	68.99	75.6	68.17	70.14	70.72	68.54	59.57	80.48	57.89	66.62	0.94	1	62.57	64.84	66.65	64.69	54.13	61.62	50.49	55.41	0.86	1	50.51	53.76	44.94	49.74	45.04	42.48	50.27	45.93	0.92	1	36.17	39.15	44.42	39.91	38.68	36.67	37.67	0.94	1
Solyc01g094570.2.1	Calmodulin-binding heat-sho	12.27	18.24	11.12	10.58	13.05	13.64	13.23	15.54	11.92	13.58	1.04	1	10.58	16.65	16.17	14.47	14.84	18.59	19.52	17.65	1.22	0.783084	5.08	9.51	5.05	6.55	6.88	4.88	7.63	6.46	0.99	1	7.66	12.6	13.8	11.35	7.94	10.36	9.15	0.81	1
Solyc01g094580.2.1	Beta-amylase 9 (AHRD V1 ***	16.59	13.16	14.09	17.97	15.45	10.43	9.95	12.38	14.36	11.78	0.76	0.735963	14.9	16.81	21.22	17.64	18.41	18.4	16.39	17.73	1.01	1	9.42	13.67	10.91	11.33	10.4	7.33	12.43	10.05	0.89	1	11.59	9.64	11.22	10.82	8.85	10.14	9.5	0.88	1
Solyc01g094590.2.1	Gibberellin 20-oxidase-like pr	3.63	5.39	3.21	2.57	3.7	3.7	1.19	4.73	1.52	2.79	0.75	0.863123	3.66	1.84	3.83	3.11	1.68	2.66	2.38	2.24	0.72	1	2.93	2.71	2.7	2.78	0.8	2.06	1.32	1.39	0.5	1	3.44	3.3	2.79	3.18	2.77	1.59	2.18	0.69	1
Solyc01g094600.2.1	Pentatricopeptide repeat-con	0.43	0.19	0.38	0.61	0.4	0.24	0.68	0.69	0.23	0.46	1.14	1	0.7	0.37	0.91	0.66	0.75	0.5	1.01	0.75	1.14	1	0.52	0.18	0.53	0.41	0.28	0.33	0.08	0.23	0.56	1	0.15	0.23	0.33	0.24	0.2	0.57	0.39	1.63	1
Solyc01g094610.2.1	Rop guanine nucleotide excha	1.66	1.84	0.98	0	1.12	0.15	0.44	0.25	0.5	0.33	0.3	0.377456	0.51	0.6	0.12	0.41	0.38	0.29	0.43	0.37	0.89	1	0	0.22	0.14	0.12	0.27	0	0.2	0.16	1.31	1	0.2	0	0.21	0.14	0	0	0	0.07	1
Solyc01g094620.2.1	Mediator of RNA polymerase	2.59	3.53	5.74	6.56	4.61	2.75	2.21	1.96	2.74	2.42	0.52	0.363789	3.93	3.65	6.82	4.8	4.27	2.88	3.23	3.46	0.72	0.825568	4.59	4.89	7.96	5.81	4.16	3.89	3.42	3.82	0.66	1	11.44	7.88	9.5	9.61	5.42	5.93	5.67	0.59	0.737112
Solyc01g094630.2.1	Unknown Protein (AHRD V1)	22.81	26.34	29.1	26.16	26.1	33.58	28.23	42.52	23.02	31.84	1.22	0.833895	32.12	19.29	29.68	27.03	14.11	17.24	25.68	19.01	0.7	0.70879	18.43	15.57	22.64	18.88	14.18	14.95	13.03	14.05	0.74	1	33.31	29.11	27.49	29.97	20.88	26.49	23.68	0.79	1
Solyc01g094640.2.1	(related) (AHRD V1 ***- Q2H	31.36	37.32	41.81	43.97	38.62	31.64	31.09	34.3	40.77	34.45	0.89	1	31.74	45.1	56.25	44.36	50.02	44.57	44.41	46.33	1.04	1	26.9	22.74	30.65	26.76	26.5	27.33	23.56	25.8	0.96	1	66.2	38.83	44.4	49.81	36.99	50.94	43.97	0.88	1
Solyc01g094650.2.1	Protein PAIR1 (AHRD V1 *-*-	0.3	0.4	0.53	0.42	0.41	0.33	0.24	0.82	0.86	0.56	1.36	1	0.97	0.52	0.38	0.62	0.41	0.54	0.82	0.59	0.95	1	0	0.24	0	0.08	0	0	0	0	0.12	1	0.21	0.11	0.23	0.18	0.27	0.13	0.2	1.09	1
Solyc01g094660.2.1	Receptor-like protein kinase (	10.91	12.66	17.31	17.72	14.65	9.67	11.46	12.67	12.38	11.55	0.79	0.860017	14.74	15.83	19.3	16.62	13.55	13.66	11.88	13.03	0.78	0.801969	10.72	12.25	14.56	12.51	11.19	9.72	10.21	10.37	0.83	1	16.87	18.58	17.91	17.79	15.45	17	16.23	0.91	1
Solyc01g094670.2.1	Aberrant root formation prot	5.5	4.59	5.69	6.19	5.49	3.99	5.4	4.33	5.96	4.92	0.9	1	7.31	6.05	8.91	7.42	5.37	6.45	5.91	5.91	0.8	0.956426	3.35	6.13	4.23	4.57	5.38	4.68	5.97	5.34	1.17	1	0.48	2.36	2.07	1.64	2.46	2.56	2.51	1.53	1
Solyc01g094680.2.1	Signal peptide peptidase (AH	33.72	38.93	35.6	41.47	37.43	24.72	24.11	28.87	32.01	27.43	0.73	0.519096	27.27	40.36	36.94	34.86	22.2	28.91	31.59	27.57	0.79	0.781406	16.48	15.83	19.4	17.24	24.8	17.13	16.83	19.59	1.14	1	22.75	27.95	29.12	26.61	25.05	28.17	26.61	1	1
Solyc01g094690.2.1	Aquaporin (AHRD V1 ***- Q4	46.83	36.95	38.92	49.27	42.99	33.85	26.59	22.27	22.19	26.22	0.61	0.100785	22.74	31.08	34.68	29.5	24.96	17.68	13.96	18.87	0.64	0.345781	16.59	26.31	19.23	20.71	9.24	13.64	12.94	11.94	0.58	1	4.26	4.69	4.61	4.52	4.86	5.06	4.96	1.1	1
Solyc01g094700.2.1	Glycerol-3-phosphate acyltra	100.67	104.21	86.82	72.31	91	74.65	57.89	70.44	91.89	73.72	0.81	0.702987	84.47	59.97	68.37	70.94	24.7	27.63	29.43	27.25	0.38	5.93E-06	2.12	1.39	0.26	1.26	1.29	0.6	0.96	0.95	0.76	1	0.19	0.29	0.61	0.36	0.12	0.58	0.35	0.96	1
Solyc01g094710.1.1	Unknown Protein (AHRD V1)	0	0	0	0	0	0.61	0	0	0	0.15	15.25	1	0	0	0	0	0.38	0	0	0.13	12.67	1	0	0	0	0	0	0	0	0	1	NA	0	0	0	0	0	0	0	1	NA
Solyc01g094720.2.1	Vesicular glutamate transpor	24.35	24.76	22.18	26.29	24.39	35.76	35.61	28.88	34.1	33.59	1.38	0.232286	22.65	20.31	25.52	22.83	22.99	24.93	26.05	24.66	1.08	1	20.55	19.84	21.25	20.55	17.64	19.85	21.15	19.55	0.95	1	32.91	9.79	12.87	18.52	27.66	22.01	24.84	1.34	1
Solyc01g094740.1.1	Ulp1 protease family C-termi	0.24	0.21	0.28	0.09	0.2	0.4	0.38	0.5	0.22	0.38	1.83	0.833008	0.22	0.31	0.25	0.26	0	0.03	0	0.01	0.04	0.119047	0.05	0.05	0.18	0.09	0.08	0	0	0.03	0.29	1	0.09	0.17	0.09	0.12	0	0.11	0.06	0.47	1
Solyc01g094750.2.1	Cytochrome P450	4.63	5.9	4.58	1.59	4.18	4.87	2.67	4.37	4.76	4.17	1	1	4.77	2.5	4.12	3.8	0.69	0.97	1.08	0.91	0.24	0.001504	0.1	0.1	0.25	0.15	0.16	0	0	0.05	0.36	1	0	0.09	0.19	0.09	0	0	0	0.11	1
Solyc01g094760.2.1	Origin recognition complex 5	4.64	4.35	5.62	6.05	5.17	3.8	4.44	4.83	4.21	4.32	0.84	1	4.55	3.5	4.64	4.23	2.37	2.59	3.46	2.81	0.66	0.657415	1.58	0.9	0.49	0.99	1.28	0.28	1.07	0.88	0.89	1	1.91	0.62	0.94	1.16	3.58	2.36	2.97	2.57	0.483107
Solyc01g094770.2.1	Unknown Protein (AHRD V1)	120.19	105.1	105.55	85.65	104.12	100.12	97.51	105.8	115.05	104.62	1	1	145.15	142.19	118.57	135.3	147.09	146.3	146.06	146.48	1.08	0.985957	152.84	160.54	106.45	139.94	137.54	126.72	152.56	138.94	0.99	1	194.45	142.65	115.34	150.81	128.5	145.8	137.15	0.91	1
Solyc01g094780.2.1	Unknown Protein (AHRD V1)	3.04	4.42	5.21	4.69	4.34	5.22	3.49	4.31	4.95	4.49	1.04	1	5.91	2.88	5.13	4.64	1.91	4	4.12	3.34	0.72	0.985963	4.24	5.4	2.47	4.04	4.17	2.72	4.02	3.64	0.9	1	0.78	1.8	0	0.86	0.76	1.45	1.1	1.28	1
Solyc01g094790.2.1	Cysteine synthase (AHRD V1 *	37.41	35.92	58.92	34.41	41.66	32.77	34.92	25.41	27.52	30.15	0.72	0.54609	54.17	61.35	54.32	56.61	62.37	58.96	58.51	59.95	1.06	1	38.56	40.69	86.7	55.32	39.1	46.37	93.46	59.64	1.08	1	59.74	155.12	96.63	103.83	87.55	53.8	70.67	0.68	1
Solyc01g094800.2.1	Chromodomain-helicase-DNA	10.88	11.36	16.42	15.52	13.55	7.61	7.43	9.52	10.97	8.88	0.66	0.254994	12.48	14.2	21.99	16.22	17.69	14.03	13.05	14.92	0.92	1	9.34	11.7	12.88	11.31	11.78	10.77	10.34	10.96	0.97	1	29.6	23.5	27.48	26.86	19.38	21.33	20.35	0.76	1
Solyc01g094810.2.1	Ubiquitin-conjugating enzyme	106.27	92.04	110.23	64.56	93.28	133.58	99.1	157.71	107.53	124.48	1.33	0.406444	116.1	118.41	122.89	119.13	186.49	183.71	172.63	180.94	1.52	0.066286	23.34	31.96	29.73	28.34	33.5	26.22	36.64	32.12	1.13	1	29.91	30.04	35.64	31.86	36.23	35.64	35.94	1.13	1
Solyc01g094820.2.1	ARID/BRIGHT DNA-binding do	2.37	3.91	3.22	2.52	3	2.9	2.62	4.36	3.15	3.26	1.08	1	4.64	4.44	3.81	4.3	2.8	3.38	4.57	3.58	0.83	1	2.78	3.29	2.36	2.81	2.81	1.8	3.11	2.57	0.92	1	6.41	5.34	7.66	6.47	2.17	3.33	2.75	0.43	0.49975
Solyc01g094830.2.1	Receptor-like protein kinase (	4.61	5.75	3.5	3.59	4.36	4.83	4.93	4.24	5.13	4.78	1.1	1	4.83	4	4.2	4.34	4	5.46	4.99	4.82	1.11	0.975922	1.71	2.16	1.16	1.68	1.29	1.12	1.81	1.41	0.84	1	1.28	2.12	1.47	1.62	1.02	1.72	1.37	0.84	1
Solyc01g094840.2.1	RING finger protein 13 (AHRD	15.04	16.43	12.1	11.6	13.79	15.58	10.52	29.86	13.74	17.43	1.26	0.934161	14.31	10.1	15.88	13.43	14.7	14.34	17.19	15.41	1.15	1	11.87	10.58	11.2	11.22	9.88	12.18	10.96	11.01	0.98	1	33.05	31.28	18.4	27.58	13.45	17.68	15.56	0.56	0.683081
Solyc01g094850.1.1	Pentatricopeptide repeat-con	1.09	0.88	1.42	0.62	1	0.61	0.86	0.6	0.94	0.75	0.75	1	0.61	0.67	0.28	0.52	0.46	0.4	0.86	0.57	1.1	1	0.27	0.53	0.11	0.3	0.5	0.5	0.96	0.65	2.15	1	0.16	0.08	0	0.08	0.3	0.67	0.48	6.06	0.943939
Solyc01g094860.2.1	Transmembrane protein 8B (	5.11	5.69	7.73	9.15	6.92	3.11	3.53	3.3	2.92	3.22	0.46	0.017308	4.83	10	8.77	7.87	4.82	3.14	5.02	4.33	0.55	0.272636	5.29	4.56	4.52	4.79	3.88	3.21	3.43	3.51	0.73	1	6.13	7.95	6.39	6.82	3.87	4.55	4.21	0.62	0.811923
Solyc01g094870.1.1	MRNA clone RAFL21-92-I07 (	2.38	1.59	2.81	3.37	2.54	4.64	4.71	2.56	2.28	3.55	1.4	0.723836	1.66	1.55	1.34	1.52	0.69	0.72	0.94	0.78	0.52	0.815644	1.44	2.27	2.56	2.09	0.65	0.45	1.16	0.75	0.36	1	0	0	0.31	0.1	0	0.35	0.17	1.69	1
Solyc01g094880.2.1	Protein RIC1 homolog (AHRD	13.24	16.89	8.99	16.16	13.82	11.66	13	13.36	13.29	12.83	0.93	1	14.17	15.38	14.45	14.67	7.76	10.86	14.55	11.06	0.75	0.607231	10.22	7.69	8.67	8.86	6.77	6.71	7.6	7.03	0.79	1	6.66	6.72	6.67	6.68	5.52	7.04	6.28	0.94	1
Solyc01g094890.2.1	Ferric reductase oxidase (AHR	0.55	1.07	1.74	0	0.84	0.62	0.51	0.68	0.2	0.5	0.6	0.880428	0.86	0.72	0.08	0.55	0.7	0.43	0.73	0.62	1.12	1	0.9	0	0.09	0.33	0	0.14	0.13	0.09	0.27	1	0	0	0	0	0	0	0	1	NA
Solyc01g094900.1.1	Ferric reductase (AHRD V1 **	0	0	0	0	0	0	0	0	0	0	1	NA	0.31	0.29	0	0.2	0.47	0	0.26	0.24	1.22	1	0.27	0	0	0.09	0	0	0.25	0.08	0.93	1	0	0	0	0	0	0	0	1	NA
Solyc01g094910.2.1	Ferric reductase oxidase (AHR	0	0	0	0	0	0	0	0	0	0	1	NA	0.6	1.19	0.7	0.83	1.91	0.85	0.58	1.11	1.34	1	0.15	0.15	0	0.1	0.36	0.07	0.13	0.19	1.87	1	0.26	0	0.14	0.13	0	0	0	0.07	1
Solyc01g094920.2.1	Receptor-like kinase (AHRD V	38.05	50.56	58.5	89.39	59.12	27.94	30.04	30.78	34.3	30.77	0.52	0.272461	70.02	114.82	128.92	104.59	71.76	69.39	62.1	67.75	0.65	0.378408	59.13	65.53	150.68	91.78	83.76	71.16	37.04	63.99	0.7	1	210.9	164.63	210.58	195.37	221.47	278.01	249.74	1.28	1
Solyc01g094930.2.1	CAAX prenyl protease 1 (AHR	38.34	45.31	37.91	37.55	39.78	36.06	33.33	37.11	36.22	35.68	0.9	1	44.47	44.26	32.51	40.41	26.38	31.83	37.94	32.05	0.79	0.813994	74.12	50.47	57.01	60.53	43.64	58.83	47.28	49.92	0.82	1	27.37	46.67	44.64	39.56	42.91	40.83	41.87	1.06	1
Solyc01g094940.2.1	Receptor-like protein kinase (	16.16	16.61	19.07	15.06	16.72	12.12	10.58	10.63	10.57	10.97	0.66	0.258331	15.65	15.65	25.86	19.05	23.06	29.39	16.94	23.13	1.21	0.878357	3.93	8.03	3.65	5.2	7.86	7.27	5.55	6.89	1.32	1	14.2	8.88	15.36	12.81	30.32	48.62	39.47	3.08	0.003411
Solyc01g094950.2.1	Protein BPS1, chloroplastic (A	313.31	382.95	276.71	272.43	311.35	317.41	276.58	394.72	287.71	319.11	1.02	1	426.81	466.82	371.42	421.68	395.01	458.93	533.15	462.36	1.1	0.962977	277.79	352.71	281.08	303.86	271.93	295.08	302.65	289.89	0.95	1	783.98	618.75	837.01	746.58	376.94	400.06	388.5	0.52	0.377594
Solyc01g094960.2.1	Mitogen-activated protein kin	17.11	15.38	11.65	14.72	14.71	17.65	18.8	21.73	17.35	18.88	1.28	0.578585	18.05	15.49	12.22	15.25	14.07	14.25	16.22	14.85	0.97	1	9.98	8.48	6.48	8.31	7.53	10.43	9.25	9.07	1.09	1	18.81	27.93	34.72	27.15	19.86	26.29	23.07	0.85	1
Solyc01g094970.2.1	Glycoside hydrolase family 28	0.32	0	0	0	0.08	0	0.13	0.3	0	0.11	1.34	1	0.6	0	0.14	0.25	0	0.42	0	0.14	0.57	1	0	0.26	0	0.09	0	0.12	0.35	0.16	1.81	1	0	0	0	0	0.15	0	0.07	7.5	1
Solyc01g094980.1.1	UDP-rhamnose soyasaponin I	0	0	0.53	0.64	0.29	0	0	0.83	0	0.21	0.71	1	0	0.39	0	0.13	0	0.23	0	0.08	0.59	1	0	0	0	0	0	0	0	0	1	1	0	0.33	0	0.11	0.41	0	0.2	1.86	1
Solyc01g094990.2.1	Glycosyltransferase-like prote	0	0.24	0	0	0.06	0	0	0.25	0.1	0.09	1.46	1	0	0.12	0	0.04	0.09	0.07	0.1	0.09	2.17	1	0	0	0	0	0	0	0	0	1	NA	0	0	0	0	0	0	0	1	NA
Solyc01g095000.2.1	Homology to unknown gene (	16.9	16.95	12.34	6.07	13.06	35.18	22.87	38.71	24.07	30.21	2.31	0.01197	16.71	24.01	14.72	18.48	24.37	26.94	29.29	26.87	1.45	0.302736	16.9	14.86	9.85	13.87	25.9	23.17	21.89	23.65	1.71	1	6.86	14.41	6.6	9.29	15.71	17.16	16.44	1.77	0.963653
Solyc01g095010.2.1	Diacylglycerol kinase catalytic	7.53	9.49	8.24	7.8	8.26	12.46	16.54	14.02	12.27	13.82	1.67	0.104637	8.72	10.22	8.69	9.21	12.87	13.99	14.59	13.82	1.5	0.296588	22.27	28.15	14.23	21.55	22.21	16.3	21.16	19.89	0.92	1	4.18	9.88	5.37	6.48	8.75	12.08	10.41	1.61	1
Solyc01g095020.2.1	cDNA clone J023132J12 full i	12.61	15	23.18	20.98	17.94	11.98	12.01	10.55	14.15	12.17	0.68	0.470551	14.68	18.4	27.57	20.22	28.53	19.92	21.22	23.22	1.15	1	23.55	24.27	24.28	24.03	22.06	17.29	22.03	20.46	0.85	1	23.34	16.29	19.59	19.74	20.71	28.89	24.8	1.26	1
Solyc01g095030.2.1	MYB transcription factor (Fra	41.73	45.98	44.07	17.99	37.44	57.65	28.08	61.2	49.5	49.11	1.31	0.809307	64.14	51.47	42.8	52.8	40.93	44.14	38.26	41.11	0.78	0.814166	30.82	25.26	24.06	26.71	42.29	43.98	24.91	37.06	1.39	1	86.45	61.05	75.17	74.22	62.57	72.83	67.7	0.91	1
Solyc01g095040.2.1	Glyoxylate/hydroxypyruvate r	37.06	39.85	65.76	58.03	50.18	28.43	30.08	32.63	31.92	30.77	0.61	0.217916	39.03	54.26	59.07	50.79	51.84	37.24	36.83	41.97	0.83	0.911565	19.23	29.94	26.01	25.06	31.2	17.72	22.06	23.66	0.94	1	77.77	45.79	49.44	57.67	43.1	59.12	51.11	0.89	1
Solyc01g095050.2.1	Negatively light-regulated pro	3201.14	3052.58	2801.22	2720.99	2943.98	2700.26	2537.94	2977.82	2649.19	2716.3	0.92	1	3377.37	3545.88	2694.39	3205.88	3048.98	3110.57	2893.13	3017.56	0.94	1	2317.74	2439.69	1712.94	2156.79	2511.61	2358.95	2333.16	2401.24	1.11	1	1733.06	1894.21	1852.59	1826.62	1646.91	1634.73	1640.82	0.9	1
Solyc01g095060.2.1	Unknown Protein (AHRD V1)	9.77	13.05	14.41	19.01	14.06	4.07	8.68	8.99	8.77	7.63	0.54	0.717678	15.91	11.68	12.38	13.32	12.71	13.92	8.64	11.76	0.88	1	10.86	14.93	10.91	12.23	8.83	7.41	17.81	11.35	0.93	1	29.53	13.31	28.18	23.67	8.95	6.43	7.69	0.32	0.703437
Solyc01g095070.2.1	Heavy metal-associated doma	283.14	278.68	253.61	121	234.11	301.05	185.65	293.7	266.27	261.67	1.12	1	239	96.86	96.24	144.03	100.49	132.71	145.86	126.35	0.88	1	98.95	101.86	62.35	87.72	118.4	111.01	112.94	114.12	1.3	1	61.04	95.6	84.94	80.53	54.03	40.68	47.36	0.59	0.544234
Solyc01g095080.2.1	1-aminocyclopropane-1-carbo	0.41	0.72	0.16	2.5	0.95	1.66	0.64	0.37	0.1	0.69	0.73	1	0.38	0.47	0.34	0.4	1.32	0.35	0.85	0.84	2.12	0.819942	583.87	246.89	715.32	515.36	462.63	480.67	573.13	505.48	0.95	1	55.2	70.69	59.28	61.72	71.95	29.04	50.5	0.82	1
Solyc01g095090.2.1	E3 ubiquitin-protein ligase RN	0.58	0.62	1.24	0.82	0.81	1.3	1.1	0.86	1	1.06	1.31	0.955345	0.76	0.71	0.59	0.69	1.21	1.27	0.73	1.07	1.56	0.857418	13.65	6.84	7.87	9.45	6.81	5.57	10.36	7.58	0.8	1	0.17	0.59	0	0.25	0.32	0.1	0.21	0.83	1
Solyc01g095100.2.1	WRKY transcription factor 23	0.19	0.33	0	0.52	0.26	0.41	0	0	0	0.1	0.39	0.97992	0.17	0.32	1.41	0.63	0.9	0.48	0.73	0.7	1.11	1	0.15	0.76	0.37	0.43	1.83	0.56	0.81	1.07	2.5	1	1.05	0.67	0.28	0.67	0.17	0.49	0.33	0.5	1
Solyc01g095110.2.1	Pentatricopeptide repeat-con	4.24	3.54	3.98	4.29	4.01	3.85	4.37	2.39	2.92	3.38	0.84	1	3.62	2.63	3.07	3.11	3.84	3.68	4.49	4	1.29	0.808014	1.61	2.47	2.58	2.22	2.33	1.51	2.52	2.12	0.95	1	3.94	3.4	4.53	3.96	3.33	4.17	3.75	0.95	1
Solyc01g095120.2.1	Alpha/beta hydrolase fold (AH	17.29	16.87	24.18	21.02	19.84	13.53	17.6	12.48	17.01	15.16	0.76	0.778353	16.92	18.93	26.94	20.93	24.43	19.96	18.62	21	1	1	8.8	13.88	13.86	12.18	13.89	11.75	11.3	12.31	1.01	1	22.04	16.37	13.39	17.27	18.53	21.45	19.99	1.16	1
Solyc01g095130.2.1	Transcription factor (AHRD V1	0.42	0.38	0.25	0.6	0.41	0.47	0.67	1.17	1.06	0.84	2.04	0.797239	1.57	0.55	1.25	1.12	0.29	1.2	0.66	0.72	0.64	1	0.34	0.34	0.21	0.3	0	0	0	0	0.03	1	0	0	0	0	0	0	0	1	NA
Solyc01g095140.2.1	Late embryogenesis abundan	86.46	140.4	55.59	106.65	97.28	12.75	51.76	22.98	109.43	49.23	0.51	0.614698	132.64	243.28	132.21	169.38	25.01	18.03	20.61	21.22	0.13	0.00013	27.15	71.12	43.11	47.13	55.99	27.64	9.85	31.16	0.66	1	75.15	89.58	107.75	90.83	117.03	129.34	123.19	1.36	0.999777
Solyc01g095150.2.1	Late embryogenesis abundan	601.66	681.75	552.38	1006.74	710.63	310.09	533.62	477.97	526.67	462.09	0.65	0.50126	628.38	846.45	734.88	736.57	236.36	227.78	269.59	244.58	0.33	9.71E-10	450.14	464.47	465.86	460.16	470.53	358.58	219.22	349.44	0.76	1	800.76	766.37	771.57	779.57	713.99	831.61	772.8	0.99	1
Solyc01g095160.2.1	Pentatricopeptide repeat-con	1.52	1.18	1.57	2.15	1.6	1.37	1.8	1.57	1.36	1.52	0.95	1	1.85	1.48	2.56	1.96	2.1	2.31	1.79	2.07	1.05	1	1.07	2.16	0.85	1.36	1.49	1.65	1.93	1.69	1.24	1	0.81	1.72	0.87	1.13	1.82	2.74	2.28	2.01	0.987148
Solyc01g095170.2.1	Harpin-induced protein (AHRD	16.27	21.99	12.69	5.76	14.18	13.89	10.1	16.83	13.56	13.6	0.96	1	29.2	35.12	19.64	27.99	24.19	20.16	17.36	20.57	0.73	0.789355	33.81	52.1	18.46	34.79	23.67	18.28	13.34	18.43	0.53	1	5.37	4.85	20.11	10.11	4.52	3.31	3.92	0.39	1
Solyc01g095180.2.1	RING finger protein 38 (AHRD	5.84	6.55	8.08	5.74	6.55	6.77	4.81	6.77	7.65	6.5	0.99	1	12.98	7.06	17.36	12.47	7.92	7.23	6.18	7.11	0.57	0.567763	5.53	13.65	10.83	10	8	4.62	4.07	5.56	0.56	1	1.8	2.76	3.51	2.69	3.25	3.56	3.41	1.27	1
Solyc01g095190.1.1	Os10g0578600 protein (Fragm	0	0	0	0	0	0.17	0.12	0	0	0.07	7.25	1	0	0	0.13	0.04	0	0.08	0	0.03	0.62	1	0	0.12	0	0.04	0.2	0	0	0.07	1.67	1	0	0	0	0	0	0.52	0.26	26	1
Solyc01g095200.2.1	Reticulon family protein (AHR	296.12	310.25	244.7	192.64	260.93	259.16	213.85	282.83	212.59	242.11	0.93	1	344.95	327.57	277.76	316.76	221.33	235.35	230.55	229.08	0.72	0.433962	357.54	374.47	297.65	343.22	248.05	237.7	256.39	247.38	0.72	1	353.52	364.26	319.71	345.83	309.27	313.53	311.4	0.9	1
Solyc01g095210.2.1	Golgin candidate 4 (AHRD V1	20.63	21.07	20.43	23.88	21.5	18.38	28.1	23.93	21.62	23.01	1.07	0.932009	23.32	21.33	26.54	23.73	16.06	20.59	21.75	19.47	0.82	0.90752	23.98	25.81	18.23	22.67	25.1	20.25	22.39	22.58	1	1	26.11	23.33	21.01	23.48	19.28	19.54	19.41	0.83	1
Solyc01g095220.2.1	Unknown Protein (AHRD V1)	92.96	133.44	86.68	67.87	95.24	118.33	73.39	144.75	58.13	98.65	1.04	1	104.6	87.32	79.78	90.57	47.84	44.25	57.74	49.94	0.55	0.147859	26.65	30.54	25.3	27.5	18.71	14.77	24.04	19.17	0.7	1	6.4	34.85	34.58	25.28	13.73	18.4	16.06	0.64	1
Solyc01g095230.2.1	Programmed cell death 7 (AH	19.86	17.24	18.7	21.06	19.21	15.66	15.42	21.2	17.59	17.47	0.91	1	18.02	18.93	18.53	18.49	16.99	19.57	14.72	17.09	0.92	1	7.76	14.74	4.93	9.14	11.99	10.92	12.37	11.76	1.29	1	13.2	13.03	16.99	14.41	13.05	15.72	14.39	1	1
Solyc01g095240.2.1	Unknown Protein (AHRD V1)	0	0.65	0.86	0.34	0.46	0.54	0.19	0.67	0	0.35	0.76	1	0.67	0.84	0.82	0.78	0.34	0.25	0.38	0.32	0.42	0.920648	0.2	0.2	0.48	0.29	0.95	0	0.7	0.55	1.87	1	0.34	0.18	0.37	0.3	0	0	0	0.03	1
Solyc01g095250.1.1	Acidic chitinase (AHRD V1 ***	0	0	0	0	0	0	0	0	0	0	1	NA	0	0	0	0	0	0	0	0	1	NA	0	0	0	0	0	0	0	0	1	NA	0	0	0	0	0	0	0	1	NA
Solyc01g095260.1.1	Acidic chitinase (AHRD V1 **-	0	0	0	0	0	0	0	0	0	0	1	NA	0	0	0	0	0	0	0	0	1	NA	0	0	0	0	0	0	0	0	1	NA	0	0	0	0	0	0	0	1	NA
Solyc01g095270.2.1	SOUL heme-binding protein-li	19.81	22.44	21.25	26.43	22.48	24.03	18.84	24.22	26.83	23.48	1.04	1	20.11	26.42	18.26	21.6	33.89	27.63	25.48	29	1.34	0.459454	18.19	21.28	19.67	19.71	20.71	22.53	23.95	22.4	1.14	1	42.9	39.41	32.55	38.29	32.57	28.23	30.4	0.79	1
Solyc01g095280.2.1	Protein ABIL1 (AHRD V1 **--	40.1	33.58	34.85	37.2	36.43	33.31	36.76	39.45	31.57	35.27	0.97	1	24.47	24.36	27.5	25.44	20.64	21.54	23.21	21.8	0.86	1	11.84	16.87	11.13	13.28	10.91	10.29	14.77	11.99	0.9	1	20.84	16.13	13.26	16.74	15.39	15.69	15.54	0.93	1
Solyc01g095290.2.1	1 3-alpha-D-xylosyltransferas	8.18	12.29	8.79	12.08	10.33	12.73	11.48	11.7	12.26	12.04	1.17	0.866794	12.34	12.16	7.21	10.57	9.83	10.54	9	9.79	0.93	1	11.75	12.74	12.45	12.31	7.88	7.58	8.48	7.98	0.65	1	4.39	5.94	3.63	4.65	4.16	7.65	5.91	1.27	1
Solyc01g095300.2.1	Glycosyl transferase group 1 (	9.65	12.09	9.96	11.32	10.76	8.75	10.98	9.87	8.71	9.58	0.89	1	12.05	7.01	9.81	9.62	5.46	6.67	7.19	6.44	0.67	0.490742	1.69	1.71	0.63	1.34	1.38	0.43	0.97	0.93	0.69	1	0.09	0.28	0.78	0.38	0	0.17	0.09	0.22	1
Solyc01g095310.1.1	Acidic chitinase (AHRD V1 **-	0	0	0	0.31	0.08	0.24	0.17	0.2	0	0.15	1.97	1	0	0.19	0.37	0.19	0	0.11	0	0.04	0.2	1	0	0	0.22	0.07	0.14	0	0	0.05	0.64	1	0	0	0	0	0	0	0	1	NA
Solyc01g095320.2.1	BCL-2-associated athanogene	402.47	434.53	226.54	161.79	306.33	367.72	331.28	522.72	1307.94	632.41	2.06	0.63005	555.12	3302.92	1520.1	1792.71	1123.54	1196.88	920.04	1080.15	0.6	0.972863	3167.89	3089.76	4099.81	3452.49	5183.96	4400.59	3214.19	4266.25	1.24	1	3100.14	5245.3	3296.86	3880.77	5596.58	3026.66	4311.62	1.11	1
Solyc01g095330.2.1	Unknown Protein (AHRD V1)	2.72	4.84	3.21	8.66	4.86	4.54	1.61	8.13	1.95	4.06	0.84	1	3.16	25.42	42.51	23.7	2.83	3.17	3.21	3.07	0.13	0.329601	21.98	5.54	122.86	50.13	6.7	10.31	7.43	8.15	0.16	1	11.6	20.25	13.6	15.15	16.81	14.31	15.56	1.03	1
Solyc01g095340.2.1	Unknown Protein (AHRD V1)	1609.93	1654.29	1022.74	674.49	1240.36	1712.37	1320.34	2149.57	4690.53	2468.2	1.99	0.537842	2242.51	13282.43	6614.16	7379.7	4049.02	4134.26	3127.06	3770.11	0.51	0.872088	11885.57	12487.79	18302.85	14225.4	18149.7	15813.63	11297.39	15086.91	1.06	1	14316.47	21064.99	12960.26	16113.91	21721.51	11552.28	16636.9	1.03	1
Solyc01g095350.1.1	Unknown Protein (AHRD V1);	0	0.29	0	0	0.07	0.72	0.26	1.19	0	0.54	7.48	0.828944	0.3	0.28	0	0.19	0	0	0.76	0.25	1.31	1	0.26	0	0	0.09	0	0	0	0	0.12	1	0	0	0	0	0	0	0	1	NA
Solyc01g095360.2.1	C2 domain-containing protein	1.46	2.17	2.58	3.1	2.33	2.44	2.69	1.12	2.1	2.09	0.9	1	0.91	3.17	1.23	1.77	1.86	1.13	1.34	1.44	0.82	1	0.2	1.59	0	0.6	0.16	0.18	0.18	0.17	0.29	1	0	0	0	0	0	0.64	0.32	32	1
Solyc01g095370.2.1	F-box family protein (AHRD V	8.31	10.58	11.92	8.76	9.89	7.24	7.72	7.79	6.78	7.38	0.75	0.711682	11.67	7.79	9.91	9.79	7.68	8.6	9.98	8.75	0.89	1	14.74	11.95	9.38	12.02	7.28	7.78	9.02	8.03	0.67	1	5.79	4.26	3.01	4.35	4.47	6.57	5.52	1.27	1
Solyc01g095380.1.1	Unknown Protein (AHRD V1)	0	0	0	0	0	0	0	0	0	0	1	NA	0	0	0	0	0	0	0	0	1	NA	0	0	0	0	0	0	0	0	1	NA	0	0	0	0	0	0	0	1	NA
Solyc01g095390.2.1	SKIP interacting protein 7 (Fra	11.09	9.05	10.22	10.95	10.33	10.15	15.69	10.73	8.29	11.21	1.09	0.961776	8.28	13.26	10.15	10.56	9.3	9.57	9.9	9.59	0.91	1	3.83	4.52	2.75	3.7	3.64	3.5	2.78	3.31	0.89	1	5.91	8.22	11.55	8.56	8.57	6.79	7.68	0.9	1
Solyc01g095400.2.1	Kelch-like protein 6 (AHRD V1	44.35	47.14	66.22	57.4	53.78	38.08	35.01	41	36.37	37.62	0.7	0.3563	53.57	56.5	54.28	54.78	54.01	40.51	45.93	46.82	0.85	0.962977	37.89	40.89	38.95	39.24	29.21	31.22	33.11	31.18	0.79	1	35.24	35.91	32.13	34.43	28.92	31.85	30.39	0.88	1
Solyc01g095410.2.1	Eukaryotic translation initiati	423.12	423.15	331.19	310.19	371.91	326.71	355.96	489.43	419.4	397.88	1.07	1	421.51	371.77	318.18	370.49	334.49	427.56	410.83	390.96	1.06	1	818.22	552.03	735.66	701.97	716.13	770.71	774.82	753.89	1.07	1	188.89	430.91	491.14	370.31	682.62	445.19	563.9	1.52	1
Solyc01g095420.2.1	Unknown Protein (AHRD V1)	45.42	44.28	36.05	27.15	38.23	38.17	34.37	43.25	43.29	39.77	1.04	1	36.47	35.96	35.17	35.87	32.63	45.08	41.01	39.57	1.1	0.963879	47.18	49.57	41.06	45.94	56.3	42.01	50.64	49.65	1.08	1	61.89	67.75	62.78	64.14	52.72	59.5	56.11	0.87	1
Solyc01g095430.2.1	Threonine endopeptidase (AH	386.95	350.22	225.76	174.08	284.25	791.21	718.5	629.97	647.01	696.67	2.45	8.93E-07	264.84	272.99	217.02	251.62	518.3	366.74	451.44	445.49	1.77	0.003908	98.49	110.34	70.08	92.97	110.25	118.13	141.69	123.36	1.33	1	57.59	107.88	72.38	79.28	89.35	78.48	83.91	1.06	1
Solyc01g095440.1.1	Unknown Protein (AHRD V1);	0	0	0	0	0	0	0	0	0	0	1	NA	0	0	0	0	0	0	0	0	1	NA	0	0	0	0	0	0	0	0	1	NA	0	0	0	0	0	0	0	1	NA
Solyc01g095450.2.1	GDSL esterase/lipase At4g011	0	0.31	0	0	0.08	0.19	0.55	0	0.13	0.22	2.81	1	0.16	0	0	0.05	0.12	0.09	0.14	0.12	2.19	1	0	0	0	0	0	0	0	0	1	NA	0	0	0	0	0	0	0	1	NA
Solyc01g095460.2.1	G-box binding factor 3 (AHRD	52.27	43.3	56.84	31.4	45.95	33.95	21.26	44.44	38.43	34.52	0.75	0.614698	39.69	34.9	40.83	38.47	20.97	21.44	19.8	20.74	0.54	0.019149	11.19	19.63	28.13	19.65	22.65	19.45	12.05	18.05	0.92	1	46.34	83.14	71.18	66.89	63.42	69.99	66.7	1	1
Solyc01g095470.2.1	Aldose 1-epimerase family pr	40.35	43.32	33	38.96	38.91	51.02	66.24	65.96	58.24	60.36	1.55	0.063475	55.61	38.97	36.97	43.85	33.91	37.58	49.66	40.38	0.92	1	180.77	83.68	162.96	142.47	55.68	97.41	107.04	86.71	0.61	1	10.62	89.16	43.99	47.92	35.57	20.22	27.89	0.58	1
Solyc01g095480.2.1	Ribonuclease PH (AHRD V1 **	53.26	41.15	57.1	64.63	54.03	40.87	53.58	23.62	55.13	43.3	0.8	0.983202	49.8	54.76	54.12	52.89	70.7	55.3	51.84	59.28	1.12	0.951718	13.76	31.95	18.52	21.41	23.27	22.75	30.14	25.39	1.19	1	53.43	30.83	42.82	42.36	58.66	77.28	67.97	1.6	0.560665
Solyc01g095490.2.1	Ubiquitin-conjugating enzyme	57.12	63.05	48.58	40.08	52.21	93.51	86.69	72.87	61.35	78.61	1.51	0.198611	73.72	53.5	40.76	55.99	51.28	45.85	57.76	51.63	0.92	1	135.93	128.8	110.31	125.01	91.33	119.8	106.47	105.87	0.85	1	170.6	149.21	137.53	152.45	90.79	85.02	87.91	0.58	0.58776
Solyc01g095500.2.1	Ethylene-responsive transcrip	4.04	5.79	6.35	7.3	5.87	3.49	5.67	5.57	3.71	4.61	0.79	0.991557	11.9	3.71	4.17	6.59	1.4	1.86	1.23	1.5	0.23	0.434894	0.18	0.73	0.22	0.38	0.29	0	0	0.1	0.26	1	0.32	0	0.35	0.22	0.21	0.2	0.21	0.92	1
Solyc01g095510.2.1	Mitochondrial carrier family (	8.1	9.95	10.71	5.04	8.45	19.28	9.21	14.75	13.85	14.27	1.69	0.240697	12.36	2.54	3.83	6.24	3.26	2.69	2.68	2.88	0.46	0.914035	4.85	3.04	4.5	4.13	1.49	2.95	1.77	2.07	0.5	1	0.46	0.94	0.5	0.63	1.63	1.71	1.67	2.64	1
Solyc01g095520.2.1	Arabinogalactan peptide 16 (	0.97	0.86	0	0	0.46	1.08	0	0	1.39	0.62	1.35	1	0.9	0.84	0.82	0.85	1.35	1.51	0	0.95	1.12	1	0	0	0	0	0.64	0	2.12	0.92	92	1	0	0	0	0	0	0	0	1	NA
Solyc01g095530.2.1	Unknown Protein (AHRD V1)	383.26	339.7	194.58	255.51	293.26	524.67	414.18	380.55	956.83	569.06	1.94	0.306278	680.86	599.42	519.4	599.89	1983.31	2426	3131.05	2513.45	4.19	5.71E-09	726.51	872.28	401.47	666.75	1525.1	894.07	1312.88	1244.02	1.87	0.6616	13.4	23.96	31.4	22.92	10.78	30.3	20.54	0.9	1
Solyc01g095540.2.1	UDP-galactose transporter 6 (	9.37	16.85	11.29	9.3	11.7	13.51	14.95	15.03	15.6	14.77	1.26	0.719575	16.31	14.38	13.47	14.72	12.58	13	11.28	12.29	0.83	1	22.22	32.48	24.53	26.41	21.08	25.83	24.98	23.96	0.91	1	9.92	9.99	12.57	10.83	15.87	17.48	16.68	1.54	0.880638
Solyc01g095550.2.1	Receptor-interacting serine/t	0.84	0.75	0.44	1.33	0.84	1.25	2.15	1.29	1.75	1.61	1.92	0.265561	0.09	0.08	0.08	0.08	0	0	0	0	0.12	0.962977	0.15	0.08	0.28	0.17	0.06	0.14	0.27	0.16	0.92	1	0	0	0	0	0	0	0	1	NA
Solyc01g095560.2.1	Unknown Protein (AHRD V1)	1.78	0.53	2.1	0.84	1.31	1.98	2.35	3.28	2.13	2.44	1.86	0.872143	2.76	2.07	1.51	2.11	4.95	1.54	2.34	2.94	1.39	1	3.84	8.72	3.54	5.37	4.29	4.5	5.2	4.66	0.87	1	1.69	2.16	1.83	1.89	1.09	0	0.55	0.29	1
Solyc01g095570.2.1	Mitochondrial carrier-like pro	27.06	33.32	39.57	31.45	32.85	30.07	33.06	25.73	30.2	29.77	0.91	1	31.37	21.26	24.57	25.73	20.69	19.29	20.97	20.32	0.79	0.84953	12.88	14.04	9.93	12.28	13.27	9.41	9.72	10.8	0.88	1	25.56	38.76	33.63	32.65	15.44	25.51	20.48	0.63	0.732071
Solyc01g095580.2.1	GH3 family protein (AHRD V1	20.41	30.57	36.8	22.97	27.69	26.36	15.55	21.8	18.88	20.65	0.75	0.551227	18.18	30.99	31.2	26.79	44.76	46.06	46.08	45.63	1.7	0.017835	40.18	50.71	46.08	45.66	56.93	59.59	62	59.51	1.3	1	97.68	76.94	54.37	76.33	60.52	44.61	52.56	0.69	0.989607
Solyc01g095590.2.1	Extra-large G-protein-like (AH	15.4	16.07	23.08	23.81	19.59	11.21	13.4	13.47	11.74	12.46	0.64	0.203886	11.85	13.74	17.33	14.31	11.53	13.87	13.71	13.04	0.91	1	4.46	5.28	4.19	4.64	6.49	3.41	4.62	4.84	1.04	1	3.29	3.56	4.51	3.79	4.06	4.18	4.12	1.09	1
Solyc01g095600.2.1	Pentatricopeptide repeat-con	0.24	0.77	0.65	0.44	0.53	0.17	0.62	0.94	0.51	0.56	1.07	1	0.44	0.55	0.66	0.55	0.6	0.53	0.74	0.62	1.13	1	0.64	0.58	0.55	0.59	0.26	0.48	0.4	0.38	0.64	1	0.56	0.69	0.48	0.58	0.5	0.55	0.53	0.91	1

Solyc01g095610.2.1	Leaf senescence protein-like (	3.64	3.71	5.88	1.71	3.74	2.84	2.34	2.6	2.8	2.64	0.71	0.741315	1.75	3.16	3.64	2.85	3.17	2.16	3.81	3.05	1.07	1	0.54	1.21	0.27	0.67	0.27	0.1	0.49	0.29	0.43	1	0	0	0	0	0	0	0	1	NA
Solyc01g095620.2.1	UDP-glucosyltransferase (AH	70.67	80.99	121.89	71.21	86.19	45.66	35.49	81.13	53.92	54.05	0.63	0.360279	67.03	41.12	37.26	48.47	48.63	28.79	40.54	39.32	0.81	1	4.47	52.97	3.02	20.15	23.9	6.08	11.39	13.79	0.68	1	1.18	0.3	0	0.49	0	0	0	0.02	1
Solyc01g095630.2.1	WRKY transcription factor (A	6.26	5.23	5.08	6.37	5.74	1.96	6.03	3.78	2.95	3.68	0.64	0.637708	1.82	3.74	5.46	3.67	3.12	2.33	2.15	2.53	0.69	0.914307	2.37	3.99	22.54	9.63	5.92	6.68	2.43	5.01	0.52	1	88.79	119.86	147.2	118.62	35.66	22.14	28.9	0.24	0.000155
Solyc01g095640.1.1	Myb family transcription fact	0.69	0	0	0	0.17	0.77	0	0.64	0	0.35	2.04	1	0	0	0	0	0	0	0	0	1	NA	0	0	0	0	0	0	0	0	1	NA	0	0	0	0	0	0	0	1	NA
Solyc01g095650.2.1	Histone H3 (AHRD V1 ***- B7	2.27	1.62	1.61	0	1.38	0.51	0	0.84	0.33	0.42	0.31	0.628721	1.27	0	1.15	0.81	0.32	0.47	0.36	0.38	0.48	1	0	0	0	0	0	0	0	0	1	1	0	0	0.7	0.23	0	0	0	0.04	1
Solyc01g095660.1.1	Unknown Protein (AHRD V1)	0.5	0.44	0	0.71	0.41	0	0.39	0.46	0	0.21	0.52	1	0	0.43	0.84	0.42	0.69	0.52	0	0.4	0.95	1	0	0.41	0	0.14	0	0	0	0	0.07	1	0	0.36	0	0.12	0	0	0	0.08	1
Solyc01g095670.2.1	Rhodanese-related sulfurtran	40.65	38.17	38.48	32.65	37.49	49	54.67	40.8	52.17	49.16	1.31	0.362324	38.51	39.74	42.01	40.09	53.3	44.57	40.84	46.24	1.15	0.844156	17.81	19.05	17.74	18.2	24.16	23.59	19.97	22.57	1.24	1	8.26	15.9	11.22	11.79	30.95	26.24	28.59	2.42	0.177443
Solyc01g095680.2.1	Unknown Protein (AHRD V1)	36.97	30.07	36.1	30.2	33.33	43.93	41.26	47.18	44.39	44.19	1.33	0.423563	35.57	27.43	27.99	30.33	41.78	44.39	57.1	47.76	1.57	0.107924	54.33	61.03	50.93	55.43	52.76	53.14	68.74	58.21	1.05	1	31.72	99.53	75.96	69.07	65.93	48.34	57.14	0.83	1
Solyc01g095690.2.1	Pentatricopeptide repeat-con	0.28	0.25	0.49	0.49	0.38	0.15	0.16	0.44	0.3	0.26	0.7	1	0.06	0.3	0.29	0.22	0.96	0.36	0.87	0.73	3.37	0.227359	0	0.11	0.27	0.13	0	0	0.1	0.03	0.26	1	0	0	0	0	0	0	0	1	NA
Solyc01g095700.2.1	Abscisic acid receptor PYL8 (A	133.35	158.25	124.03	86.83	125.61	93.72	43.17	94.39	77	77.07	0.61	0.096321	108.95	92.61	55.72	85.76	63.37	53.34	94.26	70.32	0.82	1	13.4	9.49	5.6	9.5	8.11	7.22	5.66	7	0.74	1	16.05	12.3	10.31	12.89	4.2	3.4	3.8	0.29	0.197889
Solyc01g095710.2.1	ATP-dependent RNA helicase	1.47	1.14	1.86	1.6	1.52	1.53	1.36	1.58	1.38	1.46	0.96	1	1.55	1.75	2.28	1.86	3.51	1.96	2.05	2.51	1.35	0.832635	5.88	4.21	4.78	4.96	4.36	3.73	4.3	4.13	0.83	1	6.43	6.57	5.22	6.07	4.91	5.13	5.02	0.83	1
Solyc01g095720.2.1	Lipase (AHRD V1 *-*- C1FIH0_	5.27	7.2	8.22	6.39	6.77	4.39	3.49	6.14	6.42	5.11	0.75	0.760084	4.81	5.15	4.85	4.94	3.26	3.02	2.59	2.96	0.6	0.342796	1.44	0.92	2.15	1.5	1.11	1.71	0.62	1.15	0.76	1	0.8	0.41	0.87	0.69	0.26	0.17	0.22	0.31	1
Solyc01g095730.2.1	Os12g0114200 protein (Fragr	8.73	8.77	11.17	10.27	9.73	6.16	8.44	6.76	7.85	7.3	0.75	0.678681	8.85	9.55	13.67	10.69	11.02	10.62	9.06	10.23	0.96	1	9.39	9.15	11.73	10.09	9.22	8.51	7.54	8.42	0.83	1	15.9	11.72	12.14	13.25	11.67	11.96	11.82	0.89	1
Solyc01g095740.2.1	ATP-dependent RNA helicase	21.66	14.56	20.02	16.71	18.24	11.28	17.04	14.42	21.59	16.08	0.88	1	15.92	18.57	24.12	19.54	32.31	31.06	27.98	30.45	1.56	0.054095	6.91	14.44	9.32	10.22	11.89	11.83	14.3	12.67	1.24	1	19.92	21.73	21.78	21.14	26.49	28.62	27.55	1.3	1
Solyc01g095750.2.1	Long-chain-fatty-acid-CoA lig	1	0.98	0.12	1.27	0.84	1.55	1.1	1.56	1.14	1.34	1.59	0.687442	1.67	0.95	1.01	1.21	0.41	0.57	0.39	0.46	0.38	0.350405	0.89	0.16	0.49	0.51	0.13	0.38	0.29	0.27	0.52	1	0	0	0	0	0.09	0	0.04	4.5	1
Solyc01g095760.2.1	UDP-glucosyltransferase (AH	0	0.13	0	0	0.03	0.16	0	0.27	0	0.11	3.31	1	0.14	0	0	0.05	0	0	0.11	0.04	0.79	1	0	0.12	0	0.04	0	0	0	0	0.25	1	0	0	0.22	0.07	0	0	0	0.14	1
Solyc01g095770.2.1	Cyclic nucleotide gated chann	0	0.08	0	0.26	0.09	0	0	0	0	0	0.12	0.90174	0	0	0.08	0.03	0	0.05	0	0.02	0.62	1	0	0.08	0	0.03	0	0	0	0	0.38	1	0	0.07	0	0.02	0	0	0	0.43	1
Solyc01g095780.2.1	Non-specific lipid-transfer pro	0	1.04	1.38	0.83	0.81	0	0.46	0	0	0.12	0.14	0.856058	0	0	0	0	0	0	0	0	1	NA	0	0	0.58	0.19	0	0	0	0	0.05	NA	0	0	0	0	0	0	0	1	NA
Solyc01g095790.2.1	Splicing factor 3a subunit 3 (A	110.77	103.93	130.53	87.52	108.19	80.62	67.93	92.3	95.21	84.02	0.78	0.60848	112.18	123.96	113.63	116.59	220.16	170.22	153.28	181.22	1.55	0.043076	76.58	84.81	66.3	75.9	125.44	87.58	98.84	103.95	1.37	1	340.23	271.92	363.36	325.17	172.92	207.06	189.99	0.58	0.646262
Solyc01g095800.2.1	F-box/LRR-repeat protein (AH	9.2	7.37	4.19	4.83	6.4	6.87	8.09	7.97	7.45	7.59	1.19	0.917343	6.36	5.94	5.11	5.8	4.65	5.38	5.99	5.34	0.92	1	4.99	3.86	4.04	4.3	3.63	3.69	4.88	4.07	0.95	1	2.24	3.53	2.63	2.8	3.25	3	3.12	1.12	1
Solyc01g095810.2.1	Ring H2 finger protein (AHRD	0	0	0	0	0	0	0.35	0	0	0.09	8.75	1	0	0	0	0	0.15	0.11	0	0.09	8.67	1	0	0	0	0	0.15	0	0	0.05	5	1	0	0	0	0	0	0	0	1	NA
Solyc01g095820.2.1	RING finger protein (AHRD V1	0	0	0	0	0	0	0	0	0	0	1	NA	0	0	0	0	0	0	0	0	1	NA	0	0	0	0	0	0	0	0	1	NA	0	0	0	0	0	0	0	1	NA
Solyc01g095830.2.1	Ring H2 finger protein (AHRD	0	0	0	0	0	0	0	0	0	0	1	NA	0	0	0	0	0	0	0	0	1	NA	0	0	0	0	0	0	0	0	1	NA	0	0	0	0	0	0	0	1	NA
Solyc01g095840.1.1	RING finger protein (AHRD V1	0	0	0	0	0	0	0	0	0	0	1	NA	0	0	0	0	0	0	0	0	1	NA	0	0	0	0	0	0	0	0	1	NA	0	0	0	0	0	0	0	1	NA
Solyc01g095850.1.1	Ring H2 finger protein (AHRD	0	0	0	0	0	0	0	0	0	0	1	NA	0	0	0	0	0	0	0	0	1	NA	0	0	0	0	0	0	0	0	1	NA	0	0	0	0	0	0	0	1	NA
Solyc01g095860.2.1	RING finger protein (AHRD V1	0	0	0	0	0	0	0	0	0	0	1	NA	0	0	0	0	0	0	0	0	1	NA	0	0	0	0	0	0	0	0	1	NA	0	0	0	0	0	0	0	1	NA
Solyc01g095870.1.1	Unknown Protein (AHRD V1)	0	0	0	0	0	0	0	0	0	0	1	NA	0	0	0	0	0	0	0	0	1	NA	0	0	0	0	0	0	0	0	1	NA	0	0	0	0	0	0	0	1	NA
Solyc01g095880.1.1	B3 domain-containing protei	0	0	0	0	0	0	0	0	0	0	1	NA	0	0	0	0	0	0	0	0	1	NA	0	0	0	0	0	0	0	0	1	NA	0	0	0	0	0	0	0	1	NA
Solyc01g095890.2.1	Histone-lysine N-methyltrans	7.17	7.61	7.79	4.8	6.84	6.78	5.66	7.23	5.82	6.37	0.93	1	8.25	7.71	9.24	8.4	11.08	10.81	9.74	10.54	1.26	0.615659	7.52	7.74	5.99	7.08	8.47	5.96	6.87	7.1	1	1	3.84	7.17	5.13	5.38	4.95	6.4	5.68	1.05	1
Solyc01g095900.2.1	Protein TIF31 homolog (AHRD	8.79	9.47	10.58	8.9	9.43	15.69	11.74	12.18	13.33	13.23	1.4	0.20585	10.35	11.33	14.41	12.03	25.75	18.34	17.81	20.63	1.72	0.057434	76.16	53.19	78.52	69.29	48.66	66.65	87.94	67.75	0.98	1	9.39	26.38	20.15	18.64	49.73	29.26	39.49	2.12	0.778209
Solyc01g095910.1.1	Membrane protein-like (AHRI	0	0.17	0	0	0.04	0.21	0	0.35	0.21	0.19	4.53	0.844985	0	0.17	0.08	0.08	0	0.15	0.08	0.08	0.92	1	0.08	0	0.1	0.06	0.19	0.07	0.21	0.16	2.61	1	0	0.07	0	0.02	0.18	0	0.09	3.86	1
Solyc01g095920.2.1	O-methyltransferase family 3	2.03	0.77	0.34	0	0.78	0.32	0.69	3.19	2.29	1.62	2.07	0.889786	1.88	1.26	1.47	1.54	1	0.9	1.36	1.09	0.71	1	53.3	39.14	33.32	41.92	14.45	15.13	14.76	14.78	0.35	1	2.06	5.46	4	3.84	6.36	1.52	3.94	1.03	1
Solyc01g095930.2.1	O-acyltransferase WSD1 (AH	88.35	88.89	74.57	22.19	68.5	226.66	87.23	359.91	143.41	204.3	2.98	0.251154	148.26	133.74	67.22	116.41	79.67	95.55	113.3	96.17	0.83	1	946.33	414.27	636.05	665.55	259.73	429.03	316.01	334.92	0.5	1	62.64	189.31	120.25	124.07	234.8	91.82	163.31	1.32	1
Solyc01g095940.2.1	O-acyltransferase WSD1 (AH	90.53	89.14	84.65	26.56	72.72	223.21	84.02	340.91	129.64	194.45	2.67	0.320977	138.41	141.07	76.46	118.65	73.48	84.77	104.82	87.69	0.74	0.824422	952.76	424.57	722.69	700.01	271.74	440.16	301.78	337.89	0.48	1	59.56	180.82	114.78	118.39	230.1	91.2	160.65	1.36	1
Solyc01g095950.1.1	DNA-directed RNA polymeras	0	0	0	0	0	0	0	0.52	0	0.13	13	1	0	0	0.48	0.16	0	0	0	0	0.06	1	5.05	1.39	2.82	3.09	0	0	0.41	0.14	0.04	1	3.23	0	0	1.08	0.52	0	0.26	0.24	1
Solyc01g095960.2.1	Diacylglycerol O-acyltransfera	0	0	0	0	0	0	0	1.92	0.19	0.53	52.75	0.715627	0	0	0	0	0	0	0	0	1	NA	3.79	0.32	2.85	2.32	0.17	0.79	0.19	0.38	0.17	1	0	0	0	0	0.24	0	0.12	12	1
Solyc01g095970.2.1	Dynamin 2 (AHRD V1 ***- Q4	18.46	17.39	14.65	25.96	19.12	9.84	18.16	11.81	14.19	13.5	0.71	0.674429	18.88	26	24.17	23.02	11.16	12.08	14.76	12.67	0.55	0.022302	22.33	24.02	22.72	23.02	19.58	19.07	20.38	19.68	0.85	1	20.11	27.92	31.05	26.36	28.76	28.5	28.63	1.09	1
Solyc01g095980.2.1	Acid phosphatase/vanadium-	5.06	8.18	8.68	10.08	8	14.82	10.53	11.84	9.9	11.77	1.47	0.36763	5.77	6.79	6.21	6.26	9.25	12.26	8.31	9.94	1.59	0.292525	7.06	10.11	7.53	8.23	7.55	7.49	7.71	7.58	0.92	1	1.63	2.34	3.18	2.38	5.05	3.63	4.34	1.82	1
Solyc01g095990.2.1	Phosphatidylinositol kinase fa	25.49	24.74	27.21	31.98	27.36	19.36	19.49	20.15	22.19	20.3	0.74	0.609763	27.36	24.76	34.09	28.74	23.76	28.01	30.09	27.29	0.95	1	25.53	22.27	40.3	29.37	25.23	24.13	19.16	22.84	0.78	1	42.14	60.15	74.31	58.87	40.96	36.61	38.78	0.66	0.831395
Solyc01g096000.2.1	Harpin-induced protein (AHRI	54.76	59.59	60.32	60.66	58.83	48.4	44.01	46.1	57.31	48.95	0.83	0.924458	75.79	69.96	64.91	70.22	54.22	69.34	57.81	60.46	0.86	1	56.5	70.96	55.33	60.93	77.36	97.25	84.13	86.25	1.42	0.872389	44.55	46.24	41.09	43.96	95.72	87.05	91.38	2.08	0.230245
Solyc01g096010.2.1	Unknown Protein (AHRD V1)	12.24	13.49	11.2	11.22	12.04	11.69	13.2	15.82	15.61	14.08	1.17	0.74168	12.34	11.35	13.12	12.27	14.82	16.37	16.66	15.95	1.3	0.444495	20.09	15.65	21.47	19.07	21.08	20.61	22.09	21.26	1.11	1	10.89	21.59	16.34	16.27	18.71	17.78	18.25	1.12	1
Solyc01g096020.2.1	E3 ubiquitin-protein ligase sir	15.6	13.71	14.68	13.41	14.35	10.32	9.82	15.07	12.48	11.92	0.83	0.946674	13.96	8.24	12.51	11.57	13.01	10.94	11.64	11.86	1.03	1	12.29	15.45	11.76	13.17	5.58	10.78	9.64	8.67	0.66	1	10.11	15.49	8.2	11.27	9.58	10.74	10.16	0.9	1
Solyc01g096030.2.1	ENTH/VHS domain-containing	7.57	7.18	7.05	5.78	6.9	6.2	4.88	6.87	6.44	6.1	0.88	1	6.03	4.42	6.31	5.59	5.25	5.42	5.95	5.54	0.99	1	4.99	5.2	7.02	5.74	3.01	3.32	4.87	3.73	0.65	1	5.67	5.36	5.67	5.57	5.2	4.19	4.7	0.84	1
Solyc01g096040.2.1	Aspartic proteinase nepenthe	232.27	210.98	283.39	360.29	271.73	201.43	181.32	172.15	171.27	181.54	0.67	0.304292	144.88	97.2	130.52	124.2	47.28	65.76	76.15	63.06	0.51	0.017069	209.88	189.16	322.93	240.66	133.43	172.31	196.98	167.57	0.7	1	4.48	11.03	8	7.84	46.11	29.76	37.94	4.84	0.015147
Solyc01g096050.2.1	Transcription factor (AHRD V	44.74	33.52	70.38	21.56	42.55	16.58	19.68	19.32	19.3	18.72	0.44	0.249273	57.11	30.76	32.09	39.99	30.5	30.49	55.27	38.75	0.97	1	72.3	66.35	97.25	78.63	101.7	78.88	54.02	78.2	0.99	1	79.25	97.57	125.22	100.68	70.06	70.82	70.44	0.7	0.953402
Solyc01g096060.2.1	Nuclear envelope protein tha	14.8	14.92	17.7	18.07	16.37	9.99	11.95	11.37	12.18	11.37	0.69	0.412494	19.11	18.63	20.1	19.28	16.88	17.04	16.63	16.85	0.87	1	15.67	19.5	19.59	18.25	15.01	14.65	16.01	15.22	0.83	1	12.97	19.78	21.1	17.95	19.8	25.31	22.55	1.26	1
Solyc01g096070.2.1	Auxin response factor 9 (AHR	2.01	2.81	3.5	1.22	2.39	1.7	0.98	2.46	1.3	1.61	0.68	0.645368	1.87	1.83	2.18	1.96	0.99	0.99	0.98	0.99	0.5	0.390116	1.85	1.71	3.23	2.26	2.51	4.35	0.98	2.61	1.15	1	6.25	8.75	5.73	6.91	8.14	2.6	5.37	0.78	1
Solyc01g096080.2.1	Unknown Protein (AHRD V1)	52.65	68.64	40.93	42.06	51.07	62.66	44.99	88.56	44.03	60.06	1.18	0.988051	35.15	28	23.02	28.72	37.59	29.19	29.7	32.16	1.12	0.97353	27.27	38.87	15.68	27.27	21.77	18.31	22.57	20.88	0.77	1	155.89	71.54	58.58	95.34	24.1	21.72	22.91	0.24	0.672787
Solyc01g096090.2.1	Fiber protein Fb11 (AHRD V1	359.84	532.87	233.5	353.47	369.92	418.88	430.08	484.33	325.8	414.77	1.12	0.996578	387.72	382.32	236.39	335.48	262.75	285.16	249.8	265.9	0.79	0.896625	491.8	447.17	306.58	415.18	234.42	343.19	365.65	314.42	0.76	1	89.53	270.79	247.47	202.6	189.41	201.57	195.49	0.96	1
Solyc01g096100.2.1	AT2G46550 protein (Fragmer	60.55	69.22	72.1	81.14	70.75	53.88	50.33	50.31	52.85	51.84	0.73	0.485191	77.77	76.06	94.55	82.79	107.89	94.56	92.77	98.41	1.19	0.728444	187.95	142.26	174.94	168.38	162.31	176.95	167.9	169.05	1	1	790.07	259.85	333.6	461.17	303.61	304.68	304.14	0.66	1
Solyc01g096110.2.1	WD repeat protein (AHRD V1	2.67	3.5	3.9	4.19	3.57	2.1	3.33	2.53	3.26	2.8	0.79	0.895648	4.17	4.6	4.54	4.44	5.15	4.52	4.53	4.73	1.07	1	5.85	5.47	6.71	6.01	3.88	4.39	4.87	4.38	0.73	1	6.19	4.13	5.97	5.43	5.66	6.09	5.88	1.08	1
Solyc01g096120.2.1	Dof zinc finger protein 2 (AH	0	0.21	0	0	0.05	0	0	0	0	0	0.19	1	0	0	0	0	0.17	0.12	0	0.1	9.67	1	0	0	0	0	0	0	0	0	1	1	0	0	0	0	0	0	0	1	NA
Solyc01g096130.2.1	ORM1-like protein 2 (AHRD V	79.84	80.4	78.33	67.16	76.43	71.93	58.39	67.22	72.7	67.56	0.88	1	82.86	90.22	66.37	79.82	99.38	84.2	74.96	86.18	1.08	1	62.8	60.61	44.63	56.01	51.34	49.36	55.42	52.04	0.93	1	89.62	50.36	38.77	59.58	56.54	56.74	56.64	0.95	1
Solyc01g096140.2.1	Aluminum-activated malate t	0	0	0	0	0	0.29	0.1	0	0	0.1	9.75	0.985318	0	0	0	0	0	0	0	0	1	NA	0	0	0.13	0.04	0	0	0	0	0.23	1	0	0	0	0	0	0	0	1	NA
Solyc01g096150.2.1	NC domain-containing protei	21.21	27.17	15.87	22.32	21.64	25.31	24.92	26.17	25.45	25.46	1.18	0.845205	17.33	18.67	13.77	16.59	13.1	9.38	15.86	12.78	0.77	0.814091	40.34	24.67	27.73	30.91	20.56	33.92	31.86	28.78	0.93	1	0.74	7.33	4.77	4.28	5.68	1.81	3.75	0.88	1
Solyc01g096160.2.1	Predicted kinase (AHRD V1 *-	5.32	6	5.4	6.13	5.71	4.75	4.06	4.21	5.2	4.55	0.8	0.847844	5.24	5.38	4.91	5.18	4.76	4.2	3.65	4.2	0.81	0.964678	5.3	4.19	4.18	4.56	4.11	3.42	3.34	3.62	0.8	1	6.86	4.99	5.81	5.89	5.78	5.05	5.42	0.92	1
Solyc01g096170.2.1	Serine/threonine protein kina	0.34	0.31	0.3	0.61	0.39	0.29	0.34	0.39	0.43	0.36	0.93	1	0.16	0.52	0.65	0.44	0.48	0.71	0.74	0.64	1.45	1	0.42	0.77	0.68	0.62	0.23	0.26	0.19	0.23	0.36	1	0.24	0.37	0.53	0.38	0.16	0.38	0.27	0.71	1
Solyc01g096180.2.1	Arginine/serine-rich splicing f	27.89	30.31	19.3	20.1	24.4	28.92	25.19	25.15	32.73	28	1.15	0.922871	29.93	39.4	29.22	32.85	28.83	31.24	40.04	33.37	1.02	1	26.65	18.36	15.17	20.06	19.91	19.31	22.67	20.63	1.03	1	34.32	30.39	33.82	32.84	23.81	26.1	24.95	0.76	1
Solyc01g096190.2.1	Calcium-transporting ATPase	1.7	3.02	3.71	4.36	3.2	0.84	1.19	1.85	1.85	1.43	0.45	0.164079	2.05	2.41	3.72	2.73	1.57	1.14	0.89	1.2	0.44	0.092783	0.76	0.51	0.69	0.65	0.5	0.38	0.09	0.32	0.49	1	0.18	0.55	0.29	0.34	0.17	0.28	0.23	0.66	1
Solyc01g096200.2.1	Pentatricopeptide repeat-con	7.39	6.66	9.42	12.54	9	5.75	7.25	5.94	5.47	6.1	0.68	0.552588	6.14	9.83	10.26	8.74	6.08	5.09	5.22	5.46	0.62	0.191194	3.88	6.06	17.14	9.03	8.73	5.25	5.62	6.53	0.72	1	3.81	7.64	9.3	6.92	4.61	4.41	4.51	0.65	0.976684
Solyc01g096210.2.1	Pentatricopeptide repeat-con	6.61	6.41	8.35	7.51	7.22	7.22	5.87	7.38	8.56	7.26	1.01	1	7.57	8	8.37	7.98	7.37	9.65	7.42	8.15	1.02	1	4.48	5.19	6.09	5.25	4.81	3.76	6.11	4.89	0.93	1	8.22	7.63	6.53	7.46	6.48	8.59	7.54	1.01	1
Solyc01g096220.2.1	Ras-related protein Rab-25 (A	62.19	52.7	45.3	65.02	56.3	65.22	89.65	84.27	73.96	78.27	1.39	0.191176	60.11	30.32	30.14	40.19	23.07	20.44	24.86	22.79	0.57	0.614547	150.07	210.62	111.11	157.27	102.31	65.17	198.6	122.03	0.78	1	0.77	8.25	7.48	5.5	11.88	10.9	11.39	2.07	0.82649
Solyc01g096230.2.1	ATPase AAA family protein ex	14.08	13.01	16.45	15.17	14.68	15.35	14.4	11.34	11.94	13.26	0.9	1	16.96	21.21	19.14	19.1	16.55	18.19	14.86	16.53	0.87	1	99.88	96.7	85.31	93.96	94.6	65.45	116.86	92.3	0.98	1	34.13	31.94	24.54	30.2	34.25	24.57	29.41	0.97	1
Solyc01g096240.2.1	Homology to unknown gene (	236.02	240.15	194.72	206.17	219.26	401.08	370.03	372.04	423.01	391.54	1.79	0.00236	198.05	215.31	194.72	202.69	357.29	338.25	351.5	349.01	1.72	0.005254	56.38	84.42	54.84	65.21	141.28	126.18	158.97	142.14	2.18	0.05235	13.21	40.82	35.52	29.85	67.15	59.93	63.54	2.13	0.30764
Solyc01g096250.2.1	Unknown Protein (AHRD V1)	4.53	2.69	4.58	11.59	5.85	5.27	15.32	1.98	6.04	7.15	1.22	0.982309	3.01	3.37	5.83	4.07	4.64	5.47	2.37	4.16	1.02	1	0.52	0.88	0	0.47	1.13	0.49	1.1	0.91	1.94	1	23.6	0	0.99	8.2	1.78	2.84	2.31	0.28	1
Solyc01g096260.2.1	Unknown Protein (AHRD V1)	1.31	0.47	0	0.37	0.54	3.2	3.72	2.65	1.5	2.77	5.15	0.040809	0.73	0.23	0	0.32	0.73	0.27	0.21	0.4	1.26	1	0	0	0	0	0	0	0	0	1	NA	0	0	0	0	0	0	0	1	NA
Solyc01g096270.2.1	Unknown Protein (AHRD V1)	1017.55	953.66	853.79	920.43	936.36	901.09	1024.29	887.79	988.15	950.33	1.01	1	994.86	1106.11	855.23	985.4	1143.9	1075.44	1241.13	1153.49	1.17	0.779363	1309.04	1221.76	1037.79	1189.53	1312.63	1731.87	1271.05	1438.52	1.21	1	1199.47	995.33	868.45	1021.08	1441.14	1538.09	1489.62	1.46	0.83791
Solyc01g096280.1.1	Cytochrome P450	1.59	0.65	0	0	0.56	1.09	0.29	0.68	0.79	0.71	1.27	1	0.8	0.85	0.62	0.76	0.51	0.44	0.87	0.61	0.8	1	0.99	1.1	0.73	0.94	0.96	0.74	0.54	0.75	0.79	1	0.35	0.18	0.56	0.36	0.34	0.64	0.49	1.35	1
Solyc01g096290.2.1	Ubiquitin (AHRD V1 ***- C0S	648.46	630.83	522.5	515.77	579.39	513.32	473.19	596.69	486.11	517.33	0.89	0.977921	508.52	478.78	409.48	465.59	375.63	336.01	368.94	360.19	0.77	0.662803	333.01	329.63	287.31	316.65	275.82	300.01	305.96	293.93	0.93	1	181.78	367.72	367.25	305.58	358.13	443.01	400.57	1.31	1
Solyc01g096300.2.1	Cysteine-type endopeptidase	3.99	7.31	6.65	1.33	4.82	6.52	6.3	8.42	5.39	6.66	1.38	0.802621	6.33	4.08	2.78	4.4	2.93	3.4	8.12	4.82	1.1	1	3.98	2.1	0.47	2.18	0.77	1.07	2.4	1.41	0.65	1	2.34	1.53	1.44	1.77	0.86	1.44	1.15	0.65	1
Solyc01g096310.1.1	Unknown Protein (AHRD V1)	0	0	0	0	0	0	0	0	0	0	1	NA	0	0	0	0	0	0	0	0	1	NA	0	0	0	0	0	0	0	0	1	NA	0	0	0	0	0	0	0	1	NA
Solyc01g096320.2.1	Homeobox leucine zipper pro	44.26	61.16	78.16	12.05	48.91	99	114.36	253.49	156.89	155.94	3.19	0.039316	102.79	41.1	19.27	54.39	34.32	29.18	53.36	38.95	0.72	1	138.58	81.98	29.47	83.34	44.96	46.04	108.17	66.39	0.8	1	14.85	27.15	14.79	18.93	26.92	13.98	20.45	1.08	1
Solyc01g096330.2.1	Unknown Protein (AHRD V1)	0	2.35	0.78	0.93	1.01	0	1.56	0.61	0.47	0.66	0.65	1	0.61	1.72	0.56	0.96	1.37	1.02	1.56	1.32	1.37	1	2.13	1.08	0.65	1.29	2.17	2.5	0	1.56	1.21	1	0	0.48	3.04	1.17	1.21	0.58	0.9	0.76	1
Solyc01g096340.2.1	Auxin-induced SAUR-like prot	39.58	50.71	38.92	22.9	38.03	81.33	47.35	125.17	63.72	79.39	2.09	0.209704	58.58	55.78	41.54	51.97	42.43	54.65	89.08	62.05	1.19	1	328.03	252.17	226.21	268.8	232.45	246.37	309.42	262.75	0.98	1	13.64	139.39	99.6	84.21	79.61	63.63	71.62	0.85	1
Solyc01g096350.2.1	Calcium dependent protein ki	28.51	28.16	35.62	36.06	32.09	20.38	20.04	19.64	23.68	20.94	0.65	0.239513	29.12	35.67	37.87	34.22	24.97	25.36	26.27	25.53	0.75	0.577473	53.3	48.38	76.36	59.35	50.32	46.87	39.52	45.57	0.77	1	95.39	88.2	91.65	91.75	64.59	66.86	65.72	0.72	0.989607
Solyc01g096360.2.1	Rho GTPase activating protei	8.11	6.98	6.12	9.97	7.79	3.99	9.24	5.01	6.02	6.07	0.78	0.951897	5.44	5.2	6.18	5.61	2.68	3.58	3.87	3.38	0.6	0.410746	0.54	0.87	0.4	0.6	0.26	0.3	0	0.19	0.31	1	0	0.48	1.23	0.57	0.37	0.35	0.36	0.63	1
Solyc01g096370.2.1	BHLH transcription factor (AH	9.65	6.25	7.77	6.21	7.47	4.72	7.62	11.97	12.81	9.28	1.24	0.78597	9.25	2.29	1.98	4.51	3.75	2.04	3.1	2.96	0.66	1	0	0.12	0	0.04	0.1	0	0.11	0.07	1.75	1	0	0	0	0	0.13	0	0.07	6.5	1
Solyc01g096380.1.1	Unknown Protein (AHRD V1)	0.42	0	0	0.3	0.18	0	0	0	0	0	0.06	0.907108	0.19	0.55	0.35	0.36	0	0.11	0.16	0.09	0.25	0.94937	0	1.19	0.21	0.47	0.14	0.32	0	0.15	0.33	1	0.3	0	0	0.1	0	0	0	0.1	1
Solyc01g096390.2.1	DNA-directed RNA polymeras	10.92	12.41	13.44	10.17	11.73	10.39	9.03	10.62	9.96	10	0.85	0.912648	9.83	10.79	16.23	12.28	11.85	12.31	10.04	11.4	0.93	1	4.44	6.21	5.27	5.31	6.94	4.26	5.44	5.55	1.05	1	8.04	9.22	11.54	9.6	8.99	10.82	9.91	1.03	1
Solyc01g096400.2.1	Unknown Protein (AHRD V1)	1.24	0.97	1.84	1.98	1.51	1.21	1.35	1.43	1.68	1.42	0.94	1	1.01	1.49	1.84	1.45	2.16	2.34	1.96	2.15	1.49	0.835633	1.51	3.17	2.47	2.38	1.43	2.12	2.38	1.98	0.83	1	0.88	1.36	0.48	0.91	1.28	0.68	0.98	1.08	1
Solyc01g096410.2.1	Phosphatidylinositol-4-phosp	0.59	1.51	1.3	1.2	1.15	2.91	3	2.02	3.28	2.8	2.44	0.017308	0.71	1.03	0.71	0.82	0.65	0.35	0.66	0.55	0.68	0.961037	0.07	0.28	0	0.12	0.06	0.06	0.12	0.08	0.69	1	0	0	0.13	0.04	0.08	0	0.04	0.92	1
Solyc01g096420.2.1	Predicted NADPH-dependent	3.4	4.84	5.62	15.87	7.43	1.89	4.03	2.81	4.39	3.28	0.44	0.807096	4.11	1.48	2.3	2.63	0.71	1.76	5.08	2.52	0.96	1	0.27	0.83	0	0.37	0.22	0	0	0.07	0.2	1	0	0	0	0	0	0	0	1	NA
Solyc01g096430.2.1	Predicted NADPH-dependent	71.72	74.07	91	149.31	96.53	50.77	65.7	62.52	61.94	60.23	0.62	0.585007	54.44	68.82	81.72	68.33	51.39	37.49	40.15	43.01	0.63	0.136183	33.62	49.62	40.63	41.29	20	20.51	30.08	23.53	0.57	1	4.33	10.32	7.28	7.31	17.34	29.37	23.36	3.19	0.117916
Solyc01g096440.2.1	Pentatricopeptide repeat-con	1.36	1.01	1.07	0.48	0.98	1.26	1.43	1.04	1.79	1.38	1.41	0.858261	0.63	1.68	0.86	1.06	1.65	1.29	1.16	1.37	1.29	1	0.18	0.37	0.45	0.33	0.3	0.52	0.66	0.49	1.48	1	0.81	0.33	0.17	0.44	0.42	0.3	0.36	0.82	1
Solyc01g096450.2.1	Aspartic proteinase nepenthe	56.91	53.29	55.57	124.84	72.65	60.45	110.85	24.04	44.8	60.03	0.83	1	15.89	8.91	15.06	13.29	5.87	5.08	3.74	4.9	0.37	0.015065	0.84	1.82	1.18	1.28	0.59	0.56	0.65	0.6	0.47	1	1.48	0.54	0.91	0.98	1.77	2.87	2.32	2.38	0.760024
Solyc01g096460.2.1	U-box domain-containing pro	14.8	15.05	9.23	12.86	12.98	7.52	8.35	13.41	13.21	10.62	0.82	0.926825	16.32	15.44	15.36	15.71	18.48	15.98	15.29	16.58	1.06	1	7.35	12.23	7.77	9.12	5.97	6.41	8.63	7	0.77	1	44.19	31.64	49.1	41.64	18	30.22	24.11	0.58	0.794331
Solyc01g096470.2.1	Transcription factor (AHRD V	23.78	24.07	28.96	18.99	23.95	32.83	23.92	30.5	20.65	26.98	1.13	0.963476	21.23	25.34	37.58	28.05	32.19	29.29	18.39	26.62	0.95	1	11.93	12.19	7.23	10.45	14.87	9.16	7.27	10.43	1	1	11.18	13.23	12.09	12.17	8.78	13.28	11.03	0.91	1
Solyc01g096480.2.1	Thioredoxin protein (AHRD V	49.85	40.7	59.26	48.25	49.52	81.67	82.27	63.41	67.55	73.72	1.49	0.117292	34.17	50.5	50.34	45	100.35	82.68	78.06	87.03	1.93	0.002858	19.08	24.74	23.07	22.3	44.32	38.07	33.32	38.57	1.73	0.283167	28.78	29.95	25.94	28.22	62.79	53.57	58.18	2.06	0.32208
Solyc01g096490.2.1	Kinase family protein (AHRD	10.21	10.64	12.81	14.62	12.07	6.37	7.43	6.35	8.35	7.12	0.59	0.090112	10.21	13.48	18.62	14.1	13.44	12.65	11.77	12.62	0.89	1	13.15	15.96	19.28	16.13	13.82	13.86	13.68	13.79	0.85	1	23.13	16.19	22.23	20.52	17.52	20.46	18.99	0.93	1
Solyc01g096500.2.1	D-erythro-sphingosine kinase	22.55	19.21	24.84	36.01	25.65	15.86	16.69	15.22	18.46	16.56	0.65	0.40572	20.97	26.03	33.77	26.92	26.45	27.32	21.39	25.05	0.93	1	12.67	20.17	19.15	17.33	13.42	9.81	11.75	11.66	0.67	1	28.55	23.29	22.48	24.77	16.69	20.08	18.38	0.74	1
Solyc01g096510.2.1	Sigma factor binding protein	0.41	1.11	0	0.59	0.53	0	0	0.38	0.6	0.24	0.46	1	0	0.72	0.35	0.36	0.29	0	1.3	0.53	1.49	1	0	0	0	0	0.27	0	0	0.09	9	1	0.59	0.6	0	0.4	0	0	0	0.03	1
Solyc01g096520.2.1	Peptidyl-prolyl cis-trans isom	136.78	117.43	121.43	136.31	127.99	108.65	90.53	99.4	135.14	108.43	0.85	0.960351	101.53	116.2	106.77	108.17	103.97	110.52	104.78	106.42	0.98	1	72.27	89.78	76.8	79.62	86.68	79.32	80.54	82.18	1.03	1	71.7	101.85	82.33	85.29	116.99	113.87	115.43	1.35	1
Solyc01g096530.2.1	Trafficking protein particle co	56.09	50.24	37.04	38.92	45.57	34.9	43.73	41.19	44.32	41.03	0.9	1	49.55	38.95	29.8	39.43	26.67	30.14	34.66	30.49	0.77	0.828763	48.74	37.95	28.71	38.47	31.06	38.77	38.56	36.13	0.94	1	28.09	54.58	55.88	46.18	31.65	36.81	34.23	0.74	1
Solyc01g096540.2.1	AT5g06970/MOJ9_14 (AHRD	12.29	14.69	14.78	15.37	14.28	10.3	13.74	13.98	12.41	12.61	0.88	1	12.43	13.83	13.84	13.37	10.05	10.05	11.53	10.54	0.79	0.780946	7.92	8.76	9.14	8.61	7.29	7.7	7.53	7.51	0.87	1	9.71	17.85	15.71	14.42	10.27	11.66	10.96	0.76	1
Solyc01g096550.2.1	Protein arginine methyltransf	9.91	8.16	9.45	7.16	8.67	4.57	7.07	5.23	7.41	6.07	0.7	0.561164	8.53	6.78	7.39	7.57	6.8	8.24	8.7	7.91	1.05	1	4.34	4.64	4.29	4.42	5.05	3.83	4.3	4.39	0.99	1	12.44	8.19	12.15	10.93	11.48	14.04	12.76	1.17	1
Solyc01g096560.1.1	Subtilisin-like protease (AHRD	0.18	0.16	0	0	0.08	0.3	0.42	0.16	0.19	0.27	3.15	0.777577	0.08	0.16	0	0.08	0.06	0.14	0.14	0.11	1.42	1	0.07	0	0.09	0.05	0.06	0	0.07	0.04	0.81	1	0	0.65	0	0.22	0.16	0	0.08	0.37	1
Solyc01g096570.2.1	Exonuclease family protein ex	201.11	198.18	222.38	239.96	215.41	183.06	189.16	164.49	208.83	186.39	0.87	1	212.62	205.37	224.23	214.07	227.67	187.66	190.31	201.88	0.94	1	142.47	185.22	130.65	152.78	123.72	142.74	166.8	144.42	0.95	1	201.45	122.98	121.1	148.51	159.38	135.99	147.69	0.99	1
Solyc01g096580.2.1	30S ribosomal protein S10 (A	1498.89	1156.86	1325.12	1326.98	1326.96	874.39	993.54	632.78	1098.16	899.72	0.68	0.244915	1232.5	1047.32	997.31	1092.38	902.71	805.02	688.92	798.88	0.73	0.482547	525.76	744.86	576.02	615.55	570.75	610.61	589.11	590.16	0.96	1	462.63	550.21	657.68	556.84	745.16	806.37	775.76	1.39	0.924156
Solyc01g096590.2.1	30S ribosomal protein S10 (A	1531.31	1226.03	1435.93	1390.25	1395.88	931.57	1069.9	639.46	1190.48	957.85	0.69	0.281526	1231.46	1074.05	1073.81	1126.44	992.29	890.12	710.45	864.29	0.77	0.669164	556.47	799.07	621.53	659.02	646.77	641.97	640.85	643.2	0.98	1	472.96	568.08	682.62	574.55	778.42	820.09	799.25	1.39	0.926601
Solyc01g096600.2.1	THO complex subunit 1 (AHRI	33.47	36.06	39.62	36.62	36.44	33.6	30.7	39.94	38.73	35.74	0.98	1	37.79	39.55	40.35	39.23	37.27	38.31	37.71	37.76	0.96	1	29.67	26.75	30.21	28.88	38.64	30.62	29.05	32.77	1.13	1	62.22	56.96	62.47	60.55	47.8	54.42	51.11	0.84	1
Solyc01g096610.2.1	UPF0468 protein C16orf80 hc	36.65	41.15	41.58	33.9	38.32	49.51	31.16	47.95	38.66	41.82	1.09	1	39.07	29.65	25.72	31.48	25.03	22.04	27.25	24.77	0.79	0.846643	40.77	31.24	27.98	33.33	22.97	34.62	25.48	27.69	0.83	1	24.45	29.9	26.01	26.79	15.74	18.29	17.02	0.64	0.854307
Solyc01g096620.2.1	Unknown Protein (AHRD V1)	67.88	78.29	101.14	33.81	70.28	60.93	53.84	96.99	63.61	68.84	0.98	1	71.44	46.82	36.61	51.62	26.53	28.71	39.07	31.44	0.61	0.567141	31.41	26.75	24.04	27.4	27.85	20.29	25.08	24.41	0.89	1	12.23	30.55	20.58	21.12	16.34	20.96	18.65	0.88	1
Solyc01g096630.2.1	ACI112 (AHRD V1 ***- Q3SC8	88.32	108.9	171.36	54.18	105.69	79.25	76.02	117.4	94.72	91.85	0.87	1	114.81	72.61	51.68	79.7	38.01	34.71	50.59	41.1	0.52	0.43428	49	43.79	33.8	42.2	37.75	35.08	33.34	35.39	0.84	1	21.94	38.25	20.45	26.88	25.33	28.92	27.12	1.01	1
Solyc01g096640.2.1	Unknown Protein (AHRD V1)	118.93	131.49	209.38	70.97	132.69	97.09	104.9	129.86	109.4	110.31	0.83	0.982298	140.02	89.66	72.99	100.89	51.56	29.83	59.88	47.09	0.47	0.208529	70.56	50.73	40.58	53.96	43.34	45.07	42.66	43.69	0.81	1	31.71	47.24	21.43	33.46	28.92	39.92	34.42	1.03	1
Solyc01g096650.2.1	RING finger protein (AHRD V1	0	0	0	0	0	0	0	0	0	0	1	NA	0	0	0	0	0	0	0	0	1	NA	0	0	0	0	0	0	0	0	1	NA	0	0	0	0	0	0	0	1	NA
Solyc01g096660.2.1	Genomic DNA chromosome 5	117.04	101.71	85.97	64.42	92.29	191.77	163.88	124.87	223.93	176.11	1.91	0.016132	115.28	107.26	72.04	98.19	180.15	131.06	140.78	150.66	1.53	0.123139	259.13	237.07	150.14	215.45	231.38	313.61	226.09	257.03	1.19	1	86.34	133.32	87.44	102.37	263.29	151.5	207.4	2.03	0.698088

Solyc01g096670.2.1	Cytochrome P450	9.35	6.5	14.21	9.6	9.92	5.41	4.85	4.83	7.63	5.68	0.57	0.313823	9.77	5.79	4.65	6.74	3.73	1.99	3.62	3.11	0.46	0.318905	8.18	8.66	4.45	7.1	3.03	2.33	2.05	2.47	0.35	1	39.89	1.4	4.92	15.4	3.64	4.49	4.07	0.26	1
Solyc01g096680.2.1	Microtubule-associated prote	1.04	0.62	0.68	0.98	0.83	0.26	0.55	0.21	0.75	0.44	0.53	0.846866	0.22	0.8	1.27	0.76	0.56	0.48	0.55	0.53	0.69	1	0.56	0.38	0	0.31	0.08	0.09	0.08	0.08	0.27	0.834099	0	0	0	0	0.11	0	0.06	5.5	1
Solyc01g096690.1.1	Receptor-like kinase (AHRD V	0	0	0.12	0	0.03	0.11	0	0	0	0.03	0.92	1	0	0	0	0	0	0	0	0	1	NA	0	0	0	0	0	0	0	0	1	NA	0	0	0	0	0	0	0	1	NA
Solyc01g096700.2.1	Chaperone protein dnaJ 1 (AH	370.45	377.17	492.09	489.49	432.3	401.71	329.03	376.35	377.97	371.27	0.86	0.977792	354.53	325.97	444.12	374.87	375.56	398.99	362.15	378.9	1.01	1	255.76	281.37	303.73	280.29	345.22	283.07	364.37	330.89	1.18	1	182.99	192.6	225.31	200.3	269.14	241.5	255.32	1.27	1
Solyc01g096710.2.1	Nuclear transcription factor Y	95.03	87.75	96.94	92.31	93.01	90.28	96.08	90	85.66	90.5	0.97	1	85.29	94.82	86.7	88.94	78.63	81.76	82.17	80.85	0.91	1	120.07	107.9	104.49	110.82	113.43	102.89	110.55	108.96	0.98	1	118.52	106.38	124.52	116.47	99.63	95.64	97.63	0.84	1
Solyc01g096720.2.1	Major facilitator superfamily	0	0	0	0.39	0.1	0	0	0	0	0	0.1	1	0	0.12	0.12	0.08	0	0	0	0	0.12	1	0.11	0	0.14	0.08	0.18	0	0.2	0.13	1.52	1	0	0	0	0	0.13	0	0.07	6.5	1
Solyc01g096730.2.1	Major facilitator superfamily	1.23	1.22	1.13	3.49	1.77	0.3	0.97	1.01	0.49	0.69	0.39	0.595668	0.77	1.79	1.74	1.43	0.57	0.43	0.75	0.58	0.41	0.401947	1.77	0.89	3.95	2.2	4.05	1.56	0.8	2.14	0.97	1	0.59	1.59	2.32	1.5	1.51	1.2	1.35	0.9	1
Solyc01g096740.2.1	Transporter major facilitator	1.36	0.85	2.57	1.92	1.67	1.97	0.64	2.25	1.46	1.58	0.94	1	0.51	0.83	1.15	0.83	0.19	0.77	0.32	0.43	0.51	0.944042	3.96	1.88	6.75	4.2	3.93	4.85	2.38	3.72	0.89	1	3.29	4.74	3.14	3.72	15.69	10.5	13.09	3.52	0.03286
Solyc01g096750.1.1	Argonaute 4-like protein (AH	0	0.07	0	0	0.02	0	0.18	0	0.11	0.07	4.14	1	0	0.46	0.63	0.36	0.73	1.28	0.47	0.83	2.28	0.594027	0	0.3	0	0.1	0	0	0.27	0.09	0.9	1	0	0	0	0	0	0.2	0.1	10	1
Solyc01g096760.2.1	Pre-rRNA-processing protein	44.37	43.61	48.7	40.38	44.27	26.16	26.5	28.02	34.07	28.69	0.65	0.285628	48.09	42.59	41.06	43.91	34.52	45.74	40.93	40.4	0.92	1	31.48	31.44	33.32	32.08	31.93	27.78	29.52	29.74	0.93	1	55.4	82.94	86.62	74.99	34.96	46.68	40.82	0.54	0.479394
Solyc01g096770.2.1	Unknown Protein (AHRD V1)	0	0.78	0	2.49	0.82	0	0	0	0	0	0.01	0.90174	0	0	1.49	0.5	0	0	0	0	0.02	1	0	0	0	0	0	0	0	0	1	NA	0	0	0	0	0	0	0	1	NA
Solyc01g096780.2.1	Sperm-associated SUN domai	64.69	65.96	52.31	51.2	58.54	47.62	38.55	53.57	49.52	47.31	0.81	0.715721	65.75	65.17	61.69	64.2	60.61	63.32	52.69	58.87	0.92	1	34.26	41.02	26.58	33.95	35.18	22.89	30.9	29.66	0.87	1	67.61	51.8	65.55	61.65	35.69	45.45	40.57	0.66	0.941183
Solyc01g096790.2.1	B3 domain-containing protein	3.55	5.05	5.86	3.01	4.37	6.31	5.6	5.22	0.51	4.41	1.01	1	3.96	1.23	4.79	3.33	4.92	6.61	4.46	5.33	1.6	0.896625	1.15	0.58	0.7	0.81	0	0	1.55	0.52	0.64	1	1.01	3.09	2.18	2.09	1.3	1.87	1.58	0.76	1
Solyc01g096800.2.1	SWI4 1 Peter Pan-like protein	21.74	17.99	15.07	14.29	17.27	22.47	24.24	27.87	13.55	22.03	1.28	0.676591	21.64	18.4	12.08	17.37	12.17	15.7	24.27	17.38	1	1	14.48	14.6	11.17	13.42	12.78	10.12	14.59	12.5	0.93	1	15.45	15.09	16.13	15.56	9.95	16.22	13.08	0.84	1
Solyc01g096810.2.1	Ethylene insensitive 3 class tr	45.81	40.96	61.28	66.19	53.56	24.48	23.53	22.17	22.58	23.19	0.43	0.000511	38.33	67.25	68.4	57.99	70.08	57.36	47.97	58.47	1.01	1	40.81	50.9	61.68	51.13	41.97	45.35	42.8	43.37	0.85	1	161.42	118.99	124.2	134.87	84.77	83.5	84.13	0.62	0.759291
Solyc01g096820.2.1	Calcium-dependent protein k	1.28	1.14	0.6	0	0.76	0.85	0.71	1.41	1.28	1.06	1.41	1	0.83	0.67	0.43	0.64	0.62	1.45	0.4	0.82	1.28	1	1.34	1.35	1.14	1.28	0.17	0	0.37	0.18	0.14	0.897251	0	0.37	0.39	0.25	0.23	0	0.12	0.45	1
Solyc01g096830.2.1	Calcium-transporting ATPase	22.94	22.61	27.79	28.18	25.38	18.91	21.91	16.31	19.32	19.11	0.75	0.619391	24.88	27.97	33.7	28.85	30.7	24.89	24.19	26.59	0.92	1	25.9	35.25	35.55	32.23	29.77	24.64	29.44	27.95	0.87	1	41.69	33.41	37.43	37.51	36.52	38.41	37.47	1	1
Solyc01g096840.2.1	Os02g0794300 protein (Fragi	0	0.2	0	0.16	0.09	0	0.09	0.1	0.08	0.07	0.75	1	0	0.2	0	0.07	0.16	0.17	0	0.11	1.65	1	0	0	0.11	0.04	0	0	0.08	0.03	0.73	1	0	0	0.17	0.06	0.1	0	0.05	0.88	1
Solyc01g096850.2.1	Protein FAR1-RELATED SEQU	6.48	6.1	7.01	6.47	6.51	7.54	5.65	7.42	5.58	6.55	1	1	5.74	6.56	8.81	7.04	6.34	6.94	7.54	6.94	0.99	1	6.03	5.15	4.31	5.16	5.6	5.31	5.38	5.43	1.05	1	7.04	7.86	7.15	7.35	4.74	5.61	5.18	0.7	1
Solyc01g096860.1.1	AP2-like ethylene-responsive	0.59	0.53	2.45	0.21	0.95	0	0.12	0	0.11	0.06	0.06	0.378057	0	0.26	0.13	0.13	0	0	0	0	0.08	0.964678	0	0	0	0	0	0	0	0	1	NA	0.42	0	0	0.14	0	0	0	0.07	1
Solyc01g096870.2.1	Prolyl-tRNA synthetase (AHR	57.54	53.14	50.42	55	54.03	67.98	82.12	67.3	82.11	74.88	1.39	0.16671	54.87	66.35	69.85	63.69	125.08	101.6	91.49	106.06	1.67	0.012549	27.72	48.36	38.24	38.11	58.05	54.29	53.43	55.26	1.45	0.71417	61.79	81.67	75.9	73.12	107.78	112.84	110.31	1.51	0.844615
Solyc01g096880.2.1	Nitrate transporter (AHRD V1	3.9	2.45	0.95	1.46	2.19	0.89	1.54	1.16	0.99	1.15	0.52	0.560738	0.53	0.4	0.39	0.44	0.48	0.53	0.63	0.55	1.24	1	0.19	0.28	0	0.16	0	0.09	0	0.03	0.19	1	0	0	0	0	0	0.1	0.05	5	1
Solyc01g096890.2.1	Genomic DNA chromosome 5	5.93	5.6	7.28	6.51	6.33	6.59	9.56	7.79	5.39	7.33	1.16	0.858577	6.64	7.37	5.93	6.65	5.04	6.14	5.99	5.72	0.86	1	6.26	8.19	5.53	6.66	6.04	6.51	7.14	6.56	0.99	1	3.96	6.24	5.77	5.32	4.99	4.99	4.99	0.94	1
Solyc01g096900.2.1	DNA gyrase subunit A family	8.92	10.15	9.87	10.37	9.83	9.84	9.94	10.3	11.32	10.35	1.05	1	10.03	8.96	12.73	10.57	14.2	13.82	13.31	13.78	1.3	0.491744	4.53	7.33	5.35	5.74	5.86	5.77	7.05	6.23	1.09	1	11.41	8.34	11.91	10.55	13.23	15.4	14.32	1.36	0.938102
Solyc01g096910.2.1	Vacuolar protein sorting 36 fa	11.72	13.24	18.79	14.24	14.5	9.02	6.64	9.4	9.82	8.72	0.6	0.185181	12.44	16.33	17.01	15.26	14.38	10.27	11.93	12.19	0.8	0.857418	5.83	8.45	8.2	7.49	8	5.24	7.34	6.86	0.92	1	9.61	12.77	13.87	12.08	7.57	8.3	7.94	0.66	0.953653
Solyc01g096920.2.1	Polyadenylation factor I comp	16.13	16.91	16.33	12.69	15.51	13.16	16.81	21.33	14.53	16.46	1.06	1	15.45	15.93	16.13	15.84	17.94	18.73	19.21	18.63	1.18	0.820371	6.4	8.87	5.02	6.76	8.22	7.74	7.51	7.82	1.16	1	24.3	15.05	13.8	17.72	16.52	17.16	16.84	0.95	1
Solyc01g096930.2.1	Phospholipid-transporting AT	0.9	1.15	2.26	2.55	1.71	1.13	0.4	1.34	1.21	1.02	0.59	0.741122	1.73	2.1	2.95	2.26	2.38	2.18	2.25	2.27	1	1	3.09	4.08	4.91	4.03	2.48	2.22	3.2	2.63	0.65	1	0.4	1.76	1.38	1.18	0.57	0.99	0.78	0.66	1
Solyc01g096940.2.1	Receptor like kinase, RLK	2.12	4.37	3.1	3.35	3.23	4.9	3.21	4.2	2.86	3.79	1.17	0.974475	2.28	1.69	1.79	1.92	1.7	1.97	1.46	1.71	0.89	1	0.34	0	0.08	0.14	0	0	0.31	0.1	0.74	1	0	0.06	0.39	0.15	0.08	0	0.04	0.27	1
Solyc01g096950.2.1	Retinoblastoma-binding prote	5.29	7.28	5.05	6.59	6.05	6.58	5.96	5.79	5.78	6.03	1	1	6.56	5.69	6.17	6.14	4.72	5.74	6.73	5.73	0.93	1	6.31	6.36	4.75	5.81	5.38	5.92	6.61	5.97	1.03	1	6.27	6.68	6	6.32	5.53	6.84	6.19	0.98	1
Solyc01g096960.2.1	22.7 kDa class IV heat shock p	1.94	1.73	1.58	1.48	1.68	1.49	1.3	1.65	2.57	1.75	1.04	1	0.83	0.91	1.26	1	2.38	0.31	0.23	0.97	0.97	1	0.72	1.46	0	0.73	0.39	0.68	0.65	0.57	0.79	1	0	0.87	0	0.29	0.14	0.13	0.14	0.47	1
Solyc01g096970.2.1	Unknown Protein (AHRD V1);	19.24	30.99	24.86	23.33	24.61	35.65	28.93	56.44	32.89	38.48	1.56	0.364896	46.88	46.2	28.63	40.57	36.86	51.24	54.73	47.61	1.17	0.936234	33.31	44.05	16.37	31.24	15.05	18.75	35.39	23.06	0.74	1	15.63	29.28	19.73	21.55	23.49	32.94	28.21	1.31	1
Solyc01g096980.1.1	Restricted tev movement 2 (A	0.38	1.18	0.67	0	0.56	0.42	0.15	0.35	0.27	0.3	0.53	0.905847	0.35	0.33	0.48	0.39	0.26	0	0	0.09	0.22	0.855159	0.15	0.31	0	0.15	0	0	0.28	0.09	0.61	1	0	0.14	0	0.05	0	0	0	0.21	1
Solyc01g096990.2.1	Guanine nucleotide exchange	21.72	24.28	26.42	27.65	25.02	18.56	25.83	20.97	20.08	21.36	0.85	1	25.88	28.44	25.8	26.71	25.51	19.61	18.88	21.33	0.8	0.838837	30.49	31.75	30.45	30.9	24.79	27.26	26.21	26.09	0.84	1	41.69	30.07	31.52	34.43	25.47	25.47	25.47	0.74	1
Solyc01g097000.2.1	Beta-D-glucosidase (AHRD V1	9.85	8.94	4.81	12.53	9.03	10.18	13.51	6.77	10.64	10.27	1.14	0.919524	6.2	3.55	4.79	4.85	2.55	3.71	4.85	3.7	0.76	0.930441	0.08	1.3	0.3	0.56	0.26	0	0.73	0.33	0.59	1	0	0.07	0	0.02	0	0.09	0.04	1.93	1
Solyc01g097010.2.1	Nucleic acid binding protein (	139.19	130.33	141.77	147.66	139.74	117.25	140.08	104.82	140.14	125.57	0.9	1	123.24	135.65	141.14	133.34	142.05	121.05	117.65	126.92	0.95	1	113.33	114.31	111.72	113.12	123.82	114.7	110.68	116.4	1.03	1	260.78	186.13	188.12	211.68	162.09	172.1	167.09	0.79	1
Solyc01g097020.2.1	Unknown Protein (AHRD V1)	239.31	348.83	163.29	226.89	244.58	419.86	320.67	581.59	225.67	386.95	1.58	0.536383	182.89	191.16	145.31	173.12	102.28	116.54	184	134.27	0.78	0.784149	543.44	309.9	256.79	370.04	175.9	345.11	276.19	265.73	0.72	1	102.99	259.31	212.84	191.71	136.17	116.82	126.49	0.66	0.918278
Solyc01g097030.2.1	Mutator-like transposase (AH	6.28	7.2	11.26	9.73	8.62	3.92	2.78	3.88	3.77	3.59	0.42	0.002541	7.36	8.67	13.66	9.9	9.6	7.75	6.83	8.06	0.81	0.9964	5	10.51	9.82	8.44	10.11	7.63	8.15	8.63	1.02	1	20.54	12.43	15.34	16.1	17.16	16.06	16.61	1.03	1
Solyc01g097040.2.1	IFA-binding protein-like (AHR	5.79	5.6	5.29	6.04	5.68	3.86	3.6	4.39	3.74	3.9	0.69	0.462613	4.1	5.6	6.29	5.33	5.87	6.44	5.57	5.96	1.12	0.990055	4.03	3.6	2.51	3.38	3.47	2.36	1.84	2.56	0.76	1	4.94	4.26	6.56	5.25	2.45	3.3	2.88	0.55	0.813587
Solyc01g097050.2.1	Nucleic acid binding protein (	0.4	0	0	0	0.1	0	0	0.37	0	0.09	0.92	1	0	0	0	0	0.28	0	0	0.09	9.33	1	0	0	0	0	0	0	0	0	1	NA	0	0	0	0	0	0	0	1	NA
Solyc01g097060.2.1	Unknown Protein (AHRD V1)	0	0.54	0	0	0.14	0	0	1.67	0.43	0.53	3.89	1	0	0	0	0	0	0	0	0	1	NA	0	0.49	0	0.16	0.4	0.46	0.44	0.43	2.65	1	0	0	0	0	0	0	0	1	NA
Solyc01g097070.2.1	DNA topoisomerase (AHRD V	1.75	2.53	3.09	2.47	2.46	2.19	1.55	5.42	3.14	3.08	1.25	0.951897	2.03	0.76	1.85	1.55	0.91	0.68	0.17	0.59	0.38	0.632461	0.35	1.07	0.22	0.55	0.14	0.17	0	0.1	0.19	1	0.31	0.16	0.34	0.27	0.6	0	0.3	1.11	1
Solyc01g097080.1.1	Unknown Protein (AHRD V1)	0.25	0	0	0.35	0.15	0	0	0	0	0	0.07	1	0	0	0	0	0	0	0	0	1	NA	0	0	0	0	0	0	0	0	1	NA	0	0	0	0	0	0	0	1	NA
Solyc01g097090.1.1	Unknown Protein (AHRD V1)	0	0	0	0	0	0	0	0	0	0	1	NA	0	0	0	0	0	0	0	0	1	NA	0	0	0	0	0	0	0	0	1	NA	0	0	0	0	0	0	0	1	NA
Solyc01g097100.1.1	Unknown Protein (AHRD V1)	0	0	0.18	0	0.04	0	0	0	0.11	0.03	0.61	1	0	0	0	0	0	0	0	0	1	NA	0	0	0	0	0	0	0	0	1	1	0	0	0	0	0	0	0	1	NA
Solyc01g097110.1.1	Fanconi anemia group D2 pro	0.18	0.39	0	0.25	0.21	0.2	0.28	0	0.22	0.18	0.85	1	0.2	0.19	0.33	0.24	0.15	0.07	0.1	0.11	0.44	0.784149	0	0.07	0	0.02	0.03	0	0.1	0.04	1.86	1	0.06	0	0	0.02	0.08	0.08	0.08	4	1
Solyc01g097120.2.1	Os02g0200800 protein (Fragi	91.22	81.71	99.54	81.13	88.4	66.15	61.51	90.89	81.94	75.12	0.85	0.933538	85.58	82.19	78.54	82.1	64.09	67.4	62.92	64.8	0.79	0.797888	42.86	47.38	38.08	42.77	47.33	28.78	36.52	37.54	0.88	1	68.19	54.17	54.49	58.95	30.28	35.53	32.91	0.56	0.572547
Solyc01g097130.2.1	Pentatricopeptide repeat-con	8.01	10.01	10.57	11.33	9.98	8.27	8.81	9.9	10.47	9.36	0.94	1	10.73	10.28	11.03	10.68	13.89	13.08	12.06	13.01	1.22	0.740794	7.39	8.31	7.76	7.82	10.9	7.37	8.41	8.89	1.14	1	8.4	9.07	8.5	8.66	10.12	10.03	10.07	1.16	1
Solyc01g097140.2.1	Dual-specificity tyrosine-phos	85.32	82.11	59.11	53.32	69.97	64.51	82.24	105.35	79.77	82.97	1.19	0.804781	75.21	104.78	56.56	78.85	102.18	95.28	102.52	99.99	1.27	0.583654	59.71	89.33	50.71	66.58	58.65	57.9	72.29	62.95	0.95	1	64.18	69.53	65.6	66.44	56.76	63.06	59.91	0.9	1
Solyc01g097150.2.1	Unknown Protein (AHRD V1)	119.95	120.99	106.13	101.8	112.22	137.78	129.42	176.45	139.22	145.72	1.3	0.624879	137.61	152.93	126.64	139.06	158.07	169.75	150.82	159.55	1.15	0.937607	109.83	120.55	99.22	109.87	99.81	107.58	106.35	104.58	0.95	1	130.75	165.52	92.22	129.5	80.52	82.38	81.45	0.63	0.832328
Solyc01g097160.2.1	FAD dependent oxidoreducta	26.07	29.53	27.19	24.4	26.8	37.91	35.14	28.94	38.19	35.05	1.31	0.40381	23.15	32.16	32.14	29.15	42.92	34.09	34.67	37.23	1.28	0.55479	6.06	6.97	10.98	8	10.81	10.78	11.9	11.16	1.39	0.788174	7.86	5.93	4.45	6.08	10.85	9.93	10.39	1.71	0.76924
Solyc01g097170.2.1	Unknown Protein (AHRD V1)	49.09	42.99	43.09	46.66	45.46	38.41	28.21	31.41	36.93	33.74	0.74	0.603917	44.93	68.61	68.32	60.62	45.76	58.56	49.68	51.33	0.85	1	35.53	64	58.08	52.54	66.81	43.75	54.37	54.98	1.05	1	84.86	86.13	66.42	79.14	90.59	103.29	96.94	1.22	1
Solyc01g097180.2.1	Os11g0282300 protein (Fragi	0	0	0	0	0	0	0	0	0	0	1	NA	0	0	0	0	0	0.17	0	0.06	5.67	1	0	0	0	0	0.21	0	0	0.07	7	1	0	0	0	0	0.29	0	0.14	14.5	1
Solyc01g097190.2.1	Ankyrin repeat protein-like (A	58.17	46.72	46.03	37.93	47.21	32.64	29.68	35.22	35.75	33.32	0.71	0.305029	47.9	50.07	51.05	49.67	49.5	49.29	43.15	47.31	0.95	1	80.81	105.09	106.74	97.55	128.71	105.92	89.43	108.02	1.11	1	88.96	114.17	114.1	105.74	93.77	91.93	92.85	0.88	1
Solyc01g097200.1.1	Ankyrin repeat protein-like (A	1.11	1.97	1.96	0	1.26	0	0	0.51	0.4	0.23	0.18	0.62652	3.09	1.45	0.47	1.67	1.15	1.44	3.05	1.88	1.13	1	8.96	6.32	9.9	8.39	7.28	8.82	4.04	6.71	0.8	1	25.22	22.95	34.95	27.71	26.39	24.3	25.34	0.91	1
Solyc01g097210.2.1	Ankyrin-like protein (AHRD V	3.82	7.76	5.15	4.63	5.34	1.82	5.6	3.51	1.96	3.22	0.6	0.788057	5.07	4.74	2.3	4.04	3.78	2.82	5.57	4.06	1	1	11.45	11.11	17.32	13.29	11.82	12.4	7.15	10.46	0.79	1	50.39	34.06	45.27	43.24	20.96	17.69	19.33	0.45	0.456139
Solyc01g097220.1.1	Ankyrin repeat protein-like (A	0.63	1.26	2.04	2.45	1.6	0	0.37	0.43	0.45	0.31	0.2	0.083422	2.34	2.19	0.93	1.82	0.33	0.73	0.49	0.52	0.28	0.176517	1.27	0.38	1.72	1.12	0	0	0	0	0.01	0.635824	0	0	0	0	0.29	0	0.14	14.5	1
Solyc01g097230.2.1	PRA1 family protein E (AHRD	11.4	17.76	19.22	13.25	15.41	19.92	14.79	14.98	16.66	16.59	1.08	1	17.42	17.7	19.95	18.36	24.57	28.46	15.36	22.8	1.24	0.754244	16.78	31.86	14.15	20.93	23.27	19.14	34.42	25.61	1.22	1	34.74	36.38	39.45	36.86	45.48	57.13	51.3	1.39	1
Solyc01g097240.2.1	Pathogenesis-related protein	21.07	17.12	44.86	10.37	23.36	78.42	10.49	44.65	28.61	40.54	1.74	0.869554	55.4	54.56	35.21	48.39	143.62	61.2	31.68	78.83	1.63	0.941492	161.75	228.84	123.69	171.43	111.36	107.64	137.88	118.96	0.69	1	17.59	65.88	23.95	35.81	29.36	32.54	30.95	0.86	1
Solyc01g097250.2.1	Genomic DNA chromosome 5	32.02	25.56	33.65	25.52	29.19	28.04	26.04	24.62	33.71	28.1	0.96	1	25.63	28.67	26.88	27.06	28.93	21.9	29.89	26.91	0.99	1	27.59	30.83	23.69	27.37	27.15	30.74	30.68	29.52	1.08	1	22.62	15.22	12.53	16.79	20.43	17.52	18.98	1.13	1
Solyc01g097260.2.1	Unknown Protein (AHRD V1)	4.47	1.99	2.64	1.58	2.67	0	6.18	2.06	2.41	2.66	1	1	4.16	3.89	0	2.68	0.78	1.16	2.64	1.53	0.57	1	5.42	2.73	0	2.72	2.94	0.85	1.63	1.81	0.67	1	3.18	5.68	3.44	4.1	4.09	0.98	2.54	0.62	1
Solyc01g097270.2.1	Chitinase (Fragment) (AHRD V	12.08	7.99	10.6	10.95	10.41	14.12	10.03	7.4	6	9.39	0.9	1	8.64	2.96	5.49	5.7	4.08	3.53	1.46	3.02	0.53	0.813188	9.01	80.01	0.31	29.78	1.02	0.47	2.26	1.25	0.04	1	0	0.45	0	0.15	0.57	1.09	0.83	5.53	1
Solyc01g097280.2.1	Chitinase (Fragment) (AHRD V	0	0	0	0	0	0	0	0	0	0	1	NA	0.31	0	0	0.1	0	0	0	0	0.1	1	0	1.34	0	0.45	0	0	0	0	0.02	1	0	0	0	0	0	0	0	1	NA
Solyc01g097290.2.1	Auxin responsive protein (AH	105.5	115.8	131.88	74.06	106.81	197.01	129.59	222.65	130.62	169.97	1.59	0.196566	23.13	32.31	48.87	34.77	21.06	33.21	52.46	35.58	1.02	1	1.69	1.49	1.82	1.67	0.34	0	0.57	0.3	0.18	1	0.37	0.76	0.81	0.65	0	0.46	0.23	0.36	1
Solyc01g097300.2.1	Prefoldin subunit 4 (AHRD V1	119.67	122.43	101.36	109.23	113.17	97.78	113.45	148.58	122.26	120.52	1.06	1	108.93	100.91	92	100.61	84.77	91.62	73.95	83.45	0.83	0.982244	83.46	74.18	59.39	72.34	75.59	72.87	74.16	74.21	1.03	1	47.26	89.14	69.99	68.8	90.35	87.44	88.89	1.29	1
Solyc01g097310.2.1	Sec-independent protein tran	100.8	104.66	93.79	104.33	100.89	162.61	169.19	168.78	130.61	157.8	1.56	0.051991	85.38	76.58	69.56	77.17	103.16	93.56	118.82	105.18	1.36	0.3406	131.76	111.86	110.22	117.95	123.56	150.63	137.35	137.18	1.16	1	61.75	114.88	94.52	90.38	133.91	113.89	123.9	1.37	1
Solyc01g097320.2.1	Mediator of RNA polymerase	2.91	2.85	5.78	5.83	4.34	2.29	1.89	2.13	2.48	2.2	0.51	0.282302	3.53	4.35	6.09	4.66	5.64	4.12	4.4	4.72	1.01	1	3.45	3.36	6.21	4.34	2.88	2.9	3.46	3.08	0.71	1	9.47	5.03	6.51	7	6.47	7	6.73	0.96	1
Solyc01g097330.2.1	Opaque 2 (Fragment) (AHRD	19.57	17.81	20.87	26.25	21.12	18.05	19.23	17.06	14.99	17.33	0.82	0.924872	15.91	18.02	18.37	17.43	18.4	15.77	17.43	17.2	0.99	1	10.19	13.11	9.93	11.08	14.1	10.22	10.67	11.66	1.05	1	31.75	15.27	15.16	20.73	9.29	11.82	10.55	0.51	1
Solyc01g097340.2.1	NAD-dependent epimerase/d	100.79	112.44	126.35	111.38	112.74	212.8	126.68	162.5	182.92	171.22	1.52	0.057147	75.36	57.04	63.41	65.27	58.99	48.56	59.82	55.79	0.85	0.973115	68.28	31.94	37.69	45.97	37.59	41.91	33.03	37.51	0.82	1	1.74	8.77	4.57	5.03	43.57	25.78	34.67	6.9	0.033249
Solyc01g097350.2.1	Calcium-binding EF hand fam	89.91	111.52	103.7	134.28	109.85	54.78	58.12	91.72	68.59	68.3	0.62	0.101466	87.93	123.44	95.98	102.45	50.92	50.4	64.49	55.27	0.54	0.006473	50.54	40.65	55.7	48.96	53.78	45.12	21.84	40.25	0.82	1	58.05	94.52	108.25	86.94	96.48	104.58	100.53	1.16	1
Solyc01g097360.2.1	Unknown Protein (AHRD V1)	50.63	39.6	49.78	49.91	47.48	59.69	50.27	76.88	47.91	58.69	1.24	0.709275	46.38	36.01	53.78	45.39	45.76	48.06	42.2	45.34	1	1	44.62	41.26	45.69	43.86	30.24	34.3	38.57	34.37	0.78	1	65.44	46.8	62.52	58.25	46.35	63.22	54.78	0.94	1
Solyc01g097370.1.1	Ammonium transporter (AHR	0	0	0	0	0	0	0	0	0	0	1	NA	0	0	0	0	0	0	0	0	1	NA	0	0	0	0	0	0	0	0	1	NA	0	0	0	0	0	0	0	1	NA
Solyc01g097380.1.1	Aldo/keto reductase family p	3.36	0.88	1.86	2.24	2.08	2.2	2.81	3.27	1.84	2.53	1.21	0.98192	0.92	1.03	1.17	1.04	0.41	0.41	0.62	0.48	0.46	0.833891	0	0.48	0	0.16	0.13	0	0.14	0.09	0.56	1	0.84	0.14	1.82	0.93	0	0.35	0.17	0.19	1
Solyc01g097390.2.1	Aldo/keto reductase family p	12.81	11.23	9.02	11.63	11.17	11.27	13.74	16.35	11.67	13.26	1.19	0.830543	5.69	4.65	5.65	5.33	1.99	1.98	3.16	2.38	0.45	0.118054	0.31	0.78	0.76	0.62	0.25	0.29	0.56	0.37	0.59	1	0.27	0.14	0.29	0.23	0	0.17	0.09	0.36	1
Solyc01g097400.1.1	Calcium-transporting ATPase	0	0	0	0	0	0	0	0	0	0	1	NA	0	0	0	0	0	0	0	0	1	NA	0.19	0	0	0.06	0	0	0	0	0.16	1	0	0.17	0.37	0.18	0	0	0	0.06	1
Solyc01g097410.2.1	Unknown Protein (AHRD V1)	0	0	0	0	0	0	0	0	0	0	1	NA	0	0	0	0	0	0	0	0	1	NA	0	0	0	0	0	0	0	0	1	NA	0	0	0	0	0	0	0	1	NA
Solyc01g097420.1.1	Calcium ATPase (AHRD V1 **	0	0	0	0	0	0	0	0	0	0	1	NA	0	0	0	0	0	0	0	0	1	NA	0	0.49	0	0.16	0	0	0	0	0.06	1	0	0	0	0	0	0	0	1	NA
Solyc01g097430.2.1	ABC transporter G family mer	0	0	0	0	0	0	0	0	0	0	1	NA	0	0	0	0	0	0.04	0	0.01	1.33	1	0.13	0	0.08	0.07	0	0.12	0.06	0.06	0.86	1	0	0.23	0.36	0.2	0.22	0.07	0.15	0.74	1
Solyc01g097440.2.1	Peptide-N(4)-(N-acetyl-beta-g	20.17	21.59	17.15	12.98	17.97	17.78	16.28	23.39	20.48	19.48	1.08	1	17.92	20.06	16.73	18.24	17.33	17.46	22.85	19.21	1.05	1	17.55	16.51	15.51	16.52	14.45	14.26	16.09	14.93	0.9	1	12.53	14.98	12.44	13.32	17.39	17.13	17.26	1.3	1
Solyc01g097450.2.1	Thioredoxin-like protein 1 (AH	267.61	275.66	183.37	192.43	229.77	383.65	332.8	434.19	349.73	375.09	1.63	0.027676	259.67	195.05	135.37	196.7	171.91	198.19	212.94	194.35	0.99	1	248.43	241.15	148.72	212.77	148.62	209.89	203.43	187.31	0.88	1	72.6	154.84	126.09	117.84	179.47	133.66	156.56	1.33	1
Solyc01g097460.2.1	Ribose-5-phosphate isomeras	274.88	273.4	213.64	212.34	243.56	472.67	414.18	472.25	423.68	445.69	1.83	0.001853	259.65	206.96	169.06	211.89	249.79	259.9	264.16	257.95	1.22	0.580074	217.73	216.86	162.37	198.99	210.5	233.85	223.69	222.68	1.12	1	87.1	149.45	113.52	116.69	231.5	177.66	204.58	1.75	0.608977
Solyc01g097470.2.1	Neurogenic locus notch prote	27.94	43.59	9.52	26.08	26.78	39.39	37.75	44.5	55.64	44.32	1.65	0.23723	79.32	63.86	46.96	63.38	27.37	29.98	29.21	28.85	0.46	0.021889	0.47	0.94	1.72	1.04	0.76	1.75	2.52	1.68	1.61	1	0	0.84	0	0.28	3.69	2.27	2.98	10.64	0.44705
Solyc01g097480.1.1	Unknown Protein (AHRD V1)	0.48	0	0	0	0.12	0	0	0	0	0	0.08	1	0	0	0.4	0.13	0.33	0	0	0.11	0.83	1	0	0	0.47	0.16	0	0	0	0	0.06	NA	0	0	0	0	0	0	0	1	NA
Solyc01g097490.1.1	IQ calmodulin-binding motif f	0.25	0.45	0	0	0.17	0	0.2	0.7	0	0.22	1.29	1	0	0.22	0.43	0.22	0.18	0.13	0	0.1	0.48	1	0	0	0	0	0	0	0	0	1	1	0	0.37	0	0.12	0	0.22	0.11	0.89	1
Solyc01g097500.2.1	Serine/threonine kinase (AHR	41.53	39.94	51.04	63.03	48.88	36.79	55.84	42.06	36.1	42.7	0.87	1	30.48	37.13	36.9	34.84	21.53	23.69	32.55	25.92	0.74	0.513115	54.08	37.28	43.15	44.84	27.57	47.79	34.05	36.47	0.81	1	28.29	32.68	28.44	29.8	45.94	44.19	45.06	1.51	0.854433
Solyc01g097510.2.1	Annexin 3 (AHRD V1 ***- A9)	192.12	192.52	248.14	113.21	186.5	120.23	89.47	234.45	195.98	160.03	0.86	1	112.53	78.2	55.55	82.09	40.31	31.69	69.23	47.08	0.57	0.541116	7.35	11.46	7.18	8.66	7.06	3.92	6.93	5.97	0.69	1	2.65	2.7	1.59	2.31	1.51	4.35	2.93	1.27	1
Solyc01g097520.2.1	Annexin 11 (AHRD V1 ***- D6	823.7	660.3	970.32	583.15	759.37	292.87	385.28	767.1	724.78	542.51	0.71	0.738841	583.45	412.83	260.5	418.93	176.24	104.52	286.37	189.04	0.45	0.294864	19.67	63.76	21.9	35.11	45.94	19.4	20.62	28.65	0.82	1	13.02	15.12	16	14.71	15.24	18.79	17.02	1.16	1
Solyc01g097530.1.1	Photosystem Q(B) protein (AH	1.22	1.09	0.72	2.59	1.41	0.68	1.45	2.25	2.63	1.75	1.25	1	1.14	1.06	2.58	1.59	0	0.95	0.96	0.64	0.4	0.974398	1.97	1	0.61	1.19	0.8	0.46	0.45	0.57	0.48	1	1.74	1.33	0	1.02	0.56	0.54	0.55	0.54	1
Solyc01g097540.2.1	Annexin 3 (AHRD V1 ***- A9)	14.06	24.11	14.88	17.26	17.58	15.63	12.57	17.11	18.11	15.86	0.9	1	18.47	19.06	16.24	17.92	12.91	19.17	19.83	17.3	0.97	1	44.61	39.43	37.15	40.4	29.48	49.53	42.8	40.6	1.01	1	40.28	54.67	42.93	45.96	47.71	46.24	46.98	1.02	1
Solyc01g097550.2.1	Annexin 11 (AHRD V1 ***- D6	0	0.19	0	0	0.05	0.23	0	0	0	0.06	1.21	1	0	0.18	0	0.06	0	0	0	0	0.17	1	0	0.17	0	0.06	0	0	0	0	0.18	1	0	0	0	0	0	0	0	1	NA
Solyc01g097560.1.1	Unknown Protein (AHRD V1)	0	0	0	0.8	0.2	0	0.89	0	0.81	0.43	2.12	1	0	0	0	0	0.39	0.88	0.44	0.57	57	0.923054	0.46	0	0	0.15	0	0	0	0	0.07	1	1.6	0	0	0.53	0	0	0	0.02	1
Solyc01g097570.2.1	Unknown Protein (AHRD V1)	1.04	0.92	0.61	0	0.64	1.73	1.23	2.87	1.12	1.74	2.7	0.817178	1.45	0	0.44	0.63	0.36	0.54	0.41	0.44	0.69	1	1.26	0.85	0.52	0.88	0	0.39	0	0.13	0.15	1	0	1.89	1.6	1.16	0	0	0	0.01	1
Solyc01g097580.1.1	Unknown Protein (AHRD V1)	0	0	0	0	0	0	0	0	0	0	1	NA	0	0	0	0	0	0	0	0	1	NA	0	0	0	0	0	0	0	0	1	NA	0	0	0	0	0	0	0	1	NA
Solyc01g097590.2.1	Pseudouridine synthase (AHR	11.95	12.85	11.75	7.04	10.9	9.69	9.43	9.16	9.83	9.53	0.87	1	11.35	8.44	12.41	10.73	5.53	7.86	7.04	6.81	0.63	0.556835	5.23	5.07	7.41	5.9	1.31	1.7	4.72	2.58	0.44	1	4.96	3.07	4.98	4.34	1.6	2.84	2.22	0.51	1
Solyc01g097600.1.1	tRNA pseudouridine synthase	5.2	6.68	5.6	4.55	5.51	6.3	6.28	5.63	7.36	6.39	1.16	0.943718	6.26	4.92	3.75	4.98	2.02	4.44	6.38	4.28	0.86	1	2.6	2.49	3.95	3.01	1.61	1.04	2.57	1.74	0.58	1	0.44	2	2.35	1.6	1.4	1.21	1.3	0.82	1
Solyc01g097610.2.1	F-box family protein (AHRD V	15.17	17.24	17.46	12.67	15.63	19.91	11.25	14.45	15.09	15.18	0.97	1	16.83	16.68	15.13	16.21	19.14	20.72	17.52	19.13	1.18	0.832635	23.33	22.93	17.05	21.1	18.25	19.67	22.77	20.23	0.96	1	12.47	14.2	8.71	11.79	12.21	11.38	11.8	1	1
Solyc01g097620.1.1	Unknown Protein (AHRD V1)	0	0	0	0	0	0	0	0	0	0	1	NA	0	0	0	0	0	0	0	0	1	NA	0	0	0	0	0	0	0	0	1	NA	0	0	0	0	0	0	0	1	NA
Solyc01g097630.1.1	BAC19.2 (AHRD V1 ***- Q9FY	0.45	0.4	0	0.63	0.37	0	0	0	0.48	0.12	0.32	0.994382	0.42	0	0	0.14	0.78	0.58	0.18	0.51	3.67	0.835418	0.54	0.37	0.45	0.45	0.15	0.34	0.65	0.38	0.84	1	0.64	1.47	0.35	0.82	0.41	0.79	0.6	0.73	1
Solyc01g097640.2.1	Ribosome biogenesis protein	9.59	8.53	11.75	9.01	9.72	6.07	8.96	9.26	9.42	8.43	0.87	1	10.06	7.59	10.45	9.37	8.47	9.87	10.98	9.77	1.04	1	5.32	6.07	5.53	5.64	8.13	5.15	6.24	6.51	1.15	1	13.78	9.49	13.67	12.31	11.18	14.46	12.82	1.04	1
Solyc01g097650.2.1	Serine incorporator 1 (AHRD V	0.81	1.15	0.96	0.69	0.9	0.54	0.64	1.79	1.28	1.06	1.18	1	1.66	1.27	1.1	1.34	0.34	0.84	0.38	0.52	0.39	0.604902	2.88	3.43	1.45	2.59	1.6	2.46	3.19	2.42	0.93	1	0.46	0.82	0.5	0.59	0.45	2.42	1.44	2.42	1
Solyc01g097660.2.1	Pectinesterase inhibitor (AHR	0	0	0	0	0	0	0	0	0	0	1	NA	0	0	0	0	0	0	0	0	1	NA	0	0	0	0	0	0	0	0	1	NA	0	0	0	0	0	0	0	1	NA
Solyc01g097670.2.1	Pentatricopeptide repeat-con	1.8	0.9	1.47	0.99	1.29	1.39	1.79	1.22	1.18	1.4	1.08	1	1.38	1.09	1.38	1.28	1.68	1.82	2.27	1.92	1.5	0.673688	0.95	1.59	1.08	1.21	1.03	0.89	1.54	1.15	0.96	1	1.67	2.44	3	2.37	4.57	2.46	3.52	1.48	1
Solyc01g097680.2.1	Extensin-like protein Dif10 (F	0.43	0	0.76	0.3	0.37	0	0.51	0	0	0.13	0.34	0.992609	0.6	0.75	0.36	0.57	0	0.45	0.17	0.21	0.36	0.974398	0	0.18	0.21	0.13	0.14	0	0.31	0.15	1.15	1	0	0	0	0	0	0.19	0.1	9.5	1
Solyc01g097690.2.1	Extensin-like protein Dif54 (A	1.24	0.18	0.73	0.88	0.76	0	0.98	0	0.15	0.28	0.37	0.832993	1.54	0.54	0.7	0.93	0.29	0.54	0.49	0.44	0.47	0.943066	0	0.17	0.21	0.13	0.41	0.16	0.3	0.29	2.29	1	0	0	0	0	0.38	0.18	0.28	28	1
Solyc01g097700.1.1	Extensin-like protein Dif10 (F	0.21	0	0	0	0.05	0	0.33	0	0.15	0.12	2.29	1	0.38	0	0	0.13	0.14	0.11	0	0.08	0.66	1	0	0	0	0	0.14	0.16	0.3	0.2	20	1	0	0	0	0	0	0	0	1	NA
Solyc01g097710.1.1	Extensin-like protein Dif10 (F	0.18	0.16	0.43	0.77	0.39	0.4	1.57	0.67	0.52	0.79	2.05	0.688274	1.01	0.79	1.07	0.96	0.38	0.66	1.57	0.87	0.91	1	0	0.15	0.18	0.11	0.12	0	0.13	0.08	0.76	1	0	0	0	0	0	0.16	0.08	8	1
Solyc01g097720.1.1	Extensin-like protein Dif10 (F	0.91	0.16	1.07	0.51	0.66	0.61	0.86	0.33	1.17	0.74	1.12	1	1.01	1.26	0.61	0.96	0.76	1.04	1	0.93	0.97	1	0	0.3	0.18	0.16	0.48	0.28	0.53	0.43	2.69	1	0	0	0	0	0.33	0.16	0.24	24.5	1

Solyc01g097730.2.1	Pentatricopeptide repeat-con	6.02	6.52	7.1	6.11	6.44	7.86	6.41	8.79	5.26	7.08	1.1	1	6.7	6.37	6.41	6.49	6.9	6.78	8.33	7.34	1.13	1	2.64	3.2	2.6	2.81	3.61	1.88	3.15	2.88	1.02	1	4.47	3.23	6.24	4.65	4.43	6.08	5.25	1.13	1
Solyc01g097740.2.1	Tetratricopeptide repeat prot	55.23	56.76	47.06	62.62	55.42	48.73	75.1	47.76	45.95	54.38	0.98	1	51.17	46.11	39.62	45.63	26.91	37.22	36.34	33.49	0.73	0.624496	50.82	50.9	44.16	48.63	48.38	41.36	44.56	44.77	0.92	1	62.01	84.05	84.26	76.77	52.16	51.5	51.83	0.68	0.852617
Solyc01g097750.1.1	Unknown Protein (AHRD V1)	0	0	0	0	0	0	0	0	0	0	1	NA	0	0	0	0	0	0	0	0	1	NA	0	0	0	0	0	0	0	0	1	NA	0	0	0	0	0	0	0	1	NA
Solyc01g097760.2.1	Ribosomal protein L7a (AHRD	646.72	542.03	607.03	612.14	601.98	528.05	536.71	467.69	559.75	523.05	0.87	0.990049	556.31	539.82	521.28	539.14	465.33	504.19	502.83	490.78	0.91	1	309.09	360.74	324.63	331.49	412.97	349.05	401.11	387.71	1.17	1	264.37	287.49	307.08	286.31	412.71	440.63	426.67	1.49	0.80103
Solyc01g097770.2.1	Serine/threonine protein kina	101.85	118.1	128.15	108.15	114.06	170.14	125.19	175.88	168.74	159.99	1.4	0.153936	117.02	110.08	130.33	119.14	131.81	136.59	126.95	131.78	1.11	0.919014	0.22	2.02	1.16	1.13	0.95	0.84	2.21	1.33	1.18	1	0.49	0.7	0.11	0.43	0.63	1.21	0.92	2.12	1
Solyc01g097780.1.1	Inositol monophosphatase fa	0	0	0	0	0	0	0	0	0	0	1	NA	0	0	0	0	0	0	0	0	1	NA	0	0	0	0	0	0	0	0	1	NA	0	0	0	0	0	0	0	1	NA
Solyc01g097790.2.1	AT5G28150-like protein (Frag	18.69	23.29	21.53	19.6	20.78	23.47	15.62	30.33	17.68	21.77	1.05	1	20.66	23.13	19.32	21.04	21.51	24.25	22.98	22.91	1.09	1	21.5	22.76	17.46	20.57	19.79	23	29.97	24.25	1.18	1	12.51	14.21	13.53	13.42	16.31	19.67	17.99	1.34	1
Solyc01g097800.2.1	Galactose-6-phosphate isome	28.03	26.26	34.07	32.5	30.22	29.75	37.27	25.58	35.56	32.04	1.06	0.944449	36.27	40.12	34.71	37.03	42.83	41.69	37.77	40.76	1.1	0.972234	25.03	45.34	23.22	31.2	27.42	30.51	39.47	32.47	1.04	1	40.46	31.38	29.82	33.89	43.41	45.83	44.62	1.32	1
Solyc01g097810.2.1	Zeta-carotene desaturase	39.76	42.17	41.76	35.49	39.8	53.79	44.65	42.63	55.32	49.1	1.23	0.553086	35.84	35.93	32.92	34.9	49.17	45.35	45.51	46.68	1.34	0.324188	50.22	43.99	67.15	53.79	56.8	65.45	62.36	61.54	1.14	1	39.81	70.04	55.12	54.99	85	65.21	75.1	1.37	1
Solyc01g097820.2.1	SOUL heme-binding protein (	2.1	6.32	5.58	2.97	4.24	7.89	4.77	10.63	5.28	7.14	1.68	0.500333	4.4	3.43	3.33	3.72	3.65	3.4	3.51	3.52	0.95	1	1.49	2.14	0.78	1.47	2.42	0.4	1.72	1.51	1.03	1	1.49	2.86	1.62	1.99	2.65	2.53	2.59	1.3	1
Solyc01g097830.2.1	AT5G28150-like protein (Frag	0	0.17	0	0.27	0.11	0	0.3	0	0.14	0.11	1	1	0.18	0.83	0.48	0.5	1.98	1.48	0.3	1.25	2.52	0.704699	1.08	3.41	0.57	1.69	1.62	0.14	2.77	1.51	0.9	1	0	0.55	0.29	0.28	1.74	6	3.87	13.82	0.391746
Solyc01g097840.2.1	Serine/threonine protein kina	4.58	5.64	7.48	13.95	7.91	5.87	8.55	6.72	5.19	6.58	0.83	1	4.34	5.59	8.03	5.99	4.22	5.61	4.57	4.8	0.8	0.967182	9.25	13.78	9.44	10.82	9.32	7.74	8.15	8.4	0.78	1	14.15	14.84	21.94	16.98	10.88	11.58	11.23	0.66	0.961777
Solyc01g097850.1.1	MADS-box transcription facto	0	0	0	0	0	0	0	0	0	0	1	NA	0	0	0	0	0	0	0	0	1	NA	0	0	0	0	0	0	0	0	1	NA	0	0	0	0	0	0	0	1	NA
Solyc01g097860.2.1	Membrane protein (AHRD V1	52.5	60.75	52.52	44.56	52.58	95.92	79.48	80.6	97.51	88.38	1.68	0.018709	69.43	64.69	70.76	68.29	186.98	123.75	121.63	144.12	2.11	0.017549	9.59	16.93	8.61	11.71	11.1	5.71	14.97	10.59	0.9	1	5.19	6.3	8.07	6.52	12.96	27.42	20.19	3.1	0.222244
Solyc01g097870.2.1	40S ribosomal protein S24 (A	895.04	937.67	989.16	1099.59	980.37	1121.32	881.52	1243.45	896.7	1035.75	1.06	0.996377	803.24	764.34	773.02	780.2	742.34	811.37	977.7	843.8	1.08	1	218.76	264.78	247.76	243.77	314.69	233.22	357.68	301.86	1.24	1	167.3	188.08	205.92	187.1	250.58	378.57	314.57	1.68	0.541065
Solyc01g097880.2.1	Deaminase (AHRD V1 *-*- D6	131.59	156.68	129.03	125.25	135.64	148.23	174.91	155.7	176.03	163.72	1.21	0.613591	147.67	160.08	117.1	141.62	154.15	134.99	130.86	140	0.99	1	2539.52	1491.13	1544.61	1858.42	1249.63	1485.61	1679.5	1471.58	0.79	1	170.04	357.95	263.65	263.88	400.13	220.54	310.33	1.18	1
Solyc01g097890.2.1	Os03g0370250 protein (Fragi	18.31	13.45	23.83	29.39	21.24	11.31	16.98	11.36	12	12.91	0.61	0.476728	15.14	21.6	27.49	21.41	23.18	18.13	14.85	18.72	0.87	1	9.98	20.13	15	15.04	15.08	9.47	12.92	12.49	0.83	1	51.65	19.84	29.27	33.59	23.54	28.03	25.79	0.77	1
Solyc01g097900.1.1	Unknown Protein (AHRD V1)	0	0.51	0	0	0.13	0	0	0	0	0	0.08	1	0	0	0	0	0	0	0	0	1	NA	0	0	0	0	0	0	0	0	1	NA	0	0	0	0	0	0	0	1	NA
Solyc01g097910.2.1	Rubredoxin family protein (A	82.22	78.57	74.82	51.39	71.75	143.83	119.77	116.04	137.74	129.35	1.8	0.005729	57.51	83.94	65.68	69.04	173.3	163.36	129.24	155.3	2.25	1.5E-05	57.93	53.24	25.31	45.49	68.88	50.73	65.03	61.55	1.35	1	46.22	36.8	19.6	34.21	35.3	35.21	35.25	1.03	1
Solyc01g097920.2.1	Cysteine synthase (AHRD V1	1.85	0.66	1.53	2.61	1.66	1.23	2.48	1.02	0.13	1.22	0.73	1	1.2	0.48	1.25	0.98	1.67	2.01	2.32	2	2.05	0.561283	0.6	1.96	0.37	0.98	0.24	0.42	1.21	0.62	0.64	1	1.31	0.27	1.42	1	0.68	0.16	0.42	0.42	1
Solyc01g097930.2.1	Cysteine synthase (AHRD V1	0.41	0.18	0.24	0.86	0.42	0	0.48	0	0.15	0.16	0.37	0.997392	0	0	0	0	0	0.42	0.32	0.25	24.67	0.837384	0	0	0	0	0	0.15	0	0.05	5	1	0	0	0	0	0	0.18	0.09	9	1
Solyc01g097940.1.1	Cysteine synthase (AHRD V1	0.33	0	0	0	0.08	0	0	0	0.24	0.06	0.73	1	0	0	0	0	0	0	0	0	1	NA	0	0	0	0	0	0	0	0	1	NA	0	0	0	0	0	0	0	1	NA
Solyc01g097950.2.1	Cysteine synthase (AHRD V1	13.85	11.78	10.57	17.28	13.37	5.88	15.11	9.17	10.52	10.17	0.76	0.923608	8.33	8.14	7.74	8.07	3.11	7.38	6.08	5.52	0.68	0.779245	0.16	0.66	0.4	0.41	0.13	0	0.15	0.09	0.23	1	0.29	0	0	0.1	0.19	0.36	0.28	2.84	1
Solyc01g097960.2.1	Late embryogenesis abundan	0	0	0	0	0	0	0	0	0	0	1	NA	0	0	0	0	0	0	0	0	1	NA	0	0	0	0	0	0	0	0	1	NA	0	0	0	0	0	0	0	1	NA
Solyc01g097970.2.1	Deoxyuridine 5&apos-triphos	9.9	5.45	7.78	9.33	8.12	9.95	14.14	10.83	8.46	10.85	1.34	0.72699	7.01	7.78	3.98	6.26	4.58	1.95	1.85	2.79	0.45	0.425424	1.14	4.99	3.27	3.13	1.55	1.79	2.75	2.03	0.65	1	4.02	1.37	1.45	2.28	7.33	7.02	7.17	3.15	0.578724
Solyc01g097980.2.1	Kinase family protein (AHRD	15.37	16.19	17.58	19.56	17.18	14.7	11.87	13.76	15.61	13.98	0.81	0.863584	17.35	24.09	25.85	22.43	23.13	23.49	21.1	22.57	1.01	1	16.48	22.64	26.85	21.99	19.93	22.18	17.65	19.92	0.91	1	34.92	28.13	29.41	30.82	32.31	30.54	31.43	1.02	1
Solyc01g097990.2.1	Acetyltransferase GNAT famil	7	7.12	3.54	11.31	7.24	6.67	12.63	3.68	7.89	7.72	1.07	1	12.09	5.21	6.75	8.02	6.24	5.69	2.36	4.76	0.59	0.964678	2.42	4.07	1.98	2.82	0.66	0	0.73	0.46	0.16	1	0	0	0	0	0	0	0	1	NA
Solyc01g098000.2.1	Elongation factor-like protein	95.41	83.7	84.59	77.89	85.4	82.73	75.33	89.98	86.9	83.74	0.98	1	80.38	56.16	52.96	63.17	39.89	44.04	61.27	48.4	0.77	0.825508	36.12	41.76	30.58	36.15	36.18	32.03	40.92	36.38	1.01	1	18.22	19.21	18.03	18.49	43.67	36.57	40.12	2.17	0.24932
Solyc01g098010.2.1	Acetyltransferase GNAT famil	4.6	1.02	1.36	2.71	2.42	1.28	3.94	3.17	3.58	2.99	1.24	0.986666	1.07	2	0.65	1.24	2.13	2.38	3.01	2.51	2.02	0.790445	0	0	0	0	0.25	0	0	0.08	8.33	1	0	0	0	0	0	0.34	0.17	17	1
Solyc01g098020.2.1	Acetyltransferase GNAT famil	13.83	16.17	9.79	8.94	12.18	24.59	14.03	19.25	17.87	18.93	1.55	0.417151	11.02	13.74	8.01	10.92	17.54	12.27	15.83	15.21	1.39	0.695849	1.91	0.32	0.78	1	1.3	0.6	0.86	0.92	0.92	1	1.12	2.87	0	1.33	0.72	2.77	1.75	1.31	1
Solyc01g098030.2.1	Contains similarity to RNA-bi	37.86	36.17	56.65	55.91	46.65	25.99	36.59	23.42	37.77	30.94	0.66	0.473689	39.49	44.64	56.66	46.93	67.77	44.68	43.2	51.88	1.11	1	30.92	38.79	42.14	37.28	38.77	41.46	33.67	37.97	1.02	1	65.5	64.59	75.55	68.55	71.73	81.88	76.81	1.12	1
Solyc01g098040.2.1	Methyltransferase type 12 (A	50.19	46.29	55.87	79.86	58.05	42.06	42.34	34.24	41.55	40.05	0.69	0.479329	49.13	49.61	57.43	52.06	54.32	42.96	46.76	48.01	0.92	1	20.27	39.74	24.51	28.17	33.77	26.38	32.74	30.96	1.1	1	68.77	32.63	35.48	45.63	40.57	58.2	49.39	1.08	1
Solyc01g098050.1.1	MADS-box transcription facto	0	0	0	0	0	0	0	0	0	0	1	NA	0	0	0	0	0	0	0	0	1	NA	0	0	0	0	0	0	0	0	1	NA	0	0	0	0	0	0	0	1	NA
Solyc01g098060.1.1	MADS-box transcription facto	0	0	0	0	0	0	0	0	0	0	1	NA	0	0	0	0	0	0	0	0	1	NA	0	0	0	0	0	0	0	0	1	NA	0	0	0	0	0	0	0	1	NA
Solyc01g098070.1.1	MADS-box family protein (AH	0	0	0	0	0	0	0	0	0	0	1	NA	0	0	0	0	0	0	0	0	1	NA	0	0	0	0	0	0	0	0	1	NA	0	0	0	0	0	0	0	1	NA
Solyc01g098080.2.1	BY-inesin-like protein 5 (AHR	6.37	6.04	7.64	9.15	7.3	8.35	10.88	9.31	7.87	9.1	1.25	0.564428	5.05	4.81	6.61	5.49	6.23	6.32	7.71	6.75	1.23	0.857418	4.47	4.42	1.66	3.52	3.5	1.66	3.35	2.84	0.81	1	1.78	2.2	1.28	1.75	1.62	0.92	1.27	0.72	1
Solyc01g098090.2.1	WD repeat-containing protein	21.74	22.49	33.54	31.41	27.29	18.28	20.91	17.94	23.68	20.2	0.74	0.650008	28.18	39.01	44.5	37.23	52.14	42.29	32.86	42.43	1.14	0.961157	29.95	28.24	26.29	28.16	27.47	24.16	23.77	25.13	0.89	1	84.55	58.65	51.72	64.97	43.55	56.62	50.08	0.77	1
Solyc01g098100.2.1	60S ribosome subunit biogen	51.58	40.91	29.39	33.75	38.91	20.67	29.09	38.72	28.97	29.36	0.75	0.623604	47.98	38.74	30.23	38.98	21.9	27.44	37.5	28.95	0.74	0.77615	30.05	27.45	19.51	25.67	25.36	20.48	25.06	23.63	0.92	1	15.97	29.05	25.9	23.64	16.06	17.54	16.8	0.71	1
Solyc01g098110.2.1	Hydrolase alpha/beta fold far	6.22	6.92	9.17	4.95	6.82	30.46	16.49	24.39	20.44	22.95	3.37	1.72E-05	8.68	5.66	5.17	6.5	8.56	10.61	15.04	11.4	1.75	0.2002	141.45	74.66	110.91	109.01	79.78	113.63	107.9	100.44	0.92	1	10.36	61.11	26.15	32.54	157.96	47.36	102.66	3.15	1
Solyc01g098120.2.1	Unknown Protein (AHRD V1)	3.63	5.01	4.07	2.06	3.69	6.26	4.59	2.34	5.09	4.57	1.24	0.96065	3.72	1.11	1.54	2.12	0.63	0.94	1.71	1.09	0.51	0.882396	14.24	6.96	5.95	9.05	4.89	7.58	3.18	5.22	0.58	1	9.82	5.94	2.52	6.09	2.83	0.48	1.66	0.27	0.808314
Solyc01g098130.2.1	Unknown Protein (AHRD V1)	0	0.83	0	0	0.21	0	0	0	0	0	0.05	1	0	0	0	0	0	0	0	0	1	NA	0	0	0	0	0	0	0	0	1	NA	0	0	0	0	0	0	0	1	NA
Solyc01g098140.2.1	Neutral cholesterol ester hyd	0	0.09	0	0	0.02	0	0	0	0.07	0.02	0.78	1	0.09	0.18	0	0.09	0.07	0.11	0.32	0.17	1.85	1	6.24	1.91	1.31	3.15	0.73	1.62	3.04	1.8	0.57	1	0	0.07	0	0.02	0	0	0	0.43	1
Solyc01g098150.2.1	Pentatricopeptide repeat-con	3	3.9	2.3	2.98	3.04	5.14	4.16	4.85	5.07	4.8	1.58	0.258674	4	4.94	3.5	4.15	4.78	5.8	5.93	5.5	1.33	0.613814	4.65	3.97	4.91	4.51	4.64	4.37	5.62	4.88	1.08	1	1.27	2.95	2.12	2.11	2.75	2.7	2.73	1.29	1
Solyc01g098160.2.1	Cell division protein kinase 12	39.4	30.27	33.65	36.61	34.98	23.3	37.25	30.22	37.58	32.09	0.92	1	32.49	34.35	34.25	33.7	24.94	26.69	28.71	26.78	0.79	0.809015	43.86	37.05	36.95	39.29	42.51	40.02	40.73	41.09	1.05	1	53.58	53.65	55.48	54.24	44.45	41.98	43.22	0.8	1
Solyc01g098170.2.1	Exportin 5 (Fragment) (AHRD	6.71	10.08	12.45	11.9	10.29	8.13	7.94	9.1	8.76	8.48	0.82	0.93597	9.92	9.32	10.54	9.93	9.65	8.77	9.94	9.45	0.95	1	7.11	6.41	7.86	7.13	5.74	7.21	5.57	6.17	0.87	1	9.85	12.06	14.71	12.21	9.01	11.28	10.14	0.83	1
Solyc01g098180.2.1	Translation initiation factor e	21.96	20.27	21.56	20.11	20.98	17.71	14.5	17.96	21.52	17.92	0.85	0.963119	23.44	23.63	27.52	24.86	28.28	24	22.99	25.09	1.01	1	21.38	23.47	22.96	22.6	19.57	20.74	18.76	19.69	0.87	1	24.19	30.59	40	31.59	26.12	32.75	29.44	0.93	1
Solyc01g098190.2.1	Sodium/hydrogen exchanger	126.54	143.01	177.84	124.42	142.95	299.2	185.54	216.76	251.1	238.15	1.67	0.018021	81.82	55.75	57.17	64.91	46.1	61.95	67.58	58.54	0.9	1	318.4	231.75	200.1	250.08	123.48	147.3	185.49	152.09	0.61	1	7.51	21.86	20.21	16.53	45.55	40.61	43.08	2.61	0.063424
Solyc01g098200.2.1	Vesicle-associated membrane	42.04	44.08	35.85	27.67	37.41	29.15	38.45	40.18	49.45	39.31	1.05	1	31.75	23.16	26.72	27.21	27.71	34.7	32.06	31.49	1.16	0.841123	24.72	23.23	15.49	21.15	27.75	24.61	30.33	27.56	1.3	1	17.46	19.8	21.76	19.67	28.96	29.74	29.35	1.49	0.909785
Solyc01g098210.2.1	Transmembrane protein adip	10.96	17.17	6.46	6.82	10.35	11.21	8.65	12.49	13.53	11.47	1.11	1	14.88	18.29	13.33	15.5	12.16	13.28	14.99	13.48	0.87	1	20.36	17.68	12.4	16.81	14.54	19.1	18.85	17.5	1.04	1	9.66	10.51	13.14	11.1	10.23	9.61	9.92	0.89	1
Solyc01g098220.1.1	LOB domain protein 20 (AHR	0	0	0	0	0	0	0	0	0	0	1	NA	0	0	0	0	0	0	0	0	1	NA	0	0	0	0	0	0	0	0	1	NA	0	0	0	0	0	0	0	1	NA
Solyc01g098230.2.1	Mitochondrial import inner m	19.57	21.3	20.59	16.64	19.53	19.25	18.89	24.19	19.27	20.4	1.04	1	21.03	24.02	21.55	22.2	17.42	24.3	19.43	20.38	0.92	1	21.68	23.27	22.05	22.33	29.53	21.64	23.7	24.96	1.12	1	32.89	33.61	35.58	34.03	29.53	28.17	28.85	0.85	1
Solyc01g098240.1.1	RNA polymerase Rpb1 C-term	0	0	0.05	0.24	0.07	0	0.03	0.04	0	0.02	0.24	1	0	0.08	0	0.03	0.03	0.04	0	0.02	0.88	1	0	0	0	0	0.06	0	0	0.02	2	1	0	0.03	0.2	0.08	0	0.04	0.02	0.26	1
Solyc01g098250.2.1	Chaperone protein dnaJ 49 (A	11.28	10.16	12.68	15.02	12.29	6.42	6.5	7.81	9.67	7.6	0.62	0.203886	11.61	12.63	16.99	13.74	13.81	12.71	12.83	13.12	0.95	1	10.46	11.09	12.65	11.4	13.91	11.92	11.51	12.45	1.09	1	21.89	19.14	20.82	20.62	14.03	17.4	15.71	0.76	1
Solyc01g098260.1.1	Unknown Protein (AHRD V1)	0	0	0	0	0	0	0	0	0	0	1	NA	0	0	0	0	0	0	0	0	1	NA	0	0	0	0	0	0	0	0	1	NA	0	0	0	0	0	0	0	1	NA
Solyc01g098270.1.1	Unknown Protein (AHRD V1)	0	0	0	0	0	0	0	0	0	0	1	NA	0	0	0	0	0.13	0	0	0.04	4.33	1	0	0	0.19	0.06	0.37	0.29	0.14	0.27	4.21	1	0	0.41	0	0.14	0.17	0.17	0.17	1.24	1
Solyc01g098280.2.1	Heat shock protein DnaJ (AHF	0.2	0	0	0	0.05	0	0	0	0	0	0.2	1	0	0	0	0	0	0	0	0	1	NA	0	0	0	0	0	0	0	0	1	NA	0	0	0.3	0.1	0	0	0	0.1	1
Solyc01g098290.1.1	Unknown Protein (AHRD V1)	0	0	0	0	0	0	0	0	0	0	1	NA	0	0	0	0	0	0	0	0	1	NA	0	0	0	0	0	0	0	0	1	NA	0	0	0	0	0	0	0	1	NA
Solyc01g098300.1.1	Heat shock protein DnaJ N-te	0	0	0	0	0	0	0	0	0	0	1	NA	0	0	0	0	0	0	0	0	1	NA	0	0	0	0	0.11	0.12	0	0.08	7.67	1	0	0.23	0.25	0.16	0	0	0	0.06	1
Solyc01g098310.1.1	DNAJ heat shock N-terminal d	0	0	0	0	0	0	0	0	0	0	1	NA	0	0	0	0	0	0	0	0	1	NA	0	0	0	0	0	0	0	0	1	NA	0	0	0	0	0	0	0	1	NA
Solyc01g098320.2.1	DNA-directed RNA polymeras	35.88	26.52	28.33	30.7	30.36	26.7	33.81	36.98	26.59	31.02	1.02	1	36.07	31.44	28.35	31.95	28.1	30.41	37.97	32.16	1.01	1	20.1	24.29	15.51	19.97	13.12	16.67	16.02	15.27	0.76	1	11.93	13.87	17.8	14.53	10.59	14.21	12.4	0.85	1
Solyc01g098330.1.1	DNAJ heat shock N-terminal d	0.16	0	0	0	0.04	0	0	0	0	0	0.25	1	0	0	0	0	0	0	0	0	1	NA	0	0	0	0	0	0	0	0	1	NA	0	0	0	0	0	0	0	1	NA
Solyc01g098340.2.1	DNAJ heat shock N-terminal d	8.98	10.12	14.77	10.48	11.09	10.71	7.02	11.36	10.94	10.01	0.9	1	11.49	11.96	12.87	12.11	12	12.32	11	11.77	0.97	1	4.66	7.52	5.07	5.75	5.57	4.62	7.08	5.76	1	1	3.98	4.13	4.43	4.18	3.02	5.34	4.18	1	1
Solyc01g098350.2.1	Cyclin-dependent protein kin	31.52	28.05	24.24	27.1	27.73	27.31	28.66	31.39	23.18	27.63	1	1	26.87	30.49	25.19	27.52	24.32	23.76	28.78	25.62	0.93	1	19.1	17.88	13.8	16.93	18.57	17.1	16.1	17.26	1.02	1	23.94	29.62	35.87	29.81	18.95	23.98	21.46	0.72	1
Solyc01g098360.1.1	Unknown Protein (AHRD V1)	0	0	0	0	0	0	0	0	0	0	1	NA	0	0	0	0	0	0	0	0	1	NA	0	0	0	0	0	0	0	0	1	NA	0	0	0	0	0	0	0	1	NA
Solyc01g098370.1.1	LRR receptor-like serine/thre	0	0	0	0	0	0	0	0	0	0	1	NA	0	0	0.06	0.02	0	0	0	0	0.5	1	0	0	0	0	0	0	0	0	1	1	0	0	0	0	0	0	0	1	NA
Solyc01g098380.2.1	Dihydrodipicolinate reductase	56.26	57.13	47.68	59.97	55.26	69.15	71.63	69.95	76.38	71.78	1.3	0.36141	45.33	34.27	38.49	39.36	38.75	45.65	52	45.47	1.16	0.855973	22.89	32.45	22.42	25.92	32.41	29.28	36.81	32.83	1.27	1	49.57	31.8	22.85	34.74	39.17	39.6	39.39	1.13	1
Solyc01g098390.2.1	GID1-like gibberellin receptor	3.77	6.04	9.12	4.8	5.93	5.66	3.27	5.03	3.11	4.27	0.72	0.737127	7.54	8.52	11.78	9.28	8.89	6.44	6.96	7.43	0.8	0.953169	1.52	3.07	1.31	1.97	0.99	1	1.79	1.26	0.64	1	10.72	3.29	4.35	6.12	4.49	3.47	3.98	0.65	1
Solyc01g098400.2.1	Histidine phosphotransfer pro	42.5	46.1	40.22	22.55	37.84	86.62	61.87	109.51	56.58	78.64	2.08	0.044334	9.06	5.77	4.11	6.31	5.22	3.44	4.18	4.28	0.68	0.931153	1.43	1.08	1.76	1.42	1.45	2.68	0.97	1.7	1.19	1	49.1	32.8	10.21	30.7	24.72	8.93	16.82	0.55	1
Solyc01g098410.2.1	Receptor-like kinase (AHRD V	2.29	1.83	1.48	1.13	1.68	2.03	1.26	2.31	3.28	2.22	1.32	0.853475	1.7	1.49	1.26	1.48	0.63	0.59	1.26	0.83	0.56	0.673953	0.18	0.56	0.23	0.32	0.08	0.09	0.08	0.08	0.26	1	0	0	0	0	0	0	0	1	NA
Solyc01g098420.1.1	Receptor-like protein kinase	0	0	0	0	0	0.59	0.42	1.47	0.38	0.71	71.5	0.689959	0.99	0	0.45	0.48	0.37	0.55	0	0.31	0.64	1	0	0	0	0	0	0	0	0	1	NA	0	0	0	0	0	0	0	1	NA
Solyc01g098430.2.1	Breast carcinoma amplified se	15.5	20.55	20.82	18.32	18.8	15.74	13.46	17.96	16.72	15.97	0.85	0.924458	20.41	18.38	21.94	20.24	18.99	17.83	17.62	18.15	0.9	1	26.84	25.87	31.98	28.23	21.51	25.23	24.21	23.65	0.84	1	19.27	21.93	20.85	20.68	21.81	20.18	20.99	1.02	1
Solyc01g098440.1.1	Serine/threonine protein kina	0	0	0	0	0	0.15	0	0	0	0.04	3.75	1	0	0	0	0	0	0	0	0	1	NA	0	0	0	0	0	0	0	0	1	NA	0	0	0	0	0	0	0	1	NA
Solyc01g098450.2.1	Genomic DNA chromosome 3	4.21	4.91	5.91	4.73	4.94	3.15	3.44	5.54	4.33	4.12	0.83	0.980282	6.01	5.49	4.97	5.49	5.34	5.49	6.34	5.72	1.04	1	3.64	3.08	2.02	2.91	2.63	2.48	3.12	2.74	0.94	1	5.58	4.27	8.49	6.11	4.06	5.61	4.83	0.79	1
Solyc01g098460.2.1	Ribosomal RNA large subunit	9.41	13.63	10.94	8.17	10.54	11.35	10.71	11.86	11.58	11.38	1.08	1	12.5	10.75	11.01	11.42	14.91	14.12	14.15	14.39	1.26	0.673688	9.23	11.71	8.4	9.78	9.36	9.37	8.13	8.95	0.92	1	21.4	16.88	15.91	18.06	14.51	17.55	16.03	0.89	1
Solyc01g098470.2.1	Unknown Protein (AHRD V1)	3.14	0	3.71	13.33	5.04	2.33	4.13	1.93	0.75	2.29	0.45	1	0	0.91	1.77	0.89	0.73	0.54	0.82	0.7	0.78	1	0.85	2.56	3.12	2.18	1.38	0	0.76	0.71	0.33	1	0	1.52	0	0.51	0.96	1.84	1.4	2.76	1
Solyc01g098480.1.1	Pentatricopeptide repeat-con	0.54	1.65	1.28	1.54	1.25	1.45	0.52	1	0.55	0.88	0.7	0.864145	0.81	0.66	0.55	0.67	1.06	0.85	0.68	0.86	1.28	1	0.88	0.98	0.97	0.94	0.79	0.58	0.95	0.77	0.82	1	0.46	0.4	0	0.29	1	0.95	0.97	3.4	0.915671
Solyc01g098490.2.1	Solute carrier family 2 facilita	3.29	3.31	2.19	1.21	2.5	1.43	1.92	3.82	3.29	2.62	1.05	1	9.72	11.94	7.74	9.8	5.96	8.89	7.76	7.54	0.77	0.905697	13.53	10.96	6.11	10.2	6.39	6.18	8.97	7.18	0.7	0.753079	0.2	0.94	1.54	0.89	0.65	2.01	1.33	1.49	1
Solyc01g098500.2.1	Glucose transporter 8 (AHRD	98.24	117.81	97.21	85.44	99.67	162.38	127.73	154	140.75	146.22	1.47	0.126947	112.5	130.96	106.86	116.77	120.04	139.07	137.58	132.23	1.13	0.855973	31.49	39.91	26.65	32.68	25.05	26.52	34.96	28.84	0.88	1	3.05	15.35	10.32	9.57	20.26	26.42	23.34	2.44	0.356947
Solyc01g098510.2.1	Unknown Protein (AHRD V1)	4.91	5.62	1.66	11.91	6.03	0.78	7.76	5.81	3.53	4.47	0.74	1	7.83	16.47	16	13.43	2.43	5.45	6.62	4.83	0.36	0.096211	2.84	2.86	20.9	8.87	8.76	4.26	1.02	4.68	0.53	1	13.97	12.23	20.51	15.57	23.77	14.15	18.96	1.22	1
Solyc01g098520.2.1	Unknown Protein (AHRD V1);	10.88	23	11.23	25.01	17.53	12.1	13.96	22.51	12.7	15.32	0.87	1	40.49	57.35	39.06	45.63	10.85	20.07	21.38	17.43	0.38	0.002657	10.99	13.3	60.08	28.12	20.11	27.32	4.46	17.3	0.62	1	57.05	75.08	96.22	76.12	51.67	68.58	60.12	0.79	1
Solyc01g098530.2.1	CTP synthase like protein (AH	19.51	15.71	28.5	36.8	25.13	11.36	16.87	20.5	20.01	17.19	0.68	0.867252	43.21	49.27	32.96	41.81	12.89	11.55	13.87	12.77	0.31	0.000997	12.01	13.63	16.6	14.08	14.04	14.08	4.06	10.73	0.76	1	39.63	16.2	32.86	29.56	28.92	50.51	39.72	1.34	1
Solyc01g098540.2.1	Unknown Protein (AHRD V1)	3.22	9.31	13.29	13.66	9.87	4.47	5.72	11.1	8.09	7.34	0.74	1	14.97	11.19	13.59	13.25	17.3	13.75	11.38	14.14	1.07	1	11.05	16.39	19.96	15.8	13.74	17.68	9.97	13.8	0.87	1	32.03	19.28	17.32	22.88	16.94	19.75	18.34	0.8	1
Solyc01g098550.2.1	Tryptophan synthase alpha ch	55.36	41.67	39.14	43.98	45.04	45.94	54.78	38.69	56.62	49.01	1.09	0.917809	40.03	40.87	36.51	39.14	46.94	47.31	43.05	45.77	1.17	0.804182	58.51	61.33	52.69	57.51	63.39	71.98	63.41	66.26	1.15	1	33.42	35.25	37.31	35.33	55.53	52.03	53.78	1.52	0.813587
Solyc01g098560.2.1	Glucose transporter 8 (AHRD	7.36	9.58	8.85	8.81	8.65	10.23	7.04	11.06	9.45	9.45	1.09	1	7.9	13.29	11.36	10.85	10.9	13.93	13.02	12.62	1.16	0.911351	13.84	11.88	7.87	11.2	7.63	7.4	6.6	7.21	0.64	1	1.01	3.39	5.01	3.14	2.46	2.48	2.47	0.79	1
Solyc01g098570.2.1	Unknown Protein (AHRD V1)	5.89	3.07	5.75	3.45	4.54	11.06	6.57	8.77	4.37	7.69	1.69	0.425198	3.59	2.12	4.11	3.27	12.39	9.35	6.06	9.27	2.83	0.03627	190.4	142.25	90.87	141.17	71.21	70.62	96.89	79.57	0.56	1	45.89	55.88	46.2	49.32	37.72	27.41	32.56	0.66	0.809299
Solyc01g098580.2.1	Phosphoglycerate mutase fan	9.61	7.78	3.35	6.49	6.81	8.26	7.76	8.44	5.18	7.41	1.09	1	5.08	5.32	4.8	5.07	6.06	5.32	5.15	5.51	1.09	1	11.82	8.9	8.24	9.65	8.47	8.28	7.48	8.08	0.84	1	5.9	12.22	12.09	10.07	8.4	8.62	8.51	0.85	1
Solyc01g098590.2.1	Phosphoglycerate mutase fan	212.26	159.87	156.76	289.12	204.5	84.4	123.54	176.65	145.08	132.42	0.65	0.566195	142.97	424.77	256.12	274.62	170.34	141.18	158.99	156.84	0.57	0.691518	63.4	49.04	60.2	57.55	74.21	59.83	32.72	55.59	0.97	1	131.65	148.28	191.15	157.03	105.74	130.91	118.32	0.75	1
Solyc01g098600.2.1	Unknown Protein (AHRD V1);	46.18	48.65	30.02	35.99	40.21	71.76	63.99	74.95	59.2	67.47	1.68	0.064909	38.13	44.65	39	40.59	15.03	34.65	37.77	29.15	0.72	0.743157	55.58	18.43	25.26	33.09	20.12	14.29	26.1	20.17	0.61	1	0	1.03	2.17	1.07	2.16	2.89	2.53	2.37	1
Solyc01g098610.2.1	Glutathione synthetase (AHR	24.6	23.29	22.62	21.5	23	23.33	28.28	22.4	24.68	24.67	1.07	0.980869	18.17	19.81	20.48	19.49	21	20.54	21.33	20.96	1.08	1	12.57	14.05	13.41	13.34	10.22	10.79	10.02	10.34	0.78	1	4.8	7.88	5.01	5.9	8.83	8.78	8.8	1.49	1
Solyc01g098620.2.1	Unknown Protein (AHRD V1)	0.54	0.72	0.32	0	0.4	0	0.21	0.74	0.77	0.43	1.09	1	0	0.23	0.23	0.15	0.37	0.14	0.21	0.24	1.57	1	0	0	0.27	0.09	0.53	0.2	0	0.24	2.7	1	0	0	0	0	0	0	0	1	NA
Solyc01g098630.2.1	Import inner membrane trans	20.89	19.38	13.23	16.17	17.42	21.27	26.91	20.62	21.47	22.57	1.3	0.562961	16.16	19.5	17.27	17.64	25.01	29.94	29.9	28.28	1.6	0.093549	12.61	17.74	14.41	14.92	12.13	17.17	22.43	17.24	1.16	1	19.07	21.88	24.69	21.88	31.2	35.28	33.24	1.52	0.860853
Solyc01g098640.2.1	UPF0603 protein At1g54780,	82.58	74.91	62.27	59.53	69.82	207.61	177.08	168.16	174.91	181.94	2.61	9.16E-08	53.11	69.21	64.56	62.29	147.84	126.27	141.59	138.57	2.22	2.36E-05	7.35	13.9	11.52	10.92	13.9	9.14	19.4	14.15	1.3	1	1.29	6.11	1.75	3.05	9.37	5.58	7.47	2.45	0.975227
Solyc01g098650.2.1	GDSL esterase/lipase At1g547	55.8	57.87	29.63	36.43	44.93	50.01	39.78	29.82	38.57	39.54	0.88	0.962949	75.26	85.26	68.74	76.42	72.88	69.8	84.2	75.63	0.99	1	61	59.59	53.49	58.03	24.75	45.3	38.58	36.21	0.62	1	25.31	39.83	22.98	29.37	30.12	28.29	29.2	0.99	1
Solyc01g098660.1.1	Unknown Protein (AHRD V1)	0	0	0	0	0	0	0	0	0	0	1	NA	0	0	0	0	0	0	0	0	1	NA	0	0	0	0	0	0	0	0	1	NA	0	0	0	0	0	0	0	1	NA
Solyc01g098670.1.1	Kinesin 1-like (AHRD V1 ***-	0	0	0	0	0	0.18	0	0	0	0.04	4.5	1	0	0.42	0.27	0.23	0	0.08	0.13	0.07	0.3	1	0	1.04	0.32	0.45	0	0	0.23	0.08	0.17	1	0.23	0.12	0.49	0.28	0.58	0	0.29	1.04	1
Solyc01g098680.2.1	LRR receptor-like serine/thre	0	0	0	0	0	0	0	0	0	0	1	NA	0.14	0.06	0.06	0.09	0.05	0	0.06	0.04	0.42	1	0	0	0	0	0.05	0	0	0.02	1.67	1	0	0.05	0	0.02	0	0.13	0.07	3.9	1
Solyc01g098690.2.1	LRR receptor-like serine/thre	0.14	0.5	0.58	1.3	0.63	0	0.06	0.13	0.15	0.08	0.13	0.27545	1.05	1.04	2.03	1.37	0.54	0.73	0.56	0.61	0.44	0.269253	0.29	1.04	1.89	1.07	0.51	0.21	0.21	0.31	0.29	1	0.6	1.23	0.98	0.94	1.23	3.84	2.54	2.71	0.854686
Solyc01g098700.2.1	Branched-chain amino acid ar	20.01	19.95	20.02	27.94	21.98	12.81	20.99	13.27	16.45	15.88	0.72	0.618924	18.02	16.29	16.04	16.78	15.9	12.59	12.14	13.54	0.81	0.907488	39.69	36.73	49.52	41.98	32.36	40.88	39.77	37.67	0.9	1	37.45	37.25	33.88	36.19	67.46	61.17	64.31	1.78	0.457959
Solyc01g098710.2.1	Aspartyl protease family prot	5.77	5.49	6.45	8.87	6.64	2.02	4.07	4	3.2	3.32	0.5	0.081979	5.46	8.09	7.26	6.94	1.54	1.62	3.02	2.06	0.3	2.64E-05	0.98	1.07	1.1	1.05	1.33	1.07	0.29	0.9	0.85	1	13.24	6.69	5.45	8.46	26.12	26.17	26.15	3.09	0.004552
Solyc01g098720.2.1	Transcription factor (Fragmer	1	0.89	0.47	0	0.59	0.22	0.79	0.37	0.29	0.42	0.71	1	0	0	0.17	0.06	0	0	0.16	0.05	0.94	1	0	0	0.2	0.07	0.13	0	0	0.04	0.65	1	0.29	0.29	0	0.19	2.75	5.45	4.1	21.21	0.010972
Solyc01g098730.2.1	Unknown Protein (AHRD V1)	0.78	4.14	4.58	3.29	3.2	0.86	1.84	3.57	0	1.57	0.49	0.852496	0	0.67	1.97	0.88	1.08	1.21	3.66	1.98	2.25	1	2.51	3.16	5.39	3.69	2.04	2.94	1.7	2.23	0.6	1	48.55	9.58	5.97	21.37	9.24	5.44	7.34	0.34	1
Solyc01g098740.2.1	Receptor protein kinase-like p	2.28	2.3	3.23	2.37	2.54	0.84	1.8	1.96	1.2	1.45	0.57	0.596874	1.41	3.57	3.08	2.69	2.21	1.34	2.15	1.9	0.71	0.885463	41.14	47.07	43.6	43.94	42.34	57.26	42.76	47.45	1.08	1	24.42	18.11	13.56	18.7	49.54	23.99	36.77	1.97	0.848276
Solyc01g098750.2.1	RING finger protein B (AHRD	13.98	15.47	16.09	22.42	16.99	15.73	14	19.44	12.97	15.54	0.91	1	19.5	25.04	31.82	25.45	25.66	22.68	24.65	24.33	0.96	1	16.94	23.89	42.98	27.94	27.1	27.52	20	24.87	0.89	1	75.86	68.59	67.63	70.69	69.44	66.66	68.05	0.96	1
Solyc01g098760.2.1	Heavy metal-associated doma	163.08	141.57	169.3	205.16	169.78	54.66	85.79	82.48	98.49	80.36	0.47	0.000823	96.06	135.06	144.12	125.08	80.61	104.71	112.9	99.41	0.79	0.749201	23.04	37.43	21.57	27.35	44.55	20.91	17.86	27.77	1.02	1	7.01	23.06	23.7	17.92	12.7	19.73	16.21	0.9	1
Solyc01g098770.1.1	Xylanase inhibitor (Fragment)	0.31	0.27	0.73	0.22	0.38	0	0	0	0	0	0.03	0.249829	0	0.27	0.13	0.13	0	0.08	0	0.03	0.2	1	0	0	0.31	0.1	0	0	0	0	0.1	NA	0	0	0	0	0	0	0	1	NA
Solyc01g098780.2.1	Signal peptidase complex sub	128.02	134.55	93.58	109.42	116.39	115.24	116.57	118.42	103.46	113.42	0.97	1	120.17	126.64	82.59	109.8	73.26	77.67	98.44	83.12	0.76	0.649961	131.66	141.11	107.58	126.78	84.1	127.38	95.29	102.26	0.81	1	83.19	93.26	89.97	88.81	121.86	102.95	112.41	1.27	1

Solyc01g098790.1.1	Heat shock protein-like prote	0.41	0.24	0.32	0	0.24	0.3	0.64	0.25	0.29	0.37	1.53	1	0.13	0	0.11	0.08	0.09	0.14	0.42	0.22	2.71	1	0	0	0	0	0	0	0	0	1	NA	0	0	0	0	0	0	0	1	NA
Solyc01g098800.2.1	Sodium/calcium exchanger fa	7.36	14.26	8.12	20.48	12.55	10.42	13.77	12.55	9.11	11.46	0.91	1	12.14	21.04	24.75	19.31	13.41	17.94	14.08	15.14	0.78	0.958973	28.76	26.59	59.71	38.35	33.52	31.84	19.28	28.21	0.74	1	24.29	53.6	64.23	47.37	37.58	41.82	39.7	0.84	1
Solyc01g098810.2.1	Heat shock protein-like prote	11.99	12.96	13.81	18.58	14.34	13.97	12.63	13.13	11.69	12.86	0.9	1	13.55	7.45	6.27	9.09	8.52	9.76	9.65	9.31	1.02	1	7.38	8.61	6.8	7.6	7.5	3.9	5.83	5.74	0.76	1	5.68	8.09	8.34	7.37	1.05	1.5	1.27	0.17	0.06999
Solyc01g098820.2.1	Phototropic-responsive NPH3	0.24	0.53	0.28	0.17	0.3	0.26	0.47	0.55	0.43	0.43	1.4	1	0.66	0.93	0.1	0.56	0.41	0.62	1.31	0.78	1.38	1	0.19	0	0	0.06	0.23	0	0.17	0.13	2.11	1	0	0.35	0	0.12	0.33	0.94	0.64	5.44	1
Solyc01g098830.2.1	RING zinc finger protein-like p	11	12.18	6.92	8.99	9.77	15.21	12.35	14.15	8.77	12.62	1.29	0.792227	11.59	15.51	9.08	12.06	11.35	13.28	13.83	12.82	1.06	1	20.53	19.71	15.04	18.43	12.84	12.96	14.6	13.47	0.73	1	14.59	20.05	22.17	18.94	10.96	10.07	10.52	0.56	0.700272
Solyc01g098840.2.1	Mevalonate kinase (AHRD V1	25.52	25.1	28.32	30.87	27.45	19.59	19.08	20.22	22.54	20.36	0.74	0.630237	18.27	19.41	18.29	18.66	13.97	8.69	13.72	12.13	0.65	0.251201	37.02	43.36	29.49	36.62	27.02	20.99	20.43	22.81	0.62	1	15.75	14.87	16.52	15.71	9.22	8.54	8.88	0.57	0.739583
Solyc01g098850.2.1	Short-chain dehydrogenase/r	0	0	0	0	0	0	0.15	0	0	0.04	3.75	1	0.18	0	0.49	0.22	0	0	0	0	0.04	0.83581	0	0	0	0	0.13	0	0	0.04	4.33	1	0	0	0	0	0	0	0	1	NA
Solyc01g098860.2.1	Uncharacterized GPI-anchore	3.74	3.63	2.01	1.44	2.7	1.13	3.49	2.19	4.64	2.86	1.06	1	4.11	3.55	0.57	2.74	3.77	2.99	2.14	2.97	1.08	1	0.55	0.55	0.68	0.59	1.12	1.55	1.24	1.3	2.2	1	0.97	1.98	2.09	1.68	0.62	0	0.31	0.18	0.926032
Solyc01g098870.2.1	Nucleolar GTPase (AHRD V1 *	23.57	26.58	22.66	29.82	25.66	18.99	26.26	24.56	24.88	23.67	0.92	1	26.54	24.17	24.71	25.14	19.58	22.27	27.14	23	0.91	1	32.35	33.32	32.17	32.61	30.6	27.82	33.86	30.76	0.94	1	25.58	30.23	33.91	29.91	23.24	24.06	23.65	0.79	1
Solyc01g098880.2.1	Seryl-tRNA synthetase (AHRD	60.23	55.04	82.7	78.11	69.02	39.94	46.73	41.97	55.19	45.96	0.67	0.299131	60.82	57.48	68.28	62.19	55.25	43.61	45.25	48.04	0.77	0.669164	44.61	54	61.39	53.33	47.59	56.03	53.01	52.21	0.98	1	66.37	81.5	77.9	75.26	92.34	92.32	92.33	1.23	1
Solyc01g098890.1.1	Unknown Protein (AHRD V1)	0	0	0	0	0	0	0	0	0	0	1	NA	0	0	0	0	0	0	0	0	1	NA	0	0	0	0	0	0	0	0	1	NA	0	0	0	0	0	0	0	1	NA
Solyc01g098900.2.1	Yip1 domain-containing prote	47.66	50.57	31.02	53.47	45.68	41.23	72.84	44.25	43.32	50.41	1.1	0.953259	55.69	50.41	40.65	48.92	29.8	37.32	38.56	35.23	0.72	0.549114	42.68	50.7	42.64	45.34	37.02	40.39	48.59	42	0.93	1	25.7	25.92	24.91	25.51	27.72	32.94	30.33	1.19	1
Solyc01g098910.2.1	Mitochondrial carrier protein	28.84	23.45	24.23	28.17	26.17	14.07	17.85	21.55	17.57	17.76	0.68	0.392017	36.28	39.49	32.42	36.06	27.48	18.26	24.78	23.51	0.65	0.206728	60.51	78.27	95.55	78.11	77.14	70.13	52	66.42	0.85	1	74.33	149.02	132.07	118.47	121.92	110.41	116.16	0.98	1
Solyc01g098920.2.1	Mitochondrial Rho GTPase 1 (	70.67	78.07	109.04	88.26	86.51	62.32	56.36	61.5	67.16	61.84	0.71	0.40274	94.52	84.69	96.17	91.79	119.84	109.77	107.3	112.3	1.22	0.590698	58.41	65.32	73.39	65.71	76.55	68.73	66.68	70.65	1.08	1	161.46	158.28	186.88	168.87	204.07	252.41	228.24	1.35	0.946284
Solyc01g098930.2.1	Cyclin (AHRD V1 **** D8UD8	3.2	6.72	1.37	2.06	3.34	3.55	1.84	4.01	3.76	3.29	0.99	1	2.97	1.77	3.19	2.64	1.21	1.35	2.74	1.77	0.67	0.962977	4.23	0.95	2.6	2.59	1.15	0.22	1.06	0.81	0.31	1	0.83	2.32	2.68	1.94	1.06	0.76	0.91	0.47	1
Solyc01g098940.2.1	Pectinesterase (AHRD V1 ***	0	0	0	0	0	0	0	0	0	0	1	NA	0	0	0	0	0	0	0	0	1	NA	0	0	0	0	0	0	0	0	1	NA	0	0	0	0	0	0	0	1	NA
Solyc01g098950.1.1	Glyceraldehyde-3-phosphate	51.31	40.83	47.15	65.89	51.3	29.44	42.19	27.61	57.72	39.24	0.76	0.93457	49.93	39.46	57.32	48.9	62.98	65.13	51.1	59.74	1.22	0.637217	96.66	117.15	138.94	117.58	101.43	105.74	114.76	107.31	0.91	1	51.1	64.75	60.36	58.74	108.66	124.81	116.73	1.99	0.26615
Solyc01g098960.1.1	Glycogenin-like protein (AHRI	0.36	0	0	0	0.09	0	0	0	0	0	0.11	0.977607	0.11	0	0.1	0.07	0.08	0	0	0.03	0.38	1	0	0	0.12	0.04	0	0	0	0	0.25	NA	0	0	0	0	0	0	0	1	NA
Solyc01g098970.1.1	Glycogenin-like protein (AHRI	0	0	0	0	0	0	0	0	0	0	1	NA	0	0	0	0	0	0	0	0	1	NA	0	0	0	0	0	0	0	0	1	NA	0	0	0	0	0	0	0	1	NA
Solyc01g098980.2.1	Kinesin (AHRD V1 *-*- A6YTD	0.73	0.8	0.58	0.23	0.58	0.09	0.42	0.26	0.41	0.29	0.5	0.445413	0.53	0.21	0.65	0.46	0.37	0.34	0.29	0.33	0.72	1	0.16	0.2	0.04	0.13	0.08	0.03	0.12	0.08	0.57	1	0.06	0.15	0.19	0.13	0.04	0.07	0.06	0.41	1
Solyc01g098990.2.1	Os06g0146300 protein (Fragi	58.5	56.24	57.68	47.41	54.96	94.45	81.75	72.63	78.94	81.94	1.49	0.112296	45.08	55.31	52.47	50.95	69.6	75.17	67.95	70.91	1.39	0.226346	30.7	54.45	37.72	40.96	48.95	34.79	44.34	42.69	1.04	1	30.05	37.99	23.64	30.56	40.46	38.94	39.7	1.3	1
Solyc01g099000.1.1	Pentatricopeptide repeat-con	1.58	1.49	1.63	1.96	1.67	1.76	1.25	0.73	1.28	1.25	0.75	0.95493	1.47	1.89	1.5	1.62	2.19	1.99	2.1	2.09	1.29	0.938793	1.52	0.8	0.88	1.07	0.84	0.67	2.16	1.22	1.15	1	0.56	1.36	0.61	0.84	0.99	1.13	1.06	1.26	1
Solyc01g099010.2.1	GDSL esterase/lipase At1g28	0.18	0.16	0.41	0.25	0.25	0.98	0.55	0.65	0.38	0.64	2.56	0.751152	0.49	0.76	0.3	0.52	0.61	1	1.24	0.95	1.84	0.906465	6.38	3.43	2.61	4.14	1.61	2.93	1.79	2.11	0.51	1	0.75	1.91	0.54	1.07	0.32	1.23	0.78	0.73	1
Solyc01g099020.2.1	GDSL esterase/lipase At5g03	4.85	5.24	0.41	1.47	2.99	9.05	6.01	6.21	3.85	6.28	2.1	0.469358	2.25	2.11	1.75	2.04	5.4	4.12	6.26	5.26	2.58	0.032547	8.81	1.13	2.23	4.06	4.44	3.41	4.42	4.09	1.01	1	0	0.13	0	0.04	0	0	0	0.23	1
Solyc01g099030.2.1	GDSL esterase/lipase At1g28	0.7	2.19	1.03	0.5	1.1	0.39	1.25	1.13	0.76	0.88	0.8	1	0.65	0.15	0.15	0.32	0	0.36	0.55	0.3	0.96	1	0	0.57	0	0.19	0.12	0.27	0.26	0.22	1.14	1	0	0.13	0	0.04	0	0	0	0.23	1
Solyc01g099040.2.1	GDSL esterase/lipase At5g03	0.17	0.31	0.2	0.73	0.35	3.46	0.55	2.07	0.12	1.55	4.4	0.595448	0	0.15	0	0.05	0.48	0.54	0.54	0.52	10.4	0.462111	294.33	215.03	163.1	224.15	196.22	200.79	315.45	237.49	1.06	1	18.71	228.13	197.83	148.22	56.9	28.54	42.72	0.29	0.858186
Solyc01g099050.2.1	GDSL esterase/lipase At1g28	0	0	0	0	0	0	0.28	0.16	0.13	0.14	14.25	0.919129	0.32	0	0.29	0.2	0.36	0	0.41	0.26	1.26	1	0.28	0.43	0.17	0.29	0.46	0.13	0.76	0.45	1.53	1	0	0.13	0	0.04	0.16	0	0.08	1.85	1
Solyc01g099060.1.1	GDSL esterase/lipase At1g28	0	0	0	0	0	0	0	0	0.12	0.03	3	1	0	0	0	0	0	0	0	0	1	NA	0.42	0	0	0.14	0	0	0.38	0.13	0.9	1	0.25	0	0	0.08	0	0	0	0.12	1
Solyc01g099070.1.1	GDSL esterase/lipase At5g03	0.84	0	0	0	0.21	0	0	0	0	0	0.05	1	0	0	0	0	0.58	0.43	0	0.34	33.67	1	0	0	0.83	0.28	0	0	0	0	0.04	NA	0	0	0	0	0	0	0	1	NA
Solyc01g099080.2.1	GDSL esterase/lipase At5g45	34.29	47.1	61.56	61.68	51.16	39.36	32.07	37.01	36.93	36.34	0.71	0.534444	35.36	42.44	71.46	49.75	42.14	42.26	42.18	42.19	0.85	1	31.92	29.16	47.35	36.14	29.63	35.31	43.46	36.13	1	1	20.14	23.01	18.91	20.69	23.2	19.93	21.56	1.04	1
Solyc01g099090.2.1	Beta-mannosidase (AHRD V1	35.98	45.82	52.24	51.95	46.5	43.77	40.22	39.9	38.96	40.71	0.88	1	36.57	49.19	62.1	49.29	48.78	48.1	44.58	47.15	0.96	1	37.42	45.39	43.81	42.21	34.09	40.57	48.57	41.08	0.97	1	19.92	21.19	18.09	19.73	21.85	21.71	21.78	1.1	1
Solyc01g099100.2.1	Long-chain-fatty-acid coa liga	38.05	42.03	44.89	38.56	40.88	32.4	40.87	33.42	46.82	38.38	0.94	1	47.37	56.52	53.88	52.59	57.31	55.03	54.6	55.65	1.06	1	206.39	188.42	198.04	197.62	191.14	180.78	182.16	184.69	0.93	1	59.75	112.05	94.9	88.9	138.46	103.65	121.06	1.36	1
Solyc01g099110.2.1	Xaa-pro aminopeptidase (AH	21.52	21.56	21.55	19.24	20.97	36.77	27.66	26.9	27.83	29.79	1.42	0.215352	21.76	23	21.09	21.95	25.79	26.82	29.22	27.28	1.24	0.613646	21.45	21.41	19.98	20.95	16.96	21.26	21.8	20.01	0.96	1	18.28	16.99	10.6	15.29	19.45	19.2	19.32	1.26	1
Solyc01g099120.2.1	Protein AUXIN RESPONSE 4 (	31.95	31.21	26.97	32.33	30.61	22.52	41.01	21.97	25.36	27.71	0.91	1	35.03	36.35	34.22	35.2	32.97	37.25	34.29	34.84	0.99	1	22.66	35.56	22.69	26.97	30.2	20.08	26.69	25.66	0.95	1	9.23	12.45	11.58	11.09	14.27	20.27	17.27	1.56	0.975227
Solyc01g099130.2.1	LysM domain containing prot	56.16	52.6	64.52	56.89	57.54	60.25	50.53	52.09	69.9	58.19	1.01	1	48.13	64.6	61.11	57.95	76.88	58.16	54.16	63.07	1.09	1	89.76	72.14	63.53	75.14	73.79	83.09	59.87	72.25	0.96	1	132.15	86.57	86.74	101.82	104.84	101.71	103.28	1.01	1
Solyc01g099140.2.1	Transmembrane protein 49 (	0.62	1.66	2.2	0.88	1.34	1.73	0.61	0.71	1.11	1.04	0.78	1	1.59	1.89	0.66	1.38	1.29	0.96	0.85	1.03	0.75	1	9.53	5.82	6.01	7.12	2.65	2.71	3.73	3.03	0.43	1	0.22	0.79	0.24	0.42	0	0.14	0.07	0.17	1
Solyc01g099150.2.1	Lipoxygenase (AHRD V1 ****	0.31	0.54	1.89	0.97	0.93	1.36	0.72	0.77	1.1	0.99	1.06	1	0.92	1.2	0.77	0.96	1.54	1.27	1.32	1.38	1.43	0.83287	58.55	32.08	42.04	44.22	23	36.04	29.69	29.58	0.67	1	22.28	25.04	21.98	23.1	27.01	34.45	30.73	1.33	1
Solyc01g099160.2.1	Lipoxygenase (AHRD V1 ****	1.91	2.59	5.5	2.16	3.04	4.59	1.75	2.74	4.56	3.41	1.12	1	4.34	3.06	2.84	3.41	3.98	4.16	5.17	4.44	1.3	0.721851	75.35	48.71	51.52	58.53	25.36	35.05	32.92	31.11	0.53	1	66.95	53.74	49.02	56.57	53.11	83.11	68.11	1.2	1
Solyc01g099170.2.1	Lipoxygenase (AHRD V1 ***-	1.68	1.03	3.65	1.28	1.91	2.58	1.02	0.95	2.41	1.74	0.91	1	2.04	1.46	2.5	2	1.25	1.8	2.84	1.96	0.98	1	37.68	24.46	29.67	30.6	11.77	14.36	15.21	13.78	0.45	1	37.92	27.89	25.16	30.32	26.18	39.88	33.03	1.09	1
Solyc01g099180.2.1	Lipoxygenase (AHRD V1 ****	2.08	3.5	2	2.73	2.58	3.77	5.48	3.05	2.71	3.75	1.46	0.499368	10.84	10.2	15.58	12.21	14.72	13.62	15.28	14.54	1.19	0.820481	242.12	163.41	215.06	206.86	193.17	307.38	238.18	246.24	1.19	1	41.47	122.11	81.51	81.7	95.92	57.44	76.68	0.94	1
Solyc01g099190.2.1	Lipoxygenase (AHRD V1 ****	69.35	99.79	100.85	99.94	92.48	90.75	132.36	82.55	92.3	99.49	1.08	0.904124	351.55	375.79	549.16	425.5	600.58	518.61	389.29	502.83	1.18	0.88053	6500.5	4828.23	8202.28	6510.34	8998.81	10931.87	8021.36	9317.35	1.43	1	1429.57	3132	2089.76	2217.11	3476.5	2174.52	2825.51	1.27	1
Solyc01g099200.2.1	Lipoxygenase (AHRD V1 ****	1.72	1.46	1.39	1.44	1.5	1.31	1.67	2.02	1.18	1.54	1.03	1	3.13	2.25	2.05	2.48	4.08	2.72	3.76	3.52	1.42	0.605865	18.42	8.56	46.27	24.42	38.19	49.47	15.93	34.53	1.41	1	97.92	17.53	13.86	43.1	11.12	5.5	8.31	0.19	1
Solyc01g099210.2.1	Lipoxygenase (AHRD V1 ****	0.15	0.14	0.09	0.32	0.17	0.08	0.06	0.07	0.16	0.09	0.53	1	0	0	0.06	0.02	0.05	0	0	0.02	0.83	1	0.19	0	0	0.06	0.05	0.06	0	0.04	0.58	1	0	0	0	0	0	0	0	1	NA
Solyc01g099220.2.1	DNA polymerase (AHRD V1 *	3.08	4.49	4.87	3.78	4.06	2.52	2.78	4.02	3.23	3.14	0.77	0.737671	4.79	5.62	6.01	5.47	5.8	5.56	6.47	5.94	1.09	1	3.37	3.37	3.66	3.47	3.14	3.7	3.55	3.46	1	1	7.61	5.24	5.57	6.14	5.58	6.38	5.98	0.97	1
Solyc01g099230.2.1	Zinc finger protein VAR3, chlo	6.82	8.39	7.05	6.07	7.08	4.68	6.64	8.51	6.1	6.48	0.92	1	8.53	9.22	8.81	8.85	8.17	8.76	9.26	8.73	0.99	1	4.35	3.77	4.51	4.21	5.64	3.25	4.72	4.54	1.08	1	14	9.84	13.2	12.35	8.01	10.85	9.43	0.76	1
Solyc01g099240.2.1	3-hydroxyisobutyrate dehydr	15.65	9.22	18.96	29.01	18.21	15.28	17.19	10.5	11.84	13.7	0.75	1	13.37	16.91	23.57	17.95	19.65	14.23	11.3	15.06	0.84	1	8.71	12.58	13.01	11.43	7.92	8.65	6.32	7.63	0.67	1	9.02	9.37	7.8	8.73	9.48	10.2	9.84	1.13	1
Solyc01g099250.1.1	LRR receptor-like serine/thre	0	0.07	0	0.11	0.04	0	0.06	0	0	0.01	0.33	1	0	0	0	0	0	0	0.12	0.04	4	1	0	0	0	0	0	0	0	0	1	NA	0	0.05	0	0.02	0	0	0	0.6	1
Solyc01g099260.2.1	Chaperone protein dnaJ (AHR	69.61	83.11	63.81	41.17	64.42	102.72	80.8	99.9	87.06	92.62	1.44	0.248939	66.33	64.09	62.48	64.3	60.22	72.58	79.48	70.76	1.1	0.969905	44.48	55.14	28.17	42.6	50.35	43.2	43.3	45.62	1.07	1	24.48	34.53	27.97	28.99	40.85	43.59	42.22	1.46	1
Solyc01g099270.2.1	Microtubule binding protein (	0	0.36	0.47	0.38	0.3	0	0.11	0.49	0.19	0.2	0.65	1	0.12	0.12	0.11	0.12	0.09	0.28	0.1	0.16	1.34	1	0	0.11	0.13	0.08	0	0.1	0	0.03	0.42	1	0	0.1	0.2	0.1	0	0	0	0.1	1
Solyc01g099280.2.1	5&apos-AMP-activated prote	22.35	24.99	19.62	23.14	22.53	23.18	25.93	23.56	23.88	24.14	1.07	0.994418	24.85	20.74	23.6	23.06	16.54	19.9	23.35	19.93	0.86	1	27.64	26.88	26.52	27.01	20.68	22.52	25.73	22.98	0.85	1	12.79	17.82	16.98	15.86	16.84	14.1	15.47	0.98	1
Solyc01g099290.2.1	Cullin-like protein1 (AHRD V1	54.36	54.1	44.89	35.65	47.25	66.47	107.87	92.46	87.81	88.65	1.88	0.002166	98.6	40.16	38.61	59.12	29.13	26.23	37.28	30.88	0.52	0.761396	1.2	4.03	0.49	1.91	0.97	0.75	1.44	1.05	0.55	1	1.41	1.62	3.04	2.02	1.58	3.9	2.74	1.35	1
Solyc01g099300.2.1	MORC family CW-type zinc fir	12.47	13.57	14.5	12.29	13.21	14.38	13.42	13	14.06	13.71	1.04	1	13.32	12.93	17.01	14.42	12.5	13.54	16.12	14.05	0.97	1	12.99	10.77	12.66	12.14	8.38	10.36	11.04	9.93	0.82	1	19.71	18.05	23.73	20.5	12.77	16.36	14.56	0.71	1
Solyc01g099310.2.1	RNA polymerase II holoenzym	10.23	6.6	8.75	5.11	7.67	10.7	10.61	9.59	8.35	9.81	1.28	0.756842	7.46	6.62	6.1	6.73	8.07	7.99	6.78	7.61	1.13	1	5.83	8.66	6.97	7.15	4.61	6.38	4.24	5.08	0.71	1	1.14	2.48	1.54	1.72	1.47	2.99	2.23	1.3	1
Solyc01g099320.2.1	Nuclear transcription factor Y	0	0	0	0	0	0	0	0	0	0	1	NA	0	0	0	0	0	0	0	0	1	NA	0	0	0	0	0	0	0	0	1	NA	0	0	0	0	0	0	0	1	NA
Solyc01g099330.2.1	Os10g0578600 protein (Fragi	0	0.18	0.35	0.42	0.24	0.33	0.08	0.54	0.14	0.27	1.15	1	0.09	0.26	0.08	0.14	0.14	0.05	0.16	0.12	0.81	1	0	0	0	0	0	0	0.07	0.02	2.33	1	0.14	0.07	0	0.07	0.09	0	0.04	0.64	1
Solyc01g099340.2.1	Zinc finger protein (AHRD V1	35.86	42.14	36.26	35.57	37.46	46.93	40.93	66.94	38.78	48.4	1.29	0.487043	38.98	41.56	36.58	39.04	57.6	57.3	62.95	59.28	1.52	0.082965	14.92	18.85	12.94	15.57	12.64	9.41	17.76	13.27	0.85	1	11.04	21.08	26.97	19.7	3.98	3.67	3.83	0.19	0.047858
Solyc01g099350.2.1	Acyl-CoA binding protein 4 ac	34.56	34.53	36.82	44.43	37.59	25.61	37.55	26.16	33.72	30.76	0.82	0.926661	35.08	31.41	34.43	33.64	22.13	22.07	26.15	23.45	0.7	0.331806	25.06	35.06	27.85	29.32	27.88	26.05	27.05	26.99	0.92	1	28.7	28.32	30.74	29.25	29.38	35.48	32.43	1.11	1
Solyc01g099360.2.1	PWWP domain-containing pr	10.28	11.42	16.99	14.26	13.24	7.42	9.51	8.85	7.75	8.38	0.63	0.210659	13.03	13.14	16.47	14.21	12.85	12.4	11.62	12.29	0.86	1	11.1	10.88	11.17	11.05	12.21	8.53	11.3	10.68	0.97	1	19.26	14.69	16.4	16.78	11.5	11.99	11.75	0.7	1
Solyc01g099370.2.1	SRC2 homolog (Fragment) (AI	18.92	18.94	13.11	33.45	21.11	9.46	11.2	10.65	14.09	11.35	0.54	0.426362	17.16	16.65	38.74	24.18	10.5	16.65	7.25	11.47	0.47	0.648607	13.75	16.19	100.22	43.39	25.47	25.99	8.96	20.14	0.46	1	103.21	171.4	146.9	140.5	107.58	81.27	94.42	0.67	0.751248
Solyc01g099380.2.1	U4/U6.U5 tri-snRNP-associat	11.12	9.68	7.87	9.09	9.44	6.32	9.36	15.45	7.1	9.56	1.01	1	12.18	10.09	10.02	10.76	8.74	10.75	7.96	9.15	0.85	1	14.57	9.87	14.47	12.97	10.39	9.18	13.14	10.9	0.84	1	6.67	24.41	23.18	18.09	6.11	7.37	6.74	0.37	0.682649
Solyc01g099390.2.1	SRC2-like protein (AHRD V1 *	1.47	0.37	2.22	1.18	1.31	1.63	1.16	1.54	0.75	1.27	0.97	1	0.97	2.37	2.12	1.82	3.05	1.73	1.15	1.98	1.09	1	0.51	1.37	0	0.63	0.28	0	0.76	0.35	0.55	1	0	0.3	0	0.1	0	0.37	0.18	1.85	1
Solyc01g099400.2.1	WD-repeat domain phosphoi	78	74.22	85.42	95.66	83.32	75.64	47.01	73.63	75.81	68.02	0.82	0.83913	77.19	102.07	111.62	96.96	116.26	106.66	92.28	105.07	1.08	0.996305	83.64	105.99	125.82	105.15	102.76	88.52	104.01	98.43	0.94	1	177.27	107.88	107.19	130.78	120.02	140.6	130.31	1	1
Solyc01g099410.2.1	Histone H2A (AHRD V1 ***- (	245.93	223.27	199.61	352.95	255.44	158.14	301.51	199.71	180.75	210.03	0.82	1	282.2	275.03	290.35	282.53	140.38	220.51	168.58	176.49	0.62	0.120605	105.51	220.15	44.55	123.4	40.1	18.03	85.36	47.83	0.39	0.430098	60.62	35.85	42.79	46.42	142.14	71.22	106.68	2.3	0.58776
Solyc01g099420.1.1	Catalase (AHRD V1 ***- Q52	0	1.22	2.43	0.97	1.16	0	1.09	1.26	1.97	1.08	0.94	1	2.56	1.19	1.16	1.64	1.91	2.49	1.08	1.83	1.12	1	2.22	2.8	5.46	3.49	4.51	4.69	1	3.4	0.97	1	11.72	1.5	4.23	5.82	13.84	19.28	16.56	2.85	0.30302
Solyc01g099430.2.1	ATP-dependent DNA helicase	1	0	0	0.71	0.43	1.11	0	0	0	0.28	0.65	1	0.93	1.3	0.84	1.02	0	0.78	1.96	0.91	0.89	1	1.62	0.41	0	0.68	0.66	0	0	0.22	0.33	1	2.84	1.45	0.77	1.69	3.66	3.94	3.8	2.25	1
Solyc01g099440.2.1	ATP-dependent DNA helicase	0.45	3.2	2.12	1.27	1.76	1.5	1.42	3.3	2.58	2.2	1.25	1	6.68	5.85	3.03	5.19	2.49	3.49	3.53	3.17	0.61	0.892119	1.09	3.66	9.35	4.7	4.13	8.16	1.96	4.75	1.01	1	18.5	12.06	16.56	15.71	20.54	35.02	27.78	1.77	0.663989
Solyc01g099450.2.1	Unknown Protein (AHRD V1)	0	0.44	0.59	0.7	0.43	0	0	0	0.71	0.18	0.41	1	0.46	0	0.42	0.29	0.69	1.03	0	0.57	1.95	1	1.2	0.4	3.45	1.68	0.98	1.13	0	0.7	0.42	1	3.53	2.16	5.34	3.68	4.99	8.26	6.62	1.8	0.998877
Solyc01g099460.1.1	ATP-dependent DNA helicase	1.78	2.14	1.74	1.97	1.91	1.81	1.47	1.64	1.39	1.58	0.83	0.994784	1.66	2.16	1.96	1.93	1.18	1.57	1.1	1.28	0.67	0.821464	0.38	0.57	0.69	0.55	0.92	0.29	0.4	0.54	0.98	1	0.66	0.68	0.36	0.57	0.64	1.16	0.9	1.59	1
Solyc01g099470.2.1	ATP-dependent DNA helicase	9.34	5.72	6.2	5.78	6.76	1.95	3.69	5.37	4.61	3.91	0.58	0.722281	3.8	5.07	4.93	4.6	1.62	3.02	5.51	3.38	0.74	1	1.89	1.43	5.22	2.85	2.3	1.77	0.43	1.5	0.53	1	4.98	2.54	0.9	2.81	1.6	2.56	2.08	0.74	1
Solyc01g099480.2.1	Unknown Protein (AHRD V1);	0	2.84	3.77	1.51	2.03	0	0	0.98	0	0.24	0.12	0.637958	0	2.77	1.8	1.52	0	0.55	2.51	1.02	0.67	1	0.86	0.87	1.06	0.93	3.5	2.42	0	1.97	2.12	1	7.56	2.32	1.64	3.84	0.97	3.73	2.35	0.61	1
Solyc01g099490.1.1	Unknown Protein (AHRD V1)	0	0	0	0	0	1.88	0	0.78	0.61	0.82	81.75	0.89692	0	1.47	0	0.49	0	1.31	0.66	0.66	1.34	1	1.37	0	0.84	0.74	1.11	1.28	1.23	1.21	1.64	1	12.03	2.46	3.9	6.13	1.55	3.71	2.63	0.43	1
Solyc01g099500.1.1	ATP-dependent DNA helicase	0.79	0.94	0.31	2.23	1.07	0.29	0.21	0.97	1.13	0.65	0.61	1	0.98	1.37	1.78	1.38	0.18	0.68	1.03	0.63	0.46	0.833891	0.42	1.5	3.13	1.68	1.73	1.39	0.19	1.1	0.66	1	11.58	5.15	4.45	7.06	7.7	5.99	6.85	0.97	1
Solyc01g099510.2.1	Unknown Protein (AHRD V1)	0	0	0	0	0	0	0	0	0	0	1	NA	0	0	0	0	0	0	0	0	1	NA	0	0	2.05	0.68	0	0	0	0	0.01	1	0	0	0	0	0	0	0	1	NA
Solyc01g099520.2.1	Rapid alkalinization factor 2 (	123.34	122.53	113.7	195.56	138.78	140.93	176.18	178.28	145.38	160.19	1.15	0.655245	125.4	136.64	176.37	146.14	135.28	169.01	133.89	146.06	1	1	194.19	208.49	244.85	215.84	275.11	206.09	218.21	233.14	1.08	1	1028.51	716.23	924.49	889.74	1098.58	1326.36	1212.47	1.36	0.866396
Solyc01g099530.2.1	Pentatricopeptide repeat-con	1.54	1.68	1.34	1.54	1.53	1.98	1.72	2.41	1.67	1.95	1.28	0.786661	2.12	1.33	1.72	1.72	2.3	2.49	2.69	2.49	1.45	0.486223	1.92	1.33	1.23	1.49	1.63	1.58	1.84	1.68	1.13	1	1.27	2.48	1.52	1.76	1.22	1.48	1.35	0.77	1
Solyc01g099540.2.1	NTM19 protein (AHRD V1 ***	0	0	0	0	0	0	0	0	0	0	1	NA	0	0	0	0	0	0	0	0	1	NA	0	0	0	0	0	0	0	0	1	NA	0	0	0	0	0	0	0	1	NA
Solyc01g099560.2.1	Carbonyl reductase 3 (AHRD V	1.11	2.31	1.31	1.05	1.45	0.41	1.46	0.34	0.53	0.69	0.47	0.854164	0.34	2.9	0.63	1.29	0.51	0.96	1.17	0.88	0.68	1	0	0	0	0	0	0	0.54	0.18	18	1	0	0	0	0	0	0	0	1	NA
Solyc01g099570.2.1	Desiccation-related protein P	0	0	0	0	0	0	0	0	0	0	1	NA	0	0	0	0	0	0	0	0	1	NA	0	0	0	0	0	0	0	0	1	NA	0	0	0.56	0.19	0	0.32	0.16	0.86	1
Solyc01g099580.1.1	Desiccation-related protein P	5.27	2.34	1.62	0.65	2.47	4.71	2.35	4.84	1.89	3.45	1.4	1	4.15	2.89	3.29	3.44	2.94	3.32	3.87	3.38	0.98	1	4.81	3.55	4.66	4.34	5.79	6.77	7.68	6.75	1.55	1	3.75	10.32	11.8	8.62	5.14	3.62	4.38	0.51	0.8695
Solyc01g099590.2.1	Glutathione-S-transferase (AH	173.58	218.68	111.04	84.19	146.87	321.03	187.27	451.55	185.66	286.38	1.95	0.404581	223.1	269.15	201.09	231.11	239.36	387.27	480.43	369.02	1.6	0.33065	322.4	358.62	337.31	339.44	275.05	324.9	500.4	366.78	1.08	1	187.6	436.13	342.66	322.13	237.54	178.67	208.1	0.65	0.848276
Solyc01g099600.2.1	Copper chaperone (AHRD V1	54.22	71.67	67.69	70.48	66.02	116.04	108.04	89.69	62.73	94.12	1.43	0.257913	43.58	64.97	68.32	58.96	78.86	92.48	78.87	83.4	1.41	0.170248	101.35	112.52	107.63	107.17	261.99	207.45	244.21	237.88	2.22	0.001523	188.09	312.09	279.52	259.9	141.5	140.15	140.82	0.54	0.277633
Solyc01g099610.1.1	F-box-containing protein 1 (A	2.02	3	2.38	1.9	2.32	2.24	4.78	2.48	4.83	3.58	1.54	0.919033	3.76	4.1	4.55	4.14	9.34	3.49	4.76	5.86	1.42	1	0	1.65	0.67	0.77	0.44	0.51	0.98	0.64	0.83	1	8.61	0.49	1.04	3.38	0	0	0	0	1
Solyc01g099620.2.1	Respiratory burst oxidase-like	0.08	0.07	0.09	0	0.06	0	0	0	0	0	0.17	0.917319	0.07	0	0.13	0.07	0	0	0	0	0.15	0.962977	0.25	0.19	0.3	0.25	0.05	0.06	0.06	0.06	0.23	1	77.23	3.87	12.77	31.29	2.37	4.41	3.39	0.11	1
Solyc01g099630.2.1	Xyloglucan endotransglucosyl	250.78	257.79	266.52	412.54	296.91	265.67	336.52	169.51	234.1	251.45	0.85	1	67.77	19.31	41.09	42.72	4.47	17.14	9.43	10.35	0.24	0.288357	23.51	31.68	24.47	26.55	5.84	3.37	9.87	6.36	0.24	8.61E-05	1.26	0.65	2.39	1.43	3.86	6.82	5.34	3.73	0.180284
Solyc01g099640.2.1	Unknown Protein (AHRD V1)	48.87	54.36	40.36	38.02	45.4	67.9	81.02	76.38	52.62	69.48	1.53	0.531578	63.64	53.1	28.89	48.54	37.28	43.01	63.37	47.89	0.99	1	175.69	129.42	167.34	157.48	131.62	229.64	135.32	165.53	1.05	1	264.02	188.11	169.08	207.07	203.54	201.38	202.46	0.98	1
Solyc01g099650.2.1	Formin 3 (AHRD V1 *-*- D0Q	1.24	2.34	1.55	3.2	2.08	1.7	2.77	2.01	1.78	2.06	0.99	1	0.27	0.13	0.43	0.28	0.15	0.23	0.06	0.15	0.53	1	0	0	0	0	0	0	0	0	1	NA	0	0	0	0	0	0	0	1	NA
Solyc01g099660.2.1	Heat shock protein (AHRD V1	0	0.26	0.12	0	0.1	0.11	0	0.18	0.14	0.11	1.13	1	0	0.17	0	0.06	0.41	0.36	0.08	0.28	5	0.662334	2.47	7.3	5.57	5.11	3.82	4.18	6.17	4.72	0.92	1	0.84	2.36	1.51	1.57	4.15	2.76	3.46	2.2	0.807831
Solyc01g099670.2.1	40S ribosomal protein S10-lik	596.98	526.18	609.05	548.68	570.22	544.18	574.33	432.55	501.32	513.1	0.9	1	585.82	493.88	450.94	510.21	416.31	384.88	388.69	396.63	0.78	0.693876	319.21	363.38	344.23	342.27	319.95	331.43	312.09	321.16	0.94	1	433.11	388.03	446.25	422.46	454.41	505.1	479.75	1.14	1
Solyc01g099680.2.1	REF-like stress related protein	331.3	330.65	370.73	412.14	361.21	340.93	341.51	320.57	336.31	334.83	0.93	1	446.24	470.6	431.35	449.4	462.58	435.66	387.02	428.42	0.95	1	377.53	335.51	427.64	380.23	447.07	469.97	358.24	425.09	1.12	1	667.69	473.48	401.34	514.17	580.33	528.19	554.26	1.08	1
Solyc01g099690.2.1	ATP-dependent Clp protease	190.45	172.01	171.37	142.97	169.2	278.98	255.48	259.5	316.47	277.61	1.64	0.013342	190.48	210.45	180.96	193.96	379.06	301.15	310.91	330.37	1.7	0.00665	292.57	338.49	292.37	307.81	422.38	549.86	437.81	470.02	1.53	0.97019	306.35	436.22	352.12	364.9	576.76	447.71	512.24	1.4	1
Solyc01g099700.2.1	Os07g0673000 protein (Fragi	54.51	57.46	52.44	65.43	57.46	43.37	40.33	67.13	39.71	47.63	0.83	0.854164	45.46	63.29	55.89	54.88	43.25	39.26	44.26	42.26	0.77	0.672688	50.21	50.97	63.69	54.96	43.07	37.81	42.21	41.03	0.75	1	57.48	58.14	68.04	61.22	40.69	38.62	39.66	0.65	0.852617
Solyc01g099710.2.1	Tafazzin (AHRD V1 *-*- Q6PU	47.86	43.36	37.98	38.02	41.8	38.66	37.56	42.02	37.15	38.85	0.93	1	41.04	41.35	36.54	39.64	29.71	35.42	47.21	37.45	0.94	1	40.76	31.5	31.67	34.64	38.33	44.55	39.8	40.89	1.18	1	37.81	50.63	47.3	45.25	41.05	39.07	40.06	0.89	1
Solyc01g099720.2.1	Alpha subunit of F-actin capp	72.12	77.4	74.83	69.61	73.49	95.97	101.32	98.88	88.48	96.16	1.31	0.365245	70.44	71.55	65.57	69.19	75.18	62.71	73.85	70.58	1.02	1	82.78	88.33	77.69	82.93	73.72	86.34	84.55	81.54	0.98	1	70.27	70.25	65.57	68.7	82.14	76.26	79.2	1.15	1
Solyc01g099730.2.1	Unknown Protein (AHRD V1)	30.7	31.02	22.52	20.54	26.2	33.21	29.48	51.5	33.96	37.04	1.41	0.425574	33.58	28.49	26.98	29.68	25.32	29	32.93	29.08	0.98	1	38.88	35.84	31.71	35.48	24.26	36.16	33.56	31.33	0.88	1	21.24	33.45	37.64	30.78	30.01	27.28	28.65	0.93	1
Solyc01g099740.2.1	Small nuclear ribonucleoprote	151.47	179.23	142.86	144.27	154.46	166.44	152.82	197.93	154.86	168.01	1.09	0.985318	138.07	161.48	138.2	145.92	150.81	164.94	170.88	162.21	1.11	0.946596	140.96	154.91	132.26	142.71	152.83	136.85	135.37	141.68	0.99	1	96.88	188.82	181.54	155.75	142.63	142.7	142.66	0.92	1
Solyc01g099750.2.1	Heparan-alpha-glucosaminide	13.51	19.4	16.97	15.83	16.43	18.09	13.31	17.64	12.62	15.41	0.94	1	17.57	33.6	32.16	27.78	50.39	35.66	32.09	39.38	1.42	0.603713	14.56	23.56	16.88	18.33	18.04	16.19	23.61	19.28	1.05	1	48.96	56.15	43.35	49.49	42.7	40.9	41.8	0.84	1
Solyc01g099760.2.1	26S protease regulatory subu	162.91	157.59	199.35	190.59	177.61	130.75	137.09	128.32	146.67	135.71	0.76	0.632198	180.62	191.25	221.44	197.77	192.95	149.04	142.21	161.4	0.82	0.830872	141.54	160	148.57	150.04	140.44	142.46	143.77	142.22	0.95	1	381.38	271.1	280.65	311.04	254.81	313.63	284.22	0.91	1
Solyc01g099770.2.1	Translationally-controlled tur	16803.5	14801.82	10622.91	9869.39	13024.4	13885.35	12776.91	12694.37	15020.86	13594.37	1.04	1	13875.03	18040.3	13461.02	15125.45	11961.31	23494.12	21804.56	19086.66	1.26	0.784393	9733.13	12833.11	6819.84	9795.36	20765.28	17125.66	10343.02	16077.99	1.64	1	8313.57	11381.11	9701.07	9798.58	12945.36	12162.35	12553.85	1.28	1
Solyc01g099780.2.1	Translationally-controlled tur	5369.42	4416.4	4240.23	3767.97	4448.51	4082.29	4205.45	3596.3	5050.95	4233.75	0.95	1	4411.74	6124.28	5540.1	5358.71	4810.08	8739.28	6595.33	6714.9	1.25	0.71481	3025.4	4680.09	2568.92	3424.8	8018.64	6134.5	3838.81	5997.32	1.75	1	3191.89	3987.16	3013.39	3397.48	5387.62	4946.06	5166.84	1.52	0.844615
Solyc01g099790.2.1	AT3G05545 protein (Fragmen	83.58	80.44	69.45	92.66	81.53	71.44	83.92	69.46	78.09	75.73	0.93	1	76.31	90.25	79.08	81.88	84.44	74.87	78.68	79.33	0.97	1	97.86	88.88	108.11	98.28	96.22	85.6	88.72	90.18	0.92	1	112.47	86.85	90.76	96.69	94.9	94.42	94.66	0.98	1
Solyc01g099800.2.1	Unknown Protein (AHRD V1)	164.4	183.6	176.67	91.83	154.12	214.18	102.51	218.55	136.82	168.01	1.09	1	186.04	240.31	210.5	212.28	175.04	168.08	205.73	182.95	0.86	0.998648	412.85	360.94	435.51	403.1	304.06	291.28	348.73	314.69	0.78	1	326.06	435.04	362.31	374.47	228.14	234.12	231.13	0.62	0.634102
Solyc01g099810.2.1	Arginine/serine-rich splicing f	95.35	97.71	89.58	38.38	80.25	91.04	59.55	90.87	79.35	80.2	1	1	128.06	136.34	108.5	124.3	135.37	120.26	122.28	125.97	1.01	1	246.58	231.06	216.46	231.37	193.96	189.25	233.66	205.62	0.89	1	218.04	334.5	336.88	296.47	162.48	201.25	181.87	0.61	0.594996
Solyc01g099820.2.1	Hypoxia induced protein cons	6.53	7.56	9.25	8.32	7.92	12.34	24.75	22.22	15.94	18.81	2.38	0.029448	9.72	5.68	1.66	5.69	3.17	4.06	2.57	3.27	0.57	0.990265	1.06	0	0.65	0.57	1.29	0.5	0.48	0.76	1.33	1	0	0.47	0	0.16	0	0	0	0.06	1
Solyc01g099830.2.1	60S ribosomal protein L22-2 (	1366.54	1168.69	1271.87	1288.65	1273.94	1115.69	967.66	1010.46	1026.56	1030.09	0.81	0.738956	1274.54	1018.14	946.81	1079.83	770.23	800.8	967.36	846.13	0.78	0.692373	571.9	561.47	506.71	546.69	451.27	537.71	527.29	505.42	0.92	1	202.48	235.01	238.32	225.27	369.53	370.01	369.77	1.64	0.623395
Solyc01g099840.2.1	Auxin-repressed protein (AHR	439.81	416.91	716.78	460.79	508.57	480.92	245.48	452.34	351	382.44	0.75	0.701696	465.23	496.57	545.11	502.3	464.3	343.43	462.18	423.3	0.84	0.88712	99.99	137.94	175.63	137.85	57.78	44.57	91.3	64.55	0.47	1	5.09	8.91	6.29	6.76	7.49	12.11	9.8	1.45	1
Solyc01g099850.2.1	Multi-pass transmembrane pr	26.25	33.55	34.42	31.11	31.33	21.45	20.9	27.65	21.59	22.9	0.73	0.533277	27.97	29.45	29.38	28.93	24.9	19.87	25.04	23.27	0.8	0.857418	21.21	23.22	18.71	21.05	14.74	18.71	17.99	17.15	0.81	1	29.99	38.79	33.47	34.08	29.37	34.43	31.9	0.94	1

Solyc01g099860.2.1	Serine/threonine-protein pho	14.41	21.37	20.69	21.56	19.51	25.99	17.31	27.06	21.28	22.91	1.17	0.815099	22.14	17.42	18.34	19.3	20.42	22.27	24.45	22.38	1.16	0.903176	40.14	36.41	30.25	35.6	31.41	24.68	38.79	31.63	0.89	1	30.57	31.08	29.21	30.29	29.24	28.37	28.8	0.95	1
Solyc01g099870.1.1	RAG1-activating protein 1 ho	0	0.32	0	0	0.08	1.18	0.56	1.3	0.51	0.89	11.09	0.516876	0	0	0	0	0	0	0	0	1	NA	0	0	0	0	0	0	0	0	1	NA	0	0	0	0	0	0	0	1	NA
Solyc01g099880.2.1	RAG1-activating protein 1 ho	0	0.2	0.26	0.32	0.2	0.75	0.71	1.86	0.64	0.99	5.08	0.319152	0.42	0	0	0	0.14	0.31	0	0.1	0.74	1	0	0	0	0	0	0	0.33	0.11	11	1	0	0	0	0	0	0	0	1	NA
Solyc01g099890.2.1	Malignant T cell amplified se	36.61	58.71	32.5	40.51	42.08	55.2	61.53	62.99	48.14	56.97	1.35	0.432164	49.56	40.96	30.62	40.38	36.45	45.42	50.71	44.19	1.09	1	67.94	68.82	55.77	64.18	66.44	59.62	63.39	63.15	0.98	1	24.74	50.81	61.31	45.62	63.05	59.11	61.08	1.34	1
Solyc01g099900.2.1	Ribosomal protein L18 (AHRD	1631.73	1354.79	1632.82	1667.28	1571.65	1097.25	1189.04	926.94	1205.47	1104.67	0.7	0.330709	1553.71	1480.96	1478.38	1504.35	1112.75	1089.95	919.14	1040.61	0.69	0.277961	836.05	961.06	888.19	895.1	844.41	827.95	755.63	809.33	0.9	1	912.03	966.71	1237.33	1038.69	1317.99	1832.5	1575.24	1.52	0.698088
Solyc01g099910.2.1	Epoxide hydrolase (AHRD V1	0	0.19	0.25	0.3	0.18	0.23	0	0	0.15	0.1	0.51	1	0	0	0	0	0	0	0	0	1	NA	0.17	0.34	0	0.17	0.14	0	0	0.05	0.27	1	0	0	0	0	0	0	0	1	NA
Solyc01g099920.2.1	Photoassimilate-responsive p	105.94	59.93	44.89	46.08	64.21	46.25	54.03	26.93	31.64	39.71	0.62	0.573771	141.32	195.12	156.3	164.25	181.13	144.57	141.98	155.89	0.95	1	261.18	183.13	213.02	219.11	135.23	162.04	190.9	162.72	0.74	1	13.23	12.72	13.47	13.14	10.02	14.16	12.09	0.92	1
Solyc01g099930.2.1	Pectinesterase (Fragment) (A	150.21	144.3	130.6	108.08	133.3	154.12	133.3	145.3	122.82	138.88	1.04	1	155.32	166.86	131.27	151.15	112.45	139.33	130.05	127.28	0.84	0.982244	153.27	144.57	144.28	147.37	175.31	137.01	139.06	150.46	1.02	1	268.67	208.44	228.68	235.26	162.75	182.02	172.38	0.73	1
Solyc01g099940.2.1	Pectinesterase (AHRD V1 ***	0	0	0.17	0	0.04	0	0	0	0	0	0.24	1	0	0	0	0	0	0	0	0	1	NA	0	0	0	0	0	0	0	0	1	NA	0	0	0	0	0	0	0	1	NA
Solyc01g099950.1.1	Pectinesterase (AHRD V1 ***	0	0	0	0	0	0	0	0	0	0	1	NA	0	0	0	0	0	0	0	0	1	NA	0	0	0	0	0.08	0	0	0	1	1	0	0	0.19	0.06	0	0	0	0.16	1
Solyc01g099960.2.1	Pectinesterase (AHRD V1 ***	1.95	2.04	2.84	1.95	2.19	2.04	2.72	2	1.32	2.02	0.92	1	2.77	2.69	4.26	3.24	3.18	2.61	2.25	2.68	0.83	1	1.21	2.43	2.39	2.01	1.88	2	1.67	1.85	0.92	1	5.55	2.92	3.88	4.12	1.47	1.01	1.24	0.3	0.277633
Solyc01g099970.2.1	F-box/LRR-repeat protein 14	7.87	9.28	12.3	6.32	8.94	7.69	9.07	9.29	10.16	9.05	1.01	1	12.87	12.95	10.87	12.23	10.77	12.51	12.71	12	0.98	1	11.43	9.19	7.6	9.41	7.69	6.94	8.3	7.64	0.81	1	16.34	12.13	13.42	13.96	9.94	12.22	11.08	0.79	1
Solyc01g099980.2.1	Glycine-rich protein (AHRD V	56.01	61.95	65.8	28.34	53.02	70.99	56.89	104.83	93.97	81.67	1.54	0.258674	54.61	36.47	39.89	43.66	33.16	33.79	43.1	36.68	0.84	0.962977	14.96	18.24	7.09	13.43	5.77	4.8	10.67	7.08	0.53	0.822283	1.67	4.43	2.34	2.81	3.11	2.67	2.89	1.03	1
Solyc01g099990.2.1	F-box protein PP2-B1 (AHRD	0.23	0.81	0.8	0.64	0.62	0.76	0.36	0.63	0	0.44	0.71	1	0.63	1.58	0.77	0.99	1.73	1.76	0.89	1.46	1.47	0.982244	5.49	5.73	5.63	5.62	6.11	5.15	7.76	6.34	1.13	1	8.06	8.73	12.2	9.66	7.89	3.78	5.83	0.6	0.988071
Solyc01g100000.2.1	F-box protein PP2-B1 (AHRD	29.1	39.93	17.45	35.34	30.46	44.75	42.32	52.63	30.3	42.5	1.4	0.414113	24.59	21.51	11.27	19.12	19.35	25.12	35.64	26.7	1.4	0.742046	28.8	32.61	17.33	26.25	20.08	11.58	30.58	20.75	0.79	1	12.76	21.67	17.16	17.2	6.44	11.27	8.86	0.51	0.521798
Solyc01g100010.2.1	F-box protein PP2-B1 (AHRD	8.99	4	4.46	7.02	6.12	0.79	3.17	3.48	3.06	2.62	0.43	0.222201	2.2	3.29	2.4	2.63	2.13	1.84	2.79	2.25	0.86	1	0.76	1.93	1.41	1.37	2.02	0.54	1.72	1.43	1.04	1	13.45	6.7	5.82	8.66	0.22	1.45	0.83	0.1	0.16106
Solyc01g100020.2.1	Phospholipase D (AHRD V1 *	1.57	1.84	1.03	1.33	1.44	1.95	1.78	1.9	2.43	2.02	1.4	0.636919	1.63	1.2	1.32	1.38	1.04	0.81	0.84	0.9	0.65	0.772143	0.4	0.56	0.25	0.4	0.08	0.09	0.23	0.13	0.33	1	1.16	0.41	0.19	0.59	0.11	0.27	0.19	0.32	1
Solyc01g100030.2.1	Deoxyuridine 5&apos-triphos	34.54	21.07	22.43	23.6	25.41	22.43	32.17	25.33	26.19	26.53	1.04	1	29.96	10.79	21.63	20.79	9.42	5.22	7.01	7.22	0.35	0.209993	1.88	14.23	4.24	6.78	1.27	0.29	1.7	1.09	0.16	1	0	0	0	0	3.91	5.44	4.68	467.5	0.025773
Solyc01g100040.2.1	Integrin-linked kinase-associa	47.86	44.19	43.36	34.23	42.41	25.31	29.01	50.6	21.75	31.67	0.75	0.574209	28.35	28.99	20.9	26.08	12.81	10.95	18.31	14.02	0.54	0.023187	3.77	3.65	3.2	3.54	1.88	3.8	3.14	2.94	0.83	1	0.76	4.04	1.38	2.06	0.98	0.94	0.96	0.47	1
Solyc01g100050.2.1	WD-40 repeat protein (AHRD	47.16	48.76	62.86	56.75	53.88	39.16	34.8	36.35	38.71	37.26	0.69	0.287937	49.77	61.5	78.69	63.32	83.52	68.38	62.64	71.51	1.13	0.923189	71.69	76.01	99.06	82.25	76.46	73.26	83.59	77.77	0.95	1	108.45	109.57	120.34	112.79	96.37	104.25	100.31	0.89	1
Solyc01g100060.2.1	Pre-mRNA-splicing factor 38A	80.48	71.32	58.68	64.98	68.87	54.33	52.63	60.18	71.79	59.73	0.87	0.971416	67.31	67.11	64.18	66.2	72.23	75.11	65.42	70.92	1.07	1	67.67	67.69	55.88	63.75	83.27	73.82	64.18	73.76	1.16	1	67.28	105.11	86.84	86.41	75.88	79.5	77.69	0.9	1
Solyc01g100070.2.1	Unknown Protein (AHRD V1)	0	0	0	0	0	0.25	0	0	0	0.06	6.25	1	0	0	0	0	0	0	0	0	1	NA	0	0	0	0	0	0	0	0	1	NA	0	0	0	0	0	0	0	1	NA
Solyc01g100080.2.1	Single-stranded DNA binding	29.67	24.44	27.93	29.47	27.88	27.72	33.14	22.93	28.78	28.14	1.01	1	27.31	23.6	27.99	26.3	26.49	35.79	24.81	29.03	1.1	0.962977	15.81	17.75	12.85	15.47	15.55	12.2	16.1	14.62	0.94	1	27.38	28.66	31.55	29.2	44.5	50.92	47.71	1.63	0.724231
Solyc01g100090.1.1	Wall-associated receptor kina	0	0	0	0	0	0	0	0	0	0	1	NA	0	0	0	0	0	0	0	0	1	NA	0	0	0	0	0	0	0	0	1	NA	0	0	0	0	0	0	0	1	NA
Solyc01g100100.2.1	RING finger protein (AHRD V1	0	0.43	0	0	0.11	0	1.15	0	0.35	0.38	3.49	1	0	0	0	0	0.34	0	0	0.11	11.33	1	0	0	0	0	0	0	0	0	1	NA	0	0.35	0	0.12	0	0	0	0.09	1
Solyc01g100110.2.1	Integrin-linked kinase-associa	59.19	63.24	48.37	60.39	57.8	52.6	48.68	54.85	51.57	51.92	0.9	1	67.53	89.91	112.35	89.93	60.51	98.88	95.09	84.83	0.94	1	34.74	38.06	30.29	34.36	39.09	29.79	29.54	32.81	0.95	1	37.91	80.16	80.59	66.22	68.43	90.28	79.36	1.2	1
Solyc01g100120.2.1	Kinesin (AHRD V1 ***- Q5MN	0	0.06	0.08	0	0.04	0	0.05	0	0	0.01	0.36	1	0	0.06	0	0.02	0	0	0.05	0.02	0.83	1	0	0	0	0	0	0	0	0	1	NA	0	0	0	0	0	0	0	1	NA
Solyc01g100130.2.1	PHD-finger family protein (AH	10.4	10.02	11.35	10.68	10.61	8.24	7.48	10.61	9.31	8.91	0.84	0.942758	7.55	11.46	12.28	10.43	11.96	12.25	12.15	12.12	1.16	0.886539	19.28	17.82	18.46	18.52	22.18	18.41	20.45	20.35	1.1	1	34.95	39.95	43.05	39.32	27.59	27.73	27.66	0.7	0.945561
Solyc01g100140.2.1	Protein DEHYDRATION-INDU	158.89	187.72	140.32	135.37	155.57	165.77	147.45	179.83	141.09	158.54	1.02	1	157.3	164.77	130.67	150.91	155.64	161.89	205.19	174.24	1.15	0.861151	91.21	86.69	63.64	80.51	75.69	65.14	93.1	77.98	0.97	1	184.95	129.91	151.46	155.44	93.56	98.98	96.27	0.62	0.819748
Solyc01g100150.2.1	Unknown Protein (AHRD V1)	0	0	3.38	0	0.84	0	0	0	0	0	0.01	1	0	0	0	0	0	0	0	0	1	NA	0	0	0	2.85	0.95	0	0	0	0.01	NA	0	0	0	0	0	0	0	1	NA
Solyc01g100160.2.1	Protein DEHYDRATION-INDU	69.7	97.07	33.27	25.59	56.41	74.51	59.64	103.21	67.22	76.15	1.35	0.917407	79.18	67.06	29.21	58.48	67.53	91.73	108.29	89.18	1.52	0.482313	42.98	33.82	16.37	31.06	33.55	27.82	48.46	36.61	1.18	1	44.63	66.85	85.9	65.79	28.73	33.12	30.92	0.47	0.340058
Solyc01g100170.2.1	Soluble NSF Attachment Prot	23.34	26.56	24.67	31.3	26.47	23.47	34.94	28.93	24.48	27.96	1.06	0.983962	27.19	34.41	33.6	31.73	23.09	26.28	26.48	25.28	0.8	0.855973	38.54	41.02	36.07	38.54	36	26.31	36.68	33	0.86	1	20.2	36.56	29.65	28.8	23.97	24.56	24.27	0.84	1
Solyc01g100180.2.1	DNA polymerase III polC-type	9.83	9.82	7.1	8.23	8.75	13.15	14.41	15.86	14.4	14.46	1.65	0.133853	7.09	7.5	6.94	7.18	8.07	10.17	12.29	10.18	1.42	0.556156	11.34	9.48	7.36	9.39	10.8	9.27	11.69	10.59	1.13	1	4.85	6.7	2.16	4.57	6.98	6.15	6.57	1.44	1
Solyc01g100190.2.1	Misato homolog 1 (AHRD V1	7.11	8.17	7.58	7.3	7.54	8.42	7.07	5.8	6.75	7.01	0.93	1	8.22	7.08	8.43	7.91	10.19	7.78	10.55	9.51	1.2	0.896625	5.01	8.51	7.06	6.86	4.22	5.83	6.1	5.38	0.78	1	6.69	6.5	6.53	6.57	7.25	9.66	8.46	1.29	1
Solyc01g100200.2.1	GRAS family transcription fac	5.44	2.2	3.03	2.24	3.23	0.66	1.48	1	1.07	1.05	0.33	0.109732	3.68	2.92	3.09	3.23	3.36	3.23	3.73	3.44	1.07	1	7.27	6.93	9.92	8.04	9.03	11.47	9.73	10.08	1.25	1	8.72	10.78	8.06	9.19	4.26	3.9	4.08	0.44	0.24935
Solyc01g100210.2.1	Glycosyltransferase (AHRD V1	0.19	0	0	0.26	0.11	0.62	0.15	0.17	0.27	0.3	2.69	0.9636	0.35	0.16	0	0.17	0	0.1	0.44	0.18	1.06	1	0.3	0.3	0.18	0.26	0	0	0	0	0.04	1	0	0	0	0	0	0	0	1	NA
Solyc01g100220.2.1	GATA transcription factor 22	5.72	5.09	0.52	3.73	3.76	3.42	6.94	2.43	3.16	3.99	1.06	1	4.09	2.68	2.23	3	1.22	3.64	3.8	2.89	0.96	1	0	1.08	1.75	0.94	0.58	0.67	1.6	0.95	1.01	1	0.63	0.96	0.68	0.76	1.21	1.93	1.57	2.07	1
Solyc01g100230.2.1	Chaperone protein dnaJ (AHR	16.79	11.03	6.6	9.05	10.87	16.44	6.63	11.76	4.88	9.93	0.91	1	13.02	14.6	16.89	14.84	12.76	18.22	15.71	15.56	1.05	1	19.7	49.52	77.78	49	72.23	77.28	80.12	76.54	1.56	1	22.74	58.95	41.19	40.96	49.41	42.07	45.74	1.12	1
Solyc01g100240.2.1	Calmodulin-binding protein (	5.4	4.91	3.54	10.01	5.96	4	3.6	4.19	4.31	4.02	0.67	0.829018	3.12	8.65	10.23	7.33	5.82	5.53	6.03	5.79	0.79	1	6.88	5.86	34.17	15.64	10.16	11	5.16	8.77	0.56	1	25.75	81.11	62.71	56.52	52.81	28.71	40.76	0.72	1
Solyc01g100250.2.1	ABC transporter permease pr	1.82	2.11	3.23	1.29	2.11	2.43	3.02	2.68	3.4	2.88	1.36	0.807096	2.54	1.43	2.77	2.25	3.03	1.98	4.3	3.1	1.38	0.949319	3.39	4.6	4.52	4.17	2.39	3.73	3.19	3.1	0.74	1	3.11	7.15	4.48	4.91	4.17	3.04	3.6	0.73	1
Solyc01g100260.2.1	Guanosine-5&apos-triphosph	12.75	14.89	14.21	15.06	14.23	13.78	16.53	21.94	18.47	17.68	1.24	0.553086	17.19	13.52	14.52	15.08	19.47	18.17	17.19	18.28	1.21	0.730082	19.56	18.01	14.51	17.36	17.6	17.91	20.03	18.51	1.07	1	25.28	31.69	30.4	29.12	31.69	27.08	29.38	1.01	1
Solyc01g100270.2.1	3&apos-5&apos exonuclease	1.14	1.57	2.33	0.88	1.48	1.5	2.21	1.91	2.09	1.93	1.3	0.843786	0.58	0.81	1.23	0.87	0.5	1.02	0.82	0.78	0.89	1	0.75	0.51	0.51	0.59	0.27	0.24	0.76	0.42	0.72	1	0.29	0.3	0	0.2	0	0.09	0.04	0.23	1
Solyc01g100280.2.1	ATP-DEPENDENT DNA HELICA	0.84	1.25	1.1	1.32	1.13	1.35	1.55	0.86	1.55	1.33	1.18	0.988499	1.31	1.38	1.26	1.32	0.58	0.97	1.18	0.91	0.69	0.954502	0.45	1.22	0.37	0.68	0.43	0.21	0.34	0.33	0.48	1	0.4	0.34	0.43	0.39	0.43	0.41	0.42	1.08	1
Solyc01g100290.2.1	Unknown Protein (AHRD V1)	7.31	7.67	8.45	13.02	9.11	3.33	6.98	5.51	5.72	5.38	0.59	0.390294	7.75	7.37	9.93	8.35	4.46	5.83	5.22	5.17	0.62	0.227408	6.26	10.6	7.4	8.09	6.05	4.15	7.29	5.83	0.72	1	6.96	6.31	8.43	7.23	5.22	5.06	5.14	0.71	1
Solyc01g100300.2.1	Unknown Protein (AHRD V1)	2.64	0	4.67	7.47	3.7	2.93	1.04	0	2.84	1.7	0.46	1	3.68	3.44	4.46	3.86	0.92	2.05	2.07	1.68	0.44	0.97732	6.4	2.15	7.86	5.47	0.87	0	1.92	0.93	0.17	1	1.88	0	0	0.63	1.21	1.16	1.19	1.89	1
Solyc01g100310.2.1	Calmodulin-binding protein (	61.55	67.98	77.83	105.82	78.3	58.95	77.5	92.29	50.76	69.88	0.89	1	61.07	52.41	75.45	62.98	61.52	71.42	66.23	66.39	1.05	1	149.69	113.69	105.9	123.09	166.1	120.82	135.02	140.65	1.14	1	34.11	43.56	38.35	38.67	56.92	45.94	51.43	1.33	1
Solyc01g100320.2.1	Thioredoxin/protein disulfide	363.94	329.12	283.01	365.23	335.32	260.53	411.89	309.01	331.54	328.24	0.98	1	335.29	280.84	280	298.71	214.29	263.39	254.33	244	0.82	0.886944	1260.36	1285.1	1307.1	1284.19	1243.21	1165.73	1368.24	1259.06	0.98	1	307.19	497.78	389.16	398.04	627.89	530.38	579.13	1.45	0.968857
Solyc01g100330.2.1	Zinc finger matrin type 2 (AH	33.8	37.89	34.12	27.12	33.23	28.9	31.3	37.95	37.33	33.87	1.02	1	32.65	32.49	29.64	31.59	23.44	29.28	30.14	27.62	0.87	1	45.16	43.43	26.45	38.35	30.32	33.74	36.94	33.67	0.88	1	36.5	39.18	37.48	37.72	33.91	37.61	35.76	0.95	1
Solyc01g100340.2.1	Pentatricopeptide repeat-con	1.9	1.33	2.89	1.54	1.92	2.27	2.68	3	2.25	2.55	1.33	0.799385	1.77	2.13	2.76	2.22	5.28	2.82	2.46	3.52	1.59	0.73309	1.1	2.22	1.35	1.56	1.7	2.27	1.78	1.92	1.23	1	4.64	2.57	4.39	3.87	4.61	5.61	5.11	1.32	1
Solyc01g100350.2.1	ADP-ribosylation factor-like p	779.41	687.62	991.76	905.26	841.01	616.9	550.63	569.36	740.22	619.28	0.74	0.517227	783.83	915.49	936.05	878.46	1275.98	1135.83	1085.27	1165.69	1.33	0.291382	552.22	643.98	568.41	588.2	753.96	648.73	668.08	690.26	1.17	1	1313.65	728.61	801.12	947.79	1151.78	1444.28	1298.03	1.37	0.897443
Solyc01g100360.2.1	Dihydrolipoyl dehydrogenase	77.52	72.9	73.47	98.38	80.57	70.12	90.39	67.67	91.66	79.96	0.99	1	57.6	56.34	61.77	58.57	41.35	57.07	50.39	49.6	0.85	0.995526	57.78	72.12	65.37	65.09	49.49	59.98	71.06	60.18	0.92	1	5.75	12.67	17.41	11.94	29.4	25.43	27.41	2.3	0.127167
Solyc01g100370.2.1	Universal stress protein (AHR	100.34	108.99	231.17	221.28	165.44	74.21	148.76	128.21	88.49	109.92	0.66	0.834458	181.89	378.42	279.94	280.08	317.28	232.76	191.86	247.3	0.88	1	942.14	642.84	785.39	790.12	932.43	1191.95	808.33	977.57	1.24	1	9155.65	1587.8	4802.01	5181.82	5499.87	3722.02	4610.94	0.89	1
Solyc01g100380.2.1	Calreticulin 2 calcium-binding	793.23	683.92	629.55	776.89	720.9	510.17	777	499.47	568.91	588.89	0.82	0.833287	591.63	571.28	627.73	596.88	436.98	482.54	435.97	451.83	0.76	0.5907	941.65	1359.5	940.88	1080.68	1080.56	784.23	1103.06	989.28	0.92	1	337.27	468.72	370.11	392.03	534.7	618.69	576.7	1.47	0.920332
Solyc01g100390.2.1	Pyrophosphate-energized pro	0	0	0	0.12	0.03	0	0	0.16	0	0.04	1.33	1	0	0.15	0.15	0.1	0	0.09	0.07	0.05	0.53	1	0.07	0	0.09	0.05	0	0	0	0	0.19	1	0	0.06	0	0.02	0	0	0	0.5	1
Solyc01g100400.2.1	Pseudouridine synthase famil	3.5	5.02	7.12	3.3	4.74	1.51	5.53	3.4	4.33	3.69	0.78	1	5.25	3.89	5.26	4.8	4.86	4.23	5.05	4.71	0.98	1	1.73	2.85	2.32	2.3	1.79	1.33	1.99	1.7	0.74	1	1.11	1.55	1.5	1.39	1.78	2.9	2.34	1.69	1
Solyc01g100410.2.1	Iron-sulfur cluster insertion p	14.34	27.35	17.4	11.59	17.67	31.43	18.44	28.26	18.83	24.24	1.37	0.751827	16.39	17.45	11.42	15.09	16.77	19.94	24.8	20.5	1.36	0.65557	53.96	33.39	35.79	41.05	24.76	45.03	36.13	35.31	0.86	1	9.9	32.44	25.83	22.72	22.13	17.96	20.05	0.88	1
Solyc01g100420.2.1	Peptide chain release factor 2	9.48	8.68	8.11	5.44	7.93	6.87	12.26	7.71	10.16	9.25	1.17	0.860394	10.36	9.68	7.78	9.27	8.67	8.82	11.34	9.61	1.04	1	13.1	14.45	13.23	13.59	11.92	14.69	15.22	13.94	1.03	1	10.55	8.68	9.3	9.51	13.34	14.46	13.9	1.46	0.939829
Solyc01g100430.2.1	Ribosomal RNA small subunit	17.47	16.37	15.35	14.46	15.91	12.39	14.08	16.92	10.27	13.41	0.84	0.924872	14.52	16.64	11.14	14.1	10.57	8.37	13.73	10.89	0.77	0.798909	7.21	9.09	10.88	9.06	7.94	7.04	8.53	7.84	0.86	1	8.19	14.44	16	12.88	12.08	13.69	12.88	1	1
Solyc01g100440.1.1	Transcription regulatory prot	0.13	0	0.3	0	0.11	0	0.1	0	0	0.03	0.23	1	0	0	0	0	0	0	0	0	1	NA	0	0	0	0	0.08	0	0	0.03	2.67	1	0	0	0	0	0	0	0	1	NA
Solyc01g100450.1.1	Pentatricopeptide repeat-con	1.85	2.33	2.83	1.69	2.17	1.94	1.29	3.1	2.81	2.29	1.05	1	2.43	1.7	2.21	2.11	2.19	2.09	2.22	2.17	1.03	1	0.97	1.06	1.51	1.18	1.36	0.91	1.51	1.26	1.07	1	1.55	2.06	2.51	2.04	1.59	1.24	1.42	0.69	1
Solyc01g100460.2.1	BZIP transcription factor (AH	3.32	5.49	3.36	1.34	3.38	5.28	3.37	5.67	3.41	4.43	1.31	1	6.18	3.71	3.21	4.37	5.27	5.65	5.59	5.5	1.26	1	21.09	28.62	25.91	25.21	27.44	31.66	38.04	32.38	1.28	1	73.55	24.82	10.95	36.44	11.3	8.74	10.02	0.27	1
Solyc01g100470.2.1	Unknown Protein (AHRD V1)	0	0	0	0	0	0	0	0	0	0	1	NA	0	0	0	0	0	0	0	0	1	NA	0	0	0	0	0	0	0	0	1	NA	0	0	0	0	0	0	0	1	NA
Solyc01g100480.2.1	Unknown Protein (AHRD V1)	23.75	24.13	22.93	22.4	23.3	14.67	13.83	20.07	19.12	16.92	0.73	0.576139	24.99	24.82	27.96	25.92	18.14	23.48	25.07	22.23	0.86	1	33.53	36.36	35.96	35.28	35.62	31.27	26.05	30.98	0.88	1	49.8	74.22	73.04	65.69	44.36	31.97	38.16	0.58	0.543916
Solyc01g100490.2.1	Nicotianamine synthase (AHR	0	0	0	0	0	0	0	0	0	0	1	NA	0.19	0	0	0.06	0	0	0.16	0.05	0.84	1	0.34	0.17	0.21	0.24	0	0	0.15	0.05	0.21	1	0.59	0.6	0.32	0.5	0	0	0	0.02	0.989909
Solyc01g100500.2.1	Microfibrillar-associated prot	55.72	49.45	71.33	65.39	60.47	38.79	38.55	51.09	53.08	45.38	0.75	0.636919	54.82	63.87	81.19	66.63	104.15	91.85	84.56	93.52	1.4	0.190345	76.18	71.09	84.63	77.3	118.05	92.93	88.79	99.92	1.29	1	114.69	194	171.35	160.01	146.42	128.17	137.29	0.86	1
Solyc01g100510.2.1	Knotted-like homeobox prote	2.16	2.27	1.16	1.39	1.75	1.31	3.1	4.69	3.66	3.19	1.83	0.420511	3.65	2.05	2.16	2.62	4.49	5.79	4.47	4.92	1.88	0.235884	0.95	1.76	1.17	1.29	2.97	2.83	2.15	2.65	2.05	1	0.84	1.85	1.81	1.5	2.52	2.41	2.46	1.64	1
Solyc01g100520.2.1	ATP-dependent Clp protease	166.69	147.57	142.69	139.04	149	239.8	268.4	209.67	217.39	233.81	1.57	0.031157	121.09	135.34	144.87	133.77	242.2	218.84	241.42	234.15	1.75	0.004796	184.77	234.92	288.12	235.94	250.35	319.51	295.66	288.51	1.22	1	196.66	312.89	230.28	246.61	397.57	315.63	356.6	1.45	0.989607
Solyc01g100530.2.1	Protein EFR3 homolog A (AHR	1.49	1.87	0.96	1.44	1.44	1.65	2.03	1.62	1.46	1.69	1.17	0.991164	1.13	1.06	1.09	1.09	0.52	0.77	0.9	0.73	0.67	0.894674	0.11	0.22	0	0.11	0	0	0	0	0.09	1	0.48	0.39	0.73	0.53	0.06	0.06	0.06	0.11	0.587072
Solyc01g100540.2.1	PAPA-1-like conserved region	4.25	4.31	5.43	3.67	4.41	4.59	2.98	5.21	3.47	4.06	0.92	1	4.17	4.72	5.28	4.72	7.28	7.03	5.94	6.75	1.43	0.475116	4.48	3.27	2.11	3.29	3.33	2.42	2.49	2.75	0.84	1	19.63	7.8	8.89	12.11	6.7	7.24	6.97	0.58	1
Solyc01g100570.2.1	Nucleolar protein (AHRD V1	145.64	166.71	160.02	138.96	152.83	142.03	123.63	196.31	133.45	148.85	0.97	1	160.61	156.41	158.01	158.34	150.65	145.63	160.4	152.23	0.96	1	77.64	77.73	69.97	75.11	84.02	62.19	90.07	78.76	1.05	1	125.63	89.43	123.6	112.89	111.95	142.83	127.39	1.13	1
Solyc01g100580.2.1	Pentatricopeptide repeat-con	10.93	11.79	8.86	6.57	9.54	11.24	7.98	13.56	9.12	10.47	1.1	1	9.78	10.45	11.31	10.51	13.45	12.09	13.42	12.99	1.24	0.785436	6.51	7.46	5.15	6.37	4.94	6.63	8.16	6.58	1.03	1	8.93	5.06	6.93	6.97	5.5	9.34	7.42	1.06	1
Solyc01g100590.2.1	Unknown Protein (AHRD V1)	43.49	40.66	44.2	42.65	42.75	20.92	30.87	33.94	40.28	31.5	0.74	0.611389	42.84	48.13	36.43	42.47	35.24	33.78	36.77	35.26	0.83	0.942539	33.81	40.5	27.93	34.08	38.66	32	34.36	35.01	1.03	1	27.02	43.54	42.71	37.76	31.12	25	28.06	0.74	1
Solyc01g100600.2.1	Kelch domain-containing prot	6.73	7.16	2.65	5.79	5.58	11.59	23.54	12.74	13.07	15.23	2.73	0.023025	7.61	3.56	3.57	4.91	1.1	4.58	1.56	2.41	0.49	0.855513	0	0.11	0	0.04	0.09	0.1	0	0.06	1.73	1	0	0	0	0.12	0	0	0.06	6	1
Solyc01g100610.2.1	Solute carrier family 40 mem	19.06	25.19	19.56	12.44	19.06	34.85	24.66	31.31	25.91	29.18	1.53	0.159939	24.13	20.78	14.38	19.76	20.18	21.37	23.13	21.56	1.09	0.98802	15.94	15.25	13.1	14.76	17.78	16.07	17.01	16.95	1.15	1	31.27	11.63	15.43	19.44	21.78	18.79	20.29	1.04	1
Solyc01g100620.1.1	Non-histone chromosomal pr	0.64	0.14	0.19	0.45	0.35	0.53	0.63	0.29	0	0.36	1.02	1	0.15	0.14	0	0.1	0	0.17	0	0.06	0.59	1	0	0	0	0	0	0	0	0	1	NA	0	0	0	0	0	0	0	1	NA
Solyc01g100630.1.1	Catalase (AHRD V1 ***- Q25	6.22	12.19	0	3.52	5.48	9.69	8.84	14.88	11.62	11.26	2.05	0.636919	9.26	6.49	4.21	6.65	12.09	12.89	22.5	15.83	2.38	0.340797	20.11	15.21	7.41	14.24	4.91	12.26	12.7	9.96	0.7	1	63.71	64.19	76.56	68.15	29.63	32.74	31.19	0.46	0.548489
Solyc01g100640.2.1	Catalase (AHRD V1 ***- O245	5	6.48	5.37	6.44	5.82	8.09	5.75	6.69	8.82	7.34	1.26	0.923329	12.27	17.8	13.07	14.38	13.88	16.02	19.31	16.4	1.14	1	16.54	8.53	16.71	13.93	14.65	12.76	12.6	13.34	0.96	1	76.98	76.99	86.75	80.24	52.06	60.24	56.15	0.7	1
Solyc01g100650.2.1	NHL repeat containing 2 (AH	20.75	20.86	16.82	16.19	18.66	32.04	31.94	27.85	28.29	30.03	1.61	0.035137	18.16	21.44	22.95	20.85	28.65	30.42	29.82	29.63	1.42	0.170304	14.34	13.07	13.73	13.71	14.63	14.89	14.14	14.55	1.06	1	20.45	18	19.76	19.4	19.83	19.42	19.62	1.01	1
Solyc01g100660.2.1	Transcription factor (AHRD V	29.96	23.26	83.1	68.97	51.32	37.2	56.86	28.13	26.08	37.07	0.72	1	25.79	24.66	21.53	23.99	32.49	19.8	31.81	28.03	1.17	0.962977	19.57	28.05	14.24	20.62	20.52	14.73	18.35	17.87	0.87	1	175.81	35.42	41.66	84.3	36.47	49.18	42.83	0.51	1
Solyc01g100670.1.1	3&apos-5&apos exonuclease	0	0	0	0	0	0	0	0	0	0	1	NA	0	0	0	0	0	0	0	0	1	NA	0	0	0	0	0	0	0	0	1	NA	0	0	0	0	0	0	0	1	NA
Solyc01g100680.1.1	3&apos-5&apos exonuclease	0	0	0	0	0	0	0	0	0	0	1	NA	0	0	0	0	0	0	0	0	1	NA	0	0	0	0	0	0	0	0	1	NA	0	0	0	0	0	0	0	1	NA
Solyc01g100690.2.1	Unknown Protein (AHRD V1)	35.07	32.14	28.27	35.85	32.83	40.53	46.33	40.22	38.64	41.43	1.26	0.564332	34.88	34.41	41.64	36.98	27.94	38.11	36.29	34.11	0.92	1	19.36	30.27	27.92	25.85	32.39	31.58	29.86	31.28	1.21	1	400.37	130.63	188.48	239.83	103.62	117.51	110.56	0.46	1
Solyc01g100700.2.1	Unknown Protein (AHRD V1)	44.77	61.86	77.83	74.99	64.86	45.18	56.27	56.87	59.2	54.38	0.84	1	63.01	62.47	66.17	63.88	50.66	49.1	49.53	49.76	0.78	0.83287	66.15	54.24	51.45	57.28	38.7	50	44.21	44.3	0.77	1	41.87	46.2	42.57	43.55	33.43	36.67	35.05	0.8	1
Solyc01g100710.2.1	Unknown Protein (AHRD V1)	161.38	215.89	210.75	343.12	232.78	156.82	109.77	134.38	115.13	129.02	0.55	0.230441	273.78	356.44	247.74	292.65	204.14	257.5	197.01	219.55	0.75	0.69002	132.02	63.68	76.56	90.75	79.34	121.54	64.35	88.41	0.97	1	226.56	205.67	190.41	207.55	92.91	133.48	113.19	0.55	0.529658
Solyc01g100720.2.1	Importin alpha-1b subunit (A	87.92	93.39	104.31	92.97	94.65	65.79	63.47	69.5	73.57	68.08	0.72	0.387555	94.28	98.19	90.92	94.46	68.25	63.59	67.06	66.3	0.7	0.317397	75.61	69.23	74.17	73	82.46	78.6	60.47	73.84	1.01	1	142.01	117.77	113.96	124.58	85.79	97.52	91.66	0.74	1
Solyc01g100730.2.1	UPF0176 protein yceA (AHRD	7.23	6.15	7.9	9.77	7.76	5.52	7.59	4.77	6.43	6.08	0.78	0.926149	5.52	6.38	7.39	6.43	7.71	7.49	8.15	7.78	1.21	0.871865	6.11	6.69	5.57	6.12	5.11	4.5	5.67	5.09	0.83	1	10.9	6.04	7.31	8.08	8.5	7.1	7.8	0.96	1
Solyc01g100740.2.1	Xaa-pro dipeptidase (AHRD V	3.98	5.25	5.18	4.66	4.77	2.59	4.44	4.67	2.86	3.64	0.76	0.875124	4.59	5.84	5.56	5.33	3.9	4.12	3.99	4	0.75	0.899465	3.99	3.91	4.08	3.99	3.69	3.95	3.8	3.81	0.95	1	6.05	5.58	6.12	5.92	5.65	5.29	5.47	0.92	1
Solyc01g100750.2.1	Susceptibility homeodomain	1.21	2.15	0.95	0.57	1.22	3.58	1.59	5.55	2.02	3.18	2.61	0.49454	0.37	0.35	1.02	0.58	0	0	0	0	0.02	0.704874	3.25	0	2	1.75	0.26	0.61	0.29	0.39	0.22	1	0	0.29	0	0.1	0	0	0	0.1	1
Solyc01g100760.2.1	Susceptibility homeodomain	39.18	61.2	15.57	10.75	31.67	96.89	33.14	108.08	63.72	75.46	2.38	0.369379	31.24	20.16	12.49	21.3	3.05	9.52	18.85	10.47	0.49	0.677375	952.91	111.75	561.16	541.94	198.31	402.44	233.67	278.14	0.51	1	16.49	60.11	45.5	40.7	42.09	16.48	29.29	0.72	1
Solyc01g100770.2.1	Susceptibility homeodomain	39.58	57.65	26.89	27.71	37.96	63.55	50.5	80.14	52.24	61.61	1.62	0.198407	49.09	45.53	46.6	47.07	25.51	23.19	23.57	24.09	0.51	0.024169	282	102.3	236.13	206.81	76.43	144.55	100.81	107.26	0.52	1	3.41	4.36	2.46	3.41	8.78	7.36	8.07	2.37	0.846703
Solyc01g100780.2.1	Agenet domain-containing pr	16.9	12.89	12.34	14.51	14.16	9.39	8.26	10.54	11.7	9.97	0.7	0.568572	18.15	12.59	18.52	16.42	16.04	18.02	13.44	15.83	0.96	1	3.9	9.83	4.39	6.04	10.17	4.12	6.6	6.96	1.15	1	3.15	2.78	4.64	3.52	5.16	7.05	6.11	1.73	0.879616
Solyc01g100790.1.1	Pentatricopeptide repeat-con	0.27	0.55	0.1	0.75	0.42	0.59	0.63	0.65	0.13	0.5	1.2	1	0.49	0.15	0.45	0.36	0.31	0.41	0.21	0.31	0.85	1	0	0.14	0.26	0.13	0.29	0.27	0.26	0.27	2.05	1	0.13	0.19	0.41	0.24	0.4	0.31	0.35	1.46	1
Solyc01g100800.1.1	Pentatricopeptide repeat-con	1.62	0.87	0.77	0.92	1.05	1.2	1.2	0.8	1.01	1.05	1.01	1	1.01	0.56	1.19	0.92	1.13	1.35	1.02	1.17	1.27	1	1.49	0.71	1.18	1.13	1.21	0.25	1.5	0.99	0.88	1	0.46	1.1	0.67	0.74	0.79	0.76	0.78	1.04	1
Solyc01g100810.2.1	Neutral invertase (AHRD V1 *	12.91	11.4	20.23	7.7	13.06	15.7	9.8	14.75	11.88	13.03	1	1	5.16	5.09	6.48	5.58	8.05	5.22	7.13	6.8	1.22	0.900689	6.28	9.21	6.91	7.47	7.75	6.5	7.72	7.32	0.98	1	13.05	12.09	13.81	12.98	6.56	6.37	6.46	0.5	0.457416
Solyc01g100820.2.1	AP-3 complex subunit mu-2 (	23.63	27.24	16.65	20.41	21.98	35.61	30.92	28.58	23.91	29.75	1.35	0.440401	24.19	22.47	15.14	20.6	15.73	20.45	19.82	18.67	0.91	1	19.35	19.77	13.69	17.6	14.27	14.54	15.25	14.69	0.83	1	11.95	14.51	14.88	13.78	15.97	14.74	15.36	1.11	1
Solyc01g100830.1.1	Galactinol synthase (AHRD V1	0	0	0	0	0	0	0	0	0	0	1	NA	0	0	0	0	0	0	0	0	1	NA	0	0	0	0	0	0	0	0	1	NA	0	0	0	0	0	0	0	1	NA
Solyc01g100840.2.1	Trafficking protein particle co	31.13	38.04	15.9	12.49	24.39	36.15	32.64	32.04	29.02	32.46	1.33	0.801363	32.41	28.27	15.7	25.46	12.89	23.33	34.32	23.51	0.92	1	38.66	18.93	14.76	24.12	14.65	21.13	17.94	17.91	0.74	1	15.85	27.33	33.58	25.59	14.03	16.29	15.16	0.59	0.873714
Solyc01g100850.2.1	ABC transporter ATP-binding	31.48	29.98	20.58	28.65	27.67	41.24	49.39	48.49	45.63	46.19	1.67	0.026021	26.67	22.31	16.77	21.92	22.53	22.11	28.84	24.49	1.12	0.983693	33.86	32.68	13.93	26.82	19.76	15.81	23.67	19.75	0.74	1	4.85	12.38	9.56	8.93	11.56	8.97	10.27	1.15	1
Solyc01g100860.2.1	ADP-ribosylation factor (AHR	2647.91	2351.46	2415.36	2401.09	2453.95	1527.08	1670.37	1497.74	2076.66	1692.96	0.69	0.264067	2352.78	2196.21	1952.37	2167.12	1968.02	1945.1	2215.08	2042.73	0.94	1	2041.16	2092.71	1734.92	1956.26	1975.95	1903.9	2044.99	1974.95	1.01	1	1563.12	1399.81	1410.22	1457.72	2114	2378.86	2246.43	1.54	0.692162
Solyc01g100870.2.1	ADP-ribosylation factor (AHR	1556.1	1331.22	1442.29	1432.91	1440.63	884.5	981.92	870.46	1230.3	991.8	0.69	0.272758	1364.28	1242.61	1145.05	1250.65	1160.38	1141.12	1340.59	1214.03	0.97	1	1132.91	1142.92	969.82	1081.88	1083.05	1077.76	1153.09	1104.63	1.02	1	764.61	724.86	685.13	724.87	1091.89	1242.93	1167.41	1.61	0.611808
Solyc01g100880.2.1	MtN21 nodulin protein-like (	0.55	1.31	2.82	0.26	1.23	0	0.29	0	0	0.07	0.06	0.227795	2.56	3.19	1.4	2.38	1.27	2.19	2.02	1.83	0.77	1	0.45	1.65	1.09	1.06	0.24	0.28	0.8	0.44	0.41	1	0	0.13	0	0.04	0	0	0	0.23	1
Solyc01g100890.1.1	MtN21 nodulin protein-like (	0	0.31	0	0	0.08	0.39	0.41	0	0.13	0.23	3	1	0.33	0.3	0.3	0.31	0	0.27	0	0.09	0.29	1	0	0	0.17	0.06	0	0	0	0	0.18	NA	0	0	0	0	0.16	0.15	0.15	15.5	1
Solyc01g100900.2.1	MtN21 nodulin protein-like (	4.47	8.59	0.84	0.76	3.66	6.16	6.35	13.97	4.88	7.84	2.14	0.686269	5.16	3.26	3.47	3.96	4.59	5.09	2.81	4.16	1.05	1	2.46	4.08	0.89	2.48	1.53	1.22	5.21	2.65	1.07	1	0.51	1.56	1.92	1.33	0.98	0.63	0.8	0.61	1
Solyc01g100910.1.1	MtN21 nodulin protein-like (	0.36	0.16	0	0	0.13	0	0	0.17	0.13	0.08	0.58	1	0.33	0	0	0.11	0	0.19	0	0.06	0.58	1	0.15	0.15	0	0.1	0	0	0	0	0.1	1	0	0	0	0	0	0	0	1	NA
Solyc01g100920.2.1	Nodulin-like protein (AHRD V	7.16	13.82	3.09	1.23	6.33	9.89	6.2	10.43	8.9	8.86	1.4	1	10.71	7.28	4.13	7.37	3.99	5.24	7.41	5.55	0.75	1	2.12	3.98	0	2.03	1.03	0.26	2.29	1.19	0.59	1	0.99	1.27	0.81	1.02	0.8	0.61	0.71	0.69	1
Solyc01g100930.2.1	GDSL esterase/lipase At1g09	22.88	27.62	21.76	18.06	22.58	41.59	29.26	26.09	28.9	31.46	1.39	0.371768	28.04	15.42	11.08	18.18	4.67	8.91	10.31	7.96	0.44	0.50507	1	2.02	0.35	1.12	0.12	0.4	1.16	0.56	0.5	1	0	0.26	0.27	0.18	0.16	0.31	0.23	1.33	1

Solyc01g100940.2.1	Unknown Protein (AHRD V1)	2.37	2.96	0.56	1.34	1.81	0.53	1.87	5.67	2.04	2.53	1.4	1	2.21	1.65	2	1.95	1.32	1.23	1.12	1.22	0.63	1	0.77	1.55	1.41	1.24	2.18	0.36	1.04	1.19	0.96	1	1.35	1.03	0	0.79	0.87	0	0.43	0.55	1
Solyc01g100950.2.1	Glucose-6-phosphate 1-dehyd	19.22	17.87	24.36	21.1	20.64	23.91	29.89	27.69	22.24	25.93	1.26	0.584808	19.48	17.7	18.41	18.53	11.74	17.08	13.98	14.27	0.77	0.915385	20.62	20.57	19.93	20.37	13	20	15.26	16.09	0.79	1	24.47	33.33	28.23	28.68	28.36	23.39	25.88	0.9	1
Solyc01g100960.2.1	Glucose-6-phosphate 1-dehyd	16.16	16.95	17.25	16.32	16.67	19.24	26.27	25.11	20.44	22.77	1.37	0.351557	17	14.71	13.81	15.17	11.07	13.94	9.98	11.66	0.77	0.907845	17.56	17.55	14.89	16.67	11.5	18.37	14.16	14.68	0.88	1	18.6	30.73	23.96	24.43	23.06	19.73	21.39	0.88	1
Solyc01g100970.2.1	Pyrrolidone-carboxylate pept	585.04	509.48	558.62	525.11	544.56	429.99	380.44	383.79	505.92	425.04	0.78	0.644387	502.64	554.97	551.64	536.42	647.45	461.44	526.93	545.27	1.02	1	222.4	238.73	239.31	233.48	230.48	237.73	212.62	226.94	0.97	1	478.79	389.64	361.52	409.98	397.75	405.95	401.85	0.98	1
Solyc01g100980.2.1	Polygalacturonase (AHRD V1	0	0	0	0	0	0	0	0	0	0	1	NA	0	0	0.12	0.04	0	0	0	0	0.25	1	0	0	0	0	0	0	0	0	1	NA	0	0	0	0	0	0.12	0.06	6	1
Solyc01g100990.2.1	Zinc finger matrin type 5 (AH	20.53	19.44	20.1	27.19	21.82	24.28	21.04	16.87	23.52	21.43	0.98	1	26.42	25.45	21.03	24.3	21.82	20.59	19.91	20.77	0.85	1	27.88	34.89	23.42	28.73	16.93	23.51	22.92	21.12	0.74	1	23.6	24.43	21.5	23.18	18.4	23.75	21.07	0.91	1
Solyc01g101000.2.1	Kinase family protein (AHRD \	333.4	323.64	313.23	273.8	311.02	329.28	256.73	297.66	303	296.67	0.95	1	348.13	381.01	338.95	356.03	353.55	358.07	396.93	369.52	1.04	1	440.17	391.77	385.39	405.78	395.33	416.6	409.1	407.01	1	1	350.71	310.63	305.08	322.14	358.51	340	349.25	1.08	1
Solyc01g101010.2.1	AGAP008431-PA (Fragment) (	15.27	19.45	24.26	22.02	20.25	17.53	12.61	21.56	17.27	17.24	0.85	0.965954	18.01	19.95	22.34	20.1	21.53	20.98	21.57	21.36	1.06	1	23.06	17.4	23.18	21.21	18.16	20.12	20.51	19.6	0.92	1	39.73	37.91	34.07	37.24	25.67	25.73	25.7	0.69	0.923889
Solyc01g101020.2.1	FACT complex subunit SPT16	23.16	24.35	30.61	33.73	27.96	15.59	17.46	16.81	20.8	17.66	0.63	0.175074	27.29	33.24	41.16	33.9	33.37	30.38	32.18	31.98	0.94	1	29.34	33.62	38.32	33.76	39.24	28.93	32.09	33.42	0.99	1	55.33	60.75	68.39	61.49	44.73	49.01	46.87	0.76	1
Solyc01g101030.2.1	FACT complex subunit SPT16	1.88	2.25	1.53	3.33	2.25	1.11	1.49	2.38	2.87	1.96	0.87	1	2.46	1.59	2.19	2.08	0.9	1.16	1.29	1.12	0.54	0.279786	1.76	2.11	1.46	1.78	0.97	1.12	0.64	0.91	0.51	0.78784	0.92	1.03	0.81	0.92	1.89	2.01	1.95	2.12	0.742285
Solyc01g101040.2.1	ATP-citrate lyase A-3 (AHRD \	158.55	158.83	148.69	166.6	158.17	136.11	169.76	130.47	139.63	143.99	0.91	1	156.89	131.74	123.01	137.21	86.22	98.28	89.14	91.21	0.66	0.209766	86.48	119.31	73.36	93.05	60.51	56.49	69.16	62.05	0.67	1	51.99	65.09	52.4	56.49	45.15	52.52	48.84	0.86	1
Solyc01g101050.2.1	Peroxidase 1 (AHRD V1 ****	0	0	0	0	0	0	0	0	0	0	1	NA	0	0	0	0	0	0	0	0	1	NA	0	0	0	0	0	0	0	0	1	NA	0	0.15	0	0.05	0	0	0	0.2	1
Solyc01g101060.2.1	S-adenosylmethionine syntha	2137.2	1853.21	3312.6	2899.66	2550.67	1527.13	1366.31	1137.53	1768.27	1449.81	0.57	0.148715	3147.99	2342.65	2867.07	2785.9	4702.78	3676.62	3659.79	4013.06	1.44	0.098117	289.35	615.21	391.9	432.15	834.21	477.3	505.88	605.8	1.4	1	1764.77	1090.13	1484.88	1446.59	1991.97	3523.85	2757.91	1.91	0.355355
Solyc01g101070.2.1	ABC transporter G family mer	4.6	6.57	7.69	6.57	6.36	6.13	5.14	6.53	5.66	5.87	0.92	1	9.58	9.23	10.31	9.71	8.25	9.48	9.74	9.16	0.94	1	4.6	5.46	5.16	5.07	3.53	2.76	3.78	3.36	0.66	1	6.87	5.2	4.48	5.52	3.71	5.19	4.45	0.81	1
Solyc01g101080.1.1	Ulp1 protease family C-termi	2.38	1.59	0.7	1.26	1.48	3.3	3.28	2.46	0.85	2.47	1.67	0.843786	1.38	2.07	1.76	1.74	0.82	2.62	2.8	2.08	1.2	1	1.2	0	0.29	0.5	0	0.9	0	0.3	0.6	1	0.42	0.22	0	0.21	1.09	0.26	0.68	3.16	1
Solyc01g101090.2.1	TBC1 domain family member	15.51	16.97	13.45	23	17.23	19.53	14.61	18.21	15.7	17.01	0.99	1	19.47	21.93	23.68	21.69	13.87	18.19	17.48	16.51	0.76	0.783417	27.03	23.3	46	32.11	33.64	27.62	20.97	27.41	0.85	1	15.94	39.09	33.62	29.55	41.91	29.49	35.7	1.21	1
Solyc01g101100.2.1	Receptor-like protein kinase (	29.03	25.95	26.61	39.05	30.16	21.9	28.96	22.17	25.5	24.63	0.82	0.935492	20.98	15.21	23.76	19.98	14.29	15.43	15.69	15.14	0.76	0.720252	11.2	11.93	13.51	12.21	9.88	9.53	11.24	10.22	0.84	1	8.85	8.85	10.56	9.42	12.93	14.43	13.68	1.45	0.945561
Solyc01g101110.2.1	Serine/threonine-protein kina	1.7	1.23	1.38	1.2	1.38	1.06	0.67	0.78	0.99	0.88	0.64	0.77103	1.38	1.38	0.99	1.25	0.96	0.99	0.75	0.9	0.72	1	0.26	0.17	0.32	0.25	0.28	0.16	0.62	0.35	1.41	1	0.6	0.62	0.33	0.52	0.58	0.74	0.66	1.28	1
Solyc01g101120.2.1	Glucan endo-1 3-beta-glucosi	12.19	6.38	9.52	4.56	8.16	14.15	5.52	7.25	5.28	8.05	0.99	1	5.34	5.92	13.32	8.19	7.34	7.05	3.66	6.02	0.73	1	3.91	2.92	7.65	4.83	1.18	0.95	1.18	1.1	0.23	0.12092	26.51	7.03	5.79	13.11	2.63	2.04	2.33	0.18	0.980812
Solyc01g101130.2.1	Plant IF-like protein (AHRD V1	0	0.53	0.23	0.28	0.26	0	0.16	0.73	0.85	0.43	1.67	1	0.73	0.69	0.33	0.58	1.37	0.92	0.31	0.87	1.49	1	1.76	0.97	2.55	1.76	2.08	0.9	1.87	1.62	0.92	1	9.83	11.19	9.11	10.04	9.22	7.1	8.16	0.81	1
Solyc01g101140.2.1	LOC100005105 protein (Frag	49	45.68	57.57	75.88	57.03	34.56	43.43	30.42	41.61	37.51	0.66	0.359418	57.84	81.35	84.56	74.58	84.86	57.4	62.69	68.32	0.92	1	63.77	71.45	77.62	70.95	70.13	71.17	70.58	70.63	1	1	88.1	87.03	77.79	84.31	65.31	73.27	69.29	0.82	1
Solyc01g101150.1.1	Pentatricopeptide repeat-con	0.99	2.42	0.58	1.92	1.48	1.92	1.07	1.59	0.98	1.39	0.94	1	0.8	1.83	1.77	1.47	1.37	2.24	2.43	2.01	1.37	0.911351	0.5	0.2	0.25	0.32	0.32	0.09	0.09	0.17	0.53	1	0.18	0.36	0.19	0.24	0.45	0.22	0.34	1.38	1
Solyc01g101160.2.1	Unknown Protein (AHRD V1)	41.61	41.2	46.88	49.04	44.68	44.37	42.17	38.43	41.44	41.6	0.93	1	49.68	47.25	46.06	47.66	54.99	46.58	36.09	45.89	0.96	1	38.01	42	36.83	38.95	41	36.65	42.34	40	1.03	1	47.43	46.96	49.57	47.99	38.43	35.99	37.21	0.78	1
Solyc01g101170.2.1	Alpha-humulene/(-)-(E)-beta-	0.12	0	0	0	0.03	0	0	0	0	0	0.33	1	0	0	0	0	0	0	0	0	1	NA	0	0.19	0	0.06	0	0	0	0	0.16	1	0	0	0	0	0	0	0	1	NA
Solyc01g101180.2.1	Alpha-humulene/(-)-(E)-beta-	0	0	0	0	0	0	0	0	0	0	1	NA	0	0	0	0	0	0	0	0	1	NA	0	0	0	0	0	0	0	0	1	NA	0	0	0	0	0	0	0	1	NA
Solyc01g101190.2.1	Alpha-humulene/(-)-(E)-beta-	0	0	0	0	0	0	0	0	0	0	1	NA	0	0	0	0	0	0	0	0	1	NA	0	0	0	0	0	0	0	0	1	NA	0	0	0	0	0	0	0	1	NA
Solyc01g101200.2.1	Unknown Protein (AHRD V1)	0	0	0	0	0	0	0	0	0	0	1	NA	0	0	0	0	0	0	0	0	1	NA	0	0	0	0	0	0	0	0	1	NA	0	0	0	0	0	0	0	1	NA
Solyc01g101210.2.1	Alpha-humulene/(-)-(E)-beta-	0	0	0	0	0	0	0	0	0	0	1	NA	0	0	0	0	0	0	0	0	1	NA	0	0	0	0	0	0	0	0	1	NA	0	0	0	0	0	0	0	1	NA
Solyc01g101220.1.1	Sesquiterpene synthase 1 (AH	0	0	0	0	0	0	0	0	0	0	1	NA	0	0	0	0	0	0	0	0	1	NA	0	0	0	0	0	0	0	0	1	NA	0	0	0	0	0	0	0	1	NA
Solyc01g101240.2.1	Aspartic proteinase (AHRD V1	201.04	228.25	201.56	205.41	209.06	303.89	201.96	276.96	250.73	258.38	1.24	0.556169	310.82	310.72	291.57	304.37	596.36	438.46	480.82	505.21	1.66	0.010496	103.86	155.65	81.63	113.71	122.32	88.56	132.56	114.48	1.01	1	81.77	90.92	75.03	82.57	97.2	193.84	145.52	1.76	0.715254
Solyc01g101250.2.1	D111/G-patch domain-contai	76.52	73.49	53.48	56.18	64.92	78.32	77.95	107.49	84.09	86.96	1.34	0.35925	74.1	63.78	50.41	62.76	37.97	55.77	51.84	48.53	0.77	0.796002	47.13	41.87	32.52	40.51	45.99	28.06	34.99	36.35	0.9	1	61.57	62.26	75.22	66.35	40.88	44.86	42.87	0.65	0.856201
Solyc01g101260.2.1	Unknown Protein (AHRD V1)	0	4.7	1.04	2.49	2.06	0.98	2.08	3.23	1.89	2.04	0.99	1	1.64	2.29	2.97	2.3	1.22	2.73	2.07	2.01	0.87	1	0	2.87	0	0.96	2.89	0.67	2.56	2.04	2.13	1	6.25	0.64	12.17	6.35	1.61	3.08	2.34	0.37	1
Solyc01g102260.2.1	Pheres1 (Fragment) (AHRD V1	0.79	0	0.62	1.87	0.82	0.29	0.42	0.49	0.38	0.4	0.48	1	0.98	0.69	1.34	1	2.01	2.6	2.7	2.44	2.43	0.463006	0	0.22	0	0.07	0.35	0	0	0.12	1.59	1	0.38	0.96	0.41	0.58	0.24	1.16	0.7	1.2	1
Solyc01g102270.2.1	Anaphase promoting complex	37.51	40.29	13.74	18.3	27.46	40.26	23.49	79.69	48.29	47.93	1.75	0.625191	21.66	22.49	14.2	19.45	8.07	18.08	35.58	20.58	1.06	1	10.45	10.54	0	7	5.95	5.88	11.31	7.71	1.1	1	1.84	0.94	0	0.93	4.74	6.81	5.78	6.23	0.942876
Solyc01g102280.2.1	Unknown Protein (AHRD V1)	21.99	24.91	7.08	8.48	15.61	22.22	17.36	27.57	22.96	22.53	1.44	0.930095	52.07	34.76	20.26	35.7	40.21	21.74	26.71	29.55	0.83	1	40.38	29.32	25.8	31.83	17.07	27.27	10.2	18.18	0.57	1	0	30.49	15.37	15.29	10.98	7.01	9	0.59	1
Solyc01g102290.2.1	Stress-induced hydrophobic p	223.38	518.12	120.16	96.59	239.56	450.28	202.85	626.78	354.8	408.68	1.71	0.804781	715.93	662.01	150.54	509.49	158.94	334.98	508.44	334.12	0.66	0.995707	612.47	153.73	276.86	347.69	269.15	328.08	150.97	249.4	0.72	1	343.92	762.44	815.13	640.5	262.75	306.46	284.61	0.44	0.460838
Solyc01g102300.2.1	BHLH transcription factor (AH	64.77	64.45	85.44	61.39	69.01	54.49	26.91	37.11	49.68	42.05	0.61	0.06085	42.6	69.6	81.48	64.56	84.55	61.78	58.14	68.16	1.06	1	23.23	22.6	19.66	21.83	25.6	24.16	23.37	24.38	1.12	1	52.4	35.74	35.28	41.14	51.96	57.6	54.78	1.33	0.975227
Solyc01g102310.2.1	Unknown Protein (AHRD V1)	89.48	86.43	106.4	118.26	100.14	75.67	99.53	97.2	95.17	91.89	0.92	1	58.43	73.44	137.67	89.85	64.25	83.12	77.42	74.93	0.83	1	3.26	6.15	3.31	4.24	6.74	3.26	4.6	4.87	1.15	1	233.12	106.41	146.77	162.1	126.48	220.06	173.27	1.07	1
Solyc01g102320.1.1	Unknown Protein (AHRD V1);	0	0	0	0	0	0	0	0	0	0	1	NA	0	0	0	0	0	0	0	0	1	NA	0	0	0	0	0	0	0	0	1	NA	0	0	0	0	0	0	0	1	NA
Solyc01g102330.2.1	Acetyl xylan esterase A (AHRI	299.45	237.47	214.4	314.98	266.57	222.45	386.84	170.92	259.52	259.93	0.98	1	241.58	259.45	260.17	253.73	171.97	228.06	194.66	198.23	0.78	0.752529	97.09	193.05	73.76	121.3	135.33	86.62	152.8	124.92	1.03	1	145.75	114.53	96.92	119.07	136.27	178.04	157.16	1.32	1
Solyc01g102340.2.1	Myb-like transcription factor	16.07	17.05	24.61	27.54	21.32	15.97	30.86	19.89	13.31	20.01	0.94	1	12.5	16.92	22.58	17.33	9.75	10.16	16.39	12.1	0.7	0.657526	0.75	1.13	0.31	0.73	0.41	0	0.11	0.17	0.24	1	1.54	2.36	2.38	2.09	1.98	1.9	1.94	0.93	1
Solyc01g102350.2.1	Pectinacetylesterase like prot	3.81	8.89	2.25	2.69	4.41	11.28	6.76	6.71	6.6	7.84	1.78	0.56717	14.75	15.3	12.19	14.08	23.65	15.93	14.71	18.1	1.29	0.698541	4.36	7.24	1.73	4.44	4.79	2.76	2.54	3.36	0.76	1	25.03	15.32	17.56	19.3	33.53	42.55	38.04	1.97	0.219768
Solyc01g102360.1.1	Peptide transporter-like prote	0	0	0	0	0	0	0	0	0	0	1	NA	0	0	0	0	0	0	0	0	1	NA	0	0	0	0	0	0	0	0	1	NA	0	0	0	0	0	0	0	1	NA
Solyc01g102370.2.1	Flap structure specific endonu	25.66	23.3	30.28	25.34	26.15	21.44	22.71	28.35	19.29	22.95	0.88	1	23.55	26.05	28.08	25.89	16.13	20.33	16.79	17.75	0.69	0.447793	10.58	17.4	10.94	12.97	8.03	9.92	11.8	9.92	0.76	1	13.96	19.52	20.13	17.87	20.5	22.05	21.27	1.19	1
Solyc01g102380.2.1	Germin-like protein (AHRD V1	0	0	0	0	0	0	0	0	0	0	1	NA	0	0	0	0	0	0	0	0	1	NA	0	0	0	0	0	0	0	0	1	NA	0	0	0	0	0	0	0	1	NA
Solyc01g102390.2.1	Germin-like protein (AHRD V1	22.39	19.39	11.97	25.33	19.77	10.29	16.73	15.92	10.07	13.25	0.67	0.510093	11.66	19.72	17.14	16.17	9.73	8.81	5.16	7.9	0.49	0.079455	12.78	8.03	8.59	9.8	8.43	8.6	7.4	8.14	0.83	1	2.97	5.42	3.67	4.02	5.47	6.28	5.88	1.46	1
Solyc01g102400.2.1	Germin-like protein (AHRD V1	16.12	12.49	9.86	17.31	13.95	4.65	9.19	9.88	7.07	7.7	0.55	0.226771	8.05	16.09	13.87	12.67	8.7	9.58	4.22	7.5	0.59	0.619503	10.85	8.76	9.48	9.7	7.65	6.56	6.09	6.77	0.7	1	2.55	5.85	3.21	3.87	5.74	7.07	6.41	1.66	1
Solyc01g102410.2.1	Glutaminyl-tRNA synthetase (	35.26	34.71	56.4	51.63	44.5	20.58	27.27	20.79	28.63	24.32	0.55	0.083422	36.36	43.65	55.42	45.14	49.65	43.1	38.06	43.6	0.97	1	41.21	47.88	59.21	49.43	68.2	58.3	52.03	59.51	1.2	1	577.47	193.41	274.63	348.5	205.38	230.59	217.99	0.63	1
Solyc01g102420.1.1	Unknown Protein (AHRD V1)	0	0	0.71	0	0.18	0.67	0	0	0	0.17	0.94	1	0	0	0	0	0	0	0	0	1	NA	0	0	0	0	0	0	0	0	1	NA	0	0	0	0	0	0	0	1	NA
Solyc01g102430.2.1	Unknown Protein (AHRD V1)	0	0	0	0	0	0	0	0	0	0	1	NA	0	0	0	0	0	0	0	0	1	NA	0	0	0	0	0	0	0	0	1	NA	0	0	0	0	0	0	0	1	NA
Solyc01g102440.2.1	Organic solute transporter-lik	7.27	5.09	6.13	6.61	6.27	5.2	5.74	4.78	7.46	5.8	0.92	1	4.83	4.06	3.51	4.13	6.12	4.3	0.82	3.75	0.91	1	1.26	4.66	7.22	4.38	4.44	4.72	2.27	3.81	0.87	1	6.65	4.53	2.4	4.53	6.66	10.02	8.34	1.84	1
Solyc01g102450.2.1	Transmembrane protein 34 (A	1.77	4.72	1.39	0.83	2.18	2.62	0.93	3.79	4.23	2.89	1.33	1	1.1	1.54	1.49	1.38	2.45	2.44	3.24	2.71	1.97	0.918057	1.43	0.48	1.75	1.22	0.39	0.89	2.15	1.14	0.94	1	2.51	1.28	0	1.26	1.62	3.62	2.62	2.07	1
Solyc01g102460.2.1	Unknown Protein (AHRD V1)	15.03	20.07	15.27	17.13	16.88	11.14	17.47	30.33	17.39	19.08	1.13	0.974779	12.82	15.24	11.99	13.35	13.61	24	10.83	16.15	1.21	0.925402	15.52	20.07	13.26	16.28	18.65	14.87	19.78	17.77	1.09	1	7.72	23.65	21.83	17.73	12.61	12.81	12.71	0.72	1
Solyc01g102470.2.1	Pentatricopeptide repeat-con	6.11	5.8	6.25	5.76	5.98	6.34	7.39	8.24	7.89	7.46	1.25	0.781689	5.3	6.19	6.54	6.01	7.63	6.54	6.88	7.02	1.17	1	4.77	7.13	4.45	5.45	11.1	5.86	9.35	8.77	1.61	0.673384	4.34	6.8	7.2	6.11	12.49	10.53	11.51	1.88	0.737405
Solyc01g102480.2.1	Transmembrane protein 34 (A	20.46	24.77	20.67	19.68	21.4	24.57	19	20.47	18.33	20.59	0.96	1	22.08	20.92	19.08	20.69	20.1	21.7	24.06	21.95	1.06	1	13.89	14.94	21.77	16.87	13.55	14.39	18.97	15.64	0.93	1	23.74	30.91	26.43	27.03	19.78	21.96	20.87	0.77	1
Solyc01g102490.2.1	NADPH adrenodoxin oxidored	17.33	17.07	21.84	33.33	22.39	18.33	17.01	21.35	18.97	18.91	0.84	1	21.45	36.43	33.12	30.33	20.08	21.96	21.82	21.29	0.7	0.432873	21.48	23.31	49.61	31.47	28.3	25.38	17.41	23.7	0.75	1	111.74	108.42	138.45	119.54	72.56	64.48	68.52	0.57	0.586532
Solyc01g102500.2.1	Genomic DNA chromosome 5	24.72	23.39	27.52	27.08	25.68	21.86	25.01	20.65	17.75	21.32	0.83	0.920738	21.21	29.2	25.29	25.23	17.87	15.5	15.73	16.37	0.65	0.253066	22.64	26.1	21.94	23.56	25.61	17.41	24.61	22.54	0.96	1	9.16	16.81	13.12	13.03	11.47	14.96	13.21	1.01	1
Solyc01g102510.2.1	Genomic DNA chromosome 5	15.31	15.96	26.58	28.87	21.68	11.6	16.44	9.3	13.83	12.79	0.59	0.433037	18.17	21.14	26.99	22.1	20.25	16.16	14.56	16.99	0.77	0.67425	14.11	19.18	19.26	17.52	13.71	12.09	14.77	13.52	0.77	1	34.58	16.16	17.96	22.9	23.95	28.62	26.29	1.15	1
Solyc01g102520.2.1	MLO-like protein 1 (AHRD V1	1.52	1.23	0	0	0.69	0.92	0.55	0.76	1	0.81	1.17	1	1.29	0.12	0	0.47	0.58	0.22	0.22	0.34	0.72	1	0.11	0.11	0.14	0.12	0.18	0.11	0.4	0.23	1.92	1	0	0	0	0	0	0	0	1	NA
Solyc01g102530.2.1	SNARE associated Golgi prote	14.71	23.09	12.05	10.98	15.21	19.3	9.84	17.28	13.2	14.91	0.98	1	16.72	15.8	13.11	15.21	8.07	10.47	11.56	10.03	0.66	0.46554	17.33	13.15	12.37	14.28	11.94	9.44	12.5	11.29	0.79	1	5.23	10.83	10.36	8.81	7.48	5.37	6.43	0.73	1
Solyc01g102540.2.1	Nucleoporin (AHRD V1 *-*- C	7.75	9.87	13.22	11.97	10.7	9.14	9.08	10.85	8.43	9.38	0.88	1	9.48	9.32	11.02	9.94	9.63	10.09	11.8	10.51	1.06	1	5.1	5.73	8.81	6.55	4.27	5.1	5.73	5.03	0.77	1	8.3	8.79	8.8	8.63	8.78	10.14	9.46	1.1	1
Solyc01g102550.2.1	Nucleoporin-like protein (AH	5.84	7.93	9.56	9.86	8.3	3.42	6.01	5.22	8.84	5.87	0.71	0.803901	6.33	10.57	10.4	9.1	9.44	10.15	8.41	9.33	1.03	1	7.07	5.15	6.44	6.22	5.54	4.05	4.96	4.85	0.78	1	5.76	4.71	6.23	5.57	6.97	11.8	9.38	1.69	0.780653
Solyc01g102560.2.1	Eukaryotic translation initiati	64.91	49.46	95.85	81.91	73.03	36.78	41.87	36.86	53.3	42.2	0.58	0.216261	59.39	61.96	91.91	71.09	80.48	58.54	61.18	66.73	0.94	1	37.53	53.58	62.96	51.36	56.02	49.36	50.7	52.03	1.01	1	94.1	76.88	104.38	91.79	95.93	109.92	102.93	1.12	1
Solyc01g102570.2.1	Eukaryotic translation initiati	230.9	187.52	296.68	287.29	250.6	130.39	174.54	145.34	187.32	159.4	0.64	0.174974	227.71	218.76	297.07	247.85	251.02	223.08	191.94	222.01	0.9	1	159.75	194.64	224.96	193.12	234.34	181.61	190	201.98	1.05	1	479.79	351.29	407.21	412.76	406.62	443.41	425.01	1.03	1
Solyc01g102580.2.1	Endo-1 4-beta-glucanase (AH	35.65	34.3	37.91	59.19	41.76	27.89	33.2	23.75	28.66	28.38	0.68	0.530724	34.06	38.14	46.25	39.48	35.61	32.06	24.08	30.58	0.77	0.737351	28.03	32.79	40.26	33.69	31.14	30.42	23.57	28.38	0.84	1	158.45	73.35	80.11	103.97	88.92	96.03	92.47	0.89	1
Solyc01g102590.2.1	Eukaryotic translation initiati	42.05	33.71	54.92	47.68	44.59	28.37	34.47	24.79	35.95	30.89	0.69	0.441284	43.45	39.63	50.57	44.55	45.42	36.98	36.83	39.74	0.89	1	29.57	38.45	49.39	39.14	43.76	40.15	38.25	40.72	1.04	1	56.6	58.99	75.53	63.71	63.33	69.95	66.64	1.05	1
Solyc01g102600.2.1	Eukaryotic translation initiati	55.07	41.61	78.05	60.38	58.78	21.36	33.85	33.81	45.37	33.6	0.57	0.232011	56.21	49.64	72.89	59.58	63.57	58.56	48.94	57.02	0.96	1	37.17	45.63	47.51	43.44	64.7	40.76	53.46	52.97	1.22	1	66.74	76.1	85.82	76.22	86.95	96.57	91.76	1.2	1
Solyc01g102610.2.1	Ferric reductase oxidase (AHR	49.41	64.18	73.53	24.68	52.95	154.38	69.02	164.53	124.38	128.08	2.42	0.035509	65.59	50.46	36.47	50.84	47.61	43.7	52.47	47.93	0.94	1	128.32	52.89	31.81	71.01	61.18	61.27	49.76	57.4	0.81	1	1.77	4.33	1.78	2.63	10.68	5.39	8.04	3.06	0.658256
Solyc01g102620.1.1	Unknown Protein (AHRD V1)	2	0	0.79	0.94	0.93	1.48	1.05	1.84	0	1.09	1.17	1	0	3.48	1.13	1.54	0.46	0	0.52	0.33	0.21	0.863933	1.08	0	0	0.36	0.44	0.51	0	0.32	0.88	1	0	0	1.02	0.34	0.61	0	0.3	0.9	1
Solyc01g102630.2.1	Unknown Protein (AHRD V1)	0.23	0	0	0.32	0.14	0.51	0	0	0	0.13	0.93	1	0.21	0.4	0.39	0.33	0	0	0.18	0.06	0.18	0.911351	0	0	0.23	0.08	0.15	0	0	0.05	0.65	1	0	0	0.7	0.23	0	0.2	0.1	0.43	1
Solyc01g102640.2.1	Nucleoporin (Fragment) (AHR	1.22	5.44	1.44	0	2.02	2.72	1.93	5.62	3.51	3.44	1.7	1	5.68	3.19	7.22	5.36	0.85	1.9	0.96	1.24	0.23	0.553579	1.97	3.98	1.21	2.39	0	0.93	0.89	0.61	0.25	1	3.47	1.77	0	1.75	1.12	1.07	1.1	0.63	1
Solyc01g102650.2.1	Unknown Protein (AHRD V1)	0.94	1.68	2.22	0	1.21	1.05	0.74	1.73	0.68	1.05	0.87	1	3.51	6.55	2.39	4.15	3.27	3.42	3.7	3.46	0.83	1	1.52	1.54	5.61	2.89	1.24	1.43	0.69	1.12	0.39	1	2.68	0	0	0.89	0.86	0	0.43	0.48	1
Solyc01g102660.2.1	Maleylacetoacetate isomeras	1125.06	1012.09	876.4	1052.84	1016.6	912.21	1274.51	767.31	991	986.26	0.97	1	1366.54	1209.18	1176.13	1250.62	1257.58	1154.62	1105.48	1172.56	0.94	1	1445.27	1306.82	1341.65	1364.58	1316.48	1494.44	1166.71	1325.88	0.97	1	1012.85	1021.04	742.43	925.44	1165.56	1046.35	1105.95	1.2	1
Solyc01g102670.2.1	HhH-GPD (AHRD V1 *--- Q2IN	6	5.1	7.72	7.71	6.63	6.06	3.98	4.64	5.19	4.97	0.75	0.812718	6.59	6.52	5.64	6.25	9.83	5.65	8.14	7.87	1.26	0.844566	2.53	3.22	2.71	2.82	3.49	3	4.17	3.55	1.26	1	6.78	5.15	7.96	6.63	4.37	5.26	4.81	0.73	1
Solyc01g102680.2.1	Receptor like kinase, RLK	0.14	0	0.08	0.1	0.08	0.08	0.11	0	0	0.05	0.59	1	0.13	0	0	0.04	0	0.07	0	0.02	0.54	1	0	0	0	0	0.05	0	0	0.02	1.67	1	0	0	0	0	0	0	0	1	NA
Solyc01g102690.2.1	Pentatricopeptide repeat-con	2.09	0.97	2.56	2.36	1.99	1.3	2.31	1.23	0.84	1.42	0.71	0.917957	1.55	1.31	1.97	1.61	2.49	2.16	2.3	2.32	1.44	0.70879	0.61	0.88	0.99	0.83	0.6	0.95	0.79	0.78	0.94	1	1.42	0.97	1.28	1.22	1.15	1.54	1.34	1.1	1
Solyc01g102700.2.1	Receptor like kinase, RLK	3.87	2.3	2.51	5.29	3.49	0.93	1.53	1.78	1.57	1.45	0.42	0.165925	0.96	4.48	3.81	3.08	1.7	1.6	1.47	1.59	0.52	0.728754	1.77	3	3.9	2.89	2.58	1.12	0.66	1.45	0.5	1	3.58	3.37	3.17	3.37	0.89	1.75	1.32	0.39	0.322514
Solyc01g102710.2.1	Receptor like kinase, RLK	0.42	0.56	0.16	0.29	0.36	0.31	0.27	0.25	0.35	0.3	0.83	1	0.06	0.36	0.12	0.18	0.29	0.25	0.22	0.25	1.41	1	0.06	0	0	0.02	0.14	0	0.1	0.08	4	1	0	0.1	0	0.03	0	0	0	0.3	1
Solyc01g102720.2.1	START domain containing 10	1.64	2.44	1.61	1.55	1.81	1.83	2.16	1.76	0.98	1.68	0.93	1	1.53	2.38	1.16	1.69	0.38	0.57	0.43	0.46	0.27	0.434894	0.44	0	0	0.15	0.18	0	0	0.06	0.41	1	0	0.2	0	0.07	0	0.24	0.12	1.8	1
Solyc01g102730.2.1	Cinnamoyl-CoA reductase fan	12.51	7.54	19.28	16.27	13.9	10.32	13.54	9.09	11.88	11.21	0.81	1	8.44	9.47	10.91	9.61	8.54	6.37	4.28	6.4	0.67	0.614744	3.59	5.1	7.41	5.37	3.58	3.67	3.38	3.54	0.66	1	2.87	2.78	1.86	2.5	4.62	4.42	4.52	1.81	1
Solyc01g102740.2.1	NAC domain protein IPR0034	0	0.28	0	0	0.07	0.18	0	0.44	0.11	0.18	2.61	1	0.15	0.28	0.27	0.23	0.66	0.17	0	0.28	1.19	1	0.26	0.13	0.16	0.18	0.63	0.24	0.12	0.33	1.8	1	0	0	0.25	0.08	0.29	0.42	0.35	4.26	1
Solyc01g102750.2.1	PHD finger family protein (AH	53.3	50.49	53.86	45.16	50.7	65.71	49.58	73.76	60.62	62.42	1.23	0.62951	55.47	55.28	56.61	55.79	70.65	72.41	72.17	71.74	1.29	0.490742	117.9	88.38	124.94	110.41	105.75	113	89.03	102.59	0.93	1	70.54	94.11	84	82.88	93.73	71.72	82.72	1	1
Solyc01g102760.2.1	PHD finger family protein (AH	51.4	62.25	69.7	58.46	60.45	66.26	52.08	63.09	55.16	59.15	0.98	1	62.35	55.27	60.42	59.35	47.78	57.34	49.55	51.56	0.87	1	79.63	56.78	74.42	70.28	70.49	52.61	52.9	58.67	0.83	1	52.74	69.08	72.11	64.64	43.41	45.76	44.58	0.69	0.926881
Solyc01g102770.1.1	Photosystem II reaction cente	2.09	5.59	2.47	0	2.54	13.97	4.13	2.89	6.01	6.75	2.66	0.741275	1.95	0	0.88	0.94	3.63	3.8	5.76	4.4	4.66	0.498747	0.85	0.85	0	0.57	2.06	3.97	2.29	2.77	4.89	1	5.96	3.8	3.22	4.33	0.96	0	0.48	0.11	0.92427
Solyc01g102790.2.1	Pentatricopeptide repeat-con	1.56	1.7	0.62	1.23	1.28	0.97	0.89	0.32	1.12	0.82	0.65	0.745821	0.97	0.76	1.32	1.02	1.33	1.13	0.48	0.98	0.96	1	0.98	0.64	0.52	0.71	0.46	0.79	0.95	0.73	1.03	1	0.49	1.58	1.6	1.22	1.11	0.91	1.01	0.83	1
Solyc01g102800.2.1	Histidyl-tRNA synthetase (AH	13.63	13.6	16.08	14.72	14.51	13.23	12.06	11.43	13.71	12.61	0.87	1	15.46	14.06	14.23	14.58	13.19	12.1	14.89	13.39	0.92	1	16.03	17.02	17.84	16.96	13.48	12.94	15.44	13.95	0.82	1	11.5	10.82	11.17	11.16	15.49	14.84	15.16	1.36	1
Solyc01g102810.2.1	DNA polymerase III gamma a	4.09	4.42	5.25	7.18	5.23	2.48	4.7	3.19	4.05	3.6	0.69	0.678531	3.03	3.87	7.17	4.69	3.56	3.91	4.68	4.05	0.86	1	0.67	1.31	0.36	0.78	1.53	0.55	1.06	1.05	1.34	1	0.96	1.54	1.11	1.2	0.81	1.86	1.33	1.11	1
Solyc01g102820.2.1	2-C-methyl-D-erythritol 4-ph	32.77	33.55	23	23.63	28.24	36.67	40.41	41.55	40.13	39.69	1.41	0.291608	22.71	23.65	18.09	21.48	18.42	21.07	27.44	22.31	1.04	1	6.58	12.74	9.35	9.56	10.55	11.36	10.77	10.89	1.14	1	3.05	5.91	5.27	4.74	8.82	7.32	8.07	1.7	0.993057
Solyc01g102830.2.1	Unknown Protein (AHRD V1)	469.13	472.97	277.82	382.34	400.56	378.88	495.51	401.85	376.27	413.13	1.03	1	379.27	356.03	251.52	328.94	254.2	237.21	261.38	250.93	0.76	0.665923	388.64	382.28	240.1	337.01	206.46	266.67	294.45	255.86	0.76	1	191.5	312.78	229.66	244.65	227.3	208.07	217.69	0.89	1
Solyc01g102840.2.1	Tir-nbs, resistance protein fra	3.36	3.28	2.56	6.75	3.99	2.17	3.08	2.19	1.17	2.15	0.54	0.570216	2.62	3.4	5.41	3.81	3.24	2.98	2.9	3.04	0.8	0.983079	7.36	5.31	15.08	9.25	5.06	5.84	5.06	5.32	0.58	1	21.29	27.27	19.69	22.75	10.43	5.9	8.16	0.36	0.027473
Solyc01g102850.1.1	Tir-nbs-lrr, resistance protein	0	0	0	0.08	0.02	0	0	0	0	0	0.5	1	0	0	0	0	0	0	0	0	1	NA	0	0	0	0	0	0	0	0	1	NA	0	0	0	0	0	0	0	1	NA
Solyc01g102860.2.1	Unknown Protein (AHRD V1)	24.65	29.68	24.23	22.9	25.37	52.38	40.25	35.77	38.51	41.73	1.65	0.062454	18.43	20.38	21.23	20.01	45.42	35.78	45.77	42.32	2.11	0.001272	4.49	8.86	4.08	5.81	6.67	4.4	11.8	7.62	1.31	1	0.34	0.53	0.37	0.41	0.22	0.21	0.21	0.52	1
Solyc01g102870.1.1	LRR receptor-like serine/thre	0.08	0.29	0.1	0.12	0.15	0	0.52	0.15	0.3	0.24	1.64	0.985318	0.46	0.43	0.14	0.34	0.17	0.26	0.45	0.29	0.85	1	0.2	0.13	0.08	0.14	0.05	0.06	0	0.04	0.27	1	0.12	0.3	0.25	0.22	0.15	0.07	0.11	0.49	1
Solyc01g102880.1.1	Tir-nbs-lrr, resistance protein	1.93	1.65	3.16	3.59	2.58	0.87	1.21	1.91	1.13	1.28	0.5	0.27678	1.73	2.92	3.8	2.82	1.98	2.16	1.94	2.03	0.72	0.813188	1.36	1.37	1.63	1.45	0.96	0.6	0.7	0.75	0.52	1	1.37	1.4	1.92	1.56	0.62	0.94	0.78	0.5	0.76924
Solyc01g102890.2.1	Germin-like protein 4 (AHRD	0	0	0	0	0	0	0	0	0	0	1	NA	0	0	0	0	0	0	0	0	1	NA	0	0	0	0	0	0	0	0	1	NA	0	0.42	0	0.14	0.26	0.25	0.26	1.82	1
Solyc01g102900.2.1	Germin-like protein 4 (AHRD	0	0	0	0	0	0	0	0	0	0	1	NA	0	0	0	0	0	0	0	0	1	NA	0	0	0	0	0	0	0	0	1	NA	0	0	0	0	0	0	0	1	NA
Solyc01g102910.2.1	Germin-like protein 4 (AHRD	0	0	0	0	0	0	0	0	0	0	1	NA	0	0	0	0	0	0	0	0	1	NA	0	0	0	0	0	0	0	0	1	NA	0	0.42	0	0.14	0	0.25	0.12	0.89	1
Solyc01g102920.2.1	Tir-nbs, resistance protein fra	0	0	0	0	0	0	0	0	0	0	1	NA	0	0	0	0	0	0	0	0	1	NA	0	0	0	0	0	0	0	0	1	NA	0	0	0	0	0	0	0	1	NA
Solyc01g102930.1.1	Lrr, resistance protein fragm	0	0	0	0	0	0	0	0	0	0	1	NA	0	0	0	0	0	0	0	0	1	NA	0	0	0	0	0	0	0	0	1	NA	0	0	0	0	0	0	0	1	NA
Solyc01g102940.2.1	Methionine s-methyltransfera	10.1	10.83	14	13.69	12.15	11.5	5.48	12.37	11.19	10.13	0.83	0.927034	7.47	5.66	5.29	6.14	5.19	3.72	6.31	5.07	0.83	0.928227	3.88	3.87	2.96	3.57	1.32	0.65	2.39	1.45	0.41	1	0.17	0.04	0	0.07	0.17	0.16	0.17	2.36	1
Solyc01g102950.2.1	FAD dependent oxidoreducta	2.49	3.18	4.47	2.14	3.07	24.16	12.38	19.79	14.13	17.61	5.74	1.31E-06	1.21	1.41	1	1.21	2.18	2.91	2.8	2.63	2.18	0.132927	1.31	0.53	0.64	0.83	3.06	2.38	3.15	2.86	3.46	0.019074	0.31	0.08	0	0.13	0.2	0.47	0.33	2.58	1
Solyc01g102960.2.1	class IV heat shock protein (A	9.03	9.27	2.46	15.72	9.12	12.35	59.21	24.26	136.85	58.17	6.38	0.30082	20.02	103.83	32.26	52.04	72.01	35.78	16.92	41.57	0.8	1	1051.94	1115.33	733.06	966.78	452.32	449.75	1194.77	698.95	0.72	1	32.09	88.01	29.37	49.82	88.04	31.66	59.85	1.2	1
Solyc01g102970.2.1	RNA-binding protein (AHRD V	15.24	16.03	17.07	15.14	15.87	16.77	15.5	16.92	17.52	16.68	1.05	1	19.83	18.34	20.42	19.53	19.49	20.85	19.66	20	1.02	1	8.71	12.86	8.86	10.14	13.86	10.27	12.12	12.08	1.19	1	19.92	17.22	18.94	18.69	15.78	23.89	19.84	1.06	1
Solyc01g102980.2.1	Zinc finger-homeodomain pro	0	0	0	0.32	0.08	0	0.18	0	0	0.04	0.56	1	0	0	0	0	0	0	0	0	1	NA	0	0	0	0	0	0	0	0	1	NA	0	0	0.35	0.12	0	0	0	0.09	1
Solyc01g102990.2.1	ATP-dependent Clp protease	13.46	13.73	11.97	12.01	12.79	11.86	13.16	15.14	12.71	13.22	1.03	1	13.29	12.24	14.78	13.44	11.78	13.03	11.23	12.01	0.89	1	8.62	9.28	8.84	8.91	8.91	8.48	8.68	8.69	0.97	1	15.02	14.52	16.4	15.31	12.61	12.99	12.8	0.84	1
Solyc01g103000.1.1	Unknown Protein (AHRD V1)	0	3.79	2.51	1.51	1.95	2.37	0.84	4.89	3.06	2.79	1.43	1	0	0	0	0	0	0	0	0	1	NA	0	0	0	0	0	0	0	0	1	NA	0	0	0	0	0	0	0	1	NA

Solyc01g103010.2.1	Cullin-associated NEDD8-diss	26.16	29.21	25.11	23.21	25.92	25.41	23.52	24.05	29.2	25.55	0.99	1	28.16	25	26.49	26.55	29.68	29.73	33.75	31.05	1.17	0.795604	20.92	23.92	23.76	22.87	22.99	22.58	23.44	23	1.01	1	21.49	22.7	22.74	22.31	28.06	34.81	31.44	1.41	0.936669
Solyc01g103020.2.1	Survival of motor neuron-rela	58.49	62.43	64.34	60.03	61.32	53.78	44.1	65.1	51.31	53.57	0.87	0.977921	61.77	60.79	62.96	61.84	52.62	51.01	53.75	52.46	0.85	0.976242	47.44	41.21	39.07	42.57	42.47	36.33	30.29	36.36	0.85	1	47.21	26.51	34.15	35.96	32.21	34.13	33.17	0.92	1
Solyc01g103030.2.1	Nitrate transporter (AHRD V1	0.33	0	0	0	0.08	0	0	0.1	0.23	0.08	1	1	0.61	0.57	0.64	0.61	1.81	0.9	1.11	1.27	2.1	0.517564	2.11	0.71	1.29	1.37	1.43	1.65	1.03	1.37	1	1	0	0	0	0	0	0.1	0.05	5	1
Solyc01g103040.2.1	Mitotic checkpoint protein M	6.12	6.83	8.09	6.72	6.94	5.18	5.7	6.13	7.74	6.19	0.89	1	7.99	8.42	6.41	7.61	6.15	7.43	8.76	7.45	0.98	1	3.54	5.14	3.27	3.98	4.92	4.36	4.33	4.54	1.14	1	5.85	9.62	8.15	7.87	6.52	6.81	6.67	0.85	1
Solyc01g103050.2.1	Auxin response factor 1 (AHR	52.58	58.71	62.62	70.66	61.14	32.58	53.91	42.51	49.03	44.51	0.73	0.540931	68.11	76.87	86.18	77.05	60.43	57.78	64.11	60.77	0.79	0.70879	19.12	16.58	15.8	17.17	22.5	23.74	17.84	21.36	1.24	1	77.62	52.3	62.42	64.11	48.04	75.77	61.91	0.97	1
Solyc01g103060.2.1	Non-specific lipid-transfer pro	0	0	0	0.58	0.14	0	0	0	0.29	0.07	0.5	1	1.14	0	0	0.38	0	0.21	0	0.07	0.18	1	0.33	0	0	0.11	0	0	0	0	0.09	1	0	0	0	0	0	0	0	1	NA
Solyc01g103070.2.1	Unknown Protein (AHRD V1)	0	0.36	0	0	0.09	0	0	0	0	0	0.11	1	0	0	0	0	0	0.21	0	0.07	7	1	0	0	0	0	0	0	0	0	1	1	0	0	0	0	0	0	0	1	NA
Solyc01g103080.2.1	ATP-dependent RNA helicase	28.93	32.14	40.8	29.63	32.88	24.99	19.16	29.04	24.54	24.43	0.74	0.472509	36.44	29.95	40.95	35.78	49.45	48.79	49.28	49.17	1.37	0.223171	17.46	17.99	20.66	18.7	24.95	18.04	25.98	22.99	1.23	1	45.86	42.89	51.64	46.8	36.83	43.56	40.2	0.86	1
Solyc01g103090.2.1	Splicing factor U2af subunit (	13.09	12.55	11.49	10.21	11.84	9.52	11	15.74	10.79	11.76	0.99	1	11.16	12.11	14.82	12.7	9.66	12.04	12.33	11.34	0.89	1	8.88	8.28	7.16	8.11	9.7	9.1	8.56	9.12	1.12	1	15.99	17.25	24.01	19.08	8.91	9.42	9.16	0.48	0.32208
Solyc01g103100.2.1	Short-chain dehydrogenase/r	19.96	17.39	14.87	18.13	17.59	25.22	30.19	18.54	24.44	24.6	1.4	0.298528	21.3	19.18	14.37	18.28	15.15	18.17	14.36	15.89	0.87	1	13.75	15.41	11.05	13.4	11.04	13.22	11.79	12.02	0.9	1	11.95	15.11	10.02	12.36	17.12	15.47	16.3	1.32	1
Solyc01g103110.2.1	Unknown Protein (AHRD V1);	13.99	18.36	20.67	11.39	16.1	32.98	21.08	35.2	24.34	28.4	1.76	0.037218	15.24	18.3	14.32	15.95	29.49	26.92	28.42	28.28	1.77	0.015065	12.67	15.08	12.9	13.55	15.41	17.35	17.09	16.62	1.23	1	17.88	34.06	29.76	27.23	24.05	25.89	24.97	0.92	1
Solyc01g103120.2.1	Dynamin-2A (AHRD V1 ***- B	1.78	1.19	0.79	1.58	1.33	1.98	2.29	1.64	1.76	1.92	1.44	0.901986	0.83	0.97	1.32	1.04	0.31	0.69	0	0.33	0.32	0.755535	0.18	0.36	0.44	0.33	0.15	0	0.16	0.1	0.32	1	0	0.16	0	0.05	0	0.2	0.1	1.88	1
Solyc01g103130.1.1	Dynamin-2A (AHRD V1 ***- B	0.76	0.87	0.13	1.23	0.75	0.36	1.11	0.6	1.25	0.83	1.11	1	0.71	0.38	0.37	0.49	0.53	0.34	0.34	0.4	0.83	1	0.44	0.8	0	0.41	0	0	0.24	0.08	0.19	1	0	0	0	0	0	0.1	0.05	5	1
Solyc01g103140.2.1	Voltage-gated chloride chann	52.54	59.32	54.06	49.06	53.75	63.88	72.96	62.14	76.41	68.85	1.28	0.380274	71.8	87.72	88.97	82.83	69.05	81.65	73.16	74.62	0.9	1	84.82	143.75	61.18	96.58	59.87	42.85	120.8	74.51	0.77	1	3.87	4.82	4.97	4.55	8.17	8.65	8.41	1.85	0.609317
Solyc01g103150.2.1	Os03g0731050 protein (Frag	9.95	13.13	10.56	7.27	10.23	13.82	12.69	17.37	11.54	13.85	1.35	0.563997	16.81	15.85	15.54	16.07	9.43	11.07	12.51	11	0.68	0.488583	19.95	17.29	11.35	16.2	13.18	10.68	14.13	12.66	0.78	1	4.95	17.1	12.23	11.43	8.65	10.32	9.48	0.83	1
Solyc01g103160.2.1	Pentatricopeptide repeat-con	17.52	14.21	17.16	17.42	16.58	12.18	17.63	9.83	17.05	14.17	0.85	1	18.82	19.41	21.03	19.75	24.59	17.9	20.05	20.85	1.06	1	11.04	13.51	13.43	12.66	17.51	15.74	13.36	15.54	1.23	1	26.65	20.98	19.04	22.22	30.91	37.38	34.15	1.54	0.737112
Solyc01g103170.2.1	Lysine ketoglutarate reductas	5.23	5.47	8.16	5.22	6.02	6.15	4.49	8.48	5.85	6.24	1.04	1	6.87	10.29	10.91	9.36	13.65	15.37	9.06	12.69	1.36	0.54195	21.24	11.15	34.04	22.14	26.06	24.24	19.83	23.38	1.06	1	37.17	57.86	67.63	54.22	51.66	50.7	51.18	0.94	1
Solyc01g103180.2.1	Protein FAR1-RELATED SEQU	4.15	6.58	6.58	6.16	5.87	5.41	5.52	5.03	5.17	5.28	0.9	1	7.45	5.02	6.42	6.3	6.19	5.46	5.81	5.82	0.92	1	3.27	5.2	5.13	4.53	4.26	5.15	4.43	4.61	1.02	1	7.78	5.52	6.39	6.56	5.94	6.4	6.17	0.94	1
Solyc01g103190.2.1	COX assembly mitochondrial	82.06	78.05	116.75	116.08	98.23	128.78	99.08	169.39	96.46	123.43	1.26	0.565784	89.05	83.22	90.38	87.55	73.66	80.4	72.71	75.59	0.86	1	107.25	97.03	100.62	101.63	88.26	83.54	74.46	82.09	0.81	1	83.5	112.78	106.4	100.89	91.32	103.69	97.5	0.97	1
Solyc01g103200.2.1	Unknown Protein (AHRD V1)	13.49	27.4	20.76	19.08	20.18	23.14	15.51	17.79	18.94	18.84	0.93	1	23.73	26	29.22	26.32	30.91	44.78	33.64	36.44	1.38	0.322067	15.87	14.57	17.75	16.06	18.68	13.11	19.87	17.22	1.07	1	12.51	35.14	62.22	36.62	24.42	32.65	28.54	0.78	1
Solyc01g103210.2.1	Unknown Protein (AHRD V1)	27.26	37.85	66.25	53.98	46.34	47.27	26.26	26.57	28.18	32.07	0.69	0.744225	49.19	59.72	96.24	68.38	76.39	74.81	55.27	68.82	1.01	1	27.75	42.65	47.08	39.16	43.34	34.3	48.07	41.9	1.07	1	77.52	69.69	97.25	81.49	73.87	114.25	94.06	1.15	1
Solyc01g103220.2.1	Cytochrome c (AHRD V1 ***-	816.76	630.78	755.59	1020.69	805.96	776.8	1172.55	601.19	878.93	857.37	1.06	0.935473	619.62	599.86	553.76	591.08	408.97	415.03	412.92	412.31	0.7	0.307017	948.05	921.01	884.88	917.98	948.84	925.25	789.18	887.76	0.97	1	525.43	550.39	547.63	541.15	895.17	614.26	754.71	1.39	0.945561
Solyc01g103230.2.1	Non-lysosomal glucosylceram	16.51	17.09	16.71	18.08	17.1	14.05	17.34	13.08	17.66	15.53	0.91	1	13.82	15.67	15.99	15.16	11.64	12.63	11.45	11.91	0.79	0.814166	4.19	5.74	3.29	4.41	3.31	3.82	5.29	4.14	0.94	1	4.52	2.81	5.84	4.39	5	6.24	5.62	1.28	1
Solyc01g103240.2.1	Protein serine/threonine kina	0.73	0.82	0.43	2.07	1.01	2.65	3.04	3.37	2.37	2.86	2.82	0.061219	0.34	1.75	0.46	0.85	0.13	0.76	0.58	0.49	0.58	1	0	0	0	0	0	0	0	0	1	NA	0	0	0	0	0	0	0	1	NA
Solyc01g103250.2.1	Histone-lysine N-methyltrans	86.82	89.6	78.3	78.95	83.42	70.84	101.35	129.8	101.73	100.93	1.21	0.566756	95.12	97.83	97.29	96.75	73.94	97.28	110.84	94.02	0.97	1	22.92	33.87	12.83	23.21	39.7	16.2	29.52	28.47	1.23	1	5.44	9.01	13.8	9.42	6.28	18.63	12.46	1.32	1
Solyc01g103260.2.1	Unknown Protein (AHRD V1)	0	0	0	0	0	0	0.95	1.1	0	0.51	51.25	1	1.12	0	1.01	0.71	0	0	0	0	0.01	1	0	0	0	0	0	0	0	0	1	NA	0	0	0	0	0	0	0	1	NA
Solyc01g103270.2.1	Aquaporin PIP1-3 (AHRD V1 -	11.87	8.22	14.01	1.87	8.99	8.8	9.37	9.7	9.47	9.34	1.04	1	14.73	12.62	8.92	12.09	6.41	8.88	7.26	7.52	0.62	0.962977	3.2	3.23	5.24	3.89	4.33	6	0.96	3.76	0.97	1	3.75	3.83	2.03	3.2	2.42	1.16	1.79	0.56	1
Solyc01g103280.2.1	Unknown Protein (AHRD V1)	9.73	8.66	11.48	1.53	7.85	10.82	8.54	8.95	8.54	9.21	1.17	1	15.09	11.28	10.96	12.44	6	7.84	5.95	6.6	0.53	0.748186	3.49	2.64	4.29	3.47	3.55	4.92	2.36	3.61	1.04	1	3.08	3.14	1.66	2.63	1.98	0.95	1.46	0.56	1
Solyc01g103290.2.1	Unknown Protein (AHRD V1)	11.47	6.38	10.15	16.23	11.06	9.57	10.19	9.23	13.39	10.59	0.96	1	13.34	16.21	32.71	20.75	2.98	5.94	11.27	6.73	0.32	0.251978	1.16	5.84	11.39	6.13	4.71	3.26	3.14	3.7	0.6	1	8.16	0	4.41	4.19	1.31	6.29	3.8	0.91	1
Solyc01g103300.1.1	SWIB complex BAF60b domai	9.9	8.22	7	7.47	8.15	11	10.94	16.38	8.05	11.59	1.42	0.790594	11.05	8.03	12.82	10.63	7.78	8.54	4.67	7	0.66	0.911351	5.86	2.15	4.58	4.2	6.07	6.5	4.81	5.79	1.38	1	3.75	4.31	5.07	4.38	3.02	2.31	2.67	0.61	1
Solyc01g103310.2.1	Unknown Protein (AHRD V1)	29.84	33.55	35.21	17.77	29.09	36.67	21.08	36.1	24.81	29.66	1.02	1	33.6	28.67	31.84	31.37	16.34	21.96	20.98	19.76	0.63	0.77615	26.65	17.92	18.71	21.09	17.54	21.43	16.02	18.33	0.87	1	31.27	14.83	16.91	21	14.38	16.53	15.46	0.74	1
Solyc01g103320.2.1	Zinc finger CCCH domain-con	25.24	30.34	24.55	20.35	25.12	31.99	40.72	41.12	29.56	35.85	1.43	0.279072	29.45	28.29	28.05	28.6	17.5	23.96	26.79	22.75	0.8	0.87925	27.19	18.4	15.6	20.4	28.5	19.97	27.91	25.46	1.25	1	22.98	20.1	27.23	23.44	19.86	22.9	21.38	0.91	1
Solyc01g103330.2.1	Unknown Protein (AHRD V1)	0	0	0	0	0	0	0	0	0	0	1	NA	0	0	0	0	0	0	0	0	1	NA	0	0	0	0	0	0	0	0	1	NA	0	0	0	0	0	1.81	0.91	90.5	1
Solyc01g103340.2.1	Zinc finger CCCH domain-con	18.27	29.27	19.64	17.23	21.1	29.33	26.44	39.57	19.53	28.72	1.36	0.582886	19.45	22.06	19.72	20.41	14.78	17.34	19.14	17.09	0.84	0.994358	21.65	15.55	15.72	17.64	12.93	14	14.79	13.91	0.79	1	18.47	17.99	15.92	17.46	16.72	16.37	16.55	0.95	1
Solyc01g103350.2.1	Cell division protein kinase 13	11.25	11.85	10.19	12.86	11.54	11.1	12.51	10.92	14.6	12.28	1.06	0.967871	14.57	15.02	15.58	15.06	17.04	14.59	16.2	15.94	1.06	1	15.12	15.4	15.99	15.5	14.84	8.53	14.63	12.67	0.82	1	1.28	4.05	2.63	2.65	3.3	4.66	3.98	1.5	1
Solyc01g103360.2.1	Expressed protein (Fragment)	0.24	0	0	0	0.06	0	0	0.22	0.17	0.1	1.63	1	0	0	0	0	0	0	0	0	1	NA	0	0	0	0	0	0	0	0	1	NA	0	0	0	0	0	0	0	1	NA
Solyc01g103370.2.1	Ras-related protein Rab-1A (A	127.07	135.65	116.17	104.35	120.81	120.29	94.67	126.69	132.75	118.6	0.98	1	123.33	100.85	93.05	105.74	86.33	77.57	86.58	83.49	0.79	0.784393	136.27	129.76	101.29	122.44	137.7	127.1	146.83	137.21	1.12	1	63.76	87.33	69.46	73.52	114.24	104.57	109.41	1.49	0.939829
Solyc01g103380.2.1	Ras-related protein Rab-25 (A	37.43	28.95	39.82	61.63	41.96	31.04	41.67	29.06	31.28	33.26	0.79	0.990049	38.53	34.14	44.84	39.17	28.94	30.34	29.9	29.73	0.76	0.750352	34.95	44.01	38.38	39.11	28.83	29.3	26.83	28.32	0.72	1	35.77	51.48	47.66	44.97	41.01	41.7	41.36	0.92	1
Solyc01g103390.2.1	Flavin oxidoreductase/NADH	4.08	3.3	2.62	3.41	3.35	2.06	3.95	5.62	3.86	3.87	1.16	0.984437	1.9	1.93	2.97	2.27	1.54	2.11	2.33	1.99	0.88	1	0.45	0.6	0.92	0.66	0.73	0.14	0.27	0.38	0.58	1	0.26	0.27	0.28	0.27	0	0	0	0.04	1
Solyc01g103400.2.1	Unknown Protein (AHRD V1)	132.56	181.68	103.53	132.05	137.45	197.86	119.4	225.46	140.75	170.87	1.24	0.804781	121.57	153.11	106.71	127.13	106.19	121.48	155.05	127.57	1	1	127.21	125.77	81.54	111.51	77.27	80.66	104.77	87.57	0.79	1	75.22	82.27	76.56	78.02	53.55	64.39	58.97	0.76	1
Solyc01g103410.2.1	Nucleic acid binding protein (	496.66	452.29	564.76	527.27	510.25	376.8	379.61	380.46	541.78	419.66	0.82	0.904728	445.77	492.11	425.38	454.42	540.15	655.66	540.79	578.87	1.27	0.370615	478.6	505.89	442.94	475.81	506.14	445.34	469.01	473.5	1	1	539.53	498.82	526.62	521.66	515.28	520.58	517.93	0.99	1
Solyc01g103420.2.1	Upf3 regulator of nonsense tr	45	43.47	55.44	46.38	47.57	39.99	40.45	47.72	39.93	42.02	0.88	1	51.61	48.46	52.92	51	39.59	42.13	39.54	40.42	0.79	0.812097	50.83	38.07	51.25	46.72	47.96	38.23	44.88	43.69	0.94	1	63.41	65.08	69.4	65.96	43.74	52.02	47.88	0.73	1
Solyc01g103430.2.1	Glycogen synthase kinase (AH	218.5	213.06	179.4	195.01	201.49	206.65	211.29	199.46	232.22	212.41	1.05	1	213.98	260.3	210.5	228.26	240.9	246.13	228.34	238.46	1.04	1	31.88	49.31	27.4	36.2	55.84	40.54	54.32	50.23	1.39	1	181.39	131.62	134.06	149.02	86.8	127.03	106.91	0.72	1
Solyc01g103440.2.1	Phosphoribosylamine--glycine	18.83	16.76	18.14	19.75	18.37	20.36	17.9	15.78	19.59	18.41	1	1	14.66	14.81	16.66	15.38	18.92	19.83	20.03	19.59	1.27	0.594579	15.42	18.27	18.56	17.42	18.68	18.93	22.96	20.19	1.16	1	15.85	28.47	26.79	23.7	35.3	33.81	34.55	1.46	0.984877
Solyc01g103450.2.1	Chaperone DnaK (AHRD V1 *	348.41	291.32	328.35	284.32	313.1	374.99	348.6	333.18	371.57	357.08	1.14	0.771098	278.5	263.68	327.76	289.98	450.35	403.15	439.87	431.12	1.49	0.071746	139.53	238.24	231.98	203.25	307.43	266.06	290.15	287.88	1.42	1	153.49	228.35	229.84	203.89	402.75	351.33	377.04	1.85	0.353489
Solyc01g103460.2.1	3&apos-5&apos exonuclease	37.92	31.28	47.28	44.48	40.24	32.21	41.86	32.88	31.21	34.54	0.86	1	24.66	31.62	34.63	30.3	30.58	33.04	29.8	31.14	1.03	1	14.69	21.1	8.26	14.68	15.8	9.11	11.01	11.97	0.82	1	3.51	3.13	1.42	2.69	3.95	5.68	4.81	1.79	1
Solyc01g103470.2.1	Unknown Protein (AHRD V1)	5.64	8.16	5.41	17.47	9.17	3.14	12.81	6.81	5.07	6.96	0.76	1	7.55	3.99	5.36	5.63	1.71	2.19	2.77	2.22	0.39	0.229716	0.86	0	0.7	0.52	0	0.27	0	0.09	0.17	1	2.51	6.41	6.51	5.14	0.32	4.64	2.48	0.48	0.989607
Solyc01g103480.2.1	Coatomer subunit delta (AHR	148.81	138.78	154.04	168.78	152.6	100.41	155.06	104.43	125.31	121.3	0.79	0.800101	142.6	130.77	124.53	132.63	95.93	90.76	104.67	97.12	0.73	0.441263	101.75	136.43	102.44	113.54	110.48	99.53	120.64	110.22	0.97	1	134.12	135.49	146.6	138.74	164.93	178.02	171.48	1.24	1
Solyc01g103490.2.1	Necrotic spotted lesions 1 (Fr	15.78	15.9	16.29	20.31	17.07	10.84	15.75	14.81	10.86	13.06	0.77	0.729949	17.43	26.39	22.86	22.23	16.27	15.83	18.86	16.99	0.76	0.665104	5.58	5.8	6.42	5.93	11.23	6.89	5.91	8.01	1.35	1	23.84	32	36.4	30.75	17.15	21.14	19.14	0.62	0.744205
Solyc01g103500.2.1	Unknown Protein (AHRD V1)	0.62	0	0	0	0.15	0.69	1.47	0	0	0.54	3.48	1	1.16	0.54	0	0.57	0	0	0	0	0.02	0.962977	0	0.51	0	0.17	0	0	0	0	0.06	1	0	0	0	0	1.71	0	0.85	85.5	1
Solyc01g103510.2.1	Ribosomal protein L3-like (AH	25.26	20.76	27.35	27.53	25.23	15.86	23.93	18.72	10.67	17.29	0.69	0.370783	11.75	19.12	17.32	16.06	5.15	5.77	5.37	5.43	0.34	4.39E-05	7.92	8.96	7.82	8.23	5.56	3.83	6.06	5.15	0.63	1	6.34	7.55	10.05	7.98	12.24	15.11	13.68	1.71	0.698088
Solyc01g103530.2.1	Receptor like kinase, RLK	1.72	1.83	1.09	1.5	1.53	1.25	1.36	1.64	1.28	1.38	0.9	1	3.02	1.5	2.52	2.35	3.12	1.58	0.78	1.83	0.78	1	0.86	0.59	0.53	0.66	0.3	0.25	0.43	0.33	0.49	1	1.32	1.2	0.92	1.15	0.12	0.46	0.29	0.25	0.425599
Solyc01g103540.2.1	YTH domain family 2 (Predict	706.93	610.05	856.57	632.23	701.45	610.53	396.52	492.01	585.21	521.07	0.74	0.435181	673.91	907.45	1002.67	861.34	1328	1222.3	1125.85	1225.38	1.42	0.124346	394.85	672.65	581.68	549.73	1011.59	899.67	715.49	875.58	1.59	1	1940.9	1690.21	2497.12	2042.74	2596.36	3165.84	2881.1	1.41	0.796983
Solyc01g103550.1.1	Transcription factor MADS-bo	0	0	0	0.3	0.07	0	0.17	0	0	0.04	0.57	1	0.2	0	0	0.07	0	0	0	0	0.15	1	0	0	0	0	0	0	0	0	1	1	0	0	0	0	0	0	0	1	NA
Solyc01g103560.2.1	tRNA pseudouridine synthase	7.1	6.62	9.18	8.14	7.76	6.02	3.87	6.22	6.56	5.67	0.73	0.760084	6.29	6.32	10.14	7.58	8.8	6.57	6.91	7.43	0.98	1	2.87	5.24	3.19	3.77	3.78	3.08	3.57	3.48	0.92	1	4.09	3.44	4.94	4.16	5.73	6.67	6.2	1.49	1
Solyc01g103570.2.1	DNA-binding bromodomain-c	13.83	11.19	16.73	10.51	13.06	11.57	8.58	16.92	15.27	13.09	1	1	15.1	15.21	12.84	14.38	10.31	14.03	12.58	12.31	0.86	1	7.39	6.61	6.01	6.67	9.01	6.67	7	7.56	1.13	1	15.12	16.77	17.41	16.43	5.55	9.62	7.58	0.46	0.277633
Solyc01g103580.2.1	Genomic DNA chromosome 5	6.51	8.43	5.93	5.02	6.47	8.22	7.47	5.98	7.01	7.17	1.11	1	7.15	7.71	3.5	6.12	5.95	7.66	7.21	6.94	1.13	1	3.82	6.27	2.35	4.15	4.47	3.14	4.53	4.05	0.98	1	1.68	3.01	2.73	2.47	1.62	1.56	1.59	0.64	1
Solyc01g103590.2.1	Glyoxalase/bleomycin resista	48.38	76.46	48.43	42.3	53.89	86.37	33.8	135.06	59.99	78.81	1.46	0.845857	21.27	16.06	12.88	16.74	3.05	5.31	10.6	6.32	0.38	0.043616	1.9	15.53	3.2	6.88	2.5	2	13.67	6.06	0.88	1	2.08	1.7	0.9	1.56	0.27	0.26	0.27	0.17	0.867156
Solyc01g103600.2.1	Protein TIFY 3A (AHRD V1 **	5.58	11.74	4.19	4.31	6.46	18.62	11.22	20.53	8.74	14.78	2.29	0.223346	8.02	11.03	5.57	8.21	2.46	5.26	5.18	4.3	0.52	0.585009	2.87	5.38	3.02	3.76	3	2.31	2.96	2.76	0.73	1	8.66	8.11	7.8	8.19	4.18	4.45	4.31	0.53	1
Solyc01g103610.2.1	Nbs-lrr, resistance protein	2.47	2.93	1.82	2.76	2.5	2.4	2.43	2.27	2.07	2.29	0.92	1	3.06	3.31	3.12	3.16	0.78	1.49	1.05	1.11	0.35	0.027321	1	1	1.22	1.07	0.74	1.01	0.97	0.91	0.84	1	1.61	1.34	2.21	1.72	1.69	1.89	1.79	1.04	1
Solyc01g103620.2.1	Sodium Bile acid symporter fa	6.94	8.9	5.33	7.3	7.12	13.99	9.04	12.46	10.54	11.51	1.62	0.200081	6	8.27	5.31	6.53	3.8	6.1	7.1	5.67	0.87	1	4.82	2.5	5.12	4.15	3.07	3.42	3.76	3.42	0.82	1	0.69	1.87	1.98	1.51	1.62	1.98	1.8	1.19	1
Solyc01g103630.2.1	Glyoxalase/bleomycin resista	3.59	8.14	9.25	5.54	6.63	3.27	0.77	4.2	5.39	3.41	0.51	0.390314	3.04	1.7	1.38	2.04	0.23	0.34	0.77	0.45	0.22	0.274606	0.26	1.6	0.65	0.84	0.21	0.5	2.38	1.03	1.23	1	0	0	0	0	0	0.29	0.14	14.5	1
Solyc01g103640.2.1	A_IG002N01.30 protein (Frag	5	3.56	5.31	5.3	4.79	3.89	4.73	3.91	3.59	4.03	0.84	1	3.49	5.87	6.33	5.23	7.97	4.79	6.28	6.35	1.21	1	1.41	5.09	4.46	3.65	4.27	3.03	2.19	3.16	0.87	1	2.13	3.63	4.61	3.46	5.03	4.16	4.6	1.33	1
Solyc01g103650.2.1	Hydrolase alpha/beta fold far	7.15	8.88	14.23	13.48	10.94	4.63	4.47	5.61	5.88	5.15	0.47	0.065166	5.26	4.4	4.02	4.56	2.79	1.08	2.22	2.03	0.45	0.084383	0.12	1.21	0.74	0.69	0.59	0	0.22	0.27	0.39	1	0	0.65	0.69	0.45	0.27	0.39	0.33	0.74	1
Solyc01g103660.2.1	Alpha/beta hydrolase fold (AI	3.59	3.08	1.41	1.86	2.48	3.72	2.83	2.75	3	3.08	1.24	0.966482	1.56	0.94	1.01	1.17	0.17	0.19	0.09	0.15	0.13	0.025096	1.16	0.68	0.71	0.85	0.08	0.18	0.09	0.12	0.14	1	0	0	0	0	0	0	0	1	NA
Solyc01g103670.2.1	Alpha/beta hydrolase fold-1 d	15.1	19.06	19.28	17.68	17.78	16.65	13.09	15.36	15.37	15.12	0.85	0.944449	14.28	14.42	16.1	14.93	15.11	17.17	15.56	15.95	1.07	1	18.1	18.05	16.22	17.46	16.67	16.7	17.32	16.9	0.97	1	6.69	12.32	9.9	9.64	16.54	13.24	14.89	1.55	0.981546
Solyc01g103680.2.1	Coiled-coil domain containing	106.26	98.21	123.35	116.23	111.01	101	91.59	82.81	101.91	94.33	0.85	0.953747	114.77	133.58	138.25	128.87	137.82	121.02	107.83	122.22	0.95	1	121.92	147	129	132.64	164.88	128.85	129.35	141.03	1.06	1	270.16	180.85	181.02	210.68	240.24	254.23	247.24	1.17	1
Solyc01g103690.2.1	ATP-dependent RNA helicase	8.39	8.98	8.33	9.08	8.7	7.34	7.2	7.5	6.58	7.15	0.82	0.853324	9.13	12.75	13.09	11.66	11.03	11.23	9.66	10.64	0.91	1	8.22	8.67	10.28	9.06	10.51	9.03	8.65	9.4	1.04	1	19.82	15.48	16.07	17.12	12.84	15.4	14.12	0.82	1
Solyc01g103700.2.1	Unknown Protein (AHRD V1);	0	0.26	0	0	0.07	0	0	0	0	0	0.15	1	0	0	0	0	0	0	0	0	1	NA	0	0	0	0	0	0	0	0	1	NA	0	0	0	0	0	0	0	1	NA
Solyc01g103710.2.1	Fe/S biogenesis protein nfuA	78.23	76.12	59.99	40.94	63.82	107.24	72.26	111.96	100.53	98	1.54	0.118323	74.95	64.6	46.91	62.15	78.49	80.6	95.08	84.72	1.36	0.290226	104.28	106.45	87.48	99.4	104.49	139.33	122.75	122.19	1.23	1	99.73	121.97	88.21	103.3	110.04	74.53	92.28	0.89	1
Solyc01g103720.2.1	Ribosome biogenesis protein	106.87	120.36	136.21	110.11	118.39	89.47	70.83	70.97	89.04	80.08	0.68	0.2951	120.28	112.97	119.24	117.5	89.68	89.83	110.95	96.82	0.82	0.911351	60.76	61.82	67.45	63.34	55.47	54.5	52.4	54.12	0.85	1	91.92	107.33	129.85	109.7	87.57	131.88	109.72	1	1
Solyc01g103730.2.1	Unknown Protein (AHRD V1)	0	0	3.38	0	0.84	0	0	0	0	0	0.01	1	0	0	4.85	1.62	0	0	0	0	0.01	1	0	0	0	0	0	0	0	0	1	1	0	0	0	0	0	0	0	1	NA
Solyc01g103740.2.1	Ribosome biogenesis protein	58.52	60.23	71.98	53.22	60.99	38.19	45.17	51.41	57.59	48.09	0.79	0.737799	62.22	58.03	62.12	60.79	64.78	66.62	63.14	64.85	1.07	1	29.97	39.92	34.1	34.66	48.83	34.85	40.84	41.51	1.2	1	71.32	69.83	81.76	74.3	68.4	93.01	80.71	1.09	1
Solyc01g103750.2.1	ATP phosphoribosyltransferas	50.94	44.15	48.87	57.17	50.28	54.58	72.52	37.93	56.83	55.47	1.1	0.821768	46.45	55.02	54.03	51.83	63.02	50.16	51.06	54.75	1.06	1	18.22	35.25	28.02	27.16	29.95	25.82	32.01	29.26	1.08	1	38.47	38.82	36.98	38.09	42.35	50.67	46.51	1.22	1
Solyc01g103760.2.1	DUF803 domain membrane p	22.37	26.43	18.18	24.19	22.79	24.03	41.1	27.48	26.99	29.9	1.31	0.411384	22.03	18.46	13.65	18.05	9.65	16.93	19.35	15.31	0.85	1	17.77	17	12.69	15.82	15.8	13.96	13.83	14.53	0.92	1	6.68	13.24	13.01	10.98	13.25	15.33	14.29	1.3	1
Solyc01g103770.2.1	Protein p8 MTCP-1 (AHRD V1	17.25	45.16	25.15	40.2	31.94	25.95	28.04	53.19	24.05	32.81	1.03	1	23.6	25.59	35.15	28.11	38.01	27.85	33.5	33.12	1.18	1	26.24	28.12	19.14	24.5	22	26.15	24.41	24.19	0.99	1	21.65	21.38	32.78	25.27	13.01	17.8	15.41	0.61	1
Solyc01g103780.2.1	TCP family transcription facto	72.33	47.92	95.63	91.43	76.83	59.52	72.17	35.09	61.59	57.09	0.74	0.859519	62.88	76.25	106.32	81.82	109.36	92.98	69.86	90.73	1.11	1	43.62	66.05	55.94	55.2	60.15	49.1	49.58	52.94	0.96	1	94.01	65.86	57.32	72.4	70.68	72.63	71.66	0.99	1
Solyc01g103790.2.1	Wall-associated receptor kina	47.12	57.88	12.23	30.66	36.97	40.86	42.41	81.45	33.83	49.64	1.34	0.953095	20.16	22.94	17.51	20.2	10.46	12.2	20	14.22	0.7	0.863884	13.71	13.82	5.61	11.05	7.43	12.14	6.87	8.81	0.8	1	5.36	6.16	13.04	8.19	4.31	4.13	4.22	0.52	1
Solyc01g103800.2.1	40S ribosomal protein S12 (A	484.7	541.07	455.64	408.9	472.58	462.4	375.07	481.46	381.86	425.2	0.9	0.976183	542.06	458.38	288.28	429.57	293.6	386.17	429.88	369.88	0.86	1	225.78	218.02	155.07	199.62	167.33	148.27	213.66	176.42	0.88	1	98.36	124.11	181.76	134.74	128.75	175.13	151.94	1.13	1
Solyc01g103810.1.1	Zinc finger-homeodomain pro	0	0	0	0	0	0	0	0	0	0	1	NA	0	0	0	0	0	0	0	0	1	NA	0	0	0	0	0	0	0	0	1	NA	0	0	0	0	0	0	0	1	NA
Solyc01g103820.1.1	Zinc finger-homeodomain pro	0	0	0	0	0	0	0	0	0	0	1	NA	0	0	0	0	0	0	0	0	1	NA	0	0	0	0	0	0	0	0	1	NA	0	0	0	0	0	0	0	1	NA
Solyc01g103830.1.1	Zinc finger-homeodomain pro	0	0	0	0	0	0	0	0	0	0	1	NA	0	0	0	0	0	0.16	0	0.05	5.33	1	0.25	0	0	0.08	0	0	0	0	0.12	1	0	0	0	0	0	0	0	1	NA
Solyc01g103840.1.1	Zinc finger-homeodomain pro	0	0	0	0	0	0	0	0	0	0	1	NA	0	0	0	0	0	0	0	0	1	NA	0	0	0	0	0	0	0	0	1	NA	0	0	0	0	0	0	0	1	NA
Solyc01g103850.2.1	Ubiquitin-like domain contair	21.33	20.04	22.1	20.86	21.08	19.5	15.73	21.26	20.18	19.17	0.91	1	26.33	26.16	27.94	26.81	24.33	19.71	20.36	21.47	0.8	0.872742	17.23	19.49	15.43	17.38	16.76	18.58	18.3	17.88	1.03	1	35.14	22.15	25.74	27.68	25.73	29.01	27.37	0.99	1
Solyc01g103860.2.1	Ch-cobra (AHRD V1 ***- B7S	0	0	0.37	0	0.09	0	0.12	0	0.34	0.12	1.24	1	0.87	0.68	0.26	0.6	1.51	1.05	0.24	0.93	1.55	0.96542	0.13	1.01	0.46	0.53	0.41	0.24	1.81	0.82	1.54	1	0	0	0	0	0	0	0	1	NA
Solyc01g103870.1.1	SRF-type transcription factor	0.52	1.4	0.62	0	0.64	0	0.83	2.41	3.01	1.56	2.46	0.838674	1.46	0.91	0	0.79	1.45	0.81	1.65	1.3	1.65	1	0.42	0.43	0	0.28	0.34	0.4	0.76	0.5	1.76	1	0.74	0	0	0.25	0.48	0	0.24	0.97	1
Solyc01g103880.2.1	BZIP transcription factor fami	9.68	18.01	19.72	18.66	16.52	19.56	21.53	13.75	10.1	16.23	0.98	1	12.27	13	17.83	14.37	15.86	14.57	17.97	16.13	1.12	1	14.21	22.22	15.72	17.38	15.6	16	15.38	15.66	0.9	1	30.02	55.58	48.69	44.76	31.4	31.62	31.51	0.7	1
Solyc01g103890.2.1	Magnesium transporter MRS	35.65	43.8	49.97	47.63	44.26	39.12	29.35	34.04	41.13	35.91	0.81	0.838602	39.54	47.41	52.25	46.4	42.9	39.92	38.79	40.54	0.87	1	40	39.11	42.5	40.54	38.33	34.64	36.42	36.46	0.9	1	41.59	55.33	54.35	50.42	37.38	39.94	38.66	0.77	1
Solyc01g103900.2.1	Phospholipase D (AHRD V1 **	0.4	0	0	0	0.1	0	0	0	0	0	0.1	1	0	0	0	0	0	0	0	0	1	NA	0	0	0	0	0	0	0.29	0.1	9.67	1	0	0	0	0	0	0	0	1	NA
Solyc01g103910.1.1	Phospholipase D (AHRD V1 **	0	0	0	0	0	0	0	0	0	0	1	NA	0	0	0	0	0	0	0	0	1	NA	0	0	0	0	0.06	0	0	0.02	2	1	0	0	0.15	0.05	0	0	0	0.2	1
Solyc01g103920.2.1	Ferredoxin I (AHRD V1 ***- Q	167.5	147.41	59.62	48.31	105.71	724.48	473.18	715.83	644.26	639.44	6.05	1.9E-17	120.55	169.6	77.06	122.4	603.27	414.25	512.2	509.91	4.17	5.07E-09	38.56	301.19	25.08	121.61	135.24	120.57	360.73	205.51	1.69	1	41.24	73.06	55.4	56.57	123.35	235.47	179.41	3.17	0.156027
Solyc01g103930.2.1	Transmembrane 9 superfamil	34.72	45.75	36.74	45.79	40.75	36.48	39.53	38.41	34.58	37.25	0.91	1	36.05	36.12	26.87	33.01	27.19	25.98	33.62	28.93	0.88	1	16.11	23.18	12.23	17.17	14.79	11.88	15.83	14.17	0.82	1	8.92	11.63	12.06	10.87	17.53	23.27	20.4	1.88	0.458854
Solyc01g103940.2.1	Serine/threonine protein kina	332.77	305.25	203.61	223.49	266.28	277.53	397.09	404.49	332.98	353.02	1.33	0.320347	287.5	258.38	205.49	250.46	188.33	234.67	248.2	223.73	0.89	1	99.53	123.26	79.77	100.85	110.5	97.24	102.52	103.42	1.03	1	72.81	121.38	107.05	100.41	98.76	107.86	103.31	1.03	1
Solyc01g103950.2.1	Thiamine-phosphate pyropho	15.72	13.35	13.63	16.51	14.8	21.44	20.02	21.24	15.56	19.57	1.32	0.415403	13.51	17.08	18.1	16.23	15.36	15.6	13.01	14.66	0.9	1	6.06	8.93	7.09	7.36	5.32	5.96	6.25	5.84	0.79	1	7.28	4.5	6.96	6.25	7.85	10.96	9.41	1.51	0.841325
Solyc01g103960.2.1	ATP-dependent DNA helicase	4.08	3.43	3.49	3.48	3.62	2.61	2.34	2.67	4.25	2.97	0.82	0.968446	3.33	3.98	5.57	4.29	3.95	4.83	5	4.59	1.07	1	0.81	1	0.55	0.79	0.66	0.8	0.61	0.69	0.88	1	4.21	3.85	3.52	3.86	3.27	3.28	3.28	0.85	1
Solyc01g103970.2.1	RING finger protein 170 (AHR	28.93	35.28	22.53	31.51	29.56	44.71	34.04	43.9	29.91	38.14	1.29	0.602214	30.95	38.48	34.46	34.63	32.51	33.71	38.66	34.96	1.01	1	36.7	35.6	41.64	37.98	36.12	36.84	39.43	37.46	0.99	1	55.13	104.45	91.66	83.75	42.38	43.64	43.01	0.51	0.255056
Solyc01g103980.2.1	RING finger protein 170 (AHR	40.89	42.39	41.78	33.69	39.69	61.62	40.43	60.66	51.45	53.54	1.35	0.380958	45.88	41.22	43.97	43.69	45.17	48.78	61.29	51.75	1.18	0.813994	48.53	43.45	35.77	42.58	35.03	41.22	43.01	39.75	0.93	1	36.86	28.63	24.96	30.15	26.05	23.47	24.76	0.82	1
Solyc01g103990.2.1	T-complex protein 11 (AHRD	90.49	91.17	124.69	76.97	95.83	76.45	61.29	110.95	116.51	91.3	0.95	1	100.08	67.83	81.69	83.2	61.51	59.4	47.13	56.01	0.67	0.31805	178.3	138.97	175.84	164.37	130.86	126.53	148.84	135.41	0.82	1	141.17	180.78	181.24	167.73	127.82	117.46	122.64	0.73	0.963653
Solyc01g104000.2.1	Serine hydroxymethyltransfer	19.29	19.05	21.45	29.33	22.28	11.09	23.55	15.09	16.48	16.55	0.74	0.824686	24.65	17.45	21.73	21.28	18.23	20.9	18.12	19.08	0.9	1	37.35	36.14	48.2	40.56	36.13	34.03	38.7	36.29	0.89	1	21.75	37.77	37	32.17	24.87	27.12	26	0.81	1
Solyc01g104010.2.1	F2E2.8 (AHRD V1 ***- Q9LM	0.41	0	0.48	0	0.22	0.23	0	0.19	0.15	0.14	0.64	1	0	0	0	0	0.14	0.11	0	0.08	8.33	1	0.16	0	0	0.05	0.13	0.15	0	0.09	1.75	1	0	0.15	0.63	0.26	0	0	0	0.04	1
Solyc01g104020.2.1	Embryo-specific 3 (AHRD V1	45.64	42.1	22.03	24.65	33.61	65.95	73.7	55.7	38.72	58.52	1.74	0.192974	37.82	28.13	18.92	28.29	37.99	34.8	33.26	35.35	1.25	0.70488	50.95	63.22	34.59	49.59	43.34	38.05	62.59	47.99	0.97	1	4.72	21.4	16.59	14.24	20.51	21.83	21.17	1.49	1
Solyc01g104030.2.1	Inward rectifier potassium ch	33.93	37.69	31.02	23.05	31.42	37.54	38.53	51.61	44.54	43.05	1.37	0.283437	56.02	38.66	33.25	42.64	57.44	50.38	65.83	57.88	1.36	0.361823	29.51	44.9	20.16	31.52	35.17	27.69	37.35	33.4	1.06	1	9.85	20.07	18.04	15.99	55.85	82.62	69.23	4.33	2.93E-05
Solyc01g104040.2.1	UDP-glucose glycoprotein glu	24.09	26.91	30.46	20.45	25.48	17.63	15.37	21.97	22.83	19.45	0.76	0.552365	22.41	19.16	21.6	21.06	13.56	12.56	15.56	13.89	0.66	0.162169	12.65	12.76	11.76	12.39	11.21	9.5	10.33	10.35	0.84	1	10.45	12.48	11.86	11.6	9.37	11.83	10.6	0.91	1
Solyc01g104050.2.1	Receptor-like kinase (AHRD V	8.36	6.81	6.14	6.35	6.91	4.99	4.11	3.28	4.1	4.12	0.6	0.180525	8.44	6.2	6.63	7.09	6.93	7.44	8.09	7.49	1.06	1	6.26	6.07	5.97	6.1	7.44	4.25	4.68	5.46	0.89	1	6.52	21.48	18.66	15.55	8.68	7.42	8.05	0.52	0.844805
Solyc01g104060.2.1	Aminomethyltransferase (AH	40.73	32.84	29.96	28.24	32.94	51.85	49.18	44.79	59.17	51.25	1.56	0.060356	39.37	41.52	33.59	38.16	67.03	65.75	63.52	65.43	1.71	0.010321	30.07	33.97	33.14	32.39	53.1	44.67	41.44	46.4	1.43	0.845869	21.35	49.19	33.16	34.57	43.28	47.48	45.38	1.31	1
Solyc01g104070.2.1	Importin beta (AHRD V1 **--	19.16	22.26	24.09	19.64	21.29	14.39	13.69	14.85	18.18	15.28	0.72	0.423563	25.48	17.59	24.09	22.39	21.75	20.3	24.19	22.08	0.99	1	11.93	14.34	16.4	14.22	13.87	12.43	13.87	13.39	0.94	1	20.46	17.93	24.73	21.04	31.63	41.76	36.7	1.74	0.373488

Solyc01g104080.2.1	Hypothetical membrane span	41.09	54.45	27.74	25.19	37.12	31.21	19.46	36.52	28.52	28.93	0.78	0.710137	43.32	62.51	36.63	47.49	46.86	49.52	47	47.79	1.01	1	58.09	51.15	39.43	49.56	38.39	48.1	47.91	44.8	0.9	1	29.86	49.08	46.69	41.88	32.39	33.62	33	0.79	1
Solyc01g104090.2.1	Pentatricopeptide repeat-con	1.05	0.38	0.37	0.75	0.64	0.35	0.67	0.29	0.61	0.48	0.75	1	0.49	0.55	0.89	0.64	0.37	0.65	0.5	0.51	0.79	1	0.26	0.69	0.1	0.35	0.07	0.24	0.61	0.31	0.88	1	0	0.15	0.32	0.16	0.29	0.28	0.29	1.82	1
Solyc01g104100.2.1	Legumin-like protein (AHRD V	0	0	0	0	0	0	0	0	0	0	1	NA	0	0	0	0	0	0	0	0	1	NA	0	0.15	0	0.05	0	0	0	0	0.2	1	0	0.26	0.28	0.18	0.17	0	0.09	0.47	1
Solyc01g104110.2.1	Legumin 11S-globulin (AHRD	0	0	0	0	0	0	0	0	0.13	0.03	3.25	1	0.68	0	0.15	0.28	0.5	0.19	0.71	0.47	1.69	1	0.88	0.59	0.54	0.67	1.07	0.69	0.53	0.76	1.14	1	0	2.64	2.24	1.63	0.83	0.48	0.66	0.4	1
Solyc01g104120.1.1	MuDR family transposase dor	0.22	0.2	0	0.31	0.18	0	0.18	0	0	0.04	0.25	1	0	0.19	0.38	0.19	0.15	0	0	0.05	0.26	1	0	0.18	0	0.06	0	0	0	0	0.17	1	0	0.16	0	0.05	0	0	0	0.19	1
Solyc01g104130.2.1	Oxidoreductase 2OG-Fe(II) ox	7.33	8.15	6.49	3.89	6.46	7.81	6.27	10.81	7.46	8.09	1.25	0.854164	32.39	50.05	33.28	38.57	72.87	51.55	59.17	61.2	1.59	0.108052	173.96	150.7	83.67	136.11	161.22	127.32	189.96	159.5	1.17	1	38.43	66.55	51.19	52.06	71.71	51.55	61.63	1.18	1
Solyc01g104140.2.1	Phosphatidylinositol-4-phosp	14.22	16.06	14.36	12.28	14.23	13.06	16.94	16.75	13.69	15.11	1.06	1	18.13	18.57	18.12	18.27	14.94	19.26	18.2	17.47	0.96	1	40.71	33.23	36.25	36.73	35.23	27.94	35.36	32.84	0.89	1	16.1	33.51	33.12	27.58	23.38	25.15	24.27	0.88	1
Solyc01g104150.2.1	Unknown Protein (AHRD V1)	0	0	0	0	0	0	0	0	0.93	0.23	23.25	1	0	1.12	0	0.37	0	0.67	0	0.22	0.6	1	0	0	0	0	0	0	0	0	1	1	0	0	0	0	0	0	0	1	NA
Solyc01g104160.2.1	Uncharacterised protein At1g	123.58	159.19	134.84	70.64	122.06	169.9	121.77	252.66	121.41	166.44	1.36	0.773304	142.82	166.99	143.62	151.14	106.01	147.94	152.43	135.46	0.9	1	129.31	98.81	105.14	111.09	109.11	80.81	94.62	94.85	0.85	1	214.02	253.24	265.76	244.34	106.32	126.28	116.3	0.48	0.173651
Solyc01g104170.2.1	Ankyrin repeat domain-conta	538.32	447.4	499.68	474.84	490.06	456.96	518.45	365.5	548.18	472.27	0.96	1	477.42	507.11	523.46	502.66	671.57	594.05	522.3	595.97	1.19	0.686065	307.61	357.26	308.89	324.59	436.11	393.58	373.6	401.1	1.24	1	483.46	514.06	477.96	491.83	589.24	608.17	598.7	1.22	1
Solyc01g104180.2.1	Elongation of very long chain	0	0	0	0	0	0	0	0	0	0	1	NA	0	0	0	0	0	0	0	0	1	NA	1.14	0.19	1.64	0.99	0	0	0	0	0.01	1	0	0	0	0	0	0	0	1	NA
Solyc01g104190.2.1	Unknown Protein (AHRD V1)	79.08	78.93	38.14	50.06	61.55	70.9	67.6	80.34	81.86	75.17	1.22	0.748687	73.15	61.15	37.27	57.19	43.33	52.03	59.74	51.7	0.9	1	31.06	42.72	17.78	30.52	23.25	17.55	32.16	24.32	0.8	1	2.48	12.38	8.06	7.64	11.2	13.02	12.11	1.59	1
Solyc01g104200.2.1	Fatty acyl coA reductase (AHF	0	0	0	0	0	0	0.12	0	0	0.03	3	1	0	0	0	0	0	0.08	0	0.03	2.67	1	0	0	0	0	0	0	0	0	1	NA	0	0	0	0	0	0	0	1	NA
Solyc01g104210.2.1	Elongation of very long chain	0	0	0	0	0	0.26	0	0.22	0	0.12	12	1	0	0	0	0	0	0	0	0	1	NA	100.29	33.33	104.21	79.28	14.29	35.84	10.51	20.21	0.25	1	0	0.17	0.36	0.18	0.22	0	0.11	0.62	1
Solyc01g104220.2.1	Transmembrane emp24 doma	88.27	76.36	80.02	81.93	81.64	74.71	77.02	78.03	83.38	78.28	0.96	1	88.73	87.77	78.48	84.99	94.73	93.33	82.76	90.27	1.06	1	105.85	110.8	84.85	100.5	98.68	102.59	92.31	97.86	0.97	1	67.82	87.96	83.35	79.71	106.6	100.21	103.41	1.3	1
Solyc01g104230.2.1	F-box family protein (AHRD V	0.56	0.33	0.66	0.79	0.59	0	0.29	0	0.13	0.1	0.18	0.576735	0.52	0.49	0.63	0.55	0.13	0	0.15	0.09	0.17	0.5741	0.15	0.15	0	0.1	0	0	0.14	0.05	0.47	1	0	0	0	0	0	0	0	1	NA
Solyc01g104240.2.1	Hydroxyproline-rich glycoprot	9.99	6.76	8.3	9.55	8.65	8.44	6.11	5.17	4.85	6.14	0.71	0.571581	10.47	9.17	11.64	10.43	11.32	9.54	13.27	11.38	1.09	1	12.5	12.61	14.1	13.07	12.2	11.73	9.94	11.29	0.86	1	22.8	17.06	19.03	19.63	11.59	15.66	13.62	0.69	1
Solyc01g104250.2.1	Rhomboid-like protein (AHRD	21.39	25.62	26.42	30.82	26.06	28.44	29.83	25.18	26.55	27.5	1.06	0.991155	27.15	33.19	34.11	31.48	40.21	34.16	25.14	33.17	1.05	1	36.5	44.64	38.3	39.81	32.18	30.15	34.53	32.29	0.81	1	27.86	27.73	29.2	28.26	28.55	29.27	28.91	1.02	1
Solyc01g104260.2.1	RING finger protein 13 (AHRD	0	0.2	0	0	0.05	0.24	0.17	0	0.31	0.18	3.6	1	0	0	0	0	0	0.23	0.17	0.13	13.33	1	0	0	0	0	0	0	0	0	1	1	0	0.16	0	0.05	0	0	0	0.19	1
Solyc01g104270.2.1	Os12g0283800 protein (Fragr	44.99	41.83	42.1	36.97	41.47	51.96	47.22	41.94	56.2	49.33	1.19	0.665681	46.99	51.12	54.87	50.99	89.86	79.18	72.48	80.51	1.58	0.03408	54.21	57.96	52.39	54.85	72.97	60.86	70.05	67.96	1.24	1	75.75	74.02	66.47	72.08	97.89	93.32	95.6	1.33	1
Solyc01g104280.2.1	Elongation of very long chain	14.22	11.35	19.1	17.34	15.5	37.89	23.81	27.96	22.36	28	1.81	0.03461	11.63	13.43	15.74	13.6	16.33	18.67	16	17	1.25	0.763865	11.69	14.39	12.41	12.83	14.18	14.87	14.48	14.51	1.13	1	8.72	3.74	5.28	5.91	16.16	9.25	12.71	2.15	0.56166
Solyc01g104290.1.1	Thaumatin-like protein 12104	0	0	0	0	0	0	0	0.38	0	0.1	9.5	1	0	0	0	0	0	0	0	0	1	NA	0.17	0.17	0	0.11	0	0	0.15	0.05	0.44	1	0	0	0	0	0.38	0	0.19	19	1
Solyc01g104300.2.1	Unknown Protein (AHRD V1);	15.88	16.39	20.42	11.45	16.04	15.71	14.92	22.04	15.45	17.03	1.06	1	19.64	14.01	15.82	16.49	17.95	14.47	15.72	16.05	0.97	1	11.99	17.4	11.11	13.5	14.39	13.06	12.79	13.41	0.99	1	10.84	14.18	11.6	12.21	14.39	12.32	13.36	1.09	1
Solyc01g104310.2.1	Patatin-like phospholipase do	52.23	47.77	57.86	51.97	52.46	62.32	58.51	60.95	64.11	61.47	1.17	0.678681	61.66	68.18	68	65.95	80.32	84.28	72.18	78.93	1.2	0.674862	46.72	53.63	42.81	47.72	56.14	44.31	59.48	53.31	1.12	1	37.02	41.65	41.76	40.14	49.42	51.26	50.34	1.25	1
Solyc01g104320.2.1	PAP fibrillin (AHRD V1 ***- A	18.06	25.34	23.21	14.73	20.34	54.29	32.89	57.46	33.24	44.47	2.19	0.018147	21.1	21.13	20.33	20.85	16.86	17.98	22.01	18.95	0.91	1	27.3	16.6	15.85	19.92	9.28	12.46	12.99	11.58	0.58	1	4.94	6.72	7.83	6.5	7.63	9.13	8.38	1.29	1
Solyc01g104330.1.1	Unknown Protein (AHRD V1)	0	0	0	0	0	0	0	0	0	0	1	NA	0	0	0	0	0	0	0	0	1	NA	0	0	0	0	0	0	0	0	1	NA	0	0	0	0	0	0	0	1	NA
Solyc01g104340.1.1	Transmembrane protein 222	2.7	3.37	0.32	1.53	1.98	2.4	1.92	1.49	0.78	1.65	0.83	1	1.26	1.41	0.46	1.04	0.75	1.12	2.97	1.61	1.55	1	0.44	0.66	0.54	0.55	0	0.41	0.59	0.33	0.61	1	0.38	0.59	0	0.32	0	0	0	0.03	1
Solyc01g104350.2.1	Unknown Protein (AHRD V1)	26.32	26.88	27.47	26.49	26.79	26.44	21.85	25.29	23.62	24.3	0.91	1	26.37	26.99	27.5	26.95	22.82	30.58	30.89	28.1	1.04	1	43.08	36.98	37.85	39.3	40.79	33.38	32.71	35.63	0.91	1	23.27	28.92	29.06	27.08	26.72	20.86	23.79	0.88	1
Solyc01g104360.1.1	Unknown Protein (AHRD V1)	0	0	0	0	0	0	0	0	0	0	1	NA	0	0	0	0	0	0	0	0	1	NA	0	0	0	0	0	0	0	0	1	NA	0	0	0	0	0	0	0	1	NA
Solyc01g104370.2.1	60S acidic ribosomal protein	495.66	427.1	427.73	404.64	438.78	512.03	467.82	535.17	297.17	453.05	1.03	1	466.48	395.85	302.27	388.2	238.9	236.99	223.9	233.26	0.6	0.091882	307.06	310.18	257.29	291.51	277.29	253.99	268.02	266.43	0.91	1	81.35	274.77	232.52	196.21	294.47	261.06	277.76	1.42	1
Solyc01g104380.2.1	Blue copper protein (AHRD V	4.83	10.5	12.02	3.79	7.79	0	8.05	0.49	0.77	2.33	0.3	0.408774	0	0	0	0	0	0	0	0	1	NA	0	0	0	0	0	0	0	0	1	NA	0	0	0	0	0	0	0	1	NA
Solyc01g104390.1.1	Blue copper protein (AHRD V	0	0	0	0	0	0	0	0	0	0	1	NA	0	0	0	0	0	0	0	0	1	NA	0	0	0	0	0	0	0	0	1	NA	0	0	0	0	0	0	0	1	NA
Solyc01g104400.2.1	Blue copper protein (AHRD V	6.88	9.7	10.15	8.11	8.71	3.83	1.81	5.8	5.35	4.2	0.48	0.374536	1.6	0.5	3.88	1.99	2.39	1.78	0	1.39	0.7	1	0	0	0	0	0	0	1.25	0.42	41.67	1	0	0	0	0	0	0	0	1	NA
Solyc01g104410.2.1	Sterol 3-beta-glucosyltransfer	8.44	9.11	8.32	7.43	8.32	9.69	10.15	12.72	6.45	9.75	1.17	0.919033	8.36	7.33	7.24	7.64	5.47	8.09	9.56	7.71	1.01	1	7.93	6.76	7.41	7.37	4.91	4.82	5.24	4.99	0.68	1	10.03	7.33	7.23	8.2	6.96	7.4	7.18	0.88	1
Solyc01g104420.1.1	Unknown Protein (AHRD V1)	0	0	0	0	0	1	0	0	0	0.25	25	1	0	0	3.82	1.27	0.63	0.94	0	0.52	0.41	1	0	0	0.9	0.3	0.59	0.68	0	0.42	1.41	1	0	0	6.95	2.32	4.14	0.79	2.46	1.06	1
Solyc01g104430.1.1	Unknown Protein (AHRD V1)	5.97	4.68	4.97	7.94	5.89	3.12	2.77	2.9	3.53	3.08	0.52	0.496226	5.55	10.98	10.67	9.07	2.92	8.54	4.14	5.2	0.57	0.755535	4.82	6	4.88	5.23	4.84	4.26	1.79	3.63	0.69	1	9.98	6.12	19.43	11.84	12.53	21.54	17.04	1.44	0.994665
Solyc01g104440.1.1	Unknown Protein (AHRD V1)	0	0	0	0	0	0	0	0	0	0	1	NA	0	0	0	0	0	0	0	0	1	NA	0	0	0	0	0	0	0	0	1	NA	0	0	0	0	0	0	0	1	NA
Solyc01g104450.2.1	Unknown Protein (AHRD V1)	1.43	0	0	4.06	1.37	0	0	0	2.06	0.52	0.38	1	1.33	0	0	0.44	0	0	0	0	0.02	1	2.32	0	0	0.77	0	0	2.09	0.7	0.9	1	0	0	2.21	0.74	0	0	0	0.01	1
Solyc01g104460.2.1	Partner of Y14 and mago (AH	26.27	32.73	31.68	30.55	30.31	19.47	25.35	30.06	20.96	23.96	0.79	0.804781	29.05	27.4	31.81	29.42	21.66	22.82	22.25	22.24	0.76	0.77615	23.82	31.64	25.79	27.08	29.53	24.34	27.87	27.25	1.01	1	36.52	39.01	32.32	35.95	35.81	34.55	35.18	0.98	1
Solyc01g104470.2.1	mRNA turnover protein 4 hor	918.15	946.57	693.72	683.64	810.52	721.2	521.73	721.55	666.37	657.71	0.81	0.625191	930.61	1015.79	872.15	939.52	718.37	923.74	1074.09	905.4	0.96	1	569.83	566.09	391.47	509.13	520.06	591.97	510.21	540.75	1.06	1	445.74	573.11	719.39	579.41	399.1	470.79	434.95	0.75	1
Solyc01g104480.2.1	Unknown Protein (AHRD V1)	33.23	36.53	30.84	34.73	33.83	24.58	27.01	27.41	30.26	27.32	0.81	0.804781	35.11	36.64	41.38	37.71	41.94	39.6	37.06	39.53	1.05	1	32.67	33.45	35.41	33.84	35.31	37.13	32.05	34.83	1.03	1	48.98	52.09	44.52	48.53	41.94	44.45	43.2	0.89	1
Solyc01g104490.2.1	Separin (AHRD V1 *--- C5JQX	0.62	0.99	0.91	0.75	0.82	0.79	0.78	1.03	0.87	0.87	1.06	1	0.37	0.81	0.63	0.6	0.43	0.66	0.41	0.5	0.83	1	0.13	0.2	0.12	0.15	0.08	0.07	0.02	0.06	0.38	1	0	0.09	0.14	0.08	0.14	0.08	0.11	1.43	1
Solyc01g104500.2.1	Os07g0419800 protein (Fragr	107.92	128.57	120.94	132.56	122.5	97.02	93.48	120.69	107.35	104.63	0.85	0.955025	117.69	113.81	117.73	116.41	129.76	131.9	106.88	122.85	1.06	1	545.01	444.4	469.12	486.18	472.95	437.96	503.91	471.61	0.97	1	372.3	375.7	401.62	383.21	402.91	306.71	354.81	0.93	1
Solyc01g104510.2.1	Histone-binding protein RBBP	34.77	26.8	34.25	33.59	32.35	26.57	32.72	30.4	23.29	28.24	0.87	1	35.95	29.55	32.78	32.76	25.3	24.75	24.76	24.94	0.76	0.708092	28.22	30.36	30.04	29.54	30.49	24.12	25.9	26.84	0.91	1	39.06	32.02	37	36.03	35.24	37.16	36.2	1	1
Solyc01g104520.1.1	Kinetochore protein (AHRD V	0.39	0.35	0.46	0	0.3	0	0.31	0.71	0.28	0.33	1.08	1	0	1.69	1.31	1	0	0.2	1.22	0.47	0.47	0.990265	0	0.32	0	0.11	0	0	0.28	0.09	0.88	1	0	0	0	0	0.36	0.34	0.35	35	1
Solyc01g104530.2.1	Protein serine/threonine kina	26.35	25.92	42.89	35.45	32.65	18.72	20.1	21.86	26.88	21.89	0.67	0.382085	28.66	33.41	39.82	33.96	41.29	31.7	31.68	34.89	1.03	1	37.37	37.94	39.53	38.28	37.81	38.29	38.83	38.31	1	1	80.84	61.37	66.56	69.59	49.78	55.58	52.68	0.76	1
Solyc01g104540.2.1	Unknown Protein (AHRD V1)	25.94	28.75	28.84	30.16	28.42	24.26	24.52	26.03	26.5	25.33	0.89	1	35.5	35.34	37.5	36.11	34.86	38.1	32.05	35	0.97	1	43.73	51.73	38	44.49	41.37	37.43	47.26	42.02	0.94	1	34.93	38.68	34.9	36.17	37.1	37.72	37.41	1.03	1
Solyc01g104550.2.1	WRKY transcription factor 32	0	0	0	0	0	0	0	0	0	0	1	NA	0	0	0	0	0	0	0	0	1	NA	0	0	0	0	0	0	0	0	1	NA	0	0	0	0	0	0	0	1	NA
Solyc01g104560.2.1	Pinin/SDK/memA protein (AH	90.93	86.3	94.52	73.98	86.43	85.88	73.42	109.33	69.96	84.65	0.98	1	85.17	75.59	85.26	82.01	59.32	73.67	62.84	65.28	0.8	0.83287	42.51	49.74	35.82	42.69	44.28	30.96	39.15	38.13	0.89	1	120.47	121.35	129.8	123.87	64.17	87.95	76.06	0.61	0.698088
Solyc01g104570.2.1	Kinetochore protein ndc80 (A	8.67	4.77	7.27	8.72	7.36	3.81	4.87	5.67	7.13	5.37	0.73	0.802015	6.16	6.75	5.88	6.26	3.25	5.91	4.13	4.43	0.71	0.791879	0.92	2.05	0.79	1.25	1.27	1.21	1.25	1.24	0.99	1	0.81	0.99	1.23	1.01	2.09	1.7	1.9	1.88	1
Solyc01g104580.2.1	Harpin-induced protein 1 con	27.49	27.81	22.53	24.15	25.49	34.41	36.09	31.92	33.65	34.02	1.33	0.453578	26.11	20.37	20.04	22.17	21.07	34.91	32.97	29.65	1.34	0.462948	7.95	8.49	2.58	6.34	12.55	6.14	10.12	9.6	1.51	1	27.97	15.13	20.47	21.19	15.1	22.58	18.84	0.89	1
Solyc01g104590.2.1	Ribosomal protein L3 (AHRD V	769.32	638.63	807.86	744.13	739.99	540.21	494.12	478.1	554.5	516.73	0.7	0.284723	670.45	562.89	681.51	638.28	497.45	495.11	539.98	510.85	0.8	0.763865	274.15	319.79	314.48	302.81	353.71	295.27	310.63	319.87	1.06	1	341.52	389.34	430.24	387.03	574.95	747.5	661.23	1.71	0.458934
Solyc01g104600.2.1	COP9 signalosome complex su	33.65	24.02	30.66	27.81	29.04	36.67	28.67	33.39	28.94	31.92	1.1	0.980103	25.1	28.67	20.82	24.86	20.1	25.88	24.88	23.62	0.95	1	51.95	36.11	57.54	48.53	38.52	62.63	39.33	46.83	0.96	1	32.21	32.19	26.64	30.35	40.26	38.56	39.41	1.3	1
Solyc01g104610.1.1	Unknown Protein (AHRD V1)	0	0	0	0	0	0	0	0	0	0	1	NA	0	0	0	0	0	0	0	0	1	NA	0	0	0	0	0	0	0	0	1	NA	0	0	0	0	0	0	0	1	NA
Solyc01g104620.2.1	Unknown Protein (AHRD V1)	0	0	0	0	0	0	0	0	0	0	1	NA	0	1.47	0	0.49	1.17	0	1.33	0.83	1.7	1	1.37	6.89	1.68	3.31	1.11	3.85	3.7	2.89	0.87	1	7.22	3.69	5.2	5.37	1.55	2.97	2.26	0.42	1
Solyc01g104630.2.1	Pentatricopeptide repeat-con	4.34	4.99	4.87	5.54	4.93	5.3	4.68	5.06	5.17	5.05	1.02	1	5.22	5.06	4.11	4.8	4.77	7.07	6.32	6.05	1.26	0.745982	2.31	3.71	2.63	2.88	2.71	2.57	4.17	3.15	1.09	1	1.51	3	5.21	3.24	1.65	2.23	1.94	0.6	1
Solyc01g104640.2.1	Helicase (AHRD V1 *--- D3B2	16.51	13.69	15.28	17.52	15.75	12.6	14.76	10.79	13.6	12.94	0.82	0.920923	15.96	17.24	19.35	17.52	15.33	15.09	15.14	15.19	0.87	1	10.4	11.41	10.77	10.86	10.53	10.55	13.27	11.45	1.05	1	5.64	6.82	6.97	6.48	11.86	12.21	12.04	1.86	0.516967
Solyc01g104650.2.1	BZIP transcription factor (AHF	36.53	37.24	46.49	27.56	36.96	32.24	20.27	40.71	23.2	29.11	0.79	0.678217	55.39	65.43	61.89	60.9	97.81	74.5	81.59	84.63	1.39	0.258774	121.08	287.01	263.69	223.93	394.41	373.84	394.92	387.72	1.73	0.369348	81.52	286.85	176.34	181.57	123.01	129.47	126.24	0.7	1
Solyc01g104660.2.1	Exocyst subunit Exo70-intera	18.85	24.71	28	22.14	23.43	24.13	15.94	34.35	21.26	23.92	1.02	1	32.09	40.82	32.84	35.25	49.14	42.26	41.93	44.44	1.26	0.576963	80.22	66.65	79.68	75.52	82.59	88.65	86.09	85.78	1.14	1	86.38	81.04	82.3	83.24	61.47	73.34	67.41	0.81	1
Solyc01g104670.2.1	Tubby-like F-box protein 8 (A	64.28	55.15	52.74	69.55	60.43	49.86	50.14	40.67	42.6	45.82	0.76	0.586137	48.75	57.35	71.99	59.36	55.27	51.72	50.63	52.54	0.89	1	56.91	56.4	66.09	59.8	59.74	67.17	50.88	59.26	0.99	1	70.57	68.96	73	70.84	65.05	70.28	67.66	0.96	1
Solyc01g104680.2.1	GTP-binding nuclear protein F	501.32	429.25	384.6	611.18	481.59	283.09	583.47	334.43	373.14	393.53	0.82	0.999332	442.27	398.85	441.25	427.46	423.14	560.52	548.81	510.82	1.2	0.647547	369.49	617.97	370.77	452.74	425.38	480.42	503.69	469.83	1.04	1	433.5	375.55	367.86	392.3	568.27	600.31	584.29	1.49	0.775382
Solyc01g104690.2.1	Unknown Protein (AHRD V1)	11.43	21.92	13.49	23.64	17.62	7.82	7.64	8.9	8.21	8.14	0.46	0.244449	20.46	11.47	11.89	14.61	4.27	6.38	3.46	4.7	0.32	0.119151	9.95	22.22	6.98	13.05	8.67	4	9.61	7.43	0.57	1	70.04	60.05	60.87	63.65	16.91	13.88	15.39	0.24	0.018582
Solyc01g104700.2.1	GTP-binding nuclear protein F	345.01	296.48	258.74	303.91	301.04	222.64	390.15	244.26	254.09	277.78	0.92	1	287.2	280.28	265.55	277.68	284.43	333.11	322.51	313.35	1.13	0.855973	364.93	470.02	324.17	386.37	444.12	497.09	427.47	456.23	1.18	1	1139.94	619.66	684.99	814.86	703.47	689.42	696.44	0.85	1
Solyc01g104710.2.1	Unknown Protein (AHRD V1)	213.75	293.57	363.22	225.2	273.94	659.99	409.02	808.75	446.47	581.06	2.12	0.030924	183.28	271.44	171.62	208.78	252.57	259.14	235.03	248.91	1.19	0.733007	25.58	50.89	34.92	37.13	41.6	38.67	66.66	48.98	1.32	1	40.02	34.5	20.29	31.6	41.07	57.07	49.07	1.55	0.994665
Solyc01g104720.2.1	Unknown Protein (AHRD V1)	13394.96	14358.91	7171.66	10443.38	11342.23	6486.74	8796.77	8049.08	7724.79	7764.35	0.68	0.304262	18595.35	22669.37	11226.15	17496.96	19426.1	16080.39	12462.74	15989.74	0.91	1	14072.34	14286.46	5239.51	11199.44	7877.08	10268.88	12442.24	10196.07	0.91	1	1406.96	2784.55	2085.45	2092.32	2894.43	3380.49	3137.46	1.5	0.941685
Solyc01g104730.2.1	MAPK activating protein (AHF	117.28	132.65	129.72	67.97	111.91	185.59	110.6	162.5	111.1	142.45	1.27	0.794927	139.4	148.68	112.82	133.63	111.28	166.57	190.1	155.98	1.17	0.855973	43.43	65.7	48.51	52.55	46.01	83.34	81.9	70.42	1.34	1	46.32	51.46	45.09	47.62	54.43	68.55	61.49	1.29	1
Solyc01g104740.2.1	Multiprotein bridging factor 1	20.64	18.77	16.94	16.5	18.21	29.93	8.15	16.09	17.72	17.97	0.99	1	24.63	29.65	32.6	28.96	34.55	29.28	15.17	26.33	0.91	1	10.88	36.2	60.58	35.89	48.94	61.91	52.33	54.39	1.52	0.468126	33.18	24.77	23.46	27.14	34.92	32.27	33.59	1.24	1
Solyc01g104750.2.1	MtN21 nodulin protein-like (A	0	0.15	0.4	0	0.14	0	0	0	0	0	0.07	0.914991	0	0	0	0	0	0.09	0	0.03	3	1	0.14	0	0	0.05	0	0	0.12	0.04	0.86	1	0	0	0	0	0	0	0	1	NA
Solyc01g104760.2.1	Homeodomain-like (AHRD V1	0.86	0.61	0.4	0.73	0.65	0.76	0.14	0.47	0.62	0.5	0.77	1	0.16	0.15	0.14	0.15	0.36	0.18	0.27	0.27	1.8	1	0.42	0.14	0.34	0.3	0	0.13	0	0.04	0.14	1	0	0.25	0	0.08	0	0	0	0.12	1
Solyc01g104770.2.1	Actin (AHRD V1 ***- Q6F4H4	138.05	164.34	209.61	139.97	162.99	80.7	80.88	118.26	103.5	95.83	0.59	0.029215	158.61	144.28	147.56	150.15	124.07	137.99	158.83	140.3	0.93	1	108.85	122.02	94.07	108.31	104.1	101.99	109.76	105.28	0.97	1	219.85	227.41	252.01	233.09	175.42	217.29	196.35	0.84	1
Solyc01g104780.2.1	Nodulin-related integral mem	7.12	9.1	2.92	8.32	6.87	3.79	7.09	9.68	8	7.14	1.04	1	17.29	27.73	12.03	19.02	10.53	5.29	1.7	5.84	0.31	0.163542	2.5	3.53	1.23	2.42	3.87	3.52	2.03	3.14	1.3	1	4.4	6.52	9.05	6.66	1.98	1.9	1.94	0.29	0.431941
Solyc01g104790.2.1	Unknown Protein (AHRD V1)	12.28	10.57	21.01	18.7	15.64	7.8	11.4	10.54	10.75	10.12	0.65	0.537431	10.79	11.38	14.93	12.37	12.35	12.43	14.31	13.03	1.05	1	8.94	12.09	10.58	10.54	8.42	8.79	9.54	8.92	0.85	1	12.66	10.68	12.03	11.79	10.99	12.89	11.94	1.01	1
Solyc01g104800.2.1	Integral membrane protein (A	0	0	0	0	0	0	0	0	0	0	1	NA	0	0	0	0	0	0	0	0	1	NA	0	0	0	0	0	0	0	0	1	NA	0	0	0	0	0	0	0	1	NA
Solyc01g104810.2.1	Integral membrane protein (A	0	0	0	0	0	0	0	0	0	0	1	NA	0	0	0	0	0	0	0	0	1	NA	0	0	0	0	0	0	0	0	1	NA	0	0	0	0	0	0	0	1	NA
Solyc01g104820.2.1	Integral membrane protein (A	0	0	0	0	0	0	0	0	0	0	1	NA	0	0	0	0	0	0	0	0	1	NA	0	0	0	0	0	0	0	0	1	NA	0	0	0	0	0	0	0	1	NA
Solyc01g104830.2.1	Nodulin-related integral mem	0	0	0	0	0	0	0	0	0	0	1	NA	0	0	0	0	0	0	0	0	1	NA	0	0	0	0	0	0	0	0	1	NA	0	0	0	0	0	0	0	1	NA
Solyc01g104840.2.1	RNA-binding protein RZ-1 (AH	346.94	310.87	266.16	274.12	299.52	227.53	241.58	234.5	281.64	246.31	0.82	0.800811	319.43	284.18	249.5	284.37	271.88	303.15	282.14	285.72	1	1	174.41	187.67	153.47	171.85	196.76	188.4	191.87	192.34	1.12	1	193.04	177.76	188.85	186.55	269.33	334.66	302	1.62	0.58776
Solyc01g104850.2.1	Serine carboxypeptidase F13S	20.38	29.54	7.47	13.25	17.66	22.2	12.94	11.4	11.77	14.58	0.83	0.942758	17.81	26.7	35.83	26.78	24.55	22.96	22.4	23.3	0.87	1	0.11	0.56	0.14	0.27	0.81	0.52	0.3	0.54	2.01	1	0	0.2	0	0.07	0	0	0	0.15	1
Solyc01g104860.2.1	Peroxidase (AHRD V1 **** Q	4.32	10.24	8.73	7.56	7.71	4.34	3.73	6.05	3.39	4.38	0.57	0.241245	2.49	2.14	1.22	1.95	0	0.53	0.97	0.5	0.26	0.166827	0	0	0	0	0	0	0	0	1	NA	0	0	0	0	0	0	0	1	NA
Solyc01g104870.2.1	Phosphatidylinositol binding	0	0	0	0	0	0	0	0	0	0	1	NA	0	0	0	0	0	0	0	0	1	NA	0	0	0	0	0	0	0	0	1	NA	0	0	0	0	0	0	0	1	NA
Solyc01g104880.2.1	Phosphatidylserine decarboxy	23.79	28.41	20.51	20.56	23.32	21.58	18.38	25.7	18.54	21.05	0.9	0.993327	23.07	33.31	29.19	28.52	15.72	20.37	22.45	19.51	0.68	0.301793	10.84	11.43	13.32	11.86	11.15	11.48	10	10.88	0.92	1	27.6	28.28	29.39	28.42	21.03	20.14	20.59	0.72	1
Solyc01g104890.2.1	Unknown Protein (AHRD V1)	140.19	155.96	70.54	160.39	131.77	206.25	136.72	153.54	119.89	154.1	1.17	0.951897	145.74	168.46	125.37	146.52	138.7	170.79	187.94	165.81	1.13	0.974398	129.92	156.23	79.81	121.99	101.57	137.5	112.67	117.25	0.96	1	49.83	74.87	41.21	55.3	58.5	57.84	58.17	1.05	1
Solyc01g104900.2.1	NAC domain protein IPR0034	2.65	4.43	2.35	1.41	2.71	7.37	4.45	8.23	3.09	5.79	2.13	0.347976	7.09	5.48	3.92	5.5	10.12	10.81	18.5	13.14	2.39	0.083612	4.55	3.24	1.97	3.25	1.31	0.75	0.48	0.85	0.26	1	0	1.93	0.51	0.81	0	0	0	0.01	1
Solyc01g104910.2.1	Os11g0223400 protein (Fragr	76.18	95.59	181.69	76.84	107.58	79.06	45.49	65.39	80.07	67.5	0.63	0.639388	60.12	57.17	70.87	62.72	76.86	68.74	56.06	67.22	1.07	1	149.15	127.17	124.62	133.65	67.65	78.52	83.3	76.49	0.57	1	16.71	36.09	16.8	23.2	39.91	41.3	40.6	1.75	0.796983
Solyc01g104920.2.1	26S protease regulatory subu	61.5	68.37	79.97	79.18	72.25	35.13	42.46	43.15	54.68	43.86	0.61	0.085026	73.76	87.39	85.31	82.15	66.22	67.67	62.24	65.38	0.8	0.784393	40.69	42.74	39.97	41.13	44.65	44.09	33.16	40.63	0.99	1	73.63	60.57	56.21	63.47	60.39	68.53	64.46	1.02	1
Solyc01g104930.1.1	Vesicle-fusing ATPase (AHRD	0.36	2.55	2.96	5.07	2.73	1.2	0.85	0.33	0.77	0.79	0.29	0.441679	2.33	2.18	3.33	2.61	2.74	2.04	2.82	2.53	0.97	1	0.58	0.88	1.42	0.96	2.59	0.54	1.83	1.65	1.72	1	1.53	0.26	0.55	0.78	1.64	4.09	2.87	3.67	0.773191
Solyc01g104940.1.1	F-box (AHRD V1 *-*- Q84KQ8	0	0	0	0	0	0	0	0	0	0	1	NA	0.19	0	0.35	0.18	0	0	0	0	0.06	0.962977	0	0	0	0	0	0	0	0	1	1	0.29	0	0	0.1	0	0	0	0.1	1
Solyc01g104950.2.1	Alpha-L-arabinofuranosidase/	30.83	42.13	44.76	35.7	38.36	40.01	41.99	28.73	40.92	37.91	0.99	1	17.72	14.87	19.1	17.23	11.75	11.44	13.24	12.14	0.7	0.421474	14.85	6.21	8.91	9.99	2.39	2.31	3.46	2.72	0.27	1	0	0.68	0.65	0.44	0.47	0.45	0.46	1.04	1
Solyc01g104960.1.1	Unknown Protein (AHRD V1)	0	0	0	0	0	0	0	0	0	0	1	NA	0	0	0	0	0	0	0	0	1	NA	0	0	0	0	0	0	0	0	1	NA	0	0	0	0	0	0	0	1	NA
Solyc01g104970.2.1	Receptor like kinase, RLK	14.01	13.7	13.75	20.57	15.51	9.75	9.54	6.59	8.44	8.58	0.55	0.057419	11.74	16.73	16.53	15	10.38	7.7	11.01	9.7	0.65	0.201318	8.47	6.8	5.41	6.89	5.9	4.05	2.73	4.23	0.61	1	3.5	5.36	5.26	4.71	2.74	4.31	3.52	0.75	1
Solyc01g104980.2.1	Kelch-like protein 14 (AHRD V	33.82	37.25	45.46	34.5	37.76	29.43	21.45	23.57	33.63	27.02	0.72	0.428133	39.71	42.41	43.2	41.77	49.12	47.65	41.93	46.23	1.11	0.948678	32.92	38.85	31.37	34.38	33.05	34.03	39.59	35.56	1.03	1	59.71	51.74	57.12	56.19	43.9	51.52	47.71	0.85	1
Solyc01g104990.2.1	Trimethylguanosine synthase	2.99	4.59	3.84	2.69	3.53	3.32	0.86	6.49	3.9	3.64	1.03	1	3.79	2.36	3.21	3.12	3.58	4.36	4.27	4.07	1.3	0.962977	1.54	1.99	1.08	1.54	1.07	1.65	0.59	1.1	0.72	1	1.54	1.58	0.42	1.18	1.74	1.9	1.82	1.54	1
Solyc01g105000.2.1	Copper chaperone (AHRD V1	0	0	0	0	0	0	0.29	0	0.26	0.14	13.75	1	0	0	0	0	0	0	0	0	1	NA	0	0	0	0	0	0	0	0	1	NA	0	0	0	0	0	0	0	1	NA
Solyc01g105010.2.1	Non-specific lipid-transfer pro	33.87	34.54	32.88	67.87	42.29	29.41	65.73	27.57	28.11	37.7	0.89	1	42	43.55	49.17	44.91	36.21	46.76	41.03	41.33	0.92	1	57.57	59.79	48.67	55.34	79.49	44.92	51.93	58.78	1.06	1	10.53	16.91	9.22	12.22	29.39	26.6	28	2.29	0.44125
Solyc01g105020.2.1	Phosphatase PTC7 homolog (A	4.58	5.3	2.81	2.66	3.84	8.71	5.2	6.98	7.29	7.04	1.84	0.043079	4.96	2.34	2.86	3.39	2.57	3.57	4.63	3.59	1.06	1	1.87	2.25	1.12	1.75	1.03	1.14	2.47	1.55	0.89	1	0.45	0.91	1.16	0.84	1.09	0.93	1.01	1.2	1
Solyc01g105030.2.1	Chlorophyll a-b binding prote	242.58	186.65	208.36	175.74	203.33	302.17	217.45	166.86	210.27	224.19	1.1	0.980348	190.06	141.92	161.53	164.5	158.63	102.21	117.21	126.02	0.77	0.680536	44.38	47.69	30.07	40.71	41.15	21.4	52.56	38.37	0.94	1	1.09	3.36	4.34	2.93	7.75	9.23	8.49	2.9	0.372318
Solyc01g105040.2.1	A_IG002N01.30 protein (Frag	2.2	4.48	6.87	4.23	4.45	4.73	3.23	3.76	2.94	3.67	0.82	0.998465	3.81	4.93	5.98	4.91	5.9	4.4	4.45	4.92	1	1	3.56	3.85	2.03	3.15	4.45	2.15	3.56	3.39	1.08	1	5.15	6.18	6.05	5.79	3.17	3.59	3.38	0.58	0.935807
Solyc01g105050.2.1	Chlorophyll a-b binding prote	149.65	130.68	105.09	81.33	116.69	200.88	148.34	92.99	134.16	144.09	1.23	0.899057	93.12	74.98	92.15	86.75	116.97	61.27	76.86	85.03	0.98	1	24.06	20.08	16.82	20.32	25.29	15.56	30.68	23.84	1.17	1	0.36	2.42	1.58	1.45	0.23	0.68	0.46	0.31	0.982171
Solyc01g105060.2.1	(3R)-hydroxymyristoyl- (AHRD	51.96	51.18	27.21	44.18	43.63	73.01	82.5	50.99	74.82	70.33	1.61	0.056806	39.37	35.02	22.93	32.44	35.43	45.95	42.67	41.35	1.27	0.548678	36.32	43.77	28.11	36.07	35.48	38.5	36.37	36.78	1.02	1	2.49	11.24	7.18	6.97	26.99	23.8	25.39	3.64	0.049168
Solyc01g105070.2.1	Peroxidase (AHRD V1 **** Q	2.58	4.58	7.95	14.29	7.35	1.76	10.64	2.19	5.26	4.96	0.68	1	18.06	22.56	18.07	19.56	19.79	5.64	4.83	10.09	0.52	0.503316	0.32	0.81	0.39	0.51	1.3	1.35	1.01	1.22	2.41	1	3.94	2.73	2.74	3.14	1.99	2.26	2.12	0.68	1
Solyc01g105080.2.1	Receptor like kinase, RLK	0	0	0	0	0	0	0	0	0	0	1	NA	0	0	0	0	0	0	0	0	1	NA	0	0	0	0	0	0	0	0	1	NA	0	0	0	0	0	0	0	1	NA
Solyc01g105090.2.1	Chloroplastic group IIA intron	13.69	15.93	22.56	10.42	15.65	13.65	8.03	14.2	14.61	12.62	0.81	0.888972	12.89	14.03	13.19	13.37	21.54	20.17	17.93	19.88	1.49	0.217609	5.95	6.15	5.75	5.95	7.15	4.65	6.52	6.11	1.03	1	30.93	18.73	17.27	22.31	14.13	15.54	14.84	0.66	1
Solyc01g105100.2.1	Endoplasmic reticulum-Golgi	49.09	59.32	66.37	62.69	59.37	55.33	43.2	67.24	40.51	51.57	0.87	0.95632	53.28	61.64	66.38	60.43	51.31	50.93	64.71	55.65	0.92	1	54.54	50.98	54.15	53.22	43.9	38.76	45.09	42.58	0.8	1	94.04	93.85	89.39	92.43	45.72	59.94	52.83	0.57	0.528529
Solyc01g105110.1.1	Unknown Protein (AHRD V1)	0	0	0	0	0	0	0	0	0	0	1	NA	0	0	0	0	1.73	0	0	0.58	57.67	1	0	0	0	0	0	0	0	0	1	NA	0	0	0	0	0	0	0	1	NA
Solyc01g105120.2.1	Os01g0498200 protein (Fragr	42.01	42.57	40.95	17.99	35.88	68.4	30.91	63.19	56.78	54.82	1.53	0.478089	47.42	20.49	15.74	27.88	13.61	10.26	22.01	15.29	0.55	0.833891	34.66	13.34	20.95	22.98	11.47	8.07	13.44	10.99	0.48	1	27.48	31.59	35.03	31.37	17.51	27.93	22.72	0.72	1
Solyc01g105130.2.1	SKIP interacting protein 15 (A	55.82	47.42	48.4	41.87	48.38	49.83	57.09	53.27	51.92	53.03	1.1	0.965954	45.63	56.98	39.29	47.3	33.14	38.76	43.2	38.37	0.81	0.930424	38.95	43.42	29.8	37.39	33.62	42.95	32.67	36.41	0.97	1	30.66	110.27	91.69	77.54	46.84	50.06	48.45	0.62	1

Solyc01g105140.2.1	RNA-binding protein (AHRD V	103.23	75.29	83.02	76.48	84.51	66.72	69.65	62.56	73.92	68.21	0.81	0.787474	81.58	102.33	96.58	93.5	101.92	98.86	82.75	94.51	1.01	1	84.65	124.8	111.7	107.05	135.28	112.36	105.57	117.74	1.1	1	112.92	100.34	125.71	112.99	114.62	118.41	116.52	1.03	1
Solyc01g105150.2.1	Potassium transporter family	8.27	7.7	6.23	7.79	7.5	6.21	7.49	5.42	6.54	6.41	0.86	0.994382	7.47	7.72	8.27	7.82	6.1	6.62	7.88	6.87	0.88	1	6.68	5.68	6	6.12	5.18	5.92	5.63	5.58	0.91	1	7.94	7.95	7.53	7.81	6.66	6.37	6.52	0.83	1
Solyc01g105160.2.1	Copper-transporting P-type A	8.71	8.56	11.56	8.6	9.36	10.39	8.1	10.35	10.62	9.87	1.05	1	9.45	9.7	9.81	9.65	11.39	11.51	8.84	10.58	1.1	0.985957	6.01	9.91	9.01	8.31	7.99	7.7	7.01	7.57	0.91	1	14.32	8.7	8.37	10.46	8.56	10.82	9.69	0.93	1
Solyc01g105170.2.1	Copper-exporting P-type ATP	85.62	79.2	75.51	76.64	79.24	94.86	124.78	89.56	110.44	104.91	1.32	0.297366	83.04	56.37	67.1	68.84	75.35	72.53	78.54	75.47	1.1	0.976783	49.9	56.96	46.53	51.13	53.3	47.24	54.62	51.72	1.01	1	29.94	46.9	32.81	36.55	43.94	39.63	41.78	1.14	1
Solyc01g105180.2.1	LIM zinc-binding protein (AHF	18.78	13.55	9.38	13.4	13.78	6.02	4.41	13.84	7.04	7.83	0.57	0.312066	7.55	19.7	12.43	13.23	5.63	4.29	7.98	5.97	0.45	0.33385	1.91	1.79	1.68	1.79	2.56	1.54	0.86	1.65	0.92	1	38.48	6.02	9.36	17.95	1.55	2.22	1.89	0.1	0.948615
Solyc01g105190.2.1	Unknown Protein (AHRD V1)	0	0	0	0	0	0	0	0	0	0	1	NA	0	0	0	0	0	0	0	0	1	NA	0.72	0	0	0.24	0	0	0	0	0.04	1	0	0.65	1.37	0.67	0	0	0	0.01	1
Solyc01g105200.2.1	Zinc ion binding protein (AHR	0	0	0	0	0	0	0	0	0.29	0.07	7.25	1	0	0	0.34	0.11	0	0	0	0	0.09	1	0	0	0.4	0.13	0	0	0	0	0.07	NA	0	0.29	0	0.1	0	0	0	0.1	1
Solyc01g105210.2.1	Zinc ion binding protein (AHR	1.62	0.96	0	0.76	0.83	0	0	0	1.16	0.29	0.35	0.995762	2.01	2.35	0.91	1.76	0.38	0.28	0.85	0.5	0.29	0.749236	0.44	0.88	0.54	0.62	0.36	0.41	0	0.26	0.41	1	2.31	0.79	0	1.03	0.5	0.47	0.48	0.47	1
Solyc01g105220.2.1	Alternative oxidase (AHRD V1	3.4	4.71	4.91	4.55	4.39	3.36	2.24	3.65	2.58	2.96	0.67	0.689959	4.04	5.92	4.15	4.7	2.88	3.43	4.01	3.44	0.73	0.91417	2.6	3.39	1.69	2.56	3.23	2.58	1.38	2.4	0.94	1	6.72	8.37	5.81	6.97	4.5	4.81	4.66	0.67	1
Solyc01g105230.2.1	Calmodulin-binding transcript	16.22	16.13	16.67	23.22	18.06	12.47	12.96	17.01	13.92	14.09	0.78	0.76553	16.86	19.17	18.89	18.31	13.27	13.95	15.59	14.27	0.78	0.727965	16.64	15.49	24.66	18.93	16.9	17.2	15.75	16.62	0.88	1	41.18	53.25	64.32	52.92	31.21	33.73	32.47	0.61	0.682649
Solyc01g105240.2.1	Ubiquitin carboxyl-terminal h	50.69	63.51	61.93	67.63	60.94	47.56	45.84	50.74	44.14	47.07	0.77	0.641705	58.69	59.69	58.61	59	58.72	50.54	43.23	50.83	0.86	1	61.43	59.47	59.06	59.99	33.89	43.05	39.69	38.88	0.65	1	69.52	55.23	53.35	59.37	55.12	46.97	51.05	0.86	1
Solyc01g105250.1.1	Unknown Protein (AHRD V1)	0	0	0	0	0	0	0	0	0	0	1	NA	0	0	0	0	0	0	0	0	1	NA	0	0	0	0	0	0	0	0	1	NA	0	0	0	0	0	0	0	1	NA
Solyc01g105260.1.1	Integral membrane protein (A	0	0	0	0	0	0	0	0	0	0	1	NA	0	0	0	0	0	0	0	0	1	NA	0	0	0	0	0	0	0	0	1	NA	0	0	0	0	0	0	0	1	NA
Solyc01g105270.2.1	Tobamovirus multiplication p	9.52	11.43	6.53	4.7	8.04	19.19	11.71	16.69	9.54	14.28	1.78	0.312786	10.5	10.39	7.67	9.52	8.44	12.04	14.96	11.81	1.24	0.838211	8.76	12.27	7.47	9.5	8.87	7.38	9.68	8.64	0.91	1	4.09	9.65	12.6	8.78	7.7	10.09	8.89	1.01	1
Solyc01g105280.2.1	Protein phosphatase 2C-like (	28.34	35.56	37.27	39.1	35.07	27.37	36.68	43.14	35.02	35.55	1.01	1	34.36	49.07	46.3	43.24	38.02	38.25	47.65	41.31	0.96	1	57.61	61.71	68.71	62.68	73.25	80.81	64.83	72.96	1.16	1	88.84	161.97	157.17	135.99	116.32	104.69	110.5	0.81	1
Solyc01g105290.2.1	Genomic DNA chromosome 5	20.97	20.03	30.37	22.91	23.57	23.88	16.15	26.64	17.87	21.14	0.9	1	18.55	23.41	27.21	23.06	21.75	19.15	14.2	18.37	0.8	0.890501	22.31	18.75	22.34	21.13	22.29	12.79	13.42	16.17	0.76	1	24.72	18.7	20.18	21.2	12.95	19.58	16.27	0.77	1
Solyc01g105300.2.1	Metacaspase (AHRD V1 ***-	2.37	1.95	1.08	1.55	1.74	1.22	1.01	1.17	0.52	0.98	0.56	0.650008	0.34	0.16	0.46	0.32	1.14	0.09	0.14	0.46	1.43	1	0.59	0.59	0.9	0.69	0.36	0.14	0.4	0.3	0.43	1	3.11	0.4	0	1.17	0	0	0	0.01	1
Solyc01g105310.2.1	Metacaspase (AHRD V1 ***-	1.45	1.78	3.65	1.8	2.17	0.61	1.29	0.84	1.17	0.98	0.45	0.480467	2.37	6.16	5.99	4.84	2.52	2.16	0.57	1.75	0.36	0.284943	2.2	3.41	0.54	2.05	3.22	3.03	0.53	2.26	1.1	1	0.52	0	1.12	0.55	0.33	0.8	0.57	1.03	1
Solyc01g105320.2.1	Metacaspase (AHRD V1 ***-	0.37	0.49	0.22	0.79	0.47	1.03	1.02	0.85	0.27	0.79	1.7	0.931315	1.55	1.61	0.94	1.37	2.7	0.67	0.15	1.17	0.86	1	0.45	0.15	0	0.2	0.24	0.42	0.68	0.45	2.23	1	1.58	1.48	1.14	1.4	0.51	0.81	0.66	0.47	1
Solyc01g105330.2.1	Methyl binding domain prote	5.57	5.28	8.22	6.74	6.45	4.07	3.7	5.32	4.95	4.51	0.7	0.564428	6.07	6.82	7.99	6.96	6.76	6.68	6.68	6.71	0.96	1	6.04	4.6	5.02	5.22	6.16	4.39	4.65	5.07	0.97	1	14.59	9.26	10.82	11.56	8.32	8.87	8.59	0.74	1
Solyc01g105340.2.1	Chaperone protein dnaJ (AHR	459.93	398.72	647.28	640.56	536.62	507.52	546.28	397.69	545.07	499.14	0.93	1	545.7	600.36	784.34	643.47	887.2	879.99	675.59	814.26	1.27	0.415933	518.83	746.23	831.85	698.97	927.49	786.46	703.59	805.85	1.15	1	360.88	617.62	645.36	541.29	918.99	840.19	879.59	1.62	0.672787
Solyc01g105350.1.1	UDP-glucuronosyltransferase	5.53	8.64	4.62	2.86	5.41	14.1	3.73	27.29	13.95	14.77	2.73	0.404581	2.51	1.06	0.57	1.38	1.03	4.26	3.18	2.82	2.05	0.682007	2.29	0.99	0.8	1.36	1.6	3.78	3.15	2.84	2.09	1	1.92	6.47	3.73	4.04	8.27	5.91	7.09	1.75	1
Solyc01g105360.2.1	UDP-glucuronosyltransferase	21.95	27.51	19.1	15.9	21.12	38.67	40.36	68.16	52.17	49.84	2.36	0.000655	13.82	1.51	1.92	5.75	11.42	7.41	9.99	9.61	1.67	0.904471	0.65	2.4	0.53	1.19	0.88	0.51	0.59	0.66	0.55	1	0	0.39	0.21	0.2	0.61	0.47	0.54	2.7	1
Solyc01g105370.2.1	Enoyl-(Acyl-carrier-protein) re	9.73	10.05	11.02	4.4	8.8	6.49	7.07	7.16	7.68	7.1	0.81	0.883084	7.06	4.06	3.78	4.97	2.43	3.33	3.21	2.99	0.6	0.641696	9.75	16.33	5.79	10.62	5.63	5.16	10.35	7.05	0.66	1	1.94	2.12	2.09	2.05	1.6	2.73	2.17	1.06	1
Solyc01g105380.1.1	Unknown Protein (AHRD V1)	20.55	15.4	16.59	13.77	16.58	19.23	30.74	30.83	14.75	23.89	1.44	0.680258	23.14	25.38	15.53	21.35	57.76	25.76	19.55	34.36	1.61	0.878616	20.1	29.96	16.1	22.05	22.02	27.87	22.85	24.25	1.1	1	18.45	87.19	58.21	54.62	23.76	24.65	24.2	0.44	0.868616
Solyc01g105390.2.1	Phospho-2-dehydro-3-deoxyh	40.07	33.51	48.08	32.25	38.48	43	35.72	32.39	42.65	38.44	1	1	31.81	38.43	45.22	38.49	86.24	47.75	49.13	61.04	1.59	0.55075	33.49	56.01	54.51	48	38.57	49.48	45.81	44.62	0.93	1	58.44	85.8	54.18	66.14	65.45	61.78	63.62	0.96	1
Solyc01g105400.2.1	ABC transporter G family mer	0	0	0	0	0	0	0	0.52	0	0.13	13	1	0	0.49	0.48	0.32	0	0	0	0	0.03	1	0	0	0	0	0	0	0	0	1	NA	0.8	0	0	0.27	0	0	0	0.04	1
Solyc01g105410.2.1	Os06g0220000 protein (Fragr	63.13	79.91	114.43	56.85	78.58	44.44	25.87	57.04	38.74	41.52	0.53	0.132177	123.34	76.91	80.81	93.69	80.76	51.8	73.13	68.56	0.73	0.774176	16.59	45.04	35.56	32.4	37.74	18.05	6.84	20.88	0.64	1	79.82	52.99	84.65	72.49	59.39	80.83	70.11	0.97	1
Solyc01g105420.2.1	Phospho-2-dehydro-3-deoxyh	94.07	83.72	103.78	92.16	93.43	119.05	93.56	96.6	104.08	103.32	1.11	0.908006	78.79	82.48	106.97	89.41	185.85	113.02	118.73	139.2	1.56	0.387168	82.25	129.08	114.8	108.71	88.28	118.52	112.17	106.32	0.98	1	132.59	220.05	157.18	169.94	149.49	129.61	139.55	0.82	1
Solyc01g105430.2.1	Unknown Protein (AHRD V1)	4.63	3.09	1.37	0.82	2.48	3.22	3.65	4.26	2.91	3.51	1.42	1	2.69	4.02	2.44	3.05	1.61	5.39	2.73	3.24	1.06	1	1.87	1.41	0.57	1.28	1.9	0.44	1.27	1.2	0.94	1	0.82	2.52	0.89	1.41	2.12	2.03	2.08	1.47	1
Solyc01g105440.1.1	Unknown Protein (AHRD V1)	0	0	0	0	0	0	0	0	0	0	1	NA	0	0	0	0	0	0	0	0	1	NA	0	0	0	0	0	0	0	0	1	1	0	0	0	0	0	0	0	1	NA
Solyc01g105450.2.1	ABC transporter G family mer	1.79	3.09	2.48	1.93	2.32	4.44	2.32	4.73	5.28	4.19	1.81	0.165925	0.2	0.46	0.18	0.28	0	0	0	0	0.04	0.270372	0.34	0.17	0.1	0.2	0	0.08	0.08	0.05	0.26	1	0	0	0.16	0.05	0	0	0	0.19	1
Solyc01g105460.2.1	1 4-dihydroxy-2-naphthoate	15.99	20.52	19.66	19.52	18.92	32.92	31.49	24.45	29.12	29.5	1.56	0.083412	18	18.58	16.77	17.78	23.58	21.61	25.92	23.7	1.33	0.497007	14.41	24.27	14.2	17.63	16.36	15.18	21.34	17.63	1	1	4.55	7.82	6.73	6.37	11.09	9.74	10.41	1.64	0.95816
Solyc01g105470.2.1	Unknown Protein (AHRD V1)	11.23	8.74	14.9	3.97	9.71	14.04	27.7	7.74	15.12	16.15	1.66	0.65798	11.75	12.2	16.6	13.52	8.76	8.72	8.82	8.77	0.65	0.960051	5.67	6.86	8.36	6.96	0	4.26	2.05	2.1	0.3	1	3.99	2.04	6.48	4.17	2.57	4.92	3.75	0.9	1
Solyc01g105480.2.1	Unknown Protein (AHRD V1)	58.64	45.67	84.75	53.92	60.75	48.89	55.55	37.74	33.68	43.97	0.72	0.748411	69.55	61.17	52.01	60.91	45.76	39.47	34.56	39.93	0.66	0.628165	29.61	33.45	27.65	30.24	26.97	26.67	20.3	24.65	0.82	1	72.95	10.65	13.53	32.38	25.5	25.71	25.61	0.79	1
Solyc01g105490.2.1	Unknown Protein (AHRD V1)	78.84	81.63	72.14	79.66	78.07	87.64	92.73	112.44	91.26	96.02	1.23	0.760208	118.24	107.7	129.11	118.35	99.32	124.97	136.57	120.29	1.02	1	29.9	35.4	43.12	36.14	34.88	43.9	43.38	40.72	1.13	1	70.92	50.25	39.59	53.59	30.93	74.77	52.85	0.99	1
Solyc01g105500.2.1	Endo-1 4-beta-xylanase (AHR	0	0.2	0.81	0	0.25	0	0	0	0	0	0.04	0.667623	0.21	0	0.1	0.1	0	0	0	0	0.1	0.962977	0	0	0	0	0	0	0	0	1	1	0	0	0	0	0	0	0	1	NA
Solyc01g105510.2.1	LMBR1 domain protein (AHR	10.3	8.45	8.32	10.48	9.39	9.84	9.77	10.8	11.29	10.43	1.11	0.90174	9.16	11.47	11.53	10.72	7.46	9.17	9.3	8.64	0.81	0.911351	4.31	6.11	3.23	4.55	3.38	2.05	3.36	2.93	0.64	1	4.37	3.61	5.14	4.37	2.32	3.41	2.87	0.66	1
Solyc01g105520.2.1	DNA replication licensing fact	4.39	3.71	4.66	3.73	4.12	5	5.03	2.93	4.41	4.34	1.05	1	3.98	3.53	4.73	4.08	2.66	3.3	1.73	2.56	0.63	0.626814	1.06	1.34	1.42	1.27	0.07	0.67	0.8	0.51	0.4	1	0.78	2.63	1.86	1.76	3.11	3.08	3.09	1.76	1
Solyc01g105530.2.1	DNA replication licensing fact	14.76	11.59	12.29	9.82	12.12	8.68	14.39	4.79	12.46	10.08	0.83	1	20.99	9.81	15.4	15.4	4.21	10.34	1.36	5.3	0.34	0.34075	2.81	4.95	3.45	3.74	0.57	0	1.9	0.82	0.22	1	1.23	7.57	0	2.93	7.95	5.33	6.64	2.26	1
Solyc01g105540.2.1	2-oxoglutarate/malate transl	34.89	38.56	48.39	45.6	41.86	56.76	51.9	38.77	46.2	48.41	1.16	0.706335	32.24	27.12	41.57	33.64	32.61	35.83	34.87	34.44	1.02	1	18.2	25.33	25	22.84	20.34	16.46	21.13	19.31	0.85	1	6.41	6.63	7.11	6.72	12.8	16.61	14.71	2.19	0.236663
Solyc01g105550.1.1	Hydroxycinnamoyl CoA quina	0	0	0	0	0	0	0	0	0	0	1	NA	0	0	0	0	0	0	0	0	1	NA	0	0	0	0	0	0	0	0	1	NA	0	0	0	0	0.13	0	0.07	6.5	1
Solyc01g105560.2.1	3 4-dihydroxy-2-butanone 4-p	34.83	32.28	22.24	38.64	32	34.21	36.54	24.68	28.18	30.9	0.97	1	20.09	24.47	28.62	24.39	33.68	33.17	29.63	32.16	1.32	0.409992	45.11	52.4	82.89	60.13	72.81	66.19	68.59	69.2	1.15	1	170.12	195.56	168.41	178.03	83.43	71.54	77.49	0.44	0.068532
Solyc01g105570.2.1	Genomic DNA chromosome 5	22.6	24.33	22.64	21.56	22.78	25.13	23.24	26.58	20.38	23.83	1.05	1	25.67	23.08	24.86	24.54	22.24	20.14	21.48	21.29	0.87	1	20.17	20.35	21.39	20.64	15.02	16.73	19.92	17.22	0.83	1	21.67	15.65	19	18.77	16.36	19.13	17.74	0.95	1
Solyc01g105580.1.1	Hydroxycinnamoyl CoA quina	0	0	0	0	0	0	0	0	0	0	1	NA	0	0	0	0	0	0	0	0	1	NA	0	0	0	0	0	0	0	0	1	NA	0	0	0	0	0	0	0	1	NA
Solyc01g105590.2.1	Hydroxycinnamoyl CoA quina	0	0	0	0	0	0	0	0	0	0	1	NA	0	0	0	0	0	0	0	0	1	NA	0	0	0	0	0	0	0	0	1	NA	0	0	0	0	0	0	0	1	NA
Solyc01g105600.2.1	Unknown Protein (AHRD V1)	0	0.55	1.46	0	0.5	0	0	0	0	0	0.02	0.645395	0	0	0.26	0.09	0.21	0.16	0.49	0.29	3.31	1	2.5	0	1.84	1.45	1.63	0.94	0.68	1.08	0.75	1	0.44	2.02	0.48	0.98	0.28	0.27	0.28	0.28	1
Solyc01g105610.1.1	RING finger protein (AHRD V1	0	0	0	0	0	0	0	0	0	0	1	NA	0	0	0	0	0	0	0	0	1	NA	0	0	0	0	0	0	0	0	1	NA	0	0	0	0	0	0	0	1	NA
Solyc01g105620.2.1	RING finger protein (AHRD V1	1.87	3.66	3.31	1.32	2.54	2.08	0.59	1.89	1.34	1.48	0.58	0.530724	5.22	3.41	2.05	3.56	1.3	1.45	1.76	1.5	0.42	0.340797	2.42	3.35	2.23	2.67	0.49	0.85	1.09	0.81	0.3	1	1.86	3.26	2.87	2.66	0.51	0.33	0.42	0.16	0.231088
Solyc01g105630.2.1	Calmodulin (AHRD V1 ***- Q	3.57	1.59	2.1	0	1.81	0	0.35	2.46	0	0.7	0.39	0.762692	2.49	0.39	3.01	1.96	0	0.46	0.35	0.27	0.14	0.339946	0.36	1.09	0	0.48	0.59	0.34	0	0.31	0.64	1	0	2.91	2.06	1.66	0.82	1.95	1.39	0.84	1
Solyc01g105640.2.1	Ulp1 protease family C-termi	1.14	1.01	1.34	1.61	1.27	5.06	4.49	2.09	0	2.91	2.28	0.929705	1.06	1.98	3.84	2.29	1.58	4.71	2.68	2.99	1.3	1	2.76	0	0	0.92	0	0.86	0	0.29	0.31	1	0	0.83	0	0.28	2.08	0	1.04	3.76	1
Solyc01g105650.2.1	1-aminocyclopropane-1-carb	2.59	5.59	11.55	1.31	5.26	0.41	2.48	8.84	1.86	3.4	0.65	1	14.61	1.12	2.81	6.18	5.77	0.57	1.6	2.65	0.43	1	0	5.42	0.55	1.99	2.91	0.28	0.94	1.38	0.69	1	0.26	0	0	0.09	0	0	0	0.12	1
Solyc01g105660.2.1	1-aminocyclopropane-1-carb	253.38	240.27	259.8	111.04	216.12	269.36	254.74	252.11	256.52	258.18	1.19	0.754404	262.77	247.65	213.7	241.37	265.08	279.18	283.99	276.08	1.14	0.819112	483.71	487.42	317.53	429.55	394.65	402.11	409.57	402.11	0.94	1	183.4	356.15	207.14	248.9	303.64	289.86	296.75	1.19	1
Solyc01g105670.2.1	Calcium-dependent protein k	43.24	41.26	35.41	29.21	37.28	33.2	35.66	42.08	42.24	38.3	1.03	1	36.07	35.78	29.62	33.82	33.29	37.39	39.23	36.64	1.08	1	44.66	40.68	25.66	37	31.82	31.76	36.27	33.28	0.9	1	19.44	29.25	31.99	26.89	21.35	16.55	18.95	0.7	0.968537
Solyc01g105680.2.1	Phototropic-responsive NPH3	0	0	0.12	0	0.03	0	0	0	0	0	0.33	1	0	0	0.08	0.03	0.2	0.05	0	0.08	3.12	1	0	0.08	0	0.03	0	0	0	0	0.38	1	0	0.07	0	0.02	0	0	0	0.43	1
Solyc01g105690.2.1	Transcription factor/ transcri	23.61	22.58	17.18	14.98	19.59	23.3	26.3	22.92	21.86	23.6	1.2	0.805735	22.99	20.14	17.14	20.09	20.81	21.82	21.85	21.49	1.07	1	3.92	8.81	5.69	6.14	6.67	6.02	8.04	6.91	1.13	1	2.82	7.37	3.05	4.41	8.08	11.03	9.55	2.17	0.811923
Solyc01g105700.2.1	Unknown Protein (AHRD V1)	57.42	42.4	38.92	50.11	47.21	74.69	59.8	44.93	56.13	58.89	1.25	0.778997	65.91	39.29	49.53	51.58	58.47	66.42	64.33	63.07	1.22	0.844156	16.78	25.88	12.13	18.26	20.87	13.89	31.16	21.97	1.2	1	8.68	16.86	5.64	10.39	19.01	26.78	22.9	2.2	0.989607
Solyc01g105710.2.1	Peptidyl-prolyl cis-trans isom	499.16	398.01	458.04	622.66	494.47	412.74	504.23	341.93	415.36	418.57	0.85	0.990049	432.81	408.03	414.7	418.51	237.69	259.05	219.77	238.84	0.57	0.02453	201.87	194.57	162.84	186.43	133.08	154.87	152.31	146.75	0.79	1	82.18	105.58	80.8	89.52	118.64	120.29	119.47	1.33	1
Solyc01g105720.2.1	Unknown Protein (AHRD V1)	375.81	376.8	361.77	285.38	349.94	500.86	565.96	618.93	442.01	531.94	1.52	0.059233	451.75	100.64	108.2	220.2	92.4	147.49	172.9	137.6	0.62	1	12.55	11.87	10.65	11.69	9.05	5.46	9.68	8.06	0.69	1	4.78	5.47	4.68	4.98	4.4	5.62	5.01	1.01	1
Solyc01g105730.1.1	Periaxin-like protein (AHRD V	0	0	0	0	0	0	0	0	0	0	1	NA	0	0	0	0	0	0	0	0	1	NA	0	0	0	0	0	0	0	0	1	NA	0	0	0	0	0	0	0	1	NA
Solyc01g105740.1.1	Unknown Protein (AHRD V1)	0	0	0	0	0	0	0	0	0	0	1	NA	0	0	0	0	0	0	0	0	1	NA	0	0	0	0	0	0	0	0	1	NA	0	0	0	0	0	0	0	1	NA
Solyc01g105750.1.1	Unknown Protein (AHRD V1)	0	0	0	0	0	0	0	0	0	0	1	NA	0	0	0	0	0	0	0	0	1	NA	0	0	0	0	0	0	0	0	1	NA	0	0	0	0	0	0	0	1	NA
Solyc01g105760.1.1	Unknown Protein (AHRD V1)	0	0	0	0	0	0	0	0	0	0	1	NA	0	0	0	0	0	0	0	0	1	NA	0	0	0	0	0	0	0	0	1	NA	0	0	0	0	0	0	0	1	NA
Solyc01g105770.2.1	Unknown Protein (AHRD V1)	0.17	0	0	0	0.04	0.19	0.26	0	0	0.11	2.65	1	1.71	0	0.14	0.62	0	0.43	0	0.14	0.23	1	0.14	0.14	0.33	0.2	0	0	0.37	0.12	0.61	1	0	0	0	0	0	0	0	1	NA
Solyc01g105780.2.1	DnaJ (Fragment) (AHRD V1 *-	16.45	14.23	20.27	20.53	17.87	11.75	12.55	12.67	12.6	12.39	0.69	0.440401	17.18	19.49	24.52	20.4	19.05	21.41	15.38	18.61	0.91	1	11.95	13.78	14.41	13.38	17.26	13.51	13.66	14.81	1.11	1	31.35	31.7	36.88	33.31	20.35	23.93	22.14	0.66	0.896992
Solyc01g105790.1.1	Unknown Protein (AHRD V1)	0	0	0	0	0	0	0	0	0	0	1	NA	0	0	0	0	0	0	0	0	1	NA	0	0	0	0	0	0	0	0	1	NA	0	0	0	0	0	0	0	1	NA
Solyc01g105800.2.1	MADS box transcription facto	2.36	1.26	0	1.67	1.32	0.79	0.37	2.17	1.69	1.25	0.95	1	1.53	1.64	1	1.39	0.33	0.61	0.56	0.5	0.36	0.654609	0.19	0.19	0	0.13	0	0	0	0	0.08	1	0.33	0.34	0	0.22	0	0	0	0.04	1
Solyc01g105810.2.1	Rab-GDP dissociation inhibito	0	0	0.17	0	0.04	0	0.12	0	0	0.03	0.71	1	0	0	0	0	0	0.08	0	0.03	2.67	1	0	0.12	0	0.04	0	0	0	0	0.25	1	0	0	0	0	0	0	0	1	NA
Solyc01g105820.2.1	Ulp1 protease family protein	41.18	60.75	62.09	60.95	56.24	64.01	54.19	72.58	56.4	61.8	1.1	0.939969	56.4	86.52	86.96	76.63	46.29	51.93	67.73	55.32	0.72	0.487928	55.46	44.44	71.16	57.02	40.58	43.64	38.63	40.95	0.72	1	127.96	95	131.94	118.3	74.35	81.92	78.13	0.66	0.958249
Solyc01g105830.2.1	Sentrin-specific protease (AH	9.24	13.78	13.93	13.68	12.66	14.93	12.29	16.6	12.72	14.13	1.12	0.912075	12.73	19.41	19.51	17.22	10.39	11.7	15.2	12.43	0.72	0.477412	12.58	9.97	15.97	12.84	9.11	9.79	8.67	9.19	0.72	1	28.83	21.69	29.74	26.75	16.68	18.53	17.61	0.66	0.946284
Solyc01g105840.2.1	Unknown Protein (AHRD V1)	0	0	0	0	0	0	0	0	0	0	1	NA	0	0	0	0	0	0	0	0	1	NA	0	0	0	0	0	0	0	0	1	NA	0	0	0	0	0	0	0	1	NA
Solyc01g105850.2.1	(E)-beta-ocimene synthase (A	0	0	0	0	0	0	0.19	0	0	0.05	4.75	1	0.22	0	0.2	0.14	0	0.12	0	0.04	0.29	1	0	0	0	0	0	0	0	0	1	1	0	0	0	0	0	0.21	0.1	10.5	1
Solyc01g105860.2.1	(R)-limonene synthase (AHRD	0	0	0	0	0	0	0	0	0	0	1	NA	0	0	0	0	0	0	0	0	1	NA	0	0	0	0	0	0	0	0	1	NA	0	0	0	0	0	0	0	1	NA
Solyc01g105870.2.1	(E)-beta-ocimene synthase (A	0	0.1	0.13	0	0.06	0	0	0	0	0	0.17	1	0.2	0	0	0.07	0	0	0	0	0.15	1	0	0	0	0	0	0	0	0	1	NA	0	0	0	0	0	0	0	1	NA
Solyc01g105880.2.1	(E)-beta-ocimene synthase (A	0	0.1	0	0	0.03	0.12	0.09	0.1	0.32	0.16	6.3	0.852967	0.1	0	0.09	0.06	0	0	0	0	0.16	1	0	0	0	0	0	0	0	0	1	NA	0	0	0	0	0	0	0	1	NA
Solyc01g105890.2.1	(E)-beta-ocimene synthase (A	1.3	1.64	6.25	0.31	2.38	0.96	0	1.29	0.39	0.66	0.28	0.678841	0.91	0	0	0.3	0	0	0	0	0.03	0.904978	0	0.09	0	0.03	0	0	0	0	0.33	1	0	0	0	0	0	0	0	1	NA
Solyc01g105900.2.1	(E)-beta-ocimene synthase (A	0.12	0.11	0.15	0	0.1	0.14	0	0.23	0.18	0.14	1.45	1	0	0	0	0	0	0	0	0	1	NA	0	0	0	0	0	0	0	0	1	NA	0	0	0	0	0	0	0	1	NA
Solyc01g105910.1.1	D-limonene synthase (AHRD V	0	0	0	0	0	0	0	0	0	0	1	NA	0	0	0	0	0	0	0	0	1	NA	0	0	0	0	0	0	0	0	1	NA	0	0	0	0	0	0	0	1	NA
Solyc01g105920.2.1	(E)-beta-ocimene synthase (A	0.11	0.2	0.13	0	0.11	0.37	0.09	0.72	0.64	0.45	4.14	0.498543	0	0	0	0	0	0	0	0	1	NA	0.09	0	0	0.03	0	0	0	0	0.33	1	0	0	0	0	0	0	0	1	NA
Solyc01g105930.1.1	D-limonene synthase (AHRD V	0	0	0	0	0	0	0	0	0	0	1	NA	0	0	0	0	0	0	0	0	1	NA	0	0	0	0	0	0	0	0	1	NA	0	0	0	0	0	0	0	1	NA
Solyc01g105940.2.1	Limonene synthase (AHRD V1	0	0.55	1.47	0.88	0.72	2.08	0	1.14	0	0.81	1.11	1	0.58	1.08	0.53	0.73	0	0	0	0	0.01	0.833891	0	0	0.62	0.21	0.41	1.42	0.45	0.76	3.68	1	0	0	0.96	0.32	0.57	0.55	0.56	1.75	1
Solyc01g105950.2.1	(R)-limonene synthase (AHRD	0	0.38	1.02	0.61	0.5	1.44	0	0.79	0	0.56	1.11	1	0.4	0.75	0.36	0.5	0	0	0	0	0.02	0.833891	0	0	0.43	0.14	0.28	0.98	0.31	0.52	3.65	1	0	0	0.66	0.22	0.39	0.38	0.39	1.75	1
Solyc01g105960.2.1	(E)-beta-ocimene synthase (A	0	0	0	0	0	0	0	0	0	0	1	NA	0	0	0	0	0	0	0	0	1	NA	0	0	0	0	0	0	0	0	1	NA	0	0	0	0	0	0	0	1	NA
Solyc01g105970.2.1	Magnesium-dependent phos	405.9	337.76	352.7	328.57	356.23	436.52	364.58	492.27	432.24	431.4	1.21	0.595088	343.64	410.79	348.11	367.51	421.44	338.34	430.31	396.7	1.08	1	288.93	300.48	286.05	291.82	420.16	379.22	308.94	369.44	1.27	1	572.66	787.32	639.11	666.36	587.69	473.09	530.39	0.8	1
Solyc01g105980.2.1	Unknown Protein (AHRD V1)	51.46	58.13	51.37	58.79	54.94	42.9	48.96	43.37	50.68	46.48	0.85	0.965954	52.77	46.45	49.59	49.6	34.09	42.53	44.59	40.4	0.81	0.931153	41.84	41.39	31.43	38.22	33.59	33	37.26	34.62	0.91	1	18.29	20.36	20.29	19.65	28.69	24	26.34	1.34	1
Solyc01g105990.2.1	Os01g0107900 protein (Fragr	23.99	26.1	24.82	22.08	24.25	27.51	28.18	28.8	28.84	28.33	1.17	0.753896	24.35	22.43	21.95	22.91	26.28	27.6	27.98	27.29	1.19	0.742767	17.07	21.49	13.32	17.29	18.51	18.69	19.35	18.85	1.09	1	29.37	25.37	25.18	26.64	19.35	18.11	18.73	0.7	0.989607
Solyc01g106000.2.1	Isochorismatase hydrolase (A	231.17	199.77	191.42	157.42	194.94	188.7	182.08	211.8	175.2	189.44	0.97	1	204.8	176.94	160.37	180.7	153.45	168.01	169.23	163.56	0.91	1	259.34	332.82	173.02	255.06	151.16	161.79	226.3	179.75	0.7	1	65.58	79.27	62.68	69.18	126.43	123.44	124.94	1.81	0.498249
Solyc01g106010.2.1	Fructose-1 6-bisphosphatase	19.08	27.45	15.36	12.61	18.62	35.28	24.35	40	26.63	31.56	1.69	0.134731	16.59	18.91	13.85	16.45	18.55	22.99	32.65	24.73	1.5	0.349573	11.09	9.73	6.55	9.12	6.91	9.46	10	8.79	0.96	1	2.03	5.57	3.29	3.63	3.92	2.5	3.21	0.88	1
Solyc01g106020.2.1	Nucleic acid binding protein (	10.4	9.44	10.47	9.52	9.96	10.2	12.07	9.66	8.78	10.18	1.02	1	8.44	11.26	12.06	10.59	12.09	10.88	8.97	10.65	1.01	1	4.45	6.65	5.47	5.52	6.63	5.64	5.65	5.97	1.08	1	6.23	9.18	11.29	8.9	6.81	7.78	7.29	0.82	1
Solyc01g106030.2.1	Pseudo response regulator (A	36.92	29.99	39.99	34.24	35.29	35.66	38.23	40.81	39.25	38.49	1.09	0.917957	38.66	42.79	36.3	39.25	34	36.04	37.26	35.77	0.91	1	37.81	37.32	39.25	38.13	34.99	40.83	35.87	37.23	0.98	1	55.93	43.23	43.12	47.43	52.45	56.43	54.44	1.15	1
Solyc01g106040.2.1	GATA transcription factor 25	5.4	4.65	2.67	3.2	3.98	4.45	3.44	5.12	3.5	4.13	1.04	1	4.37	3.93	3.53	3.94	3.62	3.97	3.97	3.85	0.98	1	1.41	1.42	0.52	1.12	0.8	0.53	1.52	0.95	0.85	1	0.49	0	0.54	0.34	0.48	0.92	0.7	2.04	1
Solyc01g106050.2.1	Dynamin-2 (AHRD V1 ***- D	24.05	25.61	21.56	23.58	23.7	25.48	22.66	24.32	22.67	23.78	1	1	28.26	27.25	27.08	27.53	16.23	20.67	21.73	19.54	0.71	0.425229	29.98	27.7	30	29.23	22.8	22.6	23.31	22.9	0.78	1	33.39	33.01	34.27	33.56	27.22	31.77	29.49	0.88	1
Solyc01g106060.2.1	Glutamine amidotransferase	56.2	62.82	65.66	60.29	61.24	36.21	52.08	72.89	40.54	50.43	0.82	0.858496	31.82	37.05	33.25	34.04	49.9	37.67	44.38	43.98	1.29	0.587914	19.08	29.87	27.22	25.39	43.87	44.45	38.18	42.17	1.66	1	63.3	113.18	128.58	101.69	128.24	134.01	131.12	1.29	1
Solyc01g106070.1.1	Unknown Protein (AHRD V1);	0	0	0	0	0	0	0	0	0	0	1	NA	0	0	0	0	0	0	0	0	1	NA	0	0	0	0	0	0	0	0	1	NA	0	0	0	0	0	0	0	1	NA
Solyc01g106080.2.1	Methylmalonate-semialdehy	34.33	38.52	33.71	37.99	36.14	32.3	45.79	29.31	41.02	37.11	1.03	1	40.04	45.2	38.32	41.19	46.69	46.67	43.65	45.67	1.11	0.94301	81.43	87.13	78.26	82.27	77.09	76.02	79.62	77.58	0.94	1	35.74	59.76	52.61	49.37	61.96	53.54	57.75	1.17	1
Solyc01g106090.2.1	Photosystem II oxygen evolvi	19.7	20.39	15.67	15.55	17.83	27.5	26.04	28.64	19.73	25.48	1.43	0.353546	17.9	17.12	11.8	15.61	20.65	19.93	20.16	20.25	1.3	0.615146	28.5	27.07	27.06	27.54	24.53	29.51	30.71	28.25	1.03	1	8.79	16.14	10.92	11.95	16.57	12.85	14.71	1.23	1
Solyc01g106100.2.1	DUF593-containing protein 2	9.6	8.73	5.66	3.84	6.96	8.35	6.1	14.4	11.24	10.02	1.44	0.678531	6.41	7.62	5.64	6.56	4.92	7.24	11.98	8.05	1.23	1	5.57	5.27	2.69	4.51	5.76	2.69	7	5.15	1.14	1	2.08	3.94	2.25	2.76	2.87	3.66	3.27	1.18	1
Solyc01g106110.2.1	Unknown Protein (AHRD V1)	51.74	65.62	70.2	29.28	54.21	71.89	71.49	84.44	62.22	72.51	1.34	0.657728	50.54	64.09	54.63	56.42	53.83	59.61	63.03	58.82	1.04	1	47.03	55.87	25.68	42.86	46.74	42.16	43.36	44.09	1.03	1	51.5	52.61	41.77	48.63	37.89	35.16	36.52	0.75	1
Solyc01g106120.2.1	Unknown Protein (AHRD V1)	22.69	28.65	28.64	12.69	23.17	32.85	30.83	40.76	26.14	32.64	1.41	0.535676	23.07	27.07	23.18	24.44	22.33	25.41	26.96	24.9	1.02	1	20.47	23.22	13.1	18.93	19.76	18	19.23	19	1	1	21.01	23	20.29	21.43	15.94	14.81	15.38	0.72	1
Solyc01g106130.1.1	F-box protein family-like (AH	0	0	0	0	0	0	0	0	0	0	1	NA	0	0	0	0	0	0	0	0	1	NA	0	0	0	0	0	0	0	0	1	NA	0	0	0	0	0	0	0	1	NA
Solyc01g106140.1.1	F-box protein family-like (AH	0	0	0	0	0	0	0	0	0	0	1	NA	0	0	0	0	0	0	0	0	1	NA	0	0	0	0	0	0	0	0	1	NA	0	0	0	0	0	0	0	1	NA
Solyc01g106150.1.1	Interferon-related developme	0.34	0.15	0.2	0.48	0.29	1.14	0.67	0.94	0.61	0.84	2.87	0.537092	0.16	0.74	0.43	0.44	0.71	0.53	0.54	0.59	1.34	1	0.41	0.56	0.17	0.38	0	0.52	1.24	0.59	1.54	1	0.73	0.37	0.79	0.63	0.94	0.75	0.84	1.34	1
Solyc01g106160.1.1	Receptor protein kinase-like p	0	0	0	0	0	0	0	0	0	0	1	NA	0	0	0	0	0	0	0	0	1	NA	0	0	0	0	0	0	0	0	1	NA	0	0	0	0	0	0	0	1	NA
Solyc01g106170.2.1	MADS-box transcription facto	0	0.39	0	0	0.1	0	0	0	0	0	0.1	1	0	0.38	0.37	0.25	0	0	0	0	0.04	1	0.36	0.36	0	0.24	0	0	0	0	0.04	1	0	0	0.68	0.23	0.41	0	0.2	0.9	1
Solyc01g106180.1.1	CK25 (AHRD V1 ***- Q1W1G	0.35	0.32	0	1.51	0.54	0	0.28	0	0.25	0.13	0.24	0.980526	0.33	0.62	0.6	0.52	0	0.18	0	0.06	0.12	0.913634	0	0	0	0	0.23	0	0	0.08	7.67	1	0	0	0	0	0	0	0	1	NA
Solyc01g106190.2.1	Os11g0167200 protein (Fragr	3.39	4.86	6.89	5.07	5.05	5.24	7.44	5.37	2.98	5.26	1.04	1	4.56	5.57	5.41	5.18	6.28	8	4.74	6.34	1.22	0.88712	4.87	5.99	3.55	4.8	2.85	5	3.43	3.76	0.78	1	9.38	5.34	5.51	6.74	3.8	6.78	5.29	0.78	1

Solyc01g106200.1.1	Oligopeptide transporter 4 (A	0	0	0	0	0	0	0	0	0	0	1	NA	0	0	0	0	0	0	0	0	1	NA	0	0	0	0	0	0	0	0	1	NA	0	0	0	0	0	0	0	1	NA
Solyc01g106210.2.1	Chaperone DnaK (AHRD V1 *	51.27	46.66	58.2	57.06	53.3	33.55	39.71	38.51	44.65	39.1	0.73	0.537431	58.58	55.08	66.42	60.03	61.12	53.74	55.27	56.71	0.94	1	63.54	102.23	98.7	88.16	91.19	90.47	93.4	91.69	1.04	1	87.88	114.59	120.34	107.6	151.33	156.73	154.03	1.43	0.910144
Solyc01g106220.2.1	DNA polymerase V (AHRD V1	29.53	33.37	36.58	38.71	34.55	25.44	26.65	27.83	27.56	26.87	0.78	0.688515	31.84	34.7	47.04	37.86	43.23	34.29	39.29	38.94	1.03	1	19.56	25.61	21.72	22.3	25.22	19.58	23.86	22.89	1.03	1	62.98	39.53	56.68	53.06	42.19	59.37	50.78	0.96	1
Solyc01g106230.2.1	B3 domain-containing protei	3.92	5.75	3	1.66	3.58	1.74	2.78	2.7	2.11	2.33	0.65	0.620872	2.37	3.91	2.31	2.86	2.17	4.56	5.69	4.14	1.45	0.830489	2.06	1.6	1.17	1.61	3.6	1.63	1	2.08	1.29	1	3.06	5.69	8.73	5.83	2.87	3.6	3.24	0.56	0.993057
Solyc01g106240.2.1	DNA polymerase V (AHRD V1	29.8	33.46	36.96	39.16	34.84	25.44	26.73	27.93	27.64	26.93	0.77	0.670345	32.23	35.11	47.7	38.35	43.74	34.62	39.45	39.27	1.02	1	19.85	25.83	21.83	22.5	25.6	19.62	24.02	23.08	1.03	1	63.28	39.53	56.85	53.22	42.77	60.25	51.51	0.97	1
Solyc01g106250.2.1	B3 domain-containing protei	3.92	5.75	2.77	1.38	3.46	1.74	2.63	2.52	2.11	2.25	0.65	0.636919	2.19	3.74	2.15	2.69	2.17	4.46	5.23	3.95	1.47	0.77615	1.9	1.6	1.17	1.56	3.47	1.63	0.86	1.99	1.28	1	3.06	5.69	8.43	5.73	2.51	3.6	3.05	0.53	0.942833
Solyc01g106260.2.1	Chaperone DnaK (AHRD V1 *	26.45	24.41	33.99	46.03	32.72	15.52	25.69	20.07	26.19	21.87	0.67	0.653306	27.71	26.75	35.96	30.14	28.44	25.91	26.5	26.95	0.89	1	27.8	35.41	45.96	36.39	36.04	30.25	38.4	34.9	0.96	1	38.58	49.91	51.1	46.53	62.46	62.41	62.44	1.34	1
Solyc01g106270.1.1	DNA polymerase V family (AH	0	0	0	0	0	0	0	0	0	0	1	NA	0	0	0.33	0.11	0	0	0	0	0.09	1	0	0	0	0	0	0	0	0	1	NA	0	0	0	0	0	0	0	1	NA
Solyc01g106280.2.1	DNA-binding bromodomain-c	140.04	129.5	192.45	190.35	163.08	124.17	114.79	98.28	106.35	110.9	0.68	0.26622	157.84	193.06	237.85	196.25	117.84	135.33	103.69	118.95	0.61	0.063434	80.88	130.07	136.5	115.82	116.36	90.47	92	99.61	0.86	1	129.73	253.16	260.43	214.44	177.47	207.96	192.72	0.9	1
Solyc01g106290.2.1	Unknown Protein (AHRD V1)	12.08	11.58	6.58	13.14	10.85	19.62	8.07	23.92	13.34	16.24	1.5	0.727058	18.15	21.81	21.97	20.64	13.53	22.13	14.6	16.75	0.81	1	4.5	11.36	15.68	10.51	17.7	9.86	12.86	13.47	1.28	1	6.61	33.74	18.57	19.64	17.01	22	19.51	0.99	1
Solyc01g106300.2.1	Unknown Protein (AHRD V1)	0	0	0	0	0	0	0	0	0	0	1	NA	0	0	0	0	0	0	0	0	1	NA	0	0	0	0	0	0	0	0	1	NA	0	0	0	0	0	0	0	1	NA
Solyc01g106310.2.1	Unknown Protein (AHRD V1)	5.42	10.46	2.13	1.28	4.82	6.03	2.14	7.48	6.49	5.54	1.15	1	8.41	3.14	4.58	5.38	3.76	4.21	7.81	5.26	0.98	1	10.22	10.31	7.18	9.24	5.94	7.53	7.9	7.12	0.77	1	3.85	13.13	27.79	14.92	7.45	15.85	11.65	0.78	1
Solyc01g106320.2.1	Octicosapeptide/Phox/Bem1p	138.09	106.14	180.08	222.33	161.66	118.46	140.43	83.74	128.38	117.75	0.73	0.835351	154.9	179.01	225.93	186.61	183.49	150.87	114.08	149.48	0.8	0.894392	108	146.47	148.86	134.44	121.42	111.64	101.89	111.65	0.83	1	307.97	197.48	220.14	241.86	163.69	197.41	180.55	0.75	1
Solyc01g106330.2.1	Glutamyl-tRNA(Gln) amidotra	68.1	76.36	38.48	61.21	61.04	95.6	107.03	83.39	70.93	89.24	1.46	0.175074	69.3	43.36	41.5	51.39	17.3	34.54	33.17	28.34	0.55	0.42138	4.39	5.47	3.6	4.49	1.26	1.37	1.01	1.21	0.27	0.014212	1.06	0.7	1.64	1.13	1.95	4.11	3.03	2.67	0.361958
Solyc01g106340.2.1	Glutamyl-tRNA(Gln) amidotra	13.28	16.34	8.15	6.87	11.16	14.28	11.34	16.28	12.64	13.63	1.22	0.908506	11.45	8.92	8.94	9.77	3.15	4.03	4.41	3.86	0.4	0.001417	1.05	0.7	0.43	0.73	0.28	0.25	0.31	0.28	0.39	0.971257	0.31	0.08	0.5	0.3	0.3	0.09	0.2	0.66	1
Solyc01g106350.2.1	Glutamyl-tRNA(Gln) amidotra	5.05	3.32	3.5	4.35	4.05	3.05	2.6	3.84	3.63	3.28	0.81	0.9707	2.14	1.81	2.97	2.31	1.22	1.71	1.64	1.52	0.66	0.819112	0.27	0.27	0.22	0.25	0.29	0.17	0.16	0.21	0.82	1	0.16	0.08	0	0.08	0.3	0.1	0.2	2.5	1
Solyc01g106360.2.1	Glutamyl-tRNA(Gln) amidotra	61.31	64.88	63.9	73.24	65.83	56.35	67.22	60.16	67.35	62.77	0.95	1	69.63	72.41	76.95	73	88.06	80.71	76.7	81.82	1.12	0.905397	56.1	63.38	62.04	60.51	49.67	52.71	55.81	52.73	0.87	1	39.27	45.24	42.31	42.27	58.21	62.02	60.12	1.42	0.943939
Solyc01g106370.2.1	Os06g0207500 protein (Frag	11.57	12.95	16.19	10.52	12.81	11.58	9.92	10.34	12.11	10.99	0.86	0.9965	12.46	12.07	14.52	13.02	10.66	10.62	14.29	11.86	0.91	1	12.16	11.72	11.49	11.79	10.43	8.27	9.28	9.33	0.79	1	11.99	9.85	9.41	10.42	9.99	11.02	10.5	1.01	1
Solyc01g106380.2.1	Genomic DNA chromosome 5	20.62	16.68	17.25	10.6	16.29	24.17	29.59	24.81	30.95	27.38	1.68	0.109732	13.95	8.8	9.5	10.75	20.28	21.15	18.26	19.9	1.85	0.07543	13.63	14.05	9.3	12.33	15.76	13.35	18.57	15.89	1.29	1	3.2	5.44	2.31	3.65	7.55	8.22	7.88	2.16	0.95453
Solyc01g106390.2.1	Glutamyl-tRNA reductase (AH	4.65	4.25	2.81	2.87	3.65	3.71	3.01	3.73	3.51	3.49	0.96	1	4.33	3.94	5.04	4.44	4.3	3.83	2.91	3.68	0.83	1	3.08	4.57	8.53	5.39	6.43	6.6	6.95	6.66	1.23	1	25.78	16.55	16.88	19.74	15.83	12.66	14.25	0.72	1
Solyc01g106400.2.1	Peptide methionine sulfoxide	3.45	4.44	5.88	4.88	4.66	16.63	10.3	11.99	14.87	13.45	2.88	0.003181	4.64	8.67	8.75	7.35	18.89	13.11	8.74	13.58	1.85	0.407034	0.93	2.5	1.14	1.52	0.5	0	0.84	0.45	0.29	1	0	0.28	1.18	0.49	0	0	0	0.02	1
Solyc01g106410.1.1	Cc-nbs-lrr, resistance protein	0.17	0.2	0.13	0.48	0.24	0.25	0.18	0.26	0.04	0.18	0.74	1	0.05	0.2	0.19	0.15	0.08	0.21	0.09	0.13	0.86	1	0.14	0	0	0.05	0.04	0.04	0.08	0.05	1.14	1	0.24	0.04	0.17	0.15	0.16	0.4	0.28	1.87	1
Solyc01g106420.2.1	Major facilitator superfamily	2.56	6.62	2.3	1.9	3.35	3.12	2.98	3.25	2.45	2.95	0.88	1	4.54	5.72	4.63	4.96	3.13	3.41	4.6	3.71	0.75	0.850712	5.42	4.27	2.42	4.04	1.92	1.94	2.22	2.03	0.5	1	1.56	2.75	2.06	2.12	1.9	1.5	1.7	0.8	1
Solyc01g106430.2.1	Inorganic pyrophosphatase fa	158.92	146.28	160.48	147.51	153.3	215.46	223.54	189.06	249.99	219.51	1.43	0.102493	121.46	133.22	140.16	131.61	179.72	145.79	158.03	161.18	1.22	0.643152	35.72	68.91	26.78	43.8	47.06	39	61.79	49.28	1.13	1	5.48	15.31	10.46	10.42	17.43	23.06	20.24	1.94	0.718618
Solyc01g106440.2.1	Ankyrin repeat-containing pr	15.07	15.57	13.47	15.47	14.89	14.72	15.35	15.53	14.65	15.06	1.01	1	18.19	15.74	14.98	16.3	13.57	13.65	14.32	13.85	0.85	1	12.17	11.48	13.27	12.31	10.93	12.71	11.33	11.66	0.95	1	11.23	12.53	10.65	11.47	10.12	12.78	11.45	1	1
Solyc01g106450.2.1	L-galactose dehydrogenase (A	63.92	68.01	59.7	67.52	64.79	83.58	71.98	91.17	70.9	79.41	1.23	0.601223	52.98	57.35	49.32	53.22	55.7	58.45	63.28	59.14	1.11	0.958764	38.07	47.75	39.45	41.76	36.34	40.84	44.04	40.41	0.97	1	31.46	38.24	23.31	31	39.57	44.37	41.97	1.35	1
Solyc01g106460.2.1	Transcription factor (AHRD V	0	0	0	0	0	0	0	0	0	0	1	NA	0	0	0	0	0	0.14	0	0.05	4.67	1	0	0	0	0	0	0	0	0	1	NA	0	0	0	0	0	0	0	1	NA
Solyc01g106470.2.1	Genomic DNA chromosome 5	39.86	43.08	35.67	32.62	37.81	43.8	43.04	34.4	35.96	39.3	1.04	1	50	33.32	33.28	38.87	34.42	32.26	36.48	34.39	0.88	1	106.6	103.41	108.38	106.13	68.52	76.51	135.99	93.67	0.88	1	13.24	29.73	26.77	23.25	43.76	43.25	43.5	1.87	0.522987
Solyc01g106480.2.1	Malate dehydrogenase (AHRD	60.26	71.51	66.74	81.85	70.09	206.07	157.85	153	185.74	175.66	2.51	8.75E-08	55.02	68.08	62.1	61.73	142.89	112.3	98.62	117.94	1.91	0.002932	14.14	18.02	17.38	16.51	14.89	12.01	20.95	15.95	0.97	1	6.81	10.04	4.25	7.03	11.3	11.96	11.63	1.65	1
Solyc01g106490.1.1	Unknown Protein (AHRD V1)	0	0	0	0	0	0	0	0	0	0	1	NA	0	0	0	0	0	0	0	0	1	NA	0	0	0	0	0	0	0	0	1	NA	0	0.8	0	0.27	0	0	0	0.04	1
Solyc01g106500.2.1	LRR receptor-like serine/thre	1.98	2.25	2.45	4.08	2.69	0.6	1.14	0.75	0.45	0.74	0.27	0.006783	1.76	4.39	3.12	3.09	3.63	3.22	3.12	3.32	1.08	1	4.15	2.94	8.5	5.2	4.44	5.26	2.56	4.09	0.79	1	24.6	21.4	29.1	25.03	13.37	13.91	13.64	0.54	0.572811
Solyc01g106510.2.1	F-box protein family-like (AH	0	0	0	0	0	0	0	0	0.12	0.03	3	1	0	0.15	0	0.05	0	0	0	0	0.2	1	0	0	0	0	0	0.13	0	0.04	4.33	NA	0	0	0	0	0	0	0	1	NA
Solyc01g106520.1.1	Unknown Protein (AHRD V1);	0	0	0	0	0	0	0	0	0	0	1	NA	0	0	0	0	0	0	0	0	1	NA	0	0	0	0	0	0	0	0	1	NA	0	0	0	0	0	0	0	1	NA
Solyc01g106530.1.1	Unknown Protein (AHRD V1);	0	0	0	0	0	0	0	0	0	0	1	NA	0	0	0	0	0	0	0	0	1	NA	0	0	0	0	0	0	0	0	1	NA	0	0	0	0	0	0	0	1	NA
Solyc01g106540.2.1	Unknown Protein (AHRD V1)	0.59	0.35	0.23	0	0.29	0.11	0.23	0.09	0.14	0.14	0.49	0.906298	0.18	0	0	0.06	0.27	0	0	0.09	1.5	1	0	0	0	0	0	0	0	0	1	NA	0	0	0	0	0	0	0	1	NA
Solyc01g106550.1.1	Unknown Protein (AHRD V1)	0	0	0	0	0	0	0	0	0	0	1	NA	0	0	0	0	0	0	0	0	1	NA	0	0	0	0	0	0	0	0	1	NA	0	0	0	0	0	0	0	1	NA
Solyc01g106560.2.1	AT4g33690/T16L1_180 (Frag	21.2	16.58	16.93	18.48	18.3	17.38	11.16	18.71	12.46	14.93	0.82	0.838602	15.14	19.92	15.56	16.87	17.08	20.29	16.33	17.9	1.06	1	9.69	15.36	10.63	11.89	12.1	10.71	13.42	12.08	1.02	1	37.46	30.8	38.54	35.6	20.98	28.55	24.77	0.7	1
Solyc01g106570.2.1	Magnesium and cobalt efflux	11.02	15.72	16.21	11.74	13.67	20.14	15.37	14.16	13.22	15.72	1.15	0.924458	15.45	16.8	17.47	16.57	14.45	17.43	15.9	15.93	0.96	1	9.64	13.9	7.04	10.19	7.44	7.55	11.77	8.92	0.88	1	5.58	8.22	12.77	8.86	6.91	10.46	8.69	0.98	1
Solyc01g106580.2.1	Unknown Protein (AHRD V1)	87.56	92.65	93.78	129.84	100.96	70.87	111.01	69.75	96.32	86.99	0.86	1	92.14	75.06	70.72	79.31	44.08	58.08	50.89	51.02	0.64	0.187243	31.29	64.61	21.23	39.04	25.98	17.58	30.87	24.81	0.64	0.929608	2.01	1.47	2.17	1.88	4.62	8.49	6.55	3.48	0.166883
Solyc01g106590.2.1	Leaf senescence protein-like (	11.16	7.63	8.01	9.15	8.99	8.45	10.08	5.65	8.47	8.16	0.91	1	7.22	4.64	8.19	6.68	6.17	5.44	7.5	6.37	0.95	1	5.23	6.59	4.01	5.28	3.61	3.92	5.42	4.32	0.82	1	6.67	3.41	4.48	4.85	4	5.25	4.62	0.95	1
Solyc01g106600.2.1	Pathogenesis-related protein	0	0	0.48	0	0.12	0	0	0	0	0	0.08	1	0	0	0	0	0	0	0	0	1	NA	0	0	0	0	0	0	0	0	1	NA	0	0	0	0	0	0	0	1	NA
Solyc01g106610.2.1	Pathogenesis-related protein-	0	0	0	0	0	0	0	0	0	0	1	NA	0	0	0	0	0	0	0	0	1	NA	0	0	0	0	0	0	0	0	1	NA	0	0	0	0	0	0	0	1	NA
Solyc01g106620.2.1	Pathogenesis-related protein	0	0	0	0	0	0	0	1.35	0	0.34	33.75	0.993291	0	0	0	0	0	0	0	0	1	NA	4.74	198.01	1.46	68.07	0.24	0.56	0.8	0.53	0.01	1	1.04	1.86	0	0.97	2.35	0.64	1.5	1.55	1
Solyc01g106630.2.1	Unknown Protein (AHRD V1)	0	0	0	0	0	0	0	0	0	0	1	NA	0	0	0	0	0	0	0	0	1	NA	0	0	0	0	0	0	0	0	1	NA	0	0	0	0	0	0	0	1	NA
Solyc01g106640.2.1	Pathogenesis-related protein	0	0	0	0	0	0	0	0	0	0	1	NA	0	0	0	0	0	0	0	0	1	NA	0	0	0	0	0	0	0	0	1	NA	0	0	0	0	0	0	0	1	NA
Solyc01g106650.2.1	Xyloglucan endotransglucosyl	0.9	0.8	0.26	0.32	0.57	0.5	0.35	0.62	1.13	0.65	1.14	1	0	0	0.57	0.19	0.78	0.23	0.18	0.4	2.09	1	0.36	0	0	0.12	0.44	0.34	0.33	0.37	3.08	1	0	0.33	0	0.11	0	0	0	0.09	1
Solyc01g106660.1.1	Ulp1 protease family C-termi	0	0	0	0.09	0.02	0.15	0.32	0.55	0.43	0.36	16.11	0.035772	0	0	0	0	0.92	0.72	0.73	0.79	79	2.64E-05	0.11	0	0	0.04	2.97	2.22	2.04	2.41	65.73	0.000894	0	0.05	0.1	0.05	2.99	3.62	3.31	66.1	2.5E-08
Solyc01g106670.1.1	Mutator-like transposase (AH	0	0	0.15	0	0.04	0.28	0	0.59	0.28	0.29	7.67	0.586335	0.36	0.11	0	0.16	0.18	0.2	0.1	0.16	1.02	1	0	0.21	0	0.07	0.34	0	0.09	0.14	2.05	1	0	0.09	0.2	0.1	0.35	0.22	0.28	2.95	1
Solyc01g106680.2.1	Unknown Protein (AHRD V1)	43.73	42.61	36.77	40.88	41	34.36	33.83	45.98	42.49	39.16	0.96	1	40.68	46.31	51.37	46.12	50.9	58.49	51.55	53.65	1.16	0.790845	33.3	47.72	32.68	37.9	53.16	42.86	52.29	49.44	1.3	1	74.14	103.13	88.31	88.53	63.04	63.24	63.14	0.71	0.896874
Solyc01g106690.2.1	Unknown Protein (AHRD V1);	11.62	15.18	11.22	22.63	15.16	9.73	9.06	7.77	8.35	8.73	0.58	0.303909	9.41	13.65	9.31	10.79	11.31	14.15	8.54	11.33	1.05	1	31.47	18.08	27.72	25.76	15.37	19.91	22.77	19.35	0.75	1	83.92	30.92	34.59	49.81	15.06	20.52	17.79	0.36	0.969193
Solyc01g106700.2.1	MADS-box transcription facto	0	0.27	0.36	0	0.16	0.34	0.24	1.13	0.44	0.54	3.41	0.916928	0.86	0.54	0	0.47	0.21	0.16	0.24	0.2	0.44	1	20.17	7.03	16.52	14.57	8.1	12.38	13.48	11.32	0.78	1	0.44	0.45	0.47	0.45	0.56	0.27	0.42	0.92	1
Solyc01g106710.1.1	MADS box transcription facto	0	0	0	0	0	0	0	0	0	0	1	NA	0	0	0	0	0	0	0	0	1	NA	0	0	0	0	0	0	0	0	1	NA	0	0	0	0	0	0	0	1	NA
Solyc01g106720.1.1	MADS-box transcription facto	0	0	0	0	0	0	0	0	0	0	1	NA	0	0	0	0	0	0	0	0	1	NA	0	0	0	0	0	0	0	0	1	NA	0	0	0	0	0	0	0	1	NA
Solyc01g106730.1.1	MADS box transcription facto	0	0	0	0	0	0	0	0	0	0	1	NA	0	0	0	0	0	0	0	0	1	NA	0	0	0	0	0	0	0	0	1	NA	0	0	0	0	0	0	0	1	NA
Solyc01g106740.2.1	Aspartyl-tRNA synthetase (AH	18.71	19.98	13.71	15.32	16.93	17.18	23.24	21.55	18.45	20.11	1.19	0.756218	16.58	18.74	16.72	17.35	18.3	23.91	22.05	21.42	1.23	0.621989	20.05	23.67	20.13	21.28	24.45	20.75	27.41	24.2	1.14	1	28.14	23.53	25.74	25.8	29.65	26.76	28.2	1.09	1
Solyc01g106750.1.1	Unknown Protein (AHRD V1)	0.08	0.22	0	0.24	0.14	0	0.2	0	0.18	0.1	0.7	1	0	0	0.28	0.09	0.06	0.04	0	0.03	0.36	1	0	0	0	0	0	0	0	0	1	NA	0	0	0	0	0	0	0	1	NA
Solyc01g106760.2.1	Unknown Protein (AHRD V1)	0	0	0	0	0	0	0	0	0	0	1	NA	0	0	0	0	0	0	0	0	1	NA	0	0	0	0	0	0	0	0	1	NA	0	0	0	0	0	0	0	1	NA
Solyc01g106770.2.1	Serine/threonine-protein kina	9.77	11.1	11.97	10.11	10.74	7.14	6.83	7.85	9.69	7.88	0.73	0.492591	10.34	11.15	14.77	12.09	13.52	11.71	13.26	12.83	1.06	1	10.27	10.43	11.14	10.61	9.32	9.29	9.25	9.29	0.88	1	14.08	11.24	14.82	13.38	13.7	14.3	14	1.05	1
Solyc01g106780.2.1	Pyruvate kinase (AHRD V1 **	50.05	47.3	27.02	18.47	35.71	34.88	32.01	47.49	50.82	41.3	1.16	1	42.08	29.77	22.64	31.5	18.59	22.77	27.72	23.03	0.73	0.838613	8.98	12.13	7.39	9.5	11.06	10.68	12.69	11.48	1.21	1	1.63	9.48	6.16	5.76	10.07	6.23	8.15	1.42	1
Solyc01g106790.2.1	Tubulin alpha-6 chain (AHRD	21.59	28.41	18.65	8.6	19.31	43.93	22.56	53.95	36.67	39.28	2.03	0.165323	21.49	19.29	15.66	18.81	21.51	34.79	28.19	28.16	1.5	0.199984	12.28	15.61	5.73	11.21	16.18	14.52	19.72	16.81	1.5	1	1.73	7.07	5.14	4.65	7.24	11.73	9.48	2.04	0.910144
Solyc01g106800.2.1	Amino acid permease 6 (AHR	3.82	4.66	4.18	2.2	3.71	3.15	2.68	3.77	2.24	2.96	0.8	0.879877	5.79	3.08	4.3	4.39	3.04	3.23	4.01	3.43	0.78	0.980282	5.95	3.58	5.06	4.86	2.33	3.33	1.75	2.47	0.51	1	6.64	4.42	4.14	5.07	1.56	1.99	1.77	0.35	0.378811
Solyc01g106810.2.1	Unknown Protein (AHRD V1)	10.17	9.06	6.2	7.89	8.33	5.84	7.51	8.45	10.37	8.04	0.97	1	9.46	10.27	15.25	11.66	10.02	10.88	10.58	10.49	0.9	1	4.77	5.62	4.89	5.09	6.47	4.98	5.98	5.81	1.14	1	3.27	8.11	2.52	4.63	5.71	5.18	5.45	1.18	1
Solyc01g106820.2.1	Peptidase M50 family (AHRD	1.12	1.5	3.71	2.06	2.1	2.37	2.04	2.06	1.77	2.06	0.98	1	3.13	6.24	6.16	5.18	15.17	11.62	12.61	13.13	2.54	0.000169	14.5	28.52	32.74	25.25	20.49	21.68	25.43	22.53	0.89	1	1.28	5.22	7.94	4.81	7.39	4.03	5.71	1.19	1
Solyc01g106830.2.1	Phytosulfokines 4 (AHRD V1 *	0	0	0	0	0	0	0	0	0.56	0.14	14	1	0	0	0	0	0	0	0	0	1	NA	0	0	0	0	0	0	0	0	1	NA	0	0	1.21	0.4	0	0	0	0.02	1
Solyc01g106840.1.1	Protein kinase (AHRD V1 ***-	0	1.29	0	0	0.32	0.81	1.72	4	0.52	1.76	5.47	0.705151	0.67	0	0	0.22	1.01	1.13	2.28	1.47	6.6	0.803356	1.17	0	1.44	0.87	0.48	1.65	1.06	1.06	1.22	1	0	1.58	2.23	1.27	0	0	0	0.01	1
Solyc01g106850.1.1	Unknown Protein (AHRD V1)	0	0	0	0	0	0	0	0	0	0	1	NA	0	0	0	0	0	0	0	0	1	NA	0	0	0	0	0	0	0	0	1	NA	0	0	0	0	0	0	0	1	NA
Solyc01g106860.2.1	1-phosphatidylinositol 3-kina	582.1	607.86	479.81	369.97	509.94	531.44	431.37	645.1	629.08	559.25	1.1	0.982067	546.51	487.11	509.35	514.32	503.86	523.21	586.76	537.94	1.05	1	387.51	507.22	500.75	465.16	422.68	422.95	471.69	439.11	0.94	1	301.26	347.81	391.46	346.84	377.26	355.9	366.58	1.06	1
Solyc01g106870.2.1	Receptor kinase 3 (AHRD V1 *	62.09	77.46	46.44	57.24	60.81	77.65	74.4	87.67	63.28	75.75	1.25	0.703053	68.58	67.95	53.85	63.46	48.45	63.44	87.57	66.49	1.05	1	48.37	62.34	58.88	56.53	42.97	49.58	62.21	51.59	0.91	1	40.99	54.36	34.1	43.15	47.7	46.18	46.94	1.09	1
Solyc01g106880.2.1	Ribosomal RNA small subunit	9.48	9.13	9.13	10.4	9.54	7.31	5.5	11.92	10.28	8.75	0.92	1	6.3	7.9	9.15	7.78	4.96	5.01	5.63	5.2	0.67	0.627841	5	3.47	4.22	4.23	4.07	4.4	2.54	3.67	0.87	1	5.78	10.82	10.11	8.9	4.78	3.05	3.92	0.44	0.433553
Solyc01g106890.2.1	Unknown Protein (AHRD V1)	45.29	40.31	31.37	55.71	43.17	28.46	52.08	56.13	31.1	41.94	0.97	1	42.13	49.64	29.11	40.29	41.66	46.39	31.73	39.93	0.99	1	101.03	70.61	43	71.55	59.51	80.6	98.3	79.47	1.11	1	140	223.84	221.06	194.97	55.88	59.57	57.73	0.3	0.006001
Solyc01g106900.2.1	Magnesium transporter MRS2	41.91	42.59	59.11	53.67	49.32	38.8	32.88	32.58	36.92	35.3	0.72	0.443469	44.52	55.39	65.44	55.12	80.6	60.79	55.17	65.52	1.19	0.820955	38.56	49.02	36.87	41.48	45.6	44.89	39.98	43.49	1.05	1	107.52	72.4	68.72	82.88	80.12	86.47	83.3	1.01	1
Solyc01g106910.2.1	Unknown Protein (AHRD V1)	0.35	1.56	2.07	0	0.99	0.78	0.83	0	0.76	0.59	0.6	1	1.31	1.22	0.89	1.14	0.24	0.55	1.1	0.63	0.55	1	0	0	0	0	0	0	0	0	1	NA	0.5	0	0	0.17	0.64	1.23	0.94	5.61	1
Solyc01g106920.2.1	Nucleobase ascorbate transp	0.37	0.11	0	0.18	0.16	0	0.3	0	0	0.07	0.45	1	0.35	0	0	0.12	0.09	0.13	0	0.07	0.63	1	0	0	0	0	0.08	0	0.09	0.06	5.67	1	0	0	0	0	0	0	0	1	NA
Solyc01g106930.2.1	Unknown Protein (AHRD V1)	0	0	0	0	0	0	0	0	0	0	1	NA	0	0	0	0	0	0	0	0	1	NA	0	0	0	0	0	0	0	0	1	NA	0	0	0	0	0	0	0	1	NA
Solyc01g106940.2.1	Single myb histone 4 (Fragme	23.01	21.85	16.81	19.22	20.22	22.41	15.4	22.57	23.29	20.92	1.03	1	18.35	22.29	19.62	20.09	26.91	24.51	19.98	23.8	1.18	0.832635	18.77	23.75	15.66	19.39	15.84	11.79	18.37	15.33	0.79	1	26.8	21.65	23.93	24.13	16.85	20.18	18.52	0.77	1
Solyc01g106950.2.1	Os03g0431932 protein (Frag	11.21	12.95	16.28	13.27	13.43	11.38	13.45	17.44	12.69	13.74	1.02	1	12.23	17.99	21.58	17.27	13.16	15.57	15.08	14.6	0.85	0.985299	13.76	13.62	22.9	16.76	21.88	14.79	13.2	16.62	0.99	1	52.26	77.74	101.61	77.2	56.34	70.7	63.52	0.82	1
Solyc01g106960.2.1	Unknown Protein (AHRD V1)	1.29	3.45	6.1	0	2.71	2.88	4.08	2.38	2.79	3.03	1.12	1	1.2	3.37	1.09	1.89	1.79	5.36	2.03	3.06	1.62	1	1.05	1.05	1.28	1.13	0	0.98	1.89	0.96	0.85	1	1.84	0.94	5.97	2.92	4.74	6.81	5.78	1.98	1
Solyc01g106970.1.1	F-box/kelch-repeat protein At	0	0	0	0	0	0	0	0	0	0	1	NA	0	0	0	0	0	0.05	0	0.02	1.67	1	0	0	0	0	0	0	0	0	1	NA	0	0	0	0	0	0	0	1	NA
Solyc01g106980.1.1	Endo-1 4-beta-xylanase (AHR	0	0	0	0	0	0	0	0	0	0	1	NA	0	0	0	0	0	0	0	0	1	NA	0	0	0	0	0	0	0	0	1	NA	0	0	0	0	0	0	0	1	NA
Solyc01g106990.1.1	Endo-1 4-beta-xylanase (AHR	0	0	0.13	0	0.03	0	0	0	0	0	0.31	1	0	0	0	0	0	0	0	0	1	NA	0	0	0	0	0	0	0	0	1	NA	0	0	0	0	0	0	0	1	NA
Solyc01g107000.1.1	Endo-1 4-beta-xylanase (AHR	0	0	0	0	0	0	0	0	0	0	1	NA	0	0	0	0	0	0	0	0	1	NA	0	0	0	0	0	0	0	0	1	NA	0	0	0	0	0	0	0	1	NA
Solyc01g107010.2.1	DNA polymerase IV (AHRD V1	3.93	5.45	5.08	4.88	4.83	4.79	4.61	5.63	4.61	4.91	1.02	1	5.97	6.74	8.9	7.2	5.64	6.1	7.3	6.35	0.88	1	17.56	14.75	17.3	16.54	15.35	14.66	12.28	14.1	0.85	1	24.78	27.82	30.33	27.64	23.14	24.77	23.95	0.87	1
Solyc01g107020.2.1	Predicted DNA damage induc	16.79	13.17	7.08	7.92	11.24	20.89	17.68	16.17	18.37	18.28	1.63	0.31274	13.76	16.68	7.77	12.74	23.85	31.67	27.96	27.83	2.18	0.006274	7.43	13.36	7.94	9.58	13.66	10.61	14.28	12.85	1.34	1	1.71	4.07	1.23	2.34	6.59	4.56	5.58	2.39	1
Solyc01g107030.2.1	Somatic embryogenesis relate	84.33	66.97	66.41	46.52	66.06	81.18	80.43	103.51	89.8	88.73	1.34	0.341162	65.31	71.9	63.39	66.87	72.2	73.43	86.25	77.29	1.16	0.852548	63.89	65.1	39.45	56.15	85.22	73.4	78.07	78.9	1.41	1	61.1	59.64	54.29	58.34	57.8	57.14	57.47	0.99	1
Solyc01g107040.2.1	Ribosomal RNA large subunit	31.15	31.77	36.95	29.95	32.45	29.18	22.79	27.83	25.63	26.36	0.81	0.800841	31.09	36.05	28.08	31.74	26.94	31.53	27.09	28.52	0.9	1	21.91	26.81	27.96	25.56	18.39	22.41	22.57	21.12	0.83	1	15.43	19.19	15.88	16.83	25.15	28.08	26.61	1.58	0.860061
Solyc01g107050.2.1	N-hydroxycinnamoyl/benzoyl	0	0	0	0	0	0	0	0	0	0	1	NA	0	0	0	0	0.28	0	0	0.09	9.33	1	0	0	0	0	0	0	0	0	1	NA	0	0	0	0	0	0	0	1	NA
Solyc01g107060.2.1	Unknown Protein (AHRD V1)	0	0	0	0	0	0	0	0	0	0	1	NA	0	0	0	0	0	0	0	0	1	NA	0	0	0	0	0	0	0	0	1	NA	0	0	0	0	0	0	0	1	NA
Solyc01g107070.2.1	N-hydroxycinnamoyl/benzoyl	18.13	15.11	19.57	14.73	16.88	26.16	17.97	24.48	15.79	21.1	1.25	0.800112	11.84	7.71	6.52	8.69	5.89	7.79	11.52	8.4	0.97	1	4.68	4.4	2.3	3.79	5.07	3.51	4.78	4.45	1.17	1	18.1	11.21	11.87	13.73	9.89	7.1	8.5	0.62	1
Solyc01g107080.2.1	Hydroxycinnamoyl transferas	0.31	0	0.18	0	0.12	0	0.12	0.14	0.11	0.09	0.76	1	0.58	0.95	1.46	1	2.07	1.87	2.83	2.26	2.26	0.353877	0.13	0.26	0.16	0.18	0	0	0	0	0.05	1	2.01	0.23	0	0.75	0.14	0.14	0.14	0.19	1
Solyc01g107090.2.1	Beta-1 4-xylosidase (AHRD V1	2.22	1.97	2.62	3.14	2.49	2.46	1.75	1.02	2.39	1.91	0.77	1	1.55	0	1.87	1.14	2.31	1.44	0.87	1.54	1.35	1	0	1.36	0.55	0.64	0.73	0.42	0	0.38	0.6	1	267.99	0.81	0	89.6	0	0.49	0.24	0	0.949536
Solyc01g107100.2.1	Beta-1 4-xylosidase (AHRD V1	1.27	0	1.5	4.49	1.81	2.12	2.5	0.58	0.91	1.53	0.84	1	1.77	1.65	0.54	1.32	5.72	3.61	4.99	4.77	3.62	0.294342	0	1.55	0.63	0.73	0	0	0	0	0.01	1	919.94	3.23	27.31	316.83	30.2	75.08	52.64	0.17	1
Solyc01g107110.2.1	Unknown Protein (AHRD V1)	0	0	0	0	0	0	0	0	0	0	1	NA	0	0	0	0	0	0	0	0	1	NA	0	0	0	0	0	0	0	0	1	NA	0	0	0	0	0	0	0	1	NA
Solyc01g107120.2.1	Legume lectin beta domain (A	0	0.09	0	0.14	0.06	0	0.16	0.09	0.15	0.1	1.74	1	0.09	0.18	0.09	0.12	0.14	0.16	0.08	0.13	1.06	1	0	0	0	0	0	0	0	0	1	1	0	0	0	0	0	0	0	1	NA
Solyc01g107130.2.1	Vacuolar sorting receptor (AH	71.3	72.67	74.99	70.25	72.3	56.02	51.5	58.64	64.4	57.64	0.8	0.72385	71.19	76.89	82.21	76.76	91.29	82.44	71.24	81.66	1.06	1	86.24	96.53	89.04	90.6	99.11	82.41	90.81	90.78	1	1	135.12	117.7	107.23	120.02	102.88	103.13	103	0.86	1
Solyc01g107140.1.1	BHLH transcription factor (AH	0	0	0	0	0	0	0	0	0	0	1	NA	0	0	0	0	0	0	0	0	1	NA	0	0	0	0	0	0	0	0	1	NA	0	0	0	0	0	0	0	1	NA
Solyc01g107150.2.1	Zinc finger protein-like 1 (AH	46.77	47.55	41.5	47.33	45.79	41.21	62.14	41.25	44.36	47.24	1.03	1	48.63	37.18	41.43	42.41	29.14	35.04	42.43	35.54	0.84	0.962977	52.38	48.34	41.33	47.35	35.47	44.53	39.76	39.92	0.84	1	36.76	29.41	22.51	29.56	34.63	33.51	34.07	1.15	1
Solyc01g107160.2.1	Mitotic checkpoint protein BU	29.41	23.42	25.11	27.37	26.33	18.49	21.69	25.26	22.64	22.02	0.84	0.955025	29.69	21.19	22.55	24.48	21.74	18.63	18.09	19.49	0.8	0.895788	15.47	16.71	16.71	16.3	21.35	19.8	16.92	19.36	1.19	1	31.08	25.71	27.96	28.25	26.03	30.02	28.02	0.99	1
Solyc01g107170.2.1	Zinc finger protein (AHRD V1	7.42	4.91	9.01	4.8	6.54	8.96	5.69	9.95	8.22	8.21	1.26	0.804025	10.06	10.69	9.67	10.14	8.97	8.68	4.67	7.44	0.73	0.88089	109.34	108.55	155.81	124.57	82.91	92.93	117.94	97.93	0.79	1	122.75	91.82	67.85	94.14	46.22	33.66	39.94	0.42	0.341712
Solyc01g107180.2.1	Phototropic-responsive NPH3	8.1	11.14	5.71	7.74	8.17	10.88	7.48	13.16	8.38	9.97	1.22	0.843786	9.88	12.71	8.98	10.52	8.54	7.41	11.08	9.01	0.86	0.997169	3.15	3.6	1.46	2.74	2.97	3.11	2.91	3	1.1	1	8.83	3.74	5.18	5.92	2.89	7.38	5.13	0.87	1
Solyc01g107190.2.1	LOB domain protein 38 (AHRD	6.66	3.23	8.57	6.42	6.22	2.69	0.96	1.95	1.09	1.67	0.27	0.008509	4.22	7.63	10.74	7.53	1.47	4.39	4.76	3.54	0.47	0.475775	8.07	7.89	12.32	9.43	3.78	5.05	2.43	3.75	0.4	0.204461	3.01	3.52	3.72	3.42	2.22	8.23	5.23	1.53	1
Solyc01g107200.2.1	Os02g0794300 protein (Frag	4.22	4.12	5.95	6.84	5.28	4.23	5.61	2.74	4.14	4.18	0.79	0.980869	3.35	2.42	2.61	2.79	2.78	2.82	2.43	2.68	0.96	1	1.25	2.1	0.82	1.39	1.01	0.86	1.65	1.17	0.84	1	0.59	0.15	0.32	0.35	0.47	0.54	0.51	1.43	1
Solyc01g107210.2.1	AT2G14850 protein (Fragmen	15.19	17.76	14.44	23.09	17.62	14.11	13.07	12.3	13.18	13.17	0.75	0.73223	14.76	15.37	13.02	14.38	10.38	13.73	14.79	12.97	0.9	1	16.67	14.96	12.83	14.82	14.45	14.43	12.72	13.87	0.94	1	12.89	17.95	16.38	15.74	13.7	13.32	13.51	0.86	1
Solyc01g107220.2.1	Unknown Protein (AHRD V1)	0	0	0	0	0	0	0	0	0	0	1	NA	0	0	0	0	0	0	0	0	1	NA	0	0	0	0	0	0	0	0	1	NA	0	0	0	0	0	0	0	1	NA
Solyc01g107230.2.1	Unknown Protein (AHRD V1)	0	0	0	0	0	0	0	0	0	0	1	NA	0	0	0	0	0	0	0	0	1	NA	0	0	0	0	0	0	0	0	1	NA	0	0	0	0	0	0	0	1	NA
Solyc01g107240.2.1	Peroxisomal membrane MPV	7.48	5.75	6.82	3.37	5.86	6.43	7.25	6.57	7.81	7.02	1.2	0.975665	6.64	5.32	4.02	5.33	3.77	4.93	6.41	5.04	0.95	1	3.57	2.77	2.03	2.79	1.79	2.58	2.73	2.37	0.85	1	0	2.96	2.61	1.86	2.8	2.39	2.59	1.4	1
Solyc01g107250.2.1	LRR receptor-like serine/thre	31.78	37.44	42.84	37.24	37.33	30.27	21.98	37.01	22.59	27.96	0.75	0.579908	28.71	36.83	17.64	27.73	10.7	12.69	19.98	14.46	0.52	0.319618	17.85	13.81	10.21	13.96	7.95	13.07	8.38	9.8	0.7	1	2.58	15.39	7.45	8.47	5.54	5.04	5.29	0.62	1

Solyc01g107260.2.1	Transport protein-related (AH	0.08	0.27	0.09	0.43	0.22	0	0.18	0.07	0.11	0.09	0.41	0.951897	0.14	0	0.13	0.09	0	0.12	0.12	0.08	0.89	1	0.06	0	0	0.02	0.1	0	0	0.03	1.67	1	0	0	0	0	0	0	0	1	NA
Solyc01g107270.2.1	Phosphoribosylformylglycinar	1.34	1.32	1.63	1.26	1.39	0.66	1.17	1.09	1.31	1.06	0.76	0.952743	1.56	1.33	2	1.63	1.78	2.36	2.29	2.14	1.31	0.750424	0.4	0.56	0.44	0.47	0.65	0.56	0.43	0.55	1.17	1	0.98	1.33	1.67	1.33	1.58	1.91	1.75	1.32	1
Solyc01g107280.2.1	Unknown Protein (AHRD V1)	0	0.53	0	0.84	0.34	0	0	0	0	0	0.03	1	0	0	0	0	0	0.31	1.4	0.57	57	0.978434	0	0	0	0	0	0	0	0	1	NA	0	0	0	0	0	0	0	1	NA
Solyc01g107290.2.1	RING-H2 finger protein (AHRI	28.58	15.05	12.93	35.04	22.9	18	22.56	13.14	19.49	18.3	0.8	1	17.72	21.74	24.74	21.4	23.79	32.18	20.59	25.52	1.19	0.773095	30.02	39.2	64.77	44.66	29.1	38.45	34.02	33.86	0.76	1	102.6	84.24	75.44	87.43	42.08	43.02	42.55	0.49	0.257307
Solyc01g107300.2.1	Phosphatase 2C family protei	9.75	15.32	10.83	10.95	11.71	9.57	7.02	8.44	6.8	7.96	0.68	0.501175	9.07	12.22	11.63	10.97	6.96	9.95	8.34	8.42	0.77	0.961157	9.04	14.26	24.2	15.83	10.93	15	8.57	11.5	0.73	1	37.93	65.83	81.16	61.64	33.35	35.71	34.53	0.56	0.67165
Solyc01g107310.2.1	Phosphatase 2C family protei	19.51	26.2	20.09	23.08	22.22	16.95	12.04	16.96	11.97	14.48	0.65	0.327866	16.5	23.12	21.87	20.5	15.74	19.47	16.17	17.13	0.84	1	18.05	23.41	48.58	30.01	23.77	31.99	15.51	23.76	0.79	1	71.1	146.32	178.9	132.11	70.13	75.88	73	0.55	0.812129
Solyc01g107320.2.1	Pentatricopeptide repeat-con	4.26	3.37	4.84	3.3	3.94	4.38	3.75	3.62	5.37	4.28	1.09	0.993465	4.42	4.27	5.5	4.73	7.03	6.21	5.75	6.33	1.34	0.575579	5.46	7.28	5.51	6.08	6.03	5.98	8.68	6.9	1.13	1	1.72	4.03	2.47	2.74	5.38	4.09	4.73	1.73	0.98046
Solyc01g107330.2.1	SWIB/MDM2 domain protein	78.54	67.1	70.95	97.76	78.59	119.73	134.99	126.68	109.55	122.74	1.56	0.041594	68.46	55.01	70.88	64.78	117.97	88.3	112.86	106.38	1.64	0.0461	24.66	46.44	40.98	37.36	19.75	32.31	53.96	35.34	0.95	1	9.57	15.97	13.8	13.11	39.44	36.2	37.82	2.88	0.117917
Solyc01g107340.2.1	Unknown Protein (AHRD V1)	15.45	13.17	29.23	50.98	27.21	18.24	31.5	12.13	12.71	18.64	0.69	1	11.67	5.59	10.6	9.29	2.46	5.33	3.29	3.69	0.4	0.175855	5.72	16.26	10.22	10.73	8.46	6.1	3.75	6.1	0.57	1	0.46	0	0	0.15	0.29	0.28	0.29	1.86	1
Solyc01g107350.2.1	Os12g0581300 protein (Fragr	6.86	11.47	6.11	14.66	9.78	4.2	9.51	4.25	4.73	5.67	0.58	0.623277	6.51	5.48	8.87	6.95	4.57	4.28	4.07	4.31	0.62	0.410746	1.24	1.83	0.42	1.16	0.92	0.42	0.71	0.68	0.59	1	0.6	0.71	0.65	0.65	0.9	0.98	0.94	1.44	1
Solyc01g107360.2.1	Coatomer subunit beta&apos	4.99	4.87	4.53	3.7	4.52	5	3.23	3.83	4.28	4.08	0.9	1	5.93	6.82	7.53	6.76	6.41	8.04	5.91	6.79	1	1	10.1	14.12	12.17	12.13	9.45	11.96	11.26	10.89	0.9	1	4.53	8.67	8.55	7.25	10.33	12.4	11.37	1.57	0.904864
Solyc01g107370.2.1	Gibberellin-regulated family p	0	0	0	0	0	0	0	0	0	0	1	NA	0	0	0	0	0	0	0	0	1	NA	0.59	0	0	0.2	0	0	0	0	0.05	1	0	0	0	0	0	0	0	1	NA
Solyc01g107380.2.1	Seed maturation protein PM2	140.71	138.98	143.04	133.27	139	176.89	163.31	175.19	183.15	174.63	1.26	0.443991	131.54	161.98	150.86	148.13	211.14	177.33	182.84	190.44	1.29	0.433962	147	164.85	152.91	154.92	147.07	163.3	153.04	154.47	1	1	229.5	212.81	197.5	213.27	192.09	201.83	196.96	0.92	1
Solyc01g107390.2.1	Auxin-responsive GH3 produc	5.63	3.34	3.64	2.96	3.89	3.19	6.27	1.93	1.58	3.24	0.83	1	3.8	1.63	4.29	3.24	1.15	0.8	0.78	0.91	0.28	0.073644	17.74	15.46	22.56	18.59	7.75	11.29	10.21	9.75	0.52	1	5.18	16.51	6.96	9.55	4.75	4.74	4.75	0.5	0.910144
Solyc01g107400.2.1	Indole-3-acetic acid-amido sy	2.49	6.09	11.01	12.32	7.98	6.92	0.98	8.01	8.94	6.21	0.78	1	0.58	3.79	1.58	1.98	2.59	2.26	4.4	3.08	1.55	1	1100.66	56.29	1301	819.32	206.06	487.28	243.54	312.29	0.38	1	24.78	50.18	94.75	56.57	101.98	44.2	73.09	1.29	1
Solyc01g107410.2.1	PHD-finger family homeodom	11.01	11.12	14.45	14.75	12.83	10.49	8.23	13.09	10.76	10.64	0.83	0.936624	12.17	11.87	15.8	13.28	10.51	12.77	11.51	11.6	0.87	1	6.82	7.08	9.12	7.67	6.47	9.66	5.67	7.27	0.95	1	25.3	11.18	16.04	17.51	14.02	15.6	14.81	0.85	1
Solyc01g107420.2.1	TIP41-like protein (AHRD V1 *	6.28	9.51	8.15	4.44	7.09	13.97	8.43	8.09	9.47	9.99	1.41	0.866794	7.01	17.48	9.55	11.35	9.15	11.39	19.26	13.27	1.17	1	9.64	6.14	3.74	6.51	5.78	9.52	6.87	7.39	1.14	1	7.15	10.04	11.59	9.59	8.63	9.92	9.28	0.97	1
Solyc01g107430.2.1	Zinc finger protein 7 (AHRD V	9.86	9.45	1.34	6.44	6.77	14.33	17.06	4.88	5.72	10.5	1.55	0.898546	6	4.61	3.52	4.71	5.26	5.69	7.75	6.23	1.32	0.953962	0	0	0.38	0.13	0	0	0	0	0.08	1	5.39	0.28	1.17	2.28	0.69	1.66	1.18	0.52	1
Solyc01g107440.2.1	Unknown Protein (AHRD V1)	0	0	0	0	0	0	0	0	0	0	1	NA	0	0	0	0	0	0	0	0	1	NA	0	0	0	0	0	0	0	0	1	NA	0	0	0	0	0	0	0	1	NA
Solyc01g107450.2.1	Unknown Protein (AHRD V1)	68.01	33.29	49.75	90.43	60.37	33.26	96.11	26.26	70.31	56.48	0.94	1	57.57	50.25	47.68	51.83	54.25	54.93	34.74	47.97	0.93	1	53.3	48.22	41.85	47.79	46.02	52.58	54.52	51.04	1.07	1	15.47	22.23	15.69	17.8	49.81	42.34	46.08	2.59	0.271321
Solyc01g107460.2.1	Unknown Protein (AHRD V1)	0	0.42	0	0	0.1	9.43	3.35	14.73	9.13	9.16	87.24	2.12E-05	1.75	0.82	0	0.86	0.98	2.2	2.96	2.05	2.39	0.809114	7.61	5.38	0	4.33	5.88	2.86	3.78	4.17	0.96	1	0	0.34	0	0.11	0	0	0	0.09	1
Solyc01g107470.2.1	Unknown Protein (AHRD V1)	31	29.71	30.95	24.73	29.1	22.97	23.22	27.77	28.53	25.62	0.88	1	34.01	22.11	25.51	27.21	20.4	15.64	15.62	17.22	0.63	0.593914	21.19	30.44	12.62	21.42	15.14	13.25	17.96	15.45	0.72	1	5.65	11.55	8.56	8.59	6.55	9.06	7.8	0.91	1
Solyc01g107490.1.1	BHLH transcription factor (AH	0	0	0	0	0	0	0	0	0.19	0.05	4.75	1	0	0	0.23	0.08	0	0.14	0	0.05	0.61	1	0.22	0	0	0.07	0	0	0	0	0.14	1	0	0.19	0	0.06	0	0	0	0.16	1
Solyc01g107500.2.1	Unknown Protein (AHRD V1)	24.36	22.58	14.37	33.02	23.58	16.92	15.22	16.8	16.76	16.43	0.7	0.808522	16.99	35.29	27.43	26.57	23.93	24.17	15.95	21.35	0.8	1	13.12	15.71	25.19	18.01	40.67	38.46	15.53	31.55	1.75	1	38.96	36.86	42.14	39.32	49.24	49.83	49.53	1.26	1
Solyc01g107510.2.1	DNA polymerase IV (AHRD V1	16.9	15.77	13.54	23.91	17.53	11.08	12.15	12.87	12.27	12.09	0.69	0.390891	15.25	26.43	24.66	22.11	12.07	16.13	14.18	14.13	0.64	0.328156	10.65	11.67	20.6	14.31	18.02	18.93	8.46	15.14	1.06	1	27.89	34.4	38.89	33.73	34.47	32.74	33.61	1	1
Solyc01g107520.2.1	PBSP domain-containing prot	0.57	0.76	0.33	0	0.42	0.63	0.45	1.04	0.2	0.58	1.4	1	1.84	0.49	0.24	0.86	0	0.73	0.67	0.47	0.54	1	0	0	0	0	0	0	0	0	1	NA	0	0	0	0	0.26	0	0.13	13	1
Solyc01g107530.1.1	Unknown Protein (AHRD V1)	0	0	0	0	0	0	0	0	0	0	1	NA	0	0	0	0	0	0	0	0	1	NA	0	0	0	0	0	0	0	0	1	NA	0	0	0	0	0	0	0	1	NA
Solyc01g107540.2.1	PBSP domain-containing prot	0	0	0	0	0	0	0	0.26	0.2	0.12	11.5	1	0	0	0	0	0.2	0	0	0.07	6.67	1	0	0	0	0	0	0	0	0	1	NA	0	0	0	0	0	0	0	1	NA
Solyc01g107550.2.1	Methylthioribose kinase (AHF	78.66	80.89	83.01	76.25	79.7	75.07	66.19	69.88	82.12	73.31	0.92	1	85.27	78.6	71.74	78.54	75.75	67.83	75.86	73.15	0.93	1	76.09	83.38	91.15	83.54	73.4	79.93	84.5	79.28	0.95	1	69.51	82.4	66.27	72.73	89.22	81.2	85.21	1.17	1
Solyc01g107560.2.1	UDP-glucosyltransferase 1 (A	6.89	4.33	6.54	4.59	5.59	7.81	5.66	7.83	7.08	7.1	1.27	0.720514	8.43	7.29	6.97	7.56	9.19	8.33	6.91	8.14	1.08	1	1.31	1.76	1.48	1.52	1.51	1.64	2.66	1.94	1.28	1	1.54	2.85	2.7	2.36	1.24	0.95	1.09	0.46	0.86722
Solyc01g107570.1.1	Unknown Protein (AHRD V1)	0	0	0	0	0	0	0	0	0	0	1	NA	0	0	0	0	0	0	0	0	1	NA	0	0	0	0	0	0	0	0	1	NA	0	0	0	0	0	0	0	1	NA
Solyc01g107580.2.1	Pseudouridine synthase famil	3.1	2.38	2.66	1.99	2.53	0.63	3.67	2.33	1.92	2.14	0.84	1	3.02	2.33	2.26	2.54	1.47	1.9	1.66	1.68	0.66	0.883675	1.71	1.84	0.84	1.46	1.39	0.96	1.03	1.13	0.77	1	1.8	1.84	3.03	2.22	1.94	1.85	1.9	0.85	1
Solyc01g107590.2.1	Cinnamyl alcohol dehydrogen	12.35	18.86	10.22	6.26	11.92	15.16	17.75	21.35	15.35	17.4	1.46	0.438826	16.46	11.53	9.81	12.6	5.11	9.35	14.34	9.6	0.76	0.988612	2.53	1.95	0.37	1.62	0.97	0.42	0.94	0.78	0.48	1	0.52	1.07	1.13	0.91	1.18	0.48	0.83	0.92	1
Solyc01g107600.2.1	Unc-50 homolog (AHRD V1 **	37.92	37.68	20.22	28.66	31.12	34.36	35.06	37.73	33.19	35.09	1.13	0.959699	33.82	35.45	25.89	31.72	26.66	31.87	33.47	30.67	0.97	1	37.98	40.21	38.67	38.95	35.83	34.45	43.15	37.81	0.97	1	29.54	33.58	41.54	34.89	29.24	30.29	29.77	0.85	1
Solyc01g107610.2.1	PRKR interacting protein 1 (A	29.03	30.87	33.8	29.33	30.76	21.37	24.11	22.88	33.02	25.34	0.82	0.968545	36.82	29.82	37.25	34.63	30.85	31.03	20.74	27.54	0.8	0.954908	25.28	31.64	24.7	27.21	28.73	28.57	28.57	28.62	1.05	1	26.8	33.13	22.61	27.51	24.5	29.75	27.12	0.99	1
Solyc01g107620.2.1	Reticulon family protein (AHF	15.59	23.75	12.38	14	16.43	15.33	10.18	18.75	12.06	14.08	0.86	0.914991	17.01	17.73	13.68	16.14	15.18	13.35	17.91	15.48	0.96	1	17.93	13.2	16.97	16.03	13	13.18	10.93	12.37	0.77	1	17.48	22	29.05	22.84	8.24	13.67	10.96	0.48	0.523714
Solyc01g107630.2.1	Cell division cycle protein 123	8.22	10.8	7.39	7.48	8.47	9.36	6.03	11.34	7.59	8.58	1.01	1	9.1	7.49	5.79	7.46	5.3	4.97	9.08	6.45	0.86	1	3.8	3.51	2.33	3.21	2.44	2.52	3	2.65	0.83	1	4.73	7.68	6.92	6.44	1.43	2.4	1.92	0.3	0.202689
Solyc01g107640.2.1	Tic21 (AHRD V1 **-- Q09S70_	80.61	75.05	108.25	129.79	98.42	135.56	133.87	99.53	121.07	122.51	1.24	0.361647	76.06	83.19	97.32	85.52	122.77	103.07	103.69	109.84	1.28	0.47981	82.58	99	124.74	102.11	74.47	110.04	92.09	92.2	0.9	1	24.77	36.27	30.01	30.35	77.4	65.99	71.69	2.36	0.120227
Solyc01g107650.2.1	Receptor like kinase, RLK	0.6	1.14	1.01	0.97	0.93	0.57	0.88	0.79	0.37	0.65	0.7	0.924458	0.4	0.67	0.79	0.62	0.71	0.4	0.27	0.46	0.74	1	0	0.07	0	0.02	0.06	0	0	0.02	0.86	1	0	0.06	0	0.02	0	0	0	0.5	1
Solyc01g107660.2.1	Stress enhanced protein 1 (Fr	105.93	102.54	85.51	82.8	94.2	203.99	164.32	145.23	157.43	167.74	1.78	0.008267	104.15	115.5	81.23	100.29	139.85	129.89	132.9	134.21	1.34	0.348195	53.3	63.98	63.18	60.15	50.36	73.24	72.79	65.46	1.09	1	18.5	25.64	19.29	21.14	42.95	41.14	42.05	1.99	0.580515
Solyc01g107670.2.1	LRR receptor-like serine/thre	15.16	18.24	15.44	15.59	16.11	20.49	12.65	19.48	16.32	17.23	1.07	1	22.91	28.6	30.26	27.26	21.86	35.94	30.46	29.42	1.08	1	6.12	8.84	5.13	6.7	7.81	8.36	7.41	7.86	1.17	1	21.06	20.27	29.67	23.67	35.16	53.46	44.31	1.87	0.297221
Solyc01g107680.2.1	ATP-dependent Clp protease	2.75	9.79	11.89	9.07	8.38	3.06	12.3	9.27	7.89	8.13	0.97	1	8.52	10.35	17.8	12.22	7.63	8.54	6.48	7.55	0.62	0.843955	2.22	3.73	3.64	3.2	3.61	4.17	3.34	3.71	1.16	1	5.21	3.33	4.23	4.26	8.39	13.66	11.03	2.59	0.850203
Solyc01g107690.2.1	Unknown Protein (AHRD V1)	65.97	118.54	64.62	61.62	77.69	103.77	80.58	124.75	73.28	95.59	1.23	0.899014	77.57	113.61	125.11	105.43	48.35	99.25	113.47	87.02	0.83	1	31.17	32.46	50.66	38.1	30.26	30.19	27.21	29.22	0.77	1	69.02	52.44	70.82	64.09	83.18	103.68	93.43	1.46	0.938102
Solyc01g107700.2.1	Kynurenine formamidase (AH	56.23	50.48	57.73	71.97	59.1	53.04	67.27	46.56	43.87	52.69	0.89	1	71.55	78.3	81.83	77.23	89.66	69.58	59.88	73.04	0.95	1	180.74	167.22	204.18	184.05	154.32	153.33	131.45	146.37	0.8	1	115.6	144.63	134.01	131.41	140.84	145.99	143.42	1.09	1
Solyc01g107710.2.1	Root cap protein 3 (Fragment	0	0	0	0	0	0	0	0	0	0	1	NA	0	0	0	0	0	0	0	0	1	NA	0	0	0	0	0	0	0	0	1	NA	0	0	0	0	0	0	0	1	NA
Solyc01g107720.2.1	Root cap protein 3 (Fragment	0	0	0	0	0	0	0	0	0	0	1	NA	0	0	0	0	0	0	0	0	1	NA	0	0	0	0	0	0	0	0	1	NA	0	0	0	0	0	0	0	1	NA
Solyc01g107730.2.1	Cyclin (AHRD V1 ***- Q1XGF	24.58	42.15	20.68	28.12	28.88	47.82	23.54	42.71	25.69	34.94	1.21	0.988316	29.25	18.23	14.66	20.71	8.4	15.35	15.91	13.22	0.64	0.786426	0.44	0.29	0.36	0.36	0.12	0	0.26	0.13	0.35	1	0	0.13	0	0.04	0.17	0	0.09	1.96	1
Solyc01g107740.2.1	Protein unc-13 homolog C (A	17.94	16.71	18.89	26.16	19.93	19.94	22.45	12.69	13.48	17.14	0.86	1	25.93	31.67	24.48	27.36	30.06	36.73	21.04	29.28	1.07	1	48.84	39.81	89.04	59.23	69.27	76.78	47.67	64.57	1.09	1	192.3	138.13	158.75	163.06	170.31	140.86	155.59	0.95	1
Solyc01g107750.2.1	Phosphatase and tensin-like A	51.5	50.38	74.32	62.91	59.78	57.84	66.87	71.71	52.48	62.23	1.04	0.990044	64.53	58.64	69.56	64.24	64.06	62.14	61.29	62.5	0.97	1	64.9	70.93	69.28	68.37	65.22	62.26	61.95	63.14	0.92	1	132.22	91.43	90.48	104.71	76.17	75.94	76.06	0.73	1
Solyc01g107760.2.1	Cathepsin B-like cysteine prot	0	0.12	0.33	0	0.11	0	0	0	0.1	0.03	0.22	1	0	0	0	0	0.29	0.07	0	0.12	12	0.987668	0	0	0	0	0	0	0	0	1	NA	0	0	0	0	0	0	0	1	NA
Solyc01g107770.1.1	UDP-glucosyltransferase fami	0	0	0	0	0	0	0	0	0	0	1	NA	0	0	0	0	0	0	0	0	1	NA	0	0	0	0	0	0	0	0	1	NA	0	0	0	0	0	0	0	1	NA
Solyc01g107780.2.1	UDP-glucosyltransferase fami	5.16	3.14	2.08	2.3	3.17	2.26	2.68	2.25	1.46	2.16	0.68	0.671972	0.63	2.71	1.15	1.5	3.95	1.05	3.2	2.73	1.83	0.890998	3.18	4.76	2.02	3.32	1.96	0.62	0.89	1.16	0.35	0.753079	0.39	0.99	2.3	1.23	0.99	0.6	0.79	0.65	1
Solyc01g107790.2.1	LRR receptor-like serine/thre	0	0	0	0	0	0	0	0	0	0	1	NA	0	0	0	0	0	0.09	0	0.03	3	1	0	0	0	0	0	0	0	0	1	NA	0	0	0	0	0	0	0	1	NA
Solyc01g107800.2.1	Expressed protein (Fragment)	48.8	56.29	73.63	75.54	63.56	57.5	79.86	87.61	55.33	70.08	1.1	0.821423	43.44	42.42	47.41	44.42	30.21	34.59	28.47	31.09	0.7	0.464069	32.83	86.01	29.93	49.59	45.4	20.16	91.26	52.27	1.05	1	0.3	6.24	2.58	3.04	5.37	6.98	6.18	2.03	1
Solyc01g107810.2.1	UDP-glucosyltransferase fami	0.82	0.61	0	0.39	0.45	0.76	0.22	0.5	0.29	0.44	0.97	1	0.38	0.12	0	0.17	0.09	0.28	0	0.12	0.74	1	0.11	0	0	0.04	0	0.1	0	0.03	0.91	1	0	0	0.21	0.07	0	0	0	0.14	1
Solyc01g107820.2.1	UDP-glucosyltransferase fami	26.05	29.42	28.75	35.46	29.92	15.26	17.91	33.1	18	21.07	0.7	0.47293	25.28	39.57	36.32	33.72	26.42	30.1	27.74	28.09	0.83	0.953962	27.84	22.14	43.52	31.17	19.6	11.15	10.82	13.86	0.44	1	36.05	86.47	85.94	69.49	9.1	17.68	13.39	0.19	0.043322
Solyc01g107830.2.1	UDP-glucosyltransferase fami	9.18	8.55	8.33	4.2	7.57	5.18	4.91	6.36	3.96	5.1	0.67	0.384559	16.56	21.74	15.39	17.9	20.77	20.77	14.99	18.84	1.05	1	179.64	117.76	260.39	185.93	196.37	210.28	251.18	219.28	1.18	1	34.14	71.82	39.97	48.64	68.15	33.19	50.67	1.04	1
Solyc01g107840.2.1	Immediate-early salicylate-in	1.35	2.4	0.79	1.9	1.61	1.5	2.13	2.48	2.42	2.13	1.32	1	13.15	4.68	5.69	7.84	13.54	14.64	6.35	11.51	1.47	0.833891	81.04	21.39	54.79	52.41	46.43	43.88	86.34	58.88	1.12	1	9.57	24.45	17.6	17.21	9.24	1.77	5.5	0.32	0.360559
Solyc01g107850.2.1	UDP-glucosyltransferase HvU	1.21	3.23	2.14	3.42	2.5	1.35	3.34	5.56	3.04	3.32	1.33	1	14.07	10	9.71	11.26	18.89	20.37	9.04	16.1	1.43	0.677701	107.58	35.51	105.13	82.74	69.58	70.19	143.78	94.52	1.14	1	16.35	36.49	24.2	25.68	12.74	4.25	8.5	0.33	0.21158
Solyc01g107860.2.1	CBS domain-containing prote	83.35	79.98	75.91	94.85	83.52	80.88	96.45	98.1	113.24	97.17	1.16	0.645136	75.76	83.78	68.79	76.11	52.72	51.87	68.27	57.62	0.76	0.605865	84.01	87.16	104.29	91.82	107.9	116.05	87.64	103.86	1.13	1	30.88	62.9	76.4	56.73	74.06	86.17	80.12	1.41	0.984911
Solyc01g107870.2.1	Poly(A) RNA binding protein (	415.29	419.66	758.38	775.22	592.14	400.25	437.53	411.03	489.58	434.6	0.73	0.833796	486.44	457.98	676.87	540.43	641.87	583.81	549.04	591.57	1.09	0.962977	369.94	415.45	561.17	448.85	426.05	419.03	402.06	415.71	0.93	1	296.39	316.28	319.46	310.71	381	413.43	397.22	1.28	1
Solyc01g107880.2.1	Membrane-associated ring fir	37.7	42.84	51.12	48.38	45.01	32.94	29.67	25.94	35.62	31.04	0.69	0.301201	43.45	53.12	63.78	53.45	56.24	48.15	45.28	49.89	0.93	1	37.02	38.35	48.83	41.4	38.7	36.16	29.85	34.9	0.84	1	77.11	44.34	50.4	57.28	60.5	74.73	67.62	1.18	1
Solyc01g107890.2.1	Filament-like plant protein (F	48.41	52.84	54.87	55.76	52.97	51.24	45.18	50.12	55.77	50.58	0.95	1	47.4	28.32	31.9	35.87	19.62	27.8	27.74	25.05	0.7	0.720252	0.33	0.67	0.74	0.58	0.76	0.31	0.6	0.56	0.96	1	0.59	0.12	0.13	0.28	0.15	0.43	0.29	1.04	1
Solyc01g107900.2.1	Acyltransferase WS/DGAT/M	23.23	23.46	36.14	31.24	28.52	22.49	20.7	21.22	27.11	22.88	0.8	0.899951	28.89	53.63	51.87	44.8	63.45	48.17	42.66	51.43	1.15	0.991848	50.77	42.73	56.29	49.93	42.22	39.85	36.13	39.4	0.79	1	151.33	122.12	113.94	129.13	130.69	125.3	128	0.99	1
Solyc01g107910.2.1	Caffeoyl CoA 3-O-methyltrans	5.83	13.44	5.94	2.62	6.96	13.55	8.58	12.91	6.66	10.43	1.5	0.865654	7.64	5.07	2.69	5.13	1.1	3.02	3.96	2.69	0.52	0.762128	3.21	1.94	1.31	2.15	1.22	2.01	1.35	1.53	0.71	1	0	1.35	3.26	1.54	0	0	0	0.01	0.852617
Solyc01g107920.2.1	Os07g0175100 protein (Fragr	2.07	2.26	2.25	0.45	1.76	1.24	2.01	3.22	2.62	2.27	1.29	0.957736	5.18	4.15	2.42	3.92	2.32	3.05	4.87	3.41	0.87	1	5.01	6.35	2.05	4.47	4.59	3.74	4.87	4.4	0.98	1	0.23	3.12	0.98	1.44	3.2	2.09	2.65	1.83	1
Solyc01g107930.2.1	RING-finger protein like (AHR	6.42	6.12	10.18	8.05	7.69	7.14	6.3	4.98	7.11	6.38	0.83	0.992609	11.44	8.37	10.54	10.12	11.65	9.36	9.45	10.15	1	1	11.12	9.79	10.2	10.37	9.95	8.9	9.23	9.36	0.9	1	9	9.26	9.17	9.14	10.84	11.1	10.97	1.2	1
Solyc01g107940.2.1	RING-finger protein like (AHR	8.76	7.21	6.93	5.8	7.17	8.7	5.81	6.85	7.29	7.16	1	1	8.93	10.29	11.25	10.16	8.6	8.11	8.57	8.43	0.83	0.962977	6.62	5.24	4.99	5.62	3.98	3.95	6.11	4.68	0.83	1	8.48	9.54	7.02	8.35	4.78	6.62	5.7	0.68	0.95812
Solyc01g107950.1.1	Transcription factor bHLH95 (	0	0.19	0	0	0.05	0	0	0	0	0	0.21	1	0	0	0	0	0	0	0	0	1	NA	0	0	0	0	0	0	0	0	1	NA	0	0	0	0	0	0	0	1	NA
Solyc01g107960.2.1	Transcription factor bHLH95 (	0	0	0	0	0	0	0	0	0	0	1	NA	0	0	0	0	0	0	0	0	1	NA	0	0	0	0	0	0	0	0	1	NA	0	0	0	0	0	0	0	1	NA
Solyc01g107970.1.1	Transcription factor bHLH95 (	0	0	0	0	0	0	0	0	0	0	1	NA	0	0	0	0	0	0	0	0	1	NA	0	0	0	0	0	0	0	0	1	NA	0	0	0	0	0	0	0	1	NA
Solyc01g107980.2.1	U-box domain-containing pro	7.29	5.5	11.23	12.79	9.2	4.94	6.64	4.23	3.87	4.92	0.53	0.35801	7.23	10.75	12.32	10.1	6.49	4.68	6.36	5.84	0.58	0.167762	7.82	8.14	9.44	8.47	5.42	6.01	4.62	5.35	0.63	1	7.22	5.18	6.83	6.41	7.4	7.23	7.32	1.14	1
Solyc01g107990.2.1	MAPprotein kinase-like prote	16.9	14.61	20.69	20.03	18.06	13.41	12.58	11.98	13.75	12.93	0.72	0.585705	21.85	19.58	18.08	19.84	11.38	16.25	15.05	14.23	0.72	0.6319	14.56	21.11	11.02	15.56	6.66	8.17	7.86	7.56	0.49	0.151413	1.83	1.99	0.74	1.52	1.03	0.85	0.94	0.62	1
Solyc01g108000.2.1	Receptor-like kinase (AHRD V	0.89	0.7	0.81	1.11	0.88	0.66	0.7	0.45	0.42	0.56	0.64	0.902661	0.18	0.86	0.17	0.4	0.48	0.2	0.39	0.36	0.88	1	0	0.08	0.2	0.09	0	0.22	0.07	0.1	1.04	1	0.28	0.14	0.45	0.29	0.99	0.26	0.62	2.16	1
Solyc01g108010.2.1	Limkain b1-like protein (AHRI	9.37	12.51	12.1	13.07	11.76	9.01	8.3	7.8	10.45	8.89	0.76	0.739052	11.42	12.11	15.08	12.87	12.82	12.33	11.34	12.16	0.95	1	11.76	13.93	14.71	13.47	10.07	13.05	12.27	11.8	0.88	1	24.66	19.41	19.67	21.25	17.16	15.65	16.41	0.77	1
Solyc01g108020.2.1	Thioredoxin m (AHRD V1 ***	117	114.18	101.05	85.58	104.45	131.67	155.38	128.01	121.94	134.25	1.29	0.469358	119.34	76.04	73.88	89.75	99.35	94.92	109.13	101.13	1.13	0.929939	37.4	52.87	28.58	39.62	31.61	31.77	33.2	32.19	0.81	1	10.36	19.32	14.57	14.75	18.02	16.94	17.48	1.19	1
Solyc01g108030.2.1	Methylcrotonoyl-CoA carbox	34.89	38.56	57.93	35.46	41.71	30.82	24.9	31.76	33.93	30.35	0.73	0.482562	46.86	39.47	47.62	44.65	39.11	41.16	42.4	40.89	0.92	1	65.81	51.97	62.62	60.13	41.44	47.02	44.54	44.33	0.74	1	5.09	28.11	14.92	16.04	19.14	13.37	16.25	1.01	1
Solyc01g108040.1.1	Dynamin-related protein 3B (	4.53	4.61	1.53	10.98	5.41	4.31	3.57	8.33	4.18	5.1	0.94	1	10.23	11.24	8.19	9.89	3.14	4.35	8.13	5.21	0.53	0.542235	3.14	3.16	1.93	2.74	5.52	4.9	1.89	4.1	1.5	1	6.44	4.23	7.96	6.21	6.51	13.04	9.77	1.57	1
Solyc01g108050.2.1	Unknown Protein (AHRD V1)	24.63	32.1	17.64	28.62	25.75	12.71	6.94	18.6	6.31	11.14	0.43	0.048356	28.64	30.59	28.23	29.15	4.88	15.03	17.97	12.63	0.43	0.082965	14.92	5.02	17.46	12.47	5.78	6	4.49	5.42	0.44	1	27.51	28.75	27.05	27.77	16.91	26.22	21.56	0.78	1
Solyc01g108060.1.1	Propionyl-CoA carboxylase (A	3.67	2.17	10.09	10.37	6.57	6.79	7.72	3.37	6.14	6	0.91	1	5.68	5.31	11.35	7.45	12.71	10.12	10.56	11.13	1.49	0.953391	9.87	16.92	12.13	12.97	12.04	8.33	10.68	10.35	0.8	1	0	0.89	0	0.3	4.47	3.21	3.84	12.94	1
Solyc01g108070.2.1	4-methyl-5(B-hydroxyethyl)-t	13.94	10.68	14.72	14.9	13.56	22.88	23.67	18.18	16.06	20.2	1.49	0.164078	12.97	11.84	14.51	13.11	17.99	16.2	14.52	16.24	1.24	0.735236	6.55	10.83	7.08	8.15	7.35	6.39	6.97	6.9	0.85	1	55.08	9.77	17.7	27.52	9.35	8.53	8.94	0.32	1
Solyc01g108080.2.1	BZIP transcription factor (AHF	78.37	76.4	92.46	49.24	74.12	51.24	36.4	74.83	66.65	57.28	0.77	0.698121	76.6	62.32	58.81	65.91	55.35	62.22	53.34	56.97	0.86	1	27.74	45.73	26.04	33.17	35.92	25.66	27.34	29.64	0.89	1	63.29	71.58	70.16	68.34	50.93	61.75	56.34	0.82	1
Solyc01g108090.1.1	Unknown Protein (AHRD V1);	0	0	0	0	0	0	0	0	0	0	1	NA	0	0	0	0	0	0	0	0	1	NA	0	0	0	0	0	0	0	0	1	NA	0	0	0	0	0	0	0	1	NA
[truncated: 4,966,818 more chars]
